# Dissecting organ-specific transcriptomes through RNA-sequencing

**DOI:** 10.1186/1746-4811-9-42

**Published:** 2013-10-25

**Authors:** Shan Guan, Yingqing Lu

**Affiliations:** 1 State Key Laboratory of Systematic and Evolutionary Botany, Institute of Botany, Chinese Academy of Sciences, 20 Nan Xin Cun, Xiangshan, Beijing 100093, China

**Keywords:** Paired-end reads, Transcriptome assembly, Organ development, Molecular network, Within-genet genomic variation

## Abstract

**Background:**

Organ-specific gene expression contains rich information about *in vivo* biological processes. This kind of information, previously gathered through microarray profiling, has been proven fruitful to the understanding of specific mutants, regulatory events, signaling, and development. With the advent of the next-generation sequencing (NGS) of RNAs, more quantitative and detailed information of gene expressions than previously available can now be collected for each organ or organ developmental stages. The combination of an object-oriented experimental design and an efficient treatment of the high volume information generated through a NGS platform may offer a powerful tool for inferring previously intractable developmental processes.

**Results:**

We collected transcriptomic data over a Solexa/Illumina platform on samples of *Ipomoea* leaf, sepal, and petals (at three developmental stages), and presented a method for analyzing transcriptomic variations within and between organs. We demonstrated that *in vivo* signals of transcriptomes can be retrieved *de novo* through the NGS techniques, proper data handling, bioinformatic tools, and the current understanding of molecular networks. We found that numbers of transcribed genes from both nuclear and chloroplast genomes decreased by the same order of leaf → sepal →petal. Petal resembled leaf in cell division patterns and abundance level of commonly expressed organelle genes. Its chloroplast transcripts constituted a subset of those in leaf. Moreover, reconstructions of multiple metabolic networks for each organ enabled inferences of substance flow, providing transcript evidence for the path of sucrose in leaf to anthocyanin synthesis in petal.

**Conclusion:**

Our results attest that developmental transcriptomes are highly informative for exploring connections between morphological traits and the associated molecular networks. Significant hypotheses have been developed, including that the petal is a derived organ of leaf and that its color can be modified by fluctuations of substance flow within the associated metabolic networks among organs.

## Background

Organ differentiation in multi-cellular organisms depends on highly organized molecular events and brings enhanced functionalities to the organisms for their adaptations to natural environments. This differentiation appears to be closely linked to genomic gene expression, as organs contribute most significantly to gene expression variation when compared to all other experimental variables [1]. Organs are connected by substance flow to maintain their overall functions, but the details of which have yet to be made clear at cellular and molecular levels. Knowing these details has significant bearings on understanding how molecular systems control cellular activities and associated phenotypes. Although a significant spot light has been given to gene regulation in interpreting developmental and phenotypic variation [2-4], much is yet to be known on how such gene regulation is realized on the genomic scale and what information is conveyed between cells and organs to keep the integrity of a genet. Here, we focus on the logic and ways of extracting information from sequence-based transcriptomes to shed light on relevant issues.

Key issues on organ functionality include how substance flow connects organs and how the flow is regulated at the whole plant level. These issues concern numerous biological processes at cellular and organ levels, requesting solutions of genomic scope. A genome-based transcriptome has been known to be sensitive to both developmental and environmental signals [5,6], thus is a natural starting point for explorations of organ-specific expressions. The collective gene expressions may provide information at two levels. One is an *in vivo* profile of transcript abundances of the living cells, and the other is a snapshot of genomic regulation. These kinds of information are highly useful in the light of recent findings that significant correlations exist between transcriptomic and protein profiles in roots of *Arabidopsis*[7] and human stem cells [8]. At least in these cases, it is possible to trace back dynamics of cellular and molecular activities to changes in transcriptomes. In a large picture, the correlations are expected to be held broadly in living cells, because they allow genomic regulations to be effective without involving other mechanisms for many molecular systems. Even in cases of post-transcriptional or post-translational modifications, knowing transcript dynamics is still a prerequisite for analysis of the modifications. Systematic analysis of transcriptomes may therefore yield useful inferences on both venues and mechanisms of various molecular systems in cells.

Technically, new biotechnologies such as the next generation sequencing (NGS) have dramatically increased the likelihood of obtaining *in vivo* information embedded in cells. The most cost-effective platforms in NGS are ones capable of producing short-read sequences in high throughputs and at an affordable price [9,10]. The reliability of the NGS data has been confirmed in several cases [11], but analysis of genomic expression patterns awaits further improvements. Obviously, an effective analysis of transcriptomes critically replies on both the sampling scheme and the quality of transcriptome reconstructions. We show that it is attainable now to carry out quantitative analysis of transcriptomes collected at cell and organ levels with NGS short-read assembly methods [12-14]. More significantly, the transcriptomic reconstruction can be performed in a species without genome reference. The *de novo* approach is highly attractive for genetically less studied or refractory species, potentially accelerating scientific discoveries.

Examples are given here to illustrate how to use the *de novo* approach to address issues on cellular activities and substance flow within a developing organ and between organs. Three organs - leaf, sepal, and petal - in the common morning glory (*Ipomoea purpurea*) are considered here, and an experiment is designed to focus on petal developmental stages, particularly the onset and progressive anthocyanin synthesis in the corolla. The transcriptomes of the sepal and the leaf on the same genet serve as comparison bases for analysis of the petal transcriptomes. By reconstructing developmental and organ-specific transcriptomes and connecting well-built transcriptomes to the current knowledge on cellular/molecular activities, we are able to reveal unprecedented details on cellular dynamics during the petal development, providing concrete evidence for inferring substance flow between organs and within petal developmental stages. The inferences shed much light on the origin of petals, while providing a rich context in which different molecular systems are interconnected and regulated to render specific functions.

## Results and discussion

### Transcriptomic reconstruction, classification, evaluation, and quantification

**Figure 1:**
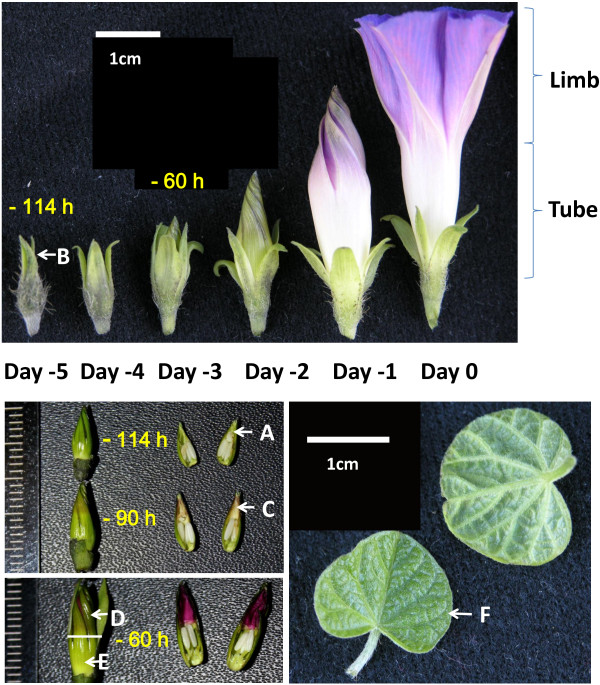
**Sampling scheme for organ-specific transcriptomic reconstructions in *Ipomoea purpurea*.** Three developmental stages (114, 90, and 60 hrs before floral opening (-114, -90, -60)) of petals **(A, C, D, E)**, one stage of sepal **(B)** and that of leaf **(F)** were collected in late September on a genet growing in the field. Flowers of the plant typically opened around 4 am daily, and the time was set as hour (h) 0 Day 0 for a given petal development series as shown. Leaves were sampled at 10 am along with petals **(A and C)** and sepals **(B)**, whereas petal samples **(D and E)** were taken on the same day at 4 pm from the same floral buds.

Petals and leaves are excellent model systems for ontogenetic investigations of various biological processes [15,16]. Petal's developmental stage can be easily determined through morphological characteristics, and leaves may be classified by size to ensure a repeatable sampling. To characterize organ-specific gene expressions and substance flow between organs on a genet of the common morning glory, we focused on three developmental stages of petals collected in four samples, taking one stage of sepal and another of leaf as reference samples (Figure 1). The *de novo* assemblies via Trinity [14] incorporated 83% - 89% of the raw data in the scaffold building (Table 1). The depths of the assembled transcriptomes were estimated between 25 to 38 for samples A→F (A:25.3, B:26.2, C:28.1, D:33.1, E:33.0, F:37.9). To aid sample comparisons, we lumped all contigs across the samples to generate a final assembly using TGICL [17], which yielded 90451 scaffold sequences in total (accession GALY01000000). About 70% of the scaffolds were annotated by the reference of uniref50 (ftp.ebi.ac.uk/pub/databases/uniprot/, up to April of 2012), and about half of the reads could be associated with known functions. Each sample was then mapped to the final assembly via Bowtie (Version 0.12.9) to obtain the reads distribution among the entries. Since these reconstructions contain comprehensive sequence information, they provided more details on petal development than those collected previously from microarrays [18,19].

**Table 1:** Results of Solexa/Illumina paired-end reads (75nt in length) assembled by Trinity

**Sample**	**Number of raw reads**	**Number of contig entries**	**N50**	**Reads assembled**	**% Reads usage**	Annotated (full+partial)*
						**(1e-5)**
A	19874108	50284	1454	17483903	88%	15707+22815
B	20599558	55164	1288	17812061	87%	14954+27058
C	19710038	52538	1221	17177667	87%	12816+26672
D	20406514	46799	1214	17847745	88%	11134+24364
E	23228336	48568	1336	20655630	89%	14143+22263
F	38954816	63811	1426	32331937	83%	18431+27754
Total		90451	1209			23474+39539

Each sample was handled with the default parameters in Trinity from cleaned raw data. The sample "Total" was obtained by combining all contigs of the six samples above and assembling them further via TGICL. The number for reads integrated into assemblies was calculated from the number of reads that are alignable to contigs at levels of more than 93% (>=70/75).

*Annotated in full are the contigs showing both ATG and STOP codons, while partial refers to the rest of the contigs. All comparisons were performed against the reference sequences at the e-value indicated.

We evaluated the quality of the transcriptomic assemblies by independent means. The accuracy of the final assembly was examined via cDNA cloning and sequencing of several commonly used genes such as ones encoding GAPDH2 (JN882353), SK (JQ256515), DAHPS (JQ256519), and ACTIN4 (JN882352), and of metabolic network genes such as those encoding PAL (KC794954), C4H (KC794955), 4CL (KC794953), CHS-D (AF358655), CHI (AF028238), DFR-B (U90432), F3H (U74081), F3'H (EU032626), ANS (EU032612), 3GT (EU032615), 3GGT (KC794956), MYB1 (AB232769), bHLH2 (EU032619), and WDR1(EU032621) in the flavonoid network. In all cases, the sequences amplified via RT-PCRs matched nearly perfectly to the final assembly.

**Figure 2:**
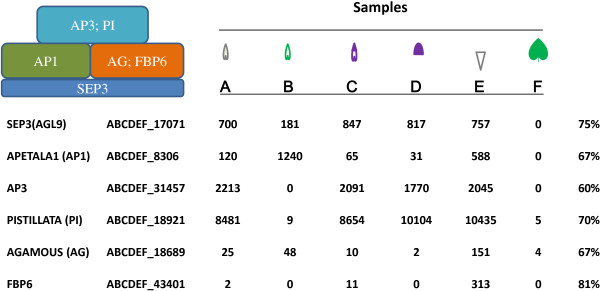
**Homolog expressions of MADs box genes across six samples.** The upper left corner shows the ABCE model. The first column lists MADs gene products in *Arabidopsis thaliana* and petunia FBP6, followed by the corresponding scaffold numbers in the final assembly. The original read level for each scaffold is shown for each of the samples (A→F). The last column displays amino acid identity between a homolog of *Ipomoea* and that of *Arabidopsis* (petunia in case of FBP6).

The precision of the transcriptomic reconstructions was assessed by transcript distributions of known organ-identity genes. As floral organ identity genes such as MADs box types are relatively well-known in *Arabidopsis*[20], expressions of their homologs in *Ipomoea* were taken as the criteria for this evaluation. These homologs largely followed the predictions of the ABC model [21] and the derived ABCE model [22]. For instance, homolog expression of the class E gene - *SEPALLATA 3* (*SEP3*) - was essentially absent in leaf, but present in floral samples in various quantities (Figure 2). The best example was found with the homolog of the class-B gene- *AP3*, which was absent in both sepal and leaf, but present in all petal samples, as previously seen with *Aquilegia AP3*-*3*[23]. Similarly, homologs of class-C genes, including *AGAMOUS* (*AG*) and FLORAL BINDING PROTEIN6 (*FBP6*), were found expressed in all tube-containing samples (A, C, and E), due to a partial fusion of *Ipomoea* filaments to the floral tube. The restricted distributions of *SEP3* and *AP3* indicate a high precision associated with our transcriptomic reconstructions (Figure 2).

To quantitatively compare the assemblies, we adapted a normalized procedure of Robinson and Oshlack [24]. In comparison to previous data handlings such as reads per kilobase per million mapped (RPKM) and quantile normalization [25], the normalization considers not only the relationship between read number and gene length but also sample characters. It does so by taking the common set of genes across samples as the base for the normalization, effectively excluding transcripts selectively expressed in each organ and the associated bias (Additional file 1). To make sure that the normalized gene expressions still reflected sampled tissues, we took independent samples of petals at the same stage (sample D) on the same genet, and collected data on transcript estimates of ten genes on the anthocyanin pathway through the conventional real-time quantitative PCRs. We observed a significant correlation (Pearson correlation coefficient = 0.77, P < 0.05, *t*-test, n = 10) between the two sets of data (Additional file 2), and conclude that the normalization procedure did not cause significant shifts of patterns among samples.

We started the data analysis by classifying each assembly into three groups - transcripts from nuclear, mitochondrial, and chloroplast genomes to reveal features of subcellular genome usages. Since mitochondrial and chloroplast genomes are relatively small and have been known for many species, this treatment helps to make a better use of the resources available in general. By taking the known chloroplastic genome of the common morning glory [26] and the mitochondrial genome from a closely related species *Nicotiana tobacum*[27] as references, we mapped each assembly to these genomes to obtain lists of chloroplastic and mitochondrial genes that were longer than 200-bp (the sequencing library length). The set of nuclear genes was obtained by excluding the organelle genomes from each assembly (Table 2). This classification permitted explicit comparisons of the six samples, leading to an effective analysis of organ-specific gene usage in each subcellular genome.

**Table 2:** **Comparisons of subcellular classifications in scaffold number and expression level (normalized in parentheses) among leaf, sepal, and corolla (=limb + tube) transcriptomes of *Ipomoea purpurea***

**Genome**	**Samples**					
	**A**	**B**	**C**	**D**	**E**	**F**
	**Corolla**	**Sepal**	**Corolla**	**Limb**	**Tube**	**Leaf**
	**(-114 hr)**		**(-90 hr)**	**(-60 hr)**	**(-60 hr)**	
Chloroplast	93	117	102	91	93	151
	(337)	(779)	(441)	(580)	(471)	(2126)
Mitochondrion	60	48	72	58	54	61
	(354)	(624)	(422)	(823)	(548)	(481)
Nucleus	64564	65373	63660	60938	63394	73187
	(238)	(217)	(238)	(365)	(407)	(406)

### Gene usages of chloroplastic, mitochondrial, and nuclear genomes

**Figure 3:**
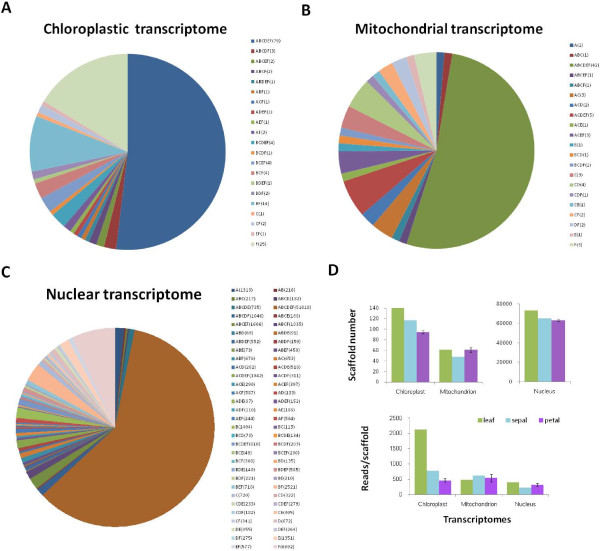
**Classification of *Ipomoea purpurea *transcriptomes. (A-C)** The sample distributions of scaffolds for three subcellular transcriptomes. The scaffold number for each sample combination is indicated in the parentheses. **(D)** Gene expressions of the three subcellular genomes were compared among organs in scaffold numbers and normalized expression levels. The standard errors were shown in bars based on four petal samples.

The organs were compared in the sequence, scaffold number, and transcript abundance of the subcellular genomes. A scaffold was referred to as a transcript here after being examined to contain such a sequence. We first examined the common features shared by the organs. For the 113 annotated units of the known *Ipomoea* chloroplast genome, 94 were detected bearing transcript expressions in our samples, and 52% of the expressions were shared among petal, sepal, and leaf (Figure 3A; Additional file 3). Some scaffolds still maintained prokaryotic characters. A chloroplast scaffold (ABCDEF_16731), for instance, has multiple units including ribosomal RNAs and two tRNAs for isoleucine and alanine, a configuration previously observed in cyanobacteria [28]. This scaffold reached the highest expression level (51617 raw reads) in the leaf sample. Similarly, for the 169 annotated units of the *Nicotiana* mitochondrial genome, 42 homologs were detected having expressions in our samples (Additional file 4), of which 53% were commonly found among the organs (Figure 3B). While the total homolog number might vary with inter-specific mitochondrial mapping, the percentage of shared transcripts could still effectively expose the genome usage pattern among organs since all organs shared the same mitochondrial genome, being on the same genet. A highly expressed scaffold (ABCDEF_41509) of mitochondrial genome also exhibited a cistron-like structure, including ribosomal RNAs and tRNA of phenylalanine. Little can be found in the literatures about this gene arrangement. Meanwhile, the highest similarity of gene expressions among organs (60%) was found within the nuclear genome (Figure 3C), suggesting the importance of nuclear genome in basic cellular functions. These commonly expressed genes were further inspected in transcript abundance. For both chloroplasts and mitochondria, the highest correlation in transcript abundance level was found between colored limb and green leaf (Pearson correlation coefficient r = 0.95, P < 0.001, *t*-test, n = 70 for chloroplasts; r = 0.99, P < 0.001, *t*-test, n = 35 for mitochondria). Many of these transcripts participate in basic biological processes such as protein synthesis. For nucleus, however, the highest correlation in transcript abundance was observed between sepal and leaf (r =0.86, P < 0.001, *z*-test, n = 51018), suggesting their similarity in nuclear expression.

We found that organ-specific gene expressions made up less than 50% of the total transcripts for each of the subcellular genomes. Relatively speaking, leaf expressed 59% more chloroplastic genes and 16% more nuclear genes than the average of petals, presumably to support cellular activities of the whole plant; petal at a later developmental stage consumed a high volume of mitochondrial transcripts to assist its function; sepal engaged the least mitochondrial genes, but shared features with both petals and leaves in other expression categories (Table 2). Significantly, the nuclear transcriptome generated 518 petal-specific scaffolds, implying a major role of nuclear genes in petal diversification (Figure 3C). The chloroplast genome showed the most striking diversification in both transcribed gene number and expression level (Figure 3D).

When regarding petal and sepal as representative parts of flower, we observed a significantly different nuclear gene usage between leaf and flower (Wilcoxon rank test, P = 0.03). The same significant differences were also seen for chloroplastic gene usage and the average transcript abundance between leaf and flower. Furthermore, a sequential reduction of transcribed gene number was found in the order of leaf → sepal → petal for both nuclear and chloroplastic genomes. This pattern is noteworthy since it appears to connect to one in *Arabidopsis*, where pollen exhibited a reduction of about 1/3 of commonly transcribed genes relative to vegetative organs [29]. Also during senescence, the transcriptomic profile of *Arabidopsis* silique is more similar to that of its petal than that of the leaf [30]. Whether or not this trend of a reduced genomic usage toward the center of a flower is ubiquitous requires additional data. The kind of information has significant bearings. One is on deciphering evolutionary histories of the organs. Floral developmental program has been considered to share a deep homology in angiosperms [31], however, the emergence of petals in land plants is still an enigma [32] despite of examinations from multiple angles [33,34]. The similarities found here between petal and leaf in chloroplast gene usage and expression patterns of common transcripts suggests a deep connection between petal and leaf.

Our data further shows that petals are partially autotrophic and costly to maintain. During petal development, two photosystem I genes (ABCDEF_39040 encoding *psaI* and ABCDEF_49598 encoding *psaA*) expressed only temporarily after the petal's exposure to light, coinciding with the temporary greening of petals prior to blooming (Figure 1). During the petal pigmentation period as seen in samples A, C, and D, ribosomal genes (e.g., ABCDEF_17426, ABCDEF_41509) of the mitochondrial genome enhanced their expressions, and ATP synthase transcripts (ABCDEF_38143) increased nearly three folds. These patterns indicate that gene usages are dynamic in subcellular genomes, which may vary by genome type, organ type, the developmental stage of an organ, and the environment.

### Cellular dynamics depicted by transcript abundance levels

The known networks depicted by the Kyoto encyclopedia of genes and genomes (KEGG) database provide a basic framework for reconstructing molecular pathways. To link gene expressions to metabolites, we mapped all annotated entries of the final assembly to KEGG (Additional file 5). We made two assumptions while making our inferences. One is a positive correlation between transcript abundance and protein expression level; the other is one between protein abundance and enzyme activity. The first correlation has gained support from tissue or cell-specific studies [7,8,35]. The second one can be derived from Michaelis-Menten kinetics, which suggests that richness of an enzyme (E) is positively correlated with the enzyme's activity until the substrate (S) is exhausted in forming ES. Both types of correlation may hold broadly.

### Gene expression patterns of petal cell division

**Figure 4:**
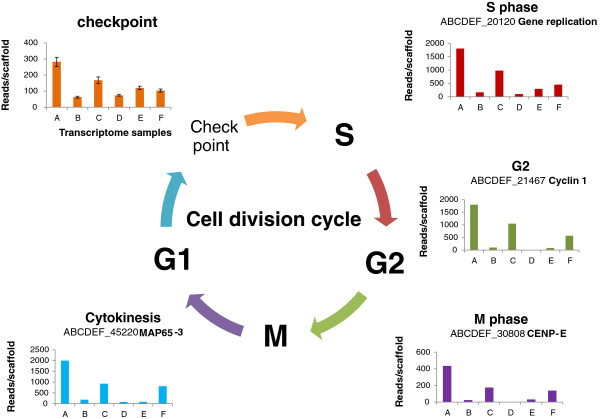
**Cellular division patterns among organs.** Patterns of cell division cycle were exemplified by homologs of known genes among samples A→F. For the case of checkpoint, multiple homologs were found, and the associated bars simply represent the transcript level variations among these homologs. The colors of bar graphs march roughly those of cell-cycle stages.

We depicted patterns of cell division using the homolog expressions of genes known to participate in cell cycle (Figure 4A). Specifically, high expression of cyclin-A2 is known to signal cell transition from G2 to M phase [36] and the transcripts get rapidly destroyed after cells enter the M phase [37]. Its homolog (ABCDEF_21549) here displayed a declining pattern along the developing petal series, suggesting reduced cells at metaphase with petal’s enlargement; consistently, B-type cyclin transcripts (ABCDEF_21467) also decreased, complementing the same G2-M phase transition [38]. The same pattern held for a homolog (ABCDEF_45220) of a gene encoding microtubule-associated protein (MAP65-3) with a known role in cell plate formation [39]. Along with the cell division features, we further examined the relative ratios of the transcripts participating in M and those in cytokinesis, and found that these ratios decreased from sample A to sample C and again from sample C to sample D, implying leaf-kind endoreplication in young petals. In addition, comparisons between samples D (limb) and E [18] of these cell cycle transcripts indicate enhanced transcription activities in tube (Figure 4), suggesting a basiplastic pattern of cell cycling, as seen in leaf [40]. These data imply that the petals are similar to developing leaves in cell formation, and young petals of sample C are closer to young leaves of sample F than to other samples taken here. Such a deep homology can be hardly diagnosed by previous comparisons [41], but complements with the lack of evidence for stamen origin of petal [23]. In contrast, sepal has the least number of dividing cells, agreeing with its mature size at the time of sampling. The cell division patterns, in additional to the common features of subcellular genome usages shared between leaf and petal, strongly favor a leaf origin of petal. This hypothesis is proposed here as it deserves close examinations in future.

### Active RNA synthesis and translation in petals

**Figure 5:**
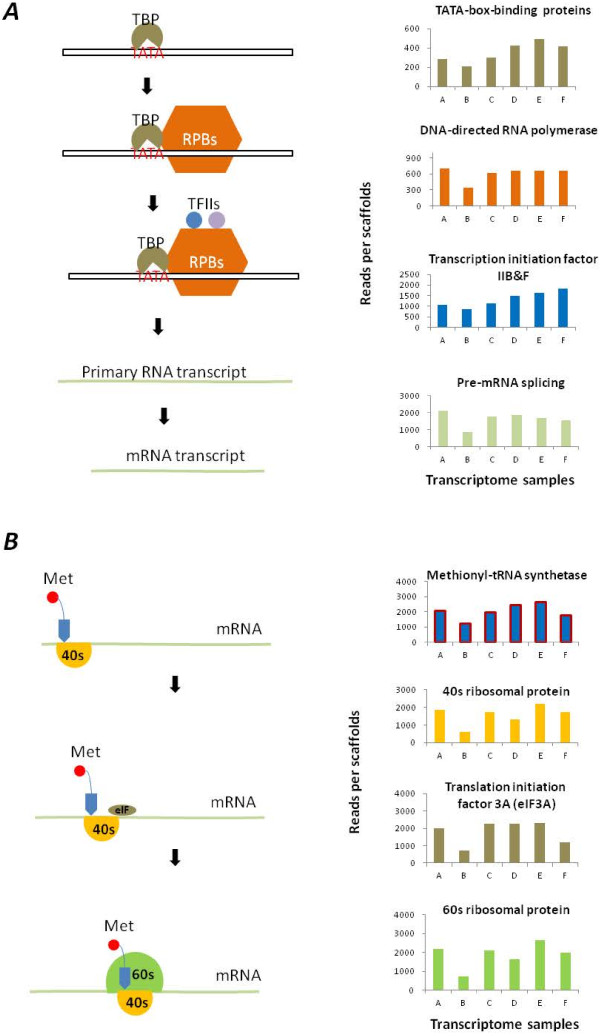
**Comparisons of RNA and protein syntheses. (A)** Patterns of RNA synthesis were decomposed into homolog expression levels of the major components including the TATA-binding protein (TBP), the DNA-directed RNA polymerase subunit II (RPB), the transcription initiation factor II (TFII), and pre-RNA splicing factors. **(B)** Patterns of protein synthesis were characterized by the homolog transcript level of the methionyl-tRNA synthase (Met) and those of a translation initiation factor (eIF3A) and ribosomal RNAs (40s & 60s). The numbers of reads per scaffold have been normalized.

Since petals appear to have distinctive patterns in genomic usages, we wanted to know whether their basic transcription and translation processes would differ from those in other organs. Homolog expressions of the components known to be part of the basal apparatus for transcription were examined to compare RNA synthesis in samples. They included topoisomerase I (ABCDEF_4627), TATA-box-binding proteins, and various components involved in the synthesis of RNAs (Figure 5A). Petals showed slightly more expressed homologs for transcription initiation factors (TFIIB and TFIIF) toward a later development. The translation process followed a pattern similar to those of TFIIB and TFIIF genes, with petal samples (with the exception of the sample D) showing higher transcripts for all the ribosome subunits and the translation initiation factor than leaf and sepal samples (Figure 5B). This phenomenon could be associated with the high-volume synthesis of petal-specific metabolites [42], although the likelihood cannot be ruled out that different quantities of the components for the basal apparatus might be needed for organ-specific transcription and translation.

### Epigenetic events differ between organs

The current understanding of epigenetic processes permits preliminary comparisons of some components across organs. These components diverged at both DNA and protein levels, suggesting that epigenetic processes are likely to be among the key events for organ differentiation (Additional file 6). In petal, we observed a highly expressed homolog (ABCDEF_38770) of *ARGONAUTE 1* (*AGO1*), along with a Dicer homolog (ABCDEF_40338). AGO1 participates in single-stranded RNA cleavage via microRNA [43], while Dicer is known to cut double-stranded RNAs to influence DNA methylation status [44]. Their expression patterns suggest different dynamics of small RNA processes between organs. In a similar pattern, genes for histone deacetylases (e.g., ABCDEF_42471) were more actively transcribed in petals while protein phosphotase 2C gene (ABCDEF_3211) was expressed more in leaves and sepals. For the colored limb (Figure 1), transcripts (ABCDEF_4131) highly resembling a maize AC transposase gene [45] were about 4–8 folds higher than those in leaves and sepals. It appears that different epigenetic strategies have been taken between organs.

### Connecting metabolic pathways to infer substance flow

As metabolic processes are the basis for the substance flow among organs, they are compared among organs and organ developmental stages to extract information. We organized the annotated final transcriptome by the classification of KEGG (Additional file 5) and compared the file against known network topologies to establish data-supported networks. As indicated above, over 50% of transcriptomic components are shared among organs, and many of the known metabolic networks can be compared at a transcript level across samples. To make inferences on substance flow, we mapped the *in vivo* transcript accumulation patterns across the six samples along metabolic networks using sign and color. Examples are given here focusing on glycolysis, amino acid pathways, and synthetic pathways to flavonoids and terpenoids, since our experimental design particularly targets petal pigmentation process (Figure 1). Anthocyanins are known to be the major pigments in the petal of *Ipomoea*[46]. Our initial concern was to know how the anthocyanin pathway had been related to other metabolic processes *in vivo*.

### Reconstruction of metabolic networks via organ transcriptomes

**Figure 6:**
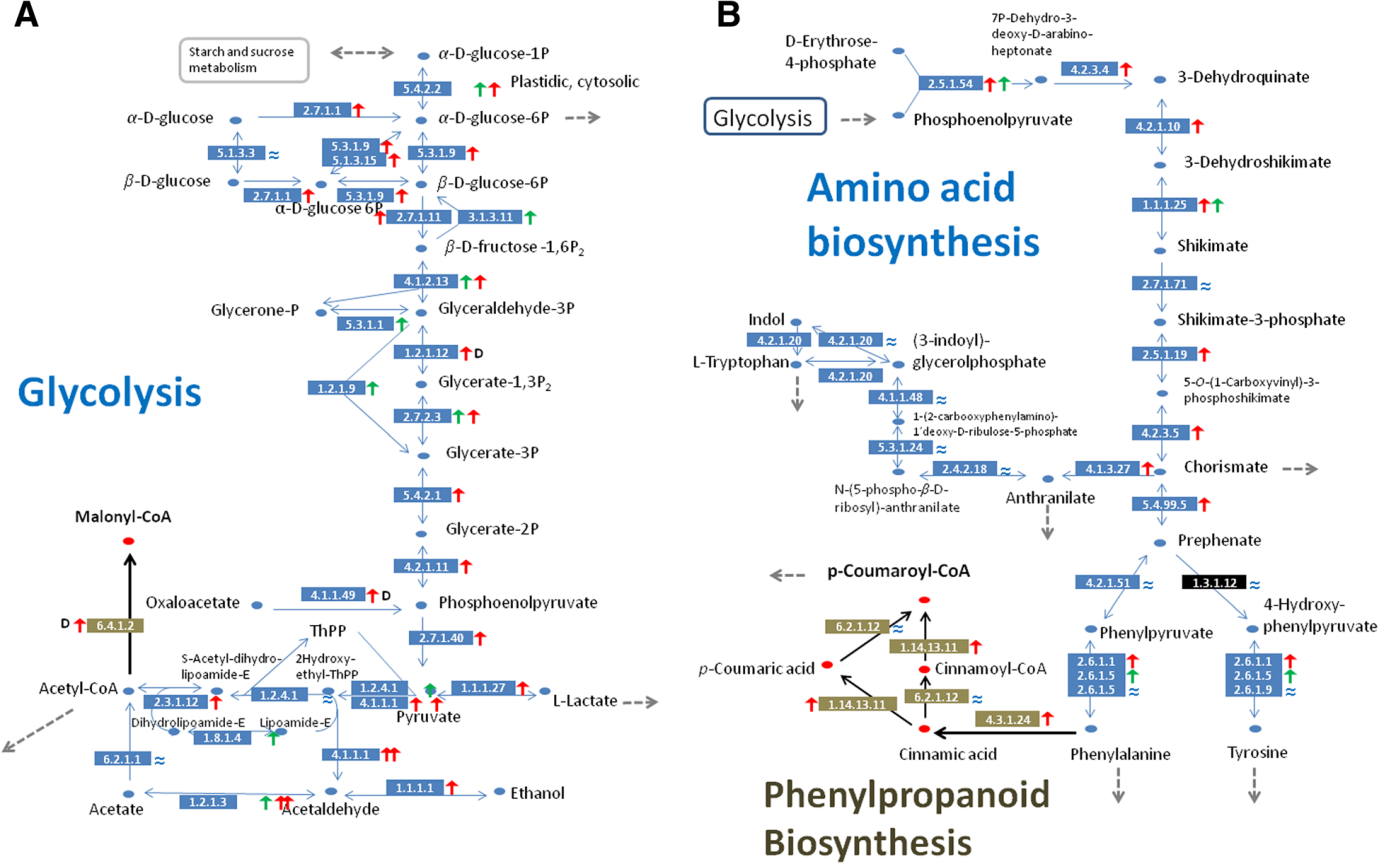
**Patterns of gene expressions on primary metabolic networks. (A)** Glycolysis was reconstructed based on transcripts of enzymes (in EC numbers) mapped to KEGG. Variation among the samples was indicated by stunt arrows, with up-pointing ones showing increasing gene expression levels (> 2 fold). Red arrows refer to the petal samples relative to leaf and green ones referring to leaf gene expression relative to the mean of petals. Green and red arrows in parallel are for cases where alternative forms of transcripts were found for leaf and flower, respectively. Approximation sign (≈) shows within 2-fold variation among the samples. Blue thin arrows display known functions of enzymes in glycolysis and the black long arrow shows the bridge to the biosynthesis of malonyl-CoA. **(B)** Amino acid syntheses were similarly rebuilt to show gene expression patterns leading to the synthesis of *p*-coumaroyl CoA in phenylpropanoid synthesis (partial). The enzyme in black rectangle refers to the case where the transcript was identified via individual searching of the *Arabidopsis* homolog (AEE29352) and absent in the initial annotation. Enzymes in brown rectangles are transcripts associated with phenylpropanoid synthesis.

We reconstructed glycolysis (KEGG #00010) for all samples, and compared the transcript abundance levels at each catalytic step among the samples along the reconstruction. The transcript distributions indicate an active substance flow from D-glucose to acetyl-CoA in petals (Figure 6A). Along the portions of the network indicated by red arrows, the transcript level of every enzyme showed an increasing pattern along the petal development when compared to its counterpart in leaf. Following the synthesis of glyceraldehyde 3-phosphate, the network took a slightly different path in leaves, where different forms of enzymes were involved. To know the details on these enzymes, we examined all entries under a GO process (0006006) for glucose metabolism, and found that petals engaged more cytoplasmic glyceraldehyde-3- phosphate dehydrogenase (G3PDH, EC 1.2.1.12, ABCDEF_27515) while leaves and sepals were dependent more on chloroplastic G3PDH (EC 1.2.1.9, ABCDEF_28550). In addition to glucose, the network in color limb cells also actively catalyzed oxaloacetate (a product largely from citric acid cycle) into phosphoenolpyruvate to supplement the substance flow.

To seek the connection between glycolysis and other networks, we extended the network reconstruction around glycolysis, and recruited acetyl-CoA carboxylase (EC 6.4.1.2). This enzyme requires one ATP for synthesizing one malonyl-CoA from one acetyl-CoA, while malonyl-CoA is one of the precursors for anthocyanins. We found that the transcripts of acetyl-CoA carboxylase increased about 4-fold between samples A and D. As a result, an enhanced substance flow was channeled into production of malonyl-CoA during petal pigmentation via this linking enzyme between glycolysis and flavonoid synthesis (Figure 6A). This also answered to some extent why much energy was consumed in petals.

Since anthocyanin pigments are known to be from three malonyl-CoA and one 4-coumaroyl-CoA [47] by the catalytic reaction of chalcone synthase (CHS), we further explored the *in vivo* source of 4-coumaroyl-CoA in petal. The mapping of amino acid biosynthesis (Figure 6B) suggested that it came directly from phenylalanine metabolism (KEGG #00360) via highly expressed transcripts for phenylalanine ammonia-lyase (4.3.1.24). The enzyme had two types of isoforms in samples. Both increased along with petal development. In comparisons to their counterparts in leaf and sepal, the shorter one increased about ten folds in tube, and the longer ones boosted their levels by 2–4 folds in colored limbs. Since the floral tube of *Ipomoea* fuses with the anther filaments, it is difficult to make a judgment on the significance of these alternative transcripts. For our purpose of inferring substance flow, these expression patterns suggested a continuous substance flow from the branch of phenylalanine synthesis to the *in vivo* production of 4-coumaroyl-CoA during development of limb pigmentation, and the substance flow could be stronger in the floral tube.

**Figure 7:**
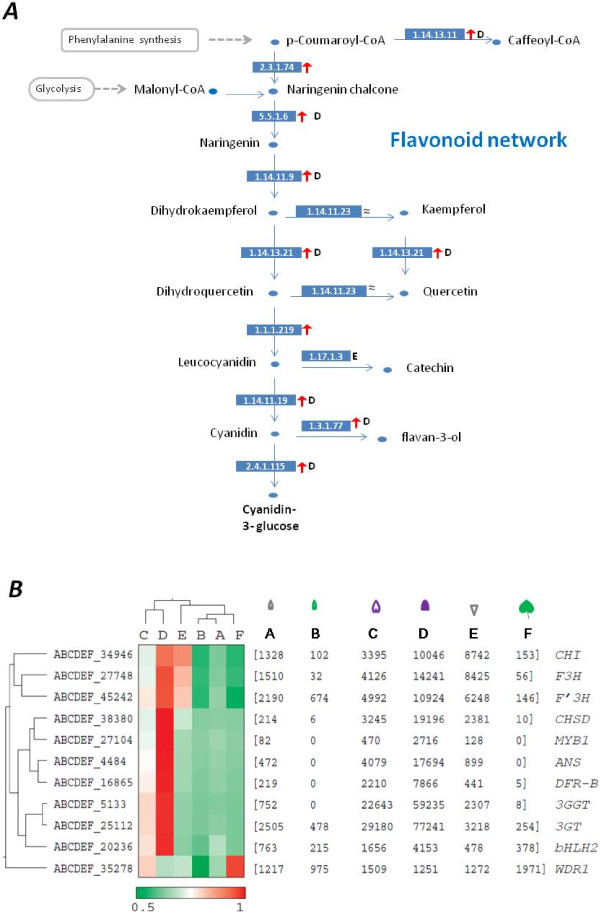
**Patterns of gene expressions on the flavonoid network. (A)** Sample comparisons on the flavonoid network. All enzymes are expressed as EC numbers in blue rectangles. Green and red arrows refer to enhanced transcript abundances (>2 fold) in leaf and flower, respectively, with each other as reference. The letter in bold following an arrow indicates the sample with the highest expression level. The pigment products are also shown in bold. **(B)** A close-up of expression patterns of known genes on the anthocyanin pathway with normalized expression levels shown in the parentheses. The horizontal cladogram indicates gene expression similarities across samples and the vertical cladogram suggests similarities of samples across genes, as generated by Cluto (version 2.0) using the matrix of Pearson correlation coefficient in a scale of 0.5 to 1.0. Alleles enlisted include *CHS-D_mex9* (AF358655), *CHI_fl1* (AF028238), *F3H_1* (U74081), *F3'H_blue* (EU032626), *DFR-B_fl1* (U90432), *ANS_c* (EU032612), *3GT_b* (EU032615), *3GGT_a* (KC794956), *Ipmyb1_a* (AB232769), *bHLH2_bh2b* (EU032619), and *WDR1_Ipwd1a* (AB232777).

The flavonoid metabolic network (KEGG #00941) was subsequently mapped and modified from data (Figure 7A). The reconstruction suggests that all enzymes on the network were up-regulated relative to their counterparts in leaf and sepal, which is consistent with the microarray results of petunia petals [42]. To reveal organ-specific expression patterns on the anthocyanin pathway, we performed a clustering analysis by Cluto (version 2.1.1) on the transcript abundance levels among the six samples. The data indicates that the lack of anthocyanin synthesis in *Ipomoea* leaf and sepal is due to lack of the expression of regulatory gene *MYB1* at organ level, which led to reduced expressions of key enzyme genes on the pathway including *CHS-D*, *DFR-B*, *ANS*, and *3GGT* in these organs (Figure 7B). Naturally, these expressed genes constitute part of petal-specific transcripts that define the function of petal. In contrast, two collaborating regulators, bHLH2 [48] and WDR1 [49], were found present more or less in all three organs compared here.

### Inferring substance flow between organs

The identification of the connections between glycolysis and the anthocyanin pathway was significant as it allowed deciphering of extended substance flow involving the supply of glucose in petal. By reconstructing the network involving starch and sucrose (KEGG #00500), we noticed that relative to leaf, petal had a 4-fold higher transcript level for alpha-glucosidase (EC3.2.1.20, ABCDEF_11272) in samples D&E and 18-fold higher transcripts for beta-glucosidase (EC3.2.1.21, ABCDEF_52515) in sample E alone. These enzymes could take different forms of glucoside to generate glucose to meet the high demand in petals. The final anthocyanin molecule in *Ipomoea* can be added with five glucoses to maintain its stability in vacuole [46,50]. Moreover, sucrose from leaf could replenish glucose in petal when the demand of glucose in petal outpaced the local supply. The evidence for this scenario came from the increasing patterns (2- to 4-fold) of homolog transcripts (ABCDEF_25171, ABCDEF_41696) of two sucrose transporter genes (*SUC1* and *SUC2*) in the petal developmental series. The homolog expression of beta-fructofuranosidase (EC3.2.1.26, ABCDEF_35900) was most informative, as it increased over three magnitudes in samples D&E versus the leaf sample. This enzyme holds a 64% identity to the vacuolar form of acid invertase in bean (*Vicia faba*), which is known to convert sucrose into glucose and fructofuranose. It is possible that leaf-produced sucrose can be transported via the vascular systems in floral tube to satisfy the need of glucose during petal pigmentation process. Although the concrete molecular path connecting different tissues still requires characterizations, externally supplied sucrose have been known to enhance anthocyanin accumulation in *Eustoma*[51] and *Arabidopsis*[52,53]. The *in vivo* substance flow detected in the petal series indicates how floral color can be altered by fluctuation of upstream metabolites.

### Reconstructing signaling networks

**Figure 8:**
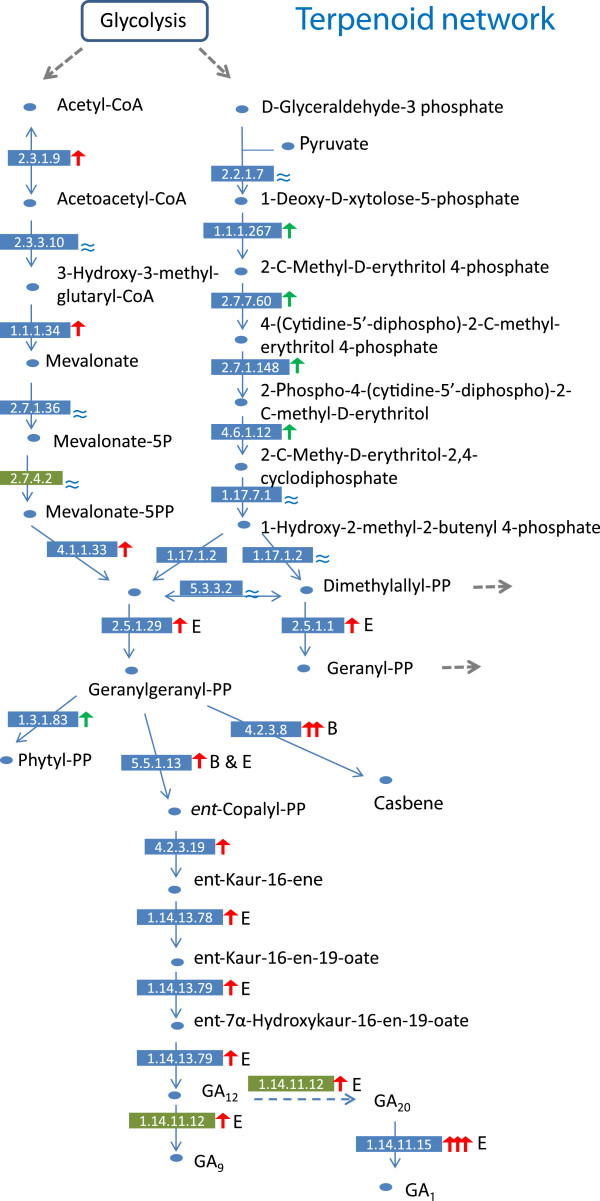
**Patterns of gene expressions of terpenoid biosynthesis.** The network is compared among samples to indicate at least 2-fold changes of gene expressions (petals in red arrow and leaves in green arrow) during the petal developmental stages, with the highest expression level shown by the sample letter and variation less than 2-fold shown as ≈. The increases of more than 10 folds and 100 folds are indicated by double and triple up-pointing arrows, respectively. Dotted grey arrows show connections to other networks, and a dotted blue one indicates a suspected reaction. The major products are in bold. The enzymes are shown in blue rectangles with the EC numbers provided by the annotated final assembly, and ones in green rectangles were detected via individual blast searches.

Because plant hormones may play significant roles in floral development, we inspected how gibberellins (GAs) were manufactured in different organs. Based on the known metabolic pathway of terpenoid backbone synthesis (KEGG#00900), we mapped the patterns of transcript levels at each step to reconstruct the network among organs. The precursors (acetyl-CoA and D-glyceraldehyde-3-phosphate) of terpenoid backbone synthesis could be traced back to glycolysis (Figure 8). We followed the branch from geranylgeranyl-diphosphate (geranylgeranyl-PP) to different GAs. Floral tube was found to be the hot spot for the synthesis of cytosol gibberellin (GA_9_ & GA_20_), since the gibberellin 3*β*-hydroxylase (ABCDEF_75035) transcripts of sample E were over 100-fold higher than other samples (Figure 8), suggesting that geranylgeranyl-PP was likely enriched in the floral tube. This GA-production hotspot is confined to filament rather than petal tissue *per se* since the enzyme leading to GA_1_ production is virtually absent in both limb and sepal, but highly expressed in tube with developing filaments. This expression pattern is also consistent with GA effect on *Arabidopsis* filament growth [54] and on development of androecium in general [55]. GA_9_ and GA_20_ may be further metabolized into GA_3_ and GA_1_ in cytosol, respectively, to influence relevant developmental processes [56].

GA synthesis in sepals, however, appeared to follow a different path of substance flow---the transcript level of 5-epi-aristolochene synthase (EC 4.2.3.8, ABCDEF_11886, 59% identity to NaEAS37) increased a 24-fold relative to petals and about three folds relative to leaves (Figure 8). This may cause a high accumulation of a capsidiol-like phytoalexin in sepal. As casbene can reduce fungal infection [57], a likely high accumulation in sepal may provide a general mechanism for the protective role of sepal during developments of floral buds and young fruits. The phenomenon certainly deserves more close examinations.

## Conclusion

Since transcriptomic reconstruction may provide rich information on cellular activities and substance flow in organs (or cells in cases of microscopic samples), it should be an integral part of biological investigations in future. We provide here the logic and ways of extracting transcriptome information. Cellular activities may be largely reconstructed from patterns of genomic usages and transcript abundance among samples under an object-oriented experimental design. Inferences may be drawn from known topology of networks and their validated components. Our analysis shows that while leaf and sepal are both photosynthetic, leaf is an essential organ for energy capturing and primary metabolism, as more genes were transcribed in higher quantities than those in sepal. Though photosynthetic and possibly autotropic to some degree, sepal has been specialized in other functions such as protection by accumulating organ-specific secondary compounds such as phytoalexins. Petal pigmentation process can be visualized step-by-step from glycolysis to anthocyanin pathway by linking relevant KEGG metabolic topologies via known biochemical reactions. The continuity in substance flow is therefore clearly visible between the primary and secondary metabolic networks. A less continuous image, however, also emerged in our analysis, showing that sucrose could be transported from leaf to petal to replenish glucose during petal pigmentation process. While the missing links between molecular systems still depend on future laboratory discoveries, the integration of NGS technique, bioinformatic analysis, and an appropriate experimental design may certainly help to orient the search directions to more likely domains.

## Methods

### Sampling scheme and data collection

Six samples of *Ipomoea purpurea* were taken on 25th September 2009 from a healthy individual of a selfing line (III6Da) grown naturally at the Botanical Garden of the Institute of Botany, CAS, at Beijing. Young leaves, sepals, and petals (Figure 1) were frozen immediately in liquid nitrogen and the whole RNAs were subsequently extracted using Trizol reagent (Life Technologies). The quality of the extracted RNAs was examined with Agilent 2100 Bioanalyzer (Agilent Technologies) and the samples were processed following the sample preparation guide for mRNA sequencing (Illumina 2009). The preparation involved purification of mRNAs by poly-T oligos attached to magnetic beads, and cDNA synthesis in two strands. The cDNAs were added with A bases at 3' ends and ligated with adapters for further PCR amplifications. The amplified cDNAs were sheared into 200 bp in length to make the sequencing library. The processed samples each in 10 ng were run in parallel at the platform Hiseq2000 of Beijing Genomics Institute (BGI). Paired-end reads of 75-nt were collected up to 1.2 giga bases (Gb) for each sample. Low quality reads were excluded from the raw data, and clean data sets (>1 Gb) were subject to the assembly pipeline described below.

### Transcriptome reconstruction and annotation

The cleaned data of each sample was assembled using Trinity of the latest version (trinityrnaseq_r2012-06-08.tgz). The command line followed: Trinity.pl --seqType fa --JM 40G --left A.Left --right A.Right --CPU 8. Details on the parameters may be found in the associated manual (http://trinityrnaseq.sourceforge.net/#sample_data). The resulting assemblies were subsequently labeled as A, B, C, D, E, and F (Table 1), corresponding to the samples of Figure 1. The six samples were further combined to produce a final assembly using TGICL (TGICL-2.0.tar.gz) under the parameters: -p 95 -l 50 -v 6 -O '-h 3 -k 0 -o 50 -p 95'.

We annotated the final scaffolds of the combined data to a reference database containing a total of 2961141 uniref50 sequences (ftp.ebi.ac.uk/pub/databases/uniprot/, January 2012) by blastx under the parameters of -e 1e-5 –b 10. The resulted annotations were then re-organized by our local Perl scripts (Additional file 7) to extract information according to their GO (http://www.geneontology.org) and KEGG classifications (http://www.genome.jp/kegg/). The assembly of each sample was mapped back to the final assembly via Bowtie [58] to obtain the reads distribution among the entries. When the read number for an entry was less than two, we considered the result fortuitous, and took the expression as zero.

### Normalization of multiple samples

Following the original notations of the TMM (trimmed mean of log (base 2) expression ratio (M value)) method [24], we applied the formula below to compute the normalization factor R:

(1) $$R={\mathrm{TMM}}_{k}^{r}$$

$$\log\left({\mathrm{TMM}}_{k}^{r}\right)=\left(\sum_{g\in G^{*}}w_{\mathrm{gk}}^{r}M_{\mathrm{gk}}^{r}\right)/\sum_{g\in G^{*}}w_{\mathrm{gk}}^{r}$$

Mgkr=logYgkNk/logYgrNr

$$w_{\mathrm{gk}}^{r}=\frac{N_{k}-Y_{\mathrm{gk}}}{N_{k}Y_{\mathrm{gk}}}+\frac{N_{r}-Y_{\mathrm{gr}}}{N_{r}Y_{\mathrm{gr}}}\approx \frac{1}{Y_{\mathrm{gk}}}+\frac{1}{Y_{\mathrm{gr}}}$$

Here, G* is a trimmed set (defined below) of genes with log-transformed abundance levels, and g is any gene in the set. The abundance levels of gene g in samples r and k, is represented by Y_gk_ and Y_gr_ respectively. The total abundance levels of all genes in samples r and k are N_k_ and N_r,_ respectively. The abundance level for each scaffold g in sample k (Y_gk_) was estimated by dividing the number of paired-end reads for the scaffold by the length of the scaffold and the total paired-end read number for the sample (Additional file 1). For comparison of n-samples, G* was obtained in the following steps. Genes expressed in all n samples were chosen to constitute G, and Y_g_ was computed as:

$$Y_{g}=\left(\sum_{k=1}^{n}Y_{\mathrm{gk}}\right)/n,\mathrm{where}\;g\in G,Y_{\mathrm{gk}}\neq 0$$

$$N=\sum_{g\in G}Y_{g}$$

Y_g_(g ∈ G) was regarded as the reference sample. For each scaffold (g) in the reference sample, we calculated its M_g_ and A_g_ for the corresponding gene in sample k:

Y_g_(g ∈ G) was regarded as the reference sample. For each scaffold (g) in the reference sample, we calculated its M_g_ and A_g_ for the corresponding gene in sample k:

$$\begin{array}{l} M_{g}=\log\frac{Y_{\mathrm{gk}}/N_{k}}{Y_{g}/N}\\ A_{g}=\frac{1}{2}\log\left[\left(Y_{\mathrm{gk}}/N_{k}\right)\left(Y_{g}/N\right)\right] \end{array}$$

We listed the maximum M_g_ (M _max_), the minimum M_g_ (M _min_), the maximum A_g_ (A _max_), and the minimum A_g_ (A _min_). If , $M_{\mathrm{lower}}<\frac{M_{g}-M_{\min}}{M_{\max}-M_{\min}}<M_{\mathrm{upper}}$, and $A_{\mathrm{lower}}<\frac{A_{g}-A_{\min}}{A_{\max}-A_{\min}}<A_{\mathrm{upper}},$ then g was kept. These chosen genes formed G* (in our calculations: M_lower_ = 0.3, M_upper_ = 1 - 0.3; A_lower_ = 0.2, A_upper_ = 1 - 0.2). From G* and the abundance levels of gene g in each sample (including the reference sample), we obtained the normalization factor R of each sample relative to the reference sample using the equation (1) above.

### Chloroplast & mitochondrial mapping

The mapping of scaffolds of each sample to the chloroplast genome was performed by querying the known genome of the common morning glory using blastn under 1e-5 and parameters of identity ≥95%, and length ≥ 80%. For the mitochondrial genome, a similar query was made under 1e-5 and identity ≥ 80%, and length ≥ 50% against *Nicotiana* mitochondrial genome. The mapping results were then examined to exclude misplacements based on the blast results. We developed a series of scripts to partition and organize data files to classify subcellular genomic expressions, and to sort out the transcript distribution among samples (Additional file 8), which allowed later charting of data via Excel.

### Verifications by sequencing and real-time qPCR

Petal cDNAs were obtained as previously mentioned. Primers were designed for the coding regions of target genes (Additional file 9; Additional file 3 of [59]) based on the final NGS data assembly, and applied to RT-PCR reactions using a standard protocol (Life Technologies). The amplified fragments were cloned and taken as the templates in sequencing reactions with big dye (Life Technologies). Samples for real-time qPCRs were similarly prepared and followed a previous protocol [59]. The results were expressed as numbers of transcript copies per pg cDNA (Additional file 9).

### Mapping sample patterns on molecular networks

All scaffolds were screened for connections to the known pathways in the KEGG database (Additional file 6). For each KEGG pathway, expressions of its components were first labeled to indicate the presences in the final assembly. When a specific pathway was in focus, the known topology was scrutinized at each component for its expression pattern among samples. Besides annotation from uniprot, the identity of a scaffold involved in the pathway was further verified via blast searches to NCBI databases (http://www.ncbi.nih.gov/) and its function cited by the enzyme nomenclature (http://www.chem.qmul.ac.uk/) and literatures. The close examinations allowed establishments of species-specific pathways with all of the components supported by the transcriptomic data. Organ-specific expressions were based on normalized scaffold levels and presented by labeling each component with a sign(s) representative of different expression patterns among organs. When a scaffold abundance level varied more than 2-fold between samples, we tentatively marked it as changing significantly. Assuming that a transcript level was reflective of the protein level to a significant degree, we were able to detect the direction of metabolic flux using criteria of the catalytic features of the enzymes and the expression patterns between the target transcriptomes (A, C, D, E) and reference ones (B, F) marked on the pathways.

### Statistical analysis

Pearson correlation coefficients were computed for gene expression data, and a standard *t*-test was applied to the correlation coefficients. A non-parametric Wilcoxon rank test was conducted for comparing the mean of a sample in a small size to a specific value. Pearson correlation coefficients were also calculated between expression levels of scaffolds across samples, or between samples across scaffolds, which allowed clustering analysis via the agglomerative method implemented in Cluto (http://glaros.dtc.umn.edu/gkhome/fetch/sw/cluto/cluto-2.1.1.tar.gz) using maxtf scaling as our row model.

### Availability of supporting data

All cleaned short reads have been submitted to GenBank (http://www.ncbi.nih.gov/) under the SRA accession number SRP015900. The final assembly of the sequences has also been submitted under the TSA accession number GALY01000000. Other supporting data are available in the additional files online.

## Competing interests

The authors declare that they have no competing interests.

## Authors’ contributions

SG assembled the NGS data, annotated and mapped the final assembly, and wrote the Perl scripts. YL designed the study, collected tissue samples, and drafted the manuscript. Both have contributed to the data analysis and writing of the manuscript. Both authors read and approved the final manuscript.

## Supplementary Material

Additional file 1 Perl scripts for normalization. Click here for file

Additional file 2 qPCR primers and expression estimation sets. Click here for file

Additional file 3 Chloroplast scaffolds and annotations. Click here for file

Additional file 4 Mitochondrial scaffolds and annotations. Click here for file

Additional file 5 KEGG mapping and expression results. Click here for file

Additional file 6 Epigenetic events during petal pigmentation. Click here for file

Additional file 7 Perl scripts for extracting and organizing KEGG information. Click here for file

Additional file 8 Perl scripts for organelle genome mapping. Click here for file

Additional file 9 Additional primers for sequence verifications. Click here for file
