# Supplementary material for: Dissecting organ-specific transcriptomes through RNA-sequencing
Source: Plant Methods. 2013 Oct 25;9:42. doi: 10.1186/1746-4811-9-42 (PMC3819660; doi:10.1186/1746-4811-9-42)
Supplement: Additional file 1 — Perl scripts for normalization. [file 1746-4811-9-42-S1.pdf]

Additional file 1

Perl scripts and command lines for transcript normalization

I. Applying bowtie (http://bowtie-bio.sourceforge.net/index.shtml): bowtie -a -p 10 -v 3 trinity -f A.PER --suppress 5,6,7 A_PER_bowtie_trinity.av3, to the final assembly file. PER is for paired-end reads. The outcome file is called bowtieResult and used in the next command line:

ReadMap.pl AssembledCtg bowtieResult RdLen MinD MaxD MinCatenatedPEC ReadMap

Here, AssembledCtg is the final assemby in fasta format, RdLen is the length of read, MinD and MaxD are the minimum and maximum lengths of fragments in the sequencing library, respectively. MinCatenatedPEC is the predefined number for minimum PERs that are consecutively mapped to the same scaffold (we used 5), and ReadMap records the positions and number of reads on each scaffold.

II. Normalization of reads by TMM (Robinson and Oshlack, 2010) method is carried out by:

gsExprn.pl ReadMapList AssembledCtg MTrim ATrim Exprn

Here, ReadMapList is a list of ReadMap from each sample handled above, AssembledCtg refers to the assembly of each sample (fasta format), MTrim and ATrim are the pre-defined cutting standards of the house keeping gene set (we used 0.3 and 0.2, respectively), and Exprn lists for each scaffold the number of normalized abundance level.

III. The abundance level file is then combined with information of KEGG:

Map2Exprn.pl MapTitle Map2EC EC2Name Gene2ECOnE Expression Result

Here, MapTitle refers to the name of pathway or network map, downloadable from KEGG (map_title.tab). Map2EC, EC2Name, Gene2ECOnE, and Expression (from gsExprn.pl) are the output files from the corresponding Perl scripts. The final output is Result, which lists the R factors for the samples and three sets of measures for abundance levels including raw, normalized and RPKM for each scaffold.

<u>Perl scripts</u>

```
**********ReadMap.pl***********
#!/usr/bin/perl -w
use strict;

@ARGV or die "$0 AssembledCtg bowtieResult RdLen MinD MaxD MinCatenatedPEC
ReadMap\n";
```

```
my ($RdLen,$MinD,$MaxD,$MinPECN)=($ARGV[2],$ARGV[3],$ARGV[4],$ARGV[5]);
my (%AsL,$CtgID,%Rd2Seq,$RdID,%RdHit);

open(IN,"<$ARGV[0]") or die "Can't open $ARGV[0]!\n";
while(<IN>){
	chomp;
	if($_=~/^>(\S+)\s+Len=(\d+)/){$AsL{$1}=$2;}
}
close(IN);

open(IN,"<$ARGV[1]") or die "Can't open $ARGV[1]!\n";
while(<IN>){if($_=~/^(\S+)_(\d)\s+(\S)\s+(\S+)\s+(\d+)/){$RdHit{$4}{$1}{$2}{$5}=$3;}}
close(IN);

open(OUT,">$ARGV[6]") or die "Can't open $ARGV[6]!\n";
for $CtgID (keys %RdHit){
	my ($S,$E,%PERHit,$Pos1,$Pos2,$i,$j);
	for $RdID (keys %{$RdHit{$CtgID}}){
		if(defined $RdHit{$CtgID}{$RdID}{1}&&defined $RdHit{$CtgID}{$RdID}{2}){
			for $Pos1 (keys %{$RdHit{$CtgID}{$RdID}{1}}){
				for $Pos2 (keys %{$RdHit{$CtgID}{$RdID}{2}}){
					$S=$E=0;
					if($RdHit{$CtgID}{$RdID}{1}{$Pos1} eq
"+"&&$RdHit{$CtgID}{$RdID}{2}{$Pos2} eq "-"&&$Pos1<$Pos2){
						$S=$Pos1; $E=$Pos2+$RdLen;
					}elsif($RdHit{$CtgID}{$RdID}{1}{$Pos1} eq
"-"&&$RdHit{$CtgID}{$RdID}{2}{$Pos2} eq "+"&&$Pos2<$Pos1){
						$S=$Pos2; $E=$Pos1+$RdLen;
					}
					if($E-$S>=$ARGV[3]&&$E-$S<=$ARGV[4]){$PERHit{$S}.="($RdID $E)";}
				}
			}
		}
	}

	if(%PERHit){
		$i=0; $S="";
		my ($HitN,%Ac,$Flag);
		while($i<$AsL{$CtgID}){
			if(defined $PERHit{$i}){
				if($S eq ""){$S=$E=$i;}
				while($PERHit{$i}=~/\(\S+\s+(\d+)\)/g){
					$HitN++;
					if($1>$E){$E=$1;}
```

```
				}
				$Flag=0; $j=$i+1;
				while($j<=$i+$RdLen){
					if(defined $PERHit{$j}){$Flag++; last;}
					else{$j++;}
				}
				if($Flag==0){
					if($HitN>=$ARGV[5]){$Ac{$S}=$E;}
					$S="";
				}
				$i=$j;
			}else{$i++;}
		}

		for($i=0;$i<$AsL{$CtgID};$i++){
			if(defined $Ac{$i}){
				print OUT ">$CtgID\n";
				$HitN=0;
				for($j=$i;$j<$Ac{$i};$j++){
					if(defined $PERHit{$j}){
						while($PERHit{$j}=~/\((\S+)\s+(\d+)\)/g){
							$HitN++;
							print OUT "\t$j\t$2\t$1\n";
						}
					}
				}
				print OUT "\tPERHitN=$HitN\n";
			}
		}
	}
}
close(OUT);
```

**********gsExprn.pl*************

```
#!/usr/bin/perl -w
use strict;
my (%PERN,%PERNInGene,%GeneL,$Gene,$Sample,%R);

sub Read{
	open(IN,"<$ARGV[0]") or die "Can't open $ARGV[0]!\n";
	my (%List,$File);
	while(<IN>){chomp; $List{$_}++;}
	close(IN);
```

```
	for $File (keys %List){
		$File=~/^(\w+?)\./; $Sample=$1;
		print "$File\n";
		open(IN,"<$File") or die "Can't open $File!\n";
		my (%Repeat,%Gene2PER,$PER);
		while(<IN>){
			if($_=~/^>(\S+?)_\d+_\d+$/){$Gene=$1;}
			elsif($_=~/^\s+\d+\s+\d+\s+(\S+)/){$Repeat{$1}++; $Gene2PER{$Gene}{$1}++;}
		}
		close(IN);

		for $Gene (keys %Gene2PER){
			for $PER (keys %{$Gene2PER{$Gene}}){
				$PERNInGene{$Gene}{$Sample}+=$Gene2PER{$Gene}{$PER}/$Repeat{$PER};
			}
			$PERN{$Sample}+=$PERNInGene{$Gene}{$Sample};
		}
	}

	open(IN,"<$ARGV[1]") or die "Can't open $ARGV[1]!\n";
	while(<IN>){if($_=~/^>(\S+)\s+Len=(\d+)/){$GeneL{$1}=$2;}}
	close(IN);
}

sub TMM{
	print "TMM...\n";
	my
(%N,%Y,%Mean,$MeanN,%Mvalue,%Avalue,$Flag,%MaxM,%MinM,%MaxA,%MinA,$m,$y,$a,%w)
;
	for $Gene (keys %PERNInGene){
		$Flag=1;
		for $Sample (keys %{$PERNInGene{$Gene}}){$Flag*=$PERNInGene{$Gene}{$Sample};}
		if($Flag!=0){
			for $Sample (keys %{$PERNInGene{$Gene}}){
				$Y{$Gene}{$Sample}=$PERNInGene{$Gene}{$Sample}/$GeneL{$Gene};
				$N{$Sample}+=$PERNInGene{$Gene}{$Sample};
			}
		}
	}

	for $Gene (keys %Y){
		for $Sample (keys %{$Y{$Gene}}){$Mean{$Gene}+=$Y{$Gene}{$Sample};}
		$Mean{$Gene}/=keys %{$Y{$Gene}};
```

```
	}
	for $Sample (keys %N){$MeanN+=$N{$Sample};}
	$MeanN/=keys %N;

	for $Sample (keys %N){
		$MaxM{$Sample}=$MinM{$Sample}=0;
		$MaxA{$Sample}=-100; $MinA{$Sample}=100;
	}
	for $Gene (keys %Mean){
		$m=$Mean{$Gene}/$MeanN;
		for $Sample (keys %{$Y{$Gene}}){
			$y=$Y{$Gene}{$Sample}/$N{$Sample};
			$Mvalue{$Sample}{$Gene}=log($y/$m);
			$Avalue{$Sample}{$Gene}=0.5*log($y*$m);

	if($Mvalue{$Sample}{$Gene}>$MaxM{$Sample}){$MaxM{$Sample}=$Mvalue{$Sample}{$Ge
ne};}

	if($Avalue{$Sample}{$Gene}>$MaxA{$Sample}){$MaxA{$Sample}=$Avalue{$Sample}{$Gene
};}

	if($Mvalue{$Sample}{$Gene}<$MinM{$Sample}){$MinM{$Sample}=$Mvalue{$Sample}{$Ge
ne};}

	if($Avalue{$Sample}{$Gene}<$MinA{$Sample}){$MinA{$Sample}=$Avalue{$Sample}{$Gene}
;}
		}
	}

	for $Sample (keys %Mvalue){
		for $Gene (keys %{$Mvalue{$Sample}}){

	$m=($Mvalue{$Sample}{$Gene}-$MinM{$Sample})/($MaxM{$Sample}-$MinM{$Sample});

	$a=($Avalue{$Sample}{$Gene}-$MinA{$Sample})/($MaxA{$Sample}-$MinA{$Sample});
			if(($m>1-$ARGV[2]||$m<$ARGV[2])&&($a>1-$ARGV[3]||$a<$ARGV[3])){
				delete $Mvalue{$Sample}{$Gene};
				delete $Avalue{$Sample}{$Gene};
			}
		}
	}

	for $Sample (keys %Mvalue){
		my $HouseN=0;
```

```
		for $Gene (keys %{$Mvalue{$Sample}}){
			$w{$Sample}{$Gene}=1/$Y{$Gene}{$Sample}+1/$Mean{$Gene};
			$HouseN++;
		}
		print "$Sample:\t$HouseN\n";
	}
	for $Sample (keys %Mvalue){
		my ($Denominator,$Numerator);
		for $Gene (keys %{$Mvalue{$Sample}}){

	$Numerator+=$w{$Sample}{$Gene}*log(($Y{$Gene}{$Sample}/$N{$Sample})/($Mean{$Gen
e}/$MeanN));
			$Denominator+=$w{$Sample}{$Gene};
		}
		$R{$Sample}=exp($Numerator/$Denominator);
	}
}

sub Print{
	print "Print...\n";
	open(OUT,">$ARGV[4]") or die "Can't open $ARGV[4]\n";
	for $Sample (sort keys %PERN){printf OUT
"$Sample:\t%15.2f\tR:%10.2f\n",$PERN{$Sample},$R{$Sample};}
	printf OUT "%20s"," ";
	for $Sample (sort keys %PERN){printf OUT "%-8s",$Sample;}
	printf OUT "\n";

	my ($RPKM,$Tmp);
	for $Gene (sort keys %PERNInGene){
		printf OUT "%-15s%-5s",$Gene,"Raw:";
		for $Sample (sort keys %PERN){
			if(defined $PERNInGene{$Gene}{$Sample}){printf OUT
"%-8d",$PERNInGene{$Gene}{$Sample};}
			else{printf OUT "%-8d",0;}
		}

		printf OUT "%-8s","Normal:";
		for $Sample (sort keys %PERN){
			if(defined $PERNInGene{$Gene}{$Sample}){
				$Tmp=$PERNInGene{$Gene}{$Sample}/$R{$Sample};
				printf OUT "%-8d",$Tmp;
			}else{printf OUT "%-8d",0;}
		}
```

```
		printf OUT "%-6s","RPKM:";
		for $Sample (sort keys %PERN){
			my $Denominator=$PERN{$Sample}*$GeneL{$Gene};
			if(defined $PERNInGene{$Gene}{$Sample}){
				$RPKM=1e9*$PERNInGene{$Gene}{$Sample}/$Denominator;
				printf OUT "%-8d",$RPKM;
			}else{printf OUT "%-8d",0;}
		}
		printf OUT "\n";
	}
	close(OUT);
}

#main
@ARGV or die "Usage:$0 ReadMapList AssembledCtg MTrim ATrim Exprn\n";
Read();
TMM();
Print();

*************Map2Exprn.pl***************
#!/usr/bin/perl -w
use strict;
my (%MapTitle,%Map2EC,%EC2Name,%GeneOfEC,%Exprn,$Map,$EC,$Unigene,$ECGene);

sub Read{
	open(IN,"<$ARGV[0]") or die "Can't open $ARGV[0]\n";
	while(<IN>){chomp; $_=~/^(\S+)\s+(\w.+)/; $MapTitle{$1}=$2;}
	close(IN);

	open(IN,"<$ARGV[1]") or die "Can't open $ARGV[1]\n";
	while(<IN>){
		chomp;
		if($_=~/^>(\S+)/){$Map=$1;}
		elsif($_=~/^\s+(\S+)/){$Map2EC{$Map}{$1}++;}
	}
	close(IN);

	open(IN,"<$ARGV[2]") or die "Can't open $ARGV[2]\n";
	while(<IN>){chomp; $_=~/^(\S+)\s+(\w.+)/; $EC2Name{$1}=$2;}
	close(IN);

	open(IN,"<$ARGV[3]") or die "Can't open $ARGV[3]\n";
	while(<IN>){
		$_=~s/\(.+\)//g;
```

```
		$_=~/^(\S+)\s+(\S+)\s+(\S+)\s+(\S+)/;
		$Unigene=$1; $ECGene=$2; $EC=$3;
		if(!defined
$GeneOfEC{$EC}{$Unigene}{$ECGene}||$GeneOfEC{$EC}{$Unigene}{$ECGene}>$4){
			$GeneOfEC{$EC}{$Unigene}{$ECGene}=$4;
		}
	}
	close(IN);

	open(IN,"<$ARGV[4]") or die "Can't open $ARGV[4]\n";
	while(<IN>){chomp; if($_=~/^(\S+?_\d+)\s+(\S.+?\S)\s+BlastAnno/){$Exprn{$1}=$2;}}
	close(IN);
}

sub Write{
	open(OUT,">$ARGV[5]") or die "Can't open $ARGV[5]\n";
	for $Map (sort keys %Map2EC){
		printf OUT ">$Map\t$MapTitle{$Map}\n";
		for $EC (sort keys %{$Map2EC{$Map}}){
			if(defined $EC2Name{$EC}){printf OUT "\t$EC($EC2Name{$EC})\n";}
			else{printf OUT "\t$EC(No Name)\n";}
			if(defined $GeneOfEC{$EC}){
				my $MinE=10;
				for $Unigene (keys %{$GeneOfEC{$EC}}){
					for $ECGene (keys %{$GeneOfEC{$EC}{$Unigene}}){
						if($GeneOfEC{$EC}{$Unigene}{$ECGene}<$MinE&&defined
$Exprn{$Unigene}){
							$MinE=$GeneOfEC{$EC}{$Unigene}{$ECGene};
						}
					}
				}
				for $Unigene (keys %{$GeneOfEC{$EC}}){
					for $ECGene (keys %{$GeneOfEC{$EC}{$Unigene}}){
						if($GeneOfEC{$EC}{$Unigene}{$ECGene}==$MinE&&defined
$Exprn{$Unigene}){
							printf OUT
"\t\t%-20s%-30s%-12s\t$Exprn{$Unigene}\n",$Unigene,$ECGene,$GeneOfEC{$EC}{$Unigene}{$E
CGene};
							last;
						}
					}
				}
			}else{printf OUT "\t\tNo Correspond Unigene\n";}
		}
```

```
	}
	close(OUT);
}

# main program
@ARGV or die "Usage:Map2Exprn.pl MapTitle Map2EC EC2Name Unigene2EC Expression
Result\n";
Read();
Write();
```
