# Supplementary material for: Dissecting organ-specific transcriptomes through RNA-sequencing
Source: Plant Methods. 2013 Oct 25;9:42. doi: 10.1186/1746-4811-9-42 (PMC3819660; doi:10.1186/1746-4811-9-42)
Supplement: Additional file 2 — qPCR primers and expression estimation sets. [file 1746-4811-9-42-S2.pdf]

Additional file 2 Real-time qPCR primers and expression estimation results. Transcript abundance levels via qPCR-based estimates and RNA-sequencing were collected on genotype III6D grown in the field in 2009. The samples were taken at 60 hours before floral opening on the 1st and 25th of September, respectively. The significance of the correlation was based on a *t*-test.

Gene name	Primer pair (forward/reverse, 5'→3')	Estimate by qPCR (copies/pg cDNA)	Standard error	Estimate by normalized read number	Pearson correlation coefficient	P-value
CHS-D	atggtgaccgtcgaggaggtc/ gtcgggcttatgctgggacg	1062.5	90.4	19196	0.774	<0.05
CHI	atgtctgcgccgccg/ ggctgcagttatttagagacgacgaccg	537.4	75.8	10046		
F3H	atgacgacggtgtcaaccttа/ cgcatatatatactttgtgactttactgca	214.7	8.5	14241		
F3'H	atggctaccttaacctta/ cgctgcagttaattgagagtatagagat	92.5	1.8	10924		
DFR-B	atggtggacggtaatcatcctcttc/ gcgctgcagcttctcaagcttttaa	344.1	8.5	7866		
ANS	atgctgtctactattactgcaactgttc/ tataagcttctaatttgattgatgatcatcatc	422.7	27.9	17694		
3GT	atgggcagttcagagtgc/ gcgctgcagaattccagcttatca	1150.8	11.5	77241		
MYB1	tatggatccatcgttaattcttctgcaag/ tatctcgagttaaatggttgtgtctaaaag	14.1	1.9	2716		
bHLH2	gatggatccatggcggaaaccccctg/ ggactcgagctaaaactgaggaattatrctatg	1.0	0.1	4153		
WDR1	caaagatggtgaactcaac/ catctccttacacttttagc	3.3	0.2	1251		
