# Supplementary material for: Dissecting organ-specific transcriptomes through RNA-sequencing
Source: Plant Methods. 2013 Oct 25;9:42. doi: 10.1186/1746-4811-9-42 (PMC3819660; doi:10.1186/1746-4811-9-42)
Supplement: Additional file 3 — Chloroplast scaffolds and annotations. [file 1746-4811-9-42-S3.pdf]

Additional file 3. Expression of *Ipomoea* chloroplastic genome

********************format explanation****************
>SeqID
col2~7: normalized expression abundance; clo8: SeqID; col9: SeqLength; col10:uniref50 proteinID; col11:uniref50 protein SeqLength; col12~21: blast result;
**********Hit in genome ****************
col1: SeqID; col2: SeqLength; col3: genome ID; col4: genome SeqLength; col5~: blast result
**********Hit in annotated cds***************
cdsID
col1: SeqID; col2: SeqLength; col3: cds ID; col4: cds SeqLength; col5~: blast result
annotation for cds
//
****

>ABCDEF_29648
Normal: 0 8 0 17 12 58 ABCDEF_29648 383 UniRef50_Q9MTL3 517 80.95 126 24 0 378 1 375 500 2e-66 217

**********Hit in genome EU118126****************
ABCDEF_29648 383 EU118126 162046 100.00 383 0 0 1 383 60853 60471 0.0 759
**********Hit in cds accD****************
accD
ABCDEF_29648 383 accD 1530 100.00 383 0 0 1 383 1487 1105 0.0 759
codon_start=1 transl_table=11 product="acetyl-CoA carboxylase carboxyltransferase beta protein_id="ABV02357.1" 59366..60895
//

>ABCDEF_81864
Normal: 0 0 0 0 0 14 No annotation
**********Hit in genome EU118126****************
ABCDEF_81864 254 EU118126 162046 100.00 254 0 0 1 254 29454 29707 7e-145 504
*************no hit in cds*****************
//

>ABCDEF_50365
Normal: 0 11 0 0 0 52 ABCDEF_50365 442 UniRef50_Q49CB5 1815 85.04 127 18 1 382 2 1324 1449 3e-64 220

**********Hit in genome EU118126****************
ABCDEF_50365 442 EU118126 162046 100.00 440 0 0 3 442 93804 93365 0.0 872
ABCDEF_50365 442 EU118126 162046 100.00 440 0 0 3 442 156415 156854 0.0 872
ABCDEF_50365 442 EU118126 162046 96.55 29 1 0 399 427 93366 93338 6e-08 50.1
ABCDEF_50365 442 EU118126 162046 96.55 29 1 0 399 427 156853 156881 6e-08 50.1
ABCDEF_50365 442 EU118126 162046 96.15 26 1 0 395 420 93388 93363 4e-06 44.1
ABCDEF_50365 442 EU118126 162046 96.15 26 1 0 395 420 156831 156856 4e-06 44.1
**********Hit in cds ycf2****************
ycf2
ABCDEF_50365 442 ycf2 6594 100.00 440 0 0 3 442 5199 4760 0.0 872
ABCDEF_50365 442 ycf2 6594 96.55 29 1 0 399 427 4761 4733 3e-08 50.1
ABCDEF_50365 442 ycf2 6594 96.15 26 1 0 395 420 4783 4758 2e-06 44.1
codon_start=1 transl_table=11 product="hypothetical chloroplast RF21" protein_id="ABV02391.1" codon_start=1 transl_table=11 product="hypothetical chloroplast RF21" protein_id="ABV02414.1" complement(155019..161612)
//

>ABCDEF_9087
Normal: 0 11 0 0 0 24 ABCDEF_9087 280 UniRef50_Q9XQV2 776 52.38 84 39 1 32 280 278 361 1e-11 66.2
**********Hit in genome EU118126****************
ABCDEF_9087 280 EU118126 162046 100.00 280 0 0 1 280 40532 40253 3e-160 555
ABCDEF_9087 280 EU118126 162046 88.31 77 9 0 188 264 42569 42493 1e-17 81.8
**********Hit in cds psaB****************
psaB
ABCDEF_9087 280 psaB 2205 100.00 280 0 0 1 280 619 898 1e-160 555
codon_start=1 transl_table=11 product="photosystem I P700 apoprotein A2" protein_id="ABV02347.1" complement(38945..41149)
//

>ABCDEF_39804
Normal: 16 0 0 5 17 60 No annotation
**********Hit in genome EU118126****************
ABCDEF_39804 467 EU118126 162046 100.00 467 0 0 1 467 130501 130035 0.0 926
**********Hit in cds ndhA****************

```
ndhA
ABCDEF_39804   467     ndhA           2532    100.00  467     0       0       1       467     1150    1616    0.0     926
codon_start=1 transl_table=11 product="NADH-plastoquinone oxidoreductase subunit 1" protein_id="ABV02406.1"       complement(129118..131649)
//

>ABCDEF_82407
Normal: 0       0       0       0       0       14           ABCDEF_82407        214     UniRef50_Q9GDV0          164     65.71   35      12      0       102     206     6       40      4e-08   52.4
**********Hit in genome EU118126****************
ABCDEF_82407   214     EU118126       162046  99.07   214     0       1       1       214     63262   63473   2e-114  402
**********Hit in cds cemA****************
cemA
ABCDEF_82407   214     cemA           690     100.00  123     0       0       92      214     5       127     5e-67   244
codon_start=1 transl_table=11 product="chloroplast envelope membrane protein" protein_id="ABV02360.1"      63346..64035
//

>ABCDEF_39040
Normal: 0       9       10      0       10      70           ABCDEF_39040        445     UniRef50_P56768          37      90.32   31      3       0       256     164     6       36      2e-10   58.9
**********Hit in genome EU118126****************
ABCDEF_39040   445     EU118126       162046  99.77   439     1       0       1       439     61891   61453   0.0     862
**********Hit in cds psaI****************
psaI
ABCDEF_39040   445     psaI           102     99.02   102     1       0       157     258     102     1       9e-52   194
codon_start=1 transl_table=11 product="photosystem I subunit VIII" protein_id="ABV02358.1"  61633..61734
//

>ABCDEF_83105
Normal: 5       11      0       0       0       40           ABCDEF_83105        432     UniRef50_Q9XQV2          776     44.37   142     70      2       6       431     490     622     1e-28   117
**********Hit in genome EU118126****************
ABCDEF_83105   432     EU118126       162046  100.00  432     0       0       1       432     42082   41651   0.0     856
**********Hit in cds psaA****************
psaA
ABCDEF_83105   432     psaA           2253    100.00  432     0       0       1       432     1347    1778    0.0     856
codon_start=1 transl_table=11 product="photosystem I P700 apoprotein A1" protein_id="ABV02348.1"   complement(41175..43427)
//

>ABCDEF_9003
Normal: 0       9       8       0       0       20           ABCDEF_9003         447     UniRef50_Q32836          2110    67.31   52      17      0       284     439     416     467     1e-12   71.6
**********Hit in genome EU118126****************
ABCDEF_9003    447     EU118126       162046  100.00  447     0       0       1       447     90349   90795   0.0     886
ABCDEF_9003    447     EU118126       162046  100.00  447     0       0       1       447     159870  159424  0.0     886
**********Hit in cds ycf2****************
ycf2
ABCDEF_9003    447     ycf2           6594    100.00  447     0       0       1       447     1744    2190    0.0     886
codon_start=1 transl_table=11 product="hypothetical chloroplast RF21" protein_id="ABV02391.1" codon_start=1 transl_table=11 product="hypothetical chloroplast RF21" protein_id="ABV02414.1"    compleme
//

>ABCDEF_81631
Normal: 0       0       0       0       0       64   No annotation
**********Hit in genome EU118126****************
ABCDEF_81631   249     EU118126       162046  100.00  249     0       0       1       249     77740   77492   7e-142  494
**********Hit in cds petB****************
petB
ABCDEF_81631   249     petB           1401    100.00  235     0       0       1       235     235     1       8e-134  466
codon_start=1 transl_table=11 product="cytochrome b6" protein_id="ABV02378.1"       77505..78905
//

>ABCDEF_85310
Normal: 0       0       0       0       0       10           ABCDEF_85310        201     UniRef50_Q7V510          776     72.73   66      18      0       201     4       70      135     4e-28   112
**********Hit in genome EU118126****************
ABCDEF_85310   201     EU118126       162046  100.00  201     0       0       1       201     43038   43238   2e-113  398
**********Hit in cds psaA****************
psaA
ABCDEF_85310   201     psaA           2253    100.00  201     0       0       1       201     391     191     1e-113  398
codon_start=1 transl_table=11 product="photosystem I P700 apoprotein A1" protein_id="ABV02348.1"   complement(41175..43427)
//

>ABCDEF_82436
Normal: 0       9       0       0       0       162  No annotation
**********Hit in genome EU118126****************
ABCDEF_82436   331     EU118126       162046  100.00  331     0       0       1       331     8671    8341    0.0     656
```

**********Hit in cds trnS-GCU****************
trnS-GCU
ABCDEF_82436 331 trnS-GCU 88 100.00 27 0 0 305 331 1 27 1e-09 54.0
complement(8279..8366)
//

>ABCDEF_88540
Normal: 0 0 0 0 0 12 ABCDEF_88540 229 UniRef50_Q9TL05 863 37.84 74 44 1 227 6 408 479 4e-08 55.5
**********Hit in genome EU118126****************
ABCDEF_88540 229 EU118126 162046 100.00 229 0 0 1 229 22387 22615 6e-130 454
**********Hit in cds rpoC1****************
rpoC1
ABCDEF_88540 229 rpoC1 2874 100.00 229 0 0 1 229 1911 1683 3e-130 454
codon_start=1 transl_table=11 product="RNA polymerase beta subunit" protein_id="ABV02339.1" complement(21423..24296)
//

>ABCDEF_81392
Normal: 0 0 0 0 0 20 No annotation
**********Hit in genome EU118126****************
ABCDEF_81392 254 EU118126 162046 100.00 254 0 0 1 254 30645 30898 7e-145 504
*************no hit in cds*****************
//

>ABCDEF_69106
Normal: 0 0 0 0 0 5 No annotation
**********Hit in genome EU118126****************
ABCDEF_69106 196 EU118126 162046 99.49 196 1 0 1 196 62745 62940 6e-108 381
*************no hit in cds*****************
//

>ABCDEF_5439
Normal: 0 13 0 0 0 28 ABCDEF_5439 314 UniRef50_Q0ZJ09 186 84.00 25 4 0 75 1 1 25 4e-06 48.5
**********Hit in genome EU118126****************
ABCDEF_5439 314 EU118126 162046 100.00 314 0 0 1 314 62252 61939 1e-180 622
**********Hit in cds ycf4****************
ycf4
ABCDEF_5439 314 ycf4 555 100.00 74 0 0 1 74 74 1 1e-37 147
codon_start=1 transl_table=11 product="photosystem I assembly protein ycf4" protein_id="ABV02359.1" 62178..62732
//

>ABCDEF_83642
Normal: 0 0 0 0 0 20 No annotation
**********Hit in genome EU118126****************
ABCDEF_83642 196 EU118126 162046 100.00 196 0 0 1 196 32327 32522 2e-110 389
*************no hit in cds*****************
//

>ABCDEF_18922
Normal: 10 29 19 0 0 108 ABCDEF_18922 635 UniRef50_Q06R82 73 71.93 57 16 0 111 281 1 57 2e-08 55.1
**********Hit in genome EU118126****************
ABCDEF_18922 635 EU118126 162046 100.00 628 0 0 8 635 122368 122995 0.0 1245
**********Hit in cds rpl32****************
rpl32
ABCDEF_18922 635 rpl32 174 100.00 174 0 0 112 285 1 174 5e-97 345
codon_start=1 transl_table=11 product="ribosomal protein L32" protein_id="ABV02399.1" 122471..122644
//

>ABCDEF_88972
Normal: 0 0 0 0 0 26 ABCDEF_88972 355 UniRef50_Q3AC82 140 39.77 88 52 1 2 265 30 116 7e-15 72.8
**********Hit in genome EU118126****************
ABCDEF_88972 355 EU118126 162046 100.00 355 0 0 1 355 128911 128557 0.0 704
**********Hit in cds ndhI****************
ndhI
ABCDEF_88972 355 ndhI 504 100.00 355 0 0 1 355 127 481 0.0 704
codon_start=1 transl_table=11 product="NADH-plastoquinone oxidoreductase subunit I" protein_id="ABV02405.1" complement(128533..129036)
//

>ABCDEF_86702
Normal: 0 0 0 0 0 28 ABCDEF_86702 389 UniRef50_Q49CB5 1815 43.31 127 41 2 381 1 883 978 3e-18 87.4
**********Hit in genome EU118126****************

ABCDEF_86702 389 EU118126 162046 99.74 389 1 0 1 389 92344 91956 0.0 763
ABCDEF_86702 389 EU118126 162046 99.74 389 1 0 1 389 157875 158263 0.0 763
**********Hit in cds ycf2***************
ycf2
ABCDEF_86702 389 ycf2 6594 99.74 389 1 0 1 389 3739 3351 0.0 763
codon_start=1 transl_table=11 product="hypothetical chloroplast RF21" protein_id="ABV02391.1" codon_start=1 transl_table=11 product="hypothetical chloroplast RF21" protein_id="ABV02414.1" compleme
//

>ABCDEF_39039
Normal: 0 8 0 0 0 18 No annotation
**********Hit in genome EU118126***************
ABCDEF_39039 276 EU118126 162046 99.64 276 1 0 1 276 62030 61755 1e-155 539
*************no hit in cds****************
//

>ABCDEF_66272
Normal: 0 0 8 0 0 0 No annotation
**********Hit in genome EU118126***************
ABCDEF_66272 216 EU118126 162046 98.61 216 3 0 1 216 61943 61728 4e-115 404
*************no hit in cds****************
//

>ABCDEF_78964
Normal: 0 14 12 20 5 62 No annotation
**********Hit in genome EU118126***************
ABCDEF_78964 518 EU118126 162046 100.00 518 0 0 1 518 74545 75062 0.0 1027
**********Hit in cds psbB***************
psbB
ABCDEF_78964 518 psbB 1527 100.00 51 0 0 468 518 1 51 1e-23 101
codon_start=1 transl_table=11 product="photosystem II CP47 chlorophyll apoprotein" protein_id="ABV02374.1" 75011..76537
//

>ABCDEF_79735
Normal: 0 0 0 0 0 10 ABCDEF_79735 227 UniRef50_A4QM92 46 59.46 37 15 0 97 207 7 43 4e-08 51.2
**********Hit in genome EU118126***************
ABCDEF_79735 227 EU118126 162046 100.00 227 0 0 1 227 37824 38050 9e-129 450
ABCDEF_79735 227 EU118126 162046 96.30 27 1 0 181 207 9826 9852 5e-07 46.1
**********Hit in cds trnG-GCC***************
trnG-GCC
ABCDEF_79735 227 trnG-GCC 71 100.00 71 0 0 139 209 1 71 6e-36 141
37961..38031
//

>ABCDEF_49916
Normal: 0 26 37 20 7 266 ABCDEF_49916 560 UniRef50_Q8M9U2 315 55.32 141 55 4 5 415 178 314 1e-41 150
**********Hit in genome EU118126***************
ABCDEF_49916 560 EU118126 162046 100.00 560 0 0 1 560 124281 124840 0.0 1110
**********Hit in cds ccsA***************
ccsA
ABCDEF_49916 560 ccsA 954 100.00 434 0 0 1 434 521 954 0.0 860
codon_start=1 transl_table=11 product="cytochrome c heme attachment protein" protein_id="ABV02400.1" 123760..124713
//

>ABCDEF_85690
Normal: 0 11 0 0 0 48 ABCDEF_85690 344 UniRef50_Q9XQV2 776 44.25 113 52 2 344 9 333 435 2e-19 89.7
**********Hit in genome EU118126***************
ABCDEF_85690 344 EU118126 162046 100.00 344 0 0 1 344 39993 40336 0.0 682
ABCDEF_85690 344 EU118126 162046 86.76 68 9 0 277 344 42493 42560 3e-12 63.9
**********Hit in cds psaB***************
psaB
ABCDEF_85690 344 psaB 2205 100.00 344 0 0 1 344 1158 815 0.0 682
codon_start=1 transl_table=11 product="photosystem I P700 apoprotein A2" protein_id="ABV02347.1" complement(38945..41149)
//

>ABCDEF_33012
Normal: 0 0 0 0 0 46 ABCDEF_33012 435 UniRef50_Q9TJZ2 144 92.00 50 4 0 152 3 35 84 3e-23 96.3
**********Hit in genome EU118126***************
ABCDEF_33012 435 EU118126 162046 99.77 435 0 1 1 435 22858 23291 0.0 846
**********Hit in cds rpoC1***************
rpoC1

ABCDEF_33012 435 rpoC1 2874 99.77 435 0 1 1 435 1440 1007 0.0 846
codon_start=1 transl_table=11 product="RNA polymerase beta subunit" protein_id="ABV02339.1" complement(21423..24296)
//

>ABCDEF_39022
Normal: 0 89 26 30 7 422 ABCDEF_39022 1115 UniRef50_Q7V4E4 563 52.43 309 146 1 3 926 194 502 2e-102 322
**********Hit in genome EU118126****************
ABCDEF_39022 1115 EU118126 162046 99.73 1114 3 0 2 1115 125996 124883 0.0 2185
**********Hit in cds ndhD****************
ndhD
ABCDEF_39022 1115 ndhD 1512 99.70 999 3 0 2 1000 514 1512 0.0 1957
exception="RNA editing" codon_start=1 transl_table=11 product="NADH-plastoquinone oxidoreductase subunit 4" protein_id="ABV02401.1" complement(124997..126508)
//

>ABCDEF_85923
Normal: 0 0 0 0 0 18 ABCDEF_85923 385 UniRef50_Q9MUS6 668 72.66 128 35 0 2 385 421 548 1e-57 196
**********Hit in genome EU118126****************
ABCDEF_85923 385 EU118126 162046 100.00 385 0 0 1 385 22254 21870 0.0 763
**********Hit in cds rpoC1****************
rpoC1
ABCDEF_85923 385 rpoC1 2874 100.00 385 0 0 1 385 2044 2428 0.0 763
codon_start=1 transl_table=11 product="RNA polymerase beta subunit" protein_id="ABV02339.1" complement(21423..24296)
//

>ABCDEF_68080
Normal: 0 12 8 0 6 38 ABCDEF_68080 347 UniRef50_P56786 2294 49.67 153 29 4 319 2 157 308 6e-27 112
**********Hit in genome EU118126****************
ABCDEF_68080 347 EU118126 162046 100.00 329 0 0 1 329 89414 89086 0.0 652
ABCDEF_68080 347 EU118126 162046 100.00 329 0 0 1 329 160805 161133 0.0 652
**********Hit in cds ycf2****************
ycf2
ABCDEF_68080 347 ycf2 6594 100.00 329 0 0 1 329 809 481 0.0 652
codon_start=1 transl_table=11 product="hypothetical chloroplast RF21" protein_id="ABV02391.1" codon_start=1 transl_table=11 product="hypothetical chloroplast RF21" protein_id="ABV02414.1" compleme
//

>ABCDEF_23201
Normal: 0 0 8 0 0 14 ABCDEF_23201 299 UniRef50_Q85C71 1445 77.22 79 18 0 2 238 1304 1382 3e-32 126
**********Hit in genome EU118126****************
ABCDEF_23201 299 EU118126 162046 100.00 298 0 0 2 299 17480 17183 5e-171 591
**********Hit in cds rpoC2****************
rpoC2
ABCDEF_23201 299 rpoC2 4206 100.00 298 0 0 2 299 3782 4079 3e-171 591
codon_start=1 transl_table=11 product="RNA polymerase beta' subunit" protein_id="ABV02338.1" complement(17055..21260)
//

>ABCDEF_43545
Normal: 0 49 0 0 0 114 ABCDEF_43545 438 UniRef50_P62090 81 100.00 81 0 0 271 29 1 81 1e-52 169
**********Hit in genome EU118126****************
ABCDEF_43545 438 EU118126 162046 100.00 438 0 0 1 438 126599 127036 0.0 868
**********Hit in cds psaC****************
psaC
ABCDEF_43545 438 psaC 246 100.00 246 0 0 27 272 246 1 4e-140 488
codon_start=1 transl_table=11 product="photosystem I subunit VII" protein_id="ABV02402.1" complement(126624..126869)
//

>ABCDEF_12433
Normal: 0 8 7 0 0 32 ABCDEF_12433 518 UniRef50_Q01923 1520 70.35 172 50 1 2 517 1179 1349 2e-74 250
**********Hit in genome EU118126****************
ABCDEF_12433 518 EU118126 162046 100.00 518 0 0 1 518 18099 17582 0.0 1027
**********Hit in cds rpoC2****************
rpoC2
ABCDEF_12433 518 rpoC2 4206 100.00 518 0 0 1 518 3163 3680 0.0 1027
codon_start=1 transl_table=11 product="RNA polymerase beta' subunit" protein_id="ABV02338.1" complement(17055..21260)
//

>ABCDEF_82700
Normal: 0 0 0 0 0 30 ABCDEF_82700 373 UniRef50_Q01923 1520 86.40 125 15 2 1 372 158 281 6e-67 226
**********Hit in genome EU118126****************
ABCDEF_82700 373 EU118126 162046 100.00 373 0 0 1 373 20777 20405 0.0 739
**********Hit in cds rpoC2****************

```
rpoC2
ABCDEF_82700   373     rpoC2          4206    100.00  373     0       0       1       373     485     857     0.0     739
codon_start=1 transl_table=11 product="RNA polymerase beta' subunit" protein_id="ABV02338.1"       complement(17055..21260)
//

>ABCDEF_70241
Normal: 3       11      2       0       0       42           ABCDEF_70241        231     UniRef50_Q9T3G4          512     94.52   73      4       0       221     3       116     188     2e-40   145
**********Hit in genome EU118126****************
ABCDEF_70241   231     EU118126       162046  99.55   220     1       0       1       220     97785   98004   3e-122  428
ABCDEF_70241   231     EU118126       162046  99.55   220     1       0       1       220     152434  152215  3e-122  428
**********Hit in cds ndhB****************
ndhB
ABCDEF_70241   231     ndhB           2198    99.55   220     1       0       1       220     565     346     2e-122  428
codon_start=1 transl_table=11 product="NADH-plastoquinone oxidoreductase subunit 2" protein_id="ABV02392.1" codon_start=1 transl_table=11 product="NADH-plastoquinone oxidoreductase subunit 2" prot
//

>ABCDEF_32387
Normal: 9       52      41      68      0       212          ABCDEF_32387        749     UniRef50_Q7G6A4          341     88.89   63      7       0       236     48      19      81      2e-29   120
**********Hit in genome EU118126****************
ABCDEF_32387   749     EU118126       162046  100.00  749     0       0       1       749     75672   74924   0.0     1485
**********Hit in cds psbB****************
psbB
ABCDEF_32387   749     psbB           1527    100.00  661     0       0       1       661     661     1       0.0     1310
codon_start=1 transl_table=11 product="photosystem II CP47 chlorophyll apoprotein" protein_id="ABV02374.1" 75011..76537
//

>ABCDEF_12578
Normal: 0       13      12      0       0       50           ABCDEF_12578        377     UniRef50_Q49CB5          1815    39.37   127     40      3       1       372     340     432     3e-14   75.5
**********Hit in genome EU118126****************
ABCDEF_12578   377     EU118126       162046  100.00  377     0       0       1       377     90053   90429   0.0     747
ABCDEF_12578   377     EU118126       162046  100.00  377     0       0       1       377     160166  159790  0.0     747
**********Hit in cds ycf2****************
ycf2
ABCDEF_12578   377     ycf2           6594    100.00  377     0       0       1       377     1448    1824    0.0     747
codon_start=1 transl_table=11 product="hypothetical chloroplast RF21" protein_id="ABV02391.1" codon_start=1 transl_table=11 product="hypothetical chloroplast RF21" protein_id="ABV02414.1"    compleme
//

>ABCDEF_81344
Normal: 0       0       0       0       0       42           ABCDEF_81344        272     UniRef50_Q1KXP9          53      90.62   32      3       0       97      2       8       39      5e-10   57.0
**********Hit in genome EU118126****************
ABCDEF_81344   272     EU118126       162046  100.00  272     0       0       1       272     100951  101222  1e-155  539
ABCDEF_81344   272     EU118126       162046  100.00  272     0       0       1       272     149268  148997  1e-155  539
**********Hit in cds ycf15****************
ycf15
ABCDEF_81344   272     ycf15          171     100.00  96      0       0       1       96      96      1       8e-51   190
codon_start=1 transl_table=11 product="hypothetical chloroplast RF15" protein_id="ABV02394.1" codon_start=1 transl_table=11 product="hypothetical chloroplast RF15" protein_id="ABV02410.1"    149172..
//

>ABCDEF_86953
Normal: 0       0       0       0       0       32   No annotation
**********Hit in genome EU118126****************
ABCDEF_86953   344     EU118126       162046  100.00  344     0       0       1       344     46778   47121   0.0     682
**********Hit in cds trnS-GGA****************
trnS-GGA
ABCDEF_86953   344     trnS-GGA       87      100.00  78      0       0       267     344     1       78      6e-40   155
47043..47129
//

>ABCDEF_9232
Normal: 12      0       7       0       0       26           ABCDEF_9232         407     UniRef50_Q9TKV3          169     43.48   115     58      3       407     69      18      127     8e-13   68.2
**********Hit in genome EU118126****************
ABCDEF_9232    407     EU118126       162046  99.00   402     1       1       6       407     127753  128151  0.0     761
**********Hit in cds ndhG****************
ndhG
ABCDEF_9232    407     ndhG           531     99.00   402     1       1       6       407     460     62      0.0     761
codon_start=1 transl_table=11 product="NADH-plastoquinone oxidoreductase subunit 6" protein_id="ABV02404.1"       complement(127681..128211)
//

>ABCDEF_87102
Normal: 0       0       0       0       0       34   No annotation
```

```
**********Hit in genome EU118126****************
ABCDEF_87102	364	EU118126	162046	99.73	364	0	1	1	364	59327	58965	0.0	706
*************no hit in cds*****************
//

>ABCDEF_80628
Normal: 0	9	0	0	0	72	No annotation
**********Hit in genome EU118126****************
ABCDEF_80628	278	EU118126	162046	100.00	278	0	0	1	278	53230	52953	4e-159	551
*************no hit in cds*****************
//

>ABCDEF_86343
Normal: 9	0	0	0	0	76	No annotation
**********Hit in genome EU118126****************
ABCDEF_86343	215	EU118126	162046	100.00	215	0	0	1	215	58662	58876	1e-121	426
ABCDEF_86343	215	EU118126	162046	100.00	22	0	0	16	37	58698	58677	2e-06	44.1
*************no hit in cds*****************
//

>ABCDEF_2037
Normal: 5	16	28	0	10	208	ABCDEF_2037	598	UniRef50_Q49CB5	1815	61.26	111	31	3	338	6	654	752	1e-32	132
**********Hit in genome EU118126****************
ABCDEF_2037	598	EU118126	162046	100.00	598	0	0	1	598	91506	90909	0.0	1185
ABCDEF_2037	598	EU118126	162046	100.00	598	0	0	1	598	158713	159310	0.0	1185
**********Hit in cds ycf2****************
ycf2
ABCDEF_2037	598	ycf2	6594	100.00	598	0	0	1	598	2901	2304	0.0	1185
codon_start=1 transl_table=11 product="hypothetical chloroplast RF21" protein_id="ABV02391.1" codon_start=1 transl_table=11 product="hypothetical chloroplast RF21" protein_id="ABV02414.1"	compleme
//

>ABCDEF_9339
Normal: 0	0	8	0	0	26	ABCDEF_9339	295	UniRef50_Q85C71	1445	44.44	72	38	2	2	211	1358	1429	2e-07	54.3
**********Hit in genome EU118126****************
ABCDEF_9339	295	EU118126	162046	100.00	295	0	0	1	295	17319	17025	3e-169	585
**********Hit in cds rpoC2****************
rpoC2
ABCDEF_9339	295	rpoC2	4206	100.00	264	0	0	1	264	3943	4206	5e-151	523
codon_start=1 transl_table=11 product="RNA polymerase beta' subunit" protein_id="ABV02338.1"	complement(17055..21260)
//

>ABCDEF_88476
Normal: 0	0	0	0	0	20	ABCDEF_88476	260	UniRef50_P56770	328	52.43	103	29	3	3	260	123	222	6e-10	60.1
**********Hit in genome EU118126****************
ABCDEF_88476	260	EU118126	162046	100.00	260	0	0	1	260	124115	124374	2e-148	515
**********Hit in cds ccsA****************
ccsA
ABCDEF_88476	260	ccsA	954	100.00	260	0	0	1	260	355	614	1e-148	515
codon_start=1 transl_table=11 product="cytochrome c heme attachment protein" protein_id="ABV02400.1"	123760..124713
//

>ABCDEF_18609
Normal: 27	64	71	0	15	266	ABCDEF_18609	480	UniRef50_Q8M9V7	334	39.22	153	91	1	479	27	182	334	3e-24	102
**********Hit in genome EU118126****************
ABCDEF_18609	480	EU118126	162046	100.00	480	0	0	1	480	80470	80949	0.0	952
**********Hit in cds rpoA****************
rpoA
ABCDEF_18609	480	rpoA	1014	100.00	471	0	0	10	480	1014	544	0.0	934
codon_start=1 transl_table=11 product="RNA polymerase alpha subunit" protein_id="ABV02380.1"	complement(80478..81491)
//

>ABCDEF_84333
Normal: 0	9	0	0	0	50	ABCDEF_84333	275	UniRef50_Q32RY3	834	41.67	60	35	0	181	2	731	790	8e-06	49.3
**********Hit in genome EU118126****************
ABCDEF_84333	275	EU118126	162046	100.00	275	0	0	1	275	21563	21837	2e-157	545
**********Hit in cds rpoC1****************
rpoC1
ABCDEF_84333	275	rpoC1	2874	100.00	275	0	0	1	275	2735	2461	1e-157	545
codon_start=1 transl_table=11 product="RNA polymerase beta subunit" protein_id="ABV02339.1" complement(21423..24296)
//
```

```
>ABCDEF_12598
Normal: 0	23	0	0	0	58		ABCDEF_12598	561	UniRef50_Q9XQV2		776	44.04	193	87	4	5	553	585	766	1e-42	158
**********Hit in genome EU118126****************
ABCDEF_12598	561	EU118126	162046	99.82	561	1	0	1	561	41769	41209	0.0	1104
**********Hit in cds psaA****************
psaA
ABCDEF_12598	561	psaA		2253	99.82	561	1	0	1	561	1660	2220	0.0	1104
codon_start=1 transl_table=11 product="photosystem I P700 apoprotein A1" protein_id="ABV02348.1"	complement(41175..43427)
//

>ABCDEF_29504
Normal: 0	13	4	0	10	47		ABCDEF_29504	253	UniRef50_Q60F87		154	89.47	57	6	0	176	6	1	57	1e-27	105
**********Hit in genome EU118126****************
ABCDEF_29504	253	EU118126	162046	100.00	239	0	0	3	241	80057	79819	7e-136	474
**********Hit in cds petD****************
petD
ABCDEF_29504	253	petD		1183	100.00	239	0	0	3	241	945	707	3e-136	474
codon_start=1 transl_table=11 product="cytochrome b6/f complex subunit IV" protein_id="ABV02379.1"	79112..80294
//

>ABCDEF_81421
Normal: 0	9	0	0	0	32		ABCDEF_81421	304	UniRef50_Q9XQV2		776	49.44	89	45	0	6	272	20	108	7e-22	96.3
**********Hit in genome EU118126****************
ABCDEF_81421	304	EU118126	162046	100.00	304	0	0	1	304	41130	40827	1e-174	603
**********Hit in cds psaB****************
psaB
ABCDEF_81421	304	psaB		2205	100.00	304	0	0	1	304	21	324	7e-175	603
codon_start=1 transl_table=11 product="photosystem I P700 apoprotein A2" protein_id="ABV02347.1"	complement(38945..41149)
//

>ABCDEF_78551
Normal: 12	0	0	0	0	66		ABCDEF_78551	701	UniRef50_Q01923		1520	78.11	233	51	0	3	701	293	525	3e-112	359
**********Hit in genome EU118126****************
ABCDEF_78551	701	EU118126	162046	100.00	701	0	0	1	701	20389	19689	0.0	1390
**********Hit in cds rpoC2****************
rpoC2
ABCDEF_78551	701	rpoC2		4206	100.00	701	0	0	1	701	873	1573	0.0	1390
codon_start=1 transl_table=11 product="RNA polymerase beta' subunit" protein_id="ABV02338.1"	complement(17055..21260)
//

>ABCDEF_89928
Normal: 0	26	3	0	0	76		ABCDEF_89928	585	UniRef50_Q9MRV3		62	79.03	62	13	0	399	214	1	62	3e-18	82.0
**********Hit in genome EU118126****************
ABCDEF_89928	585	EU118126	162046	99.66	585	2	0	1	585	126110	126694	0.0	1144
**********Hit in cds ndhD****************
ndhD
ABCDEF_89928	585	ndhD		1512	99.75	397	1	0	1	397	400	4	0.0	779
exception="RNA editing" codon_start=1 transl_table=11 product="NADH-plastoquinone oxidoreductase subunit 4" protein_id="ABV02401.1"	complement(124997..126508)
//

>ABCDEF_75464
Normal: 5	31	0	22	20	366		ABCDEF_75464	258	UniRef50_Q8CM04		261	51.79	56	26	1	5	169	18	73	4e-11	49.3
**********Hit in genome EU118126****************
ABCDEF_75464	258	EU118126	162046	98.82	255	3	0	4	258	107691	107437	3e-138	482
ABCDEF_75464	258	EU118126	162046	98.82	255	3	0	4	258	142528	142782	3e-138	482
**********Hit in cds rrn23****************
rrn23
ABCDEF_75464	258	rrn23		2810	98.82	255	3	0	4	258	1964	1710	1e-138	482
complement(141681..144490)
//

>ABCDEF_53348
Normal: 0	0	0	0	0	52		ABCDEF_53348	284	UniRef50_Q85WY0		45	88.89	27	3	0	83	3	11	37	5e-08	51.6
**********Hit in genome EU118126****************
ABCDEF_53348	284	EU118126	162046	100.00	284	0	0	1	284	50246	50529	1e-162	563
**********Hit in cds trnF-GAA****************
trnF-GAA
ABCDEF_53348	284	trnF-GAA	73	100.00	73	0	0	12	84	1	73	4e-37	145
50256..50328
```

```
//

>ABCDEF_39738
Normal: 0	28	0	2	0	42		ABCDEF_39738	528	UniRef50_Q9XQV2		776	33.48	230	95	8	1	528	82	307	6e-24	105
**********Hit in genome EU118126****************
ABCDEF_39738	528	EU118126	162046	99.81	528	1	0	1	528	40939	40412	0.0	1039
**********Hit in cds psaB****************
psaB
ABCDEF_39738	528	psaB		2205	99.81	528	1	0	1	528	212	739	0.0	1039
codon_start=1 transl_table=11 product="photosystem I P700 apoprotein A2" protein_id="ABV02347.1"	complement(38945..41149)
//

>ABCDEF_89422
Normal: 0	0	0	0	0	34		ABCDEF_89422	251	UniRef50_P36387		205	78.26	23	5	0	214	146	1	23	9e-06	47.0
**********Hit in genome EU118126****************
ABCDEF_89422	251	EU118126	162046	100.00	251	0	0	1	251	74286	74536	5e-143	498
**********Hit in cds clpP****************
clpP
ABCDEF_89422	251	clpP		2028	100.00	215	0	0	1	215	215	1	7e-122	426
codon_start=1 transl_table=11 product="clp protease proteolytic subunit" protein_id="ABV02373.1"	complement(72472..74499)
//

>ABCDEF_83431
Normal: 0	0	0	0	0	14		ABCDEF_83431	253	UniRef50_B9PBB8		74	78.26	46	5	2	8	130	29	74	3e-12	63.2
**********Hit in genome EU118126****************
ABCDEF_83431	253	EU118126	162046	100.00	253	0	0	1	253	95779	96031	3e-144	502
ABCDEF_83431	253	EU118126	162046	100.00	253	0	0	1	253	154440	154188	3e-144	502
*************no hit in cds*****************
//

>ABCDEF_61184
Normal: 0	4	0	0	0	17	No annotation
**********Hit in genome EU118126****************
ABCDEF_61184	323	EU118126	162046	99.38	320	2	0	1	320	43919	43600	2e-179	618
*************no hit in cds*****************
//

>ABCDEF_83738
Normal: 0	0	0	0	0	16		ABCDEF_83738	266	UniRef50_Q9XQV2		776	52.87	87	30	2	6	266	481	556	1e-16	80.5
**********Hit in genome EU118126****************
ABCDEF_83738	266	EU118126	162046	100.00	266	0	0	1	266	39879	39614	5e-152	527
**********Hit in cds psaB****************
psaB
ABCDEF_83738	266	psaB		2205	100.00	266	0	0	1	266	1272	1537	3e-152	527
codon_start=1 transl_table=11 product="photosystem I P700 apoprotein A2" protein_id="ABV02347.1"	complement(38945..41149)
//

>ABCDEF_87004
Normal: 0	0	0	0	0	12	No annotation
**********Hit in genome EU118126****************
ABCDEF_87004	263	EU118126	162046	100.00	263	0	0	1	263	29289	29551	3e-150	521
*************no hit in cds*****************
//

>ABCDEF_72501
Normal: 4	0	0	0	7	20		ABCDEF_72501	239	UniRef50_P56775		37	100.00	36	0	0	158	51	1	36	5e-16	72.0
**********Hit in genome EU118126****************
ABCDEF_72501	239	EU118126	162046	100.00	219	0	0	21	239	68481	68263	5e-124	434
**********Hit in cds petG****************
petG
ABCDEF_72501	239	petG		114	100.00	114	0	0	44	157	114	1	1e-61	226
codon_start=1 transl_table=11 product="cytochrome b6/f complex subunit V" protein_id="ABV02367.1"	68344..68457
//

>ABCDEF_84961
Normal: 0	14	0	0	0	28		ABCDEF_84961	451	UniRef50_Q9WWM4		101	60.20	98	39	0	294	1	1	98	9e-12	64.3
**********Hit in genome EU118126****************
ABCDEF_84961	451	EU118126	162046	100.00	451	0	0	1	451	127147	127597	0.0	894
**********Hit in cds ndhE****************
ndhE
```

```
ABCDEF_84961   451   ndhE   306   100.00   295   0   0   1   295   295   1   2e-169   585
codon_start=1 transl_table=11 product="NADH-plastoquinone oxidoreductase subunit 4L" protein_id="ABV02403.1"   complement(127135..127440)
//

>ABCDEF_87310
Normal: 0   0   0   0   0   10   ABCDEF_87310   254   UniRef50_Q9LHB6   86   79.17   24   5   0   74   3   1   24   6e-06   46.2
**********Hit in genome EU118126****************
ABCDEF_87310   254   EU118126   162046   100.00   254   0   0   1   254   16768   17021   7e-145   504
**********Hit in cds rps2****************
rps2
ABCDEF_87310   254   rps2   711   100.00   75   0   0   1   75   75   1   3e-38   149
codon_start=1 transl_table=11 product="ribosomal protein S2" protein_id="ABV02337.1"   complement(16131..16841)
//

>ABCDEF_72099
Normal: 0   8   0   10   0   43   ABCDEF_72099   247   UniRef50_Q9T3G4   512   97.53   81   2   0   245   3   182   262   5e-46   160
**********Hit in genome EU118126****************
ABCDEF_72099   247   EU118126   162046   98.31   236   4   0   12   247   97574   97809   1e-124   436
ABCDEF_72099   247   EU118126   162046   98.31   236   4   0   12   247   152645   152410   1e-124   436
**********Hit in cds ndhB****************
ndhB
ABCDEF_72099   247   ndhB   2198   98.31   236   4   0   12   247   776   541   7e-125   436
codon_start=1 transl_table=11 product="NADH-plastoquinone oxidoreductase subunit 2" protein_id="ABV02392.1" codon_start=1 transl_table=11 product="NADH-plastoquinone oxidoreductase subunit 2" prot
//

>ABCDEF_49973
Normal: 21   84   21   10   0   200   ABCDEF_49973   1181   UniRef50_Q7V511   749   41.31   426   192   9   11   1177   73   477   1e-83   278
**********Hit in genome EU118126****************
ABCDEF_49973   1181   EU118126   162046   99.92   1181   1   0   1   1181   43149   41969   0.0   2333
ABCDEF_49973   1181   EU118126   162046   88.31   77   9   0   581   657   40345   40269   5e-17   81.8
**********Hit in cds psaA****************
psaA
ABCDEF_49973   1181   psaA   2253   99.92   1181   1   0   1   1181   280   1460   0.0   2333
codon_start=1 transl_table=11 product="photosystem I P700 apoprotein A1" protein_id="ABV02348.1"   complement(41175..43427)
//

>ABCDEF_18825
Normal: 0   11   7   25   0   52   ABCDEF_18825   386   UniRef50_Q8WJ12   336   56.36   55   23   1   372   208   135   188   9e-08   55.1
**********Hit in genome EU118126****************
ABCDEF_18825   386   EU118126   162046   100.00   386   0   0   1   386   60046   59661   0.0   765
**********Hit in cds accD****************
accD
ABCDEF_18825   386   accD   1530   100.00   386   0   0   1   386   680   295   0.0   765
codon_start=1 transl_table=11 product="acetyl-CoA carboxylase carboxyltransferase beta protein_id="ABV02357.1"   59366..60895
//

>ABCDEF_85855
Normal: 0   6   0   0   0   64   ABCDEF_85855   305   UniRef50_Q8HBW0   40   92.50   40   3   0   205   86   1   40   5e-19   80.9
**********Hit in genome EU118126****************
ABCDEF_85855   305   EU118126   162046   100.00   305   0   0   1   305   46009   46313   3e-175   605
**********Hit in cds ycf3****************
ycf3
ABCDEF_85855   305   ycf3   1999   100.00   206   0   0   1   206   206   1   2e-116   408
codon_start=1 transl_table=11 product="hypothetical chloroplast RF34" protein_id="ABV02349.1"   complement(44215..46213)
//

>ABCDEF_74466
Normal: 0   0   0   0   7   6   ABCDEF_74466   276   UniRef50_Q9T3G4   512   98.63   73   1   0   3   221   253   325   2e-40   146   An
**********Hit in genome EU118126****************
ABCDEF_74466   276   EU118126   162046   99.21   252   2   0   24   275   96902   96651   7e-139   484
ABCDEF_74466   276   EU118126   162046   99.21   252   2   0   24   275   153317   153568   7e-139   484
ABCDEF_74466   276   EU118126   162046   100.00   23   0   0   1   23   97596   97574   6e-07   46.1
ABCDEF_74466   276   EU118126   162046   100.00   23   0   0   1   23   152623   152645   6e-07   46.1
**********Hit in cds ndhB****************
ndhB
ABCDEF_74466   276   ndhB   2198   99.21   252   2   0   24   275   1448   1699   4e-139   484
ABCDEF_74466   276   ndhB   2198   100.00   23   0   0   1   23   754   776   3e-07   46.1
codon_start=1 transl_table=11 product="NADH-plastoquinone oxidoreductase subunit 2" protein_id="ABV02392.1" codon_start=1 transl_table=11 product="NADH-plastoquinone oxidoreductase subunit 2" prot
//
```

```
>ABCDEF_49598
Normal: 0	44	34	7	17	157		ABCDEF_49598	861	UniRef50_Q7V510		776	68.18	88	28	0	265	2	20	107	6e-35	140
**********Hit in genome EU118126****************
ABCDEF_49598	861	EU118126	162046	100.00	861	0	0	1	861	43124	43984	0.0	1707
**********Hit in cds psaA****************
psaA
ABCDEF_49598	861	psaA		2253	100.00	305	0	0	1	305	305	1	5e-175	605
codon_start=1 transl_table=11 product="photosystem I P700 apoprotein A1" protein_id="ABV02348.1"	complement(41175..43427)
//

>ABCDEF_82650
Normal: 0	0	0	0	0	16		ABCDEF_82650	268	UniRef50_Q1ACE5		159	68.75	64	20	0	76	267	1	64	3e-27	104
**********Hit in genome EU118126****************
ABCDEF_82650	268	EU118126	162046	100.00	268	0	0	1	268	129111	128844	3e-153	531
**********Hit in cds ndhI****************
ndhI
ABCDEF_82650	268	ndhI		504	100.00	194	0	0	75	268	1	194	3e-109	385
codon_start=1 transl_table=11 product="NADH-plastoquinone oxidoreductase subunit I" protein_id="ABV02405.1"	complement(128533..129036)
//

>ABCDEF_12288
Normal: 7	14	17	7	0	238	No annotation
**********Hit in genome EU118126****************
ABCDEF_12288	561	EU118126	162046	100.00	561	0	0	1	561	119601	119041	0.0	1112
*************no hit in cds*****************
//

>ABCDEF_80335
Normal: 0	16	10	0	20	158	No annotation
**********Hit in genome EU118126****************
ABCDEF_80335	208	EU118126	162046	100.00	208	0	0	1	208	30825	31032	2e-117	412
*************no hit in cds*****************
//

>ABCDEF_16731
Normal: 7495	35030	12253	30038	12766	103299		ABCDEF_16731	7557	UniRef50_Q5W7C9		265	95.28	127	6	0	1049	669	3	129	3e-60	218
**********Hit in genome EU118126****************
ABCDEF_16731	7557	EU118126	162046	99.91	7533	2	5	1	7528	101625	109157	0.0	1.484e+04
ABCDEF_16731	7557	EU118126	162046	99.91	7533	2	5	1	7528	148594	141062	0.0	1.484e+04
**********Hit in cds rrn23****************
rrn23
ABCDEF_16731	7557	rrn23		2810	99.96	2805	0	1	4105	6908	6	2810	0.0	5545
complement(141681..144490)
//

>ABCDEF_38059
Normal: 7449	20106	6978	6819	10389	50205		ABCDEF_38059	3486	UniRef50_Q9C5A9		559	69.43	471	139	3	3274	1868	83	550	0.0	651
**********Hit in genome EU118126****************
ABCDEF_38059	3486	EU118126	162046	99.97	3487	0	1	1	3486	53290	56776	0.0	6897
**********Hit in cds atpB****************
atpB
ABCDEF_38059	3486	atpB		1500	99.93	1500	0	1	1825	3323	1500	1	0.0	2958
codon_start=1 transl_table=11 product="ATP synthase CF1 beta subunit" protein_id="ABV02355.1"	complement(55113..56612)
//

>ABCDEF_16495
Normal: 2566	5068	3021	1730	3374	31924		ABCDEF_16495	3492	UniRef50_Q06FK5		141	73.53	68	18	0	2830	2627	40	107	2e-19	94.0
**********Hit in genome EU118126****************
ABCDEF_16495	3492	EU118126	162046	99.97	3492	1	0	1	3492	73285	69794	0.0	6914
**********Hit in cds clpP****************
clpP
ABCDEF_16495	3492	clpP		2028	99.88	813	1	0	1	813	1216	2028	0.0	1604
codon_start=1 transl_table=11 product="clp protease proteolytic subunit" protein_id="ABV02373.1"	complement(72472..74499)
//

>ABCDEF_20092
Normal: 1302	2835	2547	1678	1530	10122		ABCDEF_20092	5651	UniRef50_Q85UK5		166	77.11	166	38	0	3570	3073	1	166	8e-76	258
**********Hit in genome EU118126****************
ABCDEF_20092	5651	EU118126	162046	100.00	2910	0	0	2742	5651	85279	88188	0.0	5769
ABCDEF_20092	5651	EU118126	162046	100.00	2741	0	0	1	2741	81522	84262	0.0	5434
```

**********Hit in cds rpl2****************
rpl2
ABCDEF_20092 5651 rpl2 825 100.00 825 0 0 4431 5255 825 1 0.0 1635
codon_start=1 transl_table=11 product="ribosomal protein L2" protein_id="ABV02389.1" complement(86967..87791)
//

>ABCDEF_12303
Normal: 19 51 64 15 2 194 ABCDEF_12303 499 UniRef50_Q8M9V7 334 46.06 165 89 0 499 5 55 219 5e-48 166
**********Hit in genome EU118126****************
ABCDEF_12303 499 EU118126 162046 99.80 499 1 0 1 499 80831 81329 0.0 981
**********Hit in cds rpoA****************
rpoA
ABCDEF_12303 499 rpoA 1014 99.80 499 1 0 1 499 662 164 0.0 981
codon_start=1 transl_table=11 product="RNA polymerase alpha subunit" protein_id="ABV02380.1" complement(80478..81491)
//

>ABCDEF_41159
Normal: 801 1955 1009 763 1180 9265 ABCDEF_41159 1274 UniRef50_Q9M3M7 83 67.86 56 17 1 81 248 1 55 5e-10 62.8
**********Hit in genome EU118126****************
ABCDEF_41159 1274 EU118126 162046 100.00 1274 0 0 1 1274 32182 30909 0.0 2526
**********Hit in cds psbM****************
psbM
ABCDEF_41159 1274 psbM 105 100.00 105 0 0 998 1102 1 105 2e-55 208
codon_start=1 transl_table=11 product="photosystem II protein M" protein_id="ABV02342.1" complement(31080..31184)
//

>ABCDEF_8496
Normal: 36 56 34 15 35 1366 No annotation
**********Hit in genome EU118126****************
ABCDEF_8496 400 EU118126 162046 100.00 400 0 0 1 400 9861 9462 0.0 793
ABCDEF_8496 400 EU118126 162046 96.30 27 1 0 10 36 38030 38004 8e-07 46.1
**********Hit in cds trnG-UCC****************
trnG-UCC
ABCDEF_8496 400 trnG-UCC 48 100.00 48 0 0 8 55 48 1 5e-22 95.6
9806..9853
//

>ABCDEF_20094
Normal: 1042 2249 2087 1236 1206 8780 ABCDEF_20094 3800 UniRef50_Q85UK5 166 77.11 166 38 0 1718 1221 1 166 1e-76 258
**********Hit in genome EU118126****************
ABCDEF_20094 3800 EU118126 162046 99.97 3800 0 1 1 3800 84390 88188 0.0 7517
ABCDEF_20094 3800 EU118126 162046 88.89 63 7 0 383 445 63 1 6e-13 69.9
**********Hit in cds rpl16****************
rpl16
ABCDEF_20094 3800 rpl16 1460 100.00 899 0 0 1 899 899 1 0.0 1782
codon_start=1 transl_table=11 product="ribosomal protein L16" protein_id="ABV02385.1" complement(83828..85287)
//

>ABCDEF_8255
Normal: 817 2782 1484 1071 1524 7908 ABCDEF_8255 877 UniRef50_P56769 44 93.18 44 3 0 460 329 1 44 8e-19 84.3
**********Hit in genome EU118126****************
ABCDEF_8255 877 EU118126 162046 99.89 877 1 0 1 877 69905 69029 0.0 1731
ABCDEF_8255 877 EU118126 162046 95.74 47 1 1 770 816 69067 69022 1e-13 69.9
**********Hit in cds psaJ****************
psaJ
ABCDEF_8255 877 psaJ 135 100.00 135 0 0 325 459 135 1 1e-73 268
codon_start=1 transl_table=11 product="photosystem I subunit IX" protein_id="ABV02368.1" 69446..69580
//

>ABCDEF_73115
Normal: 10 31 34 31 4 312 ABCDEF_73115 353 UniRef50_Q32992 67 70.45 44 13 0 317 186 24 67 7e-11 60.5
**********Hit in genome EU118126****************
ABCDEF_73115 353 EU118126 162046 100.00 314 0 0 1 314 104859 105172 2e-180 622
ABCDEF_73115 353 EU118126 162046 100.00 314 0 0 1 314 145360 145047 2e-180 622
ABCDEF_73115 353 EU118126 162046 100.00 48 0 0 306 353 105089 105136 9e-22 95.6
ABCDEF_73115 353 EU118126 162046 100.00 48 0 0 306 353 145130 145083 9e-22 95.6
*************no hit in cds*****************
//

>ABCDEF_12792

```
Normal: 603	2221	985	577	888	6477		ABCDEF_12792	5038	UniRef50_Q93SW9		333	55.15	330	131	4	4773	3835	4	333	6e-111	363
**********Hit in genome EU118126****************
ABCDEF_12792	5038	EU118126	162046	99.96	5038	2	0	1	5038	69046	64009	0.0	9971
**********Hit in cds petA****************
petA
ABCDEF_12792	5038	petA	963	100.00	963	0	0	3831	4793	963	1	0.0	1909
codon_start=1 transl_table=11 product="cytochrome f" protein_id="ABV02361.1"64253..65215
//

>ABCDEF_16712
Normal: 416	2590	1018	591	818	6251		ABCDEF_16712	4554	UniRef50_UPI00019842D3	358	92.50	160	10	1	1996	1517	20	177	5e-93	312
**********Hit in genome EU118126****************
ABCDEF_16712	4554	EU118126	162046	99.87	4554	3	2	1	4554	37906	33356	0.0	8966
**********Hit in cds psbC****************
psbC
ABCDEF_16712	4554	psbC	1422	100.00	1422	0	0	1099	2520	1422	1	0.0	2819
codon_start=1 transl_table=11 product="photosystem II CP43 chlorophyll apoprotein" protein_id="ABV02344.1" 35387..36808
//

>ABCDEF_25914
Normal: 27	74	44	27	41	1601 No annotation
**********Hit in genome EU118126****************
ABCDEF_25914	799	EU118126	162046	100.00	799	0	0	1	799	8633	9431	0.0	1584
*************no hit in cds*****************
//

>ABCDEF_28404
Normal: 88	219	197	93	51	2649		ABCDEF_28404	726	UniRef50_A4QM70		49	64.29	42	15	0	221	96	3	44	2e-09	57.8
**********Hit in genome EU118126****************
ABCDEF_28404	726	EU118126	162046	100.00	724	0	0	1	724	49987	49264	0.0	1435
**********Hit in cds trnL-UAA****************
trnL-UAA
ABCDEF_28404	726	trnL-UAA	50	100.00	50	0	0	103	152	50	1	6e-23	99.6
49835..49884
//

>ABCDEF_6665
Normal: 363	1495	714	290	657	5105		ABCDEF_6665	3324	UniRef50_Q4JUJ8		545	56.20	468	197	1	1383	4	4	471	7e-167	512
**********Hit in genome EU118126****************
ABCDEF_6665	3324	EU118126	162046	99.95	1854	1	0	1	1854	10751	12604	0.0	3667
ABCDEF_6665	3324	EU118126	162046	99.93	1472	1	0	1853	3324	13300	14771	0.0	2910
**********Hit in cds atpA****************
atpA
ABCDEF_6665	3324	atpA	1524	99.93	1387	1	0	1	1387	1387	1	0.0	2742
codon_start=1 transl_table=11 product="ATP synthase CF1 alpha subunit" protein_id="ABV02333.1"	complement(10613..12136)
//

>ABCDEF_11286
Normal: 1076	1004	1081	713	1065	4939		ABCDEF_11286	1177	UniRef50_Q9M3J5		1833	48.93	233	110	4	1060	374	1603	1830	6e-47	180
**********Hit in genome EU118126****************
ABCDEF_11286	1177	EU118126	162046	100.00	1177	0	0	1	1177	116977	115801	0.0	2333
ABCDEF_11286	1177	EU118126	162046	100.00	1177	0	0	1	1177	133242	134418	0.0	2333
ABCDEF_11286	1177	EU118126	162046	100.00	96	0	0	1082	1177	115848	115753	7e-50	190
ABCDEF_11286	1177	EU118126	162046	100.00	96	0	0	1082	1177	115872	115777	7e-50	190
ABCDEF_11286	1177	EU118126	162046	100.00	96	0	0	1082	1177	134347	134442	7e-50	190
ABCDEF_11286	1177	EU118126	162046	100.00	96	0	0	1082	1177	134371	134466	7e-50	190
ABCDEF_11286	1177	EU118126	162046	98.95	95	1	0	1082	1176	115824	115730	7e-47	180
ABCDEF_11286	1177	EU118126	162046	98.95	95	1	0	1082	1176	134395	134489	7e-47	180
ABCDEF_11286	1177	EU118126	162046	96.84	95	3	0	1082	1176	115800	115706	4e-42	165
ABCDEF_11286	1177	EU118126	162046	96.84	95	3	0	1082	1176	134419	134513	4e-42	165
ABCDEF_11286	1177	EU118126	162046	95.12	82	4	0	1082	1163	115776	115695	6e-32	131
ABCDEF_11286	1177	EU118126	162046	95.12	82	4	0	1082	1163	134443	134524	6e-32	131
**********Hit in cds ycf1****************
ycf1
ABCDEF_11286	1177	ycf1	6174	100.00	824	0	0	354	1177	6174	5351	0.0	1633
ABCDEF_11286	1177	ycf1	6174	100.00	96	0	0	1082	1177	5398	5303	4e-50	190
ABCDEF_11286	1177	ycf1	6174	100.00	96	0	0	1082	1177	5422	5327	4e-50	190
ABCDEF_11286	1177	ycf1	6174	98.95	95	1	0	1082	1176	5374	5280	3e-47	180
ABCDEF_11286	1177	ycf1	6174	96.84	95	3	0	1082	1176	5350	5256	2e-42	165
ABCDEF_11286	1177	ycf1	6174	95.12	82	4	0	1082	1163	5326	5245	3e-32	131
```

codon_start=1 transl_table=11 product="hypothetical chloroplast RF19" protein_id="ABV02395.1" codon_start=1 transl_table=11 product="hypothetical chloroplast RF19" protein_id="ABV02409.1" compleme
//

>ABCDEF_38101
Normal: 193 868 327 479 300 4925 ABCDEF_38101 1900 UniRef50_Q95EV1 468 95.09 468 23 0 280 1683 1 468 0.0 895
**********Hit in genome EU118126****************
ABCDEF_38101 1900 EU118126 162046 100.00 1900 0 0 1 1900 56769 58668 0.0 3766
**********Hit in cds rbcL****************
rbcL
ABCDEF_38101 1900 rbcL 1443 100.00 1443 0 0 260 1702 1 1443 0.0 2861
codon_start=1 transl_table=11 product="ribulose 1,5-bisphosphate carboxylase/oxygenase protein_id="ABV02356.1" 57027..58469
//

>ABCDEF_52796
Normal: 50 123 106 50 28 1378 No annotation
**********Hit in genome EU118126****************
ABCDEF_52796 685 EU118126 162046 100.00 685 0 0 1 685 28404 29088 0.0 1358
**********Hit in cds trnC-GCA****************
trnC-GCA
ABCDEF_52796 685 trnC-GCA 72 100.00 72 0 0 397 468 1 72 4e-36 143
28799..28870
//

>ABCDEF_49719
Normal: 9 72 18 7 6 291 No annotation
**********Hit in genome EU118126****************
ABCDEF_49719 760 EU118126 162046 100.00 760 0 0 1 760 79666 78907 0.0 1507
**********Hit in cds petD****************
petD
ABCDEF_49719 760 petD 1183 100.00 554 0 0 1 554 554 1 0.0 1098
codon_start=1 transl_table=11 product="cytochrome b6/f complex subunit IV" protein_id="ABV02379.1" 79112..80294
//

>ABCDEF_44395
Normal: 938 420 1008 400 722 4098 ABCDEF_44395 2042 UniRef50_Q2L951 1892 47.35 245 113 7 1905 1207 14 254 2e-49 194
**********Hit in genome EU118126****************
ABCDEF_44395 2042 EU118126 162046 100.00 2035 0 0 8 2042 112373 110339 0.0 4034
ABCDEF_44395 2042 EU118126 162046 100.00 2035 0 0 8 2042 137846 139880 0.0 4034
ABCDEF_44395 2042 EU118126 162046 98.41 63 1 0 1 63 112785 112723 1e-27 117
ABCDEF_44395 2042 EU118126 162046 98.41 63 1 0 1 63 137434 137496 1e-27 117
ABCDEF_44395 2042 EU118126 162046 98.39 62 1 0 1 62 112830 112769 6e-27 115
ABCDEF_44395 2042 EU118126 162046 98.39 62 1 0 1 62 137389 137450 6e-27 115
ABCDEF_44395 2042 EU118126 162046 96.77 62 2 0 1 62 112650 112589 1e-24 107
ABCDEF_44395 2042 EU118126 162046 96.77 62 2 0 1 62 137569 137630 1e-24 107
ABCDEF_44395 2042 EU118126 162046 95.24 63 3 0 1 63 112560 112498 9e-23 101
ABCDEF_44395 2042 EU118126 162046 95.24 63 3 0 1 63 112695 112633 9e-23 101
ABCDEF_44395 2042 EU118126 162046 95.24 63 3 0 1 63 137524 137586 9e-23 101
ABCDEF_44395 2042 EU118126 162046 95.24 63 3 0 1 63 137659 137721 9e-23 101
ABCDEF_44395 2042 EU118126 162046 95.16 62 3 0 1 62 112425 112364 3e-22 99.6
ABCDEF_44395 2042 EU118126 162046 95.16 62 3 0 1 62 137794 137855 3e-22 99.6
ABCDEF_44395 2042 EU118126 162046 94.64 56 3 0 8 63 112598 112543 1e-18 87.7
ABCDEF_44395 2042 EU118126 162046 94.64 56 3 0 8 63 137621 137676 1e-18 87.7
ABCDEF_44395 2042 EU118126 162046 92.06 63 5 0 1 63 112470 112408 5e-18 85.7
ABCDEF_44395 2042 EU118126 162046 92.06 63 5 0 1 63 137749 137811 5e-18 85.7
ABCDEF_44395 2042 EU118126 162046 100.00 41 0 0 2002 2042 110243 110203 8e-17 81.8
ABCDEF_44395 2042 EU118126 162046 100.00 41 0 0 2002 2042 110311 110271 8e-17 81.8
ABCDEF_44395 2042 EU118126 162046 100.00 41 0 0 2002 2042 139908 139948 8e-17 81.8
ABCDEF_44395 2042 EU118126 162046 100.00 41 0 0 2002 2042 139976 140016 8e-17 81.8
ABCDEF_44395 2042 EU118126 162046 92.86 56 4 0 8 63 112508 112453 3e-16 79.8
ABCDEF_44395 2042 EU118126 162046 92.86 56 4 0 8 63 137711 137766 3e-16 79.8
ABCDEF_44395 2042 EU118126 162046 92.73 55 4 0 8 62 112733 112679 1e-15 77.8
ABCDEF_44395 2042 EU118126 162046 92.73 55 4 0 8 62 137486 137540 1e-15 77.8
**********Hit in cds ycf1****************
ycf1
ABCDEF_44395 2042 ycf1 6174 100.00 1923 0 0 8 1930 1923 1 0.0 3812
ABCDEF_44395 2042 ycf1 6174 98.41 63 1 0 1 63 2335 2273 8e-28 117
ABCDEF_44395 2042 ycf1 6174 98.39 62 1 0 1 62 2380 2319 3e-27 115
ABCDEF_44395 2042 ycf1 6174 96.77 62 2 0 1 62 2200 2139 7e-25 107
ABCDEF_44395 2042 ycf1 6174 95.24 63 3 0 1 63 2110 2048 4e-23 101
ABCDEF_44395 2042 ycf1 6174 95.24 63 3 0 1 63 2245 2183 4e-23 101

```
ABCDEF_44395   2042    ycf1           6174    95.16   62      3       0       1       62      1975    1914    2e-22   99.6
ABCDEF_44395   2042    ycf1           6174    94.64   56      3       0       8       63      2148    2093    7e-19   87.7
ABCDEF_44395   2042    ycf1           6174    92.06   63      5       0       1       63      2020    1958    3e-18   85.7
ABCDEF_44395   2042    ycf1           6174    92.86   56      4       0       8       63      2058    2003    2e-16   79.8
ABCDEF_44395   2042    ycf1           6174    92.73   55      4       0       8       62      2283    2229    6e-16   77.8
codon_start=1 transl_table=11 product="hypothetical chloroplast RF19" protein_id="ABV02395.1" codon_start=1 transl_table=11 product="hypothetical chloroplast RF19" protein_id="ABV02409.1"    compleme
//

>ABCDEF_25574
Normal: 39      42      28      7       51      280   No annotation
**********Hit in genome EU118126****************
ABCDEF_25574   396     EU118126       162046  100.00  396     0       0       1       396     122362  121967  0.0     785
*************no hit in cds*****************
//

>ABCDEF_17969
Normal: 59      89      176     20      28      798           ABCDEF_17969        1479    UniRef50_Q85C71          1445    31.37   459     291     12      1440    76      493     931     2e-49   190
**********Hit in genome EU118126****************
ABCDEF_17969   1479    EU118126       162046  99.93   1479    1       0       1       1479    18333   19811   0.0     2924
**********Hit in cds rpoC2****************
rpoC2
ABCDEF_17969   1479    rpoC2          4206    99.93   1479    1       0       1       1479    2929    1451    0.0     2924
codon_start=1 transl_table=11 product="RNA polymerase beta' subunit" protein_id="ABV02338.1"       complement(17055..21260)
//

>ABCDEF_16493
Normal: 285     690     423     311     279     3924          ABCDEF_16493        4972    UniRef50_Q9T3G4          512     96.14   259     10      0       2687    3463    1       259     1e-149  477
**********Hit in genome EU118126****************
ABCDEF_16493   4972    EU118126       162046  99.41   3882    21      2       1091    4972    99944   96065   0.0     7497
ABCDEF_16493   4972    EU118126       162046  99.41   3882    21      2       1091    4972    150275  154154  0.0     7497
ABCDEF_16493   4972    EU118126       162046  99.91   1090    1       0       1       1090    73285   72196   0.0     2153
**********Hit in cds ndhB****************
ndhB
ABCDEF_16493   4972    ndhB           2198    99.59   2198    9       0       2688    4885    1       2198    0.0     4286
codon_start=1 transl_table=11 product="NADH-plastoquinone oxidoreductase subunit 2" protein_id="ABV02392.1" codon_start=1 transl_table=11 product="NADH-plastoquinone oxidoreductase subunit 2" prot
//

>ABCDEF_883
Normal: 821     379     1182    687     918     3696          ABCDEF_883          1441    UniRef50_Q7YJS6          1828    49.67   457     194     13      141     1439    668     1112    6e-101  340
**********Hit in genome EU118126****************
ABCDEF_883     1441    EU118126       162046  99.65   1441    5       0       1       1441    112733  114173  0.0     2817
ABCDEF_883     1441    EU118126       162046  99.65   1441    5       0       1       1441    137486  136046  0.0     2817
ABCDEF_883     1441    EU118126       162046  99.00   100     1       0       1       100     112688  112787  8e-50   190
ABCDEF_883     1441    EU118126       162046  99.00   100     1       0       1       100     137531  137432  8e-50   190
ABCDEF_883     1441    EU118126       162046  95.92   98      4       0       3       100     112465  112562  2e-41   163
ABCDEF_883     1441    EU118126       162046  95.92   98      4       0       3       100     137754  137657  2e-41   163
ABCDEF_883     1441    EU118126       162046  95.00   100     5       0       1       100     112598  112697  3e-40   159
ABCDEF_883     1441    EU118126       162046  95.00   100     5       0       1       100     137621  137522  3e-40   159
ABCDEF_883     1441    EU118126       162046  93.88   98      6       0       3       100     112555  112652  1e-36   147
ABCDEF_883     1441    EU118126       162046  93.88   98      6       0       3       100     137664  137567  1e-36   147
ABCDEF_883     1441    EU118126       162046  93.00   100     7       0       1       100     112328  112427  2e-35   143
ABCDEF_883     1441    EU118126       162046  93.00   100     7       0       1       100     137891  137792  2e-35   143
ABCDEF_883     1441    EU118126       162046  92.00   100     8       0       1       100     112373  112472  4e-33   135
ABCDEF_883     1441    EU118126       162046  92.00   100     8       0       1       100     112508  112607  4e-33   135
ABCDEF_883     1441    EU118126       162046  92.00   100     8       0       1       100     137711  137612  4e-33   135
ABCDEF_883     1441    EU118126       162046  92.00   100     8       0       1       100     137846  137747  4e-33   135
ABCDEF_883     1441    EU118126       162046  91.84   98      8       0       3       100     112420  112517  7e-32   131
ABCDEF_883     1441    EU118126       162046  91.84   98      8       0       3       100     137799  137702  7e-32   131
ABCDEF_883     1441    EU118126       162046  90.82   98      9       0       3       100     112645  112742  2e-29   123
ABCDEF_883     1441    EU118126       162046  90.82   98      9       0       3       100     137574  137477  2e-29   123
ABCDEF_883     1441    EU118126       162046  96.92   65      2       0       36      100     112318  112382  2e-26   113
ABCDEF_883     1441    EU118126       162046  96.92   65      2       0       36      100     137901  137837  2e-26   113
**********Hit in cds ycf1****************
ycf1
ABCDEF_883     1441    ycf1           6174    99.65   1441    5       0       1       1441    2283    3723    0.0     2817
ABCDEF_883     1441    ycf1           6174    99.00   100     1       0       1       100     2238    2337    4e-50   190
ABCDEF_883     1441    ycf1           6174    95.92   98      4       0       3       100     2015    2112    1e-41   163
ABCDEF_883     1441    ycf1           6174    95.00   100     5       0       1       100     2148    2247    2e-40   159
ABCDEF_883     1441    ycf1           6174    93.88   98      6       0       3       100     2105    2202    6e-37   147
ABCDEF_883     1441    ycf1           6174    93.00   100     7       0       1       100     1878    1977    9e-36   143
```

```
ABCDEF_883     1441    ycf1           6174    92.00   100     8       0       1       100     1923    2022    2e-33   135
ABCDEF_883     1441    ycf1           6174    92.00   100     8       0       1       100     2058    2157    2e-33   135
ABCDEF_883     1441    ycf1           6174    91.84   98      8       0       3       100     1970    2067    4e-32   131
ABCDEF_883     1441    ycf1           6174    90.82   98      9       0       3       100     2195    2292    9e-30   123
ABCDEF_883     1441    ycf1           6174    96.92   65      2       0       36      100     1868    1932    8e-27   113
codon_start=1 transl_table=11 product="hypothetical chloroplast RF19" protein_id="ABV02395.1" codon_start=1 transl_table=11 product="hypothetical chloroplast RF19" protein_id="ABV02409.1"    compleme
//

>ABCDEF_45495
Normal: 21      52      59      12      15      418         ABCDEF_45495        1062    UniRef50_Q7YLQ7         212     58.64   220     54      4       892     320     1       212     6e-72   231
**********Hit in genome EU118126****************
ABCDEF_45495   1062    EU118126       162046  100.00  1062    0       0       1       1062    47127   48188   0.0     2105
**********Hit in cds rps4****************
rps4
ABCDEF_45495   1062    rps4           612     100.00  612     0       0       315     926     612     1       0.0     1213
codon_start=1 transl_table=11 product="ribosomal protein S4" protein_id="ABV02350.1"        complement(47440..48051)
//

>ABCDEF_11857
Normal: 19      57      19      10      41      338         ABCDEF_11857        1027    UniRef50_Q1ACN8         29      86.21   29      4       0       941     855     1       29      3e-06   50.4
**********Hit in genome EU118126****************
ABCDEF_11857   1027    EU118126       162046  100.00  1027    0       0       1       1027    30739   29713   0.0     2036
**********Hit in cds petN****************
petN
ABCDEF_11857   1027    petN           90      100.00  90      0       0       851     940     90      1       1e-46   178
codon_start=1 transl_table=11 product="cytochrome b6/f complex subunit VIII" protein_id="ABV02341.1"       29799..29888
//

>ABCDEF_3422
Normal: 318     499     590     258     402     3225        ABCDEF_3422         1540    UniRef50_Q85FT1         158     64.00   75      27      0       332     556     43      117     1e-21   98.2
**********Hit in genome EU118126****************
ABCDEF_3422    1540    EU118126       162046  99.87   1540    2       0       1       1540    45694   44155   0.0     3037
ABCDEF_3422    1540    EU118126       162046  100.00  42      0       0       252     293     100025  99984   2e-17   83.8
ABCDEF_3422    1540    EU118126       162046  100.00  42      0       0       252     293     150194  150235  2e-17   83.8
ABCDEF_3422    1540    EU118126       162046  97.62   42      1       0       255     296     129735  129694  4e-15   75.8
**********Hit in cds ycf3****************
ycf3
ABCDEF_3422    1540    ycf3           1999    99.86   1479    2       0       1       1479    521     1999    0.0     2916
codon_start=1 transl_table=11 product="hypothetical chloroplast RF34" protein_id="ABV02349.1"      complement(44215..46213)
//

>ABCDEF_19547
Normal: 19      39      15      7       15      215         ABCDEF_19547        709     UniRef50_Q8LYW3         370     36.11   108     56      4       350     42      232     331     3e-08   58.9
**********Hit in genome EU118126****************
ABCDEF_19547   709     EU118126       162046  100.00  709     0       0       1       709     80284   79576   0.0     1405
**********Hit in cds petD****************
petD
ABCDEF_19547   709     petD           1183    100.00  709     0       0       1       709     1172    464     0.0     1405
codon_start=1 transl_table=11 product="cytochrome b6/f complex subunit IV" protein_id="ABV02379.1" 79112..80294
//

>ABCDEF_45324
Normal: 48      336     114     128     114     1390        ABCDEF_45324        1361    UniRef50_Q9F721         428     40.35   228     130     4       471     1148    68      291     8e-50   182
**********Hit in genome EU118126****************
ABCDEF_45324   1361    EU118126       162046  99.85   1344    2       0       1       1344    77776   79119   0.0     2648
**********Hit in cds petB****************
petB
ABCDEF_45324   1361    petB           1401    99.82   1131    2       0       1       1131    271     1401    0.0     2226
codon_start=1 transl_table=11 product="cytochrome b6" protein_id="ABV02378.1"       77505..78905
//

>ABCDEF_22271
Normal: 9       29      50      32      12      260  No annotation
**********Hit in genome EU118126****************
ABCDEF_22271   667     EU118126       162046  99.85   667     1       0       1       667     5383    6049    0.0     1314
**********Hit in cds rps16****************
rps16
ABCDEF_22271   667     rps16          1071    99.85   667     1       0       1       667     782     116     0.0     1314
codon_start=1 transl_table=11 product="ribosomal protein S16" protein_id="ABV02330.1"      complement(5093..6163)
//
```

```
>ABCDEF_3420
Normal: 298	474	544	234	373	2770	ABCDEF_3420	1606	UniRef50_Q8HBW0	40	92.50	40	3	0	134	253	1	40	1e-16	81.3
**********Hit in genome EU118126****************
ABCDEF_3420	1606	EU118126	162046	99.93	1349	1	0	258	1606	45365	44017	0.0	2666
ABCDEF_3420	1606	EU118126	162046	100.00	258	0	0	1	258	46346	46089	2e-146	511
**********Hit in cds ycf3****************
ycf3
ABCDEF_3420	1606	ycf3	1999	99.91	1150	1	0	258	1407	850	1999	0.0	2272
ABCDEF_3420	1606	ycf3	1999	100.00	126	0	0	133	258	1	126	6e-68	250
codon_start=1 transl_table=11 product="hypothetical chloroplast RF34" protein_id="ABV02349.1"	complement(44215..46213)
//

>ABCDEF_28359
Normal: 20	32	61	7	12	193	ABCDEF_28359	709	UniRef50_Q2JP53	159	50.00	76	38	0	31	258	1	76	7e-17	81.3
**********Hit in genome EU118126****************
ABCDEF_28359	709	EU118126	162046	100.00	639	0	0	71	709	5313	4675	0.0	1267
ABCDEF_28359	709	EU118126	162046	100.00	71	0	0	1	71	6193	6123	3e-35	141
**********Hit in cds rps16****************
rps16
ABCDEF_28359	709	rps16	1071	100.00	220	0	0	71	290	852	1071	2e-124	436
ABCDEF_28359	709	rps16	1071	100.00	42	0	0	30	71	1	42	4e-18	83.8
codon_start=1 transl_table=11 product="ribosomal protein S16" protein_id="ABV02330.1"	complement(5093..6163)
//

>ABCDEF_7069
Normal: 286	578	544	284	330	2744	ABCDEF_7069	1935	UniRef50_Q49CB5	1815	53.47	202	70	6	1302	709	8	189	1e-39	163
**********Hit in genome EU118126****************
ABCDEF_7069	1935	EU118126	162046	100.00	1490	0	0	1	1490	89950	88461	0.0	2954
ABCDEF_7069	1935	EU118126	162046	100.00	1490	0	0	1	1490	160269	161758	0.0	2954
ABCDEF_7069	1935	EU118126	162046	98.99	297	3	0	1432	1728	88469	88173	2e-162	565
ABCDEF_7069	1935	EU118126	162046	98.99	297	3	0	1432	1728	161750	162046	2e-162	565
ABCDEF_7069	1935	EU118126	162046	100.00	206	0	0	1729	1934	1	206	3e-115	408
ABCDEF_7069	1935	EU118126	162046	89.39	66	7	0	1726	1791	84837	84772	5e-15	75.8
ABCDEF_7069	1935	EU118126	162046	88.64	44	0	1	1390	1428	88476	88433	1e-06	48.1
ABCDEF_7069	1935	EU118126	162046	88.64	44	0	1	1390	1428	161743	161786	1e-06	48.1
**********Hit in cds ycf2****************
ycf2
ABCDEF_7069	1935	ycf2	6594	100.00	1345	0	0	1	1345	1345	1	0.0	2666
codon_start=1 transl_table=11 product="hypothetical chloroplast RF21" protein_id="ABV02391.1" codon_start=1 transl_table=11 product="hypothetical chloroplast RF21" protein_id="ABV02414.1"	compleme
//

>ABCDEF_45325
Normal: 18	88	28	57	12	329	ABCDEF_45325	995	UniRef50_Q9F721	428	41.78	225	125	4	45	713	71	291	6e-52	185
**********Hit in genome EU118126****************
ABCDEF_45325	995	EU118126	162046	99.77	857	2	0	53	909	78263	79119	0.0	1683
ABCDEF_45325	995	EU118126	162046	100.00	87	0	0	909	995	79819	79905	1e-44	172
ABCDEF_45325	995	EU118126	162046	100.00	53	0	0	1	53	77458	77510	3e-24	105
**********Hit in cds petB****************
petB
ABCDEF_45325	995	petB	1401	99.69	644	2	0	53	696	758	1401	0.0	1261
codon_start=1 transl_table=11 product="cytochrome b6" protein_id="ABV02378.1"	77505..78905
//

>ABCDEF_75073
Normal: 303	678	520	368	303	2730	ABCDEF_75073	1205	UniRef50_Q14EM8	171	74.56	169	43	0	670	164	1	169	2e-75	240
**********Hit in genome EU118126****************
ABCDEF_75073	1205	EU118126	162046	100.00	1175	0	0	31	1205	87014	88188	0.0	2329
ABCDEF_75073	1205	EU118126	162046	100.00	39	0	0	1	39	87035	87073	7e-16	77.8
**********Hit in cds rpl2****************
rpl2
ABCDEF_75073	1205	rpl2	825	100.00	779	0	0	31	809	779	1	0.0	1544
ABCDEF_75073	1205	rpl2	825	100.00	39	0	0	1	39	758	720	4e-16	77.8
codon_start=1 transl_table=11 product="ribosomal protein L2" protein_id="ABV02389.1"	complement(86967..87791)
//

>ABCDEF_16494
Normal: 221	378	300	253	254	2640	ABCDEF_16494	2234	UniRef50_Q8DI44	156	53.69	149	68	1	1381	1827	8	155	2e-47	171
**********Hit in genome EU118126****************
ABCDEF_16494	2234	EU118126	162046	100.00	1282	0	0	1	1282	100994	99713	0.0	2541
```

ABCDEF_16494 2234 EU118126 162046 100.00 1282 0 0 1 1282 149225 150506 0.0 2541
ABCDEF_16494 2234 EU118126 162046 99.79 952 0 2 1283 2234 99176 98227 0.0 1855
ABCDEF_16494 2234 EU118126 162046 99.79 952 0 2 1283 2234 151043 151992 0.0 1855
ABCDEF_16494 2234 EU118126 162046 100.00 39 0 0 973 1011 45440 45402 1e-15 77.8
ABCDEF_16494 2234 EU118126 162046 97.44 39 1 0 973 1011 129735 129697 3e-13 69.9
**********Hit in cds rps7****************
rps7
ABCDEF_16494 2234 rps7 468 99.79 469 0 1 1363 1831 1 468 0.0 914
codon_start=1 transl_table=11 product="ribosomal protein S7" protein_id="ABV02393.1" codon_start=1 transl_table=11 product="ribosomal protein S7" protein_id="ABV02412.1" 151122..151589
//

>ABCDEF_42381
Normal: 66 295 208 106 133 1725 ABCDEF_42381 536 UniRef50_Q8YXD6 38 72.22 36 10 0 407 300 1 36 3e-10 58.9
**********Hit in genome EU118126****************
ABCDEF_42381 536 EU118126 162046 100.00 536 0 0 1 536 8440 7905 0.0 1063
ABCDEF_42381 536 EU118126 162046 100.00 28 0 0 134 161 47101 47128 1e-09 56.0
ABCDEF_42381 536 EU118126 162046 93.33 30 2 0 137 166 37081 37052 4e-06 44.1
**********Hit in cds psbI****************
psbI
ABCDEF_42381 536 psbI 111 100.00 111 0 0 296 406 111 1 2e-59 220
codon_start=1 transl_table=11 product="photosystem II protein I" protein_id="ABV02332.1" 8034..8144
//

>ABCDEF_6664
Normal: 187 684 345 99 363 2543 ABCDEF_6664 1899 UniRef50_Q8MA06 189 51.11 45 22 0 547 413 13 57 3e-07 56.6
**********Hit in genome EU118126****************
ABCDEF_6664 1899 EU118126 162046 99.89 1899 1 1 1 1899 12874 14771 0.0 3741
**********Hit in cds atpF****************
atpF
ABCDEF_6664 1899 atpF 1252 99.83 572 1 0 1 572 572 1 0.0 1126
codon_start=1 transl_table=11 product="ATP synthase CF0 subunit I" protein_id="ABV02334.1" complement(12193..13444)
//

>ABCDEF_32123
Normal: 18 37 70 43 15 382 No annotation
**********Hit in genome EU118126****************
ABCDEF_32123 600 EU118126 162046 100.00 600 0 0 1 600 6471 7070 0.0 1189
**********Hit in cds trnQ-UUG****************
trnQ-UUG
ABCDEF_32123 600 trnQ-UUG 72 100.00 47 0 0 554 600 72 26 3e-21 93.7
complement(7023..7094)
//

>ABCDEF_71831
Normal: 36 106 61 76 47 867 ABCDEF_71831 313 UniRef50_Q7Y618 75 93.75 64 4 0 269 78 12 75 9e-33 117
**********Hit in genome EU118126****************
ABCDEF_71831 313 EU118126 162046 98.64 295 4 0 1 295 99286 99580 1e-159 553
ABCDEF_71831 313 EU118126 162046 98.64 295 4 0 1 295 150933 150639 1e-159 553
**********Hit in cds rps12****************
rps12
ABCDEF_71831 313 rps12 908 98.64 295 4 0 1 295 772 478 6e-160 553
trans_splicing codon_start=1 transl_table=11 product="ribosomal protein S12" protein_id="ABV02372.1" trans_splicing codon_start=1 transl_table=11 product="ribosomal protein S12" protein_id="ABV024
//

>ABCDEF_36658
Normal: 19 134 27 26 53 453 ABCDEF_36658 518 UniRef50_Q5SD38 521 56.12 139 61 0 21 437 383 521 1e-46 166
**********Hit in genome EU118126****************
ABCDEF_36658 518 EU118126 162046 100.00 501 0 0 18 518 63613 64113 0.0 993
**********Hit in cds cemA****************
cemA
ABCDEF_36658 518 cemA 690 100.00 424 0 0 18 441 267 690 0.0 841
codon_start=1 transl_table=11 product="chloroplast envelope membrane protein" protein_id="ABV02360.1" 63346..64035
//

>ABCDEF_7068
Normal: 219 498 471 258 263 2376 ABCDEF_7068 877 UniRef50_Q49CB5 1815 64.79 71 14 2 240 28 8 67 7e-15 80.9
**********Hit in genome EU118126****************
ABCDEF_7068 877 EU118126 162046 96.22 741 3 2 1 741 88888 88173 0.0 1277
ABCDEF_7068 877 EU118126 162046 96.22 741 3 2 1 741 161331 162046 0.0 1277
ABCDEF_7068 877 EU118126 162046 100.00 136 0 0 742 877 1 136 7e-74 270

ABCDEF_7068 877 EU118126 162046 89.39 66 7 0 739 804 84837 84772 2e-15 75.8
**********Hit in cds ycf2****************
ycf2
ABCDEF_7068 877 ycf2 6594 100.00 283 0 0 1 283 283 1 7e-162 561
codon_start=1 transl_table=11 product="hypothetical chloroplast RF21" protein_id="ABV02391.1" codon_start=1 transl_table=11 product="hypothetical chloroplast RF21" protein_id="ABV02414.1" compleme
//

>ABCDEF_68539
Normal: 27 93 46 57 33 616 ABCDEF_68539 395 UniRef50_Q85WW1 65 71.05 38 8 1 177 73 1 38 3e-08 53.5
**********Hit in genome EU118126****************
ABCDEF_68539 395 EU118126 162046 98.23 395 1 1 1 395 101711 102099 0.0 729
ABCDEF_68539 395 EU118126 162046 98.23 395 1 1 1 395 148508 148120 0.0 729
**********Hit in cds rrn16****************
rrn16
ABCDEF_68539 395 rrn16 1490 100.00 265 0 0 131 395 1 265 2e-151 525
complement(146894..148383)
//

>ABCDEF_16492
Normal: 214 479 250 161 193 2127 ABCDEF_16492 4424 UniRef50_Q9T3G4 512 96.14 259 10 0 2151 2927 1 259 9e-151 477
**********Hit in genome EU118126****************
ABCDEF_16492 4424 EU118126 162046 99.65 3102 9 2 1323 4424 99176 96077 0.0 6046
ABCDEF_16492 4424 EU118126 162046 99.65 3102 9 2 1323 4424 151043 154142 0.0 6046
ABCDEF_16492 4424 EU118126 162046 99.91 1090 1 0 1 1090 73285 72196 0.0 2153
ABCDEF_16492 4424 EU118126 162046 100.00 232 0 0 1091 1322 99944 99713 2e-130 460
ABCDEF_16492 4424 EU118126 162046 100.00 232 0 0 1091 1322 150275 150506 2e-130 460
**********Hit in cds ndhB****************
ndhB
ABCDEF_16492 4424 ndhB 2198 99.59 2198 9 0 2152 4349 1 2198 0.0 4286
codon_start=1 transl_table=11 product="NADH-plastoquinone oxidoreductase subunit 2" protein_id="ABV02392.1" codon_start=1 transl_table=11 product="NADH-plastoquinone oxidoreductase subunit 2" prot
//

>ABCDEF_8304
Normal: 23 147 40 31 61 449 ABCDEF_8304 721 UniRef50_Q5SD38 521 49.77 217 105 2 2 640 305 521 5e-65 218
**********Hit in genome EU118126****************
ABCDEF_8304 721 EU118126 162046 100.00 721 0 0 1 721 63393 64113 0.0 1429
**********Hit in cds cemA****************
cemA
ABCDEF_8304 721 cemA 690 100.00 644 0 0 1 644 47 690 0.0 1277
codon_start=1 transl_table=11 product="chloroplast envelope membrane protein" protein_id="ABV02360.1" 63346..64035
//

>ABCDEF_6666
Normal: 131 564 209 94 213 1741 ABCDEF_6666 2588 UniRef50_Q4JUJ8 545 54.56 504 219 3 772 2256 4 506 7e-174 523
**********Hit in genome EU118126****************
ABCDEF_6666 2588 EU118126 162046 99.92 2589 1 1 1 2588 12905 10317 0.0 5109
**********Hit in cds atpA****************
atpA
ABCDEF_6666 2588 atpA 1524 99.93 1524 1 0 768 2291 1 1524 0.0 3013
codon_start=1 transl_table=11 product="ATP synthase CF1 alpha subunit" protein_id="ABV02333.1" complement(10613..12136)
//

>ABCDEF_3421
Normal: 39 52 52 27 44 490 ABCDEF_3421 679 UniRef50_Q3EC19 72 47.37 38 16 1 395 294 16 53 8e-08 40.8
**********Hit in genome EU118126****************
ABCDEF_3421 679 EU118126 162046 99.71 678 2 0 2 679 45438 46115 0.0 1328
**********Hit in cds ycf3****************
ycf3
ABCDEF_3421 679 ycf3 1999 99.71 678 2 0 2 679 777 100 0.0 1328
codon_start=1 transl_table=11 product="hypothetical chloroplast RF34" protein_id="ABV02349.1" complement(44215..46213)
//

>ABCDEF_38377
Normal: 88 122 179 139 94 1591 ABCDEF_38377 1963 UniRef50_Q8WKM1 505 64.30 507 174 4 239 1744 1 505 0.0 592
**********Hit in genome EU118126****************
ABCDEF_38377 1963 EU118126 162046 100.00 1963 0 0 1 1963 3869 1907 0.0 3891
**********Hit in cds matK****************
matK
ABCDEF_38377 1963 matK 1509 100.00 1509 0 0 238 1746 1 1509 0.0 2991
codon_start=1 transl_table=11 product="maturase K" protein_id="ABV02329.1" complement(2123..3631)

//

>ABCDEF_52753
Normal: 23 67 35 25 15 269 ABCDEF_52753 813 UniRef50_Q9TKZ3 183 52.87 174 82 0 739 218 10 183 3e-43 153
**********Hit in genome EU118126****************
ABCDEF_52753 813 EU118126 162046 100.00 813 0 0 1 813 62946 62134 0.0 1612
**********Hit in cds ycf4****************
ycf4
ABCDEF_52753 813 ycf4 555 100.00 555 0 0 214 768 555 1 0.0 1100
codon_start=1 transl_table=11 product="photosystem I assembly protein ycf4" protein_id="ABV02359.1" 62178..62732
//

>ABCDEF_10925
Normal: 85 358 118 83 133 1450 ABCDEF_10925 2371 UniRef50_Q8DKZ4 237 68.04 194 62 0 1817 1236 8 201 1e-89 290
**********Hit in genome EU118126****************
ABCDEF_10925 2371 EU118126 162046 99.92 2371 1 1 1 2371 50426 52795 0.0 4676
**********Hit in cds ndhK****************
ndhK
ABCDEF_10925 2371 ndhK 708 99.86 708 1 0 1105 1812 708 1 0.0 1396
codon_start=1 transl_table=11 product="NADH-plastoquinone oxidoreductase subunit K" protein_id="ABV02352.1" complement(51529..52236)
//

>ABCDEF_11535
Normal: 112 176 203 162 159 1725 No annotation
**********Hit in genome EU118126****************
ABCDEF_11535 853 EU118126 162046 100.00 853 0 0 1 853 4714 3862 0.0 1691
**********Hit in cds trnK-UUU****************
trnK-UUU
ABCDEF_11535 853 trnK-UUU 37 100.00 37 0 0 261 297 1 37 4e-15 73.8
complement(4417..4453)
//

>ABCDEF_18781
Normal: 56 184 86 55 84 854 ABCDEF_18781 605 UniRef50_Q9F1K9 46 73.68 38 10 0 281 168 9 46 9e-11 60.8
**********Hit in genome EU118126****************
ABCDEF_18781 605 EU118126 162046 99.83 605 1 0 1 605 7808 7204 0.0 1191
**********Hit in cds psbK****************
psbK
ABCDEF_18781 605 psbK 180 99.44 180 1 0 164 343 180 1 3e-98 349
codon_start=1 transl_table=11 product="photosystem II protein K" protein_id="ABV02331.1" 7465..7644
//

>ABCDEF_39919
Normal: 23 19 16 10 7 104 ABCDEF_39919 1053 UniRef50_Q8M9U5 670 48.72 351 129 7 1048 5 294 596 1e-92 299
**********Hit in genome EU118126****************
ABCDEF_39919 1053 EU118126 162046 100.00 1053 0 0 1 1053 119871 120923 0.0 2087
**********Hit in cds ndhF****************
ndhF
ABCDEF_39919 1053 ndhF 2133 100.00 1053 0 0 1 1053 1928 876 0.0 2087
codon_start=1 transl_table=11 product="NADH-plastoquinone oxidoreductase subunit 5" protein_id="ABV02398.1" complement(119665..121797)
//

>ABCDEF_38301
Normal: 19 125 51 62 26 279 ABCDEF_38301 1691 UniRef50_Q7G6A4 341 81.37 322 17 1 1691 726 61 339 1e-176 511
**********Hit in genome EU118126****************
ABCDEF_38301 1691 EU118126 162046 100.00 1292 0 0 400 1691 76854 75563 0.0 2561
ABCDEF_38301 1691 EU118126 162046 100.00 405 0 0 1 405 77297 76893 0.0 803
**********Hit in cds psbB****************
psbB
ABCDEF_38301 1691 psbB 1527 100.00 976 0 0 716 1691 1527 552 0.0 1935
codon_start=1 transl_table=11 product="photosystem II CP47 chlorophyll apoprotein" protein_id="ABV02374.1" 75011..76537
//

>ABCDEF_76537
Normal: 4 10 18 11 9 58 ABCDEF_76537 304 UniRef50_B9PB42 188 93.10 29 2 0 303 217 160 188 2e-08 54.7
**********Hit in genome EU118126****************
ABCDEF_76537 304 EU118126 162046 100.00 304 0 0 1 304 130864 131167 1e-174 603
**********Hit in cds ndhA****************
ndhA
ABCDEF_76537 304 ndhA 2532 100.00 304 0 0 1 304 787 484 7e-175 603

codon_start=1 transl_table=11 product="NADH-plastoquinone oxidoreductase subunit 1" protein_id="ABV02406.1" complement(129118..131649)
//

>ABCDEF_38300
Normal: 27 148 63 57 30 383 ABCDEF_38300 1730 UniRef50_Q7G6A4 341 81.50 319 16 1 1726 770 64 339 1e-174 507
**********Hit in genome EU118126****************
ABCDEF_38300 1730 EU118126 162046 100.00 1730 0 0 1 1730 77297 75568 0.0 3429
ABCDEF_38300 1730 EU118126 162046 95.24 42 2 0 395 436 76862 76903 1e-12 67.9
**********Hit in cds psbB****************
psbB
ABCDEF_38300 1730 psbB 1527 100.00 971 0 0 760 1730 1527 557 0.0 1925
codon_start=1 transl_table=11 product="photosystem II CP47 chlorophyll apoprotein" protein_id="ABV02374.1" 75011..76537
//

>ABCDEF_80369
Normal: 8 11 23 6 10 83 ABCDEF_80369 589 UniRef50_UPI000008E4AB 247 78.31 166 36 0 545 48 70 235 2e-88 268
**********Hit in genome EU118126****************
ABCDEF_80369 589 EU118126 162046 99.83 589 1 0 1 589 129110 129698 0.0 1160
**********Hit in cds ndhA****************
ndhA
ABCDEF_80369 589 ndhA 2532 99.83 580 1 0 10 589 2532 1953 0.0 1142
codon_start=1 transl_table=11 product="NADH-plastoquinone oxidoreductase subunit 1" protein_id="ABV02406.1" complement(129118..131649)
//

>ABCDEF_29523
Normal: 5 21 17 10 12 68 ABCDEF_29523 638 UniRef50_Q95BN8 161 78.88 161 34 0 606 124 1 161 5e-89 268
**********Hit in genome EU118126****************
ABCDEF_29523 638 EU118126 162046 99.69 638 2 0 1 638 16080 16717 0.0 1249
**********Hit in cds rps2****************
rps2
ABCDEF_29523 638 rps2 711 99.66 586 2 0 53 638 711 126 0.0 1146
codon_start=1 transl_table=11 product="ribosomal protein S2" protein_id="ABV02337.1" complement(16131..16841)
//

>ABCDEF_88386
Normal: 16 46 61 12 25 156 ABCDEF_88386 267 UniRef50_A4GYQ3 56 72.73 44 8 1 228 109 12 55 3e-10 57.8
**********Hit in genome EU118126****************
ABCDEF_88386 267 EU118126 162046 100.00 267 0 0 1 267 33178 33444 1e-152 529
*************no hit in cds*****************
//

>ABCDEF_3875
Normal: 220 415 350 114 284 1272 ABCDEF_3875 1527 UniRef50_Q9XQV2 776 69.09 220 64 2 1526 876 558 776 1e-95 315
**********Hit in genome EU118126****************
ABCDEF_3875 1527 EU118126 162046 99.74 1527 4 0 1 1527 38085 39611 0.0 2995
**********Hit in cds psaB****************
psaB
ABCDEF_3875 1527 psaB 2205 100.00 666 0 0 862 1527 2205 1540 0.0 1320
codon_start=1 transl_table=11 product="photosystem I P700 apoprotein A2" protein_id="ABV02347.1" complement(38945..41149)
//

>ABCDEF_42459
Normal: 34 113 55 27 48 318 ABCDEF_42459 1160 UniRef50_Q9LHB7 202 60.64 249 49 3 971 231 1 202 3e-75 240
**********Hit in genome EU118126****************
ABCDEF_42459 1160 EU118126 162046 99.91 1093 1 0 68 1160 14991 16083 0.0 2159
ABCDEF_42459 1160 EU118126 162046 100.00 68 0 0 1 68 14992 15059 3e-33 135
**********Hit in cds atpI****************
atpI
ABCDEF_42459 1160 atpI 744 99.87 744 1 0 229 972 744 1 0.0 1467
codon_start=1 transl_table=11 product="ATP synthase CFO subunit IV" protein_id="ABV02336.1" complement(15151..15894)
//

>ABCDEF_32284
Normal: 16 18 41 53 23 188 ABCDEF_32284 531 UniRef50_Q9MTL3 517 65.14 175 48 1 1 525 244 405 1e-71 233
**********Hit in genome EU118126****************
ABCDEF_32284 531 EU118126 162046 100.00 531 0 0 1 531 60044 60574 0.0 1053
**********Hit in cds accD****************
accD
ABCDEF_32284 531 accD 1530 100.00 531 0 0 1 531 678 1208 0.0 1053
codon_start=1 transl_table=11 product="acetyl-CoA carboxylase carboxyltransferase beta protein_id="ABV02357.1" 59366..60895

```
//

>ABCDEF_20093
Normal: 166	385	236	215	195	1095		ABCDEF_20093	1312	UniRef50_Q8YPI9		122	70.49	122	36	0	548	183	1	122	9e-55	186
**********Hit in genome EU118126****************
ABCDEF_20093	1312	EU118126	162046	100.00	1312	0	0	1	1312	83178	84489	0.0	2601
**********Hit in cds rpl16****************
rpl16
ABCDEF_20093	1312	rpl16	1460	100.00	661	0	0	652	1312	1460	800	0.0	1310
codon_start=1 transl_table=11 product="ribosomal protein L16" protein_id="ABV02385.1"	complement(83828..85287)
//

>ABCDEF_21812
Normal: 90	188	169	91	100	952		ABCDEF_21812	1015	UniRef50_Q8M9U5		670	56.33	300	131	0	116	1015	1	300	9e-97	309
**********Hit in genome EU118126****************
ABCDEF_21812	1015	EU118126	162046	100.00	1015	0	0	1	1015	121912	120898	0.0	2012
**********Hit in cds ndhF****************
ndhF
ABCDEF_21812	1015	ndhF	2133	100.00	901	0	0	115	1015	1	901	0.0	1786
codon_start=1 transl_table=11 product="NADH-plastoquinone oxidoreductase subunit 5" protein_id="ABV02398.1"	complement(119665..121797)
//

>ABCDEF_75877
Normal: 57	41	73	27	48	259		ABCDEF_75877	388	UniRef50_A7Y3K2		2057	83.72	129	3	1	388	2	163	273	2e-51	183
**********Hit in genome EU118126****************
ABCDEF_75877	388	EU118126	162046	100.00	343	0	0	46	388	111280	110938	0.0	680
ABCDEF_75877	388	EU118126	162046	100.00	343	0	0	46	388	138939	139281	0.0	680
ABCDEF_75877	388	EU118126	162046	100.00	49	0	0	1	49	111271	111223	2e-22	97.6
ABCDEF_75877	388	EU118126	162046	100.00	49	0	0	1	49	138948	138996	2e-22	97.6
**********Hit in cds ycf1****************
ycf1
ABCDEF_75877	388	ycf1	6174	100.00	343	0	0	46	388	830	488	0.0	680
ABCDEF_75877	388	ycf1	6174	100.00	49	0	0	1	49	821	773	1e-22	97.6
codon_start=1 transl_table=11 product="hypothetical chloroplast RF19" protein_id="ABV02395.1" codon_start=1 transl_table=11 product="hypothetical chloroplast RF19" protein_id="ABV02409.1"	compleme
//

>ABCDEF_45384
Normal: 336	108	370	261	367	940		ABCDEF_45384	956	UniRef50_Q68RU8		1919	54.69	320	127	7	15	947	1195	1505	7e-83	281
**********Hit in genome EU118126****************
ABCDEF_45384	956	EU118126	162046	100.00	956	0	0	1	956	114209	115164	0.0	1895
ABCDEF_45384	956	EU118126	162046	100.00	956	0	0	1	956	136010	135055	0.0	1895
ABCDEF_45384	956	EU118126	162046	92.00	75	6	0	873	947	115309	115383	4e-23	101
ABCDEF_45384	956	EU118126	162046	92.00	75	6	0	873	947	134910	134836	4e-23	101
ABCDEF_45384	956	EU118126	162046	89.29	84	9	0	873	956	115168	115251	2e-21	95.6
ABCDEF_45384	956	EU118126	162046	89.29	84	9	0	873	956	135051	134968	2e-21	95.6
**********Hit in cds ycf1****************
ycf1
ABCDEF_45384	956	ycf1	6174	100.00	956	0	0	1	956	3759	4714	0.0	1895
ABCDEF_45384	956	ycf1	6174	92.00	75	6	0	873	947	4859	4933	2e-23	101
ABCDEF_45384	956	ycf1	6174	89.29	84	9	0	873	956	4718	4801	1e-21	95.6
codon_start=1 transl_table=11 product="hypothetical chloroplast RF19" protein_id="ABV02395.1" codon_start=1 transl_table=11 product="hypothetical chloroplast RF19" protein_id="ABV02409.1"	compleme
//

>ABCDEF_28360
Normal: 29	51	79	41	30	271		ABCDEF_28360	827	UniRef50_Q7TTU5		140	45.16	62	34	0	191	376	15	76	1e-08	58.5
**********Hit in genome EU118126****************
ABCDEF_28360	827	EU118126	162046	100.00	827	0	0	1	827	5501	4675	0.0	1639
**********Hit in cds rps16****************
rps16
ABCDEF_28360	827	rps16	1071	100.00	408	0	0	1	408	664	1071	0.0	809
codon_start=1 transl_table=11 product="ribosomal protein S16" protein_id="ABV02330.1"	complement(5093..6163)
//

>ABCDEF_50169
Normal: 150	70	219	83	230	638		ABCDEF_50169	408	UniRef50_Q7YJS6		1828	50.00	112	49	2	11	346	1408	1512	1e-14	77.4
**********Hit in genome EU118126****************
ABCDEF_50169	408	EU118126	162046	100.00	408	0	0	1	408	115311	115718	0.0	809
ABCDEF_50169	408	EU118126	162046	100.00	408	0	0	1	408	134908	134501	0.0	809
ABCDEF_50169	408	EU118126	162046	91.78	73	6	0	1	73	115083	115155	3e-22	97.6
ABCDEF_50169	408	EU118126	162046	91.78	73	6	0	1	73	135136	135064	3e-22	97.6
```

ABCDEF_50169 408 EU118126 162046 89.04 73 8 0 1 73 115170 115242 2e-17 81.8
ABCDEF_50169 408 EU118126 162046 89.04 73 8 0 1 73 135049 134977 2e-17 81.8
ABCDEF_50169 408 EU118126 162046 100.00 24 0 0 385 408 115719 115742 2e-07 48.1
ABCDEF_50169 408 EU118126 162046 100.00 24 0 0 385 408 134500 134477 2e-07 48.1
**********Hit in cds ycf1****************
ycf1
ABCDEF_50169 408 ycf1 6174 100.00 408 0 0 1 408 4861 5268 0.0 809
ABCDEF_50169 408 ycf1 6174 91.78 73 6 0 1 73 4633 4705 1e-22 97.6
ABCDEF_50169 408 ycf1 6174 89.04 73 8 0 1 73 4720 4792 8e-18 81.8
ABCDEF_50169 408 ycf1 6174 100.00 24 0 0 385 408 5269 5292 1e-07 48.1
codon_start=1 transl_table=11 product="hypothetical chloroplast RF19" protein_id="ABV02395.1" codon_start=1 transl_table=11 product="hypothetical chloroplast RF19" protein_id="ABV02409.1" compleme
//

>ABCDEF_77121
Normal: 9 20 14 22 11 78 ABCDEF_77121 330 UniRef50_Q2WGF1 133 42.25 71 40 1 217 8 1 71 5e-09 55.8
**********Hit in genome EU118126****************
ABCDEF_77121 330 EU118126 162046 99.69 327 0 1 1 326 82865 83191 0.0 632
**********Hit in cds rps8****************
rps8
ABCDEF_77121 330 rps8 405 100.00 218 0 0 1 218 218 1 2e-123 432
codon_start=1 transl_table=11 product="ribosomal protein S8" protein_id="ABV02383.1" complement(82677..83081)
//

>ABCDEF_14758
Normal: 88 402 107 159 138 762 ABCDEF_14758 1531 UniRef50_Q3BAR3 114 73.68 114 30 0 104 445 1 114 9e-41 149
**********Hit in genome EU118126****************
ABCDEF_14758 1531 EU118126 162046 100.00 1531 0 0 1 1531 477 2007 0.0 3035
**********Hit in cds psbA****************
psbA
ABCDEF_14758 1531 psbA 1062 100.00 1062 0 0 54 1115 1062 1 0.0 2105
codon_start=1 transl_table=11 product="photosystem II protein D1" protein_id="ABV02328.1" complement(529..1590)
//

>ABCDEF_5023
Normal: 6 11 10 6 10 39 ABCDEF_5023 391 UniRef50_UPI000008E4AB 247 83.78 111 18 0 340 8 70 180 9e-61 195
**********Hit in genome EU118126****************
ABCDEF_5023 391 EU118126 162046 99.74 391 1 0 1 391 129315 129705 0.0 767
**********Hit in cds ndhA****************
ndhA
ABCDEF_5023 391 ndhA 2532 99.74 391 1 0 1 391 2336 1946 0.0 767
codon_start=1 transl_table=11 product="NADH-plastoquinone oxidoreductase subunit 1" protein_id="ABV02406.1" complement(129118..131649)
//

>ABCDEF_49556
Normal: 23 29 82 43 48 140 No annotation
**********Hit in genome EU118126****************
ABCDEF_49556 434 EU118126 162046 100.00 434 0 0 1 434 130786 130353 0.0 860
ABCDEF_49556 434 EU118126 162046 90.00 40 4 0 273 312 130475 130514 2e-07 48.1
**********Hit in cds ndhA****************
ndhA
ABCDEF_49556 434 ndhA 2532 100.00 434 0 0 1 434 865 1298 0.0 860
ABCDEF_49556 434 ndhA 2532 90.00 40 4 0 273 312 1176 1137 1e-07 48.1
codon_start=1 transl_table=11 product="NADH-plastoquinone oxidoreductase subunit 1" protein_id="ABV02406.1" complement(129118..131649)
//

>ABCDEF_11521
Normal: 61 64 111 84 56 282 ABCDEF_11521 1090 UniRef50_Q9MUV8 229 55.00 160 71 1 609 1088 25 183 2e-47 168
**********Hit in genome EU118126****************
ABCDEF_11521 1090 EU118126 162046 100.00 899 0 0 1 899 74233 73335 0.0 1782
ABCDEF_11521 1090 EU118126 162046 99.48 191 1 0 900 1090 72723 72533 3e-104 371
**********Hit in cds clpP****************
clpP
ABCDEF_11521 1090 clpP 2028 100.00 899 0 0 1 899 268 1166 0.0 1782
ABCDEF_11521 1090 clpP 2028 99.48 191 1 0 900 1090 1778 1968 2e-104 371
codon_start=1 transl_table=11 product="clp protease proteolytic subunit" protein_id="ABV02373.1" complement(72472..74499)
//

>ABCDEF_68774
Normal: 6 2 10 9 6 10 ABCDEF_68774 363 UniRef50_Q979M3 369 48.72 78 40 0 27 260 208 285 2e-20 90.5
**********Hit in genome EU118126****************

```
ABCDEF_68774   363     EU118126       162046  99.45   364     1       1       1       363     117993  118356  0.0     698
ABCDEF_68774   363     EU118126       162046  99.45   364     1       1       1       363     132226  131863  0.0     698
**********Hit in cds ndhH****************
ndhH
ABCDEF_68774   363     ndhH           1182    99.45   364     1       1       1       363     608     971     0.0     698
codon_start=1 transl_table=11 product="NADH-plastoquinone oxidoreductase subunit 7" protein_id="ABV02397.1" codon_start=1 transl_table=11 product="NADH-plastoquinone oxidoreductase subunit 7" prot
//

>ABCDEF_44500
Normal: 226     138     205     193     129     499          ABCDEF_44500        2484    UniRef50_Q979M3          369     43.83   381     194     1       575     1717    9       369     1e-100  325
**********Hit in genome EU118126****************
ABCDEF_44500   2484    EU118126       162046  99.72   2483    7       0       1       2483    133370  130888  0.0     4867
ABCDEF_44500   2484    EU118126       162046  99.95   2206    1       0       1       2206    116849  119054  0.0     4365
ABCDEF_44500   2484    EU118126       162046  100.00  31      0       0       2451    2481    130894  130924  9e-11   61.9
**********Hit in cds ndhH****************
ndhH
ABCDEF_44500   2484    ndhH           1182    99.92   1182    1       0       538     1719    1       1182    0.0     2335
codon_start=1 transl_table=11 product="NADH-plastoquinone oxidoreductase subunit 7" protein_id="ABV02397.1" codon_start=1 transl_table=11 product="NADH-plastoquinone oxidoreductase subunit 7" prot
//

>ABCDEF_44502
Normal: 125     74      101     121     85      319          ABCDEF_44502        1814    UniRef50_Q979M3          369     43.83   381     194     1       90      1232    9       369     4e-103  325
**********Hit in genome EU118126****************
ABCDEF_44502   1814    EU118126       162046  99.89   1814    2       0       1       1814    117334  119147  0.0     3580
ABCDEF_44502   1814    EU118126       162046  99.88   1721    2       0       1       1721    132885  131165  0.0     3396
**********Hit in cds ndhH****************
ndhH
ABCDEF_44502   1814    ndhH           1182    99.83   1182    2       0       53      1234    1       1182    0.0     2327
codon_start=1 transl_table=11 product="NADH-plastoquinone oxidoreductase subunit 7" protein_id="ABV02397.1" codon_start=1 transl_table=11 product="NADH-plastoquinone oxidoreductase subunit 7" prot
//

>ABCDEF_5112
Normal: 16      33      39      20      23      64           ABCDEF_5112         406     UniRef50_Q8M9V7          334     62.86   70      26      0       212     3       14      83      2e-26   107
**********Hit in genome EU118126****************
ABCDEF_5112    406     EU118126       162046  99.75   406     1       0       1       406     81241   81646   0.0     797
**********Hit in cds rpoA****************
rpoA
ABCDEF_5112    406     rpoA           1014    99.60   252     1       0       1       252     252     1       2e-141  492
codon_start=1 transl_table=11 product="RNA polymerase alpha subunit" protein_id="ABV02380.1"       complement(80478..81491)
//

>ABCDEF_70133
Normal: 18      16      24      12      16      39           ABCDEF_70133        213     UniRef50_Q9BBN6          1793    54.24   59      25      1       35      211     13      69      2e-09   59.3
**********Hit in genome EU118126****************
ABCDEF_70133   213     EU118126       162046  99.53   213     1       0       1       213     110442  110654  4e-118  414
ABCDEF_70133   213     EU118126       162046  99.53   213     1       0       1       213     139777  139565  4e-118  414
**********Hit in cds ycf1****************
ycf1
ABCDEF_70133   213     ycf1           6174    100.00  204     0       0       10      213     1       204     2e-115  404
codon_start=1 transl_table=11 product="hypothetical chloroplast RF19" protein_id="ABV02395.1" codon_start=1 transl_table=11 product="hypothetical chloroplast RF19" protein_id="ABV02409.1"    compleme
//

>ABCDEF_44501
Normal: 17      11      10      22      11      30           ABCDEF_44501        398     UniRef50_Q7NI12          393     82.11   123     22      0       28      396     8       130     4e-69   221
**********Hit in genome EU118126****************
ABCDEF_44501   398     EU118126       162046  100.00  361     0       0       38      398     117418  117778  0.0     716
ABCDEF_44501   398     EU118126       162046  100.00  361     0       0       38      398     132801  132441  0.0     716
**********Hit in cds ndhH****************
ndhH
ABCDEF_44501   398     ndhH           1182    100.00  361     0       0       38      398     33      393     0.0     716
codon_start=1 transl_table=11 product="NADH-plastoquinone oxidoreductase subunit 7" protein_id="ABV02397.1" codon_start=1 transl_table=11 product="NADH-plastoquinone oxidoreductase subunit 7" prot
//



***************Undetected cds******************
>trnT-UGU      complement(48509..48581)
>atpH   codon_start=1 transl_table=11 product="ATP synthase CFO subunit III" protein_id="ABV02335.1"       complement(13795..14040)
>trnR-ACG      complement(140802..140875)
>trnT-GGU      32984..33055
```

>rpl20 codon_start=1 transl_table=11 product="ribosomal protein L20" protein_id="ABV02371.1" complement(71031..71381)
>trnS-UGA complement(37056..37145)
>trnfM-CAU complement(38302..38375)
>rrn5 complement(141115..141235)
>psbF codon_start=1 transl_table=11 product="photosystem II cytochrome b559 beta subunit" protein_id="ABV02364.1" complement(66515..66634)
>psbE codon_start=1 transl_table=11 product="photosystem II cytochrome b559 alpha subunit" protein_id="ABV02365.1" complement(66647..66898)
>trnL-UAG 123556..123635
>trnV-UAC complement(54222..54259)
>rps3 codon_start=1 transl_table=11 product="ribosomal protein S3" protein_id="ABV02386.1" complement(85451..86107)
>trnL-CAA 154593..154673
>trnM-CAU 54434..54506
>trnA-UGC complement(145493..145530)
>psbT codon_start=1 transl_table=11 product="photosystem II protein T" protein_id="ABV02375.1" 76738..76839
>trnD-GUC complement(31845..31918)
>rpoB codon_start=1 transl_table=11 product="RNA polymerase beta subunit" protein_id="ABV02340.1" complement(24302..27505)
>rpl36 codon_start=1 transl_table=11 product="ribosomal protein L36" protein_id="ABV02382.1" complement(82076..82189)
>atpE codon_start=1 transl_table=11 product="ATP synthase CF1 epsilon subunit" protein_id="ABV02354.1" complement(54715..55116)
>psbN codon_start=1 transl_table=11 product="photosystem II protein N" protein_id="ABV02376.1" complement(76905..77036)
>rps11 codon_start=1 transl_table=11 product="ribosomal protein S11" protein_id="ABV02381.1" complement(81568..81984)
>trnP-UGG complement(68829..68902)
>rrn4.5 complement(141477..141579)
>trnI-CAU 161868..161941
>rps18 codon_start=1 transl_table=11 product="ribosomal protein S18" protein_id="ABV02370.1" 70429..70776
>ndhC codon_start=1 transl_table=11 product="NADH-plastoquinone oxidoreductase subunit 3" protein_id="ABV02353.1" complement(52302..52664)
>psbL exception="RNA editing" codon_start=1 transl_table=11 product="photosystem II protein L" protein_id="ABV02363.1" complement(66376..66492)
>trnE-UUC complement(32164..32236)
>rpl33 codon_start=1 transl_table=11 product="ribosomal protein L33" protein_id="ABV02369.1" 70038..70238
>petL codon_start=1 transl_table=11 product="cytochrome b6/f complex subunit VI" protein_id="ABV02366.1" 68021..68116
>rpl23 codon_start=1 transl_table=11 product="ribosomal protein L23" protein_id="ABV02390.1" complement(87796..88074)
>rps19 codon_start=1 transl_table=11 product="ribosomal protein S19" protein_id="ABV02388.1" complement(86637..86918)
>rpl14 codon_start=1 transl_table=11 product="ribosomal protein L14" protein_id="ABV02384.1" complement(83357..83725)
>psbJ codon_start=1 transl_table=11 product="photosystem II protein J" protein_id="ABV02362.1" complement(66119..66241)
>rpl22 codon_start=1 transl_table=11 product="ribosomal protein L22" protein_id="ABV02387.1" complement(86092..86571)
>trnW-CCA complement(68586..68659)
>psbH codon_start=1 transl_table=11 product="photosystem II phosphoprotein" protein_id="ABV02377.1" 77153..77374
>trnY-GUA complement(32025..32108)
>ndhJ codon_start=1 transl_table=11 product="NADH-plastoquinone oxidoreductase subunit J" protein_id="ABV02351.1" complement(50950..51447)
>psbD codon_start=1 transl_table=11 product="photosystem II protein D2" protein_id="ABV02343.1" 34378..35439
>trnI-GAU complement(146559..146595)
>trnR-UCU 10436..10507
>psbZ codon_start=1 transl_table=11 product="photosystem II protein Z" protein_id="ABV02345.1" 37497..37685
>trnH-GUG complement(62..136)
>rps14 codon_start=1 transl_table=11 product="ribosomal protein S14" protein_id="ABV02346.1" complement(38517..38819)
>trnN-GUU 140170..140241
>trnV-GAC complement(148605..148676)
>rps15 codon_start=1 transl_table=11 product="ribosomal protein S15" protein_id="ABV02396.1" codon_start=1 transl_table=11 product="ribosomal protein S15" protein_id="ABV02408.1" complement(13295
