# Supplementary material for: Dissecting organ-specific transcriptomes through RNA-sequencing
Source: Plant Methods. 2013 Oct 25;9:42. doi: 10.1186/1746-4811-9-42 (PMC3819660; doi:10.1186/1746-4811-9-42)
Supplement: Additional file 4 — Mitochondrial scaffolds and annotations. [file 1746-4811-9-42-S4.pdf]

Additional file 4. Mitochondrial expression and annotation for *Ipomoea purpurea*

********************format explanation****************
>SeqID
col2~7: normalized expression abundance corresponding to samples A-F; clo8: SeqID; col9: SeqLength; col10:uniref50 proteinID; col11:uniref50 protein SeqLength; col12~21: blast result;
**********Hit in genome ****************
col1: SeqID; col2: SeqLength; col3: genome ID; col4: genome SeqLength; col5~: blast result
**********Hit in annotated cds***************
cdsID
col1: SeqID; col2: SeqLength; col3: cds ID; col4: cds SeqLength; col5~: blast result
annotation for cds
//
****

>ABCDEF_84114
Normal: 0 0 0 0 0 10 ABCDEF_84114 243 UniRef50_Q9TGE6 213 81.48 81 15 0 1 243 123 203
4e-24 97.4
**********Hit in genome BA000042****************
ABCDEF_84114 243 BA000042 430597 97.53 243 6 0 1 243 129852 129610 1e-123 434
ABCDEF_84114 243 BA000042 430597 100.00 31 0 0 122 152 372595 372565 2e-11 61.9
**********Hit in cds cox1****************
cox1
ABCDEF_84114 243 cox1 1584 97.53 243 6 0 1 243 437 679 3e-124 434
exception="RNA editing" note="Protein sequence is in conflict with the conceptual codon_start=1 product="cytochrome oxidase subunit 1" protein_id="BAD83459.2" complement(128704..>130287)
//

>ABCDEF_25225
Normal: 41 0 34 0 10 12 No annotation
**********Hit in genome BA000042****************
ABCDEF_25225 470 BA000042 430597 96.65 209 3 1 262 470 375856 375652 5e-100 357
ABCDEF_25225 470 BA000042 430597 97.87 47 1 0 1 47 375958 375912 3e-18 85.7
*************no hit in cds*****************
//

>ABCDEF_84592
Normal: 0 0 0 0 0 10 ABCDEF_84592 280 UniRef50_Q35783 265 67.92 53 17 0 279 121 44 96
3e-14 71.2
**********Hit in genome BA000042****************
ABCDEF_84592 280 BA000042 430597 96.07 280 11 0 1 280 113973 114252 1e-133 468
**********Hit in cds orf25(atp4)****************
orf25(atp4)
ABCDEF_84592 280 orf25(atp4) 597 96.07 280 11 0 1 280 478 199 2e-134 468
exception="RNA editing" note="Protein sequence is in conflict with the conceptual codon_start=1 product="hypothetical protein" protein_id="BAD83452.2" complement(113853..114449)
//

>ABCDEF_73776
Normal: 0 0 0 0 12 0 ABCDEF_73776 253 UniRef50_B9TA70 89 89.29 56 6 0 239 72 34 89
3e-27 102
**********Hit in genome BA000042****************
ABCDEF_73776 253 BA000042 430597 96.84 253 8 0 1 253 377313 377061 1e-124 438
*************no hit in cds*****************
//

>ABCDEF_39778
Normal: 9 0 10 0 0 0 ABCDEF_39778 253 UniRef50_Q9MFB4 129 85.71 35 5 0 8 112 95 129
2e-12 65.1
**********Hit in genome BA000042****************
ABCDEF_39778 253 BA000042 430597 93.99 233 13 1 4 235 221818 221586 4e-96 343
**********Hit in cds orf147****************
orf147
ABCDEF_39778 253 orf147 444 93.99 233 13 1 4 235 167 399 8e-97 343
note="orf149 in Arabidopsis" codon_start=1 product="hypothetical protein" protein_id="BAD83499.1" complement(221540..221983)
//

>ABCDEF_53495
Normal: 0 0 14 0 23 12 No annotation
**********Hit in genome BA000042****************
ABCDEF_53495 411 BA000042 430597 92.48 359 14 2 52 410 199572 199917 3e-144 504
ABCDEF_53495 411 BA000042 430597 98.21 56 1 0 1 56 199487 199542 1e-23 103
**********Hit in cds trnN(guu)****************
trnN(guu)
ABCDEF_53495 411 trnN(guu) 72 96.30 27 1 0 1 27 46 72 4e-07 46.1
199441..199512
//

>ABCDEF_16238
Normal: 34 0 30 27 0 0 ABCDEF_16238 506 UniRef50_Q6YSR1 115 92.78 97 6 1 219 506 1 97
1e-56 181
**********Hit in genome BA000042****************
ABCDEF_16238 506 BA000042 430597 98.42 507 7 1 1 506 194566 195072 0.0 934
**********Hit in cds orf116****************
orf116
ABCDEF_16238 506 orf116 351 97.92 288 5 1 220 506 1 288 2e-148 515
note="identical to orf270/2 in Beta vulgaris" codon_start=1 product="hypothetical protein" protein_id="BAD83488.1" 194784..195134
//

>ABCDEF_39110
Normal: 27 0 50 25 20 16 No annotation
**********Hit in genome BA000042****************
ABCDEF_39110 393 BA000042 430597 94.66 393 11 4 1 392 202663 203046 4e-171 593
**********Hit in cds orf162****************
orf162
ABCDEF_39110 393 orf162 489 96.49 57 2 0 336 392 1 57 1e-22 97.6
codon_start=1 product="hypothetical protein" protein_id="BAD83491.1" 202989..203477
//

>ABCDEF_29634
Normal: 14 0 39 0 17 0 ABCDEF_29634 439 UniRef50_B9TAI0 55 86.67 45 6 0 150 284 1 45
1e-19 84.3
**********Hit in genome BA000042****************
ABCDEF_29634 439 BA000042 430597 93.36 286 9 2 1 285 272597 272873 6e-118 416
*************no hit in cds*****************
//

>ABCDEF_62901
Normal: 0 8 0 0 0 0 No annotation
**********Hit in genome BA000042****************
ABCDEF_62901 273 BA000042 430597 96.70 273 9 0 1 273 325985 326257 3e-134 470
**********Hit in cds trnS(gcu)****************
trnS(gcu)
ABCDEF_62901 273 trnS(gcu) 88 100.00 88 0 0 165 252 88 1 5e-46 174
complement(326148..326235)
//

>ABCDEF_69399
Normal: 0 0 8 0 0 0 ABCDEF_69399 250 UniRef50_B9TB32 115 94.29 35 2 0 105 1 21 55
2e-13 67.0
**********Hit in genome BA000042****************
ABCDEF_69399 250 BA000042 430597 97.60 250 6 0 1 250 173215 173464 1e-127 448
ABCDEF_69399 250 BA000042 430597 91.49 47 4 0 189 235 241700 241654 2e-11 61.9
ABCDEF_69399 250 BA000042 430597 93.94 33 2 0 189 221 200091 200123 9e-08 50.1
ABCDEF_69399 250 BA000042 430597 100.00 22 0 0 200 221 336995 336974 5e-06 44.1
*************no hit in cds*****************
//

>ABCDEF_72106
Normal: 0 0 0 22 0 10 ABCDEF_72106 374 UniRef50_Q9G8V8 483 69.35 124 38 0 372 1 205 328
2e-56 190
**********Hit in genome BA000042****************
ABCDEF_72106 374 BA000042 430597 97.99 348 7 0 27 374 184763 184416 0.0 634
ABCDEF_72106 374 BA000042 430597 100.00 25 0 0 1 25 187927 187903 1e-07 50.1
**********Hit in cds nad4****************
nad4
ABCDEF_72106 374 nad4 1488 97.86 374 8 0 1 374 1001 628 0.0 678
exception="RNA editing" note="Protein sequence is in conflict with the conceptual codon_start=1 product="NADH dehydrogenase subunit 4" protein_id="BAD83480.2" order(182370..182830,184249..1847
//

>ABCDEF_57011
Normal: 10 0 0 0 0 0 ABCDEF_57011 258 UniRef50_B9TA70 89 89.19 37 4 0 136 26 1 37
2e-14 69.7
**********Hit in genome BA000042****************
ABCDEF_57011 258 BA000042 430597 99.61 258 1 0 1 258 376988 376731 2e-144 504
*************no hit in cds*****************
//

>ABCDEF_46427
Normal: 14 0 12 30 0 0 ABCDEF_46427 407 UniRef50_Q5M9Z4 122 80.26 76 12 1 22 249 1 73
1e-30 114
**********Hit in genome BA000042****************
ABCDEF_46427 407 BA000042 430597 94.61 408 10 4 1 407 189537 189933 8e-179 618
**********Hit in cds orf122****************
orf122
ABCDEF_46427 407 orf122 369 97.63 337 6 2 63 398 31 366 1e-170 589
codon_start=1 product="hypothetical protein" protein_id="BAD83484.1" 189558..189926
//

>ABCDEF_8597
Normal: 34 23 52 0 0 54 ABCDEF_8597 392 UniRef50_UPI00019842D3 358 89.80 49 5 0 392 246 310 358
1e-17 83.2
**********Hit in genome BA000042****************
ABCDEF_8597 392 BA000042 430597 96.22 397 9 2 1 392 289083 288688 0.0 660
**********Hit in cds nad1****************
nad1
ABCDEF_8597 392 nad1 385 97.95 146 3 0 247 392 385 240 2e-73 266
exception="RNA editing" trans_splicing note="Protein sequence is in conflict with the conceptual codon_start=1 product="NADH dehydrogenase subunit 1" protein_id="BAD83532.2" 288448..288832
//

>ABCDEF_15495
Normal: 9 0 17 11 15 17 ABCDEF_15495 375 UniRef50_Q35302 72 60.00 70 21 2 315 109 9 72
6e-15 71.6
**********Hit in genome BA000042****************
ABCDEF_15495 375 BA000042 430597 94.17 326 8 3 58 375 360834 360512 2e-139 488
ABCDEF_15495 375 BA000042 430597 91.18 68 1 1 25 87 132784 132717 6e-19 87.7
ABCDEF_15495 375 BA000042 430597 90.14 71 2 1 22 87 152105 152175 2e-18 85.7
ABCDEF_15495 375 BA000042 430597 88.73 71 3 1 22 87 364059 363989 6e-16 77.8
**********Hit in cds orf159b****************
orf159b
ABCDEF_15495 375 orf159b 480 98.41 252 4 0 124 375 1 252 3e-134 468
note="orf168 in Marchantia" codon_start=1 product="hypothetical protein" protein_id="BAD83550.1" complement(360283..360762)
//

>ABCDEF_59859
Normal: 0 0 0 12 0 16 No annotation
**********Hit in genome BA000042****************
ABCDEF_59859 240 BA000042 430597 92.41 145 11 0 96 240 330463 330319 4e-53 200
*************no hit in cds*****************
//

>ABCDEF_67704
Normal: 0 0 25 15 0 0 No annotation
**********Hit in genome BA000042****************

ABCDEF_67704 294 BA000042 430597 95.32 299 4 2 1 294 232223 231930 8e-138 482
*************no hit in cds*****************
//

>ABCDEF_66069
Normal: 0 0 14 10 0 0 ABCDEF_66069 345 UniRef50_P51428 110 88.46 26 3 0 264 187 85 110
1e-06 49.3
**********Hit in genome BA000042****************
ABCDEF_66069 345 BA000042 430597 94.36 337 14 1 14 345 130306 130642 2e-148 517
**********Hit in cds rps10****************
rps10
ABCDEF_66069 345 rps10 363 97.35 113 3 0 155 267 363 251 1e-53 200
exception="RNA editing" note="Protein sequence is in conflict with the conceptual codon_start=1 product="ribosomal protein S10" protein_id="BAD83460.2"
complement(order(130451..130563,131338..1
//

>ABCDEF_49785
Normal: 16 0 52 0 17 14 ABCDEF_49785 495 UniRef50_Q6R9K4 105 76.47 34 6 1 296 391 45 78
6e-08 53.9
**********Hit in genome BA000042****************
ABCDEF_49785 495 BA000042 430597 91.67 504 28 4 1 495 202273 202771 0.0 654
*************no hit in cds*****************
//

>ABCDEF_59287
Normal: 28 13 25 0 0 0 No annotation
**********Hit in genome BA000042****************
ABCDEF_59287 283 BA000042 430597 89.29 280 8 3 1 280 158568 158825 2e-92 331
ABCDEF_59287 283 BA000042 430597 91.89 37 3 0 7 43 173299 173335 1e-07 50.1
ABCDEF_59287 283 BA000042 430597 96.30 27 1 0 17 43 231772 231746 2e-06 46.1
ABCDEF_59287 283 BA000042 430597 100.00 22 0 0 22 43 355265 355244 6e-06 44.1
ABCDEF_59287 283 BA000042 430597 100.00 22 0 0 204 225 361607 361586 6e-06 44.1
*************no hit in cds*****************
//

>ABCDEF_60339
Normal: 0 8 8 15 0 0 ABCDEF_60339 254 UniRef50_P42792 393 95.16 62 3 0 2 187 332 393
5e-26 104
**********Hit in genome BA000042****************
ABCDEF_60339 254 BA000042 430597 96.46 254 9 0 1 254 41055 40802 6e-123 432
**********Hit in cds cob****************
cob
ABCDEF_60339 254 cob 1182 96.30 189 7 0 1 189 994 1182 1e-89 319
exception="RNA editing" note="Protein sequence is in conflict with the conceptual codon_start=1 product="apocytochrome b" protein_id="BAD83429.2"complement(40866..42047)
//

>ABCDEF_5259
Normal: 39 0 48 22 23 18 ABCDEF_5259 504 UniRef50_Q9MFB4 129 89.83 59 4 1 181 357 31 87
9e-37 107
**********Hit in genome BA000042****************
ABCDEF_5259 504 BA000042 430597 97.99 498 10 0 1 498 222193 221696 0.0 908
ABCDEF_5259 504 BA000042 430597 100.00 23 0 0 267 289 379857 379835 3e-06 46.1
**********Hit in cds orf166a****************
orf166a
ABCDEF_5259 504 orf166a 501 97.77 404 9 0 95 498 1 404 0.0 729
codon_start=1 product="hypothetical protein" protein_id="BAD83500.1" complement(221598..222098)
//

>ABCDEF_68984
Normal: 3 0 17 0 0 0 ABCDEF_68984 399 UniRef50_Q9MGB5 273 61.65 133 49 2 3 398 117 248
4e-59 191
**********Hit in genome BA000042****************
ABCDEF_68984 399 BA000042 430597 95.51 312 14 0 88 399 158094 158405 2e-145 507
ABCDEF_68984 399 BA000042 430597 98.80 83 1 0 1 83 156647 156729 8e-40 157
**********Hit in cds cox2****************
cox2
ABCDEF_68984 399 cox2 783 95.74 399 17 0 1 399 295 693 0.0 656
exception="RNA editing" note="Protein sequence is in conflict with the conceptual codon_start=1 product="cytochrome oxidase subunit 2" protein_id="BAD83476.2"
order(156352..156733,158094..1584
//

>ABCDEF_65644
Normal: 0 0 12 0 0 10 No annotation
**********Hit in genome BA000042****************
ABCDEF_65644 311 BA000042 430597 84.02 169 27 0 67 235 137079 136911 4e-29 121
*************no hit in cds*****************
//

>ABCDEF_66415
Normal: 0 0 8 12 0 0 No annotation
**********Hit in genome BA000042****************
ABCDEF_66415 235 BA000042 430597 91.59 226 14 2 1 225 43037 42816 2e-79 287
ABCDEF_66415 235 BA000042 430597 100.00 34 0 0 111 144 309772 309805 3e-13 67.9
ABCDEF_66415 235 BA000042 430597 94.74 38 2 0 113 150 236564 236527 9e-11 60.0
ABCDEF_66415 235 BA000042 430597 96.30 27 1 0 115 141 393467 393493 1e-06 46.1
*************no hit in cds*****************
//

>ABCDEF_66132
Normal: 0 0 10 12 0 2 ABCDEF_66132 281 UniRef50_Q6RUR5 218 52.22 90 37 2 1 258 59 146
5e-14 70.5
**********Hit in genome BA000042****************
ABCDEF_66132 281 BA000042 430597 86.05 258 36 0 1 258 308181 308438 8e-61 226
*************no hit in cds*****************
//

>ABCDEF_82711

```
Normal: 0	0	0	0	0	3		ABCDEF_82711	202	UniRef50_UPI000198487F	1272	91.04	67	6	0	2	202	687	753
3e-32	125
**********Hit in genome BA000042****************
ABCDEF_82711	202	BA000042	430597	91.04	201	18	0	1	201	220481	220681	6e-70	256
**********Hit in cds orf114a****************
orf114a
ABCDEF_82711	202	orf114a	345	91.04	201	18	0	1	201	11	211	1e-70	256
note="a part of phosphoribosylformylglycinamide synthase" codon_start=1 product="hypothetical protein" protein_id="BAD83497.1"	220470..220814
//

>ABCDEF_65497
Normal: 0	0	8	0	0	0	No annotation
**********Hit in genome BA000042****************
ABCDEF_65497	267	BA000042	430597	96.36	275	2	1	1	267	275991	275717	7e-135	472
*************no hit in cds*****************
//

>ABCDEF_2044
Normal: 0	9	26	35	0	38		ABCDEF_2044	260	UniRef50_P60497	205	86.11	36	5	0	109	2	164	199
6e-13	67.0
**********Hit in genome BA000042****************
ABCDEF_2044	260	BA000042	430597	95.96	99	4	0	1	99	312866	312964	2e-42	165
ABCDEF_2044	260	BA000042	430597	95.79	95	4	0	166	260	113877	113971	5e-40	157
**********Hit in cds nad6****************
nad6
ABCDEF_2044	260	nad6	687	95.96	99	4	0	1	99	599	501	4e-43	165
exception="RNA editing" note="Protein sequence is in conflict with the conceptual codon_start=1 product="NADH dehydrogenase subunit 6" protein_id="BAD83537.2"	complement(312777..313463)
//

>ABCDEF_87491
Normal: 0	0	28	0	0	20	No annotation
**********Hit in genome BA000042****************
ABCDEF_87491	255	BA000042	430597	91.86	258	7	2	1	252	196257	196506	4e-99	353
*************no hit in cds*****************
//

>ABCDEF_18931
Normal: 12	0	25	0	23	16		ABCDEF_18931	379	UniRef50_Q0ZCF0	1450	96.00	25	1	0	77	3	30	54
2e-06	52.0
**********Hit in genome BA000042****************
ABCDEF_18931	379	BA000042	430597	96.14	363	14	0	17	379	202042	202404	7e-176	609
*************no hit in cds*****************
//

>ABCDEF_69543
Normal: 0	0	10	0	0	0		ABCDEF_69543	293	UniRef50_A5AMW0	564	83.87	31	5	0	292	200	271	301
7e-09	58.2
**********Hit in genome BA000042****************
ABCDEF_69543	293	BA000042	430597	93.62	298	11	2	4	293	188336	188633	4e-124	436
ABCDEF_69543	293	BA000042	430597	95.65	46	2	0	186	231	354847	354892	2e-15	75.8
ABCDEF_69543	293	BA000042	430597	96.43	28	1	0	194	221	142751	142778	4e-07	48.1
ABCDEF_69543	293	BA000042	430597	100.00	23	0	0	158	180	354684	354706	2e-06	46.1
*************no hit in cds*****************
//

>ABCDEF_35992
Normal: 30	0	37	38	46	10		ABCDEF_35992	501	UniRef50_Q6JP41	285	60.00	55	21	1	321	485	208	261
2e-08	57.4
**********Hit in genome BA000042****************
ABCDEF_35992	501	BA000042	430597	92.70	233	12	1	274	501	44964	44732	5e-91	327
ABCDEF_35992	501	BA000042	430597	95.12	41	2	0	313	353	184126	184166	3e-12	65.9
ABCDEF_35992	501	BA000042	430597	100.00	29	0	0	240	268	144548	144520	7e-10	58.0
*************no hit in cds*****************
//

>ABCDEF_72981
Normal: 7	0	18	16	10	13		ABCDEF_72981	386	UniRef50_Q35302	72	60.00	70	21	3	313	107	9	72
2e-14	70.5
**********Hit in genome BA000042****************
ABCDEF_72981	386	BA000042	430597	92.92	339	13	3	56	386	360834	360499	3e-135	474
ABCDEF_72981	386	BA000042	430597	91.18	68	1	1	23	85	132784	132717	6e-19	87.7
ABCDEF_72981	386	BA000042	430597	90.14	71	2	1	20	85	152105	152175	2e-18	85.7
ABCDEF_72981	386	BA000042	430597	88.73	71	3	1	20	85	364059	363989	6e-16	77.8
**********Hit in cds orf159b****************
orf159b
ABCDEF_72981	386	orf159b	480	96.60	265	9	0	122	386	1	265	5e-130	454
note="orf168 in Marchantia" codon_start=1 product="hypothetical protein" protein_id="BAD83550.1"	complement(360283..360762)
//

>ABCDEF_68259
Normal: 9	0	12	0	0	0		ABCDEF_68259	326	UniRef50_Q9ZZQ0	666	72.22	108	30	0	324	1	220	327
3e-48	169
**********Hit in genome BA000042****************
ABCDEF_68259	326	BA000042	430597	97.85	326	7	0	1	326	193973	193648	1e-170	591
*************no hit in cds*****************
//

>ABCDEF_42529
Normal: 25	6	17	0	15	12	No annotation
**********Hit in genome BA000042****************
ABCDEF_42529	325	BA000042	430597	99.28	139	1	0	72	210	230616	230754	3e-73	268
ABCDEF_42529	325	BA000042	430597	98.36	61	1	0	1	61	230516	230576	9e-27	113
*************no hit in cds*****************
//

>ABCDEF_72526
Normal: 0	0	8	20	0	0		ABCDEF_72526	270	UniRef50_Q9MGK2	650	44.58	83	45	1	261	13	418	499
```

2e-06 50.8
**********Hit in genome BA000042****************
ABCDEF_72526 270 BA000042 430597 98.26 172 3 0 99 270 194439 194268 3e-88 317
ABCDEF_72526 270 BA000042 430597 98.70 77 1 0 1 77 172799 172723 2e-36 145
ABCDEF_72526 270 BA000042 430597 100.00 22 0 0 78 99 222166 222187 6e-06 44.1
**********Hit in cds nad5****************
nad5
ABCDEF_72526 270 nad5 22 100.00 21 0 0 79 99 22 2 4e-06 42.1
exception="RNA editing" trans_splicing note="Protein sequence is in conflict with the conceptual codon_start=1 product="NADH dehydrogenase subunit 5" protein_id="BAD83486.2" complement(222166..
//

>ABCDEF_1271
Normal: 9215 20340 13106 25308 17375 14098 ABCDEF_1271 259 UniRef50_P93276 107 96.55 58 2 0 187 14 1 58
4e-35 123
**********Hit in genome BA000042****************
ABCDEF_1271 259 BA000042 430597 100.00 242 0 0 15 256 66867 67108 3e-137 480
ABCDEF_1271 259 BA000042 430597 100.00 242 0 0 15 256 417617 417858 3e-137 480
**********Hit in cds rrn26****************
rrn26
ABCDEF_1271 259 rrn26 3406 100.00 242 0 0 15 256 14 255 5e-138 480
417603..421008
//

>ABCDEF_41509
Normal: 3357 4884 5229 11712 4049 6307 ABCDEF_41509 2658 UniRef50_Q6R9A5 111 80.95 84 13 1 1449 1691 28 111
4e-36 140
**********Hit in genome BA000042****************
ABCDEF_41509 2658 BA000042 430597 99.52 1263 5 1 2 1264 108334 109595 0.0 2448
ABCDEF_41509 2658 BA000042 430597 97.35 680 9 3 1334 2005 109622 110300 0.0 1193
ABCDEF_41509 2658 BA000042 430597 95.00 220 3 1 2063 2282 110306 110517 1e-98 355
ABCDEF_41509 2658 BA000042 430597 97.96 98 1 1 1877 1974 216697 216793 4e-43 170
ABCDEF_41509 2658 BA000042 430597 94.85 97 5 0 2458 2554 271094 271190 9e-38 153
ABCDEF_41509 2658 BA000042 430597 90.48 84 2 1 2003 2080 325769 325686 2e-23 105
ABCDEF_41509 2658 BA000042 430597 92.68 41 3 0 1265 1305 97082 97122 4e-09 58.0
ABCDEF_41509 2658 BA000042 430597 100.00 29 0 0 686 714 216668 216696 4e-09 58.0
ABCDEF_41509 2658 BA000042 430597 100.00 28 0 0 49 76 66139 66166 2e-08 56.0
ABCDEF_41509 2658 BA000042 430597 100.00 28 0 0 49 76 416889 416916 2e-08 56.0
ABCDEF_41509 2658 BA000042 430597 100.00 26 0 0 50 75 107129 107104 2e-07 52.0
ABCDEF_41509 2658 BA000042 430597 90.70 43 1 1 23 65 23542 23503 1e-06 50.1
**********Hit in cds rrn18****************
rrn18
ABCDEF_41509 2658 rrn18 1902 99.52 1262 5 1 3 1264 1 1261 0.0 2446
ABCDEF_41509 2658 rrn18 1902 98.05 615 8 1 1334 1944 1288 1902 0.0 1122
108334..110235
//

>ABCDEF_17426
Normal: 2757 2473 2979 4971 2797 2683 ABCDEF_17426 3142 UniRef50_Q9PLI5 122 71.25 80 23 0 239 478 43 122
7e-25 108
**********Hit in genome BA000042****************
ABCDEF_17426 3142 BA000042 430597 98.42 3173 15 8 1 3142 70253 67085 0.0 5884
ABCDEF_17426 3142 BA000042 430597 98.42 3173 15 8 1 3142 421003 417835 0.0 5884
ABCDEF_17426 3142 BA000042 430597 94.23 52 3 0 7 58 112672 112723 1e-15 79.8
ABCDEF_17426 3142 BA000042 430597 100.00 25 0 0 2864 2888 196944 196968 1e-06 50.1
ABCDEF_17426 3142 BA000042 430597 100.00 24 0 0 333 356 116523 116500 5e-06 48.1
ABCDEF_17426 3142 BA000042 430597 96.88 32 0 1 2940 2971 326788 326818 5e-06 48.1
**********Hit in cds rrn26****************
rrn26
ABCDEF_17426 3142 rrn26 3406 98.42 3173 15 8 1 3142 3400 232 0.0 5884
417603..421008
//

>ABCDEF_47863
Normal: 1304 402 2134 934 963 1227 ABCDEF_47863 2518 UniRef50_Q9G868 85 44.59 74 37 1 662 883 3 72
7e-11 66.6
**********Hit in genome BA000042****************
ABCDEF_47863 2518 BA000042 430597 94.47 1518 53 8 629 2133 152191 153690 0.0 2325
ABCDEF_47863 2518 BA000042 430597 95.17 352 17 0 2167 2518 153694 154045 3e-161 563
ABCDEF_47863 2518 BA000042 430597 98.39 62 1 0 1532 1593 192784 192845 2e-26 115
ABCDEF_47863 2518 BA000042 430597 88.00 75 5 1 1890 1964 166838 166768 2e-14 75.8
ABCDEF_47863 2518 BA000042 430597 92.50 40 3 0 1558 1597 379061 379022 1e-08 56.0
ABCDEF_47863 2518 BA000042 430597 94.29 35 2 0 388 422 316990 316956 6e-08 54.0
ABCDEF_47863 2518 BA000042 430597 84.06 69 11 0 105 173 317128 317060 9e-07 50.1
ABCDEF_47863 2518 BA000042 430597 88.64 44 5 0 2405 2448 193082 193125 4e-06 48.1
**********Hit in cds rps19****************
rps19
ABCDEF_47863 2518 rps19 285 96.45 282 10 0 654 935 1 282 6e-137 480
exception="RNA editing" note="Protein sequence is in conflict with the conceptual codon_start=1 product="ribosomal protein S19" protein_id="BAD83473.2" 152215..152499
//

>ABCDEF_11805
Normal: 66 4 53 7 30 70 ABCDEF_11805 635 UniRef50_Q8HCM5 173 64.34 129 38 3 603 223 50 172
9e-19 86.7
**********Hit in genome BA000042****************
ABCDEF_11805 635 BA000042 430597 95.94 641 13 3 2 635 204233 204867 0.0 1061
**********Hit in cds orf214****************
orf214
ABCDEF_11805 635 orf214 645 98.59 71 1 0 2 72 575 645 4e-33 133
codon_start=1 product="hypothetical protein" protein_id="BAD83492.1" 203658..204302
//

>ABCDEF_47862
Normal: 611 412 1318 856 699 1021 ABCDEF_47862 2136 UniRef50_Q95749 556 84.85 363 49 3 1048 2136 1 357
4e-166 498
**********Hit in genome BA000042****************
ABCDEF_47862 2136 BA000042 430597 95.86 1015 36 1 1122 2136 154062 155070 0.0 1681

```
ABCDEF_47862   2136    BA000042       430597  96.21   396     15      0       726     1121    152191  152586  0.0     666
ABCDEF_47862   2136    BA000042       430597  94.29   35      2       0       485     519     316990  316956  5e-08   54.0
ABCDEF_47862   2136    BA000042       430597  84.06   69      11      0       202     270     317128  317060  8e-07   50.1
**********Hit in cds rps3****************
rps3
ABCDEF_47862   2136    rps3           1692    95.77   1088    40      1       1049    2136    1       1082    0.0     1794
exception="RNA editing" note="Protein sequence is in conflict with the conceptual codon_start=1 product="ribosomal protein S3" protein_id="BAD83474.2"   order(152513..152586,154062..155679)
//

>ABCDEF_24875
Normal: 213     24      311     187     143     96          ABCDEF_24875         1445     UniRef50_UPI0001985C72    238      92.86    28       2        0         474      391      1         28
3e-11   58.5
**********Hit in genome BA000042****************
ABCDEF_24875   1445    BA000042       430597  96.05   1419    41      5       1       1418    378153  379557  0.0     2349
ABCDEF_24875   1445    BA000042       430597  92.50   40      3       0       883     922     153159  153120  9e-09   56.0
ABCDEF_24875   1445    BA000042       430597  100.00  27      0       0       1419    1445    380473  380499  3e-08   54.0
ABCDEF_24875   1445    BA000042       430597  100.00  25      0       0       1282    1306    176729  176753  5e-07   50.1
**********Hit in cds orf125f****************
orf125f
ABCDEF_24875   1445    orf125f        378     98.94   378     4       0       814     1191    1       378     0.0     718
codon_start=1 product="hypothetical protein" protein_id="BAD83555.1" 378952..379329
//

>ABCDEF_45647
Normal: 52      9       104     22      20      38          ABCDEF_45647         1031     UniRef50_B9IKH7           83       70.59    34       10       0         639      538      44        77
7e-08   55.8
**********Hit in genome BA000042****************
ABCDEF_45647   1031    BA000042       430597  94.54   843     31      3       1       838     376811  375979  0.0     1306
ABCDEF_45647   1031    BA000042       430597  98.15   54      1       0       899     952     375965  375912  5e-22   99.6
ABCDEF_45647   1031    BA000042       430597  94.44   36      2       0       92      127     315413  315378  6e-09   56.0
*************no hit in cds*****************
//

>ABCDEF_15309
Normal: 52      16      61      149     46      16          ABCDEF_15309         618      UniRef50_Q85FT9           122      61.16    121      47       0         253      615      1         121
1e-47   160
**********Hit in genome BA000042****************
ABCDEF_15309   618     BA000042       430597  96.44   618     22      0       1       618     86548   87165   0.0     1051
ABCDEF_15309   618     BA000042       430597  93.13   233     16      0       1       233     288048  288280  3e-93   335
**********Hit in cds rps12****************
rps12
ABCDEF_15309   618     rps12          378     98.36   365     6       0       254     618     1       365     0.0     676
exception="RNA editing" note="Protein sequence is in conflict with the conceptual codon_start=1 product="ribosomal protein S12" protein_id="BAD83444.2"  86800..87177
//

>ABCDEF_14872
Normal: 77      13      118     63      64      56          ABCDEF_14872         838      UniRef50_Q5M9S2           109      95.88    97       4        0         440      150      1         97
2e-56   185
**********Hit in genome BA000042****************
ABCDEF_14872   838     BA000042       430597  95.38   845     22      4       1       838     380382  379548  0.0     1360
ABCDEF_14872   838     BA000042       430597  100.00  27      0       0       527     553     221927  221901  2e-08   54.0
ABCDEF_14872   838     BA000042       430597  93.55   31      2       0       653     683     301095  301125  5e-06   46.1
**********Hit in cds orf109c****************
orf109c
ABCDEF_14872   838     orf109c        330     97.89   331     6       1       109     439     330     1       2e-171  593
codon_start=1 product="hypothetical protein" protein_id="BAD83556.1" 379944..380273
//

>ABCDEF_38799
Normal: 61      9       77      38      38      26          ABCDEF_38799         941      UniRef50_B9SBA7           136      60.38    53       18       1         404      553      84        136
1e-07   55.8
**********Hit in genome BA000042****************
ABCDEF_38799   941     BA000042       430597  96.09   947     30      2       2       941     142564  141618  0.0     1578
ABCDEF_38799   941     BA000042       430597  97.83   46      1       0       819     864     331626  331581  2e-17   83.8
ABCDEF_38799   941     BA000042       430597  88.46   78      6       1       570     644     361956  362033  4e-16   79.8
*************no hit in cds*****************
//

>ABCDEF_32283
Normal: 197     28      210     93      166     106   No annotation
**********Hit in genome BA000042****************
ABCDEF_32283   553     BA000042       430597  97.83   553     9       1       1       553     173655  174204  0.0     997
*************no hit in cds*****************
//

>ABCDEF_35059
Normal: 571     79      451     271     297     250         ABCDEF_35059         866      UniRef50_Q2V397           122      63.41    41       15       0         284      162      1         41
9e-07   52.8
**********Hit in genome BA000042****************
ABCDEF_35059   866     BA000042       430597  99.19   862     7       0       1       862     192372  193233  0.0     1653
ABCDEF_35059   866     BA000042       430597  97.33   75      1       1       108     182     121418  121491  7e-30   125
ABCDEF_35059   866     BA000042       430597  96.77   62      2       0       413     474     153094  153155  2e-24   107
**********Hit in cds orf103a****************
orf103a
ABCDEF_35059   866     orf103a        312     98.95   285     3       0       1       285     285     1       6e-156  541
codon_start=1 product="hypothetical protein" protein_id="BAD83487.1" complement(192344..192655)
//

>ABCDEF_22396
Normal: 41      28      78      11      15      65          ABCDEF_22396         572      UniRef50_P93313           495      100.00   29       0        0         454      540      467       495
6e-11   66.6
**********Hit in genome BA000042****************
ABCDEF_22396   572     BA000042       430597  94.60   352     11      3       221     572     190538  190881  5e-153  533
ABCDEF_22396   572     BA000042       430597  96.43   28      1       0       1       28      190523  190550  8e-07   48.1
**********Hit in cds nad4****************
nad4
ABCDEF_22396   572     nad4           1488    94.38   89      5       0       456     544     1400    1488    2e-34   137
```

exception="RNA editing" note="Protein sequence is in conflict with the conceptual codon_start=1 product="NADH dehydrogenase subunit 4" protein_id="BAD83480.2"
order(182370..182830,184249..1847
//

>ABCDEF_25485
Normal: 95 14 61 53 82 34 ABCDEF_25485 616 UniRef50_A5BL70 201 98.15 54 1 0 28 189 1 54
3e-43 107
**********Hit in genome BA000042****************
ABCDEF_25485 616 BA000042 430597 98.38 616 9 1 1 616 156761 157375 0.0 1134
*************no hit in cds*****************
//

>ABCDEF_25057
Normal: 45 16 91 45 87 108 ABCDEF_25057 800 UniRef50_Q9TCB4 99 51.28 78 38 0 799 566 22 99
6e-18 83.2
**********Hit in genome BA000042****************
ABCDEF_25057 800 BA000042 430597 95.06 445 16 2 357 800 43197 43636 0.0 700
ABCDEF_25057 800 BA000042 430597 93.43 137 1 1 2 130 329904 329768 2e-54 206
ABCDEF_25057 800 BA000042 430597 100.00 46 0 0 85 130 361500 361545 9e-20 91.7
**********Hit in cds rps14****************
rps14
ABCDEF_25057 800 rps14 360 94.65 299 10 2 503 800 360 67 6e-131 458
exception="RNA editing" note="Protein sequence is in conflict with the conceptual codon_start=1 product="ribosomal protein S14" protein_id="BAD83430.2" complement(43342..43701)
//

>ABCDEF_49067
Normal: 148 23 136 25 74 130 ABCDEF_49067 862 UniRef50_Q6R9K0 99 89.29 28 3 0 750 833 72 99
3e-08 56.6
**********Hit in genome BA000042****************
ABCDEF_49067 862 BA000042 430597 94.53 859 33 5 13 862 204820 205673 0.0 1308
ABCDEF_49067 862 BA000042 430597 100.00 26 0 0 420 445 334200 334175 8e-08 52.0
*************no hit in cds*****************
//

>ABCDEF_17645
Normal: 236 173 343 593 364 460 ABCDEF_17645 1256 UniRef50_Q95749 556 71.68 226 51 4 1218 544 343 556
3e-89 289
**********Hit in genome BA000042****************
ABCDEF_17645 1256 BA000042 430597 95.38 1125 37 2 132 1256 156088 154979 0.0 1828
**********Hit in cds rps3****************
rps3
ABCDEF_17645 1256 rps3 1692 93.58 717 31 2 540 1256 1692 991 0.0 1066
exception="RNA editing" note="Protein sequence is in conflict with the conceptual codon_start=1 product="ribosomal protein S3" protein_id="BAD83474.2" order(152513..152586,154062..155679)
//

>ABCDEF_38143
Normal: 188 240 462 512 364 584 ABCDEF_38143 1786 UniRef50_Q2S432 551 56.01 491 196 4 1632 220 7 497 0.0
560
**********Hit in genome BA000042****************
ABCDEF_38143 1786 BA000042 430597 97.23 1518 42 0 133 1650 262801 261284 0.0 2676
ABCDEF_38143 1786 BA000042 430597 88.89 90 6 2 1 90 263209 263124 3e-18 87.7
ABCDEF_38143 1786 BA000042 430597 90.24 41 4 0 1610 1650 7757 7797 7e-07 50.1
ABCDEF_38143 1786 BA000042 430597 91.67 36 3 0 1702 1737 257582 257547 3e-06 48.1
**********Hit in cds atp1****************
atp1
ABCDEF_38143 1786 atp1 1530 97.41 1505 39 0 133 1637 1514 10 0.0 2674
exception="RNA editing" note="Protein sequence is in conflict with the conceptual codon_start=1 product="ATP synthase F1 subunit 1" protein_id="BAD83524.2" 261287..262816
//

>ABCDEF_42174
Normal: 126 24 136 81 110 74 ABCDEF_42174 594 UniRef50_Q5M9Y8 105 92.42 66 4 1 396 593 24 88
3e-31 117
**********Hit in genome BA000042****************
ABCDEF_42174 594 BA000042 430597 94.43 610 15 2 1 594 203636 203030 0.0 957
**********Hit in cds orf162****************
orf162
ABCDEF_42174 594 orf162 489 93.36 452 11 2 159 594 489 41 0.0 676
codon_start=1 product="hypothetical protein" protein_id="BAD83491.1" 202989..203477
//

>ABCDEF_45787
Normal: 173 47 264 109 141 144 ABCDEF_45787 951 UniRef50_B9T9Z0 75 96.00 25 1 0 1 75 48 72
2e-07 54.3
**********Hit in genome BA000042****************
ABCDEF_45787 951 BA000042 430597 98.56 348 5 0 1 348 130516 130863 0.0 650
ABCDEF_45787 951 BA000042 430597 96.15 390 8 2 413 799 130857 131242 0.0 648
ABCDEF_45787 951 BA000042 430597 100.00 87 0 0 865 951 131252 131338 3e-44 172
ABCDEF_45787 951 BA000042 430597 87.91 91 6 1 340 425 315296 315386 3e-20 93.7
**********Hit in cds rps10****************
rps10
ABCDEF_45787 951 rps10 363 100.00 49 0 0 1 49 299 251 3e-22 97.6
exception="RNA editing" note="Protein sequence is in conflict with the conceptual codon_start=1 product="ribosomal protein S10" protein_id="BAD83460.2"
complement(order(130451..130563,131338..1
//

>ABCDEF_35583
Normal: 61 16 89 38 69 76 No annotation
**********Hit in genome BA000042****************
ABCDEF_35583 730 BA000042 430597 93.99 749 23 4 2 730 158110 157364 0.0 1132
*************no hit in cds*****************
//

>ABCDEF_11263
Normal: 74 21 115 76 53 52 ABCDEF_11263 847 UniRef50_B9TC59 86 70.59 51 13 1 511 657 36 86
4e-12 66.6
**********Hit in genome BA000042****************
ABCDEF_11263 847 BA000042 430597 97.42 853 15 3 1 847 182918 183769 0.0 1501

*************no hit in cds*****************
//

>ABCDEF_49437
Normal: 28 16 30 65 12 26 ABCDEF_49437 469 UniRef50_Q8W9N0 261 62.00 150 57 0 19 468 53 202
1e-51 173
**********Hit in genome BA000042****************
ABCDEF_49437 469 BA000042 430597 97.23 469 13 0 1 469 258991 259459 0.0 827
**********Hit in cds cox3****************
cox3
ABCDEF_49437 469 cox3 798 97.23 469 13 0 1 469 147 615 0.0 827
exception="RNA editing" note="Protein sequence is in conflict with the conceptual codon_start=1 product="cytochrome oxidase subunit 3" protein_id="BAD83521.2" 258844..259641
//

>ABCDEF_32577
Normal: 34 16 57 32 61 84 No annotation
**********Hit in genome BA000042****************
ABCDEF_32577 319 BA000042 430597 98.02 101 0 1 1 101 70202 70300 2e-46 178
ABCDEF_32577 319 BA000042 430597 98.02 101 0 1 1 101 420952 421050 2e-46 178
ABCDEF_32577 319 BA000042 430597 93.48 46 3 0 1 46 112717 112672 5e-13 67.9
ABCDEF_32577 319 BA000042 430597 91.84 49 4 0 192 240 171703 171751 2e-12 65.9
ABCDEF_32577 319 BA000042 430597 92.11 38 3 0 97 134 54924 54887 3e-08 52.0
**********Hit in cds rrn26****************
rrn26
ABCDEF_32577 319 rrn26 3406 100.00 58 0 0 1 58 3349 3406 4e-28 115
417603..421008
//

>ABCDEF_30884
Normal: 211 59 304 243 202 60 ABCDEF_30884 2206 UniRef50_Q9T711 745 44.66 412 132 17 2120 1086 3 385
2e-67 244
**********Hit in genome BA000042****************
ABCDEF_30884 2206 BA000042 430597 96.35 2000 73 0 207 2206 4054 6053 0.0 3386
ABCDEF_30884 2206 BA000042 430597 100.00 30 0 0 44 73 361615 361586 8e-10 60.0
**********Hit in cds mat-R****************
mat-R
ABCDEF_30884 2206 mat-R 1977 96.28 1960 73 0 207 2166 1960 1 0.0 3307
exception="RNA editing" note="Protein sequence is in conflict with the conceptual codon_start=1 product="maturase-related protein" protein_id="BAD83417.2" complement(4036..6012)
//

>ABCDEF_49108
Normal: 83 29 134 88 92 28 ABCDEF_49108 873 UniRef50_Q6JP41 285 100.00 40 0 0 873 754 168 207
2e-16 83.2
**********Hit in genome BA000042****************
ABCDEF_49108 873 BA000042 430597 96.42 810 29 0 1 810 5923 6732 0.0 1376
ABCDEF_49108 873 BA000042 430597 96.83 63 2 0 811 873 142903 142965 4e-25 109
**********Hit in cds mat-R****************
mat-R
ABCDEF_49108 873 mat-R 1977 96.70 91 3 0 1 91 91 1 4e-40 157
exception="RNA editing" note="Protein sequence is in conflict with the conceptual codon_start=1 product="maturase-related protein" protein_id="BAD83417.2" complement(4036..6012)
//

>ABCDEF_18034
Normal: 41 13 61 35 51 58 ABCDEF_18034 849 UniRef50_Q9SMX4 164 80.00 65 13 0 652 846 68 132
2e-28 114
**********Hit in genome BA000042****************
ABCDEF_18034 849 BA000042 430597 95.31 768 36 0 82 849 44262 43495 0.0 1237
ABCDEF_18034 849 BA000042 430597 96.88 32 1 0 623 654 394243 394212 5e-09 56.0
**********Hit in cds rpl5****************
rpl5
ABCDEF_18034 849 rpl5 555 94.41 555 31 0 86 640 1 555 0.0 854
exception="RNA editing" note="Protein sequence is in conflict with the conceptual codon_start=1 product="ribosomal protein L5" protein_id="BAD83432.2" complement(43703..44257)
//

>ABCDEF_28688
Normal: 32 16 75 65 35 30 ABCDEF_28688 697 UniRef50_B9RWY9 213 58.43 89 7 1 191 457 151 209
5e-20 91.3
**********Hit in genome BA000042****************
ABCDEF_28688 697 BA000042 430597 94.41 465 21 2 1 461 196382 196845 0.0 706
ABCDEF_28688 697 BA000042 430597 91.01 178 11 3 525 697 196856 197033 2e-54 206
*************no hit in cds*****************
//

>ABCDEF_18390
Normal: 27 8 37 30 17 18 ABCDEF_18390 495 UniRef50_B9TAA3 86 83.87 31 4 1 241 333 34 63
6e-06 47.8
**********Hit in genome BA000042****************
ABCDEF_18390 495 BA000042 430597 97.58 495 12 0 1 495 195797 196291 0.0 886
*************no hit in cds*****************
//

>ABCDEF_18291
Normal: 97 23 104 40 43 44 ABCDEF_18291 801 UniRef50_C5YVV7 69 51.35 74 19 2 462 262 6 69
5e-09 57.8
**********Hit in genome BA000042****************
ABCDEF_18291 801 BA000042 430597 96.57 757 11 3 44 794 202000 201253 0.0 1294
ABCDEF_18291 801 BA000042 430597 96.77 31 1 0 667 697 328080 328050 2e-08 54.0
*************no hit in cds*****************
//

>ABCDEF_45105
Normal: 239 180 436 295 374 434 ABCDEF_45105 1708 UniRef50_Q8M1D7 146 40.32 124 64 5 611 255 1 119
3e-07 56.6
**********Hit in genome BA000042****************
ABCDEF_45105 1708 BA000042 430597 91.19 885 29 9 822 1698 330611 329768 0.0 1144
ABCDEF_45105 1708 BA000042 430597 91.54 615 40 4 1 611 258343 257737 0.0 791
ABCDEF_45105 1708 BA000042 430597 96.49 57 2 0 555 611 85531 85475 3e-21 97.6

ABCDEF_45105 1708 BA000042 430597 96.49 57 2 0 555 611 287031 286975 3e-21 97.6
ABCDEF_45105 1708 BA000042 430597 100.00 46 0 0 1653 1698 361500 361545 2e-19 91.7
ABCDEF_45105 1708 BA000042 430597 95.74 47 2 0 1456 1502 361433 361479 3e-15 77.8
ABCDEF_45105 1708 BA000042 430597 97.06 34 1 0 695 728 25850 25883 6e-10 60.0
ABCDEF_45105 1708 BA000042 430597 97.06 34 1 0 695 728 145368 145401 6e-10 60.0
ABCDEF_45105 1708 BA000042 430597 97.06 34 1 0 695 728 156246 156213 6e-10 60.0
**********Hit in cds orfB****************
orfB
ABCDEF_45105 1708 orfB 471 92.24 477 31 1 134 610 471 1 0.0 654
exception="RNA editing" note="Protein sequence is in conflict with the conceptual codon_start=1 product="orfB protein" protein_id="BAD83520.2" 257737..258207
//

>ABCDEF_26005
Normal: 47 21 89 68 38 74 ABCDEF_26005 518 UniRef50_Q35301 74 60.27 73 24 3 420 208 3 72
1e-11 63.9
**********Hit in genome BA000042****************
ABCDEF_26005 518 BA000042 430597 95.98 523 12 3 1 518 184296 183778 0.0 858
ABCDEF_26005 518 BA000042 430597 90.24 41 4 0 131 171 44880 44920 2e-07 50.1
**********Hit in cds nad4****************
nad4
ABCDEF_26005 518 nad4 1488 100.00 47 0 0 1 47 508 462 3e-21 93.7
exception="RNA editing" note="Protein sequence is in conflict with the conceptual codon_start=1 product="NADH dehydrogenase subunit 4" protein_id="BAD83480.2"
order(182370..182830,184249..1847
//

>ABCDEF_87306
Normal: 12 22 22 8 7 27 ABCDEF_87306 307 UniRef50_P93313 495 90.91 33 3 0 175 273 463 495
2e-11 65.5
**********Hit in genome BA000042****************
ABCDEF_87306 307 BA000042 430597 94.42 269 10 2 37 305 190618 190881 2e-114 404
ABCDEF_87306 307 BA000042 430597 89.47 38 4 0 83 120 153939 153976 7e-06 44.1
**********Hit in cds nad4****************
nad4
ABCDEF_87306 307 nad4 1488 94.38 89 5 0 189 277 1400 1488 1e-34 137
exception="RNA editing" note="Protein sequence is in conflict with the conceptual codon_start=1 product="NADH dehydrogenase subunit 4" protein_id="BAD83480.2"
order(182370..182830,184249..1847
//

>ABCDEF_4076
Normal: 27 18 55 43 30 62 ABCDEF_4076 502 UniRef50_Q5K5G0 239 97.96 49 1 0 150 4 1 49
6e-23 98.2
**********Hit in genome BA000042****************
ABCDEF_4076 502 BA000042 430597 95.18 415 9 4 3 407 258991 258578 0.0 646
ABCDEF_4076 502 BA000042 430597 91.22 148 10 2 147 292 257202 257056 1e-45 176
ABCDEF_4076 502 BA000042 430597 97.87 47 1 0 325 371 257056 257010 3e-18 85.7
**********Hit in cds cox3****************
cox3
ABCDEF_4076 502 cox3 798 100.00 147 0 0 3 149 147 1 5e-81 291
exception="RNA editing" note="Protein sequence is in conflict with the conceptual codon_start=1 product="cytochrome oxidase subunit 3" protein_id="BAD83521.2" 258844..259641
//

>ABCDEF_1640
Normal: 41 13 35 20 43 16 No annotation
**********Hit in genome BA000042****************
ABCDEF_1640 418 BA000042 430597 93.60 297 15 1 126 418 258524 258228 6e-124 436
ABCDEF_1640 418 BA000042 430597 95.65 46 2 0 274 319 275501 275546 3e-15 75.8
*************no hit in cds*****************
//

>ABCDEF_57153
Normal: 84 52 118 59 120 170 ABCDEF_57153 526 UniRef50_B9STB6 58 71.11 45 10 1 409 275 17 58
2e-08 54.7
**********Hit in genome BA000042****************
ABCDEF_57153 526 BA000042 430597 94.32 475 18 2 52 526 329937 330402 0.0 726
ABCDEF_57153 526 BA000042 430597 95.74 47 2 0 61 107 361479 361433 8e-16 77.8
*************no hit in cds*****************
//

>ABCDEF_8797
Normal: 23 28 39 20 17 52 ABCDEF_8797 361 UniRef50_Q9TAK8 453 44.66 103 56 1 1 309 348 449
5e-21 93.2
**********Hit in genome BA000042****************
ABCDEF_8797 361 BA000042 430597 97.93 241 5 0 1 241 188084 188324 1e-124 438
ABCDEF_8797 361 BA000042 430597 95.76 118 5 0 242 359 190764 190881 4e-51 194
**********Hit in cds nad4****************
nad4
ABCDEF_8797 361 nad4 1488 96.68 331 11 0 1 331 1158 1488 1e-164 569
exception="RNA editing" note="Protein sequence is in conflict with the conceptual codon_start=1 product="NADH dehydrogenase subunit 4" protein_id="BAD83480.2"
order(182370..182830,184249..1847
//

>ABCDEF_8186
Normal: 39 28 70 45 28 24 No annotation
**********Hit in genome BA000042****************
ABCDEF_8186 334 BA000042 430597 88.14 354 18 4 1 334 182323 181974 4e-106 377
*************no hit in cds*****************
//

>ABCDEF_5199
Normal: 9 16 25 17 17 12 No annotation
**********Hit in genome BA000042****************
ABCDEF_5199 299 BA000042 430597 94.78 134 5 2 168 299 356999 356866 3e-51 194
ABCDEF_5199 299 BA000042 430597 94.29 35 2 0 102 136 335916 335950 7e-09 54.0
*************no hit in cds*****************
//

>ABCDEF_25150

Normal: 75 31 86 65 79 68 ABCDEF_25150 586 UniRef50_Q9MFC6 107 85.19 108 15 1 554 231 1 107 5e-56 181
**********Hit in genome BA000042****************
ABCDEF_25150 586 BA000042 430597 97.95 585 12 0 1 585 200564 201148 0.0 1065
**********Hit in cds rpl2****************
rpl2
ABCDEF_25150 586 rpl2 996 96.00 25 1 0 232 256 390 414 1e-05 42.1
exception="RNA editing" note="Protein sequence is in conflict with the conceptual codon_start=1 product="ribosomal protein L2" protein_id="BAD83551.2" complement(order(361051..361168,363071..3
//

>ABCDEF_53379
Normal: 18 18 16 20 25 18 ABCDEF_53379 281 UniRef50_Q8RVA9 223 54.17 72 28 3 65 280 132 198 2e-12 65.9
**********Hit in genome BA000042****************
ABCDEF_53379 281 BA000042 430597 93.95 281 11 1 1 281 259577 259851 2e-120 424
**********Hit in cds orf125e****************
orf125e
ABCDEF_53379 281 orf125e 378 93.95 281 11 1 1 281 8 282 3e-121 424
exception="RNA editing" note="Protein sequence is in conflict with the conceptual codon_start=1 product="hypothetical protein" protein_id="BAD83522.2" 259569..259946
//

***************Undetected cds*****************
>orf215 note="A part of retroelement polyprotein" codon_start=1 product="hypothetical protein" protein_id="BAD83419.1" 8801..9448
>orf134b codon_start=1 product="hypothetical protein" protein_id="BAD83564.1" complement(406181..406585)
>orf108 codon_start=1 product="hypothetical protein" protein_id="BAD83450.1" complement(110055..110381)
>orf134 codon_start=1 product="hypothetical protein" protein_id="BAD83482.1" 187937..188341
>orf112b codon_start=1 product="hypothetical protein" protein_id="BAD83428.1" complement(34105..34443)
>orf110b codon_start=1 product="hypothetical protein" protein_id="BAD83546.1" complement(345553..345885)
>orf125d note="identical to orf124 in Beta vulgaris" codon_start=1 product="hypothetical protein" protein_id="BAD83519.1" 257199..257576
>orf138c codon_start=1 product="hypothetical protein" protein_id="BAD83505.1" note="orf138c; identical to orf138b" codon_start=1 product="hypothetical protein" protein_id="BAD83567.1" complement(417511..417927)
>orf109b note="a part of putative invertase" codon_start=1 product="hypothetical protein" protein_id="BAD83540.1" complement(318502..318831)
>orf102a codon_start=1 product="hypothetical protein" protein_id="BAD83455.1" 115384..115692
>orf132 codon_start=1 product="hypothetical protein" protein_id="BAD83434.1" note="a part of non-LTR retroelement" codon_start=1 product="hypothetical protein" protein_id="BAD83457.1" 122839..123237
>orf125b codon_start=1 product="hypothetical protein" protein_id="BAD83445.1" 95890..96267
>rrn5 110399..110517
>orf101b codon_start=1 product="hypothetical protein" protein_id="BAD83489.1" complement(197310..197615)
>orf151 note="a part of copia-like polyprotein" codon_start=1 product="hypothetical protein" protein_id="BAD83447.1" complement(101746..102201)
>rpl16 exception="RNA editing" note="Protein sequence is in conflict with the conceptual codon_start=1 product="ribosomal protein L16" protein_id="BAD83475.2" <155609..156085
>trnC(gca) 197450..197520
>orf130a codon_start=1 product="hypothetical protein" protein_id="BAD83451.1" 110888..111280
>orf210 note="homologous to orf239 in Phaseolus vulgaris" codon_start=1 product="hypothetical protein" protein_id="BAD83561.1" complement(394481..395113)
>orf143 codon_start=1 product="hypothetical protein" protein_id="BAD83501.1" 226196..226627
>orf111c note="a part of ndhB in chloroplast" codon_start=1 product="hypothetical protein" protein_id="BAD83518.1" 256039..256374
>orf190 codon_start=1 product="hypothetical protein" protein_id="BAD83517.1" 255551..256123
>nad3 exception="RNA editing" note="Protein sequence is in conflict with the conceptual codon_start=1 product="NADH dehydrogenase subunit 3" protein_id="BAD83443.2" codon_start=1 product="NADH dehydrogenase subunit 3" protein_id="BAD83531.1" 287895..288251
>orf265a note="orf87 in N. sylbestris is a part of orf265a" codon_start=1 product="hypothetical protein" protein_id="BAD83442.1" 85475..86272
>orf297a note="A part of DNA polymerase homolog" codon_start=1 protein_id="BAD83421.1" complement(10811..11704)
>orf120 codon_start=1 product="hypothetical protein" protein_id="BAD83436.1" 58774..59136
>orf118 codon_start=1 product="hypothetical protein" protein_id="BAD83507.1" complement(229726..230082)
>trnF(gaa) complement(325701..325774)
>atp9 exception="RNA editing" note="Protein sequence is in conflict with the conceptual codon_start=1 product="ATP synthase F0 subunit 9" protein_id="BAD83468.2" complement(145767..145991)
>orf161 codon_start=1 product="hypothetical protein" protein_id="BAD83433.1" 48550..49035
>rps4 exception="RNA editing" note="Protein sequence is in conflict with the conceptual codon_start=1 product="ribosomal protein S4" protein_id="BAD83538.2" complement(314124..>315173)
>orf136 codon_start=1 product="hypothetical protein" protein_id="BAD83477.1" complement(163069..163479)
>orf103d codon_start=1 product="hypothetical protein" protein_id="BAD83548.1" complement(357325..357636)
>orf112c codon_start=1 product="hypothetical protein" protein_id="BAD83563.1" 401787..402125
>ccmC exception="RNA editing" note="Protein sequence is in conflict with the conceptual codon_start=1 product="cytochrome c maturation protein CcmC" protein_id="BAD83423.2" complement(19014..19766)
>orf138b codon_start=1 product="hypothetical protein" protein_id="BAD83437.1" complement(66761..67177)
>orf114b codon_start=1 product="hypothetical protein" protein_id="BAD83502.1" 226673..227017
>orf125a codon_start=1 product="hypothetical protein" protein_id="BAD83415.1" 825..1202
>orf102 codon_start=1 product="hypothetical protein" protein_id="BAD83560.1" complement(392345..392653)
>orf115 codon_start=1 product="hypothetical protein" protein_id="BAD83535.1" complement(306250..306597)
>orf265b note="orf265b; identical to orf265a" codon_start=1 product="hypothetical protein" protein_id="BAD83530.1" 286975..287772
>orf222 codon_start=1 product="hypothetical protein" protein_id="BAD83454.1" complement(114996..115664)
>orf144 note="orf105c in Beta vulgaris" codon_start=1 product="hypothetical protein" protein_id="BAD83506.1" 229441..229875
>orf101a codon_start=1 product="hypothetical protein" protein_id="BAD83471.1" complement(150521..150826)
>trnH(gug) 331955..332028
>orf120b codon_start=1 product="hypothetical protein" protein_id="BAD83526.1" note="orf120b; identical to orf120a" codon_start=1 product="hypothetical protein" protein_id="BAD83566.1" 409524..409886
>trnW(cca) 337782..337855
>orf122b codon_start=1 product="hypothetical protein" protein_id="BAD83544.1" 330483..330851
>orf116b note="a part of orf110b in Beta vulgaris" codon_start=1 product="hypothetical protein" protein_id="BAD83543.1" 325478..325828
>orf173 codon_start=1 product="hypothetical protein" protein_id="BAD83441.1" codon_start=1 product="hypothetical protein" protein_id="BAD83570.1" 428417..428938
>orf119b codon_start=1 product="hypothetical protein" protein_id="BAD83494.1" complement(210050..210409)
>orf121c codon_start=1 product="hypothetical protein" protein_id="BAD83562.1" complement(400893..401258)
>orf101d note="a part of ndhB in chloroplast" codon_start=1 product="hypothetical protein" protein_id="BAD83516.1" 254902..255207
>orf112a codon_start=1 product="hypothetical protein" protein_id="BAD83424.1" 19804..20142
>orf158 codon_start=1 product="hypothetical protein" protein_id="BAD83464.1" 139417..139893
>orf125g codon_start=1 product="hypothetical protein" protein_id="BAD83558.1" complement(382408..382785)
>orf114d codon_start=1 product="hypothetical protein" protein_id="BAD83514.1" 244197..244541
>orf104c codon_start=1 product="hypothetical protein" protein_id="BAD83533.1" complement(299287..299601)
>trnK(uuu) complement(356762..356834)
>orf129b codon_start=1 product="hypothetical protein" protein_id="BAD83446.1" 100135..100524
>orf166b note="a part of orf291 in Arabidopsis" codon_start=1 product="hypothetical protein" protein_id="BAD83542.1" complement(324805..325305)
>orf216 exception="RNA editing" note="Protein sequence is in conflict with the conceptual codon_start=1 product="hypothetical protein" protein_id="BAD83485.2" 191161..191811
>orf103b note="a part of ABC transporter-like protein" codon_start=1 product="hypothetical protein" protein_id="BAD83493.1" 207255..207566
>atp6 exception="RNA editing" note="Protein sequence is in conflict with the conceptual codon_start=1 product="ATP synthase F0 subunit 6" protein_id="BAD83425.2" complement(24618..25766)
>trnE(uuc) complement(329387..329458)

>orf107 note="a part of ccmF protein" codon_start=1 product="hypothetical protein" protein_id="BAD83513.1" complement(240552..240875)
>orf121a codon_start=1 product="hypothetical protein" protein_id="BAD83431.1" 43423..43788
>trnS(gga) complement(178217..178303)
>orf114c codon_start=1 product="hypothetical protein" protein_id="BAD83512.1" 238858..239202
>orf131c codon_start=1 product="hypothetical protein" protein_id="BAD83541.1" 319970..320365
>orf159a codon_start=1 product="hypothetical protein" protein_id="BAD83469.1" complement(146237..146716)
>orf315 note="a part of rpl2" codon_start=1 product="hypothetical protein" protein_id="BAD83461.1" complement(131729..132676)
>orf141 note="a part of reverse transcriptase" codon_start=1 product="hypothetical protein" protein_id="BAD83559.1" complement(385463..385888)
>trnQ(uug) complement(228472..228543)
>orf125 note="non-LTR retroelement" codon_start=1 product="hypothetical protein" protein_id="BAD83511.1" 236931..237308
>trnI(cau) 121049..121122
>orf133 note="identical to exon1 of chloroplast rpl2" codon_start=1 product="hypothetical protein" protein_id="BAD83422.1" complement(18091..18492)
>orf111 codon_start=1 product="hypothetical protein" protein_id="BAD83472.1" 150547..150882
>nad2 exception="RNA editing" trans_splicing note="Protein sequence is in conflict with the conceptual codon_start=1 product="NADH dehydrogenase subunit 2" protein_id="BAD83439.2" complement(427219..427371)
>trnP(ugg) 337551..337624
>orf197 codon_start=1 product="hypothetical protein" protein_id="BAD83438.1" complement(74704..75297)
>orf105 codon_start=1 product="hypothetical protein" protein_id="BAD83490.1" codon_start=1 product="hypothetical protein" protein_id="BAD83553.1" 373057..373374
>orf152 note="a part of polyprotein" codon_start=1 product="hypothetical protein" protein_id="BAD83449.1" complement(104658..105116)
>orf177 codon_start=1 product="hypothetical protein" protein_id="BAD83427.1" 31683..32216
>orf102b codon_start=1 product="hypothetical protein" protein_id="BAD83456.1" 121986..122294
>orf274 note="CDS is reported in Acc# AF121903" codon_start=1 product="hypothetical protein" protein_id="BAD83523.1" 260411..261235
>orf109a codon_start=1 product="hypothetical protein" protein_id="BAD83510.1" complement(234001..234330)
>orf155 codon_start=1 product="hypothetical protein" protein_id="BAD83498.1" 220922..221389
>orf111b codon_start=1 product="hypothetical protein" protein_id="BAD83503.1" 227311..227646
>orf160 codon_start=1 product="hypothetical protein" protein_id="BAD83508.1" 231140..231622
>orf101c codon_start=1 product="hypothetical protein" protein_id="BAD83495.1" complement(211251..211556)
>orf239 codon_start=1 product="hypothetical protein" protein_id="BAD83478.1" 171890..172609
>orfX exception="RNA editing" note="Protein sequence is in conflict with the conceptual codon_start=1 product="hypothetical protein" protein_id="BAD83547.2" complement(350541..>351371)
>orf123 codon_start=1 product="hypothetical protein" protein_id="BAD83552.1" complement(364374..364745)
>orf306 codon_start=1 product="hypothetical protein" protein_id="BAD83496.1" 215367..216287
>orf137 codon_start=1 product="hypothetical protein" protein_id="BAD83539.1" complement(315416..315829)
>ccmFc exception="RNA editing" note="Protein sequence is in conflict with the conceptual codon_start=1 product="cytochrome c maturation protein CcmFc" protein_id="BAD83536.2" order(307057..307824,308778..309327)
>orf110a codon_start=1 product="hypothetical protein" protein_id="BAD83426.1" complement(31411..31743)
>ccmB exception="RNA editing" note="Protein sequence is in conflict with the conceptual codon_start=1 product="cytochrome c maturation protein CcmB" protein_id="BAD83549.2" 359373..359993
>orf175 codon_start=1 product="hypothetical protein" protein_id="BAD83448.1" complement(102481..103008)
>nad4L exception="RNA editing" note="Protein sequence is in conflict with the conceptual codon_start=1 product="NADH dehydrogenase subunit 4L" protein_id="BAD83453.2" complement(114638..114940)
>orf119c note="orf119c; identical to orf119a" codon_start=1 product="hypothetical protein" protein_id="BAD83565.1" complement(408917..409276)
>orf171a codon_start=1 product="hypothetical protein" protein_id="BAD83418.1" complement(7187..7702)
>orf129d note="orf139 in Arabidopsis" codon_start=1 product="hypothetical protein" protein_id="BAD83515.1" 251000..251389
>orf105a codon_start=1 product="hypothetical protein" protein_id="BAD83463.1" complement(138190..138507)
>orf129a codon_start=1 product="hypothetical protein" protein_id="BAD83420.1" 9998..10387
>orf202 codon_start=1 product="hypothetical protein" protein_id="BAD83534.1" complement(304155..304763)
>nad9 exception="RNA editing" note="Protein sequence is in conflict with the conceptual codon_start=1 product="NADH dehydrogenase subunit 9" protein_id="BAD83545.2" 336111..336683
>orf197b note="orf197b;identical to orf197a" codon_start=1 product="hypothetical protein" protein_id="BAD83568.1" complement(425454..426047)
>trnfM(cau) 416951..417024
>trnG(gcc) complement(231805..231876)
>sdh3 exception="RNA editing" note="Protein sequence is in conflict with the conceptual codon_start=1 product="succinate dehydrogenase subunit 3" protein_id="BAD83440.2" codon_start=1 product="succinate dehydrogenase subunit 3" protein_id="BAD83529.1" codon_start=1 product="succinate dehydrogenase subunit 3" protein_id="BAD83569.1" complement(427948..428274)
>trnM(cau) 272515..272587
>orf171 codon_start=1 product="hypothetical protein" protein_id="BAD83481.1" 184337..184852
>orf119a codon_start=1 product="hypothetical protein" protein_id="BAD83435.1" complement(58167..58526)
>orf121b codon_start=1 product="hypothetical protein" protein_id="BAD83525.1" 264195..264560
>orf104 codon_start=1 product="hypothetical protein" protein_id="BAD83483.1" 189316..189630
>orf103c codon_start=1 product="hypothetical protein" protein_id="BAD83504.1" complement(227753..228064)
>orf142 note="homologous to orf117 in Beta vulgaris" codon_start=1 product="hypothetical protein" protein_id="BAD83466.1" complement(142756..143184)
>pseudo-trnD(guc) complement(166843..166908)
>orf129 codon_start=1 product="hypothetical protein" protein_id="BAD83465.1" 140970..141359
>nad7 exception="RNA editing" note="Protein sequence is in conflict with the conceptual codon_start=1 product="NADH dehydrogenase subunit 7" protein_id="BAD83554.2" complement(order(375325..375586,377323..378033,379489..379557,380473..380615))
>trnY(gua) 200481..200563
>orf131b codon_start=1 product="hypothetical protein" protein_id="BAD83527.1" codon_start=1 product="hypothetical protein" protein_id="BAD83528.1" 275051..275446
>orf138a codon_start=1 product="hypothetical protein" protein_id="BAD83416.1" 2387..2803
>trnS(uga) 99558..99644
>orf237 codon_start=1 product="hypothetical protein" protein_id="BAD83470.1" complement(147138..147851)
>orf106b codon_start=1 product="hypothetical protein" protein_id="BAD83479.1" complement(177519..177839)
>orf103e codon_start=1 product="hypothetical protein" protein_id="BAD83557.1" complement(381966..382277)
>orf106a codon_start=1 product="hypothetical protein" protein_id="BAD83462.1" 134607..134927
>rps13 exception="RNA editing" note="Protein sequence is in conflict with the conceptual codon_start=1 product="ribosomal protein S13" protein_id="BAD83467.2" complement(144929..145279)
>trnD(guc) complement(179000..179073)
>ccmFN exception="RNA editing" note="Protein sequence is in conflict with the conceptual codon_start=1 product="cytochrome c maturation protein CcmFN" protein_id="BAD83458.2" complement(124378..126102)
>orf104b note="a part of poly polyprotein" codon_start=1 product="hypothetical protein" protein_id="BAD83509.1" 232685..232999
