# Supplementary material for: Dissecting organ-specific transcriptomes through RNA-sequencing
Source: Plant Methods. 2013 Oct 25;9:42. doi: 10.1186/1746-4811-9-42 (PMC3819660; doi:10.1186/1746-4811-9-42)
Supplement: Additional file 6 — Epigenetic events during petal pigmentation. [file 1746-4811-9-42-S6.pdf]

Additional file 6

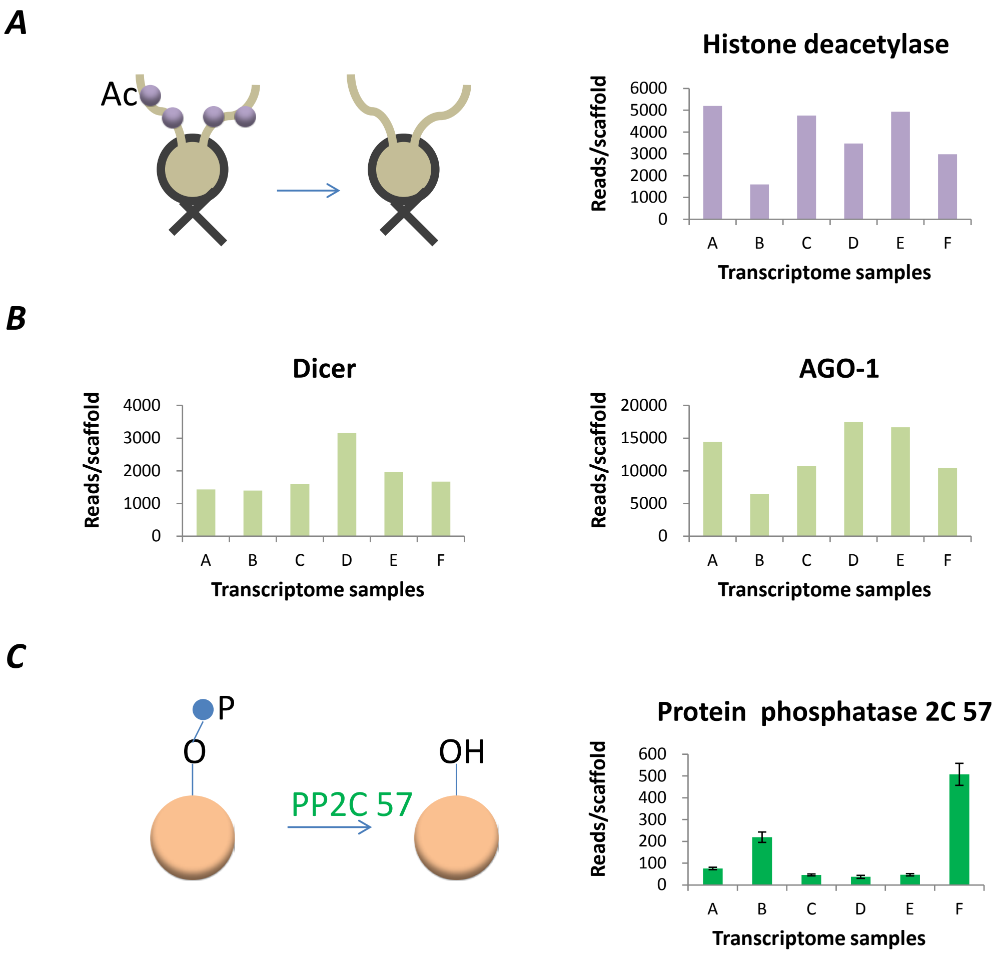

Epigenetic events detected among samples A -> F

(*A*) Histone deacetylase was found in multiple copies, with the bars indicating the variations of their expression levels. (*B*) Contrasting expression patterns of homologs for dicer (ABCDEF_40338) and argonaute 1 (AGO-1; ABCDEF_38770). (*C*) Expression pattern of a protein phosphatase across samples, with variations of abundance levels from multiple copies shown by bars.
