# Supplementary material for: Dissecting organ-specific transcriptomes through RNA-sequencing
Source: Plant Methods. 2013 Oct 25;9:42. doi: 10.1186/1746-4811-9-42 (PMC3819660; doi:10.1186/1746-4811-9-42)
Supplement: Additional file 7 — Perl scripts for extracting and organizing KEGG information. [file 1746-4811-9-42-S7.pdf]

Additional file 7

Perl scripts and command lines for extracting KEGG information

I. Two data files may be downloaded from KEGG (ftp://ftp.genome.jp/pub/kegg/): enzyme and ec_map.tab, which provide EC numbers and the corresponding enzyme names.
Following two command lines: EC2Name.pl enzyme EC2Name, and Map2EC.pl ec_map.tab Map2EC, the output file "Map2EC" will list all entries of enzymes under each numbered KEGG pathway/network from the two files.

II. From blastx search of uniref50, an annotation file may be generated to provide information on GO process and EC number. The following command lines: BlastAnnotation.pl Assembly_blastx_uniref50 uniref50.fasta uniref50.dat BlastAnnotation, and Gene2ECOnE.pl BlastAnnotation Threshold Gene2ECOnE, will lead to an organized file with the columns showing scaffold ID, the corresponding uniref50 sequence ID, the EC number, and the e-value of the blastx. The threshold is the pre-defined e-value for the marching search.

<u>Perl scripts</u>

```
************EC2Name.pl*****************
#!/usr/bin/perl -w
use strict;
my (%EC2Name,$EC);

sub ReadEnzyme{
	open(IN,"<$ARGV[0]") or die "Can't open $ARGV[0]\n";
	while(<IN>){
		chomp;
		if($_=~/^ENTRY\s+EC\s+(\S+)/){
			$EC=$1;
			$_=<IN>;
			if($_=~/^NAME\s+(\S.+)/||$_=~/^CLASS\s+(\S.+)/){$EC2Name{$EC}=$1;}
			else{print "$EC:No name\n";}
		}
	}
	close(IN);
}

sub Write{
	open(OUT,">$ARGV[1]") or die "Can't open $ARGV[1]\n";
	for $EC (keys %EC2Name){printf OUT "$EC\t$EC2Name{$EC}\n";}
	close(OUT);
```

```
}

# main program
@ARGV or die "Usage:EC2Name.pl enzyme Gene2EC\n";
ReadEnzyme();
Write();
```

****************Map2EC.pl****************

```
#!/usr/bin/perl -w
use strict;
my (%Map2EC,$Map,$EC,$Tmp);

sub Read{
	open(IN,"<$ARGV[0]") or die "Can't open $ARGV[0]\n";
	while(<IN>){
		if($_=~/^(\d+\.\S+\.\S+\.\S+)\s+(\d.+)/){
			$EC=$1; $Tmp=$2;
			while($Tmp=~/(\d+)/g){$Map2EC{$1}{$EC}++;}
		}
	}
	close(IN);
}

sub Write{
	open(OUT,">$ARGV[1]") or die "Can't open $ARGV[1]\n";
	for $Map (sort keys %Map2EC){
		printf OUT ">$Map\n";
		for $EC (sort keys %{$Map2EC{$Map}}){printf OUT "\t$EC\n";}
	}
	close(OUT);
}

# main program
@ARGV or die "Usage:Map2EC.pl ec_map.tab Map2EC\n";
Read();
Write();
```

*************BlastAnnotation.pl************

```
#!/usr/bin/perl -w
use strict;
my (%Blast,%Anno,$Gene,$Pep);

sub ReadBlast_m8{
```

```
	print "Reading blast ...\n";
	open(IN,"<$ARGV[0]") or die "Can't open $ARGV[0]!\n";
	while(<IN>){
		chomp;
		my @Tmp1=split(/\s+/,$_);
		if(!defined $Blast{$Tmp1[0]}{$Tmp1[2]}){$Blast{$Tmp1[0]}{$Tmp1[2]}=$_;}
		else{
			my @Tmp2=split(/\s+/,$Blast{$Tmp1[0]}{$Tmp1[2]});
			if($Tmp1[12]<$Tmp2[12]){$Blast{$Tmp1[0]}{$Tmp1[2]}=$_;}
		}
	}
	close(IN);
}

sub ReadAnnotation{
	print "Reading Title ...\n";
	open(IN,"<$ARGV[1]") or die "Can't open $ARGV[1]!\n";
	while(<IN>){
		chomp;
		if($_=~/^>UniRef50_(\S+)\s+(\S.+)/){$Anno{$1}=$2;}
	}
	close(IN);

	open(IN,"<$ARGV[2]") or die "Can't open $ARGV[2]\n";
	print "Reading annotation ...\n";
	while(<IN>){
		if($_=~/^AC\s+(\w+)/&&defined $Anno{$1}){
			$Pep=$1;
			while(<IN>){
				chomp;
				if($_=~/^\/\//){last;}
				elsif($_=~/^DR\s+(GO; GO.+)/){$Anno{$Pep}.=" | $1";}
				elsif($_=~/^CC\s+.+?(SUBCELLULAR LOCATION.+)/){$Anno{$Pep}.=" | $1";}
				elsif($_=~/^DR\s+(KEGG; .+)/){$Anno{$Pep}.=" | $1";}
				elsif($_=~/^DE\s+(EC=.+?);/){$Anno{$Pep}.=" | $1";}
			}
		}
	}
	close(IN);
}

sub Print{
	open(OUT,">$ARGV[3]") or die "Can't open $ARGV[3]\n";
	for $Gene (sort keys %Blast){
```

```
		for $Pep (sort keys %{$Blast{$Gene}}){
			printf OUT "$Blast{$Gene}{$Pep}\tAnnotation:";
			$Pep=~/^UniRef50_(\S+)/;
			if(defined $Anno{$1}){print OUT "$Anno{$1}\n";}
			else{print OUT "no annotation\n";}
		}
	}
	close(OUT);
}

#main
@ARGV or die "Usage:$0 blast_m8 Title Uniprot.dat Result\n";
ReadBlast_m8();
ReadAnnotation();
Print();
```

****************Gene2ECOnE.pl******************

```
#!/usr/bin/perl -w
use strict;

my (%Gene2EC,$Gene,$Pep,$EC,$Evalue);
@ARGV or die "Usage:$0 BlastAnnotation Threshold Gene2ECOnE\n";
open(IN,"<$ARGV[0]") or die "Can't open $ARGV[0]\n";
while(<IN>){
	if($_=~/^(\S+)\s+\S+\s+(\S+).+?(\S+)\s+\S+\s+Annotation.+?EC=(\S+)/&&$3<=$ARGV[1])
{
		$Gene2EC{$1}{$2}{$4}{$3}++;
	}
}
close(IN);

open(OUT,">$ARGV[2]") or die "Can't open $ARGV[2]\n";
for $Gene (sort keys %Gene2EC){
	for $Pep (keys %{$Gene2EC{$Gene}}){
		for $EC (keys %{$Gene2EC{$Gene}{$Pep}}){
			for $Evalue (keys %{$Gene2EC{$Gene}{$Pep}{$EC}}){
				print OUT "$Gene\t$Pep\t$EC\t$Evalue\n";
			}
		}
	}
}
close(OUT);
```
