# Supplementary material for: Dissecting organ-specific transcriptomes through RNA-sequencing
Source: Plant Methods. 2013 Oct 25;9:42. doi: 10.1186/1746-4811-9-42 (PMC3819660; doi:10.1186/1746-4811-9-42)
Supplement: Additional file 8 — Perl scripts for organelle genome mapping. [file 1746-4811-9-42-S8.pdf]

Additional file 8

Mapping of chloroplast and mitochondrion transcripts

The data-handling details of subcellular genomic classifications are explained here, corresponding to these summarized in Table 2 and pie-chart (Figure 2) in the text.

Input files include the final assembly "trinity.tgicl", the expression abundance level file "trinity.gsExprn5", chloroplast genome sequence file "IpChlo.fa", and mitochondrial genome sequence file "NicoMito.fa".

For Table 2, three phases of data handling are involved.

(1). A survey of the normalized numbers for each scaffold of the samples is done to determine expression patterns among tissues.

Usage:final2tissue.pl gsExprn CoverageTH

Here, gsExprn is from the normalization procedure, and CoverageTH is for the threshold of the lowest normalized number (we used 2). The output generates six list files corresponding to the samples. Each of these output files takes the place of List on the next line:

Usage:SelectFasta.pl List Input Output

where Input is trinity.tgicl, and Output is a fasta file for sequences that have satisfied the minimum normalization number for each sample.

(2). Each of the six fasta files from SelectFasta.pl is queried against IpChlo.fa and NicoMito.fa, respectively, using blastn:

Usage:Blastn.pl Query Database EValue BlastnOutput b_value

Query is the fasta file, and Database is one of the organelle sequence files. Evalue and b_value are transmitted, respectively, to the -e and -b parameter of blastn (we used EValue=1e-5, b_value=1).

BlastnOutput is then reorganized.

Usage： gsBlastOut.pl Query Database BlastnOutput gsBlastOutput

where Query, Database, and BlastnOutput are the same as in Blastn.pl, the output gsBlastOutput has additional information on lengths of the query and database sequences. This file is further cleaned with criteria below:

Usage:QryAlnL.pl gsBlastOutput IdentityTH AlnLTH Result

For IpChlo: IdentityTH: Identity⩾95%, AlnLTH: AlignedLength⩾0.8;
For NicoMito: IdentityTH:Identity⩾80%, AlnLTH: AlignedLength⩾0.5.

As there is a likelihood of a contig being simultaneously included in two subcellular genomes after QryAlnL.pl, visual examinations are required here for the results. In case of double listing, the classification of the scaffold involved was determined by a better blast result to whichever organelle genome.

A Linux command is then used to create a list of 6 files from QryAlnL.pl for organelle genome:

```
ls ?_Blastn_IpChlo.QrlAln.95_0.8 > chlo.list
ls ?_Blastn_NicoMito.QrlAln.80_0.5 > mito.list
```

Here ?_Blastn_IpChlo.QrlAln.95_0.8 and ?_Blastn_NicoMito.QrlAln.80_0.5 are results from QryAlnL.pl, from which the ambiguities have been excluded. The sequences not included in the two lists are considered nuclear.

(3). Assortments of scaffolds among samples and subcellular genomes.

Usage:final2tissueAboundance.pl gsExprn CoverageTH chlo.list mito.list result

Input file gsExprn and parameter CoverageTH are the same as those defined in final2tissue.pl. The output "result" summaries scaffold numbers and average expression levels of *Ipomoea purpurea* transcriptomes in three organs (Table 2 in the text).

II. Classification of transcript distributions (data for the pie-chart making)

To know transcript distribution among samples, a collective data file is created on the outputs after QryAlnL.pl by a Linux command:

```
cat ?_Blastn_IpChlo.QrlAln.95_0.8 > trinity_Hit_Chlo.QryAln.95_0.8
cat ?_Blastn_NicoMito.QrlAln.80_0.5 > trinity_Hit_Mito.QryAln.80_0.5
```

Here, ?_Blastn_IpChlo.QrlAln.95_0.8 and ?_Blastn_NicoMito.QrlAln.80_0.5 are the same as the above, including information on scaffold ID and organelle genome ID.

Their transcript abundance levels can be obtained as the following:

Usage:SelectExprn.pl list expression Result

Here list is from trinity_Hit_Chlo.QryAln.95_0.8 and trinity_Hit_Mito.QryAln.80_0.5, and expression is trinity.gsExprn5.

A pie graph based on Result above can be made based on data from Summary:

Usage:final2Pie.pl gsExprn MinCoverageTH Summary

Here, gsExprn is from SelectExprn.pl, MinCoverageTH is a pre-defined minimum number of normalized read value (we used 2), Summary records the scaffold numbers for each sample combination of transcript distribution.

Perl scripts:

*************final2tissue.pl************

```
#!/usr/bin/perl -w
use strict;
```

```
my (%Coverage,$Tissue,$ID);
my @Tissue=qw(A B C D E F);

sub Read{
	open(IN,"<$ARGV[0]") or die "Can't open $ARGV[0]!\n";
	while(<IN>){
		if($_=~/^(\S+)\s+.+?Normal\:\s+(\S+)\s+(\S+)\s+(\S+)\s+(\S+)\s+(\S+)\s+(\S+)/){
			$Coverage{$1}{A}=$2;
			$Coverage{$1}{B}=$3;
			$Coverage{$1}{C}=$4;
			$Coverage{$1}{D}=$5;
			$Coverage{$1}{E}=$6;
			$Coverage{$1}{F}=$7;
		}
	}
	close(IN);
	my $GeneN=keys %Coverage;
	print "GeneN=$GeneN\n";
}

sub Print{
	my $FileA="A.final";
	my $FileB="B.final";
	my $FileC="C.final";
	my $FileD="D.final";
	my $FileE="E.final";
	my $FileF="F.final";
	open(A,">$FileA") or die "Can't open $FileA\n";
	open(B,">$FileB") or die "Can't open $FileB\n";
	open(C,">$FileC") or die "Can't open $FileC\n";
	open(D,">$FileD") or die "Can't open $FileD\n";
	open(E,">$FileE") or die "Can't open $FileE\n";
	open(F,">$FileF") or die "Can't open $FileF\n";
	for $ID (keys %Coverage){
		for $Tissue (@Tissue){
			if($Coverage{$ID}{$Tissue}>$ARGV[1]){
				if($Tissue eq "A"){print A "$ID\n";}
				elsif($Tissue eq "B"){print B "$ID\n";}
				elsif($Tissue eq "C"){print C "$ID\n";}
				elsif($Tissue eq "D"){print D "$ID\n";}
				elsif($Tissue eq "E"){print E "$ID\n";}
				elsif($Tissue eq "F"){print F "$ID\n";}
			}
		}
```

```
	}
	close(A);
	close(B);
	close(C);
	close(D);
	close(E);
	close(F);
}

#main
@ARGV or die "Usage:$0 gsExprn CoverageTH\n";
Read();
Print();

*************SelectFasta.pl************
#! /usr/bin/perl -w

use strict;

@ARGV or die "Usage:$0 List Input Output\n";
my(%List,%Seq,$ID);

open(IN,"<$ARGV[0]") or die "Can not open $ARGV[0]\n";
while(<IN>){if($_=~/^(\S+)/){$List{$1}++;}}
close(IN);

open(IN,"<$ARGV[1]") or die "Can not open $ARGV[1]\n";
while(<IN>){
	if($_=~/^>(\S+)/){$ID=$1; $Seq{$ID}=$_;}
	else{chomp; $Seq{$ID}.=$_;}
}
close(IN);

open(OUT,">$ARGV[2]") or die "Can not open $ARGV[2]\n";
for $ID (keys %Seq){
	if(defined $List{$ID}){print OUT "$Seq{$ID}\n";}
}
close(OUT);


*****************gsBlastOut.pl*****************
#!/usr/bin/perl -w
use strict;
my (%QueryL,%SubjectL,%Record,$ID,$Num,$i,$j);
```

```
sub ReadSeqL{
	my ($File,$Ptr)=@_;
	open(IN,"<$File") or die "Can't open $File!\n";
	my %Seq;
	while(<IN>){
		chomp;
		if($_=~/^>(\S+)/){$ID=$1;}
		else{$Seq{$ID}.=$_; }
	}
	close(IN);
	for $ID (keys %Seq){$Ptr->{$ID}=length($Seq{$ID});}
}

sub ReadBlast_m8{
	my ($File,$Ptr)=@_;
	open(IN,"<$File") or die "Can't open $File!\n";
	$Num=0;
	while(<IN>){chomp; $Ptr->{$Num++}=$_;}
	close(IN);
}

sub Print{
	open(OUT,">$ARGV[3]") or die "Can't open $ARGV[3]\n";
	for($i=0;$i<$Num;$i++){
		my @Tmp=split(/\s+/,$Record{$i});
		printf OUT
"%-20s%-8s%-25s%-8s",$Tmp[0],$QueryL{$Tmp[0]},$Tmp[1],$SubjectL{$Tmp[1]};
		printf OUT "%-20s%-8s%-35s",$Tmp[0],$QueryL{$Tmp[0]},$Tmp[1];
		for($j=2;$j<=11;$j++){printf OUT "%-8s",$Tmp[$j];}
		printf OUT "\n";
	}
	close(OUT);
}
```

*************QryAlnL.pl*************

```
#! /usr/bin/perl -w
use strict;
my (%QryL,%Hit,%QAlnL,$Qry,$Sbjt,$Pos);

sub Read{
	open(IN,"<$ARGV[0]")or die "Can not open $ARGV[0]\n";
	while(<IN>){
		my @Tmp=split(/\s+/,$_);
```

```
		$QryL{$Tmp[0]}=$Tmp[1];
		if($Tmp[4]>=$ARGV[1]){$Hit{$Tmp[0]}{$Tmp[2]}{$Tmp[8]." ".$Tmp[9]}++;}
	}
	close(IN);

	for $Qry (keys %Hit){
		my ($MaxSbjt,$MaxL);
		$MaxL=0; $MaxSbjt="";
		for $Sbjt (keys %{$Hit{$Qry}}){
			my ($L,@Map,$i,$Start,$End);
			for($i=0;$i<$QryL{$Qry};$i++){$Map[$i]=" ";}
			for $Pos (keys %{$Hit{$Qry}{$Sbjt}}){
				$Pos=~/^(\d+)\s(\d+)/;
				if($1<=$2){$Start=$1; $End=$2;}
				else{$Start=$2; $End=$1;}
				for($i=$Start;$i<=$End;$i++){$Map[$i]="+";}
			}
			$L=0;
			for($i=0;$i<$QryL{$Qry};$i++){if($Map[$i] eq "+"){$L++;}}
			if($L>$MaxL){$MaxL=$L; $MaxSbjt=$Sbjt;}
		}
		if($MaxL!=0){$QAlnL{$Qry}{$MaxSbjt}=$MaxL/$QryL{$Qry};}
	}
}

sub Print{
	open(OUT,">$ARGV[3]") or die "Can not open $ARGV[3]\n";
	for $Qry (keys %QAlnL){
		for $Sbjt (keys %{$QAlnL{$Qry}}){
			if($QAlnL{$Qry}{$Sbjt}>=$ARGV[2]){
				printf OUT "$Qry\t$Sbjt\t%6.3f\n",$QAlnL{$Qry}{$Sbjt};
			}
		}
	}
	close(OUT);
}

# main program
@ARGV or die "Usage:$0 Blast.gs_m8 IdentityTH AlnLTH Result\n";
Read();
Print();

**************final2tissueAboundance.pl*************
#!/usr/bin/perl -w
```

```
use strict;
my (%Exprn,%Chlo,%Mito,$Tissue,$ID,%Table);

sub Read{
	open(IN,"<$ARGV[0]") or die "Can't open $ARGV[0]!\n";
	while(<IN>){
		if($_=~/^(\S+)\s+.+?Normal\:\s+(\S+)\s+(\S+)\s+(\S+)\s+(\S+)\s+(\S+)\s+(\S+)/){
			if($2>$ARGV[1]){$Exprn{A}{$1}=$2;}
			if($3>$ARGV[1]){$Exprn{B}{$1}=$3;}
			if($4>$ARGV[1]){$Exprn{C}{$1}=$4;}
			if($5>$ARGV[1]){$Exprn{D}{$1}=$5;}
			if($6>$ARGV[1]){$Exprn{E}{$1}=$6;}
			if($7>$ARGV[1]){$Exprn{F}{$1}=$7;}
		}
	}
	close(IN);
	my $GeneN;
	$GeneN=keys %{$Exprn{A}}; print "GeneN of A=$GeneN\n";
	$GeneN=keys %{$Exprn{B}}; print "GeneN of B=$GeneN\n";
	$GeneN=keys %{$Exprn{C}}; print "GeneN of C=$GeneN\n";
	$GeneN=keys %{$Exprn{D}}; print "GeneN of D=$GeneN\n";
	$GeneN=keys %{$Exprn{E}}; print "GeneN of E=$GeneN\n";
	$GeneN=keys %{$Exprn{F}}; print "GeneN of F=$GeneN\n";

	my (%List,$File);
	open(IN,"<$ARGV[2]") or die "Can't open $ARGV[2]!\n";
	while(<IN>){$_=~/^(\S+)/; $List{$1}++;}
	close(IN);

	for $File (keys %List){
		$File=~/^(\w)_/; $Tissue=$1;
		open(IN,"<$File") or die "Can't open $File\n";
		while(<IN>){$_=~/^(\S+)/; $Chlo{$Tissue}{$1}++;}
		close(IN);
	}

	%List=();
	open(IN,"<$ARGV[3]") or die "Can't open $ARGV[3]!\n";
	while(<IN>){$_=~/^(\S+)/; $List{$1}++;}
	close(IN);

	for $File (keys %List){
		$File=~/^(\w)_/; $Tissue=$1;
		open(IN,"<$File") or die "Can't open $File\n";
```

```
		while(<IN>){$_=~/^(\S+)/; $Mito{$Tissue}{$1}++;}
		close(IN);
	}
}

sub Print{
	my ($Sum,$GeneN,$Mean);
	for $Tissue (keys %Chlo){
		$Sum=$GeneN=0;
		for $ID (keys %{$Chlo{$Tissue}}){
			$GeneN++; $Sum+=$Exprn{$Tissue}{$ID};
		}
		$Mean=$Sum/$GeneN;
		$Table{"Chlo"}{$Tissue}="$GeneN $Mean";
	}

	for $Tissue (keys %Mito){
		$Sum=$GeneN=0;
		for $ID (keys %{$Mito{$Tissue}}){
			$GeneN++; $Sum+=$Exprn{$Tissue}{$ID};
		}
		$Mean=$Sum/$GeneN;
		$Table{"Mito"}{$Tissue}="$GeneN $Mean";
	}

	for $Tissue (keys %Exprn){
		$Sum=$GeneN=0;
		for $ID (keys %{$Exprn{$Tissue}}){
			if(!defined $Chlo{$Tissue}{$ID}&&!defined $Mito{$Tissue}{$ID}){
				$GeneN++; $Sum+=$Exprn{$Tissue}{$ID};
			}
		}
		$Mean=$Sum/$GeneN;
		$Table{"Nucleus"}{$Tissue}="$GeneN $Mean";
	}

	open(OUT,">$ARGV[4]") or die "Can't open $ARGV[4]\n";
	for $ID (sort keys %Table){
		printf OUT "%-8s",$ID;
		for $Tissue (sort keys %{$Table{$ID}}){
			$Table{$ID}{$Tissue}=~/^(\S+)\s+(\S+)/;
			printf OUT "%10d(%-10.2f)",$1,$2;
		}
		print OUT "\n";
```

```
	}
	close(OUT);
}

#main
@ARGV or die "Usage:$0 gsExprn CoverageTH final2chloro.list final2mito.list result\n";
Read();
Print();


******************SelectExprn.pl*****************
#!/usr/bin/perl -w
use strict;
my (%List,$Num);

sub Read{
	open(IN,"<$ARGV[0]") or die "Can't open $ARGV[0]!\n";
	while(<IN>){if($_=~/^(\S+)/){$List{$1}++;}}
	close(IN);
	$Num=keys %List;
	print "List Num=$Num\n";
}

sub Print{
	open(IN,"<$ARGV[1]") or die "Can't open $ARGV[1]!\n";
	open(OUT,">$ARGV[2]") or die "Can't open $ARGV[2]\n";
	$Num=0;
	while(<IN>){
		if($_=~/^(\w+_\d+)/&&defined $List{$1}){print OUT $_; $Num++;}
	}
	close(OUT);
	print "Annotated Num=$Num\n";
}

#main
@ARGV or die "Usage:$0 list expression Result\n";
Read();
Print();


******************final2Pie.pl****************
#!/usr/bin/perl -w
use strict;
my (%Exprn,$ID,%F2T,$Tissue,$Total,$GeneN); #Final2Tissue
```

```
sub Read{
	open(IN,"<$ARGV[0]") or die "Can't open $ARGV[0]!\n";
	while(<IN>){if($_=~/^(\S+)\s+.+?Normal\:\s+(\S.+?\S)\s+RPKM/){$Exprn{$1}=$2;}}
	close(IN);
	$Total=keys %Exprn;
	print "Total=$Total\n";
}

sub Print{
	for $ID (sort keys %Exprn){
		$Exprn{$ID}=~/^(\S+)\s+(\S+)\s+(\S+)\s+(\S+)\s+(\S+)\s+(\S+)/;

	if($1>=$ARGV[1]&&$2>=$ARGV[1]&&$3>=$ARGV[1]&&$4>=$ARGV[1]&&$5>=$ARGV[1]&&
$6>=$ARGV[1]){$F2T{"ABCDEF"}{$ID}++;}

	elsif($1>=$ARGV[1]&&$2>=$ARGV[1]&&$3>=$ARGV[1]&&$4>=$ARGV[1]&&$5>=$ARGV[1])
{$F2T{"ABCDE"}{$ID}++;} #F=0

	elsif($1>=$ARGV[1]&&$2>=$ARGV[1]&&$3>=$ARGV[1]&&$4>=$ARGV[1]&&$6>=$ARGV[1])
{$F2T{"ABCDF"}{$ID}++;} #E=0

	elsif($1>=$ARGV[1]&&$2>=$ARGV[1]&&$3>=$ARGV[1]&&$5>=$ARGV[1]&&$6>=$ARGV[1])
{$F2T{"ABCEF"}{$ID}++;} #D=0

	elsif($1>=$ARGV[1]&&$2>=$ARGV[1]&&$4>=$ARGV[1]&&$5>=$ARGV[1]&&$6>=$ARGV[1])
{$F2T{"ABDEF"}{$ID}++;} #C=0

	elsif($1>=$ARGV[1]&&$3>=$ARGV[1]&&$4>=$ARGV[1]&&$5>=$ARGV[1]&&$6>=$ARGV[1])
{$F2T{"ACDEF"}{$ID}++;} #B=0

	elsif($2>=$ARGV[1]&&$3>=$ARGV[1]&&$4>=$ARGV[1]&&$5>=$ARGV[1]&&$6>=$ARGV[1])
{$F2T{"BCDEF"}{$ID}++;} #A=0

	elsif($1>=$ARGV[1]&&$2>=$ARGV[1]&&$3>=$ARGV[1]&&$4>=$ARGV[1]){$F2T{"ABCD"}{$I
D}++;} #F=E=0

	elsif($1>=$ARGV[1]&&$2>=$ARGV[1]&&$3>=$ARGV[1]&&$5>=$ARGV[1]){$F2T{"ABCE"}{$I
D}++;} #F=D=0

	elsif($1>=$ARGV[1]&&$2>=$ARGV[1]&&$4>=$ARGV[1]&&$5>=$ARGV[1]){$F2T{"ABDE"}{$I
D}++;} #F=C=0

	elsif($1>=$ARGV[1]&&$3>=$ARGV[1]&&$4>=$ARGV[1]&&$5>=$ARGV[1]){$F2T{"ACDE"}{$I
```

```
D}++;} #F=B=0
        elsif($2>=$ARGV[1]&&$3>=$ARGV[1]&&$4>=$ARGV[1]&&$5>=$ARGV[1]){$F2T{"BCDE"}{$I
D}++;} #F=A=0
        elsif($1>=$ARGV[1]&&$2>=$ARGV[1]&&$3>=$ARGV[1]&&$6>=$ARGV[1]){$F2T{"ABCF"}{$I
D}++;} #E=D=0
        elsif($1>=$ARGV[1]&&$2>=$ARGV[1]&&$4>=$ARGV[1]&&$6>=$ARGV[1]){$F2T{"ABDF"}{$I
D}++;} #E=C=0
        elsif($1>=$ARGV[1]&&$3>=$ARGV[1]&&$4>=$ARGV[1]&&$6>=$ARGV[1]){$F2T{"ACDF"}{$I
D}++;} #E=B=0
        elsif($2>=$ARGV[1]&&$3>=$ARGV[1]&&$4>=$ARGV[1]&&$6>=$ARGV[1]){$F2T{"BCDF"}{$I
D}++;} #E=A=0
        elsif($1>=$ARGV[1]&&$2>=$ARGV[1]&&$5>=$ARGV[1]&&$6>=$ARGV[1]){$F2T{"ABEF"}{$I
D}++;} #D=C=0
        elsif($1>=$ARGV[1]&&$3>=$ARGV[1]&&$5>=$ARGV[1]&&$6>=$ARGV[1]){$F2T{"ACEF"}{$I
D}++;} #D=B=0
        elsif($2>=$ARGV[1]&&$3>=$ARGV[1]&&$5>=$ARGV[1]&&$6>=$ARGV[1]){$F2T{"BCEF"}{$ID
}++;} #D=A=0
        elsif($1>=$ARGV[1]&&$4>=$ARGV[1]&&$5>=$ARGV[1]&&$6>=$ARGV[1]){$F2T{"ADEF"}{$I
D}++;} #C=B=0
        elsif($2>=$ARGV[1]&&$4>=$ARGV[1]&&$5>=$ARGV[1]&&$6>=$ARGV[1]){$F2T{"BDEF"}{$I
D}++;} #C=A=0
        elsif($3>=$ARGV[1]&&$4>=$ARGV[1]&&$5>=$ARGV[1]&&$6>=$ARGV[1]){$F2T{"CDEF"}{$I
D}++;} #B=A=0
                elsif($1>=$ARGV[1]&&$2>=$ARGV[1]&&$3>=$ARGV[1]){$F2T{"ABC"}{$ID}++;}
#F=E=D=0
                elsif($1>=$ARGV[1]&&$2>=$ARGV[1]&&$4>=$ARGV[1]){$F2T{"ABD"}{$ID}++;}
#F=E=C=0
                elsif($1>=$ARGV[1]&&$3>=$ARGV[1]&&$4>=$ARGV[1]){$F2T{"ACD"}{$ID}++;}
#F=E=B=0
                elsif($2>=$ARGV[1]&&$3>=$ARGV[1]&&$4>=$ARGV[1]){$F2T{"BCD"}{$ID}++;}
#F=E=A=0
                elsif($1>=$ARGV[1]&&$2>=$ARGV[1]&&$5>=$ARGV[1]){$F2T{"ABE"}{$ID}++;}
#F=D=C=0
```

```
		elsif($1>=$ARGV[1]&&$3>=$ARGV[1]&&$5>=$ARGV[1]){$F2T{"ACE"}{$ID}++;}
#F=D=B=0
		elsif($2>=$ARGV[1]&&$3>=$ARGV[1]&&$5>=$ARGV[1]){$F2T{"BCE"}{$ID}++;}
#F=D=A=0
		elsif($1>=$ARGV[1]&&$4>=$ARGV[1]&&$5>=$ARGV[1]){$F2T{"ADE"}{$ID}++;}
#F=C=B=0
		elsif($2>=$ARGV[1]&&$4>=$ARGV[1]&&$5>=$ARGV[1]){$F2T{"BDE"}{$ID}++;}
#F=C=A=0
		elsif($3>=$ARGV[1]&&$4>=$ARGV[1]&&$5>=$ARGV[1]){$F2T{"CDE"}{$ID}++;}
#F=B=A=0
		elsif($1>=$ARGV[1]&&$2>=$ARGV[1]&&$6>=$ARGV[1]){$F2T{"ABF"}{$ID}++;}
#E=D=C=0
		elsif($1>=$ARGV[1]&&$3>=$ARGV[1]&&$6>=$ARGV[1]){$F2T{"ACF"}{$ID}++;}
#E=D=B=0
		elsif($2>=$ARGV[1]&&$3>=$ARGV[1]&&$6>=$ARGV[1]){$F2T{"BCF"}{$ID}++;}
#E=D=A=0
		elsif($1>=$ARGV[1]&&$4>=$ARGV[1]&&$6>=$ARGV[1]){$F2T{"ADF"}{$ID}++;}
#E=C=B=0
		elsif($2>=$ARGV[1]&&$4>=$ARGV[1]&&$6>=$ARGV[1]){$F2T{"BDF"}{$ID}++;}
#E=C=A=0
		elsif($3>=$ARGV[1]&&$4>=$ARGV[1]&&$6>=$ARGV[1]){$F2T{"CDF"}{$ID}++;}
#E=B=A=0
		elsif($1>=$ARGV[1]&&$5>=$ARGV[1]&&$6>=$ARGV[1]){$F2T{"AEF"}{$ID}++;}
#D=C=B=0
		elsif($2>=$ARGV[1]&&$5>=$ARGV[1]&&$6>=$ARGV[1]){$F2T{"BEF"}{$ID}++;}
#D=C=A=0
		elsif($4>=$ARGV[1]&&$5>=$ARGV[1]&&$6>=$ARGV[1]){$F2T{"DEF"}{$ID}++;}
#C=B=A=0
		elsif($1>=$ARGV[1]&&$2>=$ARGV[1]){$F2T{"AB"}{$ID}++;} #F=E=D=C=0
		elsif($1>=$ARGV[1]&&$3>=$ARGV[1]){$F2T{"AC"}{$ID}++;} #F=E=D=B=0
		elsif($1>=$ARGV[1]&&$4>=$ARGV[1]){$F2T{"AD"}{$ID}++;} #F=E=C=B=0
		elsif($1>=$ARGV[1]&&$5>=$ARGV[1]){$F2T{"AE"}{$ID}++;}
		elsif($1>=$ARGV[1]&&$6>=$ARGV[1]){$F2T{"AF"}{$ID}++;}
		elsif($2>=$ARGV[1]&&$3>=$ARGV[1]){$F2T{"BC"}{$ID}++;}
		elsif($2>=$ARGV[1]&&$4>=$ARGV[1]){$F2T{"BD"}{$ID}++;}
		elsif($2>=$ARGV[1]&&$5>=$ARGV[1]){$F2T{"BE"}{$ID}++;}
		elsif($2>=$ARGV[1]&&$6>=$ARGV[1]){$F2T{"BF"}{$ID}++;}
		elsif($3>=$ARGV[1]&&$4>=$ARGV[1]){$F2T{"CD"}{$ID}++;}
		elsif($3>=$ARGV[1]&&$5>=$ARGV[1]){$F2T{"CE"}{$ID}++;}
		elsif($3>=$ARGV[1]&&$6>=$ARGV[1]){$F2T{"CF"}{$ID}++;}
		elsif($4>=$ARGV[1]&&$5>=$ARGV[1]){$F2T{"DE"}{$ID}++;}
		elsif($4>=$ARGV[1]&&$6>=$ARGV[1]){$F2T{"DF"}{$ID}++;}
		elsif($5>=$ARGV[1]&&$6>=$ARGV[1]){$F2T{"EF"}{$ID}++;}
		elsif($1>=$ARGV[1]){$F2T{"A"}{$ID}++;}
```

```
        elsif($2>=$ARGV[1]){$F2T{"B"}{$ID}++;}
        elsif($3>=$ARGV[1]){$F2T{"C"}{$ID}++;}
        elsif($4>=$ARGV[1]){$F2T{"D"}{$ID}++;}
        elsif($5>=$ARGV[1]){$F2T{"E"}{$ID}++;}
        elsif($6>=$ARGV[1]){$F2T{"F"}{$ID}++;}
    }

    open(OUT,">$ARGV[2]") or die "Can't open $ARGV[2]\n";
    for $Tissue (sort keys %F2T){
        $GeneN=keys %{$F2T{$Tissue}};
        my $R=$GeneN/$Total;
        printf OUT ">$Tissue($GeneN)\t%5.3f\n",$R;
        for $ID (sort keys %{$F2T{$Tissue}}){print OUT "\t$ID\n";}
    }
    close(OUT);
}

#main
@ARGV or die "Usage:$0 gsExprn MinCoverageTH Summary\n";
Read();
Print();
```
