# Supplementary material for: Dissecting organ-specific transcriptomes through RNA-sequencing
Source: Plant Methods. 2013 Oct 25;9:42. doi: 10.1186/1746-4811-9-42 (PMC3819660; doi:10.1186/1746-4811-9-42)
Supplement: Additional file 9 — Additional primers for sequence verifications. [file 1746-4811-9-42-S9.pdf]

Additional file 9 Additional primers used to verify some of the sequences obtained from RNA-sequencing.

Gene name	Genbank ID	Primer sequence (forward primer, 5'→3')	Primer sequence (reverse primer, 5'→3')
4CL	KC794953	aaactccctgatatttacatccc	acaacaaaagccacnggnac
PAL	KC794954	atggagggcgccattgaaaac	ttaacagataggaagaggagcacc
C4H	KC794955	tcgtagccataatagtcgc	actgaattgccctccttt
3GGT	KC794956	gcagaaagctagctagcttg	gctaaaaccattctagctcagg
