# Supplementary material for: Dissecting organ-specific transcriptomes through RNA-sequencing
Source: Plant Methods. 2013 Oct 25;9:42. doi: 10.1186/1746-4811-9-42 (PMC3819660; doi:10.1186/1746-4811-9-42)
Supplement: Additional file 5 — KEGG mapping and expression results. [file 1746-4811-9-42-S5.pdf]

Additional file 5. Mapping results of scaffolds of the final assembly to KEGG

Format note
> KEGG # pathway
EC number (protein name)
Scaffold ID; referenceID; blast e-value; normalized reads corresponding to samples A->F; blast query sequence length; reference protein length; blast results

>00010 Glycolysis / Gluconeogenesis

1.1.1.1(alcohol dehydrogenase;)																					
ABCDEF_31928	UniRef50_B9RHN6	0.0	Normal: 721	714	784	1329	1570	1819	BlastAnno: 1711	UniRef50_B9RHN6	437	77.55	392	87	1	360	1535	46	436	0.0	610
ABCDEF_10335	UniRef50_Q8LEB2	0.0	Normal: 285	158	514	810	352	183	BlastAnno: 1547	UniRef50_Q8LEB2	381	74.93	379	92	1	1317	181	5	380	0.0	598
1.1.1.2(alcohol dehydrogenase (NADP+);)																					
ABCDEF_27285	UniRef50_O57380	5e-94	Normal: 119	81	124	95	252	18	BlastAnno: 1489	UniRef50_O57380	373	46.11	373	197	4	1279	161	3	371	5e-94	298
1.1.1.27(L-lactate dehydrogenase;)																					
ABCDEF_38409	UniRef50_P22988	5e-157	Normal: 468	369	408	758	1349	382	BlastAnno: 1380	UniRef50_P22988	356	66.67	357	111	3	121	1170	1	356	5e-157	458
1.1.2.7(methanol dehydrogenase (cytochrome c);)																					
No Correspond Unigene																					
1.1.2.8(alcohol dehydrogenase (cytochrome c);)																					
No Correspond Unigene																					
1.2.1.12(glyceraldehyde-3-phosphate dehydrogenase (phosphorylating);)																					
ABCDEF_18261	UniRef50_P04406	2e-161	Normal: 2109	632	2645	7938	6844	656	BlastAnno: 1748	UniRef50_P04406	335	71.08	332	95	1	493	1488	2	332	2e-161	473
1.2.1.3(aldehyde dehydrogenase (NAD+);)																					
ABCDEF_52483	UniRef50_Q70E96	0.0	Normal: 28	851	41	0	3965	420	BlastAnno: 1750	UniRef50_Q70E96	484	54.56	482	219	0	107	1552	3	484	0.0	548
ABCDEF_17638	UniRef50_Q70E96	0.0	Normal: 580	718	749	2424	1317	775	BlastAnno: 1970	UniRef50_Q70E96	484	62.21	471	177	1	283	1692	2	472	0.0	625
ABCDEF_403	UniRef50_Q8W033	0.0	Normal: 69	355	98	39	223	1111	BlastAnno: 1749	UniRef50_Q8W033	550	65.96	473	161	0	1523	105	74	546	0.0	625
ABCDEF_45143	UniRef50_Q0WSF1	0.0	Normal: 1445	874	1200	1789	1940	1504	BlastAnno: 2450	UniRef50_Q0WSF1	596	79.97	594	115	2	2220	451	1	594	0.0	1015
1.2.1.5(aldehyde dehydrogenase [NAD(P)+];)																					
ABCDEF_81997	UniRef50_P43353	5e-53	Normal: 32	81	25	16	59	314	BlastAnno: 770	UniRef50_P43353	468	45.37	205	106	3	746	138	261	461	5e-53	186
1.2.1.59(glyceraldehyde-3-phosphate dehydrogenase (NAD(P)+) (phosphorylating);)																					
No Correspond Unigene																					
1.2.1.9(glyceraldehyde-3-phosphate dehydrogenase (NADP+);)																					
ABCDEF_14573	UniRef50_Q1WIQ6	0.0	Normal: 2547	9518	1266	309	441	22896	BlastAnno: 1872	UniRef50_Q1WIQ6	496	88.71	496	56	0	138	1625	1	496	0.0	899
1.2.4.1(pyruvate dehydrogenase (acetyl-transferring);)																					
ABCDEF_28990	UniRef50_Q8H1Y0	0.0	Normal: 569	351	802	1550	2572	992	BlastAnno: 1596	UniRef50_Q8H1Y0	393	80.62	356	69	0	1369	302	38	393	0.0	628
ABCDEF_11397	UniRef50_O24458	0.0	Normal: 1628	1528	1619	3502	3214	2274	BlastAnno: 1766	UniRef50_O24458	406	82.74	394	58	4	1349	180	19	406	0.0	610
ABCDEF_35811	UniRef50_Q8H1Y0	0.0	Normal: 1865	1480	2772	5588	6415	2489	BlastAnno: 1647	UniRef50_Q8H1Y0	393	77.66	394	83	3	138	1307	1	393	0.0	621
ABCDEF_15406	UniRef50_O24458	0.0	Normal: 297	358	305	389	271	1259	BlastAnno: 1486	UniRef50_O24458	406	80.73	410	72	4	19	1239	1	406	0.0	647
ABCDEF_49154	UniRef50_O24458	0.0	Normal: 304	369	317	391	293	1358	BlastAnno: 1412	UniRef50_O24458	406	80.73	410	72	4	1394	174	1	406	0.0	647
ABCDEF_63617	UniRef50_O24458	0.0	Normal: 518	490	543	916	834	1436	BlastAnno: 1398	UniRef50_O24458	406	80.68	409	72	4	1397	180	2	406	0.0	618
1.2.7.1(pyruvate synthase;)																					
No Correspond Unigene																					
1.2.7.5(aldehyde ferredoxin oxidoreductase;)																					
No Correspond Unigene																					
1.2.7.6(glyceraldehyde-3-phosphate dehydrogenase (ferredoxin);)																					
No Correspond Unigene																					
1.8.1.4(dihydrolipoyl dehydrogenase;)																					
ABCDEF_573	UniRef50_P72740	0.0	Normal: 1422	809	1248	2548	1822	4200	BlastAnno: 2189	UniRef50_P72740	474	61.95	473	173	5	469	1878	5	473	0.0	556
2.3.1.12(dihydrolipoyllysine-residue acetyltransferase;)																					
ABCDEF_52034	UniRef50_Q0WQF7	0.0	Normal: 759	582	1123	2258	2501	768	BlastAnno: 2694	UniRef50_Q0WQF7	637	64.17	642	208	9	254	2146	1	631	0.0	743
ABCDEF_14485	UniRef50_Q5M729	0.0	Normal: 1032	504	1377	2545	3005	1098	BlastAnno: 2220	UniRef50_Q5M729	539	67.09	556	159	8	2092	446	1	539	0.0	719
ABCDEF_7230	UniRef50_Q5M729	0.0	Normal: 1333	844	1864	3619	4766	1386	BlastAnno: 2252	UniRef50_Q5M729	539	65.23	558	169	7	2120	465	1	539	0.0	702
2.7.1.1(hexokinase;)																					
ABCDEF_27834	UniRef50_P93834	0.0	Normal: 998	404	1410	4185	6485	623	BlastAnno: 2374	UniRef50_P93834	502	72.77	470	128	0	2024	615	25	494	0.0	655
ABCDEF_51305	UniRef50_P93834	0.0	Normal: 443	123	280	151	521	245	BlastAnno: 2179	UniRef50_P93834	502	76.47	289	68	0	1813	947	25	313	0.0	467
ABCDEF_420	UniRef50_Q1WM16	0.0	Normal: 453	101	514	402	696	75	BlastAnno: 1554	UniRef50_Q1WM16	463	61.66	433	159	3	3	1295	20	447	0.0	538
ABCDEF_86444	UniRef50_Q1WM16	0.0	Normal: 293	76	186	81	353	207	BlastAnno: 1684	UniRef50_Q1WM16	463	66.83	404	129	3	1404	196	63	462	0.0	530
ABCDEF_592	UniRef50_P93834	0.0	Normal: 378	177	486	1038	1819	341	BlastAnno: 1859	UniRef50_P93834	502	59.87	456	180	3	1618	260	37	492	0.0	537
ABCDEF_83497	UniRef50_Q1WM16	0.0	Normal: 290	84	185	81	350	338	BlastAnno: 1860	UniRef50_Q1WM16	463	66.83	404	129	3	1404	196	63	462	0.0	529
ABCDEF_51306	UniRef50_Q1WM16	0.0	Normal: 446	118	281	150	539	247	BlastAnno: 2108	UniRef50_Q1WM16	463	66.96	451	144	3	1685	336	16	462	0.0	578
ABCDEF_593	UniRef50_P93834	0.0	Normal: 403	191	504	1097	1940	353	BlastAnno: 1902	UniRef50_P93834	502	59.87	456	180	3	1648	290	37	492	0.0	537
2.7.1.11(6-phosphofructokinase;)																					
ABCDEF_48463	UniRef50_Q94AA4	0.0	Normal: 273	212	275	545	518	593	BlastAnno: 1885	UniRef50_Q94AA4	489	71.85	437	122	1	403	1713	17	452	0.0	681
ABCDEF_48462	UniRef50_Q9FKG3	0.0	Normal: 197	165	201	395	395	441	BlastAnno: 1773	UniRef50_Q9FKG3	530	76.95	295	61	2	316	1197	44	332	0.0	469
ABCDEF_29376	UniRef50_Q94AA4	0.0	Normal: 0	44	0	0	20	222	BlastAnno: 1555	UniRef50_Q94AA4	489	81.00	458	82	3	99	1472	8	460	0.0	756
ABCDEF_49371	UniRef50_Q94AA4	0.0	Normal: 16	353	32	5	82	64	BlastAnno: 1513	UniRef50_Q94AA4	489	77.23	470	98	3	30	1421	10	476	0.0	752
ABCDEF_48301	UniRef50_Q8VYN6	0.0	Normal: 298	135	430	474	1016	200	BlastAnno: 2035	UniRef50_Q8VYN6	537	78.25	538	112	3	128	1729	1	537	0.0	844
ABCDEF_41341	UniRef50_Q94AA4	0.0	Normal: 889	655	932	1360	2142	1363	BlastAnno: 2130	UniRef50_Q94AA4	489	80.89	450	85	1	1832	483	6	454	0.0	755
2.7.1.146(ADP-specific phosphofructokinase;)																					
No Correspond Unigene																					
2.7.1.147(ADP-specific glucokinase;)																					

No Correspond Unigene																					
2.7.1.2(glucokinase;)																					
No Correspond Unigene																					
2.7.1.40(pyruvate kinase;)																					
ABCDEF_27872	UniRef50_Q40545	0.0	Normal: 1220	1217	1378	1640	1974	4469	BlastAnno: 2233	UniRef50_Q40545	593	81.65	594	96	4	1951	206	1	593	0.0	979
ABCDEF_34901	UniRef50_B9EYF5	0.0	Normal: 3919	1884	3179	3852	4333	5138	BlastAnno: 2384	UniRef50_B9EYF5	606	80.36	555	84	2	2021	432	52	606	0.0	922
ABCDEF_10813	UniRef50_Q2QXR7	0.0	Normal: 2566	2379	2864	4497	6222	4202	BlastAnno: 2019	UniRef50_Q2QXR7	408	85.57	402	58	0	174	1379	1	402	0.0	727
ABCDEF_35590	UniRef50_Q9M057	0.0	Normal: 2212	2122	2749	5601	7830	2637	BlastAnno: 1980	UniRef50_Q9M057	510	69.58	503	148	4	1830	334	1	502	0.0	687
ABCDEF_27885	UniRef50_Q2QXR7	0.0	Normal: 924	752	1120	2229	3166	928	BlastAnno: 2102	UniRef50_Q2QXR7	408	87.31	402	51	0	182	1387	1	402	0.0	729
ABCDEF_21818	UniRef50_Q40545	0.0	Normal: 1982	1309	1439	2275	1999	2303	BlastAnno: 2062	UniRef50_Q40545	593	69.25	491	147	2	1594	134	103	593	0.0	716
ABCDEF_31300	UniRef50_Q40545	0.0	Normal: 1326	1019	1016	1433	1396	1865	BlastAnno: 1805	UniRef50_Q40545	593	91.70	494	41	0	1802	321	100	593	0.0	920
ABCDEF_45529	UniRef50_B9EYF5	0.0	Normal: 1474	625	1452	3814	2571	3121	BlastAnno: 2114	UniRef50_B9EYF5	606	78.56	569	89	3	1810	179	46	606	0.0	912
ABCDEF_45536	UniRef50_Q2QXR7	0.0	Normal: 1644	1099	1596	1290	5936	335	BlastAnno: 1959	UniRef50_Q2QXR7	408	81.59	402	74	0	1862	657	1	402	0.0	687
ABCDEF_27886	UniRef50_Q2QXR7	0.0	Normal: 771	640	954	1807	2731	814	BlastAnno: 1787	UniRef50_Q2QXR7	408	86.82	349	46	0	54	1100	54	402	0.0	632
ABCDEF_18187	UniRef50_Q9M057	0.0	Normal: 3137	1498	3397	5489	6703	1765	BlastAnno: 1912	UniRef50_Q9M057	510	67.21	494	158	3	216	1685	17	510	0.0	681
ABCDEF_45028	UniRef50_B9EYF5	0.0	Normal: 273	415	192	360	372	144	BlastAnno: 1785	UniRef50_B9EYF5	606	73.94	495	105	2	372	1784	111	605	0.0	729
2.7.1.41(glucose-1-phosphate phosphodismutase)																					
No Correspond Unigene																					
2.7.1.63(polyphosphate---glucose phosphotransferase;)																					
No Correspond Unigene																					
2.7.1.69(protein-Npi-phosphohistidine---sugar phosphotransferase;)																					
No Correspond Unigene																					
2.7.2.3(phosphoglycerate kinase;)																					
ABCDEF_38934	UniRef50_B8LKQ1	0.0	Normal: 7822	4290	7908	11136	16389	8199	BlastAnno: 1790	UniRef50_B8LKQ1	472	76.90	407	58	2	261	1481	97	467	0.0	580
ABCDEF_28557	UniRef50_B8LKQ1	0.0	Normal: 2514	10526	2985	3890	5050	32250	BlastAnno: 2074	UniRef50_B8LKQ1	472	77.70	408	56	1	1523	300	98	470	0.0	630
3.1.3.10(glucose-1-phosphatase)																					
No Correspond Unigene																					
3.1.3.11(fructose-bisphosphatase;)																					
ABCDEF_28302	UniRef50_P25851	0.0	Normal: 1013	1937	653	1129	1293	5773	BlastAnno: 1619	UniRef50_P25851	417	66.49	382	125	2	1417	281	36	417	0.0	544
ABCDEF_35263	UniRef50_Q9XGG5	0.0	Normal: 72	1508	41	126	415	7941	BlastAnno: 1577	UniRef50_Q9XGG5	329	92.62	325	24	0	373	1347	1	325	0.0	627
ABCDEF_7505	UniRef50_P25851	0.0	Normal: 1194	5939	568	263	269	21425	BlastAnno: 1662	UniRef50_P25851	417	81.75	389	66	3	1369	218	29	417	0.0	613
3.1.3.13(bisphosphoglycerate phosphatase;)																					
No Correspond Unigene																					
3.1.3.9(glucose-6-phosphatase;)																					
No Correspond Unigene																					
3.2.1.86(6-phospho-beta-glucosidase;)																					
No Correspond Unigene																					
4.1.1.1(pyruvate decarboxylase;)																					
ABCDEF_48236	UniRef50_B9RLM7	0.0	Normal: 90	85	194	639	3867	102	BlastAnno: 1939	UniRef50_B9RLM7	595	73.01	541	145	1	1938	319	50	590	0.0	782
4.1.1.32(phosphoenolpyruvate carboxykinase (GTP);)																					
No Correspond Unigene																					
4.1.1.49(phosphoenolpyruvate carboxykinase (ATP);)																					
ABCDEF_2793	UniRef50_P10963	0.0	Normal: 99	228	79	1160	39	318	BlastAnno: 2459	UniRef50_P10963	549	52.91	567	219	11	402	2084	24	548	0.0	564
ABCDEF_81273	UniRef50_Q9T074	0.0	Normal: 86	204	69	1021	35	282	BlastAnno: 2611	UniRef50_Q9T074	671	76.69	369	78	5	2606	1512	8	372	0.0	565
ABCDEF_81242	UniRef50_Q9T074	0.0	Normal: 95	225	76	1100	40	293	BlastAnno: 2538	UniRef50_Q9T074	671	75.83	480	102	7	2533	1124	8	483	0.0	693
ABCDEF_2794	UniRef50_Q9T074	0.0	Normal: 114	289	99	1477	44	337	BlastAnno: 2733	UniRef50_Q9T074	671	76.69	369	78	5	2614	1520	8	372	0.0	565
ABCDEF_2796	UniRef50_Q9T074	0.0	Normal: 127	322	105	1687	47	387	BlastAnno: 2614	UniRef50_Q9T074	671	90.23	399	39	0	1572	376	273	671	0.0	738
ABCDEF_85588	UniRef50_Q9T074	0.0	Normal: 41	99	29	443	15	186	BlastAnno: 2024	UniRef50_Q9T074	671	80.55	329	35	1	1362	376	372	671	0.0	550
ABCDEF_79512	UniRef50_Q9T074	0.0	Normal: 104	244	82	1189	43	318	BlastAnno: 2451	UniRef50_Q9T074	671	75.83	480	102	7	2446	1037	8	483	0.0	693
ABCDEF_2797	UniRef50_P10963	0.0	Normal: 136	339	120	1738	54	398	BlastAnno: 2486	UniRef50_P10963	549	55.58	538	220	10	1971	376	24	548	0.0	576
ABCDEF_89373	UniRef50_Q9T074	0.0	Normal: 55	130	40	611	21	222	BlastAnno: 1864	UniRef50_Q9T074	671	84.11	428	39	1	1659	376	273	671	0.0	721
ABCDEF_2795	UniRef50_Q9T074	0.0	Normal: 49	120	40	536	20	212	BlastAnno: 1946	UniRef50_Q9T074	671	88.33	300	35	0	1275	376	372	671	0.0	565
ABCDEF_2792	UniRef50_Q9T074	0.0	Normal: 88	218	76	1085	36	308	BlastAnno: 2532	UniRef50_Q9T074	671	88.33	300	35	0	1258	2157	372	671	0.0	565
4.1.2.13(fructose-bisphosphate aldolase;)																					
ABCDEF_812	UniRef50_Q40677	0.0	Normal: 2391	1243	3255	6494	7322	1031	BlastAnno: 1720	UniRef50_Q40677	388	81.39	360	67	0	362	1441	29	388	0.0	612
ABCDEF_4038	UniRef50_Q40677	0.0	Normal: 2914	23962	1997	525	623	51538	BlastAnno: 1570	UniRef50_Q40677	388	82.87	397	53	3	123	1301	3	388	0.0	641
ABCDEF_35511	UniRef50_B9FU33	0.0	Normal: 18	1991	3	12	56	2290	BlastAnno: 1432	UniRef50_B9FU33	559	71.11	398	101	3	1423	245	171	559	0.0	536
ABCDEF_14742	UniRef50_Q40677	0.0	Normal: 4220	14155	2670	281	395	26738	BlastAnno: 1587	UniRef50_Q40677	388	89.94	348	35	0	1291	248	41	388	0.0	657
4.2.1.11(phosphopyruvate hydratase;)																					
ABCDEF_204	UniRef50_A5B6U7	0.0	Normal: 577	318	663	998	1166	662	BlastAnno: 1992	UniRef50_A5B6U7	527	68.14	452	89	3	196	1551	1	397	0.0	613
ABCDEF_51801	UniRef50_Q6BI20	0.0	Normal: 883	270	1626	3195	3612	300	BlastAnno: 1822	UniRef50_Q6BI20	439	64.27	431	143	6	1472	201	9	435	0.0	537
ABCDEF_4784	UniRef50_P06733	0.0	Normal: 8739	4441	8587	11774	22445	7368	BlastAnno: 1781	UniRef50_P06733	434	70.81	442	115	8	1588	272	2	432	0.0	618
ABCDEF_48464	UniRef50_P06733	0.0	Normal: 3032	1633	3013	4940	8155	2527	BlastAnno: 1829	UniRef50_P06733	434	71.27	442	113	8	242	1558	2	432	0.0	621
5.1.3.15(glucose-6-phosphate 1-epimerase)																					
ABCDEF_45578	UniRef50_Q40784	2e-175	Normal: 1700	1490	1815	3984	4819	2683	BlastAnno: 1495	UniRef50_Q40784	329	78.57	308	64	1	172	1089	22	329	2e-175	505
5.1.3.3(aldose 1-epimerase;)																					
ABCDEF_6488	UniRef50_A9EUS1	5e-103	Normal: 169	166	191	162	198	287	BlastAnno: 1643	UniRef50_A9EUS1	415	48.67	339	164	7	1221	223	81	415	5e-103	325
5.3.1.1(triose-phosphate isomerase;)																					
ABCDEF_25548	UniRef50_Q9SKP6	8e-167	Normal: 879	1226	1007	1772	2081	5167	BlastAnno: 1419	UniRef50_Q9SKP6	315	75.95	316	70	3	1221	289	1	315	8e-167	482
5.3.1.9(glucose-6-phosphate isomerase;)																					
ABCDEF_21891	UniRef50_B5EBB8	0.0	Normal: 2382	2326	3109	5032	5330	3316	BlastAnno: 2692	UniRef50_B5EBB8	530	60.59	510	196	2	2176	647	4	508	0.0	624
ABCDEF_4218	UniRef50_B7GDV0	0.0	Normal: 982	971	1324	2230	3149	3125	BlastAnno: 2219	UniRef50_B7GDV0	786	56.56	564	238	4	2045	375	188	751	0.0	639

5.4.2.1(phosphoglycerate mutase;)																					
ABCDEF_45789	UniRef50_O04499	0.0	Normal: 6087	3713	6051	11349	14624	5679	BlastAnno: 2153	UniRef50_O04499	557	83.81	556	90	0	1962	295	2	557	0.0	942
5.4.2.2(phosphoglucomutase;)																					
ABCDEF_935	UniRef50_P47244	0.0	Normal: 5430	4330	5743	12110	19002	6223	BlastAnno: 2144	UniRef50_P47244	572	53.66	587	246	11	2064	328	4	572	0.0	585
ABCDEF_24641	UniRef50_P47244	0.0	Normal: 416	659	414	959	874	2109	BlastAnno: 2341	UniRef50_P47244	572	54.17	576	222	11	386	2035	13	572	0.0	588
ABCDEF_24640	UniRef50_P47244	0.0	Normal: 389	596	384	834	777	1859	BlastAnno: 2422	UniRef50_P47244	572	51.74	603	222	12	386	2116	13	572	0.0	574
5.4.2.4(bisphosphoglycerate mutase;)																					
No Correspond Unigene																					
6.2.1.1(acetate---CoA ligase;)																					
ABCDEF_65173	UniRef50_Q9VP61	0.0	Normal: 208	202	219	336	383	409	BlastAnno: 2249	UniRef50_Q9VP61	670	54.63	540	236	3	539	2131	4	543	0.0	629
ABCDEF_51889	UniRef50_Q9VP61	0.0	Normal: 268	268	315	436	574	651	BlastAnno: 2148	UniRef50_Q9VP61	670	55.64	638	273	4	2060	174	26	662	0.0	723
ABCDEF_51888	UniRef50_A8JFR9	0.0	Normal: 348	343	408	583	709	775	BlastAnno: 2704	UniRef50_A8JFR9	667	56.57	647	273	5	2090	168	20	664	0.0	712
ABCDEF_59631	UniRef50_Q9VP61	0.0	Normal: 132	127	125	187	249	287	BlastAnno: 1733	UniRef50_Q9VP61	670	55.41	518	222	3	89	1615	26	543	0.0	610
6.2.1.13(acetate---CoA ligase (ADP-forming);)																					
No Correspond Unigene																					
>00020 Citrate cycle (TCA cycle)																					
1.1.1.37(malate dehydrogenase;)																					
ABCDEF_25169	UniRef50_Q9SN86	0.0	Normal: 938	614	1116	1317	4446	973	BlastAnno: 1856	UniRef50_Q9SN86	403	75.50	400	88	2	1616	417	2	391	0.0	585
ABCDEF_31746	UniRef50_Q9ZP05	0.0	Normal: 611	6985	354	98	71	19818	BlastAnno: 1631	UniRef50_Q9ZP05	354	83.38	349	54	2	274	1317	9	354	0.0	578
ABCDEF_32230	UniRef50_P57106	0.0	Normal: 8408	4627	7877	10286	18779	11078	BlastAnno: 1523	UniRef50_P57106	332	93.07	332	23	0	262	1257	1	332	0.0	617
ABCDEF_57415	UniRef50_Q9ZP05	0.0	Normal: 403	336	343	761	845	611	BlastAnno: 1190	UniRef50_Q9ZP05	354	83.29	353	54	2	1058	3	1	349	0.0	579
ABCDEF_17941	UniRef50_Q9ZP05	0.0	Normal: 616	554	586	1025	1231	910	BlastAnno: 1567	UniRef50_Q9ZP05	354	83.63	342	51	2	1336	314	17	354	0.0	563
1.1.1.41(isocitrate dehydrogenase (NAD+);)																					
ABCDEF_21302	UniRef50_O81796	0.0	Normal: 183	57	179	277	419	72	BlastAnno: 1440	UniRef50_O81796	368	80.24	329	65	0	268	1254	40	368	0.0	523
ABCDEF_1070	UniRef50_O81796	0.0	Normal: 1007	782	1421	2225	4003	1338	BlastAnno: 1406	UniRef50_O81796	368	82.83	361	57	2	1293	217	1	358	0.0	563
1.1.1.42(isocitrate dehydrogenase (NADP+);)																					
ABCDEF_49419	UniRef50_O75874	0.0	Normal: 5183	5221	5740	12435	20083	7734	BlastAnno: 1704	UniRef50_O75874	414	68.12	389	123	1	1491	328	10	398	0.0	566
ABCDEF_51976	UniRef50_O75874	0.0	Normal: 551	408	828	1291	1909	1088	BlastAnno: 1799	UniRef50_O75874	414	66.17	405	135	2	321	1529	10	414	0.0	568
1.2.4.1(pyruvate dehydrogenase (acetyl-transferring);)																					
ABCDEF_28990	UniRef50_Q8H1Y0	0.0	Normal: 569	351	802	1550	2572	992	BlastAnno: 1596	UniRef50_Q8H1Y0	393	80.62	356	69	0	1369	302	38	393	0.0	628
ABCDEF_11397	UniRef50_O24458	0.0	Normal: 1628	1528	1619	3502	3214	2274	BlastAnno: 1766	UniRef50_O24458	406	82.74	394	58	4	1349	180	19	406	0.0	610
ABCDEF_35811	UniRef50_Q8H1Y0	0.0	Normal: 1865	1480	2772	5588	6415	2489	BlastAnno: 1647	UniRef50_Q8H1Y0	393	77.66	394	83	3	138	1307	1	393	0.0	621
ABCDEF_15406	UniRef50_O24458	0.0	Normal: 297	358	305	389	271	1259	BlastAnno: 1486	UniRef50_O24458	406	80.73	410	72	4	19	1239	1	406	0.0	647
ABCDEF_49154	UniRef50_O24458	0.0	Normal: 304	369	317	391	293	1358	BlastAnno: 1412	UniRef50_O24458	406	80.73	410	72	4	1394	174	1	406	0.0	647
ABCDEF_63617	UniRef50_O24458	0.0	Normal: 518	490	543	916	834	1436	BlastAnno: 1398	UniRef50_O24458	406	80.68	409	72	4	1397	180	2	406	0.0	618
1.2.4.2(oxoglutarate dehydrogenase (succinyl-transferring);)																					
ABCDEF_21623	UniRef50_O61199	0.0	Normal: 2615	1939	3031	10740	12054	2537	BlastAnno: 3717	UniRef50_O61199	1029	48.29	996	440	18	3334	491	49	1017	0.0	931
ABCDEF_30869	UniRef50_O61199	0.0	Normal: 1668	226	1504	1974	2525	280	BlastAnno: 3680	UniRef50_O61199	1029	46.61	1047	463	23	3319	326	18	1017	0.0	911
1.2.7.1(pyruvate synthase;)																					
No Correspond Unigene																					
1.2.7.3(2-oxoglutarate synthase;)																					
No Correspond Unigene																					
1.3.5.1(succinate dehydrogenase (ubiquinone);)																					
ABCDEF_47748	UniRef50_Q4N0B9	0.0	Normal: 903	791	1034	2782	3604	958	BlastAnno: 2360	UniRef50_Q4N0B9	658	59.87	628	201	7	2104	365	13	637	0.0	682
ABCDEF_47751	UniRef50_Q4N0B9	0.0	Normal: 776	655	916	2143	2907	810	BlastAnno: 2266	UniRef50_Q4N0B9	658	59.87	628	201	7	189	1928	13	637	0.0	682
ABCDEF_47750	UniRef50_Q4N0B9	0.0	Normal: 1478	889	1772	2151	3099	1241	BlastAnno: 2236	UniRef50_Q4N0B9	658	59.71	628	202	6	2012	273	13	637	0.0	684
1.3.99.1(succinate dehydrogenase;)																					
ABCDEF_47748	UniRef50_Q6F8K9	0.0	Normal: 903	791	1034	2782	3604	958	BlastAnno: 2360	UniRef50_Q6F8K9	632	51.24	603	252	8	1990	290	36	632	0.0	584
ABCDEF_47751	UniRef50_Q6F8K9	0.0	Normal: 776	655	916	2143	2907	810	BlastAnno: 2266	UniRef50_Q6F8K9	632	51.24	603	252	8	303	2003	36	632	0.0	585
ABCDEF_47750	UniRef50_Q6F8K9	0.0	Normal: 1478	889	1772	2151	3099	1241	BlastAnno: 2236	UniRef50_Q6F8K9	632	51.00	602	255	7	1898	198	36	632	0.0	571
1.8.1.4(dihydrolipoyl dehydrogenase;)																					
ABCDEF_573	UniRef50_P72740	0.0	Normal: 1422	809	1248	2548	1822	4200	BlastAnno: 2189	UniRef50_P72740	474	61.95	473	173	5	469	1878	5	473	0.0	556
2.3.1.12(dihydrolipoyllysine-residue acetyltransferase;)																					
ABCDEF_52034	UniRef50_Q0WQF7	0.0	Normal: 759	582	1123	2258	2501	768	BlastAnno: 2694	UniRef50_Q0WQF7	637	64.17	642	208	9	254	2146	1	631	0.0	743
ABCDEF_14485	UniRef50_Q5M729	0.0	Normal: 1032	504	1377	2545	3005	1098	BlastAnno: 2220	UniRef50_Q5M729	539	67.09	556	159	8	2092	446	1	539	0.0	719
ABCDEF_7230	UniRef50_Q5M729	0.0	Normal: 1333	844	1864	3619	4766	1386	BlastAnno: 2252	UniRef50_Q5M729	539	65.23	558	169	7	2120	465	1	539	0.0	702
2.3.1.61(dihydrolipoyllysine-residue succinyltransferase;)																					
ABCDEF_27826	UniRef50_Q9FLQ4	0.0	Normal: 465	236	709	1274	2770	464	BlastAnno: 1861	UniRef50_Q9FLQ4	464	67.93	474	132	4	1703	306	3	464	0.0	577
ABCDEF_7861	UniRef50_Q9FLQ4	0.0	Normal: 1628	1000	2134	3927	6737	1296	BlastAnno: 1904	UniRef50_Q9FLQ4	464	71.13	471	127	5	142	1554	3	464	0.0	579
ABCDEF_66196	UniRef50_Q9FLQ4	0.0	Normal: 343	184	521	947	1904	361	BlastAnno: 1450	UniRef50_Q9FLQ4	464	76.71	395	90	1	1312	134	70	464	0.0	566
2.3.3.1(citrate (Si)-synthase;)																					
ABCDEF_734	UniRef50_O75390	0.0	Normal: 720	410	930	1540	2753	660	BlastAnno: 1840	UniRef50_O75390	466	61.59	440	168	1	242	1558	27	466	0.0	589
ABCDEF_735	UniRef50_O75390	0.0	Normal: 781	455	1022	1706	2969	688	BlastAnno: 1784	UniRef50_O75390	466	61.59	440	168	1	1606	290	27	466	0.0	589
ABCDEF_795	UniRef50_Q9LXS6	0.0	Normal: 1322	1369	1619	3657	3459	1871	BlastAnno: 1908	UniRef50_Q9LXS6	514	84.68	483	71	1	1775	327	34	513	0.0	832
2.3.3.8(ATP citrate synthase;)																					
ABCDEF_877	UniRef50_Q2QNG7	0.0	Normal: 2303	1262	2637	3893	6172	3374	BlastAnno: 1806	UniRef50_Q2QNG7	423	84.73	419	64	0	1525	269	1	419	0.0	725
ABCDEF_38288	UniRef50_Q2QNG7	0.0	Normal: 923	1139	998	1780	3136	1375	BlastAnno: 1899	UniRef50_Q2QNG7	423	90.02	421	42	0	1697	435	1	421	0.0	793
ABCDEF_38287	UniRef50_Q2QNG7	0.0	Normal: 1012	1211	1083	1973	3362	1439	BlastAnno: 1842	UniRef50_Q2QNG7	423	90.02	421	42	0	146	1408	1	421	0.0	793
ABCDEF_42417	UniRef50_A8I8X8	0.0	Normal: 4805	3069	5468	10656	12827	8213	BlastAnno: 2396	UniRef50_A8I8X8	534	64.13	605	142	3	192	2003	3	533	0.0	771
4.1.1.32(phosphoenolpyruvate carboxykinase (GTP);)																					
No Correspond Unigene																					
4.1.1.49(phosphoenolpyruvate carboxykinase (ATP);)																					

ABCDEF_2793	UniRef50_P10963	0.0	Normal: 99	228	79	1160	39	318	BlastAnno: 2459	UniRef50_P10963	549	52.91	567	219	11	402	2084	24	548	0.0	564
ABCDEF_81273	UniRef50_Q9T074	0.0	Normal: 86	204	69	1021	35	282	BlastAnno: 2611	UniRef50_Q9T074	671	76.69	369	78	5	2606	1512	8	372	0.0	565
ABCDEF_81242	UniRef50_Q9T074	0.0	Normal: 95	225	76	1100	40	293	BlastAnno: 2538	UniRef50_Q9T074	671	75.83	480	102	7	2533	1124	8	483	0.0	693
ABCDEF_2794	UniRef50_Q9T074	0.0	Normal: 114	289	99	1477	44	337	BlastAnno: 2733	UniRef50_Q9T074	671	76.69	369	78	5	2614	1520	8	372	0.0	565
ABCDEF_2796	UniRef50_Q9T074	0.0	Normal: 127	322	105	1687	47	387	BlastAnno: 2614	UniRef50_Q9T074	671	90.23	399	39	0	1572	376	273	671	0.0	738
ABCDEF_85588	UniRef50_Q9T074	0.0	Normal: 41	99	29	443	15	186	BlastAnno: 2024	UniRef50_Q9T074	671	80.55	329	35	1	1362	376	372	671	0.0	550
ABCDEF_79512	UniRef50_Q9T074	0.0	Normal: 104	244	82	1189	43	318	BlastAnno: 2451	UniRef50_Q9T074	671	75.83	480	102	7	2446	1037	8	483	0.0	693
ABCDEF_2797	UniRef50_P10963	0.0	Normal: 136	339	120	1738	54	398	BlastAnno: 2486	UniRef50_P10963	549	55.58	538	220	10	1971	376	24	548	0.0	576
ABCDEF_89373	UniRef50_Q9T074	0.0	Normal: 55	130	40	611	21	222	BlastAnno: 1864	UniRef50_Q9T074	671	84.11	428	39	1	1659	376	273	671	0.0	721
ABCDEF_2795	UniRef50_Q9T074	0.0	Normal: 49	120	40	536	20	212	BlastAnno: 1946	UniRef50_Q9T074	671	88.33	300	35	0	1275	376	372	671	0.0	565
ABCDEF_2792	UniRef50_Q9T074	0.0	Normal: 88	218	76	1085	36	308	BlastAnno: 2532	UniRef50_Q9T074	671	88.33	300	35	0	1258	2157	372	671	0.0	565
4.1.3.6(citrate (pro-3S)-lyase;)																					
No Correspond Unigene																					
4.2.1.2(fumarate hydratase;)																					
ABCDEF_34405	UniRef50_Q92PB6	0.0	Normal: 238	139	294	638	963	297	BlastAnno: 1885	UniRef50_Q92PB6	463	65.23	463	157	3	1703	321	2	462	0.0	605
ABCDEF_34407	UniRef50_Q92PB6	0.0	Normal: 230	132	282	615	1071	309	BlastAnno: 2193	UniRef50_Q92PB6	463	65.01	463	158	3	1860	478	2	462	0.0	603
ABCDEF_34406	UniRef50_Q92PB6	0.0	Normal: 208	123	332	593	935	301	BlastAnno: 2063	UniRef50_Q92PB6	463	65.01	463	158	3	204	1586	2	462	0.0	605
ABCDEF_34404	UniRef50_Q92PB6	0.0	Normal: 305	139	336	555	956	377	BlastAnno: 1811	UniRef50_Q92PB6	463	65.01	463	158	3	226	1608	2	462	0.0	603
4.2.1.3(aconitate hydratase;)																					
ABCDEF_73976	UniRef50_Q90875	0.0	Normal: 1872	1426	2211	5893	8147	1851	BlastAnno: 3209	UniRef50_Q90875	889	61.77	892	335	3	3045	370	3	888	0.0	1114
ABCDEF_35907	UniRef50_Q90875	0.0	Normal: 1591	876	1677	4081	5832	1425	BlastAnno: 3358	UniRef50_Q90875	889	61.84	891	334	3	3181	509	3	887	0.0	1109
ABCDEF_17819	UniRef50_Q90875	0.0	Normal: 1842	942	1937	4916	6347	1527	BlastAnno: 3866	UniRef50_Q90875	889	61.84	891	334	3	3181	509	3	887	0.0	1110
ABCDEF_52401	UniRef50_Q90875	0.0	Normal: 2227	1825	2713	7829	9659	2079	BlastAnno: 3665	UniRef50_Q90875	889	61.77	892	335	3	3045	370	3	888	0.0	1114
6.2.1.4(succinate---CoA ligase (GDP-forming);)																					
ABCDEF_45671	UniRef50_Q869S7	2e-148	Normal: 1899	1293	2339	3905	6798	2137	BlastAnno: 1668	UniRef50_Q869S7	420	57.14	399	166	3	230	1420	23	418	2e-148	442
6.2.1.5(succinate---CoA ligase (ADP-forming);)																					
ABCDEF_17836	UniRef50_O82662	0.0	Normal: 367	77	365	530	1198	144	BlastAnno: 1491	UniRef50_O82662	421	85.48	420	61	0	131	1390	1	420	0.0	741
ABCDEF_45671	UniRef50_O82662	0.0	Normal: 1899	1293	2339	3905	6798	2137	BlastAnno: 1668	UniRef50_O82662	421	87.38	420	53	0	164	1423	1	420	0.0	753
6.4.1.1(pyruvate carboxylase;)																					
ABCDEF_45829	UniRef50_O27939	4e-167	Normal: 2364	1703	2036	5443	4075	6768	BlastAnno: 2081	UniRef50_O27939	491	54.80	469	207	2	396	1790	4	471	4e-167	498
>00030 Pentose phosphate pathway																					
1.1.1.215(gluconate 2-dehydrogenase;)																					
No Correspond Unigene																					
1.1.1.43(phosphogluconate 2-dehydrogenase;)																					
No Correspond Unigene																					
1.1.1.44(phosphogluconate dehydrogenase (decarboxylating);)																					
ABCDEF_4346	UniRef50_B8B1K3	0.0	Normal: 2255	809	3044	3718	2317	1112	BlastAnno: 2100	UniRef50_B8B1K3	475	66.90	426	117	6	228	1502	2	404	0.0	552
ABCDEF_3900	UniRef50_B8B1K3	0.0	Normal: 1843	1331	2740	5139	3536	1587	BlastAnno: 1978	UniRef50_B8B1K3	475	74.88	414	85	4	1721	480	4	398	0.0	609
ABCDEF_38637	UniRef50_B8B1K3	0.0	Normal: 1655	1399	2681	4781	5856	2437	BlastAnno: 1907	UniRef50_B8B1K3	475	73.56	416	88	5	1711	467	4	398	0.0	588
1.1.1.47(glucose 1-dehydrogenase;)																					
No Correspond Unigene																					
1.1.1.49(glucose-6-phosphate dehydrogenase;)																					
ABCDEF_328	UniRef50_B9FFT3	0.0	Normal: 317	217	403	1011	1197	77	BlastAnno: 1924	UniRef50_B9FFT3	473	73.87	486	88	4	291	1748	26	472	0.0	732
ABCDEF_3893	UniRef50_B9FFT3	0.0	Normal: 420	902	719	2614	3215	1238	BlastAnno: 2131	UniRef50_B9FFT3	473	73.98	492	89	4	291	1766	20	472	0.0	737
ABCDEF_330	UniRef50_B9FFT3	0.0	Normal: 298	220	395	1039	1218	65	BlastAnno: 1870	UniRef50_B9FFT3	473	73.87	486	88	4	237	1694	26	472	0.0	732
ABCDEF_60678	UniRef50_C1FH53	0.0	Normal: 119	335	60	38	62	427	BlastAnno: 2449	UniRef50_C1FH53	552	67.94	262	83	1	1300	2082	288	549	0.0	398
ABCDEF_47341	UniRef50_C1FH53	0.0	Normal: 110	325	58	37	56	390	BlastAnno: 2674	UniRef50_C1FH53	552	68.16	380	120	1	1171	2307	170	549	0.0	569
ABCDEF_47349	UniRef50_Q8L743	0.0	Normal: 68	187	32	19	34	251	BlastAnno: 1382	UniRef50_Q8L743	599	86.94	314	41	0	80	1021	283	596	0.0	578
ABCDEF_329	UniRef50_B9FFT3	0.0	Normal: 301	232	393	1017	1193	65	BlastAnno: 1871	UniRef50_B9FFT3	473	73.87	486	88	4	238	1695	26	472	0.0	732
ABCDEF_62298	UniRef50_Q8L743	0.0	Normal: 69	208	35	25	40	262	BlastAnno: 1913	UniRef50_Q8L743	599	77.54	236	48	1	545	1252	111	341	0.0	381
ABCDEF_47347	UniRef50_C1FH53	0.0	Normal: 113	317	51	33	60	447	BlastAnno: 2638	UniRef50_C1FH53	552	63.92	413	146	2	533	1765	60	471	0.0	554
ABCDEF_3894	UniRef50_B9FFT3	0.0	Normal: 449	941	777	2782	3454	1344	BlastAnno: 2213	UniRef50_B9FFT3	473	73.98	492	89	4	330	1805	20	472	0.0	737
ABCDEF_47345	UniRef50_C5X1U9	0.0	Normal: 124	344	62	39	67	436	BlastAnno: 2363	UniRef50_C5X1U9	627	55.56	486	205	3	542	1996	150	625	0.0	574
ABCDEF_71258	UniRef50_Q9FJI5	0.0	Normal: 83	55	85	312	404	13	BlastAnno: 1043	UniRef50_Q9FJI5	515	83.76	314	48	2	111	1043	1	314	0.0	537
ABCDEF_45319	UniRef50_C5X1U9	0.0	Normal: 630	468	546	883	837	630	BlastAnno: 2119	UniRef50_C5X1U9	627	63.73	477	171	2	402	1829	151	626	0.0	616
ABCDEF_351	UniRef50_C5X1U9	0.0	Normal: 966	364	1313	3355	4586	650	BlastAnno: 2309	UniRef50_C5X1U9	627	51.23	529	235	6	565	2112	107	625	0.0	550
ABCDEF_47342	UniRef50_Q8L743	0.0	Normal: 105	317	56	36	51	381	BlastAnno: 2760	UniRef50_Q8L743	599	85.17	263	39	0	1611	2399	334	596	0.0	475
1.1.3.4(glucose oxidase;)																					
No Correspond Unigene																					
1.1.3.5(hexose oxidase)																					
No Correspond Unigene																					
1.1.5.2(quinoprotein glucose dehydrogenase;)																					
No Correspond Unigene																					
1.1.99.10(glucose dehydrogenase (acceptor);)																					
No Correspond Unigene																					
1.1.99.3(gluconate 2-dehydrogenase (acceptor);)																					
No Correspond Unigene																					
1.2.7.5(aldehyde ferredoxin oxidoreductase;)																					
No Correspond Unigene																					
2.2.1.1(transketolase;)																					
ABCDEF_33843	UniRef50_P45694	0.0	Normal: 1647	3396	1878	3084	2916	10247	BlastAnno: 2902	UniRef50_P45694	667	52.71	664	298	8	2559	583	1	653	0.0	620
ABCDEF_33839	UniRef50_Q43848	0.0	Normal: 564	1182	798	1094	1060	3501	BlastAnno: 1311	UniRef50_Q43848	741	92.11	304	23	1	1	909	426	729	0.0	537

ABCDEF_33840	UniRef50_P45694	0.0	Normal: 1558	3002	1682	2815	2569	8787	BlastAnno: 2829	UniRef50_P45694	667	52.71	664	298	8	345	2321	1	653	0.0	619
2.2.1.2(transaldolase;)																					
ABCDEF_38820	UniRef50_B9T2V8	8e-147	Normal: 1172	1318	1457	2746	2877	3296	BlastAnno: 1468	UniRef50_B9T2V8	367	66.29	350	94	5	1314	265	38	363	8e-147	434
2.7.1.-(Transferases;)																					
ABCDEF_19669	UniRef50_O04059	0.0	Normal: 81	66	74	92	96	76	BlastAnno: 1254	UniRef50_O04059	594	81.65	376	40	1	3	1130	84	430	0.0	571
ABCDEF_19662	UniRef50_O04059	0.0	Normal: 103	74	68	108	100	97	BlastAnno: 1553	UniRef50_O04059	594	89.77	430	44	0	1419	130	1	430	0.0	752
ABCDEF_47654	UniRef50_Q86C65	0.0	Normal: 2310	2222	1853	2946	2297	3238	BlastAnno: 6212	UniRef50_Q86C65	2380	48.37	2030	881	32	6204	241	476	2380	0.0	1769
ABCDEF_19665	UniRef50_O04059	0.0	Normal: 100	80	57	109	104	104	BlastAnno: 1759	UniRef50_O04059	594	89.21	380	41	0	1390	251	60	439	0.0	655
ABCDEF_19655	UniRef50_O04059	0.0	Normal: 197	122	157	224	212	154	BlastAnno: 2166	UniRef50_O04059	594	86.71	572	76	0	2032	317	1	572	0.0	914
ABCDEF_19659	UniRef50_O04059	0.0	Normal: 200	133	145	233	228	183	BlastAnno: 2278	UniRef50_O04059	594	86.89	572	75	0	2032	317	1	572	0.0	916
ABCDEF_19671	UniRef50_O04059	0.0	Normal: 130	125	102	159	150	145	BlastAnno: 1767	UniRef50_O04059	594	89.77	430	44	0	202	1491	1	430	0.0	751
2.7.1.11(6-phosphofructokinase;)																					
ABCDEF_48463	UniRef50_Q94AA4	0.0	Normal: 273	212	275	545	518	593	BlastAnno: 1885	UniRef50_Q94AA4	489	71.85	437	122	1	403	1713	17	452	0.0	681
ABCDEF_48462	UniRef50_Q9FKG3	0.0	Normal: 197	165	201	395	395	441	BlastAnno: 1773	UniRef50_Q9FKG3	530	76.95	295	61	2	316	1197	44	332	0.0	469
ABCDEF_29376	UniRef50_Q94AA4	0.0	Normal: 0	44	0	0	20	222	BlastAnno: 1555	UniRef50_Q94AA4	489	81.00	458	82	3	99	1472	8	460	0.0	756
ABCDEF_49371	UniRef50_Q94AA4	0.0	Normal: 16	353	32	5	82	64	BlastAnno: 1513	UniRef50_Q94AA4	489	77.23	470	98	3	30	1421	10	476	0.0	752
ABCDEF_48301	UniRef50_Q8VYN6	0.0	Normal: 298	135	430	474	1016	200	BlastAnno: 2035	UniRef50_Q8VYN6	537	78.25	538	112	3	128	1729	1	537	0.0	844
ABCDEF_41341	UniRef50_Q94AA4	0.0	Normal: 889	655	932	1360	2142	1363	BlastAnno: 2130	UniRef50_Q94AA4	489	80.89	450	85	1	1832	483	6	454	0.0	755
2.7.1.12(gluconokinase;)																					
ABCDEF_58507	UniRef50_Q111A8	3e-30	Normal: 22	26	22	32	51	28	BlastAnno: 707	UniRef50_Q111A8	170	38.10	168	90	3	142	645	3	156	3e-30	118
ABCDEF_70017	UniRef50_Q111A8	3e-30	Normal: 15	24	19	42	45	25	BlastAnno: 685	UniRef50_Q111A8	170	38.10	168	90	3	123	626	3	156	3e-30	117
2.7.1.13(dehydrogluconokinase;)																					
No Correspond Unigene																					
2.7.1.15(ribokinase;)																					
ABCDEF_35167	UniRef50_Q22KI6	1e-46	Normal: 123	13	112	104	300	534	BlastAnno: 1508	UniRef50_Q22KI6	337	37.54	333	179	5	365	1285	4	333	1e-46	173
2.7.1.45(2-dehydro-3-deoxygluconokinase;)																					
No Correspond Unigene																					
2.7.4.23(ribose 1,5-bisphosphate phosphokinase;)																					
No Correspond Unigene																					
2.7.6.1(ribose-phosphate diphosphokinase;)																					
ABCDEF_48760	UniRef50_Q42581	0.0	Normal: 973	566	683	870	1272	1444	BlastAnno: 1493	UniRef50_Q42581	403	82.77	354	55	3	145	1206	48	395	0.0	596
ABCDEF_48326	UniRef50_Q93Z66	0.0	Normal: 385	227	399	743	698	724	BlastAnno: 1568	UniRef50_Q93Z66	411	72.70	381	99	3	1352	213	35	411	0.0	546
ABCDEF_38177	UniRef50_Q680A5	0.0	Normal: 875	249	761	568	782	550	BlastAnno: 1490	UniRef50_Q680A5	337	82.07	329	58	1	1198	212	10	337	0.0	562
ABCDEF_48346	UniRef50_Q42581	0.0	Normal: 106	135	82	88	251	542	BlastAnno: 1942	UniRef50_Q42581	403	81.59	364	62	3	1743	667	39	402	0.0	611
3.1.1.17(gluconolactonase;)																					
ABCDEF_17917	UniRef50_A1TXZ0	5e-64	Normal: 303	575	152	163	236	2055	BlastAnno: 1384	UniRef50_A1TXZ0	363	36.89	347	200	6	1216	200	23	358	5e-64	219
3.1.1.31(6-phosphogluconolactonase;)																					
ABCDEF_11057	UniRef50_Q9LMX8	1e-117	Normal: 96	107	140	197	114	41	BlastAnno: 1016	UniRef50_Q9LMX8	268	67.54	268	87	0	114	917	1	268	1e-117	349
3.1.3.11(fructose-bisphosphatase;)																					
ABCDEF_28302	UniRef50_P25851	0.0	Normal: 1013	1937	653	1129	1293	5773	BlastAnno: 1619	UniRef50_P25851	417	66.49	382	125	2	1417	281	36	417	0.0	544
ABCDEF_35263	UniRef50_Q9XGG5	0.0	Normal: 72	1508	41	126	415	7941	BlastAnno: 1577	UniRef50_Q9XGG5	329	92.62	325	24	0	373	1347	1	325	0.0	627
ABCDEF_7505	UniRef50_P25851	0.0	Normal: 1194	5939	568	263	269	21425	BlastAnno: 1662	UniRef50_P25851	417	81.75	389	66	3	1369	218	29	417	0.0	613
4.1.2.-(Lyases;)																					
ABCDEF_38428	UniRef50_C3KN71	1e-47	Normal: 551	427	606	695	1516	1338	BlastAnno: 1495	UniRef50_C3KN71	273	38.34	253	150	1	334	1092	7	253	1e-47	173
4.1.2.13(fructose-bisphosphate aldolase;)																					
ABCDEF_812	UniRef50_Q40677	0.0	Normal: 2391	1243	3255	6494	7322	1031	BlastAnno: 1720	UniRef50_Q40677	388	81.39	360	67	0	362	1441	29	388	0.0	612
ABCDEF_4038	UniRef50_Q40677	0.0	Normal: 2914	23962	1997	525	623	51538	BlastAnno: 1570	UniRef50_Q40677	388	82.87	397	53	3	123	1301	3	388	0.0	641
ABCDEF_35511	UniRef50_B9FU33	0.0	Normal: 18	1991	3	12	56	2290	BlastAnno: 1432	UniRef50_B9FU33	559	71.11	398	101	3	1423	245	171	559	0.0	536
ABCDEF_14742	UniRef50_Q40677	0.0	Normal: 4220	14155	2670	281	395	26738	BlastAnno: 1587	UniRef50_Q40677	388	89.94	348	35	0	1291	248	41	388	0.0	657
4.1.2.14(2-dehydro-3-deoxy-phosphogluconate aldolase;)																					
ABCDEF_11235	UniRef50_C1F496	4e-27	Normal: 18	32	12	12	50	235	BlastAnno: 1242	UniRef50_C1F496	218	35.98	214	129	4	165	797	9	217	4e-27	114
4.1.2.4(deoxyribose-phosphate aldolase;)																					
No Correspond Unigene																					
4.1.2.9(phosphoketolase;)																					
No Correspond Unigene																					
4.2.1.12(phosphogluconate dehydratase;)																					
No Correspond Unigene																					
4.2.1.39(gluconate dehydratase;)																					
No Correspond Unigene																					
4.3.1.9(glucosaminate ammonia-lyase;)																					
No Correspond Unigene																					
5.1.3.1(ribulose-phosphate 3-epimerase;)																					
ABCDEF_45834	UniRef50_Q9ZTP5	4e-147	Normal: 2109	7922	1619	1068	1971	21649	BlastAnno: 1506	UniRef50_Q9ZTP5	274	91.77	243	18	1	440	1168	32	272	4e-147	431
5.3.1.6(ribose-5-phosphate isomerase;)																					
ABCDEF_35205	UniRef50_B8FL28	3e-60	Normal: 177	481	176	230	243	502	BlastAnno: 1012	UniRef50_B8FL28	230	51.07	233	103	4	905	207	3	224	3e-60	201
5.3.1.9(glucose-6-phosphate isomerase;)																					
ABCDEF_21891	UniRef50_B5EBB8	0.0	Normal: 2382	2326	3109	5032	5330	3316	BlastAnno: 2692	UniRef50_B5EBB8	530	60.59	510	196	2	2176	647	4	508	0.0	624
ABCDEF_4218	UniRef50_B7GDV0	0.0	Normal: 982	971	1324	2230	3149	3125	BlastAnno: 2219	UniRef50_B7GDV0	786	56.56	564	238	4	2045	375	188	751	0.0	639
5.4.2.2(phosphoglucomutase;)																					
ABCDEF_935	UniRef50_P47244	0.0	Normal: 5430	4330	5743	12110	19002	6223	BlastAnno: 2144	UniRef50_P47244	572	53.66	587	246	11	2064	328	4	572	0.0	585
ABCDEF_24641	UniRef50_P47244	0.0	Normal: 416	659	414	959	874	2109	BlastAnno: 2341	UniRef50_P47244	572	54.17	576	222	11	386	2035	13	572	0.0	588
ABCDEF_24640	UniRef50_P47244	0.0	Normal: 389	596	384	834	777	1859	BlastAnno: 2422	UniRef50_P47244	572	51.74	603	222	12	386	2116	13	572	0.0	574

5.4.2.7(phosphopentomutase;)
No Correspond Unigene

>00040 Pentose and glucuronate interconversions

1.1.1.10(L-xylulose reductase;)
No Correspond Unigene

1.1.1.11(D-arabinitol 4-dehydrogenase;)
No Correspond Unigene

1.1.1.12(L-arabinitol 4-dehydrogenase;)
No Correspond Unigene

1.1.1.125(2-deoxy-D-gluconate 3-dehydrogenase;)

ABCDEF_27244	UniRef50_Q0RGH3	3e-18	Normal: 166	157	205	392	726	160	BlastAnno: 955	UniRef50_Q0RGH3	288	28.95	228	145	6	847	179	68	283	3e-18	88.2

1.1.1.127(2-dehydro-3-deoxy-D-gluconate 5-dehydrogenase;)
No Correspond Unigene

1.1.1.13(L-arabinitol 2-dehydrogenase;)
No Correspond Unigene

1.1.1.130(3-dehydro-L-gulonate 2-dehydrogenase;)
No Correspond Unigene

1.1.1.137(ribitol-5-phosphate 2-dehydrogenase;)
No Correspond Unigene

1.1.1.15(D-iditol 2-dehydrogenase;)
No Correspond Unigene

1.1.1.175(D-xylose 1-dehydrogenase;)
No Correspond Unigene

1.1.1.179(D-xylose 1-dehydrogenase (NADP+);)
No Correspond Unigene

1.1.1.19(glucuronate reductase;)
No Correspond Unigene

1.1.1.21(aldehyde reductase;)

ABCDEF_52723	UniRef50_Q6IMN8	5e-83	Normal: 1383	764	1621	1675	2066	2197	BlastAnno: 1221	UniRef50_Q6IMN8	297	44.09	313	153	5	135	1061	2	296	5e-83	265

1.1.1.22(UDP-glucose 6-dehydrogenase;)

ABCDEF_30909	UniRef50_A3AK92	0.0	Normal: 865	1783	1481	3602	5414	3960	BlastAnno: 1822	UniRef50_A3AK92	438	76.89	463	77	2	235	1617	1	435	0.0	749
ABCDEF_67405	UniRef50_A3AK92	0.0	Normal: 83	280	233	170	222	547	BlastAnno: 1342	UniRef50_A3AK92	438	77.64	407	61	2	1217	3	1	379	0.0	653
ABCDEF_48744	UniRef50_A3AK92	0.0	Normal: 89	350	273	228	296	697	BlastAnno: 1407	UniRef50_A3AK92	438	77.75	409	61	2	1221	1	1	381	0.0	658
ABCDEF_4415	UniRef50_A3AK92	0.0	Normal: 493	713	643	1516	2818	758	BlastAnno: 1410	UniRef50_A3AK92	438	76.38	381	60	2	1	1137	86	438	0.0	604
ABCDEF_30910	UniRef50_A3AK92	0.0	Normal: 920	2339	1416	3594	6463	6266	BlastAnno: 1816	UniRef50_A3AK92	438	77.54	463	74	2	1591	209	1	435	0.0	751
ABCDEF_30908	UniRef50_A3AK92	0.0	Normal: 2277	2525	3512	8495	12648	3403	BlastAnno: 1908	UniRef50_A3AK92	438	77.25	466	76	2	235	1626	1	438	0.0	755
ABCDEF_73000	UniRef50_Q9AUV6	0.0	Normal: 307	765	498	1248	2232	1778	BlastAnno: 905	UniRef50_Q9AUV6	480	90.37	301	29	0	904	2	95	395	0.0	572
ABCDEF_84803	UniRef50_A3AK92	0.0	Normal: 78	278	221	166	230	569	BlastAnno: 1468	UniRef50_A3AK92	438	77.70	408	61	2	1220	3	1	380	0.0	656

1.1.1.45(L-gulonate 3-dehydrogenase;)
No Correspond Unigene

1.1.1.56(ribitol 2-dehydrogenase;)
No Correspond Unigene

1.1.1.57(fructuronate reductase;)
No Correspond Unigene

1.1.1.58(tagaturonate reductase;)
No Correspond Unigene

1.1.1.9(D-xylulose reductase;)
No Correspond Unigene

1.1.2.2(mannitol dehydrogenase (cytochrome);)
No Correspond Unigene

1.2.1.3(aldehyde dehydrogenase (NAD+);)

ABCDEF_52483	UniRef50_Q70E96	0.0	Normal: 28	851	41	0	3965	420	BlastAnno: 1750	UniRef50_Q70E96	484	54.56	482	219	0	107	1552	3	484	0.0	548
ABCDEF_17638	UniRef50_Q70E96	0.0	Normal: 580	718	749	2424	1317	775	BlastAnno: 1970	UniRef50_Q70E96	484	62.21	471	177	1	283	1692	2	472	0.0	625
ABCDEF_403	UniRef50_Q8W033	0.0	Normal: 69	355	98	39	223	1111	BlastAnno: 1749	UniRef50_Q8W033	550	65.96	473	161	0	1523	105	74	546	0.0	625
ABCDEF_45143	UniRef50_Q0WSF1	0.0	Normal: 1445	874	1200	1789	1940	1504	BlastAnno: 2450	UniRef50_Q0WSF1	596	79.97	594	115	2	2220	451	1	594	0.0	1015

2.4.1.17(glucuronosyltransferase;)
No Correspond Unigene

2.7.1.16(ribulokinase;)

ABCDEF_37919	UniRef50_B5KEZ7	2e-126	Normal: 330	404	294	332	355	960	BlastAnno: 2134	UniRef50_B5KEZ7	545	40.90	555	295	8	2002	347	11	535	2e-126	395

2.7.1.17(xylulokinase;)

ABCDEF_37900	UniRef50_O75191	2e-160	Normal: 421	465	277	337	600	2935	BlastAnno: 1910	UniRef50_O75191	536	46.42	545	264	9	129	1754	11	530	2e-160	480

2.7.1.43(glucuronokinase;)
No Correspond Unigene

2.7.1.45(2-dehydro-3-deoxygluconokinase;)
No Correspond Unigene

2.7.1.47(D-ribulokinase;)
No Correspond Unigene

2.7.1.5(rhamnulokinase;)
No Correspond Unigene

2.7.1.53(L-xylulokinase;)
No Correspond Unigene

2.7.7.40(D-ribitol-5-phosphate cytidylyltransferase;)

	No Correspond Unigene																				
2.7.7.44(glucuronate-1-phosphate uridylyltransferase;)																					
	No Correspond Unigene																				
2.7.7.9(UTP---glucose-1-phosphate uridylyltransferase;)																					
	ABCDEF_42404	UniRef50_P57751	0.0	Normal: 2120	1934	2860	5861	9792	3375	BlastAnno: 1928	UniRef50_P57751	470	84.13	460	71	2	207	1583	6	464	0.0	756
	ABCDEF_68465	UniRef50_P57751	0.0	Normal: 1684	1594	2449	5480	7890	2628	BlastAnno: 1863	UniRef50_P57751	470	84.13	460	71	2	1678	302	6	464	0.0	756
3.1.1.11(pectinesterase;)																					
	ABCDEF_48873	UniRef50_B9N4L5	0.0	Normal: 32	42	68	373	320	42	BlastAnno: 945	UniRef50_B9N4L5	502	77.96	304	67	0	33	944	195	498	0.0	538
	ABCDEF_45683	UniRef50_Q1JPL7	0.0	Normal: 6813	6115	6504	5953	20996	376	BlastAnno: 1883	UniRef50_Q1JPL7	557	55.89	560	231	6	59	1702	2	557	0.0	606
	ABCDEF_39037	UniRef50_Q43111	0.0	Normal: 0	832	0	1248	88	3353	BlastAnno: 2135	UniRef50_Q43111	581	56.23	594	237	12	1987	236	1	581	0.0	595
	ABCDEF_48615	UniRef50_C5XIT6	0.0	Normal: 5939	5402	4656	2532	11870	5247	BlastAnno: 2220	UniRef50_C5XIT6	576	56.60	530	205	7	382	1902	49	576	0.0	590
	ABCDEF_4025	UniRef50_Q8L7Q7	0.0	Normal: 1134	2265	1101	1078	864	1623	BlastAnno: 2149	UniRef50_Q8L7Q7	602	56.41	601	236	7	1972	239	5	602	0.0	655
	ABCDEF_21060	UniRef50_Q9M3B0	0.0	Normal: 302	2377	237	1706	3680	2294	BlastAnno: 2050	UniRef50_Q9M3B0	598	68.76	541	156	6	4	1596	60	597	0.0	714
	ABCDEF_11423	UniRef50_O22149	0.0	Normal: 5	87	0	0	0	308	BlastAnno: 1565	UniRef50_O22149	511	68.57	509	143	7	46	1563	9	503	0.0	679
	ABCDEF_41398	UniRef50_Q9SMY7	0.0	Normal: 443	103	478	614	2563	224	BlastAnno: 1931	UniRef50_Q9SMY7	525	61.71	525	173	8	228	1739	8	525	0.0	601
	ABCDEF_37616	UniRef50_Q8L7Q7	0.0	Normal: 137	194	120	365	277	132	BlastAnno: 1799	UniRef50_Q8L7Q7	602	56.19	509	210	6	220	1713	91	597	0.0	569
	ABCDEF_17702	UniRef50_Q56W36	0.0	Normal: 1505	3569	1047	677	2800	2803	BlastAnno: 2042	UniRef50_Q56W36	381	73.56	382	80	2	1267	182	1	381	0.0	572
	ABCDEF_71054	UniRef50_Q43111	0.0	Normal: 116	287	99	507	262	1043	BlastAnno: 1410	UniRef50_Q43111	581	61.18	456	162	7	27	1373	134	581	0.0	566
	ABCDEF_45132	UniRef50_O48711	0.0	Normal: 72	1016	82	124	1126	92	BlastAnno: 1987	UniRef50_O48711	547	65.12	516	171	5	302	1831	35	547	0.0	694
	ABCDEF_10255	UniRef50_Q9M3B0	0.0	Normal: 125	2405	248	1499	1355	2608	BlastAnno: 2207	UniRef50_Q9M3B0	598	65.28	530	169	6	1861	284	79	597	0.0	652
	ABCDEF_14881	UniRef50_O22149	0.0	Normal: 45	67	46	969	2833	312	BlastAnno: 1739	UniRef50_O22149	511	63.10	496	165	10	1628	156	29	511	0.0	620
	ABCDEF_3485	UniRef50_Q8LPF3	0.0	Normal: 791	22	562	292	19	213	BlastAnno: 1188	UniRef50_Q8LPF3	362	79.18	317	64	1	86	1030	46	362	0.0	532
	ABCDEF_40958	UniRef50_Q9SMY7	0.0	Normal: 139	1736	370	2525	4188	716	BlastAnno: 2342	UniRef50_Q9SMY7	525	64.09	504	173	3	1690	179	28	523	0.0	606
	ABCDEF_38400	UniRef50_Q9LVQ0	0.0	Normal: 559	448	446	416	590	768	BlastAnno: 1239	UniRef50_Q9LVQ0	317	77.56	312	70	0	1154	219	6	317	0.0	515
3.1.1.68(xylono-1,4-lactonase;)																					
	No Correspond Unigene																				
3.2.1.15(polygalacturonase;)																					
	ABCDEF_49100	UniRef50_A7PZL3	0.0	Normal: 1045	1066	1246	2395	2653	2235	BlastAnno: 1908	UniRef50_A7PZL3	491	76.83	436	101	0	362	1669	49	484	0.0	703
	ABCDEF_28046	UniRef50_O49432	0.0	Normal: 199	82	178	510	235	75	BlastAnno: 1684	UniRef50_O49432	481	70.34	408	119	2	376	1596	73	479	0.0	570
	ABCDEF_18085	UniRef50_A7PZL3	0.0	Normal: 1501	3298	977	345	485	4572	BlastAnno: 1766	UniRef50_A7PZL3	491	78.13	439	96	0	268	1584	51	489	0.0	745
3.2.1.31(beta-glucuronidase;)																					
	No Correspond Unigene																				
3.2.1.67(galacturan 1,4-alpha-galacturonidase;)																					
	ABCDEF_18085	UniRef50_B9RIT3	8e-165	Normal: 1501	3298	977	345	485	4572	BlastAnno: 1766	UniRef50_B9RIT3	452	52.86	454	175	4	226	1575	28	446	8e-165	486
4.1.1.34(dehydro-L-gulonate decarboxylase;)																					
	No Correspond Unigene																				
4.1.1.85(3-dehydro-L-gulonate-6-phosphate decarboxylase;)																					
	No Correspond Unigene																				
4.1.2.14(2-dehydro-3-deoxy-phosphogluconate aldolase;)																					
	ABCDEF_11235	UniRef50_C1F496	4e-27	Normal: 18	32	12	12	50	235	BlastAnno: 1242	UniRef50_C1F496	218	35.98	214	129	4	165	797	9	217	4e-27	114
4.1.2.19(rhamnulose-1-phosphate aldolase;)																					
	No Correspond Unigene																				
4.1.2.28(2-dehydro-3-deoxy-D-pentonate aldolase;)																					
	No Correspond Unigene																				
4.1.2.43(3-hexulose-6-phosphate synthase;)																					
	No Correspond Unigene																				
4.2.1.-(Lyases;)																					
	ABCDEF_48679	UniRef50_Q0RP29	5e-115	Normal: 568	325	563	941	1285	676	BlastAnno: 1424	UniRef50_Q0RP29	493	56.39	321	129	4	119	1075	39	350	5e-115	356
4.2.1.7(altronate dehydratase;)																					
	No Correspond Unigene																				
4.2.1.8(mannonate dehydratase;)																					
	No Correspond Unigene																				
4.2.1.82(xylonate dehydratase;)																					
	No Correspond Unigene																				
4.2.2.2(pectate lyase;)																					
	ABCDEF_75654	UniRef50_O24554	0.0	Normal: 350	513	804	6304	5387	805	BlastAnno: 2049	UniRef50_O24554	401	71.96	378	104	2	1718	585	26	401	0.0	536
	ABCDEF_19824	UniRef50_Q944R1	0.0	Normal: 803	280	2094	12630	14265	1063	BlastAnno: 2664	UniRef50_Q944R1	470	77.37	411	89	1	286	1506	60	470	0.0	655
	ABCDEF_19816	UniRef50_O24554	0.0	Normal: 1013	613	679	2218	2179	806	BlastAnno: 2054	UniRef50_O24554	401	71.69	378	105	1	1719	586	26	401	0.0	542
	ABCDEF_19813	UniRef50_O24554	0.0	Normal: 998	252	1345	1892	2195	1517	BlastAnno: 1975	UniRef50_O24554	401	71.16	378	107	1	348	1481	26	401	0.0	545
	ABCDEF_19821	UniRef50_O24554	0.0	Normal: 747	328	1646	8504	10810	963	BlastAnno: 2623	UniRef50_O24554	401	71.96	378	104	1	332	1465	26	401	0.0	538
	ABCDEF_19809	UniRef50_O24554	0.0	Normal: 1652	628	535	1994	1903	658	BlastAnno: 2105	UniRef50_O24554	401	71.69	378	105	1	1761	628	26	401	0.0	540
	ABCDEF_19814	UniRef50_Q944R1	0.0	Normal: 364	1604	410	3223	1779	1504	BlastAnno: 2076	UniRef50_Q944R1	470	77.24	268	56	2	1878	1090	53	320	0.0	441
	ABCDEF_34571	UniRef50_O24554	0.0	Normal: 10	4069	8	501	1290	1190	BlastAnno: 1434	UniRef50_O24554	401	75.26	380	89	3	153	1289	26	401	0.0	564
	ABCDEF_19822	UniRef50_O24554	0.0	Normal: 662	696	469	1988	1533	832	BlastAnno: 2053	UniRef50_O24554	401	71.43	378	106	1	1718	585	26	401	0.0	541
	ABCDEF_19818	UniRef50_O24554	0.0	Normal: 710	329	671	1386	1500	809	BlastAnno: 1934	UniRef50_O24554	401	70.63	378	109	1	1599	466	26	401	0.0	549
	ABCDEF_45053	UniRef50_O24554	0.0	Normal: 1051	6003	1175	13873	9036	12779	BlastAnno: 1683	UniRef50_O24554	401	75.53	380	88	2	218	1354	26	401	0.0	567
	ABCDEF_1129	UniRef50_O24554	0.0	Normal: 296	2290	179	60	7	3262	BlastAnno: 1563	UniRef50_O24554	401	79.31	377	77	1	50	1180	26	401	0.0	529
	ABCDEF_19815	UniRef50_O24554	0.0	Normal: 376	1755	411	3467	1860	1592	BlastAnno: 1985	UniRef50_O24554	401	71.43	378	106	1	1682	549	26	401	0.0	540
	ABCDEF_19820	UniRef50_O24554	0.0	Normal: 348	543	356	2224	1870	717	BlastAnno: 2049	UniRef50_O24554	401	72.75	378	101	1	332	1465	26	401	0.0	547
4.2.2.6(oligogalacturonide lyase;)																					
	No Correspond Unigene																				
4.2.2.9(pectate disaccharide-lyase;)																					

No Correspond Unigene																					
5.-.-.-(Isomerases)																					
ABCDEF_37668	UniRef50_A8DJY4	1e-109	Normal: 240	328	262	459	844	1032	BlastAnno: 2373	UniRef50_A8DJY4	512	37.90	504	291	6	1825	359	12	508	1e-109	353
5.1.3.1(ribulose-phosphate 3-epimerase;)																					
ABCDEF_45834	UniRef50_Q9ZTP5	4e-147	Normal: 2109	7922	1619	1068	1971	21649	BlastAnno: 1506	UniRef50_Q9ZTP5	274	91.77	243	18	1	440	1168	32	272	4e-147	431
5.1.3.4(L-ribulose-5-phosphate 4-epimerase;)																					
No Correspond Unigene																					
5.3.1.-(Isomerases;)																					
No Correspond Unigene																					
5.3.1.12(glucuronate isomerase;)																					
No Correspond Unigene																					
5.3.1.15(D-lyxose ketol-isomerase;)																					
No Correspond Unigene																					
5.3.1.17(4-deoxy-L-threo-5-hexosulose-uronate ketol-isomerase;)																					
No Correspond Unigene																					
5.3.1.27(6-phospho-3-hexuloisomerase;)																					
No Correspond Unigene																					
5.3.1.4(L-arabinose isomerase;)																					
No Correspond Unigene																					
5.3.1.5(xylose isomerase;)																					
ABCDEF_28861	UniRef50_Q9FKK7	0.0	Normal: 2706	3110	2180	2073	2651	9512	BlastAnno: 1972	UniRef50_Q9FKK7	477	80.79	479	89	3	1657	224	1	477	0.0	790
>00051 Fructose and mannose metabolism																					
1.1.1.-(Oxidoreductases;)																					
ABCDEF_13453	UniRef50_Q9SYM5	0.0	Normal: 225	226	398	1950	2026	421	BlastAnno: 2578	UniRef50_Q9SYM5	669	86.61	672	87	2	298	2313	1	669	0.0	1226
ABCDEF_17342	UniRef50_Q84M96	0.0	Normal: 274	406	272	327	439	625	BlastAnno: 1277	UniRef50_Q84M96	346	73.33	345	91	1	1187	156	2	346	0.0	546
ABCDEF_31147	UniRef50_Q9SYM5	0.0	Normal: 4571	1591	1491	1401	1939	4327	BlastAnno: 2465	UniRef50_Q9SYM5	669	82.76	673	107	4	2397	394	1	669	0.0	1154
ABCDEF_17344	UniRef50_Q84M96	0.0	Normal: 252	373	257	297	380	540	BlastAnno: 1350	UniRef50_Q84M96	346	68.19	371	91	2	1263	154	2	346	0.0	530
ABCDEF_44674	UniRef50_Q9SYM5	0.0	Normal: 3581	2620	4329	9505	11447	4930	BlastAnno: 2634	UniRef50_Q9SYM5	669	87.80	672	78	3	253	2265	1	669	0.0	1241
1.1.1.11(D-arabinitol 4-dehydrogenase;)																					
No Correspond Unigene																					
1.1.1.132(GDP-mannose 6-dehydrogenase;)																					
No Correspond Unigene																					
1.1.1.135(GDP-6-deoxy-D-talose 4-dehydrogenase;)																					
No Correspond Unigene																					
1.1.1.138(mannitol 2-dehydrogenase (NADP+);)																					
No Correspond Unigene																					
1.1.1.14(L-iditol 2-dehydrogenase;)																					
ABCDEF_45723	UniRef50_Q00796	1e-104	Normal: 370	1302	302	520	723	2395	BlastAnno: 1404	UniRef50_Q00796	357	47.70	348	175	4	100	1140	8	349	1e-104	325
1.1.1.140(sorbitol-6-phosphate 2-dehydrogenase;)																					
No Correspond Unigene																					
1.1.1.15(D-iditol 2-dehydrogenase;)																					
No Correspond Unigene																					
1.1.1.17(mannitol-1-phosphate 5-dehydrogenase;)																					
No Correspond Unigene																					
1.1.1.173(L-rhamnose 1-dehydrogenase)																					
No Correspond Unigene																					
1.1.1.187(GDP-4-dehydro-D-rhamnose reductase;)																					
No Correspond Unigene																					
1.1.1.21(aldehyde reductase;)																					
ABCDEF_52723	UniRef50_Q6IMN8	5e-83	Normal: 1383	764	1621	1675	2066	2197	BlastAnno: 1221	UniRef50_Q6IMN8	297	44.09	313	153	5	135	1061	2	296	5e-83	265
1.1.1.271(GDP-L-fucose synthase;)																					
ABCDEF_25046	UniRef50_O49213	0.0	Normal: 852	424	935	1459	1534	964	BlastAnno: 1394	UniRef50_O49213	312	80.26	309	61	0	112	1038	4	312	0.0	520
1.1.1.281(GDP-4-dehydro-6-deoxy-D-mannose reductase;)																					
No Correspond Unigene																					
1.1.1.67(mannitol 2-dehydrogenase;)																					
No Correspond Unigene																					
1.1.2.2(mannitol dehydrogenase (cytochrome);)																					
No Correspond Unigene																					
1.1.99.21(D-sorbitol dehydrogenase (acceptor);)																					
No Correspond Unigene																					
2.4.1.-(Transferases;)																					
ABCDEF_18004	UniRef50_Q6YWK8	0.0	Normal: 1562	1736	2009	3452	4438	2400	BlastAnno: 2298	UniRef50_Q6YWK8	570	65.27	429	136	1	1941	694	57	485	0.0	608
ABCDEF_52379	UniRef50_Q9LHZ7	0.0	Normal: 4015	4921	4546	10520	13430	4731	BlastAnno: 3479	UniRef50_Q9LHZ7	1170	48.04	893	342	16	825	3242	299	1156	0.0	787
ABCDEF_48593	UniRef50_Q9SK82	0.0	Normal: 12	420	19	343	763	253	BlastAnno: 1834	UniRef50_Q9SK82	489	60.08	481	186	4	1714	278	10	486	0.0	633
ABCDEF_13375	UniRef50_Q8VZK9	0.0	Normal: 108	123	208	526	34	294	BlastAnno: 1738	UniRef50_Q8VZK9	729	53.62	552	234	8	94	1737	18	551	0.0	582
ABCDEF_40982	UniRef50_Q96301	0.0	Normal: 1037	706	819	1249	1084	920	BlastAnno: 3418	UniRef50_Q96301	914	79.60	902	173	3	243	2948	1	891	0.0	1506
ABCDEF_47674	UniRef50_Q9LHZ7	0.0	Normal: 274	92	202	599	505	280	BlastAnno: 2526	UniRef50_Q9LHZ7	1170	75.93	777	151	8	2260	2	15	779	0.0	1177
ABCDEF_34097	UniRef50_Q9LE59	0.0	Normal: 626	613	923	1797	1829	692	BlastAnno: 2521	UniRef50_Q9LE59	673	60.92	458	178	1	1687	317	215	672	0.0	630
ABCDEF_34098	UniRef50_Q9LE59	0.0	Normal: 457	464	697	1283	1312	539	BlastAnno: 2199	UniRef50_Q9LE59	673	60.92	458	178	1	1687	317	215	672	0.0	628
ABCDEF_10848	UniRef50_Q9M156	0.0	Normal: 157	2988	163	408	772	1566	BlastAnno: 1810	UniRef50_Q9M156	480	60.09	456	176	4	1700	336	4	454	0.0	553
ABCDEF_41182	UniRef50_Q9LHZ7	0.0	Normal: 150	2169	221	814	1407	495	BlastAnno: 3604	UniRef50_Q9LHZ7	1170	51.03	870	333	15	2822	456	299	1156	0.0	826
ABCDEF_40981	UniRef50_Q96301	0.0	Normal: 156	97	110	160	179	173	BlastAnno: 1225	UniRef50_Q96301	914	86.92	344	45	0	1112	81	394	737	0.0	652

ABCDEF_9506	UniRef50_Q9LHZ7	0.0	Normal: 1302	2128	1494	3356	4644	2206	BlastAnno: 3751	UniRef50_Q9LHZ7	1170	49.38	891	343	17	927	3359	299	1161	0.0	812
ABCDEF_9500	UniRef50_Q9LHZ7	0.0	Normal: 183	188	186	576	498	260	BlastAnno: 1717	UniRef50_Q9LHZ7	1170	47.98	644	234	14	3	1715	418	1033	0.0	571
ABCDEF_100	UniRef50_Q9M8Y0	0.0	Normal: 228	309	224	416	461	141	BlastAnno: 2109	UniRef50_Q9M8Y0	977	77.49	533	120	0	1610	12	50	582	0.0	884
ABCDEF_33659	UniRef50_Q9LHZ7	0.0	Normal: 1282	1948	2248	5826	10642	2083	BlastAnno: 4289	UniRef50_Q9LHZ7	1170	48.70	887	345	15	2955	538	299	1156	0.0	788
ABCDEF_37667	UniRef50_Q9LHZ7	0.0	Normal: 248	3617	251	1237	2078	970	BlastAnno: 3161	UniRef50_Q9LHZ7	1170	49.72	885	335	14	2671	260	301	1156	0.0	810
ABCDEF_47673	UniRef50_Q9LHZ7	0.0	Normal: 876	296	828	1628	1721	1172	BlastAnno: 3878	UniRef50_Q9LHZ7	1170	80.88	1161	186	8	3642	232	15	1163	0.0	1865
ABCDEF_99	UniRef50_Q9M8Y0	0.0	Normal: 272	330	222	398	423	145	BlastAnno: 2094	UniRef50_Q9M8Y0	977	77.36	561	127	0	1683	1	50	610	0.0	929
ABCDEF_20658	UniRef50_Q8VZK9	0.0	Normal: 373	33	193	64	503	141	BlastAnno: 2316	UniRef50_Q8VZK9	729	53.35	508	221	7	47	1564	11	504	0.0	559
ABCDEF_10183	UniRef50_A7XDQ9	0.0	Normal: 422	455	346	577	378	610	BlastAnno: 2362	UniRef50_A7XDQ9	684	50.94	693	307	12	281	2287	1	684	0.0	670
ABCDEF_30745	UniRef50_A7XDQ9	0.0	Normal: 1209	351	1031	1645	1226	635	BlastAnno: 2646	UniRef50_A7XDQ9	684	57.58	554	227	6	546	2189	133	684	0.0	664
ABCDEF_13336	UniRef50_Q7PC69	0.0	Normal: 148	563	194	957	1575	816	BlastAnno: 2584	UniRef50_Q7PC69	745	71.56	559	136	6	744	2351	187	745	0.0	730
ABCDEF_21426	UniRef50_Q8RU27	0.0	Normal: 2658	1979	3417	6157	9464	2599	BlastAnno: 1530	UniRef50_Q8RU27	366	89.44	360	34	2	1358	282	1	357	0.0	667
ABCDEF_71242	UniRef50_Q9ZV71	0.0	Normal: 134	122	179	480	754	119	BlastAnno: 1835	UniRef50_Q9ZV71	409	73.35	409	103	4	231	1439	1	409	0.0	587
ABCDEF_52020	UniRef50_Q6YWK8	0.0	Normal: 1635	1534	1975	4284	10353	2289	BlastAnno: 2394	UniRef50_Q6YWK8	570	58.37	514	185	2	403	1857	57	570	0.0	607
ABCDEF_51269	UniRef50_Q6YWK8	0.0	Normal: 147	85	103	21	33	438	BlastAnno: 1864	UniRef50_Q6YWK8	570	54.28	514	204	2	1646	198	57	570	0.0	586
ABCDEF_30753	UniRef50_Q9ZV71	0.0	Normal: 290	497	417	629	1013	634	BlastAnno: 1502	UniRef50_Q9ZV71	409	72.32	401	107	2	311	1501	1	401	0.0	590
ABCDEF_4233	UniRef50_Q9ZV71	0.0	Normal: 369	412	550	936	860	750	BlastAnno: 2004	UniRef50_Q9ZV71	409	72.13	409	110	2	370	1584	1	409	0.0	613
ABCDEF_17525	UniRef50_Q8RU27	0.0	Normal: 1500	1289	2149	3711	4446	1069	BlastAnno: 1507	UniRef50_Q8RU27	366	91.35	347	30	0	1371	331	3	349	0.0	671
ABCDEF_18005	UniRef50_Q6YWK8	0.0	Normal: 1842	2042	2515	4214	5208	2842	BlastAnno: 2305	UniRef50_Q6YWK8	570	59.14	514	180	2	1948	497	57	570	0.0	642
ABCDEF_41140	UniRef50_Q6YWK8	0.0	Normal: 86	1314	93	753	1787	2076	BlastAnno: 2346	UniRef50_Q6YWK8	570	60.00	510	174	2	431	1870	57	566	0.0	669
ABCDEF_18002	UniRef50_Q9LHZ7	0.0	Normal: 376	127	367	601	1190	520	BlastAnno: 1605	UniRef50_Q9LHZ7	1170	89.75	400	41	0	1603	404	771	1170	0.0	732
ABCDEF_13339	UniRef50_Q7PC69	0.0	Normal: 127	814	183	541	1135	1144	BlastAnno: 2610	UniRef50_Q7PC69	745	70.48	559	141	6	725	2329	187	745	0.0	729
ABCDEF_41141	UniRef50_Q6YWK8	0.0	Normal: 67	1107	84	708	1690	1854	BlastAnno: 2461	UniRef50_Q6YWK8	570	60.00	510	174	2	1916	477	57	566	0.0	668
ABCDEF_44583	UniRef50_Q9LE59	0.0	Normal: 681	561	559	947	1745	789	BlastAnno: 2470	UniRef50_Q9LE59	673	59.21	456	185	1	1749	385	214	669	0.0	621
ABCDEF_3869	UniRef50_Q9LE59	0.0	Normal: 333	393	415	809	907	754	BlastAnno: 2136	UniRef50_Q9LE59	673	59.05	613	227	4	1827	1	81	673	0.0	699
ABCDEF_28947	UniRef50_Q9SK82	0.0	Normal: 0	851	3	0	0	116	BlastAnno: 1567	UniRef50_Q9SK82	489	62.40	484	176	4	1524	85	1	482	0.0	652
ABCDEF_31119	UniRef50_Q9M8Y0	0.0	Normal: 2212	1279	1917	2933	3341	1803	BlastAnno: 3695	UniRef50_Q9M8Y0	977	81.08	925	174	1	392	3163	50	974	0.0	1583
ABCDEF_37311	UniRef50_Q9LE59	0.0	Normal: 175	133	191	549	421	366	BlastAnno: 1612	UniRef50_Q9LE59	673	62.96	486	153	4	1406	3	4	480	0.0	542
ABCDEF_24190	UniRef50_Q8L7F9	0.0	Normal: 397	265	492	583	553	371	BlastAnno: 2564	UniRef50_Q8L7F9	643	63.91	640	218	4	2353	473	1	640	0.0	850
ABCDEF_30548	UniRef50_Q9FH36	0.0	Normal: 17	916	27	151	420	126	BlastAnno: 1963	UniRef50_Q9FH36	535	72.47	534	138	3	102	1676	1	534	0.0	767
ABCDEF_27416	UniRef50_Q7PC69	0.0	Normal: 575	837	875	1344	2015	936	BlastAnno: 2454	UniRef50_Q7PC69	745	66.11	416	115	4	2337	1168	126	541	0.0	518
ABCDEF_48373	UniRef50_A7XDQ9	0.0	Normal: 1364	773	1336	1451	1791	1202	BlastAnno: 3042	UniRef50_A7XDQ9	684	63.68	691	234	6	2405	360	1	683	0.0	915
ABCDEF_3868	UniRef50_Q9LE59	0.0	Normal: 224	274	301	555	686	524	BlastAnno: 1670	UniRef50_Q9LE59	673	65.31	516	176	1	1556	9	161	673	0.0	696
ABCDEF_31288	UniRef50_Q940V3	0.0	Normal: 247	309	183	101	124	655	BlastAnno: 1658	UniRef50_Q940V3	470	56.01	466	197	7	1614	232	8	470	0.0	537
ABCDEF_52574	UniRef50_Q09332	0.0	Normal: 885	474	1068	1835	1853	816	BlastAnno: 3720	UniRef50_Q09332	1548	47.89	687	310	14	2551	533	859	1511	0.0	598
ABCDEF_79752	UniRef50_Q9ZV71	0.0	Normal: 158	183	245	400	379	352	BlastAnno: 1266	UniRef50_Q9ZV71	409	71.39	360	99	2	31	1098	1	360	0.0	526
ABCDEF_9509	UniRef50_Q9LHZ7	0.0	Normal: 1296	2171	1498	3410	4683	2232	BlastAnno: 3897	UniRef50_Q9LHZ7	1170	49.38	891	343	17	1073	3505	299	1161	0.0	812
ABCDEF_48200	UniRef50_Q6YWK8	0.0	Normal: 1768	576	3156	4431	6634	491	BlastAnno: 2274	UniRef50_Q6YWK8	570	57.98	514	185	4	291	1739	57	570	0.0	642
ABCDEF_24155	UniRef50_Q9ZVI7	0.0	Normal: 503	247	607	1676	1833	578	BlastAnno: 2708	UniRef50_Q9ZVI7	619	52.33	621	264	11	306	2123	15	618	0.0	601
ABCDEF_21246	UniRef50_Q9SKT6	0.0	Normal: 92	107	188	380	834	84	BlastAnno: 1791	UniRef50_Q9SKT6	536	56.61	484	204	5	123	1562	52	533	0.0	568
ABCDEF_32317	UniRef50_Q8RU27	0.0	Normal: 12	9	3	118	800	62	BlastAnno: 1076	UniRef50_Q8RU27	366	87.02	285	37	0	1075	221	64	348	0.0	535
ABCDEF_9502	UniRef50_Q9LHZ7	0.0	Normal: 610	486	568	1331	1403	591	BlastAnno: 1961	UniRef50_Q9LHZ7	1170	48.64	625	249	13	3	1742	564	1161	0.0	570
ABCDEF_23298	UniRef50_Q9FH36	0.0	Normal: 128	527	130	223	314	864	BlastAnno: 2832	UniRef50_Q9FH36	535	83.16	196	33	0	696	1283	340	535	0.0	357
ABCDEF_40595	UniRef50_O80898	0.0	Normal: 229	135	103	25	307	325	BlastAnno: 2413	UniRef50_O80898	757	44.68	761	378	13	95	2299	10	753	0.0	587
ABCDEF_63947	UniRef50_Q9SK82	0.0	Normal: 0	267	0	0	71	0	BlastAnno: 1446	UniRef50_Q9SK82	489	58.33	468	189	4	60	1445	1	468	0.0	592
ABCDEF_67847	UniRef50_Q9M8Y0	0.0	Normal: 794	476	871	1081	1401	868	BlastAnno: 1591	UniRef50_Q9M8Y0	977	84.86	370	56	0	1589	480	605	974	0.0	685
ABCDEF_65666	UniRef50_Q09332	0.0	Normal: 800	441	946	1606	1637	735	BlastAnno: 3518	UniRef50_Q09332	1548	47.54	671	305	13	1162	3087	859	1511	0.0	586
ABCDEF_38641	UniRef50_Q9FWA4	0.0	Normal: 674	967	1162	2603	4404	2475	BlastAnno: 1599	UniRef50_Q9FWA4	561	81.35	386	72	0	1	1158	176	561	0.0	641
ABCDEF_17914	UniRef50_Q9SKT6	0.0	Normal: 784	567	886	1447	1439	1424	BlastAnno: 2049	UniRef50_Q9SKT6	536	72.95	536	140	2	204	1796	1	536	0.0	785
ABCDEF_18286	UniRef50_Q6NQB7	0.0	Normal: 1131	1108	1225	1872	1722	3329	BlastAnno: 1752	UniRef50_Q6NQB7	393	73.80	397	81	6	1527	367	10	393	0.0	607
ABCDEF_119	UniRef50_Q8RU27	0.0	Normal: 1613	1155	2249	3558	7663	1775	BlastAnno: 1532	UniRef50_Q8RU27	366	88.33	360	38	2	1306	230	1	357	0.0	663
ABCDEF_47603	UniRef50_Q9M9Y5	0.0	Normal: 2609	8779	1664	1103	2085	22991	BlastAnno: 2830	UniRef50_Q9M9Y5	589	54.04	594	267	3	2475	694	1	588	0.0	654
ABCDEF_24192	UniRef50_Q8L7F9	0.0	Normal: 391	244	465	553	497	345	BlastAnno: 2477	UniRef50_Q8L7F9	643	58.99	434	165	4	212	1474	1	434	0.0	513
ABCDEF_17482	UniRef50_Q9SKT6	0.0	Normal: 615	379	816	1068	1470	852	BlastAnno: 2083	UniRef50_Q9SKT6	536	71.11	540	147	4	1915	311	1	536	0.0	786
ABCDEF_85909	UniRef50_Q8VZK9	0.0	Normal: 28	45	9	10	44	127	BlastAnno: 1473	UniRef50_Q8VZK9	729	55.37	475	198	6	1419	1	11	473	0.0	536
ABCDEF_9503	UniRef50_Q9LHZ7	0.0	Normal: 487	544	524	1242	1320	833	BlastAnno: 2087	UniRef50_Q9LHZ7	1170	50.35	574	236	8	57	1712	615	1161	0.0	567
ABCDEF_40594	UniRef50_O80898	0.0	Normal: 256	127	102	41	331	326	BlastAnno: 2417	UniRef50_O80898	757	45.76	719	348	12	213	2291	51	753	0.0	585
ABCDEF_17141	UniRef50_Q9LHZ7	0.0	Normal: 74	1734	69	380	701	333	BlastAnno: 3337	UniRef50_Q9LHZ7	1170	50.92	868	335	14	2539	179	299	1156	0.0	816
ABCDEF_33658	UniRef50_Q9LHZ7	0.0	Normal: 384	308	395	805	1003	602	BlastAnno: 2961	UniRef50_Q9LHZ7	1170	48.76	888	343	14	204	2621	299	1156	0.0	792
ABCDEF_20579	UniRef50_Q7PC69	0.0	Normal: 1007	795	1238	2311	2309	1345	BlastAnno: 3113	UniRef50_Q7PC69	745	59.97	767	229	14	2667	535	1	745	0.0	768
ABCDEF_31118	UniRef50_Q9M8Y0	0.0	Normal: 2135	1267	1900	3004	3329	1757	BlastAnno: 3787	UniRef50_Q9M8Y0	977	81.10	709	133	1	1132	3255	266	974	0.0	1217
ABCDEF_9499	UniRef50_Q9LHZ7	0.0	Normal: 205	207	215	632	538	275	BlastAnno: 1794	UniRef50_Q9LHZ7	1170	48.88	667	240	14	11	1792	395	1033	0.0	605
ABCDEF_48199	UniRef50_Q6YWK8	0.0	Normal: 1506	467	2429	3094	4233	439	BlastAnno: 1997	UniRef50_Q6YWK8	570	63.10	439	146	2	291	1559	57	495	0.0	610
ABCDEF_40983	UniRef50_Q96301	0.0	Normal: 406	261	343	392	415	433	BlastAnno: 2265	UniRef50_Q96301	914	79.17	504	99	1	114	1625	394	891	0.0	854
ABCDEF_17140	UniRef50_Q9LHZ7	0.0	Normal: 45	1049	49	216	386	208	BlastAnno: 2293	UniRef50_Q9LHZ7	1170	52.34	770	281	13	2138	57	397	1156	0.0	739
ABCDEF_18352	UniRef50_Q9FWA4	0.0	Normal: 2427	2688	2969	5519	6547	4855	BlastAnno: 2224	UniRef50_Q9FWA4	561	69.31	505	148	3	1887	382	61	561	0.0	683
ABCDEF_27767	UniRef50_Q9LHZ7	0.0	Normal: 2576	5141	2841	10249	14583	5031	BlastAnno: 3981	UniRef50_Q9LHZ7	1170	49.66	888	337	16	2927	504	299	1156	0.0	818
ABCDEF_33661	UniRef50_Q9LHZ7	0.0	Normal: 414	340	507	879	790	783	BlastAnno: 2185	UniRef50_Q9LHZ7	1170	48.66	748	277	14	2184	169	440	1156	0.0	662
ABCDEF_14685	UniRef50_A7XDQ9	0.0	Normal: 233	308	134	573	292	334	BlastAnno: 1947	UniRef50_A7XDQ9	684	56.84	468	186	6	569	1945	1	461	0.0	545
ABCDEF_20660	UniRef50_Q8VZK9	0.0	Normal: 22	48	13	15	52	144	BlastAnno: 1473	UniRef50_Q8VZK9	729	55.16	475	199	6	55	1473	11	473	0.0	537
ABCDEF_21315	UniRef50_Q9LHZ7	0.0	Normal: 3412	252	1450	0	79	1228	BlastAnno: 3765	UniRef50_Q9LHZ7	1170	63.78	1096	340	15	308	3508	70	1137	0.0	1333
ABCDEF_533	UniRef50_Q9C8W3	0.0	Normal: 230	204	269	505	411	301	BlastAnno: 1688	UniRef50_Q9C8W3	401	70.33	364	94	2	393	1475	47	399	0.0	555

ABCDEF_34099	UniRef50_Q9LE59	0.0	Normal: 478	491	735	1356	1350	548	BlastAnno: 2158	UniRef50_Q9LE59	673	60.92	458	178	1	1610	240	215	672	0.0	630
ABCDEF_47984	UniRef50_Q7PC69	0.0	Normal: 663	457	734	1362	2147	696	BlastAnno: 2838	UniRef50_Q7PC69	745	59.27	712	226	9	373	2316	34	745	0.0	702
ABCDEF_51451	UniRef50_Q9LE59	0.0	Normal: 1883	1782	2202	4337	4350	2667	BlastAnno: 2874	UniRef50_Q9LE59	673	74.16	685	165	6	2559	505	1	673	0.0	982
ABCDEF_13370	UniRef50_Q8VZK9	0.0	Normal: 290	87	317	377	105	258	BlastAnno: 2033	UniRef50_Q8VZK9	729	51.65	635	278	11	2031	154	112	726	0.0	612
ABCDEF_48239	UniRef50_Q9LHZ7	0.0	Normal: 511	163	422	1154	1316	510	BlastAnno: 2714	UniRef50_Q9LHZ7	1170	76.20	769	156	9	478	2712	41	806	0.0	1201
ABCDEF_13338	UniRef50_Q7PC69	0.0	Normal: 335	468	417	2639	4443	616	BlastAnno: 2525	UniRef50_Q7PC69	745	62.45	727	209	12	298	2292	21	745	0.0	781
ABCDEF_24193	UniRef50_Q8L7F9	0.0	Normal: 113	72	132	203	180	119	BlastAnno: 1679	UniRef50_Q8L7F9	643	60.54	479	176	4	212	1609	1	479	0.0	592
ABCDEF_48514	UniRef50_Q9ZV71	0.0	Normal: 540	580	601	771	1033	926	BlastAnno: 1920	UniRef50_Q9ZV71	409	78.14	398	85	2	1618	431	12	409	0.0	637
2.4.1.217(mannosyl-3-phosphoglycerate synthase;)																					
No Correspond Unigene																					
2.4.1.33(alginate synthase;)																					
No Correspond Unigene																					
2.7.1.1(hexokinase;)																					
ABCDEF_27834	UniRef50_P93834	0.0	Normal: 998	404	1410	4185	6485	623	BlastAnno: 2374	UniRef50_P93834	502	72.77	470	128	0	2024	615	25	494	0.0	655
ABCDEF_51305	UniRef50_P93834	0.0	Normal: 443	123	280	151	521	245	BlastAnno: 2179	UniRef50_P93834	502	76.47	289	68	0	1813	947	25	313	0.0	467
ABCDEF_420	UniRef50_Q1WM16	0.0	Normal: 453	101	514	402	696	75	BlastAnno: 1554	UniRef50_Q1WM16	463	61.66	433	159	3	3	1295	20	447	0.0	538
ABCDEF_86444	UniRef50_Q1WM16	0.0	Normal: 293	76	186	81	353	207	BlastAnno: 1684	UniRef50_Q1WM16	463	66.83	404	129	3	1404	196	63	462	0.0	530
ABCDEF_592	UniRef50_P93834	0.0	Normal: 378	177	486	1038	1819	341	BlastAnno: 1859	UniRef50_P93834	502	59.87	456	180	3	1618	260	37	492	0.0	537
ABCDEF_83497	UniRef50_Q1WM16	0.0	Normal: 290	84	185	81	350	338	BlastAnno: 1860	UniRef50_Q1WM16	463	66.83	404	129	3	1404	196	63	462	0.0	529
ABCDEF_51306	UniRef50_Q1WM16	0.0	Normal: 446	118	281	150	539	247	BlastAnno: 2108	UniRef50_Q1WM16	463	66.96	451	144	3	1685	336	16	462	0.0	578
ABCDEF_593	UniRef50_P93834	0.0	Normal: 403	191	504	1097	1940	353	BlastAnno: 1902	UniRef50_P93834	502	59.87	456	180	3	1648	290	37	492	0.0	537
2.7.1.105(6-phosphofructo-2-kinase;)																					
ABCDEF_13741	UniRef50_P16118	2e-114	Normal: 160	493	144	98	85	1459	BlastAnno: 2314	UniRef50_P16118	471	47.57	391	188	9	865	2016	45	425	2e-114	363
2.7.1.11(6-phosphofructokinase;)																					
ABCDEF_48463	UniRef50_Q94AA4	0.0	Normal: 273	212	275	545	518	593	BlastAnno: 1885	UniRef50_Q94AA4	489	71.85	437	122	1	403	1713	17	452	0.0	681
ABCDEF_48462	UniRef50_Q9FKG3	0.0	Normal: 197	165	201	395	395	441	BlastAnno: 1773	UniRef50_Q9FKG3	530	76.95	295	61	2	316	1197	44	332	0.0	469
ABCDEF_29376	UniRef50_Q94AA4	0.0	Normal: 0	44	0	0	20	222	BlastAnno: 1555	UniRef50_Q94AA4	489	81.00	458	82	3	99	1472	8	460	0.0	756
ABCDEF_49371	UniRef50_Q94AA4	0.0	Normal: 16	353	32	5	82	64	BlastAnno: 1513	UniRef50_Q94AA4	489	77.23	470	98	3	30	1421	10	476	0.0	752
ABCDEF_48301	UniRef50_Q8VYN6	0.0	Normal: 298	135	430	474	1016	200	BlastAnno: 2035	UniRef50_Q8VYN6	537	78.25	538	112	3	128	1729	1	537	0.0	844
ABCDEF_41341	UniRef50_Q94AA4	0.0	Normal: 889	655	932	1360	2142	1363	BlastAnno: 2130	UniRef50_Q94AA4	489	80.89	450	85	1	1832	483	6	454	0.0	755
2.7.1.28(triokinase;)																					
No Correspond Unigene																					
2.7.1.3(ketohexokinase;)																					
ABCDEF_62359	UniRef50_A1WVI5	7e-18	Normal: 21	41	18	14	41	196	BlastAnno: 704	UniRef50_A1WVI5	292	32.74	168	107	2	505	8	4	167	7e-18	85.9
2.7.1.4(fructokinase;)																					
ABCDEF_28475	UniRef50_Q9M1B9	0.0	Normal: 2729	2245	2850	2149	3141	1721	BlastAnno: 1388	UniRef50_Q9M1B9	326	85.49	317	45	1	1235	285	1	316	0.0	561
2.7.1.5(rhamnulokinase;)																					
No Correspond Unigene																					
2.7.1.51(L-fuculokinase;)																					
No Correspond Unigene																					
2.7.1.52(fucokinase;)																					
ABCDEF_10588	UniRef50_Q9LNJ9	1e-172	Normal: 184	212	165	177	305	322	BlastAnno: 1507	UniRef50_Q9LNJ9	1113	69.66	445	106	2	1	1260	671	1111	1e-172	525
2.7.1.56(1-phosphofructokinase;)																					
No Correspond Unigene																					
2.7.1.69(protein-Npi-phosphohistidine---sugar phosphotransferase;)																					
No Correspond Unigene																					
2.7.1.7(mannokinase;)																					
No Correspond Unigene																					
2.7.1.90(diphosphate---fructose-6-phosphate 1-phosphotransferase;)																					
ABCDEF_34827	UniRef50_Q41141	0.0	Normal: 1212	1133	1400	2279	3006	840	BlastAnno: 2109	UniRef50_Q41141	552	82.94	545	93	0	160	1794	6	550	0.0	930
ABCDEF_31958	UniRef50_Q41141	0.0	Normal: 4394	1638	4530	13039	14541	2431	BlastAnno: 2123	UniRef50_Q41141	552	84.34	549	86	0	1979	333	2	550	0.0	943
ABCDEF_31617	UniRef50_Q41140	0.0	Normal: 4497	1992	4496	12945	10620	2829	BlastAnno: 2373	UniRef50_Q41140	617	86.68	608	81	0	2278	455	1	608	0.0	1051
2.7.7.13(mannose-1-phosphate guanylyltransferase;)																					
ABCDEF_18119	UniRef50_B8LVX3	5e-150	Normal: 2069	3222	1844	3235	5944	6676	BlastAnno: 1767	UniRef50_B8LVX3	741	58.92	370	147	4	342	1439	373	741	5e-150	459
2.7.7.22(mannose-1-phosphate guanylyltransferase (GDP);)																					
No Correspond Unigene																					
2.7.7.30(fucose-1-phosphate guanylyltransferase;)																					
No Correspond Unigene																					
3.1.1.65(L-rhamnono-1,4-lactonase;)																					
No Correspond Unigene																					
3.1.3.-(Hydrolases;)																					
ABCDEF_3128	UniRef50_Q9ZU49	5e-117	Normal: 118	143	108	91	149	56	BlastAnno: 1434	UniRef50_Q9ZU49	327	57.43	303	121	2	1184	294	20	320	5e-117	355
3.1.3.11(fructose-bisphosphatase;)																					
ABCDEF_28302	UniRef50_P25851	0.0	Normal: 1013	1937	653	1129	1293	5773	BlastAnno: 1619	UniRef50_P25851	417	66.49	382	125	2	1417	281	36	417	0.0	544
ABCDEF_35263	UniRef50_Q9XGG5	0.0	Normal: 72	1508	41	126	415	7941	BlastAnno: 1577	UniRef50_Q9XGG5	329	92.62	325	24	0	373	1347	1	325	0.0	627
ABCDEF_7505	UniRef50_P25851	0.0	Normal: 1194	5939	568	263	269	21425	BlastAnno: 1662	UniRef50_P25851	417	81.75	389	66	3	1369	218	29	417	0.0	613
3.1.3.22(mannitol-1-phosphatase;)																					
No Correspond Unigene																					
3.1.3.46(fructose-2,6-bisphosphate 2-phosphatase;)																					
ABCDEF_13741	UniRef50_A9GKW0	5e-141	Normal: 160	493	144	98	85	1459	BlastAnno: 2314	UniRef50_A9GKW0	420	52.36	403	182	6	859	2058	11	406	5e-141	430
3.1.3.54(fructose-2,6-bisphosphate 6-phosphatase;)																					
No Correspond Unigene																					

3.1.3.70(mannosyl-3-phosphoglycerate phosphatase)																						
	No Correspond Unigene																					
3.2.1.137(mannan exo-1,2-1,6-alpha-mannosidase;)																						
	No Correspond Unigene																					
3.2.1.77(mannan 1,2-(1,3)-alpha-mannosidase;)																						
	No Correspond Unigene																					
3.2.1.78(mannan endo-1,4-beta-mannosidase;)																						
	ABCDEF_11247	UniRef50_Q9LZV3	0.0	Normal: 108	260	100	2243	1575	798	BlastAnno: 1479	UniRef50_Q9LZV3	448	66.01	459	142	5	13	1383	2	448	0.0	641
	ABCDEF_14081	UniRef50_Q7Y223	0.0	Normal: 439	457	484	1130	1749	793	BlastAnno: 2025	UniRef50_Q7Y223	433	67.23	412	134	1	1538	303	7	417	0.0	613
	ABCDEF_14082	UniRef50_Q7Y223	0.0	Normal: 513	616	609	1214	1787	937	BlastAnno: 2279	UniRef50_Q7Y223	433	67.23	412	134	1	1538	303	7	417	0.0	613
	ABCDEF_38385	UniRef50_Q9FJZ3	0.0	Normal: 869	1572	510	2673	2866	3714	BlastAnno: 1810	UniRef50_Q9FJZ3	431	65.97	432	139	4	1728	454	1	431	0.0	583
3.2.1.80(fructan beta-fructosidase;)																						
	ABCDEF_7582	UniRef50_Q67XZ3	0.0	Normal: 32	301	39	111	33	545	BlastAnno: 1949	UniRef50_Q67XZ3	594	58.17	557	226	4	1835	186	23	579	0.0	677
3.6.1.21(ADP-sugar diphosphatase;)																						
	ABCDEF_6188	UniRef50_Q9SZ63	2e-140	Normal: 60	106	66	96	87	338	BlastAnno: 1230	UniRef50_Q9SZ63	309	72.14	262	73	0	1056	271	43	304	2e-140	412
4.1.2.-(Lyases;)																						
	ABCDEF_38428	UniRef50_C3KN71	1e-47	Normal: 551	427	606	695	1516	1338	BlastAnno: 1495	UniRef50_C3KN71	273	38.34	253	150	1	334	1092	7	253	1e-47	173
4.1.2.13(fructose-bisphosphate aldolase;)																						
	ABCDEF_812	UniRef50_Q40677	0.0	Normal: 2391	1243	3255	6494	7322	1031	BlastAnno: 1720	UniRef50_Q40677	388	81.39	360	67	0	362	1441	29	388	0.0	612
	ABCDEF_4038	UniRef50_Q40677	0.0	Normal: 2914	23962	1997	525	623	51538	BlastAnno: 1570	UniRef50_Q40677	388	82.87	397	53	3	123	1301	3	388	0.0	641
	ABCDEF_35511	UniRef50_B9FU33	0.0	Normal: 18	1991	3	12	56	2290	BlastAnno: 1432	UniRef50_B9FU33	559	71.11	398	101	3	1423	245	171	559	0.0	536
	ABCDEF_14742	UniRef50_Q40677	0.0	Normal: 4220	14155	2670	281	395	26738	BlastAnno: 1587	UniRef50_Q40677	388	89.94	348	35	0	1291	248	41	388	0.0	657
4.1.2.17(L-fuculose-phosphate aldolase;)																						
	No Correspond Unigene																					
4.1.2.18(2-dehydro-3-deoxy-L-pentonate aldolase;)																						
	No Correspond Unigene																					
4.1.2.19(rhamnulose-1-phosphate aldolase;)																						
	No Correspond Unigene																					
4.2.1.47(GDP-mannose 4,6-dehydratase;)																						
	ABCDEF_48679	UniRef50_P93031	0.0	Normal: 568	325	563	941	1285	676	BlastAnno: 1424	UniRef50_P93031	373	84.86	370	54	2	41	1150	6	373	0.0	667
4.2.1.67(D-fuconate dehydratase;)																						
	No Correspond Unigene																					
4.2.1.90(L-rhamnonate dehydratase;)																						
	No Correspond Unigene																					
4.2.2.11(poly(alpha-L-guluronate) lyase;)																						
	No Correspond Unigene																					
4.2.2.3(poly(beta-D-mannuronate) lyase;)																						
	No Correspond Unigene																					
5.3.1.1(triose-phosphate isomerase;)																						
	ABCDEF_25548	UniRef50_Q9SKP6	8e-167	Normal: 879	1226	1007	1772	2081	5167	BlastAnno: 1419	UniRef50_Q9SKP6	315	75.95	316	70	3	1221	289	1	315	8e-167	482
5.3.1.14(L-rhamnose isomerase;)																						
	No Correspond Unigene																					
5.3.1.25(L-fucose isomerase)																						
	No Correspond Unigene																					
5.3.1.5(xylose isomerase;)																						
	ABCDEF_28861	UniRef50_Q9FKK7	0.0	Normal: 2706	3110	2180	2073	2651	9512	BlastAnno: 1972	UniRef50_Q9FKK7	477	80.79	479	89	3	1657	224	1	477	0.0	790
5.3.1.7(mannose isomerase;)																						
	No Correspond Unigene																					
5.3.1.8(mannose-6-phosphate isomerase;)																						
	ABCDEF_3858	UniRef50_Q67YT4	1e-176	Normal: 413	826	453	537	588	677	BlastAnno: 1801	UniRef50_Q67YT4	441	58.00	450	173	5	1474	134	2	438	1e-176	517
5.4.2.8(phosphomannomutase;)																						
	ABCDEF_10400	UniRef50_A6LQV8	3e-144	Normal: 173	143	196	105	138	487	BlastAnno: 2096	UniRef50_A6LQV8	521	46.33	531	248	9	1845	274	17	517	3e-144	440
>00052 Galactose metabolism																						
1.1.1.120(galactose 1-dehydrogenase (NADP+);)																						
	No Correspond Unigene																					
1.1.1.16(galactitol 2-dehydrogenase;)																						
	No Correspond Unigene																					
1.1.1.21(aldehyde reductase;)																						
	ABCDEF_52723	UniRef50_Q6IMN8	5e-83	Normal: 1383	764	1621	1675	2066	2197	BlastAnno: 1221	UniRef50_Q6IMN8	297	44.09	313	153	5	135	1061	2	296	5e-83	265
1.1.1.251(galactitol-1-phosphate 5-dehydrogenase)																						
	No Correspond Unigene																					
1.1.1.48(galactose 1-dehydrogenase;)																						
	ABCDEF_42111	UniRef50_C3S7Q9	0.0	Normal: 765	1778	735	895	1752	2301	BlastAnno: 1523	UniRef50_C3S7Q9	327	83.54	316	52	0	267	1214	12	327	0.0	520
1.1.3.9(galactose oxidase;)																						
	ABCDEF_3956	UniRef50_B9REU4	2e-143	Normal: 220	579	212	269	497	328	BlastAnno: 1678	UniRef50_B9REU4	533	43.75	528	269	10	1559	6	23	532	2e-143	434
2.4.1.123(inositol 3-alpha-galactosyltransferase;)																						
	No Correspond Unigene																					
2.4.1.22(lactose synthase;)																						
	No Correspond Unigene																					
2.4.1.67(galactinol---raffinose galactosyltransferase;)																						
	ABCDEF_30397	UniRef50_B9RAR3	0.0	Normal: 220	234	195	252	419	1381	BlastAnno: 2854	UniRef50_B9RAR3	685	42.50	753	347	13	254	2497	1	672	0.0	590
2.4.1.82(galactinol---sucrose galactosyltransferase;)																						

ABCDEF_30395	UniRef50_Q94A08	0.0	Normal: 217	242	202	255	409	1375	BlastAnno: 2943	UniRef50_Q94A08	773	54.50	444	189	3	269	1579	1	438	0.0	514
ABCDEF_10481	UniRef50_Q5VQG4	0.0	Normal: 43	204	220	681	70	314	BlastAnno: 1963	UniRef50_Q5VQG4	783	66.42	667	200	8	1962	1	105	760	0.0	947
ABCDEF_30397	UniRef50_Q94A08	0.0	Normal: 220	234	195	252	419	1381	BlastAnno: 2854	UniRef50_Q94A08	773	54.80	781	327	7	254	2548	1	771	0.0	907
ABCDEF_13929	UniRef50_Q94A08	0.0	Normal: 853	434	395	449	276	2979	BlastAnno: 2618	UniRef50_Q94A08	773	76.46	769	179	2	183	2486	1	768	0.0	1268
2.7.1.1(hexokinase;)																					
ABCDEF_27834	UniRef50_P93834	0.0	Normal: 998	404	1410	4185	6485	623	BlastAnno: 2374	UniRef50_P93834	502	72.77	470	128	0	2024	615	25	494	0.0	655
ABCDEF_51305	UniRef50_P93834	0.0	Normal: 443	123	280	151	521	245	BlastAnno: 2179	UniRef50_P93834	502	76.47	289	68	0	1813	947	25	313	0.0	467
ABCDEF_420	UniRef50_Q1WM16	0.0	Normal: 453	101	514	402	696	75	BlastAnno: 1554	UniRef50_Q1WM16	463	61.66	433	159	3	3	1295	20	447	0.0	538
ABCDEF_86444	UniRef50_Q1WM16	0.0	Normal: 293	76	186	81	353	207	BlastAnno: 1684	UniRef50_Q1WM16	463	66.83	404	129	3	1404	196	63	462	0.0	530
ABCDEF_592	UniRef50_P93834	0.0	Normal: 378	177	486	1038	1819	341	BlastAnno: 1859	UniRef50_P93834	502	59.87	456	180	3	1618	260	37	492	0.0	537
ABCDEF_83497	UniRef50_Q1WM16	0.0	Normal: 290	84	185	81	350	338	BlastAnno: 1860	UniRef50_Q1WM16	463	66.83	404	129	3	1404	196	63	462	0.0	529
ABCDEF_51306	UniRef50_Q1WM16	0.0	Normal: 446	118	281	150	539	247	BlastAnno: 2108	UniRef50_Q1WM16	463	66.96	451	144	3	1685	336	16	462	0.0	578
ABCDEF_593	UniRef50_P93834	0.0	Normal: 403	191	504	1097	1940	353	BlastAnno: 1902	UniRef50_P93834	502	59.87	456	180	3	1648	290	37	492	0.0	537
2.7.1.101(tagatose kinase;)																					
No Correspond Unigene																					
2.7.1.11(6-phosphofructokinase;)																					
ABCDEF_48463	UniRef50_Q94AA4	0.0	Normal: 273	212	275	545	518	593	BlastAnno: 1885	UniRef50_Q94AA4	489	71.85	437	122	1	403	1713	17	452	0.0	681
ABCDEF_48462	UniRef50_Q9FKG3	0.0	Normal: 197	165	201	395	395	441	BlastAnno: 1773	UniRef50_Q9FKG3	530	76.95	295	61	2	316	1197	44	332	0.0	469
ABCDEF_29376	UniRef50_Q94AA4	0.0	Normal: 0	44	0	0	20	222	BlastAnno: 1555	UniRef50_Q94AA4	489	81.00	458	82	3	99	1472	8	460	0.0	756
ABCDEF_49371	UniRef50_Q94AA4	0.0	Normal: 16	353	32	5	82	64	BlastAnno: 1513	UniRef50_Q94AA4	489	77.23	470	98	3	30	1421	10	476	0.0	752
ABCDEF_48301	UniRef50_Q8VYN6	0.0	Normal: 298	135	430	474	1016	200	BlastAnno: 2035	UniRef50_Q8VYN6	537	78.25	538	112	3	128	1729	1	537	0.0	844
ABCDEF_41341	UniRef50_Q94AA4	0.0	Normal: 889	655	932	1360	2142	1363	BlastAnno: 2130	UniRef50_Q94AA4	489	80.89	450	85	1	1832	483	6	454	0.0	755
2.7.1.144(tagatose-6-phosphate kinase;)																					
No Correspond Unigene																					
2.7.1.2(glucokinase;)																					
No Correspond Unigene																					
2.7.1.58(2-dehydro-3-deoxygalactonokinase;)																					
No Correspond Unigene																					
2.7.1.6(galactokinase;)																					
ABCDEF_4571	UniRef50_Q9SEE5	0.0	Normal: 1190	1054	1340	2243	4224	1615	BlastAnno: 1956	UniRef50_Q9SEE5	496	75.20	500	120	2	1688	189	1	496	0.0	775
2.7.1.69(protein-Npi-phosphohistidine---sugar phosphotransferase;)																					
No Correspond Unigene																					
2.7.7.10(UTP---hexose-1-phosphate uridylyltransferase;)																					
No Correspond Unigene																					
2.7.7.12(UDP-glucose---hexose-1-phosphate uridylyltransferase;)																					
ABCDEF_27856	UniRef50_Q9FK51	5e-135	Normal: 231	186	242	335	418	292	BlastAnno: 1338	UniRef50_Q9FK51	351	64.90	339	115	3	1298	285	15	350	5e-135	401
2.7.7.9(UTP---glucose-1-phosphate uridylyltransferase;)																					
ABCDEF_42404	UniRef50_P57751	0.0	Normal: 2120	1934	2860	5861	9792	3375	BlastAnno: 1928	UniRef50_P57751	470	84.13	460	71	2	207	1583	6	464	0.0	756
ABCDEF_68465	UniRef50_P57751	0.0	Normal: 1684	1594	2449	5480	7890	2628	BlastAnno: 1863	UniRef50_P57751	470	84.13	460	71	2	1678	302	6	464	0.0	756
3.1.1.25(1,4-lactonase;)																					
No Correspond Unigene																					
3.1.3.9(glucose-6-phosphatase;)																					
No Correspond Unigene																					
3.2.1.108(lactase;)																					
No Correspond Unigene																					
3.2.1.20(alpha-glucosidase;)																					
ABCDEF_11273	UniRef50_O04931	0.0	Normal: 3776	2509	4222	13744	13893	3618	BlastAnno: 3405	UniRef50_O04931	913	45.27	846	404	11	3289	824	41	851	0.0	750
ABCDEF_7080	UniRef50_O04931	0.0	Normal: 1183	1353	1313	1469	361	1294	BlastAnno: 3133	UniRef50_O04931	913	62.89	838	294	8	2831	354	36	868	0.0	1041
ABCDEF_11272	UniRef50_O04931	0.0	Normal: 3753	2519	4151	13616	13686	3608	BlastAnno: 3398	UniRef50_O04931	913	45.27	846	404	11	3260	795	41	851	0.0	750
ABCDEF_7166	UniRef50_O04931	0.0	Normal: 153	922	185	225	929	658	BlastAnno: 2816	UniRef50_O04931	913	60.61	820	316	3	2710	272	32	851	0.0	1028
3.2.1.22(alpha-galactosidase;)																					
ABCDEF_31352	UniRef50_Q42656	0.0	Normal: 115	169	48	7	155	1183	BlastAnno: 1530	UniRef50_Q42656	378	76.76	370	85	1	242	1351	10	378	0.0	613
ABCDEF_52628	UniRef50_Q42656	0.0	Normal: 1131	2713	1338	2471	4073	4919	BlastAnno: 1435	UniRef50_Q42656	378	65.22	368	127	1	1227	124	11	377	0.0	523
ABCDEF_10850	UniRef50_Q42656	0.0	Normal: 170	155	153	167	153	166	BlastAnno: 1524	UniRef50_Q42656	378	74.66	371	94	0	389	1501	8	378	0.0	578
3.2.1.23(beta-galactosidase;)																					
ABCDEF_48113	UniRef50_Q6Z6K4	0.0	Normal: 61	2624	32	127	673	5080	BlastAnno: 2898	UniRef50_Q6Z6K4	729	63.35	704	250	4	2505	394	33	728	0.0	931
ABCDEF_23777	UniRef50_Q6Z6K4	0.0	Normal: 219	63	227	164	235	472	BlastAnno: 2261	UniRef50_Q6Z6K4	729	66.38	690	225	5	2	2071	46	728	0.0	944
ABCDEF_3074	UniRef50_Q6Z6K4	0.0	Normal: 431	1551	321	2473	394	19728	BlastAnno: 3342	UniRef50_Q6Z6K4	729	67.09	702	221	6	415	2517	35	727	0.0	994
ABCDEF_43854	UniRef50_Q6Z6K4	0.0	Normal: 1	9	1	105	49	28	BlastAnno: 1781	UniRef50_Q6Z6K4	729	58.79	512	180	6	1	1536	147	627	0.0	595
ABCDEF_44469	UniRef50_Q8W0A1	0.0	Normal: 162	225	175	701	194	597	BlastAnno: 2319	UniRef50_Q8W0A1	827	46.10	679	324	12	2236	221	183	826	0.0	583
ABCDEF_13827	UniRef50_Q6Z6K4	0.0	Normal: 219	1054	213	1958	627	863	BlastAnno: 3038	UniRef50_Q6Z6K4	729	57.90	715	270	8	419	2539	36	727	0.0	825
ABCDEF_21624	UniRef50_Q6Z6K4	0.0	Normal: 4958	5483	4088	4956	4209	6920	BlastAnno: 3330	UniRef50_Q6Z6K4	729	62.64	696	251	4	2975	888	32	718	0.0	932
ABCDEF_43857	UniRef50_Q6Z6K4	0.0	Normal: 2	9	4	110	53	30	BlastAnno: 1655	UniRef50_Q6Z6K4	729	60.07	546	187	6	1	1638	147	661	0.0	655
ABCDEF_43861	UniRef50_Q6Z6K4	0.0	Normal: 4	15	5	162	72	47	BlastAnno: 1891	UniRef50_Q6Z6K4	729	65.21	526	179	3	107	1684	34	555	0.0	719
ABCDEF_84604	UniRef50_Q6Z6K4	0.0	Normal: 34	1696	14	81	379	2927	BlastAnno: 2528	UniRef50_Q6Z6K4	729	62.99	670	240	4	394	2403	33	694	0.0	881
ABCDEF_13826	UniRef50_Q6Z6K4	0.0	Normal: 184	889	166	1515	526	703	BlastAnno: 2897	UniRef50_Q6Z6K4	729	55.30	613	243	8	2286	472	138	727	0.0	657
ABCDEF_10626	UniRef50_Q6Z6K4	0.0	Normal: 229	909	224	1002	2471	3794	BlastAnno: 3133	UniRef50_Q6Z6K4	729	57.14	700	285	8	498	2588	40	727	0.0	809
ABCDEF_27471	UniRef50_Q6Z6K4	0.0	Normal: 992	533	1105	1489	2430	1240	BlastAnno: 3113	UniRef50_Q6Z6K4	729	64.49	704	240	5	314	2422	35	729	0.0	962
ABCDEF_43858	UniRef50_Q6Z6K4	0.0	Normal: 6	15	7	186	85	54	BlastAnno: 2028	UniRef50_Q6Z6K4	729	65.35	635	213	5	107	2011	34	661	0.0	862
ABCDEF_31373	UniRef50_Q6Z6K4	0.0	Normal: 1333	1029	1160	1241	1216	1855	BlastAnno: 3006	UniRef50_Q6Z6K4	729	52.03	713	319	7	2761	626	37	727	0.0	732
ABCDEF_10066	UniRef50_Q93Z24	0.0	Normal: 999	932	1523	1674	2007	1465	BlastAnno: 3015	UniRef50_Q93Z24	697	65.89	645	208	4	2659	737	61	697	0.0	865
ABCDEF_51808	UniRef50_Q6Z6K4	0.0	Normal: 364	454	209	36	62	1109	BlastAnno: 2817	UniRef50_Q6Z6K4	729	54.56	713	306	8	2582	447	32	727	0.0	791

ABCDEF_48111	UniRef50_Q6Z6K4	0.0	Normal: 41	1700	15	77	347	2953	BlastAnno: 2607	UniRef50_Q6Z6K4	729	67.21	494	157	3	394	1875	33	521	0.0	696
ABCDEF_17519	UniRef50_A5F5U6	0.0	Normal: 1178	1482	1392	2252	1850	3281	BlastAnno: 3706	UniRef50_A5F5U6	1024	39.71	1093	547	21	3469	254	13	1014	0.0	729
ABCDEF_40816	UniRef50_Q6Z6K4	0.0	Normal: 1166	754	898	2543	5298	532	BlastAnno: 3066	UniRef50_Q6Z6K4	729	62.55	705	254	4	2970	856	35	729	0.0	936
ABCDEF_44668	UniRef50_Q8GX69	0.0	Normal: 146	74	84	327	133	152	BlastAnno: 1396	UniRef50_Q8GX69	815	66.74	463	152	2	6	1394	23	483	0.0	671
ABCDEF_43856	UniRef50_Q6Z6K4	0.0	Normal: 12	20	12	195	92	71	BlastAnno: 1873	UniRef50_Q6Z6K4	729	61.89	488	179	5	1	1464	147	627	0.0	615
ABCDEF_44470	UniRef50_Q6Z6K4	0.0	Normal: 287	387	252	1193	287	741	BlastAnno: 2917	UniRef50_Q6Z6K4	729	52.28	723	310	9	2707	551	38	729	0.0	752
ABCDEF_49396	UniRef50_Q6Z6K4	0.0	Normal: 0	452	3	48	33	6872	BlastAnno: 2312	UniRef50_Q6Z6K4	729	65.83	676	221	7	2312	288	62	728	0.0	893
ABCDEF_10631	UniRef50_Q6Z6K4	0.0	Normal: 3079	207	2624	1970	2833	969	BlastAnno: 3058	UniRef50_Q6Z6K4	729	60.74	703	267	4	2943	835	36	729	0.0	917
ABCDEF_48112	UniRef50_Q6Z6K4	0.0	Normal: 60	2628	33	127	663	5106	BlastAnno: 2977	UniRef50_Q6Z6K4	729	67.21	494	157	3	2584	1103	33	521	0.0	696
3.2.1.26(beta-fructofuranosidase;)																					
ABCDEF_7582	UniRef50_Q5JJV0	0.0	Normal: 32	301	39	111	33	545	BlastAnno: 1949	UniRef50_Q5JJV0	590	49.44	536	263	6	1799	201	56	586	0.0	543
ABCDEF_680	UniRef50_P49175	0.0	Normal: 874	868	635	497	1076	2655	BlastAnno: 2168	UniRef50_P49175	670	64.58	576	186	9	1848	172	90	664	0.0	739
ABCDEF_52101	UniRef50_P49175	0.0	Normal: 289	386	561	2596	4447	228	BlastAnno: 1888	UniRef50_P49175	670	62.33	592	207	10	17	1756	77	664	0.0	729
ABCDEF_35900	UniRef50_P49175	0.0	Normal: 21	54	37	6735	4049	2	BlastAnno: 1966	UniRef50_P49175	670	62.85	568	195	7	1674	19	91	658	0.0	719
ABCDEF_10935	UniRef50_P49175	0.0	Normal: 81	178	91	1677	595	1048	BlastAnno: 2083	UniRef50_P49175	670	58.88	552	212	6	276	1889	114	664	0.0	663
3.2.1.85(6-phospho-beta-galactosidase;)																					
No Correspond Unigene																					
3.5.1.25(N-acetylglucosamine-6-phosphate deacetylase;)																					
No Correspond Unigene																					
4.1.2.21(2-dehydro-3-deoxy-6-phosphogalactonate aldolase;)																					
No Correspond Unigene																					
4.1.2.40(tagatose-bisphosphate aldolase;)																					
No Correspond Unigene																					
4.2.1.6(galactonate dehydratase;)																					
No Correspond Unigene																					
5.1.3.2(UDP-glucose 4-epimerase;)																					
ABCDEF_52756	UniRef50_Q42605	0.0	Normal: 448	2203	636	898	950	6504	BlastAnno: 1607	UniRef50_Q42605	351	80.87	345	66	0	1262	228	6	350	0.0	596
5.3.1.-(Isomerases;)																					
No Correspond Unigene																					
5.3.1.26(galactose-6-phosphate isomerase)																					
No Correspond Unigene																					
5.4.2.2(phosphoglucomutase;)																					
ABCDEF_935	UniRef50_P47244	0.0	Normal: 5430	4330	5743	12110	19002	6223	BlastAnno: 2144	UniRef50_P47244	572	53.66	587	246	11	2064	328	4	572	0.0	585
ABCDEF_24641	UniRef50_P47244	0.0	Normal: 416	659	414	959	874	2109	BlastAnno: 2341	UniRef50_P47244	572	54.17	576	222	11	386	2035	13	572	0.0	588
ABCDEF_24640	UniRef50_P47244	0.0	Normal: 389	596	384	834	777	1859	BlastAnno: 2422	UniRef50_P47244	572	51.74	603	222	12	386	2116	13	572	0.0	574
>00053 Ascorbate and aldarate metabolism																					
1.1.1.-(Oxidoreductases;)																					
ABCDEF_13453	UniRef50_Q9SYM5	0.0	Normal: 225	226	398	1950	2026	421	BlastAnno: 2578	UniRef50_Q9SYM5	669	86.61	672	87	2	298	2313	1	669	0.0	1226
ABCDEF_17342	UniRef50_Q84M96	0.0	Normal: 274	406	272	327	439	625	BlastAnno: 1277	UniRef50_Q84M96	346	73.33	345	91	1	1187	156	2	346	0.0	546
ABCDEF_31147	UniRef50_Q9SYM5	0.0	Normal: 4571	1591	1491	1401	1939	4327	BlastAnno: 2465	UniRef50_Q9SYM5	669	82.76	673	107	4	2397	394	1	669	0.0	1154
ABCDEF_17344	UniRef50_Q84M96	0.0	Normal: 252	373	257	297	380	540	BlastAnno: 1350	UniRef50_Q84M96	346	68.19	371	91	2	1263	154	2	346	0.0	530
ABCDEF_44674	UniRef50_Q9SYM5	0.0	Normal: 3581	2620	4329	9505	11447	4930	BlastAnno: 2634	UniRef50_Q9SYM5	669	87.80	672	78	3	253	2265	1	669	0.0	1241
1.1.1.122(D-threo-aldose 1-dehydrogenase;)																					
No Correspond Unigene																					
1.1.1.129(L-threonate 3-dehydrogenase;)																					
No Correspond Unigene																					
1.1.1.130(3-dehydro-L-gulonate 2-dehydrogenase;)																					
No Correspond Unigene																					
1.1.1.19(glucuronate reductase;)																					
No Correspond Unigene																					
1.1.1.20(glucuronolactone reductase;)																					
No Correspond Unigene																					
1.1.1.22(UDP-glucose 6-dehydrogenase;)																					
ABCDEF_30909	UniRef50_A3AK92	0.0	Normal: 865	1783	1481	3602	5414	3960	BlastAnno: 1822	UniRef50_A3AK92	438	76.89	463	77	2	235	1617	1	435	0.0	749
ABCDEF_67405	UniRef50_A3AK92	0.0	Normal: 83	280	233	170	222	547	BlastAnno: 1342	UniRef50_A3AK92	438	77.64	407	61	2	1217	3	1	379	0.0	653
ABCDEF_48744	UniRef50_A3AK92	0.0	Normal: 89	350	273	228	296	697	BlastAnno: 1407	UniRef50_A3AK92	438	77.75	409	61	2	1221	1	1	381	0.0	658
ABCDEF_4415	UniRef50_A3AK92	0.0	Normal: 493	713	643	1516	2818	758	BlastAnno: 1410	UniRef50_A3AK92	438	76.38	381	60	2	1	1137	86	438	0.0	604
ABCDEF_30910	UniRef50_A3AK92	0.0	Normal: 920	2339	1416	3594	6463	6266	BlastAnno: 1816	UniRef50_A3AK92	438	77.54	463	74	2	1591	209	1	435	0.0	751
ABCDEF_30908	UniRef50_A3AK92	0.0	Normal: 2277	2525	3512	8495	12648	3403	BlastAnno: 1908	UniRef50_A3AK92	438	77.25	466	76	2	235	1626	1	438	0.0	755
ABCDEF_73000	UniRef50_Q9AUV6	0.0	Normal: 307	765	498	1248	2232	1778	BlastAnno: 905	UniRef50_Q9AUV6	480	90.37	301	29	0	904	2	95	395	0.0	572
ABCDEF_84803	UniRef50_A3AK92	0.0	Normal: 78	278	221	166	230	569	BlastAnno: 1468	UniRef50_A3AK92	438	77.70	408	61	2	1220	3	1	380	0.0	656
1.1.1.46(L-arabinose 1-dehydrogenase)																					
No Correspond Unigene																					
1.1.3.8(L-gulonolactone oxidase;)																					
ABCDEF_56600	UniRef50_P58710	4e-36	Normal: 139	110	184	219	208	252	BlastAnno: 1086	UniRef50_P58710	440	32.88	219	147	0	989	333	12	230	4e-36	143
1.10.3.3(L-ascorbate oxidase;)																					
ABCDEF_7743	UniRef50_B9SBE5	0.0	Normal: 14	19	3	205	474	195	BlastAnno: 1988	UniRef50_B9SBE5	572	50.45	555	250	11	199	1836	34	572	0.0	547
ABCDEF_44873	UniRef50_B9SBE5	0.0	Normal: 86	790	106	961	1696	540	BlastAnno: 2093	UniRef50_B9SBE5	572	48.90	544	261	5	1930	332	35	572	0.0	542
1.11.1.11(L-ascorbate peroxidase;)																					
ABCDEF_51654	UniRef50_Q42593	0.0	Normal: 451	646	708	1139	1717	2343	BlastAnno: 1665	UniRef50_Q42593	426	78.83	326	61	2	1467	490	50	367	0.0	527
ABCDEF_51655	UniRef50_Q42593	0.0	Normal: 461	726	655	1038	1365	2645	BlastAnno: 1522	UniRef50_Q42593	426	78.67	375	72	2	1324	200	50	416	0.0	609
1.13.11.13(ascorbate 2,3-dioxygenase;)																					

No Correspond Unigene																					
1.13.99.1(inositol oxygenase;)																					
ABCDEF_32645	UniRef50_Q8H1S0	7e-162	Normal: 1	74	0	0	0	1292	BlastAnno: 1181	UniRef50_Q8H1S0	317	74.38	320	77	3	84	1037	1	317	7e-162	466
1.14.-.-(Oxidoreductases;)																					
ABCDEF_44739	UniRef50_O64989	0.0	Normal: 157	87	159	718	673	189	BlastAnno: 2185	UniRef50_O64989	513	70.75	482	112	3	1867	509	30	511	0.0	706
ABCDEF_31491	UniRef50_Q93VK5	0.0	Normal: 647	1826	671	1517	1770	5953	BlastAnno: 2278	UniRef50_Q93VK5	595	79.14	556	113	2	198	1862	35	588	0.0	902
ABCDEF_40554	UniRef50_P37122	0.0	Normal: 1574	1962	1829	1150	2882	2721	BlastAnno: 1828	UniRef50_P37122	505	60.52	504	198	1	1752	244	1	504	0.0	638
ABCDEF_34049	UniRef50_O23365	0.0	Normal: 363	384	349	442	549	1416	BlastAnno: 2101	UniRef50_O23365	580	76.17	579	116	5	230	1957	19	578	0.0	904
ABCDEF_21579	UniRef50_O48928	0.0	Normal: 1005	643	1606	4372	3226	1621	BlastAnno: 1895	UniRef50_O48928	513	69.72	502	151	1	185	1690	7	507	0.0	731
ABCDEF_37741	UniRef50_P37122	0.0	Normal: 12	760	9	24	34	576	BlastAnno: 1197	UniRef50_P37122	505	65.70	379	129	1	1195	59	127	504	0.0	526
ABCDEF_35618	UniRef50_O48928	0.0	Normal: 28	2648	17	142	1234	902	BlastAnno: 1746	UniRef50_O48928	513	66.40	506	168	2	1649	132	4	507	0.0	701
ABCDEF_24522	UniRef50_P37122	0.0	Normal: 334	2190	561	268	1391	2459	BlastAnno: 2075	UniRef50_P37122	505	61.52	460	176	1	1544	165	47	505	0.0	612
ABCDEF_14592	UniRef50_O23066	0.0	Normal: 1514	402	2245	7837	4742	1248	BlastAnno: 2378	UniRef50_O23066	553	72.71	546	140	4	2111	489	1	542	0.0	821
ABCDEF_14512	UniRef50_Q9FG65	0.0	Normal: 261	872	212	291	175	789	BlastAnno: 1878	UniRef50_Q9FG65	502	56.89	501	197	9	1738	266	11	502	0.0	532
ABCDEF_44487	UniRef50_Q940V4	0.0	Normal: 3	41	53	501	262	542	BlastAnno: 1712	UniRef50_Q940V4	465	69.37	382	116	1	38	1180	14	395	0.0	572
ABCDEF_17723	UniRef50_P37122	0.0	Normal: 240	1698	219	768	592	794	BlastAnno: 1757	UniRef50_P37122	505	63.40	459	166	2	228	1601	47	504	0.0	617
ABCDEF_37901	UniRef50_Q42569	0.0	Normal: 30	149	86	255	195	255	BlastAnno: 1246	UniRef50_Q42569	472	78.27	359	73	2	64	1140	43	396	0.0	567
ABCDEF_7260	UniRef50_O81117	0.0	Normal: 314	1085	466	3248	2575	1360	BlastAnno: 1768	UniRef50_O81117	514	59.92	474	188	1	145	1566	43	514	0.0	615
ABCDEF_40548	UniRef50_P37122	0.0	Normal: 2756	2467	2752	1072	3120	1735	BlastAnno: 1947	UniRef50_P37122	505	59.72	504	202	1	241	1749	1	504	0.0	636
ABCDEF_44485	UniRef50_Q940V4	0.0	Normal: 3	55	63	574	338	660	BlastAnno: 1607	UniRef50_Q940V4	465	68.58	452	141	1	42	1394	14	465	0.0	673
ABCDEF_40552	UniRef50_P37122	0.0	Normal: 2397	1870	2304	1249	10428	2291	BlastAnno: 1812	UniRef50_P37122	505	60.12	504	200	1	108	1616	1	504	0.0	640
ABCDEF_37902	UniRef50_Q42569	0.0	Normal: 84	524	254	725	670	1049	BlastAnno: 1812	UniRef50_Q42569	472	77.47	435	92	3	1675	371	43	471	0.0	681
ABCDEF_40556	UniRef50_P37122	0.0	Normal: 1907	2365	2163	1319	3678	3308	BlastAnno: 1944	UniRef50_P37122	505	61.04	498	193	1	264	1754	1	498	0.0	635
ABCDEF_40549	UniRef50_P37122	0.0	Normal: 1574	1962	1826	1150	2882	2723	BlastAnno: 1845	UniRef50_P37122	505	60.52	504	198	1	1754	246	1	504	0.0	638
ABCDEF_20301	UniRef50_O81117	0.0	Normal: 89	308	123	176	389	249	BlastAnno: 1637	UniRef50_O81117	514	64.04	470	164	3	1498	98	47	514	0.0	586
ABCDEF_3882	UniRef50_Q42569	0.0	Normal: 84	243	257	786	1561	182	BlastAnno: 1854	UniRef50_Q42569	472	72.51	422	107	1	1852	587	59	471	0.0	615
ABCDEF_20703	UniRef50_O49858	0.0	Normal: 115	636	20	0	0	597	BlastAnno: 1815	UniRef50_O49858	527	50.10	523	248	6	48	1607	12	524	0.0	533
ABCDEF_18295	UniRef50_O22203	0.0	Normal: 3667	1934	4977	10491	4588	1917	BlastAnno: 2006	UniRef50_O22203	508	74.85	493	123	1	214	1692	17	508	0.0	803
ABCDEF_48724	UniRef50_O23066	0.0	Normal: 104	11518	163	2083	3464	6356	BlastAnno: 2162	UniRef50_O23066	553	72.96	540	143	3	227	1840	1	539	0.0	807
ABCDEF_37738	UniRef50_P37122	0.0	Normal: 26	2447	19	30	33	993	BlastAnno: 1837	UniRef50_P37122	505	64.30	479	169	2	1685	249	28	504	0.0	639
1.2.1.26(2,5-dioxovalerate dehydrogenase;)																					
No Correspond Unigene																					
1.2.1.3(aldehyde dehydrogenase (NAD+);)																					
ABCDEF_52483	UniRef50_Q70E96	0.0	Normal: 28	851	41	0	3965	420	BlastAnno: 1750	UniRef50_Q70E96	484	54.56	482	219	0	107	1552	3	484	0.0	548
ABCDEF_17638	UniRef50_Q70E96	0.0	Normal: 580	718	749	2424	1317	775	BlastAnno: 1970	UniRef50_Q70E96	484	62.21	471	177	1	283	1692	2	472	0.0	625
ABCDEF_403	UniRef50_Q8W033	0.0	Normal: 69	355	98	39	223	1111	BlastAnno: 1749	UniRef50_Q8W033	550	65.96	473	161	0	1523	105	74	546	0.0	625
ABCDEF_45143	UniRef50_Q0WSF1	0.0	Normal: 1445	874	1200	1789	1940	1504	BlastAnno: 2450	UniRef50_Q0WSF1	596	79.97	594	115	2	2220	451	1	594	0.0	1015
1.3.2.3(L-galactonolactone dehydrogenase;)																					
ABCDEF_48138	UniRef50_Q9SU56	0.0	Normal: 525	401	672	891	840	1100	BlastAnno: 2231	UniRef50_Q9SU56	610	80.48	543	105	1	1883	255	62	603	0.0	904
ABCDEF_56600	UniRef50_Q9SU56	0.0	Normal: 139	110	184	219	208	252	BlastAnno: 1086	UniRef50_Q9SU56	610	78.49	358	69	2	1055	3	85	441	0.0	552
1.3.3.12(L-galactonolactone oxidase;)																					
No Correspond Unigene																					
1.6.5.4(monodehydroascorbate reductase (NADH);)																					
ABCDEF_38434	UniRef50_Q9SR59	0.0	Normal: 5380	4983	7435	18930	15768	7886	BlastAnno: 1832	UniRef50_Q9SR59	441	67.36	432	140	1	1585	293	4	435	0.0	550
ABCDEF_25070	UniRef50_P92947	0.0	Normal: 696	605	917	1103	1696	1284	BlastAnno: 1850	UniRef50_P92947	493	77.89	493	103	2	186	1652	1	491	0.0	759
ABCDEF_31516	UniRef50_Q9SR59	0.0	Normal: 683	308	768	1214	1611	631	BlastAnno: 1753	UniRef50_Q9SR59	441	66.28	433	145	1	1542	247	4	436	0.0	553
1.8.5.1(glutathione dehydrogenase (ascorbate);)																					
No Correspond Unigene																					
2.4.1.17(glucuronosyltransferase;)																					
No Correspond Unigene																					
2.7.1.43(glucuronokinase;)																					
No Correspond Unigene																					
2.7.1.53(L-xylulokinase;)																					
No Correspond Unigene																					
2.7.1.69(protein-Npi-phosphohistidine---sugar phosphotransferase;)																					
No Correspond Unigene																					
2.7.7.44(glucuronate-1-phosphate uridylyltransferase;)																					
No Correspond Unigene																					
2.7.7.69(GDP-L-galactose phosphorylase;)																					
No Correspond Unigene																					
3.1.1.-(Hydrolases;)																					
ABCDEF_25737	UniRef50_Q9CA68	0.0	Normal: 104	11	8	393	426	18	BlastAnno: 1232	UniRef50_Q9CA68	366	69.89	352	103	2	1182	130	17	366	0.0	522
ABCDEF_525	UniRef50_Q9LIN2	0.0	Normal: 213	16	167	378	456	158	BlastAnno: 1254	UniRef50_Q9LIN2	380	69.27	358	110	0	1097	24	23	380	0.0	535
ABCDEF_28091	UniRef50_O23522	0.0	Normal: 1192	678	424	81	156	3170	BlastAnno: 1887	UniRef50_O23522	601	57.90	525	191	12	168	1721	3	504	0.0	568
ABCDEF_13432	UniRef50_Q3ECP6	0.0	Normal: 712	1725	466	588	811	6988	BlastAnno: 2479	UniRef50_Q3ECP6	408	66.83	401	104	2	2397	1282	8	408	0.0	567
ABCDEF_72624	UniRef50_Q3ECP6	0.0	Normal: 22	38	25	267	382	149	BlastAnno: 1342	UniRef50_Q3ECP6	408	66.83	401	104	2	53	1168	8	408	0.0	567
ABCDEF_4432	UniRef50_B9IQA0	0.0	Normal: 680	298	798	834	1447	644	BlastAnno: 1533	UniRef50_B9IQA0	354	78.82	321	68	0	214	1176	33	353	0.0	556
3.1.1.15(L-arabinonolactonase)																					
No Correspond Unigene																					
3.1.1.17(gluconolactonase;)																					
ABCDEF_17917	UniRef50_A1TXZ0	5e-64	Normal: 303	575	152	163	236	2055	BlastAnno: 1384	UniRef50_A1TXZ0	363	36.89	347	200	6	1216	200	23	358	5e-64	219
3.1.1.19(uronolactonase;)																					

No Correspond Unigene																					
3.1.1.25(1,4-lactonase;)																					
No Correspond Unigene																					
3.1.3.-(Hydrolases;)																					
ABCDEF_3128	UniRef50_Q9ZU49	5e-117	Normal: 118	143	108	91	149	56	BlastAnno: 1434	UniRef50_Q9ZU49	327	57.43	303	121	2	1184	294	20	320	5e-117	355
3.2.1.-(Hydrolases;)																					
ABCDEF_55518	UniRef50_Q9SGZ5	0.0	Normal: 137	145	110	155	249	211	BlastAnno: 1222	UniRef50_Q9SGZ5	767	66.34	407	136	1	1218	1	142	548	0.0	556
ABCDEF_45209	UniRef50_Q9LXA8	0.0	Normal: 1338	811	1191	1817	1167	1821	BlastAnno: 2643	UniRef50_Q9LXA8	792	47.09	790	379	11	2448	178	7	790	0.0	745
ABCDEF_34153	UniRef50_Q9LXA8	0.0	Normal: 522	668	469	700	564	936	BlastAnno: 2528	UniRef50_Q9LXA8	792	51.44	762	345	6	2397	175	30	787	0.0	804
ABCDEF_34154	UniRef50_Q9LXA8	0.0	Normal: 495	622	454	650	534	930	BlastAnno: 2619	UniRef50_Q9LXA8	792	50.53	655	301	5	2076	175	135	787	0.0	679
ABCDEF_47469	UniRef50_Q9SXC9	0.0	Normal: 80	59	95	141	105	115	BlastAnno: 1550	UniRef50_Q9SXC9	574	81.45	248	46	0	3	746	93	340	0.0	419
ABCDEF_3050	UniRef50_A8JBU2	0.0	Normal: 365	517	518	1626	1809	451	BlastAnno: 2998	UniRef50_A8JBU2	725	52.75	673	264	8	2589	604	50	679	0.0	670
ABCDEF_3049	UniRef50_A8JBU2	0.0	Normal: 528	690	724	2218	2620	674	BlastAnno: 3077	UniRef50_A8JBU2	725	52.32	711	286	7	2650	551	55	723	0.0	707
ABCDEF_34152	UniRef50_Q9LXA8	0.0	Normal: 568	815	532	816	648	1135	BlastAnno: 2581	UniRef50_Q9LXA8	792	51.24	765	348	6	2450	219	30	790	0.0	806
ABCDEF_34155	UniRef50_Q9LXA8	0.0	Normal: 543	763	515	772	621	1127	BlastAnno: 2640	UniRef50_Q9LXA8	792	50.30	658	304	5	2112	202	135	790	0.0	681
ABCDEF_21385	UniRef50_Q9LXA8	0.0	Normal: 432	334	511	873	734	380	BlastAnno: 2109	UniRef50_Q9LXA8	792	62.64	629	229	4	3	1877	152	778	0.0	775
ABCDEF_980	UniRef50_Q9LXA8	0.0	Normal: 1398	7037	1070	4974	7681	6612	BlastAnno: 2765	UniRef50_Q9LXA8	792	49.22	772	351	9	257	2470	28	792	0.0	753
ABCDEF_31427	UniRef50_Q9LXA8	0.0	Normal: 56	316	89	4893	4124	2869	BlastAnno: 2823	UniRef50_Q9LXA8	792	48.25	773	359	12	209	2425	26	791	0.0	716
ABCDEF_27305	UniRef50_Q9FG93	0.0	Normal: 247	143	251	353	378	254	BlastAnno: 1883	UniRef50_Q9FG93	624	70.43	487	123	3	2	1456	126	593	0.0	702
ABCDEF_47466	UniRef50_Q9SXC9	0.0	Normal: 69	48	83	133	95	97	BlastAnno: 1452	UniRef50_Q9SXC9	574	75.84	476	115	0	2	1429	96	571	0.0	769
ABCDEF_3403	UniRef50_Q9LXA8	0.0	Normal: 206	82	182	114	358	291	BlastAnno: 2202	UniRef50_Q9LXA8	792	55.15	515	213	4	2198	702	84	596	0.0	548
3.7.1.-(Hydrolases;)																					
No Correspond Unigene																					
4.1.1.-(Lyases;)																					
ABCDEF_57496	UniRef50_O96569	9e-20	Normal: 4	0	0	0	0	9	BlastAnno: 223	UniRef50_O96569	439	66.67	72	22	1	210	1	19	90	9e-20	87.4
4.1.1.85(3-dehydro-L-gulonate-6-phosphate decarboxylase;)																					
No Correspond Unigene																					
4.1.2.18(2-dehydro-3-deoxy-L-pentonate aldolase;)																					
No Correspond Unigene																					
4.1.2.20(2-dehydro-3-deoxyglucarate aldolase;)																					
ABCDEF_38428	UniRef50_B2U092	3e-36	Normal: 551	427	606	695	1516	1338	BlastAnno: 1495	UniRef50_B2U092	257	31.91	257	166	4	325	1092	8	256	3e-36	141
4.2.1.25(L-arabinonate dehydratase;)																					
No Correspond Unigene																					
4.2.1.40(glucarate dehydratase;)																					
No Correspond Unigene																					
4.2.1.41(5-dehydro-4-deoxyglucarate dehydratase;)																					
No Correspond Unigene																					
4.2.1.42(galactarate dehydratase;)																					
No Correspond Unigene																					
4.2.1.43(2-dehydro-3-deoxy-L-arabinonate dehydratase;)																					
No Correspond Unigene																					
5.1.3.18(GDP-mannose 3,5-epimerase;)																					
ABCDEF_4728	UniRef50_Q93VR3	0.0	Normal: 3786	7995	2857	3393	6154	12151	BlastAnno: 1535	UniRef50_Q93VR3	377	92.04	377	29	1	161	1288	1	377	0.0	735
5.1.3.22(L-ribulose-5-phosphate 3-epimerase;)																					
No Correspond Unigene																					
5.1.3.4(L-ribulose-5-phosphate 4-epimerase;)																					
No Correspond Unigene																					
>00061 Fatty acid biosynthesis																					
1.1.1.100(3-oxoacyl-[acyl-carrier-protein] reductase;)																					
ABCDEF_52950	UniRef50_P33207	1e-122	Normal: 2328	1244	2832	8339	5274	3496	BlastAnno: 1484	UniRef50_P33207	319	69.44	301	91	1	1206	304	20	319	1e-122	370
1.14.19.2(acyl-[acyl-carrier-protein] desaturase;)																					
ABCDEF_45228	UniRef50_Q40731	0.0	Normal: 698	220	747	804	913	429	BlastAnno: 1618	UniRef50_Q40731	390	81.84	358	65	0	1332	259	31	388	0.0	610
ABCDEF_31580	UniRef50_Q40731	0.0	Normal: 1231	1997	848	595	968	3576	BlastAnno: 1749	UniRef50_Q40731	390	78.15	389	79	2	1463	303	6	390	0.0	644
ABCDEF_25662	UniRef50_Q40731	0.0	Normal: 768	828	1036	1708	2315	1366	BlastAnno: 1760	UniRef50_Q40731	390	82.13	347	62	0	365	1405	42	388	0.0	617
1.3.1.-(Oxidoreductases;)																					
ABCDEF_45552	UniRef50_P52581	0.0	Normal: 213	2112	262	1058	1208	1322	BlastAnno: 1284	UniRef50_P52581	312	80.45	312	61	0	185	1120	1	312	0.0	530
ABCDEF_47970	UniRef50_Q9T0J6	0.0	Normal: 1040	443	1171	941	1234	650	BlastAnno: 2557	UniRef50_Q9T0J6	700	60.52	699	232	9	2337	271	36	700	0.0	860
1.3.1.9(enoyl-[acyl-carrier-protein] reductase (NADH);)																					
ABCDEF_45322	UniRef50_O04942	0.0	Normal: 597	353	642	576	790	2067	BlastAnno: 1624	UniRef50_O04942	390	73.52	389	95	3	197	1345	1	387	0.0	531
2.3.1.-(Transferases;)																					
ABCDEF_520	UniRef50_Q9FF86	0.0	Normal: 1672	85	2273	5056	3383	1211	BlastAnno: 1811	UniRef50_Q9FF86	484	64.06	448	149	6	1664	354	3	449	0.0	573
ABCDEF_68664	UniRef50_Q9ZVN2	0.0	Normal: 79	35	85	107	193	63	BlastAnno: 1488	UniRef50_Q9ZVN2	704	63.99	411	142	3	46	1275	298	703	0.0	540
ABCDEF_52245	UniRef50_Q9FG87	0.0	Normal: 1878	635	1833	3814	1432	1376	BlastAnno: 1758	UniRef50_Q9FG87	529	55.35	486	204	4	4	1428	34	517	0.0	530
ABCDEF_16730	UniRef50_Q3EC11	0.0	Normal: 281	204	237	365	377	458	BlastAnno: 2092	UniRef50_Q3EC11	536	59.33	536	204	8	196	1785	3	530	0.0	661
ABCDEF_4655	UniRef50_A5BMG1	0.0	Normal: 2426	1856	3606	6958	4608	2755	BlastAnno: 2261	UniRef50_A5BMG1	481	68.37	528	112	10	263	1843	3	476	0.0	667
ABCDEF_13860	UniRef50_Q9C533	0.0	Normal: 588	220	570	1451	1338	310	BlastAnno: 2648	UniRef50_Q9C533	596	63.37	606	177	12	2226	511	1	595	0.0	558
ABCDEF_28294	UniRef50_Q9SYZ0	0.0	Normal: 1038	4070	1427	6745	877	2797	BlastAnno: 1785	UniRef50_Q9SYZ0	493	52.37	485	223	3	1598	162	5	487	0.0	533
ABCDEF_47718	UniRef50_Q9C533	0.0	Normal: 1599	236	1657	2093	1231	588	BlastAnno: 2680	UniRef50_Q9C533	596	65.02	626	165	14	2280	472	1	595	0.0	634
ABCDEF_45080	UniRef50_Q9SYZ0	0.0	Normal: 1005	592	1079	2360	3762	2217	BlastAnno: 2130	UniRef50_Q9SYZ0	493	57.85	484	197	3	302	1735	5	487	0.0	533
ABCDEF_25377	UniRef50_Q9C6L5	0.0	Normal: 37	2090	138	2573	1026	1298	BlastAnno: 1334	UniRef50_Q9C6L5	492	79.74	385	76	1	1155	1	6	388	0.0	654
ABCDEF_33470	UniRef50_Q9LW26	0.0	Normal: 172	98	156	260	391	149	BlastAnno: 2993	UniRef50_Q9LW26	701	48.96	627	301	5	655	2508	85	701	0.0	581
ABCDEF_40654	UniRef50_Q9H0A0	0.0	Normal: 638	213	530	318	318	286	BlastAnno: 2216	UniRef50_Q9H0A0	1025	56.87	721	279	6	2127	13	91	795	0.0	756

ABCDEF_33460	UniRef50_Q9ZVN2	0.0	Normal: 144	71	119	216	306	97	BlastAnno: 2691	UniRef50_Q9ZVN2	704	61.50	587	191	4	379	2133	83	636	0.0	715
ABCDEF_14442	UniRef50_Q9SYZ0	0.0	Normal: 341	684	439	1040	1601	552	BlastAnno: 1779	UniRef50_Q9SYZ0	493	63.12	480	171	2	1570	149	5	484	0.0	628
ABCDEF_52626	UniRef50_B6SYP9	0.0	Normal: 815	346	1131	3015	1537	108	BlastAnno: 1861	UniRef50_B6SYP9	494	55.02	438	189	3	1538	249	51	488	0.0	536
ABCDEF_644	UniRef50_Q9SB58	0.0	Normal: 704	444	913	969	1431	848	BlastAnno: 1777	UniRef50_Q9SB58	407	64.15	424	129	8	1572	316	1	406	0.0	554
ABCDEF_27087	UniRef50_Q52T38	0.0	Normal: 324	221	233	419	343	412	BlastAnno: 1631	UniRef50_Q52T38	620	75.95	420	91	1	1409	150	1	410	0.0	681
ABCDEF_34187	UniRef50_Q8L5Y5	0.0	Normal: 497	415	588	523	291	433	BlastAnno: 2677	UniRef50_Q8L5Y5	718	57.30	733	251	15	202	2274	1	713	0.0	683
ABCDEF_47966	UniRef50_Q9SYZ0	0.0	Normal: 577	934	326	580	682	3440	BlastAnno: 2177	UniRef50_Q9SYZ0	493	56.40	484	204	3	1780	347	5	487	0.0	535
ABCDEF_51189	UniRef50_Q52T38	0.0	Normal: 135	152	128	181	219	327	BlastAnno: 1766	UniRef50_Q52T38	620	68.09	470	137	3	80	1483	130	588	0.0	641
ABCDEF_41288	UniRef50_Q9SB58	0.0	Normal: 307	183	332	637	649	322	BlastAnno: 1403	UniRef50_Q9SB58	407	67.56	410	110	6	1401	184	15	405	0.0	523
ABCDEF_33461	UniRef50_Q9ZVN2	0.0	Normal: 142	84	140	206	283	119	BlastAnno: 2904	UniRef50_Q9ZVN2	704	60.98	574	201	6	2271	559	83	636	0.0	686
ABCDEF_14334	UniRef50_Q9SYZ0	0.0	Normal: 2263	129	1724	3469	866	283	BlastAnno: 2039	UniRef50_Q9SYZ0	493	63.22	484	171	2	369	1802	5	487	0.0	627
ABCDEF_24844	UniRef50_Q9FF86	0.0	Normal: 1857	3686	2657	21940	11467	4090	BlastAnno: 1640	UniRef50_Q9FF86	484	66.89	447	135	6	1492	185	2	446	0.0	575
ABCDEF_51190	UniRef50_Q52T38	0.0	Normal: 145	169	142	210	232	335	BlastAnno: 1891	UniRef50_Q52T38	620	70.04	514	141	3	73	1608	86	588	0.0	726
ABCDEF_14085	UniRef50_Q8L5Y5	0.0	Normal: 353	41	384	527	331	280	BlastAnno: 2362	UniRef50_Q8L5Y5	718	62.07	704	220	12	2360	348	24	713	0.0	676
ABCDEF_33458	UniRef50_Q9LW26	0.0	Normal: 158	78	130	240	342	106	BlastAnno: 2822	UniRef50_Q9LW26	701	46.72	655	302	5	400	2337	85	701	0.0	568
ABCDEF_25193	UniRef50_Q9C6L5	0.0	Normal: 3256	368	3548	6230	5109	1288	BlastAnno: 1786	UniRef50_Q9C6L5	492	65.83	480	156	4	1668	241	17	492	0.0	668
ABCDEF_33457	UniRef50_Q9ZVN2	0.0	Normal: 168	83	137	250	375	112	BlastAnno: 2823	UniRef50_Q9ZVN2	704	65.12	496	168	3	379	1860	83	575	0.0	648
ABCDEF_7634	UniRef50_Q9C6L5	0.0	Normal: 2543	9303	2711	11578	5533	10032	BlastAnno: 2064	UniRef50_Q9C6L5	492	52.76	489	225	3	1734	268	1	483	0.0	535
ABCDEF_299	UniRef50_Q71N54	0.0	Normal: 569	1147	511	900	649	1879	BlastAnno: 2017	UniRef50_Q71N54	535	61.73	533	198	5	338	1924	4	534	0.0	677
ABCDEF_27086	UniRef50_Q52T38	0.0	Normal: 784	470	617	843	893	976	BlastAnno: 2398	UniRef50_Q52T38	620	70.80	637	165	4	225	2129	1	618	0.0	887
ABCDEF_51188	UniRef50_Q52T38	0.0	Normal: 273	327	255	415	349	441	BlastAnno: 2324	UniRef50_Q52T38	620	71.30	568	150	3	2041	344	32	588	0.0	815
ABCDEF_44812	UniRef50_Q6DR03	0.0	Normal: 982	902	1005	1071	959	2999	BlastAnno: 2450	UniRef50_Q6DR03	565	54.97	644	198	17	2142	220	1	555	0.0	540
2.3.1.179(beta-ketoacyl-acyl-carrier-protein synthase II;)																					
ABCDEF_35253	UniRef50_Q255V3	4e-160	Normal: 2507	1242	2168	3895	4234	5389	BlastAnno: 1928	UniRef50_Q255V3	416	57.69	416	172	4	1660	419	3	416	4e-160	475
2.3.1.180(beta-ketoacyl-acyl-carrier-protein synthase III;)																					
ABCDEF_18080	UniRef50_P49243	0.0	Normal: 492	303	581	808	1050	765	BlastAnno: 1786	UniRef50_P49243	404	70.99	393	100	4	1439	270	22	403	0.0	565
2.3.1.39(No Name)																					
ABCDEF_1295	UniRef50_Q7UYY3	4e-70	Normal: 1510	772	1882	3063	2892	1761	BlastAnno: 1510	UniRef50_Q7UYY3	309	46.28	296	151	4	1121	240	9	298	4e-70	234
2.3.1.41(beta-ketoacyl-acyl-carrier-protein synthase I;)																					
ABCDEF_34393	UniRef50_Q8L3X9	0.0	Normal: 484	120	407	363	395	323	BlastAnno: 1765	UniRef50_Q8L3X9	461	76.25	421	97	1	1433	171	44	461	0.0	630
ABCDEF_43573	UniRef50_P23902	0.0	Normal: 205	6	33	36	48	16	BlastAnno: 1467	UniRef50_P23902	462	75.50	400	96	1	1465	272	63	462	0.0	604
ABCDEF_35253	UniRef50_P23902	0.0	Normal: 2507	1242	2168	3895	4234	5389	BlastAnno: 1928	UniRef50_P23902	462	83.92	423	66	1	1681	419	40	462	0.0	746
2.3.1.85(fatty-acid synthase;)																					
ABCDEF_83274	UniRef50_P49327	1e-37	Normal: 0	0	0	0	0	12	BlastAnno: 299	UniRef50_P49327	2511	60.20	98	39	0	298	5	1976	2073	1e-37	142
2.3.1.86(fatty-acyl-CoA synthase;)																					
No Correspond Unigene																					
3.1.2.-(Hydrolases;)																					
ABCDEF_2988	UniRef50_Q39513	0.0	Normal: 684	868	676	1224	1298	1392	BlastAnno: 1728	UniRef50_Q39513	415	69.13	392	111	5	408	1574	12	396	0.0	554
ABCDEF_42638	UniRef50_Q9SHJ8	0.0	Normal: 183	172	331	565	390	214	BlastAnno: 1547	UniRef50_Q9SHJ8	395	68.62	376	94	2	196	1320	42	394	0.0	533
ABCDEF_56804	UniRef50_Q9SHJ8	0.0	Normal: 160	146	249	472	328	155	BlastAnno: 1439	UniRef50_Q9SHJ8	395	68.62	376	94	2	275	1399	42	394	0.0	533
ABCDEF_2987	UniRef50_Q39513	0.0	Normal: 608	561	472	1162	1435	954	BlastAnno: 1938	UniRef50_Q39513	415	67.26	394	119	5	408	1580	12	398	0.0	544
ABCDEF_20456	UniRef50_Q9LK08	0.0	Normal: 375	366	460	796	1014	454	BlastAnno: 1539	UniRef50_Q9LK08	418	64.52	420	140	3	1434	187	4	418	0.0	559
ABCDEF_49725	UniRef50_Q9SHJ8	0.0	Normal: 176	174	327	582	397	195	BlastAnno: 1556	UniRef50_Q9SHJ8	395	68.62	376	94	2	234	1358	42	394	0.0	533
ABCDEF_20453	UniRef50_Q9LK08	0.0	Normal: 396	358	461	824	1019	461	BlastAnno: 1565	UniRef50_Q9LK08	418	66.90	420	134	2	1459	200	4	418	0.0	586
3.1.2.14(oleoyl-[acyl-carrier-protein] hydrolase;)																					
ABCDEF_50485	UniRef50_Q42712	6e-170	Normal: 130	293	117	147	130	775	BlastAnno: 1757	UniRef50_Q42712	369	78.22	303	61	3	246	1145	65	365	6e-170	496
3.1.2.21(dodecanoyl-[acyl-carrier-protein] hydrolase;)																					
No Correspond Unigene																					
4.2.1.-(Lyases;)																					
ABCDEF_48679	UniRef50_Q0RP29	5e-115	Normal: 568	325	563	941	1285	676	BlastAnno: 1424	UniRef50_Q0RP29	493	56.39	321	129	4	119	1075	39	350	5e-115	356
4.2.1.58(crotonoyl-[acyl-carrier-protein] hydratase;)																					
No Correspond Unigene																					
4.2.1.60(3-hydroxydecanoyl-[acyl-carrier-protein] dehydratase;)																					
No Correspond Unigene																					
4.2.1.61(3-hydroxypalmitoyl-[acyl-carrier-protein] dehydratase;)																					
No Correspond Unigene																					
6.3.4.14(biotin carboxylase;)																					
ABCDEF_45829	UniRef50_A5GNN9	0.0	Normal: 2364	1703	2036	5443	4075	6768	BlastAnno: 2081	UniRef50_A5GNN9	447	64.85	441	155	0	393	1715	4	444	0.0	587
6.4.1.2(acetyl-CoA carboxylase;)																					
ABCDEF_27465	UniRef50_P78820	0.0	Normal: 4589	3602	6382	22310	7761	4482	BlastAnno: 7477	UniRef50_P78820	2280	40.33	2145	1127	43	7128	919	55	2121	0.0	1462
>00062 Fatty acid elongation in mitochondria																					
1.1.1.211(long-chain-3-hydroxyacyl-CoA dehydrogenase;)																					
ABCDEF_86264	UniRef50_Q9V397	1e-32	Normal: 0	0	0	0	0	18	BlastAnno: 294	UniRef50_Q9V397	783	63.27	98	34	2	1	291	647	743	1e-32	126
1.1.1.35(3-hydroxyacyl-CoA dehydrogenase;)																					
ABCDEF_80649	UniRef50_Q1LD07	5e-120	Normal: 527	841	443	1308	1321	1188	BlastAnno: 2586	UniRef50_Q1LD07	715	37.34	699	420	9	2358	277	22	707	5e-120	389
1.3.1.38(trans-2-enoyl-CoA reductase (NADPH);)																					
ABCDEF_48133	UniRef50_Q8LCU7	0.0	Normal: 231	152	277	172	241	384	BlastAnno: 1636	UniRef50_Q8LCU7	375	76.42	352	83	0	1446	391	24	375	0.0	574
1.3.1.8(acyl-CoA dehydrogenase (NADP+);)																					
No Correspond Unigene																					
2.3.1.16(acetyl-CoA C-acyltransferase;)																					
ABCDEF_47228	UniRef50_P09110	1e-153	Normal: 321	497	306	408	1144	369	BlastAnno: 1559	UniRef50_P09110	424	59.95	387	152	3	1397	237	37	420	1e-153	454
3.1.2.22(palmitoyl[protein] hydrolase;)																					

ABCDEF_17235	UniRef50_B7P807	2e-49	Normal: 331	120	191	300	245	314	BlastAnno: 1446	UniRef50_B7P807	330	36.00	275	163	5	1167	373	58	329	2e-49	179
4.2.1.17(enoyl-CoA hydratase;)																					
ABCDEF_21259	UniRef50_Q8W1L6	0.0	Normal: 272	276	284	384	326	305	BlastAnno: 2641	UniRef50_Q8W1L6	726	47.57	721	375	3	114	2267	6	726	0.0	633
ABCDEF_80649	UniRef50_Q8W1L6	0.0	Normal: 527	841	443	1308	1321	1188	BlastAnno: 2586	UniRef50_Q8W1L6	726	60.39	712	281	1	2403	268	1	711	0.0	818
ABCDEF_466	UniRef50_Q8W1L6	0.0	Normal: 1014	1690	1094	1677	2438	2080	BlastAnno: 2453	UniRef50_Q8W1L6	726	71.24	699	201	0	2368	272	7	705	0.0	954
ABCDEF_54834	UniRef50_Q8W1L6	0.0	Normal: 229	220	237	298	270	261	BlastAnno: 2405	UniRef50_Q8W1L6	726	47.52	606	315	3	274	2082	121	726	0.0	580
ABCDEF_34756	UniRef50_Q8W1L6	0.0	Normal: 776	726	768	2922	4039	1079	BlastAnno: 2469	UniRef50_Q8W1L6	726	68.93	708	217	2	2396	273	7	711	0.0	957
ABCDEF_4198	UniRef50_Q8W1L6	0.0	Normal: 609	929	532	1401	1568	1297	BlastAnno: 2667	UniRef50_Q8W1L6	726	60.45	713	281	1	2463	325	1	712	0.0	820
4.2.1.74(long-chain-enoyl-CoA hydratase;)																					
No Correspond Unigene																					
>00071 Fatty acid metabolism																					
1.1.1.1(alcohol dehydrogenase;)																					
ABCDEF_31928	UniRef50_B9RHN6	0.0	Normal: 721	714	784	1329	1570	1819	BlastAnno: 1711	UniRef50_B9RHN6	437	77.55	392	87	1	360	1535	46	436	0.0	610
ABCDEF_10335	UniRef50_Q8LEB2	0.0	Normal: 285	158	514	810	352	183	BlastAnno: 1547	UniRef50_Q8LEB2	381	74.93	379	92	1	1317	181	5	380	0.0	598
1.1.1.192(long-chain-alcohol dehydrogenase;)																					
No Correspond Unigene																					
1.1.1.211(long-chain-3-hydroxyacyl-CoA dehydrogenase;)																					
ABCDEF_86264	UniRef50_Q9V397	1e-32	Normal: 0	0	0	0	0	18	BlastAnno: 294	UniRef50_Q9V397	783	63.27	98	34	2	1	291	647	743	1e-32	126
1.1.1.35(3-hydroxyacyl-CoA dehydrogenase;)																					
ABCDEF_80649	UniRef50_Q1LD07	5e-120	Normal: 527	841	443	1308	1321	1188	BlastAnno: 2586	UniRef50_Q1LD07	715	37.34	699	420	9	2358	277	22	707	5e-120	389
1.1.99.20(alkan-1-ol dehydrogenase (acceptor);)																					
No Correspond Unigene																					
1.14.14.1(unspecific monooxygenase;)																					
ABCDEF_41955	UniRef50_B9S0F8	2e-174	Normal: 70	140	28	0	0	150	BlastAnno: 1118	UniRef50_B9S0F8	506	66.67	366	115	5	2	1099	138	496	2e-174	504
1.14.15.3(alkane 1-monooxygenase;)																					
No Correspond Unigene																					
1.18.1.1(rubredoxin---NAD+ reductase;)																					
No Correspond Unigene																					
1.18.1.3(ferredoxin---NAD+ reductase;)																					
No Correspond Unigene																					
1.18.1.4(rubredoxin---NAD(P)+ reductase;)																					
No Correspond Unigene																					
1.2.1.3(aldehyde dehydrogenase (NAD+);)																					
ABCDEF_52483	UniRef50_Q70E96	0.0	Normal: 28	851	41	0	3965	420	BlastAnno: 1750	UniRef50_Q70E96	484	54.56	482	219	0	107	1552	3	484	0.0	548
ABCDEF_17638	UniRef50_Q70E96	0.0	Normal: 580	718	749	2424	1317	775	BlastAnno: 1970	UniRef50_Q70E96	484	62.21	471	177	1	283	1692	2	472	0.0	625
ABCDEF_403	UniRef50_Q8W033	0.0	Normal: 69	355	98	39	223	1111	BlastAnno: 1749	UniRef50_Q8W033	550	65.96	473	161	0	1523	105	74	546	0.0	625
ABCDEF_45143	UniRef50_Q0WSF1	0.0	Normal: 1445	874	1200	1789	1940	1504	BlastAnno: 2450	UniRef50_Q0WSF1	596	79.97	594	115	2	2220	451	1	594	0.0	1015
1.2.1.48(long-chain-aldehyde dehydrogenase;)																					
No Correspond Unigene																					
1.2.99.3(aldehyde dehydrogenase (pyrroloquinoline-quinone);)																					
ABCDEF_25501	UniRef50_A5EIC3	1e-152	Normal: 1154	1584	1099	1850	736	3942	BlastAnno: 2012	UniRef50_A5EIC3	508	50.11	473	234	2	1698	280	32	502	1e-152	461
1.3.3.6(acyl-CoA oxidase;)																					
ABCDEF_21603	UniRef50_O65202	0.0	Normal: 2124	1274	2483	4743	4370	1598	BlastAnno: 2326	UniRef50_O65202	664	79.07	664	139	0	119	2110	1	664	0.0	1139
ABCDEF_72872	UniRef50_Q96329	0.0	Normal: 14	7	25	269	10	24	BlastAnno: 1147	UniRef50_Q96329	436	77.52	347	77	1	3	1043	89	434	0.0	546
ABCDEF_23427	UniRef50_O65201	0.0	Normal: 210	178	168	322	346	166	BlastAnno: 2152	UniRef50_O65201	692	77.86	646	141	2	2005	68	1	644	0.0	1083
ABCDEF_24471	UniRef50_O65202	0.0	Normal: 429	533	717	1111	1686	1212	BlastAnno: 2664	UniRef50_O65202	664	77.56	664	149	0	277	2268	1	664	0.0	1092
ABCDEF_30962	UniRef50_O65201	0.0	Normal: 372	143	289	426	378	1018	BlastAnno: 3341	UniRef50_O65201	692	83.26	639	105	1	33	1949	56	692	0.0	1147
ABCDEF_82073	UniRef50_O65202	0.0	Normal: 141	159	270	330	550	458	BlastAnno: 1373	UniRef50_O65202	664	79.77	341	69	0	207	1229	324	664	0.0	572
ABCDEF_24472	UniRef50_O65202	0.0	Normal: 316	373	448	878	1096	796	BlastAnno: 1868	UniRef50_O65202	664	77.30	511	116	0	1592	60	1	511	0.0	841
ABCDEF_64475	UniRef50_O65202	0.0	Normal: 321	390	542	801	1250	840	BlastAnno: 2286	UniRef50_O65202	664	76.85	635	147	0	143	2047	30	664	0.0	1029
ABCDEF_25385	UniRef50_Q96329	0.0	Normal: 747	1014	820	1304	1917	2681	BlastAnno: 1768	UniRef50_Q96329	436	80.93	430	82	0	112	1401	1	430	0.0	757
1.3.99.-(Oxidoreductases;)																					
ABCDEF_4448	UniRef50_Q6JQN1	0.0	Normal: 1778	1683	1752	2720	2409	2803	BlastAnno: 2780	UniRef50_Q6JQN1	1059	48.13	802	354	14	231	2564	293	1056	0.0	718
1.3.99.13(long-chain-acyl-CoA dehydrogenase;)																					
No Correspond Unigene																					
1.3.99.2(butyryl-CoA dehydrogenase;)																					
ABCDEF_90276	UniRef50_Q0AYU5	1e-61	Normal: 0	0	0	0	0	80	BlastAnno: 757	UniRef50_Q0AYU5	380	45.63	252	126	5	3	755	44	285	1e-61	206
1.3.99.3(acyl-CoA dehydrogenase;)																					
ABCDEF_90276	UniRef50_P11310	4e-115	Normal: 0	0	0	0	0	80	BlastAnno: 757	UniRef50_P11310	421	66.14	251	79	2	3	755	80	324	4e-115	345
1.3.99.7(glutaryl-CoA dehydrogenase;)																					
ABCDEF_25385	UniRef50_Q22KH3	8e-120	Normal: 747	1014	820	1304	1917	2681	BlastAnno: 1768	UniRef50_Q22KH3	411	45.15	392	213	2	232	1404	17	407	8e-120	369
2.3.1.16(acetyl-CoA C-acyltransferase;)																					
ABCDEF_47228	UniRef50_P09110	1e-153	Normal: 321	497	306	408	1144	369	BlastAnno: 1559	UniRef50_P09110	424	59.95	387	152	3	1397	237	37	420	1e-153	454
2.3.1.21(carnitine O-palmitoyltransferase;)																					
No Correspond Unigene																					
2.3.1.9(acetyl-CoA C-acetyltransferase;)																					
ABCDEF_34825	UniRef50_Q8S4Y1	0.0	Normal: 640	698	620	807	839	1164	BlastAnno: 1773	UniRef50_Q8S4Y1	403	78.11	402	87	1	1597	395	2	403	0.0	598
ABCDEF_14629	UniRef50_Q8S4Y1	0.0	Normal: 634	316	650	1021	1278	691	BlastAnno: 1514	UniRef50_Q8S4Y1	403	85.11	403	60	0	1367	159	1	403	0.0	620
ABCDEF_107	UniRef50_Q8S4Y1	0.0	Normal: 332	460	460	842	893	424	BlastAnno: 1214	UniRef50_Q8S4Y1	403	84.07	339	54	0	1212	196	42	380	0.0	596
ABCDEF_14630	UniRef50_Q8S4Y1	0.0	Normal: 562	282	568	959	1173	627	BlastAnno: 1796	UniRef50_Q8S4Y1	403	85.50	400	58	0	436	1635	4	403	0.0	642
4.2.1.17(enoyl-CoA hydratase;)																					
ABCDEF_21259	UniRef50_Q8W1L6	0.0	Normal: 272	276	284	384	326	305	BlastAnno: 2641	UniRef50_Q8W1L6	726	47.57	721	375	3	114	2267	6	726	0.0	633

ABCDEF_80649	UniRef50_Q8W1L6	0.0	Normal: 527	841	443	1308	1321	1188	BlastAnno: 2586	UniRef50_Q8W1L6	726	60.39	712	281	1	2403	268	1	711	0.0	818
ABCDEF_466	UniRef50_Q8W1L6	0.0	Normal: 1014	1690	1094	1677	2438	2080	BlastAnno: 2453	UniRef50_Q8W1L6	726	71.24	699	201	0	2368	272	7	705	0.0	954
ABCDEF_54834	UniRef50_Q8W1L6	0.0	Normal: 229	220	237	298	270	261	BlastAnno: 2405	UniRef50_Q8W1L6	726	47.52	606	315	3	274	2082	121	726	0.0	580
ABCDEF_34756	UniRef50_Q8W1L6	0.0	Normal: 776	726	768	2922	4039	1079	BlastAnno: 2469	UniRef50_Q8W1L6	726	68.93	708	217	2	2396	273	7	711	0.0	957
ABCDEF_4198	UniRef50_Q8W1L6	0.0	Normal: 609	929	532	1401	1568	1297	BlastAnno: 2667	UniRef50_Q8W1L6	726	60.45	713	281	1	2463	325	1	712	0.0	820

4.2.1.74(long-chain-enoyl-CoA hydratase;)

No Correspond Unigene

5.1.2.3(3-hydroxybutyryl-CoA epimerase;)

No Correspond Unigene

5.3.3.8(dodecenoyl-CoA isomerase;)

No Correspond Unigene

6.2.1.20(long-chain-fatty-acid---[acyl-carrier-protein] ligase;)

ABCDEF_38252	UniRef50_Q8W471	0.0	Normal: 61	71	34	101	33	359	BlastAnno: 1698	UniRef50_Q8W471	727	64.61	534	173	5	3	1598	207	726	0.0	718

6.2.1.3(long-chain-fatty-acid---CoA ligase;)

ABCDEF_34885	UniRef50_B9RF63	0.0	Normal: 772	765	888	1188	1003	1936	BlastAnno: 2346	UniRef50_B9RF63	661	75.04	685	142	1	2166	112	6	661	0.0	1059
ABCDEF_20142	UniRef50_B9RF63	0.0	Normal: 106	208	151	252	414	285	BlastAnno: 1809	UniRef50_B9RF63	661	69.44	445	107	1	1596	262	243	658	0.0	645
ABCDEF_16801	UniRef50_Q9T0A0	0.0	Normal: 441	194	490	934	478	399	BlastAnno: 2846	UniRef50_Q9T0A0	666	47.45	411	214	2	2407	1178	10	419	0.0	410
ABCDEF_28094	UniRef50_O95573	0.0	Normal: 725	734	829	1533	2207	925	BlastAnno: 2701	UniRef50_O95573	720	46.11	720	367	7	223	2352	1	709	0.0	611
ABCDEF_45200	UniRef50_Q9T0A0	0.0	Normal: 1877	2305	1821	2911	2757	2811	BlastAnno: 2501	UniRef50_Q9T0A0	666	57.38	650	276	1	2190	244	7	656	0.0	815
ABCDEF_28095	UniRef50_O95573	0.0	Normal: 743	748	836	1598	2286	971	BlastAnno: 2700	UniRef50_O95573	720	46.11	720	367	7	226	2355	1	709	0.0	611
ABCDEF_89847	UniRef50_B9RF63	0.0	Normal: 129	253	173	306	521	343	BlastAnno: 2248	UniRef50_B9RF63	661	66.22	672	165	2	2248	233	49	658	0.0	921
ABCDEF_16803	UniRef50_Q9T0A0	0.0	Normal: 516	224	569	1076	534	421	BlastAnno: 2667	UniRef50_Q9T0A0	666	47.95	657	340	2	2273	306	10	665	0.0	665
ABCDEF_42057	UniRef50_Q9T0A0	0.0	Normal: 1626	2277	1563	3103	2538	2823	BlastAnno: 2406	UniRef50_Q9T0A0	666	76.76	654	152	0	2172	211	4	657	0.0	1053
ABCDEF_20140	UniRef50_B9RF63	0.0	Normal: 97	195	138	210	357	285	BlastAnno: 2006	UniRef50_B9RF63	661	70.03	387	87	1	1392	232	301	658	0.0	565
ABCDEF_16797	UniRef50_Q9T0A0	0.0	Normal: 562	240	601	1172	553	464	BlastAnno: 2598	UniRef50_Q9T0A0	666	47.95	657	340	2	2298	331	10	665	0.0	665
ABCDEF_20144	UniRef50_B9RF63	0.0	Normal: 166	305	199	341	627	385	BlastAnno: 2435	UniRef50_B9RF63	661	68.57	684	185	2	2310	262	4	658	0.0	979
ABCDEF_16800	UniRef50_Q9T0A0	0.0	Normal: 478	207	513	1013	495	450	BlastAnno: 2707	UniRef50_Q9T0A0	666	47.45	411	214	2	2407	1178	10	419	0.0	410
ABCDEF_16802	UniRef50_Q9T0A0	0.0	Normal: 524	227	577	1093	536	414	BlastAnno: 2737	UniRef50_Q9T0A0	666	47.95	657	340	2	2298	331	10	665	0.0	665
ABCDEF_3853	UniRef50_O95573	0.0	Normal: 824	1044	807	1839	1221	2101	BlastAnno: 2292	UniRef50_O95573	720	47.99	646	322	5	340	2265	82	717	0.0	581
ABCDEF_85741	UniRef50_B9RF63	0.0	Normal: 108	212	155	253	429	278	BlastAnno: 1726	UniRef50_B9RF63	661	68.67	466	117	1	1630	233	222	658	0.0	670
ABCDEF_45201	UniRef50_Q9T0A0	0.0	Normal: 2079	2766	1849	3232	3176	3284	BlastAnno: 3354	UniRef50_Q9T0A0	666	58.06	496	206	2	669	2153	162	656	0.0	622
ABCDEF_45202	UniRef50_Q9T0A0	0.0	Normal: 1950	2768	1872	3094	3033	3247	BlastAnno: 3295	UniRef50_Q9T0A0	666	56.95	655	281	1	3163	1202	2	656	0.0	816
ABCDEF_20143	UniRef50_B9RF63	0.0	Normal: 129	234	161	258	452	341	BlastAnno: 2417	UniRef50_B9RF63	661	70.03	387	87	1	1422	262	301	658	0.0	565

6.2.1.6(glutarate---CoA ligase;)

No Correspond Unigene

>00072 Synthesis and degradation of ketone bodies

1.1.1.30(3-hydroxybutyrate dehydrogenase;)

No Correspond Unigene

2.3.1.9(acetyl-CoA C-acetyltransferase;)

ABCDEF_34825	UniRef50_Q8S4Y1	0.0	Normal: 640	698	620	807	839	1164	BlastAnno: 1773	UniRef50_Q8S4Y1	403	78.11	402	87	1	1597	395	2	403	0.0	598
ABCDEF_14629	UniRef50_Q8S4Y1	0.0	Normal: 634	316	650	1021	1278	691	BlastAnno: 1514	UniRef50_Q8S4Y1	403	85.11	403	60	0	1367	159	1	403	0.0	620
ABCDEF_107	UniRef50_Q8S4Y1	0.0	Normal: 332	460	460	842	893	424	BlastAnno: 1214	UniRef50_Q8S4Y1	403	84.07	339	54	0	1212	196	42	380	0.0	596
ABCDEF_14630	UniRef50_Q8S4Y1	0.0	Normal: 562	282	568	959	1173	627	BlastAnno: 1796	UniRef50_Q8S4Y1	403	85.50	400	58	0	436	1635	4	403	0.0	642

2.3.3.10(hydroxymethylglutaryl-CoA synthase;)

ABCDEF_52282	UniRef50_A4GKI1	0.0	Normal: 1662	333	1405	2243	3244	1685	BlastAnno: 1805	UniRef50_A4GKI1	463	57.49	454	189	2	1606	251	2	453	0.0	536
ABCDEF_4202	UniRef50_A4GKI1	0.0	Normal: 1002	1241	821	1323	1992	1786	BlastAnno: 1998	UniRef50_A4GKI1	463	57.74	452	185	3	1636	290	2	450	0.0	531

2.8.3.5(3-oxoacid CoA-transferase;)

No Correspond Unigene

4.1.1.4(acetoacetate decarboxylase;)

No Correspond Unigene

4.1.3.4(hydroxymethylglutaryl-CoA lyase;)

ABCDEF_25184	UniRef50_P35915	3e-119	Normal: 682	2198	692	1164	701	2789	BlastAnno: 1741	UniRef50_P35915	298	63.51	296	108	0	1298	411	3	298	3e-119	363

>00100 Steroid biosynthesis

1.1.1.170(sterol-4alpha-carboxylate 3-dehydrogenase (decarboxylating);)

ABCDEF_48891	UniRef50_Q67ZE1	0.0	Normal: 748	473	919	1867	2445	1160	BlastAnno: 2311	UniRef50_Q67ZE1	564	71.66	554	157	0	2162	501	9	562	0.0	790

1.1.1.270(3-keto-steroid reductase;)

No Correspond Unigene

1.1.3.6(cholesterol oxidase;)

No Correspond Unigene

1.14.-.-(Oxidoreductases;)

ABCDEF_44739	UniRef50_O64989	0.0	Normal: 157	87	159	718	673	189	BlastAnno: 2185	UniRef50_O64989	513	70.75	482	112	3	1867	509	30	511	0.0	706
ABCDEF_31491	UniRef50_Q93VK5	0.0	Normal: 647	1826	671	1517	1770	5953	BlastAnno: 2278	UniRef50_Q93VK5	595	79.14	556	113	2	198	1862	35	588	0.0	902
ABCDEF_40554	UniRef50_P37122	0.0	Normal: 1574	1962	1829	1150	2882	2721	BlastAnno: 1828	UniRef50_P37122	505	60.52	504	198	1	1752	244	1	504	0.0	638
ABCDEF_34049	UniRef50_O23365	0.0	Normal: 363	384	349	442	549	1416	BlastAnno: 2101	UniRef50_O23365	580	76.17	579	116	5	230	1957	19	578	0.0	904
ABCDEF_21579	UniRef50_O48928	0.0	Normal: 1005	643	1606	4372	3226	1621	BlastAnno: 1895	UniRef50_O48928	513	69.72	502	151	1	185	1690	7	507	0.0	731
ABCDEF_37741	UniRef50_P37122	0.0	Normal: 12	760	9	24	34	576	BlastAnno: 1197	UniRef50_P37122	505	65.70	379	129	1	1195	59	127	504	0.0	526
ABCDEF_35618	UniRef50_O48928	0.0	Normal: 28	2648	17	142	1234	902	BlastAnno: 1746	UniRef50_O48928	513	66.40	506	168	2	1649	132	4	507	0.0	701
ABCDEF_24522	UniRef50_P37122	0.0	Normal: 334	2190	561	268	1391	2459	BlastAnno: 2075	UniRef50_P37122	505	61.52	460	176	1	1544	165	47	505	0.0	612
ABCDEF_14592	UniRef50_O23066	0.0	Normal: 1514	402	2245	7837	4742	1248	BlastAnno: 2378	UniRef50_O23066	553	72.71	546	140	4	2111	489	1	542	0.0	821
ABCDEF_14512	UniRef50_Q9FG65	0.0	Normal: 261	872	212	291	175	789	BlastAnno: 1878	UniRef50_Q9FG65	502	56.89	501	197	9	1738	266	11	502	0.0	532
ABCDEF_44487	UniRef50_Q940V4	0.0	Normal: 3	41	53	501	262	542	BlastAnno: 1712	UniRef50_Q940V4	465	69.37	382	116	1	38	1180	14	395	0.0	572
ABCDEF_17723	UniRef50_P37122	0.0	Normal: 240	1698	219	768	592	794	BlastAnno: 1757	UniRef50_P37122	505	63.40	459	166	2	228	1601	47	504	0.0	617

ABCDEF_37901	UniRef50_Q42569	0.0	Normal: 30	149	86	255	195	255	BlastAnno: 1246	UniRef50_Q42569	472	78.27	359	73	2	64	1140	43	396	0.0	567
ABCDEF_7260	UniRef50_O81117	0.0	Normal: 314	1085	466	3248	2575	1360	BlastAnno: 1768	UniRef50_O81117	514	59.92	474	188	1	145	1566	43	514	0.0	615
ABCDEF_40548	UniRef50_P37122	0.0	Normal: 2756	2467	2752	1072	3120	1735	BlastAnno: 1947	UniRef50_P37122	505	59.72	504	202	1	241	1749	1	504	0.0	636
ABCDEF_44485	UniRef50_Q940V4	0.0	Normal: 3	55	63	574	338	660	BlastAnno: 1607	UniRef50_Q940V4	465	68.58	452	141	1	42	1394	14	465	0.0	673
ABCDEF_40552	UniRef50_P37122	0.0	Normal: 2397	1870	2304	1249	10428	2291	BlastAnno: 1812	UniRef50_P37122	505	60.12	504	200	1	108	1616	1	504	0.0	640
ABCDEF_37902	UniRef50_Q42569	0.0	Normal: 84	524	254	725	670	1049	BlastAnno: 1812	UniRef50_Q42569	472	77.47	435	92	3	1675	371	43	471	0.0	681
ABCDEF_40556	UniRef50_P37122	0.0	Normal: 1907	2365	2163	1319	3678	3308	BlastAnno: 1944	UniRef50_P37122	505	61.04	498	193	1	264	1754	1	498	0.0	635
ABCDEF_40549	UniRef50_P37122	0.0	Normal: 1574	1962	1826	1150	2882	2723	BlastAnno: 1845	UniRef50_P37122	505	60.52	504	198	1	1754	246	1	504	0.0	638
ABCDEF_20301	UniRef50_O81117	0.0	Normal: 89	308	123	176	389	249	BlastAnno: 1637	UniRef50_O81117	514	64.04	470	164	3	1498	98	47	514	0.0	586
ABCDEF_3882	UniRef50_Q42569	0.0	Normal: 84	243	257	786	1561	182	BlastAnno: 1854	UniRef50_Q42569	472	72.51	422	107	1	1852	587	59	471	0.0	615
ABCDEF_20703	UniRef50_O49858	0.0	Normal: 115	636	20	0	0	597	BlastAnno: 1815	UniRef50_O49858	527	50.10	523	248	6	48	1607	12	524	0.0	533
ABCDEF_18295	UniRef50_O22203	0.0	Normal: 3667	1934	4977	10491	4588	1917	BlastAnno: 2006	UniRef50_O22203	508	74.85	493	123	1	214	1692	17	508	0.0	803
ABCDEF_48724	UniRef50_O23066	0.0	Normal: 104	11518	163	2083	3464	6356	BlastAnno: 2162	UniRef50_O23066	553	72.96	540	143	3	227	1840	1	539	0.0	807
ABCDEF_37738	UniRef50_P37122	0.0	Normal: 26	2447	19	30	33	993	BlastAnno: 1837	UniRef50_P37122	505	64.30	479	169	2	1685	249	28	504	0.0	639
1.14.13.13(calcidiol 1-monooxygenase;)																					
No Correspond Unigene																					
1.14.13.70(sterol 14-demethylase;)																					
ABCDEF_22219	UniRef50_P93846	0.0	Normal: 987	426	1054	1814	2241	997	BlastAnno: 1844	UniRef50_P93846	492	79.78	455	92	0	283	1647	37	491	0.0	768
ABCDEF_15393	UniRef50_P93846	0.0	Normal: 1099	494	1164	1984	2331	1027	BlastAnno: 1845	UniRef50_P93846	492	79.78	455	92	0	1562	198	37	491	0.0	767
1.14.13.72(methylsterol monooxygenase;)																					
ABCDEF_7015	UniRef50_Q8VWZ8	1e-168	Normal: 359	292	425	1284	2284	954	BlastAnno: 1506	UniRef50_Q8VWZ8	266	87.40	262	33	0	1193	408	1	262	1e-168	486
1.14.21.6(lathosterol oxidase;)																					
ABCDEF_125	UniRef50_Q9ZT29	5e-154	Normal: 558	311	666	978	1082	613	BlastAnno: 1254	UniRef50_Q9ZT29	271	81.04	269	51	0	958	152	3	271	5e-154	446
1.14.99.7(squalene monooxygenase;)																					
No Correspond Unigene																					
1.3.1.21(7-dehydrocholesterol reductase)																					
ABCDEF_4473	UniRef50_Q9LDU6	0.0	Normal: 1606	803	1468	2192	4553	1635	BlastAnno: 1773	UniRef50_Q9LDU6	432	86.21	428	59	0	188	1471	5	432	0.0	727
1.3.1.70(Delta14-sterol reductase)																					
ABCDEF_38311	UniRef50_Q9LDR4	3e-177	Normal: 806	310	836	1586	2143	708	BlastAnno: 1511	UniRef50_Q9LDR4	369	75.96	366	88	0	1410	313	1	366	3e-177	511
1.3.1.71(Delta24(241)-sterol reductase;)																					
No Correspond Unigene																					
1.3.1.72(Delta24-sterol reductase;)																					
ABCDEF_22103	UniRef50_Q39085	0.0	Normal: 3085	1156	2154	1230	3065	1739	BlastAnno: 2154	UniRef50_Q39085	561	80.50	564	106	2	177	1865	1	561	0.0	953
ABCDEF_22506	UniRef50_Q39085	0.0	Normal: 2779	1031	2012	1149	2832	1570	BlastAnno: 2149	UniRef50_Q39085	561	80.50	564	106	2	173	1861	1	561	0.0	954
1.3.3.-(Oxidoreductases;)																					
No Correspond Unigene																					
2.1.1.143(24-methylenesterol C-methyltransferase;)																					
ABCDEF_52510	UniRef50_Q39227	0.0	Normal: 3006	3717	3296	5169	6629	4468	BlastAnno: 1520	UniRef50_Q39227	361	82.82	355	61	0	1441	377	1	355	0.0	630
2.1.1.41(sterol 24-C-methyltransferase;)																					
ABCDEF_73505	UniRef50_Q9LM02	0.0	Normal: 1317	580	1457	2979	4573	1825	BlastAnno: 1548	UniRef50_Q9LM02	336	77.25	334	76	0	1409	408	2	335	0.0	540
ABCDEF_38947	UniRef50_Q9LM02	0.0	Normal: 1513	657	1648	3276	5140	2099	BlastAnno: 1602	UniRef50_Q9LM02	336	77.01	335	77	0	1412	408	1	335	0.0	538
2.3.1.26(sterol O-acyltransferase;)																					
ABCDEF_55936	UniRef50_Q23DR5	1e-08	Normal: 10	0	8	12	0	0	BlastAnno: 246	UniRef50_Q23DR5	561	42.62	61	35	0	216	34	317	377	1e-08	57.4
2.3.1.73(diacylglycerol---sterol O-acyltransferase;)																					
No Correspond Unigene																					
2.5.1.21(squalene synthase;)																					
ABCDEF_14673	UniRef50_P53799	0.0	Normal: 380	686	376	572	1122	1175	BlastAnno: 1655	UniRef50_P53799	410	76.86	389	87	2	1481	324	1	389	0.0	641
ABCDEF_12077	UniRef50_P53799	0.0	Normal: 241	296	200	562	529	400	BlastAnno: 1427	UniRef50_P53799	410	74.75	408	99	2	1259	42	1	406	0.0	654
ABCDEF_70952	UniRef50_P53799	0.0	Normal: 230	298	192	574	526	382	BlastAnno: 1391	UniRef50_P53799	410	74.75	408	99	2	1259	42	1	406	0.0	654
ABCDEF_32228	UniRef50_P53799	0.0	Normal: 350	399	266	694	798	589	BlastAnno: 1747	UniRef50_P53799	410	74.75	408	99	2	1579	362	1	406	0.0	655
ABCDEF_14674	UniRef50_P53799	0.0	Normal: 351	640	355	556	1055	1171	BlastAnno: 1646	UniRef50_P53799	410	76.86	389	87	2	166	1323	1	389	0.0	641
ABCDEF_26257	UniRef50_P53799	0.0	Normal: 336	394	256	699	796	567	BlastAnno: 1709	UniRef50_P53799	410	74.75	408	99	2	1577	360	1	406	0.0	655
ABCDEF_67342	UniRef50_P53799	0.0	Normal: 358	629	356	544	1042	1132	BlastAnno: 1581	UniRef50_P53799	410	76.86	389	87	2	1448	291	1	389	0.0	641
3.1.1.13(sterol esterase;)																					
No Correspond Unigene																					
5.-.-.-(Isomerases)																					
ABCDEF_37668	UniRef50_A8DJY4	1e-109	Normal: 240	328	262	459	844	1032	BlastAnno: 2373	UniRef50_A8DJY4	512	37.90	504	291	6	1825	359	12	508	1e-109	353
5.3.3.5(cholestenol Delta-isomerase)																					
ABCDEF_41990	UniRef50_O48962	2e-83	Normal: 303	185	324	667	1207	509	BlastAnno: 1240	UniRef50_O48962	223	62.22	225	78	2	194	853	1	223	2e-83	263
5.4.99.17(squalene---hopene cyclase)																					
No Correspond Unigene																					
5.4.99.7(lanosterol synthase;)																					
ABCDEF_62592	UniRef50_Q10231	4e-35	Normal: 0	31	0	0	0	5	BlastAnno: 378	UniRef50_Q10231	721	50.42	119	59	0	21	377	477	595	4e-35	134
ABCDEF_60865	UniRef50_Q10231	4e-35	Normal: 0	27	0	0	0	4	BlastAnno: 363	UniRef50_Q10231	721	50.42	119	59	0	6	362	477	595	4e-35	134
5.4.99.8(cycloartenol synthase;)																					
ABCDEF_13958	UniRef50_P38605	0.0	Normal: 349	1130	313	192	228	618	BlastAnno: 2496	UniRef50_P38605	759	58.26	757	311	3	3	2270	3	755	0.0	941
ABCDEF_5907	UniRef50_P38605	0.0	Normal: 697	321	588	1357	1262	490	BlastAnno: 2728	UniRef50_P38605	759	78.66	759	161	1	2495	222	1	759	0.0	1293
ABCDEF_57951	UniRef50_P38605	0.0	Normal: 269	137	258	480	495	247	BlastAnno: 1697	UniRef50_P38605	759	76.86	389	89	1	361	1524	371	759	0.0	644
ABCDEF_63674	UniRef50_P38605	0.0	Normal: 1	316	3	0	5	68	BlastAnno: 1548	UniRef50_P38605	759	57.29	487	205	3	91	1548	1	485	0.0	598
ABCDEF_75086	UniRef50_P38605	0.0	Normal: 323	157	310	639	620	275	BlastAnno: 1970	UniRef50_P38605	759	78.25	492	106	1	1645	173	268	759	0.0	836
ABCDEF_58677	UniRef50_P38605	0.0	Normal: 242	116	229	455	471	210	BlastAnno: 1511	UniRef50_P38605	759	76.86	389	89	1	175	1338	371	759	0.0	644
ABCDEF_5897	UniRef50_P38605	0.0	Normal: 484	238	377	875	800	314	BlastAnno: 1837	UniRef50_P38605	759	80.28	426	84	0	180	1457	1	426	0.0	751

ABCDEF_41522 UniRef50_P38605 0.0 Normal: 0 1223 0 0 0 220 BlastAnno: 2570 UniRef50_P38605 759 52.11 760 360 4 2514 238 1 757 0.0 846
ABCDEF_54710 UniRef50_P38605 0.0 Normal: 266 136 258 479 491 240 BlastAnno: 1657 UniRef50_P38605 759 76.86 389 89 1 321 1484 371 759 0.0 644
ABCDEF_38912 UniRef50_P38605 0.0 Normal: 1 435 5 0 10 99 BlastAnno: 1687 UniRef50_P38605 759 57.29 487 205 3 1458 1 1 485 0.0 599
5.5.1.9(cycloeucalenol cycloisomerase;)
ABCDEF_524 UniRef50_Q9M643 2e-158 Normal: 483 183 424 690 985 722 BlastAnno: 1330 UniRef50_Q9M643 280 79.12 273 57 0 176 994 8 280 2e-158 458
>00120 Primary bile acid biosynthesis
1.1.1.181(cholest-5-ene-3beta,7alpha-diol 3beta-dehydrogenase;)
No Correspond Unigene
1.1.1.35(3-hydroxyacyl-CoA dehydrogenase;)
ABCDEF_80649 UniRef50_Q1LD07 5e-120 Normal: 527 841 443 1308 1321 1188 BlastAnno: 2586 UniRef50_Q1LD07 715 37.34 699 420 9 2358 277 22 707 5e-120 389
1.1.1.50(3alpha-hydroxysteroid dehydrogenase (B-specific);)
No Correspond Unigene
1.14.13.1(salicylate 1-monooxygenase;)
No Correspond Unigene
1.14.13.100(25-hydroxycholesterol 7alpha-hydroxylase;)
No Correspond Unigene
1.14.13.15(cholestanetriol 26-monooxygenase;)
No Correspond Unigene
1.14.13.17(cholesterol 7alpha-monooxygenase;)
No Correspond Unigene
1.14.13.96(5beta-cholestane-3alpha,7alpha-diol 12alpha-hydroxylase;)
No Correspond Unigene
1.14.13.98(cholesterol 24-hydroxylase;)
No Correspond Unigene
1.14.13.99(24-hydroxycholesterol 7alpha-hydroxylase;)
No Correspond Unigene
1.14.99.38(cholesterol 25-hydroxylase;)
No Correspond Unigene
1.17.99.3(3alpha,7alpha,12alpha-trihydroxy-5beta-cholestanoyl-CoA 24-hydroxylase;)
ABCDEF_82073 UniRef50_Q99424 4e-60 Normal: 141 159 270 330 550 458 BlastAnno: 1373 UniRef50_Q99424 681 36.76 340 207 4 207 1205 337 675 4e-60 216
1.3.1.3(Delta4-3-oxosteroid 5beta-reductase;)
No Correspond Unigene
2.3.1.176(propanoyl-CoA C-acyltransferase;)
No Correspond Unigene
2.3.1.65(bile acid-CoA:amino acid N-acyltransferase;)
No Correspond Unigene
3.5.1.24(choloylglycine hydrolase;)
No Correspond Unigene
4.2.1.107(3alpha,7alpha,12alpha-trihydroxy-5beta-cholest-24-enoyl-CoA hydratase;)
No Correspond Unigene
5.1.99.4(alpha-methylacyl-CoA racemase)
No Correspond Unigene
6.2.1.7(cholate---CoA ligase;)
No Correspond Unigene
>00121 Secondary bile acid biosynthesis
1.-.-.-(Oxidoreductases)
ABCDEF_345 UniRef50_Q6Z690 0.0 Normal: 442 146 449 340 272 314 BlastAnno: 1658 UniRef50_Q6Z690 849 63.42 462 150 5 1657 314 387 843 0.0 589
ABCDEF_24068 UniRef50_Q9FI41 0.0 Normal: 214 316 207 270 263 849 BlastAnno: 1742 UniRef50_Q9FI41 387 73.28 363 96 1 1440 355 4 366 0.0 554
ABCDEF_24070 UniRef50_Q9FI41 0.0 Normal: 189 290 182 231 232 751 BlastAnno: 1479 UniRef50_Q9FI41 387 73.28 363 96 1 1363 278 4 366 0.0 551
ABCDEF_13957 UniRef50_Q9CAE3 0.0 Normal: 399 318 392 489 456 586 BlastAnno: 2488 UniRef50_Q9CAE3 789 80.85 496 93 2 3 1487 285 779 0.0 768
ABCDEF_28410 UniRef50_Q9LIG0 0.0 Normal: 211 366 234 286 433 1504 BlastAnno: 1324 UniRef50_Q9LIG0 330 74.92 331 76 2 1147 173 1 330 0.0 521
ABCDEF_17581 UniRef50_Q8LGI2 0.0 Normal: 758 336 735 426 374 852 BlastAnno: 1543 UniRef50_Q8LGI2 454 68.78 442 136 2 71 1390 8 449 0.0 597
ABCDEF_23753 UniRef50_Q94A68 0.0 Normal: 284 559 215 224 164 519 BlastAnno: 1580 UniRef50_Q94A68 377 81.90 348 63 0 1349 306 30 377 0.0 576
ABCDEF_24071 UniRef50_Q9FI41 0.0 Normal: 235 377 230 278 289 948 BlastAnno: 2090 UniRef50_Q9FI41 387 73.28 363 96 1 1726 641 4 366 0.0 553
1.17.99.5(bile-acid 7alpha-dehydroxylase;)
No Correspond Unigene
3.1.2.26(bile-acid-CoA hydrolase)
No Correspond Unigene
3.5.1.24(choloylglycine hydrolase;)
No Correspond Unigene
3.5.1.74(chenodeoxycholoyltaurine hydrolase)
No Correspond Unigene
4.2.1.-(Lyases;)
ABCDEF_48679 UniRef50_Q0RP29 5e-115 Normal: 568 325 563 941 1285 676 BlastAnno: 1424 UniRef50_Q0RP29 493 56.39 321 129 4 119 1075 39 350 5e-115 356
4.2.1.106(bile-acid 7alpha-dehydratase;)
No Correspond Unigene
6.-.-.-(No Name)
ABCDEF_30078 UniRef50_Q6L4L4 0.0 Normal: 873 690 641 788 1014 1012 BlastAnno: 3221 UniRef50_Q6L4L4 875 50.18 815 380 18 3086 669 78 875 0.0 721
ABCDEF_30143 UniRef50_Q6L4L4 0.0 Normal: 95 103 72 139 90 112 BlastAnno: 1586 UniRef50_Q6L4L4 875 61.24 485 163 9 1467 28 3 467 0.0 618
ABCDEF_30141 UniRef50_Q6L4L4 0.0 Normal: 1434 827 1232 1415 1424 1692 BlastAnno: 5949 UniRef50_Q6L4L4 875 50.00 876 376 23 5791 3221 3 835 0.0 802
>00130 Ubiquinone and other terpenoid-quinone biosynthesis
1.1.1.237(hydroxyphenylpyruvate reductase;)

No Correspond Unigene																					
1.13.11.27(4-hydroxyphenylpyruvate dioxygenase;)																					
ABCDEF_7370	UniRef50_P93836	0.0	Normal: 512	1122	253	32	118	3444	BlastAnno: 1725	UniRef50_P93836	445	74.00	423	102	3	1616	363	23	442	0.0	604
1.14.-.-(Oxidoreductases;)																					
ABCDEF_44739	UniRef50_O64989	0.0	Normal: 157	87	159	718	673	189	BlastAnno: 2185	UniRef50_O64989	513	70.75	482	112	3	1867	509	30	511	0.0	706
ABCDEF_31491	UniRef50_Q93VK5	0.0	Normal: 647	1826	671	1517	1770	5953	BlastAnno: 2278	UniRef50_Q93VK5	595	79.14	556	113	2	198	1862	35	588	0.0	902
ABCDEF_40554	UniRef50_P37122	0.0	Normal: 1574	1962	1829	1150	2882	2721	BlastAnno: 1828	UniRef50_P37122	505	60.52	504	198	1	1752	244	1	504	0.0	638
ABCDEF_34049	UniRef50_O23365	0.0	Normal: 363	384	349	442	549	1416	BlastAnno: 2101	UniRef50_O23365	580	76.17	579	116	5	230	1957	19	578	0.0	904
ABCDEF_21579	UniRef50_O48928	0.0	Normal: 1005	643	1606	4372	3226	1621	BlastAnno: 1895	UniRef50_O48928	513	69.72	502	151	1	185	1690	7	507	0.0	731
ABCDEF_37741	UniRef50_P37122	0.0	Normal: 12	760	9	24	34	576	BlastAnno: 1197	UniRef50_P37122	505	65.70	379	129	1	1195	59	127	504	0.0	526
ABCDEF_35618	UniRef50_O48928	0.0	Normal: 28	2648	17	142	1234	902	BlastAnno: 1746	UniRef50_O48928	513	66.40	506	168	2	1649	132	4	507	0.0	701
ABCDEF_24522	UniRef50_P37122	0.0	Normal: 334	2190	561	268	1391	2459	BlastAnno: 2075	UniRef50_P37122	505	61.52	460	176	1	1544	165	47	505	0.0	612
ABCDEF_14592	UniRef50_O23066	0.0	Normal: 1514	402	2245	7837	4742	1248	BlastAnno: 2378	UniRef50_O23066	553	72.71	546	140	4	2111	489	1	542	0.0	821
ABCDEF_14512	UniRef50_Q9FG65	0.0	Normal: 261	872	212	291	175	789	BlastAnno: 1878	UniRef50_Q9FG65	502	56.89	501	197	9	1738	266	11	502	0.0	532
ABCDEF_44487	UniRef50_Q940V4	0.0	Normal: 3	41	53	501	262	542	BlastAnno: 1712	UniRef50_Q940V4	465	69.37	382	116	1	38	1180	14	395	0.0	572
ABCDEF_17723	UniRef50_P37122	0.0	Normal: 240	1698	219	768	592	794	BlastAnno: 1757	UniRef50_P37122	505	63.40	459	166	2	228	1601	47	504	0.0	617
ABCDEF_37901	UniRef50_Q42569	0.0	Normal: 30	149	86	255	195	255	BlastAnno: 1246	UniRef50_Q42569	472	78.27	359	73	2	64	1140	43	396	0.0	567
ABCDEF_7260	UniRef50_O81117	0.0	Normal: 314	1085	466	3248	2575	1360	BlastAnno: 1768	UniRef50_O81117	514	59.92	474	188	1	145	1566	43	514	0.0	615
ABCDEF_40548	UniRef50_P37122	0.0	Normal: 2756	2467	2752	1072	3120	1735	BlastAnno: 1947	UniRef50_P37122	505	59.72	504	202	1	241	1749	1	504	0.0	636
ABCDEF_44485	UniRef50_Q940V4	0.0	Normal: 3	55	63	574	338	660	BlastAnno: 1607	UniRef50_Q940V4	465	68.58	452	141	1	42	1394	14	465	0.0	673
ABCDEF_40552	UniRef50_P37122	0.0	Normal: 2397	1870	2304	1249	10428	2291	BlastAnno: 1812	UniRef50_P37122	505	60.12	504	200	1	108	1616	1	504	0.0	640
ABCDEF_37902	UniRef50_Q42569	0.0	Normal: 84	524	254	725	670	1049	BlastAnno: 1812	UniRef50_Q42569	472	77.47	435	92	3	1675	371	43	471	0.0	681
ABCDEF_40556	UniRef50_P37122	0.0	Normal: 1907	2365	2163	1319	3678	3308	BlastAnno: 1944	UniRef50_P37122	505	61.04	498	193	1	264	1754	1	498	0.0	635
ABCDEF_40549	UniRef50_P37122	0.0	Normal: 1574	1962	1826	1150	2882	2723	BlastAnno: 1845	UniRef50_P37122	505	60.52	504	198	1	1754	246	1	504	0.0	638
ABCDEF_20301	UniRef50_O81117	0.0	Normal: 89	308	123	176	389	249	BlastAnno: 1637	UniRef50_O81117	514	64.04	470	164	3	1498	98	47	514	0.0	586
ABCDEF_3882	UniRef50_Q42569	0.0	Normal: 84	243	257	786	1561	182	BlastAnno: 1854	UniRef50_Q42569	472	72.51	422	107	1	1852	587	59	471	0.0	615
ABCDEF_20703	UniRef50_O49858	0.0	Normal: 115	636	20	0	0	597	BlastAnno: 1815	UniRef50_O49858	527	50.10	523	248	6	48	1607	12	524	0.0	533
ABCDEF_18295	UniRef50_O22203	0.0	Normal: 3667	1934	4977	10491	4588	1917	BlastAnno: 2006	UniRef50_O22203	508	74.85	493	123	1	214	1692	17	508	0.0	803
ABCDEF_48724	UniRef50_O23066	0.0	Normal: 104	11518	163	2083	3464	6356	BlastAnno: 2162	UniRef50_O23066	553	72.96	540	143	3	227	1840	1	539	0.0	807
ABCDEF_37738	UniRef50_P37122	0.0	Normal: 26	2447	19	30	33	993	BlastAnno: 1837	UniRef50_P37122	505	64.30	479	169	2	1685	249	28	504	0.0	639
1.14.13.-(Oxidoreductases;)																					
ABCDEF_45538	UniRef50_O23024	3e-156	Normal: 110	6	165	774	133	36	BlastAnno: 1258	UniRef50_O23024	437	58.18	385	151	3	3	1157	37	411	3e-156	457
1.21.-.-(Oxidoreductases;)																					
ABCDEF_21236	UniRef50_Q9SVG4	6e-179	Normal: 367	1143	460	1038	1231	1282	BlastAnno: 2010	UniRef50_Q9SVG4	570	56.37	518	210	5	1763	213	34	536	6e-179	530
2.1.1.-(Transferases;)																					
ABCDEF_20806	UniRef50_Q94II3	0.0	Normal: 700	332	876	1458	1997	929	BlastAnno: 2275	UniRef50_Q94II3	600	73.57	594	153	4	2119	341	1	591	0.0	907
ABCDEF_45802	UniRef50_Q9ZPH9	0.0	Normal: 3543	3348	4296	9797	10830	5017	BlastAnno: 2642	UniRef50_Q9ZPH9	633	53.90	603	261	7	2216	456	23	624	0.0	676
ABCDEF_20070	UniRef50_Q944R7	0.0	Normal: 301	141	265	207	185	187	BlastAnno: 2675	UniRef50_Q944R7	724	58.28	719	275	9	127	2265	13	712	0.0	840
ABCDEF_34956	UniRef50_Q94II3	0.0	Normal: 1120	7269	2475	16033	22219	21461	BlastAnno: 2676	UniRef50_Q94II3	600	73.77	610	146	4	404	2230	1	597	0.0	957
ABCDEF_17121	UniRef50_Q9ZPH9	0.0	Normal: 124	77	109	284	199	132	BlastAnno: 2066	UniRef50_Q9ZPH9	633	53.24	601	259	8	161	1903	25	623	0.0	644
ABCDEF_6524	UniRef50_Q9ZPH9	0.0	Normal: 471	296	490	753	870	730	BlastAnno: 2602	UniRef50_Q9ZPH9	633	50.89	619	290	5	2221	404	6	623	0.0	659
ABCDEF_56017	UniRef50_Q9ZPH9	0.0	Normal: 302	167	286	596	748	529	BlastAnno: 2384	UniRef50_Q9ZPH9	633	51.37	619	287	5	213	2030	6	623	0.0	663
ABCDEF_44169	UniRef50_Q9SIZ3	0.0	Normal: 179	158	273	705	685	229	BlastAnno: 2300	UniRef50_Q9SIZ3	589	62.01	308	108	4	1219	296	285	583	0.0	394
ABCDEF_6521	UniRef50_Q9ZPH9	0.0	Normal: 339	198	314	641	767	582	BlastAnno: 2511	UniRef50_Q9ZPH9	633	51.37	619	287	5	2172	355	6	623	0.0	663
ABCDEF_44172	UniRef50_Q9SIZ3	0.0	Normal: 204	184	287	767	741	242	BlastAnno: 2317	UniRef50_Q9SIZ3	589	68.15	449	134	4	1637	291	144	583	0.0	641
ABCDEF_199	UniRef50_Q9SU94	0.0	Normal: 1076	275	1009	558	1247	306	BlastAnno: 1769	UniRef50_Q9SU94	390	78.45	348	74	1	284	1324	43	390	0.0	584
ABCDEF_52252	UniRef50_Q84W92	0.0	Normal: 1658	720	1653	1980	2289	1601	BlastAnno: 2170	UniRef50_Q84W92	535	72.49	469	120	3	409	1791	34	501	0.0	703
ABCDEF_51404	UniRef50_A3BMN9	0.0	Normal: 864	188	716	337	631	326	BlastAnno: 2192	UniRef50_A3BMN9	620	50.50	604	243	9	2008	251	55	620	0.0	555
ABCDEF_21386	UniRef50_Q94AI4	0.0	Normal: 2326	1250	2220	4294	2920	1983	BlastAnno: 3701	UniRef50_Q94AI4	775	47.27	842	332	16	567	2978	8	775	0.0	577
ABCDEF_35101	UniRef50_Q9MAT5	0.0	Normal: 1440	420	1268	576	1136	850	BlastAnno: 1634	UniRef50_Q9MAT5	383	69.03	352	109	0	1449	394	32	383	0.0	550
ABCDEF_960	UniRef50_Q9LN50	0.0	Normal: 3504	13198	3609	11156	19651	12155	BlastAnno: 2626	UniRef50_Q9LN50	724	49.14	525	246	5	883	2451	219	724	0.0	570
ABCDEF_48082	UniRef50_Q9LN50	0.0	Normal: 410	445	467	870	993	454	BlastAnno: 2340	UniRef50_Q9LN50	724	70.25	521	155	0	710	2272	204	724	0.0	821
ABCDEF_10473	UniRef50_Q08A71	0.0	Normal: 374	187	332	425	456	314	BlastAnno: 1764	UniRef50_Q08A71	435	79.23	366	75	1	344	1438	70	435	0.0	575
ABCDEF_7295	UniRef50_Q9ZPH9	0.0	Normal: 25	54	30	81	26	329	BlastAnno: 1191	UniRef50_Q9ZPH9	633	69.23	403	110	4	1191	22	225	626	0.0	599
ABCDEF_4160	UniRef50_Q94II3	0.0	Normal: 189	0	210	71	17	72	BlastAnno: 1424	UniRef50_Q94II3	600	77.28	449	101	1	1424	81	143	591	0.0	758
ABCDEF_44857	UniRef50_Q9ZPH9	0.0	Normal: 101	224	103	299	588	350	BlastAnno: 1582	UniRef50_Q9ZPH9	633	72.06	519	143	1	1571	21	114	632	0.0	822
ABCDEF_71541	UniRef50_Q9SIZ3	0.0	Normal: 187	167	296	765	724	237	BlastAnno: 2268	UniRef50_Q9SIZ3	589	62.01	308	108	4	1210	287	285	583	0.0	394
ABCDEF_44171	UniRef50_Q9SIZ3	0.0	Normal: 214	196	312	827	802	251	BlastAnno: 2339	UniRef50_Q9SIZ3	589	64.78	548	182	5	1978	335	47	583	0.0	717
ABCDEF_20805	UniRef50_Q94II3	0.0	Normal: 674	344	885	1417	2194	963	BlastAnno: 2400	UniRef50_Q94II3	600	78.21	436	94	1	879	2183	156	591	0.0	734
ABCDEF_18210	UniRef50_Q9SIZ3	0.0	Normal: 5584	3714	7379	17006	21892	7546	BlastAnno: 3199	UniRef50_Q9SIZ3	589	50.00	576	269	7	2145	424	27	585	0.0	574
ABCDEF_6526	UniRef50_Q9ZPH9	0.0	Normal: 627	185	837	2717	2815	1530	BlastAnno: 2611	UniRef50_Q9ZPH9	633	54.76	599	259	5	462	2222	25	623	0.0	689
ABCDEF_6525	UniRef50_Q9ZPH9	0.0	Normal: 602	165	760	2369	2605	1400	BlastAnno: 2545	UniRef50_Q9ZPH9	633	54.92	599	258	5	431	2191	25	623	0.0	689
ABCDEF_6523	UniRef50_Q9ZPH9	0.0	Normal: 314	175	304	641	784	552	BlastAnno: 2312	UniRef50_Q9ZPH9	633	52.42	599	272	4	201	1958	25	623	0.0	663
ABCDEF_67393	UniRef50_Q9ZPH9	0.0	Normal: 434	245	444	608	768	655	BlastAnno: 2346	UniRef50_Q9ZPH9	633	51.92	599	275	4	232	1989	25	623	0.0	659
ABCDEF_3579	UniRef50_O14744	0.0	Normal: 1361	326	984	568	791	440	BlastAnno: 2369	UniRef50_O14744	637	47.00	634	294	10	267	2120	30	637	0.0	553
2.1.1.114(polyprenyldihydroxybenzoate methyltransferase;)																					
ABCDEF_20755	UniRef50_O49354	1e-130	Normal: 256	141	313	384	380	278	BlastAnno: 1205	UniRef50_O49354	322	76.67	240	56	0	931	212	76	315	1e-130	387
2.1.1.64(3-demethylubiquinol 3-O-methyltransferase;)																					
ABCDEF_20755	UniRef50_B9S8D8	2e-101	Normal: 256	141	313	384	380	278	BlastAnno: 1205	UniRef50_B9S8D8	256	59.70	268	51	2	1009	206	45	255	2e-101	310
2.1.1.95(tocopherol O-methyltransferase;)																					
ABCDEF_25054	UniRef50_Q9ZSK1	6e-142	Normal: 534	923	446	857	2397	966	BlastAnno: 1610	UniRef50_Q9ZSK1	348	71.97	289	78	2	441	1298	58	346	6e-142	422
2.2.1.9(2-succinyl-5-enolpyruvyl-6-hydroxy-3-cyclohexene-1-carboxylic-acid synthase;)																					

ABCDEF_35932	UniRef50_Q15KI9	7e-111	Normal: 26	15	7	7	0	51	BlastAnno: 715	UniRef50_Q15KI9	1715	69.62	237	72	0	714	4	377	613	7e-111	356
2.4.1.194(4-hydroxybenzoate 4-O-beta-D-glucosyltransferase;)																					
No Correspond Unigene																					
2.5.1.-(Transferases;)																					
ABCDEF_48175	UniRef50_P72580	2e-123	Normal: 366	927	282	531	590	1811	BlastAnno: 1917	UniRef50_P72580	323	58.44	320	130	2	610	1569	6	322	2e-123	377
2.5.1.39(4-hydroxybenzoate polyprenyltransferase;)																					
ABCDEF_35151	UniRef50_Q93YP7	1e-83	Normal: 209	92	221	233	269	209	BlastAnno: 952	UniRef50_Q93YP7	407	76.92	169	39	0	3	509	236	404	1e-83	266
2.5.1.74(1,4-dihydroxy-2-naphthoate polyprenyltransferase)																					
ABCDEF_36928	UniRef50_P73962	4e-57	Normal: 68	73	81	57	30	262	BlastAnno: 1418	UniRef50_P73962	307	41.97	274	154	2	310	1131	22	290	4e-57	199
2.6.1.5(tyrosine transaminase;)																					
ABCDEF_52650	UniRef50_Q9LVY1	0.0	Normal: 493	358	455	302	300	892	BlastAnno: 1442	UniRef50_Q9LVY1	420	61.26	413	160	0	65	1303	5	417	0.0	539
3.1.2.-(Hydrolases;)																					
ABCDEF_2988	UniRef50_Q39513	0.0	Normal: 684	868	676	1224	1298	1392	BlastAnno: 1728	UniRef50_Q39513	415	69.13	392	111	5	408	1574	12	396	0.0	554
ABCDEF_42638	UniRef50_Q9SHJ8	0.0	Normal: 183	172	331	565	390	214	BlastAnno: 1547	UniRef50_Q9SHJ8	395	68.62	376	94	2	196	1320	42	394	0.0	533
ABCDEF_56804	UniRef50_Q9SHJ8	0.0	Normal: 160	146	249	472	328	155	BlastAnno: 1439	UniRef50_Q9SHJ8	395	68.62	376	94	2	275	1399	42	394	0.0	533
ABCDEF_2987	UniRef50_Q39513	0.0	Normal: 608	561	472	1162	1435	954	BlastAnno: 1938	UniRef50_Q39513	415	67.26	394	119	5	408	1580	12	398	0.0	544
ABCDEF_20456	UniRef50_Q9LK08	0.0	Normal: 375	366	460	796	1014	454	BlastAnno: 1539	UniRef50_Q9LK08	418	64.52	420	140	3	1434	187	4	418	0.0	559
ABCDEF_49725	UniRef50_Q9SHJ8	0.0	Normal: 176	174	327	582	397	195	BlastAnno: 1556	UniRef50_Q9SHJ8	395	68.62	376	94	2	234	1358	42	394	0.0	533
ABCDEF_20453	UniRef50_Q9LK08	0.0	Normal: 396	358	461	824	1019	461	BlastAnno: 1565	UniRef50_Q9LK08	418	66.90	420	134	2	1459	200	4	418	0.0	586
3.1.2.23(4-hydroxybenzoyl-CoA thioesterase)																					
No Correspond Unigene																					
3.2.1.-(Hydrolases;)																					
ABCDEF_55518	UniRef50_Q9SGZ5	0.0	Normal: 137	145	110	155	249	211	BlastAnno: 1222	UniRef50_Q9SGZ5	767	66.34	407	136	1	1218	1	142	548	0.0	556
ABCDEF_45209	UniRef50_Q9LXA8	0.0	Normal: 1338	811	1191	1817	1167	1821	BlastAnno: 2643	UniRef50_Q9LXA8	792	47.09	790	379	11	2448	178	7	790	0.0	745
ABCDEF_34153	UniRef50_Q9LXA8	0.0	Normal: 522	668	469	700	564	936	BlastAnno: 2528	UniRef50_Q9LXA8	792	51.44	762	345	6	2397	175	30	787	0.0	804
ABCDEF_34154	UniRef50_Q9LXA8	0.0	Normal: 495	622	454	650	534	930	BlastAnno: 2619	UniRef50_Q9LXA8	792	50.53	655	301	5	2076	175	135	787	0.0	679
ABCDEF_47469	UniRef50_Q9SXC9	0.0	Normal: 80	59	95	141	105	115	BlastAnno: 1550	UniRef50_Q9SXC9	574	81.45	248	46	0	3	746	93	340	0.0	419
ABCDEF_3050	UniRef50_A8JBU2	0.0	Normal: 365	517	518	1626	1809	451	BlastAnno: 2998	UniRef50_A8JBU2	725	52.75	673	264	8	2589	604	50	679	0.0	670
ABCDEF_3049	UniRef50_A8JBU2	0.0	Normal: 528	690	724	2218	2620	674	BlastAnno: 3077	UniRef50_A8JBU2	725	52.32	711	286	7	2650	551	55	723	0.0	707
ABCDEF_34152	UniRef50_Q9LXA8	0.0	Normal: 568	815	532	816	648	1135	BlastAnno: 2581	UniRef50_Q9LXA8	792	51.24	765	348	6	2450	219	30	790	0.0	806
ABCDEF_34155	UniRef50_Q9LXA8	0.0	Normal: 543	763	515	772	621	1127	BlastAnno: 2640	UniRef50_Q9LXA8	792	50.30	658	304	5	2112	202	135	790	0.0	681
ABCDEF_21385	UniRef50_Q9LXA8	0.0	Normal: 432	334	511	873	734	380	BlastAnno: 2109	UniRef50_Q9LXA8	792	62.64	629	229	4	3	1877	152	778	0.0	775
ABCDEF_980	UniRef50_Q9LXA8	0.0	Normal: 1398	7037	1070	4974	7681	6612	BlastAnno: 2765	UniRef50_Q9LXA8	792	49.22	772	351	9	257	2470	28	792	0.0	753
ABCDEF_31427	UniRef50_Q9LXA8	0.0	Normal: 56	316	89	4893	4124	2869	BlastAnno: 2823	UniRef50_Q9LXA8	792	48.25	773	359	12	209	2425	26	791	0.0	716
ABCDEF_27305	UniRef50_Q9FG93	0.0	Normal: 247	143	251	353	378	254	BlastAnno: 1883	UniRef50_Q9FG93	624	70.43	487	123	3	2	1456	126	593	0.0	702
ABCDEF_47466	UniRef50_Q9SXC9	0.0	Normal: 69	48	83	133	95	97	BlastAnno: 1452	UniRef50_Q9SXC9	574	75.84	476	115	0	2	1429	96	571	0.0	769
ABCDEF_3403	UniRef50_Q9LXA8	0.0	Normal: 206	82	182	114	358	291	BlastAnno: 2202	UniRef50_Q9LXA8	792	55.15	515	213	4	2198	702	84	596	0.0	548
3.2.2.26(futalosine hydrolase;)																					
No Correspond Unigene																					
4.1.1.-(Lyases;)																					
ABCDEF_57496	UniRef50_O96569	9e-20	Normal: 4	0	0	0	0	9	BlastAnno: 223	UniRef50_O96569	439	66.67	72	22	1	210	1	19	90	9e-20	87.4
4.1.3.36(1,4-dihydroxy-2-naphthoyl-CoA synthase;)																					
ABCDEF_38118	UniRef50_Q5HH38	1e-118	Normal: 167	600	45	8	27	2238	BlastAnno: 1211	UniRef50_Q5HH38	273	64.66	266	92	1	318	1115	10	273	1e-118	355
4.1.3.40(chorismate lyase;)																					
No Correspond Unigene																					
4.1.99.-(Lyases;)																					
No Correspond Unigene																					
4.2.1.113(o-succinylbenzoate synthase;)																					
No Correspond Unigene																					
4.2.99.20(2-succinyl-6-hydroxy-2,4-cyclohexadiene-1-carboxylate synthase;)																					
No Correspond Unigene																					
5.4.4.2(isochorismate synthase;)																					
ABCDEF_51061	UniRef50_Q9S7H8	8e-178	Normal: 84	86	78	98	74	350	BlastAnno: 1688	UniRef50_Q9S7H8	569	62.19	402	152	0	35	1240	159	560	8e-178	523
6.2.1.12(4-coumarate---CoA ligase;)																					
ABCDEF_52459	UniRef50_Q9LU36	0.0	Normal: 157	704	147	540	677	726	BlastAnno: 1521	UniRef50_Q9LU36	570	65.20	431	139	3	9	1274	136	564	0.0	554
ABCDEF_41628	UniRef50_Q9LU36	0.0	Normal: 140	1561	151	856	1760	1204	BlastAnno: 2088	UniRef50_Q9LU36	570	60.04	548	203	5	152	1753	21	566	0.0	660
6.2.1.26(o-succinylbenzoate---CoA ligase;)																					
ABCDEF_44022	UniRef50_B9RQJ6	0.0	Normal: 313	183	386	1078	503	658	BlastAnno: 2067	UniRef50_B9RQJ6	545	69.12	544	159	5	75	1685	1	542	0.0	757
ABCDEF_44020	UniRef50_B9RQJ6	0.0	Normal: 344	202	421	1059	572	695	BlastAnno: 2461	UniRef50_B9RQJ6	545	69.12	544	159	5	469	2079	1	542	0.0	758
ABCDEF_13237	UniRef50_B9RQJ6	0.0	Normal: 5	128	3	8	9	142	BlastAnno: 2322	UniRef50_B9RQJ6	545	75.72	313	75	1	1060	1998	231	542	0.0	496
ABCDEF_13245	UniRef50_B9RQJ6	0.0	Normal: 7	139	5	8	12	141	BlastAnno: 2142	UniRef50_B9RQJ6	545	65.52	554	170	6	155	1789	1	542	0.0	728
ABCDEF_44023	UniRef50_B9RQJ6	0.0	Normal: 319	155	356	980	488	630	BlastAnno: 2315	UniRef50_B9RQJ6	545	68.66	536	159	5	219	1805	1	534	0.0	739
ABCDEF_13239	UniRef50_B9RQJ6	0.0	Normal: 8	92	1	7	11	108	BlastAnno: 1737	UniRef50_B9RQJ6	545	67.71	446	124	5	1638	325	109	542	0.0	613
ABCDEF_84731	UniRef50_B9RQJ6	0.0	Normal: 6	136	4	9	12	140	BlastAnno: 2113	UniRef50_B9RQJ6	545	65.34	554	171	6	155	1789	1	542	0.0	728
ABCDEF_13236	UniRef50_B9RQJ6	0.0	Normal: 13	126	9	8	19	144	BlastAnno: 2285	UniRef50_B9RQJ6	545	75.40	313	76	1	1292	354	231	542	0.0	495
ABCDEF_44019	UniRef50_B9RQJ6	0.0	Normal: 229	132	310	771	378	503	BlastAnno: 1345	UniRef50_B9RQJ6	545	74.93	383	88	4	1279	152	161	542	0.0	591
ABCDEF_44024	UniRef50_B9RQJ6	0.0	Normal: 290	162	348	1000	458	625	BlastAnno: 2171	UniRef50_B9RQJ6	545	68.66	536	159	5	75	1661	1	534	0.0	739
ABCDEF_13246	UniRef50_B9RQJ6	0.0	Normal: 7	95	3	4	7	109	BlastAnno: 1766	UniRef50_B9RQJ6	545	67.71	446	124	5	100	1413	109	542	0.0	613
>00140 Steroid hormone biosynthesis																					
1.1.1.-(Oxidoreductases;)																					
ABCDEF_13453	UniRef50_Q9SYM5	0.0	Normal: 225	226	398	1950	2026	421	BlastAnno: 2578	UniRef50_Q9SYM5	669	86.61	672	87	2	298	2313	1	669	0.0	1226
ABCDEF_17342	UniRef50_Q84M96	0.0	Normal: 274	406	272	327	439	625	BlastAnno: 1277	UniRef50_Q84M96	346	73.33	345	91	1	1187	156	2	346	0.0	546

	ABCDEF_31147	UniRef50_Q9SYM5	0.0	Normal: 4571	1591	1491	1401	1939	4327	BlastAnno: 2465	UniRef50_Q9SYM5	669	82.76	673	107	4	2397	394	1	669	0.0	1154
	ABCDEF_17344	UniRef50_Q84M96	0.0	Normal: 252	373	257	297	380	540	BlastAnno: 1350	UniRef50_Q84M96	346	68.19	371	91	2	1263	154	2	346	0.0	530
	ABCDEF_44674	UniRef50_Q9SYM5	0.0	Normal: 3581	2620	4329	9505	11447	4930	BlastAnno: 2634	UniRef50_Q9SYM5	669	87.80	672	78	3	253	2265	1	669	0.0	1241
1.1.1.145(3beta-hydroxy-Delta5-steroid dehydrogenase;)																						
	No Correspond Unigene																					
1.1.1.146(11beta-hydroxysteroid dehydrogenase;)																						
	No Correspond Unigene																					
1.1.1.148(estradiol 17alpha-dehydrogenase;)																						
	No Correspond Unigene																					
1.1.1.149(20alpha-hydroxysteroid dehydrogenase;)																						
	No Correspond Unigene																					
1.1.1.152(3alpha-hydroxy-5beta-androstane-17-one 3alpha-dehydrogenase;)																						
	No Correspond Unigene																					
1.1.1.213(3alpha-hydroxysteroid dehydrogenase (A-specific))																						
	No Correspond Unigene																					
1.1.1.239(3alpha(17beta)-hydroxysteroid dehydrogenase (NAD+);)																						
	No Correspond Unigene																					
1.1.1.50(3alpha-hydroxysteroid dehydrogenase (B-specific);)																						
	No Correspond Unigene																					
1.1.1.51(3(or 17)beta-hydroxysteroid dehydrogenase;)																						
	No Correspond Unigene																					
1.1.1.53(3alpha(or 20beta)-hydroxysteroid dehydrogenase;)																						
	No Correspond Unigene																					
1.1.1.62(estradiol 17beta-dehydrogenase;)																						
	No Correspond Unigene																					
1.1.1.63(testosterone 17beta-dehydrogenase;)																						
	No Correspond Unigene																					
1.1.1.64(testosterone 17beta-dehydrogenase (NADP+);)																						
	No Correspond Unigene																					
1.14.13.100(25-hydroxycholesterol 7alpha-hydroxylase;)																						
	No Correspond Unigene																					
1.14.13.17(cholesterol 7alpha-monooxygenase;)																						
	No Correspond Unigene																					
1.14.14.1(unspecific monooxygenase;)																						
	ABCDEF_41955	UniRef50_B9S0F8	2e-174	Normal: 70	140	28	0	0	150	BlastAnno: 1118	UniRef50_B9S0F8	506	66.67	366	115	5	2	1099	138	496	2e-174	504
1.14.15.4(steroid 11beta-monooxygenase;)																						
	No Correspond Unigene																					
1.14.15.5(corticosterone 18-monooxygenase;)																						
	No Correspond Unigene																					
1.14.15.6(cholesterol monooxygenase (side-chain-cleaving);)																						
	No Correspond Unigene																					
1.14.99.10(steroid 21-monooxygenase;)																						
	No Correspond Unigene																					
1.14.99.11(estradiol 6beta-monooxygenase;)																						
	No Correspond Unigene																					
1.14.99.12(androst-4-ene-3,17-dione monooxygenase;)																						
	No Correspond Unigene																					
1.14.99.14(progesterone 11alpha-monooxygenase;)																						
	No Correspond Unigene																					
1.14.99.9(steroid 17alpha-monooxygenase;)																						
	No Correspond Unigene																					
1.3.1.3(Delta4-3-oxosteroid 5beta-reductase;)																						
	No Correspond Unigene																					
1.3.1.30(progesterone 5alpha-reductase;)																						
	No Correspond Unigene																					
1.3.99.5(3-oxo-5alpha-steroid 4-dehydrogenase;)																						
	ABCDEF_49415	UniRef50_A4HEG5	2e-49	Normal: 2476	2826	3078	6091	6485	5019	BlastAnno: 1621	UniRef50_A4HEG5	309	34.38	317	187	7	1477	557	1	306	2e-49	180
1.3.99.6(3-oxo-5beta-steroid 4-dehydrogenase;)																						
	No Correspond Unigene																					
2.1.1.6(catechol O-methyltransferase;)																						
	ABCDEF_3867	UniRef50_Q9ZTU1	4e-129	Normal: 43	1755	68	456	3254	474	BlastAnno: 1421	UniRef50_Q9ZTU1	351	51.57	351	167	2	1306	260	2	351	4e-129	387
2.4.1.17(glucuronosyltransferase;)																						
	No Correspond Unigene																					
2.8.2.15(steroid sulfotransferase;)																						
	No Correspond Unigene																					
2.8.2.2(alcohol sulfotransferase;)																						
	ABCDEF_50672	UniRef50_B9SUK2	6e-80	Normal: 233	351	355	724	181	516	BlastAnno: 1433	UniRef50_B9SUK2	317	45.26	285	138	6	1194	343	39	306	6e-80	259
2.8.2.4(estrone sulfotransferase;)																						
	ABCDEF_60620	UniRef50_B9RE91	1e-103	Normal: 240	393	445	400	902	151	BlastAnno: 1191	UniRef50_B9RE91	354	50.59	340	156	5	31	1050	27	354	1e-103	319
3.1.6.1(arylsulfatase;)																						
	ABCDEF_89419	UniRef50_B6KE10	4e-08	Normal: 15	7	10	10	17	150	BlastAnno: 346	UniRef50_B6KE10	750	46.94	49	26	0	316	170	694	742	4e-08	56.6
3.1.6.2(steryl-sulfatase;)																						

No Correspond Unigene																					
4.1.2.30(17alpha-hydroxyprogesterone aldolase;)																					
No Correspond Unigene																					
5.3.3.1(steroid Delta-isomerase;)																					
No Correspond Unigene																					
>00190 Oxidative phosphorylation																					
1.10.2.2(ubiquinol---cytochrome-c reductase;)																					
ABCDEF_14897	UniRef50_P49727	2e-131	Normal: 1092	665	1467	2166	3319	1515	BlastAnno: 1310	UniRef50_P49727	273	75.29	255	56	4	1104	361	19	273	2e-131	389
1.3.5.1(succinate dehydrogenase (ubiquinone);)																					
ABCDEF_47748	UniRef50_Q4N0B9	0.0	Normal: 903	791	1034	2782	3604	958	BlastAnno: 2360	UniRef50_Q4N0B9	658	59.87	628	201	7	2104	365	13	637	0.0	682
ABCDEF_47751	UniRef50_Q4N0B9	0.0	Normal: 776	655	916	2143	2907	810	BlastAnno: 2266	UniRef50_Q4N0B9	658	59.87	628	201	7	189	1928	13	637	0.0	682
ABCDEF_47750	UniRef50_Q4N0B9	0.0	Normal: 1478	889	1772	2151	3099	1241	BlastAnno: 2236	UniRef50_Q4N0B9	658	59.71	628	202	6	2012	273	13	637	0.0	684
1.3.99.1(succinate dehydrogenase;)																					
ABCDEF_47748	UniRef50_Q6F8K9	0.0	Normal: 903	791	1034	2782	3604	958	BlastAnno: 2360	UniRef50_Q6F8K9	632	51.24	603	252	8	1990	290	36	632	0.0	584
ABCDEF_47751	UniRef50_Q6F8K9	0.0	Normal: 776	655	916	2143	2907	810	BlastAnno: 2266	UniRef50_Q6F8K9	632	51.24	603	252	8	303	2003	36	632	0.0	585
ABCDEF_47750	UniRef50_Q6F8K9	0.0	Normal: 1478	889	1772	2151	3099	1241	BlastAnno: 2236	UniRef50_Q6F8K9	632	51.00	602	255	7	1898	198	36	632	0.0	571
1.6.5.3(NADH dehydrogenase (ubiquinone);)																					
ABCDEF_25016	UniRef50_Q23KA9	0.0	Normal: 1899	1165	2206	3428	4601	1707	BlastAnno: 2606	UniRef50_Q23KA9	718	51.85	677	314	7	310	2316	33	705	0.0	689
ABCDEF_25528	UniRef50_P49821	0.0	Normal: 3549	2878	3730	7113	10089	5235	BlastAnno: 2135	UniRef50_P49821	464	76.65	424	99	0	1766	495	28	451	0.0	683
1.6.99.3(NADH dehydrogenase;)																					
ABCDEF_10653	UniRef50_Q4Q0M3	1e-104	Normal: 347	349	328	680	1061	482	BlastAnno: 1723	UniRef50_Q4Q0M3	527	40.69	467	252	9	47	1426	11	459	1e-104	333
1.6.99.5(NADH dehydrogenase (quinone);)																					
ABCDEF_25016	UniRef50_Q2GK08	0.0	Normal: 1899	1165	2206	3428	4601	1707	BlastAnno: 2606	UniRef50_Q2GK08	692	47.10	690	346	4	310	2325	2	690	0.0	612
ABCDEF_25528	UniRef50_A8GM77	0.0	Normal: 3549	2878	3730	7113	10089	5235	BlastAnno: 2135	UniRef50_A8GM77	423	69.93	419	120	4	1745	495	2	416	0.0	612
1.9.3.1(cytochrome-c oxidase;)																					
ABCDEF_9208	UniRef50_P00395	1e-112	Normal: 0	34	0	0	7	380	BlastAnno: 1179	UniRef50_P00395	513	62.30	374	141	0	56	1177	84	457	1e-112	347
2.7.4.1(polyphosphate kinase;)																					
No Correspond Unigene																					
3.6.1.1(inorganic diphosphatase)																					
ABCDEF_20116	UniRef50_P31414	0.0	Normal: 630	882	545	2272	3365	1093	BlastAnno: 1040	UniRef50_P31414	770	81.85	314	54	1	933	1	1	314	0.0	531
ABCDEF_31327	UniRef50_Q56ZN6	0.0	Normal: 755	523	847	1414	2499	1054	BlastAnno: 2026	UniRef50_Q56ZN6	802	89.66	532	55	0	2	1597	271	802	0.0	894
ABCDEF_24196	UniRef50_P31414	0.0	Normal: 978	641	1016	2430	2729	1863	BlastAnno: 1582	UniRef50_P31414	770	93.19	411	28	0	3	1235	360	770	0.0	752
ABCDEF_20117	UniRef50_Q231W2	0.0	Normal: 1500	737	1665	2499	4244	751	BlastAnno: 1832	UniRef50_Q231W2	772	53.35	641	248	9	1829	3	80	701	0.0	615
ABCDEF_20115	UniRef50_Q231W2	0.0	Normal: 1844	2598	2369	6360	9952	3026	BlastAnno: 2052	UniRef50_Q231W2	772	58.04	634	232	7	2052	250	131	763	0.0	669
ABCDEF_20114	UniRef50_Q231W2	0.0	Normal: 3849	5919	4377	14298	21318	7030	BlastAnno: 2688	UniRef50_Q231W2	772	53.11	772	301	13	2518	302	18	761	0.0	726
ABCDEF_24197	UniRef50_P31414	0.0	Normal: 491	312	381	1119	1386	912	BlastAnno: 1051	UniRef50_P31414	770	94.00	350	21	0	1050	1	351	700	0.0	640
3.6.3.10(H+/K+-exchanging ATPase;)																					
ABCDEF_85005	UniRef50_Q9VFE5	8e-23	Normal: 0	0	0	0	0	10	BlastAnno: 279	UniRef50_Q9VFE5	212	63.74	91	33	0	2	274	119	209	8e-23	94.4
3.6.3.14(H+-transporting two-sector ATPase;)																					
ABCDEF_38143	UniRef50_C8W8D3	0.0	Normal: 188	240	462	512	364	584	BlastAnno: 1786	UniRef50_C8W8D3	557	53.40	530	217	5	1650	121	7	526	0.0	544
ABCDEF_750	UniRef50_Q3A946	0.0	Normal: 8200	4736	9322	16172	23565	9462	BlastAnno: 2234	UniRef50_Q3A946	473	71.62	451	111	3	441	1766	18	460	0.0	657
ABCDEF_31686	UniRef50_Q3A946	0.0	Normal: 4796	2678	5669	8971	9245	4913	BlastAnno: 2182	UniRef50_Q3A946	473	71.43	448	111	3	448	1764	18	457	0.0	650
ABCDEF_81842	UniRef50_P06576	0.0	Normal: 0	6	0	0	0	192	BlastAnno: 868	UniRef50_P06576	529	88.93	289	32	0	1	867	239	527	0.0	525
ABCDEF_6666	UniRef50_B8ZR40	0.0	Normal: 131	564	209	94	213	1741	BlastAnno: 2588	UniRef50_B8ZR40	558	57.06	503	206	2	772	2253	4	505	0.0	553
ABCDEF_44101	UniRef50_A2BKX6	0.0	Normal: 1138	1254	1508	2332	4942	1782	BlastAnno: 2483	UniRef50_A2BKX6	601	49.75	599	285	5	2117	327	5	589	0.0	576
ABCDEF_6665	UniRef50_C3W009	0.0	Normal: 363	1495	714	290	657	5105	BlastAnno: 3324	UniRef50_C3W009	507	71.65	462	131	0	1386	1	1	462	0.0	597
ABCDEF_44104	UniRef50_A2BKX6	0.0	Normal: 1709	1358	1590	2988	6608	3458	BlastAnno: 2470	UniRef50_A2BKX6	601	49.92	599	284	5	252	2042	5	589	0.0	562
ABCDEF_38059	UniRef50_Q3A946	0.0	Normal: 7449	20106	6978	6819	10389	50205	BlastAnno: 3486	UniRef50_Q3A946	473	70.43	460	124	2	3220	1856	18	470	0.0	659
ABCDEF_64176	UniRef50_P38606	0.0	Normal: 625	683	721	1302	2664	866	BlastAnno: 1815	UniRef50_P38606	617	75.20	496	121	2	330	1814	11	505	0.0	749
3.6.3.6(H+-exporting ATPase;)																					
ABCDEF_44312	UniRef50_P54211	0.0	Normal: 2182	2038	1959	4417	4319	3925	BlastAnno: 3226	UniRef50_P54211	1131	47.99	748	356	9	3047	864	30	764	0.0	586
ABCDEF_44314	UniRef50_P54211	0.0	Normal: 2893	3013	2474	5294	4246	5084	BlastAnno: 3442	UniRef50_P54211	1131	47.86	748	357	9	3048	865	30	764	0.0	604
ABCDEF_25612	UniRef50_Q9SJB3	0.0	Normal: 126	444	102	611	2163	508	BlastAnno: 945	UniRef50_Q9SJB3	949	86.31	314	43	0	943	2	207	520	0.0	578
ABCDEF_44315	UniRef50_P54211	0.0	Normal: 3513	2183	3696	12992	20135	5804	BlastAnno: 3554	UniRef50_P54211	1131	43.67	884	401	14	3327	928	30	900	0.0	590
ABCDEF_51718	UniRef50_B9RD09	0.0	Normal: 682	1552	1132	5228	8316	1319	BlastAnno: 2211	UniRef50_B9RD09	874	70.40	581	127	3	1	1734	336	874	0.0	774
ABCDEF_51719	UniRef50_B9RD09	0.0	Normal: 273	1121	323	1572	6028	1515	BlastAnno: 2041	UniRef50_B9RD09	874	74.76	527	129	3	114	1685	349	874	0.0	768
>00195 Photosynthesis																					
1.10.99.1(plastoquinol---plastocyanin reductase;)																					
ABCDEF_35605	UniRef50_C6TFS5	3e-100	Normal: 1521	5835	1095	1215	2271	20667	BlastAnno: 1186	UniRef50_C6TFS5	262	77.03	222	49	2	177	842	1	220	3e-100	307
1.18.1.2(ferredoxin---NADP+ reductase;)																					
ABCDEF_45849	UniRef50_Q9FKW6	0.0	Normal: 2520	11904	1714	2296	3351	38632	BlastAnno: 1521	UniRef50_Q9FKW6	360	83.58	341	46	2	1172	162	26	360	0.0	568
ABCDEF_45511	UniRef50_O04397	0.0	Normal: 1034	387	1453	2959	3056	602	BlastAnno: 1557	UniRef50_O04397	375	85.41	377	51	3	1327	200	2	375	0.0	662
ABCDEF_10881	UniRef50_O04397	0.0	Normal: 969	430	973	1263	1457	836	BlastAnno: 1698	UniRef50_O04397	375	85.45	378	50	4	1435	308	1	375	0.0	665
3.6.3.14(H+-transporting two-sector ATPase;)																					
ABCDEF_38143	UniRef50_C8W8D3	0.0	Normal: 188	240	462	512	364	584	BlastAnno: 1786	UniRef50_C8W8D3	557	53.40	530	217	5	1650	121	7	526	0.0	544
ABCDEF_750	UniRef50_Q3A946	0.0	Normal: 8200	4736	9322	16172	23565	9462	BlastAnno: 2234	UniRef50_Q3A946	473	71.62	451	111	3	441	1766	18	460	0.0	657
ABCDEF_31686	UniRef50_Q3A946	0.0	Normal: 4796	2678	5669	8971	9245	4913	BlastAnno: 2182	UniRef50_Q3A946	473	71.43	448	111	3	448	1764	18	457	0.0	650
ABCDEF_81842	UniRef50_P06576	0.0	Normal: 0	6	0	0	0	192	BlastAnno: 868	UniRef50_P06576	529	88.93	289	32	0	1	867	239	527	0.0	525
ABCDEF_6666	UniRef50_B8ZR40	0.0	Normal: 131	564	209	94	213	1741	BlastAnno: 2588	UniRef50_B8ZR40	558	57.06	503	206	2	772	2253	4	505	0.0	553
ABCDEF_44101	UniRef50_A2BKX6	0.0	Normal: 1138	1254	1508	2332	4942	1782	BlastAnno: 2483	UniRef50_A2BKX6	601	49.75	599	285	5	2117	327	5	589	0.0	576
ABCDEF_6665	UniRef50_C3W009	0.0	Normal: 363	1495	714	290	657	5105	BlastAnno: 3324	UniRef50_C3W009	507	71.65	462	131	0	1386	1	1	462	0.0	597
ABCDEF_44104	UniRef50_A2BKX6	0.0	Normal: 1709	1358	1590	2988	6608	3458	BlastAnno: 2470	UniRef50_A2BKX6	601	49.92	599	284	5	252	2042	5	589	0.0	562

ABCDEF_38059	UniRef50_Q3A946	0.0	Normal: 7449	20106	6978	6819	10389	50205	BlastAnno: 3486	UniRef50_Q3A946	473	70.43	460	124	2	3220	1856	18	470	0.0	659
ABCDEF_64176	UniRef50_P38606	0.0	Normal: 625	683	721	1302	2664	866	BlastAnno: 1815	UniRef50_P38606	617	75.20	496	121	2	330	1814	11	505	0.0	749
>00196 Photosynthesis - antenna proteins																					
4.-.-.-(Lyases)																					
ABCDEF_41738	UniRef50_A1UF85	2e-114	Normal: 1096	1526	732	626	2607	1174	BlastAnno: 1351	UniRef50_A1UF85	322	67.22	299	93	2	1186	293	26	320	2e-114	347
>00230 Purine metabolism																					
1.1.1.154(ureidoglycolate dehydrogenase)																					
No Correspond Unigene																					
1.1.1.205(IMP dehydrogenase;)																					
ABCDEF_41932	UniRef50_P47996	0.0	Normal: 2145	767	2380	2842	2987	1396	BlastAnno: 1847	UniRef50_P47996	503	66.34	508	157	6	1726	221	4	503	0.0	636
1.17.1.4(xanthine dehydrogenase;)																					
ABCDEF_13812	UniRef50_Q00519	0.0	Normal: 571	475	518	776	1049	714	BlastAnno: 2920	UniRef50_Q00519	1335	50.71	913	426	8	2912	207	418	1317	0.0	858
1.17.3.2(xanthine oxidase;)																					
No Correspond Unigene																					
1.17.4.1(ribonucleoside-diphosphate reductase;)																					
ABCDEF_20810	UniRef50_C5DY80	0.0	Normal: 2798	417	1493	203	646	708	BlastAnno: 2909	UniRef50_C5DY80	875	54.51	776	314	10	2660	405	25	785	0.0	811
ABCDEF_51669	UniRef50_C5DY80	0.0	Normal: 2176	202	1063	25	233	660	BlastAnno: 2851	UniRef50_C5DY80	875	54.12	776	317	9	2594	339	25	785	0.0	815
1.17.4.2(ribonucleoside-triphosphate reductase;)																					
No Correspond Unigene																					
1.7.1.7(GMP reductase;)																					
No Correspond Unigene																					
1.7.3.3(factor-independent urate hydroxylase;)																					
ABCDEF_25588	UniRef50_O04420	2e-147	Normal: 932	1081	941	2032	2640	744	BlastAnno: 1309	UniRef50_O04420	309	71.05	304	87	1	77	985	6	309	2e-147	431
2.1.2.2(phosphoribosylglycinamide formyltransferase;)																					
ABCDEF_21522	UniRef50_P52422	3e-110	Normal: 231	105	226	159	220	202	BlastAnno: 1269	UniRef50_P52422	292	72.69	216	58	1	904	260	76	291	3e-110	335
2.1.2.3(phosphoribosylaminoimidazolecarboxamide formyltransferase;)																					
ABCDEF_31348	UniRef50_A3DEU9	5e-160	Normal: 1610	679	1784	1352	1383	1416	BlastAnno: 2145	UniRef50_A3DEU9	514	46.50	529	261	7	1878	307	3	514	5e-160	481
2.1.2.4(glycine formimidoyltransferase;)																					
No Correspond Unigene																					
2.1.3.5(oxamate carbamoyltransferase;)																					
No Correspond Unigene																					
2.4.2.-(Transferases;)																					
ABCDEF_17257	UniRef50_Q940Q8	0.0	Normal: 176	142	236	252	196	151	BlastAnno: 1259	UniRef50_Q940Q8	415	91.67	396	33	0	1259	72	20	415	0.0	734
ABCDEF_25833	UniRef50_Q940Q8	0.0	Normal: 28	336	34	93	151	84	BlastAnno: 958	UniRef50_Q940Q8	415	88.89	306	34	0	957	40	110	415	0.0	590
ABCDEF_21016	UniRef50_B8LDL7	0.0	Normal: 775	334	669	635	559	950	BlastAnno: 2086	UniRef50_B8LDL7	570	56.33	561	212	10	1820	234	8	567	0.0	595
ABCDEF_48436	UniRef50_Q940Q8	0.0	Normal: 603	624	571	1077	1211	656	BlastAnno: 1898	UniRef50_Q940Q8	415	90.71	409	37	1	1515	292	7	415	0.0	744
2.4.2.1(purine-nucleoside phosphorylase;)																					
No Correspond Unigene																					
2.4.2.14(amidophosphoribosyltransferase;)																					
ABCDEF_521	UniRef50_P52418	0.0	Normal: 906	973	705	157	354	756	BlastAnno: 2055	UniRef50_P52418	569	81.84	501	83	7	273	1757	60	558	0.0	820
2.4.2.15(guanosine phosphorylase)																					
No Correspond Unigene																					
2.4.2.16(urate-ribonucleotide phosphorylase;)																					
No Correspond Unigene																					
2.4.2.22(xanthine phosphoribosyltransferase;)																					
No Correspond Unigene																					
2.4.2.7(adenine phosphoribosyltransferase;)																					
ABCDEF_24232	UniRef50_Q42563	1e-100	Normal: 77	13	106	429	49	89	BlastAnno: 731	UniRef50_Q42563	192	75.66	189	46	0	694	128	1	189	1e-100	299
2.4.2.8(hypoxanthine phosphoribosyltransferase;)																					
ABCDEF_18032	UniRef50_Q839B2	9e-48	Normal: 200	59	206	154	207	86	BlastAnno: 780	UniRef50_Q839B2	181	42.78	194	96	2	748	167	1	179	9e-48	164
2.7.1.113(deoxyguanosine kinase;)																					
No Correspond Unigene																					
2.7.1.20(adenosine kinase;)																					
ABCDEF_1273	UniRef50_Q9LZG0	0.0	Normal: 2238	1924	2770	6003	8780	3466	BlastAnno: 1725	UniRef50_Q9LZG0	345	83.38	337	56	0	1397	387	8	344	0.0	603
ABCDEF_37634	UniRef50_B9S254	0.0	Normal: 158	266	194	254	333	1005	BlastAnno: 1934	UniRef50_B9S254	483	78.95	361	76	0	1401	319	116	476	0.0	597
ABCDEF_7779	UniRef50_B9S254	0.0	Normal: 964	1561	771	814	1006	5937	BlastAnno: 1846	UniRef50_B9S254	483	81.97	366	66	0	1225	128	118	483	0.0	618
ABCDEF_35688	UniRef50_Q9LZG0	0.0	Normal: 936	1031	1179	2361	5308	2283	BlastAnno: 1438	UniRef50_Q9LZG0	345	81.71	339	62	0	1217	201	7	345	0.0	592
ABCDEF_80000	UniRef50_Q9LZG0	0.0	Normal: 1040	1078	1420	2841	5012	2256	BlastAnno: 1410	UniRef50_Q9LZG0	345	82.54	338	59	0	1183	170	8	345	0.0	595
ABCDEF_80547	UniRef50_Q9LZG0	0.0	Normal: 1727	1588	2234	4810	6987	2656	BlastAnno: 1565	UniRef50_Q9LZG0	345	82.30	339	60	0	1389	373	7	345	0.0	599
2.7.1.25(adenylyl-sulfate kinase;)																					
ABCDEF_31787	UniRef50_B8CBW8	2e-117	Normal: 1065	1161	1322	2258	1986	3606	BlastAnno: 2042	UniRef50_B8CBW8	968	46.74	430	200	5	1602	397	310	738	2e-117	383
2.7.1.40(pyruvate kinase;)																					
ABCDEF_27872	UniRef50_Q40545	0.0	Normal: 1220	1217	1378	1640	1974	4469	BlastAnno: 2233	UniRef50_Q40545	593	81.65	594	96	4	1951	206	1	593	0.0	979
ABCDEF_34901	UniRef50_B9EYF5	0.0	Normal: 3919	1884	3179	3852	4333	5138	BlastAnno: 2384	UniRef50_B9EYF5	606	80.36	555	84	2	2021	432	52	606	0.0	922
ABCDEF_10813	UniRef50_Q2QXR7	0.0	Normal: 2566	2379	2864	4497	6222	4202	BlastAnno: 2019	UniRef50_Q2QXR7	408	85.57	402	58	0	174	1379	1	402	0.0	727
ABCDEF_35590	UniRef50_Q9M057	0.0	Normal: 2212	2122	2749	5601	7830	2637	BlastAnno: 1980	UniRef50_Q9M057	510	69.58	503	148	4	1830	334	1	502	0.0	687
ABCDEF_27885	UniRef50_Q2QXR7	0.0	Normal: 924	752	1120	2229	3166	928	BlastAnno: 2102	UniRef50_Q2QXR7	408	87.31	402	51	0	182	1387	1	402	0.0	729
ABCDEF_21818	UniRef50_Q40545	0.0	Normal: 1982	1309	1439	2275	1999	2303	BlastAnno: 2062	UniRef50_Q40545	593	69.25	491	147	2	1594	134	103	593	0.0	716
ABCDEF_31300	UniRef50_Q40545	0.0	Normal: 1326	1019	1016	1433	1396	1865	BlastAnno: 1805	UniRef50_Q40545	593	91.70	494	41	0	1802	321	100	593	0.0	920
ABCDEF_45529	UniRef50_B9EYF5	0.0	Normal: 1474	625	1452	3814	2571	3121	BlastAnno: 2114	UniRef50_B9EYF5	606	78.56	569	89	3	1810	179	46	606	0.0	912
ABCDEF_45536	UniRef50_Q2QXR7	0.0	Normal: 1644	1099	1596	1290	5936	335	BlastAnno: 1959	UniRef50_Q2QXR7	408	81.59	402	74	0	1862	657	1	402	0.0	687
ABCDEF_27886	UniRef50_Q2QXR7	0.0	Normal: 771	640	954	1807	2731	814	BlastAnno: 1787	UniRef50_Q2QXR7	408	86.82	349	46	0	54	1100	54	402	0.0	632

ABCDEF_18187	UniRef50_Q9M057	0.0	Normal: 3137	1498	3397	5489	6703	1765	BlastAnno: 1912	UniRef50_Q9M057	510	67.21	494	158	3	216	1685	17	510	0.0	681
ABCDEF_45028	UniRef50_B9EYF5	0.0	Normal: 273	415	192	360	372	144	BlastAnno: 1785	UniRef50_B9EYF5	606	73.94	495	105	2	372	1784	111	605	0.0	729
2.7.1.73(inosine kinase;)																					
No Correspond Unigene																					
2.7.1.74(deoxycytidine kinase;)																					
No Correspond Unigene																					
2.7.1.76(deoxyadenosine kinase;)																					
No Correspond Unigene																					
2.7.2.2(carbamate kinase;)																					
No Correspond Unigene																					
2.7.4.11(No Name)																					
No Correspond Unigene																					
2.7.4.3(adenylate kinase;)																					
ABCDEF_44532	UniRef50_B9SC22	0.0	Normal: 219	680	166	190	101	1550	BlastAnno: 2258	UniRef50_B9SC22	597	76.31	536	126	1	1967	360	63	597	0.0	863
2.7.4.6(nucleoside-diphosphate kinase;)																					
ABCDEF_1220	UniRef50_O49203	3e-121	Normal: 1836	1610	2747	4707	8156	4732	BlastAnno: 1189	UniRef50_O49203	238	75.42	240	56	3	151	867	1	238	3e-121	360
2.7.4.8(guanylate kinase;)																					
ABCDEF_10178	UniRef50_Q54XF2	7e-70	Normal: 43	51	62	88	122	89	BlastAnno: 992	UniRef50_Q54XF2	202	55.10	196	88	0	395	982	7	202	7e-70	224
2.7.6.1(ribose-phosphate diphosphokinase;)																					
ABCDEF_48760	UniRef50_Q42581	0.0	Normal: 973	566	683	870	1272	1444	BlastAnno: 1493	UniRef50_Q42581	403	82.77	354	55	3	145	1206	48	395	0.0	596
ABCDEF_48326	UniRef50_Q93Z66	0.0	Normal: 385	227	399	743	698	724	BlastAnno: 1568	UniRef50_Q93Z66	411	72.70	381	99	3	1352	213	35	411	0.0	546
ABCDEF_38177	UniRef50_Q680A5	0.0	Normal: 875	249	761	568	782	550	BlastAnno: 1490	UniRef50_Q680A5	337	82.07	329	58	1	1198	212	10	337	0.0	562
ABCDEF_48346	UniRef50_Q42581	0.0	Normal: 106	135	82	88	251	542	BlastAnno: 1942	UniRef50_Q42581	403	81.59	364	62	3	1743	667	39	402	0.0	611
2.7.6.5(GTP diphosphokinase;)																					
ABCDEF_36960	UniRef50_B9S1Z6	0.0	Normal: 554	933	413	424	556	1916	BlastAnno: 2926	UniRef50_B9S1Z6	806	65.21	871	221	10	2786	222	1	805	0.0	1025
2.7.7.4(sulfate adenylyltransferase;)																					
ABCDEF_10695	UniRef50_B6KN22	0.0	Normal: 1568	429	1310	1338	1351	785	BlastAnno: 3382	UniRef50_B6KN22	1008	53.54	1003	431	10	3195	274	5	1001	0.0	1061
ABCDEF_48012	UniRef50_B6KBK6	0.0	Normal: 909	375	986	1182	1437	436	BlastAnno: 2606	UniRef50_B6KBK6	877	54.57	722	296	6	2316	247	148	869	0.0	791
ABCDEF_38032	UniRef50_Q43870	0.0	Normal: 611	1036	642	1131	1509	1791	BlastAnno: 1947	UniRef50_Q43870	476	73.00	426	115	0	1636	359	42	467	0.0	661
ABCDEF_31663	UniRef50_Q43870	0.0	Normal: 1022	1780	1065	1614	1663	3264	BlastAnno: 1893	UniRef50_Q43870	476	74.21	411	106	0	1723	491	57	467	0.0	663
ABCDEF_31787	UniRef50_Q43870	0.0	Normal: 1065	1161	1322	2258	1986	3606	BlastAnno: 2042	UniRef50_Q43870	476	76.60	483	106	3	1809	361	1	476	0.0	771
ABCDEF_10694	UniRef50_B6KN22	0.0	Normal: 1188	323	1053	981	1089	607	BlastAnno: 2848	UniRef50_B6KN22	1008	55.54	884	365	7	21	2588	118	1001	0.0	1015
2.7.7.48(RNA-directed RNA polymerase;)																					
ABCDEF_34034	UniRef50_O82504	0.0	Normal: 1488	604	1118	941	1189	712	BlastAnno: 3758	UniRef50_O82504	1133	59.59	1111	438	7	86	3406	10	1113	0.0	1348
ABCDEF_6896	UniRef50_Q8LHH9	0.0	Normal: 551	202	525	1060	1244	330	BlastAnno: 2486	UniRef50_Q8LHH9	1218	63.41	757	272	3	2483	219	465	1218	0.0	996
2.7.7.53(ATP adenylyltransferase;)																					
No Correspond Unigene																					
2.7.7.6(DNA-directed RNA polymerase;)																					
ABCDEF_41473	UniRef50_B9GKP9	0.0	Normal: 331	132	133	251	174	146	BlastAnno: 1665	UniRef50_B9GKP9	1517	55.25	581	223	5	1637	3	201	780	0.0	570
ABCDEF_52641	UniRef50_Q8L6J2	0.0	Normal: 258	64	314	187	213	114	BlastAnno: 1303	UniRef50_Q8L6J2	332	83.88	304	49	0	1302	391	29	332	0.0	551
ABCDEF_6430	UniRef50_A9UY22	0.0	Normal: 1077	541	777	657	784	387	BlastAnno: 3519	UniRef50_A9UY22	1592	42.33	1004	486	21	3288	424	271	1230	0.0	752
ABCDEF_38289	UniRef50_Q9LK40	0.0	Normal: 3533	1236	2709	3045	2599	2113	BlastAnno: 4120	UniRef50_Q9LK40	1172	65.91	1185	362	10	356	3871	16	1171	0.0	1566
ABCDEF_47593	UniRef50_B9SHS2	0.0	Normal: 1241	283	1007	794	885	416	BlastAnno: 4025	UniRef50_B9SHS2	1139	79.23	1146	221	3	3890	456	10	1139	0.0	1819
ABCDEF_17969	UniRef50_P56764	0.0	Normal: 59	89	176	20	28	798	BlastAnno: 1479	UniRef50_P56764	1376	65.04	492	158	6	1473	1	484	962	0.0	574
ABCDEF_14408	UniRef50_Q5KIB7	0.0	Normal: 3327	1620	2819	3121	2847	2381	BlastAnno: 3999	UniRef50_Q5KIB7	1193	56.06	1204	417	15	3705	238	49	1188	0.0	1315
ABCDEF_3729	UniRef50_Q9LFV6	0.0	Normal: 229	56	187	161	198	53	BlastAnno: 1749	UniRef50_Q9LFV6	1011	66.26	575	193	1	1724	3	168	742	0.0	804
ABCDEF_4016	UniRef50_B9G6G0	0.0	Normal: 416	120	403	152	266	64	BlastAnno: 1471	UniRef50_B9G6G0	1150	68.30	407	117	4	2	1213	743	1140	0.0	553
ABCDEF_37325	UniRef50_A8IRZ2	0.0	Normal: 1508	1018	1110	2111	1134	1174	BlastAnno: 3913	UniRef50_A8IRZ2	1789	56.47	1259	494	12	3708	13	1	1232	0.0	1405
ABCDEF_37205	UniRef50_Q2XSM7	0.0	Normal: 620	278	467	527	379	735	BlastAnno: 3532	UniRef50_Q2XSM7	997	39.45	834	457	17	544	2988	193	997	0.0	589
ABCDEF_18114	UniRef50_C5YGY9	0.0	Normal: 2784	1475	2612	2568	2178	2591	BlastAnno: 2404	UniRef50_C5YGY9	1857	85.71	343	44	4	2402	1383	1195	1535	0.0	602
ABCDEF_20059	UniRef50_B9F2S6	0.0	Normal: 1614	564	810	1084	542	661	BlastAnno: 5256	UniRef50_B9F2S6	2017	44.85	1710	786	31	340	5076	3	1686	0.0	1310
ABCDEF_20332	UniRef50_B8C4M1	0.0	Normal: 673	337	552	1076	603	549	BlastAnno: 3314	UniRef50_B8C4M1	1422	49.12	1132	525	17	1	3312	50	1158	0.0	1059
ABCDEF_4043	UniRef50_Q9LFV6	0.0	Normal: 164	28	131	91	64	28	BlastAnno: 1638	UniRef50_Q9LFV6	1011	66.01	456	152	2	269	1636	159	611	0.0	563
ABCDEF_10576	UniRef50_Q1L5X6	0.0	Normal: 546	239	446	510	567	478	BlastAnno: 1817	UniRef50_Q1L5X6	1138	59.70	536	211	4	1815	214	603	1135	0.0	625
2.7.7.7(DNA-directed DNA polymerase;)																					
ABCDEF_16323	UniRef50_O48653	0.0	Normal: 85	21	57	21	24	30	BlastAnno: 2598	UniRef50_O48653	1534	62.00	650	223	8	8	1903	244	887	0.0	790
ABCDEF_16314	UniRef50_B3RI52	0.0	Normal: 159	23	81	20	29	39	BlastAnno: 3553	UniRef50_B3RI52	1203	39.13	1099	524	28	3258	16	137	1108	0.0	700
ABCDEF_3831	UniRef50_Q9LVN7	0.0	Normal: 872	95	527	93	228	166	BlastAnno: 3372	UniRef50_Q9LVN7	1081	83.91	957	149	2	3372	502	61	1012	0.0	1560
ABCDEF_16320	UniRef50_B3RI52	0.0	Normal: 150	23	89	22	32	38	BlastAnno: 3613	UniRef50_B3RI52	1203	38.46	1118	525	28	296	3598	137	1108	0.0	690
ABCDEF_57123	UniRef50_Q9SGD5	0.0	Normal: 115	8	30	0	0	8	BlastAnno: 1036	UniRef50_Q9SGD5	2271	78.16	348	52	3	1	1026	528	857	0.0	565
ABCDEF_57314	UniRef50_O48653	0.0	Normal: 50	8	24	6	11	14	BlastAnno: 1773	UniRef50_O48653	1534	62.44	591	202	6	8	1726	244	832	0.0	728
ABCDEF_17221	UniRef50_O48520	0.0	Normal: 728	155	529	212	218	433	BlastAnno: 1641	UniRef50_O48520	456	74.34	456	99	3	1572	253	1	454	0.0	707
ABCDEF_48352	UniRef50_B9SQ81	0.0	Normal: 238	127	222	78	59	181	BlastAnno: 1927	UniRef50_B9SQ81	560	68.17	421	103	3	1	1248	52	446	0.0	572
ABCDEF_16321	UniRef50_B3RI52	0.0	Normal: 297	94	191	81	113	129	BlastAnno: 4416	UniRef50_B3RI52	1203	39.46	1044	490	28	1192	4269	273	1192	0.0	664
ABCDEF_16326	UniRef50_O48653	0.0	Normal: 216	127	206	114	121	136	BlastAnno: 3076	UniRef50_O48653	1534	62.00	650	223	8	3069	1174	244	887	0.0	790
ABCDEF_16319	UniRef50_C5XR76	0.0	Normal: 93	48	74	50	55	58	BlastAnno: 3092	UniRef50_C5XR76	1541	69.60	398	115	4	1144	2334	503	895	0.0	577
ABCDEF_51711	UniRef50_C5X4W2	0.0	Normal: 259	177	325	375	256	285	BlastAnno: 1806	UniRef50_C5X4W2	1714	67.79	534	152	3	1	1545	1182	1714	0.0	757
ABCDEF_16315	UniRef50_B3RI52	0.0	Normal: 173	48	108	57	52	72	BlastAnno: 4431	UniRef50_B3RI52	1203	39.83	964	435	24	2856	16	273	1108	0.0	627
ABCDEF_41802	UniRef50_Q9SGD5	0.0	Normal: 502	39	242	0	82	126	BlastAnno: 2596	UniRef50_Q9SGD5	2271	47.66	875	364	12	2	2416	1416	2266	0.0	720
ABCDEF_16322	UniRef50_B3RI52	0.0	Normal: 151	47	99	46	54	56	BlastAnno: 4047	UniRef50_B3RI52	1203	39.83	964	435	24	1192	4032	273	1108	0.0	627
ABCDEF_41210	UniRef50_B9RD63	0.0	Normal: 305	176	260	314	159	188	BlastAnno: 1568	UniRef50_B9RD63	1420	74.42	516	120	8	43	1566	769	1280	0.0	660
ABCDEF_16316	UniRef50_B3RI52	0.0	Normal: 172	41	103	47	47	63	BlastAnno: 4345	UniRef50_B3RI52	1203	40.68	944	435	23	1550	4330	273	1108	0.0	640

ABCDEF_16324	UniRef50_C5XR76	0.0	Normal: 86	44	67	46	46	48	BlastAnno: 3032	UniRef50_C5XR76	1541	69.60	398	115	4	1144	2334	503	895	0.0	577
2.7.7.8(polyribonucleotide nucleotidyltransferase;)																					
ABCDEF_27735	UniRef50_Q6MDI2	0.0	Normal: 1219	1128	977	1124	844	3456	BlastAnno: 3515	UniRef50_Q6MDI2	697	48.99	745	325	5	415	2649	5	694	0.0	683
3.1.3.5(5'-nucleotidase;)																					
ABCDEF_10187	UniRef50_A9F2C9	3e-85	Normal: 140	337	178	525	497	1064	BlastAnno: 2494	UniRef50_A9F2C9	618	34.02	535	278	12	792	2276	119	618	3e-85	292
3.1.3.6(3'-nucleotidase;)																					
No Correspond Unigene																					
3.1.4.16(2',3'-cyclic-nucleotide 2'-phosphodiesterase;)																					
No Correspond Unigene																					
3.1.4.17(3',5'-cyclic-nucleotide phosphodiesterase;)																					
No Correspond Unigene																					
3.1.4.35(3',5'-cyclic-GMP phosphodiesterase;)																					
No Correspond Unigene																					
3.1.4.53(3',5'-cyclic-AMP phosphodiesterase;)																					
No Correspond Unigene																					
3.1.5.1(dGTPase;)																					
No Correspond Unigene																					
3.1.7.2(guanosine-3',5'-bis(diphosphate) 3'-diphosphatase;)																					
ABCDEF_34721	UniRef50_P74007	2e-88	Normal: 872	938	537	1162	1230	1374	BlastAnno: 2769	UniRef50_P74007	760	46.50	357	179	4	893	1945	53	403	2e-88	306
3.2.2.1(purine nucleosidase;)																					
ABCDEF_52566	UniRef50_B9SSZ5	2e-160	Normal: 417	265	311	827	1190	504	BlastAnno: 1375	UniRef50_B9SSZ5	286	72.01	318	54	1	191	1144	1	283	2e-160	464
3.2.2.12(inosinate nucleosidase)																					
No Correspond Unigene																					
3.2.2.2(inosine nucleosidase;)																					
No Correspond Unigene																					
3.2.2.4(AMP nucleosidase;)																					
No Correspond Unigene																					
3.2.2.7(adenosine nucleosidase;)																					
No Correspond Unigene																					
3.2.2.8(ribosylpyrimidine nucleosidase;)																					
No Correspond Unigene																					
3.5.1.5(urease)																					
ABCDEF_31241	UniRef50_B2W555	0.0	Normal: 284	167	262	380	250	289	BlastAnno: 1705	UniRef50_B2W555	793	59.82	555	181	5	1703	45	279	793	0.0	647
3.5.2.-(Hydrolases;)																					
No Correspond Unigene																					
3.5.2.17(hydroxyisourate hydrolase;)																					
No Correspond Unigene																					
3.5.2.5(allantoinase)																					
ABCDEF_31977	UniRef50_Q2SMZ8	4e-37	Normal: 0	31	0	15	28	30	BlastAnno: 517	UniRef50_Q2SMZ8	455	45.03	171	84	3	3	515	175	335	4e-37	139
3.5.3.-(Hydrolases;)																					
No Correspond Unigene																					
3.5.3.19(ureidoglycolate hydrolase)																					
No Correspond Unigene																					
3.5.3.4(allantoicase)																					
No Correspond Unigene																					
3.5.3.9(allantoate deiminase;)																					
ABCDEF_7182	UniRef50_O49434	0.0	Normal: 296	247	269	247	258	625	BlastAnno: 1409	UniRef50_O49434	525	73.06	386	103	1	82	1239	139	523	0.0	586
ABCDEF_7183	UniRef50_O49434	0.0	Normal: 411	317	313	372	372	792	BlastAnno: 1855	UniRef50_O49434	525	68.08	473	120	2	274	1692	82	523	0.0	653
ABCDEF_7181	UniRef50_O49434	0.0	Normal: 441	332	343	362	388	869	BlastAnno: 1772	UniRef50_O49434	525	72.69	443	120	1	1499	171	82	523	0.0	668
3.5.4.10(IMP cyclohydrolase;)																					
No Correspond Unigene																					
3.5.4.2(adenine deaminase;)																					
No Correspond Unigene																					
3.5.4.3(guanine deaminase;)																					
ABCDEF_4553	UniRef50_B2KAX7	8e-25	Normal: 750	1061	753	1350	2399	3282	BlastAnno: 949	UniRef50_B2KAX7	163	55.10	98	44	0	759	466	12	109	8e-25	104
3.5.4.4(adenosine deaminase;)																					
No Correspond Unigene																					
3.5.4.6(AMP deaminase;)																					
ABCDEF_10722	UniRef50_A2QHI4	0.0	Normal: 188	59	201	104	254	90	BlastAnno: 1596	UniRef50_A2QHI4	991	61.07	411	160	0	2	1234	426	836	0.0	550
ABCDEF_21847	UniRef50_A2QHI4	0.0	Normal: 2350	1658	2484	1979	2471	2885	BlastAnno: 3133	UniRef50_A2QHI4	991	58.53	516	211	1	2051	513	308	823	0.0	625
3.5.4.8(aminoimidazolase;)																					
No Correspond Unigene																					
3.6.1.-(Hydrolases;)																					
ABCDEF_43677	UniRef50_B6ADS3	0.0	Normal: 265	202	294	363	341	233	BlastAnno: 2843	UniRef50_B6ADS3	1078	62.19	685	246	6	2017	2	246	930	0.0	842
ABCDEF_40935	UniRef50_A7ASE9	0.0	Normal: 1058	787	1081	1355	1280	1214	BlastAnno: 3814	UniRef50_A7ASE9	931	63.21	647	232	3	2359	428	285	928	0.0	811
ABCDEF_37918	UniRef50_Q8L831	0.0	Normal: 1344	592	1122	994	975	978	BlastAnno: 2888	UniRef50_Q8L831	772	71.74	768	214	1	209	2503	4	771	0.0	1152
ABCDEF_43683	UniRef50_B6ADS3	0.0	Normal: 186	143	232	259	238	201	BlastAnno: 1783	UniRef50_B6ADS3	1078	66.53	478	159	1	1	1431	600	1077	0.0	698
ABCDEF_34064	UniRef50_A7AVM7	0.0	Normal: 2140	1397	1695	2129	1483	1897	BlastAnno: 4387	UniRef50_A7AVM7	1016	49.83	891	398	12	2923	368	80	960	0.0	799
ABCDEF_43671	UniRef50_B6ADS3	0.0	Normal: 152	130	166	197	197	139	BlastAnno: 1982	UniRef50_B6ADS3	1078	65.79	643	211	5	80	1981	298	940	0.0	856
ABCDEF_43679	UniRef50_B6ADS3	0.0	Normal: 245	175	259	298	300	234	BlastAnno: 2502	UniRef50_B6ADS3	1078	65.13	780	263	5	2423	111	298	1077	0.0	1053
ABCDEF_43672	UniRef50_B6ADS3	0.0	Normal: 334	268	365	470	391	268	BlastAnno: 3191	UniRef50_B6ADS3	1078	62.19	685	246	6	1175	3190	246	930	0.0	842
3.6.1.11(exopolyphosphatase;)																					

No Correspond Unigene																					
3.6.1.13(ADP-ribose diphosphatase;)																					
ABCDEF_28370	UniRef50_Q3LIE5	3e-60	Normal: 117	194	111	256	248	318	BlastAnno: 1132	UniRef50_Q3LIE5	342	41.40	314	162	6	196	1080	17	327	3e-60	206
3.6.1.14(adenosine-tetraphosphatase)																					
No Correspond Unigene																					
3.6.1.15(nucleoside-triphosphatase;)																					
ABCDEF_40935	UniRef50_B7PNN0	0.0	Normal: 1058	787	1081	1355	1280	1214	BlastAnno: 3814	UniRef50_B7PNN0	1042	57.14	812	311	14	2815	413	249	1034	0.0	867
3.6.1.17(bis(5'-nucleosyl)-tetraphosphatase (asymmetrical);)																					
ABCDEF_21476	UniRef50_Q9C6Z2	4e-101	Normal: 226	171	227	417	290	227	BlastAnno: 784	UniRef50_Q9C6Z2	175	81.14	175	31	1	702	184	1	175	4e-101	301
3.6.1.19(nucleoside-triphosphate diphosphatase;)																					
ABCDEF_52501	UniRef50_B9RWM8	8e-103	Normal: 407	84	427	381	659	120	BlastAnno: 939	UniRef50_B9RWM8	284	74.60	189	48	0	136	702	12	200	8e-103	311
3.6.1.20(5'-acylphosphoadenosine hydrolase;)																					
No Correspond Unigene																					
3.6.1.21(ADP-sugar diphosphatase;)																					
ABCDEF_6188	UniRef50_Q9SZ63	2e-140	Normal: 60	106	66	96	87	338	BlastAnno: 1230	UniRef50_Q9SZ63	309	72.14	262	73	0	1056	271	43	304	2e-140	412
3.6.1.29(bis(5'-adenosyl)-triphosphatase;)																					
ABCDEF_88071	UniRef50_O76463	2e-53	Normal: 47	18	20	27	56	61	BlastAnno: 701	UniRef50_O76463	440	45.10	204	109	3	658	53	17	219	2e-53	186
3.6.1.3(adenosinetriphosphatase;)																					
ABCDEF_2970	UniRef50_A8I175	0.0	Normal: 1828	875	1448	2169	2783	1427	BlastAnno: 2494	UniRef50_A8I175	719	53.15	730	321	4	2312	138	5	718	0.0	762
ABCDEF_14294	UniRef50_Q9VA54	0.0	Normal: 804	446	725	896	1628	1178	BlastAnno: 1619	UniRef50_Q9VA54	399	81.03	390	72	1	1592	423	12	399	0.0	615
ABCDEF_43883	UniRef50_Q9W0C5	0.0	Normal: 449	407	349	525	552	824	BlastAnno: 2631	UniRef50_Q9W0C5	743	56.67	600	247	2	2064	271	132	720	0.0	593
ABCDEF_14293	UniRef50_Q9VA54	0.0	Normal: 539	271	560	607	897	657	BlastAnno: 1425	UniRef50_Q9VA54	399	81.03	390	72	1	1398	229	12	399	0.0	615
ABCDEF_33944	UniRef50_A3LSJ3	0.0	Normal: 555	486	502	746	708	807	BlastAnno: 3624	UniRef50_A3LSJ3	1067	47.40	711	345	7	813	2885	261	962	0.0	676
ABCDEF_14292	UniRef50_Q9VA54	0.0	Normal: 788	419	811	959	1142	915	BlastAnno: 1606	UniRef50_Q9VA54	399	81.03	390	72	1	212	1381	12	399	0.0	615
3.6.1.40(guanosine-5'-triphosphate,3'-diphosphate phosphatase;)																					
No Correspond Unigene																					
3.6.1.41(bis(5'-nucleosyl)-tetraphosphatase (symmetrical);)																					
No Correspond Unigene																					
3.6.1.5(apyrase;)																					
ABCDEF_21395	UniRef50_P80595	0.0	Normal: 394	79	79	136	478	850	BlastAnno: 1744	UniRef50_P80595	454	61.45	441	163	3	56	1360	7	446	0.0	566
3.6.1.6(nucleoside-diphosphatase;)																					
No Correspond Unigene																					
3.6.1.8(ATP diphosphatase;)																					
No Correspond Unigene																					
3.6.1.9(nucleotide diphosphatase;)																					
ABCDEF_27673	UniRef50_Q9SU83	0.0	Normal: 373	418	422	639	977	783	BlastAnno: 2094	UniRef50_Q9SU83	496	70.27	481	123	2	1775	393	15	495	0.0	679
ABCDEF_83044	UniRef50_Q9SU83	0.0	Normal: 191	238	231	367	469	401	BlastAnno: 1570	UniRef50_Q9SU83	496	70.42	480	122	2	162	1541	15	494	0.0	678
3.6.4.1(myosin ATPase)																					
No Correspond Unigene																					
4.1.1.-(Lyases;)																					
ABCDEF_57496	UniRef50_O96569	9e-20	Normal: 4	0	0	0	0	9	BlastAnno: 223	UniRef50_O96569	439	66.67	72	22	1	210	1	19	90	9e-20	87.4
4.1.1.21(phosphoribosylaminoimidazole carboxylase;)																					
ABCDEF_47786	UniRef50_P15567	9e-155	Normal: 863	476	1099	1055	1144	1023	BlastAnno: 2730	UniRef50_P15567	552	50.73	548	249	10	2235	613	17	550	9e-155	475
4.3.2.2(adenylosuccinate lyase;)																					
ABCDEF_6815	UniRef50_P44797	0.0	Normal: 676	385	721	847	893	1064	BlastAnno: 2055	UniRef50_P44797	456	62.56	454	170	0	1710	349	2	455	0.0	603
ABCDEF_6814	UniRef50_P44797	0.0	Normal: 625	320	680	746	875	784	BlastAnno: 2012	UniRef50_P44797	456	62.33	454	171	0	1667	306	2	455	0.0	602
4.3.2.3(ureidoglycolate lyase;)																					
No Correspond Unigene																					
4.6.1.1(adenylate cyclase;)																					
No Correspond Unigene																					
4.6.1.2(guanylate cyclase;)																					
No Correspond Unigene																					
5.1.99.3(allantoin racemase)																					
No Correspond Unigene																					
5.4.2.2(phosphoglucomutase;)																					
ABCDEF_935	UniRef50_P47244	0.0	Normal: 5430	4330	5743	12110	19002	6223	BlastAnno: 2144	UniRef50_P47244	572	53.66	587	246	11	2064	328	4	572	0.0	585
ABCDEF_24641	UniRef50_P47244	0.0	Normal: 416	659	414	959	874	2109	BlastAnno: 2341	UniRef50_P47244	572	54.17	576	222	11	386	2035	13	572	0.0	588
ABCDEF_24640	UniRef50_P47244	0.0	Normal: 389	596	384	834	777	1859	BlastAnno: 2422	UniRef50_P47244	572	51.74	603	222	12	386	2116	13	572	0.0	574
5.4.2.7(phosphopentomutase;)																					
No Correspond Unigene																					
5.4.99.18(5-(carboxyamino)imidazole ribonucleotide mutase;)																					
6.3.2.6(phosphoribosylaminoimidazolesuccinocarboxamide synthase;)																					
ABCDEF_28248	UniRef50_P38025	0.0	Normal: 383	301	494	449	518	506	BlastAnno: 1559	UniRef50_P38025	411	72.99	374	96	2	188	1297	36	408	0.0	579
6.3.3.1(phosphoribosylformylglycinamidine cyclo-ligase;)																					
ABCDEF_31416	UniRef50_Q05728	0.0	Normal: 839	516	998	878	1262	894	BlastAnno: 1544	UniRef50_Q05728	389	74.33	374	90	3	1286	183	16	389	0.0	525
6.3.4.-(Ligases;)																					
No Correspond Unigene																					
6.3.4.1(GMP synthase;)																					
No Correspond Unigene																					
6.3.4.13(phosphoribosylamine---glycine ligase;)																					
ABCDEF_34859	UniRef50_P52420	0.0	Normal: 758	354	644	487	469	780	BlastAnno: 2224	UniRef50_P52420	532	81.53	444	82	0	1640	309	83	526	0.0	732
6.3.4.18(5-(carboxyamino)imidazole ribonucleotide synthase;)																					

No Correspond Unigene																					
6.3.4.4(adenylosuccinate synthase;)																					
ABCDEF_25360	UniRef50_B7FUM7	5e-155	Normal: 1734	835	1556	1723	2625	1416	BlastAnno: 2000	UniRef50_B7FUM7	526	51.33	450	200	5	1743	403	78	511	5e-155	467
6.3.4.7(ribose-5-phosphate---ammonia ligase;)																					
No Correspond Unigene																					
6.3.5.2(GMP synthase (glutamine-hydrolysing);)																					
ABCDEF_41850	UniRef50_B6JHM4	0.0	Normal: 1048	347	842	324	406	568	BlastAnno: 1903	UniRef50_B6JHM4	531	53.48	531	233	6	1773	181	15	531	0.0	550
ABCDEF_41849	UniRef50_B6JHM4	0.0	Normal: 1080	353	863	337	404	574	BlastAnno: 1966	UniRef50_B6JHM4	531	53.67	518	226	6	1722	169	28	531	0.0	538
6.3.5.3(phosphoribosylformylglycinamidine synthase;)																					
ABCDEF_23606	UniRef50_O15067	0.0	Normal: 983	541	893	1169	1710	427	BlastAnno: 4923	UniRef50_O15067	1338	50.48	1347	625	19	423	4391	4	1332	0.0	1248
>00231 Puromycin biosynthesis																					
1.-.-.-(Oxidoreductases)																					
ABCDEF_345	UniRef50_Q6Z690	0.0	Normal: 442	146	449	340	272	314	BlastAnno: 1658	UniRef50_Q6Z690	849	63.42	462	150	5	1657	314	387	843	0.0	589
ABCDEF_24068	UniRef50_Q9FI41	0.0	Normal: 214	316	207	270	263	849	BlastAnno: 1742	UniRef50_Q9FI41	387	73.28	363	96	1	1440	355	4	366	0.0	554
ABCDEF_24070	UniRef50_Q9FI41	0.0	Normal: 189	290	182	231	232	751	BlastAnno: 1479	UniRef50_Q9FI41	387	73.28	363	96	1	1363	278	4	366	0.0	551
ABCDEF_13957	UniRef50_Q9CAE3	0.0	Normal: 399	318	392	489	456	586	BlastAnno: 2488	UniRef50_Q9CAE3	789	80.85	496	93	2	3	1487	285	779	0.0	768
ABCDEF_28410	UniRef50_Q9LIG0	0.0	Normal: 211	366	234	286	433	1504	BlastAnno: 1324	UniRef50_Q9LIG0	330	74.92	331	76	2	1147	173	1	330	0.0	521
ABCDEF_17581	UniRef50_Q8LGI2	0.0	Normal: 758	336	735	426	374	852	BlastAnno: 1543	UniRef50_Q8LGI2	454	68.78	442	136	2	71	1390	8	449	0.0	597
ABCDEF_23753	UniRef50_Q94A68	0.0	Normal: 284	559	215	224	164	519	BlastAnno: 1580	UniRef50_Q94A68	377	81.90	348	63	0	1349	306	30	377	0.0	576
ABCDEF_24071	UniRef50_Q9FI41	0.0	Normal: 235	377	230	278	289	948	BlastAnno: 2090	UniRef50_Q9FI41	387	73.28	363	96	1	1726	641	4	366	0.0	553
2.-.-.-(Transferases)																					
ABCDEF_3256	UniRef50_Q5VV42	9e-165	Normal: 1823	1179	1691	2271	1981	2383	BlastAnno: 3398	UniRef50_Q5VV42	579	50.91	497	220	6	3326	1866	10	492	9e-165	509
2.1.1.38(O-demethylpuromycin O-methyltransferase;)																					
No Correspond Unigene																					
2.3.-.-(Transferases;)																					
ABCDEF_40719	UniRef50_Q54MC0	1e-47	Normal: 79	101	105	229	268	159	BlastAnno: 1918	UniRef50_Q54MC0	492	32.48	508	267	10	1439	81	6	492	1e-47	181
3.-.-.-(No Name)																					
ABCDEF_48866	UniRef50_Q687E1	0.0	Normal: 345	607	237	367	189	838	BlastAnno: 1481	UniRef50_Q687E1	368	68.38	351	109	1	1199	147	20	368	0.0	521
ABCDEF_3986	UniRef50_Q9LYC1	0.0	Normal: 226	528	111	65	141	752	BlastAnno: 1793	UniRef50_Q9LYC1	358	74.64	347	84	3	1423	386	1	344	0.0	551
ABCDEF_34862	UniRef50_Q9MAA7	0.0	Normal: 341	401	253	317	200	932	BlastAnno: 1664	UniRef50_Q9MAA7	345	74.71	348	83	2	138	1178	1	344	0.0	553
ABCDEF_51645	UniRef50_Q687E1	0.0	Normal: 433	515	632	1120	1009	791	BlastAnno: 2234	UniRef50_Q687E1	368	74.71	348	88	0	987	2030	20	367	0.0	575
ABCDEF_24556	UniRef50_C8NK17	0.0	Normal: 7370	10762	6819	9293	10001	30701	BlastAnno: 3104	UniRef50_C8NK17	927	57.35	823	317	7	401	2791	1	815	0.0	839
>00232 Caffeine metabolism																					
1.13.12.-(Oxidoreductases;)																					
No Correspond Unigene																					
1.14.14.1(unspecific monooxygenase;)																					
ABCDEF_41955	UniRef50_B9S0F8	2e-174	Normal: 70	140	28	0	0	150	BlastAnno: 1118	UniRef50_B9S0F8	506	66.67	366	115	5	2	1099	138	496	2e-174	504
1.17.3.2(xanthine oxidase;)																					
No Correspond Unigene																					
1.17.5.-(Oxidoreductases;)																					
No Correspond Unigene																					
1.7.3.3(factor-independent urate hydroxylase;)																					
ABCDEF_25588	UniRef50_O04420	2e-147	Normal: 932	1081	941	2032	2640	744	BlastAnno: 1309	UniRef50_O04420	309	71.05	304	87	1	77	985	6	309	2e-147	431
2.1.1.158(7-methylxanthosine synthase;)																					
No Correspond Unigene																					
2.1.1.159(theobromine synthase;)																					
No Correspond Unigene																					
2.1.1.160(caffeine synthase;)																					
No Correspond Unigene																					
2.3.1.5(arylamine N-acetyltransferase;)																					
No Correspond Unigene																					
3.2.2.25(N-methyl nucleosidase;)																					
No Correspond Unigene																					
>00240 Pyrimidine metabolism																					
1.17.4.1(ribonucleoside-diphosphate reductase;)																					
ABCDEF_20810	UniRef50_C5DY80	0.0	Normal: 2798	417	1493	203	646	708	BlastAnno: 2909	UniRef50_C5DY80	875	54.51	776	314	10	2660	405	25	785	0.0	811
ABCDEF_51669	UniRef50_C5DY80	0.0	Normal: 2176	202	1063	25	233	660	BlastAnno: 2851	UniRef50_C5DY80	875	54.12	776	317	9	2594	339	25	785	0.0	815
1.17.4.2(ribonucleoside-triphosphate reductase;)																					
No Correspond Unigene																					
1.17.99.4(uracil/thymine dehydrogenase;)																					
No Correspond Unigene																					
1.3.1.1(dihydrouracil dehydrogenase (NAD+);)																					
ABCDEF_27630	UniRef50_Q01P61	9e-82	Normal: 448	273	523	806	1011	577	BlastAnno: 1916	UniRef50_Q01P61	446	41.85	368	198	4	1435	338	2	355	9e-82	273
1.3.1.14(orotate reductase (NADH);)																					
No Correspond Unigene																					
1.3.1.2(dihydropyrimidine dehydrogenase (NADP+);)																					
ABCDEF_27630	UniRef50_A9GKC4	2e-84	Normal: 448	273	523	806	1011	577	BlastAnno: 1916	UniRef50_A9GKC4	462	42.13	356	190	5	1435	374	2	343	2e-84	280
1.3.3.1(dihydroorotate oxidase;)																					
No Correspond Unigene																					
1.3.5.2(dihydroorotate dehydrogenase;)																					
ABCDEF_21312	UniRef50_Q02127	3e-108	Normal: 504	211	473	517	1139	224	BlastAnno: 1973	UniRef50_Q02127	395	51.59	378	164	4	1502	369	36	394	3e-108	341
1.8.1.9(thioredoxin-disulfide reductase;)																					

ABCDEF_41899	UniRef50_P29509	6e-131	Normal: 922	343	782	913	1401	860	BlastAnno: 1495	UniRef50_P29509	319	63.55	321	108	3	1305	358	2	318	6e-131	391
2.1.1.148(thymidylate synthase (FAD);)																					
No Correspond Unigene																					
2.1.1.45(thymidylate synthase;)																					
ABCDEF_13009	UniRef50_P04818	2e-137	Normal: 222	170	310	238	266	400	BlastAnno: 1734	UniRef50_P04818	313	65.03	286	100	0	1313	456	28	313	2e-137	410
2.1.2.8(deoxycytidylate 5-hydroxymethyltransferase;)																					
No Correspond Unigene																					
2.1.3.2(aspartate carbamoyltransferase;)																					
ABCDEF_24647	UniRef50_P49077	3e-180	Normal: 510	109	521	166	447	274	BlastAnno: 1462	UniRef50_P49077	390	79.77	341	64	2	1203	196	50	390	3e-180	519
2.4.2.1(purine-nucleoside phosphorylase;)																					
No Correspond Unigene																					
2.4.2.10(orotate phosphoribosyltransferase;)																					
ABCDEF_24791	UniRef50_P11172	2e-176	Normal: 1550	526	1529	936	1278	476	BlastAnno: 1774	UniRef50_P11172	480	55.83	480	204	3	1578	151	2	477	2e-176	517
2.4.2.2(pyrimidine-nucleoside phosphorylase;)																					
No Correspond Unigene																					
2.4.2.23(deoxyuridine phosphorylase)																					
No Correspond Unigene																					
2.4.2.3(uridine phosphorylase;)																					
No Correspond Unigene																					
2.4.2.4(thymidine phosphorylase;)																					
No Correspond Unigene																					
2.4.2.6(nucleoside deoxyribosyltransferase;)																					
No Correspond Unigene																					
2.4.2.9(uracil phosphoribosyltransferase;)																					
ABCDEF_11650	UniRef50_Q31MH4	4e-84	Normal: 602	521	1040	1098	1224	1951	BlastAnno: 1299	UniRef50_Q31MH4	217	55.35	215	94	1	386	1030	4	216	4e-84	265
2.7.1.21(thymidine kinase;)																					
ABCDEF_21938	UniRef50_B9IH47	9e-105	Normal: 1403	516	1248	906	977	1202	BlastAnno: 1705	UniRef50_B9IH47	228	79.08	196	41	0	761	1348	26	221	9e-105	323
2.7.1.48(uridine kinase;)																					
ABCDEF_21118	UniRef50_B9SJ57	0.0	Normal: 93	111	86	175	183	112	BlastAnno: 1405	UniRef50_B9SJ57	975	81.77	406	73	1	41	1255	490	895	0.0	655
ABCDEF_38924	UniRef50_B9G1X9	0.0	Normal: 1490	957	1567	1484	2150	1194	BlastAnno: 1877	UniRef50_B9G1X9	586	85.88	425	60	0	1568	294	162	586	0.0	747
ABCDEF_51908	UniRef50_B9G1X9	0.0	Normal: 300	175	331	557	912	308	BlastAnno: 1779	UniRef50_B9G1X9	586	81.51	292	54	0	648	1523	295	586	0.0	486
ABCDEF_21162	UniRef50_B9G1X9	0.0	Normal: 121	88	276	563	1240	72	BlastAnno: 1734	UniRef50_B9G1X9	586	80.82	292	56	0	1112	237	295	586	0.0	486
2.7.1.74(deoxycytidine kinase;)																					
No Correspond Unigene																					
2.7.1.83(pseudouridine kinase;)																					
No Correspond Unigene																					
2.7.4.10(nucleoside-triphosphate---adenylate kinase;)																					
ABCDEF_18288	UniRef50_B9R9Q5	6e-85	Normal: 300	267	518	1073	2376	324	BlastAnno: 1269	UniRef50_B9R9Q5	298	60.85	212	82	1	1012	380	82	293	6e-85	270
2.7.4.14(UMP/CMP kinase;)																					
ABCDEF_37317	UniRef50_O04905	4e-111	Normal: 118	167	224	480	1080	282	BlastAnno: 850	UniRef50_O04905	202	80.79	203	31	3	74	682	1	195	4e-111	328
2.7.4.22(UMP kinase;)																					
ABCDEF_45344	UniRef50_Q10Y48	3e-103	Normal: 295	300	335	395	510	898	BlastAnno: 1555	UniRef50_Q10Y48	244	63.18	239	87	1	571	1287	5	242	3e-103	318
2.7.4.4(nucleoside-phosphate kinase;)																					
No Correspond Unigene																					
2.7.4.6(nucleoside-diphosphate kinase;)																					
ABCDEF_1220	UniRef50_O49203	3e-121	Normal: 1836	1610	2747	4707	8156	4732	BlastAnno: 1189	UniRef50_O49203	238	75.42	240	56	3	151	867	1	238	3e-121	360
2.7.4.9(dTMP kinase;)																					
ABCDEF_4251	UniRef50_Q54GN2	1e-52	Normal: 248	79	225	119	291	212	BlastAnno: 1256	UniRef50_Q54GN2	222	53.68	190	86	2	338	901	16	205	1e-52	183
2.7.7.6(DNA-directed RNA polymerase;)																					
ABCDEF_41473	UniRef50_B9GKP9	0.0	Normal: 331	132	133	251	174	146	BlastAnno: 1665	UniRef50_B9GKP9	1517	55.25	581	223	5	1637	3	201	780	0.0	570
ABCDEF_52641	UniRef50_Q8L6J2	0.0	Normal: 258	64	314	187	213	114	BlastAnno: 1303	UniRef50_Q8L6J2	332	83.88	304	49	0	1302	391	29	332	0.0	551
ABCDEF_6430	UniRef50_A9UY22	0.0	Normal: 1077	541	777	657	784	387	BlastAnno: 3519	UniRef50_A9UY22	1592	42.33	1004	486	21	3288	424	271	1230	0.0	752
ABCDEF_38289	UniRef50_Q9LK40	0.0	Normal: 3533	1236	2709	3045	2599	2113	BlastAnno: 4120	UniRef50_Q9LK40	1172	65.91	1185	362	10	356	3871	16	1171	0.0	1566
ABCDEF_47593	UniRef50_B9SHS2	0.0	Normal: 1241	283	1007	794	885	416	BlastAnno: 4025	UniRef50_B9SHS2	1139	79.23	1146	221	3	3890	456	10	1139	0.0	1819
ABCDEF_17969	UniRef50_P56764	0.0	Normal: 59	89	176	20	28	798	BlastAnno: 1479	UniRef50_P56764	1376	65.04	492	158	6	1473	1	484	962	0.0	574
ABCDEF_14408	UniRef50_Q5KIB7	0.0	Normal: 3327	1620	2819	3121	2847	2381	BlastAnno: 3999	UniRef50_Q5KIB7	1193	56.06	1204	417	15	3705	238	49	1188	0.0	1315
ABCDEF_3729	UniRef50_Q9LFV6	0.0	Normal: 229	56	187	161	198	53	BlastAnno: 1749	UniRef50_Q9LFV6	1011	66.26	575	193	1	1724	3	168	742	0.0	804
ABCDEF_4016	UniRef50_B9G6G0	0.0	Normal: 416	120	403	152	266	64	BlastAnno: 1471	UniRef50_B9G6G0	1150	68.30	407	117	4	2	1213	743	1140	0.0	553
ABCDEF_37325	UniRef50_A8IRZ2	0.0	Normal: 1508	1018	1110	2111	1134	1174	BlastAnno: 3913	UniRef50_A8IRZ2	1789	56.47	1259	494	12	3708	13	1	1232	0.0	1405
ABCDEF_37205	UniRef50_Q2XSM7	0.0	Normal: 620	278	467	527	379	735	BlastAnno: 3532	UniRef50_Q2XSM7	997	39.45	834	457	17	544	2988	193	997	0.0	589
ABCDEF_18114	UniRef50_C5YGY9	0.0	Normal: 2784	1475	2612	2568	2178	2591	BlastAnno: 2404	UniRef50_C5YGY9	1857	85.71	343	44	4	2402	1383	1195	1535	0.0	602
ABCDEF_20059	UniRef50_B9F2S6	0.0	Normal: 1614	564	810	1084	542	661	BlastAnno: 5256	UniRef50_B9F2S6	2017	44.85	1710	786	31	340	5076	3	1686	0.0	1310
ABCDEF_20332	UniRef50_B8C4M1	0.0	Normal: 673	337	552	1076	603	549	BlastAnno: 3314	UniRef50_B8C4M1	1422	49.12	1132	525	17	1	3312	50	1158	0.0	1059
ABCDEF_4043	UniRef50_Q9LFV6	0.0	Normal: 164	28	131	91	64	28	BlastAnno: 1638	UniRef50_Q9LFV6	1011	66.01	456	152	2	269	1636	159	611	0.0	563
ABCDEF_10576	UniRef50_Q1L5X6	0.0	Normal: 546	239	446	510	567	478	BlastAnno: 1817	UniRef50_Q1L5X6	1138	59.70	536	211	4	1815	214	603	1135	0.0	625
2.7.7.7(DNA-directed DNA polymerase;)																					
ABCDEF_16323	UniRef50_O48653	0.0	Normal: 85	21	57	21	24	30	BlastAnno: 2598	UniRef50_O48653	1534	62.00	650	223	8	8	1903	244	887	0.0	790
ABCDEF_16314	UniRef50_B3RI52	0.0	Normal: 159	23	81	20	29	39	BlastAnno: 3553	UniRef50_B3RI52	1203	39.13	1099	524	28	3258	16	137	1108	0.0	700
ABCDEF_3831	UniRef50_Q9LVN7	0.0	Normal: 872	95	527	93	228	166	BlastAnno: 3372	UniRef50_Q9LVN7	1081	83.91	957	149	2	3372	502	61	1012	0.0	1560
ABCDEF_16320	UniRef50_B3RI52	0.0	Normal: 150	23	89	22	32	38	BlastAnno: 3613	UniRef50_B3RI52	1203	38.46	1118	525	28	296	3598	137	1108	0.0	690
ABCDEF_57123	UniRef50_Q9SGD5	0.0	Normal: 115	8	30	0	0	8	BlastAnno: 1036	UniRef50_Q9SGD5	2271	78.16	348	52	3	1	1026	528	857	0.0	565
ABCDEF_57314	UniRef50_O48653	0.0	Normal: 50	8	24	6	11	14	BlastAnno: 1773	UniRef50_O48653	1534	62.44	591	202	6	8	1726	244	832	0.0	728

ABCDEF_17221	UniRef50_O48520	0.0	Normal: 728	155	529	212	218	433	BlastAnno: 1641	UniRef50_O48520	456	74.34	456	99	3	1572	253	1	454	0.0	707
ABCDEF_48352	UniRef50_B9SQ81	0.0	Normal: 238	127	222	78	59	181	BlastAnno: 1927	UniRef50_B9SQ81	560	68.17	421	103	3	1	1248	52	446	0.0	572
ABCDEF_16321	UniRef50_B3RI52	0.0	Normal: 297	94	191	81	113	129	BlastAnno: 4416	UniRef50_B3RI52	1203	39.46	1044	490	28	1192	4269	273	1192	0.0	664
ABCDEF_16326	UniRef50_O48653	0.0	Normal: 216	127	206	114	121	136	BlastAnno: 3076	UniRef50_O48653	1534	62.00	650	223	8	3069	1174	244	887	0.0	790
ABCDEF_16319	UniRef50_C5XR76	0.0	Normal: 93	48	74	50	55	58	BlastAnno: 3092	UniRef50_C5XR76	1541	69.60	398	115	4	1144	2334	503	895	0.0	577
ABCDEF_51711	UniRef50_C5X4W2	0.0	Normal: 259	177	325	375	256	285	BlastAnno: 1806	UniRef50_C5X4W2	1714	67.79	534	152	3	1	1545	1182	1714	0.0	757
ABCDEF_16315	UniRef50_B3RI52	0.0	Normal: 173	48	108	57	52	72	BlastAnno: 4431	UniRef50_B3RI52	1203	39.83	964	435	24	2856	16	273	1108	0.0	627
ABCDEF_41802	UniRef50_Q9SGD5	0.0	Normal: 502	39	242	0	82	126	BlastAnno: 2596	UniRef50_Q9SGD5	2271	47.66	875	364	12	2	2416	1416	2266	0.0	720
ABCDEF_16322	UniRef50_B3RI52	0.0	Normal: 151	47	99	46	54	56	BlastAnno: 4047	UniRef50_B3RI52	1203	39.83	964	435	24	1192	4032	273	1108	0.0	627
ABCDEF_41210	UniRef50_B9RD63	0.0	Normal: 305	176	260	314	159	188	BlastAnno: 1568	UniRef50_B9RD63	1420	74.42	516	120	8	43	1566	769	1280	0.0	660
ABCDEF_16316	UniRef50_B3RI52	0.0	Normal: 172	41	103	47	47	63	BlastAnno: 4345	UniRef50_B3RI52	1203	40.68	944	435	23	1550	4330	273	1108	0.0	640
ABCDEF_16324	UniRef50_C5XR76	0.0	Normal: 86	44	67	46	46	48	BlastAnno: 3032	UniRef50_C5XR76	1541	69.60	398	115	4	1144	2334	503	895	0.0	577
2.7.7.8(polyribonucleotide nucleotidyltransferase;)																					
ABCDEF_27735	UniRef50_Q6MDI2	0.0	Normal: 1219	1128	977	1124	844	3456	BlastAnno: 3515	UniRef50_Q6MDI2	697	48.99	745	325	5	415	2649	5	694	0.0	683
3.1.3.5(5'-nucleotidase;)																					
ABCDEF_10187	UniRef50_A9F2C9	3e-85	Normal: 140	337	178	525	497	1064	BlastAnno: 2494	UniRef50_A9F2C9	618	34.02	535	278	12	792	2276	119	618	3e-85	292
3.1.3.6(3'-nucleotidase;)																					
No Correspond Unigene																					
3.1.4.16(2',3'-cyclic-nucleotide 2'-phosphodiesterase;)																					
No Correspond Unigene																					
3.2.2.10(pyrimidine-5'-nucleotide nucleosidase;)																					
No Correspond Unigene																					
3.2.2.3(uridine nucleosidase;)																					
ABCDEF_21724	UniRef50_Q9SJM7	8e-170	Normal: 28	80	41	258	854	278	BlastAnno: 1478	UniRef50_Q9SJM7	336	76.74	301	70	0	406	1308	36	336	8e-170	491
3.2.2.8(ribosylpyrimidine nucleosidase;)																					
No Correspond Unigene																					
3.5.1.6(beta-ureidopropionase)																					
ABCDEF_17031	UniRef50_Q9UBR1	1e-142	Normal: 273	642	328	641	1094	595	BlastAnno: 2413	UniRef50_Q9UBR1	384	58.49	383	153	3	2133	991	6	384	1e-142	434
3.5.1.95(N-malonylurea hydrolase;)																					
No Correspond Unigene																					
3.5.2.1(barbiturase)																					
No Correspond Unigene																					
3.5.2.2(dihydropyrimidinase;)																					
ABCDEF_44368	UniRef50_Q9FMP3	0.0	Normal: 229	259	223	421	456	369	BlastAnno: 1773	UniRef50_Q9FMP3	531	84.83	501	76	0	1554	52	27	527	0.0	864
ABCDEF_44370	UniRef50_Q9FMP3	0.0	Normal: 159	173	177	315	341	286	BlastAnno: 1537	UniRef50_Q9FMP3	531	85.98	435	61	0	1363	59	93	527	0.0	754
3.5.2.3(dihydroorotase;)																					
ABCDEF_13876	UniRef50_O04904	0.0	Normal: 291	140	364	513	525	230	BlastAnno: 1904	UniRef50_O04904	377	75.94	374	82	1	404	1501	3	376	0.0	590
ABCDEF_13875	UniRef50_O04904	0.0	Normal: 215	103	247	393	360	160	BlastAnno: 1555	UniRef50_O04904	377	75.94	374	82	1	420	1517	3	376	0.0	590
3.5.4.1(cytosine deaminase;)																					
No Correspond Unigene																					
3.5.4.12(dCMP deaminase;)																					
ABCDEF_57922	UniRef50_B9RAF8	2e-73	Normal: 30	0	7	0	0	10	BlastAnno: 494	UniRef50_B9RAF8	224	86.67	120	16	0	492	133	102	221	2e-73	228
3.5.4.13(dCTP deaminase;)																					
No Correspond Unigene																					
3.5.4.14(deoxycytidine deaminase)																					
No Correspond Unigene																					
3.5.4.30(dCTP deaminase (dUMP-forming))																					
No Correspond Unigene																					
3.5.4.5(cytidine deaminase;)																					
ABCDEF_21537	UniRef50_B9H8W4	3e-85	Normal: 130	250	188	236	275	331	BlastAnno: 996	UniRef50_B9H8W4	315	56.62	272	82	5	995	207	61	305	3e-85	268
3.6.1.12(dCTP diphosphatase;)																					
No Correspond Unigene																					
3.6.1.17(bis(5'-nucleosyl)-tetraphosphatase (asymmetrical);)																					
ABCDEF_21476	UniRef50_Q9C6Z2	4e-101	Normal: 226	171	227	417	290	227	BlastAnno: 784	UniRef50_Q9C6Z2	175	81.14	175	31	1	702	184	1	175	4e-101	301
3.6.1.19(nucleoside-triphosphate diphosphatase;)																					
ABCDEF_52501	UniRef50_B9RWM8	8e-103	Normal: 407	84	427	381	659	120	BlastAnno: 939	UniRef50_B9RWM8	284	74.60	189	48	0	136	702	12	200	8e-103	311
3.6.1.23(dUTP diphosphatase;)																					
ABCDEF_38681	UniRef50_Q54BW5	8e-65	Normal: 94	3	59	0	56	76	BlastAnno: 733	UniRef50_Q54BW5	179	69.13	149	43	1	549	112	27	175	8e-65	207
3.6.1.39(thymidine-triphosphatase;)																					
No Correspond Unigene																					
3.6.1.5(apyrase;)																					
ABCDEF_21395	UniRef50_P80595	0.0	Normal: 394	79	79	136	478	850	BlastAnno: 1744	UniRef50_P80595	454	61.45	441	163	3	56	1360	7	446	0.0	566
3.6.1.6(nucleoside-diphosphatase;)																					
No Correspond Unigene																					
3.6.1.8(ATP diphosphatase;)																					
No Correspond Unigene																					
4.1.1.23(orotidine-5'-phosphate decarboxylase;)																					
No Correspond Unigene																					
4.2.1.70(pseudouridylate synthase;)																					
ABCDEF_50681	UniRef50_Q6MKN0	9e-31	Normal: 33	6	13	9	17	55	BlastAnno: 1266	UniRef50_Q6MKN0	283	33.76	234	127	6	1098	400	44	250	9e-31	125
6.3.4.2(CTP synthase;)																					
ABCDEF_7425	UniRef50_Q9VUL1	0.0	Normal: 2149	300	1551	1876	2842	748	BlastAnno: 2630	UniRef50_Q9VUL1	627	55.99	559	240	3	2251	590	20	577	0.0	568

ABCDEF_9956	UniRef50_Q9VUL1	0.0	Normal: 394	186	413	343	404	451	BlastAnno: 2409	UniRef50_Q9VUL1	627	55.61	561	246	2	239	1915	1	560	0.0	573
ABCDEF_2439	UniRef50_Q9VUL1	0.0	Normal: 323	298	151	114	281	1442	BlastAnno: 4122	UniRef50_Q9VUL1	627	57.85	548	228	2	4057	2420	1	547	0.0	593
ABCDEF_68056	UniRef50_P17812	0.0	Normal: 1346	186	947	1238	1747	426	BlastAnno: 1952	UniRef50_P17812	591	57.41	526	213	4	1584	34	20	543	0.0	550
ABCDEF_2438	UniRef50_Q9VUL1	0.0	Normal: 328	298	134	127	296	1442	BlastAnno: 4003	UniRef50_Q9VUL1	627	57.25	559	236	2	66	1736	1	558	0.0	602
ABCDEF_2433	UniRef50_Q9VUL1	0.0	Normal: 372	313	157	142	353	1294	BlastAnno: 3826	UniRef50_Q9VUL1	627	57.66	548	229	2	382	2019	1	547	0.0	593
6.3.5.5(carbamoyl-phosphate synthase (glutamine-hydrolysing);)																					
ABCDEF_48168	UniRef50_A7HVJ4	0.0	Normal: 1604	1009	1521	1682	1283	1898	BlastAnno: 4056	UniRef50_A7HVJ4	1079	58.58	1096	423	10	3524	273	3	1079	0.0	1242
ABCDEF_10940	UniRef50_A7HVJ4	0.0	Normal: 464	188	321	214	331	350	BlastAnno: 1730	UniRef50_A7HVJ4	1079	58.66	583	214	6	3	1715	274	841	0.0	671
>00250 Alanine, aspartate and glutamate metabolism																					
1.2.1.16(succinate-semialdehyde dehydrogenase [NAD(P)+];)																					
ABCDEF_35184	UniRef50_Q39Q67	0.0	Normal: 907	754	991	1776	1616	1278	BlastAnno: 2107	UniRef50_Q39Q67	509	57.38	481	205	0	1853	411	29	509	0.0	578
1.2.1.24(succinate-semialdehyde dehydrogenase (NAD+);)																					
ABCDEF_35184	UniRef50_Q9SAK4	0.0	Normal: 907	754	991	1776	1616	1278	BlastAnno: 2107	UniRef50_Q9SAK4	528	75.00	528	129	2	1991	408	2	526	0.0	778
1.4.1.1(alanine dehydrogenase;)																					
No Correspond Unigene																					
1.4.1.13(glutamate synthase (NADPH);)																					
ABCDEF_9685	UniRef50_C5P2L2	0.0	Normal: 1255	805	1346	3025	1481	566	BlastAnno: 6164	UniRef50_C5P2L2	2137	57.29	1934	742	18	6164	375	250	2103	0.0	2102
ABCDEF_66114	UniRef50_C5P2L2	0.0	Normal: 1307	821	1417	3088	1567	591	BlastAnno: 6039	UniRef50_C5P2L2	2137	58.59	1862	724	16	59	5632	285	2103	0.0	2092
ABCDEF_9687	UniRef50_C5P2L2	0.0	Normal: 1563	1073	1590	4123	1731	640	BlastAnno: 7405	UniRef50_C5P2L2	2137	56.45	2117	832	25	6616	377	40	2103	0.0	2256
ABCDEF_9683	UniRef50_C5P2L2	0.0	Normal: 1525	975	1564	3728	1698	627	BlastAnno: 7188	UniRef50_C5P2L2	2137	56.45	2117	832	25	540	6779	40	2103	0.0	2258
ABCDEF_76911	UniRef50_C5P2L2	0.0	Normal: 1308	820	1416	3108	1574	593	BlastAnno: 6032	UniRef50_C5P2L2	2137	58.29	1870	733	16	6007	410	277	2103	0.0	2092
ABCDEF_9684	UniRef50_C5P2L2	0.0	Normal: 1255	813	1342	2996	1487	561	BlastAnno: 6155	UniRef50_C5P2L2	2137	57.90	1893	744	17	131	5779	254	2103	0.0	2097
ABCDEF_23790	UniRef50_A5UXJ6	0.0	Normal: 1509	3497	1157	1553	1291	9842	BlastAnno: 5662	UniRef50_A5UXJ6	1554	47.52	1553	718	23	5114	627	18	1530	0.0	1355
1.4.1.14(glutamate synthase (NADH);)																					
ABCDEF_9685	UniRef50_Q03460	0.0	Normal: 1255	805	1346	3025	1481	566	BlastAnno: 6164	UniRef50_Q03460	2194	82.36	1962	296	5	6164	306	274	2194	0.0	3175
ABCDEF_66114	UniRef50_Q03460	0.0	Normal: 1307	821	1417	3088	1567	591	BlastAnno: 6039	UniRef50_Q03460	2194	83.91	1896	291	4	41	5701	304	2194	0.0	3140
ABCDEF_9687	UniRef50_Q03460	0.0	Normal: 1563	1073	1590	4123	1731	640	BlastAnno: 7405	UniRef50_Q03460	2194	82.91	2159	351	6	6754	308	44	2194	0.0	3558
ABCDEF_9683	UniRef50_Q03460	0.0	Normal: 1525	975	1564	3728	1698	627	BlastAnno: 7188	UniRef50_Q03460	2194	82.91	2159	351	6	402	6848	44	2194	0.0	3555
ABCDEF_76911	UniRef50_Q03460	0.0	Normal: 1308	820	1416	3108	1574	593	BlastAnno: 6032	UniRef50_Q03460	2194	83.86	1896	292	4	6001	341	304	2194	0.0	3145
ABCDEF_9684	UniRef50_Q03460	0.0	Normal: 1255	813	1342	2996	1487	561	BlastAnno: 6155	UniRef50_Q03460	2194	82.84	1929	309	7	92	5848	278	2194	0.0	3147
1.4.1.2(glutamate dehydrogenase;)																					
ABCDEF_52465	UniRef50_Q5HHC7	4e-126	Normal: 404	402	745	2021	3684	1711	BlastAnno: 1651	UniRef50_Q5HHC7	414	44.01	409	227	2	213	1436	6	413	4e-126	384
1.4.1.3(glutamate dehydrogenase [NAD(P)+];)																					
ABCDEF_25196	UniRef50_Q43314	0.0	Normal: 271	16	291	548	795	8	BlastAnno: 1412	UniRef50_Q43314	411	78.84	378	80	0	1410	277	34	411	0.0	642
ABCDEF_52465	UniRef50_Q43314	0.0	Normal: 404	402	745	2021	3684	1711	BlastAnno: 1651	UniRef50_Q43314	411	79.81	411	83	0	210	1442	1	411	0.0	699
ABCDEF_24937	UniRef50_Q43314	0.0	Normal: 94	39	122	768	1065	114	BlastAnno: 1144	UniRef50_Q43314	411	87.97	349	42	0	1047	1	1	349	0.0	649
ABCDEF_32993	UniRef50_Q43314	0.0	Normal: 127	299	296	1133	320	898	BlastAnno: 1450	UniRef50_Q43314	411	89.78	411	42	0	1382	150	1	411	0.0	775
ABCDEF_42257	UniRef50_Q43314	0.0	Normal: 140	355	359	1267	415	1032	BlastAnno: 1644	UniRef50_Q43314	411	89.78	411	42	0	1454	222	1	411	0.0	775
1.4.1.4(glutamate dehydrogenase (NADP+);)																					
ABCDEF_41247	UniRef50_P43793	1e-117	Normal: 929	608	918	1231	1278	890	BlastAnno: 2684	UniRef50_P43793	449	42.26	452	250	6	950	2284	1	448	1e-117	374
1.4.3.1(D-aspartate oxidase;)																					
No Correspond Unigene																					
1.4.3.15(D-glutamate(D-aspartate) oxidase;)																					
No Correspond Unigene																					
1.4.3.16(L-aspartate oxidase;)																					
ABCDEF_10434	UniRef50_Q3B5D3	4e-157	Normal: 139	306	137	263	196	849	BlastAnno: 2600	UniRef50_Q3B5D3	537	46.32	544	264	7	1890	274	14	534	4e-157	479
1.4.3.2(L-amino-acid oxidase;)																					
ABCDEF_34035	UniRef50_B9R844	0.0	Normal: 973	600	948	1073	764	972	BlastAnno: 3436	UniRef50_B9R844	1947	69.56	611	156	9	1050	2867	1361	1946	0.0	707
1.5.1.12(1-pyrroline-5-carboxylate dehydrogenase;)																					
ABCDEF_21142	UniRef50_Q8VZC3	0.0	Normal: 587	498	633	913	1134	690	BlastAnno: 2062	UniRef50_Q8VZC3	556	76.18	571	119	3	165	1871	1	556	0.0	916
2.1.3.2(aspartate carbamoyltransferase;)																					
ABCDEF_24647	UniRef50_P49077	3e-180	Normal: 510	109	521	166	447	274	BlastAnno: 1462	UniRef50_P49077	390	79.77	341	64	2	1203	196	50	390	3e-180	519
2.3.1.17(aspartate N-acetyltransferase;)																					
No Correspond Unigene																					
2.4.2.14(amidophosphoribosyltransferase;)																					
ABCDEF_521	UniRef50_P52418	0.0	Normal: 906	973	705	157	354	756	BlastAnno: 2055	UniRef50_P52418	569	81.84	501	83	7	273	1757	60	558	0.0	820
2.6.1.1(aspartate transaminase;)																					
ABCDEF_11690	UniRef50_P46248	0.0	Normal: 1745	1815	2022	4078	5299	6095	BlastAnno: 1778	UniRef50_P46248	453	86.09	417	58	0	297	1547	37	453	0.0	733
ABCDEF_1333	UniRef50_C0PSV4	0.0	Normal: 356	544	636	1119	2495	631	BlastAnno: 1594	UniRef50_C0PSV4	462	76.43	403	95	0	1401	193	60	462	0.0	662
ABCDEF_21786	UniRef50_C0PSV4	0.0	Normal: 268	668	330	546	1267	646	BlastAnno: 1736	UniRef50_C0PSV4	462	74.14	464	113	3	50	1441	4	460	0.0	711
ABCDEF_42262	UniRef50_P46643	0.0	Normal: 916	606	1656	2699	2553	856	BlastAnno: 1722	UniRef50_P46643	430	79.49	429	85	2	1635	355	1	428	0.0	731
ABCDEF_56929	UniRef50_P37833	0.0	Normal: 173	278	312	654	1243	297	BlastAnno: 1151	UniRef50_P37833	407	81.82	308	56	0	978	55	3	310	0.0	536
2.6.1.12(alanine---oxo-acid transaminase;)																					
No Correspond Unigene																					
2.6.1.14(asparagine---oxo-acid transaminase;)																					
No Correspond Unigene																					
2.6.1.15(glutamine---pyruvate transaminase;)																					
No Correspond Unigene																					
2.6.1.16(glutamine---fructose-6-phosphate transaminase (isomerizing);)																					
ABCDEF_10689	UniRef50_Q8SRI2	0.0	Normal: 821	288	776	1014	1042	554	BlastAnno: 2595	UniRef50_Q8SRI2	699	45.60	728	317	13	168	2201	1	699	0.0	626
2.6.1.19(4-aminobutyrate transaminase;)																					
ABCDEF_3775	UniRef50_B8CY58	4e-110	Normal: 473	159	414	817	611	782	BlastAnno: 1801	UniRef50_B8CY58	437	45.37	432	223	7	1570	293	6	430	4e-110	346

2.6.1.2(alanine transaminase;)																						
	ABCDEF_32120	UniRef50_P34106	0.0	Normal: 1312	2052	1485	2115	4153	2982	BlastAnno: 2449	UniRef50_P34106	482	78.87	478	101	0	652	2085	5	482	0.0	763
2.6.1.44(alanine---glyoxylate transaminase;)																						
	ABCDEF_76743	UniRef50_Q9SR86	0.0	Normal: 0	18	0	0	254	0	BlastAnno: 1234	UniRef50_Q9SR86	481	79.37	349	72	0	1	1047	133	481	0.0	595
	ABCDEF_3774	UniRef50_Q9SR86	0.0	Normal: 546	163	404	867	677	914	BlastAnno: 1887	UniRef50_Q9SR86	481	71.46	480	124	2	1779	379	2	481	0.0	712
	ABCDEF_11684	UniRef50_Q9SR86	0.0	Normal: 963	439	718	954	1382	1798	BlastAnno: 1883	UniRef50_Q9SR86	481	62.36	441	166	0	1649	327	41	481	0.0	592
	ABCDEF_3775	UniRef50_Q9SR86	0.0	Normal: 473	159	414	817	611	782	BlastAnno: 1801	UniRef50_Q9SR86	481	71.46	480	124	2	1693	293	2	481	0.0	712
3.5.1.1(asparaginase;)																						
	ABCDEF_13981	UniRef50_Q0E667	3e-157	Normal: 943	1644	1236	3106	2580	499	BlastAnno: 1218	UniRef50_Q0E667	375	61.50	374	93	2	1108	140	2	375	3e-157	457
3.5.1.15(aspartoacylase;)																						
	No Correspend Unigene																					
3.5.1.2(glutaminase;)																						
	ABCDEF_56484	UniRef50_B0TVW4	4e-16	Normal: 9	0	0	0	0	0	BlastAnno: 289	UniRef50_B0TVW4	514	43.37	83	47	0	252	4	428	510	4e-16	79.0
3.5.1.3(omega-amidase;)																						
	ABCDEF_13454	UniRef50_Q9NQR4	3e-103	Normal: 81	70	70	59	99	154	BlastAnno: 1287	UniRef50_Q9NQR4	276	55.84	274	116	1	945	124	1	269	3e-103	317
	ABCDEF_69577	UniRef50_Q9NQR4	3e-103	Normal: 137	102	103	115	146	278	BlastAnno: 1328	UniRef50_Q9NQR4	276	55.84	274	116	1	927	106	1	269	3e-103	317
3.5.1.38(glutamin-(asparagin-)ase)																						
	No Correspend Unigene																					
3.5.1.7(ureidosuccinase)																						
	No Correspend Unigene																					
4.1.1.12(aspartate 4-decarboxylase;)																						
	No Correspend Unigene																					
4.1.1.15(glutamate decarboxylase;)																						
	ABCDEF_11564	UniRef50_Q42472	0.0	Normal: 1009	2635	783	1023	2784	3375	BlastAnno: 2048	UniRef50_Q42472	494	80.20	500	93	3	224	1723	1	494	0.0	846
	ABCDEF_38368	UniRef50_Q42472	0.0	Normal: 893	2322	667	877	2443	2996	BlastAnno: 2255	UniRef50_Q42472	494	86.09	338	46	1	2032	1019	1	337	0.0	624
	ABCDEF_38369	UniRef50_Q42472	0.0	Normal: 802	2006	619	863	2096	2472	BlastAnno: 2237	UniRef50_Q42472	494	89.35	310	33	0	1833	904	28	337	0.0	593
	ABCDEF_38367	UniRef50_Q42472	0.0	Normal: 952	2438	773	996	2638	3216	BlastAnno: 2145	UniRef50_Q42472	494	81.99	472	80	2	1741	326	28	494	0.0	815
4.3.1.1(aspartate ammonia-lyase;)																						
	No Correspend Unigene																					
4.3.2.1(argininosuccinate lyase;)																						
	ABCDEF_27228	UniRef50_Q1Q5N1	4e-163	Normal: 522	249	449	458	484	526	BlastAnno: 1943	UniRef50_Q1Q5N1	460	52.85	456	215	0	244	1611	2	457	4e-163	484
4.3.2.2(adenylosuccinate lyase;)																						
	ABCDEF_6815	UniRef50_P44797	0.0	Normal: 676	385	721	847	893	1064	BlastAnno: 2055	UniRef50_P44797	456	62.56	454	170	0	1710	349	2	455	0.0	603
	ABCDEF_6814	UniRef50_P44797	0.0	Normal: 625	320	680	746	875	784	BlastAnno: 2012	UniRef50_P44797	456	62.33	454	171	0	1667	306	2	455	0.0	602
5.1.1.13(aspartate racemase;)																						
	ABCDEF_14610	UniRef50_B6EHN1	9e-17	Normal: 103	173	62	68	189	470	BlastAnno: 1096	UniRef50_B6EHN1	237	26.07	257	156	11	1048	293	5	232	9e-17	84.3
6.3.1.1(aspartate---ammonia ligase;)																						
	No Correspend Unigene																					
6.3.1.2(glutamate---ammonia ligase;)																						
	ABCDEF_27409	UniRef50_Q56WN1	0.0	Normal: 792	857	694	764	746	4126	BlastAnno: 1892	UniRef50_Q56WN1	356	80.76	343	66	0	460	1488	1	343	0.0	547
	ABCDEF_60010	UniRef50_Q56WN1	0.0	Normal: 722	827	669	717	712	3883	BlastAnno: 1644	UniRef50_Q56WN1	356	80.76	343	66	0	290	1318	1	343	0.0	547
	ABCDEF_27406	UniRef50_Q56WN1	0.0	Normal: 1322	8376	1449	1550	2297	27785	BlastAnno: 1946	UniRef50_Q56WN1	356	79.49	356	73	0	537	1604	1	356	0.0	546
	ABCDEF_38019	UniRef50_Q56WN1	0.0	Normal: 65	126	230	858	520	162	BlastAnno: 1336	UniRef50_Q56WN1	356	85.11	356	53	0	77	1144	1	356	0.0	654
	ABCDEF_28058	UniRef50_Q56WN1	0.0	Normal: 999	55	1008	535	2834	260	BlastAnno: 1368	UniRef50_Q56WN1	356	86.30	343	47	0	93	1121	14	356	0.0	587
	ABCDEF_28620	UniRef50_Q56WN1	0.0	Normal: 2577	3235	3447	8812	12665	2225	BlastAnno: 1455	UniRef50_Q56WN1	356	91.12	338	30	0	169	1182	16	353	0.0	595
6.3.4.16(carbamoyl-phosphate synthase (ammonia);)																						
	No Correspend Unigene																					
6.3.4.4(adenylosuccinate synthase;)																						
	ABCDEF_25360	UniRef50_B7FUM7	5e-155	Normal: 1734	835	1556	1723	2625	1416	BlastAnno: 2000	UniRef50_B7FUM7	526	51.33	450	200	5	1743	403	78	511	5e-155	467
6.3.4.5(argininosuccinate synthase;)																						
	ABCDEF_57250	UniRef50_Q24ZG8	0.0	Normal: 1037	382	856	747	1026	1222	BlastAnno: 1645	UniRef50_Q24ZG8	409	62.22	397	149	1	378	1568	9	404	0.0	525
	ABCDEF_28455	UniRef50_Q9SZX3	0.0	Normal: 1243	467	989	866	1234	1550	BlastAnno: 1756	UniRef50_Q9SZX3	494	79.74	459	90	2	296	1669	36	492	0.0	756
6.3.5.4(asparagine synthase (glutamine-hydrolysing);)																						
	ABCDEF_9351	UniRef50_Q83S76	0.0	Normal: 45	29	8	0	0	5615	BlastAnno: 2191	UniRef50_Q83S76	515	53.32	557	216	7	131	1795	1	515	0.0	556
	ABCDEF_82324	UniRef50_Q83S76	0.0	Normal: 7	19	0	0	0	2399	BlastAnno: 2218	UniRef50_Q83S76	515	52.96	557	218	7	186	1850	1	515	0.0	550
	ABCDEF_11540	UniRef50_P49078	0.0	Normal: 2080	820	1576	1695	1260	2315	BlastAnno: 2183	UniRef50_P49078	584	78.38	555	120	0	1989	325	1	555	0.0	896
6.3.5.5(carbamoyl-phosphate synthase (glutamine-hydrolysing);)																						
	ABCDEF_48168	UniRef50_A7HVJ4	0.0	Normal: 1604	1009	1521	1682	1283	1898	BlastAnno: 4056	UniRef50_A7HVJ4	1079	58.58	1096	423	10	3524	273	3	1079	0.0	1242
	ABCDEF_10940	UniRef50_A7HVJ4	0.0	Normal: 464	188	321	214	331	350	BlastAnno: 1730	UniRef50_A7HVJ4	1079	58.66	583	214	6	3	1715	274	841	0.0	671
>00253 Tetracycline biosynthesis																						
1.1.1.-(Oxidoreductases;)																						
	ABCDEF_13453	UniRef50_Q9SYM5	0.0	Normal: 225	226	398	1950	2026	421	BlastAnno: 2578	UniRef50_Q9SYM5	669	86.61	672	87	2	298	2313	1	669	0.0	1226
	ABCDEF_17342	UniRef50_Q84M96	0.0	Normal: 274	406	272	327	439	625	BlastAnno: 1277	UniRef50_Q84M96	346	73.33	345	91	1	1187	156	2	346	0.0	546
	ABCDEF_31147	UniRef50_Q9SYM5	0.0	Normal: 4571	1591	1491	1401	1939	4327	BlastAnno: 2465	UniRef50_Q9SYM5	669	82.76	673	107	4	2397	394	1	669	0.0	1154
	ABCDEF_17344	UniRef50_Q84M96	0.0	Normal: 252	373	257	297	380	540	BlastAnno: 1350	UniRef50_Q84M96	346	68.19	371	91	2	1263	154	2	346	0.0	530
	ABCDEF_44674	UniRef50_Q9SYM5	0.0	Normal: 3581	2620	4329	9505	11447	4930	BlastAnno: 2634	UniRef50_Q9SYM5	669	87.80	672	78	3	253	2265	1	669	0.0	1241
1.14.-.-(Oxidoreductases;)																						
	ABCDEF_44739	UniRef50_O64989	0.0	Normal: 157	87	159	718	673	189	BlastAnno: 2185	UniRef50_O64989	513	70.75	482	112	3	1867	509	30	511	0.0	706
	ABCDEF_31491	UniRef50_Q93VK5	0.0	Normal: 647	1826	671	1517	1770	5953	BlastAnno: 2278	UniRef50_Q93VK5	595	79.14	556	113	2	198	1862	35	588	0.0	902
	ABCDEF_40554	UniRef50_P37122	0.0	Normal: 1574	1962	1829	1150	2882	2721	BlastAnno: 1828	UniRef50_P37122	505	60.52	504	198	1	1752	244	1	504	0.0	638
	ABCDEF_34049	UniRef50_O23365	0.0	Normal: 363	384	349	442	549	1416	BlastAnno: 2101	UniRef50_O23365	580	76.17	579	116	5	230	1957	19	578	0.0	904
	ABCDEF_21579	UniRef50_O48928	0.0	Normal: 1005	643	1606	4372	3226	1621	BlastAnno: 1895	UniRef50_O48928	513	69.72	502	151	1	185	1690	7	507	0.0	731

ABCDEF_37741	UniRef50_P37122	0.0	Normal: 12	760	9	24	34	576	BlastAnno: 1197	UniRef50_P37122	505	65.70	379	129	1	1195	59	127	504	0.0	526
ABCDEF_35618	UniRef50_O48928	0.0	Normal: 28	2648	17	142	1234	902	BlastAnno: 1746	UniRef50_O48928	513	66.40	506	168	2	1649	132	4	507	0.0	701
ABCDEF_24522	UniRef50_P37122	0.0	Normal: 334	2190	561	268	1391	2459	BlastAnno: 2075	UniRef50_P37122	505	61.52	460	176	1	1544	165	47	505	0.0	612
ABCDEF_14592	UniRef50_O23066	0.0	Normal: 1514	402	2245	7837	4742	1248	BlastAnno: 2378	UniRef50_O23066	553	72.71	546	140	4	2111	489	1	542	0.0	821
ABCDEF_14512	UniRef50_Q9FG65	0.0	Normal: 261	872	212	291	175	789	BlastAnno: 1878	UniRef50_Q9FG65	502	56.89	501	197	9	1738	266	11	502	0.0	532
ABCDEF_44487	UniRef50_Q940V4	0.0	Normal: 3	41	53	501	262	542	BlastAnno: 1712	UniRef50_Q940V4	465	69.37	382	116	1	38	1180	14	395	0.0	572
ABCDEF_17723	UniRef50_P37122	0.0	Normal: 240	1698	219	768	592	794	BlastAnno: 1757	UniRef50_P37122	505	63.40	459	166	2	228	1601	47	504	0.0	617
ABCDEF_37901	UniRef50_Q42569	0.0	Normal: 30	149	86	255	195	255	BlastAnno: 1246	UniRef50_Q42569	472	78.27	359	73	2	64	1140	43	396	0.0	567
ABCDEF_7260	UniRef50_O81117	0.0	Normal: 314	1085	466	3248	2575	1360	BlastAnno: 1768	UniRef50_O81117	514	59.92	474	188	1	145	1566	43	514	0.0	615
ABCDEF_40548	UniRef50_P37122	0.0	Normal: 2756	2467	2752	1072	3120	1735	BlastAnno: 1947	UniRef50_P37122	505	59.72	504	202	1	241	1749	1	504	0.0	636
ABCDEF_44485	UniRef50_Q940V4	0.0	Normal: 3	55	63	574	338	660	BlastAnno: 1607	UniRef50_Q940V4	465	68.58	452	141	1	42	1394	14	465	0.0	673
ABCDEF_40552	UniRef50_P37122	0.0	Normal: 2397	1870	2304	1249	10428	2291	BlastAnno: 1812	UniRef50_P37122	505	60.12	504	200	1	108	1616	1	504	0.0	640
ABCDEF_37902	UniRef50_Q42569	0.0	Normal: 84	524	254	725	670	1049	BlastAnno: 1812	UniRef50_Q42569	472	77.47	435	92	3	1675	371	43	471	0.0	681
ABCDEF_40556	UniRef50_P37122	0.0	Normal: 1907	2365	2163	1319	3678	3308	BlastAnno: 1944	UniRef50_P37122	505	61.04	498	193	1	264	1754	1	498	0.0	635
ABCDEF_40549	UniRef50_P37122	0.0	Normal: 1574	1962	1826	1150	2882	2723	BlastAnno: 1845	UniRef50_P37122	505	60.52	504	198	1	1754	246	1	504	0.0	638
ABCDEF_20301	UniRef50_O81117	0.0	Normal: 89	308	123	176	389	249	BlastAnno: 1637	UniRef50_O81117	514	64.04	470	164	3	1498	98	47	514	0.0	586
ABCDEF_3882	UniRef50_Q42569	0.0	Normal: 84	243	257	786	1561	182	BlastAnno: 1854	UniRef50_Q42569	472	72.51	422	107	1	1852	587	59	471	0.0	615
ABCDEF_20703	UniRef50_O49858	0.0	Normal: 115	636	20	0	0	597	BlastAnno: 1815	UniRef50_O49858	527	50.10	523	248	6	48	1607	12	524	0.0	533
ABCDEF_18295	UniRef50_O22203	0.0	Normal: 3667	1934	4977	10491	4588	1917	BlastAnno: 2006	UniRef50_O22203	508	74.85	493	123	1	214	1692	17	508	0.0	803
ABCDEF_48724	UniRef50_O23066	0.0	Normal: 104	11518	163	2083	3464	6356	BlastAnno: 2162	UniRef50_O23066	553	72.96	540	143	3	227	1840	1	539	0.0	807
ABCDEF_37738	UniRef50_P37122	0.0	Normal: 26	2447	19	30	33	993	BlastAnno: 1837	UniRef50_P37122	505	64.30	479	169	2	1685	249	28	504	0.0	639

1.14.13.38(anhydrotetracycline monooxygenase;)

No Correspond Unigene

1.14.14.-(Oxidoreductases;)

No Correspond Unigene

2.1.1.-(Transferases;)

ABCDEF_20806	UniRef50_Q94II3	0.0	Normal: 700	332	876	1458	1997	929	BlastAnno: 2275	UniRef50_Q94II3	600	73.57	594	153	4	2119	341	1	591	0.0	907
ABCDEF_45802	UniRef50_Q9ZPH9	0.0	Normal: 3543	3348	4296	9797	10830	5017	BlastAnno: 2642	UniRef50_Q9ZPH9	633	53.90	603	261	7	2216	456	23	624	0.0	676
ABCDEF_20070	UniRef50_Q944R7	0.0	Normal: 301	141	265	207	185	187	BlastAnno: 2675	UniRef50_Q944R7	724	58.28	719	275	9	127	2265	13	712	0.0	840
ABCDEF_34956	UniRef50_Q94II3	0.0	Normal: 1120	7269	2475	16033	22219	21461	BlastAnno: 2676	UniRef50_Q94II3	600	73.77	610	146	4	404	2230	1	597	0.0	957
ABCDEF_17121	UniRef50_Q9ZPH9	0.0	Normal: 124	77	109	284	199	132	BlastAnno: 2066	UniRef50_Q9ZPH9	633	53.24	601	259	8	161	1903	25	623	0.0	644
ABCDEF_6524	UniRef50_Q9ZPH9	0.0	Normal: 471	296	490	753	870	730	BlastAnno: 2602	UniRef50_Q9ZPH9	633	50.89	619	290	5	2221	404	6	623	0.0	659
ABCDEF_56017	UniRef50_Q9ZPH9	0.0	Normal: 302	167	286	596	748	529	BlastAnno: 2384	UniRef50_Q9ZPH9	633	51.37	619	287	5	213	2030	6	623	0.0	663
ABCDEF_44169	UniRef50_Q9SIZ3	0.0	Normal: 179	158	273	705	685	229	BlastAnno: 2300	UniRef50_Q9SIZ3	589	62.01	308	108	4	1219	296	285	583	0.0	394
ABCDEF_6521	UniRef50_Q9ZPH9	0.0	Normal: 339	198	314	641	767	582	BlastAnno: 2511	UniRef50_Q9ZPH9	633	51.37	619	287	5	2172	355	6	623	0.0	663
ABCDEF_44172	UniRef50_Q9SIZ3	0.0	Normal: 204	184	287	767	741	242	BlastAnno: 2317	UniRef50_Q9SIZ3	589	68.15	449	134	4	1637	291	144	583	0.0	641
ABCDEF_199	UniRef50_Q9SU94	0.0	Normal: 1076	275	1009	558	1247	306	BlastAnno: 1769	UniRef50_Q9SU94	390	78.45	348	74	1	284	1324	43	390	0.0	584
ABCDEF_52252	UniRef50_Q84W92	0.0	Normal: 1658	720	1653	1980	2289	1601	BlastAnno: 2170	UniRef50_Q84W92	535	72.49	469	120	3	409	1791	34	501	0.0	703
ABCDEF_51404	UniRef50_A3BMN9	0.0	Normal: 864	188	716	337	631	326	BlastAnno: 2192	UniRef50_A3BMN9	620	50.50	619	243	9	2008	251	55	620	0.0	555
ABCDEF_21386	UniRef50_Q94AI4	0.0	Normal: 2326	1250	2220	4294	2920	1983	BlastAnno: 3701	UniRef50_Q94AI4	775	47.27	842	332	16	567	2978	8	775	0.0	577
ABCDEF_35101	UniRef50_Q9MAT5	0.0	Normal: 1440	420	1268	576	1136	850	BlastAnno: 1634	UniRef50_Q9MAT5	383	69.03	352	109	0	1449	394	32	383	0.0	550
ABCDEF_960	UniRef50_Q9LN50	0.0	Normal: 3504	13198	3609	11156	19651	12155	BlastAnno: 2626	UniRef50_Q9LN50	724	49.14	525	246	5	883	2451	219	724	0.0	570
ABCDEF_48082	UniRef50_Q9LN50	0.0	Normal: 410	445	467	870	993	454	BlastAnno: 2340	UniRef50_Q9LN50	724	70.25	521	155	0	710	2272	204	724	0.0	821
ABCDEF_10473	UniRef50_Q08A71	0.0	Normal: 374	187	332	425	456	314	BlastAnno: 1764	UniRef50_Q08A71	435	79.23	366	75	1	344	1438	70	435	0.0	575
ABCDEF_7295	UniRef50_Q9ZPH9	0.0	Normal: 25	54	30	81	26	329	BlastAnno: 1191	UniRef50_Q9ZPH9	633	69.23	403	110	4	1191	22	225	626	0.0	599
ABCDEF_4160	UniRef50_Q94II3	0.0	Normal: 189	0	210	71	17	72	BlastAnno: 1424	UniRef50_Q94II3	600	77.28	449	101	1	1424	81	143	591	0.0	758
ABCDEF_44857	UniRef50_Q9ZPH9	0.0	Normal: 101	224	103	299	588	350	BlastAnno: 1582	UniRef50_Q9ZPH9	633	72.06	519	143	1	1571	21	114	632	0.0	822
ABCDEF_71541	UniRef50_Q9SIZ3	0.0	Normal: 187	167	296	765	724	237	BlastAnno: 2268	UniRef50_Q9SIZ3	589	62.01	308	108	4	1210	287	285	583	0.0	394
ABCDEF_44171	UniRef50_Q9SIZ3	0.0	Normal: 214	196	312	827	802	251	BlastAnno: 2339	UniRef50_Q9SIZ3	589	64.78	548	182	5	1978	335	47	583	0.0	717
ABCDEF_20805	UniRef50_Q94II3	0.0	Normal: 674	344	885	1417	2194	963	BlastAnno: 2400	UniRef50_Q94II3	600	78.21	436	94	1	879	2183	156	591	0.0	734
ABCDEF_18210	UniRef50_Q9SIZ3	0.0	Normal: 5584	3714	7379	17006	21892	7546	BlastAnno: 3199	UniRef50_Q9SIZ3	589	50.00	576	269	7	2145	424	27	585	0.0	574
ABCDEF_6526	UniRef50_Q9ZPH9	0.0	Normal: 627	185	837	2717	2815	1530	BlastAnno: 2611	UniRef50_Q9ZPH9	633	54.76	599	259	5	462	2222	25	623	0.0	689
ABCDEF_6525	UniRef50_Q9ZPH9	0.0	Normal: 602	165	760	2369	2605	1400	BlastAnno: 2545	UniRef50_Q9ZPH9	633	54.92	599	258	5	431	2191	25	623	0.0	689
ABCDEF_6523	UniRef50_Q9ZPH9	0.0	Normal: 314	175	304	641	784	552	BlastAnno: 2312	UniRef50_Q9ZPH9	633	52.42	599	272	4	201	1958	25	623	0.0	663
ABCDEF_67393	UniRef50_Q9ZPH9	0.0	Normal: 434	245	444	608	768	655	BlastAnno: 2346	UniRef50_Q9ZPH9	633	51.92	599	275	4	232	1989	25	623	0.0	659
ABCDEF_3579	UniRef50_O14744	0.0	Normal: 1361	326	984	568	791	440	BlastAnno: 2369	UniRef50_O14744	637	47.00	634	294	10	267	2120	30	637	0.0	553

2.3.1.-(Transferases;)

ABCDEF_520	UniRef50_Q9FF86	0.0	Normal: 1672	85	2273	5056	3383	1211	BlastAnno: 1811	UniRef50_Q9FF86	484	64.06	448	149	6	1664	354	3	449	0.0	573
ABCDEF_68664	UniRef50_Q9ZVN2	0.0	Normal: 79	35	85	107	193	63	BlastAnno: 1488	UniRef50_Q9ZVN2	704	63.99	411	142	3	46	1275	298	703	0.0	540
ABCDEF_52245	UniRef50_Q9FG87	0.0	Normal: 1878	635	1833	3814	1432	1376	BlastAnno: 1758	UniRef50_Q9FG87	529	55.35	486	204	4	4	1428	34	517	0.0	530
ABCDEF_16730	UniRef50_Q3EC11	0.0	Normal: 281	204	237	365	377	458	BlastAnno: 2092	UniRef50_Q3EC11	536	59.33	536	204	8	196	1785	3	530	0.0	661
ABCDEF_4655	UniRef50_A5BMG1	0.0	Normal: 2426	1856	3606	6958	4608	2755	BlastAnno: 2261	UniRef50_A5BMG1	481	68.37	528	112	10	263	1843	3	476	0.0	667
ABCDEF_13860	UniRef50_Q9C533	0.0	Normal: 588	220	570	1451	1338	310	BlastAnno: 2648	UniRef50_Q9C533	596	63.37	606	177	12	2226	511	1	595	0.0	558
ABCDEF_28294	UniRef50_Q9SYZ0	0.0	Normal: 1038	4070	1427	6745	877	2797	BlastAnno: 1785	UniRef50_Q9SYZ0	493	52.37	485	223	3	1598	162	5	487	0.0	533
ABCDEF_47718	UniRef50_Q9C533	0.0	Normal: 1599	236	1657	2093	1231	588	BlastAnno: 2680	UniRef50_Q9C533	596	65.02	626	165	14	2280	472	1	595	0.0	634
ABCDEF_45080	UniRef50_Q9SYZ0	0.0	Normal: 1005	592	1079	2360	3762	2217	BlastAnno: 2130	UniRef50_Q9SYZ0	493	57.85	484	197	3	302	1735	5	487	0.0	533
ABCDEF_25377	UniRef50_Q9C6L5	0.0	Normal: 37	2090	138	2573	1026	1298	BlastAnno: 1334	UniRef50_Q9C6L5	492	79.74	385	76	1	1155	1	6	388	0.0	654
ABCDEF_33470	UniRef50_Q9LW26	0.0	Normal: 172	98	156	260	391	149	BlastAnno: 2993	UniRef50_Q9LW26	701	48.96	627	301	5	655	2508	85	701	0.0	581
ABCDEF_40654	UniRef50_Q9H0A0	0.0	Normal: 638	213	530	318	318	286	BlastAnno: 2216	UniRef50_Q9H0A0	1025	56.87	721	279	6	2127	13	91	795	0.0	756
ABCDEF_33460	UniRef50_Q9ZVN2	0.0	Normal: 144	71	119	216	306	97	BlastAnno: 2691	UniRef50_Q9ZVN2	704	61.50	587	191	4	379	2133	83	636	0.0	715
ABCDEF_14442	UniRef50_Q9SYZ0	0.0	Normal: 341	684	439	1040	1601	552	BlastAnno: 1779	UniRef50_Q9SYZ0	493	63.12	480	171	2	1570	149	5	484	0.0	628
ABCDEF_52626	UniRef50_B6SYP9	0.0	Normal: 815	346	1131	3015	1537	108	BlastAnno: 1861	UniRef50_B6SYP9	494	55.02	438	189	3	1538	249	51	488	0.0	536

ABCDEF_644	UniRef50_Q9SB58	0.0	Normal: 704	444	913	969	1431	848	BlastAnno: 1777	UniRef50_Q9SB58	407	64.15	424	129	8	1572	316	1	406	0.0	554
ABCDEF_27087	UniRef50_Q52T38	0.0	Normal: 324	221	233	419	343	412	BlastAnno: 1631	UniRef50_Q52T38	620	75.95	420	91	1	1409	150	1	410	0.0	681
ABCDEF_34187	UniRef50_Q8L5Y5	0.0	Normal: 497	415	588	523	291	433	BlastAnno: 2677	UniRef50_Q8L5Y5	718	57.30	733	251	15	202	2274	1	713	0.0	683
ABCDEF_47966	UniRef50_Q9SYZ0	0.0	Normal: 577	934	326	580	682	3440	BlastAnno: 2177	UniRef50_Q9SYZ0	493	56.40	484	204	3	1780	347	5	487	0.0	535
ABCDEF_51189	UniRef50_Q52T38	0.0	Normal: 135	152	128	181	219	327	BlastAnno: 1766	UniRef50_Q52T38	620	68.09	470	137	3	80	1483	130	588	0.0	641
ABCDEF_41288	UniRef50_Q9SB58	0.0	Normal: 307	183	332	637	649	322	BlastAnno: 1403	UniRef50_Q9SB58	407	67.56	410	110	6	1401	184	15	405	0.0	523
ABCDEF_33461	UniRef50_Q9ZVN2	0.0	Normal: 142	84	140	206	283	119	BlastAnno: 2904	UniRef50_Q9ZVN2	704	60.98	574	201	6	2271	559	83	636	0.0	686
ABCDEF_14334	UniRef50_Q9SYZ0	0.0	Normal: 2263	129	1724	3469	866	283	BlastAnno: 2039	UniRef50_Q9SYZ0	493	63.22	484	171	2	369	1802	5	487	0.0	627
ABCDEF_24844	UniRef50_Q9FF86	0.0	Normal: 1857	3686	2657	21940	11467	4090	BlastAnno: 1640	UniRef50_Q9FF86	484	66.89	447	135	6	1492	185	2	446	0.0	575
ABCDEF_51190	UniRef50_Q52T38	0.0	Normal: 145	169	142	210	232	335	BlastAnno: 1891	UniRef50_Q52T38	620	70.04	514	141	3	73	1608	86	588	0.0	726
ABCDEF_14085	UniRef50_Q8L5Y5	0.0	Normal: 353	41	384	527	331	280	BlastAnno: 2362	UniRef50_Q8L5Y5	718	62.07	704	220	12	2360	348	24	713	0.0	676
ABCDEF_33458	UniRef50_Q9LW26	0.0	Normal: 158	78	130	240	342	106	BlastAnno: 2822	UniRef50_Q9LW26	701	46.72	655	302	5	400	2337	85	701	0.0	568
ABCDEF_25193	UniRef50_Q9C6L5	0.0	Normal: 3256	368	3548	6230	5109	1288	BlastAnno: 1786	UniRef50_Q9C6L5	492	65.83	480	156	4	1668	241	17	492	0.0	668
ABCDEF_33457	UniRef50_Q9ZVN2	0.0	Normal: 168	83	137	250	375	112	BlastAnno: 2823	UniRef50_Q9ZVN2	704	65.12	496	168	3	379	1860	83	575	0.0	648
ABCDEF_7634	UniRef50_Q9C6L5	0.0	Normal: 2543	9303	2711	11578	5533	10032	BlastAnno: 2064	UniRef50_Q9C6L5	492	52.76	489	225	3	1734	268	1	483	0.0	535
ABCDEF_299	UniRef50_Q71N54	0.0	Normal: 569	1147	511	900	649	1879	BlastAnno: 2017	UniRef50_Q71N54	535	61.73	533	198	5	338	1924	4	534	0.0	677
ABCDEF_27086	UniRef50_Q52T38	0.0	Normal: 784	470	617	843	893	976	BlastAnno: 2398	UniRef50_Q52T38	620	70.80	637	165	4	225	2129	1	618	0.0	887
ABCDEF_51188	UniRef50_Q52T38	0.0	Normal: 273	327	255	415	349	441	BlastAnno: 2324	UniRef50_Q52T38	620	71.30	568	150	3	2041	344	32	588	0.0	815
ABCDEF_44812	UniRef50_Q6DR03	0.0	Normal: 982	902	1005	1071	959	2999	BlastAnno: 2450	UniRef50_Q6DR03	565	54.97	644	198	17	2142	220	1	555	0.0	540
4.2.1.-(Lyases;)																					
ABCDEF_48679	UniRef50_Q0RP29	5e-115	Normal: 568	325	563	941	1285	676	BlastAnno: 1424	UniRef50_Q0RP29	493	56.39	321	129	4	119	1075	39	350	5e-115	356
6.3.5.-(Ligases;)																					
ABCDEF_33655	UniRef50_Q8DGC4	0.0	Normal: 195	217	224	369	275	584	BlastAnno: 2282	UniRef50_Q8DGC4	500	60.04	493	188	4	1703	246	7	497	0.0	602
ABCDEF_33653	UniRef50_Q8DGC4	0.0	Normal: 176	252	216	383	304	625	BlastAnno: 2403	UniRef50_Q8DGC4	500	59.84	493	189	4	1791	334	7	497	0.0	602
ABCDEF_45031	UniRef50_Q7X8Z8	0.0	Normal: 533	338	492	538	373	1845	BlastAnno: 2074	UniRef50_Q7X8Z8	497	73.15	447	117	2	154	1485	50	496	0.0	617
ABCDEF_33650	UniRef50_Q8DGC4	0.0	Normal: 173	192	212	343	239	489	BlastAnno: 2066	UniRef50_Q8DGC4	500	60.04	493	188	4	1526	69	7	497	0.0	604
ABCDEF_33651	UniRef50_Q8DGC4	0.0	Normal: 203	236	286	370	292	585	BlastAnno: 2693	UniRef50_Q8DGC4	500	59.84	493	189	4	2114	657	7	497	0.0	602
ABCDEF_33654	UniRef50_Q8DGC4	0.0	Normal: 163	239	184	335	284	658	BlastAnno: 2323	UniRef50_Q8DGC4	500	59.84	493	189	4	613	2070	7	497	0.0	602
6.4.1.2(acetyl-CoA carboxylase;)																					
ABCDEF_27465	UniRef50_P78820	0.0	Normal: 4589	3602	6382	22310	7761	4482	BlastAnno: 7477	UniRef50_P78820	2280	40.33	2145	1127	43	7128	919	55	2121	0.0	1462
>00260 Glycine, serine and threonine metabolism																					
1.1.1.-(Oxidoreductases;)																					
ABCDEF_13453	UniRef50_Q9SYM5	0.0	Normal: 225	226	398	1950	2026	421	BlastAnno: 2578	UniRef50_Q9SYM5	669	86.61	672	87	2	298	2313	1	669	0.0	1226
ABCDEF_17342	UniRef50_Q84M96	0.0	Normal: 274	406	272	327	439	625	BlastAnno: 1277	UniRef50_Q84M96	346	73.33	345	91	1	1187	156	2	346	0.0	546
ABCDEF_31147	UniRef50_Q9SYM5	0.0	Normal: 4571	1591	1491	1401	1939	4327	BlastAnno: 2465	UniRef50_Q9SYM5	669	82.76	673	107	4	2397	394	1	669	0.0	1154
ABCDEF_17344	UniRef50_Q84M96	0.0	Normal: 252	373	257	297	380	540	BlastAnno: 1350	UniRef50_Q84M96	346	68.19	371	91	2	1263	154	2	346	0.0	530
ABCDEF_44674	UniRef50_Q9SYM5	0.0	Normal: 3581	2620	4329	9505	11447	4930	BlastAnno: 2634	UniRef50_Q9SYM5	669	87.80	672	78	3	253	2265	1	669	0.0	1241
1.1.1.1(alcohol dehydrogenase;)																					
ABCDEF_31928	UniRef50_B9RHN6	0.0	Normal: 721	714	784	1329	1570	1819	BlastAnno: 1711	UniRef50_B9RHN6	437	77.55	392	87	1	360	1535	46	436	0.0	610
ABCDEF_10335	UniRef50_Q8LEB2	0.0	Normal: 285	158	514	810	352	183	BlastAnno: 1547	UniRef50_Q8LEB2	381	74.93	379	92	1	1317	181	5	380	0.0	598
1.1.1.103(L-threonine 3-dehydrogenase;)																					
No Correspond Unigene																					
1.1.1.29(glycerate dehydrogenase;)																					
ABCDEF_38443	UniRef50_P13443	0.0	Normal: 996	6551	645	477	900	18941	BlastAnno: 1745	UniRef50_P13443	382	89.21	380	41	0	326	1465	1	380	0.0	679
1.1.1.3(homoserine dehydrogenase;)																					
ABCDEF_30670	UniRef50_C6W787	0.0	Normal: 1382	736	1142	1007	1095	1298	BlastAnno: 3147	UniRef50_C6W787	818	40.10	828	481	7	391	2865	3	818	0.0	625
ABCDEF_31297	UniRef50_C6W787	0.0	Normal: 3828	2128	2645	751	1881	2287	BlastAnno: 3215	UniRef50_C6W787	818	41.06	828	472	8	2751	280	3	818	0.0	611
1.1.1.75(No Name)																					
No Correspond Unigene																					
1.1.1.81(hydroxypyruvate reductase;)																					
No Correspond Unigene																					
1.1.1.95(phosphoglycerate dehydrogenase;)																					
ABCDEF_13990	UniRef50_O04130	0.0	Normal: 487	278	487	447	1745	311	BlastAnno: 1815	UniRef50_O04130	624	80.46	481	93	1	1815	373	145	624	0.0	754
ABCDEF_35317	UniRef50_O04130	0.0	Normal: 3413	458	3803	2964	1953	680	BlastAnno: 1723	UniRef50_O04130	624	78.60	500	107	0	1	1500	125	624	0.0	736
ABCDEF_45312	UniRef50_O04130	0.0	Normal: 879	2767	1113	1869	8492	758	BlastAnno: 1813	UniRef50_O04130	624	83.82	513	83	0	1812	274	112	624	0.0	853
1.1.3.17(choline oxidase)																					
No Correspond Unigene																					
1.1.99.1(choline dehydrogenase;)																					
No Correspond Unigene																					
1.14.11.-(Oxidoreductases;)																					
ABCDEF_37086	UniRef50_Q8GUI6	0.0	Normal: 592	429	529	801	742	784	BlastAnno: 3943	UniRef50_Q8GUI6	954	56.60	553	227	4	613	2244	38	586	0.0	643
ABCDEF_51324	UniRef50_Q8GUI6	0.0	Normal: 461	353	534	1098	841	490	BlastAnno: 3289	UniRef50_Q8GUI6	954	60.79	584	214	5	2989	1262	12	588	0.0	698
1.14.15.7(choline monooxygenase)																					
ABCDEF_13587	UniRef50_Q9SZR0	3e-107	Normal: 46	43	49	98	99	93	BlastAnno: 977	UniRef50_Q9SZR0	422	72.00	225	59	3	114	779	186	409	3e-107	328
1.2.1.11(aspartate-semialdehyde dehydrogenase;)																					
ABCDEF_45337	UniRef50_A4YV18	9e-115	Normal: 811	241	805	357	570	636	BlastAnno: 1520	UniRef50_A4YV18	341	54.90	337	145	3	230	1237	5	335	9e-115	351
1.2.1.8(betaine-aldehyde dehydrogenase;)																					
ABCDEF_13229	UniRef50_Q9STS1	0.0	Normal: 1788	1300	2166	3891	5328	2533	BlastAnno: 1952	UniRef50_Q9STS1	503	72.00	500	135	2	1758	271	3	501	0.0	700
ABCDEF_13230	UniRef50_Q9STS1	0.0	Normal: 472	142	291	124	487	238	BlastAnno: 1273	UniRef50_Q9STS1	503	75.07	337	83	1	1273	263	168	503	0.0	533
ABCDEF_13232	UniRef50_Q9STS1	0.0	Normal: 944	656	1133	1939	2713	1255	BlastAnno: 1566	UniRef50_Q9STS1	503	73.86	440	114	1	12	1331	65	503	0.0	663
1.4.2.1(glycine dehydrogenase (cytochrome);)																					
No Correspond Unigene																					

1.4.3.21(primary-amine oxidase;)																					
ABCDEF_3522	UniRef50_Q8H1H9	0.0	Normal: 405	723	295	151	922	46	BlastAnno: 2086	UniRef50_Q8H1H9	712	64.54	595	204	6	2	1774	116	707	0.0	811
ABCDEF_3521	UniRef50_Q8H1H9	0.0	Normal: 322	637	263	137	709	53	BlastAnno: 1768	UniRef50_Q8H1H9	712	68.24	510	156	5	1630	113	200	707	0.0	744
1.4.3.3(D-amino-acid oxidase;)																					
No Correspond Unigene																					
1.4.3.4(monoamine oxidase;)																					
ABCDEF_11940	UniRef50_Q8LL67	3e-122	Normal: 41	0	82	2	541	4	BlastAnno: 1557	UniRef50_Q8LL67	496	45.61	410	204	3	2	1183	74	480	3e-122	376
1.4.4.2(glycine dehydrogenase (decarboxylating);)																					
ABCDEF_31013	UniRef50_Q8YNF9	0.0	Normal: 1168	13982	625	549	626	26766	BlastAnno: 3892	UniRef50_Q8YNF9	983	59.33	959	371	9	3107	255	32	979	0.0	1162
ABCDEF_27663	UniRef50_Q8YNF9	0.0	Normal: 1754	787	1497	3999	4393	558	BlastAnno: 3492	UniRef50_Q8YNF9	983	59.29	958	369	8	3047	201	32	977	0.0	1144
1.5.3.1(sarcosine oxidase)																					
ABCDEF_4294	UniRef50_Q9SJA7	5e-123	Normal: 327	31	305	291	692	50	BlastAnno: 1171	UniRef50_Q9SJA7	416	62.46	285	104	2	317	1168	9	291	5e-123	370
1.5.99.1(sarcosine dehydrogenase;)																					
No Correspond Unigene																					
1.5.99.2(dimethylglycine dehydrogenase;)																					
No Correspond Unigene																					
1.8.1.4(dihydrolipoyl dehydrogenase;)																					
ABCDEF_573	UniRef50_P72740	0.0	Normal: 1422	809	1248	2548	1822	4200	BlastAnno: 2189	UniRef50_P72740	474	61.95	473	173	5	469	1878	5	473	0.0	556
2.1.1.2(guanidinoacetate N-methyltransferase;)																					
No Correspond Unigene																					
2.1.1.20(glycine N-methyltransferase;)																					
No Correspond Unigene																					
2.1.1.5(betaine---homocysteine S-methyltransferase;)																					
No Correspond Unigene																					
2.1.2.1(glycine hydroxymethyltransferase;)																					
ABCDEF_41184	UniRef50_C5KPS8	0.0	Normal: 509	437	722	1807	2474	561	BlastAnno: 2201	UniRef50_C5KPS8	607	62.56	446	164	2	1762	425	164	606	0.0	590
ABCDEF_11573	UniRef50_P34897	0.0	Normal: 1903	1387	2092	2286	3254	2865	BlastAnno: 2080	UniRef50_P34897	504	60.18	457	170	4	502	1845	48	501	0.0	573
ABCDEF_37629	UniRef50_Q9LM59	0.0	Normal: 412	460	499	689	962	979	BlastAnno: 2512	UniRef50_Q9LM59	599	66.00	597	187	5	369	2126	8	599	0.0	813
ABCDEF_7224	UniRef50_C5KPS8	0.0	Normal: 360	5392	231	401	266	13616	BlastAnno: 2246	UniRef50_C5KPS8	607	61.88	446	167	2	1828	491	164	606	0.0	577
ABCDEF_51490	UniRef50_Q9LM59	0.0	Normal: 497	502	504	755	1027	1332	BlastAnno: 2919	UniRef50_Q9LM59	599	64.38	584	197	2	578	2299	17	599	0.0	754
ABCDEF_1167	UniRef50_Q9LM59	0.0	Normal: 5116	9848	6479	16268	26840	14685	BlastAnno: 1891	UniRef50_Q9LM59	599	62.07	464	171	1	1747	371	134	597	0.0	611
ABCDEF_7223	UniRef50_C5KPS8	0.0	Normal: 395	5706	275	418	261	13912	BlastAnno: 2154	UniRef50_C5KPS8	607	61.88	446	167	2	1736	399	164	606	0.0	577
ABCDEF_28355	UniRef50_P34897	0.0	Normal: 255	239	370	837	1312	358	BlastAnno: 1812	UniRef50_P34897	504	57.73	466	153	3	1810	416	81	503	0.0	553
2.1.2.10(aminomethyltransferase;)																					
ABCDEF_22217	UniRef50_O65396	0.0	Normal: 1753	5094	1871	4253	3113	17559	BlastAnno: 1818	UniRef50_O65396	408	87.47	407	51	0	363	1583	1	407	0.0	752
2.1.4.1(glycine amidinotransferase;)																					
No Correspond Unigene																					
2.3.1.178(diaminobutyrate acetyltransferase;)																					
No Correspond Unigene																					
2.3.1.29(glycine C-acetyltransferase;)																					
No Correspond Unigene																					
2.3.1.37(5-aminolevulinate synthase;)																					
No Correspond Unigene																					
2.6.1.4(glycine transaminase;)																					
No Correspond Unigene																					
2.6.1.44(alanine---glyoxylate transaminase;)																					
ABCDEF_76743	UniRef50_Q9SR86	0.0	Normal: 0	18	0	0	254	0	BlastAnno: 1234	UniRef50_Q9SR86	481	79.37	349	72	0	1	1047	133	481	0.0	595
ABCDEF_3774	UniRef50_Q9SR86	0.0	Normal: 546	163	404	867	677	914	BlastAnno: 1887	UniRef50_Q9SR86	481	71.46	480	124	2	1779	379	2	481	0.0	712
ABCDEF_11684	UniRef50_Q9SR86	0.0	Normal: 963	439	718	954	1382	1798	BlastAnno: 1883	UniRef50_Q9SR86	481	62.36	441	166	0	1649	327	41	481	0.0	592
ABCDEF_3775	UniRef50_Q9SR86	0.0	Normal: 473	159	414	817	611	782	BlastAnno: 1801	UniRef50_Q9SR86	481	71.46	480	124	2	1693	293	2	481	0.0	712
2.6.1.45(serine---glyoxylate transaminase)																					
ABCDEF_9789	UniRef50_Q56YA5	0.0	Normal: 130	3261	66	26	15	8034	BlastAnno: 1692	UniRef50_Q56YA5	401	85.29	333	49	0	352	1350	69	401	0.0	583
ABCDEF_9785	UniRef50_Q56YA5	0.0	Normal: 191	4834	126	51	21	10532	BlastAnno: 1764	UniRef50_Q56YA5	401	85.79	401	57	0	1614	412	1	401	0.0	712
2.6.1.51(serine---pyruvate transaminase;)																					
No Correspond Unigene																					
2.6.1.52(phosphoserine transaminase;)																					
ABCDEF_41302	UniRef50_Q96255	0.0	Normal: 1671	665	2009	2718	2176	624	BlastAnno: 1822	UniRef50_Q96255	430	80.05	391	73	3	1572	406	43	430	0.0	653
ABCDEF_28393	UniRef50_Q96255	0.0	Normal: 390	227	489	822	2225	158	BlastAnno: 1446	UniRef50_Q96255	430	78.83	392	79	2	5	1180	42	429	0.0	627
2.6.1.76(diaminobutyrate---2-oxoglutarate transaminase;)																					
No Correspond Unigene																					
2.7.1.31(glycerate kinase;)																					
ABCDEF_10301	UniRef50_Q944I4	0.0	Normal: 259	552	306	527	545	1533	BlastAnno: 1595	UniRef50_Q944I4	456	72.85	372	99	1	272	1387	86	455	0.0	580
ABCDEF_10303	UniRef50_Q944I4	0.0	Normal: 266	510	294	565	522	1374	BlastAnno: 1563	UniRef50_Q944I4	456	68.43	396	99	2	1292	105	86	455	0.0	565
2.7.1.39(homoserine kinase;)																					
ABCDEF_48230	UniRef50_Q11YZ8	2e-49	Normal: 290	189	319	356	361	292	BlastAnno: 1489	UniRef50_Q11YZ8	309	42.96	291	158	5	1229	360	4	287	2e-49	179
2.7.2.4(aspartate kinase;)																					
ABCDEF_20311	UniRef50_B9F9Y5	0.0	Normal: 345	128	296	177	197	425	BlastAnno: 2207	UniRef50_B9F9Y5	659	87.77	368	43	1	851	1954	269	634	0.0	667
ABCDEF_31297	UniRef50_O81852	0.0	Normal: 3828	2128	2645	751	1881	2287	BlastAnno: 3215	UniRef50_O81852	916	77.65	841	185	2	2790	271	78	916	0.0	1355
ABCDEF_20747	UniRef50_B9F9Y5	0.0	Normal: 586	335	621	488	462	628	BlastAnno: 2180	UniRef50_B9F9Y5	659	79.23	390	76	1	817	1971	269	658	0.0	627
ABCDEF_30670	UniRef50_O81852	0.0	Normal: 1382	736	1142	1007	1095	1298	BlastAnno: 3147	UniRef50_O81852	916	79.52	840	170	1	355	2874	79	916	0.0	1387
ABCDEF_20746	UniRef50_Q9LYU8	0.0	Normal: 399	257	409	338	357	522	BlastAnno: 2071	UniRef50_Q9LYU8	569	78.12	288	63	0	1002	1865	277	564	0.0	459
2.7.3.5(lombricine kinase)																					

No Correspond Unigene																					
2.7.8.8(CDP-diacylglycerol---serine O-phosphatidyltransferase;)																					
ABCDEF_69642	UniRef50_Q4QFL4	9e-30	Normal: 18	6	18	12	12	22	BlastAnno: 395	UniRef50_Q4QFL4	532	44.83	116	64	0	47	394	227	342	9e-30	118
3.1.3.3(phosphoserine phosphatase)																					
ABCDEF_73777	UniRef50_O82796	8e-138	Normal: 234	113	241	451	692	447	BlastAnno: 1098	UniRef50_O82796	295	67.71	288	93	0	95	958	8	295	8e-138	403
ABCDEF_15135	UniRef50_O82796	8e-138	Normal: 265	126	260	482	787	488	BlastAnno: 1173	UniRef50_O82796	295	66.44	295	99	0	136	1020	1	295	8e-138	404
3.1.3.38(3-phosphoglycerate phosphatase;)																					
No Correspond Unigene																					
3.5.3.3(creatinase)																					
No Correspond Unigene																					
4.1.1.86(diaminobutyrate decarboxylase;)																					
No Correspond Unigene																					
4.1.2.5(threonine aldolase;)																					
ABCDEF_67659	UniRef50_A0LG45	9e-116	Normal: 132	114	110	69	146	476	BlastAnno: 1158	UniRef50_A0LG45	353	52.33	344	160	3	83	1114	4	343	9e-116	350
4.2.1.108(ectoine synthase;)																					
No Correspond Unigene																					
4.2.1.20(tryptophan synthase;)																					
ABCDEF_49386	UniRef50_P25269	0.0	Normal: 1090	905	1057	1677	1610	1777	BlastAnno: 1723	UniRef50_P25269	475	81.07	486	62	5	41	1444	2	475	0.0	775
ABCDEF_4210	UniRef50_P25269	0.0	Normal: 5	209	3	0	0	1341	BlastAnno: 1670	UniRef50_P25269	475	62.71	421	150	3	205	1458	56	472	0.0	552
4.2.1.22(cystathionine beta-synthase;)																					
No Correspond Unigene																					
4.2.3.1(threonine synthase;)																					
ABCDEF_45313	UniRef50_Q9S7B5	0.0	Normal: 1544	576	1733	1123	1215	608	BlastAnno: 1882	UniRef50_Q9S7B5	526	86.14	469	61	2	1744	344	46	512	0.0	841
4.3.1.17(L-serine ammonia-lyase;)																					
ABCDEF_41935	UniRef50_Q54HH2	1e-91	Normal: 44	51	30	30	14	119	BlastAnno: 1061	UniRef50_Q54HH2	324	47.90	309	156	2	138	1058	12	317	1e-91	286
4.3.1.18(D-serine ammonia-lyase;)																					
No Correspond Unigene																					
4.3.1.19(threonine ammonia-lyase;)																					
ABCDEF_1551	UniRef50_Q9ZSS6	0.0	Normal: 2	241	47	0	1	1450	BlastAnno: 2188	UniRef50_Q9ZSS6	592	69.90	505	147	3	1844	333	92	592	0.0	699
ABCDEF_10612	UniRef50_Q9ZSS6	0.0	Normal: 2720	1014	2126	1813	2334	1761	BlastAnno: 2348	UniRef50_Q9ZSS6	592	68.70	591	166	5	2148	427	4	592	0.0	763
ABCDEF_84650	UniRef50_Q9ZSS6	0.0	Normal: 2	100	18	0	0	529	BlastAnno: 1546	UniRef50_Q9ZSS6	592	73.05	371	99	1	1202	90	92	461	0.0	533
4.4.1.1(cystathionine gamma-lyase;)																					
ABCDEF_72071	UniRef50_Q55DV9	1e-80	Normal: 173	154	160	241	209	225	BlastAnno: 1568	UniRef50_Q55DV9	387	47.49	259	135	1	662	1435	126	384	1e-80	260
ABCDEF_72428	UniRef50_Q55DV9	1e-80	Normal: 135	117	119	172	170	180	BlastAnno: 1527	UniRef50_Q55DV9	387	47.49	259	135	1	621	1394	126	384	1e-80	260
5.1.1.10(amino-acid racemase;)																					
No Correspond Unigene																					
5.1.1.18(serine racemase;)																					
No Correspond Unigene																					
>00270 Cysteine and methionine metabolism																					
1.1.1.27(L-lactate dehydrogenase;)																					
ABCDEF_38409	UniRef50_P22988	5e-157	Normal: 468	369	408	758	1349	382	BlastAnno: 1380	UniRef50_P22988	356	66.67	357	111	3	121	1170	1	356	5e-157	458
1.1.1.272(No Name)																					
No Correspond Unigene																					
1.1.1.3(homoserine dehydrogenase;)																					
ABCDEF_30670	UniRef50_C6W787	0.0	Normal: 1382	736	1142	1007	1095	1298	BlastAnno: 3147	UniRef50_C6W787	818	40.10	828	481	7	391	2865	3	818	0.0	625
ABCDEF_31297	UniRef50_C6W787	0.0	Normal: 3828	2128	2645	751	1881	2287	BlastAnno: 3215	UniRef50_C6W787	818	41.06	828	472	8	2751	280	3	818	0.0	611
1.13.11.20(cysteine dioxygenase;)																					
No Correspond Unigene																					
1.13.11.53(acireductone dioxygenase (Ni2+-requiring);)																					
No Correspond Unigene																					
1.13.11.54(acireductone dioxygenase [iron(II)-requiring];)																					
ABCDEF_32200	UniRef50_Q8W108	7e-123	Normal: 754	1188	901	1369	2520	1683	BlastAnno: 977	UniRef50_Q8W108	199	85.49	193	28	0	871	293	1	193	7e-123	360
1.14.17.4(aminocyclopropanecarboxylate oxidase;)																					
ABCDEF_49220	UniRef50_Q06588	9e-170	Normal: 3	25	3	1303	877	134	BlastAnno: 1224	UniRef50_Q06588	323	69.97	323	92	3	59	1012	1	323	9e-170	487
1.2.1.11(aspartate-semialdehyde dehydrogenase;)																					
ABCDEF_45337	UniRef50_A4YV18	9e-115	Normal: 811	241	805	357	570	636	BlastAnno: 1520	UniRef50_A4YV18	341	54.90	337	145	3	230	1237	5	335	9e-115	351
1.4.1.-(Oxidoreductases;)																					
No Correspond Unigene																					
1.4.3.2(L-amino-acid oxidase;)																					
ABCDEF_34035	UniRef50_B9R844	0.0	Normal: 973	600	948	1073	764	972	BlastAnno: 3436	UniRef50_B9R844	1947	69.56	611	156	9	1050	2867	1361	1946	0.0	707
1.8.1.10(CoA-glutathione reductase;)																					
No Correspond Unigene																					
1.8.1.6(cystine reductase;)																					
No Correspond Unigene																					
1.8.4.1(glutathione---homocystine transhydrogenase)																					
No Correspond Unigene																					
1.8.4.13(L-methionine (S)-S-oxide reductase;)																					
No Correspond Unigene																					
1.8.4.14(L-methionine (R)-S-oxide reductase;)																					
No Correspond Unigene																					
1.8.4.3(glutathione---CoA-glutathione transhydrogenase;)																					
No Correspond Unigene																					

1.8.4.4(glutathione---cystine transhydrogenase;)																					
No Correspond Unigene																					
2.1.1.10(homocysteine S-methyltransferase;)																					
ABCDEF_34237	UniRef50_Q8LAX0	4e-157	Normal: 257	150	294	279	292	278	BlastAnno: 1451	UniRef50_Q8LAX0	347	66.16	331	107	2	1310	330	12	341	4e-157	459
2.1.1.13(methionine synthase;)																					
No Correspond Unigene																					
2.1.1.14(5-methyltetrahydropteroyltriglutamate---homocysteine S-methyltransferase;)																					
ABCDEF_50765	UniRef50_O50008	0.0	Normal: 1445	1155	1542	2724	6925	2394	BlastAnno: 1074	UniRef50_O50008	765	92.61	284	21	0	1074	223	482	765	0.0	570
ABCDEF_50769	UniRef50_Q0S6X7	0.0	Normal: 1467	2688	2079	4226	6119	4451	BlastAnno: 2720	UniRef50_Q0S6X7	753	49.35	764	361	11	150	2420	9	753	0.0	724
ABCDEF_14591	UniRef50_O50008	0.0	Normal: 175	108	133	170	218	262	BlastAnno: 1240	UniRef50_O50008	765	80.39	413	81	0	2	1240	107	519	0.0	656
ABCDEF_50764	UniRef50_O50008	0.0	Normal: 2132	1684	1899	4094	10442	2856	BlastAnno: 1414	UniRef50_O50008	765	89.81	471	48	0	2	1414	106	576	0.0	858
ABCDEF_50758	UniRef50_Q0S6X7	0.0	Normal: 1269	1809	1557	3252	5371	3330	BlastAnno: 2581	UniRef50_Q0S6X7	753	49.48	764	360	11	100	2370	9	753	0.0	725
ABCDEF_50755	UniRef50_Q0S6X7	0.0	Normal: 1913	2156	2123	4237	9070	3515	BlastAnno: 2561	UniRef50_Q0S6X7	753	48.65	779	344	12	2451	181	9	753	0.0	736
ABCDEF_50754	UniRef50_Q0S6X7	0.0	Normal: 2088	3031	2671	5517	9518	4461	BlastAnno: 2646	UniRef50_Q0S6X7	753	48.65	779	344	12	2543	273	9	753	0.0	736
ABCDEF_50768	UniRef50_Q0S6X7	0.0	Normal: 1344	2423	1879	3794	5578	4174	BlastAnno: 2717	UniRef50_Q0S6X7	753	49.35	764	361	11	2557	287	9	753	0.0	724
ABCDEF_50766	UniRef50_Q0S6X7	0.0	Normal: 1494	2528	1939	4050	6543	4297	BlastAnno: 2694	UniRef50_Q0S6X7	753	49.48	764	360	11	150	2420	9	753	0.0	726
ABCDEF_50753	UniRef50_Q0S6X7	0.0	Normal: 1343	2204	1709	3527	5861	3896	BlastAnno: 2733	UniRef50_Q0S6X7	753	49.48	764	360	11	189	2459	9	753	0.0	725
ABCDEF_50756	UniRef50_Q0S6X7	0.0	Normal: 2120	3117	2695	5653	9766	4628	BlastAnno: 2655	UniRef50_Q0S6X7	753	48.65	779	344	12	2545	275	9	753	0.0	737
ABCDEF_57202	UniRef50_O50008	0.0	Normal: 1505	1097	1164	2664	7110	2018	BlastAnno: 1257	UniRef50_O50008	765	87.50	360	45	0	1081	2	1	360	0.0	637
ABCDEF_50760	UniRef50_Q0S6X7	0.0	Normal: 1514	2280	1931	3777	6908	3592	BlastAnno: 2637	UniRef50_Q0S6X7	753	48.65	779	344	12	81	2351	9	753	0.0	736
ABCDEF_50761	UniRef50_B6J6H8	0.0	Normal: 1122	1642	1538	3134	5532	2936	BlastAnno: 2009	UniRef50_B6J6H8	775	51.84	571	267	5	2006	300	195	759	0.0	572
ABCDEF_50767	UniRef50_O50008	0.0	Normal: 786	1169	903	2117	3654	1574	BlastAnno: 1852	UniRef50_O50008	765	88.56	568	65	0	149	1852	1	568	0.0	1018
ABCDEF_50759	UniRef50_Q0S6X7	0.0	Normal: 1559	2011	1766	3739	7427	3210	BlastAnno: 2518	UniRef50_Q0S6X7	753	48.65	779	344	12	81	2351	9	753	0.0	736
2.1.1.37(DNA (cytosine-5-)-methyltransferase;)																					
ABCDEF_17200	UniRef50_Q9M548	0.0	Normal: 526	260	477	474	582	432	BlastAnno: 2288	UniRef50_Q9M548	626	55.88	544	204	8	452	2071	112	623	0.0	569
ABCDEF_20889	UniRef50_B9GLJ3	0.0	Normal: 246	84	123	170	130	101	BlastAnno: 1828	UniRef50_B9GLJ3	973	55.26	532	219	7	284	1828	185	714	0.0	580
ABCDEF_58832	UniRef50_B8AZD1	0.0	Normal: 201	60	184	163	120	170	BlastAnno: 2660	UniRef50_B8AZD1	1325	53.89	501	202	6	194	1633	606	1098	0.0	494
ABCDEF_37098	UniRef50_P34881	0.0	Normal: 3088	389	1567	1012	929	688	BlastAnno: 5142	UniRef50_P34881	1534	59.94	1540	585	16	412	4983	6	1529	0.0	1786
ABCDEF_17201	UniRef50_Q9M548	0.0	Normal: 515	259	465	473	568	437	BlastAnno: 2341	UniRef50_Q9M548	626	55.88	544	204	8	540	2159	112	623	0.0	568
ABCDEF_30139	UniRef50_O49139	0.0	Normal: 3906	318	1547	49	348	1061	BlastAnno: 3017	UniRef50_O49139	791	49.17	785	373	9	2590	281	14	787	0.0	688
ABCDEF_37187	UniRef50_B8AZD1	0.0	Normal: 212	56	189	161	121	178	BlastAnno: 2583	UniRef50_B8AZD1	1325	54.72	720	296	7	194	2287	606	1317	0.0	740
ABCDEF_37190	UniRef50_B8AZD1	0.0	Normal: 358	103	295	316	179	261	BlastAnno: 3754	UniRef50_B8AZD1	1325	50.00	578	244	11	2683	1028	540	1098	0.0	515
ABCDEF_37191	UniRef50_B8AZD1	0.0	Normal: 356	100	302	330	187	270	BlastAnno: 3692	UniRef50_B8AZD1	1325	51.82	797	338	12	2606	297	540	1317	0.0	761
ABCDEF_37189	UniRef50_B8AZD1	0.0	Normal: 320	102	260	290	167	240	BlastAnno: 3800	UniRef50_B8AZD1	1325	53.89	501	202	6	1334	2773	606	1098	0.0	495
ABCDEF_37188	UniRef50_B8AZD1	0.0	Normal: 358	100	303	326	181	256	BlastAnno: 3723	UniRef50_B8AZD1	1325	54.72	720	296	7	2390	297	606	1317	0.0	741
2.1.1.5(betaine---homocysteine S-methyltransferase;)																					
No Correspond Unigene																					
2.3.1.30(serine O-acetyltransferase;)																					
ABCDEF_31726	UniRef50_B9S9Q4	3e-161	Normal: 94	985	55	43	120	1929	BlastAnno: 1549	UniRef50_B9S9Q4	396	63.93	377	104	6	1287	250	19	394	3e-161	473
2.3.1.31(homoserine O-acetyltransferase;)																					
No Correspond Unigene																					
2.3.1.46(homoserine O-succinyltransferase;)																					
No Correspond Unigene																					
2.4.2.28(S-methyl-5'-thioadenosine phosphorylase;)																					
ABCDEF_80728	UniRef50_Q13126	7e-27	Normal: 0	0	0	0	0	22	BlastAnno: 266	UniRef50_Q13126	283	61.73	81	30	1	245	3	168	247	7e-27	106
2.5.1.-(Transferases;)																					
ABCDEF_48175	UniRef50_P72580	2e-123	Normal: 366	927	282	531	590	1811	BlastAnno: 1917	UniRef50_P72580	323	58.44	320	130	2	610	1569	6	322	2e-123	377
2.5.1.16(spermidine synthase;)																					
ABCDEF_7812	UniRef50_Q9ZUB3	0.0	Normal: 1661	1302	2150	4493	6957	4671	BlastAnno: 1757	UniRef50_Q9ZUB3	334	81.44	334	49	3	1326	331	11	333	0.0	552
2.5.1.22(spermine synthase;)																					
No Correspond Unigene																					
2.5.1.47(cysteine synthase;)																					
ABCDEF_4276	UniRef50_P47999	0.0	Normal: 991	1556	999	1021	1482	4746	BlastAnno: 1414	UniRef50_P47999	392	77.08	349	76	1	146	1192	47	391	0.0	533
2.5.1.48(cystathionine gamma-synthase;)																					
ABCDEF_31946	UniRef50_P55217	0.0	Normal: 3795	4731	4027	7276	7940	8571	BlastAnno: 1625	UniRef50_P55217	563	84.28	407	60	1	1399	179	161	563	0.0	719
2.5.1.49(O-acetylhomoserine aminocarboxypropyltransferase;)																					
No Correspond Unigene																					
2.5.1.6(methionine adenosyltransferase;)																					
ABCDEF_38537	UniRef50_Q00266	2e-158	Normal: 2154	3715	1965	1163	2194	2892	BlastAnno: 1653	UniRef50_Q00266	395	63.45	383	133	3	189	1337	18	393	2e-158	466
2.5.1.65(O-phosphoserine sulfhydrylase;)																					
No Correspond Unigene																					
2.6.1.-(Transferases;)																					
ABCDEF_3716	UniRef50_Q9LQ10	0.0	Normal: 224	353	140	47	76	606	BlastAnno: 2230	UniRef50_Q9LQ10	557	61.35	533	189	8	250	1812	30	557	0.0	609
ABCDEF_37756	UniRef50_Q9LQ10	0.0	Normal: 197	168	149	152	186	490	BlastAnno: 1840	UniRef50_Q9LQ10	557	57.49	534	196	7	1638	109	28	554	0.0	619
2.6.1.1(aspartate transaminase;)																					
ABCDEF_11690	UniRef50_P46248	0.0	Normal: 1745	1815	2022	4078	5299	6095	BlastAnno: 1778	UniRef50_P46248	453	86.09	417	58	0	297	1547	37	453	0.0	733
ABCDEF_1333	UniRef50_C0PSV4	0.0	Normal: 356	544	636	1119	2495	631	BlastAnno: 1594	UniRef50_C0PSV4	462	76.43	403	95	0	1401	193	60	462	0.0	662
ABCDEF_21786	UniRef50_C0PSV4	0.0	Normal: 268	668	330	546	1267	646	BlastAnno: 1736	UniRef50_C0PSV4	462	74.14	464	113	3	50	1441	4	460	0.0	711
ABCDEF_42262	UniRef50_P46643	0.0	Normal: 916	606	1656	2699	2553	856	BlastAnno: 1722	UniRef50_P46643	430	79.49	429	85	2	1635	355	1	428	0.0	731
ABCDEF_56929	UniRef50_P37833	0.0	Normal: 173	278	312	654	1243	297	BlastAnno: 1151	UniRef50_P37833	407	81.82	308	56	0	978	55	3	310	0.0	536
2.6.1.3(cysteine transaminase;)																					
No Correspond Unigene																					

2.6.1.5(tyrosine transaminase;)																						
	ABCDEF_52650	UniRef50_Q9LVY1	0.0	Normal: 493	358	455	302	300	892	BlastAnno: 1442	UniRef50_Q9LVY1	420	61.26	413	160	0	65	1303	5	417	0.0	539
2.6.1.57(aromatic-amino-acid transaminase;)																						
	No Correspond Unigene																					
2.7.1.100(S-methyl-5-thioribose kinase;)																						
	ABCDEF_7722	UniRef50_A5EK80	7e-128	Normal: 769	867	891	919	1514	1562	BlastAnno: 1611	UniRef50_A5EK80	433	46.56	421	213	5	1483	233	13	425	7e-128	389
2.7.2.4(aspartate kinase;)																						
	ABCDEF_20311	UniRef50_B9F9Y5	0.0	Normal: 345	128	296	177	197	425	BlastAnno: 2207	UniRef50_B9F9Y5	659	87.77	368	43	1	851	1954	269	634	0.0	667
	ABCDEF_31297	UniRef50_O81852	0.0	Normal: 3828	2128	2645	751	1881	2287	BlastAnno: 3215	UniRef50_O81852	916	77.65	841	185	2	2790	271	78	916	0.0	1355
	ABCDEF_20747	UniRef50_B9F9Y5	0.0	Normal: 586	335	621	488	462	628	BlastAnno: 2180	UniRef50_B9F9Y5	659	79.23	390	76	1	817	1971	269	658	0.0	627
	ABCDEF_30670	UniRef50_O81852	0.0	Normal: 1382	736	1142	1007	1095	1298	BlastAnno: 3147	UniRef50_O81852	916	79.52	840	170	1	355	2874	79	916	0.0	1387
	ABCDEF_20746	UniRef50_Q9LYU8	0.0	Normal: 399	257	409	338	357	522	BlastAnno: 2071	UniRef50_Q9LYU8	569	78.12	288	63	0	1002	1865	277	564	0.0	459
2.8.1.2(3-mercaptopyruvate sulfurtransferase;)																						
	No Correspond Unigene																					
3.1.3.77(acireductone synthase;)																						
	ABCDEF_1108	UniRef50_Q6GMI7	7e-60	Normal: 1834	2463	2220	3344	4280	6662	BlastAnno: 2085	UniRef50_Q6GMI7	261	44.98	249	132	3	933	1667	5	252	7e-60	209
3.2.2.16(methylthioadenosine nucleosidase;)																						
	ABCDEF_28529	UniRef50_C3JAU7	6e-08	Normal: 258	61	271	218	107	172	BlastAnno: 872	UniRef50_C3JAU7	242	28.05	221	130	9	774	157	4	210	6e-08	58.2
3.2.2.9(adenosylhomocysteine nucleosidase;)																						
	ABCDEF_28529	UniRef50_Q7XA67	1e-115	Normal: 258	61	271	218	107	172	BlastAnno: 872	UniRef50_Q7XA67	254	71.14	246	71	0	777	40	9	254	1e-115	342
3.3.1.1(adenosylhomocysteinase;)																						
	ABCDEF_52707	UniRef50_C5C9B9	0.0	Normal: 16010	17748	20191	40391	68577	22007	BlastAnno: 1984	UniRef50_C5C9B9	519	62.10	496	160	7	115	1533	29	519	0.0	563
3.4.13.12(Met-Xaa dipeptidase;)																						
	No Correspond Unigene																					
3.5.1.31(formylmethionine deformylase)																						
	ABCDEF_3670	UniRef50_Q8I372	9e-44	Normal: 111	170	161	150	181	472	BlastAnno: 1063	UniRef50_Q8I372	241	47.02	168	88	1	279	779	65	232	9e-44	159
4.1.1.12(aspartate 4-decarboxylase;)																						
	No Correspond Unigene																					
4.1.1.50(adenosylmethionine decarboxylase;)																						
	ABCDEF_11694	UniRef50_Q9S7T9	3e-156	Normal: 1259	2802	2513	10565	16423	4466	BlastAnno: 1898	UniRef50_Q9S7T9	362	62.98	362	130	3	610	1683	1	362	3e-156	462
4.2.1.109(methylthioribulose 1-phosphate dehydratase;)																						
	ABCDEF_1108	UniRef50_Q9FN41	0.0	Normal: 1834	2463	2220	3344	4280	6662	BlastAnno: 2085	UniRef50_Q9FN41	507	74.56	515	120	2	132	1676	4	507	0.0	783
4.2.1.22(cystathionine beta-synthase;)																						
	No Correspond Unigene																					
4.3.1.17(L-serine ammonia-lyase;)																						
	ABCDEF_41935	UniRef50_Q54HH2	1e-91	Normal: 44	51	30	30	14	119	BlastAnno: 1061	UniRef50_Q54HH2	324	47.90	309	156	2	138	1058	12	317	1e-91	286
4.4.1.1(cystathionine gamma-lyase;)																						
	ABCDEF_72071	UniRef50_Q55DV9	1e-80	Normal: 173	154	160	241	209	225	BlastAnno: 1568	UniRef50_Q55DV9	387	47.49	259	135	1	662	1435	126	384	1e-80	260
	ABCDEF_72428	UniRef50_Q55DV9	1e-80	Normal: 135	117	119	172	170	180	BlastAnno: 1527	UniRef50_Q55DV9	387	47.49	259	135	1	621	1394	126	384	1e-80	260
4.4.1.10(cysteine lyase;)																						
	No Correspond Unigene																					
4.4.1.11(methionine gamma-lyase;)																						
	ABCDEF_31946	UniRef50_C0Z892	4e-92	Normal: 3795	4731	4027	7276	7940	8571	BlastAnno: 1625	UniRef50_C0Z892	402	44.84	339	185	2	1192	179	57	394	4e-92	296
4.4.1.14(1-aminocyclopropane-1-carboxylate synthase;)																						
	ABCDEF_3961	UniRef50_P31531	2e-166	Normal: 37	107	23	25	74	290	BlastAnno: 1106	UniRef50_P31531	484	70.66	317	92	1	155	1105	1	316	2e-166	483
4.4.1.15(D-cysteine desulfhydrase;)																						
	No Correspond Unigene																					
4.4.1.21(S-ribosylhomocysteine lyase;)																						
	No Correspond Unigene																					
4.4.1.24(sulfolactate sulfo-lyase;)																						
	No Correspond Unigene																					
4.4.1.25(L-cysteate sulfo-lyase;)																						
	No Correspond Unigene																					
4.4.1.8(cystathionine beta-lyase;)																						
	ABCDEF_71418	UniRef50_P53780	0.0	Normal: 160	139	136	188	195	199	BlastAnno: 1556	UniRef50_P53780	464	72.22	450	117	2	86	1435	22	463	0.0	637
	ABCDEF_40739	UniRef50_P53780	0.0	Normal: 202	178	179	256	234	247	BlastAnno: 1653	UniRef50_P53780	464	71.24	459	124	2	130	1506	13	463	0.0	644
5.1.1.10(amino-acid racemase;)																						
	No Correspond Unigene																					
5.3.1.23(S-methyl-5-thioribose-1-phosphate isomerase;)																						
	ABCDEF_27430	UniRef50_Q9BV20	5e-101	Normal: 147	111	181	294	426	159	BlastAnno: 1516	UniRef50_Q9BV20	369	54.86	350	151	5	1345	305	2	347	5e-101	317
>00280 Valine, leucine and isoleucine degradation																						
1.1.1.178(3-hydroxy-2-methylbutyryl-CoA dehydrogenase;)																						
	No Correspond Unigene																					
1.1.1.31(3-hydroxyisobutyrate dehydrogenase;)																						
	ABCDEF_3924	UniRef50_Q9SUC0	5e-156	Normal: 293	90	269	403	395	398	BlastAnno: 1399	UniRef50_Q9SUC0	347	66.38	345	115	1	131	1165	3	346	5e-156	455
1.1.1.35(3-hydroxyacyl-CoA dehydrogenase;)																						
	ABCDEF_80649	UniRef50_Q1LD07	5e-120	Normal: 527	841	443	1308	1321	1188	BlastAnno: 2586	UniRef50_Q1LD07	715	37.34	699	420	9	2358	277	22	707	5e-120	389
1.2.1.27(methylmalonate-semialdehyde dehydrogenase (acylating);)																						
	ABCDEF_18282	UniRef50_B7GBU6	0.0	Normal: 1599	1252	1838	3819	3718	2949	BlastAnno: 2054	UniRef50_B7GBU6	537	59.35	492	191	2	402	1865	50	536	0.0	571
1.2.1.3(aldehyde dehydrogenase (NAD+);)																						
	ABCDEF_52483	UniRef50_Q70E96	0.0	Normal: 28	851	41	0	3965	420	BlastAnno: 1750	UniRef50_Q70E96	484	54.56	482	219	0	107	1552	3	484	0.0	548
	ABCDEF_17638	UniRef50_Q70E96	0.0	Normal: 580	718	749	2424	1317	775	BlastAnno: 1970	UniRef50_Q70E96	484	62.21	471	177	1	283	1692	2	472	0.0	625

ABCDEF_403	UniRef50_Q8W033	0.0	Normal: 69	355	98	39	223	1111	BlastAnno: 1749	UniRef50_Q8W033	550	65.96	473	161	0	1523	105	74	546	0.0	625
ABCDEF_45143	UniRef50_Q0WSF1	0.0	Normal: 1445	874	1200	1789	1940	1504	BlastAnno: 2450	UniRef50_Q0WSF1	596	79.97	594	115	2	2220	451	1	594	0.0	1015
1.2.3.1(aldehyde oxidase;)																					
ABCDEF_37559	UniRef50_Q7G193	0.0	Normal: 208	649	188	236	120	436	BlastAnno: 2825	UniRef50_Q7G193	1368	58.36	927	352	11	2741	3	3	909	0.0	1063
ABCDEF_51652	UniRef50_Q7G193	0.0	Normal: 97	323	130	90	67	347	BlastAnno: 1575	UniRef50_Q7G193	1368	64.15	491	172	4	5	1468	871	1360	0.0	632
1.2.4.4(3-methyl-2-oxobutanoate dehydrogenase (2-methylpropanoyl-transferring);)																					
ABCDEF_38447	UniRef50_Q55FN7	1e-150	Normal: 373	434	298	528	686	1281	BlastAnno: 1558	UniRef50_Q55FN7	370	67.69	325	104	1	1251	280	46	370	1e-150	444
1.2.7.7(3-methyl-2-oxobutanoate dehydrogenase (ferredoxin);)																					
No Correspond Unigene																					
1.3.99.-(Oxidoreductases;)																					
ABCDEF_4448	UniRef50_Q6JQN1	0.0	Normal: 1778	1683	1752	2720	2409	2803	BlastAnno: 2780	UniRef50_Q6JQN1	1059	48.13	802	354	14	231	2564	293	1056	0.0	718
1.3.99.10(isovaleryl-CoA dehydrogenase;)																					
ABCDEF_11035	UniRef50_P26440	8e-175	Normal: 334	360	401	732	758	655	BlastAnno: 1628	UniRef50_P26440	423	65.87	375	126	1	1372	254	42	416	8e-175	509
1.3.99.12(2-methylacyl-CoA dehydrogenase;)																					
No Correspond Unigene																					
1.3.99.2(butyryl-CoA dehydrogenase;)																					
ABCDEF_90276	UniRef50_Q0AYU5	1e-61	Normal: 0	0	0	0	0	80	BlastAnno: 757	UniRef50_Q0AYU5	380	45.63	252	126	5	3	755	44	285	1e-61	206
1.3.99.3(acyl-CoA dehydrogenase;)																					
ABCDEF_90276	UniRef50_P11310	4e-115	Normal: 0	0	0	0	0	80	BlastAnno: 757	UniRef50_P11310	421	66.14	251	79	2	3	755	80	324	4e-115	345
1.4.1.9(leucine dehydrogenase;)																					
No Correspond Unigene																					
1.4.3.2(L-amino-acid oxidase;)																					
ABCDEF_34035	UniRef50_B9R844	0.0	Normal: 973	600	948	1073	764	972	BlastAnno: 3436	UniRef50_B9R844	1947	69.56	611	156	9	1050	2867	1361	1946	0.0	707
1.8.1.4(dihydrolipoyl dehydrogenase;)																					
ABCDEF_573	UniRef50_P72740	0.0	Normal: 1422	809	1248	2548	1822	4200	BlastAnno: 2189	UniRef50_P72740	474	61.95	473	173	5	469	1878	5	473	0.0	556
2.3.1.16(acetyl-CoA C-acyltransferase;)																					
ABCDEF_47228	UniRef50_P09110	1e-153	Normal: 321	497	306	408	1144	369	BlastAnno: 1559	UniRef50_P09110	424	59.95	387	152	3	1397	237	37	420	1e-153	454
2.3.1.168(dihydrolipoyllysine-residue (2-methylpropanoyl)transferase;)																					
ABCDEF_45084	UniRef50_P11182	2e-106	Normal: 365	280	333	559	768	703	BlastAnno: 1972	UniRef50_P11182	482	41.30	431	237	4	1528	254	62	482	2e-106	339
2.3.1.9(acetyl-CoA C-acetyltransferase;)																					
ABCDEF_34825	UniRef50_Q8S4Y1	0.0	Normal: 640	698	620	807	839	1164	BlastAnno: 1773	UniRef50_Q8S4Y1	403	78.11	402	87	1	1597	395	2	403	0.0	598
ABCDEF_14629	UniRef50_Q8S4Y1	0.0	Normal: 634	316	650	1021	1278	691	BlastAnno: 1514	UniRef50_Q8S4Y1	403	85.11	403	60	0	1367	159	1	403	0.0	620
ABCDEF_107	UniRef50_Q8S4Y1	0.0	Normal: 332	460	460	842	893	424	BlastAnno: 1214	UniRef50_Q8S4Y1	403	84.07	339	54	0	1212	196	42	380	0.0	596
ABCDEF_14630	UniRef50_Q8S4Y1	0.0	Normal: 562	282	568	959	1173	627	BlastAnno: 1796	UniRef50_Q8S4Y1	403	85.50	400	58	0	436	1635	4	403	0.0	642
2.3.3.10(hydroxymethylglutaryl-CoA synthase;)																					
ABCDEF_52282	UniRef50_A4GKI1	0.0	Normal: 1662	333	1405	2243	3244	1685	BlastAnno: 1805	UniRef50_A4GKI1	463	57.49	454	189	2	1606	251	2	453	0.0	536
ABCDEF_4202	UniRef50_A4GKI1	0.0	Normal: 1002	1241	821	1323	1992	1786	BlastAnno: 1998	UniRef50_A4GKI1	463	57.74	452	185	3	1636	290	2	450	0.0	531
2.6.1.18(beta-alanine---pyruvate transaminase;)																					
No Correspond Unigene																					
2.6.1.22(No Name)																					
No Correspond Unigene																					
2.6.1.42(branched-chain-amino-acid transaminase;)																					
ABCDEF_45709	UniRef50_Q9FYA6	1e-179	Normal: 1705	822	1358	1129	1148	2398	BlastAnno: 1596	UniRef50_Q9FYA6	415	74.08	355	91	1	1261	197	61	414	1e-179	521
2.6.1.6(leucine transaminase;)																					
No Correspond Unigene																					
2.8.3.5(3-oxoacid CoA-transferase;)																					
No Correspond Unigene																					
3.1.2.4(3-hydroxyisobutyryl-CoA hydrolase;)																					
ABCDEF_56804	UniRef50_Q6NVY1	6e-77	Normal: 160	146	249	472	328	155	BlastAnno: 1439	UniRef50_Q6NVY1	386	40.45	356	199	7	257	1306	29	377	6e-77	254
4.1.3.4(hydroxymethylglutaryl-CoA lyase;)																					
ABCDEF_25184	UniRef50_P35915	3e-119	Normal: 682	2198	692	1164	701	2789	BlastAnno: 1741	UniRef50_P35915	298	63.51	296	108	0	1298	411	3	298	3e-119	363
4.2.1.17(enoyl-CoA hydratase;)																					
ABCDEF_21259	UniRef50_Q8W1L6	0.0	Normal: 272	276	284	384	326	305	BlastAnno: 2641	UniRef50_Q8W1L6	726	47.57	721	375	3	114	2267	6	726	0.0	633
ABCDEF_80649	UniRef50_Q8W1L6	0.0	Normal: 527	841	443	1308	1321	1188	BlastAnno: 2586	UniRef50_Q8W1L6	726	60.39	712	281	1	2403	268	1	711	0.0	818
ABCDEF_466	UniRef50_Q8W1L6	0.0	Normal: 1014	1690	1094	1677	2438	2080	BlastAnno: 2453	UniRef50_Q8W1L6	726	71.24	699	201	0	2368	272	7	705	0.0	954
ABCDEF_54834	UniRef50_Q8W1L6	0.0	Normal: 229	220	237	298	270	261	BlastAnno: 2405	UniRef50_Q8W1L6	726	47.52	606	315	3	274	2082	121	726	0.0	580
ABCDEF_34756	UniRef50_Q8W1L6	0.0	Normal: 776	726	768	2922	4039	1079	BlastAnno: 2469	UniRef50_Q8W1L6	726	68.93	708	217	2	2396	273	7	711	0.0	957
ABCDEF_4198	UniRef50_Q8W1L6	0.0	Normal: 609	929	532	1401	1568	1297	BlastAnno: 2667	UniRef50_Q8W1L6	726	60.45	713	281	1	2463	325	1	712	0.0	820
4.2.1.18(methylglutaconyl-CoA hydratase;)																					
ABCDEF_35398	UniRef50_B9T286	8e-140	Normal: 536	303	623	637	616	787	BlastAnno: 1218	UniRef50_B9T286	325	67.69	325	99	2	82	1038	1	325	8e-140	411
5.1.99.1(methylmalonyl-CoA epimerase;)																					
No Correspond Unigene																					
5.4.3.7(leucine 2,3-aminomutase)																					
No Correspond Unigene																					
5.4.99.2(methylmalonyl-CoA mutase;)																					
No Correspond Unigene																					
6.4.1.3(propionyl-CoA carboxylase;)																					
ABCDEF_21606	UniRef50_Q2S388	2e-150	Normal: 386	375	365	562	565	924	BlastAnno: 2365	UniRef50_Q2S388	698	45.92	527	261	3	251	1768	152	675	2e-150	465
6.4.1.4(methylcrotonoyl-CoA carboxylase;)																					
ABCDEF_21606	UniRef50_Q9V9A7	0.0	Normal: 386	375	365	562	565	924	BlastAnno: 2365	UniRef50_Q9V9A7	578	61.32	574	204	4	164	1834	6	578	0.0	706
ABCDEF_51901	UniRef50_Q42523	0.0	Normal: 505	684	448	760	1253	3043	BlastAnno: 2679	UniRef50_Q42523	734	70.68	730	204	5	104	2278	9	733	0.0	981
ABCDEF_21607	UniRef50_Q9V9A7	0.0	Normal: 227	220	218	317	373	680	BlastAnno: 2236	UniRef50_Q9V9A7	578	65.41	477	151	2	316	1704	102	578	0.0	639

>00281 Geraniol degradation																					
1.1.1.183(geraniol dehydrogenase)																					
ABCDEF_21569	UniRef50_Q2KNL6	0.0	Normal: 2919	622	4593	6934	3099	891	BlastAnno: 1532	UniRef50_Q2KNL6	360	71.23	358	99	2	1460	399	1	358	0.0	522
1.1.1.35(3-hydroxyacyl-CoA dehydrogenase;)																					
ABCDEF_80649	UniRef50_Q1LD07	5e-120	Normal: 527	841	443	1308	1321	1188	BlastAnno: 2586	UniRef50_Q1LD07	715	37.34	699	420	9	2358	277	22	707	5e-120	389
1.2.1.-(Oxidoreductases;)																					
ABCDEF_14573	UniRef50_B5ZBH4	2e-108	Normal: 2547	9518	1266	309	441	22896	BlastAnno: 1872	UniRef50_B5ZBH4	475	42.52	461	259	4	228	1598	16	474	2e-108	343
1.3.99.-(Oxidoreductases;)																					
ABCDEF_4448	UniRef50_Q6JQN1	0.0	Normal: 1778	1683	1752	2720	2409	2803	BlastAnno: 2780	UniRef50_Q6JQN1	1059	48.13	802	354	14	231	2564	293	1056	0.0	718
2.3.1.16(acetyl-CoA C-acyltransferase;)																					
ABCDEF_47228	UniRef50_P09110	1e-153	Normal: 321	497	306	408	1144	369	BlastAnno: 1559	UniRef50_P09110	424	59.95	387	152	3	1397	237	37	420	1e-153	454
2.8.3.-(Transferases;)																					
No Correspond Unigene																					
4.1.3.26(3-hydroxy-3-isohexenylglutaryl-CoA lyase;)																					
No Correspond Unigene																					
4.1.3.4(hydroxymethylglutaryl-CoA lyase;)																					
ABCDEF_25184	UniRef50_P35915	3e-119	Normal: 682	2198	692	1164	701	2789	BlastAnno: 1741	UniRef50_P35915	298	63.51	296	108	0	1298	411	3	298	3e-119	363
4.2.1.17(enoyl-CoA hydratase;)																					
ABCDEF_21259	UniRef50_Q8W1L6	0.0	Normal: 272	276	284	384	326	305	BlastAnno: 2641	UniRef50_Q8W1L6	726	47.57	721	375	3	114	2267	6	726	0.0	633
ABCDEF_80649	UniRef50_Q8W1L6	0.0	Normal: 527	841	443	1308	1321	1188	BlastAnno: 2586	UniRef50_Q8W1L6	726	60.39	712	281	1	2403	268	1	711	0.0	818
ABCDEF_466	UniRef50_Q8W1L6	0.0	Normal: 1014	1690	1094	1677	2438	2080	BlastAnno: 2453	UniRef50_Q8W1L6	726	71.24	699	201	0	2368	272	7	705	0.0	954
ABCDEF_54834	UniRef50_Q8W1L6	0.0	Normal: 229	220	237	298	270	261	BlastAnno: 2405	UniRef50_Q8W1L6	726	47.52	606	315	3	274	2082	121	726	0.0	580
ABCDEF_34756	UniRef50_Q8W1L6	0.0	Normal: 776	726	768	2922	4039	1079	BlastAnno: 2469	UniRef50_Q8W1L6	726	68.93	708	217	2	2396	273	7	711	0.0	957
ABCDEF_4198	UniRef50_Q8W1L6	0.0	Normal: 609	929	532	1401	1568	1297	BlastAnno: 2667	UniRef50_Q8W1L6	726	60.45	713	281	1	2463	325	1	712	0.0	820
4.2.1.57(isohexenylglutaconyl-CoA hydratase;)																					
No Correspond Unigene																					
5.2.1.-(Isomerases;)																					
No Correspond Unigene																					
6.2.1.-(Ligases;)																					
ABCDEF_7378	UniRef50_Q9FFE6	0.0	Normal: 1022	1422	746	576	680	1408	BlastAnno: 2117	UniRef50_Q9FFE6	552	67.57	552	167	5	144	1778	1	547	0.0	768
ABCDEF_47513	UniRef50_Q8RU95	0.0	Normal: 411	609	570	233	749	887	BlastAnno: 2280	UniRef50_Q8RU95	598	53.15	572	246	6	119	1777	28	596	0.0	543
ABCDEF_78742	UniRef50_Q84P25	0.0	Normal: 236	207	373	576	450	306	BlastAnno: 1534	UniRef50_Q84P25	565	60.39	462	168	5	1413	49	19	472	0.0	546
ABCDEF_10753	UniRef50_Q9FFE6	0.0	Normal: 394	401	336	253	292	348	BlastAnno: 1841	UniRef50_Q9FFE6	552	51.00	551	259	5	117	1751	1	546	0.0	553
ABCDEF_4431	UniRef50_Q9FFE6	0.0	Normal: 27	358	28	98	112	914	BlastAnno: 2075	UniRef50_Q9FFE6	552	50.71	560	264	7	1774	107	1	552	0.0	551
ABCDEF_38213	UniRef50_Q84P25	0.0	Normal: 342	337	597	798	722	505	BlastAnno: 1939	UniRef50_Q84P25	565	62.34	555	194	5	1818	175	19	565	0.0	690
ABCDEF_25221	UniRef50_Q0DV32	0.0	Normal: 971	826	1003	1543	1442	1545	BlastAnno: 2009	UniRef50_Q0DV32	552	57.20	542	226	4	183	1793	9	549	0.0	613
ABCDEF_23413	UniRef50_Q84P23	0.0	Normal: 123	237	158	149	213	658	BlastAnno: 3175	UniRef50_Q84P23	562	51.74	547	243	8	3030	1435	15	555	0.0	555
ABCDEF_20486	UniRef50_Q9FFE6	0.0	Normal: 580	912	312	324	864	1565	BlastAnno: 3530	UniRef50_Q9FFE6	552	66.61	557	171	5	352	1995	1	551	0.0	781
6.4.1.5(geranoyl-CoA carboxylase;)																					
No Correspond Unigene																					
>00290 Valine, leucine and isoleucine biosynthesis																					
1.1.1.85(3-isopropylmalate dehydrogenase;)																					
ABCDEF_37402	UniRef50_Q9FMT1	0.0	Normal: 524	257	473	538	670	865	BlastAnno: 1791	UniRef50_Q9FMT1	409	78.37	393	74	3	1423	248	24	406	0.0	639
ABCDEF_37401	UniRef50_Q9FMT1	0.0	Normal: 480	209	453	430	608	915	BlastAnno: 1616	UniRef50_Q9FMT1	409	77.86	393	76	3	1424	249	24	406	0.0	633
ABCDEF_37399	UniRef50_Q9FMT1	0.0	Normal: 484	194	417	449	585	758	BlastAnno: 1783	UniRef50_Q9FMT1	409	74.76	416	94	3	404	1648	1	406	0.0	635
1.1.1.86(ketol-acid reductoisomerase;)																					
ABCDEF_45798	UniRef50_Q05758	0.0	Normal: 8016	5924	6126	3207	6323	14773	BlastAnno: 2251	UniRef50_Q05758	591	90.13	537	49	2	1881	271	59	591	0.0	980
1.2.4.1(pyruvate dehydrogenase (acetyl-transferring);)																					
ABCDEF_28990	UniRef50_Q8H1Y0	0.0	Normal: 569	351	802	1550	2572	992	BlastAnno: 1596	UniRef50_Q8H1Y0	393	80.62	356	69	0	1369	302	38	393	0.0	628
ABCDEF_11397	UniRef50_O24458	0.0	Normal: 1628	1528	1619	3502	3214	2274	BlastAnno: 1766	UniRef50_O24458	406	82.74	394	58	4	1349	180	19	406	0.0	610
ABCDEF_35811	UniRef50_Q8H1Y0	0.0	Normal: 1865	1480	2772	5588	6415	2489	BlastAnno: 1647	UniRef50_Q8H1Y0	393	77.66	394	83	3	138	1307	1	393	0.0	621
ABCDEF_15406	UniRef50_O24458	0.0	Normal: 297	358	305	389	271	1259	BlastAnno: 1486	UniRef50_O24458	406	80.73	410	72	4	19	1239	1	406	0.0	647
ABCDEF_49154	UniRef50_O24458	0.0	Normal: 304	369	317	391	293	1358	BlastAnno: 1412	UniRef50_O24458	406	80.73	410	72	4	1394	174	1	406	0.0	647
ABCDEF_63617	UniRef50_O24458	0.0	Normal: 518	490	543	916	834	1436	BlastAnno: 1398	UniRef50_O24458	406	80.68	409	72	4	1397	180	2	406	0.0	618
1.4.1.9(leucine dehydrogenase;)																					
No Correspond Unigene																					
2.2.1.6(acetolactate synthase;)																					
ABCDEF_41851	UniRef50_B2UM25	0.0	Normal: 2693	2810	2333	1636	1298	4686	BlastAnno: 2340	UniRef50_B2UM25	617	52.19	594	263	7	364	2088	10	601	0.0	597
ABCDEF_6813	UniRef50_B2UM25	0.0	Normal: 935	649	1027	751	1655	773	BlastAnno: 2146	UniRef50_B2UM25	617	51.17	598	270	8	1943	204	10	603	0.0	584
2.3.1.182(No Name)																					
No Correspond Unigene																					
2.3.3.13(2-isopropylmalate synthase;)																					
ABCDEF_4613	UniRef50_Q9C550	0.0	Normal: 1859	1016	1479	1319	1219	3623	BlastAnno: 2304	UniRef50_Q9C550	631	78.77	551	115	1	2004	358	72	622	0.0	921
ABCDEF_7468	UniRef50_Q9C550	0.0	Normal: 1169	166	1015	479	1088	334	BlastAnno: 1883	UniRef50_Q9C550	631	75.86	526	123	2	1881	316	97	622	0.0	836
2.6.1.42(branched-chain-amino-acid transaminase;)																					
ABCDEF_45709	UniRef50_Q9FYA6	1e-179	Normal: 1705	822	1358	1129	1148	2398	BlastAnno: 1596	UniRef50_Q9FYA6	415	74.08	355	91	1	1261	197	61	414	1e-179	521
2.6.1.6(leucine transaminase;)																					
No Correspond Unigene																					
2.6.1.66(valine---pyruvate transaminase;)																					
No Correspond Unigene																					
4.2.1.33(3-isopropylmalate dehydratase;)																					
ABCDEF_45739	UniRef50_Q94AR8	0.0	Normal: 1223	1188	1256	1790	2276	3678	BlastAnno: 2076	UniRef50_Q94AR8	509	82.49	497	84	3	301	1788	15	509	0.0	866

ABCDEF_58470	UniRef50_Q94AR8	0.0	Normal: 1319	1008	968	1463	1657	2483	BlastAnno: 2039	UniRef50_Q94AR8	509	82.70	497	83	3	263	1750	15	509	0.0	868
4.2.1.35(No Name)																					
No Correspond Unigene																					
4.2.1.9(dihydroxy-acid dehydratase;)																					
ABCDEF_34628	UniRef50_P39522	0.0	Normal: 1310	368	1226	708	881	847	BlastAnno: 2244	UniRef50_P39522	585	58.77	570	226	3	291	1976	16	584	0.0	642
4.3.1.19(threonine ammonia-lyase;)																					
ABCDEF_1551	UniRef50_Q9ZSS6	0.0	Normal: 2	241	47	0	1	1450	BlastAnno: 2188	UniRef50_Q9ZSS6	592	69.90	505	147	3	1844	333	92	592	0.0	699
ABCDEF_10612	UniRef50_Q9ZSS6	0.0	Normal: 2720	1014	2126	1813	2334	1761	BlastAnno: 2348	UniRef50_Q9ZSS6	592	68.70	591	166	5	2148	427	4	592	0.0	763
ABCDEF_84650	UniRef50_Q9ZSS6	0.0	Normal: 2	100	18	0	0	529	BlastAnno: 1546	UniRef50_Q9ZSS6	592	73.05	371	99	1	1202	90	92	461	0.0	533
5.4.99.3(2-acetolactate mutase;)																					
No Correspond Unigene																					
6.1.1.4(leucine---tRNA ligase;)																					
ABCDEF_13128	UniRef50_Q9P2J5	0.0	Normal: 723	291	532	505	506	528	BlastAnno: 2714	UniRef50_Q9P2J5	1176	54.04	816	358	8	281	2710	1	805	0.0	872
ABCDEF_40881	UniRef50_A5VL28	0.0	Normal: 483	833	516	706	501	3648	BlastAnno: 3260	UniRef50_A5VL28	806	52.10	879	341	11	2854	224	2	802	0.0	907
ABCDEF_13134	UniRef50_Q9P2J5	0.0	Normal: 923	312	712	643	607	538	BlastAnno: 2775	UniRef50_Q9P2J5	1176	54.37	789	344	7	421	2769	1	779	0.0	854
6.1.1.5(isoleucine---tRNA ligase;)																					
ABCDEF_4134	UniRef50_Q4DZZ4	0.0	Normal: 955	563	945	1630	2776	1357	BlastAnno: 1945	UniRef50_Q4DZZ4	810	54.46	516	209	5	219	1688	295	810	0.0	588
ABCDEF_17345	UniRef50_A4J2F4	0.0	Normal: 750	1161	806	959	890	3224	BlastAnno: 3137	UniRef50_A4J2F4	931	42.86	959	468	20	3133	296	36	927	0.0	746
ABCDEF_14625	UniRef50_Q9SV89	0.0	Normal: 2527	1610	2465	3837	4242	3087	BlastAnno: 3700	UniRef50_Q9SV89	1254	74.33	1126	265	5	3606	295	1	1124	0.0	1664
ABCDEF_4133	UniRef50_Q4DZZ4	0.0	Normal: 1845	1081	1978	2539	3059	2444	BlastAnno: 1991	UniRef50_Q4DZZ4	810	53.93	521	204	6	311	1780	295	810	0.0	587
6.1.1.9(valine---tRNA ligase;)																					
ABCDEF_43826	UniRef50_Q86KU2	0.0	Normal: 2631	1349	2593	3148	3351	2243	BlastAnno: 4357	UniRef50_Q86KU2	1072	50.25	989	460	13	3345	430	91	1064	0.0	1021
ABCDEF_51319	UniRef50_Q3A253	0.0	Normal: 606	553	547	318	342	3499	BlastAnno: 3463	UniRef50_Q3A253	899	44.77	909	469	13	271	2982	17	897	0.0	786
>00300 Lysine biosynthesis																					
1.1.1.3(homoserine dehydrogenase;)																					
ABCDEF_30670	UniRef50_C6W787	0.0	Normal: 1382	736	1142	1007	1095	1298	BlastAnno: 3147	UniRef50_C6W787	818	40.10	828	481	7	391	2865	3	818	0.0	625
ABCDEF_31297	UniRef50_C6W787	0.0	Normal: 3828	2128	2645	751	1881	2287	BlastAnno: 3215	UniRef50_C6W787	818	41.06	828	472	8	2751	280	3	818	0.0	611
1.1.1.87(homoisocitrate dehydrogenase;)																					
No Correspond Unigene																					
1.2.1.-(Oxidoreductases;)																					
ABCDEF_14573	UniRef50_B5ZBH4	2e-108	Normal: 2547	9518	1266	309	441	22896	BlastAnno: 1872	UniRef50_B5ZBH4	475	42.52	461	259	4	228	1598	16	474	2e-108	343
1.2.1.11(aspartate-semialdehyde dehydrogenase;)																					
ABCDEF_45337	UniRef50_A4YV18	9e-115	Normal: 811	241	805	357	570	636	BlastAnno: 1520	UniRef50_A4YV18	341	54.90	337	145	3	230	1237	5	335	9e-115	351
1.2.1.31(L-aminoadipate-semialdehyde dehydrogenase;)																					
ABCDEF_13666	UniRef50_P49419	0.0	Normal: 711	381	780	675	1270	1126	BlastAnno: 1942	UniRef50_P49419	511	60.16	502	198	2	1747	245	10	510	0.0	588
1.3.1.26(dihydrodipicolinate reductase;)																					
ABCDEF_77643	UniRef50_Q8LB01	3e-146	Normal: 192	125	210	149	243	603	BlastAnno: 1176	UniRef50_Q8LB01	349	76.53	277	65	0	243	1073	73	349	3e-146	427
1.4.1.16(diaminopimelate dehydrogenase;)																					
No Correspond Unigene																					
1.5.1.10(saccharopine dehydrogenase (NADP+, L-glutamate-forming);)																					
ABCDEF_33765	UniRef50_O59711	4e-87	Normal: 787	637	771	1176	2236	598	BlastAnno: 4905	UniRef50_O59711	450	36.19	478	268	9	3352	1925	4	446	4e-87	300
1.5.1.7(saccharopine dehydrogenase (NAD+, L-lysine-forming);)																					
No Correspond Unigene																					
1.5.1.8(saccharopine dehydrogenase (NADP+, L-lysine-forming);)																					
ABCDEF_33765	UniRef50_Q9SMZ4	0.0	Normal: 787	637	771	1176	2236	598	BlastAnno: 4905	UniRef50_Q9SMZ4	1064	68.10	997	312	5	4903	1922	71	1064	0.0	1354
2.3.1.117(2,3,4,5-tetrahydropyridine-2,6-dicarboxylate N-succinyltransferase;)																					
No Correspond Unigene																					
2.3.1.89(tetrahydrodipicolinate N-acetyltransferase;)																					
No Correspond Unigene																					
2.3.3.14(homocitrate synthase;)																					
No Correspond Unigene																					
2.6.1.-(Transferases;)																					
ABCDEF_3716	UniRef50_Q9LQ10	0.0	Normal: 224	353	140	47	76	606	BlastAnno: 2230	UniRef50_Q9LQ10	557	61.35	533	189	8	250	1812	30	557	0.0	609
ABCDEF_37756	UniRef50_Q9LQ10	0.0	Normal: 197	168	149	152	186	490	BlastAnno: 1840	UniRef50_Q9LQ10	557	57.49	534	196	7	1638	109	28	554	0.0	619
2.6.1.11(acetylornithine transaminase;)																					
ABCDEF_31417	UniRef50_O04866	0.0	Normal: 897	597	797	969	1103	1194	BlastAnno: 1786	UniRef50_O04866	451	71.73	467	113	4	1597	197	2	449	0.0	656
2.6.1.17(succinyldiaminopimelate transaminase;)																					
No Correspond Unigene																					
2.6.1.36(L-lysine 6-transaminase;)																					
No Correspond Unigene																					
2.6.1.39(2-aminoadipate transaminase;)																					
No Correspond Unigene																					
2.6.1.57(aromatic-amino-acid transaminase;)																					
No Correspond Unigene																					
2.6.1.83(LL-diaminopimelate aminotransferase;)																					
ABCDEF_22229	UniRef50_Q93ZN9	0.0	Normal: 1417	634	1205	894	1433	2064	BlastAnno: 1880	UniRef50_Q93ZN9	461	75.76	458	109	2	173	1540	1	458	0.0	708
ABCDEF_56912	UniRef50_Q93ZN9	0.0	Normal: 995	434	834	595	976	1393	BlastAnno: 1504	UniRef50_Q93ZN9	461	80.89	382	73	0	1410	265	77	458	0.0	646
2.7.2.-(Transferases;)																					
ABCDEF_27638	UniRef50_Q8TGY9	7e-29	Normal: 104	46	127	175	178	132	BlastAnno: 1233	UniRef50_Q8TGY9	327	31.35	252	125	3	473	1228	102	305	7e-29	121
2.7.2.4(aspartate kinase;)																					
ABCDEF_20311	UniRef50_B9F9Y5	0.0	Normal: 345	128	296	177	197	425	BlastAnno: 2207	UniRef50_B9F9Y5	659	87.77	368	43	1	851	1954	269	634	0.0	667
ABCDEF_31297	UniRef50_O81852	0.0	Normal: 3828	2128	2645	751	1881	2287	BlastAnno: 3215	UniRef50_O81852	916	77.65	841	185	2	2790	271	78	916	0.0	1355

ABCDEF_20747	UniRef50_B9F9Y5	0.0	Normal: 586	335	621	488	462	628	BlastAnno: 2180	UniRef50_B9F9Y5	659	79.23	390	76	1	817	1971	269	658	0.0	627
ABCDEF_30670	UniRef50_O81852	0.0	Normal: 1382	736	1142	1007	1095	1298	BlastAnno: 3147	UniRef50_O81852	916	79.52	840	170	1	355	2874	79	916	0.0	1387
ABCDEF_20746	UniRef50_Q9LYU8	0.0	Normal: 399	257	409	338	357	522	BlastAnno: 2071	UniRef50_Q9LYU8	569	78.12	288	63	0	1002	1865	277	564	0.0	459
3.5.1.-(Hydrolases;)																					
ABCDEF_41579	UniRef50_O81641	0.0	Normal: 384	122	265	320	313	150	BlastAnno: 1456	UniRef50_O81641	428	65.16	399	138	1	1313	120	23	421	0.0	543
ABCDEF_16924	UniRef50_Q8VYX0	0.0	Normal: 135	349	460	1325	685	527	BlastAnno: 2002	UniRef50_Q8VYX0	464	71.72	396	109	2	1612	428	68	461	0.0	549
ABCDEF_24290	UniRef50_O04373	0.0	Normal: 296	238	403	498	698	330	BlastAnno: 1710	UniRef50_O04373	440	65.24	443	151	3	67	1395	1	440	0.0	595
3.5.1.18(succinyl-diaminopimelate desuccinylase;)																					
ABCDEF_8037	UniRef50_A6TJB4	2e-16	Normal: 1106	914	1194	2498	3390	1296	BlastAnno: 1673	UniRef50_A6TJB4	402	29.08	251	159	7	1339	593	67	300	2e-16	87.4
3.5.1.47(N-acetyldiaminopimelate deacetylase;)																					
No Correspond Unigene																					
4.1.1.20(diaminopimelate decarboxylase;)																					
ABCDEF_71859	UniRef50_Q94A94	0.0	Normal: 567	297	747	645	855	984	BlastAnno: 1125	UniRef50_Q94A94	489	88.20	322	38	0	29	994	168	489	0.0	612
ABCDEF_18287	UniRef50_Q94A94	0.0	Normal: 1342	655	1638	1508	1897	1781	BlastAnno: 1747	UniRef50_Q94A94	489	82.34	453	77	2	1553	198	39	489	0.0	795
4.2.1.36(homoaconitate hydratase;)																					
No Correspond Unigene																					
4.2.1.52(dihydrodipicolinate synthase;)																					
ABCDEF_31767	UniRef50_Q9LZX6	0.0	Normal: 933	396	879	964	908	1176	BlastAnno: 1495	UniRef50_Q9LZX6	365	79.71	340	69	0	1300	281	26	365	0.0	543
5.1.1.7(diaminopimelate epimerase)																					
ABCDEF_38750	UniRef50_Q9LFG2	5e-170	Normal: 1695	769	1768	1499	1809	2185	BlastAnno: 1528	UniRef50_Q9LFG2	362	79.06	320	66	1	1255	299	43	362	5e-170	493
6.3.2.10(UDP-N-acetylmuramoyl-tripeptide---D-alanyl-D-alanine ligase;)																					
ABCDEF_37880	UniRef50_B8I6H0	3e-63	Normal: 276	207	248	218	166	354	BlastAnno: 1803	UniRef50_B8I6H0	459	32.39	457	297	7	1603	254	4	455	3e-63	223
6.3.2.13(UDP-N-acetylmuramoyl-L-alanyl-D-glutamate---2,6-diaminopimelate ligase;)																					
ABCDEF_6535	UniRef50_Q0AYR3	1e-95	Normal: 319	180	299	293	170	743	BlastAnno: 2245	UniRef50_Q0AYR3	489	40.93	474	256	6	270	1691	17	466	1e-95	314
>00310 Lysine degradation																					
1.1.1.35(3-hydroxyacyl-CoA dehydrogenase;)																					
ABCDEF_80649	UniRef50_Q1LD07	5e-120	Normal: 527	841	443	1308	1321	1188	BlastAnno: 2586	UniRef50_Q1LD07	715	37.34	699	420	9	2358	277	22	707	5e-120	389
1.13.12.2(lysine 2-monooxygenase;)																					
No Correspond Unigene																					
1.14.11.1(gamma-butyrobetaine dioxygenase;)																					
No Correspond Unigene																					
1.14.11.4(procollagen-lysine 5-dioxygenase;)																					
No Correspond Unigene																					
1.14.11.8(trimethyllysine dioxygenase;)																					
No Correspond Unigene																					
1.14.13.59(L-lysine 6-monooxygenase (NADPH);)																					
No Correspond Unigene																					
1.2.1.20(glutarate-semialdehyde dehydrogenase;)																					
No Correspond Unigene																					
1.2.1.3(aldehyde dehydrogenase (NAD+);)																					
ABCDEF_52483	UniRef50_Q70E96	0.0	Normal: 28	851	41	0	3965	420	BlastAnno: 1750	UniRef50_Q70E96	484	54.56	482	219	0	107	1552	3	484	0.0	548
ABCDEF_17638	UniRef50_Q70E96	0.0	Normal: 580	718	749	2424	1317	775	BlastAnno: 1970	UniRef50_Q70E96	484	62.21	471	177	1	283	1692	2	472	0.0	625
ABCDEF_403	UniRef50_Q8W033	0.0	Normal: 69	355	98	39	223	1111	BlastAnno: 1749	UniRef50_Q8W033	550	65.96	473	161	0	1523	105	74	546	0.0	625
ABCDEF_45143	UniRef50_Q0WSF1	0.0	Normal: 1445	874	1200	1789	1940	1504	BlastAnno: 2450	UniRef50_Q0WSF1	596	79.97	594	115	2	2220	451	1	594	0.0	1015
1.2.1.31(L-aminoadipate-semialdehyde dehydrogenase;)																					
ABCDEF_13666	UniRef50_P49419	0.0	Normal: 711	381	780	675	1270	1126	BlastAnno: 1942	UniRef50_P49419	511	60.16	502	198	2	1747	245	10	510	0.0	588
1.2.1.47(4-trimethylammoniobutyraldehyde dehydrogenase;)																					
ABCDEF_13233	UniRef50_Q9JLJ2	6e-30	Normal: 42	15	35	11	43	20	BlastAnno: 403	UniRef50_Q9JLJ2	494	44.35	124	68	1	7	378	236	358	6e-30	118
1.2.4.-(Oxidoreductases;)																					
No Correspond Unigene																					
1.2.4.2(oxoglutarate dehydrogenase (succinyl-transferring);)																					
ABCDEF_21623	UniRef50_O61199	0.0	Normal: 2615	1939	3031	10740	12054	2537	BlastAnno: 3717	UniRef50_O61199	1029	48.29	996	440	18	3334	491	49	1017	0.0	931
ABCDEF_30869	UniRef50_O61199	0.0	Normal: 1668	226	1504	1974	2525	280	BlastAnno: 3680	UniRef50_O61199	1029	46.61	1047	463	23	3319	326	18	1017	0.0	911
1.3.99.7(glutaryl-CoA dehydrogenase;)																					
ABCDEF_25385	UniRef50_Q22KH3	8e-120	Normal: 747	1014	820	1304	1917	2681	BlastAnno: 1768	UniRef50_Q22KH3	411	45.15	392	213	2	232	1404	17	407	8e-120	369
1.4.1.11(L-erythro-3,5-diaminohexanoate dehydrogenase;)																					
No Correspond Unigene																					
1.4.1.12(2,4-diaminopentanoate dehydrogenase;)																					
No Correspond Unigene																					
1.4.3.14(L-lysine oxidase;)																					
No Correspond Unigene																					
1.5.1.1(pyrroline-2-carboxylate reductase;)																					
No Correspond Unigene																					
1.5.1.10(saccharopine dehydrogenase (NADP+, L-glutamate-forming);)																					
ABCDEF_33765	UniRef50_O59711	4e-87	Normal: 787	637	771	1176	2236	598	BlastAnno: 4905	UniRef50_O59711	450	36.19	478	268	9	3352	1925	4	446	4e-87	300
1.5.1.16(D-lysopine dehydrogenase;)																					
No Correspond Unigene																					
1.5.1.21(Delta1-piperideine-2-carboxylate reductase;)																					
No Correspond Unigene																					
1.5.1.7(saccharopine dehydrogenase (NAD+, L-lysine-forming);)																					
No Correspond Unigene																					
1.5.1.8(saccharopine dehydrogenase (NADP+, L-lysine-forming);)																					

ABCDEF_33765	UniRef50_Q9SMZ4	0.0	Normal: 787	637	771	1176	2236	598	BlastAnno: 4905	UniRef50_Q9SMZ4	1064	68.10	997	312	5	4903	1922	71	1064	0.0	1354

1.5.1.9(saccharopine dehydrogenase (NAD+, L-glutamate-forming);)

No Correspond Unigene

1.5.3.7(L-pipecolate oxidase;)

No Correspond Unigene

1.5.99.3(L-pipecolate dehydrogenase;)

No Correspond Unigene

2.1.1.43(histone-lysine N-methyltransferase;)

ABCDEF_40884	UniRef50_Q5PP37	0.0	Normal: 365	167	397	316	340	393	BlastAnno: 1766	UniRef50_Q5PP37	473	62.47	493	157	5	95	1561	1	469	0.0	604
ABCDEF_51714	UniRef50_O23372	0.0	Normal: 1497	957	1612	1913	1488	1502	BlastAnno: 3391	UniRef50_O23372	2335	67.65	1085	300	8	3390	172	1289	2334	0.0	1439
ABCDEF_20508	UniRef50_P93831	0.0	Normal: 115	122	109	106	208	152	BlastAnno: 2447	UniRef50_P93831	902	49.31	799	330	15	2317	101	117	900	0.0	609
ABCDEF_56188	UniRef50_B9RNE0	0.0	Normal: 59	55	42	48	103	63	BlastAnno: 1856	UniRef50_B9RNE0	884	60.44	589	176	8	1856	252	236	821	0.0	590
ABCDEF_20604	UniRef50_Q7XJS0	0.0	Normal: 146	72	145	140	95	131	BlastAnno: 1625	UniRef50_Q7XJS0	480	63.31	477	173	1	1532	102	1	475	0.0	638
ABCDEF_20507	UniRef50_B9RNE0	0.0	Normal: 84	87	95	68	146	134	BlastAnno: 1759	UniRef50_B9RNE0	884	68.45	504	149	5	1576	89	383	884	0.0	612
ABCDEF_40533	UniRef50_B9SKN2	0.0	Normal: 1021	415	812	589	603	886	BlastAnno: 3389	UniRef50_B9SKN2	832	49.21	825	345	15	3164	780	1	781	0.0	684
ABCDEF_23892	UniRef50_O22781	0.0	Normal: 518	377	418	656	673	657	BlastAnno: 2526	UniRef50_O22781	651	55.46	687	254	7	194	2206	1	651	0.0	766
ABCDEF_23793	UniRef50_Q8GZ42	0.0	Normal: 101	52	110	134	116	122	BlastAnno: 1317	UniRef50_Q8GZ42	1043	72.42	417	110	3	1315	77	445	860	0.0	622
ABCDEF_13862	UniRef50_O64827	0.0	Normal: 528	354	503	291	230	474	BlastAnno: 2397	UniRef50_O64827	1114	50.07	761	324	12	1	2241	395	1113	0.0	717
ABCDEF_40572	UniRef50_O23372	0.0	Normal: 896	629	590	1470	559	473	BlastAnno: 4214	UniRef50_O23372	2335	41.86	1283	554	35	3669	10	131	1284	0.0	819
ABCDEF_34435	UniRef50_Q93YF5	0.0	Normal: 1254	493	976	917	952	659	BlastAnno: 2961	UniRef50_Q93YF5	704	76.94	451	92	4	1232	2581	265	704	0.0	663
ABCDEF_40656	UniRef50_P93831	0.0	Normal: 819	204	680	558	674	600	BlastAnno: 3194	UniRef50_P93831	902	58.41	868	326	16	2905	341	27	872	0.0	824
ABCDEF_38310	UniRef50_Q93YF5	0.0	Normal: 2404	1463	2061	2083	1960	1655	BlastAnno: 2712	UniRef50_Q93YF5	704	71.21	719	171	10	2486	393	1	704	0.0	953
ABCDEF_23792	UniRef50_Q9M364	0.0	Normal: 593	364	681	1102	861	691	BlastAnno: 3506	UniRef50_Q9M364	1018	49.46	1019	392	18	3240	322	77	1018	0.0	982
ABCDEF_27027	UniRef50_P0CB22	0.0	Normal: 920	514	654	836	736	837	BlastAnno: 3681	UniRef50_P0CB22	1083	58.02	1079	395	18	3341	135	51	1081	0.0	1193
ABCDEF_3630	UniRef50_O82175	0.0	Normal: 492	387	431	682	790	632	BlastAnno: 2234	UniRef50_O82175	794	60.57	530	174	7	1738	158	297	794	0.0	637
ABCDEF_20733	UniRef50_O22781	0.0	Normal: 622	447	446	588	384	470	BlastAnno: 2092	UniRef50_O22781	651	52.33	665	228	10	168	2090	1	600	0.0	676
ABCDEF_20603	UniRef50_Q7XJS0	0.0	Normal: 88	43	109	79	55	74	BlastAnno: 1330	UniRef50_Q7XJS0	480	65.23	394	137	0	83	1264	82	475	0.0	538
ABCDEF_48164	UniRef50_B9RGX3	0.0	Normal: 493	44	185	87	128	153	BlastAnno: 1671	UniRef50_B9RGX3	640	59.63	436	112	3	1279	2	137	518	0.0	545
ABCDEF_82788	UniRef50_Q93YF5	0.0	Normal: 880	337	760	739	668	551	BlastAnno: 2676	UniRef50_Q93YF5	704	72.38	717	168	8	414	2513	1	704	0.0	940
ABCDEF_23891	UniRef50_O22781	0.0	Normal: 428	312	357	587	620	594	BlastAnno: 2389	UniRef50_O22781	651	55.46	687	254	7	49	2061	1	651	0.0	766
ABCDEF_81193	UniRef50_Q93YF5	0.0	Normal: 869	333	745	729	649	547	BlastAnno: 2549	UniRef50_Q93YF5	704	72.38	717	168	8	287	2386	1	704	0.0	940

2.1.1.59(No Name)

No Correspond Unigene

2.1.1.60(calmodulin-lysine N-methyltransferase;)

ABCDEF_48898	UniRef50_Q7Z624	2e-19	Normal: 63	26	41	65	102	72	BlastAnno: 762	UniRef50_Q7Z624	323	48.98	98	47	2	478	762	118	215	2e-19	90.9

2.3.1.-(Transferases;)

ABCDEF_520	UniRef50_Q9FF86	0.0	Normal: 1672	85	2273	5056	3383	1211	BlastAnno: 1811	UniRef50_Q9FF86	484	64.06	448	149	6	1664	354	3	449	0.0	573
ABCDEF_68664	UniRef50_Q9ZVN2	0.0	Normal: 79	35	85	107	193	63	BlastAnno: 1488	UniRef50_Q9ZVN2	704	63.99	411	142	3	46	1275	298	703	0.0	540
ABCDEF_52245	UniRef50_Q9FG87	0.0	Normal: 1878	635	1833	3814	1432	1376	BlastAnno: 1758	UniRef50_Q9FG87	529	55.35	486	204	4	4	1428	34	517	0.0	530
ABCDEF_16730	UniRef50_Q3EC11	0.0	Normal: 281	204	237	365	377	458	BlastAnno: 2092	UniRef50_Q3EC11	536	59.33	536	204	8	196	1785	3	530	0.0	661
ABCDEF_4655	UniRef50_A5BMG1	0.0	Normal: 2426	1856	3606	6958	4608	2755	BlastAnno: 2261	UniRef50_A5BMG1	481	68.37	528	112	10	263	1843	3	476	0.0	667
ABCDEF_13860	UniRef50_Q9C533	0.0	Normal: 588	220	570	1451	1338	310	BlastAnno: 2648	UniRef50_Q9C533	596	63.37	606	177	12	2226	511	1	595	0.0	558
ABCDEF_28294	UniRef50_Q9SYZ0	0.0	Normal: 1038	4070	1427	6745	877	2797	BlastAnno: 1785	UniRef50_Q9SYZ0	493	52.37	485	223	3	1598	162	5	487	0.0	533
ABCDEF_47718	UniRef50_Q9C533	0.0	Normal: 1599	236	1657	2093	1231	588	BlastAnno: 2680	UniRef50_Q9C533	596	65.02	626	165	14	2280	472	1	595	0.0	634
ABCDEF_45080	UniRef50_Q9SYZ0	0.0	Normal: 1005	592	1079	2360	3762	2217	BlastAnno: 2130	UniRef50_Q9SYZ0	493	57.85	484	197	3	302	1735	5	487	0.0	533
ABCDEF_25377	UniRef50_Q9C6L5	0.0	Normal: 37	2090	138	2573	1026	1298	BlastAnno: 1334	UniRef50_Q9C6L5	492	79.74	385	76	1	1155	1	6	388	0.0	654
ABCDEF_33470	UniRef50_Q9LW26	0.0	Normal: 172	98	156	260	391	149	BlastAnno: 2993	UniRef50_Q9LW26	701	48.96	627	301	5	655	2508	85	701	0.0	581
ABCDEF_40654	UniRef50_Q9H0A0	0.0	Normal: 638	213	530	318	318	286	BlastAnno: 2216	UniRef50_Q9H0A0	1025	56.87	721	279	6	2127	13	91	795	0.0	756
ABCDEF_33460	UniRef50_Q9ZVN2	0.0	Normal: 144	71	119	216	306	97	BlastAnno: 2691	UniRef50_Q9ZVN2	704	61.50	480	191	4	379	2133	83	636	0.0	715
ABCDEF_14442	UniRef50_Q9SYZ0	0.0	Normal: 341	684	439	1040	1601	552	BlastAnno: 1779	UniRef50_Q9SYZ0	493	63.12	480	171	2	1570	149	5	484	0.0	628
ABCDEF_52626	UniRef50_B6SYP9	0.0	Normal: 815	346	1131	3015	1537	108	BlastAnno: 1861	UniRef50_B6SYP9	494	55.02	438	189	3	1538	249	51	488	0.0	536
ABCDEF_644	UniRef50_Q9SB58	0.0	Normal: 704	444	913	969	1431	848	BlastAnno: 1777	UniRef50_Q9SB58	407	64.15	424	129	8	1572	316	1	406	0.0	554
ABCDEF_27087	UniRef50_Q52T38	0.0	Normal: 324	221	233	419	343	412	BlastAnno: 1631	UniRef50_Q52T38	620	75.95	420	91	1	1409	150	1	410	0.0	681
ABCDEF_34187	UniRef50_Q8L5Y5	0.0	Normal: 497	415	588	523	291	433	BlastAnno: 2677	UniRef50_Q8L5Y5	718	57.30	733	251	15	202	2274	1	713	0.0	683
ABCDEF_47966	UniRef50_Q9SYZ0	0.0	Normal: 577	934	326	580	682	3440	BlastAnno: 2177	UniRef50_Q9SYZ0	493	56.40	484	204	3	1780	347	5	487	0.0	535
ABCDEF_51189	UniRef50_Q52T38	0.0	Normal: 135	152	128	181	219	327	BlastAnno: 1766	UniRef50_Q52T38	620	68.09	470	137	3	80	1483	130	588	0.0	641
ABCDEF_41288	UniRef50_Q9SB58	0.0	Normal: 307	183	332	637	649	322	BlastAnno: 1403	UniRef50_Q9SB58	407	67.56	410	110	6	1401	184	15	405	0.0	523
ABCDEF_33461	UniRef50_Q9ZVN2	0.0	Normal: 142	84	140	206	283	119	BlastAnno: 2904	UniRef50_Q9ZVN2	704	60.98	574	201	6	2271	559	83	636	0.0	686
ABCDEF_14334	UniRef50_Q9SYZ0	0.0	Normal: 2263	129	1724	3469	866	283	BlastAnno: 2039	UniRef50_Q9SYZ0	493	63.22	484	171	2	369	1802	5	487	0.0	627
ABCDEF_24844	UniRef50_Q9FF86	0.0	Normal: 1857	3686	2657	21940	11467	4090	BlastAnno: 1640	UniRef50_Q9FF86	484	66.89	447	135	6	1492	185	2	446	0.0	575
ABCDEF_51190	UniRef50_Q52T38	0.0	Normal: 145	169	142	210	232	335	BlastAnno: 1891	UniRef50_Q52T38	620	70.04	514	141	3	73	1608	86	588	0.0	726
ABCDEF_14085	UniRef50_Q8L5Y5	0.0	Normal: 353	41	384	527	331	280	BlastAnno: 2362	UniRef50_Q8L5Y5	718	62.07	704	220	12	2360	348	24	713	0.0	676
ABCDEF_33458	UniRef50_Q9LW26	0.0	Normal: 158	78	130	240	342	106	BlastAnno: 2822	UniRef50_Q9LW26	701	46.72	655	302	5	400	2337	85	701	0.0	568
ABCDEF_25193	UniRef50_Q9C6L5	0.0	Normal: 3256	368	3548	6230	5109	1288	BlastAnno: 1786	UniRef50_Q9C6L5	492	65.83	480	156	4	1668	241	17	492	0.0	668
ABCDEF_33457	UniRef50_Q9ZVN2	0.0	Normal: 168	83	137	250	375	112	BlastAnno: 2823	UniRef50_Q9ZVN2	704	65.12	496	168	3	379	1860	83	575	0.0	648
ABCDEF_7634	UniRef50_Q9C6L5	0.0	Normal: 2543	9303	2711	11578	5533	10032	BlastAnno: 2064	UniRef50_Q9C6L5	492	52.76	489	225	3	1734	268	1	483	0.0	535
ABCDEF_299	UniRef50_Q71N54	0.0	Normal: 569	1147	511	900	649	1879	BlastAnno: 2017	UniRef50_Q71N54	535	61.73	533	198	5	338	1924	4	534	0.0	677
ABCDEF_27086	UniRef50_Q52T38	0.0	Normal: 784	470	617	843	893	976	BlastAnno: 2398	UniRef50_Q52T38	620	70.80	637	165	4	225	2129	1	618	0.0	887
ABCDEF_51188	UniRef50_Q52T38	0.0	Normal: 273	327	255	415	349	441	BlastAnno: 2324	UniRef50_Q52T38	620	71.30	568	150	3	2041	344	32	588	0.0	815
ABCDEF_44812	UniRef50_Q6DR03	0.0	Normal: 982	902	1005	1071	959	2999	BlastAnno: 2450	UniRef50_Q6DR03	565	54.97	644	198	17	2142	220	1	555	0.0	540

2.3.1.102(N6-hydroxylysine O-acetyltransferase;)

No Correspond Unigene

2.3.1.32(lysine N-acetyltransferase;)																					
No Correspond Unigene																					
2.3.1.61(dihydrolipoyllysine-residue succinyltransferase;)																					
ABCDEF_27826	UniRef50_Q9FLQ4	0.0	Normal: 465	236	709	1274	2770	464	BlastAnno: 1861	UniRef50_Q9FLQ4	464	67.93	474	132	4	1703	306	3	464	0.0	577
ABCDEF_7861	UniRef50_Q9FLQ4	0.0	Normal: 1628	1000	2134	3927	6737	1296	BlastAnno: 1904	UniRef50_Q9FLQ4	464	71.13	471	127	5	142	1554	3	464	0.0	579
ABCDEF_66196	UniRef50_Q9FLQ4	0.0	Normal: 343	184	521	947	1904	361	BlastAnno: 1450	UniRef50_Q9FLQ4	464	76.71	395	90	1	1312	134	70	464	0.0	566
2.3.1.9(acetyl-CoA C-acetyltransferase;)																					
ABCDEF_34825	UniRef50_Q8S4Y1	0.0	Normal: 640	698	620	807	839	1164	BlastAnno: 1773	UniRef50_Q8S4Y1	403	78.11	402	87	1	1597	395	2	403	0.0	598
ABCDEF_14629	UniRef50_Q8S4Y1	0.0	Normal: 634	316	650	1021	1278	691	BlastAnno: 1514	UniRef50_Q8S4Y1	403	85.11	403	60	0	1367	159	1	403	0.0	620
ABCDEF_107	UniRef50_Q8S4Y1	0.0	Normal: 332	460	460	842	893	424	BlastAnno: 1214	UniRef50_Q8S4Y1	403	84.07	339	54	0	1212	196	42	380	0.0	596
ABCDEF_14630	UniRef50_Q8S4Y1	0.0	Normal: 562	282	568	959	1173	627	BlastAnno: 1796	UniRef50_Q8S4Y1	403	85.50	400	58	0	436	1635	4	403	0.0	642
2.4.1.50(procollagen galactosyltransferase;)																					
No Correspond Unigene																					
2.6.1.-(Transferases;)																					
ABCDEF_3716	UniRef50_Q9LQ10	0.0	Normal: 224	353	140	47	76	606	BlastAnno: 2230	UniRef50_Q9LQ10	557	61.35	533	189	8	250	1812	30	557	0.0	609
ABCDEF_37756	UniRef50_Q9LQ10	0.0	Normal: 197	168	149	152	186	490	BlastAnno: 1840	UniRef50_Q9LQ10	557	57.49	534	196	7	1638	109	28	554	0.0	619
2.6.1.21(D-amino-acid transaminase;)																					
No Correspond Unigene																					
2.6.1.39(2-aminoadipate transaminase;)																					
No Correspond Unigene																					
2.6.1.48(5-aminovalerate transaminase;)																					
No Correspond Unigene																					
2.6.1.65(N6-acetyl-beta-lysine transaminase;)																					
No Correspond Unigene																					
2.7.1.81(hydroxylysine kinase;)																					
No Correspond Unigene																					
3.4.-.-(Hydrolases;)																					
ABCDEF_21645	UniRef50_Q1PF50	0.0	Normal: 654	512	669	909	994	888	BlastAnno: 1898	UniRef50_Q1PF50	417	66.35	416	138	1	1558	317	1	416	0.0	592
3.5.1.17(acyl-lysine deacylase;)																					
No Correspond Unigene																					
3.5.1.30(5-aminopentanamidase;)																					
No Correspond Unigene																					
3.5.1.63(4-acetamidobutyrate deacetylase)																					
No Correspond Unigene																					
4.1.1.18(lysine decarboxylase;)																					
No Correspond Unigene																					
4.1.2.-(Lyases;)																					
ABCDEF_38428	UniRef50_C3KN71	1e-47	Normal: 551	427	606	695	1516	1338	BlastAnno: 1495	UniRef50_C3KN71	273	38.34	253	150	1	334	1092	7	253	1e-47	173
4.2.1.17(enoyl-CoA hydratase;)																					
ABCDEF_21259	UniRef50_Q8W1L6	0.0	Normal: 272	276	284	384	326	305	BlastAnno: 2641	UniRef50_Q8W1L6	726	47.57	721	375	3	114	2267	6	726	0.0	633
ABCDEF_80649	UniRef50_Q8W1L6	0.0	Normal: 527	841	443	1308	1321	1188	BlastAnno: 2586	UniRef50_Q8W1L6	726	60.39	712	281	1	2403	268	1	711	0.0	818
ABCDEF_466	UniRef50_Q8W1L6	0.0	Normal: 1014	1690	1094	1677	2438	2080	BlastAnno: 2453	UniRef50_Q8W1L6	726	71.24	699	201	0	2368	272	7	705	0.0	954
ABCDEF_54834	UniRef50_Q8W1L6	0.0	Normal: 229	220	237	298	270	261	BlastAnno: 2405	UniRef50_Q8W1L6	726	47.52	606	315	3	274	2082	121	726	0.0	580
ABCDEF_34756	UniRef50_Q8W1L6	0.0	Normal: 776	726	768	2922	4039	1079	BlastAnno: 2469	UniRef50_Q8W1L6	726	68.93	708	217	2	2396	273	7	711	0.0	957
ABCDEF_4198	UniRef50_Q8W1L6	0.0	Normal: 609	929	532	1401	1568	1297	BlastAnno: 2667	UniRef50_Q8W1L6	726	60.45	713	281	1	2463	325	1	712	0.0	820
5.1.1.5(lysine racemase)																					
No Correspond Unigene																					
5.1.1.9(arginine racemase)																					
No Correspond Unigene																					
5.4.3.2(lysine 2,3-aminomutase)																					
No Correspond Unigene																					
5.4.3.3(beta-lysine 5,6-aminomutase;)																					
No Correspond Unigene																					
5.4.3.4(D-lysine 5,6-aminomutase;)																					
No Correspond Unigene																					
6.2.1.6(glutarate---CoA ligase;)																					
No Correspond Unigene																					
6.3.2.27(aerobactin synthase;)																					
No Correspond Unigene																					

>00311 Penicillin and cephalosporin biosynthesis

1.14.11.26(deacetoxycephalosporin-C hydroxylase;)	
	No Correspond Unigene
1.14.20.1(deacetoxycephalosporin-C synthase;)	
	No Correspond Unigene
1.21.3.1(isopenicillin-N synthase;)	
	No Correspond Unigene
1.4.3.3(D-amino-acid oxidase;)	
	No Correspond Unigene
2.1.3.-(No Name)	
	No Correspond Unigene
2.3.1.164(isopenicillin-N N-acyltransferase;)	
	No Correspond Unigene

	2.3.1.175(deacetylcephalosporin-C acetyltransferase;)																					
	No Correspond Unigene																					
	2.6.1.74(cephalosporin-C transaminase;)																					
	No Correspond Unigene																					
	3.1.1.41(cephalosporin-C deacetylase;)																					
	No Correspond Unigene																					
	3.5.1.11(penicillin amidase;)																					
	No Correspond Unigene																					
	3.5.1.93(glutaryl-7-aminocephalosporanic-acid acylase;)																					
	No Correspond Unigene																					
	3.5.2.6(beta-lactamase;)																					
	No Correspond Unigene																					
	5.1.1.17(isopenicillin-N epimerase)																					
	ABCDEF_37784	UniRef50_Q2JW49	4e-54	Normal: 116	56	136	115	78	95	BlastAnno: 1227	UniRef50_Q2JW49	379	30.69	391	234	9	1226	75	17	377	4e-54	191
	6.3.2.26(N-(5-amino-5-carboxypentanoyl)-L-cysteinyl-D-valine synthase;)																					
	No Correspond Unigene																					
>00312	beta-Lactam resistance																					
	3.5.2.6(beta-lactamase;)																					
	No Correspond Unigene																					
>00330	Arginine and proline metabolism																					
	1.1.1.104(4-oxoproline reductase;)																					
	No Correspond Unigene																					
	1.13.12.1(arginine 2-monooxygenase;)																					
	No Correspond Unigene																					
	1.14.11.2(procollagen-proline dioxygenase;)																					
	No Correspond Unigene																					
	1.14.13.39(nitric-oxide synthase;)																					
	ABCDEF_20929	UniRef50_Q66GP9	0.0	Normal: 151	220	68	115	83	1049	BlastAnno: 2044	UniRef50_Q66GP9	561	64.41	413	126	4	1943	762	2	412	0.0	493
	1.2.1.-(Oxidoreductases;)																					
	ABCDEF_14573	UniRef50_B5ZBH4	2e-108	Normal: 2547	9518	1266	309	441	22896	BlastAnno: 1872	UniRef50_B5ZBH4	475	42.52	461	259	4	228	1598	16	474	2e-108	343
	1.2.1.19(aminobutyraldehyde dehydrogenase;)																					
	No Correspond Unigene																					
	1.2.1.3(aldehyde dehydrogenase (NAD+);)																					
	ABCDEF_52483	UniRef50_Q70E96	0.0	Normal: 28	851	41	0	3965	420	BlastAnno: 1750	UniRef50_Q70E96	484	54.56	482	219	0	107	1552	3	484	0.0	548
	ABCDEF_17638	UniRef50_Q70E96	0.0	Normal: 580	718	749	2424	1317	775	BlastAnno: 1970	UniRef50_Q70E96	484	62.21	471	177	1	283	1692	2	472	0.0	625
	ABCDEF_403	UniRef50_Q8W033	0.0	Normal: 69	355	98	39	223	1111	BlastAnno: 1749	UniRef50_Q8W033	550	65.96	473	161	0	1523	105	74	546	0.0	625
	ABCDEF_45143	UniRef50_Q0WSF1	0.0	Normal: 1445	874	1200	1789	1940	1504	BlastAnno: 2450	UniRef50_Q0WSF1	596	79.97	594	115	2	2220	451	1	594	0.0	1015
	1.2.1.38(N-acetyl-gamma-glutamyl-phosphate reductase;)																					
	ABCDEF_21769	UniRef50_Q6AV34	0.0	Normal: 1237	1001	1113	1101	1912	2251	BlastAnno: 2013	UniRef50_Q6AV34	415	72.03	404	107	3	292	1503	16	413	0.0	590
	1.2.1.41(glutamate-5-semialdehyde dehydrogenase;)																					
	ABCDEF_48785	UniRef50_B7PPZ5	1e-179	Normal: 4099	4503	2789	1378	1588	9742	BlastAnno: 2969	UniRef50_B7PPZ5	726	45.13	729	336	13	364	2481	36	723	1e-179	548
	1.2.1.54(gamma-guanidinobutyraldehyde dehydrogenase;)																					
	No Correspond Unigene																					
	1.2.1.71(succinylglutamate-semialdehyde dehydrogenase;)																					
	No Correspond Unigene																					
	1.21.4.1(D-proline reductase (dithiol))																					
	No Correspond Unigene																					
	1.4.1.12(2,4-diaminopentanoate dehydrogenase;)																					
	No Correspond Unigene																					
	1.4.1.2(glutamate dehydrogenase;)																					
	ABCDEF_52465	UniRef50_Q5HHC7	4e-126	Normal: 404	402	745	2021	3684	1711	BlastAnno: 1651	UniRef50_Q5HHC7	414	44.01	409	227	2	213	1436	6	413	4e-126	384
	1.4.1.3(glutamate dehydrogenase [NAD(P)+];)																					
	ABCDEF_25196	UniRef50_Q43314	0.0	Normal: 271	16	291	548	795	8	BlastAnno: 1412	UniRef50_Q43314	411	78.84	378	80	0	1410	277	34	411	0.0	642
	ABCDEF_52465	UniRef50_Q43314	0.0	Normal: 404	402	745	2021	3684	1711	BlastAnno: 1651	UniRef50_Q43314	411	79.81	411	83	0	210	1442	1	411	0.0	699
	ABCDEF_24937	UniRef50_Q43314	0.0	Normal: 94	39	122	768	1065	114	BlastAnno: 1144	UniRef50_Q43314	411	87.97	349	42	0	1047	1	1	349	0.0	649
	ABCDEF_32993	UniRef50_Q43314	0.0	Normal: 127	299	296	1133	320	898	BlastAnno: 1450	UniRef50_Q43314	411	89.78	411	42	0	1382	150	1	411	0.0	775
	ABCDEF_42257	UniRef50_Q43314	0.0	Normal: 140	355	359	1267	415	1032	BlastAnno: 1644	UniRef50_Q43314	411	89.78	411	42	0	1454	222	1	411	0.0	775
	1.4.1.4(glutamate dehydrogenase (NADP+);)																					
	ABCDEF_41247	UniRef50_P43793	1e-117	Normal: 929	608	918	1231	1278	890	BlastAnno: 2684	UniRef50_P43793	449	42.26	452	250	6	950	2284	1	448	1e-117	374
	1.4.3.-(Oxidoreductases;)																					
	No Correspond Unigene																					
	1.4.3.10(putrescine oxidase)																					
	No Correspond Unigene																					
	1.4.3.22(diamine oxidase;)																					
	ABCDEF_14886	UniRef50_Q59118	5e-55	Normal: 90	94	139	183	301	14	BlastAnno: 661	UniRef50_Q59118	684	49.07	216	105	3	653	6	314	524	5e-55	193
	1.4.3.3(D-amino-acid oxidase;)																					
	No Correspond Unigene																					
	1.4.3.4(monoamine oxidase;)																					
	ABCDEF_11940	UniRef50_Q8LL67	3e-122	Normal: 41	0	82	2	541	4	BlastAnno: 1557	UniRef50_Q8LL67	496	45.61	410	204	3	2	1183	74	480	3e-122	376
	1.5.-.-(Oxidoreductases;)																					
	No Correspond Unigene																					
	1.5.1.1(pyrroline-2-carboxylate reductase;)																					

No Correspond Unigene																					
1.5.1.11(D-octopine dehydrogenase;)																					
No Correspond Unigene																					
1.5.1.12(1-pyrroline-5-carboxylate dehydrogenase;)																					
ABCDEF_21142	UniRef50_Q8VZC3	0.0	Normal: 587	498	633	913	1134	690	BlastAnno: 2062	UniRef50_Q8VZC3	556	76.18	571	119	3	165	1871	1	556	0.0	916
1.5.1.19(D-nopaline dehydrogenase;)																					
No Correspond Unigene																					
1.5.1.2(pyrroline-5-carboxylate reductase;)																					
ABCDEF_10670	UniRef50_O04016	6e-157	Normal: 66	31	49	44	61	74	BlastAnno: 958	UniRef50_O04016	278	80.59	273	53	0	820	2	1	273	6e-157	449
1.5.99.6(spermidine dehydrogenase;)																					
No Correspond Unigene																					
1.5.99.8(proline dehydrogenase;)																					
ABCDEF_39814	UniRef50_P92983	2e-67	Normal: 0	14	0	0	0	94	BlastAnno: 577	UniRef50_P92983	499	59.28	194	76	2	4	576	160	353	2e-67	222
2.1.1.2(guanidinoacetate N-methyltransferase;)																					
No Correspond Unigene																					
2.1.3.11(N-succinylornithine carbamoyltransferase;)																					
No Correspond Unigene																					
2.1.3.3(ornithine carbamoyltransferase;)																					
ABCDEF_67658	UniRef50_O50039	0.0	Normal: 466	184	430	352	481	677	BlastAnno: 1391	UniRef50_O50039	375	77.49	351	73	3	145	1191	29	375	0.0	565
ABCDEF_28304	UniRef50_O50039	0.0	Normal: 685	291	658	580	653	859	BlastAnno: 1552	UniRef50_O50039	375	77.49	351	73	3	306	1352	29	375	0.0	565
2.1.3.9(N-acetylornithine carbamoyltransferase;)																					
No Correspond Unigene																					
2.1.4.1(glycine amidinotransferase;)																					
No Correspond Unigene																					
2.3.1.-(Transferases;)																					
ABCDEF_520	UniRef50_Q9FF86	0.0	Normal: 1672	85	2273	5056	3383	1211	BlastAnno: 1811	UniRef50_Q9FF86	484	64.06	448	149	6	1664	354	3	449	0.0	573
ABCDEF_68664	UniRef50_Q9ZVN2	0.0	Normal: 79	35	85	107	193	63	BlastAnno: 1488	UniRef50_Q9ZVN2	704	63.99	411	142	3	46	1275	298	703	0.0	540
ABCDEF_52245	UniRef50_Q9FG87	0.0	Normal: 1878	635	1833	3814	1432	1376	BlastAnno: 1758	UniRef50_Q9FG87	529	55.35	486	204	4	4	1428	34	517	0.0	530
ABCDEF_16730	UniRef50_Q3EC11	0.0	Normal: 281	204	237	365	377	458	BlastAnno: 2092	UniRef50_Q3EC11	536	59.33	536	204	8	196	1785	3	530	0.0	661
ABCDEF_4655	UniRef50_A5BMG1	0.0	Normal: 2426	1856	3606	6958	4608	2755	BlastAnno: 2261	UniRef50_A5BMG1	481	68.37	528	112	10	263	1843	3	476	0.0	667
ABCDEF_13860	UniRef50_Q9C533	0.0	Normal: 588	220	570	1451	1338	310	BlastAnno: 2648	UniRef50_Q9C533	596	63.37	606	177	12	2226	511	1	595	0.0	558
ABCDEF_28294	UniRef50_Q9SYZ0	0.0	Normal: 1038	4070	1427	6745	877	2797	BlastAnno: 1785	UniRef50_Q9SYZ0	493	52.37	485	223	3	1598	162	5	487	0.0	533
ABCDEF_47718	UniRef50_Q9C533	0.0	Normal: 1599	236	1657	2093	1231	588	BlastAnno: 2680	UniRef50_Q9C533	596	65.02	626	165	14	2280	472	1	595	0.0	634
ABCDEF_45080	UniRef50_Q9SYZ0	0.0	Normal: 1005	592	1079	2360	3762	2217	BlastAnno: 2130	UniRef50_Q9SYZ0	493	57.85	484	197	3	302	1735	5	487	0.0	533
ABCDEF_25377	UniRef50_Q9C6L5	0.0	Normal: 37	2090	138	2573	1026	1298	BlastAnno: 1334	UniRef50_Q9C6L5	492	79.74	385	76	1	1155	1	6	388	0.0	654
ABCDEF_33470	UniRef50_Q9LW26	0.0	Normal: 172	98	156	260	391	149	BlastAnno: 2993	UniRef50_Q9LW26	701	48.96	627	301	5	655	2508	85	701	0.0	581
ABCDEF_40654	UniRef50_Q9HOA0	0.0	Normal: 638	213	530	318	318	286	BlastAnno: 2216	UniRef50_Q9HOA0	1025	56.87	721	279	6	2127	13	91	795	0.0	756
ABCDEF_33460	UniRef50_Q9ZVN2	0.0	Normal: 144	71	119	216	306	97	BlastAnno: 2691	UniRef50_Q9ZVN2	704	61.50	587	191	4	379	2133	83	636	0.0	715
ABCDEF_14442	UniRef50_Q9SYZ0	0.0	Normal: 341	684	439	1040	1601	552	BlastAnno: 1779	UniRef50_Q9SYZ0	493	63.12	480	171	2	1570	149	5	484	0.0	628
ABCDEF_52626	UniRef50_B6SYP9	0.0	Normal: 815	346	1131	3015	1537	108	BlastAnno: 1861	UniRef50_B6SYP9	494	55.02	438	189	3	1538	249	51	488	0.0	536
ABCDEF_644	UniRef50_Q9SB58	0.0	Normal: 704	444	913	969	1431	848	BlastAnno: 1777	UniRef50_Q9SB58	407	64.15	424	129	8	1572	316	1	406	0.0	554
ABCDEF_27087	UniRef50_Q52T38	0.0	Normal: 324	221	233	419	343	412	BlastAnno: 1631	UniRef50_Q52T38	620	75.95	420	91	1	1409	150	1	410	0.0	681
ABCDEF_34187	UniRef50_Q8L5Y5	0.0	Normal: 497	415	588	523	291	433	BlastAnno: 2677	UniRef50_Q8L5Y5	718	57.30	733	251	15	202	2274	1	713	0.0	683
ABCDEF_47966	UniRef50_Q9SYZ0	0.0	Normal: 577	934	326	580	682	3440	BlastAnno: 2177	UniRef50_Q9SYZ0	493	56.40	484	204	3	1780	347	5	487	0.0	535
ABCDEF_51189	UniRef50_Q52T38	0.0	Normal: 135	152	128	181	219	327	BlastAnno: 1766	UniRef50_Q52T38	620	68.09	470	137	3	80	1483	130	588	0.0	641
ABCDEF_41288	UniRef50_Q9SB58	0.0	Normal: 307	183	332	637	649	322	BlastAnno: 1403	UniRef50_Q9SB58	407	67.56	410	110	6	1401	184	15	405	0.0	523
ABCDEF_33461	UniRef50_Q9ZVN2	0.0	Normal: 142	84	140	206	283	119	BlastAnno: 2904	UniRef50_Q9ZVN2	704	60.98	574	201	6	2271	559	83	636	0.0	686
ABCDEF_14334	UniRef50_Q9SYZ0	0.0	Normal: 2263	129	1724	3469	866	283	BlastAnno: 2039	UniRef50_Q9SYZ0	493	63.22	484	171	2	369	1802	5	487	0.0	627
ABCDEF_24844	UniRef50_Q9FF86	0.0	Normal: 1857	3686	2657	21940	11467	4090	BlastAnno: 1640	UniRef50_Q9FF86	484	66.89	447	135	6	1492	185	2	446	0.0	575
ABCDEF_51190	UniRef50_Q52T38	0.0	Normal: 145	169	142	210	232	335	BlastAnno: 1891	UniRef50_Q52T38	620	70.04	514	141	3	73	1608	86	588	0.0	726
ABCDEF_14085	UniRef50_Q8L5Y5	0.0	Normal: 353	41	384	527	331	280	BlastAnno: 2362	UniRef50_Q8L5Y5	718	62.07	704	220	12	2360	348	24	713	0.0	676
ABCDEF_33458	UniRef50_Q9LW26	0.0	Normal: 158	78	130	240	342	106	BlastAnno: 2822	UniRef50_Q9LW26	701	46.72	655	302	5	400	2337	85	701	0.0	568
ABCDEF_25193	UniRef50_Q9C6L5	0.0	Normal: 3256	368	3548	6230	5109	1288	BlastAnno: 1786	UniRef50_Q9C6L5	492	65.83	480	156	4	1668	241	17	492	0.0	668
ABCDEF_33457	UniRef50_Q9ZVN2	0.0	Normal: 168	83	137	250	375	112	BlastAnno: 2823	UniRef50_Q9ZVN2	704	65.12	496	168	3	379	1860	83	575	0.0	648
ABCDEF_7634	UniRef50_Q9C6L5	0.0	Normal: 2543	9303	2711	11578	5533	10032	BlastAnno: 2064	UniRef50_Q9C6L5	492	52.76	489	225	3	1734	268	1	483	0.0	535
ABCDEF_299	UniRef50_Q71N54	0.0	Normal: 569	1147	511	900	649	1879	BlastAnno: 2017	UniRef50_Q71N54	535	61.73	533	198	5	338	1924	4	534	0.0	677
ABCDEF_27086	UniRef50_Q52T38	0.0	Normal: 784	470	617	843	893	976	BlastAnno: 2398	UniRef50_Q52T38	620	70.80	637	165	4	225	2129	1	618	0.0	887
ABCDEF_51188	UniRef50_Q52T38	0.0	Normal: 273	327	255	415	349	441	BlastAnno: 2324	UniRef50_Q52T38	620	71.30	568	150	3	2041	344	32	588	0.0	815
ABCDEF_44812	UniRef50_Q6DR03	0.0	Normal: 982	902	1005	1071	959	2999	BlastAnno: 2450	UniRef50_Q6DR03	565	54.97	644	198	17	2142	220	1	555	0.0	540
2.3.1.1(amino-acid N-acetyltransferase;)																					
ABCDEF_20112	UniRef50_B9SH12	0.0	Normal: 382	261	427	403	709	684	BlastAnno: 2465	UniRef50_B9SH12	705	69.56	565	140	5	2074	464	53	613	0.0	768
ABCDEF_20111	UniRef50_B9SH12	0.0	Normal: 397	542	405	326	344	471	BlastAnno: 2228	UniRef50_B9SH12	705	71.22	542	135	4	1772	210	69	610	0.0	761
2.3.1.109(arginine N-succinyltransferase;)																					
No Correspond Unigene																					
2.3.1.35(glutamate N-acetyltransferase;)																					
ABCDEF_3936	UniRef50_Q8DHN4	1e-120	Normal: 961	311	718	1022	1046	615	BlastAnno: 1796	UniRef50_Q8DHN4	419	55.95	420	171	5	284	1513	4	419	1e-120	372
2.3.1.57(diamine N-acetyltransferase;)																					
ABCDEF_78620	UniRef50_A1ZMR1	1e-08	Normal: 0	0	0	0	0	12	BlastAnno: 193	UniRef50_A1ZMR1	160	42.11	57	33	0	173	3	82	138	1e-08	53.5
2.3.1.64(agmatine N4-coumaroyltransferase;)																					
No Correspond Unigene																					
2.5.1.16(spermidine synthase;)																					
ABCDEF_7812	UniRef50_Q9ZUB3	0.0	Normal: 1661	1302	2150	4493	6957	4671	BlastAnno: 1757	UniRef50_Q9ZUB3	334	81.44	334	49	3	1326	331	11	333	0.0	552

2.5.1.22(spermine synthase;)																					
No Correspond Unigene																					
2.6.1.-(Transferases;)																					
ABCDEF_3716	UniRef50_Q9LQ10	0.0	Normal: 224	353	140	47	76	606	BlastAnno: 2230	UniRef50_Q9LQ10	557	61.35	533	189	8	250	1812	30	557	0.0	609
ABCDEF_37756	UniRef50_Q9LQ10	0.0	Normal: 197	168	149	152	186	490	BlastAnno: 1840	UniRef50_Q9LQ10	557	57.49	534	196	7	1638	109	28	554	0.0	619
2.6.1.1(aspartate transaminase;)																					
ABCDEF_11690	UniRef50_P46248	0.0	Normal: 1745	1815	2022	4078	5299	6095	BlastAnno: 1778	UniRef50_P46248	453	86.09	417	58	0	297	1547	37	453	0.0	733
ABCDEF_1333	UniRef50_C0PSV4	0.0	Normal: 356	544	636	1119	2495	631	BlastAnno: 1594	UniRef50_C0PSV4	462	76.43	403	95	0	1401	193	60	462	0.0	662
ABCDEF_21786	UniRef50_C0PSV4	0.0	Normal: 268	668	330	546	1267	646	BlastAnno: 1736	UniRef50_C0PSV4	462	74.14	464	113	3	50	1441	4	460	0.0	711
ABCDEF_42262	UniRef50_P46643	0.0	Normal: 916	606	1656	2699	2553	856	BlastAnno: 1722	UniRef50_P46643	430	79.49	429	85	2	1635	355	1	428	0.0	731
ABCDEF_56929	UniRef50_P37833	0.0	Normal: 173	278	312	654	1243	297	BlastAnno: 1151	UniRef50_P37833	407	81.82	308	56	0	978	55	3	310	0.0	536
2.6.1.11(acetylornithine transaminase;)																					
ABCDEF_31417	UniRef50_O04866	0.0	Normal: 897	597	797	969	1103	1194	BlastAnno: 1786	UniRef50_O04866	451	71.73	467	113	4	1597	197	2	449	0.0	656
2.6.1.13(ornithine aminotransferase;)																					
ABCDEF_41930	UniRef50_Q92413	1e-160	Normal: 430	709	415	421	587	636	BlastAnno: 1809	UniRef50_Q92413	454	52.88	416	184	4	1561	338	22	433	1e-160	476
2.6.1.21(D-amino-acid transaminase;)																					
No Correspond Unigene																					
2.6.1.23(4-hydroxyglutamate transaminase;)																					
No Correspond Unigene																					
2.6.1.29(diamine transaminase;)																					
No Correspond Unigene																					
2.6.1.81(succinylornithine transaminase;)																					
No Correspond Unigene																					
2.6.1.82(putrescine aminotransferase;)																					
No Correspond Unigene																					
2.6.1.84(arginine---pyruvate transaminase;)																					
No Correspond Unigene																					
2.7.2.11(glutamate 5-kinase;)																					
ABCDEF_48785	UniRef50_P54888	0.0	Normal: 4099	4503	2789	1378	1588	9742	BlastAnno: 2969	UniRef50_P54888	726	76.58	713	167	0	343	2481	1	713	0.0	1071
2.7.2.2(carbamate kinase;)																					
No Correspond Unigene																					
2.7.2.8(acetylglutamate kinase;)																					
ABCDEF_25200	UniRef50_Q6V1L5	4e-113	Normal: 991	686	1022	1103	1414	1597	BlastAnno: 1480	UniRef50_Q6V1L5	301	61.17	291	112	1	1041	169	1	290	4e-113	345
2.7.3.1(guanidinoacetate kinase;)																					
No Correspond Unigene																					
2.7.3.2(creatine kinase;)																					
No Correspond Unigene																					
2.7.3.3(arginine kinase;)																					
ABCDEF_88981	UniRef50_P48610	0.0	Normal: 0	0	0	0	0	236	BlastAnno: 1022	UniRef50_P48610	356	77.41	332	74	1	3	995	25	356	0.0	532
3.4.11.5(prolyl aminopeptidase;)																					
ABCDEF_38624	UniRef50_Q8YXE3	2e-125	Normal: 944	866	797	911	1640	2337	BlastAnno: 1502	UniRef50_Q8YXE3	316	59.42	313	127	0	1180	242	2	314	2e-125	377
3.4.13.3(Xaa-His dipeptidase;)																					
No Correspond Unigene																					
3.5.1.14(aminoacylase;)																					
ABCDEF_25202	UniRef50_Q03154	1e-108	Normal: 953	855	1135	1749	2368	1242	BlastAnno: 1974	UniRef50_Q03154	408	44.42	403	204	6	316	1518	16	400	1e-108	342
3.5.1.16(acetylornithine deacetylase;)																					
ABCDEF_8037	UniRef50_P54638	5e-147	Normal: 1106	914	1194	2498	3390	1296	BlastAnno: 1673	UniRef50_P54638	447	50.68	438	202	5	1534	260	9	445	5e-147	440
3.5.1.2(glutaminase;)																					
ABCDEF_56484	UniRef50_B0TVW4	4e-16	Normal: 9	0	0	0	0	0	BlastAnno: 289	UniRef50_B0TVW4	514	43.37	83	47	0	252	4	428	510	4e-16	79.0
3.5.1.38(glutamin-(asparagin-)ase)																					
No Correspond Unigene																					
3.5.1.4(amidase;)																					
ABCDEF_3151	UniRef50_B9RF45	0.0	Normal: 113	275	72	56	28	1014	BlastAnno: 1850	UniRef50_B9RF45	500	68.47	425	133	1	131	1402	68	492	0.0	547
ABCDEF_81937	UniRef50_B9RF45	0.0	Normal: 101	261	68	53	28	927	BlastAnno: 1739	UniRef50_B9RF45	500	68.47	425	133	1	131	1402	68	492	0.0	546
3.5.1.5(urease)																					
ABCDEF_31241	UniRef50_B2W555	0.0	Normal: 284	167	262	380	250	289	BlastAnno: 1705	UniRef50_B2W555	793	59.82	555	181	5	1703	45	279	793	0.0	647
3.5.1.53(N-carbamoylputrescine amidase;)																					
ABCDEF_19204	UniRef50_Q8VYF5	0.0	Normal: 1054	396	980	1450	1650	605	BlastAnno: 1259	UniRef50_Q8VYF5	326	79.00	319	40	1	1091	216	7	325	0.0	529
ABCDEF_18679	UniRef50_Q8VYF5	0.0	Normal: 1158	431	1085	1609	1898	701	BlastAnno: 1513	UniRef50_Q8VYF5	326	79.00	319	40	1	1345	470	7	325	0.0	529
3.5.1.54(allophanate hydrolase;)																					
No Correspond Unigene																					
3.5.1.59(N-carbamoylsarcosine amidase;)																					
No Correspond Unigene																					
3.5.1.62(acetylputrescine deacetylase)																					
No Correspond Unigene																					
3.5.1.63(4-acetamidobutyrate deacetylase)																					
No Correspond Unigene																					
3.5.1.94(gamma-glutamyl-gamma-aminobutyrate hydrolase;)																					
No Correspond Unigene																					
3.5.1.96(succinylglutamate desuccinylase;)																					
No Correspond Unigene																					
3.5.2.10(creatininase;)																					

No Correspond Unigene

3.5.2.14(N-methylhydantoinase (ATP-hydrolysing);)

No Correspond Unigene

3.5.3.1(arginase;)

ABCDEF_775	UniRef50_P46637	0.0	Normal: 512	259	479	228	745	437	BlastAnno: 1302	UniRef50_P46637	342	84.02	338	51	1	1181	177	5	342	0.0	586
ABCDEF_80837	UniRef50_P46637	0.0	Normal: 0	140	843	18	9	1249	BlastAnno: 1245	UniRef50_P46637	342	78.57	336	69	1	135	1133	7	342	0.0	548
ABCDEF_44331	UniRef50_P46637	0.0	Normal: 0	200	1096	25	9	1498	BlastAnno: 1314	UniRef50_P46637	342	78.57	336	69	1	91	1089	7	342	0.0	548
ABCDEF_44332	UniRef50_P46637	0.0	Normal: 0	216	1247	30	10	1643	BlastAnno: 1328	UniRef50_P46637	342	78.57	336	69	1	91	1089	7	342	0.0	548
ABCDEF_81364	UniRef50_P46637	0.0	Normal: 0	158	1005	23	10	1402	BlastAnno: 1372	UniRef50_P46637	342	78.57	336	69	1	135	1133	7	342	0.0	548

3.5.3.11(agmatinase;)

ABCDEF_80837	UniRef50_Q1IPT1	1e-75	Normal: 0	140	843	18	9	1249	BlastAnno: 1245	UniRef50_Q1IPT1	263	51.26	277	114	8	303	1133	5	260	1e-75	244

3.5.3.12(agmatine deiminase;)

ABCDEF_58669	UniRef50_Q8GWW7	0.0	Normal: 313	126	244	406	536	288	BlastAnno: 1422	UniRef50_Q8GWW7	383	70.64	344	100	1	1099	71	5	348	0.0	522
ABCDEF_19163	UniRef50_Q8GWW7	0.0	Normal: 512	206	480	700	830	537	BlastAnno: 1767	UniRef50_Q8GWW7	383	71.43	371	105	1	1433	324	5	375	0.0	566
ABCDEF_49498	UniRef50_Q8GWW7	0.0	Normal: 583	229	575	855	1009	575	BlastAnno: 1881	UniRef50_Q8GWW7	383	71.97	371	103	1	1537	428	5	375	0.0	568
ABCDEF_53295	UniRef50_Q8GWW7	0.0	Normal: 322	138	276	440	564	285	BlastAnno: 1494	UniRef50_Q8GWW7	383	71.51	344	97	1	1150	122	5	348	0.0	526

3.5.3.23(N-succinylarginine dihydrolase;)

No Correspond Unigene

3.5.3.3(creatinase)

No Correspond Unigene

3.5.3.6(arginine deiminase;)

No Correspond Unigene

3.5.3.7(guanidinobutyrase;)

No Correspond Unigene

3.5.4.1(cytosine deaminase;)

No Correspond Unigene

3.5.4.21(creatinine deaminase;)

No Correspond Unigene

3.5.4.22(1-pyrroline-4-hydroxy-2-carboxylate deaminase;)

No Correspond Unigene

4.1.1.-(Lyases;)

ABCDEF_57496	UniRef50_O96569	9e-20	Normal: 4	0	0	0	0	9	BlastAnno: 223	UniRef50_O96569	439	66.67	72	22	1	210	1	19	90	9e-20	87.4

4.1.1.17(ornithine decarboxylase;)

ABCDEF_35775	UniRef50_Q8S3N2	3e-102	Normal: 121	135	89	32	30	308	BlastAnno: 709	UniRef50_Q8S3N2	435	69.29	241	62	3	23	709	1	241	3e-102	312

4.1.1.19(arginine decarboxylase;)

ABCDEF_42368	UniRef50_A9RKF0	0.0	Normal: 7080	6326	4216	4601	6991	18491	BlastAnno: 3023	UniRef50_A9RKF0	668	49.28	621	284	6	703	2508	21	629	0.0	588

4.1.1.3(oxaloacetate decarboxylase;)

No Correspond Unigene

4.1.1.50(adenosylmethionine decarboxylase;)

ABCDEF_11694	UniRef50_Q9S7T9	3e-156	Normal: 1259	2802	2513	10565	16423	4466	BlastAnno: 1898	UniRef50_Q9S7T9	362	62.98	362	130	3	610	1683	1	362	3e-156	462

4.1.1.75(5-guanidino-2-oxopentanoate decarboxylase;)

No Correspond Unigene

4.1.2.14(2-dehydro-3-deoxy-phosphogluconate aldolase;)

ABCDEF_11235	UniRef50_C1F496	4e-27	Normal: 18	32	12	12	50	235	BlastAnno: 1242	UniRef50_C1F496	218	35.98	214	129	4	165	797	9	217	4e-27	114

4.1.3.16(4-hydroxy-2-oxoglutarate aldolase;)

ABCDEF_58429	UniRef50_A9F631	9e-18	Normal: 6	1	0	0	8	25	BlastAnno: 519	UniRef50_A9F631	223	32.50	160	89	2	26	505	25	165	9e-18	83.6

4.3.1.12(ornithine cyclodeaminase;)

ABCDEF_16498	UniRef50_C5CJY4	3e-58	Normal: 329	333	543	667	517	468	BlastAnno: 1701	UniRef50_C5CJY4	315	40.84	311	177	5	449	1378	4	308	3e-58	204

4.3.2.1(argininosuccinate lyase;)

ABCDEF_27228	UniRef50_Q1Q5N1	4e-163	Normal: 522	249	449	458	484	526	BlastAnno: 1943	UniRef50_Q1Q5N1	460	52.85	456	215	0	244	1611	2	457	4e-163	484

5.1.1.4(proline racemase)

No Correspond Unigene

5.1.1.8(4-hydroxyproline epimerase;)

No Correspond Unigene

6.3.1.11(glutamate---putrescine ligase;)

No Correspond Unigene

6.3.1.2(glutamate---ammonia ligase;)

ABCDEF_27409	UniRef50_Q56WN1	0.0	Normal: 792	857	694	764	746	4126	BlastAnno: 1892	UniRef50_Q56WN1	356	80.76	343	66	0	460	1488	1	343	0.0	547
ABCDEF_60010	UniRef50_Q56WN1	0.0	Normal: 722	827	669	717	712	3883	BlastAnno: 1644	UniRef50_Q56WN1	356	80.76	343	66	0	290	1318	1	343	0.0	547
ABCDEF_27406	UniRef50_Q56WN1	0.0	Normal: 1322	8376	1449	1550	2297	27785	BlastAnno: 1946	UniRef50_Q56WN1	356	79.49	356	73	0	537	1604	1	356	0.0	546
ABCDEF_38019	UniRef50_Q56WN1	0.0	Normal: 65	126	230	858	520	162	BlastAnno: 1336	UniRef50_Q56WN1	356	85.11	356	53	0	77	1144	1	356	0.0	654
ABCDEF_28058	UniRef50_Q56WN1	0.0	Normal: 999	55	1008	535	2834	260	BlastAnno: 1368	UniRef50_Q56WN1	356	86.30	343	47	0	93	1121	14	356	0.0	587
ABCDEF_28620	UniRef50_Q56WN1	0.0	Normal: 2577	3235	3447	8812	12665	2225	BlastAnno: 1455	UniRef50_Q56WN1	356	91.12	338	30	0	169	1182	16	353	0.0	595

6.3.2.11(carnosine synthase;)

No Correspond Unigene

6.3.4.16(carbamoyl-phosphate synthase (ammonia);)

No Correspond Unigene

6.3.4.5(argininosuccinate synthase;)

ABCDEF_57250	UniRef50_Q24ZG8	0.0	Normal: 1037	382	856	747	1026	1222	BlastAnno: 1645	UniRef50_Q24ZG8	409	62.22	397	149	1	378	1568	9	404	0.0	525
ABCDEF_28455	UniRef50_Q9SZX3	0.0	Normal: 1243	467	989	866	1234	1550	BlastAnno: 1756	UniRef50_Q9SZX3	494	79.74	459	90	2	296	1669	36	492	0.0	756

6.3.4.6(urea carboxylase;)

No Correspond Unigene

>00331 Clavulanic acid biosynthesis

1.14.11.21(clavaminate synthase;)

No Correspond Unigene

2.5.1.66(N2-(2-carboxyethyl)arginine synthase;)

No Correspond Unigene

3.5.3.22(proclavaminate amidinohydrolase;)

No Correspond Unigene

6.3.3.4(No Name)

No Correspond Unigene

>00340 Histidine metabolism

1.1.1.23(histidinol dehydrogenase;)

ABCDEF_34398	UniRef50_Q9C5U8	0.0	Normal: 280	237	254	295	265	546	BlastAnno: 1943	UniRef50_Q9C5U8	466	80.73	441	81	1	1567	245	24	460	0.0	745
ABCDEF_34401	UniRef50_Q9C5U8	0.0	Normal: 273	228	238	285	277	474	BlastAnno: 1950	UniRef50_Q9C5U8	466	86.81	288	38	0	1101	238	173	460	0.0	527
ABCDEF_34400	UniRef50_Q9C5U8	0.0	Normal: 266	219	237	275	264	479	BlastAnno: 1932	UniRef50_Q9C5U8	466	86.81	288	38	0	1101	238	173	460	0.0	527
ABCDEF_72202	UniRef50_Q9C5U8	0.0	Normal: 202	165	189	194	203	344	BlastAnno: 1219	UniRef50_Q9C5U8	466	86.46	288	39	0	1099	236	173	460	0.0	523
ABCDEF_34399	UniRef50_Q9C5U8	0.0	Normal: 287	246	255	305	279	542	BlastAnno: 1961	UniRef50_Q9C5U8	466	80.73	441	81	1	1567	245	24	460	0.0	745

1.14.13.-(Oxidoreductases;)

ABCDEF_45538	UniRef50_O23024	3e-156	Normal: 110	6	165	774	133	36	BlastAnno: 1258	UniRef50_O23024	437	58.18	385	151	3	3	1157	37	411	3e-156	457

1.14.13.5(imidazoleacetate 4-monooxygenase;)

No Correspond Unigene

1.2.1.3(aldehyde dehydrogenase (NAD+);)

ABCDEF_52483	UniRef50_Q70E96	0.0	Normal: 28	851	41	0	3965	420	BlastAnno: 1750	UniRef50_Q70E96	484	54.56	482	219	0	107	1552	3	484	0.0	548
ABCDEF_17638	UniRef50_Q70E96	0.0	Normal: 580	718	749	2424	1317	775	BlastAnno: 1970	UniRef50_Q70E96	484	62.21	471	177	1	283	1692	2	472	0.0	625
ABCDEF_403	UniRef50_Q8W033	0.0	Normal: 69	355	98	39	223	1111	BlastAnno: 1749	UniRef50_Q8W033	550	65.96	473	161	0	1523	105	74	546	0.0	625
ABCDEF_45143	UniRef50_Q0WSF1	0.0	Normal: 1445	874	1200	1789	1940	1504	BlastAnno: 2450	UniRef50_Q0WSF1	596	79.97	594	115	2	2220	451	1	594	0.0	1015

1.2.1.5(aldehyde dehydrogenase [NAD(P)+];)

ABCDEF_81997	UniRef50_P43353	5e-53	Normal: 32	81	25	16	59	314	BlastAnno: 770	UniRef50_P43353	468	45.37	205	106	3	746	138	261	461	5e-53	186

1.4.3.22(diamine oxidase;)

ABCDEF_14886	UniRef50_Q59118	5e-55	Normal: 90	94	139	183	301	14	BlastAnno: 661	UniRef50_Q59118	684	49.07	216	105	3	653	6	314	524	5e-55	193

1.4.3.4(monoamine oxidase;)

ABCDEF_11940	UniRef50_Q8LL67	3e-122	Normal: 41	0	82	2	541	4	BlastAnno: 1557	UniRef50_Q8LL67	496	45.61	410	204	3	2	1183	74	480	3e-122	376

2.1.1.-(Transferases;)

ABCDEF_20806	UniRef50_Q94II3	0.0	Normal: 700	332	876	1458	1997	929	BlastAnno: 2275	UniRef50_Q94II3	600	73.57	594	153	4	2119	341	1	591	0.0	907
ABCDEF_45802	UniRef50_Q9ZPH9	0.0	Normal: 3543	3348	4296	9797	10830	5017	BlastAnno: 2642	UniRef50_Q9ZPH9	633	53.90	603	261	7	2216	456	23	624	0.0	676
ABCDEF_20070	UniRef50_Q944R7	0.0	Normal: 301	141	265	207	185	187	BlastAnno: 2675	UniRef50_Q944R7	724	58.28	719	275	9	127	2265	13	712	0.0	840
ABCDEF_34956	UniRef50_Q94II3	0.0	Normal: 1120	7269	2475	16033	22219	21461	BlastAnno: 2676	UniRef50_Q94II3	600	73.77	610	146	4	404	2230	1	597	0.0	957
ABCDEF_17121	UniRef50_Q9ZPH9	0.0	Normal: 124	77	109	284	199	132	BlastAnno: 2066	UniRef50_Q9ZPH9	633	53.24	601	259	8	161	1903	25	623	0.0	644
ABCDEF_6524	UniRef50_Q9ZPH9	0.0	Normal: 471	296	490	753	870	730	BlastAnno: 2602	UniRef50_Q9ZPH9	633	50.89	619	290	5	2221	404	6	623	0.0	659
ABCDEF_56017	UniRef50_Q9ZPH9	0.0	Normal: 302	167	286	596	748	529	BlastAnno: 2384	UniRef50_Q9ZPH9	633	51.37	619	287	5	213	2030	6	623	0.0	663
ABCDEF_44169	UniRef50_Q9SIZ3	0.0	Normal: 179	158	273	705	685	229	BlastAnno: 2300	UniRef50_Q9SIZ3	589	62.01	308	108	4	1219	296	285	583	0.0	394
ABCDEF_6521	UniRef50_Q9ZPH9	0.0	Normal: 339	198	314	641	767	582	BlastAnno: 2511	UniRef50_Q9ZPH9	633	51.37	619	287	5	2172	355	6	623	0.0	663
ABCDEF_44172	UniRef50_Q9SIZ3	0.0	Normal: 204	184	287	767	741	242	BlastAnno: 2317	UniRef50_Q9SIZ3	589	68.15	449	134	4	1637	291	144	583	0.0	641
ABCDEF_199	UniRef50_Q9SU94	0.0	Normal: 1076	275	1009	558	1247	306	BlastAnno: 1769	UniRef50_Q9SU94	390	78.45	348	74	1	284	1324	43	390	0.0	584
ABCDEF_52252	UniRef50_Q84W92	0.0	Normal: 1658	720	1653	1980	2289	1601	BlastAnno: 2170	UniRef50_Q84W92	535	72.49	469	120	3	409	1791	34	501	0.0	703
ABCDEF_51404	UniRef50_A3BMN9	0.0	Normal: 864	188	716	337	631	326	BlastAnno: 2192	UniRef50_A3BMN9	620	50.50	604	243	9	2008	251	55	620	0.0	555
ABCDEF_21386	UniRef50_Q94AI4	0.0	Normal: 2326	1250	2220	4294	2920	1983	BlastAnno: 3701	UniRef50_Q94AI4	775	47.27	842	332	16	567	2978	8	775	0.0	577
ABCDEF_35101	UniRef50_Q9MAT5	0.0	Normal: 1440	420	1268	576	1136	850	BlastAnno: 1634	UniRef50_Q9MAT5	383	69.03	352	109	0	1449	394	32	383	0.0	550
ABCDEF_960	UniRef50_Q9LN50	0.0	Normal: 3504	13198	3609	11156	19651	12155	BlastAnno: 2626	UniRef50_Q9LN50	724	49.14	525	246	5	883	2451	219	724	0.0	570
ABCDEF_48082	UniRef50_Q9LN50	0.0	Normal: 410	445	467	870	993	454	BlastAnno: 2340	UniRef50_Q9LN50	724	70.25	521	155	0	710	2272	204	724	0.0	821
ABCDEF_10473	UniRef50_Q08A71	0.0	Normal: 374	187	332	425	456	314	BlastAnno: 1764	UniRef50_Q08A71	435	79.23	366	75	1	344	1438	70	435	0.0	575
ABCDEF_7295	UniRef50_Q9ZPH9	0.0	Normal: 25	54	30	81	26	329	BlastAnno: 1191	UniRef50_Q9ZPH9	633	69.23	403	110	4	1191	22	225	626	0.0	599
ABCDEF_4160	UniRef50_Q94II3	0.0	Normal: 189	0	210	71	17	72	BlastAnno: 1424	UniRef50_Q94II3	600	77.28	449	101	1	1424	81	143	591	0.0	758
ABCDEF_44857	UniRef50_Q9ZPH9	0.0	Normal: 101	224	103	299	588	350	BlastAnno: 1582	UniRef50_Q9ZPH9	633	72.06	519	143	1	1571	21	114	632	0.0	822
ABCDEF_71541	UniRef50_Q9SIZ3	0.0	Normal: 187	167	296	765	724	237	BlastAnno: 2268	UniRef50_Q9SIZ3	589	62.01	308	108	4	1210	287	285	583	0.0	394
ABCDEF_44171	UniRef50_Q9SIZ3	0.0	Normal: 214	196	312	827	802	251	BlastAnno: 2339	UniRef50_Q9SIZ3	589	64.78	548	182	5	1978	335	47	583	0.0	717
ABCDEF_20805	UniRef50_Q94II3	0.0	Normal: 674	344	885	1417	2194	963	BlastAnno: 2400	UniRef50_Q94II3	600	78.21	436	94	1	879	2183	156	591	0.0	734
ABCDEF_18210	UniRef50_Q9SIZ3	0.0	Normal: 5584	3714	7379	17006	21892	7546	BlastAnno: 3199	UniRef50_Q9SIZ3	589	50.00	576	269	7	2145	424	27	585	0.0	574
ABCDEF_6526	UniRef50_Q9ZPH9	0.0	Normal: 627	185	837	2717	2815	1530	BlastAnno: 2611	UniRef50_Q9ZPH9	633	54.76	599	259	5	462	2222	25	623	0.0	689
ABCDEF_6525	UniRef50_Q9ZPH9	0.0	Normal: 602	165	760	2369	2605	1400	BlastAnno: 2545	UniRef50_Q9ZPH9	633	54.92	599	258	5	431	2191	25	623	0.0	689
ABCDEF_6523	UniRef50_Q9ZPH9	0.0	Normal: 314	175	304	641	784	552	BlastAnno: 2312	UniRef50_Q9ZPH9	633	52.42	599	272	4	201	1958	25	623	0.0	663
ABCDEF_67393	UniRef50_Q9ZPH9	0.0	Normal: 434	245	444	608	768	655	BlastAnno: 2346	UniRef50_Q9ZPH9	633	51.92	599	275	4	232	1989	25	623	0.0	659
ABCDEF_3579	UniRef50_O14744	0.0	Normal: 1361	326	984	568	791	440	BlastAnno: 2369	UniRef50_O14744	637	47.00	634	294	10	267	2120	30	637	0.0	553

2.1.1.22(carnosine N-methyltransferase)

No Correspond Unigene

2.1.1.8(histamine N-methyltransferase;)

No Correspond Unigene

2.1.2.5(glutamate formimidoyltransferase;)

ABCDEF_24160	UniRef50_Q3AE85	1e-09	Normal: 112	179	129	133	48	463	BlastAnno: 872	UniRef50_Q3AE85	305	30.00	130	74	4	712	341	10	128	1e-09	63.9

2.3.1.-(Transferases;)

ABCDEF_520	UniRef50_Q9FF86	0.0	Normal: 1672	85	2273	5056	3383	1211	BlastAnno: 1811	UniRef50_Q9FF86	484	64.06	448	149	6	1664	354	3	449	0.0	573
ABCDEF_68664	UniRef50_Q9ZVN2	0.0	Normal: 79	35	85	107	193	63	BlastAnno: 1488	UniRef50_Q9ZVN2	704	63.99	411	142	3	46	1275	298	703	0.0	540
ABCDEF_52245	UniRef50_Q9FG87	0.0	Normal: 1878	635	1833	3814	1432	1376	BlastAnno: 1758	UniRef50_Q9FG87	529	55.35	486	204	4	4	1428	34	517	0.0	530

ABCDEF_16730	UniRef50_Q3EC11	0.0	Normal: 281	204	237	365	377	458	BlastAnno: 2092	UniRef50_Q3EC11	536	59.33	536	204	8	196	1785	3	530	0.0	661
ABCDEF_4655	UniRef50_A5BMG1	0.0	Normal: 2426	1856	3606	6958	4608	2755	BlastAnno: 2261	UniRef50_A5BMG1	481	68.37	528	112	10	263	1843	3	476	0.0	667
ABCDEF_13860	UniRef50_Q9C533	0.0	Normal: 588	220	570	1451	1338	310	BlastAnno: 2648	UniRef50_Q9C533	596	63.37	606	177	12	2226	511	1	595	0.0	558
ABCDEF_28294	UniRef50_Q9SYZ0	0.0	Normal: 1038	4070	1427	6745	877	2797	BlastAnno: 1785	UniRef50_Q9SYZ0	493	52.37	485	223	3	1598	162	5	487	0.0	533
ABCDEF_47718	UniRef50_Q9C533	0.0	Normal: 1599	236	1657	2093	1231	588	BlastAnno: 2680	UniRef50_Q9C533	596	65.02	626	165	14	2280	472	1	595	0.0	634
ABCDEF_45080	UniRef50_Q9SYZ0	0.0	Normal: 1005	592	1079	2360	3762	2217	BlastAnno: 2130	UniRef50_Q9SYZ0	493	57.85	484	197	3	302	1735	5	487	0.0	533
ABCDEF_25377	UniRef50_Q9C6L5	0.0	Normal: 37	2090	138	2573	1026	1298	BlastAnno: 1334	UniRef50_Q9C6L5	492	79.74	385	76	1	1155	1	6	388	0.0	654
ABCDEF_33470	UniRef50_Q9LW26	0.0	Normal: 172	98	156	260	391	149	BlastAnno: 2993	UniRef50_Q9LW26	701	48.96	627	301	5	655	2508	85	701	0.0	581
ABCDEF_40654	UniRef50_Q9H0A0	0.0	Normal: 638	213	530	318	318	286	BlastAnno: 2216	UniRef50_Q9H0A0	1025	56.87	721	279	6	2127	13	91	795	0.0	756
ABCDEF_33460	UniRef50_Q9ZVN2	0.0	Normal: 144	71	119	216	306	97	BlastAnno: 2691	UniRef50_Q9ZVN2	704	61.50	587	191	4	379	2133	83	636	0.0	715
ABCDEF_14442	UniRef50_Q9SYZ0	0.0	Normal: 341	684	439	1040	1601	552	BlastAnno: 1779	UniRef50_Q9SYZ0	493	63.12	480	171	2	1570	149	5	484	0.0	628
ABCDEF_52626	UniRef50_B6SYP9	0.0	Normal: 815	346	1131	3015	1537	108	BlastAnno: 1861	UniRef50_B6SYP9	494	55.02	438	189	3	1538	249	51	488	0.0	536
ABCDEF_644	UniRef50_Q9SB58	0.0	Normal: 704	444	913	969	1431	848	BlastAnno: 1777	UniRef50_Q9SB58	407	64.15	424	129	8	1572	316	1	406	0.0	554
ABCDEF_27087	UniRef50_Q52T38	0.0	Normal: 324	221	233	419	343	412	BlastAnno: 1631	UniRef50_Q52T38	620	75.95	420	91	1	1409	150	1	410	0.0	681
ABCDEF_34187	UniRef50_Q8L5Y5	0.0	Normal: 497	415	588	523	291	433	BlastAnno: 2677	UniRef50_Q8L5Y5	718	57.30	733	251	15	202	2274	1	713	0.0	683
ABCDEF_47966	UniRef50_Q9SYZ0	0.0	Normal: 577	934	326	580	682	3440	BlastAnno: 2177	UniRef50_Q9SYZ0	493	56.40	484	204	3	1780	347	5	487	0.0	535
ABCDEF_51189	UniRef50_Q52T38	0.0	Normal: 135	152	128	181	219	327	BlastAnno: 1766	UniRef50_Q52T38	620	68.09	470	137	3	80	1483	130	588	0.0	641
ABCDEF_41288	UniRef50_Q9SB58	0.0	Normal: 307	183	332	637	649	322	BlastAnno: 1403	UniRef50_Q9SB58	407	67.56	410	110	6	1401	184	15	405	0.0	523
ABCDEF_33461	UniRef50_Q9ZVN2	0.0	Normal: 142	84	140	206	283	119	BlastAnno: 2904	UniRef50_Q9ZVN2	704	60.98	574	201	6	2271	559	83	636	0.0	686
ABCDEF_14334	UniRef50_Q9SYZ0	0.0	Normal: 2263	129	1724	3469	866	283	BlastAnno: 2039	UniRef50_Q9SYZ0	493	63.22	484	171	2	369	1802	5	487	0.0	627
ABCDEF_24844	UniRef50_Q9FF86	0.0	Normal: 1857	3686	2657	21940	11467	4090	BlastAnno: 1640	UniRef50_Q9FF86	484	66.89	447	135	6	1492	185	2	446	0.0	575
ABCDEF_51190	UniRef50_Q52T38	0.0	Normal: 145	169	142	210	232	335	BlastAnno: 1891	UniRef50_Q52T38	620	70.04	514	141	3	73	1608	86	588	0.0	726
ABCDEF_14085	UniRef50_Q8L5Y5	0.0	Normal: 353	41	384	527	331	280	BlastAnno: 2362	UniRef50_Q8L5Y5	718	62.07	704	220	12	2360	348	24	713	0.0	676
ABCDEF_33458	UniRef50_Q9LW26	0.0	Normal: 158	78	130	240	342	106	BlastAnno: 2822	UniRef50_Q9LW26	701	46.72	655	302	5	400	2337	85	701	0.0	568
ABCDEF_25193	UniRef50_Q9C6L5	0.0	Normal: 3256	368	3548	6230	5109	1288	BlastAnno: 1786	UniRef50_Q9C6L5	492	65.83	480	156	4	1668	241	17	492	0.0	668
ABCDEF_33457	UniRef50_Q9ZVN2	0.0	Normal: 168	83	137	250	375	112	BlastAnno: 2823	UniRef50_Q9ZVN2	704	65.12	496	168	3	379	1860	83	575	0.0	648
ABCDEF_7634	UniRef50_Q9C6L5	0.0	Normal: 2543	9303	2711	11578	5533	10032	BlastAnno: 2064	UniRef50_Q9C6L5	492	52.76	489	225	3	1734	268	1	483	0.0	535
ABCDEF_299	UniRef50_Q71N54	0.0	Normal: 569	1147	511	900	649	1879	BlastAnno: 2017	UniRef50_Q71N54	535	61.73	533	198	5	338	1924	4	534	0.0	677
ABCDEF_27086	UniRef50_Q52T38	0.0	Normal: 784	470	617	843	893	976	BlastAnno: 2398	UniRef50_Q52T38	620	70.80	637	165	4	225	2129	1	618	0.0	887
ABCDEF_51188	UniRef50_Q52T38	0.0	Normal: 273	327	255	415	349	441	BlastAnno: 2324	UniRef50_Q52T38	620	71.30	568	150	3	2041	344	32	588	0.0	815
ABCDEF_44812	UniRef50_Q6DR03	0.0	Normal: 982	902	1005	1071	959	2999	BlastAnno: 2450	UniRef50_Q6DR03	565	54.97	644	198	17	2142	220	1	555	0.0	540
2.4.2.17(ATP phosphoribosyltransferase;)																					
ABCDEF_52986	UniRef50_Q118Q1	2e-72	Normal: 1344	904	1349	1276	1326	3320	BlastAnno: 1692	UniRef50_Q118Q1	338	37.01	335	200	4	404	1393	9	337	2e-72	243
2.6.1.38(histidine transaminase;)																					
No Correspond Unigene																					
2.6.1.9(histidinol-phosphate transaminase;)																					
ABCDEF_45962	UniRef50_A7NFV2	2e-99	Normal: 595	558	686	955	1251	1312	BlastAnno: 1635	UniRef50_A7NFV2	371	48.76	363	178	4	336	1403	7	368	2e-99	313
3.1.3.15(histidinol-phosphatase;)																					
ABCDEF_35094	UniRef50_Q6NPM8	2e-146	Normal: 520	258	509	458	626	860	BlastAnno: 1352	UniRef50_Q6NPM8	346	72.28	285	79	0	1083	229	60	344	2e-146	431
3.4.13.20(beta-Ala-His dipeptidase;)																					
No Correspond Unigene																					
3.4.13.3(Xaa-His dipeptidase;)																					
No Correspond Unigene																					
3.4.13.5(Xaa-methyl-His dipeptidase;)																					
No Correspond Unigene																					
3.5.1.15(aspartoacylase;)																					
No Correspond Unigene																					
3.5.1.68(N-formylglutamate deformylase;)																					
No Correspond Unigene																					
3.5.1.8(formylaspartate deformylase;)																					
No Correspond Unigene																					
3.5.2.-(Hydrolases;)																					
No Correspond Unigene																					
3.5.2.7(imidazolonepropionase;)																					
No Correspond Unigene																					
3.5.3.13(formimidoylglutamate deiminase;)																					
No Correspond Unigene																					
3.5.3.5(formimidoylaspartate deiminase;)																					
No Correspond Unigene																					
3.5.3.8(formimidoylglutamase;)																					
No Correspond Unigene																					
3.5.4.19(phosphoribosyl-AMP cyclohydrolase;)																					
ABCDEF_34398	UniRef50_P00815	2e-135	Normal: 280	237	254	295	265	546	BlastAnno: 1943	UniRef50_P00815	799	51.29	425	195	6	1492	245	370	791	2e-135	424
3.6.1.31(phosphoribosyl-ATP diphosphatase;)																					
ABCDEF_27015	UniRef50_A8HTA8	5e-76	Normal: 97	67	137	170	192	288	BlastAnno: 1208	UniRef50_A8HTA8	308	53.19	235	98	4	959	291	68	302	5e-76	246
4.1.1.22(histidine decarboxylase;)																					
ABCDEF_41201	UniRef50_P54772	2e-173	Normal: 568	471	584	601	932	628	BlastAnno: 1918	UniRef50_P54772	413	58.29	410	163	6	413	1633	2	406	2e-173	509
4.1.1.28(aromatic-L-amino-acid decarboxylase;)																					
ABCDEF_28052	UniRef50_P54770	3e-145	Normal: 150	74	133	88	177	332	BlastAnno: 1237	UniRef50_P54770	533	55.61	374	141	2	1237	191	144	517	3e-145	433
4.2.1.19(imidazoleglycerol-phosphate dehydratase;)																					
ABCDEF_18581	UniRef50_O23346	5e-126	Normal: 305	290	313	346	397	851	BlastAnno: 1221	UniRef50_O23346	272	84.98	213	30	1	292	924	60	272	5e-126	374
4.2.1.49(urocanate hydratase;)																					

	ABCDEF_87438	UniRef50_Q96N76	2e-35	Normal: 0	0	0	0	0	14	BlastAnno: 330	UniRef50_Q96N76	676	64.08	103	37	0	310	2	536	638	2e-35	134
4.3.1.3(histidine ammonia-lyase;)																						
	ABCDEF_77305	UniRef50_B5XIY2	3e-13	Normal: 218	337	343	1269	246	230	BlastAnno: 612	UniRef50_B5XIY2	513	36.80	125	70	3	241	609	5	122	3e-13	73.9
5.3.1.16(1-(5-phosphoribosyl)-5-[(5-phosphoribosylamino)methylideneamino]imidazole-4-carboxamide isomerase;)																						
	ABCDEF_44934	UniRef50_O82782	4e-143	Normal: 158	94	141	141	213	186	BlastAnno: 1215	UniRef50_O82782	304	78.46	260	56	0	287	1066	44	303	4e-143	418
6.3.2.11(carnosine synthase;)																						
	No Correspond Unigene																					
6.3.4.8(imidazoleacetate---phosphoribosyldiphosphate ligase;)																						
	No Correspond Unigene																					
>00350 Tyrosine metabolism																						
1.1.1.1(alcohol dehydrogenase;)																						
	ABCDEF_31928	UniRef50_B9RHN6	0.0	Normal: 721	714	784	1329	1570	1819	BlastAnno: 1711	UniRef50_B9RHN6	437	77.55	392	87	1	360	1535	46	436	0.0	610
	ABCDEF_10335	UniRef50_Q8LEB2	0.0	Normal: 285	158	514	810	352	183	BlastAnno: 1547	UniRef50_Q8LEB2	381	74.93	379	92	1	1317	181	5	380	0.0	598
1.1.1.222(No Name)																						
	No Correspond Unigene																					
1.1.1.237(hydroxyphenylpyruvate reductase;)																						
	No Correspond Unigene																					
1.1.1.90(aryl-alcohol dehydrogenase;)																						
	No Correspond Unigene																					
1.10.3.1(catechol oxidase;)																						
	ABCDEF_45625	UniRef50_P43311	9e-157	Normal: 20202	1798	18188	15443	32697	6972	BlastAnno: 2080	UniRef50_P43311	607	49.32	584	252	14	1895	243	34	606	9e-157	475
1.11.1.8(iodide peroxidase;)																						
	No Correspond Unigene																					
1.13.11.15(3,4-dihydroxyphenylacetate 2,3-dioxygenase;)																						
	No Correspond Unigene																					
1.13.11.27(4-hydroxyphenylpyruvate dioxygenase;)																						
	ABCDEF_7370	UniRef50_P93836	0.0	Normal: 512	1122	253	32	118	3444	BlastAnno: 1725	UniRef50_P93836	445	74.00	423	102	3	1616	363	23	442	0.0	604
1.13.11.29(stizolobate synthase)																						
	No Correspond Unigene																					
1.13.11.30(stizolobinate synthase)																						
	No Correspond Unigene																					
1.13.11.4(gentisate 1,2-dioxygenase;)																						
	No Correspond Unigene																					
1.13.11.5(homogentisate 1,2-dioxygenase;)																						
	ABCDEF_7368	UniRef50_Q93099	6e-173	Normal: 260	416	205	300	283	1362	BlastAnno: 1849	UniRef50_Q93099	445	57.85	446	173	8	239	1552	2	440	6e-173	508
1.13.12.-(Oxidoreductases;)																						
	No Correspond Unigene																					
1.14.12.9(4-chlorophenylacetate 3,4-dioxygenase)																						
	No Correspond Unigene																					
1.14.13.18(4-hydroxyphenylacetate 1-monooxygenase;)																						
	No Correspond Unigene																					
1.14.13.3(4-hydroxyphenylacetate 3-monooxygenase;)																						
	No Correspond Unigene																					
1.14.16.2(tyrosine 3-monooxygenase;)																						
	No Correspond Unigene																					
1.14.17.1(dopamine beta-monooxygenase;)																						
	ABCDEF_20224	UniRef50_Q9VCG8	3e-15	Normal: 82	33	62	111	97	53	BlastAnno: 1165	UniRef50_Q9VCG8	748	26.72	363	218	14	1023	10	271	610	3e-15	83.6
1.14.18.-(Oxidoreductases;)																						
	No Correspond Unigene																					
1.14.18.1(monophenol monooxygenase;)																						
	No Correspond Unigene																					
1.2.1.16(succinate-semialdehyde dehydrogenase [NAD(P)+];)																						
	ABCDEF_35184	UniRef50_Q39Q67	0.0	Normal: 907	754	991	1776	1616	1278	BlastAnno: 2107	UniRef50_Q39Q67	509	57.38	481	205	0	1853	411	29	509	0.0	578
1.2.1.29(aryl-aldehyde dehydrogenase)																						
	No Correspond Unigene																					
1.2.1.45(4-carboxy-2-hydroxymuconate-6-semialdehyde dehydrogenase;)																						
	No Correspond Unigene																					
1.2.1.5(aldehyde dehydrogenase [NAD(P)+];)																						
	ABCDEF_81997	UniRef50_P43353	5e-53	Normal: 32	81	25	16	59	314	BlastAnno: 770	UniRef50_P43353	468	45.37	205	106	3	746	138	261	461	5e-53	186
1.2.1.53(4-hydroxyphenylacetaldehyde dehydrogenase;)																						
	No Correspond Unigene																					
1.2.1.60(5-carboxymethyl-2-hydroxymuconic-semialdehyde dehydrogenase;)																						
	No Correspond Unigene																					
1.2.3.1(aldehyde oxidase;)																						
	ABCDEF_37559	UniRef50_Q7G193	0.0	Normal: 208	649	188	236	120	436	BlastAnno: 2825	UniRef50_Q7G193	1368	58.36	927	352	11	2741	3	3	909	0.0	1063
	ABCDEF_51652	UniRef50_Q7G193	0.0	Normal: 97	323	130	90	67	347	BlastAnno: 1575	UniRef50_Q7G193	1368	64.15	491	172	4	5	1468	871	1360	0.0	632
1.2.3.13(4-hydroxyphenylpyruvate oxidase)																						
	No Correspond Unigene																					
1.4.3.2(L-amino-acid oxidase;)																						
	ABCDEF_34035	UniRef50_B9R844	0.0	Normal: 973	600	948	1073	764	972	BlastAnno: 3436	UniRef50_B9R844	1947	69.56	611	156	9	1050	2867	1361	1946	0.0	707
1.4.3.21(primary-amine oxidase;)																						
	ABCDEF_3522	UniRef50_Q8H1H9	0.0	Normal: 405	723	295	151	922	46	BlastAnno: 2086	UniRef50_Q8H1H9	712	64.54	595	204	6	2	1774	116	707	0.0	811

ABCDEF_3521	UniRef50_Q8H1H9	0.0	Normal: 322	637	263	137	709	53	BlastAnno: 1768	UniRef50_Q8H1H9	712	68.24	510	156	5	1630	113	200	707	0.0	744

1.4.3.4(monoamine oxidase;)

ABCDEF_11940	UniRef50_Q8LL67	3e-122	Normal: 41	0	82	2	541	4	BlastAnno: 1557	UniRef50_Q8LL67	496	45.61	410	204	3	2	1183	74	480	3e-122	376

1.4.99.4(aralkylamine dehydrogenase;)

No Correspond Unigene

2.1.1.-(Transferases;)

ABCDEF_20806	UniRef50_Q94II3	0.0	Normal: 700	332	876	1458	1997	929	BlastAnno: 2275	UniRef50_Q94II3	600	73.57	594	153	4	2119	341	1	591	0.0	907
ABCDEF_45802	UniRef50_Q9ZPH9	0.0	Normal: 3543	3348	4296	9797	10830	5017	BlastAnno: 2642	UniRef50_Q9ZPH9	633	53.90	603	261	7	2216	456	23	624	0.0	676
ABCDEF_20070	UniRef50_Q944R7	0.0	Normal: 301	141	265	207	185	187	BlastAnno: 2675	UniRef50_Q944R7	724	58.28	719	275	9	127	2265	13	712	0.0	840
ABCDEF_34956	UniRef50_Q94II3	0.0	Normal: 1120	7269	2475	16033	22219	21461	BlastAnno: 2676	UniRef50_Q94II3	600	73.77	610	146	4	404	2230	1	597	0.0	957
ABCDEF_17121	UniRef50_Q9ZPH9	0.0	Normal: 124	77	109	284	199	132	BlastAnno: 2066	UniRef50_Q9ZPH9	633	53.24	601	259	8	161	1903	25	623	0.0	644
ABCDEF_6524	UniRef50_Q9ZPH9	0.0	Normal: 471	296	490	753	870	730	BlastAnno: 2602	UniRef50_Q9ZPH9	633	50.89	619	290	5	2221	404	6	623	0.0	659
ABCDEF_56017	UniRef50_Q9ZPH9	0.0	Normal: 302	167	286	596	748	529	BlastAnno: 2384	UniRef50_Q9ZPH9	633	51.37	619	287	5	213	2030	6	623	0.0	663
ABCDEF_44169	UniRef50_Q9SIZ3	0.0	Normal: 179	158	273	705	685	229	BlastAnno: 2300	UniRef50_Q9SIZ3	589	62.01	308	108	4	1219	296	285	583	0.0	394
ABCDEF_6521	UniRef50_Q9ZPH9	0.0	Normal: 339	198	314	641	767	582	BlastAnno: 2511	UniRef50_Q9ZPH9	633	51.37	619	287	5	2172	355	6	623	0.0	663
ABCDEF_44172	UniRef50_Q9SIZ3	0.0	Normal: 204	184	287	767	741	242	BlastAnno: 2317	UniRef50_Q9SIZ3	589	68.15	449	134	4	1637	291	144	583	0.0	641
ABCDEF_199	UniRef50_Q9SU94	0.0	Normal: 1076	275	1009	558	1247	306	BlastAnno: 1769	UniRef50_Q9SU94	390	78.45	348	74	1	284	1324	43	390	0.0	584
ABCDEF_52252	UniRef50_Q84W92	0.0	Normal: 1658	720	1653	1980	2289	1601	BlastAnno: 2170	UniRef50_Q84W92	535	72.49	469	120	3	409	1791	34	501	0.0	703
ABCDEF_51404	UniRef50_A3BMN9	0.0	Normal: 864	188	716	337	631	326	BlastAnno: 2192	UniRef50_A3BMN9	620	50.50	604	243	9	2008	251	55	620	0.0	555
ABCDEF_21386	UniRef50_Q94AI4	0.0	Normal: 2326	1250	2220	4294	2920	1983	BlastAnno: 3701	UniRef50_Q94AI4	775	47.27	842	332	16	567	2978	8	775	0.0	577
ABCDEF_35101	UniRef50_Q9MAT5	0.0	Normal: 1440	420	1268	576	1136	850	BlastAnno: 1634	UniRef50_Q9MAT5	383	69.03	352	109	0	1449	394	32	383	0.0	550
ABCDEF_960	UniRef50_Q9LN50	0.0	Normal: 3504	13198	3609	11156	19651	12155	BlastAnno: 2626	UniRef50_Q9LN50	724	49.14	525	246	5	883	2451	219	724	0.0	570
ABCDEF_48082	UniRef50_Q9LN50	0.0	Normal: 410	445	467	870	993	454	BlastAnno: 2340	UniRef50_Q9LN50	724	70.25	521	155	0	710	2272	204	724	0.0	821
ABCDEF_10473	UniRef50_Q08A71	0.0	Normal: 374	187	332	425	456	314	BlastAnno: 1764	UniRef50_Q08A71	435	79.23	366	75	1	344	1438	70	435	0.0	575
ABCDEF_7295	UniRef50_Q9ZPH9	0.0	Normal: 25	54	30	81	26	329	BlastAnno: 1191	UniRef50_Q9ZPH9	633	69.23	403	110	4	1191	22	225	626	0.0	599
ABCDEF_4160	UniRef50_Q94II3	0.0	Normal: 189	0	210	71	17	72	BlastAnno: 1424	UniRef50_Q94II3	600	77.28	449	101	1	1424	81	143	591	0.0	758
ABCDEF_44857	UniRef50_Q9ZPH9	0.0	Normal: 101	224	103	299	588	350	BlastAnno: 1582	UniRef50_Q9ZPH9	633	72.06	519	143	1	1571	21	114	632	0.0	822
ABCDEF_71541	UniRef50_Q9SIZ3	0.0	Normal: 187	167	296	765	724	237	BlastAnno: 2268	UniRef50_Q9SIZ3	589	62.01	308	108	4	1210	287	285	583	0.0	394
ABCDEF_44171	UniRef50_Q9SIZ3	0.0	Normal: 214	196	312	827	802	251	BlastAnno: 2339	UniRef50_Q9SIZ3	589	64.78	548	182	5	1978	335	47	583	0.0	717
ABCDEF_20805	UniRef50_Q94II3	0.0	Normal: 674	344	885	1417	2194	963	BlastAnno: 2400	UniRef50_Q94II3	600	78.21	436	94	1	879	2183	156	591	0.0	734
ABCDEF_18210	UniRef50_Q9SIZ3	0.0	Normal: 5584	3714	7379	17006	21892	7546	BlastAnno: 3199	UniRef50_Q9SIZ3	589	50.00	576	269	7	2145	424	27	585	0.0	574
ABCDEF_6526	UniRef50_Q9ZPH9	0.0	Normal: 627	185	837	2717	2815	1530	BlastAnno: 2611	UniRef50_Q9ZPH9	633	54.76	599	259	5	462	2222	25	623	0.0	689
ABCDEF_6525	UniRef50_Q9ZPH9	0.0	Normal: 602	165	760	2369	2605	1400	BlastAnno: 2545	UniRef50_Q9ZPH9	633	54.92	599	258	5	431	2191	25	623	0.0	689
ABCDEF_6523	UniRef50_Q9ZPH9	0.0	Normal: 314	175	304	641	784	552	BlastAnno: 2312	UniRef50_Q9ZPH9	633	52.42	599	272	4	201	1958	25	623	0.0	663
ABCDEF_67393	UniRef50_Q9ZPH9	0.0	Normal: 434	245	444	608	768	655	BlastAnno: 2346	UniRef50_Q9ZPH9	633	51.92	599	275	4	232	1989	25	623	0.0	659
ABCDEF_3579	UniRef50_O14744	0.0	Normal: 1361	326	984	568	791	440	BlastAnno: 2369	UniRef50_O14744	637	47.00	634	294	10	267	2120	30	637	0.0	553

2.1.1.27(tyramine N-methyltransferase;)

No Correspond Unigene

2.1.1.28(phenylethanolamine N-methyltransferase;)

No Correspond Unigene

2.1.1.6(catechol O-methyltransferase;)

ABCDEF_3867	UniRef50_Q9ZTU1	4e-129	Normal: 43	1755	68	456	3254	474	BlastAnno: 1421	UniRef50_Q9ZTU1	351	51.57	351	167	2	1306	260	2	351	4e-129	387

2.3.1.-(Transferases;)

ABCDEF_520	UniRef50_Q9FF86	0.0	Normal: 1672	85	2273	5056	3383	1211	BlastAnno: 1811	UniRef50_Q9FF86	484	64.06	448	149	6	1664	354	3	449	0.0	573
ABCDEF_68664	UniRef50_Q9ZVN2	0.0	Normal: 79	35	85	107	193	63	BlastAnno: 1488	UniRef50_Q9ZVN2	704	63.99	411	142	3	46	1275	298	703	0.0	540
ABCDEF_52245	UniRef50_Q9FG87	0.0	Normal: 1878	635	1833	3814	1432	1376	BlastAnno: 1758	UniRef50_Q9FG87	529	55.35	486	204	4	4	1428	34	517	0.0	530
ABCDEF_16730	UniRef50_Q3EC11	0.0	Normal: 281	204	237	365	377	458	BlastAnno: 2092	UniRef50_Q3EC11	536	59.33	536	204	8	196	1785	3	530	0.0	661
ABCDEF_4655	UniRef50_A5BMG1	0.0	Normal: 2426	1856	3606	6958	4608	2755	BlastAnno: 2261	UniRef50_A5BMG1	481	68.37	528	112	10	263	1843	3	476	0.0	667
ABCDEF_13860	UniRef50_Q9C533	0.0	Normal: 588	220	570	1451	1338	310	BlastAnno: 2648	UniRef50_Q9C533	596	63.37	606	177	12	2226	511	1	595	0.0	558
ABCDEF_28294	UniRef50_Q9SYZ0	0.0	Normal: 1038	4070	1427	6745	877	2797	BlastAnno: 1785	UniRef50_Q9SYZ0	493	52.37	485	223	3	1598	162	5	487	0.0	533
ABCDEF_47718	UniRef50_Q9C533	0.0	Normal: 1599	236	1657	2093	1231	588	BlastAnno: 2680	UniRef50_Q9C533	596	65.02	626	165	14	2280	472	1	595	0.0	634
ABCDEF_45080	UniRef50_Q9SYZ0	0.0	Normal: 1005	592	1079	2360	3762	2217	BlastAnno: 2130	UniRef50_Q9SYZ0	493	57.85	484	197	3	302	1735	5	487	0.0	533
ABCDEF_25377	UniRef50_Q9C6L5	0.0	Normal: 37	2090	138	2573	1026	1298	BlastAnno: 1334	UniRef50_Q9C6L5	492	79.74	385	76	1	1155	1	6	388	0.0	654
ABCDEF_33470	UniRef50_Q9LW26	0.0	Normal: 172	98	156	260	391	149	BlastAnno: 2993	UniRef50_Q9LW26	701	48.96	627	301	5	655	2508	85	701	0.0	581
ABCDEF_40654	UniRef50_Q9H0A0	0.0	Normal: 638	213	530	318	318	286	BlastAnno: 2216	UniRef50_Q9H0A0	1025	56.87	721	279	6	2127	13	91	795	0.0	756
ABCDEF_33460	UniRef50_Q9ZVN2	0.0	Normal: 144	71	119	216	306	97	BlastAnno: 2691	UniRef50_Q9ZVN2	704	61.50	587	191	4	379	2133	83	636	0.0	715
ABCDEF_14442	UniRef50_Q9SYZ0	0.0	Normal: 341	684	439	1040	1601	552	BlastAnno: 1779	UniRef50_Q9SYZ0	493	63.12	480	171	2	1570	149	5	484	0.0	628
ABCDEF_52626	UniRef50_B6SYP9	0.0	Normal: 815	346	1131	3015	1537	108	BlastAnno: 1861	UniRef50_B6SYP9	494	55.02	438	189	3	1538	249	51	488	0.0	536
ABCDEF_644	UniRef50_Q9SB58	0.0	Normal: 704	444	913	969	1431	848	BlastAnno: 1777	UniRef50_Q9SB58	407	64.15	424	129	8	1572	316	1	406	0.0	554
ABCDEF_27087	UniRef50_Q52T38	0.0	Normal: 324	221	233	419	343	412	BlastAnno: 1631	UniRef50_Q52T38	620	75.95	420	91	1	1409	150	1	410	0.0	681
ABCDEF_34187	UniRef50_Q8L5Y5	0.0	Normal: 497	415	588	523	291	433	BlastAnno: 2677	UniRef50_Q8L5Y5	718	57.30	733	251	15	202	2274	1	713	0.0	683
ABCDEF_47966	UniRef50_Q9SYZ0	0.0	Normal: 577	934	326	580	682	3440	BlastAnno: 2177	UniRef50_Q9SYZ0	493	56.40	484	204	3	1780	347	5	487	0.0	535
ABCDEF_51189	UniRef50_Q52T38	0.0	Normal: 135	152	128	181	219	327	BlastAnno: 1766	UniRef50_Q52T38	620	68.09	470	137	3	80	1483	130	588	0.0	641
ABCDEF_41288	UniRef50_Q9SB58	0.0	Normal: 307	183	332	637	649	322	BlastAnno: 1403	UniRef50_Q9SB58	407	67.56	410	110	6	1401	184	15	405	0.0	523
ABCDEF_33461	UniRef50_Q9ZVN2	0.0	Normal: 142	84	140	206	283	119	BlastAnno: 2904	UniRef50_Q9ZVN2	704	60.98	574	201	6	2271	559	83	636	0.0	686
ABCDEF_14334	UniRef50_Q9SYZ0	0.0	Normal: 2263	129	1724	3469	866	283	BlastAnno: 2039	UniRef50_Q9SYZ0	493	63.22	484	171	2	369	1802	5	487	0.0	627
ABCDEF_24844	UniRef50_Q9FF86	0.0	Normal: 1857	3686	2657	21940	11467	4090	BlastAnno: 1640	UniRef50_Q9FF86	484	66.89	447	135	6	1492	185	2	446	0.0	575
ABCDEF_51190	UniRef50_Q52T38	0.0	Normal: 145	169	142	210	232	335	BlastAnno: 1891	UniRef50_Q52T38	620	70.04	514	141	3	73	1608	86	588	0.0	726
ABCDEF_14085	UniRef50_Q8L5Y5	0.0	Normal: 353	41	384	527	331	280	BlastAnno: 2362	UniRef50_Q8L5Y5	718	62.07	704	220	12	2360	348	24	713	0.0	676
ABCDEF_33458	UniRef50_Q9LW26	0.0	Normal: 158	78	130	240	342	106	BlastAnno: 2822	UniRef50_Q9LW26	701	46.72	655	302	5	400	2337	85	701	0.0	568
ABCDEF_25193	UniRef50_Q9C6L5	0.0	Normal: 3256	368	3548	6230	5109	1288	BlastAnno: 1786	UniRef50_Q9C6L5	492	65.83	480	156	4	1668	241	17	492	0.0	668
ABCDEF_33457	UniRef50_Q9ZVN2	0.0	Normal: 168	83	137	250	375	112	BlastAnno: 2823	UniRef50_Q9ZVN2	704	65.12	496	168	3	379	1860	83	575	0.0	648

ABCDEF_7634	UniRef50_Q9C6L5	0.0	Normal: 2543	9303	2711	11578	5533	10032	BlastAnno: 2064	UniRef50_Q9C6L5	492	52.76	489	225	3	1734	268	1	483	0.0	535
ABCDEF_299	UniRef50_Q71N54	0.0	Normal: 569	1147	511	900	649	1879	BlastAnno: 2017	UniRef50_Q71N54	535	61.73	533	198	5	338	1924	4	534	0.0	677
ABCDEF_27086	UniRef50_Q52T38	0.0	Normal: 784	470	617	843	893	976	BlastAnno: 2398	UniRef50_Q52T38	620	70.80	637	165	4	225	2129	1	618	0.0	887
ABCDEF_51188	UniRef50_Q52T38	0.0	Normal: 273	327	255	415	349	441	BlastAnno: 2324	UniRef50_Q52T38	620	71.30	568	150	3	2041	344	32	588	0.0	815
ABCDEF_44812	UniRef50_Q6DR03	0.0	Normal: 982	902	1005	1071	959	2999	BlastAnno: 2450	UniRef50_Q6DR03	565	54.97	644	198	17	2142	220	1	555	0.0	540
2.3.1.14(glutamine N-phenylacetyltransferase;)																					
No Correspond Unigene																					
2.3.1.140(rosmarinate synthase;)																					
No Correspond Unigene																					
2.6.1.1(aspartate transaminase;)																					
ABCDEF_11690	UniRef50_P46248	0.0	Normal: 1745	1815	2022	4078	5299	6095	BlastAnno: 1778	UniRef50_P46248	453	86.09	417	58	0	297	1547	37	453	0.0	733
ABCDEF_1333	UniRef50_C0PSV4	0.0	Normal: 356	544	636	1119	2495	631	BlastAnno: 1594	UniRef50_C0PSV4	462	76.43	403	95	0	1401	193	60	462	0.0	662
ABCDEF_21786	UniRef50_C0PSV4	0.0	Normal: 268	668	330	546	1267	646	BlastAnno: 1736	UniRef50_C0PSV4	462	74.14	464	113	3	50	1441	4	460	0.0	711
ABCDEF_42262	UniRef50_P46643	0.0	Normal: 916	606	1656	2699	2553	856	BlastAnno: 1722	UniRef50_P46643	430	79.49	429	85	2	1635	355	1	428	0.0	731
ABCDEF_56929	UniRef50_P37833	0.0	Normal: 173	278	312	654	1243	297	BlastAnno: 1151	UniRef50_P37833	407	81.82	308	56	0	978	55	3	310	0.0	536
2.6.1.49(dihydroxyphenylalanine transaminase;)																					
No Correspond Unigene																					
2.6.1.5(tyrosine transaminase;)																					
ABCDEF_52650	UniRef50_Q9LVY1	0.0	Normal: 493	358	455	302	300	892	BlastAnno: 1442	UniRef50_Q9LVY1	420	61.26	413	160	0	65	1303	5	417	0.0	539
2.6.1.57(aromatic-amino-acid transaminase;)																					
No Correspond Unigene																					
2.6.1.58(phenylalanine(histidine) transaminase;)																					
No Correspond Unigene																					
2.6.1.9(histidinol-phosphate transaminase;)																					
ABCDEF_45962	UniRef50_A7NFV2	2e-99	Normal: 595	558	686	955	1251	1312	BlastAnno: 1635	UniRef50_A7NFV2	371	48.76	363	178	4	336	1403	7	368	2e-99	313
3.7.1.2(fumarylacetoacetase;)																					
ABCDEF_25631	UniRef50_P16930	2e-172	Normal: 2583	1744	2457	3093	7463	3464	BlastAnno: 1676	UniRef50_P16930	419	58.89	416	167	3	1435	194	2	415	2e-172	504
3.7.1.5(acylpyruvate hydrolase)																					
ABCDEF_37171	UniRef50_Q86I22	2e-84	Normal: 58	35	54	123	307	55	BlastAnno: 1201	UniRef50_Q86I22	218	60.47	215	83	2	73	711	2	216	2e-84	265
ABCDEF_37169	UniRef50_Q86I22	2e-84	Normal: 60	27	46	117	292	44	BlastAnno: 1192	UniRef50_Q86I22	218	60.47	215	83	2	137	775	2	216	2e-84	265
4.1.1.-(Lyases;)																					
ABCDEF_57496	UniRef50_O96569	9e-20	Normal: 4	0	0	0	0	9	BlastAnno: 223	UniRef50_O96569	439	66.67	72	22	1	210	1	19	90	9e-20	87.4
4.1.1.25(tyrosine decarboxylase;)																					
ABCDEF_28052	UniRef50_Q8RY79	0.0	Normal: 150	74	133	88	177	332	BlastAnno: 1237	UniRef50_Q8RY79	490	74.93	355	89	0	1237	173	134	488	0.0	551
4.1.1.28(aromatic-L-amino-acid decarboxylase;)																					
ABCDEF_28052	UniRef50_P54770	3e-145	Normal: 150	74	133	88	177	332	BlastAnno: 1237	UniRef50_P54770	533	55.61	374	141	2	1237	191	144	517	3e-145	433
4.1.1.62(gentisate decarboxylase;)																					
No Correspond Unigene																					
4.1.1.68(5-oxopent-3-ene-1,2,5-tricarboxylate decarboxylase;)																					
No Correspond Unigene																					
4.1.1.80(4-hydroxyphenylpyruvate decarboxylase;)																					
No Correspond Unigene																					
4.1.2.-(Lyases;)																					
ABCDEF_38428	UniRef50_C3KN71	1e-47	Normal: 551	427	606	695	1516	1338	BlastAnno: 1495	UniRef50_C3KN71	273	38.34	253	150	1	334	1092	7	253	1e-47	173
4.1.99.2(tyrosine phenol-lyase;)																					
No Correspond Unigene																					
4.2.1.-(Lyases;)																					
ABCDEF_48679	UniRef50_Q0RP29	5e-115	Normal: 568	325	563	941	1285	676	BlastAnno: 1424	UniRef50_Q0RP29	493	56.39	321	129	4	119	1075	39	350	5e-115	356
4.3.1.22(3,4-dihydroxyphenylalanine reductive deaminase;)																					
No Correspond Unigene																					
5.2.1.2(maleylacetoacetate isomerase;)																					
ABCDEF_24780	UniRef50_O43708	2e-58	Normal: 315	636	278	477	702	802	BlastAnno: 1018	UniRef50_O43708	216	50.00	214	100	3	822	187	8	216	2e-58	196
ABCDEF_73734	UniRef50_O43708	2e-58	Normal: 201	411	188	302	537	754	BlastAnno: 949	UniRef50_O43708	216	50.00	214	100	3	822	187	8	216	2e-58	195
5.2.1.4(maleylpyruvate isomerase)																					
No Correspond Unigene																					
5.3.2.1(phenylpyruvate tautomerase;)																					
ABCDEF_45696	UniRef50_P81748	7e-24	Normal: 101	181	97	48	92	1318	BlastAnno: 808	UniRef50_P81748	114	44.35	115	63	1	214	558	1	114	7e-24	100
5.3.3.-(Isomerases;)																					
No Correspond Unigene																					
5.3.3.10(5-carboxymethyl-2-hydroxymuconate Delta-isomerase)																					
No Correspond Unigene																					
5.3.3.12(L-dopachrome isomerase;)																					
No Correspond Unigene																					
5.4.3.6(tyrosine 2,3-aminomutase;)																					
No Correspond Unigene																					
6.2.1.-(Ligases;)																					
ABCDEF_7378	UniRef50_Q9FFE6	0.0	Normal: 1022	1422	746	576	680	1408	BlastAnno: 2117	UniRef50_Q9FFE6	552	67.57	552	167	5	144	1778	1	547	0.0	768
ABCDEF_47513	UniRef50_Q8RU95	0.0	Normal: 411	609	570	233	749	887	BlastAnno: 2280	UniRef50_Q8RU95	598	53.15	572	246	6	119	1777	28	596	0.0	543
ABCDEF_78742	UniRef50_Q84P25	0.0	Normal: 236	207	373	576	450	306	BlastAnno: 1534	UniRef50_Q84P25	565	60.39	462	168	5	1413	49	19	472	0.0	546
ABCDEF_10753	UniRef50_Q9FFE6	0.0	Normal: 394	401	336	253	292	348	BlastAnno: 1841	UniRef50_Q9FFE6	552	51.00	551	259	5	117	1751	1	546	0.0	553
ABCDEF_4431	UniRef50_Q9FFE6	0.0	Normal: 27	358	28	98	112	914	BlastAnno: 2075	UniRef50_Q9FFE6	552	50.71	560	264	7	1774	107	1	552	0.0	551
ABCDEF_38213	UniRef50_Q84P25	0.0	Normal: 342	337	597	798	722	505	BlastAnno: 1939	UniRef50_Q84P25	565	62.34	555	194	5	1818	175	19	565	0.0	690

ABCDEF_25221	UniRef50_Q0DV32	0.0	Normal: 971	826	1003	1543	1442	1545	BlastAnno: 2009	UniRef50_Q0DV32	552	57.20	542	226	4	183	1793	9	549	0.0	613
ABCDEF_23413	UniRef50_Q84P23	0.0	Normal: 123	237	158	149	213	658	BlastAnno: 3175	UniRef50_Q84P23	562	51.74	547	243	8	3030	1435	15	555	0.0	555
ABCDEF_20486	UniRef50_Q9FFE6	0.0	Normal: 580	912	312	324	864	1565	BlastAnno: 3530	UniRef50_Q9FFE6	552	66.61	557	171	5	352	1995	1	551	0.0	781
>00351 DDT degradation																					
1.13.11.-(Oxidoreductases;)																					
No Correspond Unigene																					
1.14.99.-(Oxidoreductases;)																					
ABCDEF_66639	UniRef50_Q0DUI8	0.0	Normal: 646	675	723	1956	1163	1303	BlastAnno: 1867	UniRef50_Q0DUI8	578	85.02	414	61	1	167	1408	158	570	0.0	734
ABCDEF_52814	UniRef50_Q0DUI8	0.0	Normal: 1118	1292	1150	3508	1987	2103	BlastAnno: 2562	UniRef50_Q0DUI8	578	77.22	575	124	4	370	2094	3	570	0.0	909
1.2.-.-(Oxidoreductases;)																					
No Correspond Unigene																					
1.3.-.-(Oxidoreductases;)																					
ABCDEF_37215	UniRef50_Q9VSM5	6e-76	Normal: 470	387	380	308	475	745	BlastAnno: 2204	UniRef50_Q9VSM5	316	44.33	300	155	4	228	1091	4	303	6e-76	255
1.3.1.-(Oxidoreductases;)																					
ABCDEF_45552	UniRef50_P52581	0.0	Normal: 213	2112	262	1058	1208	1322	BlastAnno: 1284	UniRef50_P52581	312	80.45	312	61	0	185	1120	1	312	0.0	530
ABCDEF_47970	UniRef50_Q9T0J6	0.0	Normal: 1040	443	1171	941	1234	650	BlastAnno: 2557	UniRef50_Q9T0J6	700	60.52	699	232	9	2337	271	36	700	0.0	860
1.97.1.-(Oxidoreductases;)																					
No Correspond Unigene																					
3.7.1.-(Hydrolases;)																					
No Correspond Unigene																					
4.1.1.-(Lyases;)																					
ABCDEF_57496	UniRef50_O96569	9e-20	Normal: 4	0	0	0	0	9	BlastAnno: 223	UniRef50_O96569	439	66.67	72	22	1	210	1	19	90	9e-20	87.4
4.5.1.-(Lyases;)																					
No Correspond Unigene																					
4.5.1.1(DDT-dehydrochlorinase;)																					
No Correspond Unigene																					
>00360 Phenylalanine metabolism																					
1.1.1.222(No Name)																					
No Correspond Unigene																					
1.1.1.237(hydroxyphenylpyruvate reductase;)																					
No Correspond Unigene																					
1.1.1.90(aryl-alcohol dehydrogenase;)																					
No Correspond Unigene																					
1.11.1.7(peroxidase;)																					
ABCDEF_25672	UniRef50_Q9SB81	0.0	Normal: 7636	15838	4777	1134	3090	9628	BlastAnno: 1715	UniRef50_Q9SB81	330	85.71	301	43	0	342	1244	30	330	0.0	552
1.13.11.16(3-carboxyethylcatechol 2,3-dioxygenase;)																					
No Correspond Unigene																					
1.13.11.27(4-hydroxyphenylpyruvate dioxygenase;)																					
ABCDEF_7370	UniRef50_P93836	0.0	Normal: 512	1122	253	32	118	3444	BlastAnno: 1725	UniRef50_P93836	445	74.00	423	102	3	1616	363	23	442	0.0	604
1.13.12.9(phenylalanine 2-monooxygenase;)																					
No Correspond Unigene																					
1.14.12.19(3-phenylpropanoate dioxygenase;)																					
No Correspond Unigene																					
1.14.13.-(Oxidoreductases;)																					
ABCDEF_45538	UniRef50_O23024	3e-156	Normal: 110	6	165	774	133	36	BlastAnno: 1258	UniRef50_O23024	437	58.18	385	151	3	3	1157	37	411	3e-156	457
1.14.13.11(trans-cinnamate 4-monooxygenase;)																					
ABCDEF_71482	UniRef50_P92994	0.0	Normal: 2048	1264	3828	9981	3885	695	BlastAnno: 1893	UniRef50_P92994	505	84.49	490	76	0	107	1576	14	503	0.0	797
ABCDEF_41092	UniRef50_P92994	0.0	Normal: 2785	1577	4558	11362	4868	724	BlastAnno: 2059	UniRef50_P92994	505	83.70	503	82	0	1826	318	1	503	0.0	788
ABCDEF_41091	UniRef50_P92994	0.0	Normal: 946	733	2221	5924	1901	1102	BlastAnno: 1557	UniRef50_P92994	505	87.56	442	55	0	1556	231	62	503	0.0	754
ABCDEF_41093	UniRef50_P92994	0.0	Normal: 1164	920	2732	7588	2245	1278	BlastAnno: 1828	UniRef50_P92994	505	86.56	491	66	0	1722	250	14	504	0.0	814
1.14.13.14(trans-cinnamate 2-monooxygenase;)																					
No Correspond Unigene																					
1.14.13.4(melilotate 3-monooxygenase;)																					
No Correspond Unigene																					
1.14.16.1(phenylalanine 4-monooxygenase;)																					
No Correspond Unigene																					
1.17.5.1(phenylacetyl-CoA dehydrogenase;)																					
No Correspond Unigene																					
1.2.1.39(phenylacetaldehyde dehydrogenase)																					
No Correspond Unigene																					
1.2.1.5(aldehyde dehydrogenase [NAD(P)+];)																					
ABCDEF_81997	UniRef50_P43353	5e-53	Normal: 32	81	25	16	59	314	BlastAnno: 770	UniRef50_P43353	468	45.37	205	106	3	746	138	261	461	5e-53	186
1.2.1.58(phenylglyoxylate dehydrogenase (acylating))																					
No Correspond Unigene																					
1.3.1.-(Oxidoreductases;)																					
ABCDEF_45552	UniRef50_P52581	0.0	Normal: 213	2112	262	1058	1208	1322	BlastAnno: 1284	UniRef50_P52581	312	80.45	312	61	0	185	1120	1	312	0.0	530
ABCDEF_47970	UniRef50_Q9T0J6	0.0	Normal: 1040	443	1171	941	1234	650	BlastAnno: 2557	UniRef50_Q9T0J6	700	60.52	699	232	9	2337	271	36	700	0.0	860
1.3.1.11(2-coumarate reductase;)																					
No Correspond Unigene																					
1.3.1.31(2-enoate reductase;)																					
No Correspond Unigene																					
1.4.1.20(phenylalanine dehydrogenase;)																					

No Correspond Unigene																					
1.4.3.2(L-amino-acid oxidase;)																					
ABCDEF_34035	UniRef50_B9R844	0.0	Normal: 973	600	948	1073	764	972	BlastAnno: 3436	UniRef50_B9R844	1947	69.56	611	156	9	1050	2867	1361	1946	0.0	707
1.4.3.21(primary-amine oxidase;)																					
ABCDEF_3522	UniRef50_Q8H1H9	0.0	Normal: 405	723	295	151	922	46	BlastAnno: 2086	UniRef50_Q8H1H9	712	64.54	595	204	6	2	1774	116	707	0.0	811
ABCDEF_3521	UniRef50_Q8H1H9	0.0	Normal: 322	637	263	137	709	53	BlastAnno: 1768	UniRef50_Q8H1H9	712	68.24	510	156	5	1630	113	200	707	0.0	744
1.4.3.4(monoamine oxidase;)																					
ABCDEF_11940	UniRef50_Q8LL67	3e-122	Normal: 41	0	82	2	541	4	BlastAnno: 1557	UniRef50_Q8LL67	496	45.61	410	204	3	2	1183	74	480	3e-122	376
1.4.99.1(D-amino-acid dehydrogenase;)																					
No Correspond Unigene																					
1.4.99.4(aralkylamine dehydrogenase;)																					
No Correspond Unigene																					
1.5.1.18(ephedrine dehydrogenase)																					
No Correspond Unigene																					
2.1.1.-(Transferases;)																					
ABCDEF_20806	UniRef50_Q94II3	0.0	Normal: 700	332	876	1458	1997	929	BlastAnno: 2275	UniRef50_Q94II3	600	73.57	594	153	4	2119	341	1	591	0.0	907
ABCDEF_45802	UniRef50_Q9ZPH9	0.0	Normal: 3543	3348	4296	9797	10830	5017	BlastAnno: 2642	UniRef50_Q9ZPH9	633	53.90	603	261	7	2216	456	23	624	0.0	676
ABCDEF_20070	UniRef50_Q944R7	0.0	Normal: 301	141	265	207	185	187	BlastAnno: 2675	UniRef50_Q944R7	724	58.28	719	275	9	127	2265	13	712	0.0	840
ABCDEF_34956	UniRef50_Q94II3	0.0	Normal: 1120	7269	2475	16033	22219	21461	BlastAnno: 2676	UniRef50_Q94II3	600	73.77	610	146	4	404	2230	1	597	0.0	957
ABCDEF_17121	UniRef50_Q9ZPH9	0.0	Normal: 124	77	109	284	199	132	BlastAnno: 2066	UniRef50_Q9ZPH9	633	53.24	601	259	8	161	1903	25	623	0.0	644
ABCDEF_6524	UniRef50_Q9ZPH9	0.0	Normal: 471	296	490	753	870	730	BlastAnno: 2602	UniRef50_Q9ZPH9	633	50.89	619	290	5	2221	404	6	623	0.0	659
ABCDEF_56017	UniRef50_Q9ZPH9	0.0	Normal: 302	167	286	596	748	529	BlastAnno: 2384	UniRef50_Q9ZPH9	633	51.37	619	287	5	213	2030	6	623	0.0	663
ABCDEF_44169	UniRef50_Q9SIZ3	0.0	Normal: 179	158	273	705	685	229	BlastAnno: 2300	UniRef50_Q9SIZ3	589	62.01	308	108	4	1219	296	285	583	0.0	394
ABCDEF_6521	UniRef50_Q9ZPH9	0.0	Normal: 339	198	314	641	767	582	BlastAnno: 2511	UniRef50_Q9ZPH9	633	51.37	619	287	5	2172	355	6	623	0.0	663
ABCDEF_44172	UniRef50_Q9SIZ3	0.0	Normal: 204	184	287	767	741	242	BlastAnno: 2317	UniRef50_Q9SIZ3	589	68.15	449	134	4	1637	291	144	583	0.0	641
ABCDEF_199	UniRef50_Q9SU94	0.0	Normal: 1076	275	1009	558	1247	306	BlastAnno: 1769	UniRef50_Q9SU94	390	78.45	348	74	1	284	1324	43	390	0.0	584
ABCDEF_52252	UniRef50_Q84W92	0.0	Normal: 1658	720	1653	1980	2289	1601	BlastAnno: 2170	UniRef50_Q84W92	535	72.49	469	120	3	409	1791	34	501	0.0	703
ABCDEF_51404	UniRef50_A3BMN9	0.0	Normal: 864	188	716	337	631	326	BlastAnno: 2192	UniRef50_A3BMN9	620	50.50	604	243	9	2008	251	55	620	0.0	555
ABCDEF_21386	UniRef50_Q94AI4	0.0	Normal: 2326	1250	2220	4294	2920	1983	BlastAnno: 3701	UniRef50_Q94AI4	775	47.27	842	332	16	567	2978	8	775	0.0	577
ABCDEF_35101	UniRef50_Q9MAT5	0.0	Normal: 1440	420	1268	576	1136	850	BlastAnno: 1634	UniRef50_Q9MAT5	383	69.03	352	109	0	1449	394	32	383	0.0	550
ABCDEF_960	UniRef50_Q9LN50	0.0	Normal: 3504	13198	3609	11156	19651	12155	BlastAnno: 2626	UniRef50_Q9LN50	724	49.14	525	246	5	883	2451	219	724	0.0	570
ABCDEF_48082	UniRef50_Q9LN50	0.0	Normal: 410	445	467	870	993	454	BlastAnno: 2340	UniRef50_Q9LN50	724	70.25	521	155	0	710	2272	204	724	0.0	821
ABCDEF_10473	UniRef50_Q08A71	0.0	Normal: 374	187	332	425	456	314	BlastAnno: 1764	UniRef50_Q08A71	435	79.23	366	75	1	344	1438	70	435	0.0	575
ABCDEF_7295	UniRef50_Q9ZPH9	0.0	Normal: 25	54	30	81	26	329	BlastAnno: 1191	UniRef50_Q9ZPH9	633	69.23	403	110	4	1191	22	225	626	0.0	599
ABCDEF_4160	UniRef50_Q94II3	0.0	Normal: 189	0	210	71	17	72	BlastAnno: 1424	UniRef50_Q94II3	600	77.28	449	101	1	1424	81	143	591	0.0	758
ABCDEF_44857	UniRef50_Q9ZPH9	0.0	Normal: 101	224	103	299	588	350	BlastAnno: 1582	UniRef50_Q9ZPH9	633	72.06	519	143	1	1571	21	114	632	0.0	822
ABCDEF_71541	UniRef50_Q9SIZ3	0.0	Normal: 187	167	296	765	724	237	BlastAnno: 2268	UniRef50_Q9SIZ3	589	62.01	308	108	4	1210	287	285	583	0.0	394
ABCDEF_44171	UniRef50_Q9SIZ3	0.0	Normal: 214	196	312	827	802	251	BlastAnno: 2339	UniRef50_Q9SIZ3	589	64.78	548	182	5	1978	335	47	583	0.0	717
ABCDEF_20805	UniRef50_Q94II3	0.0	Normal: 674	344	885	1417	2194	963	BlastAnno: 2400	UniRef50_Q94II3	600	78.21	436	94	1	879	2183	156	591	0.0	734
ABCDEF_18210	UniRef50_Q9SIZ3	0.0	Normal: 5584	3714	7379	17006	21892	7546	BlastAnno: 3199	UniRef50_Q9SIZ3	589	50.00	576	269	7	2145	424	27	585	0.0	574
ABCDEF_6526	UniRef50_Q9ZPH9	0.0	Normal: 627	185	837	2717	2815	1530	BlastAnno: 2611	UniRef50_Q9ZPH9	633	54.76	599	259	5	462	2222	25	623	0.0	689
ABCDEF_6525	UniRef50_Q9ZPH9	0.0	Normal: 602	165	760	2369	2605	1400	BlastAnno: 2545	UniRef50_Q9ZPH9	633	54.92	599	258	5	431	2191	25	623	0.0	689
ABCDEF_6523	UniRef50_Q9ZPH9	0.0	Normal: 314	175	304	641	784	552	BlastAnno: 2312	UniRef50_Q9ZPH9	633	52.42	599	272	4	201	1958	25	623	0.0	663
ABCDEF_67393	UniRef50_Q9ZPH9	0.0	Normal: 434	245	444	608	768	655	BlastAnno: 2346	UniRef50_Q9ZPH9	633	51.92	599	275	4	232	1989	25	623	0.0	659
ABCDEF_3579	UniRef50_O14744	0.0	Normal: 1361	326	984	568	791	440	BlastAnno: 2369	UniRef50_O14744	637	47.00	634	294	10	267	2120	30	637	0.0	553
2.1.1.104(caffeoyl-CoA O-methyltransferase;)																					
ABCDEF_62277	UniRef50_O65862	2e-162	Normal: 19	362	17	166	258	203	BlastAnno: 1005	UniRef50_O65862	247	89.84	246	20	1	103	825	1	246	2e-162	462
ABCDEF_34441	UniRef50_O65862	2e-162	Normal: 22	354	16	164	260	194	BlastAnno: 992	UniRef50_O65862	247	89.84	246	20	1	103	825	1	246	2e-162	462
2.3.1.13(glycine N-acyltransferase;)																					
No Correspond Unigene																					
2.3.1.14(glutamine N-phenylacetyltransferase;)																					
No Correspond Unigene																					
2.3.1.36(D-amino-acid N-acetyltransferase;)																					
No Correspond Unigene																					
2.3.1.53(phenylalanine N-acetyltransferase;)																					
No Correspond Unigene																					
2.3.1.71(glycine N-benzoyltransferase;)																					
No Correspond Unigene																					
2.6.1.-(Transferases;)																					
ABCDEF_3716	UniRef50_Q9LQ10	0.0	Normal: 224	353	140	47	76	606	BlastAnno: 2230	UniRef50_Q9LQ10	557	61.35	533	189	8	250	1812	30	557	0.0	609
ABCDEF_37756	UniRef50_Q9LQ10	0.0	Normal: 197	168	149	152	186	490	BlastAnno: 1840	UniRef50_Q9LQ10	557	57.49	534	196	7	1638	109	28	554	0.0	619
2.6.1.1(aspartate transaminase;)																					
ABCDEF_11690	UniRef50_P46248	0.0	Normal: 1745	1815	2022	4078	5299	6095	BlastAnno: 1778	UniRef50_P46248	453	86.09	417	58	0	297	1547	37	453	0.0	733
ABCDEF_1333	UniRef50_C0PSV4	0.0	Normal: 356	544	636	1119	2495	631	BlastAnno: 1594	UniRef50_C0PSV4	462	76.43	403	95	0	1401	193	60	462	0.0	662
ABCDEF_21786	UniRef50_C0PSV4	0.0	Normal: 268	668	330	546	1267	646	BlastAnno: 1736	UniRef50_C0PSV4	462	74.14	464	113	3	50	1441	4	460	0.0	711
ABCDEF_42262	UniRef50_P46643	0.0	Normal: 916	606	1656	2699	2553	856	BlastAnno: 1722	UniRef50_P46643	430	79.49	429	85	2	1635	355	1	428	0.0	731
ABCDEF_56929	UniRef50_P37833	0.0	Normal: 173	278	312	654	1243	297	BlastAnno: 1151	UniRef50_P37833	407	81.82	308	56	0	978	55	3	310	0.0	536
2.6.1.21(D-amino-acid transaminase;)																					
No Correspond Unigene																					
2.6.1.5(tyrosine transaminase;)																					
ABCDEF_52650	UniRef50_Q9LVY1	0.0	Normal: 493	358	455	302	300	892	BlastAnno: 1442	UniRef50_Q9LVY1	420	61.26	413	160	0	65	1303	5	417	0.0	539
2.6.1.57(aromatic-amino-acid transaminase;)																					

No Correspond Unigene																					
2.6.1.58(phenylalanine(histidine) transaminase;)																					
No Correspond Unigene																					
2.6.1.9(histidinol-phosphate transaminase;)																					
ABCDEF_45962	UniRef50_A7NFV2	2e-99	Normal: 595	558	686	955	1251	1312	BlastAnno: 1635	UniRef50_A7NFV2	371	48.76	363	178	4	336	1403	7	368	2e-99	313
3.1.2.25(phenylacetyl-CoA hydrolase)																					
No Correspond Unigene																					
3.5.1.32(hippurate hydrolase)																					
ABCDEF_24290	UniRef50_B9RKD4	1e-135	Normal: 296	238	403	498	698	330	BlastAnno: 1710	UniRef50_B9RKD4	370	54.68	395	154	6	211	1395	1	370	1e-135	408
3.5.1.4(amidase;)																					
ABCDEF_3151	UniRef50_B9RF45	0.0	Normal: 113	275	72	56	28	1014	BlastAnno: 1850	UniRef50_B9RF45	500	68.47	425	133	1	131	1402	68	492	0.0	547
ABCDEF_81937	UniRef50_B9RF45	0.0	Normal: 101	261	68	53	28	927	BlastAnno: 1739	UniRef50_B9RF45	500	68.47	425	133	1	131	1402	68	492	0.0	546
3.7.1.-(Hydrolases;)																					
No Correspond Unigene																					
4.1.1.-(Lyases;)																					
ABCDEF_57496	UniRef50_O96569	9e-20	Normal: 4	0	0	0	0	9	BlastAnno: 223	UniRef50_O96569	439	66.67	72	22	1	210	1	19	90	9e-20	87.4
4.1.1.28(aromatic-L-amino-acid decarboxylase;)																					
ABCDEF_28052	UniRef50_P54770	3e-145	Normal: 150	74	133	88	177	332	BlastAnno: 1237	UniRef50_P54770	533	55.61	374	141	2	1237	191	144	517	3e-145	433
4.1.1.43(phenylpyruvate decarboxylase;)																					
No Correspond Unigene																					
4.1.1.53(phenylalanine decarboxylase;)																					
No Correspond Unigene																					
4.1.2.41(vanillin synthase;)																					
No Correspond Unigene																					
4.1.3.39(4-hydroxy-2-oxovalerate aldolase;)																					
No Correspond Unigene																					
4.2.1.101(trans-feruloyl-CoA hydratase;)																					
No Correspond Unigene																					
4.2.1.80(2-oxopent-4-enoate hydratase;)																					
No Correspond Unigene																					
4.3.1.24(phenylalanine ammonia-lyase;)																					
ABCDEF_34414	UniRef50_Q6GZ04	0.0	Normal: 1997	3260	3067	14209	6957	2185	BlastAnno: 2627	UniRef50_Q6GZ04	698	47.67	644	318	7	2160	253	48	680	0.0	591
ABCDEF_35119	UniRef50_Q6GZ04	0.0	Normal: 2644	1363	3193	6969	3673	1650	BlastAnno: 2515	UniRef50_Q6GZ04	698	46.71	683	344	7	264	2285	9	680	0.0	597
ABCDEF_21563	UniRef50_C5YCD7	0.0	Normal: 707	225	993	972	1864	182	BlastAnno: 1483	UniRef50_C5YCD7	688	74.23	489	100	1	1482	16	225	687	0.0	758
ABCDEF_59755	UniRef50_Q6GZ04	0.0	Normal: 1915	2750	3275	9429	2466	3052	BlastAnno: 2458	UniRef50_Q6GZ04	698	46.86	685	338	10	2178	163	9	680	0.0	595
ABCDEF_7556	UniRef50_Q6GZ04	0.0	Normal: 2012	2842	3468	9812	2712	3290	BlastAnno: 2512	UniRef50_Q6GZ04	698	46.43	700	349	10	285	2345	9	695	0.0	595
4.3.1.25(phenylalanine/tyrosine ammonia-lyase;)																					
No Correspond Unigene																					
5.1.1.11(phenylalanine racemase (ATP-hydrolysing);)																					
No Correspond Unigene																					
5.3.2.1(phenylpyruvate tautomerase;)																					
ABCDEF_45696	UniRef50_P81748	7e-24	Normal: 101	181	97	48	92	1318	BlastAnno: 808	UniRef50_P81748	114	44.35	115	63	1	214	558	1	114	7e-24	100
6.2.1.12(4-coumarate---CoA ligase;)																					
ABCDEF_52459	UniRef50_Q9LU36	0.0	Normal: 157	704	147	540	677	726	BlastAnno: 1521	UniRef50_Q9LU36	570	65.20	431	139	3	9	1274	136	564	0.0	554
ABCDEF_41628	UniRef50_Q9LU36	0.0	Normal: 140	1561	151	856	1760	1204	BlastAnno: 2088	UniRef50_Q9LU36	570	60.04	548	203	5	152	1753	21	566	0.0	660
6.2.1.30(phenylacetate---CoA ligase;)																					
No Correspond Unigene																					
>00361 Chlorocyclohexane and chlorobenzene degradation																					
1.13.11.1(catechol 1,2-dioxygenase;)																					
No Correspond Unigene																					
1.13.11.2(catechol 2,3-dioxygenase;)																					
No Correspond Unigene																					
1.13.11.37(hydroxyquinol 1,2-dioxygenase;)																					
No Correspond Unigene																					
1.13.11.39(biphenyl-2,3-diol 1,2-dioxygenase;)																					
No Correspond Unigene																					
1.14.-.-(Oxidoreductases;)																					
ABCDEF_44739	UniRef50_O64989	0.0	Normal: 157	87	159	718	673	189	BlastAnno: 2185	UniRef50_O64989	513	70.75	482	112	3	1867	509	30	511	0.0	706
ABCDEF_31491	UniRef50_Q93VK5	0.0	Normal: 647	1826	671	1517	1770	5953	BlastAnno: 2278	UniRef50_Q93VK5	595	79.14	556	113	2	198	1862	35	588	0.0	902
ABCDEF_40554	UniRef50_P37122	0.0	Normal: 1574	1962	1829	1150	2882	2721	BlastAnno: 1828	UniRef50_P37122	505	60.52	504	198	1	1752	244	1	504	0.0	638
ABCDEF_34049	UniRef50_O23365	0.0	Normal: 363	384	349	442	549	1416	BlastAnno: 2101	UniRef50_O23365	580	76.17	579	116	5	230	1957	19	578	0.0	904
ABCDEF_21579	UniRef50_O48928	0.0	Normal: 1005	643	1606	4372	3226	1621	BlastAnno: 1895	UniRef50_O48928	513	69.72	502	151	1	185	1690	7	507	0.0	731
ABCDEF_37741	UniRef50_P37122	0.0	Normal: 12	760	9	24	34	576	BlastAnno: 1197	UniRef50_P37122	505	65.70	379	129	1	1195	59	127	504	0.0	526
ABCDEF_35618	UniRef50_O48928	0.0	Normal: 28	2648	17	142	1234	902	BlastAnno: 1746	UniRef50_O48928	513	66.40	506	168	2	1649	132	4	507	0.0	701
ABCDEF_24522	UniRef50_P37122	0.0	Normal: 334	2190	561	268	1391	2459	BlastAnno: 2075	UniRef50_P37122	505	61.52	460	176	1	1544	165	47	505	0.0	612
ABCDEF_14592	UniRef50_O23066	0.0	Normal: 1514	402	2245	7837	4742	1248	BlastAnno: 2378	UniRef50_O23066	553	72.71	546	140	4	2111	489	1	542	0.0	821
ABCDEF_14512	UniRef50_Q9FG65	0.0	Normal: 261	872	212	291	175	789	BlastAnno: 1878	UniRef50_Q9FG65	502	56.89	501	197	9	1738	266	11	502	0.0	532
ABCDEF_44487	UniRef50_Q940V4	0.0	Normal: 3	41	53	501	262	542	BlastAnno: 1712	UniRef50_Q940V4	465	69.37	382	116	1	38	1180	14	395	0.0	572
ABCDEF_17723	UniRef50_P37122	0.0	Normal: 240	1698	219	768	592	794	BlastAnno: 1757	UniRef50_P37122	505	63.40	459	166	2	228	1601	47	504	0.0	617
ABCDEF_37901	UniRef50_Q42569	0.0	Normal: 30	149	86	255	195	255	BlastAnno: 1246	UniRef50_Q42569	472	78.27	359	73	2	64	1140	43	396	0.0	567
ABCDEF_7260	UniRef50_O81117	0.0	Normal: 314	1085	466	3248	2575	1360	BlastAnno: 1768	UniRef50_O81117	514	59.92	474	188	1	145	1566	43	514	0.0	615
ABCDEF_40548	UniRef50_P37122	0.0	Normal: 2756	2467	2752	1072	3120	1735	BlastAnno: 1947	UniRef50_P37122	505	59.72	504	202	1	241	1749	1	504	0.0	636

ABCDEF_44485	UniRef50_Q940V4	0.0	Normal: 3	55	63	574	338	660	BlastAnno: 1607	UniRef50_Q940V4	465	68.58	452	141	1	42	1394	14	465	0.0	673
ABCDEF_40552	UniRef50_P37122	0.0	Normal: 2397	1870	2304	1249	10428	2291	BlastAnno: 1812	UniRef50_P37122	505	60.12	504	200	1	108	1616	1	504	0.0	640
ABCDEF_37902	UniRef50_Q42569	0.0	Normal: 84	524	254	725	670	1049	BlastAnno: 1812	UniRef50_Q42569	472	77.47	435	92	3	1675	371	43	471	0.0	681
ABCDEF_40556	UniRef50_P37122	0.0	Normal: 1907	2365	2163	1319	3678	3308	BlastAnno: 1944	UniRef50_P37122	505	61.04	498	193	1	264	1754	1	498	0.0	635
ABCDEF_40549	UniRef50_P37122	0.0	Normal: 1574	1962	1826	1150	2882	2723	BlastAnno: 1845	UniRef50_P37122	505	60.52	504	198	1	1754	246	1	504	0.0	638
ABCDEF_20301	UniRef50_O81117	0.0	Normal: 89	308	123	176	389	249	BlastAnno: 1637	UniRef50_O81117	514	64.04	470	164	3	1498	98	47	514	0.0	586
ABCDEF_3882	UniRef50_Q42569	0.0	Normal: 84	243	257	786	1561	182	BlastAnno: 1854	UniRef50_Q42569	472	72.51	422	107	1	1852	587	59	471	0.0	615
ABCDEF_20703	UniRef50_O49858	0.0	Normal: 115	636	20	0	0	597	BlastAnno: 1815	UniRef50_O49858	527	50.10	523	248	6	48	1607	12	524	0.0	533
ABCDEF_18295	UniRef50_O22203	0.0	Normal: 3667	1934	4977	10491	4588	1917	BlastAnno: 2006	UniRef50_O22203	508	74.85	493	123	1	214	1692	17	508	0.0	803
ABCDEF_48724	UniRef50_O23066	0.0	Normal: 104	11518	163	2083	3464	6356	BlastAnno: 2162	UniRef50_O23066	553	72.96	540	143	3	227	1840	1	539	0.0	807
ABCDEF_37738	UniRef50_P37122	0.0	Normal: 26	2447	19	30	33	993	BlastAnno: 1837	UniRef50_P37122	505	64.30	479	169	2	1685	249	28	504	0.0	639
1.14.12.-(Oxidoreductases;)																					
No Correspond Unigene																					
1.14.12.3(benzene 1,2-dioxygenase;)																					
No Correspond Unigene																					
1.14.13.-(Oxidoreductases;)																					
ABCDEF_45538	UniRef50_O23024	3e-156	Normal: 110	6	165	774	133	36	BlastAnno: 1258	UniRef50_O23024	437	58.18	385	151	3	3	1157	37	411	3e-156	457
1.14.13.20(2,4-dichlorophenol 6-monooxygenase;)																					
ABCDEF_86426	UniRef50_A7HPC1	4e-23	Normal: 3	0	2	3	0	12	BlastAnno: 402	UniRef50_A7HPC1	556	48.15	108	53	2	53	373	272	377	4e-23	100
1.14.13.50(pentachlorophenol monooxygenase;)																					
ABCDEF_28561	UniRef50_B5IJ86	7e-19	Normal: 9	16	21	12	0	0	BlastAnno: 223	UniRef50_B5IJ86	563	69.64	56	17	0	218	51	320	375	7e-19	85.9
1.14.13.7(phenol 2-monooxygenase;)																					
No Correspond Unigene																					
1.2.1.61(4-hydroxymuconic-semialdehyde dehydrogenase)																					
No Correspond Unigene																					
1.3.1.-(Oxidoreductases;)																					
ABCDEF_45552	UniRef50_P52581	0.0	Normal: 213	2112	262	1058	1208	1322	BlastAnno: 1284	UniRef50_P52581	312	80.45	312	61	0	185	1120	1	312	0.0	530
ABCDEF_47970	UniRef50_Q9TOJ6	0.0	Normal: 1040	443	1171	941	1234	650	BlastAnno: 2557	UniRef50_Q9TOJ6	700	60.52	699	232	9	2337	271	36	700	0.0	860
1.3.1.19(cis-1,2-dihydrobenzene-1,2-diol dehydrogenase;)																					
No Correspond Unigene																					
1.3.1.32(maleylacetate reductase;)																					
No Correspond Unigene																					
1.6.5.7(2-hydroxy-1,4-benzoquinone reductase;)																					
No Correspond Unigene																					
1.97.1.-(Oxidoreductases;)																					
No Correspond Unigene																					
3.1.1.45(carboxymethylenebutenolidase;)																					
ABCDEF_29224	UniRef50_B9SFS0	3e-139	Normal: 809	476	821	788	1383	993	BlastAnno: 1122	UniRef50_B9SFS0	286	77.42	248	55	1	177	917	39	286	3e-139	407
3.8.1.2(No Name)																					
ABCDEF_24773	UniRef50_A9GEM6	2e-26	Normal: 161	223	191	185	211	772	BlastAnno: 1237	UniRef50_A9GEM6	202	33.67	196	122	4	257	823	5	199	2e-26	111
3.8.1.3(haloacetate dehalogenase;)																					
No Correspond Unigene																					
3.8.1.5(haloalkane dehalogenase;)																					
ABCDEF_21424	UniRef50_Q31PT8	4e-55	Normal: 204	722	162	247	246	1436	BlastAnno: 1354	UniRef50_Q31PT8	298	39.78	274	157	6	943	128	25	292	4e-55	193
4.5.1.-(Lyases;)																					
No Correspond Unigene																					
5.2.1.10(2-chloro-4-carboxymethylenebut-2-en-1,4-olide isomerase;)																					
No Correspond Unigene																					
5.5.1.1(muconate cycloisomerase;)																					
ABCDEF_21833	UniRef50_B9SQ11	6e-167	Normal: 262	353	289	450	785	549	BlastAnno: 1557	UniRef50_B9SQ11	453	64.00	375	133	2	1535	417	75	449	6e-167	489
5.5.1.11(dichloromuconate cycloisomerase)																					
No Correspond Unigene																					
5.5.1.7(chloromuconate cycloisomerase;)																					
ABCDEF_7507	UniRef50_A6Q9H5	8e-08	Normal: 6	6	11	12	0	4	BlastAnno: 234	UniRef50_A6Q9H5	359	42.42	66	37	1	233	39	163	228	8e-08	53.9
>00362 Benzoate degradation																					
1.1.1.-(Oxidoreductases;)																					
ABCDEF_13453	UniRef50_Q9SYM5	0.0	Normal: 225	226	398	1950	2026	421	BlastAnno: 2578	UniRef50_Q9SYM5	669	86.61	672	87	2	298	2313	1	669	0.0	1226
ABCDEF_17342	UniRef50_Q84M96	0.0	Normal: 274	406	272	327	439	625	BlastAnno: 1277	UniRef50_Q84M96	346	73.33	345	91	1	1187	156	2	346	0.0	546
ABCDEF_31147	UniRef50_Q9SYM5	0.0	Normal: 4571	1591	1491	1401	1939	4327	BlastAnno: 2465	UniRef50_Q9SYM5	669	82.76	673	107	4	2397	394	1	669	0.0	1154
ABCDEF_17344	UniRef50_Q84M96	0.0	Normal: 252	373	257	297	380	540	BlastAnno: 1350	UniRef50_Q84M96	346	68.19	371	91	2	1263	154	2	346	0.0	530
ABCDEF_44674	UniRef50_Q9SYM5	0.0	Normal: 3581	2620	4329	9505	11447	4930	BlastAnno: 2634	UniRef50_Q9SYM5	669	87.80	672	78	3	253	2265	1	669	0.0	1241
1.1.1.157(3-hydroxybutyryl-CoA dehydrogenase;)																					
ABCDEF_7588	UniRef50_Q45223	6e-112	Normal: 227	754	413	644	1257	2201	BlastAnno: 1204	UniRef50_Q45223	293	56.14	285	125	0	101	955	3	287	6e-112	338
1.1.1.259(3-hydroxypimeloyl-CoA dehydrogenase)																					
No Correspond Unigene																					
1.13.11.-(Oxidoreductases;)																					
No Correspond Unigene																					
1.13.11.1(catechol 1,2-dioxygenase;)																					
No Correspond Unigene																					
1.13.11.14(2,3-dihydroxybenzoate 3,4-dioxygenase;)																					
No Correspond Unigene																					
1.13.11.2(catechol 2,3-dioxygenase;)																					

No Correspond Unigene

1.13.11.3(protocatechuate 3,4-dioxygenase;)

No Correspond Unigene

1.13.11.37(hydroxyquinol 1,2-dioxygenase;)

No Correspond Unigene

1.13.11.8(protocatechuate 4,5-dioxygenase;)

No Correspond Unigene

1.14.12.10(benzoate 1,2-dioxygenase;)

No Correspond Unigene

1.14.12.14(2-aminobenzenesulfonate 2,3-dioxygenase;)

No Correspond Unigene

1.14.13.12(benzoate 4-monooxygenase;)

No Correspond Unigene

1.14.13.2(4-hydroxybenzoate 3-monooxygenase;)

No Correspond Unigene

1.14.13.23(3-hydroxybenzoate 4-monooxygenase;)

No Correspond Unigene

1.14.13.24(3-hydroxybenzoate 6-monooxygenase;)

No Correspond Unigene

1.14.13.33(4-hydroxybenzoate 3-monooxygenase [NAD(P)H];)

No Correspond Unigene

1.14.13.64(4-hydroxybenzoate 1-hydroxylase;)

No Correspond Unigene

1.14.99.15(4-methoxybenzoate monooxygenase (O-demethylating);)

No Correspond Unigene

1.14.99.23(3-hydroxybenzoate 2-monooxygenase;)

No Correspond Unigene

1.2.1.10(acetaldehyde dehydrogenase (acetylating);)

No Correspond Unigene

1.2.1.32(aminomuconate-semialdehyde dehydrogenase;)

No Correspond Unigene

1.2.1.45(4-carboxy-2-hydroxymuconate-6-semialdehyde dehydrogenase;)

No Correspond Unigene

1.3.1.25(1,6-dihydroxycyclohexa-2,4-diene-1-carboxylate dehydrogenase;)

No Correspond Unigene

1.3.1.32(maleylacetate reductase;)

No Correspond Unigene

1.3.1.62(pimeloyl-CoA dehydrogenase)

No Correspond Unigene

1.3.99.-(Oxidoreductases;)

ABCDEF_4448	UniRef50_Q6JQN1	0.0	Normal: 1778	1683	1752	2720	2409	2803	BlastAnno: 2780	UniRef50_Q6JQN1	1059	48.13	802	354	14	231	2564	293	1056	0.0	718

1.3.99.15(benzoyl-CoA reductase;)

No Correspond Unigene

1.3.99.20(4-hydroxybenzoyl-CoA reductase;)

No Correspond Unigene

1.3.99.7(glutaryl-CoA dehydrogenase;)

ABCDEF_25385	UniRef50_Q22KH3	8e-120	Normal: 747	1014	820	1304	1917	2681	BlastAnno: 1768	UniRef50_Q22KH3	411	45.15	392	213	2	232	1404	17	407	8e-120	369

2.3.1.-(Transferases;)

ABCDEF_520	UniRef50_Q9FF86	0.0	Normal: 1672	85	2273	5056	3383	1211	BlastAnno: 1811	UniRef50_Q9FF86	484	64.06	448	149	6	1664	354	3	449	0.0	573
ABCDEF_68664	UniRef50_Q9ZVN2	0.0	Normal: 79	35	85	107	193	63	BlastAnno: 1488	UniRef50_Q9ZVN2	704	63.99	411	142	3	46	1275	298	703	0.0	540
ABCDEF_52245	UniRef50_Q9FG87	0.0	Normal: 1878	635	1833	3814	1432	1376	BlastAnno: 1758	UniRef50_Q9FG87	529	55.35	486	204	4	4	1428	34	517	0.0	530
ABCDEF_16730	UniRef50_Q3EC11	0.0	Normal: 281	204	237	365	377	458	BlastAnno: 2092	UniRef50_Q3EC11	536	59.33	536	204	8	196	1785	3	530	0.0	661
ABCDEF_4655	UniRef50_A5BMG1	0.0	Normal: 2426	1856	3606	6958	4608	2755	BlastAnno: 2261	UniRef50_A5BMG1	481	68.37	528	112	10	263	1843	3	476	0.0	667
ABCDEF_13860	UniRef50_Q9C533	0.0	Normal: 588	220	570	1451	1338	310	BlastAnno: 2648	UniRef50_Q9C533	596	63.37	606	177	12	2226	511	1	595	0.0	558
ABCDEF_28294	UniRef50_Q9SYZ0	0.0	Normal: 1038	4070	1427	6745	877	2797	BlastAnno: 1785	UniRef50_Q9SYZ0	493	52.37	485	223	3	1598	162	5	487	0.0	533
ABCDEF_47718	UniRef50_Q9C533	0.0	Normal: 1599	236	1657	2093	1231	588	BlastAnno: 2680	UniRef50_Q9C533	596	65.02	626	165	14	2280	472	1	595	0.0	634
ABCDEF_45080	UniRef50_Q9SYZ0	0.0	Normal: 1005	592	1079	2360	3762	2217	BlastAnno: 2130	UniRef50_Q9SYZ0	493	57.85	484	197	3	302	1735	5	487	0.0	533
ABCDEF_25377	UniRef50_Q9C6L5	0.0	Normal: 37	2090	138	2573	1026	1298	BlastAnno: 1334	UniRef50_Q9C6L5	492	79.74	385	76	1	1155	1	6	388	0.0	654
ABCDEF_33470	UniRef50_Q9LW26	0.0	Normal: 172	98	156	260	391	149	BlastAnno: 2993	UniRef50_Q9LW26	701	48.96	627	301	5	655	2508	85	701	0.0	581
ABCDEF_40654	UniRef50_Q9H0A0	0.0	Normal: 638	213	530	318	318	286	BlastAnno: 2216	UniRef50_Q9H0A0	1025	56.87	721	279	6	2127	13	91	795	0.0	756
ABCDEF_33460	UniRef50_Q9ZVN2	0.0	Normal: 144	71	119	216	306	97	BlastAnno: 2691	UniRef50_Q9ZVN2	704	61.50	587	191	4	379	2133	83	636	0.0	715
ABCDEF_14442	UniRef50_Q9SYZ0	0.0	Normal: 341	684	439	1040	1601	552	BlastAnno: 1779	UniRef50_Q9SYZ0	493	63.12	480	171	2	1570	149	5	484	0.0	628
ABCDEF_52626	UniRef50_B6SYP9	0.0	Normal: 815	346	1131	3015	1537	108	BlastAnno: 1861	UniRef50_B6SYP9	494	55.02	438	189	3	1538	249	51	488	0.0	536
ABCDEF_644	UniRef50_Q9SB58	0.0	Normal: 704	444	913	969	1431	848	BlastAnno: 1777	UniRef50_Q9SB58	407	64.15	424	129	8	1572	316	1	406	0.0	554
ABCDEF_27087	UniRef50_Q52T38	0.0	Normal: 324	221	233	419	343	412	BlastAnno: 1631	UniRef50_Q52T38	620	75.95	420	91	1	1409	150	1	410	0.0	681
ABCDEF_34187	UniRef50_Q8L5Y5	0.0	Normal: 497	415	588	523	291	433	BlastAnno: 2677	UniRef50_Q8L5Y5	718	57.30	733	251	15	202	2274	1	713	0.0	683
ABCDEF_47966	UniRef50_Q9SYZ0	0.0	Normal: 577	934	326	580	682	3440	BlastAnno: 2177	UniRef50_Q9SYZ0	493	56.40	484	204	3	1780	347	5	487	0.0	535
ABCDEF_51189	UniRef50_Q52T38	0.0	Normal: 135	152	128	181	219	327	BlastAnno: 1766	UniRef50_Q52T38	620	68.09	470	137	3	80	1483	130	588	0.0	641
ABCDEF_41288	UniRef50_Q9SB58	0.0	Normal: 307	183	332	637	649	322	BlastAnno: 1403	UniRef50_Q9SB58	407	67.56	410	110	6	1401	184	15	405	0.0	523
ABCDEF_33461	UniRef50_Q9ZVN2	0.0	Normal: 142	84	140	206	283	119	BlastAnno: 2904	UniRef50_Q9ZVN2	704	60.98	574	201	6	2271	559	83	636	0.0	686
ABCDEF_14334	UniRef50_Q9SYZ0	0.0	Normal: 2263	129	1724	3469	866	283	BlastAnno: 2039	UniRef50_Q9SYZ0	493	63.22	484	171	2	369	1802	5	487	0.0	627
ABCDEF_24844	UniRef50_Q9FF86	0.0	Normal: 1857	3686	2657	21940	11467	4090	BlastAnno: 1640	UniRef50_Q9FF86	484	66.89	447	135	6	1492	185	2	446	0.0	575

ABCDEF_51190	UniRef50_Q52T38	0.0	Normal: 145	169	142	210	232	335	BlastAnno: 1891	UniRef50_Q52T38	620	70.04	514	141	3	73	1608	86	588	0.0	726
ABCDEF_14085	UniRef50_Q8L5Y5	0.0	Normal: 353	41	384	527	331	280	BlastAnno: 2362	UniRef50_Q8L5Y5	718	62.07	704	220	12	2360	348	24	713	0.0	676
ABCDEF_33458	UniRef50_Q9LW26	0.0	Normal: 158	78	130	240	342	106	BlastAnno: 2822	UniRef50_Q9LW26	701	46.72	655	302	5	400	2337	85	701	0.0	568
ABCDEF_25193	UniRef50_Q9C6L5	0.0	Normal: 3256	368	3548	6230	5109	1288	BlastAnno: 1786	UniRef50_Q9C6L5	492	65.83	480	156	4	1668	241	17	492	0.0	668
ABCDEF_33457	UniRef50_Q9ZVN2	0.0	Normal: 168	83	137	250	375	112	BlastAnno: 2823	UniRef50_Q9ZVN2	704	65.12	496	168	3	379	1860	83	575	0.0	648
ABCDEF_7634	UniRef50_Q9C6L5	0.0	Normal: 2543	9303	2711	11578	5533	10032	BlastAnno: 2064	UniRef50_Q9C6L5	492	52.76	489	225	3	1734	268	1	483	0.0	535
ABCDEF_299	UniRef50_Q71N54	0.0	Normal: 569	1147	511	900	649	1879	BlastAnno: 2017	UniRef50_Q71N54	535	61.73	533	198	5	338	1924	4	534	0.0	677
ABCDEF_27086	UniRef50_Q52T38	0.0	Normal: 784	470	617	843	893	976	BlastAnno: 2398	UniRef50_Q52T38	620	70.80	637	165	4	225	2129	1	618	0.0	887
ABCDEF_51188	UniRef50_Q52T38	0.0	Normal: 273	327	255	415	349	441	BlastAnno: 2324	UniRef50_Q52T38	620	71.30	568	150	3	2041	344	32	588	0.0	815
ABCDEF_44812	UniRef50_Q6DR03	0.0	Normal: 982	902	1005	1071	959	2999	BlastAnno: 2450	UniRef50_Q6DR03	565	54.97	644	198	17	2142	220	1	555	0.0	540
2.3.1.16(acetyl-CoA C-acyltransferase;)																					
ABCDEF_47228	UniRef50_P09110	1e-153	Normal: 321	497	306	408	1144	369	BlastAnno: 1559	UniRef50_P09110	424	59.95	387	152	3	1397	237	37	420	1e-153	454
2.3.1.174(3-oxoadipyl-CoA thiolase)																					
No Correspond Unigene																					
2.3.1.9(acetyl-CoA C-acetyltransferase;)																					
ABCDEF_34825	UniRef50_Q8S4Y1	0.0	Normal: 640	698	620	807	839	1164	BlastAnno: 1773	UniRef50_Q8S4Y1	403	78.11	402	87	1	1597	395	2	403	0.0	598
ABCDEF_14629	UniRef50_Q8S4Y1	0.0	Normal: 634	316	650	1021	1278	691	BlastAnno: 1514	UniRef50_Q8S4Y1	403	85.11	403	60	0	1367	159	1	403	0.0	620
ABCDEF_107	UniRef50_Q8S4Y1	0.0	Normal: 332	460	460	842	893	424	BlastAnno: 1214	UniRef50_Q8S4Y1	403	84.07	339	54	0	1212	196	42	380	0.0	596
ABCDEF_14630	UniRef50_Q8S4Y1	0.0	Normal: 562	282	568	959	1173	627	BlastAnno: 1796	UniRef50_Q8S4Y1	403	85.50	400	58	0	436	1635	4	403	0.0	642
2.8.3.6(3-oxoadipate CoA-transferase;)																					
No Correspond Unigene																					
3.1.1.24(3-oxoadipate enol-lactonase;)																					
No Correspond Unigene																					
3.1.1.57(2-pyrone-4,6-dicarboxylate lactonase)																					
No Correspond Unigene																					
3.1.2.-(Hydrolases;)																					
ABCDEF_2988	UniRef50_Q39513	0.0	Normal: 684	868	676	1224	1298	1392	BlastAnno: 1728	UniRef50_Q39513	415	69.13	392	111	5	408	1574	12	396	0.0	554
ABCDEF_42638	UniRef50_Q9SHJ8	0.0	Normal: 183	172	331	565	390	214	BlastAnno: 1547	UniRef50_Q9SHJ8	395	68.62	376	94	2	196	1320	42	394	0.0	533
ABCDEF_56804	UniRef50_Q9SHJ8	0.0	Normal: 160	146	249	472	328	155	BlastAnno: 1439	UniRef50_Q9SHJ8	395	68.62	376	94	2	275	1399	42	394	0.0	533
ABCDEF_2987	UniRef50_Q39513	0.0	Normal: 608	561	472	1162	1435	954	BlastAnno: 1938	UniRef50_Q39513	415	67.26	394	119	5	408	1580	12	398	0.0	544
ABCDEF_20456	UniRef50_Q9LK08	0.0	Normal: 375	366	460	796	1014	454	BlastAnno: 1539	UniRef50_Q9LK08	418	64.52	420	140	3	1434	187	4	418	0.0	559
ABCDEF_49725	UniRef50_Q9SHJ8	0.0	Normal: 176	174	327	582	397	195	BlastAnno: 1556	UniRef50_Q9SHJ8	395	68.62	376	94	2	234	1358	42	394	0.0	533
ABCDEF_20453	UniRef50_Q9LK08	0.0	Normal: 396	358	461	824	1019	461	BlastAnno: 1565	UniRef50_Q9LK08	418	66.90	420	134	2	1459	200	4	418	0.0	586
3.1.2.23(4-hydroxybenzoyl-CoA thioesterase)																					
No Correspond Unigene																					
3.7.1.-(Hydrolases;)																					
No Correspond Unigene																					
3.7.1.9(2-hydroxymuconate-semialdehyde hydrolase;)																					
No Correspond Unigene																					
4.1.1.-(Lyases;)																					
ABCDEF_57496	UniRef50_O96569	9e-20	Normal: 4	0	0	0	0	9	BlastAnno: 223	UniRef50_O96569	439	66.67	72	22	1	210	1	19	90	9e-20	87.4
4.1.1.44(4-carboxymuconolactone decarboxylase;)																					
No Correspond Unigene																					
4.1.1.46(o-pyrocatechuate decarboxylase;)																					
No Correspond Unigene																					
4.1.1.63(protocatechuate decarboxylase;)																					
No Correspond Unigene																					
4.1.1.70(glutaconyl-CoA decarboxylase;)																					
ABCDEF_8712	UniRef50_B6KHR8	3e-13	Normal: 16	0	5	0	35	34	BlastAnno: 338	UniRef50_B6KHR8	600	63.27	49	18	0	116	262	250	298	3e-13	71.6
4.1.1.77(4-oxalocrotonate decarboxylase;)																					
No Correspond Unigene																					
4.1.3.17(4-hydroxy-4-methyl-2-oxoglutarate aldolase;)																					
No Correspond Unigene																					
4.1.3.39(4-hydroxy-2-oxovalerate aldolase;)																					
No Correspond Unigene																					
4.2.1.-(Lyases;)																					
ABCDEF_48679	UniRef50_Q0RP29	5e-115	Normal: 568	325	563	941	1285	676	BlastAnno: 1424	UniRef50_Q0RP29	493	56.39	321	129	4	119	1075	39	350	5e-115	356
4.2.1.100(cyclohexa-1,5-dienecarbonyl-CoA hydratase;)																					
No Correspond Unigene																					
4.2.1.17(enoyl-CoA hydratase;)																					
ABCDEF_21259	UniRef50_Q8W1L6	0.0	Normal: 272	276	284	384	326	305	BlastAnno: 2641	UniRef50_Q8W1L6	726	47.57	721	375	3	114	2267	6	726	0.0	633
ABCDEF_80649	UniRef50_Q8W1L6	0.0	Normal: 527	841	443	1308	1321	1188	BlastAnno: 2586	UniRef50_Q8W1L6	726	60.39	712	281	1	2403	268	1	711	0.0	818
ABCDEF_466	UniRef50_Q8W1L6	0.0	Normal: 1014	1690	1094	1677	2438	2080	BlastAnno: 2453	UniRef50_Q8W1L6	726	71.24	699	201	0	2368	272	7	705	0.0	954
ABCDEF_54834	UniRef50_Q8W1L6	0.0	Normal: 229	220	237	298	270	261	BlastAnno: 2405	UniRef50_Q8W1L6	726	47.52	606	315	3	274	2082	121	726	0.0	580
ABCDEF_34756	UniRef50_Q8W1L6	0.0	Normal: 776	726	768	2922	4039	1079	BlastAnno: 2469	UniRef50_Q8W1L6	726	68.93	708	217	2	2396	273	7	711	0.0	957
ABCDEF_4198	UniRef50_Q8W1L6	0.0	Normal: 609	929	532	1401	1568	1297	BlastAnno: 2667	UniRef50_Q8W1L6	726	60.45	713	281	1	2463	325	1	712	0.0	820
4.2.1.80(2-oxopent-4-enoate hydratase;)																					
No Correspond Unigene																					
4.2.1.83(4-oxalmesaconate hydratase;)																					
No Correspond Unigene																					
5.3.2.-(Isomerases;)																					
No Correspond Unigene																					

5.3.3.10(5-carboxymethyl-2-hydroxymuconate Delta-isomerase)
No Correspond Unigene
5.3.3.4(muconolactone Delta-isomerase;)
No Correspond Unigene
5.5.1.1(muconate cycloisomerase;)

ABCDEF_21833	UniRef50_B9SQ11	6e-167	Normal: 262	353	289	450	785	549	BlastAnno: 1557	UniRef50_B9SQ11	453	64.00	375	133	2	1535	417	75	449	6e-167	489

5.5.1.2(3-carboxy-cis,cis-muconate cycloisomerase;)
No Correspond Unigene
5.5.1.5(carboxy-cis,cis-muconate cyclase;)
No Correspond Unigene
6.2.1.-(Ligases;)

ABCDEF_7378	UniRef50_Q9FFE6	0.0	Normal: 1022	1422	746	576	680	1408	BlastAnno: 2117	UniRef50_Q9FFE6	552	67.57	552	167	5	144	1778	1	547	0.0	768
ABCDEF_47513	UniRef50_Q8RU95	0.0	Normal: 411	609	570	233	749	887	BlastAnno: 2280	UniRef50_Q8RU95	598	53.15	572	246	6	119	1777	28	596	0.0	543
ABCDEF_78742	UniRef50_Q84P25	0.0	Normal: 236	207	373	576	450	306	BlastAnno: 1534	UniRef50_Q84P25	565	60.39	462	168	5	1413	49	19	472	0.0	546
ABCDEF_10753	UniRef50_Q9FFE6	0.0	Normal: 394	401	336	253	292	348	BlastAnno: 1841	UniRef50_Q9FFE6	552	51.00	551	259	5	117	1751	1	546	0.0	553
ABCDEF_4431	UniRef50_Q9FFE6	0.0	Normal: 27	358	28	98	112	914	BlastAnno: 2075	UniRef50_Q9FFE6	552	50.71	560	264	7	1774	107	1	552	0.0	551
ABCDEF_38213	UniRef50_Q84P25	0.0	Normal: 342	337	597	798	722	505	BlastAnno: 1939	UniRef50_Q84P25	565	62.34	555	194	5	1818	175	19	565	0.0	690
ABCDEF_25221	UniRef50_Q0DV32	0.0	Normal: 971	826	1003	1543	1442	1545	BlastAnno: 2009	UniRef50_Q0DV32	552	57.20	542	226	4	183	1793	9	549	0.0	613
ABCDEF_23413	UniRef50_Q84P23	0.0	Normal: 123	237	158	149	213	658	BlastAnno: 3175	UniRef50_Q84P23	562	51.74	547	243	8	3030	1435	15	555	0.0	555
ABCDEF_20486	UniRef50_Q9FFE6	0.0	Normal: 580	912	312	324	864	1565	BlastAnno: 3530	UniRef50_Q9FFE6	552	66.61	557	171	5	352	1995	1	551	0.0	781

6.2.1.25(benzoate---CoA ligase;)

ABCDEF_7378	UniRef50_Q9SS01	0.0	Normal: 1022	1422	746	576	680	1408	BlastAnno: 2117	UniRef50_Q9SS01	580	50.09	541	250	7	198	1769	19	556	0.0	555
ABCDEF_10753	UniRef50_Q9SS01	0.0	Normal: 394	401	336	253	292	348	BlastAnno: 1841	UniRef50_Q9SS01	580	48.93	562	262	6	117	1745	1	556	0.0	542
ABCDEF_4431	UniRef50_Q9SS01	0.0	Normal: 27	358	28	98	112	914	BlastAnno: 2075	UniRef50_Q9SS01	580	48.14	565	266	6	1774	134	1	556	0.0	539
ABCDEF_20486	UniRef50_Q9SS01	0.0	Normal: 580	912	312	324	864	1565	BlastAnno: 3530	UniRef50_Q9SS01	580	48.92	558	266	6	352	1974	1	556	0.0	556

6.2.1.27(4-hydroxybenzoate---CoA ligase;)
No Correspond Unigene

>00363 Bisphenol degradation

1.1.-.-(Oxidoreductases;)

ABCDEF_17646	UniRef50_O34948	5e-83	Normal: 372	339	320	294	487	1376	BlastAnno: 1685	UniRef50_O34948	288	47.31	279	145	2	1523	690	5	282	5e-83	269

1.1.1.-(Oxidoreductases;)

ABCDEF_13453	UniRef50_Q9SYM5	0.0	Normal: 225	226	398	1950	2026	421	BlastAnno: 2578	UniRef50_Q9SYM5	669	86.61	672	87	2	298	2313	1	669	0.0	1226
ABCDEF_17342	UniRef50_Q84M96	0.0	Normal: 274	406	272	327	439	625	BlastAnno: 1277	UniRef50_Q84M96	346	73.33	345	91	1	1187	156	2	346	0.0	546
ABCDEF_31147	UniRef50_Q9SYM5	0.0	Normal: 4571	1591	1491	1401	1939	4327	BlastAnno: 2465	UniRef50_Q9SYM5	669	82.76	673	107	4	2397	394	1	669	0.0	1154
ABCDEF_17344	UniRef50_Q84M96	0.0	Normal: 252	373	257	297	380	540	BlastAnno: 1350	UniRef50_Q84M96	346	68.19	371	91	2	1263	154	2	346	0.0	530
ABCDEF_44674	UniRef50_Q9SYM5	0.0	Normal: 3581	2620	4329	9505	11447	4930	BlastAnno: 2634	UniRef50_Q9SYM5	669	87.80	672	78	3	253	2265	1	669	0.0	1241

1.13.-.-(Oxidoreductases;)

ABCDEF_51946	UniRef50_Q949R4	2e-119	Normal: 133	259	66	108	237	1420	BlastAnno: 1085	UniRef50_Q949R4	269	66.93	254	78	3	1012	266	4	256	2e-119	355

1.13.11.41(2,4'-dihydroxyacetophenone dioxygenase;)
No Correspond Unigene
1.14.-.-(Oxidoreductases;)

ABCDEF_44739	UniRef50_O64989	0.0	Normal: 157	87	159	718	673	189	BlastAnno: 2185	UniRef50_O64989	513	70.75	482	112	3	1867	509	30	511	0.0	706
ABCDEF_31491	UniRef50_Q93VK5	0.0	Normal: 647	1826	671	1517	1770	5953	BlastAnno: 2278	UniRef50_Q93VK5	595	79.14	556	113	2	198	1862	35	588	0.0	902
ABCDEF_40554	UniRef50_P37122	0.0	Normal: 1574	1962	1829	1150	2882	2721	BlastAnno: 1828	UniRef50_P37122	505	60.52	504	198	1	1752	244	1	504	0.0	638
ABCDEF_34049	UniRef50_O23365	0.0	Normal: 363	384	349	442	549	1416	BlastAnno: 2101	UniRef50_O23365	580	76.17	579	116	5	230	1957	19	578	0.0	904
ABCDEF_21579	UniRef50_O48928	0.0	Normal: 1005	643	1606	4372	3226	1621	BlastAnno: 1895	UniRef50_O48928	513	69.72	502	151	1	185	1690	7	507	0.0	731
ABCDEF_37741	UniRef50_P37122	0.0	Normal: 12	760	9	24	34	576	BlastAnno: 1197	UniRef50_P37122	505	65.70	379	129	1	1195	59	127	504	0.0	526
ABCDEF_35618	UniRef50_O48928	0.0	Normal: 28	2648	17	142	1234	902	BlastAnno: 1746	UniRef50_O48928	513	66.40	506	168	2	1649	132	4	507	0.0	701
ABCDEF_24522	UniRef50_P37122	0.0	Normal: 334	2190	561	268	1391	2459	BlastAnno: 2075	UniRef50_P37122	505	61.52	460	176	1	1544	165	47	505	0.0	612
ABCDEF_14592	UniRef50_O23066	0.0	Normal: 1514	402	2245	7837	4742	1248	BlastAnno: 2378	UniRef50_O23066	553	72.71	546	140	4	2111	489	1	542	0.0	821
ABCDEF_14512	UniRef50_Q9FG65	0.0	Normal: 261	872	212	291	175	789	BlastAnno: 1878	UniRef50_Q9FG65	502	56.89	501	197	9	1738	266	11	502	0.0	532
ABCDEF_44487	UniRef50_Q940V4	0.0	Normal: 3	41	53	501	262	542	BlastAnno: 1712	UniRef50_Q940V4	465	69.37	382	116	1	38	1180	14	395	0.0	572
ABCDEF_17723	UniRef50_P37122	0.0	Normal: 240	1698	219	768	592	794	BlastAnno: 1757	UniRef50_P37122	505	63.40	459	166	2	228	1601	47	504	0.0	617
ABCDEF_37901	UniRef50_Q42569	0.0	Normal: 30	149	86	255	195	255	BlastAnno: 1246	UniRef50_Q42569	472	78.27	359	73	2	64	1140	43	396	0.0	567
ABCDEF_7260	UniRef50_O81117	0.0	Normal: 314	1085	466	3248	2575	1360	BlastAnno: 1768	UniRef50_O81117	514	59.92	474	188	1	145	1566	43	514	0.0	615
ABCDEF_40548	UniRef50_P37122	0.0	Normal: 2756	2467	2752	1072	3120	1735	BlastAnno: 1947	UniRef50_P37122	505	59.72	504	202	1	241	1749	1	504	0.0	636
ABCDEF_44485	UniRef50_Q940V4	0.0	Normal: 3	55	63	574	338	660	BlastAnno: 1607	UniRef50_Q940V4	465	68.58	452	141	1	42	1394	14	465	0.0	673
ABCDEF_40552	UniRef50_P37122	0.0	Normal: 2397	1870	2304	1249	10428	2291	BlastAnno: 1812	UniRef50_P37122	505	60.12	504	200	1	108	1616	1	504	0.0	640
ABCDEF_37902	UniRef50_Q42569	0.0	Normal: 84	524	254	725	670	1049	BlastAnno: 1812	UniRef50_Q42569	472	77.47	435	92	3	1675	371	43	471	0.0	681
ABCDEF_40556	UniRef50_P37122	0.0	Normal: 1907	2365	2163	1319	3678	3308	BlastAnno: 1944	UniRef50_P37122	505	61.04	498	193	1	264	1754	1	498	0.0	635
ABCDEF_40549	UniRef50_P37122	0.0	Normal: 1574	1962	1826	1150	2882	2723	BlastAnno: 1845	UniRef50_P37122	505	60.52	504	198	1	1754	246	1	504	0.0	638
ABCDEF_20301	UniRef50_O81117	0.0	Normal: 89	308	123	176	389	249	BlastAnno: 1637	UniRef50_O81117	514	64.04	470	164	3	1498	98	47	514	0.0	586
ABCDEF_3882	UniRef50_Q42569	0.0	Normal: 84	243	257	786	1561	182	BlastAnno: 1854	UniRef50_Q42569	472	72.51	422	107	1	1852	587	59	471	0.0	615
ABCDEF_20703	UniRef50_O49858	0.0	Normal: 115	636	20	0	0	597	BlastAnno: 1815	UniRef50_O49858	527	50.10	523	248	6	48	1607	12	524	0.0	533
ABCDEF_18295	UniRef50_O22203	0.0	Normal: 3667	1934	4977	10491	4588	1917	BlastAnno: 2006	UniRef50_O22203	508	74.85	493	123	1	214	1692	17	508	0.0	803
ABCDEF_48724	UniRef50_O23066	0.0	Normal: 104	11518	163	2083	3464	6356	BlastAnno: 2162	UniRef50_O23066	553	72.96	540	143	3	227	1840	1	539	0.0	807
ABCDEF_37738	UniRef50_P37122	0.0	Normal: 26	2447	19	30	33	993	BlastAnno: 1837	UniRef50_P37122	505	64.30	479	169	2	1685	249	28	504	0.0	639

1.14.13.-(Oxidoreductases;)

ABCDEF_45538	UniRef50_O23024	3e-156	Normal: 110	6	165	774	133	36	BlastAnno: 1258	UniRef50_O23024	437	58.18	385	151	3	3	1157	37	411	3e-156	457

1.14.13.84(4-hydroxyacetophenone monooxygenase;)
No Correspond Unigene
1.3.-.-(Oxidoreductases;)

	ABCDEF_37215	UniRef50_Q9VSM5	6e-76	Normal: 470	387	380	308	475	745	BlastAnno: 2204	UniRef50_Q9VSM5	316	44.33	300	155	4	228	1091	4	303	6e-76	255
1.97.1.-(Oxidoreductases;)																						
	No Correspond Unigene																					
3.1.1.-(Hydrolases;)																						
	ABCDEF_25737	UniRef50_Q9CA68	0.0	Normal: 104	11	8	393	426	18	BlastAnno: 1232	UniRef50_Q9CA68	366	69.89	352	103	2	1182	130	17	366	0.0	522
	ABCDEF_525	UniRef50_Q9LIN2	0.0	Normal: 213	16	167	378	456	158	BlastAnno: 1254	UniRef50_Q9LIN2	380	69.27	358	110	0	1097	24	23	380	0.0	535
	ABCDEF_28091	UniRef50_O23522	0.0	Normal: 1192	678	424	81	156	3170	BlastAnno: 1887	UniRef50_O23522	601	57.90	525	191	12	168	1721	3	504	0.0	568
	ABCDEF_13432	UniRef50_Q3ECP6	0.0	Normal: 712	1725	466	588	811	6988	BlastAnno: 2479	UniRef50_Q3ECP6	408	66.83	401	104	2	2397	1282	8	408	0.0	567
	ABCDEF_72624	UniRef50_Q3ECP6	0.0	Normal: 22	38	25	267	382	149	BlastAnno: 1342	UniRef50_Q3ECP6	408	66.83	401	104	2	53	1168	8	408	0.0	567
	ABCDEF_4432	UniRef50_B9IQA0	0.0	Normal: 680	298	798	834	1447	644	BlastAnno: 1533	UniRef50_B9IQA0	354	78.82	321	68	0	214	1176	33	353	0.0	556
3.1.1.2(arylesterase;)																						
	No Correspond Unigene																					
4.2.1.-(Lyases;)																						
	ABCDEF_48679	UniRef50_Q0RP29	5e-115	Normal: 568	325	563	941	1285	676	BlastAnno: 1424	UniRef50_Q0RP29	493	56.39	321	129	4	119	1075	39	350	5e-115	356
>00364 Fluorobenzoate degradation																						
1.13.11.1(catechol 1,2-dioxygenase;)																						
	No Correspond Unigene																					
1.14.12.-(Oxidoreductases;)																						
	No Correspond Unigene																					
1.14.12.10(benzoate 1,2-dioxygenase;)																						
	No Correspond Unigene																					
1.14.12.13(2-chlorobenzoate 1,2-dioxygenase;)																						
	No Correspond Unigene																					
1.14.13.50(pentachlorophenol monooxygenase;)																						
	ABCDEF_28561	UniRef50_B5IJ86	7e-19	Normal: 9	16	21	12	0	0	BlastAnno: 223	UniRef50_B5IJ86	563	69.64	56	17	0	218	51	320	375	7e-19	85.9
1.3.1.-(Oxidoreductases;)																						
	ABCDEF_45552	UniRef50_P52581	0.0	Normal: 213	2112	262	1058	1208	1322	BlastAnno: 1284	UniRef50_P52581	312	80.45	312	61	0	185	1120	1	312	0.0	530
	ABCDEF_47970	UniRef50_Q9T0J6	0.0	Normal: 1040	443	1171	941	1234	650	BlastAnno: 2557	UniRef50_Q9T0J6	700	60.52	699	232	9	2337	271	36	700	0.0	860
1.3.1.25(1,6-dihydroxycyclohexa-2,4-diene-1-carboxylate dehydrogenase;)																						
	No Correspond Unigene																					
1.3.1.32(maleylacetate reductase;)																						
	No Correspond Unigene																					
1.3.1.63(2,4-dichlorobenzoyl-CoA reductase)																						
	No Correspond Unigene																					
3.1.1.45(carboxymethylenebutenolidase;)																						
	ABCDEF_29224	UniRef50_B9SFS0	3e-139	Normal: 809	476	821	788	1383	993	BlastAnno: 1122	UniRef50_B9SFS0	286	77.42	248	55	1	177	917	39	286	3e-139	407
3.5.1.-(Hydrolases;)																						
	ABCDEF_41579	UniRef50_O81641	0.0	Normal: 384	122	265	320	313	150	BlastAnno: 1456	UniRef50_O81641	428	65.16	399	138	1	1313	120	23	421	0.0	543
	ABCDEF_16924	UniRef50_Q8VYX0	0.0	Normal: 135	349	460	1325	685	527	BlastAnno: 2002	UniRef50_Q8VYX0	464	71.72	396	109	2	1612	428	68	461	0.0	549
	ABCDEF_24290	UniRef50_O04373	0.0	Normal: 296	238	403	498	698	330	BlastAnno: 1710	UniRef50_O04373	440	65.24	443	151	3	67	1395	1	440	0.0	595
3.5.5.6(bromoxynil nitrilase)																						
	No Correspond Unigene																					
3.8.1.7(4-chlorobenzoyl-CoA dehalogenase)																						
	No Correspond Unigene																					
4.2.1.84(nitrile hydratase;)																						
	No Correspond Unigene																					
5.5.1.1(muconate cycloisomerase;)																						
	ABCDEF_21833	UniRef50_B9SQ11	6e-167	Normal: 262	353	289	450	785	549	BlastAnno: 1557	UniRef50_B9SQ11	453	64.00	375	133	2	1535	417	75	449	6e-167	489
5.5.1.7(chloromuconate cycloisomerase;)																						
	ABCDEF_7507	UniRef50_A6Q9H5	8e-08	Normal: 6	6	11	12	0	4	BlastAnno: 234	UniRef50_A6Q9H5	359	42.42	66	37	1	233	39	163	228	8e-08	53.9
6.2.1.-(Ligases;)																						
	ABCDEF_7378	UniRef50_Q9FFE6	0.0	Normal: 1022	1422	746	576	680	1408	BlastAnno: 2117	UniRef50_Q9FFE6	552	67.57	552	167	5	144	1778	1	547	0.0	768
	ABCDEF_47513	UniRef50_Q8RU95	0.0	Normal: 411	609	570	233	749	887	BlastAnno: 2280	UniRef50_Q8RU95	598	53.15	572	246	6	119	1777	28	596	0.0	543
	ABCDEF_78742	UniRef50_Q84P25	0.0	Normal: 236	207	373	576	450	306	BlastAnno: 1534	UniRef50_Q84P25	565	60.39	462	168	5	1413	49	19	472	0.0	546
	ABCDEF_10753	UniRef50_Q9FFE6	0.0	Normal: 394	401	336	253	292	348	BlastAnno: 1841	UniRef50_Q9FFE6	552	51.00	551	259	5	117	1751	1	546	0.0	553
	ABCDEF_4431	UniRef50_Q9FFE6	0.0	Normal: 27	358	28	98	112	914	BlastAnno: 2075	UniRef50_Q9FFE6	552	50.71	560	264	7	1774	107	1	552	0.0	551
	ABCDEF_38213	UniRef50_Q84P25	0.0	Normal: 342	337	597	798	722	505	BlastAnno: 1939	UniRef50_Q84P25	565	62.34	555	194	5	1818	175	19	565	0.0	690
	ABCDEF_25221	UniRef50_Q0DV32	0.0	Normal: 971	826	1003	1543	1442	1545	BlastAnno: 2009	UniRef50_Q0DV32	552	57.20	542	226	4	183	1793	9	549	0.0	613
	ABCDEF_23413	UniRef50_Q84P23	0.0	Normal: 123	237	158	149	213	658	BlastAnno: 3175	UniRef50_Q84P23	562	51.74	547	243	8	3030	1435	15	555	0.0	555
	ABCDEF_20486	UniRef50_Q9FFE6	0.0	Normal: 580	912	312	324	864	1565	BlastAnno: 3530	UniRef50_Q9FFE6	552	66.61	557	171	5	352	1995	1	551	0.0	781
6.2.1.33(4-chlorobenzoate---CoA ligase)																						
	No Correspond Unigene																					
>00380 Tryptophan metabolism																						
1.1.1.110(indolelactate dehydrogenase;)																						
	No Correspond Unigene																					
1.1.1.190(indole-3-acetaldehyde reductase (NADH);)																						
	No Correspond Unigene																					
1.1.1.191(indole-3-acetaldehyde reductase (NADPH);)																						
	No Correspond Unigene																					
1.1.1.35(3-hydroxyacyl-CoA dehydrogenase;)																						
	ABCDEF_80649	UniRef50_Q1LD07	5e-120	Normal: 527	841	443	1308	1321	1188	BlastAnno: 2586	UniRef50_Q1LD07	715	37.34	699	420	9	2358	277	22	707	5e-120	389
1.10.3.4(o-aminophenol oxidase;)																						

No Correspond Unigene																				
1.11.1.6(catalase;)																				
ABCDEF_32232	UniRef50_Q96528	0.0	Normal: 5097	16083	8563	16179	24962	32510	BlastAnno: 1961	UniRef50_Q96528	492	75.41	492	121	0	97	1572	1	492	0.0	816
ABCDEF_45231	UniRef50_Q96528	0.0	Normal: 3120	1495	2311	3940	2336	3972	BlastAnno: 1786	UniRef50_Q96528	492	86.99	492	64	0	74	1549	1	492	0.0	921
ABCDEF_12422	UniRef50_Q96528	0.0	Normal: 3094	9467	5243	8875	15494	19272	BlastAnno: 1462	UniRef50_Q96528	492	72.90	369	99	1	64	1167	122	490	0.0	597
ABCDEF_1075	UniRef50_Q96528	0.0	Normal: 205	3077	104	25	17	5494	BlastAnno: 1543	UniRef50_Q96528	492	83.74	406	66	0	1543	326	87	492	0.0	720
ABCDEF_727	UniRef50_Q96528	0.0	Normal: 671	268	532	1260	1197	440	BlastAnno: 1428	UniRef50_Q96528	492	87.81	443	54	0	98	1426	1	443	0.0	837
1.13.11.-(Oxidoreductases;)																				
No Correspond Unigene																				
1.13.11.10(7,8-dihydroxykynurenate 8,8a-dioxygenase;)																				
No Correspond Unigene																				
1.13.11.11(tryptophan 2,3-dioxygenase;)																				
No Correspond Unigene																				
1.13.11.17(indole 2,3-dioxygenase;)																				
No Correspond Unigene																				
1.13.11.23(2,3-dihydroxyindole 2,3-dioxygenase)																				
No Correspond Unigene																				
1.13.11.52(indoleamine 2,3-dioxygenase;)																				
No Correspond Unigene																				
1.13.11.6(3-hydroxyanthranilate 3,4-dioxygenase;)																				
No Correspond Unigene																				
1.13.12.3(tryptophan 2-monooxygenase)																				
No Correspond Unigene																				
1.13.99.3(tryptophan 2'-dioxygenase;)																				
No Correspond Unigene																				
1.14.-.-(Oxidoreductases;)																				
ABCDEF_44739	UniRef50_064989	0.0	Normal: 157	87	159	718	673	189	BlastAnno: 2185	UniRef50_064989	513	70.75	482	112	3	1867	509	30	511	0.0	706
ABCDEF_31491	UniRef50_Q93VK5	0.0	Normal: 647	1826	671	1517	1770	5953	BlastAnno: 2278	UniRef50_Q93VK5	595	79.14	556	113	2	198	1862	35	588	0.0	902
ABCDEF_40554	UniRef50_P37122	0.0	Normal: 1574	1962	1829	1150	2882	2721	BlastAnno: 1828	UniRef50_P37122	505	60.52	504	198	1	1752	244	1	504	0.0	638
ABCDEF_34049	UniRef50_023365	0.0	Normal: 363	384	349	442	549	1416	BlastAnno: 2101	UniRef50_023365	580	76.17	579	116	5	230	1957	19	578	0.0	904
ABCDEF_21579	UniRef50_048928	0.0	Normal: 1005	643	1606	4372	3226	1621	BlastAnno: 1895	UniRef50_048928	513	69.72	502	151	1	185	1690	7	507	0.0	731
ABCDEF_37741	UniRef50_P37122	0.0	Normal: 12	760	9	24	34	576	BlastAnno: 1197	UniRef50_P37122	505	65.70	379	129	1	1195	59	127	504	0.0	526
ABCDEF_35618	UniRef50_048928	0.0	Normal: 28	2648	17	142	1234	902	BlastAnno: 1746	UniRef50_048928	513	66.40	506	168	2	1649	132	4	507	0.0	701
ABCDEF_24522	UniRef50_P37122	0.0	Normal: 334	2190	561	268	1391	2459	BlastAnno: 2075	UniRef50_P37122	505	61.52	460	176	1	1544	165	47	505	0.0	612
ABCDEF_14592	UniRef50_023066	0.0	Normal: 1514	402	2245	7837	4742	1248	BlastAnno: 2378	UniRef50_023066	553	72.71	546	140	4	2111	489	1	542	0.0	821
ABCDEF_14512	UniRef50_Q9FG65	0.0	Normal: 261	872	212	291	175	789	BlastAnno: 1878	UniRef50_Q9FG65	502	56.89	501	197	9	1738	266	11	502	0.0	532
ABCDEF_44487	UniRef50_Q940V4	0.0	Normal: 3	41	53	501	262	542	BlastAnno: 1712	UniRef50_Q940V4	465	69.37	382	116	1	38	1180	14	395	0.0	572
ABCDEF_17723	UniRef50_P37122	0.0	Normal: 240	1698	219	768	592	794	BlastAnno: 1757	UniRef50_P37122	505	63.40	459	166	2	228	1601	47	504	0.0	617
ABCDEF_37901	UniRef50_Q42569	0.0	Normal: 30	149	86	255	195	255	BlastAnno: 1246	UniRef50_Q42569	472	78.27	359	73	2	64	1140	43	396	0.0	567
ABCDEF_7260	UniRef50_081117	0.0	Normal: 314	1085	466	3248	2575	1360	BlastAnno: 1768	UniRef50_081117	514	59.92	474	188	1	145	1566	43	514	0.0	615
ABCDEF_40548	UniRef50_P37122	0.0	Normal: 2756	2467	2752	1072	3120	1735	BlastAnno: 1947	UniRef50_P37122	505	59.72	504	202	1	241	1749	1	504	0.0	636
ABCDEF_44485	UniRef50_Q940V4	0.0	Normal: 3	55	63	574	338	660	BlastAnno: 1607	UniRef50_Q940V4	465	68.58	452	141	1	42	1394	14	465	0.0	673
ABCDEF_40552	UniRef50_P37122	0.0	Normal: 2397	1870	2304	1249	10428	2291	BlastAnno: 1812	UniRef50_P37122	505	60.12	504	200	1	108	1616	1	504	0.0	640
ABCDEF_37902	UniRef50_Q42569	0.0	Normal: 84	524	254	725	670	1049	BlastAnno: 1812	UniRef50_Q42569	472	77.47	435	92	3	1675	371	43	471	0.0	681
ABCDEF_40556	UniRef50_P37122	0.0	Normal: 1907	2365	2163	1319	3678	3308	BlastAnno: 1944	UniRef50_P37122	505	61.04	498	193	1	264	1754	1	498	0.0	635
ABCDEF_40549	UniRef50_P37122	0.0	Normal: 1574	1962	1826	1150	2882	2723	BlastAnno: 1845	UniRef50_P37122	505	60.52	504	198	1	1754	246	1	504	0.0	638
ABCDEF_20301	UniRef50_081117	0.0	Normal: 89	308	123	176	389	249	BlastAnno: 1637	UniRef50_081117	514	64.04	470	164	3	1498	98	47	514	0.0	586
ABCDEF_3882	UniRef50_Q42569	0.0	Normal: 84	243	257	786	1561	182	BlastAnno: 1854	UniRef50_Q42569	472	72.51	422	107	1	1852	587	59	471	0.0	615
ABCDEF_20703	UniRef50_049858	0.0	Normal: 115	636	20	0	0	597	BlastAnno: 1815	UniRef50_049858	527	50.10	523	248	6	48	1607	12	524	0.0	533
ABCDEF_18295	UniRef50_022203	0.0	Normal: 3667	1934	4977	10491	4588	1917	BlastAnno: 2006	UniRef50_022203	508	74.85	493	123	1	214	1692	17	508	0.0	803
ABCDEF_48724	UniRef50_023066	0.0	Normal: 104	11518	163	2083	3464	6356	BlastAnno: 2162	UniRef50_023066	553	72.96	540	143	3	227	1840	1	539	0.0	807
ABCDEF_37738	UniRef50_P37122	0.0	Normal: 26	2447	19	30	33	993	BlastAnno: 1837	UniRef50_P37122	505	64.30	479	169	2	1685	249	28	504	0.0	639
1.14.13.-(Oxidoreductases;)																				
ABCDEF_45538	UniRef50_023024	3e-156	Normal: 110	6	165	774	133	36	BlastAnno: 1258	UniRef50_023024	437	58.18	385	151	3	3	1157	37	411	3e-156	457
1.14.13.9(kynurenine 3-monooxygenase;)																				
No Correspond Unigene																				
1.14.14.1(unspecific monooxygenase;)																				
ABCDEF_41955	UniRef50_B9S0F8	2e-174	Normal: 70	140	28	0	0	150	BlastAnno: 1118	UniRef50_B9S0F8	506	66.67	366	115	5	2	1099	138	496	2e-174	504
1.14.16.-(Oxidoreductases;)																				
No Correspond Unigene																				
1.14.16.3(anthranilate 3-monooxygenase;)																				
No Correspond Unigene																				
1.14.16.4(tryptophan 5-monooxygenase;)																				
No Correspond Unigene																				
1.14.99.2(kynurenine 7,8-hydroxylase;)																				
No Correspond Unigene																				
1.2.1.-(Oxidoreductases;)																				
ABCDEF_14573	UniRef50_B5ZBH4	2e-108	Normal: 2547	9518	1266	309	441	22896	BlastAnno: 1872	UniRef50_B5ZBH4	475	42.52	461	259	4	228	1598	16	474	2e-108	343
1.2.1.3(aldehyde dehydrogenase (NAD+);)																				
ABCDEF_52483	UniRef50_Q70E96	0.0	Normal: 28	851	41	0	3965	420	BlastAnno: 1750	UniRef50_Q70E96	484	54.56	482	219	0	107	1552	3	484	0.0	548
ABCDEF_17638	UniRef50_Q70E96	0.0	Normal: 580	718	749	2424	1317	775	BlastAnno: 1970	UniRef50_Q70E96	484	62.21	471	177	1	283	1692	2	472	0.0	625
ABCDEF_403	UniRef50_Q8W033	0.0	Normal: 69	355	98	39	223	1111	BlastAnno: 1749	UniRef50_Q8W033	550	65.96	473	161	0	1523	105	74	546	0.0	625

ABCDEF_45143	UniRef50_Q0WSF1	0.0	Normal: 1445	874	1200	1789	1940	1504	BlastAnno: 2450	UniRef50_Q0WSF1	596	79.97	594	115	2	2220	451	1	594	0.0	1015
1.2.1.32(aminomuconate-semialdehyde dehydrogenase;)																					
No Correspond Unigene																					
1.2.3.1(aldehyde oxidase;)																					
ABCDEF_37559	UniRef50_Q7G193	0.0	Normal: 208	649	188	236	120	436	BlastAnno: 2825	UniRef50_Q7G193	1368	58.36	927	352	11	2741	3	3	909	0.0	1063
ABCDEF_51652	UniRef50_Q7G193	0.0	Normal: 97	323	130	90	67	347	BlastAnno: 1575	UniRef50_Q7G193	1368	64.15	491	172	4	5	1468	871	1360	0.0	632
1.2.3.7(indole-3-acetaldehyde oxidase;)																					
No Correspond Unigene																					
1.2.4.2(oxoglutarate dehydrogenase (succinyl-transferring);)																					
ABCDEF_21623	UniRef50_O61199	0.0	Normal: 2615	1939	3031	10740	12054	2537	BlastAnno: 3717	UniRef50_O61199	1029	48.29	996	440	18	3334	491	49	1017	0.0	931
ABCDEF_30869	UniRef50_O61199	0.0	Normal: 1668	226	1504	1974	2525	280	BlastAnno: 3680	UniRef50_O61199	1029	46.61	1047	463	23	3319	326	18	1017	0.0	911
1.3.1.-(Oxidoreductases;)																					
ABCDEF_45552	UniRef50_P52581	0.0	Normal: 213	2112	262	1058	1208	1322	BlastAnno: 1284	UniRef50_P52581	312	80.45	312	61	0	185	1120	1	312	0.0	530
ABCDEF_47970	UniRef50_Q9T0J6	0.0	Normal: 1040	443	1171	941	1234	650	BlastAnno: 2557	UniRef50_Q9T0J6	700	60.52	699	232	9	2337	271	36	700	0.0	860
1.3.1.18(kynurenate-7,8-dihydrodiol dehydrogenase;)																					
No Correspond Unigene																					
1.3.99.7(glutaryl-CoA dehydrogenase;)																					
ABCDEF_25385	UniRef50_Q22KH3	8e-120	Normal: 747	1014	820	1304	1917	2681	BlastAnno: 1768	UniRef50_Q22KH3	411	45.15	392	213	2	232	1404	17	407	8e-120	369
1.4.3.2(L-amino-acid oxidase;)																					
ABCDEF_34035	UniRef50_B9R844	0.0	Normal: 973	600	948	1073	764	972	BlastAnno: 3436	UniRef50_B9R844	1947	69.56	611	156	9	1050	2867	1361	1946	0.0	707
1.4.3.22(diamine oxidase;)																					
ABCDEF_14886	UniRef50_Q59118	5e-55	Normal: 90	94	139	183	301	14	BlastAnno: 661	UniRef50_Q59118	684	49.07	216	105	3	653	6	314	524	5e-55	193
1.4.3.4(monoamine oxidase;)																					
ABCDEF_11940	UniRef50_Q8LL67	3e-122	Normal: 41	0	82	2	541	4	BlastAnno: 1557	UniRef50_Q8LL67	496	45.61	410	204	3	2	1183	74	480	3e-122	376
1.5.1.-(Oxidoreductases;)																					
No Correspond Unigene																					
1.7.3.-(Oxidoreductases;)																					
No Correspond Unigene																					
1.7.3.2(acetylindoxyl oxidase;)																					
No Correspond Unigene																					
2.1.1.-(Transferases;)																					
ABCDEF_20806	UniRef50_Q94II3	0.0	Normal: 700	332	876	1458	1997	929	BlastAnno: 2275	UniRef50_Q94II3	600	73.57	594	153	4	2119	341	1	591	0.0	907
ABCDEF_45802	UniRef50_Q9ZPH9	0.0	Normal: 3543	3348	4296	9797	10830	5017	BlastAnno: 2642	UniRef50_Q9ZPH9	633	53.90	603	261	7	2216	456	23	624	0.0	676
ABCDEF_20070	UniRef50_Q944R7	0.0	Normal: 301	141	265	207	185	187	BlastAnno: 2675	UniRef50_Q944R7	724	58.28	719	275	9	127	2265	13	712	0.0	840
ABCDEF_34956	UniRef50_Q94II3	0.0	Normal: 1120	7269	2475	16033	22219	21461	BlastAnno: 2676	UniRef50_Q94II3	600	73.77	610	146	4	404	2230	1	597	0.0	957
ABCDEF_17121	UniRef50_Q9ZPH9	0.0	Normal: 124	77	109	284	199	132	BlastAnno: 2066	UniRef50_Q9ZPH9	633	53.24	601	259	8	161	1903	25	623	0.0	644
ABCDEF_6524	UniRef50_Q9ZPH9	0.0	Normal: 471	296	490	753	870	730	BlastAnno: 2602	UniRef50_Q9ZPH9	633	50.89	619	290	5	2221	404	6	623	0.0	659
ABCDEF_56017	UniRef50_Q9ZPH9	0.0	Normal: 302	167	286	596	748	529	BlastAnno: 2384	UniRef50_Q9ZPH9	633	51.37	619	287	5	213	2030	6	623	0.0	663
ABCDEF_44169	UniRef50_Q9SIZ3	0.0	Normal: 179	158	273	705	685	229	BlastAnno: 2300	UniRef50_Q9SIZ3	589	62.01	308	108	4	1219	296	285	583	0.0	394
ABCDEF_6521	UniRef50_Q9ZPH9	0.0	Normal: 339	198	314	641	767	582	BlastAnno: 2511	UniRef50_Q9ZPH9	633	51.37	619	287	5	2172	355	6	623	0.0	663
ABCDEF_44172	UniRef50_Q9SIZ3	0.0	Normal: 204	184	287	767	741	242	BlastAnno: 2317	UniRef50_Q9SIZ3	589	68.15	449	134	4	1637	291	144	583	0.0	641
ABCDEF_199	UniRef50_Q9SU94	0.0	Normal: 1076	275	1009	558	1247	306	BlastAnno: 1769	UniRef50_Q9SU94	390	78.45	348	74	1	284	1324	43	390	0.0	584
ABCDEF_52252	UniRef50_Q84W92	0.0	Normal: 1658	720	1653	1980	2289	1601	BlastAnno: 2170	UniRef50_Q84W92	535	72.49	469	120	3	409	1791	34	501	0.0	703
ABCDEF_51404	UniRef50_A3BMN9	0.0	Normal: 864	188	716	337	631	326	BlastAnno: 2192	UniRef50_A3BMN9	620	50.50	604	243	9	2008	251	55	620	0.0	555
ABCDEF_21386	UniRef50_Q94AI4	0.0	Normal: 2326	1250	2220	4294	2920	1983	BlastAnno: 3701	UniRef50_Q94AI4	775	47.27	842	332	16	567	2978	8	775	0.0	577
ABCDEF_35101	UniRef50_Q9MAT5	0.0	Normal: 1440	420	1268	576	1136	850	BlastAnno: 1634	UniRef50_Q9MAT5	383	69.03	352	109	0	1449	394	32	383	0.0	550
ABCDEF_960	UniRef50_Q9LN50	0.0	Normal: 3504	13198	3609	11156	19651	12155	BlastAnno: 2626	UniRef50_Q9LN50	724	49.14	525	246	5	883	2451	219	724	0.0	570
ABCDEF_48082	UniRef50_Q9LN50	0.0	Normal: 410	445	467	870	993	454	BlastAnno: 2340	UniRef50_Q9LN50	724	70.25	521	155	0	710	2272	204	724	0.0	821
ABCDEF_10473	UniRef50_Q08A71	0.0	Normal: 374	187	332	425	456	314	BlastAnno: 1764	UniRef50_Q08A71	435	79.23	366	75	1	344	1438	70	435	0.0	575
ABCDEF_7295	UniRef50_Q9ZPH9	0.0	Normal: 25	54	30	81	26	329	BlastAnno: 1191	UniRef50_Q9ZPH9	633	69.23	403	110	4	1191	22	225	626	0.0	599
ABCDEF_4160	UniRef50_Q94II3	0.0	Normal: 189	0	210	71	17	72	BlastAnno: 1424	UniRef50_Q94II3	600	77.28	449	101	1	1424	81	143	591	0.0	758
ABCDEF_44857	UniRef50_Q9ZPH9	0.0	Normal: 101	224	103	299	588	350	BlastAnno: 1582	UniRef50_Q9ZPH9	633	72.06	519	143	1	1571	21	114	632	0.0	822
ABCDEF_71541	UniRef50_Q9SIZ3	0.0	Normal: 187	167	296	765	724	237	BlastAnno: 2268	UniRef50_Q9SIZ3	589	62.01	308	108	4	1210	287	285	583	0.0	394
ABCDEF_44171	UniRef50_Q9SIZ3	0.0	Normal: 214	196	312	827	802	251	BlastAnno: 2339	UniRef50_Q9SIZ3	589	64.78	548	182	5	1978	335	47	583	0.0	717
ABCDEF_20805	UniRef50_Q94II3	0.0	Normal: 674	344	885	1417	2194	963	BlastAnno: 2400	UniRef50_Q94II3	600	78.21	436	94	1	879	2183	156	591	0.0	734
ABCDEF_18210	UniRef50_Q9SIZ3	0.0	Normal: 5584	3714	7379	17006	21892	7546	BlastAnno: 3199	UniRef50_Q9SIZ3	589	50.00	576	269	7	2145	424	27	585	0.0	574
ABCDEF_6526	UniRef50_Q9ZPH9	0.0	Normal: 627	185	837	2717	2815	1530	BlastAnno: 2611	UniRef50_Q9ZPH9	633	54.76	599	259	5	462	2222	25	623	0.0	689
ABCDEF_6525	UniRef50_Q9ZPH9	0.0	Normal: 602	165	760	2369	2605	1400	BlastAnno: 2545	UniRef50_Q9ZPH9	633	54.92	599	258	5	431	2191	25	623	0.0	689
ABCDEF_6523	UniRef50_Q9ZPH9	0.0	Normal: 314	175	304	641	784	552	BlastAnno: 2312	UniRef50_Q9ZPH9	633	52.42	599	272	4	201	1958	25	623	0.0	663
ABCDEF_67393	UniRef50_Q9ZPH9	0.0	Normal: 434	245	444	608	768	655	BlastAnno: 2346	UniRef50_Q9ZPH9	633	51.92	599	275	4	232	1989	25	623	0.0	659
ABCDEF_3579	UniRef50_O14744	0.0	Normal: 1361	326	984	568	791	440	BlastAnno: 2369	UniRef50_O14744	637	47.00	634	294	10	267	2120	30	637	0.0	553
2.1.1.4(acetylserotonin O-methyltransferase;)																					
No Correspond Unigene																					
2.1.1.47(indolepyruvate C-methyltransferase;)																					
No Correspond Unigene																					
2.1.1.49(amine N-methyltransferase;)																					
No Correspond Unigene																					
2.3.1.87(aralkylamine N-acetyltransferase;)																					
No Correspond Unigene																					
2.3.1.9(acetyl-CoA C-acetyltransferase;)																					
ABCDEF_34825	UniRef50_Q8S4Y1	0.0	Normal: 640	698	620	807	839	1164	BlastAnno: 1773	UniRef50_Q8S4Y1	403	78.11	402	87	1	1597	395	2	403	0.0	598
ABCDEF_14629	UniRef50_Q8S4Y1	0.0	Normal: 634	316	650	1021	1278	691	BlastAnno: 1514	UniRef50_Q8S4Y1	403	85.11	403	60	0	1367	159	1	403	0.0	620

ABCDEF_107	UniRef50_Q8S4Y1	0.0	Normal: 332	460	460	842	893	424	BlastAnno: 1214	UniRef50_Q8S4Y1	403	84.07	339	54	0	1212	196	42	380	0.0	596
ABCDEF_14630	UniRef50_Q8S4Y1	0.0	Normal: 562	282	568	959	1173	627	BlastAnno: 1796	UniRef50_Q8S4Y1	403	85.50	400	58	0	436	1635	4	403	0.0	642
2.4.1.195(N-hydroxythioamide S-beta-glucosyltransferase;)																					
No Correspond Unigene																					
2.5.1.-(Transferases;)																					
ABCDEF_48175	UniRef50_P72580	2e-123	Normal: 366	927	282	531	590	1811	BlastAnno: 1917	UniRef50_P72580	323	58.44	320	130	2	610	1569	6	322	2e-123	377
2.6.1.-(Transferases;)																					
ABCDEF_3716	UniRef50_Q9LQ10	0.0	Normal: 224	353	140	47	76	606	BlastAnno: 2230	UniRef50_Q9LQ10	557	61.35	533	189	8	250	1812	30	557	0.0	609
ABCDEF_37756	UniRef50_Q9LQ10	0.0	Normal: 197	168	149	152	186	490	BlastAnno: 1840	UniRef50_Q9LQ10	557	57.49	534	196	7	1638	109	28	554	0.0	619
2.6.1.27(tryptophan transaminase;)																					
No Correspond Unigene																					
2.6.1.7(kynurenine---oxoglutarate transaminase;)																					
No Correspond Unigene																					
2.8.2.-(Transferases;)																					
ABCDEF_60620	UniRef50_P52839	2e-85	Normal: 240	393	445	400	902	151	BlastAnno: 1191	UniRef50_P52839	326	45.95	333	168	6	58	1050	2	324	2e-85	271
3.2.1.147(thioglucosidase;)																					
No Correspond Unigene																					
3.5.1.4(amidase;)																					
ABCDEF_3151	UniRef50_B9RF45	0.0	Normal: 113	275	72	56	28	1014	BlastAnno: 1850	UniRef50_B9RF45	500	68.47	425	133	1	131	1402	68	492	0.0	547
ABCDEF_81937	UniRef50_B9RF45	0.0	Normal: 101	261	68	53	28	927	BlastAnno: 1739	UniRef50_B9RF45	500	68.47	425	133	1	131	1402	68	492	0.0	546
3.5.1.9(arylformamidase;)																					
No Correspond Unigene																					
3.5.5.1(nitrilase;)																					
ABCDEF_3989	UniRef50_P46011	0.0	Normal: 806	816	966	1114	1416	1512	BlastAnno: 1581	UniRef50_P46011	355	79.10	354	71	3	255	1310	3	355	0.0	579
3.5.99.5(2-aminomuconate deaminase)																					
No Correspond Unigene																					
3.7.1.3(kynureninase)																					
No Correspond Unigene																					
4.1.1.-(Lyases;)																					
ABCDEF_57496	UniRef50_O96569	9e-20	Normal: 4	0	0	0	0	9	BlastAnno: 223	UniRef50_O96569	439	66.67	72	22	1	210	1	19	90	9e-20	87.4
4.1.1.28(aromatic-L-amino-acid decarboxylase;)																					
ABCDEF_28052	UniRef50_P54770	3e-145	Normal: 150	74	133	88	177	332	BlastAnno: 1237	UniRef50_P54770	533	55.61	374	141	2	1237	191	144	517	3e-145	433
4.1.1.43(phenylpyruvate decarboxylase;)																					
No Correspond Unigene																					
4.1.1.45(aminocarboxymuconate-semialdehyde decarboxylase;)																					
No Correspond Unigene																					
4.1.1.74(indolepyruvate decarboxylase;)																					
No Correspond Unigene																					
4.1.99.1(tryptophanase;)																					
No Correspond Unigene																					
4.2.1.17(enoyl-CoA hydratase;)																					
ABCDEF_21259	UniRef50_Q8W1L6	0.0	Normal: 272	276	284	384	326	305	BlastAnno: 2641	UniRef50_Q8W1L6	726	47.57	721	375	3	114	2267	6	726	0.0	633
ABCDEF_80649	UniRef50_Q8W1L6	0.0	Normal: 527	841	443	1308	1321	1188	BlastAnno: 2586	UniRef50_Q8W1L6	726	60.39	712	281	1	2403	268	1	711	0.0	818
ABCDEF_466	UniRef50_Q8W1L6	0.0	Normal: 1014	1690	1094	1677	2438	2080	BlastAnno: 2453	UniRef50_Q8W1L6	726	71.24	699	201	0	2368	272	7	705	0.0	954
ABCDEF_54834	UniRef50_Q8W1L6	0.0	Normal: 229	220	237	298	270	261	BlastAnno: 2405	UniRef50_Q8W1L6	726	47.52	606	315	3	274	2082	121	726	0.0	580
ABCDEF_34756	UniRef50_Q8W1L6	0.0	Normal: 776	726	768	2922	4039	1079	BlastAnno: 2469	UniRef50_Q8W1L6	726	68.93	708	217	2	2396	273	7	711	0.0	957
ABCDEF_4198	UniRef50_Q8W1L6	0.0	Normal: 609	929	532	1401	1568	1297	BlastAnno: 2667	UniRef50_Q8W1L6	726	60.45	713	281	1	2463	325	1	712	0.0	820
4.2.1.84(nitrile hydratase;)																					
No Correspond Unigene																					
4.4.1.-(Lyases;)																					
ABCDEF_18191	UniRef50_Q8IBI5	7e-156	Normal: 1423	1090	1398	1576	1783	1230	BlastAnno: 1995	UniRef50_Q8IBI5	553	53.92	408	183	3	268	1476	128	535	7e-156	470
4.99.1.6(indoleacetaldoxime dehydratase;)																					
ABCDEF_63488	UniRef50_O49342	6e-143	Normal: 0	215	0	0	3	15	BlastAnno: 1544	UniRef50_O49342	497	43.70	476	245	6	147	1535	32	497	6e-143	429
ABCDEF_61937	UniRef50_O49342	6e-143	Normal: 0	200	0	0	3	13	BlastAnno: 1520	UniRef50_O49342	497	43.70	476	245	6	123	1511	32	497	6e-143	429
6.1.1.2(tryptophan---tRNA ligase;)																					
ABCDEF_69527	UniRef50_P23381	2e-167	Normal: 95	39	83	81	88	84	BlastAnno: 1589	UniRef50_P23381	471	62.83	382	140	1	275	1414	85	466	2e-167	492
6.3.2.-(Ligases;)																					
ABCDEF_74753	UniRef50_Q9SS90	0.0	Normal: 79	73	69	297	369	210	BlastAnno: 2013	UniRef50_Q9SS90	489	68.91	402	97	3	526	1731	116	489	0.0	558
ABCDEF_24131	UniRef50_Q9SFX2	0.0	Normal: 641	701	610	1149	1244	1067	BlastAnno: 3045	UniRef50_Q9SFX2	811	63.79	812	287	3	2785	353	6	811	0.0	930
ABCDEF_75544	UniRef50_Q9SS90	0.0	Normal: 89	73	84	302	380	211	BlastAnno: 2121	UniRef50_Q9SS90	489	68.91	402	97	3	634	1839	116	489	0.0	558
ABCDEF_51679	UniRef50_Q9SKE2	0.0	Normal: 251	584	204	224	239	675	BlastAnno: 2146	UniRef50_Q9SKE2	575	64.36	578	200	4	1935	211	1	575	0.0	798
ABCDEF_86548	UniRef50_Q9FKG6	0.0	Normal: 64	117	72	85	73	478	BlastAnno: 2670	UniRef50_Q9FKG6	845	59.04	520	208	4	459	2003	326	845	0.0	565
ABCDEF_3724	UniRef50_Q9SKE2	0.0	Normal: 606	1439	1010	1408	1489	1797	BlastAnno: 2182	UniRef50_Q9SKE2	575	49.41	589	270	5	81	1841	13	575	0.0	550
ABCDEF_28821	UniRef50_O81829	0.0	Normal: 57	509	32	0	264	10	BlastAnno: 1646	UniRef50_O81829	612	69.82	487	134	4	2	1432	129	612	0.0	699
ABCDEF_76341	UniRef50_Q9SS90	0.0	Normal: 82	73	81	294	365	252	BlastAnno: 2003	UniRef50_Q9SS90	489	68.91	402	97	3	516	1721	116	489	0.0	558
ABCDEF_37485	UniRef50_Q9SY59	0.0	Normal: 902	485	611	834	911	728	BlastAnno: 2911	UniRef50_Q9SY59	1188	63.38	852	276	12	2	2524	362	1188	0.0	1036
ABCDEF_43918	UniRef50_Q9FKG6	0.0	Normal: 82	166	96	99	81	575	BlastAnno: 3212	UniRef50_Q9FKG6	845	59.19	517	206	4	1181	2716	326	842	0.0	566
ABCDEF_41147	UniRef50_P43254	0.0	Normal: 366	581	264	197	203	1286	BlastAnno: 2369	UniRef50_P43254	675	75.04	669	150	4	199	2160	9	675	0.0	1051
ABCDEF_27699	UniRef50_Q8VZ40	0.0	Normal: 295	184	254	418	515	180	BlastAnno: 2010	UniRef50_Q8VZ40	632	51.94	618	285	4	48	1898	12	618	0.0	593
ABCDEF_13757	UniRef50_Q6NLQ8	0.0	Normal: 298	320	265	710	637	691	BlastAnno: 2276	UniRef50_Q6NLQ8	508	61.15	520	175	10	536	2050	1	508	0.0	573
ABCDEF_57048	UniRef50_Q680Q4	0.0	Normal: 75	57	53	99	74	82	BlastAnno: 1311	UniRef50_Q680Q4	884	68.23	406	113	5	86	1297	55	446	0.0	577
ABCDEF_44723	UniRef50_P43254	0.0	Normal: 52	336	37	844	1077	54	BlastAnno: 2400	UniRef50_P43254	675	69.89	631	181	4	257	2125	46	675	0.0	878

ABCDEF_194	UniRef50_Q9CAA7	0.0	Normal: 3	1075	8	365	944	1388	BlastAnno: 3532	UniRef50_Q9CAA7	1033	52.26	819	384	5	857	3304	215	1029	0.0	867
ABCDEF_7408	UniRef50_Q9SNC6	0.0	Normal: 236	153	215	384	669	437	BlastAnno: 1514	UniRef50_Q9SNC6	660	66.07	445	148	2	181	1512	1	443	0.0	538
ABCDEF_44615	UniRef50_Q6WWW4	0.0	Normal: 2132	1418	1812	2598	2560	2010	BlastAnno: 3973	UniRef50_Q6WWW4	1888	66.95	1292	395	17	2	3829	613	1888	0.0	1412
ABCDEF_17061	UniRef50_O22193	0.0	Normal: 633	749	685	1190	1059	993	BlastAnno: 2993	UniRef50_O22193	811	57.91	815	336	4	106	2541	1	811	0.0	821
ABCDEF_47713	UniRef50_Q058P4	0.0	Normal: 412	121	401	426	515	247	BlastAnno: 2051	UniRef50_Q058P4	448	73.07	401	108	0	2021	819	48	448	0.0	605
ABCDEF_44955	UniRef50_O65041	0.0	Normal: 795	337	666	944	1072	676	BlastAnno: 1906	UniRef50_O65041	454	77.39	429	96	1	167	1450	26	454	0.0	617
ABCDEF_30563	UniRef50_Q9STN8	0.0	Normal: 300	224	312	433	553	441	BlastAnno: 1194	UniRef50_Q9STN8	327	78.70	324	61	2	960	13	1	324	0.0	531
ABCDEF_30141	UniRef50_Q680Q4	0.0	Normal: 1434	827	1232	1415	1424	1692	BlastAnno: 5949	UniRef50_Q680Q4	884	55.52	888	348	18	5794	3167	1	853	0.0	937
ABCDEF_43784	UniRef50_Q8GY23	0.0	Normal: 926	619	837	1435	1355	815	BlastAnno: 4761	UniRef50_Q8GY23	3681	59.71	1447	507	25	4759	482	2290	3681	0.0	1402
ABCDEF_34095	UniRef50_Q9CAG5	0.0	Normal: 459	1078	549	527	640	1260	BlastAnno: 2698	UniRef50_Q9CAG5	782	61.29	793	271	10	2541	238	1	782	0.0	893
ABCDEF_43782	UniRef50_Q8GY23	0.0	Normal: 2056	1659	1617	3872	2505	1381	BlastAnno: 10040	UniRef50_Q8GY23	3681	58.97	3266	1200	46	10033	371	511	3681	0.0	3227
ABCDEF_4158	UniRef50_Q9SKC3	0.0	Normal: 905	712	788	1261	1565	1198	BlastAnno: 2262	UniRef50_Q9SKC3	543	57.59	474	175	6	312	1700	39	497	0.0	552
ABCDEF_47200	UniRef50_Q8RXD6	0.0	Normal: 320	115	286	377	254	272	BlastAnno: 2788	UniRef50_Q8RXD6	878	51.12	847	406	2	79	2616	39	878	0.0	785
ABCDEF_7352	UniRef50_O22785	0.0	Normal: 2411	867	2207	1789	1932	1814	BlastAnno: 2095	UniRef50_O22785	565	73.28	509	130	2	166	1692	1	503	0.0	784
ABCDEF_44725	UniRef50_P43254	0.0	Normal: 27	226	26	613	624	35	BlastAnno: 1662	UniRef50_P43254	675	66.22	444	142	3	257	1567	46	488	0.0	563
ABCDEF_17164	UniRef50_Q8VZ40	0.0	Normal: 267	385	224	286	380	543	BlastAnno: 1970	UniRef50_Q8VZ40	632	71.46	494	140	1	1762	284	133	626	0.0	691
ABCDEF_37640	UniRef50_Q9CAG5	0.0	Normal: 193	183	184	219	381	262	BlastAnno: 1660	UniRef50_Q9CAG5	782	60.64	531	195	6	1659	82	202	723	0.0	648
ABCDEF_30565	UniRef50_Q9STN8	0.0	Normal: 213	117	212	263	281	228	BlastAnno: 1138	UniRef50_Q9STN8	327	77.78	315	66	3	939	1	1	313	0.0	516
ABCDEF_41098	UniRef50_Q9SU29	0.0	Normal: 282	273	196	258	277	348	BlastAnno: 2194	UniRef50_Q9SU29	873	53.13	670	297	5	2150	144	220	873	0.0	680
ABCDEF_14590	UniRef50_Q9LZW3	0.0	Normal: 108	323	52	180	220	640	BlastAnno: 1996	UniRef50_Q9LZW3	674	57.47	616	244	8	1827	1	1	605	0.0	648
ABCDEF_17658	UniRef50_Q9SKC3	0.0	Normal: 1602	1392	1390	2197	2440	1821	BlastAnno: 2599	UniRef50_Q9SKC3	543	53.00	534	199	9	348	1913	4	497	0.0	576
ABCDEF_47205	UniRef50_Q8RXD6	0.0	Normal: 259	99	247	302	237	231	BlastAnno: 2310	UniRef50_Q8RXD6	878	54.07	675	310	0	116	2140	204	878	0.0	702
ABCDEF_30078	UniRef50_Q680Q4	0.0	Normal: 873	690	641	788	1014	1012	BlastAnno: 3221	UniRef50_Q680Q4	884	59.18	801	302	20	3086	711	75	859	0.0	875
ABCDEF_30807	UniRef50_O22193	0.0	Normal: 595	326	514	867	993	504	BlastAnno: 2532	UniRef50_O22193	811	59.21	755	286	7	3	2228	66	811	0.0	805
ABCDEF_38002	UniRef50_Q6WWW4	0.0	Normal: 355	212	182	468	192	133	BlastAnno: 2012	UniRef50_Q6WWW4	1888	81.40	457	81	1	1382	12	150	602	0.0	670
ABCDEF_33803	UniRef50_Q9LYZ7	0.0	Normal: 259	474	177	176	198	1301	BlastAnno: 3239	UniRef50_Q9LYZ7	1502	50.43	930	398	13	44	2827	634	1502	0.0	814
ABCDEF_47910	UniRef50_Q6WWW4	0.0	Normal: 752	656	516	1256	662	392	BlastAnno: 2266	UniRef50_Q6WWW4	1888	76.34	558	115	3	618	2264	96	645	0.0	728
ABCDEF_27074	UniRef50_Q8GY23	0.0	Normal: 6555	5123	5068	10428	6398	5473	BlastAnno: 9201	UniRef50_Q8GY23	3681	55.50	1128	466	10	3520	182	2570	3676	0.0	1078
ABCDEF_43915	UniRef50_Q9FKG6	0.0	Normal: 51	115	63	87	61	398	BlastAnno: 2490	UniRef50_Q9FKG6	845	59.38	517	205	4	2032	497	326	842	0.0	567
ABCDEF_30143	UniRef50_Q680Q4	0.0	Normal: 95	103	72	139	90	112	BlastAnno: 1586	UniRef50_Q680Q4	884	66.05	489	148	6	1470	16	1	475	0.0	669
ABCDEF_47201	UniRef50_Q8RXD6	0.0	Normal: 330	125	310	408	282	294	BlastAnno: 2800	UniRef50_Q8RXD6	878	50.47	846	410	3	97	2628	40	878	0.0	772
ABCDEF_78356	UniRef50_Q9SS90	0.0	Normal: 85	77	87	349	409	221	BlastAnno: 2173	UniRef50_Q9SS90	489	68.91	402	97	3	686	1891	116	489	0.0	558
ABCDEF_74039	UniRef50_Q9SS90	0.0	Normal: 95	99	85	348	383	263	BlastAnno: 1911	UniRef50_Q9SS90	489	68.91	402	97	3	424	1629	116	489	0.0	558
ABCDEF_51550	UniRef50_Q337A0	0.0	Normal: 218	155	151	172	322	220	BlastAnno: 1748	UniRef50_Q337A0	520	62.55	486	168	8	1746	319	38	519	0.0	577
ABCDEF_38308	UniRef50_Q9C9A6	0.0	Normal: 691	435	789	1167	908	930	BlastAnno: 2443	UniRef50_Q9C9A6	628	62.12	631	206	8	2302	419	23	623	0.0	668
ABCDEF_41145	UniRef50_P43254	0.0	Normal: 356	550	260	193	190	1258	BlastAnno: 2411	UniRef50_P43254	675	77.15	547	116	2	581	2200	131	675	0.0	884
ABCDEF_41425	UniRef50_Q949V6	0.0	Normal: 725	674	644	860	723	1120	BlastAnno: 2415	UniRef50_Q949V6	597	66.39	601	188	6	282	2054	1	597	0.0	815
ABCDEF_10133	UniRef50_Q6WWW4	0.0	Normal: 886	519	1063	1147	867	928	BlastAnno: 2714	UniRef50_Q6WWW4	1888	67.89	788	236	8	44	2398	1115	1888	0.0	1009
ABCDEF_17165	UniRef50_Q8VZ40	0.0	Normal: 363	553	302	370	514	687	BlastAnno: 2507	UniRef50_Q8VZ40	632	65.91	619	208	2	2174	321	10	626	0.0	765
ABCDEF_14304	UniRef50_Q8GUH1	0.0	Normal: 943	887	877	1592	1148	957	BlastAnno: 3171	UniRef50_Q8GUH1	834	52.05	855	345	10	2798	246	40	833	0.0	796
ABCDEF_7212	UniRef50_O81829	0.0	Normal: 354	228	82	27	97	240	BlastAnno: 1645	UniRef50_O81829	612	78.03	537	98	3	1559	3	1	535	0.0	845
ABCDEF_28063	UniRef50_Q9C7R6	0.0	Normal: 770	617	728	857	782	1643	BlastAnno: 2309	UniRef50_Q9C7R6	729	69.06	682	192	5	16	2019	53	729	0.0	799
ABCDEF_44308	UniRef50_Q9SKC3	0.0	Normal: 359	268	257	424	447	440	BlastAnno: 2361	UniRef50_Q9SKC3	543	55.71	490	189	7	1961	531	23	497	0.0	572
ABCDEF_51678	UniRef50_Q9SKE2	0.0	Normal: 325	750	292	343	337	819	BlastAnno: 2313	UniRef50_Q9SKE2	575	64.36	578	200	4	1935	211	1	575	0.0	800
ABCDEF_40991	UniRef50_Q8RWB8	0.0	Normal: 906	783	917	1713	1701	1010	BlastAnno: 3667	UniRef50_Q8RWB8	1029	65.18	1034	350	8	245	3337	1	1027	0.0	1346
ABCDEF_37758	UniRef50_O22785	0.0	Normal: 1008	438	871	974	982	910	BlastAnno: 1956	UniRef50_O22785	565	72.89	509	132	2	97	1623	1	503	0.0	752
ABCDEF_44311	UniRef50_Q9SKC3	0.0	Normal: 386	339	263	463	494	561	BlastAnno: 2479	UniRef50_Q9SKC3	543	55.51	490	190	7	2080	650	23	497	0.0	570
ABCDEF_10466	UniRef50_Q9LX73	0.0	Normal: 834	669	858	1149	1242	1308	BlastAnno: 2786	UniRef50_Q9LX73	804	63.49	797	269	3	244	2613	19	800	0.0	909
ABCDEF_43917	UniRef50_Q9FKG6	0.0	Normal: 96	186	105	101	99	649	BlastAnno: 3392	UniRef50_Q9FKG6	845	59.42	520	206	4	2212	668	326	845	0.0	573
ABCDEF_13727	UniRef50_Q9C895	0.0	Normal: 719	474	621	782	703	805	BlastAnno: 2718	UniRef50_Q9C895	900	53.57	799	368	2	2716	323	104	900	0.0	729
ABCDEF_30562	UniRef50_Q9STN8	0.0	Normal: 391	213	307	381	453	346	BlastAnno: 1387	UniRef50_Q9STN8	327	76.90	329	72	3	1188	208	1	327	0.0	533
ABCDEF_33719	UniRef50_Q9FGI1	0.0	Normal: 988	729	776	951	970	1151	BlastAnno: 5387	UniRef50_Q9FGI1	1873	46.92	1735	828	23	5387	294	194	1872	0.0	1405
ABCDEF_24182	UniRef50_Q9SRT0	0.0	Normal: 423	1042	441	695	609	2149	BlastAnno: 1840	UniRef50_Q9SRT0	460	59.48	464	176	6	1682	315	1	460	0.0	532
ABCDEF_33804	UniRef50_Q9LYZ7	0.0	Normal: 286	601	216	260	260	1423	BlastAnno: 4233	UniRef50_Q9LYZ7	1502	49.59	1226	539	17	4232	561	333	1481	0.0	1035
ABCDEF_20465	UniRef50_Q9SCQ2	0.0	Normal: 525	613	474	898	939	1029	BlastAnno: 3231	UniRef50_Q9SCQ2	1142	58.15	1037	400	9	3230	138	134	1142	0.0	1203
ABCDEF_34315	UniRef50_Q84JU6	0.0	Normal: 953	381	860	834	846	454	BlastAnno: 3180	UniRef50_Q84JU6	927	51.25	921	391	16	241	2916	6	897	0.0	837
ABCDEF_51379	UniRef50_Q9SS90	0.0	Normal: 102	79	93	369	481	220	BlastAnno: 2281	UniRef50_Q9SS90	489	68.91	402	97	3	794	1999	116	489	0.0	558
ABCDEF_44309	UniRef50_Q8W468	0.0	Normal: 259	206	176	313	286	306	BlastAnno: 1676	UniRef50_Q8W468	567	74.33	409	104	1	453	1676	42	450	0.0	663
ABCDEF_13924	UniRef50_Q8S8S7	0.0	Normal: 427	108	396	365	361	640	BlastAnno: 2274	UniRef50_Q8S8S7	801	62.61	476	177	1	583	2007	317	792	0.0	615
ABCDEF_52353	UniRef50_Q9SS90	0.0	Normal: 831	901	819	1213	1370	1079	BlastAnno: 1826	UniRef50_Q9SS90	489	72.80	375	93	3	1470	349	123	489	0.0	572

>00400 Phenylalanine, tyrosine and tryptophan biosynthesis

1.1.1.24(quinate dehydrogenase;)

No Correspond Unigene

1.1.1.25(shikimate dehydrogenase;)

ABCDEF_8052	UniRef50_B9I9X4	0.0	Normal: 0	119	0	0	17	1392	BlastAnno: 1731	UniRef50_B9I9X4	504	69.74	509	149	4	1643	117	1	504	0.0	653
ABCDEF_17417	UniRef50_B9I9X4	0.0	Normal: 171	396	421	286	1563	196	BlastAnno: 2089	UniRef50_B9I9X4	504	69.38	516	141	4	2004	472	1	504	0.0	632

1.1.1.282(quinate/shikimate dehydrogenase;)

No Correspond Unigene

1.1.5.8(quinate dehydrogenase (quinone);)

No Correspond Unigene

1.14.16.1(phenylalanine 4-monooxygenase;)

No Correspond Unigene

1.3.1.12(prephenate dehydrogenase;)																					
No Correspond Unigene																					
1.3.1.13(prephenate dehydrogenase (NADP+);)																					
No Correspond Unigene																					
1.3.1.43(arogenate dehydrogenase;)																					
No Correspond Unigene																					
1.4.1.-(Oxidoreductases;)																					
No Correspond Unigene																					
1.4.1.20(phenylalanine dehydrogenase;)																					
No Correspond Unigene																					
1.4.3.2(L-amino-acid oxidase;)																					
ABCDEF_34035	UniRef50_B9R844	0.0	Normal: 973	600	948	1073	764	972	BlastAnno: 3436	UniRef50_B9R844	1947	69.56	611	156	9	1050	2867	1361	1946	0.0	707
2.4.2.18(anthranilate phosphoribosyltransferase;)																					
ABCDEF_27819	UniRef50_Q02166	2e-174	Normal: 421	506	484	682	1275	1380	BlastAnno: 1795	UniRef50_Q02166	444	73.60	356	92	1	410	1471	88	443	2e-174	511
2.5.1.19(3-phosphoshikimate 1-carboxyvinyltransferase;)																					
ABCDEF_20207	UniRef50_P05466	0.0	Normal: 366	279	532	1081	712	223	BlastAnno: 2070	UniRef50_P05466	520	75.81	525	117	5	282	1841	1	520	0.0	769
ABCDEF_63939	UniRef50_P05466	0.0	Normal: 548	399	861	1112	749	371	BlastAnno: 1782	UniRef50_P05466	520	82.39	460	77	1	345	1724	65	520	0.0	739
ABCDEF_20205	UniRef50_P05466	0.0	Normal: 396	285	542	1046	667	236	BlastAnno: 1970	UniRef50_P05466	520	75.62	525	118	5	217	1776	1	520	0.0	768
ABCDEF_62439	UniRef50_P05466	0.0	Normal: 428	374	689	1352	866	301	BlastAnno: 1848	UniRef50_P05466	520	75.43	525	115	6	72	1619	1	520	0.0	735
ABCDEF_20204	UniRef50_P05466	0.0	Normal: 599	428	993	1444	878	336	BlastAnno: 2171	UniRef50_P05466	520	82.61	460	76	1	563	1942	65	520	0.0	765
ABCDEF_20206	UniRef50_P05466	0.0	Normal: 462	367	659	1026	730	271	BlastAnno: 1918	UniRef50_P05466	520	83.04	460	74	1	345	1724	65	520	0.0	732
ABCDEF_20208	UniRef50_P05466	0.0	Normal: 477	405	738	1575	930	290	BlastAnno: 2023	UniRef50_P05466	520	75.05	525	117	6	282	1829	1	520	0.0	756
2.5.1.54(3-deoxy-7-phosphoheptulonate synthase;)																					
ABCDEF_49212	UniRef50_A9HLE8	0.0	Normal: 9031	3262	12277	18615	11274	3764	BlastAnno: 1968	UniRef50_A9HLE8	470	57.62	453	190	2	368	1720	12	464	0.0	546
ABCDEF_38160	UniRef50_A9HLE8	0.0	Normal: 479	716	501	590	650	2394	BlastAnno: 1872	UniRef50_A9HLE8	470	58.19	452	188	1	274	1626	14	465	0.0	545
ABCDEF_243	UniRef50_A9HLE8	0.0	Normal: 1344	530	2947	9252	3331	472	BlastAnno: 2106	UniRef50_A9HLE8	470	58.26	448	186	1	403	1743	15	462	0.0	547
2.6.1.1(aspartate transaminase;)																					
ABCDEF_11690	UniRef50_P46248	0.0	Normal: 1745	1815	2022	4078	5299	6095	BlastAnno: 1778	UniRef50_P46248	453	86.09	417	58	0	297	1547	37	453	0.0	733
ABCDEF_1333	UniRef50_C0PSV4	0.0	Normal: 356	544	636	1119	2495	631	BlastAnno: 1594	UniRef50_C0PSV4	462	76.43	403	95	0	1401	193	60	462	0.0	662
ABCDEF_21786	UniRef50_C0PSV4	0.0	Normal: 268	668	330	546	1267	646	BlastAnno: 1736	UniRef50_C0PSV4	462	74.14	464	113	3	50	1441	4	460	0.0	711
ABCDEF_42262	UniRef50_P46643	0.0	Normal: 916	606	1656	2699	2553	856	BlastAnno: 1722	UniRef50_P46643	430	79.49	429	85	2	1635	355	1	428	0.0	731
ABCDEF_56929	UniRef50_P37833	0.0	Normal: 173	278	312	654	1243	297	BlastAnno: 1151	UniRef50_P37833	407	81.82	308	56	0	978	55	3	310	0.0	536
2.6.1.5(tyrosine transaminase;)																					
ABCDEF_52650	UniRef50_Q9LVY1	0.0	Normal: 493	358	455	302	300	892	BlastAnno: 1442	UniRef50_Q9LVY1	420	61.26	413	160	0	65	1303	5	417	0.0	539
2.6.1.57(aromatic-amino-acid transaminase;)																					
No Correspond Unigene																					
2.6.1.58(phenylalanine(histidine) transaminase;)																					
No Correspond Unigene																					
2.6.1.9(histidinol-phosphate transaminase;)																					
ABCDEF_45962	UniRef50_A7NFV2	2e-99	Normal: 595	558	686	955	1251	1312	BlastAnno: 1635	UniRef50_A7NFV2	371	48.76	363	178	4	336	1403	7	368	2e-99	313
2.7.1.71(shikimate kinase;)																					
ABCDEF_40774	UniRef50_Q00497	2e-129	Normal: 82	64	92	92	119	121	BlastAnno: 1315	UniRef50_Q00497	300	64.07	295	105	1	290	1174	1	294	2e-129	385
4.1.1.48(indole-3-glycerol-phosphate synthase;)																					
ABCDEF_35383	UniRef50_P49572	2e-177	Normal: 1020	1158	1113	1413	1760	3041	BlastAnno: 1539	UniRef50_P49572	402	72.00	350	97	1	1297	251	51	400	2e-177	514
4.1.2.-(Lyases;)																					
ABCDEF_38428	UniRef50_C3KN71	1e-47	Normal: 551	427	606	695	1516	1338	BlastAnno: 1495	UniRef50_C3KN71	273	38.34	253	150	1	334	1092	7	253	1e-47	173
4.1.3.27(anthranilate synthase;)																					
ABCDEF_24517	UniRef50_P32069	0.0	Normal: 84	594	305	1827	1284	3111	BlastAnno: 2262	UniRef50_P32069	621	68.17	443	137	2	228	1547	61	502	0.0	616
ABCDEF_13978	UniRef50_P32069	0.0	Normal: 125	89	159	206	237	341	BlastAnno: 2223	UniRef50_P32069	621	74.25	563	123	4	261	1937	63	607	0.0	809
ABCDEF_13979	UniRef50_P32069	0.0	Normal: 134	92	173	194	242	349	BlastAnno: 2184	UniRef50_P32069	621	76.14	549	123	3	261	1895	63	607	0.0	818
ABCDEF_34411	UniRef50_P32069	0.0	Normal: 236	102	252	284	482	175	BlastAnno: 1742	UniRef50_P32069	621	73.99	542	134	3	1740	130	68	607	0.0	798
ABCDEF_24516	UniRef50_P32069	0.0	Normal: 107	716	356	2060	1415	3602	BlastAnno: 2254	UniRef50_P32069	621	68.98	548	161	3	228	1847	61	607	0.0	771
4.2.1.-(Lyases;)																					
ABCDEF_48679	UniRef50_Q0RP29	5e-115	Normal: 568	325	563	941	1285	676	BlastAnno: 1424	UniRef50_Q0RP29	493	56.39	321	129	4	119	1075	39	350	5e-115	356
4.2.1.10(3-dehydroquinate dehydratase;)																					
ABCDEF_6231	UniRef50_Q9SQT8	0.0	Normal: 370	193	351	565	481	240	BlastAnno: 2058	UniRef50_Q9SQT8	603	70.83	312	91	0	190	1125	91	402	0.0	472
ABCDEF_34606	UniRef50_Q9SQT8	0.0	Normal: 1132	887	1335	2055	2189	1320	BlastAnno: 1941	UniRef50_Q9SQT8	603	67.25	513	164	1	199	1725	91	603	0.0	676
ABCDEF_6229	UniRef50_Q9SQT8	0.0	Normal: 383	215	390	667	528	269	BlastAnno: 1961	UniRef50_Q9SQT8	603	70.76	513	147	1	190	1719	91	603	0.0	702
4.2.1.20(tryptophan synthase;)																					
ABCDEF_49386	UniRef50_P25269	0.0	Normal: 1090	905	1057	1677	1610	1777	BlastAnno: 1723	UniRef50_P25269	475	81.07	486	62	5	41	1444	2	475	0.0	775
ABCDEF_4210	UniRef50_P25269	0.0	Normal: 5	209	3	0	0	1341	BlastAnno: 1670	UniRef50_P25269	475	62.71	421	150	3	205	1458	56	472	0.0	552
4.2.1.51(prephenate dehydratase;)																					
ABCDEF_44484	UniRef50_Q9SA96	9e-171	Normal: 361	182	398	451	451	520	BlastAnno: 1611	UniRef50_Q9SA96	392	63.12	404	125	8	1333	149	1	389	9e-171	497
4.2.1.91(arogenate dehydratase;)																					
ABCDEF_48233	UniRef50_Q9FNJ8	0.0	Normal: 1033	409	1709	6471	2109	566	BlastAnno: 1644	UniRef50_Q9FNJ8	425	77.60	375	65	4	1211	120	52	418	0.0	547
4.2.3.4(3-dehydroquinate synthase;)																					
ABCDEF_75947	UniRef50_Q0AJ12	2e-139	Normal: 989	1055	1193	1512	2240	1435	BlastAnno: 1491	UniRef50_Q0AJ12	369	61.40	342	125	3	1200	187	17	355	2e-139	415
ABCDEF_52737	UniRef50_Q0AJ12	2e-139	Normal: 1033	1060	1112	1314	2299	1514	BlastAnno: 1595	UniRef50_Q0AJ12	369	61.40	342	125	3	385	1398	17	355	2e-139	416
4.2.3.5(chorismate synthase;)																					
ABCDEF_15276	UniRef50_B4FTP6	0.0	Normal: 2265	2179	3919	5159	4927	4252	BlastAnno: 1792	UniRef50_B4FTP6	402	68.56	388	95	1	1555	392	41	401	0.0	538
5.3.1.24(phosphoribosylanthranilate isomerase;)																					
ABCDEF_90219	UniRef50_Q9FFF7	2e-96	Normal: 59	15	54	28	25	145	BlastAnno: 985	UniRef50_Q9FFF7	281	63.82	246	79	1	188	895	35	280	2e-96	295

5.4.99.5(chorismate mutase;)																			
ABCDEF_52500	UniRef50_P42738	6e-139	Normal: 627	562	831	1435	1045	820	BlastAnno: 1362	UniRef50_P42738	334	65.58	308	96	3	1118	225	27	334	6e-139	411
>00401 Novobiocin biosynthesis																			
1.3.1.12(prephenate dehydrogenase;)																			
No Correspond Unigene																			
1.3.1.43(arogenate dehydrogenase;)																			
No Correspond Unigene																			
2.6.1.1(aspartate transaminase;)																			
ABCDEF_11690	UniRef50_P46248	0.0	Normal: 1745	1815	2022	4078	5299	6095	BlastAnno: 1778	UniRef50_P46248	453	86.09	417	58	0	297	1547	37	453	0.0	733
ABCDEF_1333	UniRef50_C0PSV4	0.0	Normal: 356	544	636	1119	2495	631	BlastAnno: 1594	UniRef50_C0PSV4	462	76.43	403	95	0	1401	193	60	462	0.0	662
ABCDEF_21786	UniRef50_C0PSV4	0.0	Normal: 268	668	330	546	1267	646	BlastAnno: 1736	UniRef50_C0PSV4	462	74.14	464	113	3	50	1441	4	460	0.0	711
ABCDEF_42262	UniRef50_P46643	0.0	Normal: 916	606	1656	2699	2553	856	BlastAnno: 1722	UniRef50_P46643	430	79.49	429	85	2	1635	355	1	428	0.0	731
ABCDEF_56929	UniRef50_P37833	0.0	Normal: 173	278	312	654	1243	297	BlastAnno: 1151	UniRef50_P37833	407	81.82	308	56	0	978	55	3	310	0.0	536
2.6.1.5(tyrosine transaminase;)																			
ABCDEF_52650	UniRef50_Q9LVY1	0.0	Normal: 493	358	455	302	300	892	BlastAnno: 1442	UniRef50_Q9LVY1	420	61.26	413	160	0	65	1303	5	417	0.0	539
2.6.1.57(aromatic-amino-acid transaminase;)																			
No Correspond Unigene																			
2.6.1.9(histidinol-phosphate transaminase;)																			
ABCDEF_45962	UniRef50_A7NFV2	2e-99	Normal: 595	558	686	955	1251	1312	BlastAnno: 1635	UniRef50_A7NFV2	371	48.76	363	178	4	336	1403	7	368	2e-99	313
>00402 Benzoxazinoid biosynthesis																			
1.14.-.-(Oxidoreductases;)																			
ABCDEF_44739	UniRef50_O64989	0.0	Normal: 157	87	159	718	673	189	BlastAnno: 2185	UniRef50_O64989	513	70.75	482	112	3	1867	509	30	511	0.0	706
ABCDEF_31491	UniRef50_Q93VK5	0.0	Normal: 647	1826	671	1517	1770	5953	BlastAnno: 2278	UniRef50_Q93VK5	595	79.14	556	113	2	198	1862	35	588	0.0	902
ABCDEF_40554	UniRef50_P37122	0.0	Normal: 1574	1962	1829	1150	2882	2721	BlastAnno: 1828	UniRef50_P37122	505	60.52	504	198	1	1752	244	1	504	0.0	638
ABCDEF_34049	UniRef50_O23365	0.0	Normal: 363	384	349	442	549	1416	BlastAnno: 2101	UniRef50_O23365	580	76.17	579	116	5	230	1957	19	578	0.0	904
ABCDEF_21579	UniRef50_O48928	0.0	Normal: 1005	643	1606	4372	3226	1621	BlastAnno: 1895	UniRef50_O48928	513	69.72	502	151	1	185	1690	7	507	0.0	731
ABCDEF_37741	UniRef50_P37122	0.0	Normal: 12	760	9	24	34	576	BlastAnno: 1197	UniRef50_P37122	505	65.70	379	129	1	1195	59	127	504	0.0	526
ABCDEF_35618	UniRef50_O48928	0.0	Normal: 28	2648	17	142	1234	902	BlastAnno: 1746	UniRef50_O48928	513	66.40	506	168	2	1649	132	4	507	0.0	701
ABCDEF_24522	UniRef50_P37122	0.0	Normal: 334	2190	561	268	1391	2459	BlastAnno: 2075	UniRef50_P37122	505	61.52	460	176	1	1544	165	47	505	0.0	612
ABCDEF_14592	UniRef50_O23066	0.0	Normal: 1514	402	2245	7837	4742	1248	BlastAnno: 2378	UniRef50_O23066	553	72.71	546	140	4	2111	489	1	542	0.0	821
ABCDEF_14512	UniRef50_Q9FG65	0.0	Normal: 261	872	212	291	175	789	BlastAnno: 1878	UniRef50_Q9FG65	502	56.89	501	197	9	1738	266	11	502	0.0	532
ABCDEF_44487	UniRef50_Q940V4	0.0	Normal: 3	41	53	501	262	542	BlastAnno: 1712	UniRef50_Q940V4	465	69.37	382	116	1	38	1180	14	395	0.0	572
ABCDEF_17723	UniRef50_P37122	0.0	Normal: 240	1698	219	768	592	794	BlastAnno: 1757	UniRef50_P37122	505	63.40	459	166	2	228	1601	47	504	0.0	617
ABCDEF_37901	UniRef50_Q42569	0.0	Normal: 30	149	86	255	195	255	BlastAnno: 1246	UniRef50_Q42569	472	78.27	359	73	2	64	1140	43	396	0.0	567
ABCDEF_7260	UniRef50_O81117	0.0	Normal: 314	1085	466	3248	2575	1360	BlastAnno: 1768	UniRef50_O81117	514	59.92	474	188	1	145	1566	43	514	0.0	615
ABCDEF_40548	UniRef50_P37122	0.0	Normal: 2756	2467	2752	1072	3120	1735	BlastAnno: 1947	UniRef50_P37122	505	59.72	504	202	1	241	1749	1	504	0.0	636
ABCDEF_44485	UniRef50_Q940V4	0.0	Normal: 3	55	63	574	338	660	BlastAnno: 1607	UniRef50_Q940V4	465	68.58	452	141	1	42	1394	14	465	0.0	673
ABCDEF_40552	UniRef50_P37122	0.0	Normal: 2397	1870	2304	1249	10428	2291	BlastAnno: 1812	UniRef50_P37122	505	60.12	504	200	1	108	1616	1	504	0.0	640
ABCDEF_37902	UniRef50_Q42569	0.0	Normal: 84	524	254	725	670	1049	BlastAnno: 1812	UniRef50_Q42569	472	77.47	435	92	3	1675	371	43	471	0.0	681
ABCDEF_40556	UniRef50_P37122	0.0	Normal: 1907	2365	2163	1319	3678	3308	BlastAnno: 1944	UniRef50_P37122	505	61.04	498	193	1	264	1754	1	498	0.0	635
ABCDEF_40549	UniRef50_P37122	0.0	Normal: 1574	1962	1826	1150	2882	2723	BlastAnno: 1845	UniRef50_P37122	505	60.52	504	198	1	1754	246	1	504	0.0	638
ABCDEF_20301	UniRef50_O81117	0.0	Normal: 89	308	123	176	389	249	BlastAnno: 1637	UniRef50_O81117	514	64.04	470	164	3	1498	98	47	514	0.0	586
ABCDEF_3882	UniRef50_Q42569	0.0	Normal: 84	243	257	786	1561	182	BlastAnno: 1854	UniRef50_Q42569	472	72.51	422	107	1	1852	587	59	471	0.0	615
ABCDEF_20703	UniRef50_O49858	0.0	Normal: 115	636	20	0	0	597	BlastAnno: 1815	UniRef50_O49858	527	50.10	523	248	6	48	1607	12	524	0.0	533
ABCDEF_18295	UniRef50_O22203	0.0	Normal: 3667	1934	4977	10491	4588	1917	BlastAnno: 2006	UniRef50_O22203	508	74.85	493	123	1	214	1692	17	508	0.0	803
ABCDEF_48724	UniRef50_O23066	0.0	Normal: 104	11518	163	2083	3464	6356	BlastAnno: 2162	UniRef50_O23066	553	72.96	540	143	3	227	1840	1	539	0.0	807
ABCDEF_37738	UniRef50_P37122	0.0	Normal: 26	2447	19	30	33	993	BlastAnno: 1837	UniRef50_P37122	505	64.30	479	169	2	1685	249	28	504	0.0	639
1.14.11.-(Oxidoreductases;)																			
ABCDEF_37086	UniRef50_Q8GUI6	0.0	Normal: 592	429	529	801	742	784	BlastAnno: 3943	UniRef50_Q8GUI6	954	56.60	553	227	4	613	2244	38	586	0.0	643
ABCDEF_51324	UniRef50_Q8GUI6	0.0	Normal: 461	353	534	1098	841	490	BlastAnno: 3289	UniRef50_Q8GUI6	954	60.79	584	214	5	2989	1262	12	588	0.0	698
2.1.1.-(Transferases;)																			
ABCDEF_20806	UniRef50_Q94II3	0.0	Normal: 700	332	876	1458	1997	929	BlastAnno: 2275	UniRef50_Q94II3	600	73.57	594	153	4	2119	341	1	591	0.0	907
ABCDEF_45802	UniRef50_Q9ZPH9	0.0	Normal: 3543	3348	4296	9797	10830	5017	BlastAnno: 2642	UniRef50_Q9ZPH9	633	53.90	603	261	7	2216	456	23	624	0.0	676
ABCDEF_20070	UniRef50_Q944R7	0.0	Normal: 301	141	265	207	185	187	BlastAnno: 2675	UniRef50_Q944R7	724	58.28	719	275	9	127	2265	13	712	0.0	840
ABCDEF_34956	UniRef50_Q94II3	0.0	Normal: 1120	7269	2475	16033	22219	21461	BlastAnno: 2676	UniRef50_Q94II3	600	73.77	610	146	4	404	2230	1	597	0.0	957
ABCDEF_17121	UniRef50_Q9ZPH9	0.0	Normal: 124	77	109	284	199	132	BlastAnno: 2066	UniRef50_Q9ZPH9	633	53.24	601	259	8	161	1903	25	623	0.0	644
ABCDEF_6524	UniRef50_Q9ZPH9	0.0	Normal: 471	296	490	753	870	730	BlastAnno: 2602	UniRef50_Q9ZPH9	633	50.89	619	290	5	2221	404	6	623	0.0	659
ABCDEF_56017	UniRef50_Q9ZPH9	0.0	Normal: 302	167	286	596	748	529	BlastAnno: 2384	UniRef50_Q9ZPH9	633	51.37	619	287	5	213	2030	6	623	0.0	663
ABCDEF_44169	UniRef50_Q9SIZ3	0.0	Normal: 179	158	273	705	685	229	BlastAnno: 2300	UniRef50_Q9SIZ3	589	62.01	308	108	4	1219	296	285	583	0.0	394
ABCDEF_6521	UniRef50_Q9ZPH9	0.0	Normal: 339	198	314	641	767	582	BlastAnno: 2511	UniRef50_Q9ZPH9	633	51.37	619	287	5	2172	355	6	623	0.0	663
ABCDEF_44172	UniRef50_Q9SIZ3	0.0	Normal: 204	184	287	767	741	242	BlastAnno: 2317	UniRef50_Q9SIZ3	589	68.15	449	348	134	4	1637	291	144	583	0.0	641
ABCDEF_199	UniRef50_Q9SU94	0.0	Normal: 1076	275	1009	558	1247	306	BlastAnno: 1769	UniRef50_Q9SU94	390	78.45	354	74	1	284	1324	43	390	0.0	584
ABCDEF_52252	UniRef50_Q84W92	0.0	Normal: 1658	720	1653	1980	2289	1601	BlastAnno: 2170	UniRef50_Q84W92	535	72.49	469	120	3	409	1791	34	501	0.0	703
ABCDEF_51404	UniRef50_A3BMN9	0.0	Normal: 864	188	716	337	631	326	BlastAnno: 2192	UniRef50_A3BMN9	620	50.50	604	243	9	2008	251	55	620	0.0	555
ABCDEF_21386	UniRef50_Q94AI4	0.0	Normal: 2326	1250	2220	4294	2920	1983	BlastAnno: 3701	UniRef50_Q94AI4	775	47.27	842	332	16	567	2978	8	775	0.0	577
ABCDEF_35101	UniRef50_Q9MAT5	0.0	Normal: 1440	420	1268	576	1136	850	BlastAnno: 1634	UniRef50_Q9MAT5	383	69.03	352	109	0	1449	394	32	383	0.0	550
ABCDEF_960	UniRef50_Q9LN50	0.0	Normal: 3504	13198	3609	11156	19651	12155	BlastAnno: 2626	UniRef50_Q9LN50	724	49.14	525	246	5	883	2451	219	724	0.0	570
ABCDEF_48082	UniRef50_Q9LN50	0.0	Normal: 410	445	467	870	993	454	BlastAnno: 2340	UniRef50_Q9LN50	724	70.25	521	155	0	710	2272	204	724	0.0	821
ABCDEF_10473	UniRef50_Q08A71	0.0	Normal: 374	187	332	425	456	314	BlastAnno: 1764	UniRef50_Q08A71	435	79.23	366	75	1	344	1438	70	435	0.0	575
ABCDEF_7295	UniRef50_Q9ZPH9	0.0	Normal: 25	54	30	81	26	329	BlastAnno: 1191	UniRef50_Q9ZPH9	633	69.23	403	110	4	1191	22	225	626	0.0	599
ABCDEF_4160	UniRef50_Q94II3	0.0	Normal: 189	0	210	71	17	72	BlastAnno: 1424	UniRef50_Q94II3	600	77.28	449	101	1	1424	81	143	591	0.0	758
ABCDEF_44857	UniRef50_Q9ZPH9	0.0	Normal: 101	224	103	299	588	350	BlastAnno: 1582	UniRef50_Q9ZPH9	633	72.06	519	143	1	1571	21	114	632	0.0	822

	ABCDEF_71541	UniRef50_Q9SIZ3	0.0	Normal: 187	167	296	765	724	237	BlastAnno: 2268	UniRef50_Q9SIZ3	589	62.01	308	108	4	1210	287	285	583	0.0	394
	ABCDEF_44171	UniRef50_Q9SIZ3	0.0	Normal: 214	196	312	827	802	251	BlastAnno: 2339	UniRef50_Q9SIZ3	589	64.78	548	182	5	1978	335	47	583	0.0	717
	ABCDEF_20805	UniRef50_Q94II3	0.0	Normal: 674	344	885	1417	2194	963	BlastAnno: 2400	UniRef50_Q94II3	600	78.21	436	94	1	879	2183	156	591	0.0	734
	ABCDEF_18210	UniRef50_Q9SIZ3	0.0	Normal: 5584	3714	7379	17006	21892	7546	BlastAnno: 3199	UniRef50_Q9SIZ3	589	50.00	576	269	7	2145	424	27	585	0.0	574
	ABCDEF_6526	UniRef50_Q9ZPH9	0.0	Normal: 627	185	837	2717	2815	1530	BlastAnno: 2611	UniRef50_Q9ZPH9	633	54.76	599	259	5	462	2222	25	623	0.0	689
	ABCDEF_6525	UniRef50_Q9ZPH9	0.0	Normal: 602	165	760	2369	2605	1400	BlastAnno: 2545	UniRef50_Q9ZPH9	633	54.92	599	258	5	431	2191	25	623	0.0	689
	ABCDEF_6523	UniRef50_Q9ZPH9	0.0	Normal: 314	175	304	641	784	552	BlastAnno: 2312	UniRef50_Q9ZPH9	633	52.42	599	272	4	201	1958	25	623	0.0	663
	ABCDEF_67393	UniRef50_Q9ZPH9	0.0	Normal: 434	245	444	608	768	655	BlastAnno: 2346	UniRef50_Q9ZPH9	633	51.92	599	275	4	232	1989	25	623	0.0	659
	ABCDEF_3579	UniRef50_O14744	0.0	Normal: 1361	326	984	568	791	440	BlastAnno: 2369	UniRef50_O14744	637	47.00	634	294	10	267	2120	30	637	0.0	553
2.4.1.202(2,4-dihydroxy-7-methoxy-2H-1,4-benzoxazin-3(4H)-one 2-D-glucosyltransferase;)																						
	No Correspond Unigene																					
4.1.2.8(indole-3-glycerol-phosphate lyase;)																						
	ABCDEF_1123	UniRef50_P42390	1e-108	Normal: 758	562	1072	1454	1845	1821	BlastAnno: 1186	UniRef50_P42390	347	59.77	261	101	1	975	193	90	346	1e-108	332
>00410 beta-Alanine metabolism																						
1.1.1.59(3-hydroxypropionate dehydrogenase)																						
	No Correspond Unigene																					
1.2.1.15(malonate-semialdehyde dehydrogenase)																						
	No Correspond Unigene																					
1.2.1.18(malonate-semialdehyde dehydrogenase (acetylating);)																						
	ABCDEF_18282	UniRef50_Q02252	0.0	Normal: 1599	1252	1838	3819	3718	2949	BlastAnno: 2054	UniRef50_Q02252	535	60.20	510	197	2	345	1856	23	532	0.0	653
1.2.1.19(aminobutyraldehyde dehydrogenase;)																						
	No Correspond Unigene																					
1.2.1.3(aldehyde dehydrogenase (NAD+);)																						
	ABCDEF_52483	UniRef50_Q70E96	0.0	Normal: 28	851	41	0	3965	420	BlastAnno: 1750	UniRef50_Q70E96	484	54.56	482	219	0	107	1552	3	484	0.0	548
	ABCDEF_17638	UniRef50_Q70E96	0.0	Normal: 580	718	749	2424	1317	775	BlastAnno: 1970	UniRef50_Q70E96	484	62.21	471	177	1	283	1692	2	472	0.0	625
	ABCDEF_403	UniRef50_Q8W033	0.0	Normal: 69	355	98	39	223	1111	BlastAnno: 1749	UniRef50_Q8W033	550	65.96	473	161	0	1523	105	74	546	0.0	625
	ABCDEF_45143	UniRef50_Q0WSF1	0.0	Normal: 1445	874	1200	1789	1940	1504	BlastAnno: 2450	UniRef50_Q0WSF1	596	79.97	594	115	2	2220	451	1	594	0.0	1015
1.3.1.1(dihydrouracil dehydrogenase (NAD+);)																						
	ABCDEF_27630	UniRef50_Q01P61	9e-82	Normal: 448	273	523	806	1011	577	BlastAnno: 1916	UniRef50_Q01P61	446	41.85	368	198	4	1435	338	2	355	9e-82	273
1.3.1.2(dihydropyrimidine dehydrogenase (NADP+);)																						
	ABCDEF_27630	UniRef50_A9GKC4	2e-84	Normal: 448	273	523	806	1011	577	BlastAnno: 1916	UniRef50_A9GKC4	462	42.13	356	190	5	1435	374	2	343	2e-84	280
1.3.99.3(acyl-CoA dehydrogenase;)																						
	ABCDEF_90276	UniRef50_P11310	4e-115	Normal: 0	0	0	0	0	80	BlastAnno: 757	UniRef50_P11310	421	66.14	251	79	2	3	755	80	324	4e-115	345
1.4.3.21(primary-amine oxidase;)																						
	ABCDEF_3522	UniRef50_Q8H1H9	0.0	Normal: 405	723	295	151	922	46	BlastAnno: 2086	UniRef50_Q8H1H9	712	64.54	595	204	6	2	1774	116	707	0.0	811
	ABCDEF_3521	UniRef50_Q8H1H9	0.0	Normal: 322	637	263	137	709	53	BlastAnno: 1768	UniRef50_Q8H1H9	712	68.24	510	156	5	1630	113	200	707	0.0	744
1.5.99.6(spermidine dehydrogenase;)																						
	No Correspond Unigene																					
2.5.1.16(spermidine synthase;)																						
	ABCDEF_7812	UniRef50_Q9ZUB3	0.0	Normal: 1661	1302	2150	4493	6957	4671	BlastAnno: 1757	UniRef50_Q9ZUB3	334	81.44	334	49	3	1326	331	11	333	0.0	552
2.5.1.22(spermine synthase;)																						
	No Correspond Unigene																					
2.6.1.18(beta-alanine---pyruvate transaminase;)																						
	No Correspond Unigene																					
2.6.1.19(4-aminobutyrate transaminase;)																						
	ABCDEF_3775	UniRef50_B8CY58	4e-110	Normal: 473	159	414	817	611	782	BlastAnno: 1801	UniRef50_B8CY58	437	45.37	432	223	7	1570	293	6	430	4e-110	346
2.6.1.55(taurine---2-oxoglutarate transaminase;)																						
	No Correspond Unigene																					
2.8.3.3(malonate CoA-transferase;)																						
	No Correspond Unigene																					
3.1.2.4(3-hydroxyisobutyryl-CoA hydrolase;)																						
	ABCDEF_56804	UniRef50_Q6NVY1	6e-77	Normal: 160	146	249	472	328	155	BlastAnno: 1439	UniRef50_Q6NVY1	386	40.45	356	199	7	257	1306	29	377	6e-77	254
3.4.13.20(beta-Ala-His dipeptidase;)																						
	No Correspond Unigene																					
3.4.13.3(Xaa-His dipeptidase;)																						
	No Correspond Unigene																					
3.4.13.4(Xaa-Arg dipeptidase;)																						
	No Correspond Unigene																					
3.4.13.5(Xaa-methyl-His dipeptidase;)																						
	No Correspond Unigene																					
3.5.1.21(N-acetyl-beta-alanine deacetylase)																						
	No Correspond Unigene																					
3.5.1.6(beta-ureidopropionase)																						
	ABCDEF_17031	UniRef50_Q9UBR1	1e-142	Normal: 273	642	328	641	1094	595	BlastAnno: 2413	UniRef50_Q9UBR1	384	58.49	383	153	3	2133	991	6	384	1e-142	434
3.5.2.2(dihydropyrimidinase;)																						
	ABCDEF_44368	UniRef50_Q9FMP3	0.0	Normal: 229	259	223	421	456	369	BlastAnno: 1773	UniRef50_Q9FMP3	531	84.83	501	76	0	1554	52	27	527	0.0	864
	ABCDEF_44370	UniRef50_Q9FMP3	0.0	Normal: 159	173	177	315	341	286	BlastAnno: 1537	UniRef50_Q9FMP3	531	85.98	435	61	0	1363	59	93	527	0.0	754
4.1.1.11(aspartate 1-decarboxylase;)																						
	ABCDEF_80024	UniRef50_Q24062	1e-13	Normal: 0	0	0	0	0	10	BlastAnno: 237	UniRef50_Q24062	575	39.73	73	44	0	18	236	482	554	1e-13	71.2
4.1.1.15(glutamate decarboxylase;)																						
	ABCDEF_11564	UniRef50_Q42472	0.0	Normal: 1009	2635	783	1023	2784	3375	BlastAnno: 2048	UniRef50_Q42472	494	80.20	500	93	3	224	1723	1	494	0.0	846
	ABCDEF_38368	UniRef50_Q42472	0.0	Normal: 893	2322	667	877	2443	2996	BlastAnno: 2255	UniRef50_Q42472	494	86.09	338	46	1	2032	1019	1	337	0.0	624

ABCDEF_38369	UniRef50_Q42472	0.0	Normal: 802	2006	619	863	2096	2472	BlastAnno: 2237	UniRef50_Q42472	494	89.35	310	33	0	1833	904	28	337	0.0	593
ABCDEF_38367	UniRef50_Q42472	0.0	Normal: 952	2438	773	996	2638	3216	BlastAnno: 2145	UniRef50_Q42472	494	81.99	472	80	2	1741	326	28	494	0.0	815
4.1.1.9(malonyl-CoA decarboxylase;)																					
ABCDEF_47117	UniRef50_C4KDD9	1e-90	Normal: 122	80	140	109	159	107	BlastAnno: 1629	UniRef50_C4KDD9	460	43.84	349	178	5	1266	226	124	456	1e-90	294
4.2.1.17(enoyl-CoA hydratase;)																					
ABCDEF_21259	UniRef50_Q8W1L6	0.0	Normal: 272	276	284	384	326	305	BlastAnno: 2641	UniRef50_Q8W1L6	726	47.57	721	375	3	114	2267	6	726	0.0	633
ABCDEF_80649	UniRef50_Q8W1L6	0.0	Normal: 527	841	443	1308	1321	1188	BlastAnno: 2586	UniRef50_Q8W1L6	726	60.39	712	281	1	2403	268	1	711	0.0	818
ABCDEF_466	UniRef50_Q8W1L6	0.0	Normal: 1014	1690	1094	1677	2438	2080	BlastAnno: 2453	UniRef50_Q8W1L6	726	71.24	699	201	0	2368	272	7	705	0.0	954
ABCDEF_54834	UniRef50_Q8W1L6	0.0	Normal: 229	220	237	298	270	261	BlastAnno: 2405	UniRef50_Q8W1L6	726	47.52	606	315	3	274	2082	121	726	0.0	580
ABCDEF_34756	UniRef50_Q8W1L6	0.0	Normal: 776	726	768	2922	4039	1079	BlastAnno: 2469	UniRef50_Q8W1L6	726	68.93	708	217	2	2396	273	7	711	0.0	957
ABCDEF_4198	UniRef50_Q8W1L6	0.0	Normal: 609	929	532	1401	1568	1297	BlastAnno: 2667	UniRef50_Q8W1L6	726	60.45	713	281	1	2463	325	1	712	0.0	820
4.2.1.27(acetylenecarboxylate hydratase;)																					
No Correspond Unigene																					
4.3.1.6(beta-alanyl-CoA ammonia-lyase;)																					
No Correspond Unigene																					
6.3.2.1(pantoate---beta-alanine ligase;)																					
ABCDEF_17131	UniRef50_B9S544	3e-153	Normal: 493	361	542	624	685	667	BlastAnno: 2119	UniRef50_B9S544	325	67.08	322	95	2	1855	920	5	325	3e-153	456
6.3.2.11(carnosine synthase;)																					
No Correspond Unigene																					
>00430 Taurine and hypotaurine metabolism																					
1.13.11.19(cysteamine dioxygenase;)																					
No Correspond Unigene																					
1.13.11.20(cysteine dioxygenase;)																					
No Correspond Unigene																					
1.14.11.17(taurine dioxygenase;)																					
No Correspond Unigene																					
1.2.1.73(sulfoacetaldehyde dehydrogenase;)																					
No Correspond Unigene																					
1.4.1.1(alanine dehydrogenase;)																					
No Correspond Unigene																					
1.4.2.-(Oxidoreductases;)																					
No Correspond Unigene																					
1.8.1.3(hypotaurine dehydrogenase)																					
No Correspond Unigene																					
2.3.1.65(bile acid-CoA:amino acid N-acyltransferase;)																					
No Correspond Unigene																					
2.3.1.8(phosphate acetyltransferase;)																					
ABCDEF_10009	UniRef50_B6KAY1	6e-23	Normal: 14	16	19	16	34	38	BlastAnno: 693	UniRef50_B6KAY1	1296	48.51	101	51	1	154	453	1128	1228	6e-23	104
2.3.2.2(gamma-glutamyltransferase;)																					
ABCDEF_27037	UniRef50_Q60928	4e-127	Normal: 382	258	428	509	999	336	BlastAnno: 2914	UniRef50_Q60928	568	41.49	552	291	11	330	1961	39	566	4e-127	405
2.3.3.15(sulfoacetaldehyde acetyltransferase;)																					
No Correspond Unigene																					
2.6.1.77(taurine---pyruvate aminotransferase;)																					
No Correspond Unigene																					
2.7.2.1(acetate kinase;)																					
No Correspond Unigene																					
2.7.3.4(taurocyamine kinase;)																					
ABCDEF_88981	UniRef50_P16641	5e-90	Normal: 0	0	0	0	0	236	BlastAnno: 1022	UniRef50_P16641	675	45.24	336	174	4	3	995	22	352	5e-90	291
4.1.1.15(glutamate decarboxylase;)																					
ABCDEF_11564	UniRef50_Q42472	0.0	Normal: 1009	2635	783	1023	2784	3375	BlastAnno: 2048	UniRef50_Q42472	494	80.20	500	93	3	224	1723	1	494	0.0	846
ABCDEF_38368	UniRef50_Q42472	0.0	Normal: 893	2322	667	877	2443	2996	BlastAnno: 2255	UniRef50_Q42472	494	86.09	338	46	1	2032	1019	1	337	0.0	624
ABCDEF_38369	UniRef50_Q42472	0.0	Normal: 802	2006	619	863	2096	2472	BlastAnno: 2237	UniRef50_Q42472	494	89.35	310	33	0	1833	904	28	337	0.0	593
ABCDEF_38367	UniRef50_Q42472	0.0	Normal: 952	2438	773	996	2638	3216	BlastAnno: 2145	UniRef50_Q42472	494	81.99	472	80	2	1741	326	28	494	0.0	815
4.1.1.29(sulfinoalanine decarboxylase;)																					
ABCDEF_80024	UniRef50_B7QID8	2e-33	Normal: 0	0	0	0	0	10	BlastAnno: 237	UniRef50_B7QID8	238	66.67	75	24	1	15	236	143	217	2e-33	122
4.4.1.10(cysteine lyase;)																					
No Correspond Unigene																					
>00440 Phosphonate and phosphinate metabolism																					
1.1.99.-(Oxidoreductases;)																					
ABCDEF_20521	UniRef50_Q8N465	0.0	Normal: 406	283	302	461	568	486	BlastAnno: 2144	UniRef50_Q8N465	521	58.16	478	194	5	354	1784	47	519	0.0	564
2.6.1.-(Transferases;)																					
ABCDEF_3716	UniRef50_Q9LQ10	0.0	Normal: 224	353	140	47	76	606	BlastAnno: 2230	UniRef50_Q9LQ10	557	61.35	533	189	8	250	1812	30	557	0.0	609
ABCDEF_37756	UniRef50_Q9LQ10	0.0	Normal: 197	168	149	152	186	490	BlastAnno: 1840	UniRef50_Q9LQ10	557	57.49	534	196	7	1638	109	28	554	0.0	619
2.6.1.37(2-aminoethylphosphonate---pyruvate transaminase;)																					
No Correspond Unigene																					
2.7.7.14(ethanolamine-phosphate cytidylyltransferase;)																					
ABCDEF_38643	UniRef50_Q8IP80	9e-107	Normal: 795	331	818	723	945	948	BlastAnno: 1682	UniRef50_Q8IP80	381	46.70	364	181	5	233	1318	18	370	9e-107	334
2.7.7.15(choline-phosphate cytidylyltransferase;)																					
ABCDEF_32202	UniRef50_Q42555	2e-147	Normal: 520	385	514	732	930	652	BlastAnno: 1213	UniRef50_Q42555	331	76.01	271	60	2	1064	255	11	277	2e-147	430
2.7.8.-(Transferases;)																					
ABCDEF_40506	UniRef50_C0QDU7	8e-33	Normal: 129	124	156	162	222	178	BlastAnno: 1387	UniRef50_C0QDU7	251	32.58	267	143	8	1342	542	10	239	8e-33	131
2.7.8.1(ethanolaminephosphotransferase;)																					

No Correspond Unigene																					
2.7.8.2(diacylglycerol cholinephosphotransferase;)																					
ABCDEF_32672	UniRef50_Q8T0S3	2e-07	Normal: 201	91	155	307	429	270	BlastAnno: 1067	UniRef50_Q8T0S3	415	23.03	178	119	3	177	677	222	392	2e-07	58.2
3.11.1.1(phosphonoacetaldehyde hydrolase;)																					
No Correspond Unigene																					
3.11.1.2(phosphonoacetate hydrolase)																					
No Correspond Unigene																					
4.1.1.82(phosphonopyruvate decarboxylase;)																					
No Correspond Unigene																					
4.99.1.-(Lyases;)																					
No Correspond Unigene																					
5.4.2.9(phosphoenolpyruvate mutase;)																					
No Correspond Unigene																					
>00450 Selenocompound metabolism																					
1.8.1.9(thioredoxin-disulfide reductase;)																					
ABCDEF_41899	UniRef50_P29509	6e-131	Normal: 922	343	782	913	1401	860	BlastAnno: 1495	UniRef50_P29509	319	63.55	321	108	3	1305	358	2	318	6e-131	391
1.97.1.9(selenate reductase)																					
No Correspond Unigene																					
2.1.1.-(Transferases;)																					
ABCDEF_20806	UniRef50_Q94II3	0.0	Normal: 700	332	876	1458	1997	929	BlastAnno: 2275	UniRef50_Q94II3	600	73.57	594	153	4	2119	341	1	591	0.0	907
ABCDEF_45802	UniRef50_Q9ZPH9	0.0	Normal: 3543	3348	4296	9797	10830	5017	BlastAnno: 2642	UniRef50_Q9ZPH9	633	53.90	603	261	7	2216	456	23	624	0.0	676
ABCDEF_20070	UniRef50_Q944R7	0.0	Normal: 301	141	265	207	185	187	BlastAnno: 2675	UniRef50_Q944R7	724	58.28	719	275	9	127	2265	13	712	0.0	840
ABCDEF_34956	UniRef50_Q94II3	0.0	Normal: 1120	7269	2475	16033	22219	21461	BlastAnno: 2676	UniRef50_Q94II3	600	73.77	610	146	4	404	2230	1	597	0.0	957
ABCDEF_17121	UniRef50_Q9ZPH9	0.0	Normal: 124	77	109	284	199	132	BlastAnno: 2066	UniRef50_Q9ZPH9	633	53.24	601	259	8	161	1903	25	623	0.0	644
ABCDEF_6524	UniRef50_Q9ZPH9	0.0	Normal: 471	296	490	753	870	730	BlastAnno: 2602	UniRef50_Q9ZPH9	633	50.89	619	290	5	2221	404	6	623	0.0	659
ABCDEF_56017	UniRef50_Q9ZPH9	0.0	Normal: 302	167	286	596	748	529	BlastAnno: 2384	UniRef50_Q9ZPH9	633	51.37	619	287	5	213	2030	6	623	0.0	663
ABCDEF_44169	UniRef50_Q9SIZ3	0.0	Normal: 179	158	273	705	685	229	BlastAnno: 2300	UniRef50_Q9SIZ3	589	62.01	308	108	4	1219	296	285	583	0.0	394
ABCDEF_6521	UniRef50_Q9ZPH9	0.0	Normal: 339	198	314	641	767	582	BlastAnno: 2511	UniRef50_Q9ZPH9	633	51.37	619	287	5	2172	355	6	623	0.0	663
ABCDEF_44172	UniRef50_Q9SIZ3	0.0	Normal: 204	184	287	767	741	242	BlastAnno: 2317	UniRef50_Q9SIZ3	589	68.15	449	134	4	1637	291	144	583	0.0	641
ABCDEF_199	UniRef50_Q9SU94	0.0	Normal: 1076	275	1009	558	1247	306	BlastAnno: 1769	UniRef50_Q9SU94	390	78.45	348	74	1	284	1324	43	390	0.0	584
ABCDEF_52252	UniRef50_Q84W92	0.0	Normal: 1658	720	1653	1980	2289	1601	BlastAnno: 2170	UniRef50_Q84W92	535	72.49	469	120	3	409	1791	34	501	0.0	703
ABCDEF_51404	UniRef50_A3BMN9	0.0	Normal: 864	188	716	337	631	326	BlastAnno: 2192	UniRef50_A3BMN9	620	50.50	604	243	9	2008	251	55	620	0.0	555
ABCDEF_21386	UniRef50_Q94AI4	0.0	Normal: 2326	1250	2220	4294	2920	1983	BlastAnno: 3701	UniRef50_Q94AI4	775	47.27	842	332	16	567	2978	8	775	0.0	577
ABCDEF_35101	UniRef50_Q9MAT5	0.0	Normal: 1440	420	1268	576	1136	850	BlastAnno: 1634	UniRef50_Q9MAT5	383	69.03	352	109	0	1449	394	32	383	0.0	550
ABCDEF_960	UniRef50_Q9LN50	0.0	Normal: 3504	13198	3609	11156	19651	12155	BlastAnno: 2626	UniRef50_Q9LN50	724	49.14	525	246	5	883	2451	219	724	0.0	570
ABCDEF_48082	UniRef50_Q9LN50	0.0	Normal: 410	445	467	870	993	454	BlastAnno: 2340	UniRef50_Q9LN50	724	70.25	521	155	0	710	2272	204	724	0.0	821
ABCDEF_10473	UniRef50_Q08A71	0.0	Normal: 374	187	332	425	456	314	BlastAnno: 1764	UniRef50_Q08A71	435	79.23	366	75	1	344	1438	70	435	0.0	575
ABCDEF_7295	UniRef50_Q9ZPH9	0.0	Normal: 25	54	30	81	26	329	BlastAnno: 1191	UniRef50_Q9ZPH9	633	69.23	403	110	4	1191	22	225	626	0.0	599
ABCDEF_4160	UniRef50_Q94II3	0.0	Normal: 189	0	210	71	17	72	BlastAnno: 1424	UniRef50_Q94II3	600	77.28	449	101	1	1424	81	143	591	0.0	758
ABCDEF_44857	UniRef50_Q9ZPH9	0.0	Normal: 101	224	103	299	588	350	BlastAnno: 1582	UniRef50_Q9ZPH9	633	72.06	519	143	1	1571	21	114	632	0.0	822
ABCDEF_71541	UniRef50_Q9SIZ3	0.0	Normal: 187	167	296	765	724	237	BlastAnno: 2268	UniRef50_Q9SIZ3	589	62.01	308	108	4	1210	287	285	583	0.0	394
ABCDEF_44171	UniRef50_Q9SIZ3	0.0	Normal: 214	196	312	827	802	251	BlastAnno: 2339	UniRef50_Q9SIZ3	589	64.78	548	182	5	1978	335	47	583	0.0	717
ABCDEF_20805	UniRef50_Q94II3	0.0	Normal: 674	344	885	1417	2194	963	BlastAnno: 2400	UniRef50_Q94II3	600	78.21	436	94	1	879	2183	156	591	0.0	734
ABCDEF_18210	UniRef50_Q9SIZ3	0.0	Normal: 5584	3714	7379	17006	21892	7546	BlastAnno: 3199	UniRef50_Q9SIZ3	589	50.00	576	269	7	2145	424	27	585	0.0	574
ABCDEF_6526	UniRef50_Q9ZPH9	0.0	Normal: 627	185	837	2717	2815	1530	BlastAnno: 2611	UniRef50_Q9ZPH9	633	54.76	599	259	5	462	2222	25	623	0.0	689
ABCDEF_6525	UniRef50_Q9ZPH9	0.0	Normal: 602	165	760	2369	2605	1400	BlastAnno: 2545	UniRef50_Q9ZPH9	633	54.92	599	258	5	431	2191	25	623	0.0	689
ABCDEF_6523	UniRef50_Q9ZPH9	0.0	Normal: 314	175	304	641	784	552	BlastAnno: 2312	UniRef50_Q9ZPH9	633	52.42	599	272	4	201	1958	25	623	0.0	663
ABCDEF_67393	UniRef50_Q9ZPH9	0.0	Normal: 434	245	444	608	768	655	BlastAnno: 2346	UniRef50_Q9ZPH9	633	51.92	599	275	4	232	1989	25	623	0.0	659
ABCDEF_3579	UniRef50_014744	0.0	Normal: 1361	326	984	568	791	440	BlastAnno: 2369	UniRef50_014744	637	47.00	634	294	10	267	2120	30	637	0.0	553
2.1.1.12(methionine S-methyltransferase;)																					
ABCDEF_20706	UniRef50_Q9LTB2	0.0	Normal: 834	301	825	1664	2494	812	BlastAnno: 3611	UniRef50_Q9LTB2	1071	69.67	1075	316	3	131	3355	6	1070	0.0	1547
2.1.1.13(methionine synthase;)																					
No Correspond Unigene																					
2.1.1.14(5-methyltetrahydropteroyltriglutamate---homocysteine S-methyltransferase;)																					
ABCDEF_50765	UniRef50_050008	0.0	Normal: 1445	1155	1542	2724	6925	2394	BlastAnno: 1074	UniRef50_050008	765	92.61	284	21	0	1074	223	482	765	0.0	570
ABCDEF_50769	UniRef50_Q0S6X7	0.0	Normal: 1467	2688	2079	4226	6119	4451	BlastAnno: 2720	UniRef50_Q0S6X7	753	49.35	764	361	11	150	2420	9	753	0.0	724
ABCDEF_14591	UniRef50_050008	0.0	Normal: 175	108	133	170	218	262	BlastAnno: 1240	UniRef50_050008	765	80.39	413	81	0	2	1240	107	519	0.0	656
ABCDEF_50764	UniRef50_050008	0.0	Normal: 2132	1684	1899	4094	10442	2856	BlastAnno: 1414	UniRef50_050008	765	89.81	471	48	0	2	1414	106	576	0.0	858
ABCDEF_50758	UniRef50_Q0S6X7	0.0	Normal: 1269	1809	1557	3252	5371	3330	BlastAnno: 2581	UniRef50_Q0S6X7	753	49.48	764	360	11	100	2370	9	753	0.0	725
ABCDEF_50755	UniRef50_Q0S6X7	0.0	Normal: 1913	2156	2123	4237	9070	3515	BlastAnno: 2561	UniRef50_Q0S6X7	753	48.65	779	344	12	2451	181	9	753	0.0	736
ABCDEF_50754	UniRef50_Q0S6X7	0.0	Normal: 2088	3031	2671	5517	9518	4461	BlastAnno: 2646	UniRef50_Q0S6X7	753	48.65	779	344	12	2543	273	9	753	0.0	736
ABCDEF_50768	UniRef50_Q0S6X7	0.0	Normal: 1344	2423	1879	3794	5578	4174	BlastAnno: 2717	UniRef50_Q0S6X7	753	49.35	764	361	11	2557	287	9	753	0.0	724
ABCDEF_50766	UniRef50_Q0S6X7	0.0	Normal: 1494	2528	1939	4050	6543	4297	BlastAnno: 2694	UniRef50_Q0S6X7	753	49.48	764	360	11	150	2420	9	753	0.0	726
ABCDEF_50753	UniRef50_Q0S6X7	0.0	Normal: 1343	2204	1709	3527	5861	3896	BlastAnno: 2733	UniRef50_Q0S6X7	753	49.48	764	360	11	189	2459	9	753	0.0	725
ABCDEF_50756	UniRef50_Q0S6X7	0.0	Normal: 2120	3117	2695	5653	9766	4628	BlastAnno: 2655	UniRef50_Q0S6X7	753	48.65	779	344	12	2545	275	9	753	0.0	737
ABCDEF_57202	UniRef50_050008	0.0	Normal: 1505	1097	1164	2664	7110	2018	BlastAnno: 1257	UniRef50_050008	765	87.50	360	45	0	1081	2	1	360	0.0	637
ABCDEF_50760	UniRef50_Q0S6X7	0.0	Normal: 1514	2280	1931	3777	6908	3592	BlastAnno: 2637	UniRef50_Q0S6X7	753	48.65	779	344	12	81	2351	9	753	0.0	736
ABCDEF_50761	UniRef50_B6J6H8	0.0	Normal: 1122	1642	1538	3134	5532	2936	BlastAnno: 2009	UniRef50_B6J6H8	775	51.84	571	267	5	2006	300	195	759	0.0	572
ABCDEF_50767	UniRef50_050008	0.0	Normal: 786	1169	903	2117	3654	1574	BlastAnno: 1852	UniRef50_050008	765	88.56	568	65	0	149	1852	1	568	0.0	1018
ABCDEF_50759	UniRef50_Q0S6X7	0.0	Normal: 1559	2011	1766	3739	7427	3210	BlastAnno: 2518	UniRef50_Q0S6X7	753	48.65	779	344	12	81	2351	9	753	0.0	736
2.1.1.96(thioether S-methyltransferase;)																					
No Correspond Unigene																					

2.5.1.48(cystathionine gamma-synthase;)																							
	ABCDEF_31946	UniRef50_P55217	0.0	Normal: 3795	4731	4027	7276	7940	8571	BlastAnno: 1625	UniRef50_P55217	563	84.28	407	60	1	1399	179	161	563	0.0	719
2.6.1.-(Transferases;)																							
	ABCDEF_3716	UniRef50_Q9LQ10	0.0	Normal: 224	353	140	47	76	606	BlastAnno: 2230	UniRef50_Q9LQ10	557	61.35	533	189	8	250	1812	30	557	0.0	609
	ABCDEF_37756	UniRef50_Q9LQ10	0.0	Normal: 197	168	149	152	186	490	BlastAnno: 1840	UniRef50_Q9LQ10	557	57.49	534	196	7	1638	109	28	554	0.0	619
2.7.1.164(O-phosphoseryl-tRNASec kinase;)																							
	No Correspond Unigene																						
2.7.7.4(sulfate adenylyltransferase;)																							
	ABCDEF_10695	UniRef50_B6KN22	0.0	Normal: 1568	429	1310	1338	1351	785	BlastAnno: 3382	UniRef50_B6KN22	1008	53.54	1003	431	10	3195	274	5	1001	0.0	1061
	ABCDEF_48012	UniRef50_B6KBK6	0.0	Normal: 909	375	986	1182	1437	436	BlastAnno: 2606	UniRef50_B6KBK6	877	54.57	722	296	6	2316	247	148	869	0.0	791
	ABCDEF_38032	UniRef50_Q43870	0.0	Normal: 611	1036	642	1131	1509	1791	BlastAnno: 1947	UniRef50_Q43870	476	73.00	426	115	0	1636	359	42	467	0.0	661
	ABCDEF_31663	UniRef50_Q43870	0.0	Normal: 1022	1780	1065	1614	1663	3264	BlastAnno: 1893	UniRef50_Q43870	476	74.21	411	106	0	1723	491	57	467	0.0	663
	ABCDEF_31787	UniRef50_Q43870	0.0	Normal: 1065	1161	1322	2258	1986	3606	BlastAnno: 2042	UniRef50_Q43870	476	76.60	483	106	3	1809	361	1	476	0.0	771
	ABCDEF_10694	UniRef50_B6KN22	0.0	Normal: 1188	323	1053	981	1089	607	BlastAnno: 2848	UniRef50_B6KN22	1008	55.54	884	365	7	21	2588	118	1001	0.0	1015
2.7.9.3(selenide, water dikinase;)																							
	No Correspond Unigene																						
2.9.1.1(L-seryl-tRNASec selenium transferase;)																							
	No Correspond Unigene																						
2.9.1.2(O-phospho-L-seryl-tRNASec:L-selenocysteinyl-tRNA synthase;)																							
	No Correspond Unigene																						
4.4.1.1(cystathionine gamma-lyase;)																							
	ABCDEF_72071	UniRef50_Q55DV9	1e-80	Normal: 173	154	160	241	209	225	BlastAnno: 1568	UniRef50_Q55DV9	387	47.49	259	135	1	662	1435	126	384	1e-80	260
	ABCDEF_72428	UniRef50_Q55DV9	1e-80	Normal: 135	117	119	172	170	180	BlastAnno: 1527	UniRef50_Q55DV9	387	47.49	259	135	1	621	1394	126	384	1e-80	260
4.4.1.11(methionine gamma-lyase;)																							
	ABCDEF_31946	UniRef50_C0Z892	4e-92	Normal: 3795	4731	4027	7276	7940	8571	BlastAnno: 1625	UniRef50_C0Z892	402	44.84	339	185	2	1192	179	57	394	4e-92	296
4.4.1.16(selenocysteine lyase;)																							
	No Correspond Unigene																						
4.4.1.8(cystathionine beta-lyase;)																							
	ABCDEF_71418	UniRef50_P53780	0.0	Normal: 160	139	136	188	195	199	BlastAnno: 1556	UniRef50_P53780	464	72.22	450	117	2	86	1435	22	463	0.0	637
	ABCDEF_40739	UniRef50_P53780	0.0	Normal: 202	178	179	256	234	247	BlastAnno: 1653	UniRef50_P53780	464	71.24	459	124	2	130	1506	13	463	0.0	644
6.1.1.10(methionine---tRNA ligase;)																							
	ABCDEF_25209	UniRef50_B9RWD7	0.0	Normal: 2071	1224	1963	2436	2653	1743	BlastAnno: 2801	UniRef50_B9RWD7	678	75.61	492	111	4	1159	2634	196	678	0.0	692
	ABCDEF_16840	UniRef50_B9SR18	0.0	Normal: 1881	5139	1840	3474	6391	11913	BlastAnno: 3490	UniRef50_B9SR18	523	82.24	321	45	2	3305	2379	52	372	0.0	549
>00460 Cyanoamino acid metabolism																							
1.14.13.-(Oxidoreductases;)																							
	ABCDEF_45538	UniRef50_O23024	3e-156	Normal: 110	6	165	774	133	36	BlastAnno: 1258	UniRef50_O23024	437	58.18	385	151	3	3	1157	37	411	3e-156	457
1.14.13.41(tyrosine N-monooxygenase;)																							
	ABCDEF_7973	UniRef50_B9SBR6	2e-97	Normal: 150	194	5	0	0	1034	BlastAnno: 1715	UniRef50_B9SBR6	390	52.22	270	126	1	237	1046	73	339	2e-97	310
1.14.13.68(4-hydroxyphenylacetaldehyde oxime monooxygenase;)																							
	ABCDEF_61937	UniRef50_B9RAS4	9e-163	Normal: 0	200	0	0	3	13	BlastAnno: 1520	UniRef50_B9RAS4	527	49.18	486	220	7	120	1508	44	525	9e-163	481
1.4.99.5(glycine dehydrogenase (cyanide-forming);)																							
	No Correspond Unigene																						
2.1.2.1(glycine hydroxymethyltransferase;)																							
	ABCDEF_41184	UniRef50_C5KPS8	0.0	Normal: 509	437	722	1807	2474	561	BlastAnno: 2201	UniRef50_C5KPS8	607	62.56	446	164	2	1762	425	164	606	0.0	590
	ABCDEF_11573	UniRef50_P34897	0.0	Normal: 1903	1387	2092	2286	3254	2865	BlastAnno: 2080	UniRef50_P34897	504	60.18	457	170	4	502	1845	48	501	0.0	573
	ABCDEF_37629	UniRef50_Q9LM59	0.0	Normal: 412	460	499	689	962	979	BlastAnno: 2512	UniRef50_Q9LM59	599	66.00	597	187	5	369	2126	8	599	0.0	813
	ABCDEF_7224	UniRef50_C5KPS8	0.0	Normal: 360	5392	231	401	266	13616	BlastAnno: 2246	UniRef50_C5KPS8	607	61.88	446	167	2	1828	491	164	606	0.0	577
	ABCDEF_51490	UniRef50_Q9LM59	0.0	Normal: 497	502	504	755	1027	1332	BlastAnno: 2919	UniRef50_Q9LM59	599	64.38	584	197	2	578	2299	17	599	0.0	754
	ABCDEF_1167	UniRef50_Q9LM59	0.0	Normal: 5116	9848	6479	16268	26840	14685	BlastAnno: 1891	UniRef50_Q9LM59	599	62.07	464	171	1	1747	371	134	597	0.0	611
	ABCDEF_7223	UniRef50_C5KPS8	0.0	Normal: 395	5706	275	418	261	13912	BlastAnno: 2154	UniRef50_C5KPS8	607	61.88	446	167	2	1736	399	164	606	0.0	577
	ABCDEF_28355	UniRef50_P34897	0.0	Normal: 255	239	370	837	1312	358	BlastAnno: 1812	UniRef50_P34897	504	57.73	466	153	3	1810	416	81	503	0.0	553
2.3.2.2(gamma-glutamyltransferase;)																							
	ABCDEF_27037	UniRef50_Q60928	4e-127	Normal: 382	258	428	509	999	336	BlastAnno: 2914	UniRef50_Q60928	568	41.49	552	291	11	330	1961	39	566	4e-127	405
2.4.1.178(hydroxymandelonitrile glucosyltransferase;)																							
	No Correspond Unigene																						
2.4.1.63(linamarin synthase;)																							
	No Correspond Unigene																						
2.4.1.85(cyanohydrin beta-glucosyltransferase;)																							
	ABCDEF_63349	UniRef50_Q9SBL1	3e-26	Normal: 0	16	0	0	0	0	BlastAnno: 266	UniRef50_Q9SBL1	492	52.27	88	41	1	263	3	142	229	3e-26	106
3.2.1.117(amygdalin beta-glucosidase;)																							
	No Correspond Unigene																						
3.2.1.118(prunasin beta-glucosidase;)																							
	No Correspond Unigene																						
3.2.1.21(beta-glucosidase;)																							
	ABCDEF_24901	UniRef50_Q9LV34	0.0	Normal: 696	459	604	917	1518	3129	BlastAnno: 1698	UniRef50_Q9LV34	495	69.82	487	133	4	1576	131	17	494	0.0	696
	ABCDEF_3707	UniRef50_Q8L7J2	0.0	Normal: 55	614	80	478	1267	616	BlastAnno: 1950	UniRef50_Q8L7J2	521	68.39	484	143	2	365	1786	36	519	0.0	738
	ABCDEF_50691	UniRef50_O80690	0.0	Normal: 29	230	24	31	176	222	BlastAnno: 1737	UniRef50_O80690	527	56.05	512	197	4	1723	194	39	524	0.0	593
	ABCDEF_6978	UniRef50_Q9LV34	0.0	Normal: 132	59	114	74	74	76	BlastAnno: 1368	UniRef50_Q9LV34	495	65.54	444	136	5	1311	1	27	460	0.0	616
	ABCDEF_7820	UniRef50_B3H5Q1	0.0	Normal: 1199	176	254	446	677	1138	BlastAnno: 1699	UniRef50_B3H5Q1	521	55.14	486	190	4	97	1470	25	510	0.0	549
	ABCDEF_52515	UniRef50_O64882	0.0	Normal: 14	184	426	593	18106	970	BlastAnno: 1946	UniRef50_O64882	517	55.39	482	214	1	128	1570	32	513	0.0	574
	ABCDEF_50689	UniRef50_O80690	0.0	Normal: 42	287	26	32	235	244	BlastAnno: 1949	UniRef50_O80690	527	57.58	521	205	4	1711	194	5	524	0.0	608
	ABCDEF_24900	UniRef50_Q9LV34	0.0	Normal: 661	448	581	908	1414	2839	BlastAnno: 1678	UniRef50_Q9LV34	495	69.82	487	133	4	1557	112	17	494	0.0	696

ABCDEF_9495	UniRef50_O80690	0.0	Normal: 261	104	1144	3869	396	26	BlastAnno: 1691	UniRef50_O80690	527	52.12	495	196	7	220	1686	59	518	0.0	533
ABCDEF_9464	UniRef50_O80690	0.0	Normal: 733	82	1949	4567	726	6	BlastAnno: 2104	UniRef50_O80690	527	53.91	473	206	6	269	1669	59	525	0.0	542
3.5.1.1(asparaginase;)																					
ABCDEF_13981	UniRef50_Q0E667	3e-157	Normal: 943	1644	1236	3106	2580	499	BlastAnno: 1218	UniRef50_Q0E667	375	61.50	374	93	2	1108	140	2	375	3e-157	457
3.5.1.4(amidase;)																					
ABCDEF_3151	UniRef50_B9RF45	0.0	Normal: 113	275	72	56	28	1014	BlastAnno: 1850	UniRef50_B9RF45	500	68.47	425	133	1	131	1402	68	492	0.0	547
ABCDEF_81937	UniRef50_B9RF45	0.0	Normal: 101	261	68	53	28	927	BlastAnno: 1739	UniRef50_B9RF45	500	68.47	425	133	1	131	1402	68	492	0.0	546
3.5.5.1(nitrilase;)																					
ABCDEF_3989	UniRef50_P46011	0.0	Normal: 806	816	966	1114	1416	1512	BlastAnno: 1581	UniRef50_P46011	355	79.10	354	71	3	255	1310	3	355	0.0	579
3.5.5.4(cyanoalanine nitrilase;)																					
No Correspond Unigene																					
3.5.5.5(arylacetonitrilase)																					
No Correspond Unigene																					
4.1.2.10(No Name)																					
ABCDEF_11133	UniRef50_B9SN35	0.0	Normal: 2833	62	3938	10400	3516	782	BlastAnno: 2202	UniRef50_B9SN35	597	72.95	573	144	2	155	1873	35	596	0.0	873
ABCDEF_25039	UniRef50_B9SN35	0.0	Normal: 333	3731	279	134	100	6377	BlastAnno: 2149	UniRef50_B9SN35	597	57.89	532	222	2	354	1946	64	594	0.0	621
ABCDEF_78746	UniRef50_B9SN35	0.0	Normal: 1192	24	1802	4047	1606	405	BlastAnno: 1410	UniRef50_B9SN35	597	71.55	341	94	1	8	1030	245	582	0.0	528
4.1.2.11(hydroxymandelonitrile lyase;)																					
No Correspond Unigene																					
4.1.2.46(aliphatic (R)-hydroxynitrile lyase;)																					
ABCDEF_20657	UniRef50_P93243	9e-42	Normal: 62	131	56	86	228	188	BlastAnno: 726	UniRef50_P93243	422	39.32	206	108	5	59	625	11	216	9e-42	154
4.1.2.47(No Name)																					
ABCDEF_64621	UniRef50_P52705	6e-33	Normal: 0	31	0	0	0	0	BlastAnno: 357	UniRef50_P52705	258	59.55	89	36	0	273	7	5	93	6e-33	123
4.2.1.65(3-cyanoalanine hydratase;)																					
No Correspond Unigene																					
4.2.1.66(cyanide hydratase;)																					
No Correspond Unigene																					
4.2.1.84(nitrile hydratase;)																					
No Correspond Unigene																					
4.4.1.9(L-3-cyanoalanine synthase;)																					
No Correspond Unigene																					
4.99.1.5(aliphatic aldoxime dehydratase;)																					
No Correspond Unigene																					
4.99.1.7(phenylacetaldoxime dehydratase;)																					
No Correspond Unigene																					
6.3.1.1(aspartate---ammonia ligase;)																					
No Correspond Unigene																					
>00471 D-Glutamine and D-glutamate metabolism																					
1.4.1.3(glutamate dehydrogenase [NAD(P)+];)																					
ABCDEF_25196	UniRef50_Q43314	0.0	Normal: 271	16	291	548	795	8	BlastAnno: 1412	UniRef50_Q43314	411	78.84	378	80	0	1410	277	34	411	0.0	642
ABCDEF_52465	UniRef50_Q43314	0.0	Normal: 404	402	745	2021	3684	1711	BlastAnno: 1651	UniRef50_Q43314	411	79.81	411	83	0	210	1442	1	411	0.0	699
ABCDEF_24937	UniRef50_Q43314	0.0	Normal: 94	39	122	768	1065	114	BlastAnno: 1144	UniRef50_Q43314	411	87.97	349	42	0	1047	1	1	349	0.0	649
ABCDEF_32993	UniRef50_Q43314	0.0	Normal: 127	299	296	1133	320	898	BlastAnno: 1450	UniRef50_Q43314	411	89.78	411	42	0	1382	150	1	411	0.0	775
ABCDEF_42257	UniRef50_Q43314	0.0	Normal: 140	355	359	1267	415	1032	BlastAnno: 1644	UniRef50_Q43314	411	89.78	411	42	0	1454	222	1	411	0.0	775
1.4.3.15(D-glutamate(D-aspartate) oxidase;)																					
No Correspond Unigene																					
1.4.3.7(D-glutamate oxidase;)																					
No Correspond Unigene																					
2.3.2.1(D-glutamyltransferase;)																					
No Correspond Unigene																					
3.5.1.2(glutaminase;)																					
ABCDEF_56484	UniRef50_B0TVW4	4e-16	Normal: 9	0	0	0	0	0	BlastAnno: 289	UniRef50_B0TVW4	514	43.37	83	47	0	252	4	428	510	4e-16	79.0
3.5.1.35(D-glutaminase)																					
No Correspond Unigene																					
3.5.1.38(glutamin-(asparagin-)ase)																					
No Correspond Unigene																					
4.2.1.48(D-glutamate cyclase;)																					
No Correspond Unigene																					
5.1.1.10(amino-acid racemase;)																					
No Correspond Unigene																					
5.1.1.3(glutamate racemase)																					
ABCDEF_59512	UniRef50_B2JBV6	4e-21	Normal: 0	8	0	0	0	0	BlastAnno: 280	UniRef50_B2JBV6	1801	53.75	80	37	0	34	273	402	481	4e-21	94.4
6.3.2.8(UDP-N-acetylmuramate---L-alanine ligase;)																					
ABCDEF_59065	UniRef50_B8CWJ7	1e-17	Normal: 10	0	0	0	0	0	BlastAnno: 297	UniRef50_B8CWJ7	459	44.44	99	49	1	1	297	25	117	1e-17	82.8
6.3.2.9(UDP-N-acetylmuramoyl-L-alanine---D-glutamate ligase;)																					
ABCDEF_30168	UniRef50_Q1IKG8	6e-62	Normal: 85	52	102	178	150	135	BlastAnno: 1554	UniRef50_Q1IKG8	453	32.55	470	284	15	99	1493	2	443	6e-62	217
>00472 D-Arginine and D-ornithine metabolism																					
1.4.1.12(2,4-diaminopentanoate dehydrogenase;)																					
No Correspond Unigene																					
1.4.3.3(D-amino-acid oxidase;)																					
No Correspond Unigene																					
2.6.1.21(D-amino-acid transaminase;)																					

No Correspond Unigene
3.5.3.10(D-arginase)
No Correspond Unigene
5.1.1.10(amino-acid racemase;)
No Correspond Unigene
5.1.1.12(ornithine racemase)

ABCDEF_55628	UniRef50_Q3M1P5	7e-12	Normal: 10	0	0	0	0	0	BlastAnno: 214	UniRef50_Q3M1P5	2791	46.97	66	35	0	1	198	2638	2703	7e-12	66.6

5.1.1.9(arginine racemase)
No Correspond Unigene
5.4.3.5(D-ornithine 4,5-aminomutase;)
No Correspond Unigene

>00473 D-Alanine metabolism
2.6.1.21(D-amino-acid transaminase;)
No Correspond Unigene
2.6.1.41(D-methionine---pyruvate transaminase;)
No Correspond Unigene
5.1.1.1(alanine racemase;)
No Correspond Unigene
6.1.1.13(D-alanine---poly(phosphoribitol) ligase;)
No Correspond Unigene
6.3.2.16(D-alanine---alanyl-poly(glycerolphosphate) ligase;)
No Correspond Unigene
6.3.2.4(D-alanine---D-alanine ligase;)

ABCDEF_48167	UniRef50_Q11WL3	8e-43	Normal: 259	196	253	255	363	222	BlastAnno: 1996	UniRef50_Q11WL3	897	26.55	644	328	23	1991	183	350	889	8e-43	172

>00480 Glutathione metabolism
1.1.1.42(isocitrate dehydrogenase (NADP+);)

ABCDEF_49419	UniRef50_O75874	0.0	Normal: 5183	5221	5740	12435	20083	7734	BlastAnno: 1704	UniRef50_O75874	414	68.12	389	123	1	1491	328	10	398	0.0	566
ABCDEF_51976	UniRef50_O75874	0.0	Normal: 551	408	828	1291	1909	1088	BlastAnno: 1799	UniRef50_O75874	414	66.17	405	135	2	321	1529	10	414	0.0	568

1.1.1.43(phosphogluconate 2-dehydrogenase;)
No Correspond Unigene
1.1.1.44(phosphogluconate dehydrogenase (decarboxylating);)

ABCDEF_4346	UniRef50_B8B1K3	0.0	Normal: 2255	809	3044	3718	2317	1112	BlastAnno: 2100	UniRef50_B8B1K3	475	66.90	426	117	6	228	1502	2	404	0.0	552
ABCDEF_3900	UniRef50_B8B1K3	0.0	Normal: 1843	1331	2740	5139	3536	1587	BlastAnno: 1978	UniRef50_B8B1K3	475	74.88	414	85	4	1721	480	4	398	0.0	609
ABCDEF_38637	UniRef50_B8B1K3	0.0	Normal: 1655	1399	2681	4781	5856	2437	BlastAnno: 1907	UniRef50_B8B1K3	475	73.56	416	88	5	1711	467	4	398	0.0	588

1.1.1.49(glucose-6-phosphate dehydrogenase;)

ABCDEF_328	UniRef50_B9FFT3	0.0	Normal: 317	217	403	1011	1197	77	BlastAnno: 1924	UniRef50_B9FFT3	473	73.87	486	88	4	291	1748	26	472	0.0	732
ABCDEF_3893	UniRef50_B9FFT3	0.0	Normal: 420	902	719	2614	3215	1238	BlastAnno: 2131	UniRef50_B9FFT3	473	73.98	492	89	4	291	1766	20	472	0.0	737
ABCDEF_330	UniRef50_B9FFT3	0.0	Normal: 298	220	395	1039	1218	65	BlastAnno: 1870	UniRef50_B9FFT3	473	73.87	486	88	4	237	1694	26	472	0.0	732
ABCDEF_60678	UniRef50_C1FH53	0.0	Normal: 119	335	60	38	62	427	BlastAnno: 2449	UniRef50_C1FH53	552	67.94	262	83	1	1300	2082	288	549	0.0	398
ABCDEF_47341	UniRef50_C1FH53	0.0	Normal: 110	325	58	37	56	390	BlastAnno: 2674	UniRef50_C1FH53	552	68.16	380	120	1	1171	2307	170	549	0.0	569
ABCDEF_47349	UniRef50_Q8L743	0.0	Normal: 68	187	32	19	34	251	BlastAnno: 1382	UniRef50_Q8L743	599	86.94	314	41	0	80	1021	283	596	0.0	578
ABCDEF_329	UniRef50_B9FFT3	0.0	Normal: 301	232	393	1017	1193	65	BlastAnno: 1871	UniRef50_B9FFT3	473	73.87	486	88	4	238	1695	26	472	0.0	732
ABCDEF_62298	UniRef50_Q8L743	0.0	Normal: 69	208	35	25	40	262	BlastAnno: 1913	UniRef50_Q8L743	599	77.54	236	48	1	545	1252	111	341	0.0	381
ABCDEF_47347	UniRef50_C1FH53	0.0	Normal: 113	317	51	33	60	447	BlastAnno: 2638	UniRef50_C1FH53	552	63.92	413	146	2	533	1765	60	471	0.0	554
ABCDEF_3894	UniRef50_B9FFT3	0.0	Normal: 449	941	777	2782	3454	1344	BlastAnno: 2213	UniRef50_B9FFT3	473	73.98	492	89	4	330	1805	20	472	0.0	737
ABCDEF_47345	UniRef50_C5X1U9	0.0	Normal: 124	344	62	39	67	436	BlastAnno: 2363	UniRef50_C5X1U9	627	55.56	486	205	3	542	1996	150	625	0.0	574
ABCDEF_71258	UniRef50_Q9FJI5	0.0	Normal: 83	55	85	312	404	13	BlastAnno: 1043	UniRef50_Q9FJI5	515	83.76	314	48	2	111	1043	1	314	0.0	537
ABCDEF_45319	UniRef50_C5X1U9	0.0	Normal: 630	468	546	883	837	630	BlastAnno: 2119	UniRef50_C5X1U9	627	63.73	477	171	2	402	1829	151	626	0.0	616
ABCDEF_351	UniRef50_C5X1U9	0.0	Normal: 966	364	1313	3355	4586	650	BlastAnno: 2309	UniRef50_C5X1U9	627	51.23	529	235	6	565	2112	107	625	0.0	550
ABCDEF_47342	UniRef50_Q8L743	0.0	Normal: 105	317	56	36	51	381	BlastAnno: 2760	UniRef50_Q8L743	599	85.17	263	39	0	1611	2399	334	596	0.0	475

1.11.1.-(Oxidoreductases;)

ABCDEF_24877	UniRef50_Q9FIJ0	0.0	Normal: 124	402	52	48	107	1528	BlastAnno: 2551	UniRef50_Q9FIJ0	921	71.22	806	222	5	13	2421	123	921	0.0	1185
ABCDEF_10358	UniRef50_Q9FIJ0	0.0	Normal: 142	457	201	403	458	412	BlastAnno: 1774	UniRef50_Q9FIJ0	921	61.14	579	217	6	51	1772	159	734	0.0	717
ABCDEF_38416	UniRef50_Q9FIJ0	0.0	Normal: 75	161	89	114	356	140	BlastAnno: 1314	UniRef50_Q9FIJ0	921	77.61	402	87	2	1313	108	523	921	0.0	659

1.11.1.11(L-ascorbate peroxidase;)

ABCDEF_51654	UniRef50_Q42593	0.0	Normal: 451	646	708	1139	1717	2343	BlastAnno: 1665	UniRef50_Q42593	426	78.83	326	61	2	1467	490	50	367	0.0	527
ABCDEF_51655	UniRef50_Q42593	0.0	Normal: 461	726	655	1038	1365	2645	BlastAnno: 1522	UniRef50_Q42593	426	78.67	375	72	2	1324	200	50	416	0.0	609

1.11.1.12(phospholipid-hydroperoxide glutathione peroxidase;)

ABCDEF_52939	UniRef50_P52032	2e-102	Normal: 515	1528	626	1296	1804	3072	BlastAnno: 1344	UniRef50_P52032	236	86.31	168	23	0	423	926	69	236	2e-102	313

1.11.1.15(peroxiredoxin;)

ABCDEF_18144	UniRef50_Q6ER94	4e-137	Normal: 1450	2849	1716	1993	2290	18473	BlastAnno: 1296	UniRef50_Q6ER94	261	76.98	265	51	3	1076	288	5	261	4e-137	402

1.11.1.9(glutathione peroxidase;)

ABCDEF_37002	UniRef50_O23970	1e-77	Normal: 1065	641	1000	1091	1101	1152	BlastAnno: 5076	UniRef50_O23970	167	71.78	163	46	0	86	574	3	165	1e-77	262

1.17.4.1(ribonucleoside-diphosphate reductase;)

ABCDEF_20810	UniRef50_C5DY80	0.0	Normal: 2798	417	1493	203	646	708	BlastAnno: 2909	UniRef50_C5DY80	875	54.51	776	314	10	2660	405	25	785	0.0	811
ABCDEF_51669	UniRef50_C5DY80	0.0	Normal: 2176	202	1063	25	233	660	BlastAnno: 2851	UniRef50_C5DY80	875	54.12	776	317	9	2594	339	25	785	0.0	815

1.5.4.1(pyrimidodiazepine synthase;)
No Correspond Unigene
1.8.1.12(trypanothione-disulfide reductase;)
No Correspond Unigene
1.8.1.13(bis-gamma-glutamylcystine reductase;)
No Correspond Unigene

1.8.1.7(glutathione-disulfide reductase;)																					
ABCDEF_24113	UniRef50_P42770	0.0	Normal: 355	361	434	399	331	993	BlastAnno: 2125	UniRef50_P42770	565	81.71	514	87	2	200	1741	59	565	0.0	872
ABCDEF_24115	UniRef50_P42770	0.0	Normal: 476	451	573	524	506	1313	BlastAnno: 2299	UniRef50_P42770	565	81.71	514	87	2	234	1775	59	565	0.0	872
ABCDEF_24114	UniRef50_P42770	0.0	Normal: 407	395	501	469	391	1123	BlastAnno: 2166	UniRef50_P42770	565	81.71	514	87	2	200	1741	59	565	0.0	872
1.8.3.3(glutathione oxidase)																					
No Correspond Unigene																					
1.8.4.1(glutathione---homocystine transhydrogenase)																					
No Correspond Unigene																					
1.8.4.2(protein-disulfide reductase (glutathione);)																					
No Correspond Unigene																					
1.8.4.3(glutathione---CoA-glutathione transhydrogenase;)																					
No Correspond Unigene																					
1.8.4.4(glutathione---cystine transhydrogenase;)																					
No Correspond Unigene																					
1.8.4.7(enzyme-thiol transhydrogenase (glutathione-disulfide);)																					
No Correspond Unigene																					
1.8.5.1(glutathione dehydrogenase (ascorbate);)																					
No Correspond Unigene																					
2.3.1.80(cysteine-S-conjugate N-acetyltransferase)																					
No Correspond Unigene																					
2.3.2.2(gamma-glutamyltransferase;)																					
ABCDEF_27037	UniRef50_Q60928	4e-127	Normal: 382	258	428	509	999	336	BlastAnno: 2914	UniRef50_Q60928	568	41.49	552	291	11	330	1961	39	566	4e-127	405
2.3.2.4(gamma-glutamylcyclotransferase;)																					
ABCDEF_49399	UniRef50_Q9BVM4	3e-15	Normal: 101	79	61	78	51	1334	BlastAnno: 557	UniRef50_Q9BVM4	153	47.06	85	43	2	134	385	3	86	3e-15	75.9
2.5.1.16(spermidine synthase;)																					
ABCDEF_7812	UniRef50_Q9ZUB3	0.0	Normal: 1661	1302	2150	4493	6957	4671	BlastAnno: 1757	UniRef50_Q9ZUB3	334	81.44	334	49	3	1326	331	11	333	0.0	552
2.5.1.18(glutathione transferase;)																					
ABCDEF_52207	UniRef50_O80662	1e-123	Normal: 236	352	137	142	402	1670	BlastAnno: 1321	UniRef50_O80662	266	68.66	268	82	1	1087	284	1	266	1e-123	369
2.5.1.22(spermine synthase;)																					
No Correspond Unigene																					
2.8.1.3(thiosulfate---thiol sulfurtransferase;)																					
No Correspond Unigene																					
3.4.11.1(leucyl aminopeptidase;)																					
ABCDEF_44683	UniRef50_Q944P7	0.0	Normal: 2700	2424	2977	4680	5355	5752	BlastAnno: 2222	UniRef50_Q944P7	583	76.63	552	128	1	2047	392	32	582	0.0	808
ABCDEF_83556	UniRef50_Q944P7	0.0	Normal: 33	143	411	29	32	2551	BlastAnno: 2018	UniRef50_Q944P7	583	70.10	505	151	0	1712	198	78	582	0.0	664
ABCDEF_16542	UniRef50_Q944P7	0.0	Normal: 52	179	463	42	45	3265	BlastAnno: 1989	UniRef50_Q944P7	583	69.58	526	160	0	1775	198	57	582	0.0	691
ABCDEF_16541	UniRef50_Q944P7	0.0	Normal: 50	195	437	42	41	3575	BlastAnno: 2944	UniRef50_Q944P7	583	69.34	486	149	0	2730	1273	57	542	0.0	625
ABCDEF_16543	UniRef50_Q944P7	0.0	Normal: 30	162	400	29	29	2877	BlastAnno: 2973	UniRef50_Q944P7	583	70.11	465	139	0	2667	1273	78	542	0.0	599
3.4.11.2(membrane alanyl aminopeptidase;)																					
ABCDEF_6553	UniRef50_B6J1X0	0.0	Normal: 763	755	676	1025	1320	1622	BlastAnno: 3379	UniRef50_B6J1X0	901	46.32	896	468	5	491	3178	13	895	0.0	835
ABCDEF_6551	UniRef50_A7NEZ2	0.0	Normal: 672	699	656	978	1235	1416	BlastAnno: 3119	UniRef50_A7NEZ2	864	60.24	425	168	1	2861	1587	7	430	0.0	542
ABCDEF_6552	UniRef50_B6J1X0	0.0	Normal: 782	719	743	1142	1339	1581	BlastAnno: 3131	UniRef50_B6J1X0	901	46.32	896	468	5	2908	221	13	895	0.0	837
ABCDEF_6554	UniRef50_B6J1X0	0.0	Normal: 759	744	700	1058	1298	1568	BlastAnno: 3418	UniRef50_B6J1X0	901	46.32	896	468	5	2909	222	13	895	0.0	836
3.4.11.23(PepB aminopeptidase;)																					
No Correspond Unigene																					
3.4.13.3(Xaa-His dipeptidase;)																					
No Correspond Unigene																					
3.5.1.-(Hydrolases;)																					
ABCDEF_41579	UniRef50_O81641	0.0	Normal: 384	122	265	320	313	150	BlastAnno: 1456	UniRef50_O81641	428	65.16	399	138	1	1313	120	23	421	0.0	543
ABCDEF_16924	UniRef50_Q8VYX0	0.0	Normal: 135	349	460	1325	685	527	BlastAnno: 2002	UniRef50_Q8VYX0	464	71.72	396	109	2	1612	428	68	461	0.0	549
ABCDEF_24290	UniRef50_O04373	0.0	Normal: 296	238	403	498	698	330	BlastAnno: 1710	UniRef50_O04373	440	65.24	443	151	3	67	1395	1	440	0.0	595
3.5.1.78(glutathionylspermidine amidase;)																					
No Correspond Unigene																					
3.5.2.9(5-oxoprolinase (ATP-hydrolysing);)																					
ABCDEF_38325	UniRef50_Q9FIZ7	0.0	Normal: 132	100	95	142	156	116	BlastAnno: 1000	UniRef50_Q9FIZ7	1266	87.39	333	42	0	2	1000	169	501	0.0	594
ABCDEF_14175	UniRef50_Q9FIZ7	0.0	Normal: 484	438	561	565	862	746	BlastAnno: 2295	UniRef50_Q9FIZ7	1266	83.36	691	113	2	6	2075	575	1264	0.0	1159
4.1.1.17(ornithine decarboxylase;)																					
ABCDEF_35775	UniRef50_Q8S3N2	3e-102	Normal: 121	135	89	32	30	308	BlastAnno: 709	UniRef50_Q8S3N2	435	69.29	241	62	3	23	709	1	241	3e-102	312
6.3.1.8(glutathionylspermidine synthase;)																					
No Correspond Unigene																					
6.3.1.9(trypanothione synthase)																					
No Correspond Unigene																					
6.3.2.2(glutamate---cysteine ligase;)																					
ABCDEF_75950	UniRef50_Q6Z3A3	0.0	Normal: 502	257	473	531	630	722	BlastAnno: 2047	UniRef50_Q6Z3A3	496	82.33	447	79	0	422	1762	50	496	0.0	788
ABCDEF_33962	UniRef50_Q6Z3A3	0.0	Normal: 1054	569	1136	1244	1495	1227	BlastAnno: 2094	UniRef50_Q6Z3A3	496	81.88	447	81	0	433	1773	50	496	0.0	788
ABCDEF_33963	UniRef50_Q6Z3A3	0.0	Normal: 546	368	476	630	431	1064	BlastAnno: 2328	UniRef50_Q6Z3A3	496	81.88	447	81	0	455	1795	50	496	0.0	791
6.3.2.3(glutathione synthase;)																					
ABCDEF_34938	UniRef50_O22494	0.0	Normal: 425	349	637	729	694	696	BlastAnno: 1920	UniRef50_O22494	546	76.42	530	119	2	147	1733	22	546	0.0	835
>00500 Starch and sucrose metabolism																					
1.1.1.22(UDP-glucose 6-dehydrogenase;)																					
ABCDEF_30909	UniRef50_A3AK92	0.0	Normal: 865	1783	1481	3602	5414	3960	BlastAnno: 1822	UniRef50_A3AK92	438	76.89	463	77	2	235	1617	1	435	0.0	749
ABCDEF_67405	UniRef50_A3AK92	0.0	Normal: 83	280	233	170	222	547	BlastAnno: 1342	UniRef50_A3AK92	438	77.64	407	61	2	1217	3	1	379	0.0	653

	ABCDEF_48744	UniRef50_A3AK92	0.0	Normal: 89	350	273	228	296	697	BlastAnno: 1407	UniRef50_A3AK92	438	77.75	409	61	2	1221	1	1	381	0.0	658
	ABCDEF_4415	UniRef50_A3AK92	0.0	Normal: 493	713	643	1516	2818	758	BlastAnno: 1410	UniRef50_A3AK92	438	76.38	381	60	2	1	1137	86	438	0.0	604
	ABCDEF_30910	UniRef50_A3AK92	0.0	Normal: 920	2339	1416	3594	6463	6266	BlastAnno: 1816	UniRef50_A3AK92	438	77.54	463	74	2	1591	209	1	435	0.0	751
	ABCDEF_30908	UniRef50_A3AK92	0.0	Normal: 2277	2525	3512	8495	12648	3403	BlastAnno: 1908	UniRef50_A3AK92	438	77.25	466	76	2	235	1626	1	438	0.0	755
	ABCDEF_73000	UniRef50_Q9AUV6	0.0	Normal: 307	765	498	1248	2232	1778	BlastAnno: 905	UniRef50_Q9AUV6	480	90.37	301	29	0	904	2	95	395	0.0	572
	ABCDEF_84803	UniRef50_A3AK92	0.0	Normal: 78	278	221	166	230	569	BlastAnno: 1468	UniRef50_A3AK92	438	77.70	408	61	2	1220	3	1	380	0.0	656
1.1.99.13(glucoside 3-dehydrogenase;)																						
	No Correspond Unigene																					
2.4.1.1(phosphorylase;)																						
	ABCDEF_12384	UniRef50_B9HP81	0.0	Normal: 724	455	891	6099	5220	792	BlastAnno: 2057	UniRef50_B9HP81	853	84.37	531	83	0	1851	259	322	852	0.0	959
	ABCDEF_38339	UniRef50_B9HP81	0.0	Normal: 1368	863	1524	11439	9254	1364	BlastAnno: 3295	UniRef50_B9HP81	853	82.23	844	150	0	506	3037	9	852	0.0	1464
	ABCDEF_31880	UniRef50_B9HP81	0.0	Normal: 3374	1673	3674	8425	11977	3334	BlastAnno: 3302	UniRef50_B9HP81	853	68.16	402	127	1	1469	264	453	853	0.0	593
	ABCDEF_37584	UniRef50_B9HP81	0.0	Normal: 406	205	417	1204	1569	456	BlastAnno: 2809	UniRef50_B9HP81	853	48.36	823	405	7	100	2520	35	853	0.0	775
	ABCDEF_48936	UniRef50_B9HP81	0.0	Normal: 1242	2292	3026	2560	4486	5907	BlastAnno: 3247	UniRef50_B9HP81	853	65.42	402	138	1	1529	324	453	853	0.0	583
2.4.1.10(levansucrase;)																						
	No Correspond Unigene																					
2.4.1.11(glycogen(starch) synthase;)																						
	ABCDEF_49205	UniRef50_Q5K2R5	1e-109	Normal: 6418	10970	6303	5042	8449	2535	BlastAnno: 2363	UniRef50_Q5K2R5	636	42.73	543	275	13	2001	448	90	621	1e-109	357
2.4.1.12(cellulose synthase (UDP-forming);)																						
	ABCDEF_37969	UniRef50_Q84JA6	0.0	Normal: 65	948	79	203	325	216	BlastAnno: 1621	UniRef50_Q84JA6	1049	72.61	482	115	5	211	1620	28	504	0.0	701
	ABCDEF_18004	UniRef50_B9SY23	0.0	Normal: 1562	1736	2009	3452	4438	2400	BlastAnno: 2298	UniRef50_B9SY23	425	76.35	389	90	2	1758	595	2	389	0.0	635
	ABCDEF_14597	UniRef50_Q84JA6	0.0	Normal: 39	559	68	134	277	180	BlastAnno: 1140	UniRef50_Q84JA6	1049	72.75	389	97	2	1	1140	507	895	0.0	560
	ABCDEF_52379	UniRef50_Q69XK5	0.0	Normal: 4015	4921	4546	10520	13430	4731	BlastAnno: 3479	UniRef50_Q69XK5	860	48.05	847	387	15	789	3245	25	846	0.0	753
	ABCDEF_9504	UniRef50_048947	0.0	Normal: 493	478	522	1134	1240	738	BlastAnno: 1713	UniRef50_048947	1084	64.30	465	153	4	1	1377	625	1082	0.0	588
	ABCDEF_9503	UniRef50_048947	0.0	Normal: 487	544	524	1242	1320	833	BlastAnno: 2087	UniRef50_048947	1084	69.10	589	169	4	3	1751	501	1082	0.0	815
	ABCDEF_57334	UniRef50_048947	0.0	Normal: 196	267	236	615	691	367	BlastAnno: 1367	UniRef50_048947	1084	69.80	457	136	1	1	1365	490	946	0.0	655
	ABCDEF_52020	UniRef50_B9SY23	0.0	Normal: 1635	1534	1975	4284	10353	2289	BlastAnno: 2394	UniRef50_B9SY23	425	75.29	425	103	1	586	1860	2	424	0.0	645
	ABCDEF_471	UniRef50_B9SY23	0.0	Normal: 218	61	287	173	230	102	BlastAnno: 1260	UniRef50_B9SY23	425	74.40	336	86	0	1259	252	89	424	0.0	543
	ABCDEF_51269	UniRef50_B9SY23	0.0	Normal: 147	85	103	21	33	438	BlastAnno: 1864	UniRef50_B9SY23	425	74.00	423	110	0	1463	195	2	424	0.0	659
	ABCDEF_17141	UniRef50_048947	0.0	Normal: 74	1734	69	380	701	333	BlastAnno: 3337	UniRef50_048947	1084	62.85	1093	338	12	3235	128	1	1082	0.0	1318
	ABCDEF_33658	UniRef50_048947	0.0	Normal: 384	308	395	805	1003	602	BlastAnno: 2961	UniRef50_048947	1084	82.72	897	145	4	3	2669	188	1082	0.0	1448
	ABCDEF_9499	UniRef50_Q69XK5	0.0	Normal: 205	207	215	632	538	275	BlastAnno: 1794	UniRef50_Q69XK5	860	54.15	615	241	11	2	1792	119	710	0.0	640
	ABCDEF_470	UniRef50_B9SY23	0.0	Normal: 211	52	262	158	192	88	BlastAnno: 1293	UniRef50_B9SY23	425	72.05	347	86	1	1292	252	89	424	0.0	533
	ABCDEF_18005	UniRef50_B9SY23	0.0	Normal: 1842	2042	2515	4214	5208	2842	BlastAnno: 2305	UniRef50_B9SY23	425	76.42	424	99	1	1765	494	2	424	0.0	703
	ABCDEF_84749	UniRef50_048947	0.0	Normal: 209	218	289	618	1410	378	BlastAnno: 1528	UniRef50_048947	1084	82.44	467	79	3	1515	121	617	1082	0.0	716
	ABCDEF_41182	UniRef50_048947	0.0	Normal: 150	2169	221	814	1407	495	BlastAnno: 3604	UniRef50_048947	1084	62.78	1107	324	15	3536	405	1	1082	0.0	1339
	ABCDEF_48199	UniRef50_B9SY23	0.0	Normal: 1506	467	2429	3094	4233	439	BlastAnno: 1997	UniRef50_B9SY23	425	81.67	371	68	0	474	1586	2	372	0.0	655
	ABCDEF_17140	UniRef50_048947	0.0	Normal: 45	1049	49	216	386	208	BlastAnno: 2293	UniRef50_048947	1084	70.03	744	196	5	2168	6	343	1082	0.0	1038
	ABCDEF_9509	UniRef50_Q69XK5	0.0	Normal: 1296	2171	1498	3410	4683	2232	BlastAnno: 3897	UniRef50_Q69XK5	860	51.90	844	351	15	1073	3508	31	851	0.0	826
	ABCDEF_48200	UniRef50_B9SY23	0.0	Normal: 1768	576	3156	4431	6634	491	BlastAnno: 2274	UniRef50_B9SY23	425	78.96	423	89	0	474	1742	2	424	0.0	716
	ABCDEF_41140	UniRef50_B9SY23	0.0	Normal: 86	1314	93	753	1787	2076	BlastAnno: 2346	UniRef50_B9SY23	425	84.43	424	65	1	614	1885	2	424	0.0	776
	ABCDEF_27767	UniRef50_048947	0.0	Normal: 2576	5141	2841	10249	14583	5031	BlastAnno: 3981	UniRef50_048947	1084	64.05	1082	372	7	3698	453	18	1082	0.0	1365
	ABCDEF_9506	UniRef50_Q69XK5	0.0	Normal: 1302	2128	1494	3356	4644	2206	BlastAnno: 3751	UniRef50_Q69XK5	860	51.90	844	351	15	927	3362	31	851	0.0	826
	ABCDEF_33661	UniRef50_Q69XK5	0.0	Normal: 414	340	507	879	790	783	BlastAnno: 2185	UniRef50_Q69XK5	860	46.50	701	326	14	2184	166	167	846	0.0	611
	ABCDEF_9502	UniRef50_048947	0.0	Normal: 610	486	568	1331	1403	591	BlastAnno: 1961	UniRef50_048947	1084	68.95	599	173	4	3	1781	491	1082	0.0	839
	ABCDEF_9500	UniRef50_Q69XK5	0.0	Normal: 183	188	186	576	498	260	BlastAnno: 1717	UniRef50_Q69XK5	860	53.82	589	231	11	3	1715	145	710	0.0	624
	ABCDEF_41141	UniRef50_B9SY23	0.0	Normal: 67	1107	84	708	1690	1854	BlastAnno: 2461	UniRef50_B9SY23	425	84.43	424	65	1	1733	462	2	424	0.0	775
	ABCDEF_9510	UniRef50_048947	0.0	Normal: 335	388	309	907	931	421	BlastAnno: 1845	UniRef50_048947	1084	58.96	519	194	6	311	1843	1	508	0.0	583
	ABCDEF_21315	UniRef50_Q9SWW6	0.0	Normal: 3412	252	1450	0	79	1228	BlastAnno: 3765	UniRef50_Q9SWW6	1026	49.37	867	332	16	950	3505	215	989	0.0	783
	ABCDEF_56888	UniRef50_Q69XK5	0.0	Normal: 269	426	318	776	861	437	BlastAnno: 1649	UniRef50_Q69XK5	860	54.63	562	215	10	2	1636	119	657	0.0	585
	ABCDEF_33659	UniRef50_Q69XK5	0.0	Normal: 1282	1948	2248	5826	10642	2083	BlastAnno: 4289	UniRef50_Q69XK5	860	48.62	835	382	13	2955	535	31	846	0.0	763
	ABCDEF_79373	UniRef50_048947	0.0	Normal: 306	465	348	811	1100	596	BlastAnno: 1322	UniRef50_048947	1084	69.82	444	128	3	3	1322	498	939	0.0	634
	ABCDEF_56232	UniRef50_048947	0.0	Normal: 221	368	255	630	735	393	BlastAnno: 1221	UniRef50_048947	1084	70.76	407	113	3	3	1211	491	895	0.0	586
	ABCDEF_37667	UniRef50_048947	0.0	Normal: 248	3617	251	1237	2078	970	BlastAnno: 3161	UniRef50_048947	1084	69.59	822	247	2	2698	239	253	1073	0.0	1180
	ABCDEF_47673	UniRef50_Q9SWW6	0.0	Normal: 876	296	828	1628	1721	1172	BlastAnno: 3878	UniRef50_Q9SWW6	1026	49.66	892	356	15	2892	253	199	1009	0.0	835
	ABCDEF_56460	UniRef50_Q69XK5	0.0	Normal: 245	407	291	715	820	423	BlastAnno: 1572	UniRef50_Q69XK5	860	54.29	536	205	10	3	1559	145	657	0.0	568
2.4.1.13(sucrose synthase;)																						
	ABCDEF_4645	UniRef50_P49040	0.0	Normal: 157	361	98	177	1699	268	BlastAnno: 1001	UniRef50_P49040	808	84.68	333	51	0	999	1	386	718	0.0	610
	ABCDEF_10551	UniRef50_Q8DK23	0.0	Normal: 365	12010	332	149	559	1116	BlastAnno: 2926	UniRef50_Q8DK23	808	44.94	801	427	9	2604	238	6	804	0.0	684
	ABCDEF_37284	UniRef50_Q8DK23	0.0	Normal: 184	235	315	2396	1641	316	BlastAnno: 2988	UniRef50_Q8DK23	808	42.11	748	426	4	2705	480	58	804	0.0	615
	ABCDEF_45017	UniRef50_P49040	0.0	Normal: 94	221	61	185	1089	124	BlastAnno: 1095	UniRef50_P49040	808	73.35	364	97	0	1093	2	27	390	0.0	544
	ABCDEF_24805	UniRef50_B9T3H2	0.0	Normal: 37	57	43	355	379	78	BlastAnno: 1314	UniRef50_B9T3H2	799	79.14	374	78	0	1313	192	401	774	0.0	605
	ABCDEF_42297	UniRef50_Q8DK23	0.0	Normal: 1560	2040	1112	1219	10085	82	BlastAnno: 2986	UniRef50_Q8DK23	808	45.25	800	426	8	309	2675	6	804	0.0	700
	ABCDEF_41595	UniRef50_B9T3H2	0.0	Normal: 112	198	203	1565	1124	296	BlastAnno: 1694	UniRef50_B9T3H2	799	77.57	486	109	0	1692	235	313	798	0.0	784
2.4.1.14(sucrose-phosphate synthase;)																						
	ABCDEF_13503	UniRef50_P31927	0.0	Normal: 81	110	106	217	1044	202	BlastAnno: 2225	UniRef50_P31927	1068	50.76	595	265	10	54	1787	478	1061	0.0	569
	ABCDEF_34241	UniRef50_Q6EZE8	0.0	Normal: 336	96	222	1249	1381	253	BlastAnno: 3591	UniRef50_Q6EZE8	1055	51.38	1084	452	18	3411	265	4	1047	0.0	1010
	ABCDEF_13505	UniRef50_Q6EZE8	0.0	Normal: 251	336	285	842	2558	497	BlastAnno: 3804	UniRef50_Q6EZE8	1055	48.20	1083	475	19	3642	547	4	1051	0.0	927
	ABCDEF_13504	UniRef50_Q6EZE8	0.0	Normal: 231	308	267	797	2339	427	BlastAnno: 3717	UniRef50_Q6EZE8	1055	48.20	1083	475	19	3555	460	4	1051	0.0	927
	ABCDEF_73001	UniRef50_Q6EZE8	0.0	Normal: 149	58	148	575	725	134	BlastAnno: 2183	UniRef50_Q6EZE8	1055	46.55	638	299	10	226	2118	445	1047	0.0	558
	ABCDEF_13502	UniRef50_P31927	0.0	Normal: 90	139	126	276	1264	283	BlastAnno: 2312	UniRef50_P31927	1068	50.76	595	265	10	54	1787	478	1061	0.0	569
2.4.1.15(alpha,alpha-trehalose-phosphate synthase (UDP-forming);)																						

ABCDEF_17055	UniRef50_B9SNF1	0.0	Normal: 1212	1042	635	805	1067	3624	BlastAnno: 3184	UniRef50_B9SNF1	753	65.63	483	164	1	1686	244	265	747	0.0	686
ABCDEF_18296	UniRef50_O23617	0.0	Normal: 142	562	21	12	7	2465	BlastAnno: 1837	UniRef50_O23617	862	57.30	534	213	6	1588	2	1	524	0.0	637
ABCDEF_6668	UniRef50_B9SNF1	0.0	Normal: 515	762	316	870	438	4142	BlastAnno: 3448	UniRef50_B9SNF1	753	64.52	482	169	2	2181	742	266	747	0.0	676
ABCDEF_51658	UniRef50_B9SNF1	0.0	Normal: 1604	1924	1135	1862	2132	4634	BlastAnno: 3435	UniRef50_B9SNF1	753	84.60	487	75	0	1652	3112	266	752	0.0	839
ABCDEF_37526	UniRef50_B9SNF1	0.0	Normal: 645	431	558	1320	2054	113	BlastAnno: 2996	UniRef50_B9SNF1	753	74.13	487	126	0	1253	2713	265	751	0.0	775
ABCDEF_37524	UniRef50_B9SNF1	0.0	Normal: 650	448	570	1373	2141	130	BlastAnno: 3118	UniRef50_B9SNF1	753	74.13	487	126	0	1375	2835	265	751	0.0	777
ABCDEF_13946	UniRef50_B9SNF1	0.0	Normal: 1374	1367	1066	1640	1452	1760	BlastAnno: 3724	UniRef50_B9SNF1	753	63.14	491	168	3	1502	2947	266	752	0.0	653
ABCDEF_27215	UniRef50_Q9SHG0	0.0	Normal: 526	185	605	419	1542	575	BlastAnno: 3381	UniRef50_Q9SHG0	730	62.28	509	170	2	2851	1325	7	493	0.0	677
ABCDEF_47929	UniRef50_Q9SHG0	0.0	Normal: 495	346	520	1016	1747	260	BlastAnno: 3365	UniRef50_Q9SHG0	730	46.71	835	316	16	2862	361	10	716	0.0	677

2.4.1.17(glucuronosyltransferase;)

No Correspond Unigene

2.4.1.18(1,4-alpha-glucan branching enzyme;)

ABCDEF_14095	UniRef50_Q08047	0.0	Normal: 1866	843	1776	2739	2596	1094	BlastAnno: 3066	UniRef50_Q08047	799	58.07	694	264	7	2571	526	121	799	0.0	818
ABCDEF_64024	UniRef50_O49185	0.0	Normal: 976	699	820	1667	1850	473	BlastAnno: 2253	UniRef50_O49185	766	62.09	517	190	5	715	2253	24	538	0.0	664
ABCDEF_579	UniRef50_O49185	0.0	Normal: 2177	1458	2021	3615	4292	1141	BlastAnno: 3231	UniRef50_O49185	766	58.05	677	265	8	758	2770	24	687	0.0	785

2.4.1.20(cellobiose phosphorylase)

No Correspond Unigene

2.4.1.21(starch synthase;)

ABCDEF_6154	UniRef50_Q0DDE3	0.0	Normal: 264	295	260	700	706	72	BlastAnno: 1816	UniRef50_Q0DDE3	810	75.40	374	92	0	229	1350	435	808	0.0	612
ABCDEF_13962	UniRef50_Q43846	0.0	Normal: 739	1029	851	1208	959	2087	BlastAnno: 3447	UniRef50_Q43846	1230	79.76	929	188	0	155	2941	300	1228	0.0	1491
ABCDEF_36874	UniRef50_B9SSD8	0.0	Normal: 217	224	164	297	387	395	BlastAnno: 2388	UniRef50_B9SSD8	720	61.94	465	176	1	672	2066	208	671	0.0	636
ABCDEF_48380	UniRef50_Q9FNF2	0.0	Normal: 416	654	404	197	327	1843	BlastAnno: 2492	UniRef50_Q9FNF2	652	69.13	596	163	4	431	2155	56	651	0.0	852
ABCDEF_6155	UniRef50_Q0DDE3	0.0	Normal: 302	331	305	791	852	115	BlastAnno: 2336	UniRef50_Q0DDE3	810	74.06	505	131	0	423	1937	304	808	0.0	814
ABCDEF_6149	UniRef50_Q0DDE3	0.0	Normal: 428	435	443	1159	1124	112	BlastAnno: 3286	UniRef50_Q0DDE3	810	74.06	505	131	0	1305	2819	304	808	0.0	825

2.4.1.25(4-alpha-glucanotransferase;)

ABCDEF_33493	UniRef50_Q8RXD9	0.0	Normal: 29	16	36	181	95	53	BlastAnno: 2954	UniRef50_Q8RXD9	955	68.32	644	176	2	2153	234	333	952	0.0	947
ABCDEF_70560	UniRef50_Q8RXD9	0.0	Normal: 19	11	24	128	78	43	BlastAnno: 2504	UniRef50_Q8RXD9	955	71.13	620	175	1	2081	234	333	952	0.0	961
ABCDEF_33500	UniRef50_Q8RXD9	0.0	Normal: 28	16	34	175	91	53	BlastAnno: 2822	UniRef50_Q8RXD9	955	71.13	620	175	1	1993	146	333	952	0.0	961
ABCDEF_70197	UniRef50_Q8RXD9	0.0	Normal: 21	12	28	138	83	47	BlastAnno: 2576	UniRef50_Q8RXD9	955	68.48	644	175	2	2153	234	333	952	0.0	949
ABCDEF_71621	UniRef50_Q8RXD9	0.0	Normal: 17	11	23	109	67	41	BlastAnno: 2477	UniRef50_Q8RXD9	955	73.52	438	116	0	2157	844	333	770	0.0	700
ABCDEF_33494	UniRef50_Q8RXD9	0.0	Normal: 18	11	27	105	60	41	BlastAnno: 1968	UniRef50_Q8RXD9	955	70.54	404	95	1	1967	756	391	770	0.0	614
ABCDEF_33501	UniRef50_Q8RXD9	0.0	Normal: 20	11	27	132	78	45	BlastAnno: 2548	UniRef50_Q8RXD9	955	68.48	644	175	2	2153	234	333	952	0.0	949
ABCDEF_20293	UniRef50_Q8RXD9	0.0	Normal: 278	145	292	1067	1017	611	BlastAnno: 1751	UniRef50_Q8RXD9	955	76.68	476	109	1	1691	270	471	946	0.0	781
ABCDEF_33497	UniRef50_Q8RXD9	0.0	Normal: 27	16	33	169	86	51	BlastAnno: 2794	UniRef50_Q8RXD9	955	71.13	620	175	1	1993	146	333	952	0.0	961
ABCDEF_33498	UniRef50_Q8RXD9	0.0	Normal: 18	12	24	108	63	43	BlastAnno: 2389	UniRef50_Q8RXD9	955	73.52	438	116	0	2069	756	333	770	0.0	700
ABCDEF_33504	UniRef50_Q8RXD9	0.0	Normal: 26	17	33	181	96	48	BlastAnno: 2023	UniRef50_Q8RXD9	955	72.43	544	146	1	1765	146	409	952	0.0	867
ABCDEF_33505	UniRef50_Q8RXD9	0.0	Normal: 21	12	28	136	80	48	BlastAnno: 2488	UniRef50_Q8RXD9	955	68.48	644	175	2	2065	146	333	952	0.0	949
ABCDEF_20295	UniRef50_Q8RXD9	0.0	Normal: 765	439	668	3138	2472	1374	BlastAnno: 3446	UniRef50_Q8RXD9	955	71.65	924	260	1	3035	270	23	946	0.0	1415
ABCDEF_33499	UniRef50_Q8RXD9	0.0	Normal: 20	13	30	125	77	46	BlastAnno: 2577	UniRef50_Q8RXD9	955	69.48	462	117	1	2229	844	333	770	0.0	685
ABCDEF_73167	UniRef50_Q8RXD9	0.0	Normal: 21	12	28	131	75	47	BlastAnno: 2460	UniRef50_Q8RXD9	955	68.48	644	175	2	2065	146	333	952	0.0	949
ABCDEF_33503	UniRef50_Q8RXD9	0.0	Normal: 23	12	27	148	88	43	BlastAnno: 2017	UniRef50_Q8RXD9	955	69.37	568	146	2	1837	146	409	952	0.0	854
ABCDEF_41443	UniRef50_Q06801	0.0	Normal: 123	55	122	510	514	169	BlastAnno: 1435	UniRef50_Q06801	576	80.55	365	63	3	1145	54	41	398	0.0	615
ABCDEF_33506	UniRef50_Q8RXD9	0.0	Normal: 30	17	37	179	91	55	BlastAnno: 2866	UniRef50_Q8RXD9	955	68.48	644	175	2	2065	146	333	952	0.0	949
ABCDEF_33496	UniRef50_Q8RXD9	0.0	Normal: 32	26	40	220	138	69	BlastAnno: 2910	UniRef50_Q8RXD9	955	71.13	620	175	1	2081	234	333	952	0.0	961
ABCDEF_41444	UniRef50_Q06801	0.0	Normal: 495	179	476	1639	2118	711	BlastAnno: 2367	UniRef50_Q06801	576	81.22	543	94	3	2077	452	41	576	0.0	917
ABCDEF_33507	UniRef50_Q8RXD9	0.0	Normal: 20	12	29	119	72	44	BlastAnno: 2549	UniRef50_Q8RXD9	955	69.70	462	116	1	2229	844	333	770	0.0	687
ABCDEF_73059	UniRef50_Q8RXD9	0.0	Normal: 18	10	23	122	73	42	BlastAnno: 2476	UniRef50_Q8RXD9	955	71.13	620	175	1	2081	234	333	952	0.0	961
ABCDEF_33495	UniRef50_Q8RXD9	0.0	Normal: 29	16	37	186	100	55	BlastAnno: 2982	UniRef50_Q8RXD9	955	68.48	644	175	2	2153	234	333	952	0.0	949
ABCDEF_33502	UniRef50_Q8RXD9	0.0	Normal: 18	11	25	116	74	44	BlastAnno: 2505	UniRef50_Q8RXD9	955	73.29	438	117	0	2157	844	333	770	0.0	698
ABCDEF_33492	UniRef50_Q8RXD9	0.0	Normal: 35	28	44	229	140	74	BlastAnno: 2894	UniRef50_Q8RXD9	955	68.48	644	175	2	2065	146	333	952	0.0	949
ABCDEF_70690	UniRef50_Q8RXD9	0.0	Normal: 19	11	25	126	75	45	BlastAnno: 2416	UniRef50_Q8RXD9	955	71.13	620	175	1	1993	146	333	952	0.0	961
ABCDEF_73288	UniRef50_Q8RXD9	0.0	Normal: 19	11	24	121	70	43	BlastAnno: 2388	UniRef50_Q8RXD9	955	71.13	620	175	1	1993	146	333	952	0.0	961
ABCDEF_33491	UniRef50_Q8RXD9	0.0	Normal: 27	15	31	169	89	50	BlastAnno: 2882	UniRef50_Q8RXD9	955	70.97	620	176	1	2081	234	333	952	0.0	959
ABCDEF_72271	UniRef50_Q8RXD9	0.0	Normal: 18	12	25	113	68	44	BlastAnno: 2417	UniRef50_Q8RXD9	955	73.52	438	116	0	2069	756	333	770	0.0	700

2.4.1.29(cellulose synthase (GDP-forming);)

No Correspond Unigene

2.4.1.34(1,3-beta-glucan synthase;)

ABCDEF_27127	UniRef50_Q9ZT82	0.0	Normal: 2330	764	2189	4900	3133	1418	BlastAnno: 5687	UniRef50_Q9ZT82	1780	73.04	1725	446	9	2	5143	59	1775	0.0	2543
ABCDEF_56964	UniRef50_Q9SFU6	0.0	Normal: 484	207	429	571	534	355	BlastAnno: 2755	UniRef50_Q9SFU6	1931	83.15	807	125	3	88	2502	1134	1931	0.0	1351
ABCDEF_33886	UniRef50_Q9LUD7	0.0	Normal: 448	528	301	409	268	309	BlastAnno: 3386	UniRef50_Q9LUD7	1976	48.64	1178	526	16	3384	1	208	1356	0.0	1100
ABCDEF_44875	UniRef50_Q9LUD7	0.0	Normal: 8236	2430	5400	10693	8778	3765	BlastAnno: 6418	UniRef50_Q9LUD7	1976	54.75	1991	800	23	326	6127	30	1976	0.0	2140
ABCDEF_37024	UniRef50_Q9SFU6	0.0	Normal: 638	291	572	812	712	497	BlastAnno: 3978	UniRef50_Q9SFU6	1931	69.14	674	175	5	1	1962	708	1368	0.0	941
ABCDEF_44258	UniRef50_Q9ZT82	0.0	Normal: 1461	458	1144	1243	1075	746	BlastAnno: 4343	UniRef50_Q9ZT82	1780	69.94	1284	374	7	4342	503	501	1776	0.0	1749
ABCDEF_37023	UniRef50_Q9SFU6	0.0	Normal: 460	203	429	543	522	363	BlastAnno: 2832	UniRef50_Q9SFU6	1931	82.16	583	99	2	834	2579	1353	1931	0.0	933
ABCDEF_43961	UniRef50_Q9SFU6	0.0	Normal: 3114	1731	2830	5500	4751	2529	BlastAnno: 6231	UniRef50_Q9SFU6	1931	61.57	1957	664	18	277	5970	1	1928	0.0	2430
ABCDEF_44075	UniRef50_Q9ZT82	0.0	Normal: 427	346	630	1305	1677	491	BlastAnno: 4725	UniRef50_Q9ZT82	1780	45.72	1612	768	25	4721	96	206	1780	0.0	1335
ABCDEF_37025	UniRef50_Q9SFU6	0.0	Normal: 1527	753	1152	2195	1495	878	BlastAnno: 6193	UniRef50_Q9SFU6	1931	73.00	1967	437	17	6070	344	1	1931	0.0	2802

2.4.1.35(phenol beta-glucosyltransferase;)

No Correspond Unigene

2.4.1.4(amylosucrase;)

No Correspond Unigene

2.4.1.43(polygalacturonate 4-alpha-galacturonosyltransferase;)

No Correspond Unigene

2.4.1.5(dextransucrase;)

No Correspond Unigene

2.4.1.64(alpha,alpha-trehalose phosphorylase;)

No Correspond Unigene

2.4.1.7(sucrose phosphorylase;)

No Correspond Unigene

2.4.1.8(maltose phosphorylase)

No Correspond Unigene

2.4.2.24(1,4-beta-D-xylan synthase;)

No Correspond Unigene

2.7.1.1(hexokinase;)

ABCDEF_27834	UniRef50_P93834	0.0	Normal: 998	404	1410	4185	6485	623	BlastAnno: 2374	UniRef50_P93834	502	72.77	470	128	0	2024	615	25	494	0.0	655
ABCDEF_51305	UniRef50_P93834	0.0	Normal: 443	123	280	151	521	245	BlastAnno: 2179	UniRef50_P93834	502	76.47	289	68	0	1813	947	25	313	0.0	467
ABCDEF_420	UniRef50_Q1WM16	0.0	Normal: 453	101	514	402	696	75	BlastAnno: 1554	UniRef50_Q1WM16	463	61.66	433	159	3	3	1295	20	447	0.0	538
ABCDEF_86444	UniRef50_Q1WM16	0.0	Normal: 293	76	186	81	353	207	BlastAnno: 1684	UniRef50_Q1WM16	463	66.83	404	129	3	1404	196	63	462	0.0	530
ABCDEF_592	UniRef50_P93834	0.0	Normal: 378	177	486	1038	1819	341	BlastAnno: 1859	UniRef50_P93834	502	59.87	456	180	3	1618	260	37	492	0.0	537
ABCDEF_83497	UniRef50_Q1WM16	0.0	Normal: 290	84	185	81	350	338	BlastAnno: 1860	UniRef50_Q1WM16	463	66.83	404	129	3	1404	196	63	462	0.0	529
ABCDEF_51306	UniRef50_Q1WM16	0.0	Normal: 446	118	281	150	539	247	BlastAnno: 2108	UniRef50_Q1WM16	463	66.96	451	144	3	1685	336	16	462	0.0	578
ABCDEF_593	UniRef50_P93834	0.0	Normal: 403	191	504	1097	1940	353	BlastAnno: 1902	UniRef50_P93834	502	59.87	456	180	3	1648	290	37	492	0.0	537

2.7.1.10(phosphoglucokinase;)

No Correspond Unigene

2.7.1.106(glucose-1,6-bisphosphate synthase;)

No Correspond Unigene

2.7.1.2(glucokinase;)

No Correspond Unigene

2.7.1.4(fructokinase;)

ABCDEF_28475	UniRef50_Q9M1B9	0.0	Normal: 2729	2245	2850	2149	3141	1721	BlastAnno: 1388	UniRef50_Q9M1B9	326	85.49	317	45	1	1235	285	1	316	0.0	561

2.7.1.41(glucose-1-phosphate phosphodismutase)

No Correspond Unigene

2.7.1.69(protein-Npi-phosphohistidine---sugar phosphotransferase;)

No Correspond Unigene

2.7.7.27(glucose-1-phosphate adenylyltransferase;)

ABCDEF_17985	UniRef50_P55229	0.0	Normal: 1300	638	1009	329	1017	389	BlastAnno: 1576	UniRef50_P55229	522	68.95	409	126	1	1453	227	115	522	0.0	616
ABCDEF_48650	UniRef50_P55229	0.0	Normal: 350	4442	338	474	264	18127	BlastAnno: 1933	UniRef50_P55229	522	78.03	528	107	5	165	1739	1	522	0.0	857
ABCDEF_67742	UniRef50_P55229	0.0	Normal: 1112	1852	1343	1792	835	556	BlastAnno: 2247	UniRef50_P55229	522	66.92	390	129	0	545	1714	80	469	0.0	578
ABCDEF_30971	UniRef50_P55229	0.0	Normal: 1644	1077	1633	2147	2596	1547	BlastAnno: 1963	UniRef50_P55229	522	54.93	497	210	8	1623	157	32	522	0.0	550
ABCDEF_17986	UniRef50_P55229	0.0	Normal: 2123	1065	1571	652	1712	595	BlastAnno: 2169	UniRef50_P55229	522	69.53	443	134	1	1556	228	81	522	0.0	669
ABCDEF_30972	UniRef50_P55229	0.0	Normal: 581	1501	521	667	1424	5770	BlastAnno: 2058	UniRef50_P55229	522	56.85	438	181	5	1701	403	88	522	0.0	535
ABCDEF_4309	UniRef50_P55229	0.0	Normal: 1203	2072	1480	2030	879	662	BlastAnno: 2273	UniRef50_P55229	522	65.91	443	151	0	1729	401	80	522	0.0	647
ABCDEF_30969	UniRef50_P55229	0.0	Normal: 876	980	759	1045	1332	2854	BlastAnno: 2070	UniRef50_P55229	522	54.12	497	214	8	1730	264	32	522	0.0	545
ABCDEF_23917	UniRef50_P55229	0.0	Normal: 384	209	500	1079	959	244	BlastAnno: 2102	UniRef50_P55229	522	66.59	443	148	0	1579	251	80	522	0.0	644

2.7.7.33(glucose-1-phosphate cytidylyltransferase;)

No Correspond Unigene

2.7.7.34(glucose-1-phosphate guanylyltransferase;)

No Correspond Unigene

2.7.7.9(UTP---glucose-1-phosphate uridylyltransferase;)

ABCDEF_42404	UniRef50_P57751	0.0	Normal: 2120	1934	2860	5861	9792	3375	BlastAnno: 1928	UniRef50_P57751	470	84.13	460	71	2	207	1583	6	464	0.0	756
ABCDEF_68465	UniRef50_P57751	0.0	Normal: 1684	1594	2449	5480	7890	2628	BlastAnno: 1863	UniRef50_P57751	470	84.13	460	71	2	1678	302	6	464	0.0	756

3.1.1.11(pectinesterase;)

ABCDEF_48873	UniRef50_B9N4L5	0.0	Normal: 32	42	68	373	320	42	BlastAnno: 945	UniRef50_B9N4L5	502	77.96	304	67	0	33	944	195	498	0.0	538
ABCDEF_45683	UniRef50_Q1JPL7	0.0	Normal: 6813	6115	6504	5953	20996	376	BlastAnno: 1883	UniRef50_Q1JPL7	557	55.89	560	231	6	59	1702	2	557	0.0	606
ABCDEF_39037	UniRef50_Q43111	0.0	Normal: 0	832	0	1248	88	3353	BlastAnno: 2135	UniRef50_Q43111	581	56.23	594	237	12	1987	236	1	581	0.0	595
ABCDEF_48615	UniRef50_C5XIT6	0.0	Normal: 5939	5402	4656	2532	11870	5247	BlastAnno: 2220	UniRef50_C5XIT6	576	56.60	530	205	7	382	1902	49	576	0.0	590
ABCDEF_4025	UniRef50_Q8L7Q7	0.0	Normal: 1134	2265	1101	1078	864	1623	BlastAnno: 2149	UniRef50_Q8L7Q7	602	56.41	601	236	7	1972	239	5	602	0.0	655
ABCDEF_21060	UniRef50_Q9M3B0	0.0	Normal: 302	2377	237	1706	3680	2294	BlastAnno: 2050	UniRef50_Q9M3B0	598	68.76	541	156	6	4	1596	60	597	0.0	714
ABCDEF_11423	UniRef50_O22149	0.0	Normal: 5	87	0	0	0	308	BlastAnno: 1565	UniRef50_O22149	511	68.57	509	143	7	46	1563	9	503	0.0	679
ABCDEF_41398	UniRef50_Q9SMY7	0.0	Normal: 443	103	478	614	2563	224	BlastAnno: 1931	UniRef50_Q9SMY7	525	61.71	525	173	8	228	1739	8	525	0.0	601
ABCDEF_37616	UniRef50_Q8L7Q7	0.0	Normal: 137	194	120	365	277	132	BlastAnno: 1799	UniRef50_Q8L7Q7	602	56.19	509	210	6	220	1713	91	597	0.0	569
ABCDEF_17702	UniRef50_Q56W36	0.0	Normal: 1505	3569	1047	677	2800	2803	BlastAnno: 2042	UniRef50_Q56W36	381	73.56	382	80	2	1267	182	1	381	0.0	572
ABCDEF_71054	UniRef50_Q43111	0.0	Normal: 116	287	99	507	262	1043	BlastAnno: 1410	UniRef50_Q43111	581	61.18	456	162	7	27	1373	134	581	0.0	566
ABCDEF_45132	UniRef50_O48711	0.0	Normal: 72	1016	82	124	1126	92	BlastAnno: 1987	UniRef50_O48711	547	65.12	516	171	5	302	1831	35	547	0.0	694
ABCDEF_10255	UniRef50_Q9M3B0	0.0	Normal: 125	2405	248	1499	1355	2608	BlastAnno: 2207	UniRef50_Q9M3B0	598	65.28	530	169	6	1861	284	79	597	0.0	652
ABCDEF_14881	UniRef50_O22149	0.0	Normal: 45	67	46	969	2833	312	BlastAnno: 1739	UniRef50_O22149	511	63.10	496	165	10	1628	156	29	511	0.0	620
ABCDEF_3485	UniRef50_Q8LPF3	0.0	Normal: 791	22	562	292	19	213	BlastAnno: 1188	UniRef50_Q8LPF3	362	79.18	317	64	1	86	1030	46	362	0.0	532
ABCDEF_40958	UniRef50_Q9SMY7	0.0	Normal: 139	1736	370	2525	4188	716	BlastAnno: 2342	UniRef50_Q9SMY7	525	64.09	504	173	3	1690	179	28	523	0.0	606
ABCDEF_38400	UniRef50_Q9LVQ0	0.0	Normal: 559	448	446	416	590	768	BlastAnno: 1239	UniRef50_Q9LVQ0	317	77.56	312	70	0	1154	219	6	317	0.0	515

3.1.2.21(dodecanoyl-[acyl-carrier-protein] hydrolase;)

No Correspond Unigene

3.1.3.12(trehalose-phosphatase;)

ABCDEF_27215	UniRef50_A3CXN9	9e-150	Normal: 526	185	605	419	1542	575	BlastAnno: 3381	UniRef50_A3CXN9	752	37.77	728	410	13	2839	680	2	694	9e-150	475

3.1.3.24(sucrose-phosphate phosphatase;)

ABCDEF_77426	UniRef50_Q5IH14	0.0	Normal: 364	332	482	1171	1909	640	BlastAnno: 1881	UniRef50_Q5IH14	425	81.97	427	74	2	1532	255	1	425	0.0	723
ABCDEF_1276	UniRef50_Q5IH14	0.0	Normal: 492	462	654	1674	2504	868	BlastAnno: 1836	UniRef50_Q5IH14	425	81.97	427	74	2	1532	255	1	425	0.0	726
ABCDEF_66931	UniRef50_Q5IH14	0.0	Normal: 356	315	480	1138	1819	633	BlastAnno: 1588	UniRef50_Q5IH14	425	81.97	427	74	2	108	1385	1	425	0.0	728
3.1.3.9(glucose-6-phosphatase;)																					
No Correspond Unigene																					
3.2.1.1(alpha-amylase;)																					
ABCDEF_18718	UniRef50_P27933	1e-170	Normal: 0	161	0	0	10	262	BlastAnno: 1039	UniRef50_P27933	436	69.18	318	98	0	84	1037	25	342	1e-170	491
3.2.1.10(oligo-1,6-glucosidase;)																					
No Correspond Unigene																					
3.2.1.122(maltose-6'-phosphate glucosidase;)																					
No Correspond Unigene																					
3.2.1.15(polygalacturonase;)																					
ABCDEF_49100	UniRef50_A7PZL3	0.0	Normal: 1045	1066	1246	2395	2653	2235	BlastAnno: 1908	UniRef50_A7PZL3	491	76.83	436	101	0	362	1669	49	484	0.0	703
ABCDEF_28046	UniRef50_049432	0.0	Normal: 199	82	178	510	235	75	BlastAnno: 1684	UniRef50_049432	481	70.34	408	119	2	376	1596	73	479	0.0	570
ABCDEF_18085	UniRef50_A7PZL3	0.0	Normal: 1501	3298	977	345	485	4572	BlastAnno: 1766	UniRef50_A7PZL3	491	78.13	439	96	0	268	1584	51	489	0.0	745
3.2.1.2(beta-amylase;)																					
ABCDEF_34687	UniRef50_B9RSZ6	0.0	Normal: 612	1665	313	233	568	4532	BlastAnno: 1959	UniRef50_B9RSZ6	545	58.65	549	196	10	1749	148	1	533	0.0	597
ABCDEF_17390	UniRef50_Q9LIR6	0.0	Normal: 704	1193	65	148	112	6007	BlastAnno: 2019	UniRef50_Q9LIR6	575	66.49	576	152	8	171	1796	1	569	0.0	761
ABCDEF_49255	UniRef50_Q9LIR6	0.0	Normal: 59	1186	32	152	82	562	BlastAnno: 2060	UniRef50_Q9LIR6	575	62.61	444	158	3	1803	496	106	549	0.0	578
ABCDEF_17749	UniRef50_Q9LIR6	0.0	Normal: 982	1829	552	954	864	5744	BlastAnno: 2086	UniRef50_Q9LIR6	575	72.70	575	138	6	1914	220	1	566	0.0	861
ABCDEF_16927	UniRef50_065258	0.0	Normal: 530	553	556	797	791	1578	BlastAnno: 2924	UniRef50_065258	527	68.46	428	135	0	1223	2506	88	515	0.0	624
3.2.1.20(alpha-glucosidase;)																					
ABCDEF_11273	UniRef50_004931	0.0	Normal: 3776	2509	4222	13744	13893	3618	BlastAnno: 3405	UniRef50_004931	913	45.27	846	404	11	3289	824	41	851	0.0	750
ABCDEF_7080	UniRef50_004931	0.0	Normal: 1183	1353	1313	1469	361	1294	BlastAnno: 3133	UniRef50_004931	913	62.89	838	294	8	2831	354	36	868	0.0	1041
ABCDEF_11272	UniRef50_004931	0.0	Normal: 3753	2519	4151	13616	13686	3608	BlastAnno: 3398	UniRef50_004931	913	45.27	846	404	11	3260	795	41	851	0.0	750
ABCDEF_7166	UniRef50_004931	0.0	Normal: 153	922	185	225	929	658	BlastAnno: 2816	UniRef50_004931	913	60.61	820	316	3	2710	272	32	851	0.0	1028
3.2.1.21(beta-glucosidase;)																					
ABCDEF_24901	UniRef50_Q9LV34	0.0	Normal: 696	459	604	917	1518	3129	BlastAnno: 1698	UniRef50_Q9LV34	495	69.82	487	133	4	1576	131	17	494	0.0	696
ABCDEF_3707	UniRef50_Q8L7J2	0.0	Normal: 55	614	80	478	1267	616	BlastAnno: 1950	UniRef50_Q8L7J2	521	68.39	484	143	2	365	1786	36	519	0.0	738
ABCDEF_50691	UniRef50_080690	0.0	Normal: 29	230	24	31	176	222	BlastAnno: 1737	UniRef50_080690	527	56.05	512	197	4	1723	194	39	524	0.0	593
ABCDEF_6978	UniRef50_Q9LV34	0.0	Normal: 132	59	114	74	74	76	BlastAnno: 1368	UniRef50_Q9LV34	495	65.54	444	136	5	1311	1	27	460	0.0	616
ABCDEF_7820	UniRef50_B3H5Q1	0.0	Normal: 1199	176	254	446	677	1138	BlastAnno: 1699	UniRef50_B3H5Q1	521	55.14	486	190	4	97	1470	25	510	0.0	549
ABCDEF_52515	UniRef50_064882	0.0	Normal: 14	184	426	593	18106	970	BlastAnno: 1946	UniRef50_064882	517	55.39	482	214	1	128	1570	32	513	0.0	574
ABCDEF_50689	UniRef50_080690	0.0	Normal: 42	287	26	32	235	244	BlastAnno: 1949	UniRef50_080690	527	57.58	521	205	4	1711	194	5	524	0.0	608
ABCDEF_24900	UniRef50_Q9LV34	0.0	Normal: 661	448	581	908	1414	2839	BlastAnno: 1678	UniRef50_Q9LV34	495	69.82	487	133	4	1557	112	17	494	0.0	696
ABCDEF_9495	UniRef50_080690	0.0	Normal: 261	104	1144	3869	396	26	BlastAnno: 1691	UniRef50_080690	527	52.12	495	196	7	220	1686	59	518	0.0	533
ABCDEF_9464	UniRef50_080690	0.0	Normal: 733	82	1949	4567	726	6	BlastAnno: 2104	UniRef50_080690	527	53.91	473	206	6	269	1669	59	525	0.0	542
3.2.1.26(beta-fructofuranosidase;)																					
ABCDEF_7582	UniRef50_Q5JJV0	0.0	Normal: 32	301	39	111	33	545	BlastAnno: 1949	UniRef50_Q5JJV0	590	49.44	536	263	6	1799	201	56	586	0.0	543
ABCDEF_680	UniRef50_P49175	0.0	Normal: 874	868	635	497	1076	2655	BlastAnno: 2168	UniRef50_P49175	670	64.58	576	186	9	1848	172	90	664	0.0	739
ABCDEF_52101	UniRef50_P49175	0.0	Normal: 289	386	561	2596	4447	228	BlastAnno: 1888	UniRef50_P49175	670	62.33	592	207	10	17	1756	77	664	0.0	729
ABCDEF_35900	UniRef50_P49175	0.0	Normal: 21	54	37	6735	4049	2	BlastAnno: 1966	UniRef50_P49175	670	62.85	568	195	7	1674	19	91	658	0.0	719
ABCDEF_10935	UniRef50_P49175	0.0	Normal: 81	178	91	1677	595	1048	BlastAnno: 2083	UniRef50_P49175	670	58.88	552	212	6	276	1889	114	664	0.0	663
3.2.1.28(alpha,alpha-trehalase;)																					
ABCDEF_8199	UniRef50_A6MIZ4	3e-180	Normal: 843	399	872	1329	2036	512	BlastAnno: 2543	UniRef50_A6MIZ4	574	48.82	549	270	4	396	2021	19	563	3e-180	540
3.2.1.3(glucan 1,4-alpha-glucosidase;)																					
No Correspond Unigene																					
3.2.1.31(beta-glucuronidase;)																					
No Correspond Unigene																					
3.2.1.33(amylo-alpha-1,6-glucosidase;)																					
No Correspond Unigene																					
3.2.1.37(xylan 1,4-beta-xylosidase;)																					
ABCDEF_34152	UniRef50_Q2MCJ4	0.0	Normal: 568	815	532	816	648	1135	BlastAnno: 2581	UniRef50_Q2MCJ4	704	54.24	754	258	13	2450	213	30	704	0.0	789
ABCDEF_45209	UniRef50_Q9FLG1	0.0	Normal: 1338	811	1191	1817	1167	1821	BlastAnno: 2643	UniRef50_Q9FLG1	784	67.01	773	244	5	2469	172	16	784	0.0	1102
ABCDEF_34155	UniRef50_Q2MCJ4	0.0	Normal: 543	763	515	772	621	1127	BlastAnno: 2640	UniRef50_Q2MCJ4	704	52.88	643	221	10	2112	196	140	704	0.0	660
ABCDEF_34153	UniRef50_Q2MCJ4	0.0	Normal: 522	668	469	700	564	936	BlastAnno: 2528	UniRef50_Q2MCJ4	704	54.41	748	254	13	2397	178	30	698	0.0	779
ABCDEF_34154	UniRef50_Q2MCJ4	0.0	Normal: 495	622	454	650	534	930	BlastAnno: 2619	UniRef50_Q2MCJ4	704	53.06	637	217	10	2076	178	140	698	0.0	650
ABCDEF_980	UniRef50_Q9FLG1	0.0	Normal: 1398	7037	1070	4974	7681	6612	BlastAnno: 2765	UniRef50_Q9FLG1	784	63.28	749	268	2	242	2470	37	784	0.0	1008
ABCDEF_31427	UniRef50_Q9FLG1	0.0	Normal: 56	316	89	4893	4124	2869	BlastAnno: 2823	UniRef50_Q9FLG1	784	56.12	768	309	12	179	2425	25	783	0.0	849
3.2.1.39(glucan endo-1,3-beta-D-glucosidase;)																					
ABCDEF_9641	UniRef50_Q9M088	0.0	Normal: 39	29	31	60	114	42	BlastAnno: 1390	UniRef50_Q9M088	484	69.65	369	111	1	1387	281	65	432	0.0	535
ABCDEF_34545	UniRef50_Q6NKW9	0.0	Normal: 110	270	83	177	136	578	BlastAnno: 1758	UniRef50_Q6NKW9	481	75.12	434	107	1	1557	256	22	454	0.0	690
ABCDEF_28964	UniRef50_Q93Z08	0.0	Normal: 308	94	294	359	287	147	BlastAnno: 1575	UniRef50_Q93Z08	477	70.59	374	110	0	1381	260	104	477	0.0	560
ABCDEF_58766	UniRef50_B9SH66	0.0	Normal: 471	132	333	121	494	145	BlastAnno: 1610	UniRef50_B9SH66	495	76.42	441	96	1	76	1374	19	459	0.0	709
ABCDEF_66	UniRef50_Q9M088	0.0	Normal: 387	108	344	423	357	194	BlastAnno: 2002	UniRef50_Q9M088	484	64.69	456	154	4	1755	400	1	453	0.0	621
ABCDEF_42013	UniRef50_Q9FGH4	0.0	Normal: 311	87	314	98	248	164	BlastAnno: 1405	UniRef50_Q9FGH4	476	65.76	403	132	3	1403	195	63	459	0.0	528
ABCDEF_34880	UniRef50_Q9M069	0.0	Normal: 1681	3879	1397	1068	2882	2395	BlastAnno: 1882	UniRef50_Q9M069	504	66.51	427	142	1	1600	323	22	448	0.0	569
ABCDEF_13893	UniRef50_Q9ZU91	0.0	Normal: 1146	128	1050	908	523	176	BlastAnno: 2053	UniRef50_Q9ZU91	501	72.62	442	120	1	339	1661	19	460	0.0	684
ABCDEF_17976	UniRef50_B9SH66	0.0	Normal: 940	364	1031	1063	1162	2107	BlastAnno: 1857	UniRef50_B9SH66	495	77.73	440	98	0	165	1484	19	458	0.0	728
ABCDEF_41871	UniRef50_Q9M088	0.0	Normal: 1042	24	1101	860	1306	160	BlastAnno: 2071	UniRef50_Q9M088	484	63.62	437	155	3	353	1657	19	453	0.0	611
ABCDEF_41264	UniRef50_Q94CD8	0.0	Normal: 206	88	198	338	449	318	BlastAnno: 1930	UniRef50_Q94CD8	505	66.52	460	147	4	317	1690	16	470	0.0	564
ABCDEF_41265	UniRef50_Q94CD8	0.0	Normal: 284	120	257	512	537	398	BlastAnno: 2016	UniRef50_Q94CD8	505	66.52	460	147	4	1614	241	16	470	0.0	564

ABCDEF_21451	UniRef50_Q9ZU91	0.0	Normal: 369	28	326	288	383	65	BlastAnno: 1981	UniRef50_Q9ZU91	501	71.96	460	128	1	1799	423	19	478	0.0	709
ABCDEF_14564	UniRef50_Q9M069	0.0	Normal: 569	678	815	2218	3934	588	BlastAnno: 1885	UniRef50_Q9M069	504	66.29	439	136	2	274	1590	22	448	0.0	576
ABCDEF_13857	UniRef50_Q94CD8	0.0	Normal: 420	270	472	440	387	475	BlastAnno: 1871	UniRef50_Q94CD8	505	61.79	458	170	3	1670	303	23	477	0.0	537
ABCDEF_74016	UniRef50_Q9M088	0.0	Normal: 362	100	321	387	352	186	BlastAnno: 1992	UniRef50_Q9M088	484	64.69	456	154	4	239	1594	1	453	0.0	621
ABCDEF_66081	UniRef50_Q9M088	0.0	Normal: 359	102	322	398	338	186	BlastAnno: 1908	UniRef50_Q9M088	484	64.69	456	154	4	155	1510	1	453	0.0	621
ABCDEF_67	UniRef50_Q9M088	0.0	Normal: 372	108	333	415	364	200	BlastAnno: 1959	UniRef50_Q9M088	484	64.69	456	154	4	205	1560	1	453	0.0	621
ABCDEF_29183	UniRef50_Q9M088	0.0	Normal: 28	39	25	279	251	26	BlastAnno: 1391	UniRef50_Q9M088	484	63.47	427	153	2	1285	5	19	442	0.0	565
ABCDEF_14586	UniRef50_B9SH66	0.0	Normal: 748	221	574	208	681	239	BlastAnno: 1681	UniRef50_B9SH66	495	76.42	441	96	1	147	1445	19	459	0.0	709
ABCDEF_9637	UniRef50_Q9M088	0.0	Normal: 134	81	121	171	417	111	BlastAnno: 2108	UniRef50_Q9M088	484	66.38	461	145	3	660	2018	25	483	0.0	635
ABCDEF_31593	UniRef50_Q8VYE5	0.0	Normal: 1719	227	716	235	306	232	BlastAnno: 1880	UniRef50_Q8VYE5	534	64.60	452	135	2	1749	469	25	476	0.0	619
ABCDEF_44690	UniRef50_Q6NKW9	0.0	Normal: 405	102	476	1299	1942	246	BlastAnno: 2108	UniRef50_Q6NKW9	481	75.23	444	109	1	1632	301	11	453	0.0	701
ABCDEF_9643	UniRef50_Q9M088	0.0	Normal: 44	25	41	66	149	60	BlastAnno: 1325	UniRef50_Q9M088	484	65.77	371	117	3	147	1235	115	483	0.0	525
ABCDEF_68	UniRef50_Q9M088	0.0	Normal: 360	103	327	395	355	192	BlastAnno: 1903	UniRef50_Q9M088	484	64.69	456	154	4	150	1505	1	453	0.0	621
ABCDEF_77663	UniRef50_Q94CD8	0.0	Normal: 211	93	207	351	445	314	BlastAnno: 1894	UniRef50_Q94CD8	505	66.52	460	147	4	312	1685	16	470	0.0	564
ABCDEF_14300	UniRef50_Q9ZU91	0.0	Normal: 1577	97	779	540	749	298	BlastAnno: 1574	UniRef50_Q9ZU91	501	70.61	456	132	2	1363	2	19	474	0.0	676

3.2.1.4(cellulase;)

ABCDEF_3393	UniRef50_Q8LCP6	0.0	Normal: 76	90	87	120	368	68	BlastAnno: 1907	UniRef50_Q8LCP6	525	64.79	497	168	3	238	1713	11	505	0.0	627
ABCDEF_28479	UniRef50_Q8LCP6	0.0	Normal: 1557	2673	1282	1352	3092	3654	BlastAnno: 2100	UniRef50_Q8LCP6	525	67.52	508	162	3	1838	315	1	505	0.0	646
ABCDEF_4533	UniRef50_Q69NF5	0.0	Normal: 851	877	422	466	614	3806	BlastAnno: 1914	UniRef50_Q69NF5	515	68.24	466	147	1	1633	239	40	505	0.0	603
ABCDEF_27475	UniRef50_Q5NAT8	0.0	Normal: 983	1572	1206	11297	8455	3342	BlastAnno: 2759	UniRef50_Q5NAT8	640	58.16	619	226	9	2409	598	40	640	0.0	629
ABCDEF_7217	UniRef50_Q7XUK4	0.0	Normal: 2071	3000	2267	5113	7736	4502	BlastAnno: 2529	UniRef50_Q7XUK4	623	59.64	612	232	8	304	2109	1	607	0.0	646
ABCDEF_3822	UniRef50_Q5NAT8	0.0	Normal: 5993	5064	4386	11829	8287	8271	BlastAnno: 2610	UniRef50_Q5NAT8	640	60.58	619	201	9	2311	530	40	640	0.0	721
ABCDEF_33981	UniRef50_Q7XUK4	0.0	Normal: 250	471	290	1088	1153	224	BlastAnno: 2065	UniRef50_Q7XUK4	623	58.90	584	229	7	2060	330	28	607	0.0	630
ABCDEF_17855	UniRef50_Q9SRX3	0.0	Normal: 2315	264	1059	5847	8392	1518	BlastAnno: 1844	UniRef50_Q9SRX3	501	73.78	473	118	2	1688	270	35	501	0.0	728
ABCDEF_31110	UniRef50_O48766	0.0	Normal: 244	549	257	631	2060	404	BlastAnno: 1838	UniRef50_O48766	525	77.56	468	103	1	267	1670	38	503	0.0	731
ABCDEF_33986	UniRef50_Q38890	0.0	Normal: 191	410	212	893	819	179	BlastAnno: 1877	UniRef50_Q38890	621	79.26	487	98	3	1602	151	1	487	0.0	718
ABCDEF_3394	UniRef50_Q8LCP6	0.0	Normal: 91	128	92	130	478	99	BlastAnno: 2019	UniRef50_Q8LCP6	525	64.79	497	168	3	1782	307	11	505	0.0	627
ABCDEF_33983	UniRef50_Q7XUK4	0.0	Normal: 216	440	279	947	1068	212	BlastAnno: 2068	UniRef50_Q7XUK4	623	58.57	601	237	8	3	1784	12	607	0.0	644

3.2.1.48(sucrose alpha-glucosidase;)

No Correspond Unigene

3.2.1.54(cyclomaltodextrinase;)

No Correspond Unigene

3.2.1.58(glucan 1,3-beta-glucosidase;)

ABCDEF_44843	UniRef50_Q12626	9e-36	Normal: 83	260	74	237	306	909	BlastAnno: 1469	UniRef50_Q12626	435	31.28	374	179	14	1008	61	66	419	9e-36	144

3.2.1.65(levanase;)

No Correspond Unigene

3.2.1.67(galacturan 1,4-alpha-galacturonidase;)

ABCDEF_18085	UniRef50_B9RIT3	8e-165	Normal: 1501	3298	977	345	485	4572	BlastAnno: 1766	UniRef50_B9RIT3	452	52.86	454	175	4	226	1575	28	446	8e-165	486

3.2.1.74(glucan 1,4-beta-glucosidase;)

ABCDEF_51426	UniRef50_A4FNP6	2e-169	Normal: 239	636	405	2536	3194	1405	BlastAnno: 2299	UniRef50_A4FNP6	615	50.00	604	272	11	2123	330	33	612	2e-169	511

3.2.1.91(cellulose 1,4-beta-cellobiosidase;)

No Correspond Unigene

3.2.1.93(alpha,alpha-phosphotrehalase;)

No Correspond Unigene

3.6.1.21(ADP-sugar diphosphatase;)

ABCDEF_6188	UniRef50_Q9SZ63	2e-140	Normal: 60	106	66	96	87	338	BlastAnno: 1230	UniRef50_Q9SZ63	309	72.14	262	73	0	1056	271	43	304	2e-140	412

3.6.1.9(nucleotide diphosphatase;)

ABCDEF_27673	UniRef50_Q9SU83	0.0	Normal: 373	418	422	639	977	783	BlastAnno: 2094	UniRef50_Q9SU83	496	70.27	481	123	2	1775	393	15	495	0.0	679
ABCDEF_83044	UniRef50_Q9SU83	0.0	Normal: 191	238	231	367	469	401	BlastAnno: 1570	UniRef50_Q9SU83	496	70.42	480	122	2	162	1541	15	494	0.0	678

4.1.1.35(UDP-glucuronate decarboxylase;)

ABCDEF_11113	UniRef50_B9SAR7	0.0	Normal: 925	1155	753	449	2067	835	BlastAnno: 1952	UniRef50_B9SAR7	419	74.43	442	79	5	1658	342	1	411	0.0	645
ABCDEF_35392	UniRef50_B9SAR7	0.0	Normal: 298	348	433	969	1317	656	BlastAnno: 1691	UniRef50_B9SAR7	419	65.74	432	117	2	247	1524	11	417	0.0	552

5.1.3.6(UDP-glucuronate 4-epimerase;)

ABCDEF_40845	UniRef50_Q9LIS3	0.0	Normal: 657	4113	785	1682	3724	4096	BlastAnno: 1905	UniRef50_Q9LIS3	460	77.48	453	95	3	1780	437	8	458	0.0	670
ABCDEF_7044	UniRef50_Q9LIS3	0.0	Normal: 347	255	406	1043	998	1296	BlastAnno: 1923	UniRef50_Q9LIS3	460	72.75	378	93	1	1317	214	67	444	0.0	549
ABCDEF_34307	UniRef50_Q9LIS3	0.0	Normal: 375	325	466	1035	1289	406	BlastAnno: 2245	UniRef50_Q9LIS3	460	75.35	353	87	0	704	1762	92	444	0.0	568
ABCDEF_7309	UniRef50_Q9LIS3	0.0	Normal: 318	240	786	2596	2150	368	BlastAnno: 2125	UniRef50_Q9LIS3	460	76.92	351	81	0	1451	399	93	443	0.0	535
ABCDEF_40847	UniRef50_Q9LIS3	0.0	Normal: 355	521	497	2860	2581	1569	BlastAnno: 1492	UniRef50_Q9LIS3	460	76.44	450	98	1	1382	57	3	452	0.0	671
ABCDEF_40846	UniRef50_Q9LIS3	0.0	Normal: 362	1911	403	1392	2019	2084	BlastAnno: 1462	UniRef50_Q9LIS3	460	78.43	445	91	2	1366	47	8	452	0.0	670

5.3.1.9(glucose-6-phosphate isomerase;)

ABCDEF_21891	UniRef50_B5EBB8	0.0	Normal: 2382	2326	3109	5032	5330	3316	BlastAnno: 2692	UniRef50_B5EBB8	530	60.59	510	196	2	2176	647	4	508	0.0	624
ABCDEF_4218	UniRef50_B7GDV0	0.0	Normal: 982	971	1324	2230	3149	3125	BlastAnno: 2219	UniRef50_B7GDV0	786	56.56	564	238	4	2045	375	188	751	0.0	639

5.4.2.2(phosphoglucomutase;)

ABCDEF_935	UniRef50_P47244	0.0	Normal: 5430	4330	5743	12110	19002	6223	BlastAnno: 2144	UniRef50_P47244	572	53.66	587	246	11	2064	328	4	572	0.0	585
ABCDEF_24641	UniRef50_P47244	0.0	Normal: 416	659	414	959	874	2109	BlastAnno: 2341	UniRef50_P47244	572	54.17	576	222	11	386	2035	13	572	0.0	588
ABCDEF_24640	UniRef50_P47244	0.0	Normal: 389	596	384	834	777	1859	BlastAnno: 2422	UniRef50_P47244	572	51.74	603	222	12	386	2116	13	572	0.0	574

5.4.2.6(beta-phosphoglucomutase)

ABCDEF_42862	UniRef50_Q13NY2	3e-42	Normal: 97	108	194	384	621	293	BlastAnno: 1220	UniRef50_Q13NY2	230	40.55	217	124	2	876	226	7	218	3e-42	155
ABCDEF_1252	UniRef50_Q13NY2	3e-42	Normal: 116	116	197	376	625	303	BlastAnno: 1211	UniRef50_Q13NY2	230	40.55	217	124	2	867	217	7	218	3e-42	155

5.4.99.16(maltose alpha-D-glucosyltransferase;)

No Correspond Unigene

>00510 N-Glycan biosynthesis

2.4.1.-(Transferases;)

ABCDEF_18004	UniRef50_Q6YWK8	0.0	Normal: 1562	1736	2009	3452	4438	2400	BlastAnno: 2298	UniRef50_Q6YWK8	570	65.27	429	136	1	1941	694	57	485	0.0	608
ABCDEF_52379	UniRef50_Q9LHZ7	0.0	Normal: 4015	4921	4546	10520	13430	4731	BlastAnno: 3479	UniRef50_Q9LHZ7	1170	48.04	893	342	16	825	3242	299	1156	0.0	787
ABCDEF_48593	UniRef50_Q9SK82	0.0	Normal: 12	420	19	343	763	253	BlastAnno: 1834	UniRef50_Q9SK82	489	60.08	481	186	4	1714	278	10	486	0.0	633
ABCDEF_13375	UniRef50_Q8VZK9	0.0	Normal: 108	123	208	526	34	294	BlastAnno: 1738	UniRef50_Q8VZK9	729	53.62	552	234	8	94	1737	18	551	0.0	582
ABCDEF_40982	UniRef50_Q96301	0.0	Normal: 1037	706	819	1249	1084	920	BlastAnno: 3418	UniRef50_Q96301	914	79.60	902	173	3	243	2948	1	891	0.0	1506
ABCDEF_47674	UniRef50_Q9LHZ7	0.0	Normal: 274	92	202	599	505	280	BlastAnno: 2526	UniRef50_Q9LHZ7	1170	75.93	777	151	8	2260	2	15	779	0.0	1177
ABCDEF_34097	UniRef50_Q9LE59	0.0	Normal: 626	613	923	1797	1829	692	BlastAnno: 2521	UniRef50_Q9LE59	673	60.92	458	178	1	1687	317	215	672	0.0	630
ABCDEF_34098	UniRef50_Q9LE59	0.0	Normal: 457	464	697	1283	1312	539	BlastAnno: 2199	UniRef50_Q9LE59	673	60.92	458	178	1	1687	317	215	672	0.0	628
ABCDEF_10848	UniRef50_Q9M156	0.0	Normal: 157	2988	163	408	772	1566	BlastAnno: 1810	UniRef50_Q9M156	480	60.09	456	176	4	1700	336	4	454	0.0	553
ABCDEF_41182	UniRef50_Q9LHZ7	0.0	Normal: 150	2169	221	814	1407	495	BlastAnno: 3604	UniRef50_Q9LHZ7	1170	51.03	870	333	15	2822	456	299	1156	0.0	826
ABCDEF_40981	UniRef50_Q96301	0.0	Normal: 156	97	110	160	179	173	BlastAnno: 1225	UniRef50_Q96301	914	86.92	344	45	0	1112	81	394	737	0.0	652
ABCDEF_9506	UniRef50_Q9LHZ7	0.0	Normal: 1302	2128	1494	3356	4644	2206	BlastAnno: 3751	UniRef50_Q9LHZ7	1170	49.38	891	343	17	927	3359	299	1161	0.0	812
ABCDEF_9500	UniRef50_Q9LHZ7	0.0	Normal: 183	188	186	576	498	260	BlastAnno: 1717	UniRef50_Q9LHZ7	1170	47.98	644	234	14	3	1715	418	1033	0.0	571
ABCDEF_100	UniRef50_Q9M8Y0	0.0	Normal: 228	309	224	416	461	141	BlastAnno: 2109	UniRef50_Q9M8Y0	977	77.49	533	120	0	1610	12	50	582	0.0	884
ABCDEF_33659	UniRef50_Q9LHZ7	0.0	Normal: 1282	1948	2248	5826	10642	2083	BlastAnno: 4289	UniRef50_Q9LHZ7	1170	48.70	887	345	15	2955	538	299	1156	0.0	788
ABCDEF_37667	UniRef50_Q9LHZ7	0.0	Normal: 248	3617	251	1237	2078	970	BlastAnno: 3161	UniRef50_Q9LHZ7	1170	49.72	885	335	14	2671	260	301	1156	0.0	810
ABCDEF_47673	UniRef50_Q9LHZ7	0.0	Normal: 876	296	828	1628	1721	1172	BlastAnno: 3878	UniRef50_Q9LHZ7	1170	80.88	1161	186	8	3642	232	15	1163	0.0	1865
ABCDEF_99	UniRef50_Q9M8Y0	0.0	Normal: 272	330	222	398	423	145	BlastAnno: 2094	UniRef50_Q9M8Y0	977	77.36	561	127	0	1683	1	50	610	0.0	929
ABCDEF_20658	UniRef50_Q8VZK9	0.0	Normal: 373	33	193	64	503	141	BlastAnno: 2316	UniRef50_Q8VZK9	729	53.35	508	221	7	47	1564	11	504	0.0	559
ABCDEF_10183	UniRef50_A7XDQ9	0.0	Normal: 422	455	346	577	378	610	BlastAnno: 2362	UniRef50_A7XDQ9	684	50.94	693	307	12	281	2287	1	684	0.0	670
ABCDEF_30745	UniRef50_A7XDQ9	0.0	Normal: 1209	351	1031	1645	1226	635	BlastAnno: 2646	UniRef50_A7XDQ9	684	57.58	554	227	6	546	2189	133	684	0.0	664
ABCDEF_13336	UniRef50_Q7PC69	0.0	Normal: 148	563	194	957	1575	816	BlastAnno: 2584	UniRef50_Q7PC69	745	71.56	559	136	6	744	2351	187	745	0.0	730
ABCDEF_21426	UniRef50_Q8RU27	0.0	Normal: 2658	1979	3417	6157	9464	2599	BlastAnno: 1530	UniRef50_Q8RU27	366	89.44	360	34	2	1358	282	1	357	0.0	667
ABCDEF_71242	UniRef50_Q9ZV71	0.0	Normal: 134	122	179	480	754	119	BlastAnno: 1835	UniRef50_Q9ZV71	409	73.35	409	103	4	231	1439	1	409	0.0	587
ABCDEF_52020	UniRef50_Q6YWK8	0.0	Normal: 1635	1534	1975	4284	10353	2289	BlastAnno: 2394	UniRef50_Q6YWK8	570	58.37	514	185	2	403	1857	57	570	0.0	607
ABCDEF_51269	UniRef50_Q6YWK8	0.0	Normal: 147	85	103	21	33	438	BlastAnno: 1864	UniRef50_Q6YWK8	570	54.28	514	204	2	1646	198	57	570	0.0	586
ABCDEF_30753	UniRef50_Q9ZV71	0.0	Normal: 290	497	417	629	1013	634	BlastAnno: 1502	UniRef50_Q9ZV71	409	72.32	401	107	2	311	1501	1	401	0.0	590
ABCDEF_4233	UniRef50_Q9ZV71	0.0	Normal: 369	412	550	936	860	750	BlastAnno: 2004	UniRef50_Q9ZV71	409	72.13	409	110	2	370	1584	1	409	0.0	613
ABCDEF_17525	UniRef50_Q8RU27	0.0	Normal: 1500	1289	2149	3711	4446	1069	BlastAnno: 1507	UniRef50_Q8RU27	366	91.35	347	30	0	1371	331	3	349	0.0	671
ABCDEF_18005	UniRef50_Q6YWK8	0.0	Normal: 1842	2042	2515	4214	5208	2842	BlastAnno: 2305	UniRef50_Q6YWK8	570	59.14	514	180	2	1948	497	57	570	0.0	642
ABCDEF_41140	UniRef50_Q6YWK8	0.0	Normal: 86	1314	93	753	1787	2076	BlastAnno: 2346	UniRef50_Q6YWK8	570	60.00	510	174	2	431	1870	57	566	0.0	669
ABCDEF_18002	UniRef50_Q9LHZ7	0.0	Normal: 376	127	367	601	1190	520	BlastAnno: 1605	UniRef50_Q9LHZ7	1170	89.75	400	41	0	1603	404	771	1170	0.0	732
ABCDEF_13339	UniRef50_Q7PC69	0.0	Normal: 127	814	183	541	1135	1144	BlastAnno: 2610	UniRef50_Q7PC69	745	70.48	559	141	6	725	2329	187	745	0.0	729
ABCDEF_41141	UniRef50_Q6YWK8	0.0	Normal: 67	1107	84	708	1690	1854	BlastAnno: 2461	UniRef50_Q6YWK8	570	60.00	510	174	2	1916	477	57	566	0.0	668
ABCDEF_44583	UniRef50_Q9LE59	0.0	Normal: 681	561	559	947	1745	789	BlastAnno: 2470	UniRef50_Q9LE59	673	59.21	456	185	1	1749	385	214	669	0.0	621
ABCDEF_3869	UniRef50_Q9LE59	0.0	Normal: 333	393	415	809	907	754	BlastAnno: 2136	UniRef50_Q9LE59	673	59.05	613	227	4	1827	1	81	673	0.0	699
ABCDEF_28947	UniRef50_Q9SK82	0.0	Normal: 0	851	3	0	0	116	BlastAnno: 1567	UniRef50_Q9SK82	489	62.40	484	176	4	1524	85	1	482	0.0	652
ABCDEF_31119	UniRef50_Q9M8Y0	0.0	Normal: 2212	1279	1917	2933	3341	1803	BlastAnno: 3695	UniRef50_Q9M8Y0	977	81.08	925	174	1	392	3163	50	974	0.0	1583
ABCDEF_37311	UniRef50_Q9LE59	0.0	Normal: 175	133	191	549	421	366	BlastAnno: 1612	UniRef50_Q9LE59	673	62.96	486	153	4	1406	3	4	480	0.0	542
ABCDEF_24190	UniRef50_Q8L7F9	0.0	Normal: 397	265	492	583	553	371	BlastAnno: 2564	UniRef50_Q8L7F9	643	63.91	640	218	4	2353	473	1	640	0.0	850
ABCDEF_30548	UniRef50_Q9FH36	0.0	Normal: 17	916	27	151	420	126	BlastAnno: 1963	UniRef50_Q9FH36	535	72.47	534	138	3	102	1676	1	534	0.0	767
ABCDEF_27416	UniRef50_Q7PC69	0.0	Normal: 575	837	875	1344	2015	936	BlastAnno: 2454	UniRef50_Q7PC69	745	66.11	416	115	4	2337	1168	126	541	0.0	518
ABCDEF_48373	UniRef50_A7XDQ9	0.0	Normal: 1364	773	1336	1451	1791	1202	BlastAnno: 3042	UniRef50_A7XDQ9	684	63.68	691	234	6	2405	360	1	683	0.0	915
ABCDEF_3868	UniRef50_Q9LE59	0.0	Normal: 224	274	301	555	686	524	BlastAnno: 1670	UniRef50_Q9LE59	673	65.31	516	176	1	1556	9	161	673	0.0	696
ABCDEF_31288	UniRef50_Q940V3	0.0	Normal: 247	309	183	101	124	655	BlastAnno: 1658	UniRef50_Q940V3	470	56.01	466	197	7	1614	232	8	470	0.0	537
ABCDEF_52574	UniRef50_Q09332	0.0	Normal: 885	474	1068	1835	1853	816	BlastAnno: 3720	UniRef50_Q09332	1548	47.89	687	310	14	2551	533	859	1511	0.0	598
ABCDEF_79752	UniRef50_Q9ZV71	0.0	Normal: 158	183	245	400	379	352	BlastAnno: 1266	UniRef50_Q9ZV71	409	71.39	360	99	2	31	1098	1	360	0.0	526
ABCDEF_9509	UniRef50_Q9LHZ7	0.0	Normal: 1296	2171	1498	3410	4683	2232	BlastAnno: 3897	UniRef50_Q9LHZ7	1170	49.38	891	343	17	1073	3505	299	1161	0.0	812
ABCDEF_48200	UniRef50_Q6YWK8	0.0	Normal: 1768	576	3156	4431	6634	491	BlastAnno: 2274	UniRef50_Q6YWK8	570	57.98	514	185	4	291	1739	57	570	0.0	642
ABCDEF_24155	UniRef50_Q9ZVI7	0.0	Normal: 503	247	607	1676	1833	578	BlastAnno: 2708	UniRef50_Q9ZVI7	619	52.33	621	264	11	306	2123	15	618	0.0	601
ABCDEF_21246	UniRef50_Q9SKT6	0.0	Normal: 92	107	188	380	834	84	BlastAnno: 1791	UniRef50_Q9SKT6	536	56.61	484	204	5	123	1562	52	533	0.0	568
ABCDEF_32317	UniRef50_Q8RU27	0.0	Normal: 12	9	3	118	800	62	BlastAnno: 1076	UniRef50_Q8RU27	366	87.02	285	37	0	1075	221	64	348	0.0	535
ABCDEF_9502	UniRef50_Q9LHZ7	0.0	Normal: 610	486	568	1331	1403	591	BlastAnno: 1961	UniRef50_Q9LHZ7	1170	48.64	625	249	13	3	1742	564	1161	0.0	570
ABCDEF_23298	UniRef50_Q9FH36	0.0	Normal: 128	527	130	223	314	864	BlastAnno: 2832	UniRef50_Q9FH36	535	83.16	196	33	0	696	1283	340	535	0.0	357
ABCDEF_40595	UniRef50_O80898	0.0	Normal: 229	135	103	25	307	325	BlastAnno: 2413	UniRef50_O80898	757	44.68	761	378	13	95	2299	10	753	0.0	587
ABCDEF_63947	UniRef50_Q9SK82	0.0	Normal: 0	267	0	0	71	0	BlastAnno: 1446	UniRef50_Q9SK82	489	58.33	468	189	4	60	1445	1	468	0.0	592
ABCDEF_67847	UniRef50_Q9M8Y0	0.0	Normal: 794	476	871	1081	1401	868	BlastAnno: 1591	UniRef50_Q9M8Y0	977	84.86	370	56	0	1589	480	605	974	0.0	685
ABCDEF_65666	UniRef50_Q09332	0.0	Normal: 800	441	946	1606	1637	735	BlastAnno: 3518	UniRef50_Q09332	1548	47.54	671	305	13	1162	3087	859	1511	0.0	586
ABCDEF_38641	UniRef50_Q9FWA4	0.0	Normal: 674	967	1162	2603	4404	2475	BlastAnno: 1599	UniRef50_Q9FWA4	561	81.35	386	72	0	1	1158	176	561	0.0	641
ABCDEF_17914	UniRef50_Q9SKT6	0.0	Normal: 784	567	886	1447	1439	1424	BlastAnno: 2049	UniRef50_Q9SKT6	536	72.95	536	140	2	204	1796	1	536	0.0	785
ABCDEF_18286	UniRef50_Q6NQB7	0.0	Normal: 1131	1108	1225	1872	1722	3329	BlastAnno: 1752	UniRef50_Q6NQB7	393	73.80	397	81	6	1527	367	10	393	0.0	607
ABCDEF_119	UniRef50_Q8RU27	0.0	Normal: 1613	1155	2249	3558	7663	1775	BlastAnno: 1532	UniRef50_Q8RU27	366	88.33	360	38	2	1306	230	1	357	0.0	663
ABCDEF_47603	UniRef50_Q9M9Y5	0.0	Normal: 2609	8779	1664	1103	2085	22991	BlastAnno: 2830	UniRef50_Q9M9Y5	589	54.04	594	267	3	2475	694	1	588	0.0	654
ABCDEF_24192	UniRef50_Q8L7F9	0.0	Normal: 391	244	465	553	497	345	BlastAnno: 2477	UniRef50_Q8L7F9	643	58.99	434	165	4	212	1474	1	434	0.0	513
ABCDEF_17482	UniRef50_Q9SKT6	0.0	Normal: 615	379	816	1068	1470	852	BlastAnno: 2083	UniRef50_Q9SKT6	536	71.11	540	147	4	1915	311	1	536	0.0	786
ABCDEF_85909	UniRef50_Q8VZK9	0.0	Normal: 28	45	9	10	44	127	BlastAnno: 1473	UniRef50_Q8VZK9	729	55.37	475	198	6	1419	1	11	473	0.0	536
ABCDEF_9503	UniRef50_Q9LHZ7	0.0	Normal: 487	544	524	1242	1320	833	BlastAnno: 2087	UniRef50_Q9LHZ7	1170	50.35	574	236	8	57	1712	615	1161	0.0	567
ABCDEF_40594	UniRef50_O80898	0.0	Normal: 256	127	102	41	331	326	BlastAnno: 2417	UniRef50_O80898	757	45.76	719	348	12	213	2291	51	753	0.0	585
ABCDEF_17141	UniRef50_Q9LHZ7	0.0	Normal: 74	1734	69	380	701	333	BlastAnno: 3337	UniRef50_Q9LHZ7	1170	50.92	868	335	14	2539	179	299	1156	0.0	816
ABCDEF_33658	UniRef50_Q9LHZ7	0.0	Normal: 384	308	395	805	1003	602	BlastAnno: 2961	UniRef50_Q9LHZ7	1170	48.76	888	343	14	204	2621	299	1156	0.0	792
ABCDEF_20579	UniRef50_Q7PC69	0.0	Normal: 1007	795	1238	2311	2309	1345	BlastAnno: 3113	UniRef50_Q7PC69	745	59.97	767	229	14	2667	535	1	745	0.0	768

ABCDEF_31118	UniRef50_Q9M8Y0	0.0	Normal: 2135	1267	1900	3004	3329	1757	BlastAnno: 3787	UniRef50_Q9M8Y0	977	81.10	709	133	1	1132	3255	266	974	0.0	1217
ABCDEF_9499	UniRef50_Q9LHZ7	0.0	Normal: 205	207	215	632	538	275	BlastAnno: 1794	UniRef50_Q9LHZ7	1170	48.88	667	240	14	11	1792	395	1033	0.0	605
ABCDEF_48199	UniRef50_Q6YWK8	0.0	Normal: 1506	467	2429	3094	4233	439	BlastAnno: 1997	UniRef50_Q6YWK8	570	63.10	439	146	2	291	1559	57	495	0.0	610
ABCDEF_40983	UniRef50_Q96301	0.0	Normal: 406	261	343	392	415	433	BlastAnno: 2265	UniRef50_Q96301	914	79.17	504	99	1	114	1625	394	891	0.0	854
ABCDEF_17140	UniRef50_Q9LHZ7	0.0	Normal: 45	1049	49	216	386	208	BlastAnno: 2293	UniRef50_Q9LHZ7	1170	52.34	770	281	13	2138	57	397	1156	0.0	739
ABCDEF_18352	UniRef50_Q9FWA4	0.0	Normal: 2427	2688	2969	5519	6547	4855	BlastAnno: 2224	UniRef50_Q9FWA4	561	69.31	505	148	3	1887	382	61	561	0.0	683
ABCDEF_27767	UniRef50_Q9LHZ7	0.0	Normal: 2576	5141	2841	10249	14583	5031	BlastAnno: 3981	UniRef50_Q9LHZ7	1170	49.66	888	337	16	2927	504	299	1156	0.0	818
ABCDEF_33661	UniRef50_Q9LHZ7	0.0	Normal: 414	340	507	879	790	783	BlastAnno: 2185	UniRef50_Q9LHZ7	1170	48.66	748	277	14	2184	169	440	1156	0.0	662
ABCDEF_14685	UniRef50_A7XDQ9	0.0	Normal: 233	308	134	573	292	334	BlastAnno: 1947	UniRef50_A7XDQ9	684	56.84	468	186	6	569	1945	1	461	0.0	545
ABCDEF_20660	UniRef50_Q8VZK9	0.0	Normal: 22	48	13	15	52	144	BlastAnno: 1473	UniRef50_Q8VZK9	729	55.16	475	199	6	55	1473	11	473	0.0	537
ABCDEF_21315	UniRef50_Q9LHZ7	0.0	Normal: 3412	252	1450	0	79	1228	BlastAnno: 3765	UniRef50_Q9LHZ7	1170	63.78	1096	340	15	308	3508	70	1137	0.0	1333
ABCDEF_533	UniRef50_Q9C8W3	0.0	Normal: 230	204	269	505	411	301	BlastAnno: 1688	UniRef50_Q9C8W3	401	70.33	364	94	2	393	1475	47	399	0.0	555
ABCDEF_34099	UniRef50_Q9LE59	0.0	Normal: 478	491	735	1356	1350	548	BlastAnno: 2158	UniRef50_Q9LE59	673	60.92	458	178	1	1610	240	215	672	0.0	630
ABCDEF_47984	UniRef50_Q7PC69	0.0	Normal: 663	457	734	1362	2147	696	BlastAnno: 2838	UniRef50_Q7PC69	745	59.27	712	226	9	373	2316	34	745	0.0	702
ABCDEF_51451	UniRef50_Q9LE59	0.0	Normal: 1883	1782	2202	4337	4350	2667	BlastAnno: 2874	UniRef50_Q9LE59	673	74.16	685	165	6	2559	505	1	673	0.0	982
ABCDEF_13370	UniRef50_Q8VZK9	0.0	Normal: 290	87	317	377	105	258	BlastAnno: 2033	UniRef50_Q8VZK9	729	51.65	635	278	11	2031	154	112	726	0.0	612
ABCDEF_48239	UniRef50_Q9LHZ7	0.0	Normal: 511	163	422	1154	1316	510	BlastAnno: 2714	UniRef50_Q9LHZ7	1170	76.20	769	156	9	478	2712	41	806	0.0	1201
ABCDEF_13338	UniRef50_Q7PC69	0.0	Normal: 335	468	417	2639	4443	616	BlastAnno: 2525	UniRef50_Q7PC69	745	62.45	727	209	12	298	2292	21	745	0.0	781
ABCDEF_24193	UniRef50_Q8L7F9	0.0	Normal: 113	72	132	203	180	119	BlastAnno: 1679	UniRef50_Q8L7F9	643	60.54	479	176	4	212	1609	1	479	0.0	592
ABCDEF_48514	UniRef50_Q9ZV71	0.0	Normal: 540	580	601	771	1033	926	BlastAnno: 1920	UniRef50_Q9ZV71	409	78.14	398	85	2	1618	431	12	409	0.0	637
2.4.1.101(alpha-1,3-mannosyl-glycoprotein 2-beta-N-acetylglucosaminyltransferase;)																					
ABCDEF_26986	UniRef50_Q9XGM8	0.0	Normal: 106	55	92	159	243	127	BlastAnno: 1191	UniRef50_Q9XGM8	444	77.78	333	74	0	1190	192	112	444	0.0	567
ABCDEF_26985	UniRef50_Q9XGM8	0.0	Normal: 69	42	63	148	180	88	BlastAnno: 1148	UniRef50_Q9XGM8	444	77.78	333	74	0	1147	149	112	444	0.0	567
ABCDEF_26982	UniRef50_Q9XGM8	0.0	Normal: 188	115	153	310	426	221	BlastAnno: 1924	UniRef50_Q9XGM8	444	73.64	440	116	0	1776	457	5	444	0.0	705
ABCDEF_26983	UniRef50_Q9XGM8	0.0	Normal: 234	126	196	335	497	264	BlastAnno: 1964	UniRef50_Q9XGM8	444	73.64	440	116	0	1816	497	5	444	0.0	705
2.4.1.117(dolichyl-phosphate beta-glucosyltransferase;)																					
ABCDEF_5954	UniRef50_Q9Y673	1e-71	Normal: 115	73	108	240	234	128	BlastAnno: 1077	UniRef50_Q9Y673	324	47.12	278	129	4	78	908	57	317	1e-71	234
2.4.1.119(dolichyl-diphosphooligosaccharide---protein glycotransferase;)																					
ABCDEF_48073	UniRef50_B6KJL1	0.0	Normal: 2214	1214	2170	3118	4131	2301	BlastAnno: 2792	UniRef50_B6KJL1	753	45.26	738	355	7	2602	398	54	745	0.0	588
ABCDEF_28256	UniRef50_P46977	0.0	Normal: 2426	1044	2529	4068	4468	1518	BlastAnno: 2888	UniRef50_P46977	705	60.37	709	262	5	2612	498	7	700	0.0	841
2.4.1.130(dolichyl-phosphate-mannose---glycolipid alpha-mannosyltransferase;)																					
No Correspond Unigene																					
2.4.1.131(glycolipid 2-alpha-mannosyltransferase;)																					
ABCDEF_5906	UniRef50_Q2TAA5	5e-119	Normal: 45	54	41	77	101	81	BlastAnno: 1169	UniRef50_Q2TAA5	492	48.40	376	188	4	1116	7	57	432	5e-119	363
2.4.1.132(glycolipid 3-alpha-mannosyltransferase;)																					
ABCDEF_17580	UniRef50_O94738	1e-85	Normal: 56	38	59	74	107	90	BlastAnno: 996	UniRef50_O94738	455	49.82	285	123	6	817	2	3	280	1e-85	273
2.4.1.141(N-acetylglucosaminyldiphosphodolichol N-acetylglucosaminyltransferase;)																					
ABCDEF_13935	UniRef50_Q9NP73	3e-35	Normal: 75	42	79	87	117	135	BlastAnno: 826	UniRef50_Q9NP73	165	42.07	164	93	1	736	245	2	163	3e-35	132
2.4.1.142(chitobiosyldiphosphodolichol beta-mannosyltransferase;)																					
ABCDEF_21126	UniRef50_Q9BT22	1e-111	Normal: 285	250	242	469	609	458	BlastAnno: 1877	UniRef50_Q9BT22	464	43.22	472	225	11	212	1612	28	461	1e-111	352
2.4.1.143(alpha-1,6-mannosyl-glycoprotein 2-beta-N-acetylglucosaminyltransferase;)																					
ABCDEF_44902	UniRef50_Q9FT88	0.0	Normal: 685	658	903	1695	1719	1028	BlastAnno: 2042	UniRef50_Q9FT88	430	74.36	351	89	1	1373	324	71	421	0.0	576
2.4.1.144(beta-1,4-mannosyl-glycoprotein 4-beta-N-acetylglucosaminyltransferase;)																					
No Correspond Unigene																					
2.4.1.145(alpha-1,3-mannosyl-glycoprotein 4-beta-N-acetylglucosaminyltransferase;)																					
No Correspond Unigene																					
2.4.1.155(alpha-1,6-mannosyl-glycoprotein 6-beta-N-acetylglucosaminyltransferase;)																					
No Correspond Unigene																					
2.4.1.201(alpha-1,6-mannosyl-glycoprotein 4-beta-N-acetylglucosaminyltransferase;)																					
No Correspond Unigene																					
2.4.1.38(beta-N-acetylglucosaminylglycopeptide beta-1,4-galactosyltransferase;)																					
No Correspond Unigene																					
2.4.1.68(glycoprotein 6-alpha-L-fucosyltransferase;)																					
No Correspond Unigene																					
2.4.1.83(dolichyl-phosphate beta-D-mannosyltransferase;)																					
ABCDEF_4395	UniRef50_O60762	1e-110	Normal: 268	129	265	263	411	299	BlastAnno: 968	UniRef50_O60762	260	64.29	238	83	1	927	220	23	260	1e-110	330
2.4.99.1(beta-galactoside alpha-2,6-sialyltransferase;)																					
No Correspond Unigene																					
2.7.1.108(dolichol kinase;)																					
ABCDEF_37470	UniRef50_Q9UPQ8	3e-32	Normal: 120	137	120	179	209	239	BlastAnno: 1992	UniRef50_Q9UPQ8	538	39.91	233	120	6	967	317	297	525	3e-32	137
2.7.8.15(UDP-N-acetylglucosamine---dolichyl-phosphate N-acetylglucosaminephosphotransferase;)																					
ABCDEF_90440	UniRef50_Q9H3H5	2e-86	Normal: 76	43	103	162	144	94	BlastAnno: 977	UniRef50_Q9H3H5	408	51.45	311	129	5	934	41	5	306	2e-86	274
3.1.3.51(dolichyl-phosphatase;)																					
No Correspond Unigene																					
3.1.4.48(dolichylphosphate-glucose phosphodiesterase;)																					
No Correspond Unigene																					
3.2.1.106(mannosyl-oligosaccharide glucosidase;)																					
No Correspond Unigene																					
3.2.1.113(mannosyl-oligosaccharide 1,2-alpha-mannosidase;)																					
ABCDEF_25164	UniRef50_Q93Y37	0.0	Normal: 767	679	1074	2657	3100	1098	BlastAnno: 2667	UniRef50_Q93Y37	624	68.20	632	184	6	433	2310	1	621	0.0	822
3.2.1.114(mannosyl-oligosaccharide 1,3-1,6-alpha-mannosidase;)																					
ABCDEF_20466	UniRef50_Q16706	0.0	Normal: 1468	901	1501	2183	2291	1145	BlastAnno: 3954	UniRef50_Q16706	1144	38.40	1060	596	22	424	3564	128	1143	0.0	717

3.2.1.84(glucan 1,3-alpha-glucosidase;)																			
ABCDEF_28057	UniRef50_Q14697	0.0	Normal: 1705	954	1963	3447	3390	1297	BlastAnno: 3218	UniRef50_Q14697	944	42.74	1006	454	16	70	2955	16	943	0.0	799
ABCDEF_28056	UniRef50_Q14697	0.0	Normal: 1963	1109	2434	4044	3767	1328	BlastAnno: 3117	UniRef50_Q14697	944	44.34	972	452	16	3044	264	16	943	0.0	820
3.6.1.43(dolichyldiphosphatase;)																			
ABCDEF_27689	UniRef50_Q86YN1	9e-33	Normal: 134	207	107	172	456	375	BlastAnno: 1257	UniRef50_Q86YN1	238	38.94	226	125	3	1038	388	11	232	9e-33	130
>00511 Other glycan degradation																			
3.2.1.18(exo-alpha-sialidase;)																			
ABCDEF_30704	UniRef50_B0RQ59	6e-65	Normal: 92	41	67	66	82	84	BlastAnno: 1280	UniRef50_B0RQ59	355	42.63	319	163	7	145	1056	36	349	6e-65	220
3.2.1.23(beta-galactosidase;)																			
ABCDEF_48113	UniRef50_Q6Z6K4	0.0	Normal: 61	2624	32	127	673	5080	BlastAnno: 2898	UniRef50_Q6Z6K4	729	63.35	704	250	4	2505	394	33	728	0.0	931
ABCDEF_23777	UniRef50_Q6Z6K4	0.0	Normal: 219	63	227	164	235	472	BlastAnno: 2261	UniRef50_Q6Z6K4	729	66.38	690	225	5	2	2071	46	728	0.0	944
ABCDEF_3074	UniRef50_Q6Z6K4	0.0	Normal: 431	1551	321	2473	394	19728	BlastAnno: 3342	UniRef50_Q6Z6K4	729	67.09	702	221	6	415	2517	35	727	0.0	994
ABCDEF_43854	UniRef50_Q6Z6K4	0.0	Normal: 1	9	1	105	49	28	BlastAnno: 1781	UniRef50_Q6Z6K4	729	58.79	512	180	6	1	1536	147	627	0.0	595
ABCDEF_44469	UniRef50_Q8W0A1	0.0	Normal: 162	225	175	701	194	597	BlastAnno: 2319	UniRef50_Q8W0A1	827	46.10	679	324	12	2236	221	183	826	0.0	583
ABCDEF_13827	UniRef50_Q6Z6K4	0.0	Normal: 219	1054	213	1958	627	863	BlastAnno: 3038	UniRef50_Q6Z6K4	729	57.90	715	270	8	419	2539	36	727	0.0	825
ABCDEF_21624	UniRef50_Q6Z6K4	0.0	Normal: 4958	5483	4088	4956	4209	6920	BlastAnno: 3330	UniRef50_Q6Z6K4	729	62.64	696	251	4	2975	888	32	718	0.0	932
ABCDEF_43857	UniRef50_Q6Z6K4	0.0	Normal: 2	9	4	110	53	30	BlastAnno: 1655	UniRef50_Q6Z6K4	729	60.07	546	187	6	1	1638	147	661	0.0	655
ABCDEF_43861	UniRef50_Q6Z6K4	0.0	Normal: 4	15	5	162	72	47	BlastAnno: 1891	UniRef50_Q6Z6K4	729	65.21	526	179	3	107	1684	34	555	0.0	719
ABCDEF_84604	UniRef50_Q6Z6K4	0.0	Normal: 34	1696	14	81	379	2927	BlastAnno: 2528	UniRef50_Q6Z6K4	729	62.99	670	240	4	394	2403	33	694	0.0	881
ABCDEF_13826	UniRef50_Q6Z6K4	0.0	Normal: 184	889	166	1515	526	703	BlastAnno: 2897	UniRef50_Q6Z6K4	729	55.30	613	243	8	2286	472	138	727	0.0	657
ABCDEF_10626	UniRef50_Q6Z6K4	0.0	Normal: 229	909	224	1002	2471	3794	BlastAnno: 3133	UniRef50_Q6Z6K4	729	57.14	700	285	8	498	2588	40	727	0.0	809
ABCDEF_27471	UniRef50_Q6Z6K4	0.0	Normal: 992	533	1105	1489	2430	1240	BlastAnno: 3113	UniRef50_Q6Z6K4	729	64.49	704	240	5	314	2422	35	729	0.0	962
ABCDEF_43858	UniRef50_Q6Z6K4	0.0	Normal: 6	15	7	186	85	54	BlastAnno: 2028	UniRef50_Q6Z6K4	729	65.35	635	213	5	107	2011	34	661	0.0	862
ABCDEF_31373	UniRef50_Q6Z6K4	0.0	Normal: 1333	1029	1160	1241	1216	1855	BlastAnno: 3006	UniRef50_Q6Z6K4	729	52.03	713	319	7	2761	626	37	727	0.0	732
ABCDEF_10066	UniRef50_Q93Z24	0.0	Normal: 999	932	1523	1674	2007	1465	BlastAnno: 3015	UniRef50_Q93Z24	697	65.89	645	208	4	2659	737	61	697	0.0	865
ABCDEF_51808	UniRef50_Q6Z6K4	0.0	Normal: 364	454	209	36	62	1109	BlastAnno: 2817	UniRef50_Q6Z6K4	729	54.56	713	306	8	2582	447	32	727	0.0	791
ABCDEF_48111	UniRef50_Q6Z6K4	0.0	Normal: 41	1700	15	77	347	2953	BlastAnno: 2607	UniRef50_Q6Z6K4	729	67.21	494	157	3	394	1875	33	521	0.0	696
ABCDEF_17519	UniRef50_A5F5U6	0.0	Normal: 1178	1482	1392	2252	1850	3281	BlastAnno: 3706	UniRef50_A5F5U6	1024	39.71	1093	547	21	3469	254	13	1014	0.0	729
ABCDEF_40816	UniRef50_Q6Z6K4	0.0	Normal: 1166	754	898	2543	5298	532	BlastAnno: 3066	UniRef50_Q6Z6K4	729	62.55	705	254	4	2970	856	35	729	0.0	936
ABCDEF_44668	UniRef50_Q8GX69	0.0	Normal: 146	74	84	327	133	152	BlastAnno: 1396	UniRef50_Q8GX69	815	66.74	463	152	2	6	1394	23	483	0.0	671
ABCDEF_43856	UniRef50_Q6Z6K4	0.0	Normal: 12	20	12	195	92	71	BlastAnno: 1873	UniRef50_Q6Z6K4	729	61.89	488	179	5	1	1464	147	627	0.0	615
ABCDEF_44470	UniRef50_Q6Z6K4	0.0	Normal: 287	387	252	1193	287	741	BlastAnno: 2917	UniRef50_Q6Z6K4	729	52.28	723	310	9	2707	551	38	729	0.0	752
ABCDEF_49396	UniRef50_Q6Z6K4	0.0	Normal: 0	452	3	48	33	6872	BlastAnno: 2312	UniRef50_Q6Z6K4	729	65.83	676	221	7	2312	288	62	728	0.0	893
ABCDEF_10631	UniRef50_Q6Z6K4	0.0	Normal: 3079	207	2624	1970	2833	969	BlastAnno: 3058	UniRef50_Q6Z6K4	729	60.74	703	267	4	2943	835	36	729	0.0	917
ABCDEF_48112	UniRef50_Q6Z6K4	0.0	Normal: 60	2628	33	127	663	5106	BlastAnno: 2977	UniRef50_Q6Z6K4	729	67.21	494	157	3	2584	1103	33	521	0.0	696
3.2.1.24(alpha-mannosidase;)																			
ABCDEF_14245	UniRef50_O00754	0.0	Normal: 464	2145	619	2021	1148	4470	BlastAnno: 3404	UniRef50_O00754	1011	39.58	998	537	21	3125	192	52	1003	0.0	699
ABCDEF_3491	UniRef50_O00754	0.0	Normal: 2499	1743	2566	4820	3865	3088	BlastAnno: 3976	UniRef50_O00754	1011	41.63	1004	511	25	3474	526	52	1001	0.0	690
3.2.1.25(beta-mannosidase;)																			
ABCDEF_51831	UniRef50_B0RPH8	3e-27	Normal: 169	138	89	164	111	165	BlastAnno: 1075	UniRef50_B0RPH8	1154	32.87	216	133	5	394	1020	295	505	3e-27	120
3.2.1.45(glucosylceramidase;)																			
ABCDEF_44031	UniRef50_Q8LPR0	0.0	Normal: 88	125	131	209	725	66	BlastAnno: 1470	UniRef50_Q8LPR0	922	62.05	448	154	3	138	1463	448	885	0.0	544
ABCDEF_44034	UniRef50_B9FYQ4	0.0	Normal: 135	179	169	301	1067	84	BlastAnno: 2868	UniRef50_B9FYQ4	919	55.40	879	352	11	2780	168	70	916	0.0	903
ABCDEF_26879	UniRef50_Q8LPR0	0.0	Normal: 261	199	270	652	497	368	BlastAnno: 1657	UniRef50_Q8LPR0	922	57.17	523	192	6	1596	46	424	920	0.0	554
ABCDEF_74992	UniRef50_Q9LRN1	0.0	Normal: 163	140	183	229	464	205	BlastAnno: 1980	UniRef50_Q9LRN1	937	78.54	424	89	1	1483	212	516	937	0.0	650
ABCDEF_9833	UniRef50_Q9LRN1	0.0	Normal: 495	475	508	789	1098	494	BlastAnno: 3769	UniRef50_Q9LRN1	937	70.71	990	228	7	3251	309	1	937	0.0	1369
ABCDEF_26878	UniRef50_B9FYQ4	0.0	Normal: 376	308	408	1066	703	505	BlastAnno: 3016	UniRef50_B9FYQ4	919	55.35	925	370	12	2821	77	19	910	0.0	960
ABCDEF_44030	UniRef50_B9FYQ4	0.0	Normal: 166	221	229	394	1305	124	BlastAnno: 3109	UniRef50_B9FYQ4	919	54.42	928	383	11	183	2942	21	916	0.0	947
3.2.1.51(alpha-L-fucosidase;)																			
ABCDEF_3463	UniRef50_B9SCI4	0.0	Normal: 264	287	222	535	445	640	BlastAnno: 1806	UniRef50_B9SCI4	562	69.78	450	132	3	1531	191	64	512	0.0	636
ABCDEF_37241	UniRef50_Q8L7W8	0.0	Normal: 246	163	318	334	165	189	BlastAnno: 1767	UniRef50_Q8L7W8	843	64.86	498	164	3	50	1528	352	843	0.0	699
3.2.1.52(beta-N-acetylhexosaminidase;)																			
ABCDEF_16822	UniRef50_Q54K56	3e-109	Normal: 543	493	486	692	917	660	BlastAnno: 1760	UniRef50_Q54K56	564	36.42	519	294	10	1587	94	59	562	3e-109	347
3.2.1.96(mannosyl-glycoprotein endo-beta-N-acetylglucosaminidase;)																			
ABCDEF_40422	UniRef50_P0C7A1	2e-94	Normal: 258	367	236	365	262	514	BlastAnno: 2391	UniRef50_P0C7A1	728	41.33	392	221	4	2016	844	80	463	2e-94	319
3.5.1.26(N4-(beta-N-acetylglucosaminyl)-L-asparaginase;)																			
ABCDEF_30989	UniRef50_Q8MR45	6e-95	Normal: 93	190	68	85	160	443	BlastAnno: 1355	UniRef50_Q8MR45	393	52.00	325	140	7	1119	172	54	371	6e-95	300
>00512 Mucin type O-Glycan biosynthesis																			
2.4.1.-(Transferases;)																			
ABCDEF_18004	UniRef50_Q6YWK8	0.0	Normal: 1562	1736	2009	3452	4438	2400	BlastAnno: 2298	UniRef50_Q6YWK8	570	65.27	429	136	1	1941	694	57	485	0.0	608
ABCDEF_52379	UniRef50_Q9LHZ7	0.0	Normal: 4015	4921	4546	10520	13430	4731	BlastAnno: 3479	UniRef50_Q9LHZ7	1170	48.04	893	342	16	825	3242	299	1156	0.0	787
ABCDEF_48593	UniRef50_Q9SK82	0.0	Normal: 12	420	19	343	763	253	BlastAnno: 1834	UniRef50_Q9SK82	489	60.08	481	186	4	1714	278	10	486	0.0	633
ABCDEF_13375	UniRef50_Q8VZK9	0.0	Normal: 108	123	208	526	34	294	BlastAnno: 1738	UniRef50_Q8VZK9	729	53.62	552	234	8	94	1737	18	551	0.0	582
ABCDEF_40982	UniRef50_Q96301	0.0	Normal: 1037	706	819	1249	1084	920	BlastAnno: 3418	UniRef50_Q96301	914	79.60	902	173	3	243	2948	1	891	0.0	1506
ABCDEF_47674	UniRef50_Q9LHZ7	0.0	Normal: 274	92	202	599	505	280	BlastAnno: 2526	UniRef50_Q9LHZ7	1170	75.93	777	151	8	2260	2	15	779	0.0	1177
ABCDEF_34097	UniRef50_Q9LE59	0.0	Normal: 626	613	923	1797	1829	692	BlastAnno: 2521	UniRef50_Q9LE59	673	60.92	458	178	1	1687	317	215	672	0.0	630
ABCDEF_34098	UniRef50_Q9LE59	0.0	Normal: 457	464	697	1283	1312	539	BlastAnno: 2199	UniRef50_Q9LE59	673	60.92	458	178	1	1687	317	215	672	0.0	628
ABCDEF_10848	UniRef50_Q9M156	0.0	Normal: 157	2988	163	408	772	1566	BlastAnno: 1810	UniRef50_Q9M156	480	60.09	456	176	4	1700	336	4	454	0.0	553
ABCDEF_41182	UniRef50_Q9LHZ7	0.0	Normal: 150	2169	221	814	1407	495	BlastAnno: 3604	UniRef50_Q9LHZ7	1170	51.03	870	333	15	2822	456	299	1156	0.0	826
ABCDEF_40981	UniRef50_Q96301	0.0	Normal: 156	97	110	160	179	173	BlastAnno: 1225	UniRef50_Q96301	914	86.92	344	45	0	1112	81	394	737	0.0	652
ABCDEF_9506	UniRef50_Q9LHZ7	0.0	Normal: 1302	2128	1494	3356	4644	2206	BlastAnno: 3751	UniRef50_Q9LHZ7	1170	49.38	891	343	17	927	3359	299	1161	0.0	812
ABCDEF_9500	UniRef50_Q9LHZ7	0.0	Normal: 183	188	186	576	498	260	BlastAnno: 1717	UniRef50_Q9LHZ7	1170	47.98	644	234	14	3	1715	418	1033	0.0	571

ABCDEF_100	UniRef50_Q9M8Y0	0.0	Normal: 228	309	224	416	461	141	BlastAnno: 2109	UniRef50_Q9M8Y0	977	77.49	533	120	0	1610	12	50	582	0.0	884
ABCDEF_33659	UniRef50_Q9LHZ7	0.0	Normal: 1282	1948	2248	5826	10642	2083	BlastAnno: 4289	UniRef50_Q9LHZ7	1170	48.70	887	345	15	2955	538	299	1156	0.0	788
ABCDEF_37667	UniRef50_Q9LHZ7	0.0	Normal: 248	3617	251	1237	2078	970	BlastAnno: 3161	UniRef50_Q9LHZ7	1170	49.72	885	335	14	2671	260	301	1156	0.0	810
ABCDEF_47673	UniRef50_Q9LHZ7	0.0	Normal: 876	296	828	1628	1721	1172	BlastAnno: 3878	UniRef50_Q9LHZ7	1170	80.88	1161	186	8	3642	232	15	1163	0.0	1865
ABCDEF_99	UniRef50_Q9M8Y0	0.0	Normal: 272	330	222	398	423	145	BlastAnno: 2094	UniRef50_Q9M8Y0	977	77.36	561	127	0	1683	1	50	610	0.0	929
ABCDEF_20658	UniRef50_Q8VZK9	0.0	Normal: 373	33	193	64	503	141	BlastAnno: 2316	UniRef50_Q8VZK9	729	53.35	508	221	7	47	1564	11	504	0.0	559
ABCDEF_10183	UniRef50_A7XDQ9	0.0	Normal: 422	455	346	577	378	610	BlastAnno: 2362	UniRef50_A7XDQ9	684	50.94	693	307	12	281	2287	1	684	0.0	670
ABCDEF_30745	UniRef50_A7XDQ9	0.0	Normal: 1209	351	1031	1645	1226	635	BlastAnno: 2646	UniRef50_A7XDQ9	684	57.58	554	227	6	546	2189	133	684	0.0	664
ABCDEF_13336	UniRef50_Q7PC69	0.0	Normal: 148	563	194	957	1575	816	BlastAnno: 2584	UniRef50_Q7PC69	745	71.56	559	136	6	744	2351	187	745	0.0	730
ABCDEF_21426	UniRef50_Q8RU27	0.0	Normal: 2658	1979	3417	6157	9464	2599	BlastAnno: 1530	UniRef50_Q8RU27	366	89.44	360	34	2	1358	282	1	357	0.0	667
ABCDEF_71242	UniRef50_Q9ZV71	0.0	Normal: 134	122	179	480	754	119	BlastAnno: 1835	UniRef50_Q9ZV71	409	73.35	409	103	4	231	1439	1	409	0.0	587
ABCDEF_52020	UniRef50_Q6YWK8	0.0	Normal: 1635	1534	1975	4284	10353	2289	BlastAnno: 2394	UniRef50_Q6YWK8	570	58.37	514	185	2	403	1857	57	570	0.0	607
ABCDEF_51269	UniRef50_Q6YWK8	0.0	Normal: 147	85	103	21	33	438	BlastAnno: 1864	UniRef50_Q6YWK8	570	54.28	514	204	2	1646	198	57	570	0.0	586
ABCDEF_30753	UniRef50_Q9ZV71	0.0	Normal: 290	497	417	629	1013	634	BlastAnno: 1502	UniRef50_Q9ZV71	409	72.32	401	107	2	311	1501	1	401	0.0	590
ABCDEF_4233	UniRef50_Q9ZV71	0.0	Normal: 369	412	550	936	860	750	BlastAnno: 2004	UniRef50_Q9ZV71	409	72.13	409	110	2	370	1584	1	409	0.0	613
ABCDEF_17525	UniRef50_Q8RU27	0.0	Normal: 1500	1289	2149	3711	4446	1069	BlastAnno: 1507	UniRef50_Q8RU27	366	91.35	347	30	0	1371	331	3	349	0.0	671
ABCDEF_18005	UniRef50_Q6YWK8	0.0	Normal: 1842	2042	2515	4214	5208	2842	BlastAnno: 2305	UniRef50_Q6YWK8	570	59.14	514	180	2	1948	497	57	570	0.0	642
ABCDEF_41140	UniRef50_Q6YWK8	0.0	Normal: 86	1314	93	753	1787	2076	BlastAnno: 2346	UniRef50_Q6YWK8	570	60.00	510	174	2	431	1870	57	566	0.0	669
ABCDEF_18002	UniRef50_Q9LHZ7	0.0	Normal: 376	127	367	601	1190	520	BlastAnno: 1605	UniRef50_Q9LHZ7	1170	89.75	400	41	0	1603	404	771	1170	0.0	732
ABCDEF_13339	UniRef50_Q7PC69	0.0	Normal: 127	814	183	541	1135	1144	BlastAnno: 2610	UniRef50_Q7PC69	745	70.48	559	141	6	725	2329	187	745	0.0	729
ABCDEF_41141	UniRef50_Q6YWK8	0.0	Normal: 67	1107	84	708	1690	1854	BlastAnno: 2461	UniRef50_Q6YWK8	570	60.00	510	174	2	1916	477	57	566	0.0	668
ABCDEF_44583	UniRef50_Q9LE59	0.0	Normal: 681	561	559	947	1745	789	BlastAnno: 2470	UniRef50_Q9LE59	673	59.21	456	185	1	1749	385	214	669	0.0	621
ABCDEF_3869	UniRef50_Q9LE59	0.0	Normal: 333	393	415	809	907	754	BlastAnno: 2136	UniRef50_Q9LE59	673	59.05	613	227	4	1827	1	81	673	0.0	699
ABCDEF_28947	UniRef50_Q9SK82	0.0	Normal: 0	851	3	0	0	116	BlastAnno: 1567	UniRef50_Q9SK82	489	62.40	484	176	4	1524	85	1	482	0.0	652
ABCDEF_31119	UniRef50_Q9M8Y0	0.0	Normal: 2212	1279	1917	2933	3341	1803	BlastAnno: 3695	UniRef50_Q9M8Y0	977	81.08	925	174	1	392	3163	50	974	0.0	1583
ABCDEF_37311	UniRef50_Q9LE59	0.0	Normal: 175	133	191	549	421	366	BlastAnno: 1612	UniRef50_Q9LE59	673	62.96	486	153	4	1406	3	4	480	0.0	542
ABCDEF_24190	UniRef50_Q8L7F9	0.0	Normal: 397	265	492	583	553	371	BlastAnno: 2564	UniRef50_Q8L7F9	643	63.91	640	218	4	2353	473	1	640	0.0	850
ABCDEF_30548	UniRef50_Q9FH36	0.0	Normal: 17	916	27	151	420	126	BlastAnno: 1963	UniRef50_Q9FH36	535	72.47	534	138	3	102	1676	1	534	0.0	767
ABCDEF_27416	UniRef50_Q7PC69	0.0	Normal: 575	837	875	1344	2015	936	BlastAnno: 2454	UniRef50_Q7PC69	745	66.11	416	115	4	2337	1168	126	541	0.0	518
ABCDEF_48373	UniRef50_A7XDQ9	0.0	Normal: 1364	773	1336	1451	1791	1202	BlastAnno: 3042	UniRef50_A7XDQ9	684	63.68	691	234	6	2405	360	1	683	0.0	915
ABCDEF_3868	UniRef50_Q9LE59	0.0	Normal: 224	274	301	555	686	524	BlastAnno: 1670	UniRef50_Q9LE59	673	65.31	516	176	1	1556	9	161	673	0.0	696
ABCDEF_31288	UniRef50_Q940V3	0.0	Normal: 247	309	183	101	124	655	BlastAnno: 1658	UniRef50_Q940V3	470	56.01	466	197	7	1614	232	8	470	0.0	537
ABCDEF_52574	UniRef50_Q09332	0.0	Normal: 885	474	1068	1835	1853	816	BlastAnno: 3720	UniRef50_Q09332	1548	47.89	687	310	14	2551	533	859	1511	0.0	598
ABCDEF_79752	UniRef50_Q9ZV71	0.0	Normal: 158	183	245	400	379	352	BlastAnno: 1266	UniRef50_Q9ZV71	409	71.39	360	99	2	31	1098	1	360	0.0	526
ABCDEF_9509	UniRef50_Q9LHZ7	0.0	Normal: 1296	2171	1498	3410	4683	2232	BlastAnno: 3897	UniRef50_Q9LHZ7	1170	49.38	891	343	17	1073	3505	299	1161	0.0	812
ABCDEF_48200	UniRef50_Q6YWK8	0.0	Normal: 1768	576	3156	4431	6634	491	BlastAnno: 2274	UniRef50_Q6YWK8	570	57.98	514	185	4	291	1739	57	570	0.0	642
ABCDEF_24155	UniRef50_Q9ZVI7	0.0	Normal: 503	247	607	1676	1833	578	BlastAnno: 2708	UniRef50_Q9ZVI7	619	52.33	621	264	11	306	2123	15	618	0.0	601
ABCDEF_21246	UniRef50_Q9SKT6	0.0	Normal: 92	107	188	380	834	84	BlastAnno: 1791	UniRef50_Q9SKT6	536	56.61	484	204	5	123	1562	52	533	0.0	568
ABCDEF_32317	UniRef50_Q8RU27	0.0	Normal: 12	9	3	118	800	62	BlastAnno: 1076	UniRef50_Q8RU27	366	87.02	285	37	0	1075	221	64	348	0.0	535
ABCDEF_9502	UniRef50_Q9LHZ7	0.0	Normal: 610	486	568	1331	1403	591	BlastAnno: 1961	UniRef50_Q9LHZ7	1170	48.64	625	249	13	3	1742	564	1161	0.0	570
ABCDEF_23298	UniRef50_Q9FH36	0.0	Normal: 128	527	130	223	314	864	BlastAnno: 2832	UniRef50_Q9FH36	535	83.16	196	33	0	696	1283	340	535	0.0	357
ABCDEF_40595	UniRef50_O80898	0.0	Normal: 229	135	103	25	307	325	BlastAnno: 2413	UniRef50_O80898	757	44.68	761	378	13	95	2299	10	753	0.0	587
ABCDEF_63947	UniRef50_Q9SK82	0.0	Normal: 0	267	0	0	71	0	BlastAnno: 1446	UniRef50_Q9SK82	489	58.33	468	189	4	60	1445	1	468	0.0	592
ABCDEF_67847	UniRef50_Q9M8Y0	0.0	Normal: 794	476	871	1081	1401	868	BlastAnno: 1591	UniRef50_Q9M8Y0	977	84.86	370	56	0	1589	480	605	974	0.0	685
ABCDEF_65666	UniRef50_Q09332	0.0	Normal: 800	441	946	1606	1637	735	BlastAnno: 3518	UniRef50_Q09332	1548	47.54	671	305	13	1162	3087	859	1511	0.0	586
ABCDEF_38641	UniRef50_Q9FWA4	0.0	Normal: 674	967	1162	2603	4404	2475	BlastAnno: 1599	UniRef50_Q9FWA4	561	81.35	386	72	0	1	1158	176	561	0.0	641
ABCDEF_17914	UniRef50_Q9SKT6	0.0	Normal: 784	567	886	1447	1439	1424	BlastAnno: 2049	UniRef50_Q9SKT6	536	72.95	536	140	2	204	1796	1	536	0.0	785
ABCDEF_18286	UniRef50_Q6NQB7	0.0	Normal: 1131	1108	1225	1872	1722	3329	BlastAnno: 1752	UniRef50_Q6NQB7	393	73.80	397	81	6	1527	367	10	393	0.0	607
ABCDEF_119	UniRef50_Q8RU27	0.0	Normal: 1613	1155	2249	3558	7663	1775	BlastAnno: 1532	UniRef50_Q8RU27	366	88.33	360	38	2	1306	230	1	357	0.0	663
ABCDEF_47603	UniRef50_Q9M9Y5	0.0	Normal: 2609	8779	1664	1103	2085	22991	BlastAnno: 2830	UniRef50_Q9M9Y5	589	54.04	594	267	3	2475	694	1	588	0.0	654
ABCDEF_24192	UniRef50_Q8L7F9	0.0	Normal: 391	244	465	553	497	345	BlastAnno: 2477	UniRef50_Q8L7F9	643	58.99	434	165	4	212	1474	1	434	0.0	513
ABCDEF_17482	UniRef50_Q9SKT6	0.0	Normal: 615	379	816	1068	1470	852	BlastAnno: 2083	UniRef50_Q9SKT6	536	71.11	540	147	4	1915	311	1	536	0.0	786
ABCDEF_85909	UniRef50_Q8VZK9	0.0	Normal: 28	45	9	10	44	127	BlastAnno: 1473	UniRef50_Q8VZK9	729	55.37	475	198	6	1419	1	11	473	0.0	536
ABCDEF_9503	UniRef50_Q9LHZ7	0.0	Normal: 487	544	524	1242	1320	833	BlastAnno: 2087	UniRef50_Q9LHZ7	1170	50.35	574	236	8	57	1712	615	1161	0.0	567
ABCDEF_40594	UniRef50_O80898	0.0	Normal: 256	127	102	41	331	326	BlastAnno: 2417	UniRef50_O80898	757	45.76	719	348	12	213	2291	51	753	0.0	585
ABCDEF_17141	UniRef50_Q9LHZ7	0.0	Normal: 74	1734	69	380	701	333	BlastAnno: 3337	UniRef50_Q9LHZ7	1170	50.92	868	335	14	2539	179	299	1156	0.0	816
ABCDEF_33658	UniRef50_Q9LHZ7	0.0	Normal: 384	308	395	805	1003	602	BlastAnno: 2961	UniRef50_Q9LHZ7	1170	48.76	888	343	14	204	2621	299	1156	0.0	792
ABCDEF_20579	UniRef50_Q7PC69	0.0	Normal: 1007	795	1238	2311	2309	1345	BlastAnno: 3113	UniRef50_Q7PC69	745	59.97	767	229	14	2667	535	1	745	0.0	768
ABCDEF_31118	UniRef50_Q9M8Y0	0.0	Normal: 2135	1267	1900	3004	3329	1757	BlastAnno: 3787	UniRef50_Q9M8Y0	977	81.10	709	133	1	1132	3255	266	974	0.0	1217
ABCDEF_9499	UniRef50_Q9LHZ7	0.0	Normal: 205	207	215	632	538	275	BlastAnno: 1794	UniRef50_Q9LHZ7	1170	48.88	667	240	14	11	1792	395	1033	0.0	605
ABCDEF_48199	UniRef50_Q6YWK8	0.0	Normal: 1506	467	2429	3094	4233	439	BlastAnno: 1997	UniRef50_Q6YWK8	570	63.10	439	146	2	291	1559	57	495	0.0	610
ABCDEF_40983	UniRef50_Q96301	0.0	Normal: 406	261	343	392	415	433	BlastAnno: 2265	UniRef50_Q96301	914	79.17	504	99	1	114	1625	394	891	0.0	854
ABCDEF_17140	UniRef50_Q9LHZ7	0.0	Normal: 45	1049	49	216	386	208	BlastAnno: 2293	UniRef50_Q9LHZ7	1170	52.34	770	281	13	2138	57	397	1156	0.0	739
ABCDEF_18352	UniRef50_Q9FWA4	0.0	Normal: 2427	2688	2969	5519	6547	4855	BlastAnno: 2224	UniRef50_Q9FWA4	561	69.31	505	148	3	1887	382	61	561	0.0	683
ABCDEF_27767	UniRef50_Q9LHZ7	0.0	Normal: 2576	5141	2841	10249	14583	5031	BlastAnno: 3981	UniRef50_Q9LHZ7	1170	49.66	888	337	16	2927	504	299	1156	0.0	818
ABCDEF_33661	UniRef50_Q9LHZ7	0.0	Normal: 414	340	507	879	790	783	BlastAnno: 2185	UniRef50_Q9LHZ7	1170	48.66	748	277	14	2184	169	440	1156	0.0	662
ABCDEF_14685	UniRef50_A7XDQ9	0.0	Normal: 233	308	134	573	292	334	BlastAnno: 1947	UniRef50_A7XDQ9	684	56.84	468	186	6	569	1945	1	461	0.0	545
ABCDEF_20660	UniRef50_Q8VZK9	0.0	Normal: 22	48	13	15	52	144	BlastAnno: 1473	UniRef50_Q8VZK9	729	55.16	475	199	6	55	1473	11	473	0.0	537
ABCDEF_21315	UniRef50_Q9LHZ7	0.0	Normal: 3412	252	1450	0	79	1228	BlastAnno: 3765	UniRef50_Q9LHZ7	1170	63.78	1096	340	15	308	3508	70	1137	0.0	1333
ABCDEF_533	UniRef50_Q9C8W3	0.0	Normal: 230	204	269	505	411	301	BlastAnno: 1688	UniRef50_Q9C8W3	401	70.33	364	94	2	393	1475	47	399	0.0	555
ABCDEF_34099	UniRef50_Q9LE59	0.0	Normal: 478	491	735	1356	1350	548	BlastAnno: 2158	UniRef50_Q9LE59	673	60.92	458	178	1	1610	240	215	672	0.0	630
ABCDEF_47984	UniRef50_Q7PC69	0.0	Normal: 663	457	734	1362	2147	696	BlastAnno: 2838	UniRef50_Q7PC69	745	59.27	712	226	9	373	2316	34	745	0.0	702

ABCDEF_51451	UniRef50_Q9LE59	0.0	Normal: 1883	1782	2202	4337	4350	2667	BlastAnno: 2874	UniRef50_Q9LE59	673	74.16	685	165	6	2559	505	1	673	0.0	982
ABCDEF_13370	UniRef50_Q8VZK9	0.0	Normal: 290	87	317	377	105	258	BlastAnno: 2033	UniRef50_Q8VZK9	729	51.65	635	278	11	2031	154	112	726	0.0	612
ABCDEF_48239	UniRef50_Q9LHZ7	0.0	Normal: 511	163	422	1154	1316	510	BlastAnno: 2714	UniRef50_Q9LHZ7	1170	76.20	769	156	9	478	2712	41	806	0.0	1201
ABCDEF_13338	UniRef50_Q7PC69	0.0	Normal: 335	468	417	2639	4443	616	BlastAnno: 2525	UniRef50_Q7PC69	745	62.45	727	209	12	298	2292	21	745	0.0	781
ABCDEF_24193	UniRef50_Q8L7F9	0.0	Normal: 113	72	132	203	180	119	BlastAnno: 1679	UniRef50_Q8L7F9	643	60.54	479	176	4	212	1609	1	479	0.0	592
ABCDEF_48514	UniRef50_Q9ZV71	0.0	Normal: 540	580	601	771	1033	926	BlastAnno: 1920	UniRef50_Q9ZV71	409	78.14	398	85	2	1618	431	12	409	0.0	637

2.4.1.102(beta-1,3-galactosyl-O-glycosyl-glycoprotein beta-1,6-N-acetylglucosaminyltransferase;)

No Correspond Unigene

2.4.1.122(glycoprotein-N-acetylgalactosamine 3-beta-galactosyltransferase;)

No Correspond Unigene

2.4.1.147(acetylgalactosaminyl-O-glycosyl-glycoprotein beta-1,3-N-acetylglucosaminyltransferase;)

No Correspond Unigene

2.4.1.148(acetylgalactosaminyl-O-glycosyl-glycoprotein beta-1,6-N-acetylglucosaminyltransferase;)

No Correspond Unigene

2.4.1.41(polypeptide N-acetylgalactosaminyltransferase;)

No Correspond Unigene

2.4.99.3(alpha-N-acetylgalactosaminide alpha-2,6-sialyltransferase)

No Correspond Unigene

2.4.99.4(beta-galactoside alpha-2,3-sialyltransferase;)

ABCDEF_28297	UniRef50_B9SCR7	4e-167	Normal: 573	742	586	923	1062	1024	BlastAnno: 1855	UniRef50_B9SCR7	434	60.36	391	149	4	1454	294	46	434	4e-167	493

>00513 Various types of N-glycan biosynthesis

2.4.1.-(Transferases;)

ABCDEF_18004	UniRef50_Q6YWK8	0.0	Normal: 1562	1736	2009	3452	4438	2400	BlastAnno: 2298	UniRef50_Q6YWK8	570	65.27	429	136	1	1941	694	57	485	0.0	608
ABCDEF_52379	UniRef50_Q9LHZ7	0.0	Normal: 4015	4921	4546	10520	13430	4731	BlastAnno: 3479	UniRef50_Q9LHZ7	1170	48.04	893	342	16	825	3242	299	1156	0.0	787
ABCDEF_48593	UniRef50_Q9SK82	0.0	Normal: 12	420	19	343	763	253	BlastAnno: 1834	UniRef50_Q9SK82	489	60.08	481	186	4	1714	278	10	486	0.0	633
ABCDEF_13375	UniRef50_Q8VZK9	0.0	Normal: 108	123	208	526	34	294	BlastAnno: 1738	UniRef50_Q8VZK9	729	53.62	552	234	8	94	1737	18	551	0.0	582
ABCDEF_40982	UniRef50_Q96301	0.0	Normal: 1037	706	819	1249	1084	920	BlastAnno: 3418	UniRef50_Q96301	914	79.60	902	173	3	243	2948	1	891	0.0	1506
ABCDEF_47674	UniRef50_Q9LHZ7	0.0	Normal: 274	92	202	599	505	280	BlastAnno: 2526	UniRef50_Q9LHZ7	1170	75.93	777	151	8	2260	2	15	779	0.0	1177
ABCDEF_34097	UniRef50_Q9LE59	0.0	Normal: 626	613	923	1797	1829	692	BlastAnno: 2521	UniRef50_Q9LE59	673	60.92	458	178	1	1687	317	215	672	0.0	630
ABCDEF_34098	UniRef50_Q9LE59	0.0	Normal: 457	464	697	1283	1312	539	BlastAnno: 2199	UniRef50_Q9LE59	673	60.92	458	178	1	1687	317	215	672	0.0	628
ABCDEF_10848	UniRef50_Q9M156	0.0	Normal: 157	2988	163	408	772	1566	BlastAnno: 1810	UniRef50_Q9M156	480	60.09	456	176	4	1700	336	4	454	0.0	553
ABCDEF_41182	UniRef50_Q9LHZ7	0.0	Normal: 150	2169	221	814	1407	495	BlastAnno: 3604	UniRef50_Q9LHZ7	1170	51.03	870	333	15	2822	456	299	1156	0.0	826
ABCDEF_40981	UniRef50_Q96301	0.0	Normal: 156	97	110	160	179	173	BlastAnno: 1225	UniRef50_Q96301	914	86.92	344	45	0	1112	81	394	737	0.0	652
ABCDEF_9506	UniRef50_Q9LHZ7	0.0	Normal: 1302	2128	1494	3356	4644	2206	BlastAnno: 3751	UniRef50_Q9LHZ7	1170	49.38	891	343	17	927	3359	299	1161	0.0	812
ABCDEF_9500	UniRef50_Q9LHZ7	0.0	Normal: 183	188	186	576	498	260	BlastAnno: 1717	UniRef50_Q9LHZ7	1170	47.98	644	234	14	3	1715	418	1033	0.0	571
ABCDEF_100	UniRef50_Q9M8Y0	0.0	Normal: 228	309	224	416	461	141	BlastAnno: 2109	UniRef50_Q9M8Y0	977	77.49	533	120	0	1610	12	50	582	0.0	884
ABCDEF_33659	UniRef50_Q9LHZ7	0.0	Normal: 1282	1948	2248	5826	10642	2083	BlastAnno: 4289	UniRef50_Q9LHZ7	1170	48.70	887	345	15	2955	538	299	1156	0.0	788
ABCDEF_37667	UniRef50_Q9LHZ7	0.0	Normal: 248	3617	251	1237	2078	970	BlastAnno: 3161	UniRef50_Q9LHZ7	1170	49.72	885	335	14	2671	260	301	1156	0.0	810
ABCDEF_47673	UniRef50_Q9LHZ7	0.0	Normal: 876	296	828	1628	1721	1172	BlastAnno: 3878	UniRef50_Q9LHZ7	1170	80.88	1161	186	8	3642	232	15	1163	0.0	1865
ABCDEF_99	UniRef50_Q9M8Y0	0.0	Normal: 272	330	222	398	423	145	BlastAnno: 2094	UniRef50_Q9M8Y0	977	77.36	561	127	0	1683	1	50	610	0.0	929
ABCDEF_20658	UniRef50_Q8VZK9	0.0	Normal: 373	33	193	64	503	141	BlastAnno: 2316	UniRef50_Q8VZK9	729	53.35	508	221	7	47	1564	11	504	0.0	559
ABCDEF_10183	UniRef50_A7XDQ9	0.0	Normal: 422	455	346	577	378	610	BlastAnno: 2362	UniRef50_A7XDQ9	684	50.94	693	307	12	281	2287	1	684	0.0	670
ABCDEF_30745	UniRef50_A7XDQ9	0.0	Normal: 1209	351	1031	1645	1226	635	BlastAnno: 2646	UniRef50_A7XDQ9	684	57.58	554	227	6	546	2189	133	684	0.0	664
ABCDEF_13336	UniRef50_Q7PC69	0.0	Normal: 148	563	194	957	1575	816	BlastAnno: 2584	UniRef50_Q7PC69	745	71.56	559	136	6	744	2351	187	745	0.0	730
ABCDEF_21426	UniRef50_Q8RU27	0.0	Normal: 2658	1979	3417	6157	9464	2599	BlastAnno: 1530	UniRef50_Q8RU27	366	89.44	360	34	2	1358	282	1	357	0.0	667
ABCDEF_71242	UniRef50_Q9ZV71	0.0	Normal: 134	122	179	480	754	119	BlastAnno: 1835	UniRef50_Q9ZV71	409	73.35	409	103	4	231	1439	1	409	0.0	587
ABCDEF_52020	UniRef50_Q6YWK8	0.0	Normal: 1635	1534	1975	4284	10353	2289	BlastAnno: 2394	UniRef50_Q6YWK8	570	58.37	514	185	2	403	1857	57	570	0.0	607
ABCDEF_51269	UniRef50_Q6YWK8	0.0	Normal: 147	85	103	21	33	438	BlastAnno: 1864	UniRef50_Q6YWK8	570	54.28	514	204	2	1646	198	57	570	0.0	586
ABCDEF_30753	UniRef50_Q9ZV71	0.0	Normal: 290	497	417	629	1013	634	BlastAnno: 1502	UniRef50_Q9ZV71	409	72.32	401	107	2	311	1501	1	401	0.0	590
ABCDEF_4233	UniRef50_Q9ZV71	0.0	Normal: 369	412	550	936	860	750	BlastAnno: 2004	UniRef50_Q9ZV71	409	72.13	409	110	2	370	1584	1	409	0.0	613
ABCDEF_17525	UniRef50_Q8RU27	0.0	Normal: 1500	1289	2149	3711	4446	1069	BlastAnno: 1507	UniRef50_Q8RU27	366	91.35	347	30	0	1371	331	3	349	0.0	671
ABCDEF_18005	UniRef50_Q6YWK8	0.0	Normal: 1842	2042	2515	4214	5208	2842	BlastAnno: 2305	UniRef50_Q6YWK8	570	59.14	514	180	2	1948	497	57	570	0.0	642
ABCDEF_41140	UniRef50_Q6YWK8	0.0	Normal: 86	1314	93	753	1787	2076	BlastAnno: 2346	UniRef50_Q6YWK8	570	60.00	510	174	2	431	1870	57	566	0.0	669
ABCDEF_18002	UniRef50_Q9LHZ7	0.0	Normal: 376	127	367	601	1190	520	BlastAnno: 1605	UniRef50_Q9LHZ7	1170	89.75	400	41	0	1603	404	771	1170	0.0	732
ABCDEF_13339	UniRef50_Q7PC69	0.0	Normal: 127	814	183	541	1135	1144	BlastAnno: 2610	UniRef50_Q7PC69	745	70.48	559	141	6	725	2329	187	745	0.0	729
ABCDEF_41141	UniRef50_Q6YWK8	0.0	Normal: 67	1107	84	708	1690	1854	BlastAnno: 2461	UniRef50_Q6YWK8	570	60.00	510	174	2	1916	477	57	566	0.0	668
ABCDEF_44583	UniRef50_Q9LE59	0.0	Normal: 681	561	559	947	1745	789	BlastAnno: 2470	UniRef50_Q9LE59	673	59.21	456	185	1	1749	385	214	669	0.0	621
ABCDEF_3869	UniRef50_Q9LE59	0.0	Normal: 333	393	415	809	907	754	BlastAnno: 2136	UniRef50_Q9LE59	673	59.05	613	227	4	1827	1	81	673	0.0	699
ABCDEF_28947	UniRef50_Q9SK82	0.0	Normal: 0	851	3	0	0	116	BlastAnno: 1567	UniRef50_Q9SK82	489	62.40	484	176	4	1524	85	1	482	0.0	652
ABCDEF_31119	UniRef50_Q9M8Y0	0.0	Normal: 2212	1279	1917	2933	3341	1803	BlastAnno: 3695	UniRef50_Q9M8Y0	977	81.08	925	174	1	392	3163	50	974	0.0	1583
ABCDEF_37311	UniRef50_Q9LE59	0.0	Normal: 175	133	191	549	421	366	BlastAnno: 1612	UniRef50_Q9LE59	673	62.96	486	153	4	1406	3	4	480	0.0	542
ABCDEF_24190	UniRef50_Q8L7F9	0.0	Normal: 397	265	492	583	553	371	BlastAnno: 2564	UniRef50_Q8L7F9	643	63.91	640	218	4	2353	473	1	640	0.0	850
ABCDEF_30548	UniRef50_Q9FH36	0.0	Normal: 17	916	27	151	420	126	BlastAnno: 1963	UniRef50_Q9FH36	535	72.47	534	138	3	102	1676	1	534	0.0	767
ABCDEF_27416	UniRef50_Q7PC69	0.0	Normal: 575	837	875	1344	2015	936	BlastAnno: 2454	UniRef50_Q7PC69	745	66.11	416	115	4	2337	1168	126	541	0.0	518
ABCDEF_48373	UniRef50_A7XDQ9	0.0	Normal: 1364	773	1336	1451	1791	1202	BlastAnno: 3042	UniRef50_A7XDQ9	684	63.68	691	234	6	2405	360	1	683	0.0	915
ABCDEF_3868	UniRef50_Q9LE59	0.0	Normal: 224	274	301	555	686	524	BlastAnno: 1670	UniRef50_Q9LE59	673	65.31	516	176	1	1556	9	161	673	0.0	696
ABCDEF_31288	UniRef50_Q940V3	0.0	Normal: 247	309	183	101	124	655	BlastAnno: 1658	UniRef50_Q940V3	470	56.01	466	197	7	1614	232	8	470	0.0	537
ABCDEF_52574	UniRef50_Q09332	0.0	Normal: 885	474	1068	1835	1853	816	BlastAnno: 3720	UniRef50_Q09332	1548	47.89	687	310	14	2551	533	859	1511	0.0	598
ABCDEF_79752	UniRef50_Q9ZV71	0.0	Normal: 158	183	245	400	379	352	BlastAnno: 1266	UniRef50_Q9ZV71	409	71.39	360	99	2	31	1098	1	360	0.0	526
ABCDEF_9509	UniRef50_Q9LHZ7	0.0	Normal: 1296	2171	1498	3410	4683	2232	BlastAnno: 3897	UniRef50_Q9LHZ7	1170	49.38	891	343	17	1073	3505	299	1161	0.0	812
ABCDEF_48200	UniRef50_Q6YWK8	0.0	Normal: 1768	576	3156	4431	6634	491	BlastAnno: 2274	UniRef50_Q6YWK8	570	57.98	514	185	4	291	1739	57	570	0.0	642
ABCDEF_24155	UniRef50_Q9ZVI7	0.0	Normal: 503	247	607	1676	1833	578	BlastAnno: 2708	UniRef50_Q9ZVI7	619	52.33	621	264	11	306	2123	15	618	0.0	601

ABCDEF_21246	UniRef50_Q9SKT6	0.0	Normal: 92	107	188	380	834	84	BlastAnno: 1791	UniRef50_Q9SKT6	536	56.61	484	204	5	123	1562	52	533	0.0	568
ABCDEF_32317	UniRef50_Q8RU27	0.0	Normal: 12	9	3	118	800	62	BlastAnno: 1076	UniRef50_Q8RU27	366	87.02	285	37	0	1075	221	64	348	0.0	535
ABCDEF_9502	UniRef50_Q9LHZ7	0.0	Normal: 610	486	568	1331	1403	591	BlastAnno: 1961	UniRef50_Q9LHZ7	1170	48.64	625	249	13	3	1742	564	1161	0.0	570
ABCDEF_23298	UniRef50_Q9FH36	0.0	Normal: 128	527	130	223	314	864	BlastAnno: 2832	UniRef50_Q9FH36	535	83.16	196	33	0	696	1283	340	535	0.0	357
ABCDEF_40595	UniRef50_O80898	0.0	Normal: 229	135	103	25	307	325	BlastAnno: 2413	UniRef50_O80898	757	44.68	761	378	13	95	2299	10	753	0.0	587
ABCDEF_63947	UniRef50_Q9SK82	0.0	Normal: 0	267	0	0	71	0	BlastAnno: 1446	UniRef50_Q9SK82	489	58.33	468	189	4	60	1445	1	468	0.0	592
ABCDEF_67847	UniRef50_Q9M8Y0	0.0	Normal: 794	476	871	1081	1401	868	BlastAnno: 1591	UniRef50_Q9M8Y0	977	84.86	370	56	0	1589	480	605	974	0.0	685
ABCDEF_65666	UniRef50_Q09332	0.0	Normal: 800	441	946	1606	1637	735	BlastAnno: 3518	UniRef50_Q09332	1548	47.54	671	305	13	1162	3087	859	1511	0.0	586
ABCDEF_38641	UniRef50_Q9FWA4	0.0	Normal: 674	967	1162	2603	4404	2475	BlastAnno: 1599	UniRef50_Q9FWA4	561	81.35	386	72	0	1	1158	176	561	0.0	641
ABCDEF_17914	UniRef50_Q9SKT6	0.0	Normal: 784	567	886	1447	1439	1424	BlastAnno: 2049	UniRef50_Q9SKT6	536	72.95	536	140	2	204	1796	1	536	0.0	785
ABCDEF_18286	UniRef50_Q6NQB7	0.0	Normal: 1131	1108	1225	1872	1722	3329	BlastAnno: 1752	UniRef50_Q6NQB7	393	73.80	397	81	6	1527	367	10	393	0.0	607
ABCDEF_119	UniRef50_Q8RU27	0.0	Normal: 1613	1155	2249	3558	7663	1775	BlastAnno: 1532	UniRef50_Q8RU27	366	88.33	360	38	2	1306	230	1	357	0.0	663
ABCDEF_47603	UniRef50_Q9M9Y5	0.0	Normal: 2609	8779	1664	1103	2085	22991	BlastAnno: 2830	UniRef50_Q9M9Y5	589	54.04	594	267	3	2475	694	1	588	0.0	654
ABCDEF_24192	UniRef50_Q8L7F9	0.0	Normal: 391	244	465	553	497	345	BlastAnno: 2477	UniRef50_Q8L7F9	643	58.99	434	165	4	212	1474	1	434	0.0	513
ABCDEF_17482	UniRef50_Q9SKT6	0.0	Normal: 615	379	816	1068	1470	852	BlastAnno: 2083	UniRef50_Q9SKT6	536	71.11	540	147	4	1915	311	1	536	0.0	786
ABCDEF_85909	UniRef50_Q8VZK9	0.0	Normal: 28	45	9	10	44	127	BlastAnno: 1473	UniRef50_Q8VZK9	729	55.37	475	198	6	1419	1	11	473	0.0	536
ABCDEF_9503	UniRef50_Q9LHZ7	0.0	Normal: 487	544	524	1242	1320	833	BlastAnno: 2087	UniRef50_Q9LHZ7	1170	50.35	574	236	8	57	1712	615	1161	0.0	567
ABCDEF_40594	UniRef50_O80898	0.0	Normal: 256	127	102	41	331	326	BlastAnno: 2417	UniRef50_O80898	757	45.76	719	348	12	213	2291	51	753	0.0	585
ABCDEF_17141	UniRef50_Q9LHZ7	0.0	Normal: 74	1734	69	380	701	333	BlastAnno: 3337	UniRef50_Q9LHZ7	1170	50.92	868	335	14	2539	179	299	1156	0.0	816
ABCDEF_33658	UniRef50_Q9LHZ7	0.0	Normal: 384	308	395	805	1003	602	BlastAnno: 2961	UniRef50_Q9LHZ7	1170	48.76	888	343	14	204	2621	299	1156	0.0	792
ABCDEF_20579	UniRef50_Q7PC69	0.0	Normal: 1007	795	1238	2311	2309	1345	BlastAnno: 3113	UniRef50_Q7PC69	745	59.97	767	229	14	2667	535	1	745	0.0	768
ABCDEF_31118	UniRef50_Q9M8Y0	0.0	Normal: 2135	1267	1900	3004	3329	1757	BlastAnno: 3787	UniRef50_Q9M8Y0	977	81.10	709	133	1	1132	3255	266	974	0.0	1217
ABCDEF_9499	UniRef50_Q9LHZ7	0.0	Normal: 205	207	215	632	538	275	BlastAnno: 1794	UniRef50_Q9LHZ7	1170	48.88	667	240	14	11	1792	395	1033	0.0	605
ABCDEF_48199	UniRef50_Q6YWK8	0.0	Normal: 1506	467	2429	3094	4233	439	BlastAnno: 1997	UniRef50_Q6YWK8	570	63.10	439	146	2	291	1559	57	495	0.0	610
ABCDEF_40983	UniRef50_Q96301	0.0	Normal: 406	261	343	392	415	433	BlastAnno: 2265	UniRef50_Q96301	914	79.17	504	99	1	114	1625	394	891	0.0	854
ABCDEF_17140	UniRef50_Q9LHZ7	0.0	Normal: 45	1049	49	216	386	208	BlastAnno: 2293	UniRef50_Q9LHZ7	1170	52.34	770	281	13	2138	57	397	1156	0.0	739
ABCDEF_18352	UniRef50_Q9FWA4	0.0	Normal: 2427	2688	2969	5519	6547	4855	BlastAnno: 2224	UniRef50_Q9FWA4	561	69.31	505	148	3	1887	382	61	561	0.0	683
ABCDEF_27767	UniRef50_Q9LHZ7	0.0	Normal: 2576	5141	2841	10249	14583	5031	BlastAnno: 3981	UniRef50_Q9LHZ7	1170	49.66	888	337	16	2927	504	299	1156	0.0	818
ABCDEF_33661	UniRef50_Q9LHZ7	0.0	Normal: 414	340	507	879	790	783	BlastAnno: 2185	UniRef50_Q9LHZ7	1170	48.66	748	277	14	2184	169	440	1156	0.0	662
ABCDEF_14685	UniRef50_A7XDQ9	0.0	Normal: 233	308	134	573	292	334	BlastAnno: 1947	UniRef50_A7XDQ9	684	56.84	468	186	6	569	1945	1	461	0.0	545
ABCDEF_20660	UniRef50_Q8VZK9	0.0	Normal: 22	48	13	15	52	144	BlastAnno: 1473	UniRef50_Q8VZK9	729	55.16	475	199	6	55	1473	11	473	0.0	537
ABCDEF_21315	UniRef50_Q9LHZ7	0.0	Normal: 3412	252	1450	0	79	1228	BlastAnno: 3765	UniRef50_Q9LHZ7	1170	63.78	1096	340	15	308	3508	70	1137	0.0	1333
ABCDEF_533	UniRef50_Q9C8W3	0.0	Normal: 230	204	269	505	411	301	BlastAnno: 1688	UniRef50_Q9C8W3	401	70.33	364	94	2	393	1475	47	399	0.0	555
ABCDEF_34099	UniRef50_Q9LE59	0.0	Normal: 478	491	735	1356	1350	548	BlastAnno: 2158	UniRef50_Q9LE59	673	60.92	458	178	1	1610	240	215	672	0.0	630
ABCDEF_47984	UniRef50_Q7PC69	0.0	Normal: 663	457	734	1362	2147	696	BlastAnno: 2838	UniRef50_Q7PC69	745	59.27	712	226	9	373	2316	34	745	0.0	702
ABCDEF_51451	UniRef50_Q9LE59	0.0	Normal: 1883	1782	2202	4337	4350	2667	BlastAnno: 2874	UniRef50_Q9LE59	673	74.16	685	165	6	2559	505	1	673	0.0	982
ABCDEF_13370	UniRef50_Q8VZK9	0.0	Normal: 290	87	317	377	105	258	BlastAnno: 2033	UniRef50_Q8VZK9	729	51.65	635	278	11	2031	154	112	726	0.0	612
ABCDEF_48239	UniRef50_Q9LHZ7	0.0	Normal: 511	163	422	1154	1316	510	BlastAnno: 2714	UniRef50_Q9LHZ7	1170	76.20	769	156	9	478	2712	41	806	0.0	1201
ABCDEF_13338	UniRef50_Q7PC69	0.0	Normal: 335	468	417	2639	4443	616	BlastAnno: 2525	UniRef50_Q7PC69	745	62.45	727	209	12	298	2292	21	745	0.0	781
ABCDEF_24193	UniRef50_Q8L7F9	0.0	Normal: 113	72	132	203	180	119	BlastAnno: 1679	UniRef50_Q8L7F9	643	60.54	479	176	4	212	1609	1	479	0.0	592
ABCDEF_48514	UniRef50_Q9ZV71	0.0	Normal: 540	580	601	771	1033	926	BlastAnno: 1920	UniRef50_Q9ZV71	409	78.14	398	85	2	1618	431	12	409	0.0	637
2.4.1.101(alpha-1,3-mannosyl-glycoprotein 2-beta-N-acetylglucosaminyltransferase;)																					
ABCDEF_26986	UniRef50_Q9XGM8	0.0	Normal: 106	55	92	159	243	127	BlastAnno: 1191	UniRef50_Q9XGM8	444	77.78	333	74	0	1190	192	112	444	0.0	567
ABCDEF_26985	UniRef50_Q9XGM8	0.0	Normal: 69	42	63	148	180	88	BlastAnno: 1148	UniRef50_Q9XGM8	444	77.78	333	74	0	1147	149	112	444	0.0	567
ABCDEF_26982	UniRef50_Q9XGM8	0.0	Normal: 188	115	153	310	426	221	BlastAnno: 1924	UniRef50_Q9XGM8	444	73.64	440	116	0	1776	457	5	444	0.0	705
ABCDEF_26983	UniRef50_Q9XGM8	0.0	Normal: 234	126	196	335	497	264	BlastAnno: 1964	UniRef50_Q9XGM8	444	73.64	440	116	0	1816	497	5	444	0.0	705
2.4.1.119(dolichyl-diphosphooligosaccharide---protein glycotransferase;)																					
ABCDEF_48073	UniRef50_B6KJL1	0.0	Normal: 2214	1214	2170	3118	4131	2301	BlastAnno: 2792	UniRef50_B6KJL1	753	45.26	738	355	7	2602	398	54	745	0.0	588
ABCDEF_28256	UniRef50_P46977	0.0	Normal: 2426	1044	2529	4068	4468	1518	BlastAnno: 2888	UniRef50_P46977	705	60.37	709	262	5	2612	498	7	700	0.0	841
2.4.1.130(dolichyl-phosphate-mannose---glycolipid alpha-mannosyltransferase;)																					
No Correspond Unigene																					
2.4.1.131(glycolipid 2-alpha-mannosyltransferase;)																					
ABCDEF_5906	UniRef50_Q2TAA5	5e-119	Normal: 45	54	41	77	101	81	BlastAnno: 1169	UniRef50_Q2TAA5	492	48.40	376	188	4	1116	7	57	432	5e-119	363
2.4.1.132(glycolipid 3-alpha-mannosyltransferase;)																					
ABCDEF_17580	UniRef50_O94738	1e-85	Normal: 56	38	59	74	107	90	BlastAnno: 996	UniRef50_O94738	455	49.82	285	123	6	817	2	3	280	1e-85	273
2.4.1.141(N-acetylglucosaminyldiphosphodolichol N-acetylglucosaminyltransferase;)																					
ABCDEF_13935	UniRef50_Q9NP73	3e-35	Normal: 75	42	79	87	117	135	BlastAnno: 826	UniRef50_Q9NP73	165	42.07	164	93	1	736	245	2	163	3e-35	132
2.4.1.142(chitobiosyldiphosphodolichol beta-mannosyltransferase;)																					
ABCDEF_21126	UniRef50_Q9BT22	1e-111	Normal: 285	250	242	469	609	458	BlastAnno: 1877	UniRef50_Q9BT22	464	43.22	472	225	11	212	1612	28	461	1e-111	352
2.4.1.143(alpha-1,6-mannosyl-glycoprotein 2-beta-N-acetylglucosaminyltransferase;)																					
ABCDEF_44902	UniRef50_Q9FT88	0.0	Normal: 685	658	903	1695	1719	1028	BlastAnno: 2042	UniRef50_Q9FT88	430	74.36	351	89	1	1373	324	71	421	0.0	576
2.4.1.145(alpha-1,3-mannosyl-glycoprotein 4-beta-N-acetylglucosaminyltransferase;)																					
No Correspond Unigene																					
2.4.1.214(glycoprotein 3-alpha-L-fucosyltransferase;)																					
ABCDEF_10223	UniRef50_Q9LJK1	0.0	Normal: 265	147	225	306	315	236	BlastAnno: 1864	UniRef50_Q9LJK1	501	61.19	505	164	4	260	1744	19	501	0.0	615
ABCDEF_10222	UniRef50_Q9LJK1	0.0	Normal: 126	69	123	156	189	118	BlastAnno: 1275	UniRef50_Q9LJK1	501	69.09	385	118	1	36	1190	118	501	0.0	551
2.4.1.232(initiation-specific alpha-1,6-mannosyltransferase;)																					
No Correspond Unigene																					
2.4.1.244(N-acetyl-beta-glucosaminyl-glycoprotein 4-beta-N-acetylgalactosaminyltransferase;)																					
No Correspond Unigene																					
2.4.1.38(beta-N-acetylglucosaminylglycopeptide beta-1,4-galactosyltransferase;)																					
No Correspond Unigene																					

2.4.1.68(glycoprotein 6-alpha-L-fucosyltransferase;)																					
No Correspond Unigene																					
2.4.2.38(glycoprotein 2-beta-D-xylosyltransferase;)																					
ABCDEF_7085	UniRef50_Q599J2	0.0	Normal: 461	292	354	511	555	729	BlastAnno: 2027	UniRef50_Q599J2	468	60.61	528	138	8	84	1637	1	468	0.0	620
2.4.99.6(N-acetyllactosaminide alpha-2,3-sialyltransferase;)																					
No Correspond Unigene																					
2.7.8.-(Transferases;)																					
ABCDEF_40506	UniRef50_C0QDU7	8e-33	Normal: 129	124	156	162	222	178	BlastAnno: 1387	UniRef50_C0QDU7	251	32.58	267	143	8	1342	542	10	239	8e-33	131
2.8.2.-(Transferases;)																					
ABCDEF_60620	UniRef50_P52839	2e-85	Normal: 240	393	445	400	902	151	BlastAnno: 1191	UniRef50_P52839	326	45.95	333	168	6	58	1050	2	324	2e-85	271
3.2.1.113(mannosyl-oligosaccharide 1,2-alpha-mannosidase;)																					
ABCDEF_25164	UniRef50_Q93Y37	0.0	Normal: 767	679	1074	2657	3100	1098	BlastAnno: 2667	UniRef50_Q93Y37	624	68.20	632	184	6	433	2310	1	621	0.0	822
3.2.1.114(mannosyl-oligosaccharide 1,3-1,6-alpha-mannosidase;)																					
ABCDEF_20466	UniRef50_Q16706	0.0	Normal: 1468	901	1501	2183	2291	1145	BlastAnno: 3954	UniRef50_Q16706	1144	38.40	1060	596	22	424	3564	128	1143	0.0	717
3.2.1.52(beta-N-acetylhexosaminidase;)																					
ABCDEF_16822	UniRef50_Q54K56	3e-109	Normal: 543	493	486	692	917	660	BlastAnno: 1760	UniRef50_Q54K56	564	36.42	519	294	10	1587	94	59	562	3e-109	347
>00514 Other types of O-glycan biosynthesis																					
2.4.1.-(Transferases;)																					
ABCDEF_18004	UniRef50_Q6YWK8	0.0	Normal: 1562	1736	2009	3452	4438	2400	BlastAnno: 2298	UniRef50_Q6YWK8	570	65.27	429	136	1	1941	694	57	485	0.0	608
ABCDEF_52379	UniRef50_Q9LHZ7	0.0	Normal: 4015	4921	4546	10520	13430	4731	BlastAnno: 3479	UniRef50_Q9LHZ7	1170	48.04	893	342	16	825	3242	299	1156	0.0	787
ABCDEF_48593	UniRef50_Q9SK82	0.0	Normal: 12	420	19	343	763	253	BlastAnno: 1834	UniRef50_Q9SK82	489	60.08	481	186	4	1714	278	10	486	0.0	633
ABCDEF_13375	UniRef50_Q8VZK9	0.0	Normal: 108	123	208	526	34	294	BlastAnno: 1738	UniRef50_Q8VZK9	729	53.62	552	234	8	94	1737	18	551	0.0	582
ABCDEF_40982	UniRef50_Q96301	0.0	Normal: 1037	706	819	1249	1084	920	BlastAnno: 3418	UniRef50_Q96301	914	79.60	902	173	3	243	2948	1	891	0.0	1506
ABCDEF_47674	UniRef50_Q9LHZ7	0.0	Normal: 274	92	202	599	505	280	BlastAnno: 2526	UniRef50_Q9LHZ7	1170	75.93	777	151	8	2260	2	15	779	0.0	1177
ABCDEF_34097	UniRef50_Q9LE59	0.0	Normal: 626	613	923	1797	1829	692	BlastAnno: 2521	UniRef50_Q9LE59	673	60.92	458	178	1	1687	317	215	672	0.0	630
ABCDEF_34098	UniRef50_Q9LE59	0.0	Normal: 457	464	697	1283	1312	539	BlastAnno: 2199	UniRef50_Q9LE59	673	60.92	458	178	1	1687	317	215	672	0.0	628
ABCDEF_10848	UniRef50_Q9M156	0.0	Normal: 157	2988	163	408	772	1566	BlastAnno: 1810	UniRef50_Q9M156	480	60.09	456	176	4	1700	336	4	454	0.0	553
ABCDEF_41182	UniRef50_Q9LHZ7	0.0	Normal: 150	2169	221	814	1407	495	BlastAnno: 3604	UniRef50_Q9LHZ7	1170	51.03	870	333	15	2822	456	299	1156	0.0	826
ABCDEF_40981	UniRef50_Q96301	0.0	Normal: 156	97	110	160	179	173	BlastAnno: 1225	UniRef50_Q96301	914	86.92	344	45	0	1112	81	394	737	0.0	652
ABCDEF_9506	UniRef50_Q9LHZ7	0.0	Normal: 1302	2128	1494	3356	4644	2206	BlastAnno: 3751	UniRef50_Q9LHZ7	1170	49.38	891	343	17	927	3359	299	1161	0.0	812
ABCDEF_9500	UniRef50_Q9LHZ7	0.0	Normal: 183	188	186	576	498	260	BlastAnno: 1717	UniRef50_Q9LHZ7	1170	47.98	644	234	14	3	1715	418	1033	0.0	571
ABCDEF_100	UniRef50_Q9M8Y0	0.0	Normal: 228	309	224	416	461	141	BlastAnno: 2109	UniRef50_Q9M8Y0	977	77.49	533	120	0	1610	12	50	582	0.0	884
ABCDEF_33659	UniRef50_Q9LHZ7	0.0	Normal: 1282	1948	2248	5826	10642	2083	BlastAnno: 4289	UniRef50_Q9LHZ7	1170	48.70	887	345	15	2955	538	299	1156	0.0	788
ABCDEF_37667	UniRef50_Q9LHZ7	0.0	Normal: 248	3617	251	1237	2078	970	BlastAnno: 3161	UniRef50_Q9LHZ7	1170	49.72	885	335	14	2671	260	301	1156	0.0	810
ABCDEF_47673	UniRef50_Q9LHZ7	0.0	Normal: 876	296	828	1628	1721	1172	BlastAnno: 3878	UniRef50_Q9LHZ7	1170	80.88	1161	186	8	3642	232	15	1163	0.0	1865
ABCDEF_99	UniRef50_Q9M8Y0	0.0	Normal: 272	330	222	398	423	145	BlastAnno: 2094	UniRef50_Q9M8Y0	977	77.36	561	127	0	1683	1	50	610	0.0	929
ABCDEF_20658	UniRef50_Q8VZK9	0.0	Normal: 373	33	193	64	503	141	BlastAnno: 2316	UniRef50_Q8VZK9	729	53.35	508	221	7	47	1564	11	504	0.0	559
ABCDEF_10183	UniRef50_A7XDQ9	0.0	Normal: 422	455	346	577	378	610	BlastAnno: 2362	UniRef50_A7XDQ9	684	50.94	693	307	12	281	2287	1	684	0.0	670
ABCDEF_30745	UniRef50_A7XDQ9	0.0	Normal: 1209	351	1031	1645	1226	635	BlastAnno: 2646	UniRef50_A7XDQ9	684	57.58	554	227	6	546	2189	133	684	0.0	664
ABCDEF_13336	UniRef50_Q7PC69	0.0	Normal: 148	563	194	957	1575	816	BlastAnno: 2584	UniRef50_Q7PC69	745	71.56	559	136	6	744	2351	187	745	0.0	730
ABCDEF_21426	UniRef50_Q8RU27	0.0	Normal: 2658	1979	3417	6157	9464	2599	BlastAnno: 1530	UniRef50_Q8RU27	366	89.44	360	34	2	1358	282	1	357	0.0	667
ABCDEF_71242	UniRef50_Q9ZV71	0.0	Normal: 134	122	179	480	754	119	BlastAnno: 1835	UniRef50_Q9ZV71	409	73.35	409	103	4	231	1439	1	409	0.0	587
ABCDEF_52020	UniRef50_Q6YWK8	0.0	Normal: 1635	1534	1975	4284	10353	2289	BlastAnno: 2394	UniRef50_Q6YWK8	570	58.37	514	185	2	403	1857	57	570	0.0	607
ABCDEF_51269	UniRef50_Q6YWK8	0.0	Normal: 147	85	103	21	33	438	BlastAnno: 1864	UniRef50_Q6YWK8	570	54.28	514	204	2	1646	198	57	570	0.0	586
ABCDEF_30753	UniRef50_Q9ZV71	0.0	Normal: 290	497	417	629	1013	634	BlastAnno: 1502	UniRef50_Q9ZV71	409	72.32	401	107	2	311	1501	1	401	0.0	590
ABCDEF_4233	UniRef50_Q9ZV71	0.0	Normal: 369	412	550	936	860	750	BlastAnno: 2004	UniRef50_Q9ZV71	409	72.13	409	110	2	370	1584	1	409	0.0	613
ABCDEF_17525	UniRef50_Q8RU27	0.0	Normal: 1500	1289	2149	3711	4446	1069	BlastAnno: 1507	UniRef50_Q8RU27	366	91.35	347	30	0	1371	331	3	349	0.0	671
ABCDEF_18005	UniRef50_Q6YWK8	0.0	Normal: 1842	2042	2515	4214	5208	2842	BlastAnno: 2305	UniRef50_Q6YWK8	570	59.14	514	180	2	1948	497	57	570	0.0	642
ABCDEF_41140	UniRef50_Q6YWK8	0.0	Normal: 86	1314	93	753	1787	2076	BlastAnno: 2346	UniRef50_Q6YWK8	570	60.00	510	174	2	431	1870	57	566	0.0	669
ABCDEF_18002	UniRef50_Q9LHZ7	0.0	Normal: 376	127	367	601	1190	520	BlastAnno: 1605	UniRef50_Q9LHZ7	1170	89.75	400	41	0	1603	404	771	1170	0.0	732
ABCDEF_13339	UniRef50_Q7PC69	0.0	Normal: 127	814	183	541	1135	1144	BlastAnno: 2610	UniRef50_Q7PC69	745	70.48	559	141	6	725	2329	187	745	0.0	729
ABCDEF_41141	UniRef50_Q6YWK8	0.0	Normal: 67	1107	84	708	1690	1854	BlastAnno: 2461	UniRef50_Q6YWK8	570	60.00	510	174	2	1916	477	57	566	0.0	668
ABCDEF_44583	UniRef50_Q9LE59	0.0	Normal: 681	561	559	947	1745	789	BlastAnno: 2470	UniRef50_Q9LE59	673	59.21	456	185	1	1749	385	214	669	0.0	621
ABCDEF_3869	UniRef50_Q9LE59	0.0	Normal: 333	393	415	809	907	754	BlastAnno: 2136	UniRef50_Q9LE59	673	59.05	613	227	4	1827	1	81	673	0.0	699
ABCDEF_28947	UniRef50_Q9SK82	0.0	Normal: 0	851	3	0	0	116	BlastAnno: 1567	UniRef50_Q9SK82	489	62.40	484	176	4	1524	85	1	482	0.0	652
ABCDEF_31119	UniRef50_Q9M8Y0	0.0	Normal: 2212	1279	1917	2933	3341	1803	BlastAnno: 3695	UniRef50_Q9M8Y0	977	81.08	925	174	1	392	3163	50	974	0.0	1583
ABCDEF_37311	UniRef50_Q9LE59	0.0	Normal: 175	133	191	549	421	366	BlastAnno: 1612	UniRef50_Q9LE59	673	62.96	486	153	4	1406	3	4	480	0.0	542
ABCDEF_24190	UniRef50_Q8L7F9	0.0	Normal: 397	265	492	583	553	371	BlastAnno: 2564	UniRef50_Q8L7F9	643	63.91	640	218	4	2353	473	1	640	0.0	850
ABCDEF_30548	UniRef50_Q9FH36	0.0	Normal: 17	916	27	151	420	126	BlastAnno: 1963	UniRef50_Q9FH36	535	72.47	534	138	3	102	1676	1	534	0.0	767
ABCDEF_27416	UniRef50_Q7PC69	0.0	Normal: 575	837	875	1344	2015	936	BlastAnno: 2454	UniRef50_Q7PC69	745	66.11	416	115	4	2337	1168	126	541	0.0	518
ABCDEF_48373	UniRef50_A7XDQ9	0.0	Normal: 1364	773	1336	1451	1791	1202	BlastAnno: 3042	UniRef50_A7XDQ9	684	63.68	691	234	6	2405	360	1	683	0.0	915
ABCDEF_3868	UniRef50_Q9LE59	0.0	Normal: 224	274	301	555	686	524	BlastAnno: 1670	UniRef50_Q9LE59	673	65.31	516	176	1	1556	9	161	673	0.0	696
ABCDEF_31288	UniRef50_Q940V3	0.0	Normal: 247	309	183	101	124	655	BlastAnno: 1658	UniRef50_Q940V3	470	56.01	466	197	7	1614	232	8	470	0.0	537
ABCDEF_52574	UniRef50_Q09332	0.0	Normal: 885	474	1068	1835	1853	816	BlastAnno: 3720	UniRef50_Q09332	1548	47.89	687	310	14	2551	533	859	1511	0.0	598
ABCDEF_79752	UniRef50_Q9ZV71	0.0	Normal: 158	183	245	400	379	352	BlastAnno: 1266	UniRef50_Q9ZV71	409	71.39	360	99	2	31	1098	1	360	0.0	526
ABCDEF_9509	UniRef50_Q9LHZ7	0.0	Normal: 1296	2171	1498	3410	4683	2232	BlastAnno: 3897	UniRef50_Q9LHZ7	1170	49.38	891	343	17	1073	3505	299	1161	0.0	812
ABCDEF_48200	UniRef50_Q6YWK8	0.0	Normal: 1768	576	3156	4431	6634	491	BlastAnno: 2274	UniRef50_Q6YWK8	570	57.98	514	185	4	291	1739	57	570	0.0	642
ABCDEF_24155	UniRef50_Q9ZVI7	0.0	Normal: 503	247	607	1676	1833	578	BlastAnno: 2708	UniRef50_Q9ZVI7	619	52.33	621	264	11	306	2123	15	618	0.0	601
ABCDEF_21246	UniRef50_Q9SKT6	0.0	Normal: 92	107	188	380	834	84	BlastAnno: 1791	UniRef50_Q9SKT6	536	56.61	484	204	5	123	1562	52	533	0.0	568
ABCDEF_32317	UniRef50_Q8RU27	0.0	Normal: 12	9	3	118	800	62	BlastAnno: 1076	UniRef50_Q8RU27	366	87.02	285	37	0	1075	221	64	348	0.0	535
ABCDEF_9502	UniRef50_Q9LHZ7	0.0	Normal: 610	486	568	1331	1403	591	BlastAnno: 1961	UniRef50_Q9LHZ7	1170	48.64	625	249	13	3	1742	564	1161	0.0	570
ABCDEF_23298	UniRef50_Q9FH36	0.0	Normal: 128	527	130	223	314	864	BlastAnno: 2832	UniRef50_Q9FH36	535	83.16	196	33	0	696	1283	340	535	0.0	357

ABCDEF_40595	UniRef50_O80898	0.0	Normal: 229	135	103	25	307	325	BlastAnno: 2413	UniRef50_O80898	757	44.68	761	378	13	95	2299	10	753	0.0	587
ABCDEF_63947	UniRef50_Q9SK82	0.0	Normal: 0	267	0	0	71	0	BlastAnno: 1446	UniRef50_Q9SK82	489	58.33	468	189	4	60	1445	1	468	0.0	592
ABCDEF_67847	UniRef50_Q9M8Y0	0.0	Normal: 794	476	871	1081	1401	868	BlastAnno: 1591	UniRef50_Q9M8Y0	977	84.86	370	56	0	1589	480	605	974	0.0	685
ABCDEF_65666	UniRef50_Q09332	0.0	Normal: 800	441	946	1606	1637	735	BlastAnno: 3518	UniRef50_Q09332	1548	47.54	671	305	13	1162	3087	859	1511	0.0	586
ABCDEF_38641	UniRef50_Q9FWA4	0.0	Normal: 674	967	1162	2603	4404	2475	BlastAnno: 1599	UniRef50_Q9FWA4	561	81.35	386	72	0	1	1158	176	561	0.0	641
ABCDEF_17914	UniRef50_Q9SKT6	0.0	Normal: 784	567	886	1447	1439	1424	BlastAnno: 2049	UniRef50_Q9SKT6	536	72.95	536	140	2	204	1796	1	536	0.0	785
ABCDEF_18286	UniRef50_Q6NQB7	0.0	Normal: 1131	1108	1225	1872	1722	3329	BlastAnno: 1752	UniRef50_Q6NQB7	393	73.80	397	81	6	1527	367	10	393	0.0	607
ABCDEF_119	UniRef50_Q8RU27	0.0	Normal: 1613	1155	2249	3558	7663	1775	BlastAnno: 1532	UniRef50_Q8RU27	366	88.33	360	38	2	1306	230	1	357	0.0	663
ABCDEF_47603	UniRef50_Q9M9Y5	0.0	Normal: 2609	8779	1664	1103	2085	22991	BlastAnno: 2830	UniRef50_Q9M9Y5	589	54.04	594	267	3	2475	694	1	588	0.0	654
ABCDEF_24192	UniRef50_Q8L7F9	0.0	Normal: 391	244	465	553	497	345	BlastAnno: 2477	UniRef50_Q8L7F9	643	58.99	434	165	4	212	1474	1	434	0.0	513
ABCDEF_17482	UniRef50_Q9SKT6	0.0	Normal: 615	379	816	1068	1470	852	BlastAnno: 2083	UniRef50_Q9SKT6	536	71.11	540	147	4	1915	311	1	536	0.0	786
ABCDEF_85909	UniRef50_Q8VZK9	0.0	Normal: 28	45	9	10	44	127	BlastAnno: 1473	UniRef50_Q8VZK9	729	55.37	475	198	6	1419	1	11	473	0.0	536
ABCDEF_9503	UniRef50_Q9LHZ7	0.0	Normal: 487	544	524	1242	1320	833	BlastAnno: 2087	UniRef50_Q9LHZ7	1170	50.35	574	236	8	57	1712	615	1161	0.0	567
ABCDEF_40594	UniRef50_O80898	0.0	Normal: 256	127	102	41	331	326	BlastAnno: 2417	UniRef50_O80898	757	45.76	719	348	12	213	2291	51	753	0.0	585
ABCDEF_17141	UniRef50_Q9LHZ7	0.0	Normal: 74	1734	69	380	701	333	BlastAnno: 3337	UniRef50_Q9LHZ7	1170	50.92	868	335	14	2539	179	299	1156	0.0	816
ABCDEF_33658	UniRef50_Q9LHZ7	0.0	Normal: 384	308	395	805	1003	602	BlastAnno: 2961	UniRef50_Q9LHZ7	1170	48.76	888	343	14	204	2621	299	1156	0.0	792
ABCDEF_20579	UniRef50_Q7PC69	0.0	Normal: 1007	795	1238	2311	2309	1345	BlastAnno: 3113	UniRef50_Q7PC69	745	59.97	767	229	14	2667	535	1	745	0.0	768
ABCDEF_31118	UniRef50_Q9M8Y0	0.0	Normal: 2135	1267	1900	3004	3329	1757	BlastAnno: 3787	UniRef50_Q9M8Y0	977	81.10	709	133	1	1132	3255	266	974	0.0	1217
ABCDEF_9499	UniRef50_Q9LHZ7	0.0	Normal: 205	207	215	632	538	275	BlastAnno: 1794	UniRef50_Q9LHZ7	1170	48.88	667	240	14	11	1792	395	1033	0.0	605
ABCDEF_48199	UniRef50_Q6YWK8	0.0	Normal: 1506	467	2429	3094	4233	439	BlastAnno: 1997	UniRef50_Q6YWK8	570	63.10	439	146	2	291	1559	57	495	0.0	610
ABCDEF_40983	UniRef50_Q96301	0.0	Normal: 406	261	343	392	415	433	BlastAnno: 2265	UniRef50_Q96301	914	79.17	504	99	1	114	1625	394	891	0.0	854
ABCDEF_17140	UniRef50_Q9LHZ7	0.0	Normal: 45	1049	49	216	386	208	BlastAnno: 2293	UniRef50_Q9LHZ7	1170	52.34	770	281	13	2138	57	397	1156	0.0	739
ABCDEF_18352	UniRef50_Q9FWA4	0.0	Normal: 2427	2688	2969	5519	6547	4855	BlastAnno: 2224	UniRef50_Q9FWA4	561	69.31	505	148	3	1887	382	61	561	0.0	683
ABCDEF_27767	UniRef50_Q9LHZ7	0.0	Normal: 2576	5141	2841	10249	14583	5031	BlastAnno: 3981	UniRef50_Q9LHZ7	1170	49.66	888	337	16	2927	504	299	1156	0.0	818
ABCDEF_33661	UniRef50_Q9LHZ7	0.0	Normal: 414	340	507	879	790	783	BlastAnno: 2185	UniRef50_Q9LHZ7	1170	48.66	748	277	14	2184	169	440	1156	0.0	662
ABCDEF_14685	UniRef50_A7XDQ9	0.0	Normal: 233	308	134	573	292	334	BlastAnno: 1947	UniRef50_A7XDQ9	684	56.84	468	186	6	569	1945	1	461	0.0	545
ABCDEF_20660	UniRef50_Q8VZK9	0.0	Normal: 22	48	13	15	52	144	BlastAnno: 1473	UniRef50_Q8VZK9	729	55.16	475	199	6	55	1473	11	473	0.0	537
ABCDEF_21315	UniRef50_Q9LHZ7	0.0	Normal: 3412	252	1450	0	79	1228	BlastAnno: 3765	UniRef50_Q9LHZ7	1170	63.78	1096	340	15	308	3508	70	1137	0.0	1333
ABCDEF_533	UniRef50_Q9C8W3	0.0	Normal: 230	204	269	505	411	301	BlastAnno: 1688	UniRef50_Q9C8W3	401	70.33	364	94	2	393	1475	47	399	0.0	555
ABCDEF_34099	UniRef50_Q9LE59	0.0	Normal: 478	491	735	1356	1350	548	BlastAnno: 2158	UniRef50_Q9LE59	673	60.92	458	178	1	1610	240	215	672	0.0	630
ABCDEF_47984	UniRef50_Q7PC69	0.0	Normal: 663	457	734	1362	2147	696	BlastAnno: 2838	UniRef50_Q7PC69	745	59.27	712	226	9	373	2316	34	745	0.0	702
ABCDEF_51451	UniRef50_Q9LE59	0.0	Normal: 1883	1782	2202	4337	4350	2667	BlastAnno: 2874	UniRef50_Q9LE59	673	74.16	685	165	6	2559	505	1	673	0.0	982
ABCDEF_13370	UniRef50_Q8VZK9	0.0	Normal: 290	87	317	377	105	258	BlastAnno: 2033	UniRef50_Q8VZK9	729	51.65	635	278	11	2031	154	112	726	0.0	612
ABCDEF_48239	UniRef50_Q9LHZ7	0.0	Normal: 511	163	422	1154	1316	510	BlastAnno: 2714	UniRef50_Q9LHZ7	1170	76.20	769	156	9	478	2712	41	806	0.0	1201
ABCDEF_13338	UniRef50_Q7PC69	0.0	Normal: 335	468	417	2639	4443	616	BlastAnno: 2525	UniRef50_Q7PC69	745	62.45	727	209	12	298	2292	21	745	0.0	781
ABCDEF_24193	UniRef50_Q8L7F9	0.0	Normal: 113	72	132	203	180	119	BlastAnno: 1679	UniRef50_Q8L7F9	643	60.54	479	176	4	212	1609	1	479	0.0	592
ABCDEF_48514	UniRef50_Q9ZV71	0.0	Normal: 540	580	601	771	1033	926	BlastAnno: 1920	UniRef50_Q9ZV71	409	78.14	398	85	2	1618	431	12	409	0.0	637

2.4.1.109(dolichyl-phosphate-mannose-protein mannosyltransferase;)

ABCDEF_11649	UniRef50_B0EDN8	4e-39	Normal: 1465	1004	2251	2426	2774	1761	BlastAnno: 1278	UniRef50_B0EDN8	211	44.20	181	98	3	429	968	33	211	4e-39	147

2.4.1.152(4-galactosyl-N-acetylglucosaminide 3-alpha-L-fucosyltransferase;)

No Correspond Unigene

2.4.1.17(glucuronosyltransferase;)

No Correspond Unigene

2.4.1.221(peptide-O-fucosyltransferase;)

ABCDEF_10513	UniRef50_Q7XAG2	3e-136	Normal: 168	158	183	210	279	468	BlastAnno: 1813	UniRef50_Q7XAG2	444	56.53	375	145	5	1257	169	75	443	3e-136	413

2.4.1.222(O-fucosylpeptide 3-beta-N-acetylglucosaminyltransferase;)

No Correspond Unigene

2.4.1.50(procollagen galactosyltransferase;)

No Correspond Unigene

2.4.1.66(procollagen glucosyltransferase;)

No Correspond Unigene

2.4.2.-(Transferases;)

ABCDEF_17257	UniRef50_Q940Q8	0.0	Normal: 176	142	236	252	196	151	BlastAnno: 1259	UniRef50_Q940Q8	415	91.67	396	33	0	1259	72	20	415	0.0	734
ABCDEF_25833	UniRef50_Q940Q8	0.0	Normal: 28	336	34	93	151	84	BlastAnno: 958	UniRef50_Q940Q8	415	88.89	306	34	0	957	40	110	415	0.0	590
ABCDEF_21016	UniRef50_B8LDL7	0.0	Normal: 775	334	669	635	559	950	BlastAnno: 2086	UniRef50_B8LDL7	570	56.33	561	212	10	1820	234	8	567	0.0	595
ABCDEF_48436	UniRef50_Q940Q8	0.0	Normal: 603	624	571	1077	1211	656	BlastAnno: 1898	UniRef50_Q940Q8	415	90.71	409	37	1	1515	292	7	415	0.0	744

2.4.99.1(beta-galactoside alpha-2,6-sialyltransferase;)

No Correspond Unigene

2.4.99.6(N-acetyllactosaminide alpha-2,3-sialyltransferase;)

No Correspond Unigene

2.8.2.-(Transferases;)

ABCDEF_60620	UniRef50_P52839	2e-85	Normal: 240	393	445	400	902	151	BlastAnno: 1191	UniRef50_P52839	326	45.95	333	168	6	58	1050	2	324	2e-85	271

>00520 Amino sugar and nucleotide sugar metabolism

1.-.-.-(Oxidoreductases)

ABCDEF_345	UniRef50_Q6Z690	0.0	Normal: 442	146	449	340	272	314	BlastAnno: 1658	UniRef50_Q6Z690	849	63.42	462	150	5	1657	314	387	843	0.0	589
ABCDEF_24068	UniRef50_Q9FI41	0.0	Normal: 214	316	207	270	263	849	BlastAnno: 1742	UniRef50_Q9FI41	387	73.28	363	96	1	1440	355	4	366	0.0	554
ABCDEF_24070	UniRef50_Q9FI41	0.0	Normal: 189	290	182	231	232	751	BlastAnno: 1479	UniRef50_Q9FI41	387	73.28	363	96	1	1363	278	4	366	0.0	551
ABCDEF_13957	UniRef50_Q9CAE3	0.0	Normal: 399	318	392	489	456	586	BlastAnno: 2488	UniRef50_Q9CAE3	789	80.85	496	93	2	3	1487	285	779	0.0	768
ABCDEF_28410	UniRef50_Q9LIG0	0.0	Normal: 211	366	234	286	433	1504	BlastAnno: 1324	UniRef50_Q9LIG0	330	74.92	331	76	2	1147	173	1	330	0.0	521
ABCDEF_17581	UniRef50_Q8LGI2	0.0	Normal: 758	336	735	426	374	852	BlastAnno: 1543	UniRef50_Q8LGI2	454	68.78	442	136	2	71	1390	8	449	0.0	597
ABCDEF_23753	UniRef50_Q94A68	0.0	Normal: 284	559	215	224	164	519	BlastAnno: 1580	UniRef50_Q94A68	377	81.90	348	63	0	1349	306	30	377	0.0	576
ABCDEF_24071	UniRef50_Q9FI41	0.0	Normal: 235	377	230	278	289	948	BlastAnno: 2090	UniRef50_Q9FI41	387	73.28	363	96	1	1726	641	4	366	0.0	553

1.1.1.-(Oxidoreductases;)																				
ABCDEF_13453	UniRef50_Q9SYM5	0.0	Normal: 225	226	398	1950	2026	421	BlastAnno: 2578	UniRef50_Q9SYM5	669	86.61	672	87	2	298	2313	1	669	0.0	1226
ABCDEF_17342	UniRef50_Q84M96	0.0	Normal: 274	406	272	327	439	625	BlastAnno: 1277	UniRef50_Q84M96	346	73.33	345	91	1	1187	156	2	346	0.0	546
ABCDEF_31147	UniRef50_Q9SYM5	0.0	Normal: 4571	1591	1491	1401	1939	4327	BlastAnno: 2465	UniRef50_Q9SYM5	669	82.76	673	107	4	2397	394	1	669	0.0	1154
ABCDEF_17344	UniRef50_Q84M96	0.0	Normal: 252	373	257	297	380	540	BlastAnno: 1350	UniRef50_Q84M96	346	68.19	371	91	2	1263	154	2	346	0.0	530
ABCDEF_44674	UniRef50_Q9SYM5	0.0	Normal: 3581	2620	4329	9505	11447	4930	BlastAnno: 2634	UniRef50_Q9SYM5	669	87.80	672	78	3	253	2265	1	669	0.0	1241
1.1.1.132(GDP-mannose 6-dehydrogenase;)																				
No Correspond Unigene																				
1.1.1.135(GDP-6-deoxy-D-talose 4-dehydrogenase;)																				
No Correspond Unigene																				
1.1.1.136(UDP-N-acetylglucosamine 6-dehydrogenase;)																				
No Correspond Unigene																				
1.1.1.158(UDP-N-acetylmuramate dehydrogenase;)																				
ABCDEF_23349	UniRef50_Q6MAQ1	2e-65	Normal: 38	20	38	31	21	19	BlastAnno: 1176	UniRef50_Q6MAQ1	299	49.60	250	122	2	874	125	47	292	2e-65	218
1.1.1.187(GDP-4-dehydro-D-rhamnose reductase;)																				
No Correspond Unigene																				
1.1.1.22(UDP-glucose 6-dehydrogenase;)																				
ABCDEF_30909	UniRef50_A3AK92	0.0	Normal: 865	1783	1481	3602	5414	3960	BlastAnno: 1822	UniRef50_A3AK92	438	76.89	463	77	2	235	1617	1	435	0.0	749
ABCDEF_67405	UniRef50_A3AK92	0.0	Normal: 83	280	233	170	222	547	BlastAnno: 1342	UniRef50_A3AK92	438	77.64	407	61	2	1217	3	1	379	0.0	653
ABCDEF_48744	UniRef50_A3AK92	0.0	Normal: 89	350	273	228	296	697	BlastAnno: 1407	UniRef50_A3AK92	438	77.75	409	61	2	1221	1	1	381	0.0	658
ABCDEF_4415	UniRef50_A3AK92	0.0	Normal: 493	713	643	1516	2818	758	BlastAnno: 1410	UniRef50_A3AK92	438	76.38	381	60	2	1	1137	86	438	0.0	604
ABCDEF_30910	UniRef50_A3AK92	0.0	Normal: 920	2339	1416	3594	6463	6266	BlastAnno: 1816	UniRef50_A3AK92	438	77.54	463	74	2	1591	209	1	435	0.0	751
ABCDEF_30908	UniRef50_A3AK92	0.0	Normal: 2277	2525	3512	8495	12648	3403	BlastAnno: 1908	UniRef50_A3AK92	438	77.25	466	76	2	235	1626	1	438	0.0	755
ABCDEF_73000	UniRef50_Q9AUV6	0.0	Normal: 307	765	498	1248	2232	1778	BlastAnno: 905	UniRef50_Q9AUV6	480	90.37	301	29	0	904	2	95	395	0.0	572
ABCDEF_84803	UniRef50_A3AK92	0.0	Normal: 78	278	221	166	230	569	BlastAnno: 1468	UniRef50_A3AK92	438	77.70	408	61	2	1220	3	1	380	0.0	656
1.1.1.271(GDP-L-fucose synthase;)																				
ABCDEF_25046	UniRef50_O49213	0.0	Normal: 852	424	935	1459	1534	964	BlastAnno: 1394	UniRef50_O49213	312	80.26	309	61	0	112	1038	4	312	0.0	520
1.1.1.281(GDP-4-dehydro-6-deoxy-D-mannose reductase;)																				
No Correspond Unigene																				
1.1.1.305(UDP-glucuronic acid dehydrogenase (UDP-4-keto-hexauronic acid decarboxylating);)																				
No Correspond Unigene																				
1.14.18.2(CMP-N-acetylneuraminate monooxygenase;)																				
No Correspond Unigene																				
1.17.1.1(CDP-4-dehydro-6-deoxyglucose reductase;)																				
No Correspond Unigene																				
1.6.2.2(cytochrome-b5 reductase;)																				
ABCDEF_40796	UniRef50_P83291	3e-151	Normal: 359	153	426	699	1055	333	BlastAnno: 1297	UniRef50_P83291	328	72.62	325	87	2	1238	267	5	328	3e-151	441
2.1.2.-(Transferases;)																				
No Correspond Unigene																				
2.3.1.157(glucosamine-1-phosphate N-acetyltransferase)																				
No Correspond Unigene																				
2.3.1.3(glucosamine N-acetyltransferase;)																				
No Correspond Unigene																				
2.3.1.4(glucosamine-phosphate N-acetyltransferase;)																				
ABCDEF_7697	UniRef50_Q54WR8	9e-38	Normal: 50	56	70	115	142	92	BlastAnno: 812	UniRef50_Q54WR8	157	51.77	141	67	1	161	583	12	151	9e-38	138
2.4.1.16(chitin synthase;)																				
No Correspond Unigene																				
2.4.1.43(polygalacturonate 4-alpha-galacturonosyltransferase;)																				
No Correspond Unigene																				
2.4.2.24(1,4-beta-D-xylan synthase;)																				
No Correspond Unigene																				
2.5.1.56(N-acetylneuraminate synthase;)																				
No Correspond Unigene																				
2.5.1.57(N-acylneuraminate-9-phosphate synthase;)																				
No Correspond Unigene																				
2.5.1.7(UDP-N-acetylglucosamine 1-carboxyvinyltransferase;)																				
No Correspond Unigene																				
2.6.1.16(glutamine---fructose-6-phosphate transaminase (isomerizing);)																				
ABCDEF_10689	UniRef50_Q8SRI2	0.0	Normal: 821	288	776	1014	1042	554	BlastAnno: 2595	UniRef50_Q8SRI2	699	45.60	728	317	13	168	2201	1	699	0.0	626
2.6.1.87(UDP-4-amino-4-deoxy-L-arabinose aminotransferase;)																				
No Correspond Unigene																				
2.7.1.1(hexokinase;)																				
ABCDEF_27834	UniRef50_P93834	0.0	Normal: 998	404	1410	4185	6485	623	BlastAnno: 2374	UniRef50_P93834	502	72.77	470	128	0	2024	615	25	494	0.0	655
ABCDEF_51305	UniRef50_P93834	0.0	Normal: 443	123	280	151	521	245	BlastAnno: 2179	UniRef50_P93834	502	76.47	289	68	0	1813	947	25	313	0.0	467
ABCDEF_420	UniRef50_Q1WM16	0.0	Normal: 453	101	514	402	696	75	BlastAnno: 1554	UniRef50_Q1WM16	463	61.66	433	159	3	3	1295	20	447	0.0	538
ABCDEF_86444	UniRef50_Q1WM16	0.0	Normal: 293	76	186	81	353	207	BlastAnno: 1684	UniRef50_Q1WM16	463	66.83	404	129	3	1404	196	63	462	0.0	530
ABCDEF_592	UniRef50_P93834	0.0	Normal: 378	177	486	1038	1819	341	BlastAnno: 1859	UniRef50_P93834	502	59.87	456	180	3	1618	260	37	492	0.0	537
ABCDEF_83497	UniRef50_Q1WM16	0.0	Normal: 290	84	185	81	350	338	BlastAnno: 1860	UniRef50_Q1WM16	463	66.83	404	129	3	1404	196	63	462	0.0	529
ABCDEF_51306	UniRef50_Q1WM16	0.0	Normal: 446	118	281	150	539	247	BlastAnno: 2108	UniRef50_Q1WM16	463	66.96	451	144	3	1685	336	16	462	0.0	578
ABCDEF_593	UniRef50_P93834	0.0	Normal: 403	191	504	1097	1940	353	BlastAnno: 1902	UniRef50_P93834	502	59.87	456	180	3	1648	290	37	492	0.0	537
2.7.1.2(glucokinase;)																				
No Correspond Unigene																				

2.7.1.4(fructokinase;)																					
ABCDEF_28475	UniRef50_Q9M1B9	0.0	Normal: 2729	2245	2850	2149	3141	1721	BlastAnno: 1388	UniRef50_Q9M1B9	326	85.49	317	45	1	1235	285	1	316	0.0	561
2.7.1.43(glucuronokinase;)																					
No Correspond Unigene																					
2.7.1.44(galacturonokinase;)																					
ABCDEF_41759	UniRef50_Q8VYG2	7e-172	Normal: 559	467	547	814	1373	770	BlastAnno: 1707	UniRef50_Q8VYG2	424	64.67	433	141	2	131	1429	3	423	7e-172	503
2.7.1.46(L-arabinokinase;)																					
ABCDEF_3321	UniRef50_O23461	0.0	Normal: 61	242	47	58	36	421	BlastAnno: 2633	UniRef50_O23461	1039	75.26	392	94	2	3	1169	329	720	0.0	574
ABCDEF_3322	UniRef50_O23461	0.0	Normal: 52	221	39	56	32	394	BlastAnno: 2760	UniRef50_O23461	1039	75.26	392	94	2	3	1169	329	720	0.0	574
ABCDEF_444	UniRef50_O23461	0.0	Normal: 182	49	109	81	115	540	BlastAnno: 1642	UniRef50_O23461	1039	70.51	546	151	4	1640	15	500	1039	0.0	716
ABCDEF_3320	UniRef50_O23461	0.0	Normal: 124	529	66	101	93	719	BlastAnno: 3240	UniRef50_O23461	1039	78.83	978	202	4	3132	211	59	1035	0.0	1524
ABCDEF_87856	UniRef50_O23461	0.0	Normal: 50	239	43	64	39	399	BlastAnno: 2347	UniRef50_O23461	1039	73.12	733	167	5	2345	159	329	1035	0.0	1021
2.7.1.52(fucokinase;)																					
ABCDEF_10588	UniRef50_Q9LNJ9	1e-172	Normal: 184	212	165	177	305	322	BlastAnno: 1507	UniRef50_Q9LNJ9	1113	69.66	445	106	2	1	1260	671	1111	1e-172	525
2.7.1.59(N-acetylglucosamine kinase;)																					
ABCDEF_10169	UniRef50_Q54PM7	5e-59	Normal: 254	188	252	327	279	366	BlastAnno: 1175	UniRef50_Q54PM7	323	35.67	328	189	5	1088	132	6	320	5e-59	202
2.7.1.6(galactokinase;)																					
ABCDEF_4571	UniRef50_Q9SEE5	0.0	Normal: 1190	1054	1340	2243	4224	1615	BlastAnno: 1956	UniRef50_Q9SEE5	496	75.20	500	120	2	1688	189	1	496	0.0	775
2.7.1.60(N-acylmannosamine kinase;)																					
No Correspond Unigene																					
2.7.1.63(polyphosphate---glucose phosphotransferase;)																					
No Correspond Unigene																					
2.7.1.69(protein-Npi-phosphohistidine---sugar phosphotransferase;)																					
No Correspond Unigene																					
2.7.1.7(mannokinase;)																					
No Correspond Unigene																					
2.7.1.8(glucosamine kinase;)																					
No Correspond Unigene																					
2.7.7.-(Transferases;)																					
ABCDEF_9555	UniRef50_Q9VXW2	0.0	Normal: 846	473	829	909	792	562	BlastAnno: 4157	UniRef50_Q9VXW2	1224	54.00	550	235	5	1481	3127	370	902	0.0	593
ABCDEF_9553	UniRef50_Q9VXW2	0.0	Normal: 268	145	246	297	265	213	BlastAnno: 3155	UniRef50_Q9VXW2	1224	54.00	550	235	5	2586	940	370	902	0.0	593
ABCDEF_40110	UniRef50_Q9VF56	0.0	Normal: 1387	922	1252	2141	1749	1299	BlastAnno: 6349	UniRef50_Q9VF56	2183	47.61	1947	918	19	428	5971	231	2174	0.0	1825
ABCDEF_16588	UniRef50_A3EWL3	0.0	Normal: 738	287	520	261	436	448	BlastAnno: 2835	UniRef50_A3EWL3	1105	50.40	744	329	11	2779	608	381	1104	0.0	682
ABCDEF_9552	UniRef50_Q9VXW2	0.0	Normal: 336	227	385	389	378	310	BlastAnno: 3817	UniRef50_Q9VXW2	1224	54.00	550	235	5	3248	1602	370	902	0.0	593
ABCDEF_9556	UniRef50_Q9VXW2	0.0	Normal: 196	108	169	204	178	166	BlastAnno: 2471	UniRef50_Q9VXW2	1224	60.35	454	174	2	2253	895	454	902	0.0	588
ABCDEF_14324	UniRef50_Q84WJ2	0.0	Normal: 538	44	228	5	65	118	BlastAnno: 2216	UniRef50_Q84WJ2	454	70.67	450	128	3	1585	245	1	449	0.0	650
ABCDEF_21043	UniRef50_Q9FJ26	0.0	Normal: 549	92	358	86	155	149	BlastAnno: 1708	UniRef50_Q9FJ26	407	65.96	379	108	3	195	1319	26	387	0.0	533
ABCDEF_9550	UniRef50_Q9VXW2	0.0	Normal: 230	131	215	291	238	198	BlastAnno: 3080	UniRef50_Q9VXW2	1224	54.00	550	235	5	570	2216	370	902	0.0	593
ABCDEF_9551	UniRef50_Q9VXW2	0.0	Normal: 244	147	229	314	258	214	BlastAnno: 3418	UniRef50_Q9VXW2	1224	54.00	550	235	5	570	2216	370	902	0.0	593
ABCDEF_9554	UniRef50_Q9VXW2	0.0	Normal: 750	401	750	826	637	518	BlastAnno: 3842	UniRef50_Q9VXW2	1224	54.00	550	235	5	1301	2947	370	902	0.0	593
ABCDEF_9549	UniRef50_Q9VXW2	0.0	Normal: 301	176	303	350	318	283	BlastAnno: 3569	UniRef50_Q9VXW2	1224	54.00	550	235	5	570	2216	370	902	0.0	593
ABCDEF_14323	UniRef50_Q84WJ2	0.0	Normal: 456	35	201	0	65	103	BlastAnno: 2163	UniRef50_Q84WJ2	454	70.44	450	129	3	579	1919	1	449	0.0	647
2.7.7.10(UTP---hexose-1-phosphate uridylyltransferase;)																					
No Correspond Unigene																					
2.7.7.11(UTP---xylose-1-phosphate uridylyltransferase;)																					
No Correspond Unigene																					
2.7.7.12(UDP-glucose---hexose-1-phosphate uridylyltransferase;)																					
ABCDEF_27856	UniRef50_Q9FK51	5e-135	Normal: 231	186	242	335	418	292	BlastAnno: 1338	UniRef50_Q9FK51	351	64.90	339	115	3	1298	285	15	350	5e-135	401
2.7.7.13(mannose-1-phosphate guanylyltransferase;)																					
ABCDEF_18119	UniRef50_B8LVX3	5e-150	Normal: 2069	3222	1844	3235	5944	6676	BlastAnno: 1767	UniRef50_B8LVX3	741	58.92	370	147	4	342	1439	373	741	5e-150	459
2.7.7.22(mannose-1-phosphate guanylyltransferase (GDP);)																					
No Correspond Unigene																					
2.7.7.23(UDP-N-acetylglucosamine diphosphorylase;)																					
ABCDEF_45697	UniRef50_O64765	0.0	Normal: 729	951	753	2128	3172	1697	BlastAnno: 1929	UniRef50_O64765	502	82.91	474	79	1	1734	319	29	502	0.0	813
2.7.7.27(glucose-1-phosphate adenylyltransferase;)																					
ABCDEF_17985	UniRef50_P55229	0.0	Normal: 1300	638	1009	329	1017	389	BlastAnno: 1576	UniRef50_P55229	522	68.95	409	126	1	1453	227	115	522	0.0	616
ABCDEF_48650	UniRef50_P55229	0.0	Normal: 350	4442	338	474	264	18127	BlastAnno: 1933	UniRef50_P55229	522	78.03	528	107	5	165	1739	1	522	0.0	857
ABCDEF_67742	UniRef50_P55229	0.0	Normal: 1112	1852	1343	1792	835	556	BlastAnno: 2247	UniRef50_P55229	522	66.92	390	129	0	545	1714	80	469	0.0	578
ABCDEF_30971	UniRef50_P55229	0.0	Normal: 1644	1077	1633	2147	2596	1547	BlastAnno: 1963	UniRef50_P55229	522	54.93	497	210	8	1623	157	32	522	0.0	550
ABCDEF_17986	UniRef50_P55229	0.0	Normal: 2123	1065	1571	652	1712	595	BlastAnno: 2169	UniRef50_P55229	522	69.53	443	134	1	1556	228	81	522	0.0	669
ABCDEF_30972	UniRef50_P55229	0.0	Normal: 581	1501	521	667	1424	5770	BlastAnno: 2058	UniRef50_P55229	522	56.85	438	181	5	1701	403	88	522	0.0	535
ABCDEF_4309	UniRef50_P55229	0.0	Normal: 1203	2072	1480	2030	879	662	BlastAnno: 2273	UniRef50_P55229	522	65.91	443	151	0	1729	401	80	522	0.0	647
ABCDEF_30969	UniRef50_P55229	0.0	Normal: 876	980	759	1045	1332	2854	BlastAnno: 2070	UniRef50_P55229	522	54.12	497	214	8	1730	264	32	522	0.0	545
ABCDEF_23917	UniRef50_P55229	0.0	Normal: 384	209	500	1079	959	244	BlastAnno: 2102	UniRef50_P55229	522	66.59	443	148	0	1579	251	80	522	0.0	644
2.7.7.30(fucose-1-phosphate guanylyltransferase;)																					
No Correspond Unigene																					
2.7.7.33(glucose-1-phosphate cytidylyltransferase;)																					
No Correspond Unigene																					
2.7.7.34(glucose-1-phosphate guanylyltransferase;)																					
No Correspond Unigene																					
2.7.7.43(N-acylneuraminate cytidylyltransferase;)																					
No Correspond Unigene																					

2.7.7.44(glucuronate-1-phosphate uridylyltransferase;)																						
No Correspond Unigene																						
2.7.7.64(UTP-monosaccharide-1-phosphate uridylyltransferase;)																						
ABCDEF_4151	UniRef50_Q9C5I1	0.0	Normal: 1490	1023	1424	2446	3108	1795	BlastAnno: 2267	UniRef50_Q9C5I1	614	80.03	586	117	0	2038	281	27	612	0.0	979	
2.7.7.9(UTP---glucose-1-phosphate uridylyltransferase;)																						
ABCDEF_42404	UniRef50_P57751	0.0	Normal: 2120	1934	2860	5861	9792	3375	BlastAnno: 1928	UniRef50_P57751	470	84.13	460	71	2	207	1583	6	464	0.0	756	
ABCDEF_68465	UniRef50_P57751	0.0	Normal: 1684	1594	2449	5480	7890	2628	BlastAnno: 1863	UniRef50_P57751	470	84.13	460	71	2	1678	302	6	464	0.0	756	
2.7.8.30(undecaprenyl-phosphate 4-deoxy-4-formamido-L-arabinose transferase;)																						
No Correspond Unigene																						
3.1.3.29(N-acylneuraminate-9-phosphatase;)																						
No Correspond Unigene																						
3.13.1.1(UDP-sulfoquinovose synthase;)																						
ABCDEF_35370	UniRef50_O48917	0.0	Normal: 1264	1108	1131	1710	2086	4845	BlastAnno: 2024	UniRef50_O48917	477	79.04	477	93	3	1732	317	1	475	0.0	760	
3.2.1.-(Hydrolases;)																						
ABCDEF_55518	UniRef50_Q9SGZ5	0.0	Normal: 137	145	110	155	249	211	BlastAnno: 1222	UniRef50_Q9SGZ5	767	66.34	407	136	1	1218	1	142	548	0.0	556	
ABCDEF_45209	UniRef50_Q9LXA8	0.0	Normal: 1338	811	1191	1817	1167	1821	BlastAnno: 2643	UniRef50_Q9LXA8	792	47.09	790	379	11	2448	178	7	790	0.0	745	
ABCDEF_34153	UniRef50_Q9LXA8	0.0	Normal: 522	668	469	700	564	936	BlastAnno: 2528	UniRef50_Q9LXA8	792	51.44	762	345	6	2397	175	30	787	0.0	804	
ABCDEF_34154	UniRef50_Q9LXA8	0.0	Normal: 495	622	454	650	534	930	BlastAnno: 2619	UniRef50_Q9LXA8	792	50.53	655	301	5	2076	175	135	787	0.0	679	
ABCDEF_47469	UniRef50_Q9SXC9	0.0	Normal: 80	59	95	141	105	115	BlastAnno: 1550	UniRef50_Q9SXC9	574	81.45	248	46	0	3	746	93	340	0.0	419	
ABCDEF_3050	UniRef50_A8JBU2	0.0	Normal: 365	517	518	1626	1809	451	BlastAnno: 2998	UniRef50_A8JBU2	725	52.75	673	264	8	2589	604	50	679	0.0	670	
ABCDEF_3049	UniRef50_A8JBU2	0.0	Normal: 528	690	724	2218	2620	674	BlastAnno: 3077	UniRef50_A8JBU2	725	52.32	711	286	7	2650	551	55	723	0.0	707	
ABCDEF_34152	UniRef50_Q9LXA8	0.0	Normal: 568	815	532	816	648	1135	BlastAnno: 2581	UniRef50_Q9LXA8	792	51.24	765	348	6	2450	219	30	790	0.0	806	
ABCDEF_34155	UniRef50_Q9LXA8	0.0	Normal: 543	763	515	772	621	1127	BlastAnno: 2640	UniRef50_Q9LXA8	792	50.30	658	304	5	2112	202	135	790	0.0	681	
ABCDEF_21385	UniRef50_Q9LXA8	0.0	Normal: 432	334	511	873	734	380	BlastAnno: 2109	UniRef50_Q9LXA8	792	62.64	629	229	4	3	1877	152	778	0.0	775	
ABCDEF_980	UniRef50_Q9LXA8	0.0	Normal: 1398	7037	1070	4974	7681	6612	BlastAnno: 2765	UniRef50_Q9LXA8	792	49.22	772	351	9	257	2470	28	792	0.0	753	
ABCDEF_31427	UniRef50_Q9LXA8	0.0	Normal: 56	316	89	4893	4124	2869	BlastAnno: 2823	UniRef50_Q9LXA8	792	48.25	773	359	12	209	2425	26	791	0.0	716	
ABCDEF_27305	UniRef50_Q9FG93	0.0	Normal: 247	143	251	353	378	254	BlastAnno: 1883	UniRef50_Q9FG93	624	70.43	487	123	3	2	1456	126	593	0.0	702	
ABCDEF_47466	UniRef50_Q9SXC9	0.0	Normal: 69	48	83	133	95	97	BlastAnno: 1452	UniRef50_Q9SXC9	574	75.84	476	115	0	2	1429	96	571	0.0	769	
ABCDEF_3403	UniRef50_Q9LXA8	0.0	Normal: 206	82	182	114	358	291	BlastAnno: 2202	UniRef50_Q9LXA8	792	55.15	515	213	4	2198	702	84	596	0.0	548	
3.2.1.132(chitosanase)																						
No Correspond Unigene																						
3.2.1.14(chitinase;)																						
ABCDEF_2952	UniRef50_Q9SLP4	3e-116	Normal: 106	121	134	192	299	110	BlastAnno: 1032	UniRef50_Q9SLP4	314	66.54	254	84	1	771	10	30	282	3e-116	348	
3.2.1.37(xylan 1,4-beta-xylosidase;)																						
ABCDEF_34152	UniRef50_Q2MCJ4	0.0	Normal: 568	815	532	816	648	1135	BlastAnno: 2581	UniRef50_Q2MCJ4	704	54.24	754	258	13	2450	213	30	704	0.0	789	
ABCDEF_45209	UniRef50_Q9FLG1	0.0	Normal: 1338	811	1191	1817	1167	1821	BlastAnno: 2643	UniRef50_Q9FLG1	784	67.01	773	244	5	2469	172	16	784	0.0	1102	
ABCDEF_34155	UniRef50_Q2MCJ4	0.0	Normal: 543	763	515	772	621	1127	BlastAnno: 2640	UniRef50_Q2MCJ4	704	52.88	643	221	10	2112	196	140	704	0.0	660	
ABCDEF_34153	UniRef50_Q2MCJ4	0.0	Normal: 522	668	469	700	564	936	BlastAnno: 2528	UniRef50_Q2MCJ4	704	54.41	748	254	13	2397	178	30	698	0.0	779	
ABCDEF_34154	UniRef50_Q2MCJ4	0.0	Normal: 495	622	454	650	534	930	BlastAnno: 2619	UniRef50_Q2MCJ4	704	53.06	637	217	10	2076	178	140	698	0.0	650	
ABCDEF_980	UniRef50_Q9FLG1	0.0	Normal: 1398	7037	1070	4974	7681	6612	BlastAnno: 2765	UniRef50_Q9FLG1	784	63.28	749	268	2	242	2470	37	784	0.0	1008	
ABCDEF_31427	UniRef50_Q9FLG1	0.0	Normal: 56	316	89	4893	4124	2869	BlastAnno: 2823	UniRef50_Q9FLG1	784	56.12	768	309	12	179	2425	25	783	0.0	849	
3.2.1.52(beta-N-acetylhexosaminidase;)																						
ABCDEF_16822	UniRef50_Q54K56	3e-109	Normal: 543	493	486	692	917	660	BlastAnno: 1760	UniRef50_Q54K56	564	36.42	519	294	10	1587	94	59	562	3e-109	347	
3.2.1.55(alpha-N-arabinofuranosidase;)																						
ABCDEF_10664	UniRef50_Q8VZR2	0.0	Normal: 589	1567	439	1827	1493	2613	BlastAnno: 2599	UniRef50_Q8VZR2	674	62.56	673	234	10	2204	207	11	672	0.0	855	
3.4.1.-(No Name)																						
No Correspond Unigene																						
3.5.1.-(Hydrolases;)																						
ABCDEF_41579	UniRef50_O81641	0.0	Normal: 384	122	265	320	313	150	BlastAnno: 1456	UniRef50_O81641	428	65.16	399	138	1	1313	120	23	421	0.0	543	
ABCDEF_16924	UniRef50_Q8VYX0	0.0	Normal: 135	349	460	1325	685	527	BlastAnno: 2002	UniRef50_Q8VYX0	464	71.72	396	109	2	1612	428	68	461	0.0	549	
ABCDEF_24290	UniRef50_O04373	0.0	Normal: 296	238	403	498	698	330	BlastAnno: 1710	UniRef50_O04373	440	65.24	443	151	3	67	1395	1	440	0.0	595	
3.5.1.25(N-acetylglucosamine-6-phosphate deacetylase;)																						
No Correspond Unigene																						
3.5.1.33(N-acetylglucosamine deacetylase;)																						
No Correspond Unigene																						
3.5.1.41(chitin deacetylase)																						
ABCDEF_79368	UniRef50_Q9VW30	4e-27	Normal: 0	0	0	0	0	10	BlastAnno: 233	UniRef50_Q9VW30	549	62.34	77	29	0	2	232	444	520	4e-27	108	
3.5.99.6(glucosamine-6-phosphate deaminase;)																						
No Correspond Unigene																						
4.1.1.35(UDP-glucuronate decarboxylase;)																						
ABCDEF_11113	UniRef50_B9SAR7	0.0	Normal: 925	1155	753	449	2067	835	BlastAnno: 1952	UniRef50_B9SAR7	419	74.43	442	79	5	1658	342	1	411	0.0	645	
ABCDEF_35392	UniRef50_B9SAR7	0.0	Normal: 298	348	433	969	1317	656	BlastAnno: 1691	UniRef50_B9SAR7	419	65.74	432	117	2	247	1524	11	417	0.0	552	
4.1.1.67(UDP-galacturonate decarboxylase;)																						
No Correspond Unigene																						
4.1.3.3(N-acetylneuraminate lyase;)																						
No Correspond Unigene																						
4.2.-.-(Lyases;)																						
No Correspond Unigene																						
4.2.1.-(Lyases;)																						
ABCDEF_48679	UniRef50_Q0RP29	5e-115	Normal: 568	325	563	941	1285	676	BlastAnno: 1424	UniRef50_Q0RP29	493	56.39	321	129	4	119	1075	39	350	5e-115	356	
4.2.1.45(CDP-glucose 4,6-dehydratase;)																						
No Correspond Unigene																						

4.2.1.47(GDP-mannose 4,6-dehydratase;)																					
ABCDEF_48679	UniRef50_P93031	0.0	Normal: 568	325	563	941	1285	676	BlastAnno: 1424	UniRef50_P93031	373	84.86	370	54	2	41	1150	6	373	0.0	667
4.2.1.76(UDP-glucose 4,6-dehydratase;)																					
No Correspond Unigene																					
5.1.-.-(Isomerases;)																					
ABCDEF_43198	UniRef50_Q9DCG6	9e-15	Normal: 9	1	0	2	59	304	BlastAnno: 591	UniRef50_Q9DCG6	288	40.00	140	70	3	49	468	6	131	9e-15	76.3
5.1.3.-(Isomerases;)																					
No Correspond Unigene																					
5.1.3.10(CDP-paratose 2-epimerase;)																					
No Correspond Unigene																					
5.1.3.12(UDP-glucuronate 5'-epimerase;)																					
ABCDEF_34307	UniRef50_Q2RNC5	2e-87	Normal: 375	325	466	1035	1289	406	BlastAnno: 2245	UniRef50_Q2RNC5	335	45.27	338	174	5	764	1759	1	333	2e-87	287
5.1.3.14(UDP-N-acetylglucosamine 2-epimerase;)																					
No Correspond Unigene																					
5.1.3.18(GDP-mannose 3,5-epimerase;)																					
ABCDEF_4728	UniRef50_Q93VR3	0.0	Normal: 3786	7995	2857	3393	6154	12151	BlastAnno: 1535	UniRef50_Q93VR3	377	92.04	377	29	1	161	1288	1	377	0.0	735
5.1.3.2(UDP-glucose 4-epimerase;)																					
ABCDEF_52756	UniRef50_Q42605	0.0	Normal: 448	2203	636	898	950	6504	BlastAnno: 1607	UniRef50_Q42605	351	80.87	345	66	0	1262	228	6	350	0.0	596
5.1.3.5(UDP-arabinose 4-epimerase;)																					
ABCDEF_51689	UniRef50_Q9SA77	0.0	Normal: 494	114	326	186	170	325	BlastAnno: 1674	UniRef50_Q9SA77	419	83.21	411	68	1	181	1413	1	410	0.0	691
ABCDEF_3389	UniRef50_Q9SA77	0.0	Normal: 357	215	359	626	550	341	BlastAnno: 1753	UniRef50_Q9SA77	419	83.66	410	67	0	1556	327	1	410	0.0	729
5.1.3.6(UDP-glucuronate 4-epimerase;)																					
ABCDEF_40845	UniRef50_Q9LIS3	0.0	Normal: 657	4113	785	1682	3724	4096	BlastAnno: 1905	UniRef50_Q9LIS3	460	77.48	453	95	3	1780	437	8	458	0.0	670
ABCDEF_7044	UniRef50_Q9LIS3	0.0	Normal: 347	255	406	1043	998	1296	BlastAnno: 1923	UniRef50_Q9LIS3	460	72.75	378	93	1	1317	214	67	444	0.0	549
ABCDEF_34307	UniRef50_Q9LIS3	0.0	Normal: 375	325	466	1035	1289	406	BlastAnno: 2245	UniRef50_Q9LIS3	460	75.35	353	87	0	704	1762	92	444	0.0	568
ABCDEF_7309	UniRef50_Q9LIS3	0.0	Normal: 318	240	786	2596	2150	368	BlastAnno: 2125	UniRef50_Q9LIS3	460	76.92	351	81	0	1451	399	93	443	0.0	535
ABCDEF_40847	UniRef50_Q9LIS3	0.0	Normal: 355	521	497	2860	2581	1569	BlastAnno: 1492	UniRef50_Q9LIS3	460	76.44	450	98	1	1382	57	3	452	0.0	671
ABCDEF_40846	UniRef50_Q9LIS3	0.0	Normal: 362	1911	403	1392	2019	2084	BlastAnno: 1462	UniRef50_Q9LIS3	460	78.43	445	91	2	1366	47	8	452	0.0	670
5.1.3.8(N-acylglucosamine 2-epimerase;)																					
No Correspond Unigene																					
5.1.3.9(N-acylglucosamine-6-phosphate 2-epimerase;)																					
ABCDEF_21449	UniRef50_B7PPV8	2e-89	Normal: 405	273	419	292	397	607	BlastAnno: 1651	UniRef50_B7PPV8	483	44.69	320	156	4	421	1371	11	312	2e-89	291
5.3.1.8(mannose-6-phosphate isomerase;)																					
ABCDEF_3858	UniRef50_Q67YT4	1e-176	Normal: 413	826	453	537	588	677	BlastAnno: 1801	UniRef50_Q67YT4	441	58.00	450	173	5	1474	134	2	438	1e-176	517
5.3.1.9(glucose-6-phosphate isomerase;)																					
ABCDEF_21891	UniRef50_B5EBB8	0.0	Normal: 2382	2326	3109	5032	5330	3316	BlastAnno: 2692	UniRef50_B5EBB8	530	60.59	510	196	2	2176	647	4	508	0.0	624
ABCDEF_4218	UniRef50_B7GDV0	0.0	Normal: 982	971	1324	2230	3149	3125	BlastAnno: 2219	UniRef50_B7GDV0	786	56.56	564	238	4	2045	375	188	751	0.0	639
5.4.2.10(phosphoglucosamine mutase)																					
No Correspond Unigene																					
5.4.2.2(phosphoglucomutase;)																					
ABCDEF_935	UniRef50_P47244	0.0	Normal: 5430	4330	5743	12110	19002	6223	BlastAnno: 2144	UniRef50_P47244	572	53.66	587	246	11	2064	328	4	572	0.0	585
ABCDEF_24641	UniRef50_P47244	0.0	Normal: 416	659	414	959	874	2109	BlastAnno: 2341	UniRef50_P47244	572	54.17	576	222	11	386	2035	13	572	0.0	588
ABCDEF_24640	UniRef50_P47244	0.0	Normal: 389	596	384	834	777	1859	BlastAnno: 2422	UniRef50_P47244	572	51.74	603	222	12	386	2116	13	572	0.0	574
5.4.2.3(phosphoacetylglucosamine mutase;)																					
ABCDEF_45205	UniRef50_P57750	0.0	Normal: 549	397	888	915	822	635	BlastAnno: 2406	UniRef50_P57750	556	65.18	560	185	5	2020	356	1	555	0.0	722
ABCDEF_45206	UniRef50_P57750	0.0	Normal: 568	401	803	912	858	630	BlastAnno: 2260	UniRef50_P57750	556	65.36	560	184	5	241	1905	1	555	0.0	726
5.4.2.5(phosphoglucomutase (glucose-cofactor);)																					
No Correspond Unigene																					
5.4.2.8(phosphomannomutase;)																					
ABCDEF_10400	UniRef50_A6LQV8	3e-144	Normal: 173	143	196	105	138	487	BlastAnno: 2096	UniRef50_A6LQV8	521	46.33	531	248	9	1845	274	17	517	3e-144	440
>00521 Streptomycin biosynthesis																					
1.1.1.133(dTDP-4-dehydrorhamnose reductase;)																					
No Correspond Unigene																					
1.1.1.18(inositol 2-dehydrogenase;)																					
No Correspond Unigene																					
2.1.4.2(scyllo-inosamine-4-phosphate amidinotransferase;)																					
No Correspond Unigene																					
2.4.2.27(dTDP-dihydrostreptose---streptidine-6-phosphate dihydrostreptosyltransferase;)																					
No Correspond Unigene																					
2.6.1.50(glutamine---scyllo-inositol transaminase;)																					
No Correspond Unigene																					
2.6.1.56(1D-1-guanidino-3-amino-1,3-dideoxy-scyllo-inositol transaminase;)																					
No Correspond Unigene																					
2.7.1.1(hexokinase;)																					
ABCDEF_27834	UniRef50_P93834	0.0	Normal: 998	404	1410	4185	6485	623	BlastAnno: 2374	UniRef50_P93834	502	72.77	470	128	0	2024	615	25	494	0.0	655
ABCDEF_51305	UniRef50_P93834	0.0	Normal: 443	123	280	151	521	245	BlastAnno: 2179	UniRef50_P93834	502	76.47	289	68	0	1813	947	25	313	0.0	467
ABCDEF_420	UniRef50_Q1WM16	0.0	Normal: 453	101	514	402	696	75	BlastAnno: 1554	UniRef50_Q1WM16	463	61.66	433	159	3	3	1295	20	447	0.0	538
ABCDEF_86444	UniRef50_Q1WM16	0.0	Normal: 293	76	186	81	353	207	BlastAnno: 1684	UniRef50_Q1WM16	463	66.83	404	129	3	1404	196	63	462	0.0	530
ABCDEF_592	UniRef50_P93834	0.0	Normal: 378	177	486	1038	1819	341	BlastAnno: 1859	UniRef50_P93834	502	59.87	456	180	3	1618	260	37	492	0.0	537
ABCDEF_83497	UniRef50_Q1WM16	0.0	Normal: 290	84	185	81	350	338	BlastAnno: 1860	UniRef50_Q1WM16	463	66.83	404	129	3	1404	196	63	462	0.0	529
ABCDEF_51306	UniRef50_Q1WM16	0.0	Normal: 446	118	281	150	539	247	BlastAnno: 2108	UniRef50_Q1WM16	463	66.96	451	144	3	1685	336	16	462	0.0	578
ABCDEF_593	UniRef50_P93834	0.0	Normal: 403	191	504	1097	1940	353	BlastAnno: 1902	UniRef50_P93834	502	59.87	456	180	3	1648	290	37	492	0.0	537

2.7.1.2(glucokinase;)																					
No Correspond Unigene																					
2.7.1.65(scyllo-inosamine 4-kinase;)																					
No Correspond Unigene																					
2.7.1.72(streptomycin 6-kinase;)																					
No Correspond Unigene																					
2.7.7.24(glucose-1-phosphate thymidylyltransferase;)																					
No Correspond Unigene																					
3.1.3.25(inositol-phosphate phosphatase;)																					
ABCDEF_24298	UniRef50_Q94F00	4e-170	Normal: 647	536	647	1368	1728	818	BlastAnno: 1549	UniRef50_Q94F00	371	64.61	373	126	2	41	1147	1	371	4e-170	494
3.1.3.39(streptomycin-6-phosphatase;)																					
No Correspond Unigene																					
3.1.3.40(guanidinodeoxy-scyllo-inositol-4-phosphatase;)																					
No Correspond Unigene																					
4.2.1.46(dTDP-glucose 4,6-dehydratase;)																					
ABCDEF_13453	UniRef50_O95455	1e-100	Normal: 225	226	398	1950	2026	421	BlastAnno: 2578	UniRef50_O95455	350	48.21	336	165	3	316	1308	18	349	1e-100	325
5.1.3.13(dTDP-4-dehydrorhamnose 3,5-epimerase;)																					
No Correspond Unigene																					
5.4.2.2(phosphoglucomutase;)																					
ABCDEF_935	UniRef50_P47244	0.0	Normal: 5430	4330	5743	12110	19002	6223	BlastAnno: 2144	UniRef50_P47244	572	53.66	587	246	11	2064	328	4	572	0.0	585
ABCDEF_24641	UniRef50_P47244	0.0	Normal: 416	659	414	959	874	2109	BlastAnno: 2341	UniRef50_P47244	572	54.17	576	222	11	386	2035	13	572	0.0	588
ABCDEF_24640	UniRef50_P47244	0.0	Normal: 389	596	384	834	777	1859	BlastAnno: 2422	UniRef50_P47244	572	51.74	603	222	12	386	2116	13	572	0.0	574
5.5.1.4(inositol-3-phosphate synthase;)																					
ABCDEF_35470	UniRef50_Q9NPH2	0.0	Normal: 4323	922	2996	984	2481	19110	BlastAnno: 2022	UniRef50_Q9NPH2	558	59.88	506	197	4	202	1716	7	507	0.0	649
>00522 Biosynthesis of 12-, 14- and 16-membered macrolides																					
1.14.-.-(Oxidoreductases;)																					
ABCDEF_44739	UniRef50_O64989	0.0	Normal: 157	87	159	718	673	189	BlastAnno: 2185	UniRef50_O64989	513	70.75	482	112	3	1867	509	30	511	0.0	706
ABCDEF_31491	UniRef50_Q93VK5	0.0	Normal: 647	1826	671	1517	1770	5953	BlastAnno: 2278	UniRef50_Q93VK5	595	79.14	556	113	2	198	1862	35	588	0.0	902
ABCDEF_40554	UniRef50_P37122	0.0	Normal: 1574	1962	1829	1150	2882	2721	BlastAnno: 1828	UniRef50_P37122	505	60.52	504	198	1	1752	244	1	504	0.0	638
ABCDEF_34049	UniRef50_O23365	0.0	Normal: 363	384	349	442	549	1416	BlastAnno: 2101	UniRef50_O23365	580	76.17	579	116	5	230	1957	19	578	0.0	904
ABCDEF_21579	UniRef50_O48928	0.0	Normal: 1005	643	1606	4372	3226	1621	BlastAnno: 1895	UniRef50_O48928	513	69.72	502	151	1	185	1690	7	507	0.0	731
ABCDEF_37741	UniRef50_P37122	0.0	Normal: 12	760	9	24	34	576	BlastAnno: 1197	UniRef50_P37122	505	65.70	379	129	1	1195	59	127	504	0.0	526
ABCDEF_35618	UniRef50_O48928	0.0	Normal: 28	2648	17	142	1234	902	BlastAnno: 1746	UniRef50_O48928	513	66.40	506	168	2	1649	132	4	507	0.0	701
ABCDEF_24522	UniRef50_P37122	0.0	Normal: 334	2190	561	268	1391	2459	BlastAnno: 2075	UniRef50_P37122	505	61.52	460	176	1	1544	165	47	505	0.0	612
ABCDEF_14592	UniRef50_O23066	0.0	Normal: 1514	402	2245	7837	4742	1248	BlastAnno: 2378	UniRef50_O23066	553	72.71	546	140	4	2111	489	1	542	0.0	821
ABCDEF_14512	UniRef50_Q9FG65	0.0	Normal: 261	872	212	291	175	789	BlastAnno: 1878	UniRef50_Q9FG65	502	56.89	501	197	9	1738	266	11	502	0.0	532
ABCDEF_44487	UniRef50_Q940V4	0.0	Normal: 3	41	53	501	262	542	BlastAnno: 1712	UniRef50_Q940V4	465	69.37	382	116	1	38	1180	14	395	0.0	572
ABCDEF_17723	UniRef50_P37122	0.0	Normal: 240	1698	219	768	592	794	BlastAnno: 1757	UniRef50_P37122	505	63.40	459	166	2	228	1601	47	504	0.0	617
ABCDEF_37901	UniRef50_Q42569	0.0	Normal: 30	149	86	255	195	255	BlastAnno: 1246	UniRef50_Q42569	472	78.27	359	73	2	64	1140	43	396	0.0	567
ABCDEF_7260	UniRef50_O81117	0.0	Normal: 314	1085	466	3248	2575	1360	BlastAnno: 1768	UniRef50_O81117	514	59.92	474	188	1	145	1566	43	514	0.0	615
ABCDEF_40548	UniRef50_P37122	0.0	Normal: 2756	2467	2752	1072	3120	1735	BlastAnno: 1947	UniRef50_P37122	505	59.72	504	202	1	241	1749	1	504	0.0	636
ABCDEF_44485	UniRef50_Q940V4	0.0	Normal: 3	55	63	574	338	660	BlastAnno: 1607	UniRef50_Q940V4	465	68.58	452	141	1	42	1394	14	465	0.0	673
ABCDEF_40552	UniRef50_P37122	0.0	Normal: 2397	1870	2304	1249	10428	2291	BlastAnno: 1812	UniRef50_P37122	505	60.12	504	200	1	108	1616	1	504	0.0	640
ABCDEF_37902	UniRef50_Q42569	0.0	Normal: 84	524	254	725	670	1049	BlastAnno: 1812	UniRef50_Q42569	472	77.47	435	92	3	1675	371	43	471	0.0	681
ABCDEF_40556	UniRef50_P37122	0.0	Normal: 1907	2365	2163	1319	3678	3308	BlastAnno: 1944	UniRef50_P37122	505	61.04	498	193	1	264	1754	1	498	0.0	635
ABCDEF_40549	UniRef50_P37122	0.0	Normal: 1574	1962	1826	1150	2882	2723	BlastAnno: 1845	UniRef50_P37122	505	60.52	504	198	1	1754	246	1	504	0.0	638
ABCDEF_20301	UniRef50_O81117	0.0	Normal: 89	308	123	176	389	249	BlastAnno: 1637	UniRef50_O81117	514	64.04	470	164	3	1498	98	47	514	0.0	586
ABCDEF_3882	UniRef50_Q42569	0.0	Normal: 84	243	257	786	1561	182	BlastAnno: 1854	UniRef50_Q42569	472	72.51	422	107	1	1852	587	59	471	0.0	615
ABCDEF_20703	UniRef50_O49858	0.0	Normal: 115	636	20	0	0	597	BlastAnno: 1815	UniRef50_O49858	527	50.10	523	248	6	48	1607	12	524	0.0	533
ABCDEF_18295	UniRef50_O22203	0.0	Normal: 3667	1934	4977	10491	4588	1917	BlastAnno: 2006	UniRef50_O22203	508	74.85	493	123	1	214	1692	17	508	0.0	803
ABCDEF_48724	UniRef50_O23066	0.0	Normal: 104	11518	163	2083	3464	6356	BlastAnno: 2162	UniRef50_O23066	553	72.96	540	143	3	227	1840	1	539	0.0	807
ABCDEF_37738	UniRef50_P37122	0.0	Normal: 26	2447	19	30	33	993	BlastAnno: 1837	UniRef50_P37122	505	64.30	479	169	2	1685	249	28	504	0.0	639
2.1.1.-(Transferases;)																					
ABCDEF_20806	UniRef50_Q94II3	0.0	Normal: 700	332	876	1458	1997	929	BlastAnno: 2275	UniRef50_Q94II3	600	73.57	594	153	4	2119	341	1	591	0.0	907
ABCDEF_45802	UniRef50_Q9ZPH9	0.0	Normal: 3543	3348	4296	9797	10830	5017	BlastAnno: 2642	UniRef50_Q9ZPH9	633	53.90	603	261	7	2216	456	23	624	0.0	676
ABCDEF_20070	UniRef50_Q944R7	0.0	Normal: 301	141	265	207	185	187	BlastAnno: 2675	UniRef50_Q944R7	724	58.28	719	275	9	127	2265	13	712	0.0	840
ABCDEF_34956	UniRef50_Q94II3	0.0	Normal: 1120	7269	2475	16033	22219	21461	BlastAnno: 2676	UniRef50_Q94II3	600	73.77	610	146	4	404	2230	1	597	0.0	957
ABCDEF_17121	UniRef50_Q9ZPH9	0.0	Normal: 124	77	109	284	199	132	BlastAnno: 2066	UniRef50_Q9ZPH9	633	53.24	601	259	8	161	1903	25	623	0.0	644
ABCDEF_6524	UniRef50_Q9ZPH9	0.0	Normal: 471	296	490	753	870	730	BlastAnno: 2602	UniRef50_Q9ZPH9	633	50.89	619	290	5	2221	404	6	623	0.0	659
ABCDEF_56017	UniRef50_Q9ZPH9	0.0	Normal: 302	167	286	596	748	529	BlastAnno: 2384	UniRef50_Q9ZPH9	633	51.37	619	287	5	213	2030	6	623	0.0	663
ABCDEF_44169	UniRef50_Q9SIZ3	0.0	Normal: 179	158	273	705	685	229	BlastAnno: 2300	UniRef50_Q9SIZ3	589	62.01	308	108	4	1219	296	285	583	0.0	394
ABCDEF_6521	UniRef50_Q9ZPH9	0.0	Normal: 339	198	314	641	767	582	BlastAnno: 2511	UniRef50_Q9ZPH9	633	51.37	619	287	5	2172	355	6	623	0.0	663
ABCDEF_44172	UniRef50_Q9SIZ3	0.0	Normal: 204	184	287	767	741	242	BlastAnno: 2317	UniRef50_Q9SIZ3	589	68.15	449	134	4	1637	291	144	583	0.0	641
ABCDEF_199	UniRef50_Q9SU94	0.0	Normal: 1076	275	1009	558	1247	306	BlastAnno: 1769	UniRef50_Q9SU94	390	78.45	348	74	1	284	1324	43	390	0.0	584
ABCDEF_52252	UniRef50_Q84W92	0.0	Normal: 1658	720	1653	1980	2289	1601	BlastAnno: 2170	UniRef50_Q84W92	535	72.49	469	120	3	409	1791	34	501	0.0	703
ABCDEF_51404	UniRef50_A3BMN9	0.0	Normal: 864	188	716	337	631	326	BlastAnno: 2192	UniRef50_A3BMN9	620	50.50	604	243	9	2008	251	55	620	0.0	555
ABCDEF_21386	UniRef50_Q94AI4	0.0	Normal: 2326	1250	2220	4294	2920	1983	BlastAnno: 3701	UniRef50_Q94AI4	775	47.27	842	332	16	567	2978	8	775	0.0	577
ABCDEF_35101	UniRef50_Q9MAT5	0.0	Normal: 1440	420	1268	576	1136	850	BlastAnno: 1634	UniRef50_Q9MAT5	383	69.03	352	109	0	1449	394	32	383	0.0	550
ABCDEF_960	UniRef50_Q9LN50	0.0	Normal: 3504	13198	3609	11156	19651	12155	BlastAnno: 2626	UniRef50_Q9LN50	724	49.14	525	246	5	883	2451	219	724	0.0	570
ABCDEF_48082	UniRef50_Q9LN50	0.0	Normal: 410	445	467	870	993	454	BlastAnno: 2340	UniRef50_Q9LN50	724	70.25	521	155	0	710	2272	204	724	0.0	821
ABCDEF_10473	UniRef50_Q08A71	0.0	Normal: 374	187	332	425	456	314	BlastAnno: 1764	UniRef50_Q08A71	435	79.23	366	75	1	344	1438	70	435	0.0	575
ABCDEF_7295	UniRef50_Q9ZPH9	0.0	Normal: 25	54	30	81	26	329	BlastAnno: 1191	UniRef50_Q9ZPH9	633	69.23	403	110	4	1191	22	225	626	0.0	599

ABCDEF_4160	UniRef50_Q94II3	0.0	Normal: 189	0	210	71	17	72	BlastAnno: 1424	UniRef50_Q94II3	600	77.28	449	101	1	1424	81	143	591	0.0	758
ABCDEF_44857	UniRef50_Q9ZPH9	0.0	Normal: 101	224	103	299	588	350	BlastAnno: 1582	UniRef50_Q9ZPH9	633	72.06	519	143	1	1571	21	114	632	0.0	822
ABCDEF_71541	UniRef50_Q9SIZ3	0.0	Normal: 187	167	296	765	724	237	BlastAnno: 2268	UniRef50_Q9SIZ3	589	62.01	308	108	4	1210	287	285	583	0.0	394
ABCDEF_44171	UniRef50_Q9SIZ3	0.0	Normal: 214	196	312	827	802	251	BlastAnno: 2339	UniRef50_Q9SIZ3	589	64.78	548	182	5	1978	335	47	583	0.0	717
ABCDEF_20805	UniRef50_Q94II3	0.0	Normal: 674	344	885	1417	2194	963	BlastAnno: 2400	UniRef50_Q94II3	600	78.21	436	94	1	879	2183	156	591	0.0	734
ABCDEF_18210	UniRef50_Q9SIZ3	0.0	Normal: 5584	3714	7379	17006	21892	7546	BlastAnno: 3199	UniRef50_Q9SIZ3	589	50.00	576	269	7	2145	424	27	585	0.0	574
ABCDEF_6526	UniRef50_Q9ZPH9	0.0	Normal: 627	185	837	2717	2815	1530	BlastAnno: 2611	UniRef50_Q9ZPH9	633	54.76	599	259	5	462	2222	25	623	0.0	689
ABCDEF_6525	UniRef50_Q9ZPH9	0.0	Normal: 602	165	760	2369	2605	1400	BlastAnno: 2545	UniRef50_Q9ZPH9	633	54.92	599	258	5	431	2191	25	623	0.0	689
ABCDEF_6523	UniRef50_Q9ZPH9	0.0	Normal: 314	175	304	641	784	552	BlastAnno: 2312	UniRef50_Q9ZPH9	633	52.42	599	272	4	201	1958	25	623	0.0	663
ABCDEF_67393	UniRef50_Q9ZPH9	0.0	Normal: 434	245	444	608	768	655	BlastAnno: 2346	UniRef50_Q9ZPH9	633	51.92	599	275	4	232	1989	25	623	0.0	659
ABCDEF_3579	UniRef50_O14744	0.0	Normal: 1361	326	984	568	791	440	BlastAnno: 2369	UniRef50_O14744	637	47.00	634	294	10	267	2120	30	637	0.0	553

2.1.1.101(macrocin O-methyltransferase;)

No Correspond Unigene

2.1.1.102(demethylmacrocin O-methyltransferase;)

No Correspond Unigene

2.3.1.94(6-deoxyerythronolide-B synthase;)

No Correspond Unigene

2.4.1.-(Transferases;)

ABCDEF_18004	UniRef50_Q6YWK8	0.0	Normal: 1562	1736	2009	3452	4438	2400	BlastAnno: 2298	UniRef50_Q6YWK8	570	65.27	429	136	1	1941	694	57	485	0.0	608
ABCDEF_52379	UniRef50_Q9LHZ7	0.0	Normal: 4015	4921	4546	10520	13430	4731	BlastAnno: 3479	UniRef50_Q9LHZ7	1170	48.04	893	342	16	825	3242	299	1156	0.0	787
ABCDEF_48593	UniRef50_Q9SK82	0.0	Normal: 12	420	19	343	763	253	BlastAnno: 1834	UniRef50_Q9SK82	489	60.08	481	186	4	1714	278	10	486	0.0	633
ABCDEF_13375	UniRef50_Q8VZK9	0.0	Normal: 108	123	208	526	34	294	BlastAnno: 1738	UniRef50_Q8VZK9	729	53.62	552	234	8	94	1737	18	551	0.0	582
ABCDEF_40982	UniRef50_Q96301	0.0	Normal: 1037	706	819	1249	1084	920	BlastAnno: 3418	UniRef50_Q96301	914	79.60	902	173	3	243	2948	1	891	0.0	1506
ABCDEF_47674	UniRef50_Q9LHZ7	0.0	Normal: 274	92	202	599	505	280	BlastAnno: 2526	UniRef50_Q9LHZ7	1170	75.93	777	151	8	2260	2	15	779	0.0	1177
ABCDEF_34097	UniRef50_Q9LE59	0.0	Normal: 626	613	923	1797	1829	692	BlastAnno: 2521	UniRef50_Q9LE59	673	60.92	458	178	1	1687	317	215	672	0.0	630
ABCDEF_34098	UniRef50_Q9LE59	0.0	Normal: 457	464	697	1283	1312	539	BlastAnno: 2199	UniRef50_Q9LE59	673	60.92	458	178	1	1687	317	215	672	0.0	628
ABCDEF_10848	UniRef50_Q9M156	0.0	Normal: 157	2988	163	408	772	1566	BlastAnno: 1810	UniRef50_Q9M156	480	60.09	456	176	4	1700	336	4	454	0.0	553
ABCDEF_41182	UniRef50_Q9LHZ7	0.0	Normal: 150	2169	221	814	1407	495	BlastAnno: 3604	UniRef50_Q9LHZ7	1170	51.03	870	333	15	2822	456	299	1156	0.0	826
ABCDEF_40981	UniRef50_Q96301	0.0	Normal: 156	97	110	160	179	173	BlastAnno: 1225	UniRef50_Q96301	914	86.92	344	45	0	1112	81	394	737	0.0	652
ABCDEF_9506	UniRef50_Q9LHZ7	0.0	Normal: 1302	2128	1494	3356	4644	2206	BlastAnno: 3751	UniRef50_Q9LHZ7	1170	49.38	891	343	17	927	3359	299	1161	0.0	812
ABCDEF_9500	UniRef50_Q9LHZ7	0.0	Normal: 183	188	186	576	498	260	BlastAnno: 1717	UniRef50_Q9LHZ7	1170	47.98	644	234	14	3	1715	418	1033	0.0	571
ABCDEF_100	UniRef50_Q9M8Y0	0.0	Normal: 228	309	224	416	461	141	BlastAnno: 2109	UniRef50_Q9M8Y0	977	77.49	533	120	0	1610	12	50	582	0.0	884
ABCDEF_33659	UniRef50_Q9LHZ7	0.0	Normal: 1282	1948	2248	5826	10642	2083	BlastAnno: 4289	UniRef50_Q9LHZ7	1170	48.70	887	345	15	2955	538	299	1156	0.0	788
ABCDEF_37667	UniRef50_Q9LHZ7	0.0	Normal: 248	3617	251	1237	2078	970	BlastAnno: 3161	UniRef50_Q9LHZ7	1170	49.72	885	335	14	2671	260	301	1156	0.0	810
ABCDEF_47673	UniRef50_Q9LHZ7	0.0	Normal: 876	296	828	1628	1721	1172	BlastAnno: 3878	UniRef50_Q9LHZ7	1170	80.88	1161	186	8	3642	232	15	1163	0.0	1865
ABCDEF_99	UniRef50_Q9M8Y0	0.0	Normal: 272	330	222	398	423	145	BlastAnno: 2094	UniRef50_Q9M8Y0	977	77.36	561	127	0	1683	1	50	610	0.0	929
ABCDEF_20658	UniRef50_Q8VZK9	0.0	Normal: 373	33	193	64	503	141	BlastAnno: 2316	UniRef50_Q8VZK9	729	53.35	508	221	7	47	1564	11	504	0.0	559
ABCDEF_10183	UniRef50_A7XDQ9	0.0	Normal: 422	455	346	577	378	610	BlastAnno: 2362	UniRef50_A7XDQ9	684	50.94	693	307	12	281	2287	1	684	0.0	670
ABCDEF_30745	UniRef50_A7XDQ9	0.0	Normal: 1209	351	1031	1645	1226	635	BlastAnno: 2646	UniRef50_A7XDQ9	684	57.58	554	227	6	546	2189	133	684	0.0	664
ABCDEF_13336	UniRef50_Q7PC69	0.0	Normal: 148	563	194	957	1575	816	BlastAnno: 2584	UniRef50_Q7PC69	745	71.56	559	136	6	744	2351	187	745	0.0	730
ABCDEF_21426	UniRef50_Q8RU27	0.0	Normal: 2658	1979	3417	6157	9464	2599	BlastAnno: 1530	UniRef50_Q8RU27	366	89.44	360	34	2	1358	282	1	357	0.0	667
ABCDEF_71242	UniRef50_Q9ZV71	0.0	Normal: 134	122	179	480	754	119	BlastAnno: 1835	UniRef50_Q9ZV71	409	73.35	409	103	4	231	1439	1	409	0.0	587
ABCDEF_52020	UniRef50_Q6YWK8	0.0	Normal: 1635	1534	1975	4284	10353	2289	BlastAnno: 2394	UniRef50_Q6YWK8	570	58.37	514	185	2	403	1857	57	570	0.0	607
ABCDEF_51269	UniRef50_Q6YWK8	0.0	Normal: 147	85	103	21	33	438	BlastAnno: 1864	UniRef50_Q6YWK8	570	54.28	514	204	2	1646	198	57	570	0.0	586
ABCDEF_30753	UniRef50_Q9ZV71	0.0	Normal: 290	497	417	629	1013	634	BlastAnno: 1502	UniRef50_Q9ZV71	409	72.32	401	107	2	311	1501	1	401	0.0	590
ABCDEF_4233	UniRef50_Q9ZV71	0.0	Normal: 369	412	550	936	860	750	BlastAnno: 2004	UniRef50_Q9ZV71	409	72.13	409	110	2	370	1584	1	409	0.0	613
ABCDEF_17525	UniRef50_Q8RU27	0.0	Normal: 1500	1289	2149	3711	4446	1069	BlastAnno: 1507	UniRef50_Q8RU27	366	91.35	347	30	0	1371	331	3	349	0.0	671
ABCDEF_18005	UniRef50_Q6YWK8	0.0	Normal: 1842	2042	2515	4214	5208	2842	BlastAnno: 2305	UniRef50_Q6YWK8	570	59.14	514	180	2	1948	497	57	570	0.0	642
ABCDEF_41140	UniRef50_Q6YWK8	0.0	Normal: 86	1314	93	753	1787	2076	BlastAnno: 2346	UniRef50_Q6YWK8	570	60.00	510	174	2	431	1870	57	566	0.0	669
ABCDEF_18002	UniRef50_Q9LHZ7	0.0	Normal: 376	127	367	601	1190	520	BlastAnno: 1605	UniRef50_Q9LHZ7	1170	89.75	400	41	0	1603	404	771	1170	0.0	732
ABCDEF_13339	UniRef50_Q7PC69	0.0	Normal: 127	814	183	541	1135	1144	BlastAnno: 2610	UniRef50_Q7PC69	745	70.48	559	141	6	725	2329	187	745	0.0	729
ABCDEF_41141	UniRef50_Q6YWK8	0.0	Normal: 67	1107	84	708	1690	1854	BlastAnno: 2461	UniRef50_Q6YWK8	570	60.00	510	174	2	1916	477	57	566	0.0	668
ABCDEF_44583	UniRef50_Q9LE59	0.0	Normal: 681	561	559	947	1745	789	BlastAnno: 2470	UniRef50_Q9LE59	673	59.21	456	185	1	1749	385	214	669	0.0	621
ABCDEF_3869	UniRef50_Q9LE59	0.0	Normal: 333	393	415	809	907	754	BlastAnno: 2136	UniRef50_Q9LE59	673	59.05	613	227	4	1827	1	81	673	0.0	699
ABCDEF_28947	UniRef50_Q9SK82	0.0	Normal: 0	851	3	0	0	116	BlastAnno: 1567	UniRef50_Q9SK82	489	62.40	484	176	4	1524	85	1	482	0.0	652
ABCDEF_31119	UniRef50_Q9M8Y0	0.0	Normal: 2212	1279	1917	2933	3341	1803	BlastAnno: 3695	UniRef50_Q9M8Y0	977	81.08	925	174	1	392	3163	50	974	0.0	1583
ABCDEF_37311	UniRef50_Q9LE59	0.0	Normal: 175	133	191	549	421	366	BlastAnno: 1612	UniRef50_Q9LE59	673	62.96	486	153	4	1406	3	4	480	0.0	542
ABCDEF_24190	UniRef50_Q8L7F9	0.0	Normal: 397	265	492	583	553	371	BlastAnno: 2564	UniRef50_Q8L7F9	643	63.91	640	218	4	2353	473	1	640	0.0	850
ABCDEF_30548	UniRef50_Q9FH36	0.0	Normal: 17	916	27	151	420	126	BlastAnno: 1963	UniRef50_Q9FH36	535	72.47	534	138	3	102	1676	1	534	0.0	767
ABCDEF_27416	UniRef50_Q7PC69	0.0	Normal: 575	837	875	1344	2015	936	BlastAnno: 2454	UniRef50_Q7PC69	745	66.11	416	115	4	2337	1168	126	541	0.0	518
ABCDEF_48373	UniRef50_A7XDQ9	0.0	Normal: 1364	773	1336	1451	1791	1202	BlastAnno: 3042	UniRef50_A7XDQ9	684	63.68	691	234	6	2405	360	1	683	0.0	915
ABCDEF_3868	UniRef50_Q9LE59	0.0	Normal: 224	274	301	555	686	524	BlastAnno: 1670	UniRef50_Q9LE59	673	65.31	516	176	1	1556	9	161	673	0.0	696
ABCDEF_31288	UniRef50_Q940V3	0.0	Normal: 247	309	183	101	124	655	BlastAnno: 1658	UniRef50_Q940V3	470	56.01	466	197	7	1614	232	8	470	0.0	537
ABCDEF_52574	UniRef50_Q09332	0.0	Normal: 885	474	1068	1835	1853	816	BlastAnno: 3720	UniRef50_Q09332	1548	47.89	687	310	14	2551	533	859	1511	0.0	598
ABCDEF_79752	UniRef50_Q9ZV71	0.0	Normal: 158	183	245	400	379	352	BlastAnno: 1266	UniRef50_Q9ZV71	409	71.39	360	99	2	31	1098	1	360	0.0	526
ABCDEF_9509	UniRef50_Q9LHZ7	0.0	Normal: 1296	2171	1498	3410	4683	2232	BlastAnno: 3897	UniRef50_Q9LHZ7	1170	49.38	891	343	17	1073	3505	299	1161	0.0	812
ABCDEF_48200	UniRef50_Q6YWK8	0.0	Normal: 1768	576	3156	4431	6634	491	BlastAnno: 2274	UniRef50_Q6YWK8	570	57.98	514	185	4	291	1739	57	570	0.0	642
ABCDEF_24155	UniRef50_Q9ZVI7	0.0	Normal: 503	247	607	1676	1833	578	BlastAnno: 2708	UniRef50_Q9ZVI7	619	52.33	621	264	11	306	2123	15	618	0.0	601
ABCDEF_21246	UniRef50_Q9SKT6	0.0	Normal: 92	107	188	380	834	84	BlastAnno: 1791	UniRef50_Q9SKT6	536	56.61	484	204	5	123	1562	52	533	0.0	568
ABCDEF_32317	UniRef50_Q8RU27	0.0	Normal: 12	9	3	118	800	62	BlastAnno: 1076	UniRef50_Q8RU27	366	87.02	285	37	0	1075	221	64	348	0.0	535
ABCDEF_9502	UniRef50_Q9LHZ7	0.0	Normal: 610	486	568	1331	1403	591	BlastAnno: 1961	UniRef50_Q9LHZ7	1170	48.64	625	249	13	3	1742	564	1161	0.0	570
ABCDEF_23298	UniRef50_Q9FH36	0.0	Normal: 128	527	130	223	314	864	BlastAnno: 2832	UniRef50_Q9FH36	535	83.16	196	33	0	696	1283	340	535	0.0	357

ABCDEF_40595	UniRef50_O80898	0.0	Normal: 229	135	103	25	307	325	BlastAnno: 2413	UniRef50_O80898	757	44.68	761	378	13	95	2299	10	753	0.0	587
ABCDEF_63947	UniRef50_Q9SK82	0.0	Normal: 0	267	0	0	71	0	BlastAnno: 1446	UniRef50_Q9SK82	489	58.33	468	189	4	60	1445	1	468	0.0	592
ABCDEF_67847	UniRef50_Q9M8Y0	0.0	Normal: 794	476	871	1081	1401	868	BlastAnno: 1591	UniRef50_Q9M8Y0	977	84.86	370	56	0	1589	480	605	974	0.0	685
ABCDEF_65666	UniRef50_Q09332	0.0	Normal: 800	441	946	1606	1637	735	BlastAnno: 3518	UniRef50_Q09332	1548	47.54	671	305	13	1162	3087	859	1511	0.0	586
ABCDEF_38641	UniRef50_Q9FWA4	0.0	Normal: 674	967	1162	2603	4404	2475	BlastAnno: 1599	UniRef50_Q9FWA4	561	81.35	386	72	0	1	1158	176	561	0.0	641
ABCDEF_17914	UniRef50_Q9SKT6	0.0	Normal: 784	567	886	1447	1439	1424	BlastAnno: 2049	UniRef50_Q9SKT6	536	72.95	536	140	2	204	1796	1	536	0.0	785
ABCDEF_18286	UniRef50_Q6NQB7	0.0	Normal: 1131	1108	1225	1872	1722	3329	BlastAnno: 1752	UniRef50_Q6NQB7	393	73.80	397	81	6	1527	367	10	393	0.0	607
ABCDEF_119	UniRef50_Q8RU27	0.0	Normal: 1613	1155	2249	3558	7663	1775	BlastAnno: 1532	UniRef50_Q8RU27	366	88.33	360	38	2	1306	230	1	357	0.0	663
ABCDEF_47603	UniRef50_Q9M9Y5	0.0	Normal: 2609	8779	1664	1103	2085	22991	BlastAnno: 2830	UniRef50_Q9M9Y5	589	54.04	594	267	3	2475	694	1	588	0.0	654
ABCDEF_24192	UniRef50_Q8L7F9	0.0	Normal: 391	244	465	553	497	345	BlastAnno: 2477	UniRef50_Q8L7F9	643	58.99	434	165	4	212	1474	1	434	0.0	513
ABCDEF_17482	UniRef50_Q9SKT6	0.0	Normal: 615	379	816	1068	1470	852	BlastAnno: 2083	UniRef50_Q9SKT6	536	71.11	540	147	4	1915	311	1	536	0.0	786
ABCDEF_85909	UniRef50_Q8VZK9	0.0	Normal: 28	45	9	10	44	127	BlastAnno: 1473	UniRef50_Q8VZK9	729	55.37	475	198	6	1419	1	11	473	0.0	536
ABCDEF_9503	UniRef50_Q9LHZ7	0.0	Normal: 487	544	524	1242	1320	833	BlastAnno: 2087	UniRef50_Q9LHZ7	1170	50.35	574	236	8	57	1712	615	1161	0.0	567
ABCDEF_40594	UniRef50_O80898	0.0	Normal: 256	127	102	41	331	326	BlastAnno: 2417	UniRef50_O80898	757	45.76	719	348	12	213	2291	51	753	0.0	585
ABCDEF_17141	UniRef50_Q9LHZ7	0.0	Normal: 74	1734	69	380	701	333	BlastAnno: 3337	UniRef50_Q9LHZ7	1170	50.92	868	335	14	2539	179	299	1156	0.0	816
ABCDEF_33658	UniRef50_Q9LHZ7	0.0	Normal: 384	308	395	805	1003	602	BlastAnno: 2961	UniRef50_Q9LHZ7	1170	48.76	888	343	14	204	2621	299	1156	0.0	792
ABCDEF_20579	UniRef50_Q7PC69	0.0	Normal: 1007	795	1238	2311	2309	1345	BlastAnno: 3113	UniRef50_Q7PC69	745	59.97	767	229	14	2667	535	1	745	0.0	768
ABCDEF_31118	UniRef50_Q9M8Y0	0.0	Normal: 2135	1267	1900	3004	3329	1757	BlastAnno: 3787	UniRef50_Q9M8Y0	977	81.10	709	133	1	1132	3255	266	974	0.0	1217
ABCDEF_9499	UniRef50_Q9LHZ7	0.0	Normal: 205	207	215	632	538	275	BlastAnno: 1794	UniRef50_Q9LHZ7	1170	48.88	667	240	14	11	1792	395	1033	0.0	605
ABCDEF_48199	UniRef50_Q6YWK8	0.0	Normal: 1506	467	2429	3094	4233	439	BlastAnno: 1997	UniRef50_Q6YWK8	570	63.10	439	146	2	291	1559	57	495	0.0	610
ABCDEF_40983	UniRef50_Q96301	0.0	Normal: 406	261	343	392	415	433	BlastAnno: 2265	UniRef50_Q96301	914	79.17	504	99	1	114	1625	394	891	0.0	854
ABCDEF_17140	UniRef50_Q9LHZ7	0.0	Normal: 45	1049	49	216	386	208	BlastAnno: 2293	UniRef50_Q9LHZ7	1170	52.34	770	281	13	2138	57	397	1156	0.0	739
ABCDEF_18352	UniRef50_Q9FWA4	0.0	Normal: 2427	2688	2969	5519	6547	4855	BlastAnno: 2224	UniRef50_Q9FWA4	561	69.31	505	148	3	1887	382	61	561	0.0	683
ABCDEF_27767	UniRef50_Q9LHZ7	0.0	Normal: 2576	5141	2841	10249	14583	5031	BlastAnno: 3981	UniRef50_Q9LHZ7	1170	49.66	888	337	16	2927	504	299	1156	0.0	818
ABCDEF_33661	UniRef50_Q9LHZ7	0.0	Normal: 414	340	507	879	790	783	BlastAnno: 2185	UniRef50_Q9LHZ7	1170	48.66	748	277	14	2184	169	440	1156	0.0	662
ABCDEF_14685	UniRef50_A7XDQ9	0.0	Normal: 233	308	134	573	292	334	BlastAnno: 1947	UniRef50_A7XDQ9	684	56.84	468	186	6	569	1945	1	461	0.0	545
ABCDEF_20660	UniRef50_Q8VZK9	0.0	Normal: 22	48	13	15	52	144	BlastAnno: 1473	UniRef50_Q8VZK9	729	55.16	475	199	6	55	1473	11	473	0.0	537
ABCDEF_21315	UniRef50_Q9LHZ7	0.0	Normal: 3412	252	1450	0	79	1228	BlastAnno: 3765	UniRef50_Q9LHZ7	1170	63.78	1096	340	15	308	3508	70	1137	0.0	1333
ABCDEF_533	UniRef50_Q9C8W3	0.0	Normal: 230	204	269	505	411	301	BlastAnno: 1688	UniRef50_Q9C8W3	401	70.33	364	94	2	393	1475	47	399	0.0	555
ABCDEF_34099	UniRef50_Q9LE59	0.0	Normal: 478	491	735	1356	1350	548	BlastAnno: 2158	UniRef50_Q9LE59	673	60.92	458	178	1	1610	240	215	672	0.0	630
ABCDEF_47984	UniRef50_Q7PC69	0.0	Normal: 663	457	734	1362	2147	696	BlastAnno: 2838	UniRef50_Q7PC69	745	59.27	712	226	9	373	2316	34	745	0.0	702
ABCDEF_51451	UniRef50_Q9LE59	0.0	Normal: 1883	1782	2202	4337	4350	2667	BlastAnno: 2874	UniRef50_Q9LE59	673	74.16	685	165	6	2559	505	1	673	0.0	982
ABCDEF_13370	UniRef50_Q8VZK9	0.0	Normal: 290	87	317	377	105	258	BlastAnno: 2033	UniRef50_Q8VZK9	729	51.65	635	278	11	2031	154	112	726	0.0	612
ABCDEF_48239	UniRef50_Q9LHZ7	0.0	Normal: 511	163	422	1154	1316	510	BlastAnno: 2714	UniRef50_Q9LHZ7	1170	76.20	769	156	9	478	2712	41	806	0.0	1201
ABCDEF_13338	UniRef50_Q7PC69	0.0	Normal: 335	468	417	2639	4443	616	BlastAnno: 2525	UniRef50_Q7PC69	745	62.45	727	209	12	298	2292	21	745	0.0	781
ABCDEF_24193	UniRef50_Q8L7F9	0.0	Normal: 113	72	132	203	180	119	BlastAnno: 1679	UniRef50_Q8L7F9	643	60.54	479	176	4	212	1609	1	479	0.0	592
ABCDEF_48514	UniRef50_Q9ZV71	0.0	Normal: 540	580	601	771	1033	926	BlastAnno: 1920	UniRef50_Q9ZV71	409	78.14	398	85	2	1618	431	12	409	0.0	637

>00523 Polyketide sugar unit biosynthesis

1.1.1.-(Oxidoreductases;)

ABCDEF_13453	UniRef50_Q9SYM5	0.0	Normal: 225	226	398	1950	2026	421	BlastAnno: 2578	UniRef50_Q9SYM5	669	86.61	672	87	2	298	2313	1	669	0.0	1226
ABCDEF_17342	UniRef50_Q84M96	0.0	Normal: 274	406	272	327	439	625	BlastAnno: 1277	UniRef50_Q84M96	346	73.33	345	91	1	1187	156	2	346	0.0	546
ABCDEF_31147	UniRef50_Q9SYM5	0.0	Normal: 4571	1591	1491	1401	1939	4327	BlastAnno: 2465	UniRef50_Q9SYM5	669	82.76	673	107	4	2397	394	1	669	0.0	1154
ABCDEF_17344	UniRef50_Q84M96	0.0	Normal: 252	373	257	297	380	540	BlastAnno: 1350	UniRef50_Q84M96	346	68.19	371	91	2	1263	154	2	346	0.0	530
ABCDEF_44674	UniRef50_Q9SYM5	0.0	Normal: 3581	2620	4329	9505	11447	4930	BlastAnno: 2634	UniRef50_Q9SYM5	669	87.80	672	78	3	253	2265	1	669	0.0	1241

1.1.1.133(dTDP-4-dehydrorhamnose reductase;)

No Correspond Unigene

1.1.1.266(dTDP-4-dehydro-6-deoxyglucose reductase;)

No Correspond Unigene

2.6.1.33(dTDP-4-amino-4,6-dideoxy-D-glucose transaminase;)

No Correspond Unigene

2.7.7.24(glucose-1-phosphate thymidylyltransferase;)

No Correspond Unigene

4.2.1.46(dTDP-glucose 4,6-dehydratase;)

ABCDEF_13453	UniRef50_O95455	1e-100	Normal: 225	226	398	1950	2026	421	BlastAnno: 2578	UniRef50_O95455	350	48.21	336	165	3	316	1308	18	349	1e-100	325

5.1.3.-(Isomerases;)

No Correspond Unigene

5.1.3.13(dTDP-4-dehydrorhamnose 3,5-epimerase;)

No Correspond Unigene

>00524 Butirosin and neomycin biosynthesis

1.1.-.-(Oxidoreductases;)

ABCDEF_17646	UniRef50_O34948	5e-83	Normal: 372	339	320	294	487	1376	BlastAnno: 1685	UniRef50_O34948	288	47.31	279	145	2	1523	690	5	282	5e-83	269

1.1.1.-(Oxidoreductases;)

ABCDEF_13453	UniRef50_Q9SYM5	0.0	Normal: 225	226	398	1950	2026	421	BlastAnno: 2578	UniRef50_Q9SYM5	669	86.61	672	87	2	298	2313	1	669	0.0	1226
ABCDEF_17342	UniRef50_Q84M96	0.0	Normal: 274	406	272	327	439	625	BlastAnno: 1277	UniRef50_Q84M96	346	73.33	345	91	1	1187	156	2	346	0.0	546
ABCDEF_31147	UniRef50_Q9SYM5	0.0	Normal: 4571	1591	1491	1401	1939	4327	BlastAnno: 2465	UniRef50_Q9SYM5	669	82.76	673	107	4	2397	394	1	669	0.0	1154
ABCDEF_17344	UniRef50_Q84M96	0.0	Normal: 252	373	257	297	380	540	BlastAnno: 1350	UniRef50_Q84M96	346	68.19	371	91	2	1263	154	2	346	0.0	530
ABCDEF_44674	UniRef50_Q9SYM5	0.0	Normal: 3581	2620	4329	9505	11447	4930	BlastAnno: 2634	UniRef50_Q9SYM5	669	87.80	672	78	3	253	2265	1	669	0.0	1241

1.1.3.-(Oxidoreductases;)

No Correspond Unigene

1.14.14.-(Oxidoreductases;)

No Correspond Unigene

1.5.1.29(FMN reductase;)

ABCDEF_45774	UniRef50_B0EI57	0.0	Normal: 24227	11521	19203	23125	37117	17927	BlastAnno: 3112	UniRef50_B0EI57	970	54.60	837	315	5	2894	390	197	970	0.0	919
2.3.2.-(Transferases;)																					
ABCDEF_41698	UniRef50_Q9M8T3	7e-62	Normal: 110	102	151	190	213	210	BlastAnno: 870	UniRef50_Q9M8T3	182	58.29	187	63	4	740	210	1	182	7e-62	202
2.4.1.-(Transferases;)																					
ABCDEF_18004	UniRef50_Q6YWK8	0.0	Normal: 1562	1736	2009	3452	4438	2400	BlastAnno: 2298	UniRef50_Q6YWK8	570	65.27	429	136	1	1941	694	57	485	0.0	608
ABCDEF_52379	UniRef50_Q9LHZ7	0.0	Normal: 4015	4921	4546	10520	13430	4731	BlastAnno: 3479	UniRef50_Q9LHZ7	1170	48.04	893	342	16	825	3242	299	1156	0.0	787
ABCDEF_48593	UniRef50_Q9SK82	0.0	Normal: 12	420	19	343	763	253	BlastAnno: 1834	UniRef50_Q9SK82	489	60.08	481	186	4	1714	278	10	486	0.0	633
ABCDEF_13375	UniRef50_Q8VZK9	0.0	Normal: 108	123	208	526	34	294	BlastAnno: 1738	UniRef50_Q8VZK9	729	53.62	552	234	8	94	1737	18	551	0.0	582
ABCDEF_40982	UniRef50_Q96301	0.0	Normal: 1037	706	819	1249	1084	920	BlastAnno: 3418	UniRef50_Q96301	914	79.60	902	173	3	243	2948	1	891	0.0	1506
ABCDEF_47674	UniRef50_Q9LHZ7	0.0	Normal: 274	92	202	599	505	280	BlastAnno: 2526	UniRef50_Q9LHZ7	1170	75.93	777	151	8	2260	2	15	779	0.0	1177
ABCDEF_34097	UniRef50_Q9LE59	0.0	Normal: 626	613	923	1797	1829	692	BlastAnno: 2521	UniRef50_Q9LE59	673	60.92	458	178	1	1687	317	215	672	0.0	630
ABCDEF_34098	UniRef50_Q9LE59	0.0	Normal: 457	464	697	1283	1312	539	BlastAnno: 2199	UniRef50_Q9LE59	673	60.92	458	178	1	1687	317	215	672	0.0	628
ABCDEF_10848	UniRef50_Q9M156	0.0	Normal: 157	2988	163	408	772	1566	BlastAnno: 1810	UniRef50_Q9M156	480	60.09	456	176	4	1700	336	4	454	0.0	553
ABCDEF_41182	UniRef50_Q9LHZ7	0.0	Normal: 150	2169	221	814	1407	495	BlastAnno: 3604	UniRef50_Q9LHZ7	1170	51.03	870	333	15	2822	456	299	1156	0.0	826
ABCDEF_40981	UniRef50_Q96301	0.0	Normal: 156	97	110	160	179	173	BlastAnno: 1225	UniRef50_Q96301	914	86.92	344	45	0	1112	81	394	737	0.0	652
ABCDEF_9506	UniRef50_Q9LHZ7	0.0	Normal: 1302	2128	1494	3356	4644	2206	BlastAnno: 3751	UniRef50_Q9LHZ7	1170	49.38	891	343	17	927	3359	299	1161	0.0	812
ABCDEF_9500	UniRef50_Q9LHZ7	0.0	Normal: 183	188	186	576	498	260	BlastAnno: 1717	UniRef50_Q9LHZ7	1170	47.98	644	234	14	3	1715	418	1033	0.0	571
ABCDEF_100	UniRef50_Q9M8Y0	0.0	Normal: 228	309	224	416	461	141	BlastAnno: 2109	UniRef50_Q9M8Y0	977	77.49	533	120	0	1610	12	50	582	0.0	884
ABCDEF_33659	UniRef50_Q9LHZ7	0.0	Normal: 1282	1948	2248	5826	10642	2083	BlastAnno: 4289	UniRef50_Q9LHZ7	1170	48.70	887	345	15	2955	538	299	1156	0.0	788
ABCDEF_37667	UniRef50_Q9LHZ7	0.0	Normal: 248	3617	251	1237	2078	970	BlastAnno: 3161	UniRef50_Q9LHZ7	1170	49.72	885	335	14	2671	260	301	1156	0.0	810
ABCDEF_47673	UniRef50_Q9LHZ7	0.0	Normal: 876	296	828	1628	1721	1172	BlastAnno: 3878	UniRef50_Q9LHZ7	1170	80.88	1161	186	8	3642	232	15	1163	0.0	1865
ABCDEF_99	UniRef50_Q9M8Y0	0.0	Normal: 272	330	222	398	423	145	BlastAnno: 2094	UniRef50_Q9M8Y0	977	77.36	561	127	0	1683	1	50	610	0.0	929
ABCDEF_20658	UniRef50_Q8VZK9	0.0	Normal: 373	33	193	64	503	141	BlastAnno: 2316	UniRef50_Q8VZK9	729	53.35	508	221	7	47	1564	11	504	0.0	559
ABCDEF_10183	UniRef50_A7XDQ9	0.0	Normal: 422	455	346	577	378	610	BlastAnno: 2362	UniRef50_A7XDQ9	684	50.94	693	307	12	281	2287	1	684	0.0	670
ABCDEF_30745	UniRef50_A7XDQ9	0.0	Normal: 1209	351	1031	1645	1226	635	BlastAnno: 2646	UniRef50_A7XDQ9	684	57.58	554	227	6	546	2189	133	684	0.0	664
ABCDEF_13336	UniRef50_Q7PC69	0.0	Normal: 148	563	194	957	1575	816	BlastAnno: 2584	UniRef50_Q7PC69	745	71.56	559	136	6	744	2351	187	745	0.0	730
ABCDEF_21426	UniRef50_Q8RU27	0.0	Normal: 2658	1979	3417	6157	9464	2599	BlastAnno: 1530	UniRef50_Q8RU27	366	89.44	360	34	2	1358	282	1	357	0.0	667
ABCDEF_71242	UniRef50_Q9ZV71	0.0	Normal: 134	122	179	480	754	119	BlastAnno: 1835	UniRef50_Q9ZV71	409	73.35	409	103	4	231	1439	1	409	0.0	587
ABCDEF_52020	UniRef50_Q6YWK8	0.0	Normal: 1635	1534	1975	4284	10353	2289	BlastAnno: 2394	UniRef50_Q6YWK8	570	58.37	514	185	2	403	1857	57	570	0.0	607
ABCDEF_51269	UniRef50_Q6YWK8	0.0	Normal: 147	85	103	21	33	438	BlastAnno: 1864	UniRef50_Q6YWK8	570	54.28	514	204	2	1646	198	57	570	0.0	586
ABCDEF_30753	UniRef50_Q9ZV71	0.0	Normal: 290	497	417	629	1013	634	BlastAnno: 1502	UniRef50_Q9ZV71	409	72.32	401	107	2	311	1501	1	401	0.0	590
ABCDEF_4233	UniRef50_Q9ZV71	0.0	Normal: 369	412	550	936	860	750	BlastAnno: 2004	UniRef50_Q9ZV71	409	72.13	409	110	2	370	1584	1	409	0.0	613
ABCDEF_17525	UniRef50_Q8RU27	0.0	Normal: 1500	1289	2149	3711	4446	1069	BlastAnno: 1507	UniRef50_Q8RU27	366	91.35	347	30	0	1371	331	3	349	0.0	671
ABCDEF_18005	UniRef50_Q6YWK8	0.0	Normal: 1842	2042	2515	4214	5208	2842	BlastAnno: 2305	UniRef50_Q6YWK8	570	59.14	514	180	2	1948	497	57	570	0.0	642
ABCDEF_41140	UniRef50_Q6YWK8	0.0	Normal: 86	1314	93	753	1787	2076	BlastAnno: 2346	UniRef50_Q6YWK8	570	60.00	510	174	2	431	1870	57	566	0.0	669
ABCDEF_18002	UniRef50_Q9LHZ7	0.0	Normal: 376	127	367	601	1190	520	BlastAnno: 1605	UniRef50_Q9LHZ7	1170	89.75	400	41	0	1603	404	771	1170	0.0	732
ABCDEF_13339	UniRef50_Q7PC69	0.0	Normal: 127	814	183	541	1135	1144	BlastAnno: 2610	UniRef50_Q7PC69	745	70.48	559	141	6	725	2329	187	745	0.0	729
ABCDEF_41141	UniRef50_Q6YWK8	0.0	Normal: 67	1107	84	708	1690	1854	BlastAnno: 2461	UniRef50_Q6YWK8	570	60.00	510	174	2	1916	477	57	566	0.0	668
ABCDEF_44583	UniRef50_Q9LE59	0.0	Normal: 681	561	559	947	1745	789	BlastAnno: 2470	UniRef50_Q9LE59	673	59.21	456	185	1	1749	385	214	669	0.0	621
ABCDEF_3869	UniRef50_Q9LE59	0.0	Normal: 333	393	415	809	907	754	BlastAnno: 2136	UniRef50_Q9LE59	673	59.05	613	227	4	1827	1	81	673	0.0	699
ABCDEF_28947	UniRef50_Q9SK82	0.0	Normal: 0	851	3	0	0	116	BlastAnno: 1567	UniRef50_Q9SK82	489	62.40	484	176	4	1524	85	1	482	0.0	652
ABCDEF_31119	UniRef50_Q9M8Y0	0.0	Normal: 2212	1279	1917	2933	3341	1803	BlastAnno: 3695	UniRef50_Q9M8Y0	977	81.08	925	174	1	392	3163	50	974	0.0	1583
ABCDEF_37311	UniRef50_Q9LE59	0.0	Normal: 175	133	191	549	421	366	BlastAnno: 1612	UniRef50_Q9LE59	673	62.96	486	153	4	1406	3	4	480	0.0	542
ABCDEF_24190	UniRef50_Q8L7F9	0.0	Normal: 397	265	492	583	553	371	BlastAnno: 2564	UniRef50_Q8L7F9	643	63.91	640	218	4	2353	473	1	640	0.0	850
ABCDEF_30548	UniRef50_Q9FH36	0.0	Normal: 17	916	27	151	420	126	BlastAnno: 1963	UniRef50_Q9FH36	535	72.47	534	138	3	102	1676	1	534	0.0	767
ABCDEF_27416	UniRef50_Q7PC69	0.0	Normal: 575	837	875	1344	2015	936	BlastAnno: 2454	UniRef50_Q7PC69	745	66.11	416	115	4	2337	1168	126	541	0.0	518
ABCDEF_48373	UniRef50_A7XDQ9	0.0	Normal: 1364	773	1336	1451	1791	1202	BlastAnno: 3042	UniRef50_A7XDQ9	684	63.68	691	234	6	2405	360	1	683	0.0	915
ABCDEF_3868	UniRef50_Q9LE59	0.0	Normal: 224	274	301	555	686	524	BlastAnno: 1670	UniRef50_Q9LE59	673	65.31	516	176	1	1556	9	161	673	0.0	696
ABCDEF_31288	UniRef50_Q940V3	0.0	Normal: 247	309	183	101	124	655	BlastAnno: 1658	UniRef50_Q940V3	470	56.01	466	197	7	1614	232	8	470	0.0	537
ABCDEF_52574	UniRef50_Q09332	0.0	Normal: 885	474	1068	1835	1853	816	BlastAnno: 3720	UniRef50_Q09332	1548	47.89	687	310	14	2551	533	859	1511	0.0	598
ABCDEF_79752	UniRef50_Q9ZV71	0.0	Normal: 158	183	245	400	379	352	BlastAnno: 1266	UniRef50_Q9ZV71	409	71.39	360	99	2	31	1098	1	360	0.0	526
ABCDEF_9509	UniRef50_Q9LHZ7	0.0	Normal: 1296	2171	1498	3410	4683	2232	BlastAnno: 3897	UniRef50_Q9LHZ7	1170	49.38	891	343	17	1073	3505	299	1161	0.0	812
ABCDEF_48200	UniRef50_Q6YWK8	0.0	Normal: 1768	576	3156	4431	6634	491	BlastAnno: 2274	UniRef50_Q6YWK8	570	57.98	514	185	4	291	1739	57	570	0.0	642
ABCDEF_24155	UniRef50_Q9ZVI7	0.0	Normal: 503	247	607	1676	1833	578	BlastAnno: 2708	UniRef50_Q9ZVI7	619	52.33	621	264	11	306	2123	15	618	0.0	601
ABCDEF_21246	UniRef50_Q9SKT6	0.0	Normal: 92	107	188	380	834	84	BlastAnno: 1791	UniRef50_Q9SKT6	536	56.61	484	204	5	123	1562	52	533	0.0	568
ABCDEF_32317	UniRef50_Q8RU27	0.0	Normal: 12	9	3	118	800	62	BlastAnno: 1076	UniRef50_Q8RU27	366	87.02	285	37	0	1075	221	64	348	0.0	535
ABCDEF_9502	UniRef50_Q9LHZ7	0.0	Normal: 610	486	568	1331	1403	591	BlastAnno: 1961	UniRef50_Q9LHZ7	1170	48.64	625	249	13	3	1742	564	1161	0.0	570
ABCDEF_23298	UniRef50_Q9FH36	0.0	Normal: 128	527	130	223	314	864	BlastAnno: 2832	UniRef50_Q9FH36	535	83.16	196	33	0	696	1283	340	535	0.0	357
ABCDEF_40595	UniRef50_O80898	0.0	Normal: 229	135	103	25	307	325	BlastAnno: 2413	UniRef50_O80898	757	44.68	761	378	13	95	2299	10	753	0.0	587
ABCDEF_63947	UniRef50_Q9SK82	0.0	Normal: 0	267	0	0	71	0	BlastAnno: 1446	UniRef50_Q9SK82	489	58.33	468	189	4	60	1445	1	468	0.0	592
ABCDEF_67847	UniRef50_Q9M8Y0	0.0	Normal: 794	476	871	1081	1401	868	BlastAnno: 1591	UniRef50_Q9M8Y0	977	84.86	370	56	0	1589	480	605	974	0.0	685
ABCDEF_65666	UniRef50_Q09332	0.0	Normal: 800	441	946	1606	1637	735	BlastAnno: 3518	UniRef50_Q09332	1548	47.54	671	305	13	1162	3087	859	1511	0.0	586
ABCDEF_38641	UniRef50_Q9FWA4	0.0	Normal: 674	967	1162	2603	4404	2475	BlastAnno: 1599	UniRef50_Q9FWA4	561	81.35	386	72	0	1	1158	176	561	0.0	641
ABCDEF_17914	UniRef50_Q9SKT6	0.0	Normal: 784	567	886	1447	1439	1424	BlastAnno: 2049	UniRef50_Q9SKT6	536	72.95	536	140	2	204	1796	1	536	0.0	785
ABCDEF_18286	UniRef50_Q6NQB7	0.0	Normal: 1131	1108	1225	1872	1722	3329	BlastAnno: 1752	UniRef50_Q6NQB7	393	73.80	397	81	6	1527	367	10	393	0.0	607
ABCDEF_119	UniRef50_Q8RU27	0.0	Normal: 1613	1155	2249	3558	7663	1775	BlastAnno: 1532	UniRef50_Q8RU27	366	88.33	360	38	2	1306	230	1	357	0.0	663
ABCDEF_47603	UniRef50_Q9M9Y5	0.0	Normal: 2609	8779	1664	1103	2085	22991	BlastAnno: 2830	UniRef50_Q9M9Y5	589	54.04	594	267	3	2475	694	1	588	0.0	654
ABCDEF_24192	UniRef50_Q8L7F9	0.0	Normal: 391	244	465	553	497	345	BlastAnno: 2477	UniRef50_Q8L7F9	643	58.99	434	165	4	212	1474	1	434	0.0	513
ABCDEF_17482	UniRef50_Q9SKT6	0.0	Normal: 615	379	816	1068	1470	852	BlastAnno: 2083	UniRef50_Q9SKT6	536	71.11	540	147	4	1915	311	1	536	0.0	786
ABCDEF_85909	UniRef50_Q8VZK9	0.0	Normal: 28	45	9	10	44	127	BlastAnno: 1473	UniRef50_Q8VZK9	729	55.37	475	198	6	1419	1	11	473	0.0	536
ABCDEF_9503	UniRef50_Q9LHZ7	0.0	Normal: 487	544	524	1242	1320	833	BlastAnno: 2087	UniRef50_Q9LHZ7	1170	50.35	574	236	8	57	1712	615	1161	0.0	567
ABCDEF_40594	UniRef50_O80898	0.0	Normal: 256	127	102	41	331	326	BlastAnno: 2417	UniRef50_O80898	757	45.76	719	348	12	213	2291	51	753	0.0	585

ABCDEF_17141	UniRef50_Q9LHZ7	0.0	Normal: 74	1734	69	380	701	333	BlastAnno: 3337	UniRef50_Q9LHZ7	1170	50.92	868	335	14	2539	179	299	1156	0.0	816
ABCDEF_33658	UniRef50_Q9LHZ7	0.0	Normal: 384	308	395	805	1003	602	BlastAnno: 2961	UniRef50_Q9LHZ7	1170	48.76	888	343	14	204	2621	299	1156	0.0	792
ABCDEF_20579	UniRef50_Q7PC69	0.0	Normal: 1007	795	1238	2311	2309	1345	BlastAnno: 3113	UniRef50_Q7PC69	745	59.97	767	229	14	2667	535	1	745	0.0	768
ABCDEF_31118	UniRef50_Q9M8Y0	0.0	Normal: 2135	1267	1900	3004	3329	1757	BlastAnno: 3787	UniRef50_Q9M8Y0	977	81.10	709	133	1	1132	3255	266	974	0.0	1217
ABCDEF_9499	UniRef50_Q9LHZ7	0.0	Normal: 205	207	215	632	538	275	BlastAnno: 1794	UniRef50_Q9LHZ7	1170	48.88	667	240	14	11	1792	395	1033	0.0	605
ABCDEF_48199	UniRef50_Q6YWK8	0.0	Normal: 1506	467	2429	3094	4233	439	BlastAnno: 1997	UniRef50_Q6YWK8	570	63.10	439	146	2	291	1559	57	495	0.0	610
ABCDEF_40983	UniRef50_Q96301	0.0	Normal: 406	261	343	392	415	433	BlastAnno: 2265	UniRef50_Q96301	914	79.17	504	99	1	114	1625	394	891	0.0	854
ABCDEF_17140	UniRef50_Q9LHZ7	0.0	Normal: 45	1049	49	216	386	208	BlastAnno: 2293	UniRef50_Q9LHZ7	1170	52.34	770	281	13	2138	57	397	1156	0.0	739
ABCDEF_18352	UniRef50_Q9FWA4	0.0	Normal: 2427	2688	2969	5519	6547	4855	BlastAnno: 2224	UniRef50_Q9FWA4	561	69.31	505	148	3	1887	382	61	561	0.0	683
ABCDEF_27767	UniRef50_Q9LHZ7	0.0	Normal: 2576	5141	2841	10249	14583	5031	BlastAnno: 3981	UniRef50_Q9LHZ7	1170	49.66	888	337	16	2927	504	299	1156	0.0	818
ABCDEF_33661	UniRef50_Q9LHZ7	0.0	Normal: 414	340	507	879	790	783	BlastAnno: 2185	UniRef50_Q9LHZ7	1170	48.66	748	277	14	2184	169	440	1156	0.0	662
ABCDEF_14685	UniRef50_A7XDQ9	0.0	Normal: 233	308	134	573	292	334	BlastAnno: 1947	UniRef50_A7XDQ9	684	56.84	468	186	6	569	1945	1	461	0.0	545
ABCDEF_20660	UniRef50_Q8VZK9	0.0	Normal: 22	48	13	15	52	144	BlastAnno: 1473	UniRef50_Q8VZK9	729	55.16	475	199	6	55	1473	11	473	0.0	537
ABCDEF_21315	UniRef50_Q9LHZ7	0.0	Normal: 3412	252	1450	0	79	1228	BlastAnno: 3765	UniRef50_Q9LHZ7	1170	63.78	1096	340	15	308	3508	70	1137	0.0	1333
ABCDEF_533	UniRef50_Q9C8W3	0.0	Normal: 230	204	269	505	411	301	BlastAnno: 1688	UniRef50_Q9C8W3	401	70.33	364	94	2	393	1475	47	399	0.0	555
ABCDEF_34099	UniRef50_Q9LE59	0.0	Normal: 478	491	735	1356	1350	548	BlastAnno: 2158	UniRef50_Q9LE59	673	60.92	458	178	1	1610	240	215	672	0.0	630
ABCDEF_47984	UniRef50_Q7PC69	0.0	Normal: 663	457	734	1362	2147	696	BlastAnno: 2838	UniRef50_Q7PC69	745	59.27	712	226	9	373	2316	34	745	0.0	702
ABCDEF_51451	UniRef50_Q9LE59	0.0	Normal: 1883	1782	2202	4337	4350	2667	BlastAnno: 2874	UniRef50_Q9LE59	673	74.16	685	165	6	2559	505	1	673	0.0	982
ABCDEF_13370	UniRef50_Q8VZK9	0.0	Normal: 290	87	317	377	105	258	BlastAnno: 2033	UniRef50_Q8VZK9	729	51.65	635	278	11	2031	154	112	726	0.0	612
ABCDEF_48239	UniRef50_Q9LHZ7	0.0	Normal: 511	163	422	1154	1316	510	BlastAnno: 2714	UniRef50_Q9LHZ7	1170	76.20	769	156	9	478	2712	41	806	0.0	1201
ABCDEF_13338	UniRef50_Q7PC69	0.0	Normal: 335	468	417	2639	4443	616	BlastAnno: 2525	UniRef50_Q7PC69	745	62.45	727	209	12	298	2292	21	745	0.0	781
ABCDEF_24193	UniRef50_Q8L7F9	0.0	Normal: 113	72	132	203	180	119	BlastAnno: 1679	UniRef50_Q8L7F9	643	60.54	479	176	4	212	1609	1	479	0.0	592
ABCDEF_48514	UniRef50_Q9ZV71	0.0	Normal: 540	580	601	771	1033	926	BlastAnno: 1920	UniRef50_Q9ZV71	409	78.14	398	85	2	1618	431	12	409	0.0	637

2.4.2.-(Transferases;)

ABCDEF_17257	UniRef50_Q940Q8	0.0	Normal: 176	142	236	252	196	151	BlastAnno: 1259	UniRef50_Q940Q8	415	91.67	396	33	0	1259	72	20	415	0.0	734
ABCDEF_25833	UniRef50_Q940Q8	0.0	Normal: 28	336	34	93	151	84	BlastAnno: 958	UniRef50_Q940Q8	415	88.89	306	34	0	957	40	110	415	0.0	590
ABCDEF_21016	UniRef50_B8LDL7	0.0	Normal: 775	334	669	635	559	950	BlastAnno: 2086	UniRef50_B8LDL7	570	56.33	561	212	10	1820	234	8	567	0.0	595
ABCDEF_48436	UniRef50_Q940Q8	0.0	Normal: 603	624	571	1077	1211	656	BlastAnno: 1898	UniRef50_Q940Q8	415	90.71	409	37	1	1515	292	7	415	0.0	744

2.6.1.-(Transferases;)

ABCDEF_3716	UniRef50_Q9LQ10	0.0	Normal: 224	353	140	47	76	606	BlastAnno: 2230	UniRef50_Q9LQ10	557	61.35	533	189	8	250	1812	30	557	0.0	609
ABCDEF_37756	UniRef50_Q9LQ10	0.0	Normal: 197	168	149	152	186	490	BlastAnno: 1840	UniRef50_Q9LQ10	557	57.49	534	196	7	1638	109	28	554	0.0	619

2.7.1.1(hexokinase;)

ABCDEF_27834	UniRef50_P93834	0.0	Normal: 998	404	1410	4185	6485	623	BlastAnno: 2374	UniRef50_P93834	502	72.77	470	128	0	2024	615	25	494	0.0	655
ABCDEF_51305	UniRef50_P93834	0.0	Normal: 443	123	280	151	521	245	BlastAnno: 2179	UniRef50_P93834	502	76.47	289	68	0	1813	947	25	313	0.0	467
ABCDEF_420	UniRef50_Q1WM16	0.0	Normal: 453	101	514	402	696	75	BlastAnno: 1554	UniRef50_Q1WM16	463	61.66	433	159	3	3	1295	20	447	0.0	538
ABCDEF_86444	UniRef50_Q1WM16	0.0	Normal: 293	76	186	81	353	207	BlastAnno: 1684	UniRef50_Q1WM16	463	66.83	404	129	3	1404	196	63	462	0.0	530
ABCDEF_592	UniRef50_P93834	0.0	Normal: 378	177	486	1038	1819	341	BlastAnno: 1859	UniRef50_P93834	502	59.87	456	180	3	1618	260	37	492	0.0	537
ABCDEF_83497	UniRef50_Q1WM16	0.0	Normal: 290	84	185	81	350	338	BlastAnno: 1860	UniRef50_Q1WM16	463	66.83	404	129	3	1404	196	63	462	0.0	529
ABCDEF_51306	UniRef50_Q1WM16	0.0	Normal: 446	118	281	150	539	247	BlastAnno: 2108	UniRef50_Q1WM16	463	66.96	451	144	3	1685	336	16	462	0.0	578
ABCDEF_593	UniRef50_P93834	0.0	Normal: 403	191	504	1097	1940	353	BlastAnno: 1902	UniRef50_P93834	502	59.87	456	180	3	1648	290	37	492	0.0	537

2.7.1.2(glucokinase;)

No Correspond Unigene

3.1.3.-(Hydrolases;)

ABCDEF_3128	UniRef50_Q9ZU49	5e-117	Normal: 118	143	108	91	149	56	BlastAnno: 1434	UniRef50_Q9ZU49	327	57.43	303	121	2	1184	294	20	320	5e-117	355

3.5.1.-(Hydrolases;)

ABCDEF_41579	UniRef50_O81641	0.0	Normal: 384	122	265	320	313	150	BlastAnno: 1456	UniRef50_O81641	428	65.16	399	138	1	1313	120	23	421	0.0	543
ABCDEF_16924	UniRef50_Q8VYX0	0.0	Normal: 135	349	460	1325	685	527	BlastAnno: 2002	UniRef50_Q8VYX0	464	71.72	396	109	2	1612	428	68	461	0.0	549
ABCDEF_24290	UniRef50_O04373	0.0	Normal: 296	238	403	498	698	330	BlastAnno: 1710	UniRef50_O04373	440	65.24	443	151	3	67	1395	1	440	0.0	595

4.1.1.-(Lyases;)

ABCDEF_57496	UniRef50_O96569	9e-20	Normal: 4	0	0	0	0	9	BlastAnno: 223	UniRef50_O96569	439	66.67	72	22	1	210	1	19	90	9e-20	87.4

4.2.3.-(Lyases;)

ABCDEF_30616	UniRef50_Q0JA83	0.0	Normal: 514	201	395	484	549	320	BlastAnno: 3023	UniRef50_Q0JA83	739	47.55	736	339	12	660	2852	33	726	0.0	609

6.2.1.-(Ligases;)

ABCDEF_7378	UniRef50_Q9FFE6	0.0	Normal: 1022	1422	746	576	680	1408	BlastAnno: 2117	UniRef50_Q9FFE6	552	67.57	552	167	5	144	1778	1	547	0.0	768
ABCDEF_47513	UniRef50_Q8RU95	0.0	Normal: 411	609	570	233	749	887	BlastAnno: 2280	UniRef50_Q8RU95	598	53.15	572	246	6	119	1777	28	596	0.0	543
ABCDEF_78742	UniRef50_Q84P25	0.0	Normal: 236	207	373	576	450	306	BlastAnno: 1534	UniRef50_Q84P25	565	60.39	462	168	5	1413	49	19	472	0.0	546
ABCDEF_10753	UniRef50_Q9FFE6	0.0	Normal: 394	401	336	253	292	348	BlastAnno: 1841	UniRef50_Q9FFE6	552	51.00	551	259	5	117	1751	1	546	0.0	553
ABCDEF_4431	UniRef50_Q9FFE6	0.0	Normal: 27	358	28	98	112	914	BlastAnno: 2075	UniRef50_Q9FFE6	552	50.71	560	264	7	1774	107	1	552	0.0	551
ABCDEF_38213	UniRef50_Q84P25	0.0	Normal: 342	337	597	798	722	505	BlastAnno: 1939	UniRef50_Q84P25	565	62.34	555	194	5	1818	175	19	565	0.0	690
ABCDEF_25221	UniRef50_Q0DV32	0.0	Normal: 971	826	1003	1543	1442	1545	BlastAnno: 2009	UniRef50_Q0DV32	552	57.20	542	226	4	183	1793	9	549	0.0	613
ABCDEF_23413	UniRef50_Q84P23	0.0	Normal: 123	237	158	149	213	658	BlastAnno: 3175	UniRef50_Q84P23	562	51.74	547	243	8	3030	1435	15	555	0.0	555
ABCDEF_20486	UniRef50_Q9FFE6	0.0	Normal: 580	912	312	324	864	1565	BlastAnno: 3530	UniRef50_Q9FFE6	552	66.61	557	171	5	352	1995	1	551	0.0	781

>00531 Glycosaminoglycan degradation

2.3.1.78(heparan-alpha-glucosaminide N-acetyltransferase;)

No Correspond Unigene

3.1.6.12(N-acetylgalactosamine-4-sulfatase;)

No Correspond Unigene

3.1.6.13(iduronate-2-sulfatase;)

No Correspond Unigene

3.1.6.14(N-acetylglucosamine-6-sulfatase;)

No Correspond Unigene

3.1.6.18(glucuronate-2-sulfatase;)

No Correspond Unigene

3.1.6.4(N-acetylgalactosamine-6-sulfatase;)

No Correspond Unigene

3.10.1.1(N-sulfoglucosamine sulfohydrolase;)

No Correspond Unigene

3.2.-.-(Hydrolases;)

ABCDEF_27761	UniRef50_Q9FF10	0.0	Normal: 236	648	227	1008	1164	1135	BlastAnno: 2073	UniRef50_Q9FF10	543	59.23	542	214	3	1850	246	1	542	0.0	677
ABCDEF_52431	UniRef50_Q9FZP1	0.0	Normal: 208	463	314	5113	7494	638	BlastAnno: 2176	UniRef50_Q9FZP1	536	61.25	542	201	2	1902	277	3	535	0.0	699
ABCDEF_27762	UniRef50_Q9FF10	0.0	Normal: 282	803	296	1273	1279	1391	BlastAnno: 2244	UniRef50_Q9FF10	543	59.41	542	213	3	1850	246	1	542	0.0	678
ABCDEF_20425	UniRef50_Q9FF10	0.0	Normal: 166	299	167	252	186	251	BlastAnno: 2046	UniRef50_Q9FF10	543	61.95	523	192	4	363	1922	24	542	0.0	702
ABCDEF_20424	UniRef50_Q9FF10	0.0	Normal: 113	176	125	193	130	166	BlastAnno: 1439	UniRef50_Q9FF10	543	63.96	394	138	3	1313	141	24	416	0.0	556
ABCDEF_20428	UniRef50_Q9FF10	0.0	Normal: 194	273	176	256	188	303	BlastAnno: 2110	UniRef50_Q9FF10	543	61.95	523	192	4	351	1910	24	542	0.0	702
ABCDEF_27763	UniRef50_Q9FF10	0.0	Normal: 291	818	296	1258	1261	1514	BlastAnno: 2264	UniRef50_Q9FF10	543	59.41	542	213	3	438	2042	1	542	0.0	681

3.2.1.-(Hydrolases;)

ABCDEF_55518	UniRef50_Q9SGZ5	0.0	Normal: 137	145	110	155	249	211	BlastAnno: 1222	UniRef50_Q9SGZ5	767	66.34	407	136	1	1218	1	142	548	0.0	556
ABCDEF_45209	UniRef50_Q9LXA8	0.0	Normal: 1338	811	1191	1817	1167	1821	BlastAnno: 2643	UniRef50_Q9LXA8	792	47.09	790	379	11	2448	178	7	790	0.0	745
ABCDEF_34153	UniRef50_Q9LXA8	0.0	Normal: 522	668	469	700	564	936	BlastAnno: 2528	UniRef50_Q9LXA8	792	51.44	762	345	6	2397	175	30	787	0.0	804
ABCDEF_34154	UniRef50_Q9LXA8	0.0	Normal: 495	622	454	650	534	930	BlastAnno: 2619	UniRef50_Q9LXA8	792	50.53	655	301	5	2076	175	135	787	0.0	679
ABCDEF_47469	UniRef50_Q9SXC9	0.0	Normal: 80	59	95	141	105	115	BlastAnno: 1550	UniRef50_Q9SXC9	574	81.45	248	46	0	3	746	93	340	0.0	419
ABCDEF_3050	UniRef50_A8JBU2	0.0	Normal: 365	517	518	1626	1809	451	BlastAnno: 2998	UniRef50_A8JBU2	725	52.75	673	264	8	2589	604	50	679	0.0	670
ABCDEF_3049	UniRef50_A8JBU2	0.0	Normal: 528	690	724	2218	2620	674	BlastAnno: 3077	UniRef50_A8JBU2	725	52.32	711	286	7	2650	551	55	723	0.0	707
ABCDEF_34152	UniRef50_Q9LXA8	0.0	Normal: 568	815	532	816	648	1135	BlastAnno: 2581	UniRef50_Q9LXA8	792	51.24	765	348	6	2450	219	30	790	0.0	806
ABCDEF_34155	UniRef50_Q9LXA8	0.0	Normal: 543	763	515	772	621	1127	BlastAnno: 2640	UniRef50_Q9LXA8	792	50.30	658	304	5	2112	202	135	790	0.0	681
ABCDEF_21385	UniRef50_Q9LXA8	0.0	Normal: 432	334	511	873	734	380	BlastAnno: 2109	UniRef50_Q9LXA8	792	62.64	629	229	4	3	1877	152	778	0.0	775
ABCDEF_980	UniRef50_Q9LXA8	0.0	Normal: 1398	7037	1070	4974	7681	6612	BlastAnno: 2765	UniRef50_Q9LXA8	792	49.22	772	351	9	257	2470	28	792	0.0	753
ABCDEF_31427	UniRef50_Q9LXA8	0.0	Normal: 56	316	89	4893	4124	2869	BlastAnno: 2823	UniRef50_Q9LXA8	792	48.25	773	359	12	209	2425	26	791	0.0	716
ABCDEF_27305	UniRef50_Q9FG93	0.0	Normal: 247	143	251	353	378	254	BlastAnno: 1883	UniRef50_Q9FG93	624	70.43	487	123	3	2	1456	126	593	0.0	702
ABCDEF_47466	UniRef50_Q9SXC9	0.0	Normal: 69	48	83	133	95	97	BlastAnno: 1452	UniRef50_Q9SXC9	574	75.84	476	115	0	2	1429	96	571	0.0	769
ABCDEF_3403	UniRef50_Q9LXA8	0.0	Normal: 206	82	182	114	358	291	BlastAnno: 2202	UniRef50_Q9LXA8	792	55.15	515	213	4	2198	702	84	596	0.0	548

3.2.1.103(keratan-sulfate endo-1,4-beta-galactosidase;)

No Correspond Unigene

3.2.1.23(beta-galactosidase;)

ABCDEF_48113	UniRef50_Q6Z6K4	0.0	Normal: 61	2624	32	127	673	5080	BlastAnno: 2898	UniRef50_Q6Z6K4	729	63.35	704	250	4	2505	394	33	728	0.0	931
ABCDEF_23777	UniRef50_Q6Z6K4	0.0	Normal: 219	63	227	164	235	472	BlastAnno: 2261	UniRef50_Q6Z6K4	729	66.38	690	225	5	2	2071	46	728	0.0	944
ABCDEF_3074	UniRef50_Q6Z6K4	0.0	Normal: 431	1551	321	2473	394	19728	BlastAnno: 3342	UniRef50_Q6Z6K4	729	67.09	702	221	6	415	2517	35	727	0.0	994
ABCDEF_43854	UniRef50_Q6Z6K4	0.0	Normal: 1	9	1	105	49	28	BlastAnno: 1781	UniRef50_Q6Z6K4	729	58.79	512	180	6	1	1536	147	627	0.0	595
ABCDEF_44469	UniRef50_Q8W0A1	0.0	Normal: 162	225	175	701	194	597	BlastAnno: 2319	UniRef50_Q8W0A1	827	46.10	679	324	12	2236	221	183	826	0.0	583
ABCDEF_13827	UniRef50_Q6Z6K4	0.0	Normal: 219	1054	213	1958	627	863	BlastAnno: 3038	UniRef50_Q6Z6K4	729	57.90	715	270	8	419	2539	36	727	0.0	825
ABCDEF_21624	UniRef50_Q6Z6K4	0.0	Normal: 4958	5483	4088	4956	4209	6920	BlastAnno: 3330	UniRef50_Q6Z6K4	729	62.64	696	251	4	2975	888	32	718	0.0	932
ABCDEF_43857	UniRef50_Q6Z6K4	0.0	Normal: 2	9	4	110	53	30	BlastAnno: 1655	UniRef50_Q6Z6K4	729	60.07	546	187	6	1	1638	147	661	0.0	655
ABCDEF_43861	UniRef50_Q6Z6K4	0.0	Normal: 4	15	5	162	72	47	BlastAnno: 1891	UniRef50_Q6Z6K4	729	65.21	526	179	3	107	1684	34	555	0.0	719
ABCDEF_84604	UniRef50_Q6Z6K4	0.0	Normal: 34	1696	14	81	379	2927	BlastAnno: 2528	UniRef50_Q6Z6K4	729	62.99	670	240	4	394	2403	33	694	0.0	881
ABCDEF_13826	UniRef50_Q6Z6K4	0.0	Normal: 184	889	166	1515	526	703	BlastAnno: 2897	UniRef50_Q6Z6K4	729	55.30	613	243	8	2286	472	138	727	0.0	657
ABCDEF_10626	UniRef50_Q6Z6K4	0.0	Normal: 229	909	224	1002	2471	3794	BlastAnno: 3133	UniRef50_Q6Z6K4	729	57.14	700	285	8	498	2588	40	727	0.0	809
ABCDEF_27471	UniRef50_Q6Z6K4	0.0	Normal: 992	533	1105	1489	2430	1240	BlastAnno: 3113	UniRef50_Q6Z6K4	729	64.49	704	240	5	314	2422	35	729	0.0	962
ABCDEF_43858	UniRef50_Q6Z6K4	0.0	Normal: 6	15	7	186	85	54	BlastAnno: 2028	UniRef50_Q6Z6K4	729	65.35	635	213	5	107	2011	34	661	0.0	862
ABCDEF_31373	UniRef50_Q6Z6K4	0.0	Normal: 1333	1029	1160	1241	1216	1855	BlastAnno: 3006	UniRef50_Q6Z6K4	729	52.03	713	319	7	2761	626	37	727	0.0	732
ABCDEF_10066	UniRef50_Q93Z24	0.0	Normal: 999	932	1523	1674	2007	1465	BlastAnno: 3015	UniRef50_Q93Z24	697	65.89	645	208	4	2659	737	61	697	0.0	865
ABCDEF_51808	UniRef50_Q6Z6K4	0.0	Normal: 364	454	209	36	62	1109	BlastAnno: 2817	UniRef50_Q6Z6K4	729	54.56	713	306	8	2582	447	32	727	0.0	791
ABCDEF_48111	UniRef50_Q6Z6K4	0.0	Normal: 41	1700	15	77	347	2953	BlastAnno: 2607	UniRef50_Q6Z6K4	729	67.21	494	157	3	394	1875	33	521	0.0	696
ABCDEF_17519	UniRef50_A5F5U6	0.0	Normal: 1178	1482	1392	2252	1850	3281	BlastAnno: 3706	UniRef50_A5F5U6	1024	39.71	1093	547	21	3469	254	13	1014	0.0	729
ABCDEF_40816	UniRef50_Q6Z6K4	0.0	Normal: 1166	754	898	2543	5298	532	BlastAnno: 3066	UniRef50_Q6Z6K4	729	62.55	705	254	4	2970	856	35	729	0.0	936
ABCDEF_44668	UniRef50_Q8GX69	0.0	Normal: 146	74	84	327	133	152	BlastAnno: 1396	UniRef50_Q8GX69	815	66.74	463	152	2	6	1394	23	483	0.0	671
ABCDEF_43856	UniRef50_Q6Z6K4	0.0	Normal: 12	20	12	195	92	71	BlastAnno: 1873	UniRef50_Q6Z6K4	729	61.89	488	179	5	1	1464	147	627	0.0	615
ABCDEF_44470	UniRef50_Q6Z6K4	0.0	Normal: 287	387	252	1193	287	741	BlastAnno: 2917	UniRef50_Q6Z6K4	729	52.28	723	310	9	2707	551	38	729	0.0	752
ABCDEF_49396	UniRef50_Q6Z6K4	0.0	Normal: 0	452	3	48	33	6872	BlastAnno: 2312	UniRef50_Q6Z6K4	729	65.83	676	221	7	2312	288	62	728	0.0	893
ABCDEF_10631	UniRef50_Q6Z6K4	0.0	Normal: 3079	207	2624	1970	2833	969	BlastAnno: 3058	UniRef50_Q6Z6K4	729	60.74	703	267	4	2943	835	36	729	0.0	917
ABCDEF_48112	UniRef50_Q6Z6K4	0.0	Normal: 60	2628	33	127	663	5106	BlastAnno: 2977	UniRef50_Q6Z6K4	729	67.21	494	157	3	2584	1103	33	521	0.0	696

3.2.1.31(beta-glucuronidase;)

No Correspond Unigene

3.2.1.35(hyaluronoglucosaminidase;)

No Correspond Unigene

3.2.1.50(alpha-N-acetylglucosaminidase;)

ABCDEF_4067	UniRef50_P54802	0.0	Normal: 1112	811	987	1350	1683	1418	BlastAnno: 2719	UniRef50_P54802	743	40.97	725	372	13	395	2560	64	735	0.0	579

3.2.1.52(beta-N-acetylhexosaminidase;)

ABCDEF_16822	UniRef50_Q54K56	3e-109	Normal: 543	493	486	692	917	660	BlastAnno: 1760	UniRef50_Q54K56	564	36.42	519	294	10	1587	94	59	562	3e-109	347

3.2.1.76(L-iduronidase;)

No Correspond Unigene

>00532 Glycosaminoglycan biosynthesis - chondroitin sulfate

2.4.1.133(xylosylprotein 4-beta-galactosyltransferase;)

No Correspond Unigene

2.4.1.134(galactosylxylosylprotein 3-beta-galactosyltransferase;)

No Correspond Unigene

2.4.1.135(galactosylgalactosylxylosylprotein 3-beta-glucuronosyltransferase;)

ABCDEF_31804	UniRef50_B9SGQ5	5e-136	Normal: 34	377	16	116	405	124	BlastAnno: 1307	UniRef50_B9SGQ5	369	60.29	350	130	5	1160	114	1	342	5e-136	404

2.4.1.174(glucuronylgalactosylproteoglycan 4-beta-N-acetylgalactosaminyltransferase;)

No Correspond Unigene

2.4.1.175(glucuronosyl-N-acetylgalactosaminyl-proteoglycan 4-beta-N-acetylgalactosaminyltransferase;)

No Correspond Unigene

2.4.1.226(N-acetylgalactosaminyl-proteoglycan 3-beta-glucuronosyltransferase;)

No Correspond Unigene

2.4.2.26(protein xylosyltransferase;)

ABCDEF_24102	UniRef50_B9RW58	0.0	Normal: 103	123	104	121	104	183	BlastAnno: 1599	UniRef50_B9RW58	439	75.93	432	103	1	48	1340	7	438	0.0	701
ABCDEF_48808	UniRef50_B9RW58	0.0	Normal: 211	165	185	362	400	222	BlastAnno: 1513	UniRef50_B9RW58	439	73.27	434	115	1	63	1361	5	438	0.0	676

2.8.2.-(Transferases;)

ABCDEF_60620	UniRef50_P52839	2e-85	Normal: 240	393	445	400	902	151	BlastAnno: 1191	UniRef50_P52839	326	45.95	333	168	6	58	1050	2	324	2e-85	271

2.8.2.17(chondroitin 6-sulfotransferase;)

No Correspond Unigene

2.8.2.33(N-acetylgalactosamine 4-sulfate 6-O-sulfotransferase;)

No Correspond Unigene

2.8.2.5(chondroitin 4-sulfotransferase;)

No Correspond Unigene

5.1.3.19(chondroitin-glucuronate 5-epimerase;)

No Correspond Unigene

>00533 Glycosaminoglycan biosynthesis - keratan sulfate

2.4.1.-(Transferases;)

ABCDEF_18004	UniRef50_Q6YWK8	0.0	Normal: 1562	1736	2009	3452	4438	2400	BlastAnno: 2298	UniRef50_Q6YWK8	570	65.27	429	136	1	1941	694	57	485	0.0	608
ABCDEF_52379	UniRef50_Q9LHZ7	0.0	Normal: 4015	4921	4546	10520	13430	4731	BlastAnno: 3479	UniRef50_Q9LHZ7	1170	48.04	893	342	16	825	3242	299	1156	0.0	787
ABCDEF_48593	UniRef50_Q9SK82	0.0	Normal: 12	420	19	343	763	253	BlastAnno: 1834	UniRef50_Q9SK82	489	60.08	481	186	4	1714	278	10	486	0.0	633
ABCDEF_13375	UniRef50_Q8VZK9	0.0	Normal: 108	123	208	526	34	294	BlastAnno: 1738	UniRef50_Q8VZK9	729	53.62	552	234	8	94	1737	18	551	0.0	582
ABCDEF_40982	UniRef50_Q96301	0.0	Normal: 1037	706	819	1249	1084	920	BlastAnno: 3418	UniRef50_Q96301	914	79.60	902	173	3	243	2948	1	891	0.0	1506
ABCDEF_47674	UniRef50_Q9LHZ7	0.0	Normal: 274	92	202	599	505	280	BlastAnno: 2526	UniRef50_Q9LHZ7	1170	75.93	777	151	8	2260	2	15	779	0.0	1177
ABCDEF_34097	UniRef50_Q9LE59	0.0	Normal: 626	613	923	1797	1829	692	BlastAnno: 2521	UniRef50_Q9LE59	673	60.92	458	178	1	1687	317	215	672	0.0	630
ABCDEF_34098	UniRef50_Q9LE59	0.0	Normal: 457	464	697	1283	1312	539	BlastAnno: 2199	UniRef50_Q9LE59	673	60.92	458	178	1	1687	317	215	672	0.0	628
ABCDEF_10848	UniRef50_Q9M156	0.0	Normal: 157	2988	163	408	772	1566	BlastAnno: 1810	UniRef50_Q9M156	480	60.09	456	176	4	1700	336	4	454	0.0	553
ABCDEF_41182	UniRef50_Q9LHZ7	0.0	Normal: 150	2169	221	814	1407	495	BlastAnno: 3604	UniRef50_Q9LHZ7	1170	51.03	870	333	15	2822	456	299	1156	0.0	826
ABCDEF_40981	UniRef50_Q96301	0.0	Normal: 156	97	110	160	179	173	BlastAnno: 1225	UniRef50_Q96301	914	86.92	344	45	0	1112	81	394	737	0.0	652
ABCDEF_9506	UniRef50_Q9LHZ7	0.0	Normal: 1302	2128	1494	3356	4644	2206	BlastAnno: 3751	UniRef50_Q9LHZ7	1170	49.38	891	343	17	927	3359	299	1161	0.0	812
ABCDEF_9500	UniRef50_Q9LHZ7	0.0	Normal: 183	188	186	576	498	260	BlastAnno: 1717	UniRef50_Q9LHZ7	1170	47.98	644	234	14	3	1715	418	1033	0.0	571
ABCDEF_100	UniRef50_Q9M8Y0	0.0	Normal: 228	309	224	416	461	141	BlastAnno: 2109	UniRef50_Q9M8Y0	977	77.49	533	120	0	1610	12	50	582	0.0	884
ABCDEF_33659	UniRef50_Q9LHZ7	0.0	Normal: 1282	1948	2248	5826	10642	2083	BlastAnno: 4289	UniRef50_Q9LHZ7	1170	48.70	887	345	15	2955	538	299	1156	0.0	788
ABCDEF_37667	UniRef50_Q9LHZ7	0.0	Normal: 248	3617	251	1237	2078	970	BlastAnno: 3161	UniRef50_Q9LHZ7	1170	49.72	885	335	14	2671	260	301	1156	0.0	810
ABCDEF_47673	UniRef50_Q9LHZ7	0.0	Normal: 876	296	828	1628	1721	1172	BlastAnno: 3878	UniRef50_Q9LHZ7	1170	80.88	1161	186	8	3642	232	15	1163	0.0	1865
ABCDEF_99	UniRef50_Q9M8Y0	0.0	Normal: 272	330	222	398	423	145	BlastAnno: 2094	UniRef50_Q9M8Y0	977	77.36	561	127	0	1683	1	50	610	0.0	929
ABCDEF_20658	UniRef50_Q8VZK9	0.0	Normal: 373	33	193	64	503	141	BlastAnno: 2316	UniRef50_Q8VZK9	729	53.35	508	221	7	47	1564	11	504	0.0	559
ABCDEF_10183	UniRef50_A7XDQ9	0.0	Normal: 422	455	346	577	378	610	BlastAnno: 2362	UniRef50_A7XDQ9	684	50.94	693	307	12	281	2287	1	684	0.0	670
ABCDEF_30745	UniRef50_A7XDQ9	0.0	Normal: 1209	351	1031	1645	1226	635	BlastAnno: 2646	UniRef50_A7XDQ9	684	57.58	554	227	6	546	2189	133	684	0.0	664
ABCDEF_13336	UniRef50_Q7PC69	0.0	Normal: 148	563	194	957	1575	816	BlastAnno: 2584	UniRef50_Q7PC69	745	71.56	559	136	6	744	2351	187	745	0.0	730
ABCDEF_21426	UniRef50_Q8RU27	0.0	Normal: 2658	1979	3417	6157	9464	2599	BlastAnno: 1530	UniRef50_Q8RU27	366	89.44	360	34	2	1358	282	1	357	0.0	667
ABCDEF_71242	UniRef50_Q9ZV71	0.0	Normal: 134	122	179	480	754	119	BlastAnno: 1835	UniRef50_Q9ZV71	409	73.35	409	103	4	231	1439	1	409	0.0	587
ABCDEF_52020	UniRef50_Q6YWK8	0.0	Normal: 1635	1534	1975	4284	10353	2289	BlastAnno: 2394	UniRef50_Q6YWK8	570	58.37	514	185	2	403	1857	57	570	0.0	607
ABCDEF_51269	UniRef50_Q6YWK8	0.0	Normal: 147	85	103	21	33	438	BlastAnno: 1864	UniRef50_Q6YWK8	570	54.28	514	204	2	1646	198	57	570	0.0	586
ABCDEF_30753	UniRef50_Q9ZV71	0.0	Normal: 290	497	417	629	1013	634	BlastAnno: 1502	UniRef50_Q9ZV71	409	72.32	401	107	2	311	1501	1	401	0.0	590
ABCDEF_4233	UniRef50_Q9ZV71	0.0	Normal: 369	412	550	936	860	750	BlastAnno: 2004	UniRef50_Q9ZV71	409	72.13	409	110	2	370	1584	1	409	0.0	613
ABCDEF_17525	UniRef50_Q8RU27	0.0	Normal: 1500	1289	2149	3711	4446	1069	BlastAnno: 1507	UniRef50_Q8RU27	366	91.35	347	30	0	1371	331	3	349	0.0	671
ABCDEF_18005	UniRef50_Q6YWK8	0.0	Normal: 1842	2042	2515	4214	5208	2842	BlastAnno: 2305	UniRef50_Q6YWK8	570	59.14	514	180	2	1948	497	57	570	0.0	642
ABCDEF_41140	UniRef50_Q6YWK8	0.0	Normal: 86	1314	93	753	1787	2076	BlastAnno: 2346	UniRef50_Q6YWK8	570	60.00	510	174	2	431	1870	57	566	0.0	669
ABCDEF_18002	UniRef50_Q9LHZ7	0.0	Normal: 376	127	367	601	1190	520	BlastAnno: 1605	UniRef50_Q9LHZ7	1170	89.75	400	41	0	1603	404	771	1170	0.0	732
ABCDEF_13339	UniRef50_Q7PC69	0.0	Normal: 127	814	183	541	1135	1144	BlastAnno: 2610	UniRef50_Q7PC69	745	70.48	559	141	6	725	2329	187	745	0.0	729
ABCDEF_41141	UniRef50_Q6YWK8	0.0	Normal: 67	1107	84	708	1690	1854	BlastAnno: 2461	UniRef50_Q6YWK8	570	60.00	510	174	2	1916	477	57	566	0.0	668
ABCDEF_44583	UniRef50_Q9LE59	0.0	Normal: 681	561	559	947	1745	789	BlastAnno: 2470	UniRef50_Q9LE59	673	59.21	456	185	1	1749	385	214	669	0.0	621
ABCDEF_3869	UniRef50_Q9LE59	0.0	Normal: 333	393	415	809	907	754	BlastAnno: 2136	UniRef50_Q9LE59	673	59.05	613	227	4	1827	1	81	673	0.0	699
ABCDEF_28947	UniRef50_Q9SK82	0.0	Normal: 0	851	3	0	0	116	BlastAnno: 1567	UniRef50_Q9SK82	489	62.40	484	176	4	1524	85	1	482	0.0	652
ABCDEF_31119	UniRef50_Q9M8Y0	0.0	Normal: 2212	1279	1917	2933	3341	1803	BlastAnno: 3695	UniRef50_Q9M8Y0	977	81.08	925	174	1	392	3163	50	974	0.0	1583
ABCDEF_37311	UniRef50_Q9LE59	0.0	Normal: 175	133	191	549	421	366	BlastAnno: 1612	UniRef50_Q9LE59	673	62.96	486	153	4	1406	3	4	480	0.0	542
ABCDEF_24190	UniRef50_Q8L7F9	0.0	Normal: 397	265	492	583	553	371	BlastAnno: 2564	UniRef50_Q8L7F9	643	63.91	640	218	4	2353	473	1	640	0.0	850
ABCDEF_30548	UniRef50_Q9FH36	0.0	Normal: 17	916	27	151	420	126	BlastAnno: 1963	UniRef50_Q9FH36	535	72.47	534	138	3	102	1676	1	534	0.0	767
ABCDEF_27416	UniRef50_Q7PC69	0.0	Normal: 575	837	875	1344	2015	936	BlastAnno: 2454	UniRef50_Q7PC69	745	66.11	416	115	4	2337	1168	126	541	0.0	518
ABCDEF_48373	UniRef50_A7XDQ9	0.0	Normal: 1364	773	1336	1451	1791	1202	BlastAnno: 3042	UniRef50_A7XDQ9	684	63.68	691	234	6	2405	360	1	683	0.0	915
ABCDEF_3868	UniRef50_Q9LE59	0.0	Normal: 224	274	301	555	686	524	BlastAnno: 1670	UniRef50_Q9LE59	673	65.31	516	176	1	1556	9	161	673	0.0	696
ABCDEF_31288	UniRef50_Q940V3	0.0	Normal: 247	309	183	101	124	655	BlastAnno: 1658	UniRef50_Q940V3	470	56.01	466	197	7	1614	232	8	470	0.0	537
ABCDEF_52574	UniRef50_Q09332	0.0	Normal: 885	474	1068	1835	1853	816	BlastAnno: 3720	UniRef50_Q09332	1548	47.89	687	310	14	2551	533	859	1511	0.0	598
ABCDEF_79752	UniRef50_Q9ZV71	0.0	Normal: 158	183	245	400	379	352	BlastAnno: 1266	UniRef50_Q9ZV71	409	71.39	360	99	2	31	1098	1	360	0.0	526
ABCDEF_9509	UniRef50_Q9LHZ7	0.0	Normal: 1296	2171	1498	3410	4683	2232	BlastAnno: 3897	UniRef50_Q9LHZ7	1170	49.38	891	343	17	1073	3505	299	1161	0.0	812

ABCDEF_48200	UniRef50_Q6YWK8	0.0	Normal: 1768	576	3156	4431	6634	491	BlastAnno: 2274	UniRef50_Q6YWK8	570	57.98	514	185	4	291	1739	57	570	0.0	642
ABCDEF_24155	UniRef50_Q9ZVI7	0.0	Normal: 503	247	607	1676	1833	578	BlastAnno: 2708	UniRef50_Q9ZVI7	619	52.33	621	264	11	306	2123	15	618	0.0	601
ABCDEF_21246	UniRef50_Q9SKT6	0.0	Normal: 92	107	188	380	834	84	BlastAnno: 1791	UniRef50_Q9SKT6	536	56.61	484	204	5	123	1562	52	533	0.0	568
ABCDEF_32317	UniRef50_Q8RU27	0.0	Normal: 12	9	3	118	800	62	BlastAnno: 1076	UniRef50_Q8RU27	366	87.02	285	37	0	1075	221	64	348	0.0	535
ABCDEF_9502	UniRef50_Q9LHZ7	0.0	Normal: 610	486	568	1331	1403	591	BlastAnno: 1961	UniRef50_Q9LHZ7	1170	48.64	625	249	13	3	1742	564	1161	0.0	570
ABCDEF_23298	UniRef50_Q9FH36	0.0	Normal: 128	527	130	223	314	864	BlastAnno: 2832	UniRef50_Q9FH36	535	83.16	196	33	0	696	1283	340	535	0.0	357
ABCDEF_40595	UniRef50_O80898	0.0	Normal: 229	135	103	25	307	325	BlastAnno: 2413	UniRef50_O80898	757	44.68	761	378	13	95	2299	10	753	0.0	587
ABCDEF_63947	UniRef50_Q9SK82	0.0	Normal: 0	267	0	0	71	0	BlastAnno: 1446	UniRef50_Q9SK82	489	58.33	468	189	4	60	1445	1	468	0.0	592
ABCDEF_67847	UniRef50_Q9M8Y0	0.0	Normal: 794	476	871	1081	1401	868	BlastAnno: 1591	UniRef50_Q9M8Y0	977	84.86	370	56	0	1589	480	605	974	0.0	685
ABCDEF_65666	UniRef50_Q09332	0.0	Normal: 800	441	946	1606	1637	735	BlastAnno: 3518	UniRef50_Q09332	1548	47.54	671	305	13	1162	3087	859	1511	0.0	586
ABCDEF_38641	UniRef50_Q9FWA4	0.0	Normal: 674	967	1162	2603	4404	2475	BlastAnno: 1599	UniRef50_Q9FWA4	561	81.35	386	72	0	1	1158	176	561	0.0	641
ABCDEF_17914	UniRef50_Q9SKT6	0.0	Normal: 784	567	886	1447	1439	1424	BlastAnno: 2049	UniRef50_Q9SKT6	536	72.95	536	140	2	204	1796	1	536	0.0	785
ABCDEF_18286	UniRef50_Q6NQB7	0.0	Normal: 1131	1108	1225	1872	1722	3329	BlastAnno: 1752	UniRef50_Q6NQB7	393	73.80	397	81	6	1527	367	10	393	0.0	607
ABCDEF_119	UniRef50_Q8RU27	0.0	Normal: 1613	1155	2249	3558	7663	1775	BlastAnno: 1532	UniRef50_Q8RU27	366	88.33	360	38	2	1306	230	1	357	0.0	663
ABCDEF_47603	UniRef50_Q9M9Y5	0.0	Normal: 2609	8779	1664	1103	2085	22991	BlastAnno: 2830	UniRef50_Q9M9Y5	589	54.04	594	267	3	2475	694	1	588	0.0	654
ABCDEF_24192	UniRef50_Q8L7F9	0.0	Normal: 391	244	465	553	497	345	BlastAnno: 2477	UniRef50_Q8L7F9	643	58.99	434	165	4	212	1474	1	434	0.0	513
ABCDEF_17482	UniRef50_Q9SKT6	0.0	Normal: 615	379	816	1068	1470	852	BlastAnno: 2083	UniRef50_Q9SKT6	536	71.11	540	147	4	1915	311	1	536	0.0	786
ABCDEF_85909	UniRef50_Q8VZK9	0.0	Normal: 28	45	9	10	44	127	BlastAnno: 1473	UniRef50_Q8VZK9	729	55.37	475	198	6	1419	1	11	473	0.0	536
ABCDEF_9503	UniRef50_Q9LHZ7	0.0	Normal: 487	544	524	1242	1320	833	BlastAnno: 2087	UniRef50_Q9LHZ7	1170	50.35	574	236	8	57	1712	615	1161	0.0	567
ABCDEF_40594	UniRef50_O80898	0.0	Normal: 256	127	102	41	331	326	BlastAnno: 2417	UniRef50_O80898	757	45.76	719	348	12	213	2291	51	753	0.0	585
ABCDEF_17141	UniRef50_Q9LHZ7	0.0	Normal: 74	1734	69	380	701	333	BlastAnno: 3337	UniRef50_Q9LHZ7	1170	50.92	868	335	14	2539	179	299	1156	0.0	816
ABCDEF_33658	UniRef50_Q9LHZ7	0.0	Normal: 384	308	395	805	1003	602	BlastAnno: 2961	UniRef50_Q9LHZ7	1170	48.76	888	343	14	204	2621	299	1156	0.0	792
ABCDEF_20579	UniRef50_Q7PC69	0.0	Normal: 1007	795	1238	2311	2309	1345	BlastAnno: 3113	UniRef50_Q7PC69	745	59.97	767	229	14	2667	535	1	745	0.0	768
ABCDEF_31118	UniRef50_Q9M8Y0	0.0	Normal: 2135	1267	1900	3004	3329	1757	BlastAnno: 3787	UniRef50_Q9M8Y0	977	81.10	709	133	1	1132	3255	266	974	0.0	1217
ABCDEF_9499	UniRef50_Q9LHZ7	0.0	Normal: 205	207	215	632	538	275	BlastAnno: 1794	UniRef50_Q9LHZ7	1170	48.88	667	240	14	11	1792	395	1033	0.0	605
ABCDEF_48199	UniRef50_Q6YWK8	0.0	Normal: 1506	467	2429	3094	4233	439	BlastAnno: 1997	UniRef50_Q6YWK8	570	63.10	439	146	2	291	1559	57	495	0.0	610
ABCDEF_40983	UniRef50_Q96301	0.0	Normal: 406	261	343	392	415	433	BlastAnno: 2265	UniRef50_Q96301	914	79.17	504	99	1	114	1625	394	891	0.0	854
ABCDEF_17140	UniRef50_Q9LHZ7	0.0	Normal: 45	1049	49	216	386	208	BlastAnno: 2293	UniRef50_Q9LHZ7	1170	52.34	770	281	13	2138	57	397	1156	0.0	739
ABCDEF_18352	UniRef50_Q9FWA4	0.0	Normal: 2427	2688	2969	5519	6547	4855	BlastAnno: 2224	UniRef50_Q9FWA4	561	69.31	505	148	3	1887	382	61	561	0.0	683
ABCDEF_27767	UniRef50_Q9LHZ7	0.0	Normal: 2576	5141	2841	10249	14583	5031	BlastAnno: 3981	UniRef50_Q9LHZ7	1170	49.66	888	337	16	2927	504	299	1156	0.0	818
ABCDEF_33661	UniRef50_Q9LHZ7	0.0	Normal: 414	340	507	879	790	783	BlastAnno: 2185	UniRef50_Q9LHZ7	1170	48.66	748	277	14	2184	169	440	1156	0.0	662
ABCDEF_14685	UniRef50_A7XDQ9	0.0	Normal: 233	308	134	573	292	334	BlastAnno: 1947	UniRef50_A7XDQ9	684	56.84	468	186	6	569	1945	1	461	0.0	545
ABCDEF_20660	UniRef50_Q8VZK9	0.0	Normal: 22	48	13	15	52	144	BlastAnno: 1473	UniRef50_Q8VZK9	729	55.16	475	199	6	55	1473	11	473	0.0	537
ABCDEF_21315	UniRef50_Q9LHZ7	0.0	Normal: 3412	252	1450	0	79	1228	BlastAnno: 3765	UniRef50_Q9LHZ7	1170	63.78	1096	340	15	308	3508	70	1137	0.0	1333
ABCDEF_533	UniRef50_Q9C8W3	0.0	Normal: 230	204	269	505	411	301	BlastAnno: 1688	UniRef50_Q9C8W3	401	70.33	364	94	2	393	1475	47	399	0.0	555
ABCDEF_34099	UniRef50_Q9LE59	0.0	Normal: 478	491	735	1356	1350	548	BlastAnno: 2158	UniRef50_Q9LE59	673	60.92	458	178	1	1610	240	215	672	0.0	630
ABCDEF_47984	UniRef50_Q7PC69	0.0	Normal: 663	457	734	1362	2147	696	BlastAnno: 2838	UniRef50_Q7PC69	745	59.27	712	226	9	373	2316	34	745	0.0	702
ABCDEF_51451	UniRef50_Q9LE59	0.0	Normal: 1883	1782	2202	4337	4350	2667	BlastAnno: 2874	UniRef50_Q9LE59	673	74.16	685	165	6	2559	505	1	673	0.0	982
ABCDEF_13370	UniRef50_Q8VZK9	0.0	Normal: 290	87	317	377	105	258	BlastAnno: 2033	UniRef50_Q8VZK9	729	51.65	635	278	11	2031	154	112	726	0.0	612
ABCDEF_48239	UniRef50_Q9LHZ7	0.0	Normal: 511	163	422	1154	1316	510	BlastAnno: 2714	UniRef50_Q9LHZ7	1170	76.20	769	156	9	478	2712	41	806	0.0	1201
ABCDEF_13338	UniRef50_Q7PC69	0.0	Normal: 335	468	417	2639	4443	616	BlastAnno: 2525	UniRef50_Q7PC69	745	62.45	727	209	12	298	2292	21	745	0.0	781
ABCDEF_24193	UniRef50_Q8L7F9	0.0	Normal: 113	72	132	203	180	119	BlastAnno: 1679	UniRef50_Q8L7F9	643	60.54	479	176	4	212	1609	1	479	0.0	592
ABCDEF_48514	UniRef50_Q9ZV71	0.0	Normal: 540	580	601	771	1033	926	BlastAnno: 1920	UniRef50_Q9ZV71	409	78.14	398	85	2	1618	431	12	409	0.0	637

2.4.1.149(N-acetyllactosaminide beta-1,3-N-acetylglucosaminyltransferase;)

No Correspond Unigene

2.4.1.38(beta-N-acetylglucosaminylglycopeptide beta-1,4-galactosyltransferase;)

No Correspond Unigene

2.4.1.68(glycoprotein 6-alpha-L-fucosyltransferase;)

No Correspond Unigene

2.4.99.4(beta-galactoside alpha-2,3-sialyltransferase;)

ABCDEF_28297	UniRef50_B9SCR7	4e-167	Normal: 573	742	586	923	1062	1024	BlastAnno: 1855	UniRef50_B9SCR7	434	60.36	391	149	4	1454	294	46	434	4e-167	493

2.4.99.6(N-acetyllactosaminide alpha-2,3-sialyltransferase;)

No Correspond Unigene

2.8.2.-(Transferases;)

ABCDEF_60620	UniRef50_P52839	2e-85	Normal: 240	393	445	400	902	151	BlastAnno: 1191	UniRef50_P52839	326	45.95	333	168	6	58	1050	2	324	2e-85	271

2.8.2.21(keratan sulfotransferase;)

No Correspond Unigene

>00534 Glycosaminoglycan biosynthesis - heparan sulfate

2.4.1.133(xylosylprotein 4-beta-galactosyltransferase;)

No Correspond Unigene

2.4.1.134(galactosylxylosylprotein 3-beta-galactosyltransferase;)

No Correspond Unigene

2.4.1.135(galactosylgalactosylxylosylprotein 3-beta-glucuronosyltransferase;)

ABCDEF_31804	UniRef50_B9SGQ5	5e-136	Normal: 34	377	16	116	405	124	BlastAnno: 1307	UniRef50_B9SGQ5	369	60.29	350	130	5	1160	114	1	342	5e-136	404

2.4.1.223(glucuronyl-galactosyl-proteoglycan 4-alpha-N-acetylglucosaminyltransferase;)

ABCDEF_14217	UniRef50_B9SCN4	2e-124	Normal: 217	140	313	819	641	146	BlastAnno: 1802	UniRef50_B9SCN4	349	60.51	314	107	5	706	1602	38	349	2e-124	379

2.4.1.224(glucuronosyl-N-acetylglucosaminyl-proteoglycan 4-alpha-N-acetylglucosaminyltransferase;)

ABCDEF_24526	UniRef50_B9SRL1	0.0	Normal: 28	18	28	98	130	20	BlastAnno: 1648	UniRef50_B9SRL1	574	64.87	390	132	3	1406	237	183	567	0.0	548
ABCDEF_19226	UniRef50_B9SRL1	0.0	Normal: 0	4734	0	0	0	5815	BlastAnno: 1774	UniRef50_B9SRL1	574	70.08	391	114	3	390	1559	178	566	0.0	585

2.4.1.225(N-acetylglucosaminyl-proteoglycan 4-beta-glucuronosyltransferase;)

No Correspond Unigene

2.4.2.26(protein xylosyltransferase;)

ABCDEF_24102	UniRef50_B9RW58	0.0	Normal: 103	123	104	121	104	183	BlastAnno: 1599	UniRef50_B9RW58	439	75.93	432	103	1	48	1340	7	438	0.0	701
ABCDEF_48808	UniRef50_B9RW58	0.0	Normal: 211	165	185	362	400	222	BlastAnno: 1513	UniRef50_B9RW58	439	73.27	434	115	1	63	1361	5	438	0.0	676

2.8.2.-(Transferases;)

ABCDEF_60620	UniRef50_P52839	2e-85	Normal: 240	393	445	400	902	151	BlastAnno: 1191	UniRef50_P52839	326	45.95	333	168	6	58	1050	2	324	2e-85	271

2.8.2.23(No Name)

ABCDEF_42273	UniRef50_Q23AP5	3e-77	Normal: 3487	1907	4242	6030	7335	3814	BlastAnno: 1668	UniRef50_Q23AP5	430	36.34	388	222	9	271	1422	53	419	3e-77	258

2.8.2.29(No Name)

No Correspond Unigene

2.8.2.30(No Name)

No Correspond Unigene

5.1.3.17(heparosan-N-sulfate-glucuronate 5-epimerase;)

No Correspond Unigene

>00540 Lipopolysaccharide biosynthesis

2.-.-.-(Transferases)

ABCDEF_3256	UniRef50_Q5VV42	9e-165	Normal: 1823	1179	1691	2271	1981	2383	BlastAnno: 3398	UniRef50_Q5VV42	579	50.91	497	220	6	3326	1866	10	492	9e-165	509

2.3.1.-(Transferases;)

ABCDEF_520	UniRef50_Q9FF86	0.0	Normal: 1672	85	2273	5056	3383	1211	BlastAnno: 1811	UniRef50_Q9FF86	484	64.06	448	149	6	1664	354	3	449	0.0	573
ABCDEF_68664	UniRef50_Q9ZVN2	0.0	Normal: 79	35	85	107	193	63	BlastAnno: 1488	UniRef50_Q9ZVN2	704	63.99	411	142	3	46	1275	298	703	0.0	540
ABCDEF_52245	UniRef50_Q9FG87	0.0	Normal: 1878	635	1833	3814	1432	1376	BlastAnno: 1758	UniRef50_Q9FG87	529	55.35	486	204	4	4	1428	34	517	0.0	530
ABCDEF_16730	UniRef50_Q3EC11	0.0	Normal: 281	204	237	365	377	458	BlastAnno: 2092	UniRef50_Q3EC11	536	59.33	536	204	8	196	1785	3	530	0.0	661
ABCDEF_4655	UniRef50_A5BMG1	0.0	Normal: 2426	1856	3606	6958	4608	2755	BlastAnno: 2261	UniRef50_A5BMG1	481	68.37	528	112	10	263	1843	3	476	0.0	667
ABCDEF_13860	UniRef50_Q9C533	0.0	Normal: 588	220	570	1451	1338	310	BlastAnno: 2648	UniRef50_Q9C533	596	63.37	606	177	12	2226	511	1	595	0.0	558
ABCDEF_28294	UniRef50_Q9SYZ0	0.0	Normal: 1038	4070	1427	6745	877	2797	BlastAnno: 1785	UniRef50_Q9SYZ0	493	52.37	485	223	3	1598	162	5	487	0.0	533
ABCDEF_47718	UniRef50_Q9C533	0.0	Normal: 1599	236	1657	2093	1231	588	BlastAnno: 2680	UniRef50_Q9C533	596	65.02	626	165	14	2280	472	1	595	0.0	634
ABCDEF_45080	UniRef50_Q9SYZ0	0.0	Normal: 1005	592	1079	2360	3762	2217	BlastAnno: 2130	UniRef50_Q9SYZ0	493	57.85	484	197	3	302	1735	5	487	0.0	533
ABCDEF_25377	UniRef50_Q9C6L5	0.0	Normal: 37	2090	138	2573	1026	1298	BlastAnno: 1334	UniRef50_Q9C6L5	492	79.74	385	76	1	1155	1	6	388	0.0	654
ABCDEF_33470	UniRef50_Q9LW26	0.0	Normal: 172	98	156	260	391	149	BlastAnno: 2993	UniRef50_Q9LW26	701	48.96	627	301	5	655	2508	85	701	0.0	581
ABCDEF_40654	UniRef50_Q9H0A0	0.0	Normal: 638	213	530	318	318	286	BlastAnno: 2216	UniRef50_Q9H0A0	1025	56.87	721	279	6	2127	13	91	795	0.0	756
ABCDEF_33460	UniRef50_Q9ZVN2	0.0	Normal: 144	71	119	216	306	97	BlastAnno: 2691	UniRef50_Q9ZVN2	704	61.50	587	191	4	379	2133	83	636	0.0	715
ABCDEF_14442	UniRef50_Q9SYZ0	0.0	Normal: 341	684	439	1040	1601	552	BlastAnno: 1779	UniRef50_Q9SYZ0	493	63.12	480	171	2	1570	149	5	484	0.0	628
ABCDEF_52626	UniRef50_B6SYP9	0.0	Normal: 815	346	1131	3015	1537	108	BlastAnno: 1861	UniRef50_B6SYP9	494	55.02	438	189	3	1538	249	51	488	0.0	536
ABCDEF_644	UniRef50_Q9SB58	0.0	Normal: 704	444	913	969	1431	848	BlastAnno: 1777	UniRef50_Q9SB58	407	64.15	424	129	8	1572	316	1	406	0.0	554
ABCDEF_27087	UniRef50_Q52T38	0.0	Normal: 324	221	233	419	343	412	BlastAnno: 1631	UniRef50_Q52T38	620	75.95	420	91	1	1409	150	1	410	0.0	681
ABCDEF_34187	UniRef50_Q8L5Y5	0.0	Normal: 497	415	588	523	291	433	BlastAnno: 2677	UniRef50_Q8L5Y5	718	57.30	733	251	15	202	2274	1	713	0.0	683
ABCDEF_47966	UniRef50_Q9SYZ0	0.0	Normal: 577	934	326	580	682	3440	BlastAnno: 2177	UniRef50_Q9SYZ0	493	56.40	484	204	3	1780	347	5	487	0.0	535
ABCDEF_51189	UniRef50_Q52T38	0.0	Normal: 135	152	128	181	219	327	BlastAnno: 1766	UniRef50_Q52T38	620	68.09	470	137	3	80	1483	130	588	0.0	641
ABCDEF_41288	UniRef50_Q9SB58	0.0	Normal: 307	183	332	637	649	322	BlastAnno: 1403	UniRef50_Q9SB58	407	67.56	410	110	6	1401	184	15	405	0.0	523
ABCDEF_33461	UniRef50_Q9ZVN2	0.0	Normal: 142	84	140	206	283	119	BlastAnno: 2904	UniRef50_Q9ZVN2	704	60.98	574	201	6	2271	559	83	636	0.0	686
ABCDEF_14334	UniRef50_Q9SYZ0	0.0	Normal: 2263	129	1724	3469	866	283	BlastAnno: 2039	UniRef50_Q9SYZ0	493	63.22	484	171	2	369	1802	5	487	0.0	627
ABCDEF_24844	UniRef50_Q9FF86	0.0	Normal: 1857	3686	2657	21940	11467	4090	BlastAnno: 1640	UniRef50_Q9FF86	484	66.89	447	135	6	1492	185	2	446	0.0	575
ABCDEF_51190	UniRef50_Q52T38	0.0	Normal: 145	169	142	210	232	335	BlastAnno: 1891	UniRef50_Q52T38	620	70.04	514	141	3	73	1608	86	588	0.0	726
ABCDEF_14085	UniRef50_Q8L5Y5	0.0	Normal: 353	41	384	527	331	280	BlastAnno: 2362	UniRef50_Q8L5Y5	718	62.07	704	220	12	2360	348	24	713	0.0	676
ABCDEF_33458	UniRef50_Q9LW26	0.0	Normal: 158	78	130	240	342	106	BlastAnno: 2822	UniRef50_Q9LW26	701	46.72	655	302	5	400	2337	85	701	0.0	568
ABCDEF_25193	UniRef50_Q9C6L5	0.0	Normal: 3256	368	3548	6230	5109	1288	BlastAnno: 1786	UniRef50_Q9C6L5	492	65.83	480	156	4	1668	241	17	492	0.0	668
ABCDEF_33457	UniRef50_Q9ZVN2	0.0	Normal: 168	83	137	250	375	112	BlastAnno: 2823	UniRef50_Q9ZVN2	704	65.12	496	168	3	379	1860	83	575	0.0	648
ABCDEF_7634	UniRef50_Q9C6L5	0.0	Normal: 2543	9303	2711	11578	5533	10032	BlastAnno: 2064	UniRef50_Q9C6L5	492	52.76	489	225	3	1734	268	1	483	0.0	535
ABCDEF_299	UniRef50_Q71N54	0.0	Normal: 569	1147	511	900	649	1879	BlastAnno: 2017	UniRef50_Q71N54	535	61.73	533	198	5	338	1924	4	534	0.0	677
ABCDEF_27086	UniRef50_Q52T38	0.0	Normal: 784	470	617	843	893	976	BlastAnno: 2398	UniRef50_Q52T38	620	70.80	637	165	4	225	2129	1	618	0.0	887
ABCDEF_51188	UniRef50_Q52T38	0.0	Normal: 273	327	255	415	349	441	BlastAnno: 2324	UniRef50_Q52T38	620	71.30	568	150	3	2041	344	32	588	0.0	815
ABCDEF_44812	UniRef50_Q6DR03	0.0	Normal: 982	902	1005	1071	959	2999	BlastAnno: 2450	UniRef50_Q6DR03	565	54.97	644	198	17	2142	220	1	555	0.0	540

2.3.1.129(acyl-[acyl-carrier-protein]---UDP-N-acetylglucosamine O-acyltransferase;)

ABCDEF_26889	UniRef50_Q39SI8	5e-26	Normal: 37	107	31	0	69	71	BlastAnno: 836	UniRef50_Q39SI8	269	40.00	230	122	5	38	727	43	256	5e-26	110

2.4.-.-(No Name)

ABCDEF_48465	UniRef50_Q9M9U0	0.0	Normal: 993	813	1333	2641	3937	2315	BlastAnno: 1989	UniRef50_Q9M9U0	513	59.48	496	134	5	1850	555	19	511	0.0	589

2.4.1.-(Transferases;)

ABCDEF_18004	UniRef50_Q6YWK8	0.0	Normal: 1562	1736	2009	3452	4438	2400	BlastAnno: 2298	UniRef50_Q6YWK8	570	65.27	429	136	1	1941	694	57	485	0.0	608
ABCDEF_52379	UniRef50_Q9LHZ7	0.0	Normal: 4015	4921	4546	10520	13430	4731	BlastAnno: 3479	UniRef50_Q9LHZ7	1170	48.04	893	342	16	825	3242	299	1156	0.0	787
ABCDEF_48593	UniRef50_Q9SK82	0.0	Normal: 12	420	19	343	763	253	BlastAnno: 1834	UniRef50_Q9SK82	489	60.08	481	186	4	1714	278	10	486	0.0	633
ABCDEF_13375	UniRef50_Q8VZK9	0.0	Normal: 108	123	208	526	34	294	BlastAnno: 1738	UniRef50_Q8VZK9	729	53.62	552	234	8	94	1737	18	551	0.0	582
ABCDEF_40982	UniRef50_Q96301	0.0	Normal: 1037	706	819	1249	1084	920	BlastAnno: 3418	UniRef50_Q96301	914	79.60	902	173	3	243	2948	1	891	0.0	1506
ABCDEF_47674	UniRef50_Q9LHZ7	0.0	Normal: 274	92	202	599	505	280	BlastAnno: 2526	UniRef50_Q9LHZ7	1170	75.93	777	151	8	2260	2	15	779	0.0	1177
ABCDEF_34097	UniRef50_Q9LE59	0.0	Normal: 626	613	923	1797	1829	692	BlastAnno: 2521	UniRef50_Q9LE59	673	60.92	458	178	1	1687	317	215	672	0.0	630
ABCDEF_34098	UniRef50_Q9LE59	0.0	Normal: 457	464	697	1283	1312	539	BlastAnno: 2199	UniRef50_Q9LE59	673	60.92	458	178	1	1687	317	215	672	0.0	628
ABCDEF_10848	UniRef50_Q9M156	0.0	Normal: 157	2988	163	408	772	1566	BlastAnno: 1810	UniRef50_Q9M156	480	60.09	456	176	4	1700	336	4	454	0.0	553
ABCDEF_41182	UniRef50_Q9LHZ7	0.0	Normal: 150	2169	221	814	1407	495	BlastAnno: 3604	UniRef50_Q9LHZ7	1170	51.03	870	333	15	2822	456	299	1156	0.0	826
ABCDEF_40981	UniRef50_Q96301	0.0	Normal: 156	97	110	160	179	173	BlastAnno: 1225	UniRef50_Q96301	914	86.92	344	45	0	1112	81	394	737	0.0	652
ABCDEF_9506	UniRef50_Q9LHZ7	0.0	Normal: 1302	2128	1494	3356	4644	2206	BlastAnno: 3751	UniRef50_Q9LHZ7	1170	49.38	891	343	17	927	3359	299	1161	0.0	812
ABCDEF_9500	UniRef50_Q9LHZ7	0.0	Normal: 183	188	186	576	498	260	BlastAnno: 1717	UniRef50_Q9LHZ7	1170	47.98	644	234	14	3	1715	418	1033	0.0	571
ABCDEF_100	UniRef50_Q9M8Y0	0.0	Normal: 228	309	224	416	461	141	BlastAnno: 2109	UniRef50_Q9M8Y0	977	77.49	533	120	0	1610	12	50	582	0.0	884
ABCDEF_33659	UniRef50_Q9LHZ7	0.0	Normal: 1282	1948	2248	5826	10642	2083	BlastAnno: 4289	UniRef50_Q9LHZ7	1170	48.70	887	345	15	2955	538	299	1156	0.0	788
ABCDEF_37667	UniRef50_Q9LHZ7	0.0	Normal: 248	3617	251	1237	2078	970	BlastAnno: 3161	UniRef50_Q9LHZ7	1170	49.72	885	335	14	2671	260	301	1156	0.0	810
ABCDEF_47673	UniRef50_Q9LHZ7	0.0	Normal: 876	296	828	1628	1721	1172	BlastAnno: 3878	UniRef50_Q9LHZ7	1170	80.88	1161	186	8	3642	232	15	1163	0.0	1865

ABCDEF_99	UniRef50_Q9M8Y0	0.0	Normal: 272	330	222	398	423	145	BlastAnno: 2094	UniRef50_Q9M8Y0	977	77.36	561	127	0	1683	1	50	610	0.0	929
ABCDEF_20658	UniRef50_Q8VZK9	0.0	Normal: 373	33	193	64	503	141	BlastAnno: 2316	UniRef50_Q8VZK9	729	53.35	508	221	7	47	1564	11	504	0.0	559
ABCDEF_10183	UniRef50_A7XDQ9	0.0	Normal: 422	455	346	577	378	610	BlastAnno: 2362	UniRef50_A7XDQ9	684	50.94	693	307	12	281	2287	1	684	0.0	670
ABCDEF_30745	UniRef50_A7XDQ9	0.0	Normal: 1209	351	1031	1645	1226	635	BlastAnno: 2646	UniRef50_A7XDQ9	684	57.58	554	227	6	546	2189	133	684	0.0	664
ABCDEF_13336	UniRef50_Q7PC69	0.0	Normal: 148	563	194	957	1575	816	BlastAnno: 2584	UniRef50_Q7PC69	745	71.56	559	136	6	744	2351	187	745	0.0	730
ABCDEF_21426	UniRef50_Q8RU27	0.0	Normal: 2658	1979	3417	6157	9464	2599	BlastAnno: 1530	UniRef50_Q8RU27	366	89.44	360	34	2	1358	282	1	357	0.0	667
ABCDEF_71242	UniRef50_Q9ZV71	0.0	Normal: 134	122	179	480	754	119	BlastAnno: 1835	UniRef50_Q9ZV71	409	73.35	409	103	4	231	1439	1	409	0.0	587
ABCDEF_52020	UniRef50_Q6YWK8	0.0	Normal: 1635	1534	1975	4284	10353	2289	BlastAnno: 2394	UniRef50_Q6YWK8	570	58.37	514	185	2	403	1857	57	570	0.0	607
ABCDEF_51269	UniRef50_Q6YWK8	0.0	Normal: 147	85	103	21	33	438	BlastAnno: 1864	UniRef50_Q6YWK8	570	54.28	514	204	2	1646	198	57	570	0.0	586
ABCDEF_30753	UniRef50_Q9ZV71	0.0	Normal: 290	497	417	629	1013	634	BlastAnno: 1502	UniRef50_Q9ZV71	409	72.32	401	107	2	311	1501	1	401	0.0	590
ABCDEF_4233	UniRef50_Q9ZV71	0.0	Normal: 369	412	550	936	860	750	BlastAnno: 2004	UniRef50_Q9ZV71	409	72.13	409	110	2	370	1584	1	409	0.0	613
ABCDEF_17525	UniRef50_Q8RU27	0.0	Normal: 1500	1289	2149	3711	4446	1069	BlastAnno: 1507	UniRef50_Q8RU27	366	91.35	347	30	0	1371	331	3	349	0.0	671
ABCDEF_18005	UniRef50_Q6YWK8	0.0	Normal: 1842	2042	2515	4214	5208	2842	BlastAnno: 2305	UniRef50_Q6YWK8	570	59.14	514	180	2	1948	497	57	570	0.0	642
ABCDEF_41140	UniRef50_Q6YWK8	0.0	Normal: 86	1314	93	753	1787	2076	BlastAnno: 2346	UniRef50_Q6YWK8	570	60.00	510	174	2	431	1870	57	566	0.0	669
ABCDEF_18002	UniRef50_Q9LHZ7	0.0	Normal: 376	127	367	601	1190	520	BlastAnno: 1605	UniRef50_Q9LHZ7	1170	89.75	400	41	0	1603	404	771	1170	0.0	732
ABCDEF_13339	UniRef50_Q7PC69	0.0	Normal: 127	814	183	541	1135	1144	BlastAnno: 2610	UniRef50_Q7PC69	745	70.48	559	141	6	725	2329	187	745	0.0	729
ABCDEF_41141	UniRef50_Q6YWK8	0.0	Normal: 67	1107	84	708	1690	1854	BlastAnno: 2461	UniRef50_Q6YWK8	570	60.00	510	174	2	1916	477	57	566	0.0	668
ABCDEF_44583	UniRef50_Q9LE59	0.0	Normal: 681	561	559	947	1745	789	BlastAnno: 2470	UniRef50_Q9LE59	673	59.21	456	185	1	1749	385	214	669	0.0	621
ABCDEF_3869	UniRef50_Q9LE59	0.0	Normal: 333	393	415	809	907	754	BlastAnno: 2136	UniRef50_Q9LE59	673	59.05	613	227	4	1827	1	81	673	0.0	699
ABCDEF_28947	UniRef50_Q9SK82	0.0	Normal: 0	851	3	0	0	116	BlastAnno: 1567	UniRef50_Q9SK82	489	62.40	484	176	4	1524	85	1	482	0.0	652
ABCDEF_31119	UniRef50_Q9M8Y0	0.0	Normal: 2212	1279	1917	2933	3341	1803	BlastAnno: 3695	UniRef50_Q9M8Y0	977	81.08	925	174	1	392	3163	50	974	0.0	1583
ABCDEF_37311	UniRef50_Q9LE59	0.0	Normal: 175	133	191	549	421	366	BlastAnno: 1612	UniRef50_Q9LE59	673	62.96	486	153	4	1406	3	4	480	0.0	542
ABCDEF_24190	UniRef50_Q8L7F9	0.0	Normal: 397	265	492	583	553	371	BlastAnno: 2564	UniRef50_Q8L7F9	643	63.91	640	218	4	2353	473	1	640	0.0	850
ABCDEF_30548	UniRef50_Q9FH36	0.0	Normal: 17	916	27	151	420	126	BlastAnno: 1963	UniRef50_Q9FH36	535	72.47	534	138	3	102	1676	1	534	0.0	767
ABCDEF_27416	UniRef50_Q7PC69	0.0	Normal: 575	837	875	1344	2015	936	BlastAnno: 2454	UniRef50_Q7PC69	745	66.11	416	115	4	2337	1168	126	541	0.0	518
ABCDEF_48373	UniRef50_A7XDQ9	0.0	Normal: 1364	773	1336	1451	1791	1202	BlastAnno: 3042	UniRef50_A7XDQ9	684	63.68	691	234	6	2405	360	1	683	0.0	915
ABCDEF_3868	UniRef50_Q9LE59	0.0	Normal: 224	274	301	555	686	524	BlastAnno: 1670	UniRef50_Q9LE59	673	65.31	516	176	1	1556	9	161	673	0.0	696
ABCDEF_31288	UniRef50_Q940V3	0.0	Normal: 247	309	183	101	124	655	BlastAnno: 1658	UniRef50_Q940V3	470	56.01	466	197	7	1614	232	8	470	0.0	537
ABCDEF_52574	UniRef50_Q09332	0.0	Normal: 885	474	1068	1835	1853	816	BlastAnno: 3720	UniRef50_Q09332	1548	47.89	687	310	14	2551	533	859	1511	0.0	598
ABCDEF_79752	UniRef50_Q9ZV71	0.0	Normal: 158	183	245	400	379	352	BlastAnno: 1266	UniRef50_Q9ZV71	409	71.39	360	99	2	31	1098	1	360	0.0	526
ABCDEF_9509	UniRef50_Q9LHZ7	0.0	Normal: 1296	2171	1498	3410	4683	2232	BlastAnno: 3897	UniRef50_Q9LHZ7	1170	49.38	891	343	17	1073	3505	299	1161	0.0	812
ABCDEF_48200	UniRef50_Q6YWK8	0.0	Normal: 1768	576	3156	4431	6634	491	BlastAnno: 2274	UniRef50_Q6YWK8	570	57.98	514	185	4	291	1739	57	570	0.0	642
ABCDEF_24155	UniRef50_Q9ZVI7	0.0	Normal: 503	247	607	1676	1833	578	BlastAnno: 2708	UniRef50_Q9ZVI7	619	52.33	621	264	11	306	2123	15	618	0.0	601
ABCDEF_21246	UniRef50_Q9SKT6	0.0	Normal: 92	107	188	380	834	84	BlastAnno: 1791	UniRef50_Q9SKT6	536	56.61	484	204	5	123	1562	52	533	0.0	568
ABCDEF_32317	UniRef50_Q8RU27	0.0	Normal: 12	9	3	118	800	62	BlastAnno: 1076	UniRef50_Q8RU27	366	87.02	285	37	0	1075	221	64	348	0.0	535
ABCDEF_9502	UniRef50_Q9LHZ7	0.0	Normal: 610	486	568	1331	1403	591	BlastAnno: 1961	UniRef50_Q9LHZ7	1170	48.64	625	249	13	3	1742	564	1161	0.0	570
ABCDEF_23298	UniRef50_Q9FH36	0.0	Normal: 128	527	130	223	314	864	BlastAnno: 2832	UniRef50_Q9FH36	535	83.16	196	33	0	696	1283	340	535	0.0	357
ABCDEF_40595	UniRef50_O80898	0.0	Normal: 229	135	103	25	307	325	BlastAnno: 2413	UniRef50_O80898	757	44.68	761	378	13	95	2299	10	753	0.0	587
ABCDEF_63947	UniRef50_Q9SK82	0.0	Normal: 0	267	0	0	71	0	BlastAnno: 1446	UniRef50_Q9SK82	489	58.33	468	189	4	60	1445	1	468	0.0	592
ABCDEF_67847	UniRef50_Q9M8Y0	0.0	Normal: 794	476	871	1081	1401	868	BlastAnno: 1591	UniRef50_Q9M8Y0	977	84.86	370	56	0	1589	480	605	974	0.0	685
ABCDEF_65666	UniRef50_Q09332	0.0	Normal: 800	441	946	1606	1637	735	BlastAnno: 3518	UniRef50_Q09332	1548	47.54	671	305	13	1162	3087	859	1511	0.0	586
ABCDEF_38641	UniRef50_Q9FWA4	0.0	Normal: 674	967	1162	2603	4404	2475	BlastAnno: 1599	UniRef50_Q9FWA4	561	81.35	386	72	0	1	1158	176	561	0.0	641
ABCDEF_17914	UniRef50_Q9SKT6	0.0	Normal: 784	567	886	1447	1439	1424	BlastAnno: 2049	UniRef50_Q9SKT6	536	72.95	536	140	2	204	1796	1	536	0.0	785
ABCDEF_18286	UniRef50_Q6NQB7	0.0	Normal: 1131	1108	1225	1872	1722	3329	BlastAnno: 1752	UniRef50_Q6NQB7	393	73.80	397	81	6	1527	367	10	393	0.0	607
ABCDEF_119	UniRef50_Q8RU27	0.0	Normal: 1613	1155	2249	3558	7663	1775	BlastAnno: 1532	UniRef50_Q8RU27	366	88.33	360	38	2	1306	230	1	357	0.0	663
ABCDEF_47603	UniRef50_Q9M9Y5	0.0	Normal: 2609	8779	1664	1103	2085	22991	BlastAnno: 2830	UniRef50_Q9M9Y5	589	54.04	594	267	3	2475	694	1	588	0.0	654
ABCDEF_24192	UniRef50_Q8L7F9	0.0	Normal: 391	244	465	553	497	345	BlastAnno: 2477	UniRef50_Q8L7F9	643	58.99	434	165	4	212	1474	1	434	0.0	513
ABCDEF_17482	UniRef50_Q9SKT6	0.0	Normal: 615	379	816	1068	1470	852	BlastAnno: 2083	UniRef50_Q9SKT6	536	71.11	540	147	4	1915	311	1	536	0.0	786
ABCDEF_85909	UniRef50_Q8VZK9	0.0	Normal: 28	45	9	10	44	127	BlastAnno: 1473	UniRef50_Q8VZK9	729	55.37	475	198	6	1419	1	11	473	0.0	536
ABCDEF_9503	UniRef50_Q9LHZ7	0.0	Normal: 487	544	524	1242	1320	833	BlastAnno: 2087	UniRef50_Q9LHZ7	1170	50.35	574	236	8	57	1712	615	1161	0.0	567
ABCDEF_40594	UniRef50_O80898	0.0	Normal: 256	127	102	41	331	326	BlastAnno: 2417	UniRef50_O80898	757	45.76	719	348	12	213	2291	51	753	0.0	585
ABCDEF_17141	UniRef50_Q9LHZ7	0.0	Normal: 74	1734	69	380	701	333	BlastAnno: 3337	UniRef50_Q9LHZ7	1170	50.92	868	335	14	2539	179	299	1156	0.0	816
ABCDEF_33658	UniRef50_Q9LHZ7	0.0	Normal: 384	308	395	805	1003	602	BlastAnno: 2961	UniRef50_Q9LHZ7	1170	48.76	888	343	14	204	2621	299	1156	0.0	792
ABCDEF_20579	UniRef50_Q7PC69	0.0	Normal: 1007	795	1238	2311	2309	1345	BlastAnno: 3113	UniRef50_Q7PC69	745	59.97	767	229	14	2667	535	1	745	0.0	768
ABCDEF_31118	UniRef50_Q9M8Y0	0.0	Normal: 2135	1267	1900	3004	3329	1757	BlastAnno: 3787	UniRef50_Q9M8Y0	977	81.10	709	133	1	1132	3255	266	974	0.0	1217
ABCDEF_9499	UniRef50_Q9LHZ7	0.0	Normal: 205	207	215	632	538	275	BlastAnno: 1794	UniRef50_Q9LHZ7	1170	48.88	667	240	14	11	1792	395	1033	0.0	605
ABCDEF_48199	UniRef50_Q6YWK8	0.0	Normal: 1506	467	2429	3094	4233	439	BlastAnno: 1997	UniRef50_Q6YWK8	570	63.10	439	146	2	291	1559	57	495	0.0	610
ABCDEF_40983	UniRef50_Q96301	0.0	Normal: 406	261	343	392	415	433	BlastAnno: 2265	UniRef50_Q96301	914	79.17	504	99	1	114	1625	394	891	0.0	854
ABCDEF_17140	UniRef50_Q9LHZ7	0.0	Normal: 45	1049	49	216	386	208	BlastAnno: 2293	UniRef50_Q9LHZ7	1170	52.34	770	281	13	2138	57	397	1156	0.0	739
ABCDEF_18352	UniRef50_Q9FWA4	0.0	Normal: 2427	2688	2969	5519	6547	4855	BlastAnno: 2224	UniRef50_Q9FWA4	561	69.31	505	148	3	1887	382	61	561	0.0	683
ABCDEF_27767	UniRef50_Q9LHZ7	0.0	Normal: 2576	5141	2841	10249	14583	5031	BlastAnno: 3981	UniRef50_Q9LHZ7	1170	49.66	888	337	16	2927	504	299	1156	0.0	818
ABCDEF_33661	UniRef50_Q9LHZ7	0.0	Normal: 414	340	507	879	790	783	BlastAnno: 2185	UniRef50_Q9LHZ7	1170	48.66	748	277	14	2184	169	440	1156	0.0	662
ABCDEF_14685	UniRef50_A7XDQ9	0.0	Normal: 233	308	134	573	292	334	BlastAnno: 1947	UniRef50_A7XDQ9	684	56.84	468	186	6	569	1945	1	461	0.0	545
ABCDEF_20660	UniRef50_Q8VZK9	0.0	Normal: 22	48	13	15	52	144	BlastAnno: 1473	UniRef50_Q8VZK9	729	55.16	475	199	6	55	1473	11	473	0.0	537
ABCDEF_21315	UniRef50_Q9LHZ7	0.0	Normal: 3412	252	1450	0	79	1228	BlastAnno: 3765	UniRef50_Q9LHZ7	1170	63.78	1096	340	15	308	3508	70	1137	0.0	1333
ABCDEF_533	UniRef50_Q9C8W3	0.0	Normal: 230	204	269	505	411	301	BlastAnno: 1688	UniRef50_Q9C8W3	401	70.33	364	94	2	393	1475	47	399	0.0	555
ABCDEF_34099	UniRef50_Q9LE59	0.0	Normal: 478	491	735	1356	1350	548	BlastAnno: 2158	UniRef50_Q9LE59	673	60.92	458	178	1	1610	240	215	672	0.0	630
ABCDEF_47984	UniRef50_Q7PC69	0.0	Normal: 663	457	734	1362	2147	696	BlastAnno: 2838	UniRef50_Q7PC69	745	59.27	712	226	9	373	2316	34	745	0.0	702
ABCDEF_51451	UniRef50_Q9LE59	0.0	Normal: 1883	1782	2202	4337	4350	2667	BlastAnno: 2874	UniRef50_Q9LE59	673	74.16	685	165	6	2559	505	1	673	0.0	982
ABCDEF_13370	UniRef50_Q8VZK9	0.0	Normal: 290	87	317	377	105	258	BlastAnno: 2033	UniRef50_Q8VZK9	729	51.65	635	278	11	2031	154	112	726	0.0	612
ABCDEF_48239	UniRef50_Q9LHZ7	0.0	Normal: 511	163	422	1154	1316	510	BlastAnno: 2714	UniRef50_Q9LHZ7	1170	76.20	769	156	9	478	2712	41	806	0.0	1201
ABCDEF_13338	UniRef50_Q7PC69	0.0	Normal: 335	468	417	2639	4443	616	BlastAnno: 2525	UniRef50_Q7PC69	745	62.45	727	209	12	298	2292	21	745	0.0	781

ABCDEF_24193	UniRef50_Q8L7F9	0.0	Normal: 113	72	132	203	180	119	BlastAnno: 1679	UniRef50_Q8L7F9	643	60.54	479	176	4	212	1609	1	479	0.0	592
ABCDEF_48514	UniRef50_Q9ZV71	0.0	Normal: 540	580	601	771	1033	926	BlastAnno: 1920	UniRef50_Q9ZV71	409	78.14	398	85	2	1618	431	12	409	0.0	637
2.4.1.182(lipid-A-disaccharide synthase)																					
ABCDEF_27752	UniRef50_B6IST7	8e-55	Normal: 81	43	89	70	79	89	BlastAnno: 994	UniRef50_B6IST7	401	39.79	289	150	7	986	120	100	364	8e-55	192
2.4.1.44(lipopolysaccharide 3-alpha-galactosyltransferase;)																					
No Correspond Unigene																					
2.4.1.56(lipopolysaccharide N-acetylglucosaminyltransferase;)																					
No Correspond Unigene																					
2.4.1.58(lipopolysaccharide glucosyltransferase I;)																					
No Correspond Unigene																					
2.5.1.55(3-deoxy-8-phosphooctulonate synthase;)																					
ABCDEF_9723	UniRef50_Q9AV97	1e-179	Normal: 324	147	287	444	505	319	BlastAnno: 1682	UniRef50_Q9AV97	290	87.59	290	36	0	420	1289	1	290	1e-179	516
2.7.-.-(Transferases;)																					
ABCDEF_23367	UniRef50_Q9SYX2	0.0	Normal: 506	422	484	686	760	545	BlastAnno: 3695	UniRef50_Q9SYX2	1029	51.41	850	355	12	791	3214	194	1027	0.0	828
ABCDEF_45142	UniRef50_Q9MA15	0.0	Normal: 347	633	345	439	495	800	BlastAnno: 2401	UniRef50_Q9MA15	711	82.82	390	67	0	955	2124	298	687	0.0	655
ABCDEF_27575	UniRef50_Q8RWG1	0.0	Normal: 452	1600	437	812	911	2795	BlastAnno: 2280	UniRef50_Q8RWG1	682	86.14	599	74	1	1947	151	93	682	0.0	1073
ABCDEF_40580	UniRef50_Q9SYX2	0.0	Normal: 259	404	197	295	247	615	BlastAnno: 3187	UniRef50_Q9SYX2	1029	43.09	970	444	23	2788	44	51	967	0.0	620
ABCDEF_41280	UniRef50_Q9LRB0	0.0	Normal: 846	755	903	1378	1441	1073	BlastAnno: 2965	UniRef50_Q9LRB0	763	66.62	770	228	15	364	2622	1	758	0.0	939
ABCDEF_41441	UniRef50_Q9LRB0	0.0	Normal: 1112	790	991	1469	1539	1330	BlastAnno: 3089	UniRef50_Q9LRB0	763	64.25	772	241	15	2565	322	1	761	0.0	848
ABCDEF_65007	UniRef50_Q9MA15	0.0	Normal: 253	467	255	310	382	629	BlastAnno: 1826	UniRef50_Q9MA15	711	82.82	390	67	0	400	1569	298	687	0.0	655
ABCDEF_24582	UniRef50_Q9ASX5	0.0	Normal: 491	993	467	696	751	1720	BlastAnno: 1876	UniRef50_Q9ASX5	540	76.22	534	119	3	1813	221	2	530	0.0	834
2.7.1.-(Transferases;)																					
ABCDEF_19669	UniRef50_O04059	0.0	Normal: 81	66	74	92	96	76	BlastAnno: 1254	UniRef50_O04059	594	81.65	376	40	1	3	1130	84	430	0.0	571
ABCDEF_19662	UniRef50_O04059	0.0	Normal: 103	74	68	108	100	97	BlastAnno: 1553	UniRef50_O04059	594	89.77	430	44	0	1419	130	1	430	0.0	752
ABCDEF_47654	UniRef50_Q86C65	0.0	Normal: 2310	2222	1853	2946	2297	3238	BlastAnno: 6212	UniRef50_Q86C65	2380	48.37	2030	881	32	6204	241	476	2380	0.0	1769
ABCDEF_19665	UniRef50_O04059	0.0	Normal: 100	80	57	109	104	104	BlastAnno: 1759	UniRef50_O04059	594	89.21	380	41	0	1390	251	60	439	0.0	655
ABCDEF_19655	UniRef50_O04059	0.0	Normal: 197	122	157	224	212	154	BlastAnno: 2166	UniRef50_O04059	594	86.71	572	76	0	2032	317	1	572	0.0	914
ABCDEF_19659	UniRef50_O04059	0.0	Normal: 200	133	145	233	228	183	BlastAnno: 2278	UniRef50_O04059	594	86.89	572	75	0	2032	317	1	572	0.0	916
ABCDEF_19671	UniRef50_O04059	0.0	Normal: 130	125	102	159	150	145	BlastAnno: 1767	UniRef50_O04059	594	89.77	430	44	0	202	1491	1	430	0.0	751
2.7.1.130(tetraacyldisaccharide 4'-kinase;)																					
No Correspond Unigene																					
2.7.7.-(Transferases;)																					
ABCDEF_9555	UniRef50_Q9VXW2	0.0	Normal: 846	473	829	909	792	562	BlastAnno: 4157	UniRef50_Q9VXW2	1224	54.00	550	235	5	1481	3127	370	902	0.0	593
ABCDEF_9553	UniRef50_Q9VXW2	0.0	Normal: 268	145	246	297	265	213	BlastAnno: 3155	UniRef50_Q9VXW2	1224	54.00	550	235	5	2586	940	370	902	0.0	593
ABCDEF_40110	UniRef50_Q9VF56	0.0	Normal: 1387	922	1252	2141	1749	1299	BlastAnno: 6349	UniRef50_Q9VF56	2183	47.61	1947	918	19	428	5971	231	2174	0.0	1825
ABCDEF_16588	UniRef50_A3EWL3	0.0	Normal: 738	287	520	261	436	448	BlastAnno: 2835	UniRef50_A3EWL3	1105	50.40	744	329	11	2779	608	381	1104	0.0	682
ABCDEF_9552	UniRef50_Q9VXW2	0.0	Normal: 336	227	385	389	378	310	BlastAnno: 3817	UniRef50_Q9VXW2	1224	54.00	550	235	5	3248	1602	370	902	0.0	593
ABCDEF_9556	UniRef50_Q9VXW2	0.0	Normal: 196	108	169	204	178	166	BlastAnno: 2471	UniRef50_Q9VXW2	1224	60.35	454	174	2	2253	895	454	902	0.0	588
ABCDEF_14324	UniRef50_Q84WJ2	0.0	Normal: 538	44	228	5	65	118	BlastAnno: 2216	UniRef50_Q84WJ2	454	70.67	450	128	3	1585	245	1	449	0.0	650
ABCDEF_21043	UniRef50_Q9FJ26	0.0	Normal: 549	92	358	86	155	149	BlastAnno: 1708	UniRef50_Q9FJ26	407	65.96	379	108	3	195	1319	26	387	0.0	533
ABCDEF_9550	UniRef50_Q9VXW2	0.0	Normal: 230	131	215	291	238	198	BlastAnno: 3080	UniRef50_Q9VXW2	1224	54.00	550	235	5	570	2216	370	902	0.0	593
ABCDEF_9551	UniRef50_Q9VXW2	0.0	Normal: 244	147	229	314	258	214	BlastAnno: 3418	UniRef50_Q9VXW2	1224	54.00	550	235	5	570	2216	370	902	0.0	593
ABCDEF_9554	UniRef50_Q9VXW2	0.0	Normal: 750	401	750	826	637	518	BlastAnno: 3842	UniRef50_Q9VXW2	1224	54.00	550	235	5	1301	2947	370	902	0.0	593
ABCDEF_9549	UniRef50_Q9VXW2	0.0	Normal: 301	176	303	350	318	283	BlastAnno: 3569	UniRef50_Q9VXW2	1224	54.00	550	235	5	570	2216	370	902	0.0	593
ABCDEF_14323	UniRef50_Q84WJ2	0.0	Normal: 456	35	201	0	65	103	BlastAnno: 2163	UniRef50_Q84WJ2	454	70.44	450	129	3	579	1919	1	449	0.0	647
2.7.7.38(3-deoxy-manno-octulosonate cytidylyltransferase;)																					
ABCDEF_45044	UniRef50_Q9C920	6e-162	Normal: 511	349	708	1312	1776	836	BlastAnno: 1424	UniRef50_Q9C920	290	85.21	257	38	0	1240	470	34	290	6e-162	468
3.1.3.-(Hydrolases;)																					
ABCDEF_3128	UniRef50_Q9ZU49	5e-117	Normal: 118	143	108	91	149	56	BlastAnno: 1434	UniRef50_Q9ZU49	327	57.43	303	121	2	1184	294	20	320	5e-117	355
3.1.3.45(3-deoxy-manno-octulosonate-8-phosphatase)																					
No Correspond Unigene																					
3.5.1.-(Hydrolases;)																					
ABCDEF_41579	UniRef50_O81641	0.0	Normal: 384	122	265	320	313	150	BlastAnno: 1456	UniRef50_O81641	428	65.16	399	138	1	1313	120	23	421	0.0	543
ABCDEF_16924	UniRef50_Q8VYX0	0.0	Normal: 135	349	460	1325	685	527	BlastAnno: 2002	UniRef50_Q8VYX0	464	71.72	396	109	2	1612	428	68	461	0.0	549
ABCDEF_24290	UniRef50_O04373	0.0	Normal: 296	238	403	498	698	330	BlastAnno: 1710	UniRef50_O04373	440	65.24	443	151	3	67	1395	1	440	0.0	595
3.6.1.-(Hydrolases;)																					
ABCDEF_43677	UniRef50_B6ADS3	0.0	Normal: 265	202	294	363	341	233	BlastAnno: 2843	UniRef50_B6ADS3	1078	62.19	685	246	6	2017	2	246	930	0.0	842
ABCDEF_40935	UniRef50_A7ASE9	0.0	Normal: 1058	787	1081	1355	1280	1214	BlastAnno: 3814	UniRef50_A7ASE9	931	63.21	647	232	3	2359	428	285	928	0.0	811
ABCDEF_37918	UniRef50_Q8L831	0.0	Normal: 1344	592	1122	994	975	978	BlastAnno: 2888	UniRef50_Q8L831	772	71.74	768	214	1	209	2503	4	771	0.0	1152
ABCDEF_43683	UniRef50_B6ADS3	0.0	Normal: 186	143	232	259	238	201	BlastAnno: 1783	UniRef50_B6ADS3	1078	66.53	478	159	1	1	1431	600	1077	0.0	698
ABCDEF_34064	UniRef50_A7AVM7	0.0	Normal: 2140	1397	1695	2129	1483	1897	BlastAnno: 4387	UniRef50_A7AVM7	1016	49.83	891	398	12	2923	368	80	960	0.0	799
ABCDEF_43671	UniRef50_B6ADS3	0.0	Normal: 152	130	166	197	197	139	BlastAnno: 1982	UniRef50_B6ADS3	1078	65.79	640	211	5	80	1981	298	940	0.0	856
ABCDEF_43679	UniRef50_B6ADS3	0.0	Normal: 245	175	259	298	300	234	BlastAnno: 2502	UniRef50_B6ADS3	1078	65.13	780	263	5	2423	111	298	1077	0.0	1053
ABCDEF_43672	UniRef50_B6ADS3	0.0	Normal: 334	268	365	470	391	268	BlastAnno: 3191	UniRef50_B6ADS3	1078	62.19	685	246	6	1175	3190	246	930	0.0	842
5.-.-.-(Isomerases)																					
ABCDEF_37668	UniRef50_A8DJY4	1e-109	Normal: 240	328	262	459	844	1032	BlastAnno: 2373	UniRef50_A8DJY4	512	37.90	504	291	6	1825	359	12	508	1e-109	353
5.1.3.20(ADP-glyceromanno-heptose 6-epimerase)																					
No Correspond Unigene																					
6.-.-.-(No Name)																					
ABCDEF_30078	UniRef50_Q6L4L4	0.0	Normal: 873	690	641	788	1014	1012	BlastAnno: 3221	UniRef50_Q6L4L4	875	50.18	815	380	18	3086	669	78	875	0.0	721
ABCDEF_30143	UniRef50_Q6L4L4	0.0	Normal: 95	103	72	139	90	112	BlastAnno: 1586	UniRef50_Q6L4L4	875	61.24	485	163	9	1467	28	3	467	0.0	618
ABCDEF_30141	UniRef50_Q6L4L4	0.0	Normal: 1434	827	1232	1415	1424	1692	BlastAnno: 5949	UniRef50_Q6L4L4	875	50.00	876	376	23	5791	3221	3	835	0.0	802

>00550 Peptidoglycan biosynthesis

1.1.1.158(UDP-N-acetylmuramate dehydrogenase;)

ABCDEF_23349	UniRef50_Q6MAQ1	2e-65	Normal: 38	20	38	31	21	19	BlastAnno: 1176	UniRef50_Q6MAQ1	299	49.60	250	122	2	874	125	47	292	2e-65	218

2.3.2.-(Transferases;)

ABCDEF_41698	UniRef50_Q9M8T3	7e-62	Normal: 110	102	151	190	213	210	BlastAnno: 870	UniRef50_Q9M8T3	182	58.29	187	63	4	740	210	1	182	7e-62	202

2.3.2.10(UDP-N-acetylmuramoylpentapeptide-lysine N6-alanyltransferase;)

No Correspond Unigene

2.4.1.-(Transferases;)

ABCDEF_18004	UniRef50_Q6YWK8	0.0	Normal: 1562	1736	2009	3452	4438	2400	BlastAnno: 2298	UniRef50_Q6YWK8	570	65.27	429	136	1	1941	694	57	485	0.0	608
ABCDEF_52379	UniRef50_Q9LHZ7	0.0	Normal: 4015	4921	4546	10520	13430	4731	BlastAnno: 3479	UniRef50_Q9LHZ7	1170	48.04	893	342	16	825	3242	299	1156	0.0	787
ABCDEF_48593	UniRef50_Q9SK82	0.0	Normal: 12	420	19	343	763	253	BlastAnno: 1834	UniRef50_Q9SK82	489	60.08	481	186	4	1714	278	10	486	0.0	633
ABCDEF_13375	UniRef50_Q8VZK9	0.0	Normal: 108	123	208	526	34	294	BlastAnno: 1738	UniRef50_Q8VZK9	729	53.62	552	234	8	94	1737	18	551	0.0	582
ABCDEF_40982	UniRef50_Q96301	0.0	Normal: 1037	706	819	1249	1084	920	BlastAnno: 3418	UniRef50_Q96301	914	79.60	902	173	3	243	2948	1	891	0.0	1506
ABCDEF_47674	UniRef50_Q9LHZ7	0.0	Normal: 274	92	202	599	505	280	BlastAnno: 2526	UniRef50_Q9LHZ7	1170	75.93	777	151	8	2260	2	15	779	0.0	1177
ABCDEF_34097	UniRef50_Q9LE59	0.0	Normal: 626	613	923	1797	1829	692	BlastAnno: 2521	UniRef50_Q9LE59	673	60.92	458	178	1	1687	317	215	672	0.0	630
ABCDEF_34098	UniRef50_Q9LE59	0.0	Normal: 457	464	697	1283	1312	539	BlastAnno: 2199	UniRef50_Q9LE59	673	60.92	458	178	1	1687	317	215	672	0.0	628
ABCDEF_10848	UniRef50_Q9M156	0.0	Normal: 157	2988	163	408	772	1566	BlastAnno: 1810	UniRef50_Q9M156	480	60.09	456	176	4	1700	336	4	454	0.0	553
ABCDEF_41182	UniRef50_Q9LHZ7	0.0	Normal: 150	2169	221	814	1407	495	BlastAnno: 3604	UniRef50_Q9LHZ7	1170	51.03	870	333	15	2822	456	299	1156	0.0	826
ABCDEF_40981	UniRef50_Q96301	0.0	Normal: 156	97	110	160	179	173	BlastAnno: 1225	UniRef50_Q96301	914	86.92	344	45	0	1112	81	394	737	0.0	652
ABCDEF_9506	UniRef50_Q9LHZ7	0.0	Normal: 1302	2128	1494	3356	4644	2206	BlastAnno: 3751	UniRef50_Q9LHZ7	1170	49.38	891	343	17	927	3359	299	1161	0.0	812
ABCDEF_9500	UniRef50_Q9LHZ7	0.0	Normal: 183	188	186	576	498	260	BlastAnno: 1717	UniRef50_Q9LHZ7	1170	47.98	644	234	14	3	1715	418	1033	0.0	571
ABCDEF_100	UniRef50_Q9M8Y0	0.0	Normal: 228	309	224	416	461	141	BlastAnno: 2109	UniRef50_Q9M8Y0	977	77.49	533	120	0	1610	12	50	582	0.0	884
ABCDEF_33659	UniRef50_Q9LHZ7	0.0	Normal: 1282	1948	2248	5826	10642	2083	BlastAnno: 4289	UniRef50_Q9LHZ7	1170	48.70	887	345	15	2955	538	299	1156	0.0	788
ABCDEF_37667	UniRef50_Q9LHZ7	0.0	Normal: 248	3617	251	1237	2078	970	BlastAnno: 3161	UniRef50_Q9LHZ7	1170	49.72	885	335	14	2671	260	301	1156	0.0	810
ABCDEF_47673	UniRef50_Q9LHZ7	0.0	Normal: 876	296	828	1628	1721	1172	BlastAnno: 3878	UniRef50_Q9LHZ7	1170	80.88	1161	186	8	3642	232	15	1163	0.0	1865
ABCDEF_99	UniRef50_Q9M8Y0	0.0	Normal: 272	330	222	398	423	145	BlastAnno: 2094	UniRef50_Q9M8Y0	977	77.36	561	127	0	1683	1	50	610	0.0	929
ABCDEF_20658	UniRef50_Q8VZK9	0.0	Normal: 373	33	193	64	503	141	BlastAnno: 2316	UniRef50_Q8VZK9	729	53.35	508	221	7	47	1564	11	504	0.0	559
ABCDEF_10183	UniRef50_A7XDQ9	0.0	Normal: 422	455	346	577	378	610	BlastAnno: 2362	UniRef50_A7XDQ9	684	50.94	693	307	12	281	2287	1	684	0.0	670
ABCDEF_30745	UniRef50_A7XDQ9	0.0	Normal: 1209	351	1031	1645	1226	635	BlastAnno: 2646	UniRef50_A7XDQ9	684	57.58	554	227	6	546	2189	133	684	0.0	664
ABCDEF_13336	UniRef50_Q7PC69	0.0	Normal: 148	563	194	957	1575	816	BlastAnno: 2584	UniRef50_Q7PC69	745	71.56	559	136	6	744	2351	187	745	0.0	730
ABCDEF_21426	UniRef50_Q8RU27	0.0	Normal: 2658	1979	3417	6157	9464	2599	BlastAnno: 1530	UniRef50_Q8RU27	366	89.44	360	34	2	1358	282	1	357	0.0	667
ABCDEF_71242	UniRef50_Q9ZV71	0.0	Normal: 134	122	179	480	754	119	BlastAnno: 1835	UniRef50_Q9ZV71	409	73.35	409	103	4	231	1439	1	409	0.0	587
ABCDEF_52020	UniRef50_Q6YWK8	0.0	Normal: 1635	1534	1975	4284	10353	2289	BlastAnno: 2394	UniRef50_Q6YWK8	570	58.37	514	185	2	403	1857	57	570	0.0	607
ABCDEF_51269	UniRef50_Q6YWK8	0.0	Normal: 147	85	103	21	33	438	BlastAnno: 1864	UniRef50_Q6YWK8	570	54.28	514	204	2	1646	198	57	570	0.0	586
ABCDEF_30753	UniRef50_Q9ZV71	0.0	Normal: 290	497	417	629	1013	634	BlastAnno: 1502	UniRef50_Q9ZV71	409	72.32	401	107	2	311	1501	1	401	0.0	590
ABCDEF_4233	UniRef50_Q9ZV71	0.0	Normal: 369	412	550	936	860	750	BlastAnno: 2004	UniRef50_Q9ZV71	409	72.13	409	110	2	370	1584	1	409	0.0	613
ABCDEF_17525	UniRef50_Q8RU27	0.0	Normal: 1500	1289	2149	3711	4446	1069	BlastAnno: 1507	UniRef50_Q8RU27	366	91.35	347	30	0	1371	331	3	349	0.0	671
ABCDEF_18005	UniRef50_Q6YWK8	0.0	Normal: 1842	2042	2515	4214	5208	2842	BlastAnno: 2305	UniRef50_Q6YWK8	570	59.14	514	180	2	1948	497	57	570	0.0	642
ABCDEF_41140	UniRef50_Q6YWK8	0.0	Normal: 86	1314	93	772	1787	2076	BlastAnno: 2346	UniRef50_Q6YWK8	570	60.00	510	174	2	431	1870	57	566	0.0	669
ABCDEF_18002	UniRef50_Q9LHZ7	0.0	Normal: 376	127	367	601	1190	520	BlastAnno: 1605	UniRef50_Q9LHZ7	1170	89.75	400	41	0	1603	404	771	1170	0.0	732
ABCDEF_13339	UniRef50_Q7PC69	0.0	Normal: 127	814	183	541	1135	1144	BlastAnno: 2610	UniRef50_Q7PC69	745	70.48	559	141	6	725	2329	187	745	0.0	729
ABCDEF_41141	UniRef50_Q6YWK8	0.0	Normal: 67	1107	84	708	1690	1854	BlastAnno: 2461	UniRef50_Q6YWK8	570	60.00	510	174	2	1916	477	57	566	0.0	668
ABCDEF_44583	UniRef50_Q9LE59	0.0	Normal: 681	561	559	947	1745	789	BlastAnno: 2470	UniRef50_Q9LE59	673	59.21	456	185	1	1749	385	214	669	0.0	621
ABCDEF_3869	UniRef50_Q9LE59	0.0	Normal: 333	393	415	809	907	754	BlastAnno: 2136	UniRef50_Q9LE59	673	59.05	613	227	4	1827	1	81	673	0.0	699
ABCDEF_28947	UniRef50_Q9SK82	0.0	Normal: 0	851	3	0	0	116	BlastAnno: 1567	UniRef50_Q9SK82	489	62.40	484	176	4	1524	85	1	482	0.0	652
ABCDEF_31119	UniRef50_Q9M8Y0	0.0	Normal: 2212	1279	1917	2933	3341	1803	BlastAnno: 3695	UniRef50_Q9M8Y0	977	81.08	925	174	1	392	3163	50	974	0.0	1583
ABCDEF_37311	UniRef50_Q9LE59	0.0	Normal: 175	133	191	549	421	366	BlastAnno: 1612	UniRef50_Q9LE59	673	62.96	486	153	4	1406	3	4	480	0.0	542
ABCDEF_24190	UniRef50_Q8L7F9	0.0	Normal: 397	265	492	583	553	371	BlastAnno: 2564	UniRef50_Q8L7F9	643	63.91	640	218	4	2353	473	1	640	0.0	850
ABCDEF_30548	UniRef50_Q9FH36	0.0	Normal: 17	916	27	151	420	126	BlastAnno: 1963	UniRef50_Q9FH36	535	72.47	534	138	3	102	1676	1	534	0.0	767
ABCDEF_27416	UniRef50_Q7PC69	0.0	Normal: 575	837	875	1344	2015	936	BlastAnno: 2454	UniRef50_Q7PC69	745	66.11	416	115	4	2337	1168	126	541	0.0	518
ABCDEF_48373	UniRef50_A7XDQ9	0.0	Normal: 1364	773	1336	1451	1791	1202	BlastAnno: 3042	UniRef50_A7XDQ9	684	63.68	691	234	6	2405	360	1	683	0.0	915
ABCDEF_3868	UniRef50_Q9LE59	0.0	Normal: 224	274	301	555	686	524	BlastAnno: 1670	UniRef50_Q9LE59	673	65.31	516	176	1	1556	9	161	673	0.0	696
ABCDEF_31288	UniRef50_Q940V3	0.0	Normal: 247	309	183	101	124	655	BlastAnno: 1658	UniRef50_Q940V3	470	56.01	466	197	7	1614	232	8	470	0.0	537
ABCDEF_52574	UniRef50_Q09332	0.0	Normal: 885	474	1068	1835	1853	816	BlastAnno: 3720	UniRef50_Q09332	1548	47.89	687	310	14	2551	533	859	1511	0.0	598
ABCDEF_79752	UniRef50_Q9ZV71	0.0	Normal: 158	183	245	400	379	352	BlastAnno: 1266	UniRef50_Q9ZV71	409	71.39	360	99	2	31	1098	1	360	0.0	526
ABCDEF_9509	UniRef50_Q9LHZ7	0.0	Normal: 1296	2171	1498	3410	4683	2232	BlastAnno: 3897	UniRef50_Q9LHZ7	1170	49.38	891	343	17	1073	3505	299	1161	0.0	812
ABCDEF_48200	UniRef50_Q6YWK8	0.0	Normal: 1768	576	3156	4431	6634	491	BlastAnno: 2274	UniRef50_Q6YWK8	570	57.98	514	185	4	291	1739	57	570	0.0	642
ABCDEF_24155	UniRef50_Q9ZVI7	0.0	Normal: 503	247	607	1676	1833	578	BlastAnno: 2708	UniRef50_Q9ZVI7	619	52.33	621	264	11	306	2123	15	618	0.0	601
ABCDEF_21246	UniRef50_Q9SKT6	0.0	Normal: 92	107	188	380	834	84	BlastAnno: 1791	UniRef50_Q9SKT6	536	56.61	484	204	5	123	1562	52	533	0.0	568
ABCDEF_32317	UniRef50_Q8RU27	0.0	Normal: 12	9	3	118	800	62	BlastAnno: 1076	UniRef50_Q8RU27	366	87.02	285	37	0	1075	221	64	348	0.0	535
ABCDEF_9502	UniRef50_Q9LHZ7	0.0	Normal: 610	486	568	1331	1403	591	BlastAnno: 1961	UniRef50_Q9LHZ7	1170	48.64	625	249	13	3	1742	564	1161	0.0	570
ABCDEF_23298	UniRef50_Q9FH36	0.0	Normal: 128	527	130	223	314	864	BlastAnno: 2832	UniRef50_Q9FH36	535	83.16	196	33	0	696	1283	340	535	0.0	357
ABCDEF_40595	UniRef50_O80898	0.0	Normal: 229	135	103	25	307	325	BlastAnno: 2413	UniRef50_O80898	757	44.68	761	378	13	95	2299	10	753	0.0	587
ABCDEF_63947	UniRef50_Q9SK82	0.0	Normal: 0	267	0	0	71	0	BlastAnno: 1446	UniRef50_Q9SK82	489	58.33	468	189	4	60	1445	1	468	0.0	592
ABCDEF_67847	UniRef50_Q9M8Y0	0.0	Normal: 794	476	871	1081	1401	868	BlastAnno: 1591	UniRef50_Q9M8Y0	977	84.86	370	56	0	1589	480	605	974	0.0	685
ABCDEF_65666	UniRef50_Q09332	0.0	Normal: 800	441	946	1606	1637	735	BlastAnno: 3518	UniRef50_Q09332	1548	47.54	671	305	13	1162	3087	859	1511	0.0	586
ABCDEF_38641	UniRef50_Q9FWA4	0.0	Normal: 674	967	1162	2603	4404	2475	BlastAnno: 1599	UniRef50_Q9FWA4	561	81.35	386	72	0	1	1158	176	561	0.0	641
ABCDEF_17914	UniRef50_Q9SKT6	0.0	Normal: 784	567	886	1447	1439	1424	BlastAnno: 2049	UniRef50_Q9SKT6	536	72.95	536	140	2	204	1796	1	536	0.0	785
ABCDEF_18286	UniRef50_Q6NQB7	0.0	Normal: 1131	1108	1225	1872	1722	3329	BlastAnno: 1752	UniRef50_Q6NQB7	393	73.80	397	81	6	1527	367	10	393	0.0	607
ABCDEF_119	UniRef50_Q8RU27	0.0	Normal: 1613	1155	2249	3558	7663	1775	BlastAnno: 1532	UniRef50_Q8RU27	366	88.33	360	38	2	1306	230	1	357	0.0	663
ABCDEF_47603	UniRef50_Q9M9Y5	0.0	Normal: 2609	8779	1664	1103	2085	22991	BlastAnno: 2830	UniRef50_Q9M9Y5	589	54.04	594	267	3	2475	694	1	588	0.0	654
ABCDEF_24192	UniRef50_Q8L7F9	0.0	Normal: 391	244	465	553	497	345	BlastAnno: 2477	UniRef50_Q8L7F9	643	58.99	434	165	4	212	1474	1	434	0.0	513

ABCDEF_17482	UniRef50_Q9SKT6	0.0	Normal: 615	379	816	1068	1470	852	BlastAnno: 2083	UniRef50_Q9SKT6	536	71.11	540	147	4	1915	311	1	536	0.0	786
ABCDEF_85909	UniRef50_Q8VZK9	0.0	Normal: 28	45	9	10	44	127	BlastAnno: 1473	UniRef50_Q8VZK9	729	55.37	475	198	6	1419	1	11	473	0.0	536
ABCDEF_9503	UniRef50_Q9LHZ7	0.0	Normal: 487	544	524	1242	1320	833	BlastAnno: 2087	UniRef50_Q9LHZ7	1170	50.35	574	236	8	57	1712	615	1161	0.0	567
ABCDEF_40594	UniRef50_O80898	0.0	Normal: 256	127	102	41	331	326	BlastAnno: 2417	UniRef50_O80898	757	45.76	719	348	12	213	2291	51	753	0.0	585
ABCDEF_17141	UniRef50_Q9LHZ7	0.0	Normal: 74	1734	69	380	701	333	BlastAnno: 3337	UniRef50_Q9LHZ7	1170	50.92	868	335	14	2539	179	299	1156	0.0	816
ABCDEF_33658	UniRef50_Q9LHZ7	0.0	Normal: 384	308	395	805	1003	602	BlastAnno: 2961	UniRef50_Q9LHZ7	1170	48.76	888	343	14	204	2621	299	1156	0.0	792
ABCDEF_20579	UniRef50_Q7PC69	0.0	Normal: 1007	795	1238	2311	2309	1345	BlastAnno: 3113	UniRef50_Q7PC69	745	59.97	767	229	14	2667	535	1	745	0.0	768
ABCDEF_31118	UniRef50_Q9M8Y0	0.0	Normal: 2135	1267	1900	3004	3329	1757	BlastAnno: 3787	UniRef50_Q9M8Y0	977	81.10	709	133	1	1132	3255	266	974	0.0	1217
ABCDEF_9499	UniRef50_Q9LHZ7	0.0	Normal: 205	207	215	632	538	275	BlastAnno: 1794	UniRef50_Q9LHZ7	1170	48.88	667	240	14	11	1792	395	1033	0.0	605
ABCDEF_48199	UniRef50_Q6YWK8	0.0	Normal: 1506	467	2429	3094	4233	439	BlastAnno: 1997	UniRef50_Q6YWK8	570	63.10	439	146	2	291	1559	57	495	0.0	610
ABCDEF_40983	UniRef50_Q96301	0.0	Normal: 406	261	343	392	415	433	BlastAnno: 2265	UniRef50_Q96301	914	79.17	504	99	1	114	1625	394	891	0.0	854
ABCDEF_17140	UniRef50_Q9LHZ7	0.0	Normal: 45	1049	49	216	386	208	BlastAnno: 2293	UniRef50_Q9LHZ7	1170	52.34	770	281	13	2138	57	397	1156	0.0	739
ABCDEF_18352	UniRef50_Q9FWA4	0.0	Normal: 2427	2688	2969	5519	6547	4855	BlastAnno: 2224	UniRef50_Q9FWA4	561	69.31	505	148	3	1887	382	61	561	0.0	683
ABCDEF_27767	UniRef50_Q9LHZ7	0.0	Normal: 2576	5141	2841	10249	14583	5031	BlastAnno: 3981	UniRef50_Q9LHZ7	1170	49.66	888	337	16	2927	504	299	1156	0.0	818
ABCDEF_33661	UniRef50_Q9LHZ7	0.0	Normal: 414	340	507	879	790	783	BlastAnno: 2185	UniRef50_Q9LHZ7	1170	48.66	748	277	14	2184	169	440	1156	0.0	662
ABCDEF_14685	UniRef50_A7XDQ9	0.0	Normal: 233	308	134	573	292	334	BlastAnno: 1947	UniRef50_A7XDQ9	684	56.84	468	186	6	569	1945	1	461	0.0	545
ABCDEF_20660	UniRef50_Q8VZK9	0.0	Normal: 22	48	13	15	52	144	BlastAnno: 1473	UniRef50_Q8VZK9	729	55.16	475	199	6	55	1473	11	473	0.0	537
ABCDEF_21315	UniRef50_Q9LHZ7	0.0	Normal: 3412	252	1450	0	79	1228	BlastAnno: 3765	UniRef50_Q9LHZ7	1170	63.78	1096	340	15	308	3508	70	1137	0.0	1333
ABCDEF_533	UniRef50_Q9C8W3	0.0	Normal: 230	204	269	505	411	301	BlastAnno: 1688	UniRef50_Q9C8W3	401	70.33	364	94	2	393	1475	47	399	0.0	555
ABCDEF_34099	UniRef50_Q9LE59	0.0	Normal: 478	491	735	1356	1350	548	BlastAnno: 2158	UniRef50_Q9LE59	673	60.92	458	178	1	1610	240	215	672	0.0	630
ABCDEF_47984	UniRef50_Q7PC69	0.0	Normal: 663	457	734	1362	2147	696	BlastAnno: 2838	UniRef50_Q7PC69	745	59.27	712	226	9	373	2316	34	745	0.0	702
ABCDEF_51451	UniRef50_Q9LE59	0.0	Normal: 1883	1782	2202	4337	4350	2667	BlastAnno: 2874	UniRef50_Q9LE59	673	74.16	685	165	6	2559	505	1	673	0.0	982
ABCDEF_13370	UniRef50_Q8VZK9	0.0	Normal: 290	87	317	377	105	258	BlastAnno: 2033	UniRef50_Q8VZK9	729	51.65	635	278	11	2031	154	112	726	0.0	612
ABCDEF_48239	UniRef50_Q9LHZ7	0.0	Normal: 511	163	422	1154	1316	510	BlastAnno: 2714	UniRef50_Q9LHZ7	1170	76.20	769	156	9	478	2712	41	806	0.0	1201
ABCDEF_13338	UniRef50_Q7PC69	0.0	Normal: 335	468	417	2639	4443	616	BlastAnno: 2525	UniRef50_Q7PC69	745	62.45	727	209	12	298	2292	21	745	0.0	781
ABCDEF_24193	UniRef50_Q8L7F9	0.0	Normal: 113	72	132	203	180	119	BlastAnno: 1679	UniRef50_Q8L7F9	643	60.54	479	176	4	212	1609	1	479	0.0	592
ABCDEF_48514	UniRef50_Q9ZV71	0.0	Normal: 540	580	601	771	1033	926	BlastAnno: 1920	UniRef50_Q9ZV71	409	78.14	398	85	2	1618	431	12	409	0.0	637
2.4.1.129(peptidoglycan glycosyltransferase;)																					
ABCDEF_24474	UniRef50_A1HPE8	3e-29	Normal: 64	19	93	85	80	55	BlastAnno: 1051	UniRef50_A1HPE8	724	50.39	129	61	1	15	392	123	251	3e-29	125
2.4.1.227(undecaprenyldiphospho-muramoylpentapeptide beta-N-acetylglucosaminyltransferase;)																					
ABCDEF_12174	UniRef50_B9SFW3	2e-52	Normal: 19	8	19	17	0	2	BlastAnno: 364	UniRef50_B9SFW3	437	69.17	120	37	0	3	362	279	398	2e-52	178
2.5.1.7(UDP-N-acetylglucosamine 1-carboxyvinyltransferase;)																					
No Correspond Unigene																					
2.7.1.66(undecaprenol kinase;)																					
No Correspond Unigene																					
2.7.8.13(phospho-N-acetylmuramoyl-pentapeptide-transferase;)																					
ABCDEF_13385	UniRef50_B0BZI7	4e-58	Normal: 151	189	88	133	161	141	BlastAnno: 1372	UniRef50_B0BZI7	371	39.14	327	169	5	1367	408	51	354	4e-58	203
3.4.-.-(Hydrolases;)																					
ABCDEF_21645	UniRef50_Q1PF50	0.0	Normal: 654	512	669	909	994	888	BlastAnno: 1898	UniRef50_Q1PF50	417	66.35	416	138	1	1558	317	1	416	0.0	592
3.4.16.4(serine-type D-Ala-D-Ala carboxypeptidase;)																					
ABCDEF_13354	UniRef50_B6KGS5	2e-106	Normal: 284	526	325	602	535	454	BlastAnno: 3178	UniRef50_B6KGS5	1900	33.48	678	418	12	2903	915	4	663	2e-106	374
3.6.1.27(undecaprenyl-diphosphatase;)																					
No Correspond Unigene																					
6.3.2.10(UDP-N-acetylmuramoyl-tripeptide---D-alanyl-D-alanine ligase;)																					
ABCDEF_37880	UniRef50_B8I6H0	3e-63	Normal: 276	207	248	218	166	354	BlastAnno: 1803	UniRef50_B8I6H0	459	32.39	457	297	7	1603	254	4	455	3e-63	223
6.3.2.13(UDP-N-acetylmuramoyl-L-alanyl-D-glutamate---2,6-diaminopimelate ligase;)																					
ABCDEF_6535	UniRef50_Q0AYR3	1e-95	Normal: 319	180	299	293	170	743	BlastAnno: 2245	UniRef50_Q0AYR3	489	40.93	474	256	6	270	1691	17	466	1e-95	314
6.3.2.4(D-alanine---D-alanine ligase;)																					
ABCDEF_48167	UniRef50_Q11WL3	8e-43	Normal: 259	196	253	255	363	222	BlastAnno: 1996	UniRef50_Q11WL3	897	26.55	644	328	23	1991	183	350	889	8e-43	172
6.3.2.7(UDP-N-acetylmuramoyl-L-alanyl-D-glutamate---L-lysine ligase;)																					
No Correspond Unigene																					
6.3.2.8(UDP-N-acetylmuramate---L-alanine ligase;)																					
ABCDEF_59065	UniRef50_B8CWJ7	1e-17	Normal: 10	0	0	0	0	0	BlastAnno: 297	UniRef50_B8CWJ7	459	44.44	99	49	1	1	297	25	117	1e-17	82.8
6.3.2.9(UDP-N-acetylmuramoyl-L-alanine---D-glutamate ligase;)																					
ABCDEF_30168	UniRef50_Q1IKG8	6e-62	Normal: 85	52	102	178	150	135	BlastAnno: 1554	UniRef50_Q1IKG8	453	32.55	470	284	15	99	1493	2	443	6e-62	217
>00561 Glycerolipid metabolism																					
1.1.1.156(glycerol 2-dehydrogenase (NADP+);)																					
No Correspond Unigene																					
1.1.1.2(alcohol dehydrogenase (NADP+);)																					
ABCDEF_27285	UniRef50_O57380	5e-94	Normal: 119	81	124	95	252	18	BlastAnno: 1489	UniRef50_O57380	373	46.11	373	197	4	1279	161	3	371	5e-94	298
1.1.1.202(1,3-propanediol dehydrogenase;)																					
No Correspond Unigene																					
1.1.1.21(aldehyde reductase;)																					
ABCDEF_52723	UniRef50_Q6IMN8	5e-83	Normal: 1383	764	1621	1675	2066	2197	BlastAnno: 1221	UniRef50_Q6IMN8	297	44.09	313	153	5	135	1061	2	296	5e-83	265
1.1.1.6(glycerol dehydrogenase;)																					
No Correspond Unigene																					
1.1.1.72(glycerol dehydrogenase (NADP+);)																					
No Correspond Unigene																					
1.2.1.3(aldehyde dehydrogenase (NAD+);)																					
ABCDEF_52483	UniRef50_Q70E96	0.0	Normal: 28	851	41	0	3965	420	BlastAnno: 1750	UniRef50_Q70E96	484	54.56	482	219	0	107	1552	3	484	0.0	548
ABCDEF_17638	UniRef50_Q70E96	0.0	Normal: 580	718	749	2424	1317	775	BlastAnno: 1970	UniRef50_Q70E96	484	62.21	471	177	1	283	1692	2	472	0.0	625
ABCDEF_403	UniRef50_Q8W033	0.0	Normal: 69	355	98	39	223	1111	BlastAnno: 1749	UniRef50_Q8W033	550	65.96	473	161	0	1523	105	74	546	0.0	625

ABCDEF_45143	UniRef50_Q0WSF1	0.0	Normal: 1445	874	1200	1789	1940	1504	BlastAnno: 2450	UniRef50_Q0WSF1	596	79.97	594	115	2	2220	451	1	594	0.0	1015
2.3.1.134(galactolipid O-acyltransferase;)																					
No Correspond Unigene																					
2.3.1.141(galactosylacylglycerol O-acyltransferase;)																					
No Correspond Unigene																					
2.3.1.15(glycerol-3-phosphate O-acyltransferase;)																					
ABCDEF_31526	UniRef50_Q8GWG0	0.0	Normal: 854	674	926	1620	2095	1316	BlastAnno: 1574	UniRef50_Q8GWG0	376	86.52	371	49	1	1249	140	5	375	0.0	674
ABCDEF_26975	UniRef50_Q43307	0.0	Normal: 98	193	111	185	196	210	BlastAnno: 1701	UniRef50_Q43307	459	63.88	407	140	3	166	1365	53	459	0.0	546
ABCDEF_46632	UniRef50_Q9SHJ5	0.0	Normal: 0	815	0	0	0	6344	BlastAnno: 1852	UniRef50_Q9SHJ5	585	57.22	568	187	13	1682	141	20	585	0.0	561
ABCDEF_4142	UniRef50_Q9LMM0	0.0	Normal: 1866	2698	2190	6028	4010	4730	BlastAnno: 1842	UniRef50_Q9LMM0	503	77.31	498	110	2	82	1572	1	496	0.0	766
ABCDEF_26971	UniRef50_Q43307	0.0	Normal: 99	187	126	191	199	204	BlastAnno: 1761	UniRef50_Q43307	459	63.88	407	140	3	166	1365	53	459	0.0	546
ABCDEF_26970	UniRef50_Q43307	0.0	Normal: 105	209	133	218	233	251	BlastAnno: 1753	UniRef50_Q43307	459	63.88	407	140	3	187	1386	53	459	0.0	546
ABCDEF_14714	UniRef50_Q9LMM0	0.0	Normal: 50	5435	8	619	582	1352	BlastAnno: 1608	UniRef50_Q9LMM0	503	65.16	488	164	5	75	1526	10	495	0.0	642
2.3.1.158(phospholipid:diacylglycerol acyltransferase;)																					
ABCDEF_24418	UniRef50_Q9FNA9	0.0	Normal: 271	29	192	215	243	121	BlastAnno: 2015	UniRef50_Q9FNA9	671	76.82	548	125	1	1	1644	126	671	0.0	875
ABCDEF_38602	UniRef50_Q9FNA9	0.0	Normal: 2042	1712	1900	4134	4265	3258	BlastAnno: 2886	UniRef50_Q9FNA9	671	78.22	629	133	3	2534	648	47	671	0.0	1036
2.3.1.20(diacylglycerol O-acyltransferase;)																					
ABCDEF_2967	UniRef50_Q9SLD2	5e-180	Normal: 99	113	156	347	348	170	BlastAnno: 1170	UniRef50_Q9SLD2	520	76.47	323	72	2	1170	202	202	520	5e-180	520
2.3.1.22(2-acylglycerol O-acyltransferase;)																					
No Correspond Unigene																					
2.3.1.51(1-acylglycerol-3-phosphate O-acyltransferase;)																					
ABCDEF_20740	UniRef50_Q8LG50	0.0	Normal: 385	356	438	2043	1660	959	BlastAnno: 1640	UniRef50_Q8LG50	389	72.92	373	101	0	1425	307	14	386	0.0	577
ABCDEF_34747	UniRef50_Q8LG50	0.0	Normal: 1074	1007	1074	2022	1660	1853	BlastAnno: 1595	UniRef50_Q8LG50	389	72.49	378	104	0	1414	281	10	387	0.0	527
2.4.1.-(Transferases;)																					
ABCDEF_18004	UniRef50_Q6YWK8	0.0	Normal: 1562	1736	2009	3452	4438	2400	BlastAnno: 2298	UniRef50_Q6YWK8	570	65.27	429	136	1	1941	694	57	485	0.0	608
ABCDEF_52379	UniRef50_Q9LHZ7	0.0	Normal: 4015	4921	4546	10520	13430	4731	BlastAnno: 3479	UniRef50_Q9LHZ7	1170	48.04	893	342	16	825	3242	299	1156	0.0	787
ABCDEF_48593	UniRef50_Q9SK82	0.0	Normal: 12	420	19	343	763	253	BlastAnno: 1834	UniRef50_Q9SK82	489	60.08	481	186	4	1714	278	10	486	0.0	633
ABCDEF_13375	UniRef50_Q8VZK9	0.0	Normal: 108	123	208	526	34	294	BlastAnno: 1738	UniRef50_Q8VZK9	729	53.62	552	234	8	94	1737	18	551	0.0	582
ABCDEF_40982	UniRef50_Q96301	0.0	Normal: 1037	706	819	1249	1084	920	BlastAnno: 3418	UniRef50_Q96301	914	79.60	902	173	3	243	2948	1	891	0.0	1506
ABCDEF_47674	UniRef50_Q9LHZ7	0.0	Normal: 274	92	202	599	505	280	BlastAnno: 2526	UniRef50_Q9LHZ7	1170	75.93	777	151	8	2260	2	15	779	0.0	1177
ABCDEF_34097	UniRef50_Q9LE59	0.0	Normal: 626	613	923	1797	1829	692	BlastAnno: 2521	UniRef50_Q9LE59	673	60.92	458	178	1	1687	317	215	672	0.0	630
ABCDEF_34098	UniRef50_Q9LE59	0.0	Normal: 457	464	697	1283	1312	539	BlastAnno: 2199	UniRef50_Q9LE59	673	60.92	458	178	1	1687	317	215	672	0.0	628
ABCDEF_10848	UniRef50_Q9M156	0.0	Normal: 157	2988	163	408	772	1566	BlastAnno: 1810	UniRef50_Q9M156	480	60.09	456	176	4	1700	336	4	454	0.0	553
ABCDEF_41182	UniRef50_Q9LHZ7	0.0	Normal: 150	2169	221	814	1407	495	BlastAnno: 3604	UniRef50_Q9LHZ7	1170	51.03	870	333	15	2822	456	299	1156	0.0	826
ABCDEF_40981	UniRef50_Q96301	0.0	Normal: 156	97	110	160	179	173	BlastAnno: 1225	UniRef50_Q96301	914	86.92	344	45	0	1112	81	394	737	0.0	652
ABCDEF_9506	UniRef50_Q9LHZ7	0.0	Normal: 1302	2128	1494	3356	4644	2206	BlastAnno: 3751	UniRef50_Q9LHZ7	1170	49.38	891	343	17	927	3359	299	1161	0.0	812
ABCDEF_9500	UniRef50_Q9LHZ7	0.0	Normal: 183	188	186	576	498	260	BlastAnno: 1717	UniRef50_Q9LHZ7	1170	47.98	644	234	14	3	1715	418	1033	0.0	571
ABCDEF_100	UniRef50_Q9M8Y0	0.0	Normal: 228	309	224	416	461	141	BlastAnno: 2109	UniRef50_Q9M8Y0	977	77.49	533	120	0	1610	12	50	582	0.0	884
ABCDEF_33659	UniRef50_Q9LHZ7	0.0	Normal: 1282	1948	2248	5826	10642	2083	BlastAnno: 4289	UniRef50_Q9LHZ7	1170	48.70	887	345	15	2955	538	299	1156	0.0	788
ABCDEF_37667	UniRef50_Q9LHZ7	0.0	Normal: 248	3617	251	1237	2078	970	BlastAnno: 3161	UniRef50_Q9LHZ7	1170	49.72	885	335	14	2671	260	301	1156	0.0	810
ABCDEF_47673	UniRef50_Q9LHZ7	0.0	Normal: 876	296	828	1628	1721	1172	BlastAnno: 3878	UniRef50_Q9LHZ7	1170	80.88	1161	186	8	3642	232	15	1163	0.0	1865
ABCDEF_99	UniRef50_Q9M8Y0	0.0	Normal: 272	330	222	398	423	145	BlastAnno: 2094	UniRef50_Q9M8Y0	977	77.36	561	127	0	1683	1	50	610	0.0	929
ABCDEF_20658	UniRef50_Q8VZK9	0.0	Normal: 373	33	193	64	503	141	BlastAnno: 2316	UniRef50_Q8VZK9	729	53.35	508	221	7	47	1564	11	504	0.0	559
ABCDEF_10183	UniRef50_A7XDQ9	0.0	Normal: 422	455	346	577	378	610	BlastAnno: 2362	UniRef50_A7XDQ9	684	50.94	693	307	12	281	2287	1	684	0.0	670
ABCDEF_30745	UniRef50_A7XDQ9	0.0	Normal: 1209	351	1031	1645	1226	635	BlastAnno: 2646	UniRef50_A7XDQ9	684	57.58	554	227	6	546	2189	133	684	0.0	664
ABCDEF_13336	UniRef50_Q7PC69	0.0	Normal: 148	563	194	957	1575	816	BlastAnno: 2584	UniRef50_Q7PC69	745	71.56	559	136	6	744	2351	187	745	0.0	730
ABCDEF_21426	UniRef50_Q8RU27	0.0	Normal: 2658	1979	3417	6157	9464	2599	BlastAnno: 1530	UniRef50_Q8RU27	366	89.44	360	34	2	1358	282	1	357	0.0	667
ABCDEF_71242	UniRef50_Q9ZV71	0.0	Normal: 134	122	179	480	754	119	BlastAnno: 1835	UniRef50_Q9ZV71	409	73.35	409	103	4	231	1439	1	409	0.0	587
ABCDEF_52020	UniRef50_Q6YWK8	0.0	Normal: 1635	1534	1975	4284	10353	2289	BlastAnno: 2394	UniRef50_Q6YWK8	570	58.37	514	185	2	403	1857	57	570	0.0	607
ABCDEF_51269	UniRef50_Q6YWK8	0.0	Normal: 147	85	103	21	33	438	BlastAnno: 1864	UniRef50_Q6YWK8	570	54.28	514	204	2	1646	198	57	570	0.0	586
ABCDEF_30753	UniRef50_Q9ZV71	0.0	Normal: 290	497	417	629	1013	634	BlastAnno: 1502	UniRef50_Q9ZV71	409	72.32	401	107	2	311	1501	1	401	0.0	590
ABCDEF_4233	UniRef50_Q9ZV71	0.0	Normal: 369	412	550	936	860	750	BlastAnno: 2004	UniRef50_Q9ZV71	409	72.13	409	110	2	370	1584	1	409	0.0	613
ABCDEF_17525	UniRef50_Q8RU27	0.0	Normal: 1500	1289	2149	3711	4446	1069	BlastAnno: 1507	UniRef50_Q8RU27	366	91.35	347	30	0	1371	331	3	349	0.0	671
ABCDEF_18005	UniRef50_Q6YWK8	0.0	Normal: 1842	2042	2515	4214	5208	2842	BlastAnno: 2305	UniRef50_Q6YWK8	570	59.14	514	180	2	1948	497	57	570	0.0	642
ABCDEF_41140	UniRef50_Q6YWK8	0.0	Normal: 86	1314	93	753	1787	2076	BlastAnno: 2346	UniRef50_Q6YWK8	570	60.00	510	174	2	431	1870	57	566	0.0	669
ABCDEF_18002	UniRef50_Q9LHZ7	0.0	Normal: 376	127	367	601	1190	520	BlastAnno: 1605	UniRef50_Q9LHZ7	1170	89.75	400	41	0	1603	404	771	1170	0.0	732
ABCDEF_13339	UniRef50_Q7PC69	0.0	Normal: 127	814	183	541	1135	1144	BlastAnno: 2610	UniRef50_Q7PC69	745	70.48	559	141	6	725	2329	187	745	0.0	729
ABCDEF_41141	UniRef50_Q6YWK8	0.0	Normal: 67	1107	84	708	1690	1854	BlastAnno: 2461	UniRef50_Q6YWK8	570	60.00	510	174	2	1916	477	57	566	0.0	668
ABCDEF_44583	UniRef50_Q9LE59	0.0	Normal: 681	561	559	947	1745	789	BlastAnno: 2470	UniRef50_Q9LE59	673	59.21	456	185	1	1749	385	214	669	0.0	621
ABCDEF_3869	UniRef50_Q9LE59	0.0	Normal: 333	393	415	809	907	754	BlastAnno: 2136	UniRef50_Q9LE59	673	59.05	613	227	4	1827	1	81	673	0.0	699
ABCDEF_28947	UniRef50_Q9SK82	0.0	Normal: 0	851	3	0	0	116	BlastAnno: 1567	UniRef50_Q9SK82	489	62.40	484	176	4	1524	85	1	482	0.0	652
ABCDEF_31119	UniRef50_Q9M8Y0	0.0	Normal: 2212	1279	1917	2933	3341	1803	BlastAnno: 3695	UniRef50_Q9M8Y0	977	81.08	925	174	1	392	3163	50	974	0.0	1583
ABCDEF_37311	UniRef50_Q9LE59	0.0	Normal: 175	133	191	549	421	366	BlastAnno: 1612	UniRef50_Q9LE59	673	62.96	486	153	4	1406	3	4	480	0.0	542
ABCDEF_24190	UniRef50_Q8L7F9	0.0	Normal: 397	265	492	583	553	371	BlastAnno: 2564	UniRef50_Q8L7F9	643	63.91	640	218	4	2353	473	1	640	0.0	850
ABCDEF_30548	UniRef50_Q9FH36	0.0	Normal: 17	916	27	151	420	126	BlastAnno: 1963	UniRef50_Q9FH36	535	72.47	534	138	3	102	1676	1	534	0.0	767
ABCDEF_27416	UniRef50_Q7PC69	0.0	Normal: 575	837	875	1344	2015	936	BlastAnno: 2454	UniRef50_Q7PC69	745	66.11	416	115	4	2337	1168	126	541	0.0	518
ABCDEF_48373	UniRef50_A7XDQ9	0.0	Normal: 1364	773	1336	1451	1791	1202	BlastAnno: 3042	UniRef50_A7XDQ9	684	63.68	691	234	6	2405	360	1	683	0.0	915
ABCDEF_3868	UniRef50_Q9LE59	0.0	Normal: 224	274	301	555	686	524	BlastAnno: 1670	UniRef50_Q9LE59	673	65.31	516	176	1	1556	9	161	673	0.0	696
ABCDEF_31288	UniRef50_Q940V3	0.0	Normal: 247	309	183	101	124	655	BlastAnno: 1658	UniRef50_Q940V3	470	56.01	466	197	7	1614	232	8	470	0.0	537
ABCDEF_52574	UniRef50_Q09332	0.0	Normal: 885	474	1068	1835	1853	816	BlastAnno: 3720	UniRef50_Q09332	1548	47.89	687	310	14	2551	533	859	1511	0.0	598
ABCDEF_79752	UniRef50_Q9ZV71	0.0	Normal: 158	183	245	400	379	352	BlastAnno: 1266	UniRef50_Q9ZV71	409	71.39	360	99	2	31	1098	1	360	0.0	526
ABCDEF_9509	UniRef50_Q9LHZ7	0.0	Normal: 1296	2171	1498	3410	4683	2232	BlastAnno: 3897	UniRef50_Q9LHZ7	1170	49.38	891	343	17	1073	3505	299	1161	0.0	812

ABCDEF_48200	UniRef50_Q6YWK8	0.0	Normal: 1768	576	3156	4431	6634	491	BlastAnno: 2274	UniRef50_Q6YWK8	570	57.98	514	185	4	291	1739	57	570	0.0	642
ABCDEF_24155	UniRef50_Q9ZVI7	0.0	Normal: 503	247	607	1676	1833	578	BlastAnno: 2708	UniRef50_Q9ZVI7	619	52.33	621	264	11	306	2123	15	618	0.0	601
ABCDEF_21246	UniRef50_Q9SKT6	0.0	Normal: 92	107	188	380	834	84	BlastAnno: 1791	UniRef50_Q9SKT6	536	56.61	484	204	5	123	1562	52	533	0.0	568
ABCDEF_32317	UniRef50_Q8RU27	0.0	Normal: 12	9	3	118	800	62	BlastAnno: 1076	UniRef50_Q8RU27	366	87.02	285	37	0	1075	221	64	348	0.0	535
ABCDEF_9502	UniRef50_Q9LHZ7	0.0	Normal: 610	486	568	1331	1403	591	BlastAnno: 1961	UniRef50_Q9LHZ7	1170	48.64	625	249	13	3	1742	564	1161	0.0	570
ABCDEF_23298	UniRef50_Q9FH36	0.0	Normal: 128	527	130	223	314	864	BlastAnno: 2832	UniRef50_Q9FH36	535	83.16	196	33	0	696	1283	340	535	0.0	357
ABCDEF_40595	UniRef50_O80898	0.0	Normal: 229	135	103	25	307	325	BlastAnno: 2413	UniRef50_O80898	757	44.68	761	378	13	95	2299	10	753	0.0	587
ABCDEF_63947	UniRef50_Q9SK82	0.0	Normal: 0	267	0	0	71	0	BlastAnno: 1446	UniRef50_Q9SK82	489	58.33	468	189	4	60	1445	1	468	0.0	592
ABCDEF_67847	UniRef50_Q9M8Y0	0.0	Normal: 794	476	871	1081	1401	868	BlastAnno: 1591	UniRef50_Q9M8Y0	977	84.86	370	56	0	1589	480	605	974	0.0	685
ABCDEF_65666	UniRef50_Q09332	0.0	Normal: 800	441	946	1606	1637	735	BlastAnno: 3518	UniRef50_Q09332	1548	47.54	671	305	13	1162	3087	859	1511	0.0	586
ABCDEF_38641	UniRef50_Q9FWA4	0.0	Normal: 674	967	1162	2603	4404	2475	BlastAnno: 1599	UniRef50_Q9FWA4	561	81.35	386	72	0	1	1158	176	561	0.0	641
ABCDEF_17914	UniRef50_Q9SKT6	0.0	Normal: 784	567	886	1447	1439	1424	BlastAnno: 2049	UniRef50_Q9SKT6	536	72.95	536	140	2	204	1796	1	536	0.0	785
ABCDEF_18286	UniRef50_Q6NQB7	0.0	Normal: 1131	1108	1225	1872	1722	3329	BlastAnno: 1752	UniRef50_Q6NQB7	393	73.80	397	81	6	1527	367	10	393	0.0	607
ABCDEF_119	UniRef50_Q8RU27	0.0	Normal: 1613	1155	2249	3558	7663	1775	BlastAnno: 1532	UniRef50_Q8RU27	366	88.33	360	38	2	1306	230	1	357	0.0	663
ABCDEF_47603	UniRef50_Q9M9Y5	0.0	Normal: 2609	8779	1664	1103	2085	22991	BlastAnno: 2830	UniRef50_Q9M9Y5	589	54.04	594	267	3	2475	694	1	588	0.0	654
ABCDEF_24192	UniRef50_Q8L7F9	0.0	Normal: 391	244	465	553	497	345	BlastAnno: 2477	UniRef50_Q8L7F9	643	58.99	434	165	4	212	1474	1	434	0.0	513
ABCDEF_17482	UniRef50_Q9SKT6	0.0	Normal: 615	379	816	1068	1470	852	BlastAnno: 2083	UniRef50_Q9SKT6	536	71.11	540	147	4	1915	311	1	536	0.0	786
ABCDEF_85909	UniRef50_Q8VZK9	0.0	Normal: 28	45	9	10	44	127	BlastAnno: 1473	UniRef50_Q8VZK9	729	55.37	475	198	6	1419	1	11	473	0.0	536
ABCDEF_9503	UniRef50_Q9LHZ7	0.0	Normal: 487	544	524	1242	1320	833	BlastAnno: 2087	UniRef50_Q9LHZ7	1170	50.35	574	236	8	57	1712	615	1161	0.0	567
ABCDEF_40594	UniRef50_O80898	0.0	Normal: 256	127	102	41	331	326	BlastAnno: 2417	UniRef50_O80898	757	45.76	719	348	12	213	2291	51	753	0.0	585
ABCDEF_17141	UniRef50_Q9LHZ7	0.0	Normal: 74	1734	69	380	701	333	BlastAnno: 3337	UniRef50_Q9LHZ7	1170	50.92	868	335	14	2539	179	299	1156	0.0	816
ABCDEF_33658	UniRef50_Q9LHZ7	0.0	Normal: 384	308	395	805	1003	602	BlastAnno: 2961	UniRef50_Q9LHZ7	1170	48.76	888	343	14	204	2621	299	1156	0.0	792
ABCDEF_20579	UniRef50_Q7PC69	0.0	Normal: 1007	795	1238	2311	2309	1345	BlastAnno: 3113	UniRef50_Q7PC69	745	59.97	767	229	14	2667	535	1	745	0.0	768
ABCDEF_31118	UniRef50_Q9M8Y0	0.0	Normal: 2135	1267	1900	3004	3329	1757	BlastAnno: 3787	UniRef50_Q9M8Y0	977	81.10	709	133	1	1132	3255	266	974	0.0	1217
ABCDEF_9499	UniRef50_Q9LHZ7	0.0	Normal: 205	207	215	632	538	275	BlastAnno: 1794	UniRef50_Q9LHZ7	1170	48.88	667	240	14	11	1792	395	1033	0.0	605
ABCDEF_48199	UniRef50_Q6YWK8	0.0	Normal: 1506	467	2429	3094	4233	439	BlastAnno: 1997	UniRef50_Q6YWK8	570	63.10	439	146	2	291	1559	57	495	0.0	610
ABCDEF_40983	UniRef50_Q96301	0.0	Normal: 406	261	343	392	415	433	BlastAnno: 2265	UniRef50_Q96301	914	79.17	504	99	1	114	1625	394	891	0.0	854
ABCDEF_17140	UniRef50_Q9LHZ7	0.0	Normal: 45	1049	49	216	386	208	BlastAnno: 2293	UniRef50_Q9LHZ7	1170	52.34	770	281	13	2138	57	397	1156	0.0	739
ABCDEF_18352	UniRef50_Q9FWA4	0.0	Normal: 2427	2688	2969	5519	6547	4855	BlastAnno: 2224	UniRef50_Q9FWA4	561	69.31	505	148	3	1887	382	61	561	0.0	683
ABCDEF_27767	UniRef50_Q9LHZ7	0.0	Normal: 2576	5141	2841	10249	14583	5031	BlastAnno: 3981	UniRef50_Q9LHZ7	1170	49.66	888	337	16	2927	504	299	1156	0.0	818
ABCDEF_33661	UniRef50_Q9LHZ7	0.0	Normal: 414	340	507	879	790	783	BlastAnno: 2185	UniRef50_Q9LHZ7	1170	48.66	748	277	14	2184	169	440	1156	0.0	662
ABCDEF_14685	UniRef50_A7XDQ9	0.0	Normal: 233	308	134	573	292	334	BlastAnno: 1947	UniRef50_A7XDQ9	684	56.84	468	186	6	569	1945	1	461	0.0	545
ABCDEF_20660	UniRef50_Q8VZK9	0.0	Normal: 22	48	13	15	52	144	BlastAnno: 1473	UniRef50_Q8VZK9	729	55.16	475	199	6	55	1473	11	473	0.0	537
ABCDEF_21315	UniRef50_Q9LHZ7	0.0	Normal: 3412	252	1450	0	79	1228	BlastAnno: 3765	UniRef50_Q9LHZ7	1170	63.78	1096	340	15	308	3508	70	1137	0.0	1333
ABCDEF_533	UniRef50_Q9C8W3	0.0	Normal: 230	204	269	505	411	301	BlastAnno: 1688	UniRef50_Q9C8W3	401	70.33	364	94	2	393	1475	47	399	0.0	555
ABCDEF_34099	UniRef50_Q9LE59	0.0	Normal: 478	491	735	1356	1350	548	BlastAnno: 2158	UniRef50_Q9LE59	673	60.92	458	178	1	1610	240	215	672	0.0	630
ABCDEF_47984	UniRef50_Q7PC69	0.0	Normal: 663	457	734	1362	2147	696	BlastAnno: 2838	UniRef50_Q7PC69	745	59.27	712	226	9	373	2316	34	745	0.0	702
ABCDEF_51451	UniRef50_Q9LE59	0.0	Normal: 1883	1782	2202	4337	4350	2667	BlastAnno: 2874	UniRef50_Q9LE59	673	74.16	685	165	6	2559	505	1	673	0.0	982
ABCDEF_13370	UniRef50_Q8VZK9	0.0	Normal: 290	87	317	377	105	258	BlastAnno: 2033	UniRef50_Q8VZK9	729	51.65	635	278	11	2031	154	112	726	0.0	612
ABCDEF_48239	UniRef50_Q9LHZ7	0.0	Normal: 511	163	422	1154	1316	510	BlastAnno: 2714	UniRef50_Q9LHZ7	1170	76.20	769	156	9	478	2712	41	806	0.0	1201
ABCDEF_13338	UniRef50_Q7PC69	0.0	Normal: 335	468	417	2639	4443	616	BlastAnno: 2525	UniRef50_Q7PC69	745	62.45	727	209	12	298	2292	21	745	0.0	781
ABCDEF_24193	UniRef50_Q8L7F9	0.0	Normal: 113	72	132	203	180	119	BlastAnno: 1679	UniRef50_Q8L7F9	643	60.54	479	176	4	212	1609	1	479	0.0	592
ABCDEF_48514	UniRef50_Q9ZV71	0.0	Normal: 540	580	601	771	1033	926	BlastAnno: 1920	UniRef50_Q9ZV71	409	78.14	398	85	2	1618	431	12	409	0.0	637

2.4.1.157(1,2-diacylglycerol 3-glucosyltransferase;)

No Correspond Unigene

2.4.1.184(galactolipid galactosyltransferase;)

No Correspond Unigene

2.4.1.241(digalactosyldiacylglycerol synthase;)

ABCDEF_7051	UniRef50_Q8W1S1	0.0	Normal: 519	609	349	353	277	2469	BlastAnno: 2796	UniRef50_Q8W1S1	473	54.90	459	203	1	1109	2485	6	460	0.0	559
ABCDEF_85672	UniRef50_Q8W1S1	0.0	Normal: 345	448	263	228	186	1827	BlastAnno: 2117	UniRef50_Q8W1S1	473	54.90	459	203	1	1688	312	6	460	0.0	559
ABCDEF_7444	UniRef50_Q8W1S1	0.0	Normal: 417	484	385	683	772	784	BlastAnno: 1871	UniRef50_Q8W1S1	473	66.59	461	140	4	1636	290	4	462	0.0	651

2.4.1.46(monogalactosyldiacylglycerol synthase;)

ABCDEF_24265	UniRef50_O81770	0.0	Normal: 106	79	223	203	278	231	BlastAnno: 1334	UniRef50_O81770	533	79.15	355	74	0	1258	194	174	528	0.0	583
ABCDEF_24266	UniRef50_O81770	0.0	Normal: 303	214	517	596	746	577	BlastAnno: 2232	UniRef50_O81770	533	73.38	462	119	3	1572	199	67	528	0.0	666
ABCDEF_86703	UniRef50_O81770	0.0	Normal: 75	52	136	130	198	165	BlastAnno: 1440	UniRef50_O81770	533	70.25	400	74	1	1373	174	174	528	0.0	561
ABCDEF_41336	UniRef50_O82730	0.0	Normal: 219	108	174	240	156	51	BlastAnno: 1649	UniRef50_O82730	468	80.10	417	83	0	1251	1	50	466	0.0	671
ABCDEF_24788	UniRef50_O81770	0.0	Normal: 578	590	601	670	721	1759	BlastAnno: 2004	UniRef50_O81770	533	77.71	462	99	4	385	1758	67	528	0.0	722
ABCDEF_24267	UniRef50_O81770	0.0	Normal: 233	167	424	429	634	511	BlastAnno: 2345	UniRef50_O81770	533	66.86	507	119	4	642	2150	67	528	0.0	645
ABCDEF_3838	UniRef50_O82730	0.0	Normal: 105	49	61	309	542	95	BlastAnno: 1558	UniRef50_O82730	468	78.86	421	89	0	1278	16	48	468	0.0	658
ABCDEF_41335	UniRef50_O82730	0.0	Normal: 151	69	115	160	100	26	BlastAnno: 1420	UniRef50_O82730	468	80.78	333	64	0	1190	192	50	382	0.0	536

2.4.99.5(galactosyldiacylglycerol alpha-2,3-sialyltransferase)

No Correspond Unigene

2.7.1.107(diacylglycerol kinase;)

ABCDEF_20845	UniRef50_Q9FFN7	0.0	Normal: 282	208	272	189	491	288	BlastAnno: 1902	UniRef50_Q9FFN7	712	53.43	554	233	7	1901	249	181	712	0.0	570
ABCDEF_24649	UniRef50_Q9FFN7	0.0	Normal: 66	112	93	228	428	96	BlastAnno: 1529	UniRef50_Q9FFN7	712	77.72	386	84	1	49	1206	329	712	0.0	596

2.7.1.29(glycerone kinase;)

ABCDEF_19659	UniRef50_Q3LXA3	6e-108	Normal: 200	133	145	233	228	183	BlastAnno: 2278	UniRef50_Q3LXA3	575	39.45	578	322	11	2026	317	1	558	6e-108	349

2.7.1.30(glycerol kinase;)

ABCDEF_41498	UniRef50_Q54VT8	2e-176	Normal: 200	1029	207	377	343	2157	BlastAnno: 1836	UniRef50_Q54VT8	539	49.45	550	224	6	1662	124	4	536	2e-176	520

2.7.1.31(glycerate kinase;)

ABCDEF_10301	UniRef50_Q944I4	0.0	Normal: 259	552	306	527	545	1533	BlastAnno: 1595	UniRef50_Q944I4	456	72.85	372	99	1	272	1387	86	455	0.0	580
ABCDEF_10303	UniRef50_Q944I4	0.0	Normal: 266	510	294	565	522	1374	BlastAnno: 1563	UniRef50_Q944I4	456	68.43	396	99	2	1292	105	86	455	0.0	565

2.7.1.94(acylglycerol kinase;)																						
	No Correspond Unigene																					
2.7.8.20(phosphatidylglycerol---membrane-oligosaccharide glycerophosphotransferase;)																						
	No Correspond Unigene																					
3.1.1.23(acylglycerol lipase;)																						
	No Correspond Unigene																					
3.1.1.26(galactolipase;)																						
	ABCDEF_23924	UniRef50_Q9MA46	1e-51	Normal: 21	91	43	81	73	669	BlastAnno: 711	UniRef50_Q9MA46	471	45.61	239	100	7	1	699	196	410	1e-51	182
3.1.1.3(triacylglycerol lipase;)																						
	ABCDEF_24860	UniRef50_Q71DJ5	0.0	Normal: 382	475	252	296	300	1230	BlastAnno: 1458	UniRef50_Q71DJ5	393	63.14	388	134	2	129	1292	8	386	0.0	539
3.1.1.34(lipoprotein lipase;)																						
	No Correspond Unigene																					
3.1.3.21(glycerol-1-phosphatase;)																						
	No Correspond Unigene																					
3.1.3.4(phosphatidate phosphatase;)																						
	ABCDEF_3583	UniRef50_Q9FVL1	9e-133	Normal: 418	155	337	228	574	484	BlastAnno: 1631	UniRef50_Q9FVL1	322	66.36	324	105	3	1344	379	1	322	9e-133	398
3.13.1.1(UDP-sulfoquinovose synthase;)																						
	ABCDEF_35370	UniRef50_O48917	0.0	Normal: 1264	1108	1131	1710	2086	4845	BlastAnno: 2024	UniRef50_O48917	477	79.04	477	93	3	1732	317	1	475	0.0	760
3.2.1.22(alpha-galactosidase;)																						
	ABCDEF_31352	UniRef50_Q42656	0.0	Normal: 115	169	48	7	155	1183	BlastAnno: 1530	UniRef50_Q42656	378	76.76	370	85	1	242	1351	10	378	0.0	613
	ABCDEF_52628	UniRef50_Q42656	0.0	Normal: 1131	2713	1338	2471	4073	4919	BlastAnno: 1435	UniRef50_Q42656	378	65.22	368	127	1	1227	124	11	377	0.0	523
	ABCDEF_10850	UniRef50_Q42656	0.0	Normal: 170	155	153	167	153	166	BlastAnno: 1524	UniRef50_Q42656	378	74.66	371	94	0	389	1501	8	378	0.0	578
4.2.1.28(propanediol dehydratase;)																						
	No Correspond Unigene																					
4.2.1.30(glycerol dehydratase;)																						
	No Correspond Unigene																					
>00562 Inositol phosphate metabolism																						
1.1.1.18(inositol 2-dehydrogenase;)																						
	No Correspond Unigene																					
1.13.99.1(inositol oxygenase;)																						
	ABCDEF_32645	UniRef50_Q8H1S0	7e-162	Normal: 1	74	0	0	0	1292	BlastAnno: 1181	UniRef50_Q8H1S0	317	74.38	320	77	3	84	1037	1	317	7e-162	466
1.2.1.18(malonate-semialdehyde dehydrogenase (acetylating);)																						
	ABCDEF_18282	UniRef50_Q02252	0.0	Normal: 1599	1252	1838	3819	3718	2949	BlastAnno: 2054	UniRef50_Q02252	535	60.20	510	197	2	345	1856	23	532	0.0	653
1.2.1.27(methylmalonate-semialdehyde dehydrogenase (acylating);)																						
	ABCDEF_18282	UniRef50_B7GBU6	0.0	Normal: 1599	1252	1838	3819	3718	2949	BlastAnno: 2054	UniRef50_B7GBU6	537	59.35	492	191	2	402	1865	50	536	0.0	571
2.1.1.39(inositol 3-methyltransferase;)																						
	No Correspond Unigene																					
2.1.1.40(inositol 1-methyltransferase;)																						
	No Correspond Unigene																					
2.7.1.127(inositol-trisphosphate 3-kinase;)																						
	No Correspond Unigene																					
2.7.1.134(inositol-tetrakisphosphate 1-kinase;)																						
	ABCDEF_3567	UniRef50_O80568	4e-167	Normal: 264	153	279	381	535	280	BlastAnno: 1635	UniRef50_O80568	488	52.31	476	209	6	1615	191	23	481	4e-167	492
2.7.1.137(phosphatidylinositol 3-kinase;)																						
	ABCDEF_10418	UniRef50_P42339	0.0	Normal: 900	863	852	1241	985	1434	BlastAnno: 2833	UniRef50_P42339	814	83.27	819	131	4	2665	212	1	814	0.0	1293
2.7.1.140(inositol-tetrakisphosphate 5-kinase;)																						
	ABCDEF_27561	UniRef50_Q9FLT2	8e-123	Normal: 160	42	148	263	284	94	BlastAnno: 1032	UniRef50_Q9FLT2	300	58.33	288	115	1	101	949	1	288	8e-123	364
2.7.1.149(1-phosphatidylinositol-5-phosphate 4-kinase;)																						
	No Correspond Unigene																					
2.7.1.150(1-phosphatidylinositol-3-phosphate 5-kinase;)																						
	ABCDEF_33873	UniRef50_B9SJ39	0.0	Normal: 1074	595	980	1933	2451	1058	BlastAnno: 5376	UniRef50_B9SJ39	1651	59.20	1625	565	23	757	5376	5	1616	0.0	1626
	ABCDEF_16479	UniRef50_B9SJ39	0.0	Normal: 925	1132	929	1980	2071	1282	BlastAnno: 6887	UniRef50_B9SJ39	1651	46.63	1514	631	33	5421	1120	209	1625	0.0	1176
	ABCDEF_20698	UniRef50_B9SJ39	0.0	Normal: 303	536	335	1027	971	576	BlastAnno: 2864	UniRef50_B9SJ39	1651	49.32	884	371	15	2696	114	211	1040	0.0	776
	ABCDEF_24023	UniRef50_B9SJ39	0.0	Normal: 828	417	778	931	1493	523	BlastAnno: 3545	UniRef50_B9SJ39	1651	58.88	1126	417	12	17	3295	516	1628	0.0	1243
	ABCDEF_1005	UniRef50_B7P3Z6	0.0	Normal: 4748	1107	3611	3362	5199	2367	BlastAnno: 2170	UniRef50_B7P3Z6	510	55.86	546	195	5	182	1816	6	506	0.0	603
2.7.1.151(inositol-polyphosphate multikinase;)																						
	ABCDEF_27561	UniRef50_B7P880	4e-23	Normal: 160	42	148	263	284	94	BlastAnno: 1032	UniRef50_B7P880	278	31.14	289	153	12	116	928	15	275	4e-23	102
2.7.1.153(phosphatidylinositol-4,5-bisphosphate 3-kinase;)																						
	No Correspond Unigene																					
2.7.1.154(phosphatidylinositol-4-phosphate 3-kinase;)																						
	No Correspond Unigene																					
2.7.1.158(inositol-pentakisphosphate 2-kinase;)																						
	ABCDEF_40704	UniRef50_Q93YN9	7e-160	Normal: 218	146	292	431	449	258	BlastAnno: 1752	UniRef50_Q93YN9	451	55.83	446	189	4	2	1321	3	446	7e-160	473
2.7.1.159(inositol-1,3,4-trisphosphate 5/6-kinase;)																						
	No Correspond Unigene																					
2.7.1.64(inositol 3-kinase;)																						
	No Correspond Unigene																					
2.7.1.67(1-phosphatidylinositol 4-kinase;)																						
	ABCDEF_3899	UniRef50_P42356	1e-129	Normal: 1306	717	1369	1836	1717	1083	BlastAnno: 2202	UniRef50_P42356	2044	38.59	622	336	11	332	2083	1429	2042	1e-129	431
2.7.1.68(1-phosphatidylinositol-4-phosphate 5-kinase;)																						
	ABCDEF_74785	UniRef50_Q9SUI2	0.0	Normal: 220	133	179	308	487	141	BlastAnno: 1559	UniRef50_Q9SUI2	754	74.10	417	105	2	1423	173	338	751	0.0	647
	ABCDEF_27364	UniRef50_Q9SUI2	0.0	Normal: 382	275	287	577	867	229	BlastAnno: 2925	UniRef50_Q9SUI2	754	60.84	641	226	5	2663	753	4	623	0.0	788

ABCDEF_24461	UniRef50_048709	0.0	Normal: 419	166	404	583	349	340	BlastAnno: 2120	UniRef50_048709	705	57.02	598	167	9	405	2120	21	554	0.0	665
ABCDEF_3001	UniRef50_048709	0.0	Normal: 520	191	333	217	481	297	BlastAnno: 2996	UniRef50_048709	705	55.38	715	269	11	568	2673	28	705	0.0	746
ABCDEF_10457	UniRef50_048709	0.0	Normal: 678	432	865	1918	1388	448	BlastAnno: 2873	UniRef50_048709	705	54.83	755	239	10	452	2668	22	690	0.0	796
ABCDEF_21602	UniRef50_Q9M1K2	0.0	Normal: 1320	1224	1198	2705	2394	2007	BlastAnno: 2967	UniRef50_Q9M1K2	779	43.09	782	354	14	374	2665	67	775	0.0	583
ABCDEF_27366	UniRef50_Q9M1K2	0.0	Normal: 418	282	285	617	923	235	BlastAnno: 2807	UniRef50_Q9M1K2	779	40.84	786	367	18	272	2569	68	775	0.0	566

2.7.1.92(5-dehydro-2-deoxygluconokinase;)

No Correspond Unigene

2.7.8.11(CDP-diacylglycerol---inositol 3-phosphatidyltransferase;)

ABCDEF_13953	UniRef50_Q8LBA6	1e-110	Normal: 195	122	191	243	296	189	BlastAnno: 1145	UniRef50_Q8LBA6	227	71.11	225	63	1	108	776	1	225	1e-110	332

3.1.3.25(inositol-phosphate phosphatase;)

ABCDEF_24298	UniRef50_Q94F00	4e-170	Normal: 647	536	647	1368	1728	818	BlastAnno: 1549	UniRef50_Q94F00	371	64.61	373	126	2	41	1147	1	371	4e-170	494

3.1.3.26(4-phytase;)

No Correspond Unigene

3.1.3.36(phosphoinositide 5-phosphatase;)

ABCDEF_20859	UniRef50_Q9SKB7	0.0	Normal: 213	159	226	686	559	115	BlastAnno: 2293	UniRef50_Q9SKB7	1144	55.67	591	234	11	1860	145	96	677	0.0	628
ABCDEF_60625	UniRef50_Q9SKB7	0.0	Normal: 178	137	203	647	518	105	BlastAnno: 2355	UniRef50_Q9SKB7	1144	55.45	642	256	12	451	2319	96	726	0.0	676
ABCDEF_20858	UniRef50_Q9SKB7	0.0	Normal: 168	109	183	633	531	80	BlastAnno: 2229	UniRef50_Q9SKB7	1144	55.11	548	220	10	1796	210	96	636	0.0	577
ABCDEF_20857	UniRef50_Q9SKB7	0.0	Normal: 541	328	562	1285	1119	451	BlastAnno: 3848	UniRef50_Q9SKB7	1144	57.94	1008	399	11	452	3421	96	1096	0.0	1169

3.1.3.56(inositol-polyphosphate 5-phosphatase;)

ABCDEF_7365	UniRef50_Q9FUR2	0.0	Normal: 3	195	25	114	258	378	BlastAnno: 2077	UniRef50_Q9FUR2	646	53.94	571	211	10	4	1617	92	643	0.0	560
ABCDEF_20858	UniRef50_080560	0.0	Normal: 168	109	183	633	531	80	BlastAnno: 2229	UniRef50_080560	1305	58.48	525	194	10	1733	210	115	632	0.0	628
ABCDEF_20857	UniRef50_080560	0.0	Normal: 541	328	562	1285	1119	451	BlastAnno: 3848	UniRef50_080560	1305	62.03	993	351	12	515	3439	115	1099	0.0	1290
ABCDEF_60625	UniRef50_080560	0.0	Normal: 178	137	203	647	518	105	BlastAnno: 2355	UniRef50_080560	1305	59.56	596	216	11	514	2250	115	702	0.0	723
ABCDEF_7366	UniRef50_Q9FUR2	0.0	Normal: 6	238	22	118	222	314	BlastAnno: 2000	UniRef50_Q9FUR2	646	52.19	661	258	13	90	1955	2	643	0.0	593
ABCDEF_10381	UniRef50_B9RUI1	0.0	Normal: 0	171	0	0	0	377	BlastAnno: 2033	UniRef50_B9RUI1	437	75.56	356	70	5	171	1223	64	407	0.0	544
ABCDEF_20859	UniRef50_080560	0.0	Normal: 213	159	226	686	559	115	BlastAnno: 2293	UniRef50_080560	1305	58.45	568	211	11	1797	145	115	674	0.0	674
ABCDEF_34645	UniRef50_Q9LR47	0.0	Normal: 424	352	475	164	21	569	BlastAnno: 1926	UniRef50_Q9LR47	617	70.29	478	95	11	1926	541	171	617	0.0	598

3.1.3.57(inositol-1,4-bisphosphate 1-phosphatase;)

No Correspond Unigene

3.1.3.62(multiple inositol-polyphosphate phosphatase;)

No Correspond Unigene

3.1.3.64(phosphatidylinositol-3-phosphatase;)

No Correspond Unigene

3.1.3.66(phosphatidylinositol-3,4-bisphosphate 4-phosphatase;)

No Correspond Unigene

3.1.3.67(phosphatidylinositol-3,4,5-trisphosphate 3-phosphatase;)

ABCDEF_31277	UniRef50_B7Q941	5e-60	Normal: 57	15	120	203	574	22	BlastAnno: 2129	UniRef50_B7Q941	363	50.79	191	86	1	1486	914	2	184	5e-60	213

3.1.3.8(3-phytase;)

No Correspond Unigene

3.1.4.11(phosphoinositide phospholipase C;)

ABCDEF_12974	UniRef50_Q8GV43	0.0	Normal: 26	173	22	28	59	198	BlastAnno: 2360	UniRef50_Q8GV43	613	58.02	536	172	6	1792	206	123	612	0.0	598
ABCDEF_12970	UniRef50_Q8GV43	0.0	Normal: 27	158	24	26	67	201	BlastAnno: 2327	UniRef50_Q8GV43	613	58.14	528	176	6	1768	206	123	612	0.0	590
ABCDEF_7920	UniRef50_Q8GV43	0.0	Normal: 1605	1027	1541	3468	3403	3774	BlastAnno: 2223	UniRef50_Q8GV43	613	60.66	610	202	7	1983	232	15	612	0.0	736
ABCDEF_12965	UniRef50_Q8GV43	0.0	Normal: 23	172	24	31	75	233	BlastAnno: 2289	UniRef50_Q8GV43	613	53.17	647	228	11	2077	206	18	612	0.0	631
ABCDEF_40188	UniRef50_Q8GV43	0.0	Normal: 243	250	287	222	309	299	BlastAnno: 1780	UniRef50_Q8GV43	613	64.41	531	172	4	125	1711	97	612	0.0	727
ABCDEF_12975	UniRef50_Q8GV43	0.0	Normal: 41	317	40	44	113	364	BlastAnno: 2189	UniRef50_Q8GV43	613	56.12	613	227	10	1975	206	19	612	0.0	655
ABCDEF_12967	UniRef50_Q8GV43	0.0	Normal: 25	178	22	28	64	203	BlastAnno: 2286	UniRef50_Q8GV43	613	53.49	645	226	11	2071	206	19	612	0.0	640
ABCDEF_12976	UniRef50_Q8GV43	0.0	Normal: 25	157	21	23	60	175	BlastAnno: 2349	UniRef50_Q8GV43	613	51.12	669	229	12	2143	206	19	612	0.0	619
ABCDEF_83256	UniRef50_Q8GV43	0.0	Normal: 27	166	22	26	59	191	BlastAnno: 2284	UniRef50_Q8GV43	613	55.36	560	173	7	1864	206	123	612	0.0	582
ABCDEF_40192	UniRef50_Q8GV43	0.0	Normal: 342	345	344	306	420	450	BlastAnno: 2173	UniRef50_Q8GV43	613	58.64	631	222	8	1948	116	1	612	0.0	742
ABCDEF_12971	UniRef50_Q8GV43	0.0	Normal: 26	179	26	31	71	229	BlastAnno: 2264	UniRef50_Q8GV43	613	61.51	504	173	5	1696	206	123	612	0.0	613
ABCDEF_12964	UniRef50_Q8GV43	0.0	Normal: 24	167	24	31	70	227	BlastAnno: 2329	UniRef50_Q8GV43	613	57.73	537	173	6	1761	172	123	612	0.0	591
ABCDEF_89844	UniRef50_Q8GV43	0.0	Normal: 27	180	26	30	77	231	BlastAnno: 2253	UniRef50_Q8GV43	613	53.92	638	228	11	2050	206	18	612	0.0	639

3.1.4.3(phospholipase C;)

ABCDEF_24368	UniRef50_Q22P01	1e-93	Normal: 158	107	203	472	477	108	BlastAnno: 1430	UniRef50_Q22P01	500	40.35	456	211	15	1308	61	4	438	1e-93	301

3.7.1.-(Hydrolases;)

No Correspond Unigene

4.1.2.29(5-dehydro-2-deoxyphosphogluconate aldolase;)

No Correspond Unigene

4.2.1.44(myo-inosose-2 dehydratase;)

No Correspond Unigene

4.6.1.13(phosphatidylinositol diacylglycerol-lyase;)

ABCDEF_18469	UniRef50_P34024	4e-11	Normal: 284	1904	446	3367	2114	6085	BlastAnno: 1745	UniRef50_P34024	317	32.17	115	75	3	608	946	46	159	4e-11	70.1

5.3.1.-(Isomerases;)

No Correspond Unigene

5.3.1.1(triose-phosphate isomerase;)

ABCDEF_25548	UniRef50_Q9SKP6	8e-167	Normal: 879	1226	1007	1772	2081	5167	BlastAnno: 1419	UniRef50_Q9SKP6	315	75.95	316	70	3	1221	289	1	315	8e-167	482

5.5.1.4(inositol-3-phosphate synthase;)

ABCDEF_35470	UniRef50_Q9NPH2	0.0	Normal: 4323	922	2996	984	2481	19110	BlastAnno: 2022	UniRef50_Q9NPH2	558	59.88	506	197	4	202	1716	7	507	0.0	649

>00563 Glycosylphosphatidylinositol(GPI)-anchor biosynthesis

2.3.-.-(Transferases;)

ABCDEF_40719	UniRef50_Q54MC0	1e-47	Normal: 79	101	105	229	268	159	BlastAnno: 1918	UniRef50_Q54MC0	492	32.48	508	267	10	1439	81	6	492	1e-47	181

2.4.1.-(Transferases;)

ABCDEF_18004	UniRef50_Q6YWK8	0.0	Normal: 1562	1736	2009	3452	4438	2400	BlastAnno: 2298	UniRef50_Q6YWK8	570	65.27	429	136	1	1941	694	57	485	0.0	608
ABCDEF_52379	UniRef50_Q9LHZ7	0.0	Normal: 4015	4921	4546	10520	13430	4731	BlastAnno: 3479	UniRef50_Q9LHZ7	1170	48.04	893	342	16	825	3242	299	1156	0.0	787
ABCDEF_48593	UniRef50_Q9SK82	0.0	Normal: 12	420	19	343	763	253	BlastAnno: 1834	UniRef50_Q9SK82	489	60.08	481	186	4	1714	278	10	486	0.0	633
ABCDEF_13375	UniRef50_Q8VZK9	0.0	Normal: 108	123	208	526	34	294	BlastAnno: 1738	UniRef50_Q8VZK9	729	53.62	552	234	8	94	1737	18	551	0.0	582
ABCDEF_40982	UniRef50_Q96301	0.0	Normal: 1037	706	819	1249	1084	920	BlastAnno: 3418	UniRef50_Q96301	914	79.60	902	173	3	243	2948	1	891	0.0	1506
ABCDEF_47674	UniRef50_Q9LHZ7	0.0	Normal: 274	92	202	599	505	280	BlastAnno: 2526	UniRef50_Q9LHZ7	1170	75.93	777	151	8	2260	2	15	779	0.0	1177
ABCDEF_34097	UniRef50_Q9LE59	0.0	Normal: 626	613	923	1797	1829	692	BlastAnno: 2521	UniRef50_Q9LE59	673	60.92	458	178	1	1687	317	215	672	0.0	630
ABCDEF_34098	UniRef50_Q9LE59	0.0	Normal: 457	464	697	1283	1312	539	BlastAnno: 2199	UniRef50_Q9LE59	673	60.92	458	178	1	1687	317	215	672	0.0	628
ABCDEF_10848	UniRef50_Q9M156	0.0	Normal: 157	2988	163	408	772	1566	BlastAnno: 1810	UniRef50_Q9M156	480	60.09	456	176	4	1700	336	4	454	0.0	553
ABCDEF_41182	UniRef50_Q9LHZ7	0.0	Normal: 150	2169	221	814	1407	495	BlastAnno: 3604	UniRef50_Q9LHZ7	1170	51.03	870	333	15	2822	456	299	1156	0.0	826
ABCDEF_40981	UniRef50_Q96301	0.0	Normal: 156	97	110	160	179	173	BlastAnno: 1225	UniRef50_Q96301	914	86.92	344	45	0	1112	81	394	737	0.0	652
ABCDEF_9506	UniRef50_Q9LHZ7	0.0	Normal: 1302	2128	1494	3356	4644	2206	BlastAnno: 3751	UniRef50_Q9LHZ7	1170	49.38	891	343	17	927	3359	299	1161	0.0	812
ABCDEF_9500	UniRef50_Q9LHZ7	0.0	Normal: 183	188	186	576	498	260	BlastAnno: 1717	UniRef50_Q9LHZ7	1170	47.98	644	234	14	3	1715	418	1033	0.0	571
ABCDEF_100	UniRef50_Q9M8Y0	0.0	Normal: 228	309	224	416	461	141	BlastAnno: 2109	UniRef50_Q9M8Y0	977	77.49	533	120	0	1610	12	50	582	0.0	884
ABCDEF_33659	UniRef50_Q9LHZ7	0.0	Normal: 1282	1948	2248	5826	10642	2083	BlastAnno: 4289	UniRef50_Q9LHZ7	1170	48.70	887	345	15	2955	538	299	1156	0.0	788
ABCDEF_37667	UniRef50_Q9LHZ7	0.0	Normal: 248	3617	251	1237	2078	970	BlastAnno: 3161	UniRef50_Q9LHZ7	1170	49.72	885	335	14	2671	260	301	1156	0.0	810
ABCDEF_47673	UniRef50_Q9LHZ7	0.0	Normal: 876	296	828	1628	1721	1172	BlastAnno: 3878	UniRef50_Q9LHZ7	1170	80.88	1161	186	8	3642	232	15	1163	0.0	1865
ABCDEF_99	UniRef50_Q9M8Y0	0.0	Normal: 272	330	222	398	423	145	BlastAnno: 2094	UniRef50_Q9M8Y0	977	77.36	561	127	0	1683	1	50	610	0.0	929
ABCDEF_20658	UniRef50_Q8VZK9	0.0	Normal: 373	33	193	64	503	141	BlastAnno: 2316	UniRef50_Q8VZK9	729	53.35	508	221	7	47	1564	11	504	0.0	559
ABCDEF_10183	UniRef50_A7XDQ9	0.0	Normal: 422	455	346	577	378	610	BlastAnno: 2362	UniRef50_A7XDQ9	684	50.94	693	307	12	281	2287	1	684	0.0	670
ABCDEF_30745	UniRef50_A7XDQ9	0.0	Normal: 1209	351	1031	1645	1226	635	BlastAnno: 2646	UniRef50_A7XDQ9	684	57.58	554	227	6	546	2189	133	684	0.0	664
ABCDEF_13336	UniRef50_Q7PC69	0.0	Normal: 148	563	194	957	1575	816	BlastAnno: 2584	UniRef50_Q7PC69	745	71.56	559	136	6	744	2351	187	745	0.0	730
ABCDEF_21426	UniRef50_Q8RU27	0.0	Normal: 2658	1979	3417	6157	9464	2599	BlastAnno: 1530	UniRef50_Q8RU27	366	89.44	360	34	2	1358	282	1	357	0.0	667
ABCDEF_71242	UniRef50_Q9ZV71	0.0	Normal: 134	122	179	480	754	119	BlastAnno: 1835	UniRef50_Q9ZV71	409	73.35	409	103	4	231	1439	1	409	0.0	587
ABCDEF_52020	UniRef50_Q6YWK8	0.0	Normal: 1635	1534	1975	4284	10353	2289	BlastAnno: 2394	UniRef50_Q6YWK8	570	58.37	514	185	2	403	1857	57	570	0.0	607
ABCDEF_51269	UniRef50_Q6YWK8	0.0	Normal: 147	85	103	21	33	438	BlastAnno: 1864	UniRef50_Q6YWK8	570	54.28	514	204	2	1646	198	57	570	0.0	586
ABCDEF_30753	UniRef50_Q9ZV71	0.0	Normal: 290	497	417	629	1013	634	BlastAnno: 1502	UniRef50_Q9ZV71	409	72.32	401	107	2	311	1501	1	401	0.0	590
ABCDEF_4233	UniRef50_Q9ZV71	0.0	Normal: 369	412	550	936	860	750	BlastAnno: 2004	UniRef50_Q9ZV71	409	72.13	409	110	2	370	1584	1	409	0.0	613
ABCDEF_17525	UniRef50_Q8RU27	0.0	Normal: 1500	1289	2149	3711	4446	1069	BlastAnno: 1507	UniRef50_Q8RU27	366	91.35	347	30	0	1371	331	3	349	0.0	671
ABCDEF_18005	UniRef50_Q6YWK8	0.0	Normal: 1842	2042	2515	4214	5208	2842	BlastAnno: 2305	UniRef50_Q6YWK8	570	59.14	514	180	2	1948	497	57	570	0.0	642
ABCDEF_41140	UniRef50_Q6YWK8	0.0	Normal: 86	1314	93	753	1787	2076	BlastAnno: 2346	UniRef50_Q6YWK8	570	60.00	510	174	2	431	1870	57	566	0.0	669
ABCDEF_18002	UniRef50_Q9LHZ7	0.0	Normal: 376	127	367	601	1190	520	BlastAnno: 1605	UniRef50_Q9LHZ7	1170	89.75	400	41	0	1603	404	771	1170	0.0	732
ABCDEF_13339	UniRef50_Q7PC69	0.0	Normal: 127	814	183	541	1135	1144	BlastAnno: 2610	UniRef50_Q7PC69	745	70.48	559	141	6	725	2329	187	745	0.0	729
ABCDEF_41141	UniRef50_Q6YWK8	0.0	Normal: 67	1107	84	708	1690	1854	BlastAnno: 2461	UniRef50_Q6YWK8	570	60.00	510	174	2	1916	477	57	566	0.0	668
ABCDEF_44583	UniRef50_Q9LE59	0.0	Normal: 681	561	559	947	1745	789	BlastAnno: 2470	UniRef50_Q9LE59	673	59.21	456	185	1	1749	385	214	669	0.0	621
ABCDEF_3869	UniRef50_Q9LE59	0.0	Normal: 333	393	415	809	907	754	BlastAnno: 2136	UniRef50_Q9LE59	673	59.05	613	227	4	1827	1	81	673	0.0	699
ABCDEF_28947	UniRef50_Q9SK82	0.0	Normal: 0	851	3	0	0	116	BlastAnno: 1567	UniRef50_Q9SK82	489	62.40	484	176	4	1524	85	1	482	0.0	652
ABCDEF_31119	UniRef50_Q9M8Y0	0.0	Normal: 2212	1279	1917	2933	3341	1803	BlastAnno: 3695	UniRef50_Q9M8Y0	977	81.08	925	174	1	392	3163	50	974	0.0	1583
ABCDEF_37311	UniRef50_Q9LE59	0.0	Normal: 175	133	191	549	421	366	BlastAnno: 1612	UniRef50_Q9LE59	673	62.96	486	153	4	1406	3	4	480	0.0	542
ABCDEF_24190	UniRef50_Q8L7F9	0.0	Normal: 397	265	492	583	553	371	BlastAnno: 2564	UniRef50_Q8L7F9	643	63.91	640	218	4	2353	473	1	640	0.0	850
ABCDEF_30548	UniRef50_Q9FH36	0.0	Normal: 17	916	27	151	420	126	BlastAnno: 1963	UniRef50_Q9FH36	535	72.47	534	138	3	102	1676	1	534	0.0	767
ABCDEF_27416	UniRef50_Q7PC69	0.0	Normal: 575	837	875	1344	2015	936	BlastAnno: 2454	UniRef50_Q7PC69	745	66.11	416	115	4	2337	1168	126	541	0.0	518
ABCDEF_48373	UniRef50_A7XDQ9	0.0	Normal: 1364	773	1336	1451	1791	1202	BlastAnno: 3042	UniRef50_A7XDQ9	684	63.68	691	234	6	2405	360	1	683	0.0	915
ABCDEF_3868	UniRef50_Q9LE59	0.0	Normal: 224	274	301	555	686	524	BlastAnno: 1670	UniRef50_Q9LE59	673	65.31	516	176	1	1556	9	161	673	0.0	696
ABCDEF_31288	UniRef50_Q940V3	0.0	Normal: 247	309	183	101	124	655	BlastAnno: 1658	UniRef50_Q940V3	470	56.01	466	197	7	1614	232	8	470	0.0	537
ABCDEF_52574	UniRef50_Q09332	0.0	Normal: 885	474	1068	1835	1853	816	BlastAnno: 3720	UniRef50_Q09332	1548	47.89	687	310	14	2551	533	859	1511	0.0	598
ABCDEF_79752	UniRef50_Q9ZV71	0.0	Normal: 158	183	245	400	379	352	BlastAnno: 1266	UniRef50_Q9ZV71	409	71.39	360	99	2	31	1098	1	360	0.0	526
ABCDEF_9509	UniRef50_Q9LHZ7	0.0	Normal: 1296	2171	1498	3410	4683	2232	BlastAnno: 3897	UniRef50_Q9LHZ7	1170	49.38	891	343	17	1073	3505	299	1161	0.0	812
ABCDEF_48200	UniRef50_Q6YWK8	0.0	Normal: 1768	576	3156	4431	6634	491	BlastAnno: 2274	UniRef50_Q6YWK8	570	57.98	514	185	4	291	1739	57	570	0.0	642
ABCDEF_24155	UniRef50_Q9ZVI7	0.0	Normal: 503	247	607	1676	1833	578	BlastAnno: 2708	UniRef50_Q9ZVI7	619	52.33	621	264	11	306	2123	15	618	0.0	601
ABCDEF_21246	UniRef50_Q9SKT6	0.0	Normal: 92	107	188	380	834	84	BlastAnno: 1791	UniRef50_Q9SKT6	536	56.61	484	204	5	123	1562	52	533	0.0	568
ABCDEF_32317	UniRef50_Q8RU27	0.0	Normal: 12	9	3	118	800	62	BlastAnno: 1076	UniRef50_Q8RU27	366	87.02	285	37	0	1075	221	64	348	0.0	535
ABCDEF_9502	UniRef50_Q9LHZ7	0.0	Normal: 610	486	568	1331	1403	591	BlastAnno: 1961	UniRef50_Q9LHZ7	1170	48.64	625	249	13	3	1742	564	1161	0.0	570
ABCDEF_23298	UniRef50_Q9FH36	0.0	Normal: 128	527	130	223	314	864	BlastAnno: 2832	UniRef50_Q9FH36	535	83.16	196	33	0	696	1283	340	535	0.0	357
ABCDEF_40595	UniRef50_O80898	0.0	Normal: 229	135	103	25	307	325	BlastAnno: 2413	UniRef50_O80898	757	44.68	761	378	13	95	2299	10	753	0.0	587
ABCDEF_63947	UniRef50_Q9SK82	0.0	Normal: 0	267	0	0	71	0	BlastAnno: 1446	UniRef50_Q9SK82	489	58.33	468	189	4	60	1445	1	468	0.0	592
ABCDEF_67847	UniRef50_Q9M8Y0	0.0	Normal: 794	476	871	1081	1401	868	BlastAnno: 1591	UniRef50_Q9M8Y0	977	84.86	370	56	0	1589	480	605	974	0.0	685
ABCDEF_65666	UniRef50_Q09332	0.0	Normal: 800	441	946	1606	1637	735	BlastAnno: 3518	UniRef50_Q09332	1548	47.54	671	305	13	1162	3087	859	1511	0.0	586
ABCDEF_38641	UniRef50_Q9FWA4	0.0	Normal: 674	967	1162	2603	4404	2475	BlastAnno: 1599	UniRef50_Q9FWA4	561	81.35	386	72	0	1	1158	176	561	0.0	641
ABCDEF_17914	UniRef50_Q9SKT6	0.0	Normal: 784	567	886	1447	1439	1424	BlastAnno: 2049	UniRef50_Q9SKT6	536	72.95	536	140	2	204	1796	1	536	0.0	785
ABCDEF_18286	UniRef50_Q6NQB7	0.0	Normal: 1131	1108	1225	1872	1722	3329	BlastAnno: 1752	UniRef50_Q6NQB7	393	73.80	397	81	6	1527	367	10	393	0.0	607
ABCDEF_119	UniRef50_Q8RU27	0.0	Normal: 1613	1155	2249	3558	7663	1775	BlastAnno: 1532	UniRef50_Q8RU27	366	88.33	360	38	2	1306	230	1	357	0.0	663
ABCDEF_47603	UniRef50_Q9M9Y5	0.0	Normal: 2609	8779	1664	1103	2085	22991	BlastAnno: 2830	UniRef50_Q9M9Y5	589	54.04	594	267	3	2475	694	1	588	0.0	654
ABCDEF_24192	UniRef50_Q8L7F9	0.0	Normal: 391	244	465	553	497	345	BlastAnno: 2477	UniRef50_Q8L7F9	643	58.99	434	165	4	212	1474	1	434	0.0	513
ABCDEF_17482	UniRef50_Q9SKT6	0.0	Normal: 615	379	816	1068	1470	852	BlastAnno: 2083	UniRef50_Q9SKT6	536	71.11	540	147	4	1915	311	1	536	0.0	786
ABCDEF_85909	UniRef50_Q8VZK9	0.0	Normal: 28	45	9	10	44	127	BlastAnno: 1473	UniRef50_Q8VZK9	729	55.37	475	198	6	1419	1	11	473	0.0	536
ABCDEF_9503	UniRef50_Q9LHZ7	0.0	Normal: 487	544	524	1242	1320	833	BlastAnno: 2087	UniRef50_Q9LHZ7	1170	50.35	574	236	8	57	1712	615	1161	0.0	567
ABCDEF_40594	UniRef50_O80898	0.0	Normal: 256	127	102	41	331	326	BlastAnno: 2417	UniRef50_O80898	757	45.76	719	348	12	213	2291	51	753	0.0	585
ABCDEF_17141	UniRef50_Q9LHZ7	0.0	Normal: 74	1734	69	380	701	333	BlastAnno: 3337	UniRef50_Q9LHZ7	1170	50.92	868	335	14	2539	179	299	1156	0.0	816
ABCDEF_33658	UniRef50_Q9LHZ7	0.0	Normal: 384	308	395	805	1003	602	BlastAnno: 2961	UniRef50_Q9LHZ7	1170	48.76	888	343	14	204	2621	299	1156	0.0	792
ABCDEF_20579	UniRef50_Q7PC69	0.0	Normal: 1007	795	1238	2311	2309	1345	BlastAnno: 3113	UniRef50_Q7PC69	745	59.97	767	229	14	2667	535	1	745	0.0	768

ABCDEF_31118	UniRef50_Q9M8Y0	0.0	Normal: 2135	1267	1900	3004	3329	1757	BlastAnno: 3787	UniRef50_Q9M8Y0	977	81.10	709	133	1	1132	3255	266	974	0.0	1217
ABCDEF_9499	UniRef50_Q9LHZ7	0.0	Normal: 205	207	215	632	538	275	BlastAnno: 1794	UniRef50_Q9LHZ7	1170	48.88	667	240	14	11	1792	395	1033	0.0	605
ABCDEF_48199	UniRef50_Q6YWK8	0.0	Normal: 1506	467	2429	3094	4233	439	BlastAnno: 1997	UniRef50_Q6YWK8	570	63.10	439	146	2	291	1559	57	495	0.0	610
ABCDEF_40983	UniRef50_Q96301	0.0	Normal: 406	261	343	392	415	433	BlastAnno: 2265	UniRef50_Q96301	914	79.17	504	99	1	114	1625	394	891	0.0	854
ABCDEF_17140	UniRef50_Q9LHZ7	0.0	Normal: 45	1049	49	216	386	208	BlastAnno: 2293	UniRef50_Q9LHZ7	1170	52.34	770	281	13	2138	57	397	1156	0.0	739
ABCDEF_18352	UniRef50_Q9FWA4	0.0	Normal: 2427	2688	2969	5519	6547	4855	BlastAnno: 2224	UniRef50_Q9FWA4	561	69.31	505	148	3	1887	382	61	561	0.0	683
ABCDEF_27767	UniRef50_Q9LHZ7	0.0	Normal: 2576	5141	2841	10249	14583	5031	BlastAnno: 3981	UniRef50_Q9LHZ7	1170	49.66	888	337	16	2927	504	299	1156	0.0	818
ABCDEF_33661	UniRef50_Q9LHZ7	0.0	Normal: 414	340	507	879	790	783	BlastAnno: 2185	UniRef50_Q9LHZ7	1170	48.66	748	277	14	2184	169	440	1156	0.0	662
ABCDEF_14685	UniRef50_A7XDQ9	0.0	Normal: 233	308	134	573	292	334	BlastAnno: 1947	UniRef50_A7XDQ9	684	56.84	468	186	6	569	1945	1	461	0.0	545
ABCDEF_20660	UniRef50_Q8VZK9	0.0	Normal: 22	48	13	15	52	144	BlastAnno: 1473	UniRef50_Q8VZK9	729	55.16	475	199	6	55	1473	11	473	0.0	537
ABCDEF_21315	UniRef50_Q9LHZ7	0.0	Normal: 3412	252	1450	0	79	1228	BlastAnno: 3765	UniRef50_Q9LHZ7	1170	63.78	1096	340	15	308	3508	70	1137	0.0	1333
ABCDEF_533	UniRef50_Q9C8W3	0.0	Normal: 230	204	269	505	411	301	BlastAnno: 1688	UniRef50_Q9C8W3	401	70.33	364	94	2	393	1475	47	399	0.0	555
ABCDEF_34099	UniRef50_Q9LE59	0.0	Normal: 478	491	735	1356	1350	548	BlastAnno: 2158	UniRef50_Q9LE59	673	60.92	458	178	1	1610	240	215	672	0.0	630
ABCDEF_47984	UniRef50_Q7PC69	0.0	Normal: 663	457	734	1362	2147	696	BlastAnno: 2838	UniRef50_Q7PC69	745	59.27	712	226	9	373	2316	34	745	0.0	702
ABCDEF_51451	UniRef50_Q9LE59	0.0	Normal: 1883	1782	2202	4337	4350	2667	BlastAnno: 2874	UniRef50_Q9LE59	673	74.16	685	165	6	2559	505	1	673	0.0	982
ABCDEF_13370	UniRef50_Q8VZK9	0.0	Normal: 290	87	317	377	105	258	BlastAnno: 2033	UniRef50_Q8VZK9	729	51.65	635	278	11	2031	154	112	726	0.0	612
ABCDEF_48239	UniRef50_Q9LHZ7	0.0	Normal: 511	163	422	1154	1316	510	BlastAnno: 2714	UniRef50_Q9LHZ7	1170	76.20	769	156	9	478	2712	41	806	0.0	1201
ABCDEF_13338	UniRef50_Q7PC69	0.0	Normal: 335	468	417	2639	4443	616	BlastAnno: 2525	UniRef50_Q7PC69	745	62.45	727	209	12	298	2292	21	745	0.0	781
ABCDEF_24193	UniRef50_Q8L7F9	0.0	Normal: 113	72	132	203	180	119	BlastAnno: 1679	UniRef50_Q8L7F9	643	60.54	479	176	4	212	1609	1	479	0.0	592
ABCDEF_48514	UniRef50_Q9ZV71	0.0	Normal: 540	580	601	771	1033	926	BlastAnno: 1920	UniRef50_Q9ZV71	409	78.14	398	85	2	1618	431	12	409	0.0	637
2.4.1.198(phosphatidylinositol N-acetylglucosaminyltransferase;)																					
ABCDEF_56960	UniRef50_P37287	6e-164	Normal: 66	39	56	71	72	90	BlastAnno: 1530	UniRef50_P37287	484	54.12	425	190	3	1418	156	33	456	6e-164	482
2.6.-.-(Transferases;)																					
ABCDEF_3637	UniRef50_Q8LAD0	1e-121	Normal: 102	196	78	84	162	412	BlastAnno: 922	UniRef50_Q8LAD0	255	78.44	218	47	0	870	217	1	218	1e-121	358
2.7.-.-(Transferases;)																					
ABCDEF_23367	UniRef50_Q9SYX2	0.0	Normal: 506	422	484	686	760	545	BlastAnno: 3695	UniRef50_Q9SYX2	1029	51.41	850	355	12	791	3214	194	1027	0.0	828
ABCDEF_45142	UniRef50_Q9MA15	0.0	Normal: 347	633	345	439	495	800	BlastAnno: 2401	UniRef50_Q9MA15	711	82.82	390	67	0	955	2124	298	687	0.0	655
ABCDEF_27575	UniRef50_Q8RWG1	0.0	Normal: 452	1600	437	812	911	2795	BlastAnno: 2280	UniRef50_Q8RWG1	682	86.14	599	74	1	1947	151	93	682	0.0	1073
ABCDEF_40580	UniRef50_Q9SYX2	0.0	Normal: 259	404	197	295	247	615	BlastAnno: 3187	UniRef50_Q9SYX2	1029	43.09	970	444	23	2788	44	51	967	0.0	620
ABCDEF_41280	UniRef50_Q9LRB0	0.0	Normal: 846	755	903	1378	1441	1073	BlastAnno: 2965	UniRef50_Q9LRB0	763	66.62	770	228	15	364	2622	1	758	0.0	939
ABCDEF_41441	UniRef50_Q9LRB0	0.0	Normal: 1112	790	991	1469	1539	1330	BlastAnno: 3089	UniRef50_Q9LRB0	763	64.25	772	241	15	2565	322	1	761	0.0	848
ABCDEF_65007	UniRef50_Q9MA15	0.0	Normal: 253	467	255	310	382	629	BlastAnno: 1826	UniRef50_Q9MA15	711	82.82	390	67	0	400	1569	298	687	0.0	655
ABCDEF_24582	UniRef50_Q9ASX5	0.0	Normal: 491	993	467	696	751	1720	BlastAnno: 1876	UniRef50_Q9ASX5	540	76.22	534	119	3	1813	221	2	530	0.0	834
3.-.-.-(No Name)																					
ABCDEF_48866	UniRef50_Q687E1	0.0	Normal: 345	607	237	367	189	838	BlastAnno: 1481	UniRef50_Q687E1	368	68.38	351	109	1	1199	147	20	368	0.0	521
ABCDEF_3986	UniRef50_Q9LYC1	0.0	Normal: 226	528	111	65	141	752	BlastAnno: 1793	UniRef50_Q9LYC1	358	74.64	347	84	3	1423	386	1	344	0.0	551
ABCDEF_34862	UniRef50_Q9MAA7	0.0	Normal: 341	401	253	317	200	932	BlastAnno: 1664	UniRef50_Q9MAA7	345	74.71	348	83	2	138	1178	1	344	0.0	553
ABCDEF_51645	UniRef50_Q687E1	0.0	Normal: 433	515	632	1120	1009	791	BlastAnno: 2234	UniRef50_Q687E1	368	74.71	348	88	0	987	2030	20	367	0.0	575
ABCDEF_24556	UniRef50_C8NK17	0.0	Normal: 7370	10762	6819	9293	10001	30701	BlastAnno: 3104	UniRef50_C8NK17	927	57.35	823	317	7	401	2791	1	815	0.0	839
3.1.4.50(glycosylphosphatidylinositol phospholipase D;)																					
No Correspond Unigene																					
3.5.1.89(N-acetylglucosaminylphosphatidylinositol deacetylase;)																					
ABCDEF_33446	UniRef50_Q54C64	2e-46	Normal: 24	27	35	59	58	45	BlastAnno: 1183	UniRef50_Q54C64	258	35.34	249	135	1	788	120	9	257	2e-46	167
ABCDEF_33444	UniRef50_Q54C64	2e-46	Normal: 31	28	37	70	62	47	BlastAnno: 1147	UniRef50_Q54C64	258	35.34	249	135	1	788	120	9	257	2e-46	167
ABCDEF_33445	UniRef50_Q54C64	2e-46	Normal: 26	24	34	64	59	48	BlastAnno: 1181	UniRef50_Q54C64	258	35.34	249	135	1	788	120	9	257	2e-46	167
>00564 Glycerophospholipid metabolism																					
1.1.1.101(acylglycerone-phosphate reductase;)																					
No Correspond Unigene																					
1.1.1.8(glycerol-3-phosphate dehydrogenase (NAD+);)																					
ABCDEF_10630	UniRef50_O22216	0.0	Normal: 138	52	180	85	289	175	BlastAnno: 1168	UniRef50_O22216	462	83.49	315	52	0	3	947	147	461	0.0	538
ABCDEF_6818	UniRef50_O22216	0.0	Normal: 366	332	744	2748	1900	741	BlastAnno: 1994	UniRef50_O22216	462	87.05	440	57	0	319	1638	23	462	0.0	750
ABCDEF_27221	UniRef50_O22216	0.0	Normal: 218	753	340	750	835	611	BlastAnno: 1610	UniRef50_O22216	462	86.77	446	59	0	26	1363	17	462	0.0	791
ABCDEF_27224	UniRef50_O22216	0.0	Normal: 274	1068	444	1091	1198	828	BlastAnno: 2387	UniRef50_O22216	462	84.98	466	64	3	1701	310	1	462	0.0	796
ABCDEF_44741	UniRef50_Q9SCX9	0.0	Normal: 165	151	171	275	160	408	BlastAnno: 1639	UniRef50_Q9SCX9	400	84.62	351	54	0	1357	305	48	398	0.0	621
ABCDEF_27223	UniRef50_O22216	0.0	Normal: 323	1222	500	1289	1413	1042	BlastAnno: 2585	UniRef50_O22216	462	85.19	466	63	3	1701	310	1	462	0.0	798
1.1.1.94(glycerol-3-phosphate dehydrogenase [NAD(P)+];)																					
ABCDEF_30205	UniRef50_C4XLT6	1e-65	Normal: 30	25	41	6	13	111	BlastAnno: 1097	UniRef50_C4XLT6	330	44.48	290	161	0	1093	224	35	324	1e-65	219
1.1.3.21(glycerol-3-phosphate oxidase;)																					
ABCDEF_17411	UniRef50_Q2RU86	9e-132	Normal: 231	285	295	464	913	496	BlastAnno: 1965	UniRef50_Q2RU86	548	44.23	520	270	5	3	1562	37	536	9e-132	407
1.1.5.3(glycerol-3-phosphate dehydrogenase;)																					
ABCDEF_17411	UniRef50_Q9SS48	0.0	Normal: 231	285	295	464	913	496	BlastAnno: 1965	UniRef50_Q9SS48	629	83.82	544	88	0	3	1634	85	628	0.0	941
2.1.1.103(phosphoethanolamine N-methyltransferase;)																					
ABCDEF_89311	UniRef50_Q9FR44	0.0	Normal: 852	595	1093	1834	3031	584	BlastAnno: 3718	UniRef50_Q9FR44	491	80.33	488	96	0	3366	1903	1	488	0.0	838
ABCDEF_84806	UniRef50_Q9FR44	0.0	Normal: 857	592	1093	1832	3031	589	BlastAnno: 3791	UniRef50_Q9FR44	491	80.33	488	96	0	3439	1976	1	488	0.0	838
ABCDEF_84635	UniRef50_Q9FR44	0.0	Normal: 850	566	1089	1829	3029	543	BlastAnno: 3785	UniRef50_Q9FR44	491	80.33	488	96	0	3433	1970	1	488	0.0	838
ABCDEF_87399	UniRef50_Q9FR44	0.0	Normal: 837	515	1082	1827	3016	357	BlastAnno: 2708	UniRef50_Q9FR44	491	80.33	488	96	0	2356	893	1	488	0.0	838
ABCDEF_80121	UniRef50_Q9FR44	0.0	Normal: 849	566	1088	1830	3029	564	BlastAnno: 3700	UniRef50_Q9FR44	491	80.33	488	96	0	3348	1885	1	488	0.0	838
ABCDEF_16951	UniRef50_Q9FR44	0.0	Normal: 864	571	1042	1833	3035	555	BlastAnno: 3858	UniRef50_Q9FR44	491	80.33	488	96	0	353	1816	1	488	0.0	838
ABCDEF_13728	UniRef50_Q9FR44	0.0	Normal: 847	1261	624	1048	1206	988	BlastAnno: 2101	UniRef50_Q9FR44	491	79.59	490	100	0	1699	230	1	490	0.0	842
ABCDEF_16952	UniRef50_Q9FR44	0.0	Normal: 860	600	1097	1863	3056	624	BlastAnno: 3683	UniRef50_Q9FR44	491	80.33	488	96	0	3281	1818	1	488	0.0	838
ABCDEF_79861	UniRef50_Q9FR44	0.0	Normal: 842	512	1081	1821	3012	362	BlastAnno: 2781	UniRef50_Q9FR44	491	80.33	488	96	0	2429	966	1	488	0.0	838
ABCDEF_16953	UniRef50_Q9FR44	0.0	Normal: 864	596	1097	1861	3055	629	BlastAnno: 3756	UniRef50_Q9FR44	491	80.33	488	96	0	3354	1891	1	488	0.0	838

ABCDEF_85145	UniRef50_Q9FR44	0.0	Normal: 854	562	1088	1829	3028	569	BlastAnno: 3773	UniRef50_Q9FR44	491	80.33	488	96	0	3421	1958	1	488	0.0	838
2.1.1.17(phosphatidylethanolamine N-methyltransferase;)																					
No Correspond Unigene																					
2.1.1.71(phosphatidyl-N-methylethanolamine N-methyltransferase;)																					
No Correspond Unigene																					
2.3.1.-(Transferases;)																					
ABCDEF_520	UniRef50_Q9FF86	0.0	Normal: 1672	85	2273	5056	3383	1211	BlastAnno: 1811	UniRef50_Q9FF86	484	64.06	448	149	6	1664	354	3	449	0.0	573
ABCDEF_68664	UniRef50_Q9ZVN2	0.0	Normal: 79	35	85	107	193	63	BlastAnno: 1488	UniRef50_Q9ZVN2	704	63.99	411	142	3	46	1275	298	703	0.0	540
ABCDEF_52245	UniRef50_Q9FG87	0.0	Normal: 1878	635	1833	3814	1432	1376	BlastAnno: 1758	UniRef50_Q9FG87	529	55.35	486	204	4	4	1428	34	517	0.0	530
ABCDEF_16730	UniRef50_Q3EC11	0.0	Normal: 281	204	237	365	377	458	BlastAnno: 2092	UniRef50_Q3EC11	536	59.33	536	204	8	196	1785	3	530	0.0	661
ABCDEF_4655	UniRef50_A5BMG1	0.0	Normal: 2426	1856	3606	6958	4608	2755	BlastAnno: 2261	UniRef50_A5BMG1	481	68.37	528	112	10	263	1843	3	476	0.0	667
ABCDEF_13860	UniRef50_Q9C533	0.0	Normal: 588	220	570	1451	1338	310	BlastAnno: 2648	UniRef50_Q9C533	596	63.37	606	177	12	2226	511	1	595	0.0	558
ABCDEF_28294	UniRef50_Q9SYZ0	0.0	Normal: 1038	4070	1427	6745	877	2797	BlastAnno: 1785	UniRef50_Q9SYZ0	493	52.37	485	223	3	1598	162	5	487	0.0	533
ABCDEF_47718	UniRef50_Q9C533	0.0	Normal: 1599	236	1657	2093	1231	588	BlastAnno: 2680	UniRef50_Q9C533	596	65.02	626	165	14	2280	472	1	595	0.0	634
ABCDEF_45080	UniRef50_Q9SYZ0	0.0	Normal: 1005	592	1079	2360	3762	2217	BlastAnno: 2130	UniRef50_Q9SYZ0	493	57.85	484	197	3	302	1735	5	487	0.0	533
ABCDEF_25377	UniRef50_Q9C6L5	0.0	Normal: 37	2090	138	2573	1026	1298	BlastAnno: 1334	UniRef50_Q9C6L5	492	79.74	385	76	1	1155	1	6	388	0.0	654
ABCDEF_33470	UniRef50_Q9LW26	0.0	Normal: 172	98	156	260	391	149	BlastAnno: 2993	UniRef50_Q9LW26	701	48.96	627	301	5	655	2508	85	701	0.0	581
ABCDEF_40654	UniRef50_Q9H0A0	0.0	Normal: 638	213	530	318	318	286	BlastAnno: 2216	UniRef50_Q9H0A0	1025	56.87	721	279	6	2127	13	91	795	0.0	756
ABCDEF_33460	UniRef50_Q9ZVN2	0.0	Normal: 144	71	119	216	306	97	BlastAnno: 2691	UniRef50_Q9ZVN2	704	61.50	587	191	4	379	2133	83	636	0.0	715
ABCDEF_14442	UniRef50_Q9SYZ0	0.0	Normal: 341	684	439	1040	1601	552	BlastAnno: 1779	UniRef50_Q9SYZ0	493	63.12	480	171	2	1570	149	5	484	0.0	628
ABCDEF_52626	UniRef50_B6SYP9	0.0	Normal: 815	346	1131	3015	1537	108	BlastAnno: 1861	UniRef50_B6SYP9	494	55.02	438	189	3	1538	249	51	488	0.0	536
ABCDEF_644	UniRef50_Q9SB58	0.0	Normal: 704	444	913	969	1431	848	BlastAnno: 1777	UniRef50_Q9SB58	407	64.15	424	129	8	1572	316	1	406	0.0	554
ABCDEF_27087	UniRef50_Q52T38	0.0	Normal: 324	221	233	419	343	412	BlastAnno: 1631	UniRef50_Q52T38	620	75.95	420	91	1	1409	150	1	410	0.0	681
ABCDEF_34187	UniRef50_Q8L5Y5	0.0	Normal: 497	415	588	523	291	433	BlastAnno: 2677	UniRef50_Q8L5Y5	718	57.30	733	251	15	202	2274	1	713	0.0	683
ABCDEF_47966	UniRef50_Q9SYZ0	0.0	Normal: 577	934	326	580	682	3440	BlastAnno: 2177	UniRef50_Q9SYZ0	493	56.40	484	204	3	1780	347	5	487	0.0	535
ABCDEF_51189	UniRef50_Q52T38	0.0	Normal: 135	152	128	181	219	327	BlastAnno: 1766	UniRef50_Q52T38	620	68.09	470	137	3	80	1483	130	588	0.0	641
ABCDEF_41288	UniRef50_Q9SB58	0.0	Normal: 307	183	332	637	649	322	BlastAnno: 1403	UniRef50_Q9SB58	407	67.56	410	110	6	1401	184	15	405	0.0	523
ABCDEF_33461	UniRef50_Q9ZVN2	0.0	Normal: 142	84	140	206	283	119	BlastAnno: 2904	UniRef50_Q9ZVN2	704	60.98	574	201	6	2271	559	83	636	0.0	686
ABCDEF_14334	UniRef50_Q9SYZ0	0.0	Normal: 2263	129	1724	3469	866	283	BlastAnno: 2039	UniRef50_Q9SYZ0	493	63.22	484	171	2	369	1802	5	487	0.0	627
ABCDEF_24844	UniRef50_Q9FF86	0.0	Normal: 1857	3686	2657	21940	11467	4090	BlastAnno: 1640	UniRef50_Q9FF86	484	66.89	447	135	6	1492	185	2	446	0.0	575
ABCDEF_51190	UniRef50_Q52T38	0.0	Normal: 145	169	142	210	232	335	BlastAnno: 1891	UniRef50_Q52T38	620	70.04	514	141	3	73	1608	86	588	0.0	726
ABCDEF_14085	UniRef50_Q8L5Y5	0.0	Normal: 353	41	384	527	331	280	BlastAnno: 2362	UniRef50_Q8L5Y5	718	62.07	704	220	12	2360	348	24	713	0.0	676
ABCDEF_33458	UniRef50_Q9LW26	0.0	Normal: 158	78	130	240	342	106	BlastAnno: 2822	UniRef50_Q9LW26	701	46.72	655	302	5	400	2337	85	701	0.0	568
ABCDEF_25193	UniRef50_Q9C6L5	0.0	Normal: 3256	368	3548	6230	5109	1288	BlastAnno: 1786	UniRef50_Q9C6L5	492	65.83	480	156	4	1668	241	17	492	0.0	668
ABCDEF_33457	UniRef50_Q9ZVN2	0.0	Normal: 168	83	137	250	375	112	BlastAnno: 2823	UniRef50_Q9ZVN2	704	65.12	496	168	3	379	1860	83	575	0.0	648
ABCDEF_7634	UniRef50_Q9C6L5	0.0	Normal: 2543	9303	2711	11578	5533	10032	BlastAnno: 2064	UniRef50_Q9C6L5	492	52.76	489	225	3	1734	268	1	483	0.0	535
ABCDEF_299	UniRef50_Q71N54	0.0	Normal: 569	1147	511	900	649	1879	BlastAnno: 2017	UniRef50_Q71N54	535	61.73	533	198	5	338	1924	4	534	0.0	677
ABCDEF_27086	UniRef50_Q52T38	0.0	Normal: 784	470	617	843	893	976	BlastAnno: 2398	UniRef50_Q52T38	620	70.80	637	165	4	225	2129	1	618	0.0	887
ABCDEF_51188	UniRef50_Q52T38	0.0	Normal: 273	327	255	415	349	441	BlastAnno: 2324	UniRef50_Q52T38	620	71.30	568	150	3	2041	344	32	588	0.0	815
ABCDEF_44812	UniRef50_Q6DR03	0.0	Normal: 982	902	1005	1071	959	2999	BlastAnno: 2450	UniRef50_Q6DR03	565	54.97	644	198	17	2142	220	1	555	0.0	540
2.3.1.15(glycerol-3-phosphate O-acyltransferase;)																					
ABCDEF_31526	UniRef50_Q8GWG0	0.0	Normal: 854	674	926	1620	2095	1316	BlastAnno: 1574	UniRef50_Q8GWG0	376	86.52	371	49	1	1249	140	5	375	0.0	674
ABCDEF_26975	UniRef50_Q43307	0.0	Normal: 98	193	111	185	196	210	BlastAnno: 1701	UniRef50_Q43307	459	63.88	407	140	3	166	1365	53	459	0.0	546
ABCDEF_46632	UniRef50_Q9SHJ5	0.0	Normal: 0	815	0	0	0	6344	BlastAnno: 1852	UniRef50_Q9SHJ5	585	57.22	568	187	13	1682	141	20	585	0.0	561
ABCDEF_4142	UniRef50_Q9LMM0	0.0	Normal: 1866	2698	2190	6028	4010	4730	BlastAnno: 1842	UniRef50_Q9LMM0	503	77.31	498	110	2	82	1572	1	496	0.0	766
ABCDEF_26971	UniRef50_Q43307	0.0	Normal: 99	187	126	191	199	204	BlastAnno: 1761	UniRef50_Q43307	459	63.88	407	140	3	166	1365	53	459	0.0	546
ABCDEF_26970	UniRef50_Q43307	0.0	Normal: 105	209	133	218	233	251	BlastAnno: 1753	UniRef50_Q43307	459	63.88	407	140	3	187	1386	53	459	0.0	546
ABCDEF_14714	UniRef50_Q9LMM0	0.0	Normal: 50	5435	8	619	582	1352	BlastAnno: 1608	UniRef50_Q9LMM0	503	65.16	488	164	5	75	1526	10	495	0.0	642
2.3.1.23(1-acylglycerophosphocholine O-acyltransferase;)																					
ABCDEF_17404	UniRef50_Q0KHU5	2e-47	Normal: 236	330	418	625	795	763	BlastAnno: 1702	UniRef50_Q0KHU5	533	31.88	389	247	12	75	1205	50	432	2e-47	180
2.3.1.40(acyl-[acyl-carrier-protein]---phospholipid O-acyltransferase;)																					
No Correspond Unigene																					
2.3.1.42(glycerone-phosphate O-acyltransferase;)																					
No Correspond Unigene																					
2.3.1.43(phosphatidylcholine---sterol O-acyltransferase;)																					
ABCDEF_38602	UniRef50_A2R7I4	2e-86	Normal: 2042	1712	1900	4134	4265	3258	BlastAnno: 2886	UniRef50_A2R7I4	628	34.35	556	284	12	2387	726	120	596	2e-86	298
2.3.1.51(1-acylglycerol-3-phosphate O-acyltransferase;)																					
ABCDEF_20740	UniRef50_Q8LG50	0.0	Normal: 385	356	438	2043	1660	959	BlastAnno: 1640	UniRef50_Q8LG50	389	72.92	373	101	0	1425	307	14	386	0.0	577
ABCDEF_34747	UniRef50_Q8LG50	0.0	Normal: 1074	1007	1074	2022	1660	1853	BlastAnno: 1595	UniRef50_Q8LG50	389	72.49	378	104	0	1414	281	10	387	0.0	527
2.3.1.52(2-acylglycerol-3-phosphate O-acyltransferase;)																					
No Correspond Unigene																					
2.3.1.6(choline O-acetyltransferase;)																					
No Correspond Unigene																					
2.3.1.62(2-acylglycerophosphocholine O-acyltransferase;)																					
No Correspond Unigene																					
2.7.1.107(diacylglycerol kinase;)																					
ABCDEF_20845	UniRef50_Q9FFN7	0.0	Normal: 282	208	272	189	491	288	BlastAnno: 1902	UniRef50_Q9FFN7	712	53.43	554	233	7	1901	249	181	712	0.0	570
ABCDEF_24649	UniRef50_Q9FFN7	0.0	Normal: 66	112	93	228	428	96	BlastAnno: 1529	UniRef50_Q9FFN7	712	77.72	386	84	1	49	1206	329	712	0.0	596
2.7.1.32(choline kinase;)																					
ABCDEF_31120	UniRef50_Q9Y259	1e-50	Normal: 415	196	317	243	251	556	BlastAnno: 1441	UniRef50_Q9Y259	395	33.93	336	204	9	1216	260	56	390	1e-50	184
2.7.1.82(ethanolamine kinase;)																					
ABCDEF_7151	UniRef50_Q869T9	6e-70	Normal: 387	291	399	719	898	334	BlastAnno: 1516	UniRef50_Q869T9	349	35.47	344	219	2	1217	192	7	349	6e-70	235

2.7.7.14(ethanolamine-phosphate cytidylyltransferase;)																					
ABCDEF_38643	UniRef50_Q8IP80	9e-107	Normal: 795	331	818	723	945	948	BlastAnno: 1682	UniRef50_Q8IP80	381	46.70	364	181	5	233	1318	18	370	9e-107	334
2.7.7.15(choline-phosphate cytidylyltransferase;)																					
ABCDEF_32202	UniRef50_Q42555	2e-147	Normal: 520	385	514	732	930	652	BlastAnno: 1213	UniRef50_Q42555	331	76.01	271	60	2	1064	255	11	277	2e-147	430
2.7.7.39(glycerol-3-phosphate cytidylyltransferase;)																					
No Correspond Unigene																					
2.7.7.41(phosphatidate cytidylyltransferase;)																					
ABCDEF_20211	UniRef50_O04928	0.0	Normal: 290	270	272	296	395	334	BlastAnno: 1710	UniRef50_O04928	421	75.42	419	103	0	1548	292	1	419	0.0	666
ABCDEF_20213	UniRef50_O04928	0.0	Normal: 288	271	273	305	399	328	BlastAnno: 1840	UniRef50_O04928	421	75.42	419	103	0	1584	328	1	419	0.0	666
ABCDEF_20214	UniRef50_O04928	0.0	Normal: 309	294	281	314	418	347	BlastAnno: 1742	UniRef50_O04928	421	75.42	419	103	0	1625	369	1	419	0.0	666
ABCDEF_20212	UniRef50_O04928	0.0	Normal: 284	269	269	297	387	321	BlastAnno: 1760	UniRef50_O04928	421	75.42	419	103	0	1584	328	1	419	0.0	666
2.7.8.-(Transferases;)																					
ABCDEF_40506	UniRef50_C0QDU7	8e-33	Normal: 129	124	156	162	222	178	BlastAnno: 1387	UniRef50_C0QDU7	251	32.58	267	143	8	1342	542	10	239	8e-33	131
2.7.8.1(ethanolaminephosphotransferase;)																					
No Correspond Unigene																					
2.7.8.11(CDP-diacylglycerol---inositol 3-phosphatidyltransferase;)																					
ABCDEF_13953	UniRef50_Q8LBA6	1e-110	Normal: 195	122	191	243	296	189	BlastAnno: 1145	UniRef50_Q8LBA6	227	71.11	225	63	1	108	776	1	225	1e-110	332
2.7.8.2(diacylglycerol cholinephosphotransferase;)																					
ABCDEF_32672	UniRef50_Q8T0S3	2e-07	Normal: 201	91	155	307	429	270	BlastAnno: 1067	UniRef50_Q8T0S3	415	23.03	178	119	3	177	677	222	392	2e-07	58.2
2.7.8.24(phosphatidylcholine synthase;)																					
No Correspond Unigene																					
2.7.8.4(serine-phosphoethanolamine synthase;)																					
No Correspond Unigene																					
2.7.8.5(CDP-diacylglycerol---glycerol-3-phosphate 3-phosphatidyltransferase;)																					
ABCDEF_52021	UniRef50_B9SGD6	2e-86	Normal: 246	141	196	403	477	228	BlastAnno: 1410	UniRef50_B9SGD6	359	71.55	232	64	1	273	962	127	358	2e-86	278
2.7.8.8(CDP-diacylglycerol---serine O-phosphatidyltransferase;)																					
ABCDEF_69642	UniRef50_Q4QFL4	9e-30	Normal: 18	6	18	12	12	22	BlastAnno: 395	UniRef50_Q4QFL4	532	44.83	116	64	0	47	394	227	342	9e-30	118
3.1.1.-(Hydrolases;)																					
ABCDEF_25737	UniRef50_Q9CA68	0.0	Normal: 104	11	8	393	426	18	BlastAnno: 1232	UniRef50_Q9CA68	366	69.89	352	103	2	1182	130	17	366	0.0	522
ABCDEF_525	UniRef50_Q9LIN2	0.0	Normal: 213	16	167	378	456	158	BlastAnno: 1254	UniRef50_Q9LIN2	380	69.27	358	110	0	1097	24	23	380	0.0	535
ABCDEF_28091	UniRef50_O23522	0.0	Normal: 1192	678	424	81	156	3170	BlastAnno: 1887	UniRef50_O23522	601	57.90	525	191	12	168	1721	3	504	0.0	568
ABCDEF_13432	UniRef50_Q3ECP6	0.0	Normal: 712	1725	466	588	811	6988	BlastAnno: 2479	UniRef50_Q3ECP6	408	66.83	401	104	2	2397	1282	8	408	0.0	567
ABCDEF_72624	UniRef50_Q3ECP6	0.0	Normal: 22	38	25	267	382	149	BlastAnno: 1342	UniRef50_Q3ECP6	408	66.83	401	104	2	53	1168	8	408	0.0	567
ABCDEF_4432	UniRef50_B9IQA0	0.0	Normal: 680	298	798	834	1447	644	BlastAnno: 1533	UniRef50_B9IQA0	354	78.82	321	68	0	214	1176	33	353	0.0	556
3.1.1.32(phospholipase A1)																					
ABCDEF_52204	UniRef50_Q93V61	0.0	Normal: 621	312	886	815	680	334	BlastAnno: 1680	UniRef50_Q93V61	447	70.35	425	124	2	147	1418	16	439	0.0	608
3.1.1.4(phospholipase A2;)																					
ABCDEF_4059	UniRef50_B9SCE0	3e-50	Normal: 1020	374	939	865	1113	3015	BlastAnno: 767	UniRef50_B9SCE0	160	63.64	132	47	1	530	138	29	160	3e-50	170
3.1.1.5(lysophospholipase;)																					
ABCDEF_13732	UniRef50_Q5XTS1	4e-49	Normal: 597	440	687	1049	1047	576	BlastAnno: 3230	UniRef50_Q5XTS1	786	33.85	384	194	13	428	1540	420	756	4e-49	194
3.1.1.52(phosphatidylinositol deacylase;)																					
No Correspond Unigene																					
3.1.1.7(acetylcholinesterase;)																					
No Correspond Unigene																					
3.1.3.27(phosphatidylglycerophosphatase;)																					
No Correspond Unigene																					
3.1.3.4(phosphatidate phosphatase;)																					
ABCDEF_3583	UniRef50_Q9FVL1	9e-133	Normal: 418	155	337	228	574	484	BlastAnno: 1631	UniRef50_Q9FVL1	322	66.36	324	105	3	1344	379	1	322	9e-133	398
3.1.3.75(phosphoethanolamine/phosphocholine phosphatase;)																					
No Correspond Unigene																					
3.1.4.13(serine-ethanolaminephosphate phosphodiesterase;)																					
No Correspond Unigene																					
3.1.4.2(glycerophosphocholine phosphodiesterase;)																					
No Correspond Unigene																					
3.1.4.3(phospholipase C;)																					
ABCDEF_24368	UniRef50_Q22P01	1e-93	Normal: 158	107	203	472	477	108	BlastAnno: 1430	UniRef50_Q22P01	500	40.35	456	211	15	1308	61	4	438	1e-93	301
3.1.4.4(phospholipase D;)																					
ABCDEF_81656	UniRef50_B9GQ72	0.0	Normal: 911	492	1147	2677	3756	838	BlastAnno: 1912	UniRef50_B9GQ72	794	62.20	574	159	5	1911	193	107	623	0.0	759
ABCDEF_29990	UniRef50_Q9M9W8	0.0	Normal: 52	325	47	51	22	275	BlastAnno: 2175	UniRef50_Q9M9W8	1046	62.41	439	153	5	277	1581	39	469	0.0	525
ABCDEF_6945	UniRef50_B9GQ72	0.0	Normal: 130	210	114	401	396	481	BlastAnno: 2055	UniRef50_B9GQ72	794	56.12	654	223	10	2055	142	188	793	0.0	736
ABCDEF_6313	UniRef50_Q9M9W8	0.0	Normal: 258	323	267	747	653	343	BlastAnno: 3823	UniRef50_Q9M9W8	1046	62.26	1081	325	14	225	3437	39	1046	0.0	1329
ABCDEF_29986	UniRef50_Q9M9W8	0.0	Normal: 62	221	43	30	53	274	BlastAnno: 2218	UniRef50_Q9M9W8	1046	64.86	626	190	7	1	1839	438	1046	0.0	832
ABCDEF_6944	UniRef50_P93733	0.0	Normal: 45	89	67	182	186	265	BlastAnno: 1152	UniRef50_P93733	967	77.09	323	73	1	49	1014	645	967	0.0	538
ABCDEF_45705	UniRef50_Q69X21	0.0	Normal: 6254	4562	6868	11588	15822	7720	BlastAnno: 3278	UniRef50_Q69X21	842	60.81	842	298	14	2965	536	1	842	0.0	1019
ABCDEF_42077	UniRef50_B9F7I5	0.0	Normal: 2660	1447	3240	7162	9958	2163	BlastAnno: 3043	UniRef50_B9F7I5	829	55.81	869	318	13	2753	195	11	829	0.0	985
ABCDEF_35431	UniRef50_B9MX88	0.0	Normal: 830	552	1648	2900	795	1394	BlastAnno: 2809	UniRef50_B9MX88	881	46.26	802	367	16	2459	198	88	873	0.0	696
ABCDEF_29991	UniRef50_Q9M9W8	0.0	Normal: 28	100	17	10	12	121	BlastAnno: 1710	UniRef50_Q9M9W8	1046	66.99	418	121	4	1319	90	475	883	0.0	584
3.1.4.46(glycerophosphodiester phosphodiesterase;)																					
ABCDEF_1041	UniRef50_Q9FJ62	0.0	Normal: 3245	2724	2546	6164	10073	5137	BlastAnno: 2941	UniRef50_Q9FJ62	766	55.84	711	300	8	2654	546	19	723	0.0	821
3.6.1.16(CDP-glycerol diphosphatase;)																					
No Correspond Unigene																					
3.6.1.26(CDP-diacylglycerol diphosphatase;)																					

No Correspond Unigene

4.1.1.65(phosphatidylserine decarboxylase;)

ABCDEF_12029	UniRef50_Q9UG56	2e-22	Normal: 99	60	81	58	71	133	BlastAnno: 775	UniRef50_Q9UG56	408	45.05	111	56	2	157	489	301	406	2e-22	100

4.3.1.7(ethanolamine ammonia-lyase;)

No Correspond Unigene

4.3.3.-(Lyases;)

No Correspond Unigene

>00565 Ether lipid metabolism

1.1.1.101(acylglycerone-phosphate reductase;)

No Correspond Unigene

1.14.99.19(plasmanylethanolamine desaturase;)

No Correspond Unigene

2.3.1.-(Transferases;)

ABCDEF_520	UniRef50_Q9FF86	0.0	Normal: 1672	85	2273	5056	3383	1211	BlastAnno: 1811	UniRef50_Q9FF86	484	64.06	448	149	6	1664	354	3	449	0.0	573
ABCDEF_68664	UniRef50_Q9ZVN2	0.0	Normal: 79	35	85	107	193	63	BlastAnno: 1488	UniRef50_Q9ZVN2	704	63.99	411	142	3	46	1275	298	703	0.0	540
ABCDEF_52245	UniRef50_Q9FG87	0.0	Normal: 1878	635	1833	3814	1432	1376	BlastAnno: 1758	UniRef50_Q9FG87	529	55.35	486	204	4	4	1428	34	517	0.0	530
ABCDEF_16730	UniRef50_Q3EC11	0.0	Normal: 281	204	237	365	377	458	BlastAnno: 2092	UniRef50_Q3EC11	536	59.33	536	204	8	196	1785	3	530	0.0	661
ABCDEF_4655	UniRef50_A5BMG1	0.0	Normal: 2426	1856	3606	6958	4608	2755	BlastAnno: 2261	UniRef50_A5BMG1	481	68.37	528	112	10	263	1843	3	476	0.0	667
ABCDEF_13860	UniRef50_Q9C533	0.0	Normal: 588	220	570	1451	1338	310	BlastAnno: 2648	UniRef50_Q9C533	596	63.37	606	177	12	2226	511	1	595	0.0	558
ABCDEF_28294	UniRef50_Q9SYZ0	0.0	Normal: 1038	4070	1427	6745	877	2797	BlastAnno: 1785	UniRef50_Q9SYZ0	493	52.37	485	223	3	1598	162	5	487	0.0	533
ABCDEF_47718	UniRef50_Q9C533	0.0	Normal: 1599	236	1657	2093	1231	588	BlastAnno: 2680	UniRef50_Q9C533	596	65.02	626	165	14	2280	472	1	595	0.0	634
ABCDEF_45080	UniRef50_Q9SYZ0	0.0	Normal: 1005	592	1079	2360	3762	2217	BlastAnno: 2130	UniRef50_Q9SYZ0	493	57.85	484	197	3	302	1735	5	487	0.0	533
ABCDEF_25377	UniRef50_Q9C6L5	0.0	Normal: 37	2090	138	2573	1026	1298	BlastAnno: 1334	UniRef50_Q9C6L5	492	79.74	385	76	1	1155	1	6	388	0.0	654
ABCDEF_33470	UniRef50_Q9LW26	0.0	Normal: 172	98	156	260	391	149	BlastAnno: 2993	UniRef50_Q9LW26	701	48.96	627	301	5	655	2508	85	701	0.0	581
ABCDEF_40654	UniRef50_Q9H0A0	0.0	Normal: 638	213	530	318	318	286	BlastAnno: 2216	UniRef50_Q9H0A0	1025	56.87	721	279	6	2127	13	91	795	0.0	756
ABCDEF_33460	UniRef50_Q9ZVN2	0.0	Normal: 144	71	119	216	306	97	BlastAnno: 2691	UniRef50_Q9ZVN2	704	61.50	587	191	4	379	2133	83	636	0.0	715
ABCDEF_14442	UniRef50_Q9SYZ0	0.0	Normal: 341	684	439	1040	1601	552	BlastAnno: 1779	UniRef50_Q9SYZ0	493	63.12	480	171	2	1570	149	5	484	0.0	628
ABCDEF_52626	UniRef50_B6SYP9	0.0	Normal: 815	346	1131	3015	1537	108	BlastAnno: 1861	UniRef50_B6SYP9	494	55.02	438	189	3	1538	249	51	488	0.0	536
ABCDEF_644	UniRef50_Q9SB58	0.0	Normal: 704	444	913	969	1431	848	BlastAnno: 1777	UniRef50_Q9SB58	407	64.15	424	129	8	1572	316	1	406	0.0	554
ABCDEF_27087	UniRef50_Q52T38	0.0	Normal: 324	221	233	419	343	412	BlastAnno: 1631	UniRef50_Q52T38	620	75.95	420	91	1	1409	150	1	410	0.0	681
ABCDEF_34187	UniRef50_Q8L5Y5	0.0	Normal: 497	415	588	523	291	433	BlastAnno: 2677	UniRef50_Q8L5Y5	718	57.30	733	251	15	202	2274	1	713	0.0	683
ABCDEF_47966	UniRef50_Q9SYZ0	0.0	Normal: 577	934	326	580	682	3440	BlastAnno: 2177	UniRef50_Q9SYZ0	493	56.40	484	204	3	1780	347	5	487	0.0	535
ABCDEF_51189	UniRef50_Q52T38	0.0	Normal: 135	152	128	181	219	327	BlastAnno: 1766	UniRef50_Q52T38	620	68.09	470	137	3	80	1483	130	588	0.0	641
ABCDEF_41288	UniRef50_Q9SB58	0.0	Normal: 307	183	332	637	649	322	BlastAnno: 1403	UniRef50_Q9SB58	407	67.56	410	110	6	1401	184	15	405	0.0	523
ABCDEF_33461	UniRef50_Q9ZVN2	0.0	Normal: 142	84	140	206	283	119	BlastAnno: 2904	UniRef50_Q9ZVN2	704	60.98	574	201	6	2271	559	83	636	0.0	686
ABCDEF_14334	UniRef50_Q9SYZ0	0.0	Normal: 2263	129	1724	3469	866	283	BlastAnno: 2039	UniRef50_Q9SYZ0	493	63.22	484	171	2	369	1802	5	487	0.0	627
ABCDEF_24844	UniRef50_Q9FF86	0.0	Normal: 1857	3686	2657	21940	11467	4090	BlastAnno: 1640	UniRef50_Q9FF86	484	66.89	447	135	6	1492	185	2	446	0.0	575
ABCDEF_51190	UniRef50_Q52T38	0.0	Normal: 145	169	142	210	232	335	BlastAnno: 1891	UniRef50_Q52T38	620	70.04	514	141	3	73	1608	86	588	0.0	726
ABCDEF_14085	UniRef50_Q8L5Y5	0.0	Normal: 353	41	384	527	331	280	BlastAnno: 2362	UniRef50_Q8L5Y5	718	62.07	704	220	12	2360	348	24	713	0.0	676
ABCDEF_33458	UniRef50_Q9LW26	0.0	Normal: 158	78	130	240	342	106	BlastAnno: 2822	UniRef50_Q9LW26	701	46.72	655	302	5	400	2337	85	701	0.0	568
ABCDEF_25193	UniRef50_Q9C6L5	0.0	Normal: 3256	368	3548	6230	5109	1288	BlastAnno: 1786	UniRef50_Q9C6L5	492	65.83	480	156	4	1668	241	17	492	0.0	668
ABCDEF_33457	UniRef50_Q9ZVN2	0.0	Normal: 168	83	137	250	375	112	BlastAnno: 2823	UniRef50_Q9ZVN2	704	65.12	496	168	3	379	1860	83	575	0.0	648
ABCDEF_7634	UniRef50_Q9C6L5	0.0	Normal: 2543	9303	2711	11578	5533	10032	BlastAnno: 2064	UniRef50_Q9C6L5	492	52.76	489	225	3	1734	268	1	483	0.0	535
ABCDEF_299	UniRef50_Q71N54	0.0	Normal: 569	1147	511	900	649	1879	BlastAnno: 2017	UniRef50_Q71N54	535	61.73	533	198	5	338	1924	4	534	0.0	677
ABCDEF_27086	UniRef50_Q52T38	0.0	Normal: 784	470	617	843	893	976	BlastAnno: 2398	UniRef50_Q52T38	620	70.80	637	165	4	225	2129	1	618	0.0	887
ABCDEF_51188	UniRef50_Q52T38	0.0	Normal: 273	327	255	415	349	441	BlastAnno: 2324	UniRef50_Q52T38	620	71.30	568	150	3	2041	344	32	588	0.0	815
ABCDEF_44812	UniRef50_Q6DR03	0.0	Normal: 982	902	1005	1071	959	2999	BlastAnno: 2450	UniRef50_Q6DR03	565	54.97	644	198	17	2142	220	1	555	0.0	540

2.3.1.104(1-alkenylglycerophosphocholine O-acyltransferase)

No Correspond Unigene

2.3.1.105(alkylglycerophosphate 2-O-acetyltransferase;)

No Correspond Unigene

2.3.1.121(1-alkenylglycerophosphoethanolamine O-acyltransferase)

No Correspond Unigene

2.3.1.125(1-alkyl-2-acetylglycerol O-acyltransferase;)

No Correspond Unigene

2.3.1.25(plasmalogen synthase;)

No Correspond Unigene

2.3.1.63(1-alkylglycerophosphocholine O-acyltransferase)

No Correspond Unigene

2.3.1.67(1-alkylglycerophosphocholine O-acetyltransferase;)

No Correspond Unigene

2.5.1.26(alkylglycerone-phosphate synthase;)

No Correspond Unigene

2.6.-.-(Transferases;)

ABCDEF_3637	UniRef50_Q8LAD0	1e-121	Normal: 102	196	78	84	162	412	BlastAnno: 922	UniRef50_Q8LAD0	255	78.44	218	47	0	870	217	1	218	1e-121	358

2.7.1.93(alkylglycerol kinase;)

No Correspond Unigene

2.7.8.1(ethanolaminephosphotransferase;)

No Correspond Unigene

2.7.8.2(diacylglycerol cholinephosphotransferase;)

ABCDEF_32672	UniRef50_Q8T0S3	2e-07	Normal: 201	91	155	307	429	270	BlastAnno: 1067	UniRef50_Q8T0S3	415	23.03	178	119	3	177	677	222	392	2e-07	58.2

2.7.8.22(1-alkenyl-2-acylglycerol choline phosphotransferase;)

No Correspond Unigene																					
3.1.1.4(phospholipase A2;)																					
ABCDEF_4059	UniRef50_B9SCE0	3e-50	Normal: 1020	374	939	865	1113	3015	BlastAnno: 767	UniRef50_B9SCE0	160	63.64	132	47	1	530	138	29	160	3e-50	170
3.1.1.47(1-alkyl-2-acetylglycerophosphocholine esterase;)																					
ABCDEF_45371	UniRef50_B6KM18	1e-33	Normal: 539	377	486	932	1275	688	BlastAnno: 1536	UniRef50_B6KM18	311	36.28	215	126	3	605	1237	87	294	1e-33	135
3.1.3.-(Hydrolases;)																					
ABCDEF_3128	UniRef50_Q9ZU49	5e-117	Normal: 118	143	108	91	149	56	BlastAnno: 1434	UniRef50_Q9ZU49	327	57.43	303	121	2	1184	294	20	320	5e-117	355
3.1.3.4(phosphatidate phosphatase;)																					
ABCDEF_3583	UniRef50_Q9FVL1	9e-133	Normal: 418	155	337	228	574	484	BlastAnno: 1631	UniRef50_Q9FVL1	322	66.36	324	105	3	1344	379	1	322	9e-133	398
3.1.3.59(alkylacetylglycerophosphatase;)																					
No Correspond Unigene																					
3.1.4.-(Hydrolases;)																					
ABCDEF_30658	UniRef50_B0G107	3e-90	Normal: 633	283	458	191	395	752	BlastAnno: 1986	UniRef50_B0G107	514	41.27	424	236	7	272	1540	77	488	3e-90	298
3.1.4.3(phospholipase C;)																					
ABCDEF_24368	UniRef50_Q22P01	1e-93	Normal: 158	107	203	472	477	108	BlastAnno: 1430	UniRef50_Q22P01	500	40.35	456	211	15	1308	61	4	438	1e-93	301
3.1.4.39(alkylglycerophosphoethanolamine phosphodiesterase;)																					
No Correspond Unigene																					
3.1.4.4(phospholipase D;)																					
ABCDEF_81656	UniRef50_B9GQ72	0.0	Normal: 911	492	1147	2677	3756	838	BlastAnno: 1912	UniRef50_B9GQ72	794	62.20	574	159	5	1911	193	107	623	0.0	759
ABCDEF_29990	UniRef50_Q9M9W8	0.0	Normal: 52	325	47	51	22	275	BlastAnno: 2175	UniRef50_Q9M9W8	1046	62.41	439	153	5	277	1581	39	469	0.0	525
ABCDEF_6945	UniRef50_B9GQ72	0.0	Normal: 130	210	114	401	396	481	BlastAnno: 2055	UniRef50_B9GQ72	794	56.12	654	223	10	2055	142	188	793	0.0	736
ABCDEF_6313	UniRef50_Q9M9W8	0.0	Normal: 258	323	267	747	653	343	BlastAnno: 3823	UniRef50_Q9M9W8	1046	62.26	1081	325	14	225	3437	39	1046	0.0	1329
ABCDEF_29986	UniRef50_Q9M9W8	0.0	Normal: 62	221	43	30	53	274	BlastAnno: 2218	UniRef50_Q9M9W8	1046	64.86	626	190	7	1	1839	438	1046	0.0	832
ABCDEF_6944	UniRef50_P93733	0.0	Normal: 45	89	67	182	186	265	BlastAnno: 1152	UniRef50_P93733	967	77.09	323	73	1	49	1014	645	967	0.0	538
ABCDEF_45705	UniRef50_Q69X21	0.0	Normal: 6254	4562	6868	11588	15822	7720	BlastAnno: 3278	UniRef50_Q69X21	842	60.81	842	298	14	2965	536	1	842	0.0	1019
ABCDEF_42077	UniRef50_B9F7I5	0.0	Normal: 2660	1447	3240	7162	9958	2163	BlastAnno: 3043	UniRef50_B9F7I5	829	55.81	869	318	13	2753	195	11	829	0.0	985
ABCDEF_35431	UniRef50_B9MX88	0.0	Normal: 830	552	1648	2900	795	1394	BlastAnno: 2809	UniRef50_B9MX88	881	46.26	802	367	16	2459	198	88	873	0.0	696
ABCDEF_29991	UniRef50_Q9M9W8	0.0	Normal: 28	100	17	10	12	121	BlastAnno: 1710	UniRef50_Q9M9W8	1046	66.99	418	121	4	1319	90	475	883	0.0	584
3.3.2.2(alkenylglycerophosphocholine hydrolase;)																					
No Correspond Unigene																					
3.3.2.5(alkenylglycerophosphoethanolamine hydrolase)																					
No Correspond Unigene																					
>00590 Arachidonic acid metabolism																					
1.1.1.184(carbonyl reductase (NADPH);)																					
ABCDEF_34083	UniRef50_B9RL30	1e-148	Normal: 181	178	159	164	91	643	BlastAnno: 1390	UniRef50_B9RL30	325	73.65	277	69	1	1094	264	53	325	1e-148	436
ABCDEF_34084	UniRef50_B9RL30	1e-148	Normal: 156	158	148	148	96	804	BlastAnno: 1302	UniRef50_B9RL30	325	75.37	272	61	2	32	847	58	323	1e-148	434
1.1.1.188(prostaglandin-F synthase;)																					
No Correspond Unigene																					
1.1.1.189(prostaglandin-E2 9-reductase;)																					
No Correspond Unigene																					
1.1.1.196(15-hydroxyprostaglandin-D dehydrogenase (NADP+);)																					
No Correspond Unigene																					
1.1.1.232(15-hydroxyicosatetraenoate dehydrogenase;)																					
No Correspond Unigene																					
1.11.1.9(glutathione peroxidase;)																					
ABCDEF_37002	UniRef50_O23970	1e-77	Normal: 1065	641	1000	1091	1101	1152	BlastAnno: 5076	UniRef50_O23970	167	71.78	163	46	0	86	574	3	165	1e-77	262
1.13.11.-(Oxidoreductases;)																					
No Correspond Unigene																					
1.13.11.31(arachidonate 12-lipoxygenase;)																					
No Correspond Unigene																					
1.13.11.33(arachidonate 15-lipoxygenase;)																					
No Correspond Unigene																					
1.13.11.34(arachidonate 5-lipoxygenase;)																					
No Correspond Unigene																					
1.13.11.40(arachidonate 8-lipoxygenase;)																					
No Correspond Unigene																					
1.14.13.30(leukotriene-B4 20-monooxygenase;)																					
No Correspond Unigene																					
1.14.13.34(leukotriene-E4 20-monooxygenase;)																					
No Correspond Unigene																					
1.14.14.1(unspecific monooxygenase;)																					
ABCDEF_41955	UniRef50_B9S0F8	2e-174	Normal: 70	140	28	0	0	150	BlastAnno: 1118	UniRef50_B9S0F8	506	66.67	366	115	5	2	1099	138	496	2e-174	504
1.14.15.3(alkane 1-monooxygenase;)																					
No Correspond Unigene																					
1.14.99.1(prostaglandin-endoperoxide synthase;)																					
No Correspond Unigene																					
2.3.2.-(Transferases;)																					
ABCDEF_41698	UniRef50_Q9M8T3	7e-62	Normal: 110	102	151	190	213	210	BlastAnno: 870	UniRef50_Q9M8T3	182	58.29	187	63	4	740	210	1	182	7e-62	202
2.3.2.2(gamma-glutamyltransferase;)																					
ABCDEF_27037	UniRef50_Q60928	4e-127	Normal: 382	258	428	509	999	336	BlastAnno: 2914	UniRef50_Q60928	568	41.49	552	291	11	330	1961	39	566	4e-127	405
3.1.1.4(phospholipase A2;)																					
ABCDEF_4059	UniRef50_B9SCE0	3e-50	Normal: 1020	374	939	865	1113	3015	BlastAnno: 767	UniRef50_B9SCE0	160	63.64	132	47	1	530	138	29	160	3e-50	170

3.3.2.-(Hydrolases;)																					
No Correspond Unigene																					
3.3.2.10(soluble epoxide hydrolase;)																					
No Correspond Unigene																					
3.3.2.6(leukotriene-A4 hydrolase;)																					
ABCDEF_6172	UniRef50_A1ZG99	6e-130	Normal: 338	174	314	303	214	129	BlastAnno: 2698	UniRef50_A1ZG99	634	37.54	618	356	9	2149	302	44	633	6e-130	413
3.3.2.7(hepoxilin-epoxide hydrolase;)																					
No Correspond Unigene																					
4.4.1.20(leukotriene-C4 synthase;)																					
No Correspond Unigene																					
5.3.99.2(prostaglandin-D synthase;)																					
No Correspond Unigene																					
5.3.99.3(prostaglandin-E synthase;)																					
ABCDEF_7375	UniRef50_Q9H7Z7	5e-64	Normal: 535	138	606	639	1021	208	BlastAnno: 1225	UniRef50_Q9H7Z7	377	42.70	274	111	7	868	185	101	374	5e-64	218
5.3.99.4(prostaglandin-I synthase;)																					
No Correspond Unigene																					
5.3.99.5(thromboxane-A synthase;)																					
No Correspond Unigene																					
5.4.4.-(Isomerases;)																					
No Correspond Unigene																					
>00591 Linoleic acid metabolism																					
1.1.1.-(Oxidoreductases;)																					
ABCDEF_13453	UniRef50_Q9SYM5	0.0	Normal: 225	226	398	1950	2026	421	BlastAnno: 2578	UniRef50_Q9SYM5	669	86.61	672	87	2	298	2313	1	669	0.0	1226
ABCDEF_17342	UniRef50_Q84M96	0.0	Normal: 274	406	272	327	439	625	BlastAnno: 1277	UniRef50_Q84M96	346	73.33	345	91	1	1187	156	2	346	0.0	546
ABCDEF_31147	UniRef50_Q9SYM5	0.0	Normal: 4571	1591	1491	1401	1939	4327	BlastAnno: 2465	UniRef50_Q9SYM5	669	82.76	673	107	4	2397	394	1	669	0.0	1154
ABCDEF_17344	UniRef50_Q84M96	0.0	Normal: 252	257	297	380	540		BlastAnno: 1350	UniRef50_Q84M96	346	68.19	371	91	2	1263	154	2	346	0.0	530
ABCDEF_44674	UniRef50_Q9SYM5	0.0	Normal: 3581	2620	4329	9505	11447	4930	BlastAnno: 2634	UniRef50_Q9SYM5	669	87.80	672	78	3	253	2265	1	669	0.0	1241
1.13.11.12(lipoxygenase;)																					
ABCDEF_5470	UniRef50_B2ZUL4	0.0	Normal: 2034	4549	3158	2698	11160	14337	BlastAnno: 2985	UniRef50_B2ZUL4	777	50.19	787	340	14	2559	262	22	777	0.0	704
ABCDEF_27355	UniRef50_B8LLK5	0.0	Normal: 531	1196	1089	988	7503	3941	BlastAnno: 2962	UniRef50_B8LLK5	930	49.24	857	404	17	2746	239	84	930	0.0	790
ABCDEF_21194	UniRef50_C5Y999	0.0	Normal: 65	176	58	53	121	122	BlastAnno: 1236	UniRef50_C5Y999	924	66.10	413	138	2	1236	1	512	923	0.0	548
ABCDEF_36330	UniRef50_C5Y999	0.0	Normal: 27	6959	7	0	12	12445	BlastAnno: 2940	UniRef50_C5Y999	924	46.59	820	419	10	348	2765	110	924	0.0	719
ABCDEF_41794	UniRef50_B3TDK8	0.0	Normal: 1654	538	3133	5289	2109	1260	BlastAnno: 1795	UniRef50_B3TDK8	801	53.14	574	213	8	78	1793	8	527	0.0	600
ABCDEF_27360	UniRef50_B2ZUL4	0.0	Normal: 2173	3765	3190	2801	8094	12038	BlastAnno: 3307	UniRef50_B2ZUL4	777	46.95	835	343	15	2742	301	22	777	0.0	676
ABCDEF_396	UniRef50_C5Y999	0.0	Normal: 3480	11439	4278	393	854	6047	BlastAnno: 3082	UniRef50_C5Y999	924	62.20	828	304	6	2661	199	99	924	0.0	1030
ABCDEF_27354	UniRef50_B8LLK5	0.0	Normal: 1777	3877	2708	2389	9733	11715	BlastAnno: 2897	UniRef50_B8LLK5	930	48.36	856	413	15	2681	174	84	930	0.0	788
ABCDEF_27359	UniRef50_B3TDK8	0.0	Normal: 2222	3850	3300	2918	8342	12141	BlastAnno: 3326	UniRef50_B3TDK8	801	60.41	442	167	3	1704	3026	367	801	0.0	561
ABCDEF_45372	UniRef50_B2ZUL4	0.0	Normal: 2325	4034	3369	3012	8817	12832	BlastAnno: 3163	UniRef50_B2ZUL4	777	49.94	787	342	14	2598	301	22	777	0.0	696
ABCDEF_72848	UniRef50_B2ZUL4	0.0	Normal: 2737	4683	4147	3973	9757	13989	BlastAnno: 2917	UniRef50_B2ZUL4	777	49.94	787	342	14	358	2655	22	777	0.0	698
ABCDEF_27356	UniRef50_B8LLK5	0.0	Normal: 280	887	861	821	8604	1991	BlastAnno: 2897	UniRef50_B8LLK5	930	49.12	857	405	17	2681	174	84	930	0.0	792
ABCDEF_52642	UniRef50_C5Y999	0.0	Normal: 119	1782	149	71	0	3742	BlastAnno: 2986	UniRef50_C5Y999	924	50.66	829	387	9	2587	140	105	924	0.0	832
ABCDEF_27353	UniRef50_B8LLK5	0.0	Normal: 417	1225	927	844	8011	3982	BlastAnno: 2985	UniRef50_B8LLK5	930	49.36	857	403	17	2769	262	84	930	0.0	793
ABCDEF_45373	UniRef50_B2ZUL4	0.0	Normal: 2143	4290	3299	2859	9846	13267	BlastAnno: 2962	UniRef50_B2ZUL4	777	50.06	787	341	14	2536	239	22	777	0.0	700
1.13.11.33(arachidonate 15-lipoxygenase;)																					
No Correspond Unigene																					
1.13.11.44(linoleate diol synthase;)																					
No Correspond Unigene																					
1.13.11.45(linoleate 11-lipoxygenase;)																					
No Correspond Unigene																					
1.14.14.1(unspecific monooxygenase;)																					
ABCDEF_41955	UniRef50_B9S0F8	2e-174	Normal: 70	140	28	0	0	150	BlastAnno: 1118	UniRef50_B9S0F8	506	66.67	366	115	5	2	1099	138	496	2e-174	504
1.14.19.3(linoleoyl-CoA desaturase;)																					
ABCDEF_84064	UniRef50_A2QNS0	2e-20	Normal: 0	0	0	0	0	38	BlastAnno: 410	UniRef50_A2QNS0	557	34.71	121	77	2	408	46	255	373	2e-20	92.8
1.14.99.33(Delta12-fatty acid dehydrogenase;)																					
ABCDEF_17305	UniRef50_B8HN41	8e-122	Normal: 2147	7356	2747	1682	2466	18157	BlastAnno: 2000	UniRef50_B8HN41	360	54.34	346	148	4	681	1715	22	358	8e-122	375
3.1.1.4(phospholipase A2;)																					
ABCDEF_4059	UniRef50_B9SCE0	3e-50	Normal: 1020	374	939	865	1113	3015	BlastAnno: 767	UniRef50_B9SCE0	160	63.64	132	47	1	530	138	29	160	3e-50	170
3.3.2.-(Hydrolases;)																					
No Correspond Unigene																					
5.2.1.5(linoleate isomerase;)																					
No Correspond Unigene																					
>00592 alpha-Linolenic acid metabolism																					
1.-.-.-(Oxidoreductases)																					
ABCDEF_345	UniRef50_Q6Z690	0.0	Normal: 442	146	449	340	272	314	BlastAnno: 1658	UniRef50_Q6Z690	849	63.42	462	150	5	1657	314	387	843	0.0	589
ABCDEF_24068	UniRef50_Q9FI41	0.0	Normal: 214	316	207	270	263	849	BlastAnno: 1742	UniRef50_Q9FI41	387	73.28	363	96	1	1440	355	4	366	0.0	554
ABCDEF_24070	UniRef50_Q9FI41	0.0	Normal: 189	290	182	231	232	751	BlastAnno: 1479	UniRef50_Q9FI41	387	73.28	363	96	1	1363	278	4	366	0.0	551
ABCDEF_13957	UniRef50_Q9CAE3	0.0	Normal: 399	318	392	489	456	586	BlastAnno: 2488	UniRef50_Q9CAE3	789	80.85	496	93	2	3	1487	285	779	0.0	768
ABCDEF_28410	UniRef50_Q9LIG0	0.0	Normal: 211	366	234	286	433	1504	BlastAnno: 1324	UniRef50_Q9LIG0	330	74.92	331	76	2	1147	173	1	330	0.0	521
ABCDEF_17581	UniRef50_Q8LGI2	0.0	Normal: 758	336	735	426	374	852	BlastAnno: 1543	UniRef50_Q8LGI2	454	68.78	442	136	2	71	1390	8	449	0.0	597
ABCDEF_23753	UniRef50_Q94A68	0.0	Normal: 284	559	215	224	164	519	BlastAnno: 1580	UniRef50_Q94A68	377	81.90	348	63	0	1349	306	30	377	0.0	576
ABCDEF_24071	UniRef50_Q9FI41	0.0	Normal: 235	377	230	278	289	948	BlastAnno: 2090	UniRef50_Q9FI41	387	73.28	363	96	1	1726	641	4	366	0.0	553
1.13.11.12(lipoxygenase;)																					

ABCDEF_5470	UniRef50_B2ZUL4	0.0	Normal: 2034	4549	3158	2698	11160	14337	BlastAnno: 2985	UniRef50_B2ZUL4	777	50.19	787	340	14	2559	262	22	777	0.0	704
ABCDEF_27355	UniRef50_B8LLK5	0.0	Normal: 531	1196	1089	988	7503	3941	BlastAnno: 2962	UniRef50_B8LLK5	930	49.24	857	404	17	2746	239	84	930	0.0	790
ABCDEF_21194	UniRef50_C5Y999	0.0	Normal: 65	176	58	53	121	122	BlastAnno: 1236	UniRef50_C5Y999	924	66.10	413	138	2	1236	1	512	923	0.0	548
ABCDEF_36330	UniRef50_C5Y999	0.0	Normal: 27	6959	7	0	12	12445	BlastAnno: 2940	UniRef50_C5Y999	924	46.59	820	419	10	348	2765	110	924	0.0	719
ABCDEF_41794	UniRef50_B3TDK8	0.0	Normal: 1654	538	3133	5289	2109	1260	BlastAnno: 1795	UniRef50_B3TDK8	801	53.14	574	213	8	78	1793	8	527	0.0	600
ABCDEF_27360	UniRef50_B2ZUL4	0.0	Normal: 2173	3765	3190	2801	8094	12038	BlastAnno: 3307	UniRef50_B2ZUL4	777	46.95	835	343	15	2742	301	22	777	0.0	676
ABCDEF_396	UniRef50_C5Y999	0.0	Normal: 3480	11439	4278	393	854	6047	BlastAnno: 3082	UniRef50_C5Y999	924	62.20	828	304	6	2661	199	99	924	0.0	1030
ABCDEF_27354	UniRef50_B8LLK5	0.0	Normal: 1777	3877	2708	2389	9733	11715	BlastAnno: 2897	UniRef50_B8LLK5	930	48.36	856	413	15	2681	174	84	930	0.0	788
ABCDEF_27359	UniRef50_B3TDK8	0.0	Normal: 2222	3850	3300	2918	8342	12141	BlastAnno: 3326	UniRef50_B3TDK8	801	60.41	442	167	3	1704	3026	367	801	0.0	561
ABCDEF_45372	UniRef50_B2ZUL4	0.0	Normal: 2325	4034	3369	3012	8817	12832	BlastAnno: 3163	UniRef50_B2ZUL4	777	49.94	787	342	14	2598	301	22	777	0.0	696
ABCDEF_72848	UniRef50_B2ZUL4	0.0	Normal: 2737	4683	4147	3973	9757	13989	BlastAnno: 2917	UniRef50_B2ZUL4	777	49.94	787	342	14	358	2655	22	777	0.0	698
ABCDEF_27356	UniRef50_B8LLK5	0.0	Normal: 280	887	861	821	8604	1991	BlastAnno: 2897	UniRef50_B8LLK5	930	49.12	857	405	17	2681	174	84	930	0.0	792
ABCDEF_52642	UniRef50_C5Y999	0.0	Normal: 119	1782	149	71	0	3742	BlastAnno: 2986	UniRef50_C5Y999	924	50.66	829	387	9	2587	140	105	924	0.0	832
ABCDEF_27353	UniRef50_B8LLK5	0.0	Normal: 417	1225	927	844	8011	3982	BlastAnno: 2985	UniRef50_B8LLK5	930	49.36	857	403	17	2769	262	84	930	0.0	793
ABCDEF_45373	UniRef50_B2ZUL4	0.0	Normal: 2143	4290	3299	2859	9846	13267	BlastAnno: 2962	UniRef50_B2ZUL4	777	50.06	787	341	14	2536	239	22	777	0.0	700
1.14.19.-(Oxidoreductases;)																					
ABCDEF_68581	UniRef50_P46313	0.0	Normal: 449	280	996	3089	6141	798	BlastAnno: 1521	UniRef50_P46313	383	78.65	384	80	2	161	1309	1	383	0.0	635
ABCDEF_27864	UniRef50_P46313	0.0	Normal: 584	132	627	1501	1415	692	BlastAnno: 1543	UniRef50_P46313	383	72.32	383	105	1	149	1294	1	383	0.0	595
ABCDEF_17305	UniRef50_P46310	0.0	Normal: 2147	7356	2747	1682	2466	18157	BlastAnno: 2000	UniRef50_P46310	446	71.15	454	110	4	399	1733	1	442	0.0	645
ABCDEF_14699	UniRef50_P46313	0.0	Normal: 7783	2392	7230	5399	5958	7201	BlastAnno: 1602	UniRef50_P46313	383	78.12	384	81	3	1318	173	1	383	0.0	633
ABCDEF_52196	UniRef50_P46313	0.0	Normal: 538	298	1014	3158	6637	944	BlastAnno: 1569	UniRef50_P46313	383	78.65	384	80	2	1493	345	1	383	0.0	635
ABCDEF_21843	UniRef50_P46312	0.0	Normal: 900	1727	931	619	692	6984	BlastAnno: 1783	UniRef50_P46312	448	75.39	447	99	4	1489	179	1	446	0.0	696
ABCDEF_30626	UniRef50_P46310	0.0	Normal: 1035	405	871	878	1314	1183	BlastAnno: 1652	UniRef50_P46310	446	70.66	351	103	0	263	1315	87	437	0.0	537
ABCDEF_30625	UniRef50_P46310	0.0	Normal: 1075	395	893	876	1315	1191	BlastAnno: 1918	UniRef50_P46310	446	70.66	351	103	0	1390	338	87	437	0.0	538
ABCDEF_30627	UniRef50_P46310	0.0	Normal: 1267	459	1001	998	1563	1645	BlastAnno: 1594	UniRef50_P46310	446	70.66	351	103	0	205	1257	87	437	0.0	538
ABCDEF_27863	UniRef50_P46313	0.0	Normal: 576	108	647	1576	1499	622	BlastAnno: 1538	UniRef50_P46313	383	72.32	383	105	1	145	1290	1	383	0.0	595
ABCDEF_27862	UniRef50_P46313	0.0	Normal: 600	107	650	1584	1468	620	BlastAnno: 1642	UniRef50_P46313	383	72.32	383	105	1	249	1394	1	383	0.0	595
1.3.1.42(12-oxophytodienoate reductase;)																					
ABCDEF_28545	UniRef50_Q9FUP0	0.0	Normal: 316	578	828	881	1312	1187	BlastAnno: 1678	UniRef50_Q9FUP0	391	72.89	391	98	2	271	1440	8	391	0.0	603
ABCDEF_52773	UniRef50_Q8GYB8	0.0	Normal: 12	91	19	0	0	741	BlastAnno: 1158	UniRef50_Q8GYB8	374	71.74	368	104	0	55	1158	4	371	0.0	573
ABCDEF_49372	UniRef50_Q8GYB8	0.0	Normal: 316	282	413	553	590	1110	BlastAnno: 1458	UniRef50_Q8GYB8	374	79.62	368	75	0	184	1287	7	374	0.0	641
1.3.3.6(acyl-CoA oxidase;)																					
ABCDEF_21603	UniRef50_O65202	0.0	Normal: 2124	1274	2483	4743	4370	1598	BlastAnno: 2326	UniRef50_O65202	664	79.07	664	139	0	119	2110	1	664	0.0	1139
ABCDEF_72872	UniRef50_Q96329	0.0	Normal: 14	7	25	269	10	24	BlastAnno: 1147	UniRef50_Q96329	436	77.52	347	77	1	3	1043	89	434	0.0	546
ABCDEF_23427	UniRef50_O65201	0.0	Normal: 210	178	168	322	346	166	BlastAnno: 2152	UniRef50_O65201	692	77.86	646	141	2	2005	68	1	644	0.0	1083
ABCDEF_24471	UniRef50_O65202	0.0	Normal: 429	533	717	1111	1686	1212	BlastAnno: 2664	UniRef50_O65202	664	77.56	664	149	0	277	2268	1	664	0.0	1092
ABCDEF_30962	UniRef50_O65201	0.0	Normal: 372	143	289	426	378	1018	BlastAnno: 3341	UniRef50_O65201	692	83.26	639	105	1	33	1949	56	692	0.0	1147
ABCDEF_82073	UniRef50_O65202	0.0	Normal: 141	159	270	330	550	458	BlastAnno: 1373	UniRef50_O65202	664	79.77	341	69	0	207	1229	324	664	0.0	572
ABCDEF_24472	UniRef50_O65202	0.0	Normal: 316	373	448	878	1096	796	BlastAnno: 1868	UniRef50_O65202	664	77.30	511	116	0	1592	60	1	511	0.0	841
ABCDEF_64475	UniRef50_O65202	0.0	Normal: 321	390	542	801	1250	840	BlastAnno: 2286	UniRef50_O65202	664	76.85	635	147	0	143	2047	30	664	0.0	1029
ABCDEF_25385	UniRef50_Q96329	0.0	Normal: 747	1014	820	1304	1917	2681	BlastAnno: 1768	UniRef50_Q96329	436	80.93	430	82	0	112	1401	1	430	0.0	757
2.1.1.141(jasmonate O-methyltransferase;)																					
ABCDEF_38345	UniRef50_B9STB2	3e-125	Normal: 184	1398	158	237	251	979	BlastAnno: 1421	UniRef50_B9STB2	363	51.64	366	167	5	102	1184	1	361	3e-125	377
2.3.1.16(acetyl-CoA C-acyltransferase;)																					
ABCDEF_47228	UniRef50_P09110	1e-153	Normal: 321	497	306	408	1144	369	BlastAnno: 1559	UniRef50_P09110	424	59.95	387	152	3	1397	237	37	420	1e-153	454
3.1.1.32(phospholipase A1)																					
ABCDEF_52204	UniRef50_Q93V61	0.0	Normal: 621	312	886	815	680	334	BlastAnno: 1680	UniRef50_Q93V61	447	70.35	425	124	2	147	1418	16	439	0.0	608
3.1.1.4(phospholipase A2;)																					
ABCDEF_4059	UniRef50_B9SCE0	3e-50	Normal: 1020	374	939	865	1113	3015	BlastAnno: 767	UniRef50_B9SCE0	160	63.64	132	47	1	530	138	29	160	3e-50	170
3.1.2.-(Hydrolases;)																					
ABCDEF_2988	UniRef50_Q39513	0.0	Normal: 684	868	676	1224	1298	1392	BlastAnno: 1728	UniRef50_Q39513	415	69.13	392	111	5	408	1574	12	396	0.0	554
ABCDEF_42638	UniRef50_Q9SHJ8	0.0	Normal: 183	172	331	565	390	214	BlastAnno: 1547	UniRef50_Q9SHJ8	395	68.62	376	94	2	196	1320	42	394	0.0	533
ABCDEF_56804	UniRef50_Q9SHJ8	0.0	Normal: 160	146	249	472	328	155	BlastAnno: 1439	UniRef50_Q9SHJ8	395	68.62	376	94	2	275	1399	42	394	0.0	533
ABCDEF_2987	UniRef50_Q39513	0.0	Normal: 608	561	472	1162	1435	954	BlastAnno: 1938	UniRef50_Q39513	415	67.26	394	119	5	408	1580	12	398	0.0	544
ABCDEF_20456	UniRef50_Q9LK08	0.0	Normal: 375	366	460	796	1014	454	BlastAnno: 1539	UniRef50_Q9LK08	418	64.52	420	140	3	1434	187	4	418	0.0	559
ABCDEF_49725	UniRef50_Q9SHJ8	0.0	Normal: 176	174	327	582	397	195	BlastAnno: 1556	UniRef50_Q9SHJ8	395	68.62	376	94	2	234	1358	42	394	0.0	533
ABCDEF_20453	UniRef50_Q9LK08	0.0	Normal: 396	358	461	824	1019	461	BlastAnno: 1565	UniRef50_Q9LK08	418	66.90	420	134	2	1459	200	4	418	0.0	586
4.1.2.-(Lyases;)																					
ABCDEF_38428	UniRef50_C3KN71	1e-47	Normal: 551	427	606	695	1516	1338	BlastAnno: 1495	UniRef50_C3KN71	273	38.34	253	150	1	334	1092	7	253	1e-47	173
4.2.1.17(enoyl-CoA hydratase;)																					
ABCDEF_21259	UniRef50_Q8W1L6	0.0	Normal: 272	276	284	384	326	305	BlastAnno: 2641	UniRef50_Q8W1L6	726	47.57	721	375	3	114	2267	6	726	0.0	633
ABCDEF_80649	UniRef50_Q8W1L6	0.0	Normal: 527	841	443	1308	1321	1188	BlastAnno: 2586	UniRef50_Q8W1L6	726	60.39	712	281	1	2403	268	1	711	0.0	818
ABCDEF_466	UniRef50_Q8W1L6	0.0	Normal: 1014	1690	1094	1677	2438	2080	BlastAnno: 2453	UniRef50_Q8W1L6	726	71.24	699	201	0	2368	272	7	705	0.0	954
ABCDEF_54834	UniRef50_Q8W1L6	0.0	Normal: 229	220	237	298	270	261	BlastAnno: 2405	UniRef50_Q8W1L6	726	47.52	606	315	3	274	2082	121	726	0.0	580
ABCDEF_34756	UniRef50_Q8W1L6	0.0	Normal: 776	726	768	2922	4039	1079	BlastAnno: 2469	UniRef50_Q8W1L6	726	68.93	708	217	2	2396	273	7	711	0.0	957
ABCDEF_4198	UniRef50_Q8W1L6	0.0	Normal: 609	929	532	1401	1568	1297	BlastAnno: 2667	UniRef50_Q8W1L6	726	60.45	713	281	1	2463	325	1	712	0.0	820
4.2.1.92(hydroperoxide dehydratase;)																					
ABCDEF_48797	UniRef50_Q96242	3e-162	Normal: 77	133	102	15	351	316	BlastAnno: 1269	UniRef50_Q96242	518	60.88	386	150	1	1156	2	2	387	3e-162	476
5.3.3.-(Isomerases;)																					
No Correspond Unigene																					
5.3.99.6(allene-oxide cyclase)																					
ABCDEF_28227	UniRef50_B9SEJ3	1e-78	Normal: 537	205	632	478	1409	1144	BlastAnno: 1281	UniRef50_B9SEJ3	259	74.71	174	43	1	384	902	86	259	1e-78	252

6.2.1.-(Ligases;)

ABCDEF_7378	UniRef50_Q9FFE6	0.0	Normal: 1022	1422	746	576	680	1408	BlastAnno: 2117	UniRef50_Q9FFE6	552	67.57	552	167	5	144	1778	1	547	0.0	768
ABCDEF_47513	UniRef50_Q8RU95	0.0	Normal: 411	609	570	233	749	887	BlastAnno: 2280	UniRef50_Q8RU95	598	53.15	572	246	6	119	1777	28	596	0.0	543
ABCDEF_78742	UniRef50_Q84P25	0.0	Normal: 236	207	373	576	450	306	BlastAnno: 1534	UniRef50_Q84P25	565	60.39	462	168	5	1413	49	19	472	0.0	546
ABCDEF_10753	UniRef50_Q9FFE6	0.0	Normal: 394	401	336	253	292	348	BlastAnno: 1841	UniRef50_Q9FFE6	552	51.00	551	259	5	117	1751	1	546	0.0	553
ABCDEF_4431	UniRef50_Q9FFE6	0.0	Normal: 27	358	28	98	112	914	BlastAnno: 2075	UniRef50_Q9FFE6	552	50.71	560	264	7	1774	107	1	552	0.0	551
ABCDEF_38213	UniRef50_Q84P25	0.0	Normal: 342	337	597	798	722	505	BlastAnno: 1939	UniRef50_Q84P25	565	62.34	555	194	5	1818	175	19	565	0.0	690
ABCDEF_25221	UniRef50_Q0DV32	0.0	Normal: 971	826	1003	1543	1442	1545	BlastAnno: 2009	UniRef50_Q0DV32	552	57.20	542	226	4	183	1793	9	549	0.0	613
ABCDEF_23413	UniRef50_Q84P23	0.0	Normal: 123	237	158	149	213	658	BlastAnno: 3175	UniRef50_Q84P23	562	51.74	547	243	8	3030	1435	15	555	0.0	555
ABCDEF_20486	UniRef50_Q9FFE6	0.0	Normal: 580	912	312	324	864	1565	BlastAnno: 3530	UniRef50_Q9FFE6	552	66.61	557	171	5	352	1995	1	551	0.0	781

>00600 Sphingolipid metabolism

1.1.1.102(3-dehydrosphinganine reductase;)

ABCDEF_45353	UniRef50_B9RTW7	1e-160	Normal: 220	152	189	333	396	370	BlastAnno: 1251	UniRef50_B9RTW7	456	75.17	298	73	1	1150	257	29	325	1e-160	469

1.14.-.-(Oxidoreductases;)

ABCDEF_44739	UniRef50_O64989	0.0	Normal: 157	87	159	718	673	189	BlastAnno: 2185	UniRef50_O64989	513	70.75	482	112	3	1867	509	30	511	0.0	706
ABCDEF_31491	UniRef50_Q93VK5	0.0	Normal: 647	1826	671	1517	1770	5953	BlastAnno: 2278	UniRef50_Q93VK5	595	79.14	556	113	2	198	1862	35	588	0.0	902
ABCDEF_40554	UniRef50_P37122	0.0	Normal: 1574	1962	1829	1150	2882	2721	BlastAnno: 1828	UniRef50_P37122	505	60.52	504	198	1	1752	244	1	504	0.0	638
ABCDEF_34049	UniRef50_O23365	0.0	Normal: 363	384	349	442	549	1416	BlastAnno: 2101	UniRef50_O23365	580	76.17	579	116	5	230	1957	19	578	0.0	904
ABCDEF_21579	UniRef50_O48928	0.0	Normal: 1005	643	1606	4372	3226	1621	BlastAnno: 1895	UniRef50_O48928	513	69.72	502	151	1	185	1690	7	507	0.0	731
ABCDEF_37741	UniRef50_P37122	0.0	Normal: 12	760	9	24	34	576	BlastAnno: 1197	UniRef50_P37122	505	65.70	379	129	1	1195	59	127	504	0.0	526
ABCDEF_35618	UniRef50_O48928	0.0	Normal: 28	2648	17	142	1234	902	BlastAnno: 1746	UniRef50_O48928	513	66.40	506	168	2	1649	132	4	507	0.0	701
ABCDEF_24522	UniRef50_P37122	0.0	Normal: 334	2190	561	268	1391	2459	BlastAnno: 2075	UniRef50_P37122	505	61.52	460	176	1	1544	165	47	505	0.0	612
ABCDEF_14592	UniRef50_O23066	0.0	Normal: 1514	402	2245	7837	4742	1248	BlastAnno: 2378	UniRef50_O23066	553	72.71	546	140	4	2111	489	1	542	0.0	821
ABCDEF_14512	UniRef50_Q9FG65	0.0	Normal: 261	872	212	291	175	789	BlastAnno: 1878	UniRef50_Q9FG65	502	56.89	501	197	9	1738	266	11	502	0.0	532
ABCDEF_44487	UniRef50_Q940V4	0.0	Normal: 3	41	53	501	262	542	BlastAnno: 1712	UniRef50_Q940V4	465	69.37	382	116	1	38	1180	14	395	0.0	572
ABCDEF_17723	UniRef50_P37122	0.0	Normal: 240	1698	219	768	592	794	BlastAnno: 1757	UniRef50_P37122	505	63.40	459	166	2	228	1601	47	504	0.0	617
ABCDEF_37901	UniRef50_Q42569	0.0	Normal: 30	149	86	255	195	255	BlastAnno: 1246	UniRef50_Q42569	472	78.27	359	73	2	64	1140	43	396	0.0	567
ABCDEF_7260	UniRef50_O81117	0.0	Normal: 314	1085	466	3248	2575	1360	BlastAnno: 1768	UniRef50_O81117	514	59.92	474	188	1	145	1566	43	514	0.0	615
ABCDEF_40548	UniRef50_P37122	0.0	Normal: 2756	2467	2752	1072	3120	1735	BlastAnno: 1947	UniRef50_P37122	505	59.72	504	202	1	241	1749	1	504	0.0	636
ABCDEF_44485	UniRef50_Q940V4	0.0	Normal: 3	55	63	574	338	660	BlastAnno: 1607	UniRef50_Q940V4	465	68.58	452	141	1	42	1394	14	465	0.0	673
ABCDEF_40552	UniRef50_P37122	0.0	Normal: 2397	1870	2304	1249	10428	2291	BlastAnno: 1812	UniRef50_P37122	505	60.12	504	200	1	108	1616	1	504	0.0	640
ABCDEF_37902	UniRef50_Q42569	0.0	Normal: 84	524	254	725	670	1049	BlastAnno: 1812	UniRef50_Q42569	472	77.47	435	92	3	1675	371	43	471	0.0	681
ABCDEF_40556	UniRef50_P37122	0.0	Normal: 1907	2365	2163	1319	3678	3308	BlastAnno: 1944	UniRef50_P37122	505	61.04	498	193	1	264	1754	1	498	0.0	635
ABCDEF_40549	UniRef50_P37122	0.0	Normal: 1574	1962	1826	1150	2882	2723	BlastAnno: 1845	UniRef50_P37122	505	60.52	504	198	1	1754	246	1	504	0.0	638
ABCDEF_20301	UniRef50_O81117	0.0	Normal: 89	308	123	176	389	249	BlastAnno: 1637	UniRef50_O81117	514	64.04	470	164	3	1498	98	47	514	0.0	586
ABCDEF_3882	UniRef50_Q42569	0.0	Normal: 84	243	257	786	1561	182	BlastAnno: 1854	UniRef50_Q42569	472	72.51	422	107	1	1852	587	59	471	0.0	615
ABCDEF_20703	UniRef50_O49858	0.0	Normal: 115	636	20	0	0	597	BlastAnno: 1815	UniRef50_O49858	527	50.10	523	248	6	48	1607	12	524	0.0	533
ABCDEF_18295	UniRef50_O22203	0.0	Normal: 3667	1934	4977	10491	4588	1917	BlastAnno: 2006	UniRef50_O22203	508	74.85	493	123	1	214	1692	17	508	0.0	803
ABCDEF_48724	UniRef50_O23066	0.0	Normal: 104	11518	163	2083	3464	6356	BlastAnno: 2162	UniRef50_O23066	553	72.96	540	143	3	227	1840	1	539	0.0	807
ABCDEF_37738	UniRef50_P37122	0.0	Normal: 26	2447	19	30	33	993	BlastAnno: 1837	UniRef50_P37122	505	64.30	479	169	2	1685	249	28	504	0.0	639

2.3.1.24(sphingosine N-acyltransferase;)

No Correspond Unigene

2.3.1.50(serine C-palmitoyltransferase;)

ABCDEF_38491	UniRef50_O15270	6e-163	Normal: 1385	1327	1488	2541	2661	2159	BlastAnno: 1991	UniRef50_O15270	562	52.84	475	218	3	1799	393	65	539	6e-163	488

2.4.1.-(Transferases;)

ABCDEF_18004	UniRef50_Q6YWK8	0.0	Normal: 1562	1736	2009	3452	4438	2400	BlastAnno: 2298	UniRef50_Q6YWK8	570	65.27	429	136	1	1941	694	57	485	0.0	608
ABCDEF_52379	UniRef50_Q9LHZ7	0.0	Normal: 4015	4921	4546	10520	13430	4731	BlastAnno: 3479	UniRef50_Q9LHZ7	1170	48.04	893	342	16	825	3242	299	1156	0.0	787
ABCDEF_48593	UniRef50_Q9SK82	0.0	Normal: 12	420	19	343	763	253	BlastAnno: 1834	UniRef50_Q9SK82	489	60.08	481	186	4	1714	278	10	486	0.0	633
ABCDEF_13375	UniRef50_Q8VZK9	0.0	Normal: 108	123	208	526	34	294	BlastAnno: 1738	UniRef50_Q8VZK9	729	53.62	552	234	8	94	1737	18	551	0.0	582
ABCDEF_40982	UniRef50_Q96301	0.0	Normal: 1037	706	819	1249	1084	920	BlastAnno: 3418	UniRef50_Q96301	914	79.60	902	173	3	243	2948	1	891	0.0	1506
ABCDEF_47674	UniRef50_Q9LHZ7	0.0	Normal: 274	92	202	599	505	280	BlastAnno: 2526	UniRef50_Q9LHZ7	1170	75.93	777	151	8	2260	2	15	779	0.0	1177
ABCDEF_34097	UniRef50_Q9LE59	0.0	Normal: 626	613	923	1797	1829	692	BlastAnno: 2521	UniRef50_Q9LE59	673	60.92	458	178	1	1687	317	215	672	0.0	630
ABCDEF_34098	UniRef50_Q9LE59	0.0	Normal: 457	464	697	1283	1312	539	BlastAnno: 2199	UniRef50_Q9LE59	673	60.92	458	178	1	1687	317	215	672	0.0	628
ABCDEF_10848	UniRef50_Q9M156	0.0	Normal: 157	2988	163	408	772	1566	BlastAnno: 1810	UniRef50_Q9M156	480	60.09	456	176	4	1700	336	4	454	0.0	553
ABCDEF_41182	UniRef50_Q9LHZ7	0.0	Normal: 150	2169	221	814	1407	495	BlastAnno: 3604	UniRef50_Q9LHZ7	1170	51.03	870	333	15	2822	456	299	1156	0.0	826
ABCDEF_40981	UniRef50_Q96301	0.0	Normal: 156	97	110	160	179	173	BlastAnno: 1225	UniRef50_Q96301	914	86.92	344	45	0	1112	81	394	737	0.0	652
ABCDEF_9506	UniRef50_Q9LHZ7	0.0	Normal: 1302	2128	1494	3356	4644	2206	BlastAnno: 3751	UniRef50_Q9LHZ7	1170	49.38	891	343	17	927	3359	299	1161	0.0	812
ABCDEF_9500	UniRef50_Q9LHZ7	0.0	Normal: 183	188	186	576	498	260	BlastAnno: 1717	UniRef50_Q9LHZ7	1170	47.98	644	234	14	3	1715	418	1033	0.0	571
ABCDEF_100	UniRef50_Q9M8Y0	0.0	Normal: 228	309	224	416	461	141	BlastAnno: 2109	UniRef50_Q9M8Y0	977	77.49	533	120	0	1610	12	50	582	0.0	884
ABCDEF_33659	UniRef50_Q9LHZ7	0.0	Normal: 1282	1948	2248	5826	10642	2083	BlastAnno: 4289	UniRef50_Q9LHZ7	1170	48.70	887	345	15	2955	538	299	1156	0.0	788
ABCDEF_37667	UniRef50_Q9LHZ7	0.0	Normal: 248	3617	251	1237	2078	970	BlastAnno: 3161	UniRef50_Q9LHZ7	1170	49.72	885	335	14	2671	260	301	1156	0.0	810
ABCDEF_47673	UniRef50_Q9LHZ7	0.0	Normal: 876	296	828	1628	1721	1172	BlastAnno: 3878	UniRef50_Q9LHZ7	1170	80.88	1161	186	8	3642	232	15	1163	0.0	1865
ABCDEF_99	UniRef50_Q9M8Y0	0.0	Normal: 272	330	222	398	423	145	BlastAnno: 2094	UniRef50_Q9M8Y0	977	77.36	561	127	0	1683	1	50	610	0.0	929
ABCDEF_20658	UniRef50_Q8VZK9	0.0	Normal: 373	33	193	64	503	141	BlastAnno: 2316	UniRef50_Q8VZK9	729	53.35	508	221	7	47	1564	11	504	0.0	559
ABCDEF_10183	UniRef50_A7XDQ9	0.0	Normal: 422	455	346	577	378	610	BlastAnno: 2362	UniRef50_A7XDQ9	684	50.94	693	307	12	281	2287	1	684	0.0	670
ABCDEF_30745	UniRef50_A7XDQ9	0.0	Normal: 1209	351	1031	1645	1226	635	BlastAnno: 2646	UniRef50_A7XDQ9	684	57.58	554	227	6	546	2189	133	684	0.0	664
ABCDEF_13336	UniRef50_Q7PC69	0.0	Normal: 148	563	194	957	1575	816	BlastAnno: 2584	UniRef50_Q7PC69	745	71.56	559	136	6	744	2351	187	745	0.0	730
ABCDEF_21426	UniRef50_Q8RU27	0.0	Normal: 2658	1979	3417	6157	9464	2599	BlastAnno: 1530	UniRef50_Q8RU27	366	89.44	360	34	2	1358	282	1	357	0.0	667
ABCDEF_71242	UniRef50_Q9ZV71	0.0	Normal: 134	122	179	480	754	119	BlastAnno: 1835	UniRef50_Q9ZV71	409	73.35	409	103	4	231	1439	1	409	0.0	587
ABCDEF_52020	UniRef50_Q6YWK8	0.0	Normal: 1635	1534	1975	4284	10353	2289	BlastAnno: 2394	UniRef50_Q6YWK8	570	58.37	514	185	2	403	1857	57	570	0.0	607
ABCDEF_51269	UniRef50_Q6YWK8	0.0	Normal: 147	85	103	21	33	438	BlastAnno: 1864	UniRef50_Q6YWK8	570	54.28	514	204	2	1646	198	57	570	0.0	586
ABCDEF_30753	UniRef50_Q9ZV71	0.0	Normal: 290	497	417	629	1013	634	BlastAnno: 1502	UniRef50_Q9ZV71	409	72.32	401	107	2	311	1501	1	401	0.0	590

ABCDEF_4233	UniRef50_Q9ZV71	0.0	Normal: 369	412	550	936	860	750	BlastAnno: 2004	UniRef50_Q9ZV71	409	72.13	409	110	2	370	1584	1	409	0.0	613
ABCDEF_17525	UniRef50_Q8RU27	0.0	Normal: 1500	1289	2149	3711	4446	1069	BlastAnno: 1507	UniRef50_Q8RU27	366	91.35	347	30	0	1371	331	3	349	0.0	671
ABCDEF_18005	UniRef50_Q6YWK8	0.0	Normal: 1842	2042	2515	4214	5208	2842	BlastAnno: 2305	UniRef50_Q6YWK8	570	59.14	514	180	2	1948	497	57	570	0.0	642
ABCDEF_41140	UniRef50_Q6YWK8	0.0	Normal: 86	1314	93	753	1787	2076	BlastAnno: 2346	UniRef50_Q6YWK8	570	60.00	510	174	2	431	1870	57	566	0.0	669
ABCDEF_18002	UniRef50_Q9LHZ7	0.0	Normal: 376	127	367	601	1190	520	BlastAnno: 1605	UniRef50_Q9LHZ7	1170	89.75	400	41	0	1603	404	771	1170	0.0	732
ABCDEF_13339	UniRef50_Q7PC69	0.0	Normal: 127	814	183	541	1135	1144	BlastAnno: 2610	UniRef50_Q7PC69	745	70.48	559	141	6	725	2329	187	745	0.0	729
ABCDEF_41141	UniRef50_Q6YWK8	0.0	Normal: 67	1107	84	708	1690	1854	BlastAnno: 2461	UniRef50_Q6YWK8	570	60.00	510	174	2	1916	477	57	566	0.0	668
ABCDEF_44583	UniRef50_Q9LE59	0.0	Normal: 681	561	559	947	1745	789	BlastAnno: 2470	UniRef50_Q9LE59	673	59.21	456	185	1	1749	385	214	669	0.0	621
ABCDEF_3869	UniRef50_Q9LE59	0.0	Normal: 333	393	415	809	907	754	BlastAnno: 2136	UniRef50_Q9LE59	673	59.05	613	227	4	1827	1	81	673	0.0	699
ABCDEF_28947	UniRef50_Q9SK82	0.0	Normal: 0	851	3	0	0	116	BlastAnno: 1567	UniRef50_Q9SK82	489	62.40	484	176	4	1524	85	1	482	0.0	652
ABCDEF_31119	UniRef50_Q9M8Y0	0.0	Normal: 2212	1279	1917	2933	3341	1803	BlastAnno: 3695	UniRef50_Q9M8Y0	977	81.08	925	174	1	392	3163	50	974	0.0	1583
ABCDEF_37311	UniRef50_Q9LE59	0.0	Normal: 175	133	191	549	421	366	BlastAnno: 1612	UniRef50_Q9LE59	673	62.96	486	153	4	1406	3	4	480	0.0	542
ABCDEF_24190	UniRef50_Q8L7F9	0.0	Normal: 397	265	492	583	553	371	BlastAnno: 2564	UniRef50_Q8L7F9	643	63.91	640	218	4	2353	473	1	640	0.0	850
ABCDEF_30548	UniRef50_Q9FH36	0.0	Normal: 17	916	27	151	420	126	BlastAnno: 1963	UniRef50_Q9FH36	535	72.47	534	138	3	102	1676	1	534	0.0	767
ABCDEF_27416	UniRef50_Q7PC69	0.0	Normal: 575	837	875	1344	2015	936	BlastAnno: 2454	UniRef50_Q7PC69	745	66.11	416	115	4	2337	1168	126	541	0.0	518
ABCDEF_48373	UniRef50_A7XDQ9	0.0	Normal: 1364	773	1336	1451	1791	1202	BlastAnno: 3042	UniRef50_A7XDQ9	684	63.68	691	234	6	2405	360	1	683	0.0	915
ABCDEF_3868	UniRef50_Q9LE59	0.0	Normal: 224	274	301	555	686	524	BlastAnno: 1670	UniRef50_Q9LE59	673	65.31	516	176	1	1556	9	161	673	0.0	696
ABCDEF_31288	UniRef50_Q940V3	0.0	Normal: 247	309	183	101	124	655	BlastAnno: 1658	UniRef50_Q940V3	470	56.01	466	197	7	1614	232	8	470	0.0	537
ABCDEF_52574	UniRef50_Q09332	0.0	Normal: 885	474	1068	1835	1853	816	BlastAnno: 3720	UniRef50_Q09332	1548	47.89	687	310	14	2551	533	859	1511	0.0	598
ABCDEF_79752	UniRef50_Q9ZV71	0.0	Normal: 158	183	245	400	379	352	BlastAnno: 1266	UniRef50_Q9ZV71	409	71.39	360	99	2	31	1098	1	360	0.0	526
ABCDEF_9509	UniRef50_Q9LHZ7	0.0	Normal: 1296	2171	1498	3410	4683	2232	BlastAnno: 3897	UniRef50_Q9LHZ7	1170	49.38	891	343	17	1073	3505	299	1161	0.0	812
ABCDEF_48200	UniRef50_Q6YWK8	0.0	Normal: 1768	576	3156	4431	6634	491	BlastAnno: 2274	UniRef50_Q6YWK8	570	57.98	514	185	4	291	1739	57	570	0.0	642
ABCDEF_24155	UniRef50_Q9ZVI7	0.0	Normal: 503	247	607	1676	1833	578	BlastAnno: 2708	UniRef50_Q9ZVI7	619	52.33	621	264	11	306	2123	15	618	0.0	601
ABCDEF_21246	UniRef50_Q9SKT6	0.0	Normal: 92	107	188	380	834	84	BlastAnno: 1791	UniRef50_Q9SKT6	536	56.61	484	204	5	123	1562	52	533	0.0	568
ABCDEF_32317	UniRef50_Q8RU27	0.0	Normal: 12	9	3	118	800	62	BlastAnno: 1076	UniRef50_Q8RU27	366	87.02	285	37	0	1075	221	64	348	0.0	535
ABCDEF_9502	UniRef50_Q9LHZ7	0.0	Normal: 610	486	568	1331	1403	591	BlastAnno: 1961	UniRef50_Q9LHZ7	1170	48.64	625	249	13	3	1742	564	1161	0.0	570
ABCDEF_23298	UniRef50_Q9FH36	0.0	Normal: 128	527	130	223	314	864	BlastAnno: 2832	UniRef50_Q9FH36	535	83.16	196	33	0	696	1283	340	535	0.0	357
ABCDEF_40595	UniRef50_O80898	0.0	Normal: 229	135	103	25	307	325	BlastAnno: 2413	UniRef50_O80898	757	44.68	761	378	13	95	2299	10	753	0.0	587
ABCDEF_63947	UniRef50_Q9SK82	0.0	Normal: 0	267	0	0	71	0	BlastAnno: 1446	UniRef50_Q9SK82	489	58.33	468	189	4	60	1445	1	468	0.0	592
ABCDEF_67847	UniRef50_Q9M8Y0	0.0	Normal: 794	476	871	1081	1401	868	BlastAnno: 1591	UniRef50_Q9M8Y0	977	84.86	370	56	0	1589	480	605	974	0.0	685
ABCDEF_65666	UniRef50_Q09332	0.0	Normal: 800	441	946	1606	1637	735	BlastAnno: 3518	UniRef50_Q09332	1548	47.54	671	305	13	1162	3087	859	1511	0.0	586
ABCDEF_38641	UniRef50_Q9FWA4	0.0	Normal: 674	967	1162	2603	4404	2475	BlastAnno: 1599	UniRef50_Q9FWA4	561	81.35	386	72	0	1	1158	176	561	0.0	641
ABCDEF_17914	UniRef50_Q9SKT6	0.0	Normal: 784	567	886	1447	1439	1424	BlastAnno: 2049	UniRef50_Q9SKT6	536	72.95	536	140	2	204	1796	1	536	0.0	785
ABCDEF_18286	UniRef50_Q6NQB7	0.0	Normal: 1131	1108	1225	1872	1722	3329	BlastAnno: 1752	UniRef50_Q6NQB7	393	73.80	397	81	6	1527	367	10	393	0.0	607
ABCDEF_119	UniRef50_Q8RU27	0.0	Normal: 1613	1155	2249	3558	7663	1775	BlastAnno: 1532	UniRef50_Q8RU27	366	88.33	360	38	2	1306	230	1	357	0.0	663
ABCDEF_47603	UniRef50_Q9M9Y5	0.0	Normal: 2609	8779	1664	1103	2085	22991	BlastAnno: 2830	UniRef50_Q9M9Y5	589	54.04	594	267	3	2475	694	1	588	0.0	654
ABCDEF_24192	UniRef50_Q8L7F9	0.0	Normal: 391	244	465	553	497	345	BlastAnno: 2477	UniRef50_Q8L7F9	643	58.99	434	165	4	212	1474	1	434	0.0	513
ABCDEF_17482	UniRef50_Q9SKT6	0.0	Normal: 615	379	816	1068	1470	852	BlastAnno: 2083	UniRef50_Q9SKT6	536	71.11	540	147	4	1915	311	1	536	0.0	786
ABCDEF_85909	UniRef50_Q8VZK9	0.0	Normal: 28	45	9	10	44	127	BlastAnno: 1473	UniRef50_Q8VZK9	729	55.37	475	198	6	1419	1	11	473	0.0	536
ABCDEF_9503	UniRef50_Q9LHZ7	0.0	Normal: 487	544	524	1242	1320	833	BlastAnno: 2087	UniRef50_Q9LHZ7	1170	50.35	574	236	8	57	1712	615	1161	0.0	567
ABCDEF_40594	UniRef50_O80898	0.0	Normal: 256	127	102	41	331	326	BlastAnno: 2417	UniRef50_O80898	757	45.76	719	348	12	213	2291	51	753	0.0	585
ABCDEF_17141	UniRef50_Q9LHZ7	0.0	Normal: 74	1734	69	380	701	333	BlastAnno: 3337	UniRef50_Q9LHZ7	1170	50.92	868	335	14	2539	179	299	1156	0.0	816
ABCDEF_33658	UniRef50_Q9LHZ7	0.0	Normal: 384	308	395	805	1003	602	BlastAnno: 2961	UniRef50_Q9LHZ7	1170	48.76	888	343	14	204	2621	299	1156	0.0	792
ABCDEF_20579	UniRef50_Q7PC69	0.0	Normal: 1007	795	1238	2311	2309	1345	BlastAnno: 3113	UniRef50_Q7PC69	745	59.97	767	229	14	2667	535	1	745	0.0	768
ABCDEF_31118	UniRef50_Q9M8Y0	0.0	Normal: 2135	1267	1900	3004	3329	1757	BlastAnno: 3787	UniRef50_Q9M8Y0	977	81.10	709	133	1	1132	3255	266	974	0.0	1217
ABCDEF_9499	UniRef50_Q9LHZ7	0.0	Normal: 205	207	215	632	538	275	BlastAnno: 1794	UniRef50_Q9LHZ7	1170	48.88	667	240	14	11	1792	395	1033	0.0	605
ABCDEF_48199	UniRef50_Q6YWK8	0.0	Normal: 1506	467	2429	3094	4233	439	BlastAnno: 1997	UniRef50_Q6YWK8	570	63.10	439	146	2	291	1559	57	495	0.0	610
ABCDEF_40983	UniRef50_Q96301	0.0	Normal: 406	261	343	392	415	433	BlastAnno: 2265	UniRef50_Q96301	914	79.17	504	99	1	114	1625	394	891	0.0	854
ABCDEF_17140	UniRef50_Q9LHZ7	0.0	Normal: 45	1049	49	216	386	208	BlastAnno: 2293	UniRef50_Q9LHZ7	1170	52.34	770	281	13	2138	57	397	1156	0.0	739
ABCDEF_18352	UniRef50_Q9FWA4	0.0	Normal: 2427	2688	2969	5519	6547	4855	BlastAnno: 2224	UniRef50_Q9FWA4	561	69.31	505	148	3	1887	382	61	561	0.0	683
ABCDEF_27767	UniRef50_Q9LHZ7	0.0	Normal: 2576	5141	2841	10249	14583	5031	BlastAnno: 3981	UniRef50_Q9LHZ7	1170	49.66	888	337	16	2927	504	299	1156	0.0	818
ABCDEF_33661	UniRef50_Q9LHZ7	0.0	Normal: 414	340	507	879	790	783	BlastAnno: 2185	UniRef50_Q9LHZ7	1170	48.66	748	277	14	2184	169	440	1156	0.0	662
ABCDEF_14685	UniRef50_A7XDQ9	0.0	Normal: 233	308	134	573	292	334	BlastAnno: 1947	UniRef50_A7XDQ9	684	56.84	468	186	6	569	1945	1	461	0.0	545
ABCDEF_20660	UniRef50_Q8VZK9	0.0	Normal: 22	48	13	15	52	144	BlastAnno: 1473	UniRef50_Q8VZK9	729	55.16	475	199	6	55	1473	11	473	0.0	537
ABCDEF_21315	UniRef50_Q9LHZ7	0.0	Normal: 3412	252	1450	0	79	1228	BlastAnno: 3765	UniRef50_Q9LHZ7	1170	63.78	1096	340	15	308	3508	70	1137	0.0	1333
ABCDEF_533	UniRef50_Q9C8W3	0.0	Normal: 230	204	269	505	411	301	BlastAnno: 1688	UniRef50_Q9C8W3	401	70.33	364	94	2	393	1475	47	399	0.0	555
ABCDEF_34099	UniRef50_Q9LE59	0.0	Normal: 478	491	735	1356	1350	548	BlastAnno: 2158	UniRef50_Q9LE59	673	60.92	458	178	1	1610	240	215	672	0.0	630
ABCDEF_47984	UniRef50_Q7PC69	0.0	Normal: 663	457	734	1362	2147	696	BlastAnno: 2838	UniRef50_Q7PC69	745	59.27	712	226	9	373	2316	34	745	0.0	702
ABCDEF_51451	UniRef50_Q9LE59	0.0	Normal: 1883	1782	2202	4337	4350	2667	BlastAnno: 2874	UniRef50_Q9LE59	673	74.16	685	165	6	2559	505	1	673	0.0	982
ABCDEF_13370	UniRef50_Q8VZK9	0.0	Normal: 290	87	317	377	105	258	BlastAnno: 2033	UniRef50_Q8VZK9	729	51.65	635	278	11	2031	154	112	726	0.0	612
ABCDEF_48239	UniRef50_Q9LHZ7	0.0	Normal: 511	163	422	1154	1316	510	BlastAnno: 2714	UniRef50_Q9LHZ7	1170	76.20	769	156	9	478	2712	41	806	0.0	1201
ABCDEF_13338	UniRef50_Q7PC69	0.0	Normal: 335	468	417	2639	4443	616	BlastAnno: 2525	UniRef50_Q7PC69	745	62.45	727	209	12	298	2292	21	745	0.0	781
ABCDEF_24193	UniRef50_Q8L7F9	0.0	Normal: 113	72	132	203	180	119	BlastAnno: 1679	UniRef50_Q8L7F9	643	60.54	479	176	4	212	1609	1	479	0.0	592
ABCDEF_48514	UniRef50_Q9ZV71	0.0	Normal: 540	580	601	771	1033	926	BlastAnno: 1920	UniRef50_Q9ZV71	409	78.14	398	85	2	1618	431	12	409	0.0	637

2.4.1.23(sphingosine beta-galactosyltransferase;)

No Correspond Unigene

2.4.1.47(N-acylsphingosine galactosyltransferase;)

No Correspond Unigene

2.4.1.80(ceramide glucosyltransferase;)

No Correspond Unigene

2.7.1.138(ceramide kinase;)

ABCDEF_27212	UniRef50_Q8TCT0	1e-27	Normal: 56	44	79	124	120	62	BlastAnno: 781	UniRef50_Q8TCT0	537	30.97	268	133	5	719	6	286	531	1e-27	116

2.7.1.91(sphinganine kinase;)																					
ABCDEF_59409	UniRef50_Q6B516	2e-08	Normal: 77	76	89	154	124	61	BlastAnno: 770	UniRef50_Q6B516	760	31.41	191	97	7	647	84	209	368	2e-08	60.5
2.7.8.-(Transferases;)																					
ABCDEF_40506	UniRef50_C0QDU7	8e-33	Normal: 129	124	156	162	222	178	BlastAnno: 1387	UniRef50_C0QDU7	251	32.58	267	143	8	1342	542	10	239	8e-33	131
2.7.8.10(sphingosine cholinephosphotransferase;)																					
No Correspond Unigene																					
2.7.8.27(sphingomyelin synthase;)																					
No Correspond Unigene																					
2.7.8.3(ceramide cholinephosphotransferase;)																					
No Correspond Unigene																					
2.8.2.11(galactosylceramide sulfotransferase;)																					
No Correspond Unigene																					
3.1.3.-(Hydrolases;)																					
ABCDEF_3128	UniRef50_Q9ZU49	5e-117	Normal: 118	143	108	91	149	56	BlastAnno: 1434	UniRef50_Q9ZU49	327	57.43	303	121	2	1184	294	20	320	5e-117	355
3.1.3.4(phosphatidate phosphatase;)																					
ABCDEF_3583	UniRef50_Q9FVL1	9e-133	Normal: 418	155	337	228	574	484	BlastAnno: 1631	UniRef50_Q9FVL1	322	66.36	324	105	3	1344	379	1	322	9e-133	398
3.1.4.12(sphingomyelin phosphodiesterase;)																					
ABCDEF_42507	UniRef50_B0E6F9	4e-76	Normal: 1560	430	1812	2063	3935	1555	BlastAnno: 1137	UniRef50_B0E6F9	231	56.46	209	85	2	889	266	28	231	4e-76	243
ABCDEF_51291	UniRef50_B0E6F9	4e-76	Normal: 224	120	236	457	675	197	BlastAnno: 1421	UniRef50_B0E6F9	231	56.02	216	90	2	1035	391	20	231	4e-76	246
3.1.4.41(sphingomyelin phosphodiesterase D;)																					
No Correspond Unigene																					
3.1.6.1(arylsulfatase;)																					
ABCDEF_89419	UniRef50_B6KE10	4e-08	Normal: 15	7	10	10	17	150	BlastAnno: 346	UniRef50_B6KE10	750	46.94	49	26	0	316	170	694	742	4e-08	56.6
3.1.6.8(cerebroside-sulfatase;)																					
No Correspond Unigene																					
3.2.1.18(exo-alpha-sialidase;)																					
ABCDEF_30704	UniRef50_B0RQ59	6e-65	Normal: 92	41	67	66	82	84	BlastAnno: 1280	UniRef50_B0RQ59	355	42.63	319	163	7	145	1056	36	349	6e-65	220
3.2.1.22(alpha-galactosidase;)																					
ABCDEF_31352	UniRef50_Q42656	0.0	Normal: 115	169	48	7	155	1183	BlastAnno: 1530	UniRef50_Q42656	378	76.76	370	85	1	242	1351	10	378	0.0	613
ABCDEF_52628	UniRef50_Q42656	0.0	Normal: 1131	2713	1338	2471	4073	4919	BlastAnno: 1435	UniRef50_Q42656	378	65.22	368	127	1	1227	124	11	377	0.0	523
ABCDEF_10850	UniRef50_Q42656	0.0	Normal: 170	155	153	167	153	166	BlastAnno: 1524	UniRef50_Q42656	378	74.66	371	94	0	389	1501	8	378	0.0	578
3.2.1.23(beta-galactosidase;)																					
ABCDEF_48113	UniRef50_Q6Z6K4	0.0	Normal: 61	2624	32	127	673	5080	BlastAnno: 2898	UniRef50_Q6Z6K4	729	63.35	704	250	4	2505	394	33	728	0.0	931
ABCDEF_23777	UniRef50_Q6Z6K4	0.0	Normal: 219	63	227	164	235	472	BlastAnno: 2261	UniRef50_Q6Z6K4	729	66.38	690	225	5	2	2071	46	728	0.0	944
ABCDEF_3074	UniRef50_Q6Z6K4	0.0	Normal: 431	1551	321	2473	394	19728	BlastAnno: 3342	UniRef50_Q6Z6K4	729	67.09	702	221	6	415	2517	35	727	0.0	994
ABCDEF_43854	UniRef50_Q6Z6K4	0.0	Normal: 1	9	1	105	49	28	BlastAnno: 1781	UniRef50_Q6Z6K4	729	58.79	512	180	6	1	1536	147	627	0.0	595
ABCDEF_44469	UniRef50_Q8W0A1	0.0	Normal: 162	225	175	701	194	597	BlastAnno: 2319	UniRef50_Q8W0A1	827	46.10	679	324	12	2236	221	183	826	0.0	583
ABCDEF_13827	UniRef50_Q6Z6K4	0.0	Normal: 219	1054	213	1958	627	863	BlastAnno: 3038	UniRef50_Q6Z6K4	729	57.90	715	270	8	419	2539	36	727	0.0	825
ABCDEF_21624	UniRef50_Q6Z6K4	0.0	Normal: 4958	5483	4088	4956	4209	6920	BlastAnno: 3330	UniRef50_Q6Z6K4	729	62.64	696	251	4	2975	888	32	718	0.0	932
ABCDEF_43857	UniRef50_Q6Z6K4	0.0	Normal: 2	9	4	110	53	30	BlastAnno: 1655	UniRef50_Q6Z6K4	729	60.07	546	187	6	1	1638	147	661	0.0	655
ABCDEF_43861	UniRef50_Q6Z6K4	0.0	Normal: 4	15	5	162	72	47	BlastAnno: 1891	UniRef50_Q6Z6K4	729	65.21	526	179	3	107	1684	34	555	0.0	719
ABCDEF_84604	UniRef50_Q6Z6K4	0.0	Normal: 34	1696	14	81	379	2927	BlastAnno: 2528	UniRef50_Q6Z6K4	729	62.99	670	240	4	394	2403	33	694	0.0	881
ABCDEF_13826	UniRef50_Q6Z6K4	0.0	Normal: 184	889	166	1515	526	703	BlastAnno: 2897	UniRef50_Q6Z6K4	729	55.30	613	243	8	2286	472	138	727	0.0	657
ABCDEF_10626	UniRef50_Q6Z6K4	0.0	Normal: 229	909	224	1002	2471	3794	BlastAnno: 3133	UniRef50_Q6Z6K4	729	57.14	700	285	8	498	2588	40	727	0.0	809
ABCDEF_27471	UniRef50_Q6Z6K4	0.0	Normal: 992	533	1105	1489	2430	1240	BlastAnno: 3113	UniRef50_Q6Z6K4	729	64.49	704	240	5	314	2422	35	729	0.0	962
ABCDEF_43858	UniRef50_Q6Z6K4	0.0	Normal: 6	15	7	186	85	54	BlastAnno: 2028	UniRef50_Q6Z6K4	729	65.35	635	213	5	107	2011	34	661	0.0	862
ABCDEF_31373	UniRef50_Q6Z6K4	0.0	Normal: 1333	1029	1160	1241	1216	1855	BlastAnno: 3006	UniRef50_Q6Z6K4	729	52.03	713	319	7	2761	626	37	727	0.0	732
ABCDEF_10066	UniRef50_Q93Z24	0.0	Normal: 999	932	1523	1674	2007	1465	BlastAnno: 3015	UniRef50_Q93Z24	697	65.89	645	208	4	2659	737	61	697	0.0	865
ABCDEF_51808	UniRef50_Q6Z6K4	0.0	Normal: 364	454	209	36	62	1109	BlastAnno: 2817	UniRef50_Q6Z6K4	729	54.56	713	306	8	2582	447	32	727	0.0	791
ABCDEF_48111	UniRef50_Q6Z6K4	0.0	Normal: 41	1700	15	77	347	2953	BlastAnno: 2607	UniRef50_Q6Z6K4	729	67.21	494	157	3	394	1875	33	521	0.0	696
ABCDEF_17519	UniRef50_A5F5U6	0.0	Normal: 1178	1482	1392	2252	1850	3281	BlastAnno: 3706	UniRef50_A5F5U6	1024	39.71	1093	547	21	3469	254	13	1014	0.0	729
ABCDEF_40816	UniRef50_Q6Z6K4	0.0	Normal: 1166	754	898	2543	5298	532	BlastAnno: 3066	UniRef50_Q6Z6K4	729	62.55	705	254	4	2970	856	35	729	0.0	936
ABCDEF_44668	UniRef50_Q8GX69	0.0	Normal: 146	74	84	327	133	152	BlastAnno: 1396	UniRef50_Q8GX69	815	66.74	463	152	2	6	1394	23	483	0.0	671
ABCDEF_43856	UniRef50_Q6Z6K4	0.0	Normal: 12	20	12	195	92	71	BlastAnno: 1873	UniRef50_Q6Z6K4	729	61.89	488	179	5	1	1464	147	627	0.0	615
ABCDEF_44470	UniRef50_Q6Z6K4	0.0	Normal: 287	387	252	1193	287	741	BlastAnno: 2917	UniRef50_Q6Z6K4	729	52.28	723	310	9	2707	551	38	729	0.0	752
ABCDEF_49396	UniRef50_Q6Z6K4	0.0	Normal: 0	452	3	48	33	6872	BlastAnno: 2312	UniRef50_Q6Z6K4	729	65.83	676	221	7	2312	288	62	728	0.0	893
ABCDEF_10631	UniRef50_Q6Z6K4	0.0	Normal: 3079	207	2624	1970	2833	969	BlastAnno: 3058	UniRef50_Q6Z6K4	729	60.74	703	267	4	2943	835	36	729	0.0	917
ABCDEF_48112	UniRef50_Q6Z6K4	0.0	Normal: 60	2628	33	127	663	5106	BlastAnno: 2977	UniRef50_Q6Z6K4	729	67.21	494	157	3	2584	1103	33	521	0.0	696
3.2.1.45(glucosylceramidase;)																					
ABCDEF_44031	UniRef50_Q8LPR0	0.0	Normal: 88	125	131	209	725	66	BlastAnno: 1470	UniRef50_Q8LPR0	922	62.05	448	154	3	138	1463	448	885	0.0	544
ABCDEF_44034	UniRef50_B9FYQ4	0.0	Normal: 135	179	169	301	1067	84	BlastAnno: 2868	UniRef50_B9FYQ4	919	55.40	879	352	11	2780	168	70	916	0.0	903
ABCDEF_26879	UniRef50_Q8LPR0	0.0	Normal: 261	199	270	652	497	368	BlastAnno: 1657	UniRef50_Q8LPR0	922	57.17	523	192	6	1596	46	424	920	0.0	554
ABCDEF_74992	UniRef50_Q9LRN1	0.0	Normal: 163	140	183	229	464	205	BlastAnno: 1980	UniRef50_Q9LRN1	937	78.54	424	89	1	1483	212	516	937	0.0	650
ABCDEF_9833	UniRef50_Q9LRN1	0.0	Normal: 495	475	508	789	1098	494	BlastAnno: 3769	UniRef50_Q9LRN1	937	70.71	990	228	7	3251	309	1	937	0.0	1369
ABCDEF_26878	UniRef50_B9FYQ4	0.0	Normal: 376	308	408	1066	703	505	BlastAnno: 3016	UniRef50_B9FYQ4	919	55.35	925	370	12	2821	77	19	910	0.0	960
ABCDEF_44030	UniRef50_B9FYQ4	0.0	Normal: 166	221	229	394	1305	124	BlastAnno: 3109	UniRef50_B9FYQ4	919	54.42	928	383	11	183	2942	21	916	0.0	947
3.2.1.46(galactosylceramidase;)																					
No Correspond Unigene																					
3.5.1.-(Hydrolases;)																					
ABCDEF_41579	UniRef50_O81641	0.0	Normal: 384	122	265	320	313	150	BlastAnno: 1456	UniRef50_O81641	428	65.16	399	138	1	1313	120	23	421	0.0	543
ABCDEF_16924	UniRef50_Q8VYX0	0.0	Normal: 135	349	460	1325	685	527	BlastAnno: 2002	UniRef50_Q8VYX0	464	71.72	396	109	2	1612	428	68	461	0.0	549
ABCDEF_24290	UniRef50_O04373	0.0	Normal: 296	238	403	498	698	330	BlastAnno: 1710	UniRef50_O04373	440	65.24	443	151	3	67	1395	1	440	0.0	595

3.5.1.23(ceramidase;)																					
ABCDEF_74776	UniRef50_Q304B9	0.0	Normal: 617	1392	660	1092	1259	1102	BlastAnno: 3249	UniRef50_Q304B9	757	74.36	741	179	4	2585	369	26	757	0.0	1100
ABCDEF_44976	UniRef50_Q304B9	0.0	Normal: 575	1324	620	1032	1190	1064	BlastAnno: 3102	UniRef50_Q304B9	757	74.36	741	179	4	2585	369	26	757	0.0	1100
ABCDEF_44977	UniRef50_Q304B9	0.0	Normal: 653	1593	679	1168	1323	1174	BlastAnno: 3268	UniRef50_Q304B9	757	74.36	741	179	4	2631	415	26	757	0.0	1100
4.1.2.27(sphinganine-1-phosphate aldolase;)																					
ABCDEF_52129	UniRef50_Q9C509	5e-167	Normal: 56	51	46	145	361	403	BlastAnno: 975	UniRef50_Q9C509	544	79.42	277	57	0	1	831	265	541	5e-167	485
>00601 Glycosphingolipid biosynthesis - lacto and neolacto series																					
2.4.1.-(Transferases;)																					
ABCDEF_18004	UniRef50_Q6YWK8	0.0	Normal: 1562	1736	2009	3452	4438	2400	BlastAnno: 2298	UniRef50_Q6YWK8	570	65.27	429	136	1	1941	694	57	485	0.0	608
ABCDEF_52379	UniRef50_Q9LHZ7	0.0	Normal: 4015	4921	4546	10520	13430	4731	BlastAnno: 3479	UniRef50_Q9LHZ7	1170	48.04	893	342	16	825	3242	299	1156	0.0	787
ABCDEF_48593	UniRef50_Q9SK82	0.0	Normal: 12	420	19	343	763	253	BlastAnno: 1834	UniRef50_Q9SK82	489	60.08	481	186	4	1714	278	10	486	0.0	633
ABCDEF_13375	UniRef50_Q8VZK9	0.0	Normal: 108	123	208	526	34	294	BlastAnno: 1738	UniRef50_Q8VZK9	729	53.62	552	234	8	94	1737	18	551	0.0	582
ABCDEF_40982	UniRef50_Q96301	0.0	Normal: 1037	706	819	1249	1084	920	BlastAnno: 3418	UniRef50_Q96301	914	79.60	902	173	3	243	2948	1	891	0.0	1506
ABCDEF_47674	UniRef50_Q9LHZ7	0.0	Normal: 274	92	202	599	505	280	BlastAnno: 2526	UniRef50_Q9LHZ7	1170	75.93	777	151	8	2260	2	15	779	0.0	1177
ABCDEF_34097	UniRef50_Q9LE59	0.0	Normal: 626	613	923	1797	1829	692	BlastAnno: 2521	UniRef50_Q9LE59	673	60.92	458	178	1	1687	317	215	672	0.0	630
ABCDEF_34098	UniRef50_Q9LE59	0.0	Normal: 457	464	697	1283	1312	539	BlastAnno: 2199	UniRef50_Q9LE59	673	60.92	458	178	1	1687	317	215	672	0.0	628
ABCDEF_10848	UniRef50_Q9M156	0.0	Normal: 157	2988	163	408	772	1566	BlastAnno: 1810	UniRef50_Q9M156	480	60.09	456	176	4	1700	336	4	454	0.0	553
ABCDEF_41182	UniRef50_Q9LHZ7	0.0	Normal: 150	2169	221	814	1407	495	BlastAnno: 3604	UniRef50_Q9LHZ7	1170	51.03	870	333	15	2822	456	299	1156	0.0	826
ABCDEF_40981	UniRef50_Q96301	0.0	Normal: 156	97	110	160	179	173	BlastAnno: 1225	UniRef50_Q96301	914	86.92	344	45	0	1112	81	394	737	0.0	652
ABCDEF_9506	UniRef50_Q9LHZ7	0.0	Normal: 1302	2128	1494	3356	4644	2206	BlastAnno: 3751	UniRef50_Q9LHZ7	1170	49.38	891	343	17	927	3359	299	1161	0.0	812
ABCDEF_9500	UniRef50_Q9LHZ7	0.0	Normal: 183	188	186	576	498	260	BlastAnno: 1717	UniRef50_Q9LHZ7	1170	47.98	644	234	14	3	1715	418	1033	0.0	571
ABCDEF_100	UniRef50_Q9M8Y0	0.0	Normal: 228	309	224	416	461	141	BlastAnno: 2109	UniRef50_Q9M8Y0	977	77.49	533	120	0	1610	12	50	582	0.0	884
ABCDEF_33659	UniRef50_Q9LHZ7	0.0	Normal: 1282	1948	2248	5826	10642	2083	BlastAnno: 4289	UniRef50_Q9LHZ7	1170	48.70	887	345	15	2955	538	299	1156	0.0	788
ABCDEF_37667	UniRef50_Q9LHZ7	0.0	Normal: 248	3617	251	1237	2078	970	BlastAnno: 3161	UniRef50_Q9LHZ7	1170	49.72	885	335	14	2671	260	301	1156	0.0	810
ABCDEF_47673	UniRef50_Q9LHZ7	0.0	Normal: 876	296	828	1628	1721	1172	BlastAnno: 3878	UniRef50_Q9LHZ7	1170	80.88	1161	186	8	3642	232	15	1163	0.0	1865
ABCDEF_99	UniRef50_Q9M8Y0	0.0	Normal: 272	330	222	398	423	145	BlastAnno: 2094	UniRef50_Q9M8Y0	977	77.36	561	127	0	1683	1	50	610	0.0	929
ABCDEF_20658	UniRef50_Q8VZK9	0.0	Normal: 373	33	193	64	503	141	BlastAnno: 2316	UniRef50_Q8VZK9	729	53.35	508	221	7	47	1564	11	504	0.0	559
ABCDEF_10183	UniRef50_A7XDQ9	0.0	Normal: 422	455	346	577	378	610	BlastAnno: 2362	UniRef50_A7XDQ9	684	50.94	693	307	12	281	2287	1	684	0.0	670
ABCDEF_30745	UniRef50_A7XDQ9	0.0	Normal: 1209	351	1031	1645	1226	635	BlastAnno: 2646	UniRef50_A7XDQ9	684	57.58	554	227	6	546	2189	133	684	0.0	664
ABCDEF_13336	UniRef50_Q7PC69	0.0	Normal: 148	563	194	957	1575	816	BlastAnno: 2584	UniRef50_Q7PC69	745	71.56	559	136	6	744	2351	187	745	0.0	730
ABCDEF_21426	UniRef50_Q8RU27	0.0	Normal: 2658	1979	3417	6157	9464	2599	BlastAnno: 1530	UniRef50_Q8RU27	366	89.44	360	34	2	1358	282	1	357	0.0	667
ABCDEF_71242	UniRef50_Q9ZV71	0.0	Normal: 134	122	179	480	754	119	BlastAnno: 1835	UniRef50_Q9ZV71	409	73.35	409	103	4	231	1439	1	409	0.0	587
ABCDEF_52020	UniRef50_Q6YWK8	0.0	Normal: 1635	1534	1975	4284	10353	2289	BlastAnno: 2394	UniRef50_Q6YWK8	570	58.37	514	185	2	403	1857	57	570	0.0	607
ABCDEF_51269	UniRef50_Q6YWK8	0.0	Normal: 147	85	103	21	33	438	BlastAnno: 1864	UniRef50_Q6YWK8	570	54.28	514	204	2	1646	198	57	570	0.0	586
ABCDEF_30753	UniRef50_Q9ZV71	0.0	Normal: 290	497	417	629	1013	634	BlastAnno: 1502	UniRef50_Q9ZV71	409	72.32	401	107	2	311	1501	1	401	0.0	590
ABCDEF_4233	UniRef50_Q9ZV71	0.0	Normal: 369	412	550	936	860	750	BlastAnno: 2004	UniRef50_Q9ZV71	409	72.13	409	110	2	370	1584	1	409	0.0	613
ABCDEF_17525	UniRef50_Q8RU27	0.0	Normal: 1500	1289	2149	3711	4446	1069	BlastAnno: 1507	UniRef50_Q8RU27	366	91.35	347	30	0	1371	331	3	349	0.0	671
ABCDEF_18005	UniRef50_Q6YWK8	0.0	Normal: 1842	2042	2515	4214	5208	2842	BlastAnno: 2305	UniRef50_Q6YWK8	570	59.14	514	180	2	1948	497	57	570	0.0	642
ABCDEF_41140	UniRef50_Q6YWK8	0.0	Normal: 86	1314	93	753	1787	2076	BlastAnno: 2346	UniRef50_Q6YWK8	570	60.00	510	174	2	431	1870	57	566	0.0	669
ABCDEF_18002	UniRef50_Q9LHZ7	0.0	Normal: 376	127	367	601	1190	520	BlastAnno: 1605	UniRef50_Q9LHZ7	1170	89.75	400	41	0	1603	404	771	1170	0.0	732
ABCDEF_13339	UniRef50_Q7PC69	0.0	Normal: 127	814	183	541	1135	1144	BlastAnno: 2610	UniRef50_Q7PC69	745	70.48	559	141	6	725	2329	187	745	0.0	729
ABCDEF_41141	UniRef50_Q6YWK8	0.0	Normal: 67	1107	84	708	1690	1854	BlastAnno: 2461	UniRef50_Q6YWK8	570	60.00	510	174	2	1916	477	57	566	0.0	668
ABCDEF_44583	UniRef50_Q9LE59	0.0	Normal: 681	561	559	947	1745	789	BlastAnno: 2470	UniRef50_Q9LE59	673	59.21	456	185	1	1749	385	214	669	0.0	621
ABCDEF_3869	UniRef50_Q9LE59	0.0	Normal: 333	393	415	809	907	754	BlastAnno: 2136	UniRef50_Q9LE59	673	59.05	613	227	4	1827	1	81	673	0.0	699
ABCDEF_28947	UniRef50_Q9SK82	0.0	Normal: 0	851	3	0	0	116	BlastAnno: 1567	UniRef50_Q9SK82	489	62.40	484	176	4	1524	85	1	482	0.0	652
ABCDEF_31119	UniRef50_Q9M8Y0	0.0	Normal: 2212	1279	1917	2933	3341	1803	BlastAnno: 3695	UniRef50_Q9M8Y0	977	81.08	925	174	1	392	3163	50	974	0.0	1583
ABCDEF_37311	UniRef50_Q9LE59	0.0	Normal: 175	133	191	549	421	366	BlastAnno: 1612	UniRef50_Q9LE59	673	62.96	486	153	4	1406	3	4	480	0.0	542
ABCDEF_24190	UniRef50_Q8L7F9	0.0	Normal: 397	265	492	583	553	371	BlastAnno: 2564	UniRef50_Q8L7F9	643	63.91	640	218	4	2353	473	1	640	0.0	850
ABCDEF_30548	UniRef50_Q9FH36	0.0	Normal: 17	916	27	151	420	126	BlastAnno: 1963	UniRef50_Q9FH36	535	72.47	534	138	3	102	1676	1	534	0.0	767
ABCDEF_27416	UniRef50_Q7PC69	0.0	Normal: 575	837	875	1344	2015	936	BlastAnno: 2454	UniRef50_Q7PC69	745	66.11	416	115	4	2337	1168	126	541	0.0	518
ABCDEF_48373	UniRef50_A7XDQ9	0.0	Normal: 1364	773	1336	1451	1791	1202	BlastAnno: 3042	UniRef50_A7XDQ9	684	63.68	691	234	6	2405	360	1	683	0.0	915
ABCDEF_3868	UniRef50_Q9LE59	0.0	Normal: 224	274	301	555	686	524	BlastAnno: 1670	UniRef50_Q9LE59	673	65.31	516	176	1	1556	9	161	673	0.0	696
ABCDEF_31288	UniRef50_Q940V3	0.0	Normal: 247	309	183	101	124	655	BlastAnno: 1658	UniRef50_Q940V3	470	56.01	466	197	7	1614	232	8	470	0.0	537
ABCDEF_52574	UniRef50_Q09332	0.0	Normal: 885	474	1068	1835	1853	816	BlastAnno: 3720	UniRef50_Q09332	1548	47.89	687	310	14	2551	533	859	1511	0.0	598
ABCDEF_79752	UniRef50_Q9ZV71	0.0	Normal: 158	183	245	400	379	352	BlastAnno: 1266	UniRef50_Q9ZV71	409	71.39	360	99	2	31	1098	1	360	0.0	526
ABCDEF_9509	UniRef50_Q9LHZ7	0.0	Normal: 1296	2171	1498	3410	4683	2232	BlastAnno: 3897	UniRef50_Q9LHZ7	1170	49.38	891	343	17	1073	3505	299	1161	0.0	812
ABCDEF_48200	UniRef50_Q6YWK8	0.0	Normal: 1768	576	3156	4431	6634	491	BlastAnno: 2274	UniRef50_Q6YWK8	570	57.98	514	185	4	291	1739	57	570	0.0	642
ABCDEF_24155	UniRef50_Q9ZVI7	0.0	Normal: 503	247	607	1676	1833	578	BlastAnno: 2708	UniRef50_Q9ZVI7	619	52.33	621	264	11	306	2123	15	618	0.0	601
ABCDEF_21246	UniRef50_Q9SKT6	0.0	Normal: 92	107	188	380	834	84	BlastAnno: 1791	UniRef50_Q9SKT6	536	56.61	484	204	5	123	1562	52	533	0.0	568
ABCDEF_32317	UniRef50_Q8RU27	0.0	Normal: 12	9	3	118	800	62	BlastAnno: 1076	UniRef50_Q8RU27	366	87.02	285	37	0	1075	221	64	348	0.0	535
ABCDEF_9502	UniRef50_Q9LHZ7	0.0	Normal: 610	486	568	1331	1403	591	BlastAnno: 1961	UniRef50_Q9LHZ7	1170	48.64	625	249	13	3	1742	564	1161	0.0	570
ABCDEF_23298	UniRef50_Q9FH36	0.0	Normal: 128	527	130	223	314	864	BlastAnno: 2832	UniRef50_Q9FH36	535	83.16	196	33	0	696	1283	340	535	0.0	357
ABCDEF_40595	UniRef50_O80898	0.0	Normal: 229	135	103	25	307	325	BlastAnno: 2413	UniRef50_O80898	757	44.68	761	378	13	95	2299	10	753	0.0	587
ABCDEF_63947	UniRef50_Q9SK82	0.0	Normal: 0	267	0	0	71	0	BlastAnno: 1446	UniRef50_Q9SK82	489	58.33	468	189	4	60	1445	1	468	0.0	592
ABCDEF_67847	UniRef50_Q9M8Y0	0.0	Normal: 794	476	871	1081	1401	868	BlastAnno: 1591	UniRef50_Q9M8Y0	977	84.86	370	56	0	1589	480	605	974	0.0	685
ABCDEF_65666	UniRef50_Q09332	0.0	Normal: 800	441	946	1606	1637	735	BlastAnno: 3518	UniRef50_Q09332	1548	47.54	671	305	13	1162	3087	859	1511	0.0	586
ABCDEF_38641	UniRef50_Q9FWA4	0.0	Normal: 674	967	1162	2603	4404	2475	BlastAnno: 1599	UniRef50_Q9FWA4	561	81.35	386	72	0	1	1158	176	561	0.0	641
ABCDEF_17914	UniRef50_Q9SKT6	0.0	Normal: 784	567	886	1447	1439	1424	BlastAnno: 2049	UniRef50_Q9SKT6	536	72.95	536	140	2	204	1796	1	536	0.0	785
ABCDEF_18286	UniRef50_Q6NQB7	0.0	Normal: 1131	1108	1225	1872	1722	3329	BlastAnno: 1752	UniRef50_Q6NQB7	393	73.80	397	81	6	1527	367	10	393	0.0	607
ABCDEF_119	UniRef50_Q8RU27	0.0	Normal: 1613	1155	2249	3558	7663	1775	BlastAnno: 1532	UniRef50_Q8RU27	366	88.33	360	38	2	1306	230	1	357	0.0	663
ABCDEF_47603	UniRef50_Q9M9Y5	0.0	Normal: 2609	8779	1664	1103	2085	22991	BlastAnno: 2830	UniRef50_Q9M9Y5	589	54.04	594	267	3	2475	694	1	588	0.0	654
ABCDEF_24192	UniRef50_Q8L7F9	0.0	Normal: 391	244	465	553	497	345	BlastAnno: 2477	UniRef50_Q8L7F9	643	58.99	434	165	4	212	1474	1	434	0.0	513

ABCDEF_17482	UniRef50_Q9SKT6	0.0	Normal: 615	379	816	1068	1470	852	BlastAnno: 2083	UniRef50_Q9SKT6	536	71.11	540	147	4	1915	311	1	536	0.0	786
ABCDEF_85909	UniRef50_Q8VZK9	0.0	Normal: 28	45	9	10	44	127	BlastAnno: 1473	UniRef50_Q8VZK9	729	55.37	475	198	6	1419	1	11	473	0.0	536
ABCDEF_9503	UniRef50_Q9LHZ7	0.0	Normal: 487	544	524	1242	1320	833	BlastAnno: 2087	UniRef50_Q9LHZ7	1170	50.35	574	236	8	57	1712	615	1161	0.0	567
ABCDEF_40594	UniRef50_O80898	0.0	Normal: 256	127	102	41	331	326	BlastAnno: 2417	UniRef50_O80898	757	45.76	719	348	12	213	2291	51	753	0.0	585
ABCDEF_17141	UniRef50_Q9LHZ7	0.0	Normal: 74	1734	69	380	701	333	BlastAnno: 3337	UniRef50_Q9LHZ7	1170	50.92	868	335	14	2539	179	299	1156	0.0	816
ABCDEF_33658	UniRef50_Q9LHZ7	0.0	Normal: 384	308	395	805	1003	602	BlastAnno: 2961	UniRef50_Q9LHZ7	1170	48.76	888	343	14	204	2621	299	1156	0.0	792
ABCDEF_20579	UniRef50_Q7PC69	0.0	Normal: 1007	795	1238	2311	2309	1345	BlastAnno: 3113	UniRef50_Q7PC69	745	59.97	767	229	14	2667	535	1	745	0.0	768
ABCDEF_31118	UniRef50_Q9M8Y0	0.0	Normal: 2135	1267	1900	3004	3329	1757	BlastAnno: 3787	UniRef50_Q9M8Y0	977	81.10	709	133	1	1132	3255	266	974	0.0	1217
ABCDEF_9499	UniRef50_Q9LHZ7	0.0	Normal: 205	207	215	632	538	275	BlastAnno: 1794	UniRef50_Q9LHZ7	1170	48.88	667	240	14	11	1792	395	1033	0.0	605
ABCDEF_48199	UniRef50_Q6YWK8	0.0	Normal: 1506	467	2429	3094	4233	439	BlastAnno: 1997	UniRef50_Q6YWK8	570	63.10	439	146	2	291	1559	57	495	0.0	610
ABCDEF_40983	UniRef50_Q96301	0.0	Normal: 406	261	343	392	415	433	BlastAnno: 2265	UniRef50_Q96301	914	79.17	504	99	1	114	1625	394	891	0.0	854
ABCDEF_17140	UniRef50_Q9LHZ7	0.0	Normal: 45	1049	49	216	386	208	BlastAnno: 2293	UniRef50_Q9LHZ7	1170	52.34	770	281	13	2138	57	397	1156	0.0	739
ABCDEF_18352	UniRef50_Q9FWA4	0.0	Normal: 2427	2688	2969	5519	6547	4855	BlastAnno: 2224	UniRef50_Q9FWA4	561	69.31	505	148	3	1887	382	61	561	0.0	683
ABCDEF_27767	UniRef50_Q9LHZ7	0.0	Normal: 2576	5141	2841	10249	14583	5031	BlastAnno: 3981	UniRef50_Q9LHZ7	1170	49.66	888	337	16	2927	504	299	1156	0.0	818
ABCDEF_33661	UniRef50_Q9LHZ7	0.0	Normal: 414	340	507	879	790	783	BlastAnno: 2185	UniRef50_Q9LHZ7	1170	48.66	748	277	14	2184	169	440	1156	0.0	662
ABCDEF_14685	UniRef50_A7XDQ9	0.0	Normal: 233	308	134	573	292	334	BlastAnno: 1947	UniRef50_A7XDQ9	684	56.84	468	186	6	569	1945	1	461	0.0	545
ABCDEF_20660	UniRef50_Q8VZK9	0.0	Normal: 22	48	13	15	52	144	BlastAnno: 1473	UniRef50_Q8VZK9	729	55.16	475	199	6	55	1473	11	473	0.0	537
ABCDEF_21315	UniRef50_Q9LHZ7	0.0	Normal: 3412	252	1450	0	79	1228	BlastAnno: 3765	UniRef50_Q9LHZ7	1170	63.78	1096	340	15	308	3508	70	1137	0.0	1333
ABCDEF_533	UniRef50_Q9C8W3	0.0	Normal: 230	204	269	505	411	301	BlastAnno: 1688	UniRef50_Q9C8W3	401	70.33	364	94	2	393	1475	47	399	0.0	555
ABCDEF_34099	UniRef50_Q9LE59	0.0	Normal: 478	491	735	1356	1350	548	BlastAnno: 2158	UniRef50_Q9LE59	673	60.92	458	178	1	1610	240	215	672	0.0	630
ABCDEF_47984	UniRef50_Q7PC69	0.0	Normal: 663	457	734	1362	2147	696	BlastAnno: 2838	UniRef50_Q7PC69	745	59.27	712	226	9	373	2316	34	745	0.0	702
ABCDEF_51451	UniRef50_Q9LE59	0.0	Normal: 1883	1782	2202	4337	4350	2667	BlastAnno: 2874	UniRef50_Q9LE59	673	74.16	685	165	6	2559	505	1	673	0.0	982
ABCDEF_13370	UniRef50_Q8VZK9	0.0	Normal: 290	87	317	377	105	258	BlastAnno: 2033	UniRef50_Q8VZK9	729	51.65	635	278	11	2031	154	112	726	0.0	612
ABCDEF_48239	UniRef50_Q9LHZ7	0.0	Normal: 511	163	422	1154	1316	510	BlastAnno: 2714	UniRef50_Q9LHZ7	1170	76.20	769	156	9	478	2712	41	806	0.0	1201
ABCDEF_13338	UniRef50_Q7PC69	0.0	Normal: 335	468	417	2639	4443	616	BlastAnno: 2525	UniRef50_Q7PC69	745	62.45	727	209	12	298	2292	21	745	0.0	781
ABCDEF_24193	UniRef50_Q8L7F9	0.0	Normal: 113	72	132	203	180	119	BlastAnno: 1679	UniRef50_Q8L7F9	643	60.54	479	176	4	212	1609	1	479	0.0	592
ABCDEF_48514	UniRef50_Q9ZV71	0.0	Normal: 540	580	601	771	1033	926	BlastAnno: 1920	UniRef50_Q9ZV71	409	78.14	398	85	2	1618	431	12	409	0.0	637

2.4.1.149(N-acetyllactosaminide beta-1,3-N-acetylglucosaminyltransferase;)

No Correspond Unigene

2.4.1.150(N-acetyllactosaminide beta-1,6-N-acetylglucosaminyl-transferase;)

No Correspond Unigene

2.4.1.152(4-galactosyl-N-acetylglucosaminide 3-alpha-L-fucosyltransferase;)

No Correspond Unigene

2.4.1.163(beta-galactosyl-N-acetylglucosaminylgalactosylglucosyl-ceramide beta-1,3-acetylglucosaminyltransferase;)

No Correspond Unigene

2.4.1.206(lactosylceramide 1,3-N-acetyl-beta-D-glucosaminyltransferase;)

No Correspond Unigene

2.4.1.37(fucosylgalactoside 3-alpha-galactosyltransferase;)

ABCDEF_4123	UniRef50_Q54RP0	2e-14	Normal: 420	239	595	921	1421	395	BlastAnno: 1712	UniRef50_Q54RP0	648	26.44	208	133	4	651	1241	94	292	2e-14	82.4

2.4.1.40(glycoprotein-fucosylgalactoside alpha-N-acetylgalactosaminyltransferase;)

No Correspond Unigene

2.4.1.65(3-galactosyl-N-acetylglucosaminide 4-alpha-L-fucosyltransferase;)

No Correspond Unigene

2.4.1.69(galactoside 2-alpha-L-fucosyltransferase;)

ABCDEF_87540	UniRef50_B9RLW2	9e-56	Normal: 0	0	0	0	0	36	BlastAnno: 523	UniRef50_B9RLW2	551	53.76	173	78	1	522	4	296	466	9e-56	192

2.4.1.86(glucosaminylgalactosylglucosylceramide beta-galactosyltransferase;)

No Correspond Unigene

2.4.1.87(N-acetyllactosaminide 3-alpha-galactosyltransferase;)

No Correspond Unigene

2.4.99.-(Transferases;)

ABCDEF_40388	UniRef50_Q92187	2e-11	Normal: 282	215	308	417	512	350	BlastAnno: 2774	UniRef50_Q92187	359	57.53	73	29	2	2023	1808	116	187	2e-11	72.4
ABCDEF_40387	UniRef50_Q92187	2e-11	Normal: 587	417	654	1107	1268	855	BlastAnno: 2090	UniRef50_Q92187	359	57.53	73	29	2	1328	1113	116	187	2e-11	72.4

2.4.99.10(neolactotetraosylceramide alpha-2,3-sialyltransferase;)

No Correspond Unigene

2.4.99.4(beta-galactoside alpha-2,3-sialyltransferase;)

ABCDEF_28297	UniRef50_B9SCR7	4e-167	Normal: 573	742	586	923	1062	1024	BlastAnno: 1855	UniRef50_B9SCR7	434	60.36	391	149	4	1454	294	46	434	4e-167	493

2.4.99.6(N-acetyllactosaminide alpha-2,3-sialyltransferase;)

No Correspond Unigene

2.4.99.8(alpha-N-acetylneuraminate alpha-2,8-sialyltransferase;)

No Correspond Unigene

>00603 Glycosphingolipid biosynthesis - globo series

2.4.1.-(Transferases;)

ABCDEF_18004	UniRef50_Q6YWK8	0.0	Normal: 1562	1736	2009	3452	4438	2400	BlastAnno: 2298	UniRef50_Q6YWK8	570	65.27	429	136	1	1941	694	57	485	0.0	608
ABCDEF_52379	UniRef50_Q9LHZ7	0.0	Normal: 4015	4921	4546	10520	13430	4731	BlastAnno: 3479	UniRef50_Q9LHZ7	1170	48.04	893	342	16	825	3242	299	1156	0.0	787
ABCDEF_48593	UniRef50_Q9SK82	0.0	Normal: 12	420	19	343	763	253	BlastAnno: 1834	UniRef50_Q9SK82	489	60.08	481	186	4	1714	278	10	486	0.0	633
ABCDEF_13375	UniRef50_Q8VZK9	0.0	Normal: 108	123	208	526	34	294	BlastAnno: 1738	UniRef50_Q8VZK9	729	53.62	552	234	8	94	1737	18	551	0.0	582
ABCDEF_40982	UniRef50_Q96301	0.0	Normal: 1037	706	819	1249	1084	920	BlastAnno: 3418	UniRef50_Q96301	914	79.60	902	173	3	243	2948	1	891	0.0	1506
ABCDEF_47674	UniRef50_Q9LHZ7	0.0	Normal: 274	92	202	599	505	280	BlastAnno: 2526	UniRef50_Q9LHZ7	1170	75.93	777	151	8	2260	2	15	779	0.0	1177
ABCDEF_34097	UniRef50_Q9LE59	0.0	Normal: 626	613	923	1797	1829	692	BlastAnno: 2521	UniRef50_Q9LE59	673	60.92	458	178	1	1687	317	215	672	0.0	630
ABCDEF_34098	UniRef50_Q9LE59	0.0	Normal: 457	464	697	1283	1312	539	BlastAnno: 2199	UniRef50_Q9LE59	673	60.92	458	178	1	1687	317	215	672	0.0	628
ABCDEF_10848	UniRef50_Q9M156	0.0	Normal: 157	2988	163	408	772	1566	BlastAnno: 1810	UniRef50_Q9M156	480	60.09	456	176	4	1700	336	4	454	0.0	553
ABCDEF_41182	UniRef50_Q9LHZ7	0.0	Normal: 150	2169	221	814	1407	495	BlastAnno: 3604	UniRef50_Q9LHZ7	1170	51.03	870	333	15	2822	456	299	1156	0.0	826

ABCDEF_40981	UniRef50_Q96301	0.0	Normal: 156	97	110	160	179	173	BlastAnno: 1225	UniRef50_Q96301	914	86.92	344	45	0	1112	81	394	737	0.0	652
ABCDEF_9506	UniRef50_Q9LHZ7	0.0	Normal: 1302	2128	1494	3356	4644	2206	BlastAnno: 3751	UniRef50_Q9LHZ7	1170	49.38	891	343	17	927	3359	299	1161	0.0	812
ABCDEF_9500	UniRef50_Q9LHZ7	0.0	Normal: 183	188	186	576	498	260	BlastAnno: 1717	UniRef50_Q9LHZ7	1170	47.98	644	234	14	3	1715	418	1033	0.0	571
ABCDEF_100	UniRef50_Q9M8Y0	0.0	Normal: 228	309	224	416	461	141	BlastAnno: 2109	UniRef50_Q9M8Y0	977	77.49	533	120	0	1610	12	50	582	0.0	884
ABCDEF_33659	UniRef50_Q9LHZ7	0.0	Normal: 1282	1948	2248	5826	10642	2083	BlastAnno: 4289	UniRef50_Q9LHZ7	1170	48.70	887	345	15	2955	538	299	1156	0.0	788
ABCDEF_37667	UniRef50_Q9LHZ7	0.0	Normal: 248	3617	251	1237	2078	970	BlastAnno: 3161	UniRef50_Q9LHZ7	1170	49.72	885	335	14	2671	260	301	1156	0.0	810
ABCDEF_47673	UniRef50_Q9LHZ7	0.0	Normal: 876	296	828	1628	1721	1172	BlastAnno: 3878	UniRef50_Q9LHZ7	1170	80.88	1161	186	8	3642	232	15	1163	0.0	1865
ABCDEF_99	UniRef50_Q9M8Y0	0.0	Normal: 272	330	222	398	423	145	BlastAnno: 2094	UniRef50_Q9M8Y0	977	77.36	561	127	0	1683	1	50	610	0.0	929
ABCDEF_20658	UniRef50_Q8VZK9	0.0	Normal: 373	33	193	64	503	141	BlastAnno: 2316	UniRef50_Q8VZK9	729	53.35	508	221	7	47	1564	11	504	0.0	559
ABCDEF_10183	UniRef50_A7XDQ9	0.0	Normal: 422	455	346	577	378	610	BlastAnno: 2362	UniRef50_A7XDQ9	684	50.94	693	307	12	281	2287	1	684	0.0	670
ABCDEF_30745	UniRef50_A7XDQ9	0.0	Normal: 1209	351	1031	1645	1226	635	BlastAnno: 2646	UniRef50_A7XDQ9	684	57.58	554	227	6	546	2189	133	684	0.0	664
ABCDEF_13336	UniRef50_Q7PC69	0.0	Normal: 148	563	194	957	1575	816	BlastAnno: 2584	UniRef50_Q7PC69	745	71.56	559	136	6	744	2351	187	745	0.0	730
ABCDEF_21426	UniRef50_Q8RU27	0.0	Normal: 2658	1979	3417	6157	9464	2599	BlastAnno: 1530	UniRef50_Q8RU27	366	89.44	360	34	2	1358	282	1	357	0.0	667
ABCDEF_71242	UniRef50_Q9ZV71	0.0	Normal: 134	122	179	480	754	119	BlastAnno: 1835	UniRef50_Q9ZV71	409	73.35	409	103	4	231	1439	1	409	0.0	587
ABCDEF_52020	UniRef50_Q6YWK8	0.0	Normal: 1635	1534	1975	4284	10353	2289	BlastAnno: 2394	UniRef50_Q6YWK8	570	58.37	514	185	2	403	1857	57	570	0.0	607
ABCDEF_51269	UniRef50_Q6YWK8	0.0	Normal: 147	85	103	21	33	438	BlastAnno: 1864	UniRef50_Q6YWK8	570	54.28	514	204	2	1646	198	57	570	0.0	586
ABCDEF_30753	UniRef50_Q9ZV71	0.0	Normal: 290	497	417	629	1013	634	BlastAnno: 1502	UniRef50_Q9ZV71	409	72.32	401	107	2	311	1501	1	401	0.0	590
ABCDEF_4233	UniRef50_Q9ZV71	0.0	Normal: 369	412	550	936	860	750	BlastAnno: 2004	UniRef50_Q9ZV71	409	72.13	409	110	2	370	1584	1	409	0.0	613
ABCDEF_17525	UniRef50_Q8RU27	0.0	Normal: 1500	1289	2149	3711	4446	1069	BlastAnno: 1507	UniRef50_Q8RU27	366	91.35	347	30	0	1371	331	3	349	0.0	671
ABCDEF_18005	UniRef50_Q6YWK8	0.0	Normal: 1842	2042	2515	4214	5208	2842	BlastAnno: 2305	UniRef50_Q6YWK8	570	59.14	514	180	2	1948	497	57	570	0.0	642
ABCDEF_41140	UniRef50_Q6YWK8	0.0	Normal: 86	1314	93	753	1787	2076	BlastAnno: 2346	UniRef50_Q6YWK8	570	60.00	510	174	2	431	1870	57	566	0.0	669
ABCDEF_18002	UniRef50_Q9LHZ7	0.0	Normal: 376	127	367	601	1190	520	BlastAnno: 1605	UniRef50_Q9LHZ7	1170	89.75	400	41	0	1603	404	771	1170	0.0	732
ABCDEF_13339	UniRef50_Q7PC69	0.0	Normal: 127	814	183	541	1135	1144	BlastAnno: 2610	UniRef50_Q7PC69	745	70.48	559	141	6	725	2329	187	745	0.0	729
ABCDEF_41141	UniRef50_Q6YWK8	0.0	Normal: 67	1107	84	708	1690	1854	BlastAnno: 2461	UniRef50_Q6YWK8	570	60.00	510	174	2	1916	477	57	566	0.0	668
ABCDEF_44583	UniRef50_Q9LE59	0.0	Normal: 681	561	559	947	1745	789	BlastAnno: 2470	UniRef50_Q9LE59	673	59.21	456	185	1	1749	385	214	669	0.0	621
ABCDEF_3869	UniRef50_Q9LE59	0.0	Normal: 333	393	415	809	907	754	BlastAnno: 2136	UniRef50_Q9LE59	673	59.05	613	227	4	1827	1	81	673	0.0	699
ABCDEF_28947	UniRef50_Q9SK82	0.0	Normal: 0	851	3	0	0	116	BlastAnno: 1567	UniRef50_Q9SK82	489	62.40	484	176	4	1524	85	1	482	0.0	652
ABCDEF_31119	UniRef50_Q9M8Y0	0.0	Normal: 2212	1279	1917	2933	3341	1803	BlastAnno: 3695	UniRef50_Q9M8Y0	977	81.08	925	174	1	392	3163	50	974	0.0	1583
ABCDEF_37311	UniRef50_Q9LE59	0.0	Normal: 175	133	191	549	421	366	BlastAnno: 1612	UniRef50_Q9LE59	673	62.96	486	153	4	1406	3	4	480	0.0	542
ABCDEF_24190	UniRef50_Q8L7F9	0.0	Normal: 397	265	492	583	553	371	BlastAnno: 2564	UniRef50_Q8L7F9	643	63.91	640	218	4	2353	473	1	640	0.0	850
ABCDEF_30548	UniRef50_Q9FH36	0.0	Normal: 17	916	27	151	420	126	BlastAnno: 1963	UniRef50_Q9FH36	535	72.47	534	138	3	102	1676	1	534	0.0	767
ABCDEF_27416	UniRef50_Q7PC69	0.0	Normal: 575	837	875	1344	2015	936	BlastAnno: 2454	UniRef50_Q7PC69	745	66.11	416	115	4	2337	1168	126	541	0.0	518
ABCDEF_48373	UniRef50_A7XDQ9	0.0	Normal: 1364	773	1336	1451	1791	1202	BlastAnno: 3042	UniRef50_A7XDQ9	684	63.68	691	234	6	2405	360	1	683	0.0	915
ABCDEF_3868	UniRef50_Q9LE59	0.0	Normal: 224	274	301	555	686	524	BlastAnno: 1670	UniRef50_Q9LE59	673	65.31	516	176	1	1556	9	161	673	0.0	696
ABCDEF_31288	UniRef50_Q940V3	0.0	Normal: 247	309	183	101	124	655	BlastAnno: 1658	UniRef50_Q940V3	470	56.01	466	197	7	1614	232	8	470	0.0	537
ABCDEF_52574	UniRef50_Q09332	0.0	Normal: 885	474	1068	1835	1853	816	BlastAnno: 3720	UniRef50_Q09332	1548	47.89	687	310	14	2551	533	859	1511	0.0	598
ABCDEF_79752	UniRef50_Q9ZV71	0.0	Normal: 158	183	245	400	379	352	BlastAnno: 1266	UniRef50_Q9ZV71	409	71.39	360	99	2	31	1098	1	360	0.0	526
ABCDEF_9509	UniRef50_Q9LHZ7	0.0	Normal: 1296	2171	1498	3410	4683	2232	BlastAnno: 3897	UniRef50_Q9LHZ7	1170	49.38	891	343	17	1073	3505	299	1161	0.0	812
ABCDEF_48200	UniRef50_Q6YWK8	0.0	Normal: 1768	576	3156	4431	6634	491	BlastAnno: 2274	UniRef50_Q6YWK8	570	57.98	514	185	4	291	1739	57	570	0.0	642
ABCDEF_24155	UniRef50_Q9ZVI7	0.0	Normal: 503	247	607	1676	1833	578	BlastAnno: 2708	UniRef50_Q9ZVI7	619	52.33	621	264	11	306	2123	15	618	0.0	601
ABCDEF_21246	UniRef50_Q9SKT6	0.0	Normal: 92	107	188	380	834	84	BlastAnno: 1791	UniRef50_Q9SKT6	536	56.61	484	204	5	123	1562	52	533	0.0	568
ABCDEF_32317	UniRef50_Q8RU27	0.0	Normal: 12	9	3	118	800	62	BlastAnno: 1076	UniRef50_Q8RU27	366	87.02	285	37	0	1075	221	64	348	0.0	535
ABCDEF_9502	UniRef50_Q9LHZ7	0.0	Normal: 610	486	568	1331	1403	591	BlastAnno: 1961	UniRef50_Q9LHZ7	1170	48.64	625	249	13	3	1742	564	1161	0.0	570
ABCDEF_23298	UniRef50_Q9FH36	0.0	Normal: 128	527	130	223	314	864	BlastAnno: 2832	UniRef50_Q9FH36	535	83.16	196	33	0	696	1283	340	535	0.0	357
ABCDEF_40595	UniRef50_O80898	0.0	Normal: 229	135	103	25	307	325	BlastAnno: 2413	UniRef50_O80898	757	44.68	761	378	13	95	2299	10	753	0.0	587
ABCDEF_63947	UniRef50_Q9SK82	0.0	Normal: 0	267	0	0	71	0	BlastAnno: 1446	UniRef50_Q9SK82	489	58.33	468	189	4	60	1445	1	468	0.0	592
ABCDEF_67847	UniRef50_Q9M8Y0	0.0	Normal: 794	476	871	1081	1401	868	BlastAnno: 1591	UniRef50_Q9M8Y0	977	84.86	370	56	0	1589	480	605	974	0.0	685
ABCDEF_65666	UniRef50_Q09332	0.0	Normal: 800	441	946	1606	1637	735	BlastAnno: 3518	UniRef50_Q09332	1548	47.54	671	305	13	1162	3087	859	1511	0.0	586
ABCDEF_38641	UniRef50_Q9FWA4	0.0	Normal: 674	967	1162	2603	4404	2475	BlastAnno: 1599	UniRef50_Q9FWA4	561	81.35	386	72	0	1	1158	176	561	0.0	641
ABCDEF_17914	UniRef50_Q9SKT6	0.0	Normal: 784	567	886	1447	1439	1424	BlastAnno: 2049	UniRef50_Q9SKT6	536	72.95	536	140	2	204	1796	1	536	0.0	785
ABCDEF_18286	UniRef50_Q6NQB7	0.0	Normal: 1131	1108	1225	1872	1722	3329	BlastAnno: 1752	UniRef50_Q6NQB7	393	73.80	397	81	6	1527	367	10	393	0.0	607
ABCDEF_119	UniRef50_Q8RU27	0.0	Normal: 1613	1155	2249	3558	7663	1775	BlastAnno: 1532	UniRef50_Q8RU27	366	88.33	360	38	2	1306	230	1	357	0.0	663
ABCDEF_47603	UniRef50_Q9M9Y5	0.0	Normal: 2609	8779	1664	1103	2085	22991	BlastAnno: 2830	UniRef50_Q9M9Y5	589	54.04	594	267	3	2475	694	1	588	0.0	654
ABCDEF_24192	UniRef50_Q8L7F9	0.0	Normal: 391	244	465	553	497	345	BlastAnno: 2477	UniRef50_Q8L7F9	643	58.99	434	165	4	212	1474	1	434	0.0	513
ABCDEF_17482	UniRef50_Q9SKT6	0.0	Normal: 615	379	816	1068	1470	852	BlastAnno: 2083	UniRef50_Q9SKT6	536	71.11	540	147	4	1915	311	1	536	0.0	786
ABCDEF_85909	UniRef50_Q8VZK9	0.0	Normal: 28	45	9	10	44	127	BlastAnno: 1473	UniRef50_Q8VZK9	729	55.37	475	198	6	1419	1	11	473	0.0	536
ABCDEF_9503	UniRef50_Q9LHZ7	0.0	Normal: 487	544	524	1242	1320	833	BlastAnno: 2087	UniRef50_Q9LHZ7	1170	50.35	574	236	8	57	1712	615	1161	0.0	567
ABCDEF_40594	UniRef50_O80898	0.0	Normal: 256	127	102	41	331	326	BlastAnno: 2417	UniRef50_O80898	757	45.76	719	348	12	213	2291	51	753	0.0	585
ABCDEF_17141	UniRef50_Q9LHZ7	0.0	Normal: 74	1734	69	380	701	333	BlastAnno: 3337	UniRef50_Q9LHZ7	1170	50.92	868	335	14	2539	179	299	1156	0.0	816
ABCDEF_33658	UniRef50_Q9LHZ7	0.0	Normal: 384	308	395	805	1003	602	BlastAnno: 2961	UniRef50_Q9LHZ7	1170	48.76	888	343	14	204	2621	299	1156	0.0	792
ABCDEF_20579	UniRef50_Q7PC69	0.0	Normal: 1007	795	1238	2311	2309	1345	BlastAnno: 3113	UniRef50_Q7PC69	745	59.97	767	229	14	2667	535	1	745	0.0	768
ABCDEF_31118	UniRef50_Q9M8Y0	0.0	Normal: 2135	1267	1900	3004	3329	1757	BlastAnno: 3787	UniRef50_Q9M8Y0	977	81.10	709	133	1	1132	3255	266	974	0.0	1217
ABCDEF_9499	UniRef50_Q9LHZ7	0.0	Normal: 205	207	215	632	538	275	BlastAnno: 1794	UniRef50_Q9LHZ7	1170	48.88	667	240	14	11	1792	395	1033	0.0	605
ABCDEF_48199	UniRef50_Q6YWK8	0.0	Normal: 1506	467	2429	3094	4233	439	BlastAnno: 1997	UniRef50_Q6YWK8	570	63.10	439	146	2	291	1559	57	495	0.0	610
ABCDEF_40983	UniRef50_Q96301	0.0	Normal: 406	261	343	392	415	433	BlastAnno: 2265	UniRef50_Q96301	914	79.17	504	99	1	114	1625	394	891	0.0	854
ABCDEF_17140	UniRef50_Q9LHZ7	0.0	Normal: 45	1049	49	216	386	208	BlastAnno: 2293	UniRef50_Q9LHZ7	1170	52.34	770	281	13	2138	57	397	1156	0.0	739
ABCDEF_18352	UniRef50_Q9FWA4	0.0	Normal: 2427	2688	2969	5519	6547	4855	BlastAnno: 2224	UniRef50_Q9FWA4	561	69.31	505	148	3	1887	382	61	561	0.0	683
ABCDEF_27767	UniRef50_Q9LHZ7	0.0	Normal: 2576	5141	2841	10249	14583	5031	BlastAnno: 3981	UniRef50_Q9LHZ7	1170	49.66	883	337	16	2927	504	299	1156	0.0	818
ABCDEF_33661	UniRef50_Q9LHZ7	0.0	Normal: 414	340	507	879	790	783	BlastAnno: 2185	UniRef50_Q9LHZ7	1170	48.66	748	277	14	2184	169	440	1156	0.0	662
ABCDEF_14685	UniRef50_A7XDQ9	0.0	Normal: 233	308	134	573	292	334	BlastAnno: 1947	UniRef50_A7XDQ9	684	56.84	468	186	6	569	1945	1	461	0.0	545
ABCDEF_20660	UniRef50_Q8VZK9	0.0	Normal: 22	48	13	15	52	144	BlastAnno: 1473	UniRef50_Q8VZK9	729	55.16	475	199	6	55	1473	11	473	0.0	537
ABCDEF_21315	UniRef50_Q9LHZ7	0.0	Normal: 3412	252	1450	0	79	1228	BlastAnno: 3765	UniRef50_Q9LHZ7	1170	63.78	1096	340	15	308	3508	70	1137	0.0	1333

ABCDEF_533	UniRef50_Q9C8W3	0.0	Normal: 230	204	269	505	411	301	BlastAnno: 1688	UniRef50_Q9C8W3	401	70.33	364	94	2	393	1475	47	399	0.0	555
ABCDEF_34099	UniRef50_Q9LE59	0.0	Normal: 478	491	735	1356	1350	548	BlastAnno: 2158	UniRef50_Q9LE59	673	60.92	458	178	1	1610	240	215	672	0.0	630
ABCDEF_47984	UniRef50_Q7PC69	0.0	Normal: 663	457	734	1362	2147	696	BlastAnno: 2838	UniRef50_Q7PC69	745	59.27	712	226	9	373	2316	34	745	0.0	702
ABCDEF_51451	UniRef50_Q9LE59	0.0	Normal: 1883	1782	2202	4337	4350	2667	BlastAnno: 2874	UniRef50_Q9LE59	673	74.16	685	165	6	2559	505	1	673	0.0	982
ABCDEF_13370	UniRef50_Q8VZK9	0.0	Normal: 290	87	317	377	105	258	BlastAnno: 2033	UniRef50_Q8VZK9	729	51.65	635	278	11	2031	154	112	726	0.0	612
ABCDEF_48239	UniRef50_Q9LHZ7	0.0	Normal: 511	163	422	1154	1316	510	BlastAnno: 2714	UniRef50_Q9LHZ7	1170	76.20	769	156	9	478	2712	41	806	0.0	1201
ABCDEF_13338	UniRef50_Q7PC69	0.0	Normal: 335	468	417	2639	4443	616	BlastAnno: 2525	UniRef50_Q7PC69	745	62.45	727	209	12	298	2292	21	745	0.0	781
ABCDEF_24193	UniRef50_Q8L7F9	0.0	Normal: 113	72	132	203	180	119	BlastAnno: 1679	UniRef50_Q8L7F9	643	60.54	479	176	4	212	1609	1	479	0.0	592
ABCDEF_48514	UniRef50_Q9ZV71	0.0	Normal: 540	580	601	771	1033	926	BlastAnno: 1920	UniRef50_Q9ZV71	409	78.14	398	85	2	1618	431	12	409	0.0	637
2.4.1.152(4-galactosyl-N-acetylglucosaminide 3-alpha-L-fucosyltransferase;)																					
No Correspond Unigene																					
2.4.1.179(lactosylceramide beta-1,3-galactosyltransferase;)																					
No Correspond Unigene																					
2.4.1.228(lactosylceramide 4-alpha-galactosyltransferase;)																					
No Correspond Unigene																					
2.4.1.69(galactoside 2-alpha-L-fucosyltransferase;)																					
ABCDEF_87540	UniRef50_B9RLW2	9e-56	Normal: 0	0	0	0	0	36	BlastAnno: 523	UniRef50_B9RLW2	551	53.76	173	78	1	522	4	296	466	9e-56	192
2.4.1.79(globotriaosylceramide 3-beta-N-acetylgalactosaminyltransferase;)																					
No Correspond Unigene																					
2.4.1.88(globoside alpha-N-acetylgalactosaminyltransferase;)																					
No Correspond Unigene																					
2.4.99.-(Transferases;)																					
ABCDEF_40388	UniRef50_Q92187	2e-11	Normal: 282	215	308	417	512	350	BlastAnno: 2774	UniRef50_Q92187	359	57.53	73	29	2	2023	1808	116	187	2e-11	72.4
ABCDEF_40387	UniRef50_Q92187	2e-11	Normal: 587	417	654	1107	1268	855	BlastAnno: 2090	UniRef50_Q92187	359	57.53	73	29	2	1328	1113	116	187	2e-11	72.4
2.4.99.4(beta-galactoside alpha-2,3-sialyltransferase;)																					
ABCDEF_28297	UniRef50_B9SCR7	4e-167	Normal: 573	742	586	923	1062	1024	BlastAnno: 1855	UniRef50_B9SCR7	434	60.36	391	149	4	1454	294	46	434	4e-167	493
2.4.99.8(alpha-N-acetylneuraminate alpha-2,8-sialyltransferase;)																					
No Correspond Unigene																					
3.2.1.22(alpha-galactosidase;)																					
ABCDEF_31352	UniRef50_Q42656	0.0	Normal: 115	169	48	7	155	1183	BlastAnno: 1530	UniRef50_Q42656	378	76.76	370	85	1	242	1351	10	378	0.0	613
ABCDEF_52628	UniRef50_Q42656	0.0	Normal: 1131	2713	1338	2471	4073	4919	BlastAnno: 1435	UniRef50_Q42656	378	65.22	368	127	1	1227	124	11	377	0.0	523
ABCDEF_10850	UniRef50_Q42656	0.0	Normal: 170	155	153	167	153	166	BlastAnno: 1524	UniRef50_Q42656	378	74.66	371	94	0	389	1501	8	378	0.0	578
3.2.1.47(galactosylgalactosylglucosylceramidase;)																					
No Correspond Unigene																					
3.2.1.49(alpha-N-acetylgalactosaminidase;)																					
No Correspond Unigene																					
3.2.1.52(beta-N-acetylhexosaminidase;)																					
ABCDEF_16822	UniRef50_Q54K56	3e-109	Normal: 543	493	486	692	917	660	BlastAnno: 1760	UniRef50_Q54K56	564	36.42	519	294	10	1587	94	59	562	3e-109	347
>00604 Glycosphingolipid biosynthesis - ganglio series																					
2.3.1.-(Transferases;)																					
ABCDEF_520	UniRef50_Q9FF86	0.0	Normal: 1672	85	2273	5056	3383	1211	BlastAnno: 1811	UniRef50_Q9FF86	484	64.06	448	149	6	1664	354	3	449	0.0	573
ABCDEF_68664	UniRef50_Q9ZVN2	0.0	Normal: 79	35	85	107	193	63	BlastAnno: 1488	UniRef50_Q9ZVN2	704	63.99	411	142	3	46	1275	298	703	0.0	540
ABCDEF_52245	UniRef50_Q9FG87	0.0	Normal: 1878	635	1833	3814	1432	1376	BlastAnno: 1758	UniRef50_Q9FG87	529	55.35	486	204	4	4	1428	34	517	0.0	530
ABCDEF_16730	UniRef50_Q3EC11	0.0	Normal: 281	204	237	365	377	458	BlastAnno: 2092	UniRef50_Q3EC11	536	59.33	536	204	8	196	1785	3	530	0.0	661
ABCDEF_4655	UniRef50_A5BMG1	0.0	Normal: 2426	1856	3606	6958	4608	2755	BlastAnno: 2261	UniRef50_A5BMG1	481	68.37	528	112	10	263	1843	3	476	0.0	667
ABCDEF_13860	UniRef50_Q9C533	0.0	Normal: 588	220	570	1451	1338	310	BlastAnno: 2648	UniRef50_Q9C533	596	63.37	606	177	12	2226	511	1	595	0.0	558
ABCDEF_28294	UniRef50_Q9SYZ0	0.0	Normal: 1038	4070	1427	6745	877	2797	BlastAnno: 1785	UniRef50_Q9SYZ0	493	52.37	485	223	3	1598	162	5	487	0.0	533
ABCDEF_47718	UniRef50_Q9C533	0.0	Normal: 1599	236	1657	2093	1231	588	BlastAnno: 2680	UniRef50_Q9C533	596	65.02	626	165	14	2280	472	1	595	0.0	634
ABCDEF_45080	UniRef50_Q9SYZ0	0.0	Normal: 1005	592	1079	2360	3762	2217	BlastAnno: 2130	UniRef50_Q9SYZ0	493	57.85	484	197	3	302	1735	5	487	0.0	533
ABCDEF_25377	UniRef50_Q9C6L5	0.0	Normal: 37	2090	138	2573	1026	1298	BlastAnno: 1334	UniRef50_Q9C6L5	492	79.74	385	76	1	1155	1	6	388	0.0	654
ABCDEF_33470	UniRef50_Q9LW26	0.0	Normal: 172	98	156	260	391	149	BlastAnno: 2993	UniRef50_Q9LW26	701	48.96	627	301	5	655	2508	85	701	0.0	581
ABCDEF_40654	UniRef50_Q9H0A0	0.0	Normal: 638	213	530	318	318	286	BlastAnno: 2216	UniRef50_Q9H0A0	1025	56.87	721	279	6	2127	13	91	795	0.0	756
ABCDEF_33460	UniRef50_Q9ZVN2	0.0	Normal: 144	71	119	216	306	97	BlastAnno: 2691	UniRef50_Q9ZVN2	704	61.50	587	191	4	379	2133	83	636	0.0	715
ABCDEF_14442	UniRef50_Q9SYZ0	0.0	Normal: 341	684	439	1040	1601	552	BlastAnno: 1779	UniRef50_Q9SYZ0	493	63.12	480	171	2	1570	149	5	484	0.0	628
ABCDEF_52626	UniRef50_B6SYP9	0.0	Normal: 815	346	1131	3015	1537	108	BlastAnno: 1861	UniRef50_B6SYP9	494	55.02	438	189	3	1538	249	51	488	0.0	536
ABCDEF_644	UniRef50_Q9SB58	0.0	Normal: 704	444	913	969	1431	848	BlastAnno: 1777	UniRef50_Q9SB58	407	64.15	424	129	8	1572	316	1	406	0.0	554
ABCDEF_27087	UniRef50_Q52T38	0.0	Normal: 324	221	233	419	343	412	BlastAnno: 1631	UniRef50_Q52T38	620	75.95	420	91	1	1409	150	1	410	0.0	681
ABCDEF_34187	UniRef50_Q8L5Y5	0.0	Normal: 497	415	588	523	291	433	BlastAnno: 2677	UniRef50_Q8L5Y5	718	57.30	733	251	15	202	2274	1	713	0.0	683
ABCDEF_47966	UniRef50_Q9SYZ0	0.0	Normal: 577	934	326	580	682	3440	BlastAnno: 2177	UniRef50_Q9SYZ0	493	56.40	484	204	3	1780	347	5	487	0.0	535
ABCDEF_51189	UniRef50_Q52T38	0.0	Normal: 135	152	128	181	219	327	BlastAnno: 1766	UniRef50_Q52T38	620	68.09	470	137	3	80	1483	130	588	0.0	641
ABCDEF_41288	UniRef50_Q9SB58	0.0	Normal: 307	183	332	637	649	322	BlastAnno: 1403	UniRef50_Q9SB58	407	67.56	410	110	6	1401	184	15	405	0.0	523
ABCDEF_33461	UniRef50_Q9ZVN2	0.0	Normal: 142	84	140	206	283	119	BlastAnno: 2904	UniRef50_Q9ZVN2	704	60.98	574	201	6	2271	559	83	636	0.0	686
ABCDEF_14334	UniRef50_Q9SYZ0	0.0	Normal: 2263	129	1724	3469	866	283	BlastAnno: 2039	UniRef50_Q9SYZ0	493	63.22	484	171	2	369	1802	5	487	0.0	627
ABCDEF_24844	UniRef50_Q9FF86	0.0	Normal: 1857	3686	2657	21940	11467	4090	BlastAnno: 1640	UniRef50_Q9FF86	484	66.89	447	135	6	1492	185	2	446	0.0	575
ABCDEF_51190	UniRef50_Q52T38	0.0	Normal: 145	169	142	210	232	335	BlastAnno: 1891	UniRef50_Q52T38	620	70.04	514	141	3	73	1608	86	588	0.0	726
ABCDEF_14085	UniRef50_Q8L5Y5	0.0	Normal: 353	41	384	527	331	280	BlastAnno: 2362	UniRef50_Q8L5Y5	718	62.07	704	220	12	2360	348	24	713	0.0	676
ABCDEF_33458	UniRef50_Q9LW26	0.0	Normal: 158	78	130	240	342	106	BlastAnno: 2822	UniRef50_Q9LW26	701	46.72	655	302	5	400	2337	85	701	0.0	568
ABCDEF_25193	UniRef50_Q9C6L5	0.0	Normal: 3256	368	3548	6230	5109	1288	BlastAnno: 1786	UniRef50_Q9C6L5	492	65.83	480	156	4	1668	241	17	492	0.0	668
ABCDEF_33457	UniRef50_Q9ZVN2	0.0	Normal: 168	83	137	250	375	112	BlastAnno: 2823	UniRef50_Q9ZVN2	704	65.12	496	168	3	379	1860	83	575	0.0	648
ABCDEF_7634	UniRef50_Q9C6L5	0.0	Normal: 2543	9303	2711	11578	5533	10032	BlastAnno: 2064	UniRef50_Q9C6L5	492	52.76	489	225	3	1734	268	1	483	0.0	535
ABCDEF_299	UniRef50_Q71N54	0.0	Normal: 569	1147	511	900	649	1879	BlastAnno: 2017	UniRef50_Q71N54	535	61.73	533	198	5	338	1924	4	534	0.0	677
ABCDEF_27086	UniRef50_Q52T38	0.0	Normal: 784	470	617	843	893	976	BlastAnno: 2398	UniRef50_Q52T38	620	70.80	637	165	4	225	2129	1	618	0.0	887

ABCDEF_51188	UniRef50_Q52T38	0.0	Normal: 273	327	255	415	349	441	BlastAnno: 2324	UniRef50_Q52T38	620	71.30	568	150	3	2041	344	32	588	0.0	815
ABCDEF_44812	UniRef50_Q6DR03	0.0	Normal: 982	902	1005	1071	959	2999	BlastAnno: 2450	UniRef50_Q6DR03	565	54.97	644	198	17	2142	220	1	555	0.0	540
2.4.1.-(Transferases;)																					
ABCDEF_18004	UniRef50_Q6YWK8	0.0	Normal: 1562	1736	2009	3452	4438	2400	BlastAnno: 2298	UniRef50_Q6YWK8	570	65.27	429	136	1	1941	694	57	485	0.0	608
ABCDEF_52379	UniRef50_Q9LHZ7	0.0	Normal: 4015	4921	4546	10520	13430	4731	BlastAnno: 3479	UniRef50_Q9LHZ7	1170	48.04	893	342	16	825	3242	299	1156	0.0	787
ABCDEF_48593	UniRef50_Q9SK82	0.0	Normal: 12	420	19	343	763	253	BlastAnno: 1834	UniRef50_Q9SK82	489	60.08	481	186	4	1714	278	10	486	0.0	633
ABCDEF_13375	UniRef50_Q8VZK9	0.0	Normal: 108	123	208	526	34	294	BlastAnno: 1738	UniRef50_Q8VZK9	729	53.62	552	234	8	94	1737	18	551	0.0	582
ABCDEF_40982	UniRef50_Q96301	0.0	Normal: 1037	706	819	1249	1084	920	BlastAnno: 3418	UniRef50_Q96301	914	79.60	902	173	3	243	2948	1	891	0.0	1506
ABCDEF_47674	UniRef50_Q9LHZ7	0.0	Normal: 274	92	202	599	505	280	BlastAnno: 2526	UniRef50_Q9LHZ7	1170	75.93	777	151	8	2260	2	15	779	0.0	1177
ABCDEF_34097	UniRef50_Q9LE59	0.0	Normal: 626	613	923	1797	1829	692	BlastAnno: 2521	UniRef50_Q9LE59	673	60.92	458	178	1	1687	317	215	672	0.0	630
ABCDEF_34098	UniRef50_Q9LE59	0.0	Normal: 457	464	697	1283	1312	539	BlastAnno: 2199	UniRef50_Q9LE59	673	60.92	458	178	1	1687	317	215	672	0.0	628
ABCDEF_10848	UniRef50_Q9M156	0.0	Normal: 157	2988	163	408	772	1566	BlastAnno: 1810	UniRef50_Q9M156	480	60.09	456	176	4	1700	336	4	454	0.0	553
ABCDEF_41182	UniRef50_Q9LHZ7	0.0	Normal: 150	2169	221	814	1407	495	BlastAnno: 3604	UniRef50_Q9LHZ7	1170	51.03	870	333	15	2822	456	299	1156	0.0	826
ABCDEF_40981	UniRef50_Q96301	0.0	Normal: 156	97	110	160	179	173	BlastAnno: 1225	UniRef50_Q96301	914	86.92	344	45	0	1112	81	394	737	0.0	652
ABCDEF_9506	UniRef50_Q9LHZ7	0.0	Normal: 1302	2128	1494	3356	4644	2206	BlastAnno: 3751	UniRef50_Q9LHZ7	1170	49.38	891	343	17	927	3359	299	1161	0.0	812
ABCDEF_9500	UniRef50_Q9LHZ7	0.0	Normal: 183	188	186	576	498	260	BlastAnno: 1717	UniRef50_Q9LHZ7	1170	47.98	644	234	14	3	1715	418	1033	0.0	571
ABCDEF_100	UniRef50_Q9M8Y0	0.0	Normal: 228	309	224	416	461	141	BlastAnno: 2109	UniRef50_Q9M8Y0	977	77.49	533	120	0	1610	12	50	582	0.0	884
ABCDEF_33659	UniRef50_Q9LHZ7	0.0	Normal: 1282	1948	2248	5826	10642	2083	BlastAnno: 4289	UniRef50_Q9LHZ7	1170	48.70	887	345	15	2955	538	299	1156	0.0	788
ABCDEF_37667	UniRef50_Q9LHZ7	0.0	Normal: 248	3617	251	1237	2078	970	BlastAnno: 3161	UniRef50_Q9LHZ7	1170	49.72	885	335	14	2671	260	301	1156	0.0	810
ABCDEF_47673	UniRef50_Q9LHZ7	0.0	Normal: 876	296	828	1628	1721	1172	BlastAnno: 3878	UniRef50_Q9LHZ7	1170	80.88	1161	186	8	3642	232	15	1163	0.0	1865
ABCDEF_99	UniRef50_Q9M8Y0	0.0	Normal: 272	330	222	398	423	145	BlastAnno: 2094	UniRef50_Q9M8Y0	977	77.36	561	127	0	1683	1	50	610	0.0	929
ABCDEF_20658	UniRef50_Q8VZK9	0.0	Normal: 373	33	193	64	503	141	BlastAnno: 2316	UniRef50_Q8VZK9	729	53.35	508	221	7	47	1564	11	504	0.0	559
ABCDEF_10183	UniRef50_A7XDQ9	0.0	Normal: 422	455	346	577	378	610	BlastAnno: 2362	UniRef50_A7XDQ9	684	50.94	693	307	12	281	2287	1	684	0.0	670
ABCDEF_30745	UniRef50_A7XDQ9	0.0	Normal: 1209	351	1031	1645	1226	635	BlastAnno: 2646	UniRef50_A7XDQ9	684	57.58	554	227	6	546	2189	133	684	0.0	664
ABCDEF_13336	UniRef50_Q7PC69	0.0	Normal: 148	563	194	957	1575	816	BlastAnno: 2584	UniRef50_Q7PC69	745	71.56	559	136	6	744	2351	187	745	0.0	730
ABCDEF_21426	UniRef50_Q8RU27	0.0	Normal: 2658	1979	3417	6157	9464	2599	BlastAnno: 1530	UniRef50_Q8RU27	366	89.44	360	34	2	1358	282	1	357	0.0	667
ABCDEF_71242	UniRef50_Q9ZV71	0.0	Normal: 134	122	179	480	754	119	BlastAnno: 1835	UniRef50_Q9ZV71	409	73.35	409	103	4	231	1439	1	409	0.0	587
ABCDEF_52020	UniRef50_Q6YWK8	0.0	Normal: 1635	1534	1975	4284	10353	2289	BlastAnno: 2394	UniRef50_Q6YWK8	570	58.37	514	185	2	403	1857	57	570	0.0	607
ABCDEF_51269	UniRef50_Q6YWK8	0.0	Normal: 147	85	103	21	33	438	BlastAnno: 1864	UniRef50_Q6YWK8	570	54.28	514	204	2	1646	198	57	570	0.0	586
ABCDEF_30753	UniRef50_Q9ZV71	0.0	Normal: 290	497	417	629	1013	634	BlastAnno: 1502	UniRef50_Q9ZV71	409	72.32	401	107	2	311	1501	1	401	0.0	590
ABCDEF_4233	UniRef50_Q9ZV71	0.0	Normal: 369	412	550	936	860	750	BlastAnno: 2004	UniRef50_Q9ZV71	409	72.13	409	110	2	370	1584	1	409	0.0	613
ABCDEF_17525	UniRef50_Q8RU27	0.0	Normal: 1500	1289	2149	3711	4446	1069	BlastAnno: 1507	UniRef50_Q8RU27	366	91.35	347	30	0	1371	331	3	349	0.0	671
ABCDEF_18005	UniRef50_Q6YWK8	0.0	Normal: 1842	2042	2515	4214	5208	2842	BlastAnno: 2305	UniRef50_Q6YWK8	570	59.14	514	180	2	1948	497	57	570	0.0	642
ABCDEF_41140	UniRef50_Q6YWK8	0.0	Normal: 86	1314	93	753	1787	2076	BlastAnno: 2346	UniRef50_Q6YWK8	570	60.00	510	174	2	431	1870	57	566	0.0	669
ABCDEF_18002	UniRef50_Q9LHZ7	0.0	Normal: 376	127	367	601	1190	520	BlastAnno: 1605	UniRef50_Q9LHZ7	1170	89.75	400	41	0	1603	404	771	1170	0.0	732
ABCDEF_13339	UniRef50_Q7PC69	0.0	Normal: 127	814	183	541	1135	1144	BlastAnno: 2610	UniRef50_Q7PC69	745	70.48	559	141	6	725	2329	187	745	0.0	729
ABCDEF_41141	UniRef50_Q6YWK8	0.0	Normal: 67	1107	84	708	1690	1854	BlastAnno: 2461	UniRef50_Q6YWK8	570	60.00	510	174	2	1916	477	57	566	0.0	668
ABCDEF_44583	UniRef50_Q9LE59	0.0	Normal: 681	561	559	947	1745	789	BlastAnno: 2470	UniRef50_Q9LE59	673	59.21	456	185	1	1749	385	214	669	0.0	621
ABCDEF_3869	UniRef50_Q9LE59	0.0	Normal: 333	393	415	809	907	754	BlastAnno: 2136	UniRef50_Q9LE59	673	59.05	613	227	4	1827	1	81	673	0.0	699
ABCDEF_28947	UniRef50_Q9SK82	0.0	Normal: 0	851	3	0	0	116	BlastAnno: 1567	UniRef50_Q9SK82	489	62.40	484	176	4	1524	85	1	482	0.0	652
ABCDEF_31119	UniRef50_Q9M8Y0	0.0	Normal: 2212	1279	1917	2933	3341	1803	BlastAnno: 3695	UniRef50_Q9M8Y0	977	81.08	925	174	1	392	3163	50	974	0.0	1583
ABCDEF_37311	UniRef50_Q9LE59	0.0	Normal: 175	133	191	549	421	366	BlastAnno: 1612	UniRef50_Q9LE59	673	62.96	486	153	4	1406	3	4	480	0.0	542
ABCDEF_24190	UniRef50_Q8L7F9	0.0	Normal: 397	265	492	583	553	371	BlastAnno: 2564	UniRef50_Q8L7F9	643	63.91	640	218	4	2353	473	1	640	0.0	850
ABCDEF_30548	UniRef50_Q9FH36	0.0	Normal: 17	916	27	151	420	126	BlastAnno: 1963	UniRef50_Q9FH36	535	72.47	534	138	3	102	1676	1	534	0.0	767
ABCDEF_27416	UniRef50_Q7PC69	0.0	Normal: 575	837	875	1344	2015	936	BlastAnno: 2454	UniRef50_Q7PC69	745	66.11	416	115	4	2337	1168	126	541	0.0	518
ABCDEF_48373	UniRef50_A7XDQ9	0.0	Normal: 1364	773	1336	1451	1791	1202	BlastAnno: 3042	UniRef50_A7XDQ9	684	63.68	691	234	6	2405	360	1	683	0.0	915
ABCDEF_3868	UniRef50_Q9LE59	0.0	Normal: 224	274	301	555	686	524	BlastAnno: 1670	UniRef50_Q9LE59	673	65.31	516	176	1	1556	9	161	673	0.0	696
ABCDEF_31288	UniRef50_Q940V3	0.0	Normal: 247	309	183	101	124	655	BlastAnno: 1658	UniRef50_Q940V3	470	56.01	466	197	7	1614	232	8	470	0.0	537
ABCDEF_52574	UniRef50_Q09332	0.0	Normal: 885	474	1068	1835	1853	816	BlastAnno: 3720	UniRef50_Q09332	1548	47.89	687	310	14	2551	533	859	1511	0.0	598
ABCDEF_79752	UniRef50_Q9ZV71	0.0	Normal: 158	183	245	400	379	352	BlastAnno: 1266	UniRef50_Q9ZV71	409	71.39	360	99	2	31	1098	1	360	0.0	526
ABCDEF_9509	UniRef50_Q9LHZ7	0.0	Normal: 1296	2171	1498	3410	4683	2232	BlastAnno: 3897	UniRef50_Q9LHZ7	1170	49.38	891	343	17	1073	3505	299	1161	0.0	812
ABCDEF_48200	UniRef50_Q6YWK8	0.0	Normal: 1768	576	3156	4431	6634	491	BlastAnno: 2274	UniRef50_Q6YWK8	570	57.98	514	185	4	291	1739	57	570	0.0	642
ABCDEF_24155	UniRef50_Q9ZVI7	0.0	Normal: 503	247	607	1676	1833	578	BlastAnno: 2708	UniRef50_Q9ZVI7	619	52.33	621	264	11	306	2123	15	618	0.0	601
ABCDEF_21246	UniRef50_Q9SKT6	0.0	Normal: 92	107	188	380	834	84	BlastAnno: 1791	UniRef50_Q9SKT6	536	56.61	484	204	5	123	1562	52	533	0.0	568
ABCDEF_32317	UniRef50_Q8RU27	0.0	Normal: 12	9	3	118	800	62	BlastAnno: 1076	UniRef50_Q8RU27	366	87.02	285	37	0	1075	221	64	348	0.0	535
ABCDEF_9502	UniRef50_Q9LHZ7	0.0	Normal: 610	486	568	1331	1403	591	BlastAnno: 1961	UniRef50_Q9LHZ7	1170	48.64	625	249	13	3	1742	564	1161	0.0	570
ABCDEF_23298	UniRef50_Q9FH36	0.0	Normal: 128	527	130	223	314	864	BlastAnno: 2832	UniRef50_Q9FH36	535	83.16	196	33	0	696	1283	340	535	0.0	357
ABCDEF_40595	UniRef50_O80898	0.0	Normal: 229	135	103	25	307	325	BlastAnno: 2413	UniRef50_O80898	757	44.68	761	378	13	95	2299	10	753	0.0	587
ABCDEF_63947	UniRef50_Q9SK82	0.0	Normal: 0	267	0	0	71	0	BlastAnno: 1446	UniRef50_Q9SK82	489	58.33	468	189	4	60	1445	1	468	0.0	592
ABCDEF_67847	UniRef50_Q9M8Y0	0.0	Normal: 794	476	871	1081	1401	868	BlastAnno: 1591	UniRef50_Q9M8Y0	977	84.86	370	56	0	1589	480	605	974	0.0	685
ABCDEF_65666	UniRef50_Q09332	0.0	Normal: 800	441	946	1606	1637	735	BlastAnno: 3518	UniRef50_Q09332	1548	47.54	671	305	13	1162	3087	859	1511	0.0	586
ABCDEF_38641	UniRef50_Q9FWA4	0.0	Normal: 674	967	1162	2603	4404	2475	BlastAnno: 1599	UniRef50_Q9FWA4	561	81.35	386	72	0	1	1158	176	561	0.0	641
ABCDEF_17914	UniRef50_Q9SKT6	0.0	Normal: 784	567	886	1447	1439	1424	BlastAnno: 2049	UniRef50_Q9SKT6	536	72.95	536	140	2	204	1796	1	536	0.0	785
ABCDEF_18286	UniRef50_Q6NQB7	0.0	Normal: 1131	1108	1225	1872	1722	3329	BlastAnno: 1752	UniRef50_Q6NQB7	393	73.80	397	81	6	1527	367	10	393	0.0	607
ABCDEF_119	UniRef50_Q8RU27	0.0	Normal: 1613	1155	2249	3558	7663	1775	BlastAnno: 1532	UniRef50_Q8RU27	366	88.33	360	38	2	1306	230	1	357	0.0	663
ABCDEF_47603	UniRef50_Q9M9Y5	0.0	Normal: 2609	8779	1664	1103	2085	22991	BlastAnno: 2830	UniRef50_Q9M9Y5	589	54.04	594	267	3	2475	694	1	588	0.0	654
ABCDEF_24192	UniRef50_Q8L7F9	0.0	Normal: 391	244	465	553	497	345	BlastAnno: 2477	UniRef50_Q8L7F9	643	58.99	434	165	4	212	1474	1	434	0.0	513
ABCDEF_17482	UniRef50_Q9SKT6	0.0	Normal: 615	379	816	1068	1470	852	BlastAnno: 2083	UniRef50_Q9SKT6	536	71.11	540	147	4	1915	311	1	536	0.0	786
ABCDEF_85909	UniRef50_Q8VZK9	0.0	Normal: 28	45	9	10	44	127	BlastAnno: 1473	UniRef50_Q8VZK9	729	55.37	475	198	6	1419	1	11	473	0.0	536
ABCDEF_9503	UniRef50_Q9LHZ7	0.0	Normal: 487	544	524	1242	1320	833	BlastAnno: 2087	UniRef50_Q9LHZ7	1170	50.35	574	236	8	57	1712	615	1161	0.0	567
ABCDEF_40594	UniRef50_O80898	0.0	Normal: 256	127	102	41	331	326	BlastAnno: 2417	UniRef50_O80898	757	45.76	719	348	12	213	2291	51	753	0.0	585
ABCDEF_17141	UniRef50_Q9LHZ7	0.0	Normal: 74	1734	69	380	701	333	BlastAnno: 3337	UniRef50_Q9LHZ7	1170	50.92	868	335	14	2539	179	299	1156	0.0	816

ABCDEF_33658	UniRef50_Q9LHZ7	0.0	Normal: 384	308	395	805	1003	602	BlastAnno: 2961	UniRef50_Q9LHZ7	1170	48.76	888	343	14	204	2621	299	1156	0.0	792
ABCDEF_20579	UniRef50_Q7PC69	0.0	Normal: 1007	795	1238	2311	2309	1345	BlastAnno: 3113	UniRef50_Q7PC69	745	59.97	767	229	14	2667	535	1	745	0.0	768
ABCDEF_31118	UniRef50_Q9M8Y0	0.0	Normal: 2135	1267	1900	3004	3329	1757	BlastAnno: 3787	UniRef50_Q9M8Y0	977	81.10	709	133	1	1132	3255	266	974	0.0	1217
ABCDEF_9499	UniRef50_Q9LHZ7	0.0	Normal: 205	207	215	632	538	275	BlastAnno: 1794	UniRef50_Q9LHZ7	1170	48.88	667	240	14	11	1792	395	1033	0.0	605
ABCDEF_48199	UniRef50_Q6YWK8	0.0	Normal: 1506	467	2429	3094	4233	439	BlastAnno: 1997	UniRef50_Q6YWK8	570	63.10	439	146	2	291	1559	57	495	0.0	610
ABCDEF_40983	UniRef50_Q96301	0.0	Normal: 406	261	343	392	415	433	BlastAnno: 2265	UniRef50_Q96301	914	79.17	504	99	1	114	1625	394	891	0.0	854
ABCDEF_17140	UniRef50_Q9LHZ7	0.0	Normal: 45	1049	49	216	386	208	BlastAnno: 2293	UniRef50_Q9LHZ7	1170	52.34	770	281	13	2138	57	397	1156	0.0	739
ABCDEF_18352	UniRef50_Q9FWA4	0.0	Normal: 2427	2688	2969	5519	6547	4855	BlastAnno: 2224	UniRef50_Q9FWA4	561	69.31	505	148	3	1887	382	61	561	0.0	683
ABCDEF_27767	UniRef50_Q9LHZ7	0.0	Normal: 2576	5141	2841	10249	14583	5031	BlastAnno: 3981	UniRef50_Q9LHZ7	1170	49.66	888	337	16	2927	504	299	1156	0.0	818
ABCDEF_33661	UniRef50_Q9LHZ7	0.0	Normal: 414	340	507	879	790	783	BlastAnno: 2185	UniRef50_Q9LHZ7	1170	48.66	748	277	14	2184	169	440	1156	0.0	662
ABCDEF_14685	UniRef50_A7XDQ9	0.0	Normal: 233	308	134	573	292	334	BlastAnno: 1947	UniRef50_A7XDQ9	684	56.84	468	186	6	569	1945	1	461	0.0	545
ABCDEF_20660	UniRef50_Q8VZK9	0.0	Normal: 22	48	13	15	52	144	BlastAnno: 1473	UniRef50_Q8VZK9	729	55.16	475	199	6	55	1473	11	473	0.0	537
ABCDEF_21315	UniRef50_Q9LHZ7	0.0	Normal: 3412	252	1450	0	79	1228	BlastAnno: 3765	UniRef50_Q9LHZ7	1170	63.78	1096	340	15	308	3508	70	1137	0.0	1333
ABCDEF_533	UniRef50_Q9C8W3	0.0	Normal: 230	204	269	505	411	301	BlastAnno: 1688	UniRef50_Q9C8W3	401	70.33	364	94	2	393	1475	47	399	0.0	555
ABCDEF_34099	UniRef50_Q9LE59	0.0	Normal: 478	491	735	1356	1350	548	BlastAnno: 2158	UniRef50_Q9LE59	673	60.92	458	178	1	1610	240	215	672	0.0	630
ABCDEF_47984	UniRef50_Q7PC69	0.0	Normal: 663	457	734	1362	2147	696	BlastAnno: 2838	UniRef50_Q7PC69	745	59.27	712	226	9	373	2316	34	745	0.0	702
ABCDEF_51451	UniRef50_Q9LE59	0.0	Normal: 1883	1782	2202	4337	4350	2667	BlastAnno: 2874	UniRef50_Q9LE59	673	74.16	685	165	6	2559	505	1	673	0.0	982
ABCDEF_13370	UniRef50_Q8VZK9	0.0	Normal: 290	87	317	377	105	258	BlastAnno: 2033	UniRef50_Q8VZK9	729	51.65	635	278	11	2031	154	112	726	0.0	612
ABCDEF_48239	UniRef50_Q9LHZ7	0.0	Normal: 511	163	422	1154	1316	510	BlastAnno: 2714	UniRef50_Q9LHZ7	1170	76.20	769	156	9	478	2712	41	806	0.0	1201
ABCDEF_13338	UniRef50_Q7PC69	0.0	Normal: 335	468	417	2639	4443	616	BlastAnno: 2525	UniRef50_Q7PC69	745	62.45	727	209	12	298	2292	21	745	0.0	781
ABCDEF_24193	UniRef50_Q8L7F9	0.0	Normal: 113	72	132	203	180	119	BlastAnno: 1679	UniRef50_Q8L7F9	643	60.54	479	176	4	212	1609	1	479	0.0	592
ABCDEF_48514	UniRef50_Q9ZV71	0.0	Normal: 540	580	601	771	1033	926	BlastAnno: 1920	UniRef50_Q9ZV71	409	78.14	398	85	2	1618	431	12	409	0.0	637

2.4.1.62(ganglioside galactosyltransferase;)

No Correspond Unigene

2.4.1.92(No Name)

No Correspond Unigene

2.4.99.-(Transferases;)

ABCDEF_40388	UniRef50_Q92187	2e-11	Normal: 282	215	308	417	512	350	BlastAnno: 2774	UniRef50_Q92187	359	57.53	73	29	2	2023	1808	116	187	2e-11	72.4
ABCDEF_40387	UniRef50_Q92187	2e-11	Normal: 587	417	654	1107	1268	855	BlastAnno: 2090	UniRef50_Q92187	359	57.53	73	29	2	1328	1113	116	187	2e-11	72.4

2.4.99.4(beta-galactoside alpha-2,3-sialyltransferase;)

ABCDEF_28297	UniRef50_B9SCR7	4e-167	Normal: 573	742	586	923	1062	1024	BlastAnno: 1855	UniRef50_B9SCR7	434	60.36	391	149	4	1454	294	46	434	4e-167	493

2.4.99.7(alpha-N-acetylneuraminyl-2,3-beta-galactosyl-1,3-N-acetylgalactosaminide 6-alpha-sialyltransferase;)

No Correspond Unigene

2.4.99.8(alpha-N-acetylneuraminate alpha-2,8-sialyltransferase;)

No Correspond Unigene

2.4.99.9(lactosylceramide alpha-2,3-sialyltransferase;)

No Correspond Unigene

3.2.1.23(beta-galactosidase;)

ABCDEF_48113	UniRef50_Q6Z6K4	0.0	Normal: 61	2624	32	127	673	5080	BlastAnno: 2898	UniRef50_Q6Z6K4	729	63.35	704	250	4	2505	394	33	728	0.0	931
ABCDEF_23777	UniRef50_Q6Z6K4	0.0	Normal: 219	63	227	164	235	472	BlastAnno: 2261	UniRef50_Q6Z6K4	729	66.38	690	225	5	2	2071	46	728	0.0	944
ABCDEF_3074	UniRef50_Q6Z6K4	0.0	Normal: 431	1551	321	2473	394	19728	BlastAnno: 3342	UniRef50_Q6Z6K4	729	67.09	702	221	6	415	2517	35	727	0.0	994
ABCDEF_43854	UniRef50_Q6Z6K4	0.0	Normal: 1	9	1	105	49	28	BlastAnno: 1781	UniRef50_Q6Z6K4	729	58.79	512	180	6	1	1536	147	627	0.0	595
ABCDEF_44469	UniRef50_Q8W0A1	0.0	Normal: 162	225	175	701	194	597	BlastAnno: 2319	UniRef50_Q8W0A1	827	46.10	679	324	12	2236	221	183	826	0.0	583
ABCDEF_13827	UniRef50_Q6Z6K4	0.0	Normal: 219	1054	213	1958	627	863	BlastAnno: 3038	UniRef50_Q6Z6K4	729	57.90	715	270	8	419	2539	36	727	0.0	825
ABCDEF_21624	UniRef50_Q6Z6K4	0.0	Normal: 4958	5483	4088	4956	4209	6920	BlastAnno: 3330	UniRef50_Q6Z6K4	729	62.64	696	251	4	2975	888	32	718	0.0	932
ABCDEF_43857	UniRef50_Q6Z6K4	0.0	Normal: 2	9	4	110	53	30	BlastAnno: 1655	UniRef50_Q6Z6K4	729	60.07	546	187	6	1	1638	147	661	0.0	655
ABCDEF_43861	UniRef50_Q6Z6K4	0.0	Normal: 4	15	5	162	72	47	BlastAnno: 1891	UniRef50_Q6Z6K4	729	65.21	526	179	3	107	1684	34	555	0.0	719
ABCDEF_84604	UniRef50_Q6Z6K4	0.0	Normal: 34	1696	14	81	379	2927	BlastAnno: 2528	UniRef50_Q6Z6K4	729	62.99	670	240	4	394	2403	33	694	0.0	881
ABCDEF_13826	UniRef50_Q6Z6K4	0.0	Normal: 184	889	166	1515	526	703	BlastAnno: 2897	UniRef50_Q6Z6K4	729	55.30	613	243	8	2286	472	138	727	0.0	657
ABCDEF_10626	UniRef50_Q6Z6K4	0.0	Normal: 229	909	224	1002	2471	3794	BlastAnno: 3133	UniRef50_Q6Z6K4	729	57.14	700	285	8	498	2588	40	727	0.0	809
ABCDEF_27471	UniRef50_Q6Z6K4	0.0	Normal: 992	533	1105	1489	2430	1240	BlastAnno: 3113	UniRef50_Q6Z6K4	729	64.49	704	240	5	314	2422	35	729	0.0	962
ABCDEF_43858	UniRef50_Q6Z6K4	0.0	Normal: 6	15	7	186	85	54	BlastAnno: 2028	UniRef50_Q6Z6K4	729	65.35	635	213	5	107	2011	34	661	0.0	862
ABCDEF_31373	UniRef50_Q6Z6K4	0.0	Normal: 1333	1029	1160	1241	1216	1855	BlastAnno: 3006	UniRef50_Q6Z6K4	729	52.03	713	319	7	2761	626	37	727	0.0	732
ABCDEF_10066	UniRef50_Q93Z24	0.0	Normal: 999	932	1523	1674	2007	1465	BlastAnno: 3015	UniRef50_Q93Z24	697	65.89	645	208	4	2659	737	61	697	0.0	865
ABCDEF_51808	UniRef50_Q6Z6K4	0.0	Normal: 364	454	209	36	62	1109	BlastAnno: 2817	UniRef50_Q6Z6K4	729	54.56	713	306	8	2582	447	32	727	0.0	791
ABCDEF_48111	UniRef50_Q6Z6K4	0.0	Normal: 41	1700	15	77	347	2953	BlastAnno: 2607	UniRef50_Q6Z6K4	729	67.21	494	157	3	394	1875	33	521	0.0	696
ABCDEF_17519	UniRef50_A5F5U6	0.0	Normal: 1178	1482	1392	2252	1850	3281	BlastAnno: 3706	UniRef50_A5F5U6	1024	39.71	1093	547	21	3469	254	13	1014	0.0	729
ABCDEF_40816	UniRef50_Q6Z6K4	0.0	Normal: 1166	754	898	2543	5298	532	BlastAnno: 3066	UniRef50_Q6Z6K4	729	62.55	705	254	4	2970	856	35	729	0.0	936
ABCDEF_44668	UniRef50_Q8GX69	0.0	Normal: 146	74	84	327	133	152	BlastAnno: 1396	UniRef50_Q8GX69	815	66.74	463	152	2	6	1394	23	483	0.0	671
ABCDEF_43856	UniRef50_Q6Z6K4	0.0	Normal: 12	20	12	195	92	71	BlastAnno: 1873	UniRef50_Q6Z6K4	729	61.89	488	179	5	1	1464	147	627	0.0	615
ABCDEF_44470	UniRef50_Q6Z6K4	0.0	Normal: 287	387	252	1193	287	741	BlastAnno: 2917	UniRef50_Q6Z6K4	729	52.28	723	310	9	2707	551	38	729	0.0	752
ABCDEF_49396	UniRef50_Q6Z6K4	0.0	Normal: 0	452	3	48	33	6872	BlastAnno: 2312	UniRef50_Q6Z6K4	729	65.83	676	221	7	2312	288	62	728	0.0	893
ABCDEF_10631	UniRef50_Q6Z6K4	0.0	Normal: 3079	207	2624	1970	2833	969	BlastAnno: 3058	UniRef50_Q6Z6K4	729	60.74	703	267	4	2943	835	36	729	0.0	917
ABCDEF_48112	UniRef50_Q6Z6K4	0.0	Normal: 60	2628	33	127	663	5106	BlastAnno: 2977	UniRef50_Q6Z6K4	729	67.21	494	157	3	2584	1103	33	521	0.0	696

3.2.1.52(beta-N-acetylhexosaminidase;)

ABCDEF_16822	UniRef50_Q54K56	3e-109	Normal: 543	493	486	692	917	660	BlastAnno: 1760	UniRef50_Q54K56	564	36.42	519	294	10	1587	94	59	562	3e-109	347

>00620 Pyruvate metabolism

1.1.1.21(aldehyde reductase;)

ABCDEF_52723	UniRef50_Q6IMN8	5e-83	Normal: 1383	764	1621	1675	2066	2197	BlastAnno: 1221	UniRef50_Q6IMN8	297	44.09	313	153	5	135	1061	2	296	5e-83	265

1.1.1.27(L-lactate dehydrogenase;)

ABCDEF_38409	UniRef50_P22988	5e-157	Normal: 468	369	408	758	1349	382	BlastAnno: 1380	UniRef50_P22988	356	66.67	357	111	3	121	1170	1	356	5e-157	458

1.1.1.28(D-lactate dehydrogenase;)

No Correspond Unigene																					
1.1.1.37(malate dehydrogenase;)																					
ABCDEF_25169	UniRef50_Q9SN86	0.0	Normal: 938	614	1116	1317	4446	973	BlastAnno: 1856	UniRef50_Q9SN86	403	75.50	400	88	2	1616	417	2	391	0.0	585
ABCDEF_31746	UniRef50_Q9ZP05	0.0	Normal: 611	6985	354	98	71	19818	BlastAnno: 1631	UniRef50_Q9ZP05	354	83.38	349	54	2	274	1317	9	354	0.0	578
ABCDEF_32230	UniRef50_P57106	0.0	Normal: 8408	4627	7877	10286	18779	11078	BlastAnno: 1523	UniRef50_P57106	332	93.07	332	23	0	262	1257	1	332	0.0	617
ABCDEF_57415	UniRef50_Q9ZP05	0.0	Normal: 403	336	343	761	845	611	BlastAnno: 1190	UniRef50_Q9ZP05	354	83.29	353	54	2	1058	3	1	349	0.0	579
ABCDEF_17941	UniRef50_Q9ZP05	0.0	Normal: 616	554	586	1025	1231	910	BlastAnno: 1567	UniRef50_Q9ZP05	354	83.63	342	51	2	1336	314	17	354	0.0	563
1.1.1.38(malate dehydrogenase (oxaloacetate-decarboxylating);)																					
ABCDEF_4172	UniRef50_Q99KE1	0.0	Normal: 3896	8020	3800	3763	4801	9522	BlastAnno: 2536	UniRef50_Q99KE1	589	50.63	559	264	5	2021	375	8	564	0.0	559
1.1.1.39(malate dehydrogenase (decarboxylating);)																					
ABCDEF_76313	UniRef50_P37221	0.0	Normal: 942	772	1313	2259	2342	1405	BlastAnno: 2331	UniRef50_P37221	626	65.87	583	193	1	2153	423	42	624	0.0	661
ABCDEF_11069	UniRef50_P37221	0.0	Normal: 913	1050	959	1783	2411	1423	BlastAnno: 2200	UniRef50_P37221	626	87.90	628	73	2	2190	310	1	626	0.0	1046
ABCDEF_11068	UniRef50_P37221	0.0	Normal: 894	1051	947	1766	2300	1417	BlastAnno: 2244	UniRef50_P37221	626	87.90	628	73	2	2158	278	1	626	0.0	1045
ABCDEF_35547	UniRef50_P37221	0.0	Normal: 1071	798	1206	2226	2090	1293	BlastAnno: 2532	UniRef50_P37221	626	66.04	583	192	1	399	2129	42	624	0.0	661
1.1.1.40(malate dehydrogenase (oxaloacetate-decarboxylating) (NADP+);)																					
ABCDEF_7664	UniRef50_P43279	0.0	Normal: 1619	1000	1705	548	2995	3374	BlastAnno: 2126	UniRef50_P43279	639	78.41	565	117	1	131	1825	74	633	0.0	879
ABCDEF_24790	UniRef50_P43279	0.0	Normal: 452	150	359	466	361	366	BlastAnno: 1480	UniRef50_P43279	639	78.02	373	75	2	1121	3	58	423	0.0	567
ABCDEF_4172	UniRef50_Q16798	0.0	Normal: 3896	8020	3800	3763	4801	9522	BlastAnno: 2536	UniRef50_Q16798	604	50.56	540	264	3	1988	372	50	587	0.0	556
ABCDEF_4544	UniRef50_P43279	0.0	Normal: 12	3	39	281	184	0	BlastAnno: 1263	UniRef50_P43279	639	78.18	417	91	0	2	1252	223	639	0.0	660
1.1.1.77(lactaldehyde reductase;)																					
No Correspond Unigene																					
1.1.1.78(methylglyoxal reductase (NADH-dependent);)																					
No Correspond Unigene																					
1.1.1.79(glyoxylate reductase (NADP+);)																					
No Correspond Unigene																					
1.1.1.82(malate dehydrogenase (NADP+);)																					
ABCDEF_11352	UniRef50_P37229	0.0	Normal: 857	3023	663	550	982	9189	BlastAnno: 1914	UniRef50_P37229	432	83.25	400	64	2	371	1561	32	431	0.0	661
1.1.2.3(L-lactate dehydrogenase (cytochrome);)																					
ABCDEF_10989	UniRef50_P09437	8e-08	Normal: 7	13	7	12	9	21	BlastAnno: 545	UniRef50_P09437	573	35.56	90	50	1	497	228	191	272	8e-08	57.0
1.1.2.4(D-lactate dehydrogenase (cytochrome);)																					
ABCDEF_41186	UniRef50_Q94AX4	0.0	Normal: 168	194	166	266	369	409	BlastAnno: 2077	UniRef50_Q94AX4	567	84.20	481	76	0	1691	249	87	567	0.0	875
ABCDEF_20521	UniRef50_P46681	0.0	Normal: 406	283	302	461	568	486	BlastAnno: 2144	UniRef50_P46681	530	53.41	498	217	5	324	1778	35	530	0.0	535
1.1.2.5(D-lactate dehydrogenase (cytochrome c-553))																					
No Correspond Unigene																					
1.1.3.3(malate oxidase;)																					
No Correspond Unigene																					
1.1.5.4(malate dehydrogenase (quinone);)																					
No Correspond Unigene																					
1.1.99.7(lactate---malate transhydrogenase;)																					
No Correspond Unigene																					
1.13.12.4(lactate 2-monooxygenase;)																					
No Correspond Unigene																					
1.2.1.10(acetaldehyde dehydrogenase (acetylating);)																					
No Correspond Unigene																					
1.2.1.22(lactaldehyde dehydrogenase;)																					
No Correspond Unigene																					
1.2.1.23(2-oxoaldehyde dehydrogenase (NAD+);)																					
No Correspond Unigene																					
1.2.1.3(aldehyde dehydrogenase (NAD+);)																					
ABCDEF_52483	UniRef50_Q70E96	0.0	Normal: 28	851	41	0	3965	420	BlastAnno: 1750	UniRef50_Q70E96	484	54.56	482	219	0	107	1552	3	484	0.0	548
ABCDEF_17638	UniRef50_Q70E96	0.0	Normal: 580	718	749	2424	1317	775	BlastAnno: 1970	UniRef50_Q70E96	484	62.21	471	177	1	283	1692	2	472	0.0	625
ABCDEF_403	UniRef50_Q8W033	0.0	Normal: 69	355	98	39	223	1111	BlastAnno: 1749	UniRef50_Q8W033	550	65.96	473	161	0	1523	105	74	546	0.0	625
ABCDEF_45143	UniRef50_Q0WSF1	0.0	Normal: 1445	874	1200	1789	1940	1504	BlastAnno: 2450	UniRef50_Q0WSF1	596	79.97	594	115	2	2220	451	1	594	0.0	1015
1.2.1.49(2-oxoaldehyde dehydrogenase (NADP+);)																					
No Correspond Unigene																					
1.2.3.3(pyruvate oxidase;)																					
No Correspond Unigene																					
1.2.3.6(pyruvate oxidase (CoA-acetylating))																					
No Correspond Unigene																					
1.2.4.1(pyruvate dehydrogenase (acetyl-transferring);)																					
ABCDEF_28990	UniRef50_Q8H1Y0	0.0	Normal: 569	351	802	1550	2572	992	BlastAnno: 1596	UniRef50_Q8H1Y0	393	80.62	356	69	0	1369	302	38	393	0.0	628
ABCDEF_11397	UniRef50_O24458	0.0	Normal: 1628	1528	1619	3502	3214	2274	BlastAnno: 1766	UniRef50_O24458	406	82.74	394	58	4	1349	180	19	406	0.0	610
ABCDEF_35811	UniRef50_Q8H1Y0	0.0	Normal: 1865	1480	2772	5588	6415	2489	BlastAnno: 1647	UniRef50_Q8H1Y0	393	77.66	394	83	3	138	1307	1	393	0.0	621
ABCDEF_15406	UniRef50_O24458	0.0	Normal: 297	358	305	389	271	1259	BlastAnno: 1486	UniRef50_O24458	406	80.73	410	72	4	19	1239	1	406	0.0	647
ABCDEF_49154	UniRef50_O24458	0.0	Normal: 304	369	317	391	293	1358	BlastAnno: 1412	UniRef50_O24458	406	80.73	410	72	4	1394	174	1	406	0.0	647
ABCDEF_63617	UniRef50_O24458	0.0	Normal: 518	490	543	916	834	1436	BlastAnno: 1398	UniRef50_O24458	406	80.68	409	72	4	1397	180	2	406	0.0	618
1.2.5.1(pyruvate dehydrogenase (quinone);)																					
No Correspond Unigene																					
1.2.7.1(pyruvate synthase;)																					
No Correspond Unigene																					
1.2.99.3(aldehyde dehydrogenase (pyrroloquinoline-quinone);)																					
ABCDEF_25501	UniRef50_A5EIC3	1e-152	Normal: 1154	1584	1099	1850	736	3942	BlastAnno: 2012	UniRef50_A5EIC3	508	50.11	473	234	2	1698	280	32	502	1e-152	461

1.2.99.6(carboxylate reductase;)																					
No Correspond Unigene																					
1.8.1.4(dihydrolipoyl dehydrogenase;)																					
ABCDEF_573	UniRef50_P72740	0.0	Normal: 1422	809	1248	2548	1822	4200	BlastAnno: 2189	UniRef50_P72740	474	61.95	473	173	5	469	1878	5	473	0.0	556
2.3.1.12(dihydrolipoyllysine-residue acetyltransferase;)																					
ABCDEF_52034	UniRef50_Q0WQF7	0.0	Normal: 759	582	1123	2258	2501	768	BlastAnno: 2694	UniRef50_Q0WQF7	637	64.17	642	208	9	254	2146	1	631	0.0	743
ABCDEF_14485	UniRef50_Q5M729	0.0	Normal: 1032	504	1377	2545	3005	1098	BlastAnno: 2220	UniRef50_Q5M729	539	67.09	556	159	8	2092	446	1	539	0.0	719
ABCDEF_7230	UniRef50_Q5M729	0.0	Normal: 1333	844	1864	3619	4766	1386	BlastAnno: 2252	UniRef50_Q5M729	539	65.23	558	169	7	2120	465	1	539	0.0	702
2.3.1.54(formate C-acetyltransferase;)																					
No Correspond Unigene																					
2.3.1.8(phosphate acetyltransferase;)																					
ABCDEF_10009	UniRef50_B6KAY1	6e-23	Normal: 14	16	19	16	34	38	BlastAnno: 693	UniRef50_B6KAY1	1296	48.51	101	51	1	154	453	1128	1228	6e-23	104
2.3.1.9(acetyl-CoA C-acetyltransferase;)																					
ABCDEF_34825	UniRef50_Q8S4Y1	0.0	Normal: 640	698	620	807	839	1164	BlastAnno: 1773	UniRef50_Q8S4Y1	403	78.11	402	87	1	1597	395	2	403	0.0	598
ABCDEF_14629	UniRef50_Q8S4Y1	0.0	Normal: 634	316	650	1021	1278	691	BlastAnno: 1514	UniRef50_Q8S4Y1	403	85.11	403	60	0	1367	159	1	403	0.0	620
ABCDEF_107	UniRef50_Q8S4Y1	0.0	Normal: 332	460	460	842	893	424	BlastAnno: 1214	UniRef50_Q8S4Y1	403	84.07	339	54	0	1212	196	42	380	0.0	596
ABCDEF_14630	UniRef50_Q8S4Y1	0.0	Normal: 562	282	568	959	1173	627	BlastAnno: 1796	UniRef50_Q8S4Y1	403	85.50	400	58	0	436	1635	4	403	0.0	642
2.3.3.13(2-isopropylmalate synthase;)																					
ABCDEF_4613	UniRef50_Q9C550	0.0	Normal: 1859	1016	1479	1319	1219	3623	BlastAnno: 2304	UniRef50_Q9C550	631	78.77	551	115	1	2004	358	72	622	0.0	921
ABCDEF_7468	UniRef50_Q9C550	0.0	Normal: 1169	166	1015	479	1088	334	BlastAnno: 1883	UniRef50_Q9C550	631	75.86	526	123	2	1881	316	97	622	0.0	836
2.3.3.14(homocitrate synthase;)																					
No Correspond Unigene																					
2.3.3.6(2-ethylmalate synthase;)																					
No Correspond Unigene																					
2.3.3.9(malate synthase;)																					
ABCDEF_16096	UniRef50_P08216	1e-61	Normal: 12	0	12	0	0	26	BlastAnno: 316	UniRef50_P08216	568	89.52	105	11	0	2	316	237	341	1e-61	204
2.7.1.40(pyruvate kinase;)																					
ABCDEF_27872	UniRef50_Q40545	0.0	Normal: 1220	1217	1378	1640	1974	4469	BlastAnno: 2233	UniRef50_Q40545	593	81.65	594	96	4	1951	206	1	593	0.0	979
ABCDEF_34901	UniRef50_B9EYF5	0.0	Normal: 3919	1884	3179	3852	4333	5138	BlastAnno: 2384	UniRef50_B9EYF5	606	80.36	555	84	2	2021	432	52	606	0.0	922
ABCDEF_10813	UniRef50_Q2QXR7	0.0	Normal: 2566	2379	2864	4497	6222	4202	BlastAnno: 2019	UniRef50_Q2QXR7	408	85.57	402	58	0	174	1379	1	402	0.0	727
ABCDEF_35590	UniRef50_Q9M057	0.0	Normal: 2212	2122	2749	5601	7830	2637	BlastAnno: 1980	UniRef50_Q9M057	510	69.58	503	148	4	1830	334	1	502	0.0	687
ABCDEF_27885	UniRef50_Q2QXR7	0.0	Normal: 924	752	1120	2229	3166	928	BlastAnno: 2102	UniRef50_Q2QXR7	408	87.31	402	51	0	182	1387	1	402	0.0	729
ABCDEF_21818	UniRef50_Q40545	0.0	Normal: 1982	1309	1439	2275	1999	2303	BlastAnno: 2062	UniRef50_Q40545	593	69.25	491	147	2	1594	134	103	593	0.0	716
ABCDEF_31300	UniRef50_Q40545	0.0	Normal: 1326	1019	1016	1433	1396	1865	BlastAnno: 1805	UniRef50_Q40545	593	91.70	494	41	0	1802	321	100	593	0.0	920
ABCDEF_45529	UniRef50_B9EYF5	0.0	Normal: 1474	625	1452	3814	2571	3121	BlastAnno: 2114	UniRef50_B9EYF5	606	78.56	569	89	3	1810	179	46	606	0.0	912
ABCDEF_45536	UniRef50_Q2QXR7	0.0	Normal: 1644	1099	1596	1290	5936	335	BlastAnno: 1959	UniRef50_Q2QXR7	408	81.59	402	74	0	1862	657	1	402	0.0	687
ABCDEF_27886	UniRef50_Q2QXR7	0.0	Normal: 771	640	954	1807	2731	814	BlastAnno: 1787	UniRef50_Q2QXR7	408	86.82	349	46	0	54	1100	54	402	0.0	632
ABCDEF_18187	UniRef50_Q9M057	0.0	Normal: 3137	1498	3397	5489	6703	1765	BlastAnno: 1912	UniRef50_Q9M057	510	67.21	494	158	3	216	1685	17	510	0.0	681
ABCDEF_45028	UniRef50_B9EYF5	0.0	Normal: 273	415	192	360	372	144	BlastAnno: 1785	UniRef50_B9EYF5	606	73.94	495	105	2	372	1784	111	605	0.0	729
2.7.2.1(acetate kinase;)																					
No Correspond Unigene																					
2.7.2.12(acetate kinase (diphosphate);)																					
No Correspond Unigene																					
2.7.9.1(pyruvate, phosphate dikinase;)																					
ABCDEF_21316	UniRef50_B9RSV8	0.0	Normal: 3	411	12	10	27	459	BlastAnno: 1538	UniRef50_B9RSV8	872	87.65	421	49	1	284	1537	68	488	0.0	774
ABCDEF_20732	UniRef50_A9F4I0	0.0	Normal: 9	376	9	13	38	528	BlastAnno: 1760	UniRef50_A9F4I0	920	59.76	497	187	5	1760	294	407	898	0.0	588
ABCDEF_20731	UniRef50_O23404	0.0	Normal: 14	241	6	10	27	401	BlastAnno: 1677	UniRef50_O23404	963	84.32	338	52	1	401	1414	626	962	0.0	604
2.7.9.2(pyruvate, water dikinase;)																					
No Correspond Unigene																					
2.8.3.1(propionate CoA-transferase;)																					
No Correspond Unigene																					
3.1.2.1(acetyl-CoA hydrolase;)																					
No Correspond Unigene																					
3.1.2.6(hydroxyacylglutathione hydrolase;)																					
ABCDEF_48924	UniRef50_O24495	4e-157	Normal: 309	400	333	553	644	844	BlastAnno: 1266	UniRef50_O24495	331	70.44	318	93	1	111	1061	14	331	4e-157	456
3.6.1.7(acylphosphatase;)																					
ABCDEF_18227	UniRef50_C5Z842	3e-28	Normal: 191	173	224	225	257	298	BlastAnno: 956	UniRef50_C5Z842	142	62.37	93	33	2	573	301	50	142	3e-28	114
4.1.1.-(Lyases;)																					
ABCDEF_57496	UniRef50_O96569	9e-20	Normal: 4	0	0	0	0	9	BlastAnno: 223	UniRef50_O96569	439	66.67	72	22	1	210	1	19	90	9e-20	87.4
4.1.1.3(oxaloacetate decarboxylase;)																					
No Correspond Unigene																					
4.1.1.31(phosphoenolpyruvate carboxylase;)																					
ABCDEF_64086	UniRef50_Q6R2V6	0.0	Normal: 363	569	289	463	784	348	BlastAnno: 2123	UniRef50_Q6R2V6	1221	61.79	560	205	4	163	1821	664	1221	0.0	650
ABCDEF_41385	UniRef50_B5FPX1	0.0	Normal: 4314	701	3865	3687	4278	3624	BlastAnno: 3374	UniRef50_B5FPX1	883	40.60	941	490	14	3058	260	4	883	0.0	634
ABCDEF_62655	UniRef50_Q6R2V6	0.0	Normal: 335	542	261	432	710	315	BlastAnno: 2054	UniRef50_Q6R2V6	1221	61.79	560	205	4	163	1821	664	1221	0.0	650
ABCDEF_5143	UniRef50_Q6R2V6	0.0	Normal: 847	1445	623	1238	1720	785	BlastAnno: 3592	UniRef50_Q6R2V6	1221	57.46	637	243	6	1451	3292	590	1221	0.0	661
ABCDEF_34325	UniRef50_B5FPX1	0.0	Normal: 1019	396	1067	2133	3237	977	BlastAnno: 3428	UniRef50_B5FPX1	883	41.15	943	483	13	3011	210	4	883	0.0	656
ABCDEF_49210	UniRef50_Q6R2V6	0.0	Normal: 884	1485	676	1279	1833	822	BlastAnno: 3747	UniRef50_Q6R2V6	1221	57.46	637	243	6	2261	420	590	1221	0.0	661
ABCDEF_34323	UniRef50_B5FPX1	0.0	Normal: 1026	368	1024	2181	3307	986	BlastAnno: 3450	UniRef50_B5FPX1	883	41.15	943	483	13	440	3241	4	883	0.0	656
ABCDEF_13966	UniRef50_P15804	0.0	Normal: 86	248	71	71	214	123	BlastAnno: 2130	UniRef50_P15804	960	74.85	672	161	4	2130	133	291	960	0.0	1010
ABCDEF_13967	UniRef50_P15804	0.0	Normal: 75	236	61	55	191	97	BlastAnno: 2000	UniRef50_P15804	960	74.96	619	149	3	28	1872	344	960	0.0	925
4.1.1.32(phosphoenolpyruvate carboxykinase (GTP);)																					

No Correspond Unigene

4.1.1.38(phosphoenolpyruvate carboxykinase (diphosphate);)

No Correspond Unigene

4.1.1.49(phosphoenolpyruvate carboxykinase (ATP);)

ABCDEF_2793	UniRef50_P10963	0.0	Normal: 99	228	79	1160	39	318	BlastAnno: 2459	UniRef50_P10963	549	52.91	567	219	11	402	2084	24	548	0.0	564
ABCDEF_81273	UniRef50_Q9T074	0.0	Normal: 86	204	69	1021	35	282	BlastAnno: 2611	UniRef50_Q9T074	671	76.69	369	78	5	2606	1512	8	372	0.0	565
ABCDEF_81242	UniRef50_Q9T074	0.0	Normal: 95	225	76	1100	40	293	BlastAnno: 2538	UniRef50_Q9T074	671	75.83	480	102	7	2533	1124	8	483	0.0	693
ABCDEF_2794	UniRef50_Q9T074	0.0	Normal: 114	289	99	1477	44	337	BlastAnno: 2733	UniRef50_Q9T074	671	76.69	369	78	5	2614	1520	8	372	0.0	565
ABCDEF_2796	UniRef50_Q9T074	0.0	Normal: 127	322	105	1687	47	387	BlastAnno: 2614	UniRef50_Q9T074	671	90.23	399	39	0	1572	376	273	671	0.0	738
ABCDEF_85588	UniRef50_Q9T074	0.0	Normal: 41	99	29	443	15	186	BlastAnno: 2024	UniRef50_Q9T074	671	80.55	329	35	1	1362	376	372	671	0.0	550
ABCDEF_79512	UniRef50_Q9T074	0.0	Normal: 104	244	82	1189	43	318	BlastAnno: 2451	UniRef50_Q9T074	671	75.83	480	102	7	2446	1037	8	483	0.0	693
ABCDEF_2797	UniRef50_P10963	0.0	Normal: 136	339	120	1738	54	398	BlastAnno: 2486	UniRef50_P10963	549	55.58	538	220	10	1971	376	24	548	0.0	576
ABCDEF_89373	UniRef50_Q9T074	0.0	Normal: 55	130	40	611	21	222	BlastAnno: 1864	UniRef50_Q9T074	671	84.11	428	39	1	1659	376	273	671	0.0	721
ABCDEF_2795	UniRef50_Q9T074	0.0	Normal: 49	120	40	536	20	212	BlastAnno: 1946	UniRef50_Q9T074	671	88.33	300	35	0	1275	376	372	671	0.0	565
ABCDEF_2792	UniRef50_Q9T074	0.0	Normal: 88	218	76	1085	36	308	BlastAnno: 2532	UniRef50_Q9T074	671	88.33	300	35	0	1258	2157	372	671	0.0	565

4.1.1.78(acetylenedicarboxylate decarboxylase;)

No Correspond Unigene

4.1.2.36(lactate aldolase;)

No Correspond Unigene

4.1.3.-(Lyases;)

ABCDEF_21016	UniRef50_C5E1L7	1e-173	Normal: 775	334	669	635	559	950	BlastAnno: 2086	UniRef50_C5E1L7	551	49.91	549	253	8	1823	234	2	547	1e-173	517

4.2.3.3(methylglyoxal synthase;)

No Correspond Unigene

4.4.1.5(lactoylglutathione lyase;)

ABCDEF_32167	UniRef50_Q948T6	4e-164	Normal: 2749	2303	2616	4332	6488	4931	BlastAnno: 1276	UniRef50_Q948T6	291	77.66	291	64	1	1190	321	1	291	4e-164	472

5.1.2.1(lactate racemase;)

No Correspond Unigene

6.2.1.1(acetate---CoA ligase;)

ABCDEF_65173	UniRef50_Q9VP61	0.0	Normal: 208	202	219	336	383	409	BlastAnno: 2249	UniRef50_Q9VP61	670	54.63	540	236	3	539	2131	4	543	0.0	629
ABCDEF_51889	UniRef50_Q9VP61	0.0	Normal: 268	268	315	436	574	651	BlastAnno: 2148	UniRef50_Q9VP61	670	55.64	638	273	4	2060	174	26	662	0.0	723
ABCDEF_51888	UniRef50_A8JFR9	0.0	Normal: 348	343	408	583	709	775	BlastAnno: 2704	UniRef50_A8JFR9	667	56.57	647	273	5	2090	168	20	664	0.0	712
ABCDEF_59631	UniRef50_Q9VP61	0.0	Normal: 132	127	125	187	249	287	BlastAnno: 1733	UniRef50_Q9VP61	670	55.41	518	222	3	89	1615	26	543	0.0	610

6.2.1.13(acetate---CoA ligase (ADP-forming);)

No Correspond Unigene

6.4.1.1(pyruvate carboxylase;)

ABCDEF_45829	UniRef50_O27939	4e-167	Normal: 2364	1703	2036	5443	4075	6768	BlastAnno: 2081	UniRef50_O27939	491	54.80	469	207	2	396	1790	4	471	4e-167	498

6.4.1.2(acetyl-CoA carboxylase;)

ABCDEF_27465	UniRef50_P78820	0.0	Normal: 4589	3602	6382	22310	7761	4482	BlastAnno: 7477	UniRef50_P78820	2280	40.33	2145	1127	43	7128	919	55	2121	0.0	1462

>00621 Dioxin degradation

1.13.11.39(biphenyl-2,3-diol 1,2-dioxygenase;)

No Correspond Unigene

1.14.12.18(biphenyl 2,3-dioxygenase;)

No Correspond Unigene

1.14.13.1(salicylate 1-monooxygenase;)

No Correspond Unigene

1.2.1.10(acetaldehyde dehydrogenase (acetylating);)

No Correspond Unigene

1.3.1.56(cis-2,3-dihydrobiphenyl-2,3-diol dehydrogenase;)

No Correspond Unigene

3.7.1.8(2,6-dioxo-6-phenylhexa-3-enoate hydrolase;)

No Correspond Unigene

4.1.1.-(Lyases;)

ABCDEF_57496	UniRef50_O96569	9e-20	Normal: 4	0	0	0	0	9	BlastAnno: 223	UniRef50_O96569	439	66.67	72	22	1	210	1	19	90	9e-20	87.4

4.1.1.77(4-oxalocrotonate decarboxylase;)

No Correspond Unigene

4.1.3.39(4-hydroxy-2-oxovalerate aldolase;)

No Correspond Unigene

4.2.1.80(2-oxopent-4-enoate hydratase;)

No Correspond Unigene

5.3.2.-(Isomerases;)

No Correspond Unigene

>00622 Xylene degradation

1.1.1.-(Oxidoreductases;)

ABCDEF_13453	UniRef50_Q9SYM5	0.0	Normal: 225	226	398	1950	2026	421	BlastAnno: 2578	UniRef50_Q9SYM5	669	86.61	672	87	2	298	2313	1	669	0.0	1226
ABCDEF_17342	UniRef50_Q84M96	0.0	Normal: 274	406	272	327	439	625	BlastAnno: 1277	UniRef50_Q84M96	346	73.33	345	91	1	1187	156	2	346	0.0	546
ABCDEF_31147	UniRef50_Q9SYM5	0.0	Normal: 4571	1591	1491	1401	1939	4327	BlastAnno: 2465	UniRef50_Q9SYM5	669	82.76	673	107	4	2397	394	1	669	0.0	1154
ABCDEF_17344	UniRef50_Q84M96	0.0	Normal: 252	373	257	297	380	540	BlastAnno: 1350	UniRef50_Q84M96	346	68.19	371	91	2	1263	154	2	346	0.0	530
ABCDEF_44674	UniRef50_Q9SYM5	0.0	Normal: 3581	2620	4329	9505	11447	4930	BlastAnno: 2634	UniRef50_Q9SYM5	669	87.80	672	78	3	253	2265	1	669	0.0	1241

1.1.1.90(aryl-alcohol dehydrogenase;)

No Correspond Unigene

1.13.11.-(Oxidoreductases;)

No Correspond Unigene

1.13.11.2(catechol 2,3-dioxygenase;)
No Correspond Unigene
1.14.12.-(Oxidoreductases;)
No Correspond Unigene
1.14.13.-(Oxidoreductases;)
ABCDEF_45538 UniRef50_O23024 3e-156 Normal: 110 6 165 774 133 36 BlastAnno: 1258 UniRef50_O23024 437 58.18 385 151 3 3 1157 37 411 3e-156 457
1.2.1.10(acetaldehyde dehydrogenase (acetylating);)
No Correspond Unigene
1.2.1.28(benzaldehyde dehydrogenase (NAD+);)
No Correspond Unigene
1.2.1.29(aryl-aldehyde dehydrogenase)
No Correspond Unigene
1.2.1.32(aminomuconate-semialdehyde dehydrogenase;)
No Correspond Unigene
1.2.1.7(benzaldehyde dehydrogenase (NADP+);)
No Correspond Unigene
1.3.1.-(Oxidoreductases;)
ABCDEF_45552 UniRef50_P52581 0.0 Normal: 213 2112 262 1058 1208 1322 BlastAnno: 1284 UniRef50_P52581 312 80.45 312 61 0 185 1120 1 312 0.0 530
ABCDEF_47970 UniRef50_Q9T0J6 0.0 Normal: 1040 443 1171 941 1234 650 BlastAnno: 2557 UniRef50_Q9T0J6 700 60.52 699 232 9 2337 271 36 700 0.0 860
1.3.1.58(2,3-dihydroxy-2,3-dihydro-p-cumate dehydrogenase)
No Correspond Unigene
1.3.1.67(cis-1,2-dihydroxy-4-methylcyclohexa-3,5-diene-1-carboxylate dehydrogenase)
No Correspond Unigene
1.3.1.68(1,2-dihydroxy-6-methylcyclohexa-3,5-dienecarboxylate dehydrogenase)
No Correspond Unigene
3.7.1.-(Hydrolases;)
No Correspond Unigene
3.7.1.9(2-hydroxymuconate-semialdehyde hydrolase;)
No Correspond Unigene
4.1.1.-(Lyases;)
ABCDEF_57496 UniRef50_O96569 9e-20 Normal: 4 0 0 0 0 9 BlastAnno: 223 UniRef50_O96569 439 66.67 72 22 1 210 1 19 90 9e-20 87.4
4.1.1.77(4-oxalocrotonate decarboxylase;)
No Correspond Unigene
4.1.3.39(4-hydroxy-2-oxovalerate aldolase;)
No Correspond Unigene
4.2.1.80(2-oxopent-4-enoate hydratase;)
No Correspond Unigene
>00623 Toluene degradation
1.1.1.257(4-(hydroxymethyl)benzenesulfonate dehydrogenase)
No Correspond Unigene
1.1.1.35(3-hydroxyacyl-CoA dehydrogenase;)
ABCDEF_80649 UniRef50_Q1LD07 5e-120 Normal: 527 841 443 1308 1321 1188 BlastAnno: 2586 UniRef50_Q1LD07 715 37.34 699 420 9 2358 277 22 707 5e-120 389
1.1.1.90(aryl-alcohol dehydrogenase;)
No Correspond Unigene
1.1.1.97(3-hydroxybenzyl-alcohol dehydrogenase;)
No Correspond Unigene
1.13.11.-(Oxidoreductases;)
No Correspond Unigene
1.13.11.1(catechol 1,2-dioxygenase;)
No Correspond Unigene
1.14.12.-(Oxidoreductases;)
No Correspond Unigene
1.14.12.11(toluene dioxygenase;)
No Correspond Unigene
1.14.12.8(4-sulfobenzoate 3,4-dioxygenase;)
No Correspond Unigene
1.14.13.-(Oxidoreductases;)
ABCDEF_45538 UniRef50_O23024 3e-156 Normal: 110 6 165 774 133 36 BlastAnno: 1258 UniRef50_O23024 437 58.18 385 151 3 3 1157 37 411 3e-156 457
1.14.13.7(phenol 2-monooxygenase;)
No Correspond Unigene
1.17.99.1(4-cresol dehydrogenase (hydroxylating);)
No Correspond Unigene
1.2.1.28(benzaldehyde dehydrogenase (NAD+);)
No Correspond Unigene
1.2.1.62(4-formylbenzenesulfonate dehydrogenase)
No Correspond Unigene
1.2.1.64(4-hydroxybenzaldehyde dehydrogenase;)
No Correspond Unigene
1.2.1.7(benzaldehyde dehydrogenase (NADP+);)
No Correspond Unigene
1.3.1.-(Oxidoreductases;)
ABCDEF_45552 UniRef50_P52581 0.0 Normal: 213 2112 262 1058 1208 1322 BlastAnno: 1284 UniRef50_P52581 312 80.45 312 61 0 185 1120 1 312 0.0 530

	ABCDEF_47970	UniRef50_Q9T0J6	0.0	Normal: 1040	443	1171	941	1234	650	BlastAnno: 2557	UniRef50_Q9T0J6	700	60.52	699	232	9	2337	271	36	700	0.0	860
1.3.1.19(cis-1,2-dihydrobenzene-1,2-diol dehydrogenase;)																						
	No Correspond Unigene																					
1.3.1.32(maleylacetate reductase;)																						
	No Correspond Unigene																					
1.3.99.1(succinate dehydrogenase;)																						
	ABCDEF_47748	UniRef50_Q6F8K9	0.0	Normal: 903	791	1034	2782	3604	958	BlastAnno: 2360	UniRef50_Q6F8K9	632	51.24	603	252	8	1990	290	36	632	0.0	584
	ABCDEF_47751	UniRef50_Q6F8K9	0.0	Normal: 776	655	916	2143	2907	810	BlastAnno: 2266	UniRef50_Q6F8K9	632	51.24	603	252	8	303	2003	36	632	0.0	585
	ABCDEF_47750	UniRef50_Q6F8K9	0.0	Normal: 1478	889	1772	2151	3099	1241	BlastAnno: 2236	UniRef50_Q6F8K9	632	51.00	602	255	7	1898	198	36	632	0.0	571
1.3.99.21(No Name)																						
	No Correspond Unigene																					
2.3.1.-(Transferases;)																						
	ABCDEF_520	UniRef50_Q9FF86	0.0	Normal: 1672	85	2273	5056	3383	1211	BlastAnno: 1811	UniRef50_Q9FF86	484	64.06	448	149	6	1664	354	3	449	0.0	573
	ABCDEF_68664	UniRef50_Q9ZVN2	0.0	Normal: 79	35	85	107	193	63	BlastAnno: 1488	UniRef50_Q9ZVN2	704	63.99	411	142	3	46	1275	298	703	0.0	540
	ABCDEF_52245	UniRef50_Q9FG87	0.0	Normal: 1878	635	1833	3814	1432	1376	BlastAnno: 1758	UniRef50_Q9FG87	529	55.35	486	204	4	4	1428	34	517	0.0	530
	ABCDEF_16730	UniRef50_Q3EC11	0.0	Normal: 281	204	237	365	377	458	BlastAnno: 2092	UniRef50_Q3EC11	536	59.33	536	204	8	196	1785	3	530	0.0	661
	ABCDEF_4655	UniRef50_A5BMG1	0.0	Normal: 2426	1856	3606	6958	4608	2755	BlastAnno: 2261	UniRef50_A5BMG1	481	68.37	528	112	10	263	1843	3	476	0.0	667
	ABCDEF_13860	UniRef50_Q9C533	0.0	Normal: 588	220	570	1451	1338	310	BlastAnno: 2648	UniRef50_Q9C533	596	63.37	606	177	12	2226	511	1	595	0.0	558
	ABCDEF_28294	UniRef50_Q9SYZ0	0.0	Normal: 1038	4070	1427	6745	877	2797	BlastAnno: 1785	UniRef50_Q9SYZ0	493	52.37	485	223	3	1598	162	5	487	0.0	533
	ABCDEF_47718	UniRef50_Q9C533	0.0	Normal: 1599	236	1657	2093	1231	588	BlastAnno: 2680	UniRef50_Q9C533	596	65.02	626	165	14	2280	472	1	595	0.0	634
	ABCDEF_45080	UniRef50_Q9SYZ0	0.0	Normal: 1005	592	1079	2360	3762	2217	BlastAnno: 2130	UniRef50_Q9SYZ0	493	57.85	484	197	3	302	1735	5	487	0.0	533
	ABCDEF_25377	UniRef50_Q9C6L5	0.0	Normal: 37	2090	138	2573	1026	1298	BlastAnno: 1334	UniRef50_Q9C6L5	492	79.74	385	76	1	1155	1	6	388	0.0	654
	ABCDEF_33470	UniRef50_Q9LW26	0.0	Normal: 172	98	156	260	391	149	BlastAnno: 2993	UniRef50_Q9LW26	701	48.96	627	301	5	655	2508	85	701	0.0	581
	ABCDEF_40654	UniRef50_Q9H0A0	0.0	Normal: 638	213	530	318	318	286	BlastAnno: 2216	UniRef50_Q9H0A0	1025	56.87	721	279	6	2127	13	91	795	0.0	756
	ABCDEF_33460	UniRef50_Q9ZVN2	0.0	Normal: 144	71	119	216	306	97	BlastAnno: 2691	UniRef50_Q9ZVN2	704	61.50	587	191	4	379	2133	83	636	0.0	715
	ABCDEF_14442	UniRef50_Q9SYZ0	0.0	Normal: 341	684	439	1040	1601	552	BlastAnno: 1779	UniRef50_Q9SYZ0	493	63.12	480	171	2	1570	149	5	484	0.0	628
	ABCDEF_52626	UniRef50_B6SYP9	0.0	Normal: 815	346	1131	3015	1537	108	BlastAnno: 1861	UniRef50_B6SYP9	494	55.02	438	189	3	1538	249	51	488	0.0	536
	ABCDEF_644	UniRef50_Q9SB58	0.0	Normal: 704	444	913	969	1431	848	BlastAnno: 1777	UniRef50_Q9SB58	407	64.15	424	129	8	1572	316	1	406	0.0	554
	ABCDEF_27087	UniRef50_Q52T38	0.0	Normal: 324	221	233	419	343	412	BlastAnno: 1631	UniRef50_Q52T38	620	75.95	420	91	1	1409	150	1	410	0.0	681
	ABCDEF_34187	UniRef50_Q8L5Y5	0.0	Normal: 497	415	588	523	291	433	BlastAnno: 2677	UniRef50_Q8L5Y5	718	57.30	733	251	15	202	2274	1	713	0.0	683
	ABCDEF_47966	UniRef50_Q9SYZ0	0.0	Normal: 577	934	326	580	682	3440	BlastAnno: 2177	UniRef50_Q9SYZ0	493	56.40	484	204	3	1780	347	5	487	0.0	535
	ABCDEF_51189	UniRef50_Q52T38	0.0	Normal: 135	152	128	181	219	327	BlastAnno: 1766	UniRef50_Q52T38	620	68.09	470	137	3	80	1483	130	588	0.0	641
	ABCDEF_41288	UniRef50_Q9SB58	0.0	Normal: 307	183	332	637	649	322	BlastAnno: 1403	UniRef50_Q9SB58	407	67.56	410	110	6	1401	184	15	405	0.0	523
	ABCDEF_33461	UniRef50_Q9ZVN2	0.0	Normal: 142	84	140	206	283	119	BlastAnno: 2904	UniRef50_Q9ZVN2	704	60.98	574	201	6	2271	559	83	636	0.0	686
	ABCDEF_14334	UniRef50_Q9SYZ0	0.0	Normal: 2263	129	1724	3469	866	283	BlastAnno: 2039	UniRef50_Q9SYZ0	493	63.22	484	171	2	369	1802	5	487	0.0	627
	ABCDEF_24844	UniRef50_Q9FF86	0.0	Normal: 1857	3686	2657	21940	11467	4090	BlastAnno: 1640	UniRef50_Q9FF86	484	66.89	447	135	6	1492	185	2	446	0.0	575
	ABCDEF_51190	UniRef50_Q52T38	0.0	Normal: 145	169	142	210	232	335	BlastAnno: 1891	UniRef50_Q52T38	620	70.04	514	141	3	73	1608	86	588	0.0	726
	ABCDEF_14085	UniRef50_Q8L5Y5	0.0	Normal: 353	41	384	527	331	280	BlastAnno: 2362	UniRef50_Q8L5Y5	718	62.07	704	220	12	2360	348	24	713	0.0	676
	ABCDEF_33458	UniRef50_Q9LW26	0.0	Normal: 158	78	130	240	342	106	BlastAnno: 2822	UniRef50_Q9LW26	701	46.72	655	302	5	400	2337	85	701	0.0	568
	ABCDEF_25193	UniRef50_Q9C6L5	0.0	Normal: 3256	368	3548	6230	5109	1288	BlastAnno: 1786	UniRef50_Q9C6L5	492	65.83	480	156	4	1668	241	17	492	0.0	668
	ABCDEF_33457	UniRef50_Q9ZVN2	0.0	Normal: 168	83	137	250	375	112	BlastAnno: 2823	UniRef50_Q9ZVN2	704	65.12	496	168	3	379	1860	83	575	0.0	648
	ABCDEF_7634	UniRef50_Q9C6L5	0.0	Normal: 2543	9303	2711	11578	5533	10032	BlastAnno: 2064	UniRef50_Q9C6L5	492	52.76	489	225	3	1734	268	1	483	0.0	535
	ABCDEF_299	UniRef50_Q71N54	0.0	Normal: 569	1147	511	900	649	1879	BlastAnno: 2017	UniRef50_Q71N54	535	61.73	533	198	5	338	1924	4	534	0.0	677
	ABCDEF_27086	UniRef50_Q52T38	0.0	Normal: 784	470	617	843	893	976	BlastAnno: 2398	UniRef50_Q52T38	620	70.80	637	165	4	225	2129	1	618	0.0	887
	ABCDEF_51188	UniRef50_Q52T38	0.0	Normal: 273	327	255	415	349	441	BlastAnno: 2324	UniRef50_Q52T38	620	71.30	568	150	3	2041	344	32	588	0.0	815
	ABCDEF_44812	UniRef50_Q6DR03	0.0	Normal: 982	902	1005	1071	959	2999	BlastAnno: 2450	UniRef50_Q6DR03	565	54.97	644	198	17	2142	220	1	555	0.0	540
2.8.3.15(succinyl-CoA:(R)-benzylsuccinate CoA-transferase;)																						
	No Correspond Unigene																					
3.1.1.-(Hydrolases;)																						
	ABCDEF_25737	UniRef50_Q9CA68	0.0	Normal: 104	11	8	393	426	18	BlastAnno: 1232	UniRef50_Q9CA68	366	69.89	352	103	2	1182	130	17	366	0.0	522
	ABCDEF_525	UniRef50_Q9LIN2	0.0	Normal: 213	16	167	378	456	158	BlastAnno: 1254	UniRef50_Q9LIN2	380	69.27	358	110	0	1097	24	23	380	0.0	535
	ABCDEF_28091	UniRef50_O23522	0.0	Normal: 1192	678	424	81	156	3170	BlastAnno: 1887	UniRef50_O23522	601	57.90	525	191	12	168	1721	3	504	0.0	568
	ABCDEF_13432	UniRef50_Q3ECP6	0.0	Normal: 712	1725	466	588	811	6988	BlastAnno: 2479	UniRef50_Q3ECP6	408	66.83	401	104	2	2397	1282	8	408	0.0	567
	ABCDEF_72624	UniRef50_Q3ECP6	0.0	Normal: 22	38	25	267	382	149	BlastAnno: 1342	UniRef50_Q3ECP6	408	66.83	401	104	2	53	1168	8	408	0.0	567
	ABCDEF_4432	UniRef50_B9IQA0	0.0	Normal: 680	298	798	834	1447	644	BlastAnno: 1533	UniRef50_B9IQA0	354	78.82	321	68	0	214	1176	33	353	0.0	556
3.1.1.45(carboxymethylenebutenolidase;)																						
	ABCDEF_29224	UniRef50_B9SFS0	3e-139	Normal: 809	476	821	788	1383	993	BlastAnno: 1122	UniRef50_B9SFS0	286	77.42	248	55	1	177	917	39	286	3e-139	407
4.1.99.11(benzylsuccinate synthase;)																						
	No Correspond Unigene																					
4.2.1.-(Lyases;)																						
	ABCDEF_48679	UniRef50_Q0RP29	5e-115	Normal: 568	325	563	941	1285	676	BlastAnno: 1424	UniRef50_Q0RP29	493	56.39	321	129	4	119	1075	39	350	5e-115	356
5.4.99.14(4-carboxymethyl-4-methylbutenolide mutase;)																						
	No Correspond Unigene																					
5.5.1.-(Isomerases;)																						
	No Correspond Unigene																					
5.5.1.1(muconate cycloisomerase;)																						
	ABCDEF_21833	UniRef50_B9SQ11	6e-167	Normal: 262	353	289	450	785	549	BlastAnno: 1557	UniRef50_B9SQ11	453	64.00	375	133	2	1535	417	75	449	6e-167	489
5.5.1.7(chloromuconate cycloisomerase;)																						
	ABCDEF_7507	UniRef50_A6Q9H5	8e-08	Normal: 6	6	11	12	0	4	BlastAnno: 234	UniRef50_A6Q9H5	359	42.42	66	37	1	233	39	163	228	8e-08	53.9
>00624 Polycyclic aromatic hydrocarbon degradation																						
1.1.-.-(Oxidoreductases;)																						
	ABCDEF_17646	UniRef50_O34948	5e-83	Normal: 372	339	320	294	487	1376	BlastAnno: 1685	UniRef50_O34948	288	47.31	279	145	2	1523	690	5	282	5e-83	269

1.1.1.256(fluoren-9-ol dehydrogenase)
No Correspond Unigene
1.13.11.-(Oxidoreductases;)
No Correspond Unigene
1.13.11.3(protocatechuate 3,4-dioxygenase;)
No Correspond Unigene
1.13.11.38(1-hydroxy-2-naphthoate 1,2-dioxygenase;)
No Correspond Unigene
1.13.11.8(protocatechuate 4,5-dioxygenase;)
No Correspond Unigene
1.14.-.-(Oxidoreductases;)

ABCDEF_44739	UniRef50_O64989	0.0	Normal: 157	87	159	718	673	189	BlastAnno: 2185	UniRef50_O64989	513	70.75	482	112	3	1867	509	30	511	0.0	706
ABCDEF_31491	UniRef50_Q93VK5	0.0	Normal: 647	1826	671	1517	1770	5953	BlastAnno: 2278	UniRef50_Q93VK5	595	79.14	556	113	2	198	1862	35	588	0.0	902
ABCDEF_40554	UniRef50_P37122	0.0	Normal: 1574	1962	1829	1150	2882	2721	BlastAnno: 1828	UniRef50_P37122	505	60.52	504	198	1	1752	244	1	504	0.0	638
ABCDEF_34049	UniRef50_O23365	0.0	Normal: 363	384	349	442	549	1416	BlastAnno: 2101	UniRef50_O23365	580	76.17	579	116	5	230	1957	19	578	0.0	904
ABCDEF_21579	UniRef50_O48928	0.0	Normal: 1005	643	1606	4372	3226	1621	BlastAnno: 1895	UniRef50_O48928	513	69.72	502	151	1	185	1690	7	507	0.0	731
ABCDEF_37741	UniRef50_P37122	0.0	Normal: 12	760	9	24	34	576	BlastAnno: 1197	UniRef50_P37122	505	65.70	379	129	1	1195	59	127	504	0.0	526
ABCDEF_35618	UniRef50_O48928	0.0	Normal: 28	2648	17	142	1234	902	BlastAnno: 1746	UniRef50_O48928	513	66.40	506	168	2	1649	132	4	507	0.0	701
ABCDEF_24522	UniRef50_P37122	0.0	Normal: 334	2190	561	268	1391	2459	BlastAnno: 2075	UniRef50_P37122	505	61.52	460	176	1	1544	165	47	505	0.0	612
ABCDEF_14592	UniRef50_O23066	0.0	Normal: 1514	402	2245	7837	4742	1248	BlastAnno: 2378	UniRef50_O23066	553	72.71	546	140	4	2111	489	1	542	0.0	821
ABCDEF_14512	UniRef50_Q9FG65	0.0	Normal: 261	872	212	291	175	789	BlastAnno: 1878	UniRef50_Q9FG65	502	56.89	501	197	9	1738	266	11	502	0.0	532
ABCDEF_44487	UniRef50_Q940V4	0.0	Normal: 3	41	53	501	262	542	BlastAnno: 1712	UniRef50_Q940V4	465	69.37	382	116	1	38	1180	14	395	0.0	572
ABCDEF_17723	UniRef50_P37122	0.0	Normal: 240	1698	219	768	592	794	BlastAnno: 1757	UniRef50_P37122	505	63.40	459	166	2	228	1601	47	504	0.0	617
ABCDEF_37901	UniRef50_Q42569	0.0	Normal: 30	149	86	255	195	255	BlastAnno: 1246	UniRef50_Q42569	472	78.27	359	73	2	64	1140	43	396	0.0	567
ABCDEF_7260	UniRef50_O81117	0.0	Normal: 314	1085	466	3248	2575	1360	BlastAnno: 1768	UniRef50_O81117	514	59.92	474	188	1	145	1566	43	514	0.0	615
ABCDEF_40548	UniRef50_P37122	0.0	Normal: 2756	2467	2752	1072	3120	1735	BlastAnno: 1947	UniRef50_P37122	505	59.72	504	202	1	241	1749	1	504	0.0	636
ABCDEF_44485	UniRef50_Q940V4	0.0	Normal: 3	55	63	574	338	660	BlastAnno: 1607	UniRef50_Q940V4	465	68.58	452	141	1	42	1394	14	465	0.0	673
ABCDEF_40552	UniRef50_P37122	0.0	Normal: 2397	1870	2304	1249	10428	2291	BlastAnno: 1812	UniRef50_P37122	505	60.12	504	200	1	108	1616	1	504	0.0	640
ABCDEF_37902	UniRef50_Q42569	0.0	Normal: 84	524	254	725	670	1049	BlastAnno: 1812	UniRef50_Q42569	472	77.47	435	92	3	1675	371	43	471	0.0	681
ABCDEF_40556	UniRef50_P37122	0.0	Normal: 1907	2365	2163	1319	3678	3308	BlastAnno: 1944	UniRef50_P37122	505	61.04	498	193	1	264	1754	1	498	0.0	635
ABCDEF_40549	UniRef50_P37122	0.0	Normal: 1574	1962	1826	1150	2882	2723	BlastAnno: 1845	UniRef50_P37122	505	60.52	504	198	1	1754	246	1	504	0.0	638
ABCDEF_20301	UniRef50_O81117	0.0	Normal: 89	308	123	176	389	249	BlastAnno: 1637	UniRef50_O81117	514	64.04	470	164	3	1498	98	47	514	0.0	586
ABCDEF_3882	UniRef50_Q42569	0.0	Normal: 84	243	257	786	1561	182	BlastAnno: 1854	UniRef50_Q42569	472	72.51	422	107	1	1852	587	59	471	0.0	615
ABCDEF_20703	UniRef50_O49858	0.0	Normal: 115	636	20	0	0	597	BlastAnno: 1815	UniRef50_O49858	527	50.10	523	248	6	48	1607	12	524	0.0	533
ABCDEF_18295	UniRef50_O22203	0.0	Normal: 3667	1934	4977	10491	4588	1917	BlastAnno: 2006	UniRef50_O22203	508	74.85	493	123	1	214	1692	17	508	0.0	803
ABCDEF_48724	UniRef50_O23066	0.0	Normal: 104	11518	163	2083	3464	6356	BlastAnno: 2162	UniRef50_O23066	553	72.96	540	143	3	227	1840	1	539	0.0	807
ABCDEF_37738	UniRef50_P37122	0.0	Normal: 26	2447	19	30	33	993	BlastAnno: 1837	UniRef50_P37122	505	64.30	479	169	2	1685	249	28	504	0.0	639

1.14.12.-(Oxidoreductases;)
No Correspond Unigene
1.14.12.12(naphthalene 1,2-dioxygenase;)
No Correspond Unigene
1.14.12.15(terephthalate 1,2-dioxygenase;)
No Correspond Unigene
1.14.12.7(phthalate 4,5-dioxygenase;)
No Correspond Unigene
1.14.13.-(Oxidoreductases;)

ABCDEF_45538	UniRef50_O23024	3e-156	Normal: 110	6	165	774	133	36	BlastAnno: 1258	UniRef50_O23024	437	58.18	385	151	3	3	1157	37	411	3e-156	457

1.14.13.1(salicylate 1-monooxygenase;)
No Correspond Unigene
1.14.13.23(3-hydroxybenzoate 4-monooxygenase;)
No Correspond Unigene
1.2.1.-(Oxidoreductases;)

ABCDEF_14573	UniRef50_B5ZBH4	2e-108	Normal: 2547	9518	1266	309	441	22896	BlastAnno: 1872	UniRef50_B5ZBH4	475	42.52	461	259	4	228	1598	16	474	2e-108	343

1.3.-.-(Oxidoreductases;)

ABCDEF_37215	UniRef50_Q9VSM5	6e-76	Normal: 470	387	380	308	475	745	BlastAnno: 2204	UniRef50_Q9VSM5	316	44.33	300	155	4	228	1091	4	303	6e-76	255

1.3.1.-(Oxidoreductases;)

ABCDEF_45552	UniRef50_P52581	0.0	Normal: 213	2112	262	1058	1208	1322	BlastAnno: 1284	UniRef50_P52581	312	80.45	312	61	0	185	1120	1	312	0.0	530
ABCDEF_47970	UniRef50_Q9T0J6	0.0	Normal: 1040	443	1171	941	1234	650	BlastAnno: 2557	UniRef50_Q9T0J6	700	60.52	699	232	9	2337	271	36	700	0.0	860

1.3.1.19(cis-1,2-dihydrobenzene-1,2-diol dehydrogenase;)
No Correspond Unigene
1.3.1.29(cis-1,2-dihydro-1,2-dihydroxynaphthalene dehydrogenase;)
No Correspond Unigene
1.3.1.49(cis-3,4-dihydrophenanthrene-3,4-diol dehydrogenase)
No Correspond Unigene
1.3.1.53(No Name)
No Correspond Unigene
1.3.1.64(phthalate 4,5-cis-dihydrodiol dehydrogenase)
No Correspond Unigene
1.6.-.-(Oxidoreductases;)

ABCDEF_192	UniRef50_Q8GXR9	0.0	Normal: 327	311	268	361	529	963	BlastAnno: 1951	UniRef50_Q8GXR9	519	69.98	483	125	3	399	1847	57	519	0.0	664

1.6.5.-(Oxidoreductases;)

ABCDEF_44500	UniRef50_Q7NI12	0.0	Normal: 226	138	205	193	129	499	BlastAnno: 2484	UniRef50_Q7NI12	393	72.52	393	108	0	539	1717	1	393	0.0	619

ABCDEF_44502	UniRef50_Q7NI12	0.0	Normal: 125	74	101	121	85	319	BlastAnno: 1814	UniRef50_Q7NI12	393	72.52	393	108	0	54	1232	1	393	0.0	619
2.1.1.-(Transferases;)																					
ABCDEF_20806	UniRef50_Q94II3	0.0	Normal: 700	332	876	1458	1997	929	BlastAnno: 2275	UniRef50_Q94II3	600	73.57	594	153	4	2119	341	1	591	0.0	907
ABCDEF_45802	UniRef50_Q9ZPH9	0.0	Normal: 3543	3348	4296	9797	10830	5017	BlastAnno: 2642	UniRef50_Q9ZPH9	633	53.90	603	261	7	2216	456	23	624	0.0	676
ABCDEF_20070	UniRef50_Q944R7	0.0	Normal: 301	141	265	207	185	187	BlastAnno: 2675	UniRef50_Q944R7	724	58.28	719	275	9	127	2265	13	712	0.0	840
ABCDEF_34956	UniRef50_Q94II3	0.0	Normal: 1120	7269	2475	16033	22219	21461	BlastAnno: 2676	UniRef50_Q94II3	600	73.77	610	146	4	404	2230	1	597	0.0	957
ABCDEF_17121	UniRef50_Q9ZPH9	0.0	Normal: 124	77	109	284	199	132	BlastAnno: 2066	UniRef50_Q9ZPH9	633	53.24	601	259	8	161	1903	25	623	0.0	644
ABCDEF_6524	UniRef50_Q9ZPH9	0.0	Normal: 471	296	490	753	870	730	BlastAnno: 2602	UniRef50_Q9ZPH9	633	50.89	619	290	5	2221	404	6	623	0.0	659
ABCDEF_56017	UniRef50_Q9ZPH9	0.0	Normal: 302	167	286	596	748	529	BlastAnno: 2384	UniRef50_Q9ZPH9	633	51.37	619	287	5	213	2030	6	623	0.0	663
ABCDEF_44169	UniRef50_Q9SIZ3	0.0	Normal: 179	158	273	705	685	229	BlastAnno: 2300	UniRef50_Q9SIZ3	589	62.01	308	108	4	1219	296	285	583	0.0	394
ABCDEF_6521	UniRef50_Q9ZPH9	0.0	Normal: 339	198	314	641	767	582	BlastAnno: 2511	UniRef50_Q9ZPH9	633	51.37	619	287	5	2172	355	6	623	0.0	663
ABCDEF_44172	UniRef50_Q9SIZ3	0.0	Normal: 204	184	287	767	741	242	BlastAnno: 2317	UniRef50_Q9SIZ3	589	68.15	449	134	4	1637	291	144	583	0.0	641
ABCDEF_199	UniRef50_Q9SU94	0.0	Normal: 1076	275	1009	558	1247	306	BlastAnno: 1769	UniRef50_Q9SU94	390	78.45	348	74	1	284	1324	43	390	0.0	584
ABCDEF_52252	UniRef50_Q84W92	0.0	Normal: 1658	720	1653	1980	2289	1601	BlastAnno: 2170	UniRef50_Q84W92	535	72.49	469	120	3	409	1791	34	501	0.0	703
ABCDEF_51404	UniRef50_A3BMN9	0.0	Normal: 864	188	716	337	631	326	BlastAnno: 2192	UniRef50_A3BMN9	620	50.50	604	243	9	2008	251	55	620	0.0	555
ABCDEF_21386	UniRef50_Q94AI4	0.0	Normal: 2326	1250	2220	4294	2920	1983	BlastAnno: 3701	UniRef50_Q94AI4	775	47.27	842	332	16	567	2978	8	775	0.0	577
ABCDEF_35101	UniRef50_Q9MAT5	0.0	Normal: 1440	420	1268	576	1136	850	BlastAnno: 1634	UniRef50_Q9MAT5	383	69.03	352	109	0	1449	394	32	383	0.0	550
ABCDEF_960	UniRef50_Q9LN50	0.0	Normal: 3504	13198	3609	11156	19651	12155	BlastAnno: 2626	UniRef50_Q9LN50	724	49.14	525	246	5	883	2451	219	724	0.0	570
ABCDEF_48082	UniRef50_Q9LN50	0.0	Normal: 410	445	467	870	993	454	BlastAnno: 2340	UniRef50_Q9LN50	724	70.25	521	155	0	710	2272	204	724	0.0	821
ABCDEF_10473	UniRef50_Q08A71	0.0	Normal: 374	187	332	425	456	314	BlastAnno: 1764	UniRef50_Q08A71	435	79.23	366	75	1	344	1438	70	435	0.0	575
ABCDEF_7295	UniRef50_Q9ZPH9	0.0	Normal: 25	54	30	81	26	329	BlastAnno: 1191	UniRef50_Q9ZPH9	633	69.23	403	110	4	1191	22	225	626	0.0	599
ABCDEF_4160	UniRef50_Q94II3	0.0	Normal: 189	0	210	71	17	72	BlastAnno: 1424	UniRef50_Q94II3	600	77.28	449	101	1	1424	81	143	591	0.0	758
ABCDEF_44857	UniRef50_Q9ZPH9	0.0	Normal: 101	224	103	299	588	350	BlastAnno: 1582	UniRef50_Q9ZPH9	633	72.06	519	143	1	1571	21	114	632	0.0	822
ABCDEF_71541	UniRef50_Q9SIZ3	0.0	Normal: 187	167	296	765	724	237	BlastAnno: 2268	UniRef50_Q9SIZ3	589	62.01	308	108	4	1210	287	285	583	0.0	394
ABCDEF_44171	UniRef50_Q9SIZ3	0.0	Normal: 214	196	312	827	802	251	BlastAnno: 2339	UniRef50_Q9SIZ3	589	64.78	548	182	5	1978	335	47	583	0.0	717
ABCDEF_20805	UniRef50_Q94II3	0.0	Normal: 674	344	885	1417	2194	963	BlastAnno: 2400	UniRef50_Q94II3	600	78.21	436	94	1	879	2183	156	591	0.0	734
ABCDEF_18210	UniRef50_Q9SIZ3	0.0	Normal: 5584	3714	7379	17006	21892	7546	BlastAnno: 3199	UniRef50_Q9SIZ3	589	50.00	576	269	7	2145	424	27	585	0.0	574
ABCDEF_6526	UniRef50_Q9ZPH9	0.0	Normal: 627	185	837	2717	2815	1530	BlastAnno: 2611	UniRef50_Q9ZPH9	633	54.76	599	259	5	462	2222	25	623	0.0	689
ABCDEF_6525	UniRef50_Q9ZPH9	0.0	Normal: 602	165	760	2369	2605	1400	BlastAnno: 2545	UniRef50_Q9ZPH9	633	54.92	599	258	5	431	2191	25	623	0.0	689
ABCDEF_6523	UniRef50_Q9ZPH9	0.0	Normal: 314	175	304	641	784	552	BlastAnno: 2312	UniRef50_Q9ZPH9	633	52.42	599	272	4	201	1958	25	623	0.0	663
ABCDEF_67393	UniRef50_Q9ZPH9	0.0	Normal: 434	245	444	608	768	655	BlastAnno: 2346	UniRef50_Q9ZPH9	633	51.92	599	275	4	232	1989	25	623	0.0	659
ABCDEF_3579	UniRef50_O14744	0.0	Normal: 1361	326	984	568	791	440	BlastAnno: 2369	UniRef50_O14744	637	47.00	634	294	10	267	2120	30	637	0.0	553
2.8.2.-(Transferases;)																					
ABCDEF_60620	UniRef50_P52839	2e-85	Normal: 240	393	445	400	902	151	BlastAnno: 1191	UniRef50_P52839	326	45.95	333	168	6	58	1050	2	324	2e-85	271
3.1.1.35(dihydrocoumarin hydrolase)																					
No Correspond Unigene																					
3.3.2.-(Hydrolases;)																					
No Correspond Unigene																					
3.7.1.-(Hydrolases;)																					
No Correspond Unigene																					
4.1.1.-(Lyases;)																					
ABCDEF_57496	UniRef50_O96569	9e-20	Normal: 4	0	0	0	0	9	BlastAnno: 223	UniRef50_O96569	439	66.67	72	22	1	210	1	19	90	9e-20	87.4
4.1.1.55(4,5-dihydroxyphthalate decarboxylase;)																					
No Correspond Unigene																					
4.1.1.69(3,4-dihydroxyphthalate decarboxylase;)																					
No Correspond Unigene																					
4.1.2.-(Lyases;)																					
ABCDEF_38428	UniRef50_C3KN71	1e-47	Normal: 551	427	606	695	1516	1338	BlastAnno: 1495	UniRef50_C3KN71	273	38.34	253	150	1	334	1092	7	253	1e-47	173
4.1.2.34(4-(2-carboxyphenyl)-2-oxobut-3-enoate aldolase;)																					
No Correspond Unigene																					
4.1.3.-(Lyases;)																					
ABCDEF_21016	UniRef50_C5E1L7	1e-173	Normal: 775	334	669	635	559	950	BlastAnno: 2086	UniRef50_C5E1L7	551	49.91	549	253	8	1823	234	2	547	1e-173	517
5.1.2.-(Isomerases;)																					
No Correspond Unigene																					
>00625 Chloroalkane and chloroalkene degradation																					
1.1.-.-(Oxidoreductases;)																					
ABCDEF_17646	UniRef50_O34948	5e-83	Normal: 372	339	320	294	487	1376	BlastAnno: 1685	UniRef50_O34948	288	47.31	279	145	2	1523	690	5	282	5e-83	269
1.1.1.-(Oxidoreductases;)																					
ABCDEF_13453	UniRef50_Q9SYM5	0.0	Normal: 225	226	398	1950	2026	421	BlastAnno: 2578	UniRef50_Q9SYM5	669	86.61	672	87	2	298	2313	1	669	0.0	1226
ABCDEF_17342	UniRef50_Q84M96	0.0	Normal: 274	406	272	327	439	625	BlastAnno: 1277	UniRef50_Q84M96	346	73.33	345	91	1	1187	156	2	346	0.0	546
ABCDEF_31147	UniRef50_Q9SYM5	0.0	Normal: 4571	1591	1491	1401	1939	4327	BlastAnno: 2465	UniRef50_Q9SYM5	669	82.76	673	107	4	2397	394	1	669	0.0	1154
ABCDEF_17344	UniRef50_Q84M96	0.0	Normal: 252	373	257	297	380	540	BlastAnno: 1350	UniRef50_Q84M96	346	68.19	371	91	2	1263	154	2	346	0.0	530
ABCDEF_44674	UniRef50_Q9SYM5	0.0	Normal: 3581	2620	4329	9505	11447	4930	BlastAnno: 2634	UniRef50_Q9SYM5	669	87.80	672	78	3	253	2265	1	669	0.0	1241
1.1.1.1(alcohol dehydrogenase;)																					
ABCDEF_31928	UniRef50_B9RHN6	0.0	Normal: 721	714	784	1329	1570	1819	BlastAnno: 1711	UniRef50_B9RHN6	437	77.55	392	87	1	360	1535	46	436	0.0	610
ABCDEF_10335	UniRef50_Q8LEB2	0.0	Normal: 285	158	514	810	352	183	BlastAnno: 1547	UniRef50_Q8LEB2	381	74.93	379	92	1	1317	181	5	380	0.0	598
1.1.2.7(methanol dehydrogenase (cytochrome c);)																					
No Correspond Unigene																					
1.1.2.8(alcohol dehydrogenase (cytochrome c);)																					
No Correspond Unigene																					
1.13.12.-(Oxidoreductases;)																					
No Correspond Unigene																					

1.14.-.-(Oxidoreductases;)

ABCDEF_44739	UniRef50_064989	0.0	Normal: 157	87	159	718	673	189	BlastAnno: 2185	UniRef50_064989	513	70.75	482	112	3	1867	509	30	511	0.0	706
ABCDEF_31491	UniRef50_Q93VK5	0.0	Normal: 647	1826	671	1517	1770	5953	BlastAnno: 2278	UniRef50_Q93VK5	595	79.14	556	113	2	198	1862	35	588	0.0	902
ABCDEF_40554	UniRef50_P37122	0.0	Normal: 1574	1962	1829	1150	2882	2721	BlastAnno: 1828	UniRef50_P37122	505	60.52	504	198	1	1752	244	1	504	0.0	638
ABCDEF_34049	UniRef50_023365	0.0	Normal: 363	384	349	442	549	1416	BlastAnno: 2101	UniRef50_023365	580	76.17	579	116	5	230	1957	19	578	0.0	904
ABCDEF_21579	UniRef50_048928	0.0	Normal: 1005	643	1606	4372	3226	1621	BlastAnno: 1895	UniRef50_048928	513	69.72	502	151	1	185	1690	7	507	0.0	731
ABCDEF_37741	UniRef50_P37122	0.0	Normal: 12	760	9	24	34	576	BlastAnno: 1197	UniRef50_P37122	505	65.70	379	129	1	1195	59	127	504	0.0	526
ABCDEF_35618	UniRef50_048928	0.0	Normal: 28	2648	17	142	1234	902	BlastAnno: 1746	UniRef50_048928	513	66.40	506	168	2	1649	132	4	507	0.0	701
ABCDEF_24522	UniRef50_P37122	0.0	Normal: 334	2190	561	268	1391	2459	BlastAnno: 2075	UniRef50_P37122	505	61.52	460	176	1	1544	165	47	505	0.0	612
ABCDEF_14592	UniRef50_023066	0.0	Normal: 1514	402	2245	7837	4742	1248	BlastAnno: 2378	UniRef50_023066	553	72.71	546	140	4	2111	489	1	542	0.0	821
ABCDEF_14512	UniRef50_Q9FG65	0.0	Normal: 261	872	212	291	175	789	BlastAnno: 1878	UniRef50_Q9FG65	502	56.89	501	197	9	1738	266	11	502	0.0	532
ABCDEF_44487	UniRef50_Q940V4	0.0	Normal: 3	41	53	501	262	542	BlastAnno: 1712	UniRef50_Q940V4	465	69.37	382	116	1	38	1180	14	395	0.0	572
ABCDEF_17723	UniRef50_P37122	0.0	Normal: 240	1698	219	768	592	794	BlastAnno: 1757	UniRef50_P37122	505	63.40	459	166	2	228	1601	47	504	0.0	617
ABCDEF_37901	UniRef50_Q42569	0.0	Normal: 30	149	86	255	195	255	BlastAnno: 1246	UniRef50_Q42569	472	78.27	359	73	2	64	1140	43	396	0.0	567
ABCDEF_7260	UniRef50_081117	0.0	Normal: 314	1085	466	3248	2575	1360	BlastAnno: 1768	UniRef50_081117	514	59.92	474	188	1	145	1566	43	514	0.0	615
ABCDEF_40548	UniRef50_P37122	0.0	Normal: 2756	2467	2752	1072	3120	1735	BlastAnno: 1947	UniRef50_P37122	505	59.72	504	202	1	241	1749	1	504	0.0	636
ABCDEF_44485	UniRef50_Q940V4	0.0	Normal: 3	55	63	574	338	660	BlastAnno: 1607	UniRef50_Q940V4	465	68.58	452	141	1	42	1394	14	465	0.0	673
ABCDEF_40552	UniRef50_P37122	0.0	Normal: 2397	1870	2304	1249	10428	2291	BlastAnno: 1812	UniRef50_P37122	505	60.12	504	200	1	108	1616	1	504	0.0	640
ABCDEF_37902	UniRef50_Q42569	0.0	Normal: 84	524	254	725	670	1049	BlastAnno: 1812	UniRef50_Q42569	472	77.47	435	92	3	1675	371	43	471	0.0	681
ABCDEF_40556	UniRef50_P37122	0.0	Normal: 1907	2365	2163	1319	3678	3308	BlastAnno: 1944	UniRef50_P37122	505	61.04	498	193	1	264	1754	1	498	0.0	635
ABCDEF_40549	UniRef50_P37122	0.0	Normal: 1574	1962	1826	1150	2882	2723	BlastAnno: 1845	UniRef50_P37122	505	60.52	504	198	1	1754	246	1	504	0.0	638
ABCDEF_20301	UniRef50_081117	0.0	Normal: 89	308	123	176	389	249	BlastAnno: 1637	UniRef50_081117	514	64.04	470	164	3	1498	98	47	514	0.0	586
ABCDEF_3882	UniRef50_Q42569	0.0	Normal: 84	243	257	786	1561	182	BlastAnno: 1854	UniRef50_Q42569	472	72.51	422	107	1	1852	587	59	471	0.0	615
ABCDEF_20703	UniRef50_049858	0.0	Normal: 115	636	20	0	0	597	BlastAnno: 1815	UniRef50_049858	527	50.10	523	248	6	48	1607	12	524	0.0	533
ABCDEF_18295	UniRef50_022203	0.0	Normal: 3667	1934	4977	10491	4588	1917	BlastAnno: 2006	UniRef50_022203	508	74.85	493	123	1	214	1692	17	508	0.0	803
ABCDEF_48724	UniRef50_023066	0.0	Normal: 104	11518	163	2083	3464	6356	BlastAnno: 2162	UniRef50_023066	553	72.96	540	143	3	227	1840	1	539	0.0	807
ABCDEF_37738	UniRef50_P37122	0.0	Normal: 26	2447	19	30	33	993	BlastAnno: 1837	UniRef50_P37122	505	64.30	479	169	2	1685	249	28	504	0.0	639

1.14.12.11(toluene dioxygenase;)

No Correspond Unigene

1.14.13.-(Oxidoreductases;)

ABCDEF_45538	UniRef50_023024	3e-156	Normal: 110	6	165	774	133	36	BlastAnno: 1258	UniRef50_023024	437	58.18	385	151	3	3	1157	37	411	3e-156	457

1.14.13.69(alkene monooxygenase;)

No Correspond Unigene

1.18.6.1(nitrogenase)

No Correspond Unigene

1.2.1.3(aldehyde dehydrogenase (NAD+);)

ABCDEF_52483	UniRef50_Q70E96	0.0	Normal: 28	851	41	0	3965	420	BlastAnno: 1750	UniRef50_Q70E96	484	54.56	482	219	0	107	1552	3	484	0.0	548
ABCDEF_17638	UniRef50_Q70E96	0.0	Normal: 580	718	749	2424	1317	775	BlastAnno: 1970	UniRef50_Q70E96	484	62.21	471	177	1	283	1692	2	472	0.0	625
ABCDEF_403	UniRef50_Q8W033	0.0	Normal: 69	355	98	39	223	1111	BlastAnno: 1749	UniRef50_Q8W033	550	65.96	473	161	0	1523	105	74	546	0.0	625
ABCDEF_45143	UniRef50_Q0WSF1	0.0	Normal: 1445	874	1200	1789	1940	1504	BlastAnno: 2450	UniRef50_Q0WSF1	596	79.97	594	115	2	2220	451	1	594	0.0	1015

1.2.1.46(formaldehyde dehydrogenase;)

No Correspond Unigene

1.97.1.-(Oxidoreductases;)

No Correspond Unigene

1.97.1.8(tetrachloroethene reductive dehalogenase;)

No Correspond Unigene

3.3.2.10(soluble epoxide hydrolase;)

No Correspond Unigene

3.8.1.-(Hydrolases;)

No Correspond Unigene

3.8.1.2(No Name)

ABCDEF_24773	UniRef50_A9GEM6	2e-26	Normal: 161	223	191	185	211	772	BlastAnno: 1237	UniRef50_A9GEM6	202	33.67	196	122	4	257	823	5	199	2e-26	111

3.8.1.3(haloacetate dehalogenase;)

No Correspond Unigene

3.8.1.5(haloalkane dehalogenase;)

ABCDEF_21424	UniRef50_Q31PT8	4e-55	Normal: 204	722	162	247	246	1436	BlastAnno: 1354	UniRef50_Q31PT8	298	39.78	274	157	6	943	128	25	292	4e-55	193

4.1.1.-(Lyases;)

ABCDEF_57496	UniRef50_096569	9e-20	Normal: 4	0	0	0	0	9	BlastAnno: 223	UniRef50_096569	439	66.67	72	22	1	210	1	19	90	9e-20	87.4

4.2.1.112(acetylene hydratase;)

No Correspond Unigene

4.5.1.3(dichloromethane dehalogenase;)

No Correspond Unigene

>00626 Naphthalene degradation

1.1.-.-(Oxidoreductases;)

ABCDEF_17646	UniRef50_034948	5e-83	Normal: 372	339	320	294	487	1376	BlastAnno: 1685	UniRef50_034948	288	47.31	279	145	2	1523	690	5	282	5e-83	269

1.1.1.1(alcohol dehydrogenase;)

ABCDEF_31928	UniRef50_B9RHN6	0.0	Normal: 721	714	784	1329	1570	1819	BlastAnno: 1711	UniRef50_B9RHN6	437	77.55	392	87	1	360	1535	46	436	0.0	610
ABCDEF_10335	UniRef50_Q8LEB2	0.0	Normal: 285	158	514	810	352	183	BlastAnno: 1547	UniRef50_Q8LEB2	381	74.93	379	92	1	1317	181	5	380	0.0	598

1.13.-.-(Oxidoreductases;)

ABCDEF_51946	UniRef50_Q949R4	2e-119	Normal: 133	259	66	108	237	1420	BlastAnno: 1085	UniRef50_Q949R4	269	66.93	254	78	3	1012	266	4	256	2e-119	355

1.13.11.-(Oxidoreductases;)

No Correspond Unigene

1.13.11.56(1,2-dihydroxynaphthalene dioxygenase;)

No Correspond Unigene

1.14.12.-(Oxidoreductases;)

No Correspond Unigene

1.14.12.12(naphthalene 1,2-dioxygenase;)

No Correspond Unigene

1.14.13.-(Oxidoreductases;)

ABCDEF_45538	UniRef50_O23024	3e-156	Normal: 110	6	165	774	133	36	BlastAnno: 1258	UniRef50_O23024	437	58.18	385	151	3	3	1157	37	411	3e-156	457

1.14.13.1(salicylate 1-monooxygenase;)

No Correspond Unigene

1.14.99.-(Oxidoreductases;)

ABCDEF_66639	UniRef50_Q0DUI8	0.0	Normal: 646	675	723	1956	1163	1303	BlastAnno: 1867	UniRef50_Q0DUI8	578	85.02	414	61	1	167	1408	158	570	0.0	734
ABCDEF_52814	UniRef50_Q0DUI8	0.0	Normal: 1118	1292	1150	3508	1987	2103	BlastAnno: 2562	UniRef50_Q0DUI8	578	77.22	575	124	4	370	2094	3	570	0.0	909

1.2.1.-(Oxidoreductases;)

ABCDEF_14573	UniRef50_B5ZBH4	2e-108	Normal: 2547	9518	1266	309	441	22896	BlastAnno: 1872	UniRef50_B5ZBH4	475	42.52	461	259	4	228	1598	16	474	2e-108	343

1.2.1.65(salicylaldehyde dehydrogenase)

No Correspond Unigene

1.2.3.-(Oxidoreductases;)

No Correspond Unigene

1.3.1.29(cis-1,2-dihydro-1,2-dihydroxynaphthalene dehydrogenase;)

No Correspond Unigene

1.3.99.-(Oxidoreductases;)

ABCDEF_4448	UniRef50_Q6JQN1	0.0	Normal: 1778	1683	1752	2720	2409	2803	BlastAnno: 2780	UniRef50_Q6JQN1	1059	48.13	802	354	14	231	2564	293	1056	0.0	718

2.3.1.-(Transferases;)

ABCDEF_520	UniRef50_Q9FF86	0.0	Normal: 1672	85	2273	5056	3383	1211	BlastAnno: 1811	UniRef50_Q9FF86	484	64.06	448	149	6	1664	354	3	449	0.0	573
ABCDEF_68664	UniRef50_Q9ZVN2	0.0	Normal: 79	35	85	107	193	63	BlastAnno: 1488	UniRef50_Q9ZVN2	704	63.99	411	142	3	46	1275	298	703	0.0	540
ABCDEF_52245	UniRef50_Q9FG87	0.0	Normal: 1878	635	1833	3814	1432	1376	BlastAnno: 1758	UniRef50_Q9FG87	529	55.35	486	204	4	4	1428	34	517	0.0	530
ABCDEF_16730	UniRef50_Q3EC11	0.0	Normal: 281	204	237	365	377	458	BlastAnno: 2092	UniRef50_Q3EC11	536	59.33	536	204	8	196	1785	3	530	0.0	661
ABCDEF_4655	UniRef50_A5BMG1	0.0	Normal: 2426	1856	3606	6958	4608	2755	BlastAnno: 2261	UniRef50_A5BMG1	481	68.37	528	112	10	263	1843	3	476	0.0	667
ABCDEF_13860	UniRef50_Q9C533	0.0	Normal: 588	220	570	1451	1338	310	BlastAnno: 2648	UniRef50_Q9C533	596	63.37	606	177	12	2226	511	1	595	0.0	558
ABCDEF_28294	UniRef50_Q9SYZ0	0.0	Normal: 1038	4070	1427	6745	877	2797	BlastAnno: 1785	UniRef50_Q9SYZ0	493	52.37	485	223	3	1598	162	5	487	0.0	533
ABCDEF_47718	UniRef50_Q9C533	0.0	Normal: 1599	236	1657	2093	1231	588	BlastAnno: 2680	UniRef50_Q9C533	596	65.02	626	165	14	2280	472	1	595	0.0	634
ABCDEF_45080	UniRef50_Q9SYZ0	0.0	Normal: 1005	592	1079	2360	3762	2217	BlastAnno: 2130	UniRef50_Q9SYZ0	493	57.85	484	197	3	302	1735	5	487	0.0	533
ABCDEF_25377	UniRef50_Q9C6L5	0.0	Normal: 37	2090	138	2573	1026	1298	BlastAnno: 1334	UniRef50_Q9C6L5	492	79.74	385	76	1	1155	1	6	388	0.0	654
ABCDEF_33470	UniRef50_Q9LW26	0.0	Normal: 172	98	156	260	391	149	BlastAnno: 2993	UniRef50_Q9LW26	701	48.96	627	301	5	655	2508	85	701	0.0	581
ABCDEF_40654	UniRef50_Q9H0A0	0.0	Normal: 638	213	530	318	318	286	BlastAnno: 2216	UniRef50_Q9H0A0	1025	56.87	721	279	6	2127	13	91	795	0.0	756
ABCDEF_33460	UniRef50_Q9ZVN2	0.0	Normal: 144	71	119	216	306	97	BlastAnno: 2691	UniRef50_Q9ZVN2	704	61.50	587	191	4	379	2133	83	636	0.0	715
ABCDEF_14442	UniRef50_Q9SYZ0	0.0	Normal: 341	684	439	1040	1601	552	BlastAnno: 1779	UniRef50_Q9SYZ0	493	63.12	480	171	2	1570	149	5	484	0.0	628
ABCDEF_52626	UniRef50_B6SYP9	0.0	Normal: 815	346	1131	3015	1537	108	BlastAnno: 1861	UniRef50_B6SYP9	494	55.02	438	189	3	1538	249	51	488	0.0	536
ABCDEF_644	UniRef50_Q9SB58	0.0	Normal: 704	444	913	969	1431	848	BlastAnno: 1777	UniRef50_Q9SB58	407	64.15	424	129	8	1572	316	1	406	0.0	554
ABCDEF_27087	UniRef50_Q52T38	0.0	Normal: 324	221	233	419	343	412	BlastAnno: 1631	UniRef50_Q52T38	620	75.95	420	91	1	1409	150	1	410	0.0	681
ABCDEF_34187	UniRef50_Q8L5Y5	0.0	Normal: 497	415	588	523	291	433	BlastAnno: 2677	UniRef50_Q8L5Y5	718	57.30	733	251	15	202	2274	1	713	0.0	683
ABCDEF_47966	UniRef50_Q9SYZ0	0.0	Normal: 577	934	326	580	682	3440	BlastAnno: 2177	UniRef50_Q9SYZ0	493	56.40	484	204	3	1780	347	5	487	0.0	535
ABCDEF_51189	UniRef50_Q52T38	0.0	Normal: 135	152	128	181	219	327	BlastAnno: 1766	UniRef50_Q52T38	620	68.09	470	137	3	80	1483	130	588	0.0	641
ABCDEF_41288	UniRef50_Q9SB58	0.0	Normal: 307	183	332	637	649	322	BlastAnno: 1403	UniRef50_Q9SB58	407	67.56	410	110	6	1401	184	15	405	0.0	523
ABCDEF_33461	UniRef50_Q9ZVN2	0.0	Normal: 142	84	140	206	283	119	BlastAnno: 2904	UniRef50_Q9ZVN2	704	60.98	574	201	6	2271	559	83	636	0.0	686
ABCDEF_14334	UniRef50_Q9SYZ0	0.0	Normal: 2263	129	1724	3469	866	283	BlastAnno: 2039	UniRef50_Q9SYZ0	493	63.22	484	171	2	369	1802	5	487	0.0	627
ABCDEF_24844	UniRef50_Q9FF86	0.0	Normal: 1857	3686	2657	21940	11467	4090	BlastAnno: 1640	UniRef50_Q9FF86	484	66.89	447	135	6	1492	185	2	446	0.0	575
ABCDEF_51190	UniRef50_Q52T38	0.0	Normal: 145	169	142	210	232	335	BlastAnno: 1891	UniRef50_Q52T38	620	70.04	514	141	3	73	1608	86	588	0.0	726
ABCDEF_14085	UniRef50_Q8L5Y5	0.0	Normal: 353	41	384	527	331	280	BlastAnno: 2362	UniRef50_Q8L5Y5	718	62.07	704	220	12	2360	348	24	713	0.0	676
ABCDEF_33458	UniRef50_Q9LW26	0.0	Normal: 158	78	130	240	342	106	BlastAnno: 2822	UniRef50_Q9LW26	701	46.72	655	302	5	400	2337	85	701	0.0	568
ABCDEF_25193	UniRef50_Q9C6L5	0.0	Normal: 3256	368	3548	6230	5109	1288	BlastAnno: 1786	UniRef50_Q9C6L5	492	65.83	480	156	4	1668	241	17	492	0.0	668
ABCDEF_33457	UniRef50_Q9ZVN2	0.0	Normal: 168	83	137	250	375	112	BlastAnno: 2823	UniRef50_Q9ZVN2	704	65.12	496	168	3	379	1860	83	575	0.0	648
ABCDEF_7634	UniRef50_Q9C6L5	0.0	Normal: 2543	9303	2711	11578	5533	10032	BlastAnno: 2064	UniRef50_Q9C6L5	492	52.76	489	225	3	1734	268	1	483	0.0	535
ABCDEF_299	UniRef50_Q71N54	0.0	Normal: 569	1147	511	900	649	1879	BlastAnno: 2017	UniRef50_Q71N54	535	61.73	533	198	5	338	1924	4	534	0.0	677
ABCDEF_27086	UniRef50_Q52T38	0.0	Normal: 784	470	617	843	893	976	BlastAnno: 2398	UniRef50_Q52T38	620	70.80	637	165	4	225	2129	1	618	0.0	887
ABCDEF_51188	UniRef50_Q52T38	0.0	Normal: 273	327	255	415	349	441	BlastAnno: 2324	UniRef50_Q52T38	620	71.30	568	150	3	2041	344	32	588	0.0	815
ABCDEF_44812	UniRef50_Q6DR03	0.0	Normal: 982	902	1005	1071	959	2999	BlastAnno: 2450	UniRef50_Q6DR03	565	54.97	644	198	17	2142	220	1	555	0.0	540

2.8.3.-(Transferases;)

No Correspond Unigene

4.1.1.-(Lyases;)

ABCDEF_57496	UniRef50_O96569	9e-20	Normal: 4	0	0	0	0	9	BlastAnno: 223	UniRef50_O96569	439	66.67	72	22	1	210	1	19	90	9e-20	87.4

4.1.2.45(trans-o-hydroxybenzylidenepyruvate hydratase-aldolase;)

No Correspond Unigene

4.1.99.-(Lyases;)

No Correspond Unigene

4.2.1.-(Lyases;)

ABCDEF_48679	UniRef50_Q0RP29	5e-115	Normal: 568	325	563	941	1285	676	BlastAnno: 1424	UniRef50_Q0RP29	493	56.39	321	129	4	119	1075	39	350	5e-115	356

5.3.99.-(Isomerases;)

No Correspond Unigene

5.99.1.4(2-hydroxychromene-2-carboxylate isomerase;)

No Correspond Unigene

>00627 Aminobenzoate degradation

1.1.-.-(Oxidoreductases;)

ABCDEF_17646	UniRef50_O34948	5e-83	Normal: 372	339	320	294	487	1376	BlastAnno: 1685	UniRef50_O34948	288	47.31	279	145	2	1523	690	5	282	5e-83	269

1.1.3.19(4-hydroxymandelate oxidase;)

No Correspond Unigene

1.1.3.38(vanillyl-alcohol oxidase;)

No Correspond Unigene

1.1.99.31(No Name)

ABCDEF_35503	UniRef50_P20932	7e-08	Normal: 9	19	17	0	0	6	BlastAnno: 557	UniRef50_P20932	393	39.13	46	28	0	214	351	1	46	7e-08	42.7

1.13.11.-(Oxidoreductases;)

No Correspond Unigene

1.13.11.3(protocatechuate 3,4-dioxygenase;)

No Correspond Unigene

1.13.11.8(protocatechuate 4,5-dioxygenase;)

No Correspond Unigene

1.14.-.-(Oxidoreductases;)

ABCDEF_44739	UniRef50_O64989	0.0	Normal: 157	87	159	718	673	189	BlastAnno: 2185	UniRef50_O64989	513	70.75	482	112	3	1867	509	30	511	0.0	706
ABCDEF_31491	UniRef50_Q93VK5	0.0	Normal: 647	1826	671	1517	1770	5953	BlastAnno: 2278	UniRef50_Q93VK5	595	79.14	556	113	2	198	1862	35	588	0.0	902
ABCDEF_40554	UniRef50_P37122	0.0	Normal: 1574	1962	1829	1150	2882	2721	BlastAnno: 1828	UniRef50_P37122	505	60.52	504	198	1	1752	244	1	504	0.0	638
ABCDEF_34049	UniRef50_O23365	0.0	Normal: 363	384	349	442	549	1416	BlastAnno: 2101	UniRef50_O23365	580	76.17	579	116	5	230	1957	19	578	0.0	904
ABCDEF_21579	UniRef50_O48928	0.0	Normal: 1005	643	1606	4372	3226	1621	BlastAnno: 1895	UniRef50_O48928	513	69.72	502	151	1	185	1690	7	507	0.0	731
ABCDEF_37741	UniRef50_P37122	0.0	Normal: 12	760	9	24	34	576	BlastAnno: 1197	UniRef50_P37122	505	65.70	379	129	1	1195	59	127	504	0.0	526
ABCDEF_35618	UniRef50_O48928	0.0	Normal: 28	2648	17	142	1234	902	BlastAnno: 1746	UniRef50_O48928	513	66.40	506	168	2	1649	132	4	507	0.0	701
ABCDEF_24522	UniRef50_P37122	0.0	Normal: 334	2190	561	268	1391	2459	BlastAnno: 2075	UniRef50_P37122	505	61.52	460	176	1	1544	165	47	505	0.0	612
ABCDEF_14592	UniRef50_O23066	0.0	Normal: 1514	402	2245	7837	4742	1248	BlastAnno: 2378	UniRef50_O23066	553	72.71	546	140	4	2111	489	1	542	0.0	821
ABCDEF_14512	UniRef50_Q9FG65	0.0	Normal: 261	872	212	291	175	789	BlastAnno: 1878	UniRef50_Q9FG65	502	56.89	501	197	9	1738	266	11	502	0.0	532
ABCDEF_44487	UniRef50_Q940V4	0.0	Normal: 3	41	53	501	262	542	BlastAnno: 1712	UniRef50_Q940V4	465	69.37	382	116	1	38	1180	14	395	0.0	572
ABCDEF_17723	UniRef50_P37122	0.0	Normal: 240	1698	219	768	592	794	BlastAnno: 1757	UniRef50_P37122	505	63.40	459	166	2	228	1601	47	504	0.0	617
ABCDEF_37901	UniRef50_Q42569	0.0	Normal: 30	149	86	255	195	255	BlastAnno: 1246	UniRef50_Q42569	472	78.27	359	73	2	64	1140	43	396	0.0	567
ABCDEF_7260	UniRef50_O81117	0.0	Normal: 314	1085	466	3248	2575	1360	BlastAnno: 1768	UniRef50_O81117	514	59.92	474	188	1	145	1566	43	514	0.0	615
ABCDEF_40548	UniRef50_P37122	0.0	Normal: 2756	2467	2752	1072	3120	1735	BlastAnno: 1947	UniRef50_P37122	505	59.72	504	202	1	241	1749	1	504	0.0	636
ABCDEF_44485	UniRef50_Q940V4	0.0	Normal: 3	55	63	574	338	660	BlastAnno: 1607	UniRef50_Q940V4	465	68.58	452	141	1	42	1394	14	465	0.0	673
ABCDEF_40552	UniRef50_P37122	0.0	Normal: 2397	1870	2304	1249	10428	2291	BlastAnno: 1812	UniRef50_P37122	505	60.12	504	200	1	108	1616	1	504	0.0	640
ABCDEF_37902	UniRef50_Q42569	0.0	Normal: 84	524	254	725	670	1049	BlastAnno: 1812	UniRef50_Q42569	472	77.47	435	92	3	1675	371	43	471	0.0	681
ABCDEF_40556	UniRef50_P37122	0.0	Normal: 1907	2365	2163	1319	3678	3308	BlastAnno: 1944	UniRef50_P37122	505	61.04	498	193	1	264	1754	1	498	0.0	635
ABCDEF_40549	UniRef50_P37122	0.0	Normal: 1574	1962	1826	1150	2882	2723	BlastAnno: 1845	UniRef50_P37122	505	60.52	504	198	1	1754	246	1	504	0.0	638
ABCDEF_20301	UniRef50_O81117	0.0	Normal: 89	308	123	176	389	249	BlastAnno: 1637	UniRef50_O81117	514	64.04	470	164	3	1498	98	47	514	0.0	586
ABCDEF_3882	UniRef50_Q42569	0.0	Normal: 84	243	257	786	1561	182	BlastAnno: 1854	UniRef50_Q42569	472	72.51	422	107	1	1852	587	59	471	0.0	615
ABCDEF_20703	UniRef50_O49858	0.0	Normal: 115	636	20	0	0	597	BlastAnno: 1815	UniRef50_O49858	527	50.10	523	248	6	48	1607	12	524	0.0	533
ABCDEF_18295	UniRef50_O22203	0.0	Normal: 3667	1934	4977	10491	4588	1917	BlastAnno: 2006	UniRef50_O22203	508	74.85	493	123	1	214	1692	17	508	0.0	803
ABCDEF_48724	UniRef50_O23066	0.0	Normal: 104	11518	163	2083	3464	6356	BlastAnno: 2162	UniRef50_O23066	553	72.96	540	143	3	227	1840	1	539	0.0	807
ABCDEF_37738	UniRef50_P37122	0.0	Normal: 26	2447	19	30	33	993	BlastAnno: 1837	UniRef50_P37122	505	64.30	479	169	2	1685	249	28	504	0.0	639

1.14.12.-(Oxidoreductases;)

No Correspond Unigene

1.14.12.1(anthranilate 1,2-dioxygenase (deaminating, decarboxylating);)

No Correspond Unigene

1.14.13.-(Oxidoreductases;)

ABCDEF_45538	UniRef50_O23024	3e-156	Normal: 110	6	165	774	133	36	BlastAnno: 1258	UniRef50_O23024	437	58.18	385	151	3	3	1157	37	411	3e-156	457

1.14.13.12(benzoate 4-monooxygenase;)

No Correspond Unigene

1.14.13.27(4-aminobenzoate 1-monooxygenase;)

No Correspond Unigene

1.14.13.29(4-nitrophenol 2-monooxygenase;)

No Correspond Unigene

1.14.13.35(anthranilate 3-monooxygenase (deaminating);)

No Correspond Unigene

1.14.13.40(anthraniloyl-CoA monooxygenase;)

No Correspond Unigene

1.14.13.7(phenol 2-monooxygenase;)

No Correspond Unigene

1.14.13.82(vanillate monooxygenase;)

ABCDEF_52243	UniRef50_B2J2T9	3e-71	Normal: 759	1146	777	893	690	1983	BlastAnno: 2073	UniRef50_B2J2T9	451	36.13	346	203	6	418	1440	25	357	3e-71	246

1.14.14.1(unspecific monooxygenase;)

ABCDEF_41955	UniRef50_B9S0F8	2e-174	Normal: 70	140	28	0	0	150	BlastAnno: 1118	UniRef50_B9S0F8	506	66.67	366	115	5	2	1099	138	496	2e-174	504

1.14.16.6(mandelate 4-monooxygenase;)

No Correspond Unigene

1.2.1.28(benzaldehyde dehydrogenase (NAD+);)

No Correspond Unigene

1.2.1.64(4-hydroxybenzaldehyde dehydrogenase;)

No Correspond Unigene

1.2.1.67(vanillin dehydrogenase)

No Correspond Unigene

1.2.1.7(benzaldehyde dehydrogenase (NADP+);)
No Correspond Unigene
1.2.3.-(Oxidoreductases;)
No Correspond Unigene
1.3.1.57(phloroglucinol reductase)
No Correspond Unigene
1.3.99.20(4-hydroxybenzoyl-CoA reductase;)
No Correspond Unigene
1.4.-.-(Oxidoreductases;)
No Correspond Unigene
1.6.5.6(p-benzoquinone reductase (NADPH))
No Correspond Unigene
1.6.5.7(2-hydroxy-1,4-benzoquinone reductase;)
No Correspond Unigene
1.7.1.-(Oxidoreductases;)
No Correspond Unigene
1.97.1.2(pyrogallol hydroxytransferase;)
No Correspond Unigene
2.3.1.-(Transferases;)

ABCDEF_520	UniRef50_Q9FF86	0.0	Normal: 1672	85	2273	5056	3383	1211	BlastAnno: 1811	UniRef50_Q9FF86	484	64.06	448	149	6	1664	354	3	449	0.0	573
ABCDEF_68664	UniRef50_Q9ZVN2	0.0	Normal: 79	35	85	107	193	63	BlastAnno: 1488	UniRef50_Q9ZVN2	704	63.99	411	142	3	46	1275	298	703	0.0	540
ABCDEF_52245	UniRef50_Q9FG87	0.0	Normal: 1878	635	1833	3814	1432	1376	BlastAnno: 1758	UniRef50_Q9FG87	529	55.35	486	204	4	4	1428	34	517	0.0	530
ABCDEF_16730	UniRef50_Q3EC11	0.0	Normal: 281	204	237	365	377	458	BlastAnno: 2092	UniRef50_Q3EC11	536	59.33	536	204	8	196	1785	3	530	0.0	661
ABCDEF_4655	UniRef50_A5BMG1	0.0	Normal: 2426	1856	3606	6958	4608	2755	BlastAnno: 2261	UniRef50_A5BMG1	481	68.37	528	112	10	263	1843	3	476	0.0	667
ABCDEF_13860	UniRef50_Q9C533	0.0	Normal: 588	220	570	1451	1338	310	BlastAnno: 2648	UniRef50_Q9C533	596	63.37	606	177	12	2226	511	1	595	0.0	558
ABCDEF_28294	UniRef50_Q9SYZ0	0.0	Normal: 1038	4070	1427	6745	877	2797	BlastAnno: 1785	UniRef50_Q9SYZ0	493	52.37	485	223	3	1598	162	5	487	0.0	533
ABCDEF_47718	UniRef50_Q9C533	0.0	Normal: 1599	236	1657	2093	1231	588	BlastAnno: 2680	UniRef50_Q9C533	596	65.02	626	165	14	2280	472	1	595	0.0	634
ABCDEF_45080	UniRef50_Q9SYZ0	0.0	Normal: 1005	592	1079	2360	3762	2217	BlastAnno: 2130	UniRef50_Q9SYZ0	493	57.85	484	197	3	302	1735	5	487	0.0	533
ABCDEF_25377	UniRef50_Q9C6L5	0.0	Normal: 37	2090	138	2573	1026	1298	BlastAnno: 1334	UniRef50_Q9C6L5	492	79.74	385	76	1	1155	1	6	388	0.0	654
ABCDEF_33470	UniRef50_Q9LW26	0.0	Normal: 172	98	156	260	391	149	BlastAnno: 2993	UniRef50_Q9LW26	701	48.96	627	301	5	655	2508	85	701	0.0	581
ABCDEF_40654	UniRef50_Q9H0A0	0.0	Normal: 638	213	530	318	318	286	BlastAnno: 2216	UniRef50_Q9H0A0	1025	56.87	721	279	6	2127	13	91	795	0.0	756
ABCDEF_33460	UniRef50_Q9ZVN2	0.0	Normal: 144	71	119	216	306	97	BlastAnno: 2691	UniRef50_Q9ZVN2	704	61.50	587	191	4	379	2133	83	636	0.0	715
ABCDEF_14442	UniRef50_Q9SYZ0	0.0	Normal: 341	684	439	1040	1601	552	BlastAnno: 1779	UniRef50_Q9SYZ0	493	63.12	480	171	2	1570	149	5	484	0.0	628
ABCDEF_52626	UniRef50_B6SYP9	0.0	Normal: 815	346	1131	3015	1537	108	BlastAnno: 1861	UniRef50_B6SYP9	494	55.02	438	189	3	1538	249	51	488	0.0	536
ABCDEF_644	UniRef50_Q9SB58	0.0	Normal: 704	444	913	969	1431	848	BlastAnno: 1777	UniRef50_Q9SB58	407	64.15	424	129	8	1572	316	1	406	0.0	554
ABCDEF_27087	UniRef50_Q52T38	0.0	Normal: 324	221	233	419	343	412	BlastAnno: 1631	UniRef50_Q52T38	620	75.95	420	91	1	1409	150	1	410	0.0	681
ABCDEF_34187	UniRef50_Q8L5Y5	0.0	Normal: 497	415	588	523	291	433	BlastAnno: 2677	UniRef50_Q8L5Y5	718	57.30	733	251	15	202	2274	1	713	0.0	683
ABCDEF_47966	UniRef50_Q9SYZ0	0.0	Normal: 577	934	326	580	682	3440	BlastAnno: 2177	UniRef50_Q9SYZ0	493	56.40	484	204	3	1780	347	5	487	0.0	535
ABCDEF_51189	UniRef50_Q52T38	0.0	Normal: 135	152	128	181	219	327	BlastAnno: 1766	UniRef50_Q52T38	620	68.09	470	137	3	80	1483	130	588	0.0	641
ABCDEF_41288	UniRef50_Q9SB58	0.0	Normal: 307	183	332	637	649	322	BlastAnno: 1403	UniRef50_Q9SB58	407	67.56	410	110	6	1401	184	15	405	0.0	523
ABCDEF_33461	UniRef50_Q9ZVN2	0.0	Normal: 142	84	140	206	283	119	BlastAnno: 2904	UniRef50_Q9ZVN2	704	60.98	574	201	6	2271	559	83	636	0.0	686
ABCDEF_14334	UniRef50_Q9SYZ0	0.0	Normal: 2263	129	1724	3469	866	283	BlastAnno: 2039	UniRef50_Q9SYZ0	493	63.22	484	171	2	369	1802	5	487	0.0	627
ABCDEF_24844	UniRef50_Q9FF86	0.0	Normal: 1857	3686	2657	21940	11467	4090	BlastAnno: 1640	UniRef50_Q9FF86	484	66.89	447	135	6	1492	185	2	446	0.0	575
ABCDEF_51190	UniRef50_Q52T38	0.0	Normal: 145	169	142	210	232	335	BlastAnno: 1891	UniRef50_Q52T38	620	70.04	514	141	3	73	1608	86	588	0.0	726
ABCDEF_14085	UniRef50_Q8L5Y5	0.0	Normal: 353	41	384	527	331	280	BlastAnno: 2362	UniRef50_Q8L5Y5	718	62.07	704	220	12	2360	348	24	713	0.0	676
ABCDEF_33458	UniRef50_Q9LW26	0.0	Normal: 158	78	130	240	342	106	BlastAnno: 2822	UniRef50_Q9LW26	701	46.72	655	302	5	400	2337	85	701	0.0	568
ABCDEF_25193	UniRef50_Q9C6L5	0.0	Normal: 3256	368	3548	6230	5109	1288	BlastAnno: 1786	UniRef50_Q9C6L5	492	65.83	480	156	4	1668	241	17	492	0.0	668
ABCDEF_33457	UniRef50_Q9ZVN2	0.0	Normal: 168	83	137	250	375	112	BlastAnno: 2823	UniRef50_Q9ZVN2	704	65.12	496	168	3	379	1860	83	575	0.0	648
ABCDEF_7634	UniRef50_Q9C6L5	0.0	Normal: 2543	9303	2711	11578	5533	10032	BlastAnno: 2064	UniRef50_Q9C6L5	492	52.76	489	225	3	1734	268	1	483	0.0	535
ABCDEF_299	UniRef50_Q71N54	0.0	Normal: 569	1147	511	900	649	1879	BlastAnno: 2017	UniRef50_Q71N54	535	61.73	533	198	5	338	1924	4	534	0.0	677
ABCDEF_27086	UniRef50_Q52T38	0.0	Normal: 784	470	617	843	893	976	BlastAnno: 2398	UniRef50_Q52T38	620	70.80	637	165	4	225	2129	1	618	0.0	887
ABCDEF_51188	UniRef50_Q52T38	0.0	Normal: 273	327	255	415	349	441	BlastAnno: 2324	UniRef50_Q52T38	620	71.30	568	150	3	2041	344	32	588	0.0	815
ABCDEF_44812	UniRef50_Q6DR03	0.0	Normal: 982	902	1005	1071	959	2999	BlastAnno: 2450	UniRef50_Q6DR03	565	54.97	644	198	17	2142	220	1	555	0.0	540

2.7.9.-(Transferases;)
No Correspond Unigene
2.8.3.8(acetate CoA-transferase;)
No Correspond Unigene
3.1.1.-(Hydrolases;)

ABCDEF_25737	UniRef50_Q9CA68	0.0	Normal: 104	11	8	393	426	18	BlastAnno: 1232	UniRef50_Q9CA68	366	69.89	352	103	2	1182	130	17	366	0.0	522
ABCDEF_525	UniRef50_Q9LIN2	0.0	Normal: 213	16	167	378	456	158	BlastAnno: 1254	UniRef50_Q9LIN2	380	69.27	358	110	0	1097	24	23	380	0.0	535
ABCDEF_28091	UniRef50_O23522	0.0	Normal: 1192	678	424	81	156	3170	BlastAnno: 1887	UniRef50_O23522	601	57.90	525	191	12	168	1721	3	504	0.0	568
ABCDEF_13432	UniRef50_Q3ECP6	0.0	Normal: 712	1725	466	588	811	6988	BlastAnno: 2479	UniRef50_Q3ECP6	408	66.83	401	104	2	2397	1282	8	408	0.0	567
ABCDEF_72624	UniRef50_Q3ECP6	0.0	Normal: 22	38	25	267	382	149	BlastAnno: 1342	UniRef50_Q3ECP6	408	66.83	401	104	2	53	1168	8	408	0.0	567
ABCDEF_4432	UniRef50_B9IQA0	0.0	Normal: 680	298	798	834	1447	644	BlastAnno: 1533	UniRef50_B9IQA0	354	78.82	321	68	0	214	1176	33	353	0.0	556

3.1.1.57(2-pyrone-4,6-dicarboxylate lactonase)
No Correspond Unigene
3.1.3.1(alkaline phosphatase;)

ABCDEF_87884	UniRef50_P05186	1e-14	Normal: 0	0	0	0	0	16	BlastAnno: 228	UniRef50_P05186	524	46.15	78	40	1	1	228	411	488	1e-14	73.9

3.1.3.2(acid phosphatase;)

ABCDEF_48781	UniRef50_Q9LXI4	0.0	Normal: 3	205	0	171	142	375	BlastAnno: 1533	UniRef50_Q9LXI4	437	61.07	429	162	2	79	1365	5	428	0.0	540
ABCDEF_31834	UniRef50_Q9LJU7	0.0	Normal: 669	384	448	538	533	426	BlastAnno: 1818	UniRef50_Q9LJU7	437	64.83	435	148	3	344	1642	6	437	0.0	602
ABCDEF_41630	UniRef50_Q949Y3	0.0	Normal: 325	632	262	126	318	1578	BlastAnno: 1864	UniRef50_Q949Y3	475	61.81	432	162	2	1475	183	22	451	0.0	563

ABCDEF_28194	UniRef50_Q949Y3	0.0	Normal: 933	1694	869	1449	1788	3440	BlastAnno: 1940	UniRef50_Q949Y3	475	80.67	450	87	0	1654	305	20	469	0.0	759
ABCDEF_41635	UniRef50_Q949Y3	0.0	Normal: 1046	543	888	1413	1679	1200	BlastAnno: 1824	UniRef50_Q949Y3	475	59.45	439	169	4	1513	200	21	451	0.0	540
ABCDEF_45098	UniRef50_Q949Y3	0.0	Normal: 349	5170	534	294	420	27111	BlastAnno: 1905	UniRef50_Q949Y3	475	56.86	459	184	6	156	1520	4	452	0.0	537
ABCDEF_27775	UniRef50_Q949Y3	0.0	Normal: 108	180	98	136	195	803	BlastAnno: 1429	UniRef50_Q949Y3	475	60.51	428	166	2	1427	147	26	451	0.0	540
3.1.3.41(4-nitrophenylphosphatase;)																					
ABCDEF_41255	UniRef50_A8IG47	7e-77	Normal: 159	196	203	340	522	356	BlastAnno: 1248	UniRef50_A8IG47	304	48.73	275	138	2	1056	235	11	283	7e-77	249
3.1.8.1(aryldialkylphosphatase;)																					
No Correspond Unigene																					
3.5.1.4(amidase;)																					
ABCDEF_3151	UniRef50_B9RF45	0.0	Normal: 113	275	72	56	28	1014	BlastAnno: 1850	UniRef50_B9RF45	500	68.47	425	133	1	131	1402	68	492	0.0	547
ABCDEF_81937	UniRef50_B9RF45	0.0	Normal: 101	261	68	53	28	927	BlastAnno: 1739	UniRef50_B9RF45	500	68.47	425	133	1	131	1402	68	492	0.0	546
3.5.5.1(nitrilase;)																					
ABCDEF_3989	UniRef50_P46011	0.0	Normal: 806	816	966	1114	1416	1512	BlastAnno: 1581	UniRef50_P46011	355	79.10	354	71	3	255	1310	3	355	0.0	579
3.6.1.7(acylphosphatase;)																					
ABCDEF_18227	UniRef50_C5Z842	3e-28	Normal: 191	173	224	225	257	298	BlastAnno: 956	UniRef50_C5Z842	142	62.37	93	33	2	573	301	50	142	3e-28	114
3.7.1.-(Hydrolases;)																					
No Correspond Unigene																					
4.1.1.46(o-pyrocatechuate decarboxylase;)																					
No Correspond Unigene																					
4.1.1.59(gallate decarboxylase;)																					
No Correspond Unigene																					
4.1.1.61(4-hydroxybenzoate decarboxylase;)																					
No Correspond Unigene																					
4.1.1.63(protocatechuate decarboxylase;)																					
No Correspond Unigene																					
4.1.1.7(benzoylformate decarboxylase;)																					
No Correspond Unigene																					
4.2.1.17(enoyl-CoA hydratase;)																					
ABCDEF_21259	UniRef50_Q8W1L6	0.0	Normal: 272	276	284	384	326	305	BlastAnno: 2641	UniRef50_Q8W1L6	726	47.57	721	375	3	114	2267	6	726	0.0	633
ABCDEF_80649	UniRef50_Q8W1L6	0.0	Normal: 527	841	443	1308	1321	1188	BlastAnno: 2586	UniRef50_Q8W1L6	726	60.39	712	281	1	2403	268	1	711	0.0	818
ABCDEF_466	UniRef50_Q8W1L6	0.0	Normal: 1014	1690	1094	1677	2438	2080	BlastAnno: 2453	UniRef50_Q8W1L6	726	71.24	699	201	0	2368	272	7	705	0.0	954
ABCDEF_54834	UniRef50_Q8W1L6	0.0	Normal: 229	220	237	298	270	261	BlastAnno: 2405	UniRef50_Q8W1L6	726	47.52	606	315	3	274	2082	121	726	0.0	580
ABCDEF_34756	UniRef50_Q8W1L6	0.0	Normal: 776	726	768	2922	4039	1079	BlastAnno: 2469	UniRef50_Q8W1L6	726	68.93	708	217	2	2396	273	7	711	0.0	957
ABCDEF_4198	UniRef50_Q8W1L6	0.0	Normal: 609	929	532	1401	1568	1297	BlastAnno: 2667	UniRef50_Q8W1L6	726	60.45	713	281	1	2463	325	1	712	0.0	820
4.2.1.84(nitrile hydratase;)																					
No Correspond Unigene																					
5.1.2.2(mandelate racemase)																					
No Correspond Unigene																					
5.4.4.1(No Name)																					
No Correspond Unigene																					
5.5.1.-(Isomerases;)																					
No Correspond Unigene																					
6.2.1.-(Ligases;)																					
ABCDEF_7378	UniRef50_Q9FFE6	0.0	Normal: 1022	1422	746	576	680	1408	BlastAnno: 2117	UniRef50_Q9FFE6	552	67.57	552	167	5	144	1778	1	547	0.0	768
ABCDEF_47513	UniRef50_Q8RU95	0.0	Normal: 411	609	570	233	749	887	BlastAnno: 2280	UniRef50_Q8RU95	598	53.15	572	246	6	119	1777	28	596	0.0	543
ABCDEF_78742	UniRef50_Q84P25	0.0	Normal: 236	207	373	576	450	306	BlastAnno: 1534	UniRef50_Q84P25	565	60.39	462	168	5	1413	49	19	472	0.0	546
ABCDEF_10753	UniRef50_Q9FFE6	0.0	Normal: 394	401	336	253	292	348	BlastAnno: 1841	UniRef50_Q9FFE6	552	51.00	551	259	5	117	1751	1	546	0.0	553
ABCDEF_4431	UniRef50_Q9FFE6	0.0	Normal: 27	358	28	98	112	914	BlastAnno: 2075	UniRef50_Q9FFE6	552	50.71	560	264	7	1774	107	1	552	0.0	551
ABCDEF_38213	UniRef50_Q84P25	0.0	Normal: 342	337	597	798	722	505	BlastAnno: 1939	UniRef50_Q84P25	565	62.34	555	194	5	1818	175	19	565	0.0	690
ABCDEF_25221	UniRef50_Q0DV32	0.0	Normal: 971	826	1003	1543	1442	1545	BlastAnno: 2009	UniRef50_Q0DV32	552	57.20	542	226	4	183	1793	9	549	0.0	613
ABCDEF_23413	UniRef50_Q84P23	0.0	Normal: 123	237	158	149	213	658	BlastAnno: 3175	UniRef50_Q84P23	562	51.74	547	243	8	3030	1435	15	555	0.0	555
ABCDEF_20486	UniRef50_Q9FFE6	0.0	Normal: 580	912	312	324	864	1565	BlastAnno: 3530	UniRef50_Q9FFE6	552	66.61	557	171	5	352	1995	1	551	0.0	781
6.2.1.25(benzoate---CoA ligase;)																					
ABCDEF_7378	UniRef50_Q9SS01	0.0	Normal: 1022	1422	746	576	680	1408	BlastAnno: 2117	UniRef50_Q9SS01	580	50.09	541	250	7	198	1769	19	556	0.0	555
ABCDEF_10753	UniRef50_Q9SS01	0.0	Normal: 394	401	336	253	292	348	BlastAnno: 1841	UniRef50_Q9SS01	580	48.93	562	262	6	117	1745	1	556	0.0	542
ABCDEF_4431	UniRef50_Q9SS01	0.0	Normal: 27	358	28	98	112	914	BlastAnno: 2075	UniRef50_Q9SS01	580	48.14	565	266	6	1774	134	1	556	0.0	539
ABCDEF_20486	UniRef50_Q9SS01	0.0	Normal: 580	912	312	324	864	1565	BlastAnno: 3530	UniRef50_Q9SS01	580	48.92	558	266	6	352	1974	1	556	0.0	556
6.2.1.27(4-hydroxybenzoate---CoA ligase;)																					
No Correspond Unigene																					
6.2.1.32(anthranilate---CoA ligase;)																					
No Correspond Unigene																					
6.4.1.-(Ligases;)																					
No Correspond Unigene																					
>00630 Glyoxylate and dicarboxylate metabolism																					
1.1.1.26(glyoxylate reductase;)																					
ABCDEF_38443	UniRef50_A7HM61	5e-65	Normal: 996	6551	645	477	900	18941	BlastAnno: 1745	UniRef50_A7HM61	317	42.90	331	165	9	374	1363	2	309	5e-65	223
1.1.1.29(glycerate dehydrogenase;)																					
ABCDEF_38443	UniRef50_P13443	0.0	Normal: 996	6551	645	477	900	18941	BlastAnno: 1745	UniRef50_P13443	382	89.21	380	41	0	326	1465	1	380	0.0	679
1.1.1.36(acetoacetyl-CoA reductase;)																					
ABCDEF_34806	UniRef50_B7QGZ9	3e-25	Normal: 139	128	127	302	264	325	BlastAnno: 873	UniRef50_B7QGZ9	273	39.19	148	78	1	177	584	105	252	3e-25	108
1.1.1.37(malate dehydrogenase;)																					
ABCDEF_25169	UniRef50_Q9SN86	0.0	Normal: 938	614	1116	1317	4446	973	BlastAnno: 1856	UniRef50_Q9SN86	403	75.50	400	88	2	1616	417	2	391	0.0	585

ABCDEF_31746	UniRef50_Q9ZP05	0.0	Normal: 611	6985	354	98	71	19818	BlastAnno: 1631	UniRef50_Q9ZP05	354	83.38	349	54	2	274	1317	9	354	0.0	578
ABCDEF_32230	UniRef50_P57106	0.0	Normal: 8408	4627	7877	10286	18779	11078	BlastAnno: 1523	UniRef50_P57106	332	93.07	332	23	0	262	1257	1	332	0.0	617
ABCDEF_57415	UniRef50_Q9ZP05	0.0	Normal: 403	336	343	761	845	611	BlastAnno: 1190	UniRef50_Q9ZP05	354	83.29	353	54	2	1058	3	1	349	0.0	579
ABCDEF_17941	UniRef50_Q9ZP05	0.0	Normal: 616	554	586	1025	1231	910	BlastAnno: 1567	UniRef50_Q9ZP05	354	83.63	342	51	2	1336	314	17	354	0.0	563
1.1.1.60(2-hydroxy-3-oxopropionate reductase;)																					
ABCDEF_25589	UniRef50_Q1K1W6	7e-92	Normal: 1440	1755	1664	2327	3041	3552	BlastAnno: 1236	UniRef50_Q1K1W6	291	50.87	289	142	0	86	952	1	289	7e-92	287
1.1.1.77(lactaldehyde reductase;)																					
No Correspond Unigene																					
1.1.1.79(glyoxylate reductase (NADP+);)																					
No Correspond Unigene																					
1.1.1.81(hydroxypyruvate reductase;)																					
No Correspond Unigene																					
1.1.1.92(oxaloglycolate reductase (decarboxylating))																					
No Correspond Unigene																					
1.1.1.93(tartrate dehydrogenase;)																					
No Correspond Unigene																					
1.1.3.15(No Name)																					
ABCDEF_14667	UniRef50_P05414	0.0	Normal: 582	5865	429	399	781	17819	BlastAnno: 1729	UniRef50_P05414	369	89.42	359	38	0	1324	248	11	369	0.0	654
ABCDEF_44477	UniRef50_P05414	0.0	Normal: 130	143	73	78	172	264	BlastAnno: 1556	UniRef50_P05414	369	83.38	367	59	1	265	1359	3	369	0.0	634
ABCDEF_35107	UniRef50_P05414	0.0	Normal: 536	8711	472	259	172	18582	BlastAnno: 1476	UniRef50_P05414	369	91.34	358	31	0	1347	274	2	359	0.0	666
1.1.99.14(glycolate dehydrogenase;)																					
No Correspond Unigene																					
1.2.1.17(glyoxylate dehydrogenase (acylating))																					
No Correspond Unigene																					
1.2.1.2(formate dehydrogenase;)																					
ABCDEF_60816	UniRef50_P33160	7e-111	Normal: 1383	1133	1488	2129	3432	1561	BlastAnno: 1303	UniRef50_P33160	401	51.38	327	159	0	116	1096	49	375	7e-111	341
1.2.1.21(glycolaldehyde dehydrogenase;)																					
No Correspond Unigene																					
1.2.2.1(formate dehydrogenase (cytochrome);)																					
No Correspond Unigene																					
1.2.3.4(oxalate oxidase;)																					
No Correspond Unigene																					
1.2.3.5(glyoxylate oxidase)																					
No Correspond Unigene																					
1.3.1.7(meso-tartrate dehydrogenase)																					
No Correspond Unigene																					
2.2.1.5(2-hydroxy-3-oxoadipate synthase;)																					
No Correspond Unigene																					
2.3.1.9(acetyl-CoA C-acetyltransferase;)																					
ABCDEF_34825	UniRef50_Q8S4Y1	0.0	Normal: 640	698	620	807	839	1164	BlastAnno: 1773	UniRef50_Q8S4Y1	403	78.11	402	87	1	1597	395	2	403	0.0	598
ABCDEF_14629	UniRef50_Q8S4Y1	0.0	Normal: 634	316	650	1021	1278	691	BlastAnno: 1514	UniRef50_Q8S4Y1	403	85.11	403	60	0	1367	159	1	403	0.0	620
ABCDEF_107	UniRef50_Q8S4Y1	0.0	Normal: 332	460	460	842	893	424	BlastAnno: 1214	UniRef50_Q8S4Y1	403	84.07	339	54	0	1212	196	42	380	0.0	596
ABCDEF_14630	UniRef50_Q8S4Y1	0.0	Normal: 562	282	568	959	1173	627	BlastAnno: 1796	UniRef50_Q8S4Y1	403	85.50	400	58	0	436	1635	4	403	0.0	642
2.3.3.1(citrate (Si)-synthase;)																					
ABCDEF_734	UniRef50_O75390	0.0	Normal: 720	410	930	1540	2753	660	BlastAnno: 1840	UniRef50_O75390	466	61.59	440	168	1	242	1558	27	466	0.0	589
ABCDEF_735	UniRef50_O75390	0.0	Normal: 781	455	1022	1706	2969	688	BlastAnno: 1784	UniRef50_O75390	466	61.59	440	168	1	1606	290	27	466	0.0	589
ABCDEF_795	UniRef50_Q9LXS6	0.0	Normal: 1322	1369	1619	3657	3459	1871	BlastAnno: 1908	UniRef50_Q9LXS6	514	84.68	483	71	1	1775	327	34	513	0.0	832
2.3.3.12(3-propylmalate synthase;)																					
No Correspond Unigene																					
2.3.3.7(3-ethylmalate synthase;)																					
No Correspond Unigene																					
2.3.3.9(malate synthase;)																					
ABCDEF_16096	UniRef50_P08216	1e-61	Normal: 12	0	12	0	0	26	BlastAnno: 316	UniRef50_P08216	568	89.52	105	11	0	2	316	237	341	1e-61	204
2.7.1.31(glycerate kinase;)																					
ABCDEF_10301	UniRef50_Q944I4	0.0	Normal: 259	552	306	527	545	1533	BlastAnno: 1595	UniRef50_Q944I4	456	72.85	372	99	1	272	1387	86	455	0.0	580
ABCDEF_10303	UniRef50_Q944I4	0.0	Normal: 266	510	294	565	522	1374	BlastAnno: 1563	UniRef50_Q944I4	456	68.43	396	99	2	1292	105	86	455	0.0	565
2.7.2.6(formate kinase)																					
No Correspond Unigene																					
2.8.3.2(oxalate CoA-transferase;)																					
No Correspond Unigene																					
3.1.2.10(formyl-CoA hydrolase;)																					
No Correspond Unigene																					
3.1.3.18(phosphoglycolate phosphatase;)																					
ABCDEF_47957	UniRef50_B7GAG2	0.0	Normal: 899	493	1119	1449	1970	1173	BlastAnno: 2458	UniRef50_B7GAG2	623	69.82	623	164	4	2288	492	1	623	0.0	904
ABCDEF_71572	UniRef50_B7GAG2	0.0	Normal: 217	128	215	297	332	166	BlastAnno: 1404	UniRef50_B7GAG2	623	64.85	441	131	4	6	1256	140	580	0.0	576
ABCDEF_44666	UniRef50_B7GAG2	0.0	Normal: 744	424	834	996	1117	691	BlastAnno: 2381	UniRef50_B7GAG2	623	69.53	617	164	4	2108	330	1	617	0.0	890
ABCDEF_47956	UniRef50_B7GAG2	0.0	Normal: 896	509	1067	1388	1908	1181	BlastAnno: 2537	UniRef50_B7GAG2	623	69.82	623	164	4	2366	570	1	623	0.0	905
3.3.2.4(trans-epoxysuccinate hydrolase;)																					
No Correspond Unigene																					
3.5.1.10(formyltetrahydrofolate deformylase)																					
ABCDEF_11642	UniRef50_B9SXD7	5e-153	Normal: 361	592	520	793	672	2287	BlastAnno: 1364	UniRef50_B9SXD7	341	77.42	279	60	2	113	949	4	279	5e-153	447
3.5.1.27(N-formylmethionylaminoacyl-tRNA deformylase)																					

No Correspond Unigene																					
3.5.1.31(formylmethionine deformylase)																					
ABCDEF_3670	UniRef50_Q8I372	9e-44	Normal: 111	170	161	150	181	472	BlastAnno: 1063	UniRef50_Q8I372	241	47.02	168	88	1	279	779	65	232	9e-44	159
3.5.1.49(formamidase)																					
ABCDEF_60429	UniRef50_Q9SZE9	0.0	Normal: 13	40	13	16	7	67	BlastAnno: 1646	UniRef50_Q9SZE9	432	70.39	456	93	4	108	1421	1	432	0.0	619
ABCDEF_23263	UniRef50_Q9SZE9	0.0	Normal: 18	37	10	15	9	48	BlastAnno: 1541	UniRef50_Q9SZE9	432	77.32	366	63	1	1405	308	1	346	0.0	570
ABCDEF_23268	UniRef50_Q9SZE9	0.0	Normal: 19	60	15	18	9	96	BlastAnno: 1677	UniRef50_Q9SZE9	432	68.42	456	102	4	139	1452	1	432	0.0	592
ABCDEF_63714	UniRef50_Q9SZE9	0.0	Normal: 24	55	17	24	11	83	BlastAnno: 1689	UniRef50_Q9SZE9	432	80.24	420	63	1	108	1367	1	400	0.0	689
ABCDEF_23269	UniRef50_Q9SZE9	0.0	Normal: 59	149	58	65	24	188	BlastAnno: 1719	UniRef50_Q9SZE9	432	78.54	452	77	1	139	1494	1	432	0.0	719
3.5.1.56(N,N-dimethylformamidase;)																					
No Correspond Unigene																					
3.5.1.68(N-formylglutamate deformylase;)																					
No Correspond Unigene																					
3.5.1.8(formylaspartate deformylase;)																					
No Correspond Unigene																					
3.5.1.9(arylformamidase;)																					
No Correspond Unigene																					
4.1.1.2(oxalate decarboxylase;)																					
No Correspond Unigene																					
4.1.1.39(ribulose-bisphosphate carboxylase;)																					
ABCDEF_38101	UniRef50_Q8WKC9	0.0	Normal: 193	868	327	479	300	4925	BlastAnno: 1900	UniRef50_Q8WKC9	418	95.22	418	20	0	334	1587	1	418	0.0	798
4.1.1.40(hydroxypyruvate decarboxylase;)																					
No Correspond Unigene																					
4.1.1.47(tartronate-semialdehyde synthase;)																					
No Correspond Unigene																					
4.1.1.54(dihydroxyfumarate decarboxylase;)																					
No Correspond Unigene																					
4.1.1.8(oxalyl-CoA decarboxylase;)																					
ABCDEF_24878	UniRef50_P40149	4e-132	Normal: 748	881	856	1340	1426	1176	BlastAnno: 2328	UniRef50_P40149	568	41.91	556	295	10	359	2002	25	560	4e-132	413
4.1.3.1(isocitrate lyase;)																					
ABCDEF_16096	UniRef50_Q10663	4e-32	Normal: 12	0	12	0	0	26	BlastAnno: 316	UniRef50_Q10663	968	54.81	104	47	0	2	313	652	755	4e-32	125
4.1.3.13(oxalomalate lyase;)																					
No Correspond Unigene																					
4.1.3.16(4-hydroxy-2-oxoglutarate aldolase;)																					
ABCDEF_58429	UniRef50_A9F631	9e-18	Normal: 6	1	0	0	8	25	BlastAnno: 519	UniRef50_A9F631	223	32.50	160	89	2	26	505	25	165	9e-18	83.6
4.1.3.24(malyl-CoA lyase;)																					
No Correspond Unigene																					
4.2.1.3(aconitate hydratase;)																					
ABCDEF_73976	UniRef50_Q90875	0.0	Normal: 1872	1426	2211	5893	8147	1851	BlastAnno: 3209	UniRef50_Q90875	889	61.77	892	335	3	3045	370	3	888	0.0	1114
ABCDEF_35907	UniRef50_Q90875	0.0	Normal: 1591	876	1677	4081	5832	1425	BlastAnno: 3358	UniRef50_Q90875	889	61.84	891	334	3	3181	509	3	887	0.0	1109
ABCDEF_17819	UniRef50_Q90875	0.0	Normal: 1842	942	1937	4916	6347	1527	BlastAnno: 3866	UniRef50_Q90875	889	61.84	891	334	3	3181	509	3	887	0.0	1110
ABCDEF_52401	UniRef50_Q90875	0.0	Normal: 2227	1825	2713	7829	9659	2079	BlastAnno: 3665	UniRef50_Q90875	889	61.77	892	335	3	3045	370	3	888	0.0	1114
4.2.1.32(L(+)-tartrate dehydratase;)																					
No Correspond Unigene																					
4.2.1.55(3-hydroxybutyryl-CoA dehydratase;)																					
ABCDEF_31573	UniRef50_C0QBE8	2e-44	Normal: 159	151	158	345	356	262	BlastAnno: 1094	UniRef50_C0QBE8	240	40.00	220	124	3	228	875	11	226	2e-44	160
5.1.2.5(tartrate epimerase;)																					
No Correspond Unigene																					
5.1.99.1(methylmalonyl-CoA epimerase;)																					
No Correspond Unigene																					
5.3.1.22(hydroxypyruvate isomerase)																					
No Correspond Unigene																					
5.4.99.2(methylmalonyl-CoA mutase;)																					
No Correspond Unigene																					
6.2.1.8(oxalate---CoA ligase;)																					
No Correspond Unigene																					
6.2.1.9(malate---CoA ligase;)																					
ABCDEF_10877	UniRef50_P53595	5e-94	Normal: 1436	691	1429	2257	2843	867	BlastAnno: 1600	UniRef50_P53595	296	52.04	294	137	2	1323	451	2	294	5e-94	297
6.4.1.3(propionyl-CoA carboxylase;)																					
ABCDEF_21606	UniRef50_Q2S388	2e-150	Normal: 386	375	365	562	565	924	BlastAnno: 2365	UniRef50_Q2S388	698	45.92	527	261	3	251	1768	152	675	2e-150	465
>00633 Nitrotoluene degradation																					
1.-.-.-(Oxidoreductases)																					
ABCDEF_345	UniRef50_Q6Z690	0.0	Normal: 442	146	449	340	272	314	BlastAnno: 1658	UniRef50_Q6Z690	849	63.42	462	150	5	1657	314	387	843	0.0	589
ABCDEF_24068	UniRef50_Q9FI41	0.0	Normal: 214	316	207	270	263	849	BlastAnno: 1742	UniRef50_Q9FI41	387	73.28	363	96	1	1440	355	4	366	0.0	554
ABCDEF_24070	UniRef50_Q9FI41	0.0	Normal: 189	290	182	231	232	751	BlastAnno: 1479	UniRef50_Q9FI41	387	73.28	363	96	1	1363	278	4	366	0.0	551
ABCDEF_13957	UniRef50_Q9CAE3	0.0	Normal: 399	318	392	489	456	586	BlastAnno: 2488	UniRef50_Q9CAE3	789	80.85	496	93	2	3	1487	285	779	0.0	768
ABCDEF_28410	UniRef50_Q9LIG0	0.0	Normal: 211	366	234	286	433	1504	BlastAnno: 1324	UniRef50_Q9LIG0	330	74.92	331	76	2	1147	173	1	330	0.0	521
ABCDEF_17581	UniRef50_Q8LGI2	0.0	Normal: 758	336	735	426	374	852	BlastAnno: 1543	UniRef50_Q8LGI2	454	68.78	442	136	2	71	1390	8	449	0.0	597
ABCDEF_23753	UniRef50_Q94A68	0.0	Normal: 284	559	215	224	164	519	BlastAnno: 1580	UniRef50_Q94A68	377	81.90	348	63	0	1349	306	30	377	0.0	576
ABCDEF_24071	UniRef50_Q9FI41	0.0	Normal: 235	377	230	278	289	948	BlastAnno: 2090	UniRef50_Q9FI41	387	73.28	363	96	1	1726	641	4	366	0.0	553
1.1.1.-(Oxidoreductases;)																					
ABCDEF_13453	UniRef50_Q9SYM5	0.0	Normal: 225	226	398	1950	2026	421	BlastAnno: 2578	UniRef50_Q9SYM5	669	86.61	672	87	2	298	2313	1	669	0.0	1226

ABCDEF_17342 UniRef50_Q84M96 0.0 Normal: 274 406 272 327 439 625 BlastAnno: 1277 UniRef50_Q84M96 346 73.33 345 91 1 1187 156 2 346 0.0 546
ABCDEF_31147 UniRef50_Q9SYM5 0.0 Normal: 4571 1591 1491 1401 1939 4327 BlastAnno: 2465 UniRef50_Q9SYM5 669 82.76 673 107 4 2397 394 1 669 0.0 1154
ABCDEF_17344 UniRef50_Q84M96 0.0 Normal: 252 373 257 297 380 540 BlastAnno: 1350 UniRef50_Q84M96 346 68.19 371 91 2 1263 154 2 346 0.0 530
ABCDEF_44674 UniRef50_Q9SYM5 0.0 Normal: 3581 2620 4329 9505 11447 4930 BlastAnno: 2634 UniRef50_Q9SYM5 669 87.80 672 78 3 253 2265 1 669 0.0 1241

1.12.99.6(hydrogenase (acceptor);)
No Correspond Unigene
1.13.11.-(Oxidoreductases;)
No Correspond Unigene
1.13.12.-(Oxidoreductases;)
No Correspond Unigene
1.14.12.-(Oxidoreductases;)
No Correspond Unigene
1.2.7.1(pyruvate synthase;)
No Correspond Unigene
1.2.99.2(carbon-monoxide dehydrogenase (acceptor);)
No Correspond Unigene
1.7.1.-(Oxidoreductases;)
No Correspond Unigene
1.8.99.3(hydrogensulfite reductase;)
No Correspond Unigene
2.3.1.5(arylamine N-acetyltransferase;)
No Correspond Unigene
3.7.1.-(Hydrolases;)
No Correspond Unigene

>00640 Propanoate metabolism

1.1.1.27(L-lactate dehydrogenase;)
ABCDEF_38409 UniRef50_P22988 5e-157 Normal: 468 369 408 758 1349 382 BlastAnno: 1380 UniRef50_P22988 356 66.67 357 111 3 121 1170 1 356 5e-157 458

1.1.1.59(3-hydroxypropionate dehydrogenase)
No Correspond Unigene
1.1.1.80(isopropanol dehydrogenase (NADP+);)
No Correspond Unigene
1.1.2.8(alcohol dehydrogenase (cytochrome c);)
No Correspond Unigene
1.2.1.18(malonate-semialdehyde dehydrogenase (acetylating);)
ABCDEF_18282 UniRef50_Q02252 0.0 Normal: 1599 1252 1838 3819 3718 2949 BlastAnno: 2054 UniRef50_Q02252 535 60.20 510 197 2 345 1856 23 532 0.0 653

1.2.1.27(methylmalonate-semialdehyde dehydrogenase (acylating);)
ABCDEF_18282 UniRef50_B7GBU6 0.0 Normal: 1599 1252 1838 3819 3718 2949 BlastAnno: 2054 UniRef50_B7GBU6 537 59.35 492 191 2 402 1865 50 536 0.0 571

1.2.1.3(aldehyde dehydrogenase (NAD+);)
ABCDEF_52483 UniRef50_Q70E96 0.0 Normal: 28 851 41 0 3965 420 BlastAnno: 1750 UniRef50_Q70E96 484 54.56 482 219 0 107 1552 3 484 0.0 548
ABCDEF_17638 UniRef50_Q70E96 0.0 Normal: 580 718 749 2424 1317 775 BlastAnno: 1970 UniRef50_Q70E96 484 62.21 471 177 1 283 1692 2 472 0.0 625
ABCDEF_403 UniRef50_Q8W033 0.0 Normal: 69 355 98 39 223 1111 BlastAnno: 1749 UniRef50_Q8W033 550 65.96 473 161 0 1523 105 74 546 0.0 625
ABCDEF_45143 UniRef50_Q0WSF1 0.0 Normal: 1445 874 1200 1789 1940 1504 BlastAnno: 2450 UniRef50_Q0WSF1 596 79.97 594 115 2 2220 451 1 594 0.0 1015

1.2.7.1(pyruvate synthase;)
No Correspond Unigene
1.2.7.2(2-oxobutyrate synthase;)
No Correspond Unigene
1.2.99.3(aldehyde dehydrogenase (pyrroloquinoline-quinone);)
ABCDEF_25501 UniRef50_A5EIC3 1e-152 Normal: 1154 1584 1099 1850 736 3942 BlastAnno: 2012 UniRef50_A5EIC3 508 50.11 473 234 2 1698 280 32 502 1e-152 461

1.3.1.-(Oxidoreductases;)
ABCDEF_45552 UniRef50_P52581 0.0 Normal: 213 2112 262 1058 1208 1322 BlastAnno: 1284 UniRef50_P52581 312 80.45 312 61 0 185 1120 1 312 0.0 530
ABCDEF_47970 UniRef50_Q9T0J6 0.0 Normal: 1040 443 1171 941 1234 650 BlastAnno: 2557 UniRef50_Q9T0J6 700 60.52 699 232 9 2337 271 36 700 0.0 860

1.3.99.3(acyl-CoA dehydrogenase;)
ABCDEF_90276 UniRef50_P11310 4e-115 Normal: 0 0 0 0 0 80 BlastAnno: 757 UniRef50_P11310 421 66.14 251 79 2 3 755 80 324 4e-115 345

2.1.3.1(methylmalonyl-CoA carboxytransferase;)
No Correspond Unigene
2.3.1.54(formate C-acetyltransferase;)
No Correspond Unigene
2.3.1.8(phosphate acetyltransferase;)
ABCDEF_10009 UniRef50_B6KAY1 6e-23 Normal: 14 16 19 16 34 38 BlastAnno: 693 UniRef50_B6KAY1 1296 48.51 101 51 1 154 453 1128 1228 6e-23 104

2.3.1.9(acetyl-CoA C-acetyltransferase;)
ABCDEF_34825 UniRef50_Q8S4Y1 0.0 Normal: 640 698 620 807 839 1164 BlastAnno: 1773 UniRef50_Q8S4Y1 403 78.11 402 87 1 1597 395 2 403 0.0 598
ABCDEF_14629 UniRef50_Q8S4Y1 0.0 Normal: 634 316 650 1021 1278 691 BlastAnno: 1514 UniRef50_Q8S4Y1 403 85.11 403 60 0 1367 159 1 403 0.0 620
ABCDEF_107 UniRef50_Q8S4Y1 0.0 Normal: 332 460 460 842 893 424 BlastAnno: 1214 UniRef50_Q8S4Y1 403 84.07 339 54 0 1212 196 42 380 0.0 596
ABCDEF_14630 UniRef50_Q8S4Y1 0.0 Normal: 562 282 568 959 1173 627 BlastAnno: 1796 UniRef50_Q8S4Y1 403 85.50 400 58 0 436 1635 4 403 0.0 642

2.3.3.5(2-methylcitrate synthase;)
ABCDEF_735 UniRef50_Q9TEM3 2e-148 Normal: 781 455 1022 1706 2969 688 BlastAnno: 1784 UniRef50_Q9TEM3 460 51.03 435 202 5 1606 326 24 455 2e-148 445

2.6.1.18(beta-alanine---pyruvate transaminase;)
No Correspond Unigene
2.6.1.19(4-aminobutyrate transaminase;)
ABCDEF_3775 UniRef50_B8CY58 4e-110 Normal: 473 159 414 817 611 782 BlastAnno: 1801 UniRef50_B8CY58 437 45.37 432 223 7 1570 293 6 430 4e-110 346

2.7.2.1(acetate kinase;)
No Correspond Unigene

2.7.2.15(propionate kinase;)																					
No Correspond Unigene																					
2.8.3.1(propionate CoA-transferase;)																					
No Correspond Unigene																					
2.8.3.3(malonate CoA-transferase;)																					
No Correspond Unigene																					
2.8.3.8(acetate CoA-transferase;)																					
No Correspond Unigene																					
3.1.2.17(No Name)																					
No Correspond Unigene																					
3.1.2.4(3-hydroxyisobutyryl-CoA hydrolase;)																					
ABCDEF_56804	UniRef50_Q6NVY1	6e-77	Normal: 160	146	249	472	328	155	BlastAnno: 1439	UniRef50_Q6NVY1	386	40.45	356	199	7	257	1306	29	377	6e-77	254
3.5.99.7(1-aminocyclopropane-1-carboxylate deaminase;)																					
ABCDEF_28678	UniRef50_Q21K56	1e-67	Normal: 1546	1394	1804	2228	3149	3005	BlastAnno: 1576	UniRef50_Q21K56	336	40.72	334	176	8	1255	293	12	336	1e-67	229
4.1.1.4(acetoacetate decarboxylase;)																					
No Correspond Unigene																					
4.1.1.41(methylmalonyl-CoA decarboxylase;)																					
No Correspond Unigene																					
4.1.1.9(malonyl-CoA decarboxylase;)																					
ABCDEF_47117	UniRef50_C4KDD9	1e-90	Normal: 122	80	140	109	159	107	BlastAnno: 1629	UniRef50_C4KDD9	460	43.84	349	178	5	1266	226	124	456	1e-90	294
4.1.3.30(methylisocitrate lyase;)																					
ABCDEF_16200	UniRef50_Q9P6J1	5e-10	Normal: 0	0	14	55	0	0	BlastAnno: 356	UniRef50_Q9P6J1	518	35.43	127	61	4	330	1	57	179	5e-10	62.0
4.2.1.17(enoyl-CoA hydratase;)																					
ABCDEF_21259	UniRef50_Q8W1L6	0.0	Normal: 272	276	284	384	326	305	BlastAnno: 2641	UniRef50_Q8W1L6	726	47.57	721	375	3	114	2267	6	726	0.0	633
ABCDEF_80649	UniRef50_Q8W1L6	0.0	Normal: 527	841	443	1308	1321	1188	BlastAnno: 2586	UniRef50_Q8W1L6	726	60.39	712	281	1	2403	268	1	711	0.0	818
ABCDEF_466	UniRef50_Q8W1L6	0.0	Normal: 1014	1690	1094	1677	2438	2080	BlastAnno: 2453	UniRef50_Q8W1L6	726	71.24	699	201	0	2368	272	7	705	0.0	954
ABCDEF_54834	UniRef50_Q8W1L6	0.0	Normal: 229	220	237	298	270	261	BlastAnno: 2405	UniRef50_Q8W1L6	726	47.52	606	315	3	274	2082	121	726	0.0	580
ABCDEF_34756	UniRef50_Q8W1L6	0.0	Normal: 776	726	768	2922	4039	1079	BlastAnno: 2469	UniRef50_Q8W1L6	726	68.93	708	217	2	2396	273	7	711	0.0	957
ABCDEF_4198	UniRef50_Q8W1L6	0.0	Normal: 609	929	532	1401	1568	1297	BlastAnno: 2667	UniRef50_Q8W1L6	726	60.45	713	281	1	2463	325	1	712	0.0	820
4.2.1.27(acetylenecarboxylate hydratase;)																					
No Correspond Unigene																					
4.2.1.54(lactoyl-CoA dehydratase;)																					
No Correspond Unigene																					
4.2.1.79(2-methylcitrate dehydratase;)																					
No Correspond Unigene																					
4.2.1.99(2-methylisocitrate dehydratase;)																					
No Correspond Unigene																					
4.3.1.6(beta-alanyl-CoA ammonia-lyase;)																					
No Correspond Unigene																					
5.1.99.1(methylmalonyl-CoA epimerase;)																					
No Correspond Unigene																					
5.4.99.2(methylmalonyl-CoA mutase;)																					
No Correspond Unigene																					
6.2.1.-(Ligases;)																					
ABCDEF_7378	UniRef50_Q9FFE6	0.0	Normal: 1022	1422	746	576	680	1408	BlastAnno: 2117	UniRef50_Q9FFE6	552	67.57	552	167	5	144	1778	1	547	0.0	768
ABCDEF_47513	UniRef50_Q8RU95	0.0	Normal: 411	609	570	233	749	887	BlastAnno: 2280	UniRef50_Q8RU95	598	53.15	572	246	6	119	1777	28	596	0.0	543
ABCDEF_78742	UniRef50_Q84P25	0.0	Normal: 236	207	373	576	450	306	BlastAnno: 1534	UniRef50_Q84P25	565	60.39	462	168	5	1413	49	19	472	0.0	546
ABCDEF_10753	UniRef50_Q9FFE6	0.0	Normal: 394	401	336	253	292	348	BlastAnno: 1841	UniRef50_Q9FFE6	552	51.00	551	259	5	117	1751	1	546	0.0	553
ABCDEF_4431	UniRef50_Q9FFE6	0.0	Normal: 27	358	28	98	112	914	BlastAnno: 2075	UniRef50_Q9FFE6	552	50.71	560	264	7	1774	107	1	552	0.0	551
ABCDEF_38213	UniRef50_Q84P25	0.0	Normal: 342	337	597	798	722	505	BlastAnno: 1939	UniRef50_Q84P25	565	62.34	555	194	5	1818	175	19	565	0.0	690
ABCDEF_25221	UniRef50_Q0DV32	0.0	Normal: 971	826	1003	1543	1442	1545	BlastAnno: 2009	UniRef50_Q0DV32	552	57.20	542	226	4	183	1793	9	549	0.0	613
ABCDEF_23413	UniRef50_Q84P23	0.0	Normal: 123	237	158	149	213	658	BlastAnno: 3175	UniRef50_Q84P23	562	51.74	547	243	8	3030	1435	15	555	0.0	555
ABCDEF_20486	UniRef50_Q9FFE6	0.0	Normal: 580	912	312	324	864	1565	BlastAnno: 3530	UniRef50_Q9FFE6	552	66.61	557	171	5	352	1995	1	551	0.0	781
6.2.1.1(acetate---CoA ligase;)																					
ABCDEF_65173	UniRef50_Q9VP61	0.0	Normal: 208	202	219	336	383	409	BlastAnno: 2249	UniRef50_Q9VP61	670	54.63	540	236	3	539	2131	4	543	0.0	629
ABCDEF_51889	UniRef50_Q9VP61	0.0	Normal: 268	268	315	436	574	651	BlastAnno: 2148	UniRef50_Q9VP61	670	55.64	638	273	4	2060	174	26	662	0.0	723
ABCDEF_51888	UniRef50_A8JFR9	0.0	Normal: 348	343	408	583	709	775	BlastAnno: 2704	UniRef50_A8JFR9	667	56.57	647	273	5	2090	168	20	664	0.0	712
ABCDEF_59631	UniRef50_Q9VP61	0.0	Normal: 132	127	125	187	249	287	BlastAnno: 1733	UniRef50_Q9VP61	670	55.41	518	222	3	89	1615	26	543	0.0	610
6.2.1.13(acetate---CoA ligase (ADP-forming);)																					
No Correspond Unigene																					
6.2.1.17(propionate---CoA ligase;)																					
No Correspond Unigene																					
6.2.1.4(succinate---CoA ligase (GDP-forming);)																					
ABCDEF_45671	UniRef50_Q869S7	2e-148	Normal: 1899	1293	2339	3905	6798	2137	BlastAnno: 1668	UniRef50_Q869S7	420	57.14	399	166	3	230	1420	23	418	2e-148	442
6.2.1.5(succinate---CoA ligase (ADP-forming);)																					
ABCDEF_17836	UniRef50_O82662	0.0	Normal: 367	77	365	530	1198	144	BlastAnno: 1491	UniRef50_O82662	421	85.48	420	61	0	131	1390	1	420	0.0	741
ABCDEF_45671	UniRef50_O82662	0.0	Normal: 1899	1293	2339	3905	6798	2137	BlastAnno: 1668	UniRef50_O82662	421	87.38	420	53	0	164	1423	1	420	0.0	753
6.4.1.2(acetyl-CoA carboxylase;)																					
ABCDEF_27465	UniRef50_P78820	0.0	Normal: 4589	3602	6382	22310	7761	4482	BlastAnno: 7477	UniRef50_P78820	2280	40.33	2145	1127	43	7128	919	55	2121	0.0	1462
6.4.1.3(propionyl-CoA carboxylase;)																					
ABCDEF_21606	UniRef50_Q2S388	2e-150	Normal: 386	375	365	562	565	924	BlastAnno: 2365	UniRef50_Q2S388	698	45.92	527	261	3	251	1768	152	675	2e-150	465

>00642 Ethylbenzene degradation

1.1.-.-(Oxidoreductases;)

ABCDEF_17646	UniRef50_O34948	5e-83	Normal: 372	339	320	294	487	1376	BlastAnno: 1685	UniRef50_O34948	288	47.31	279	145	2	1523	690	5	282	5e-83	269

1.14.12.12(naphthalene 1,2-dioxygenase;)

No Correspond Unigene

1.17.99.2(ethylbenzene hydroxylase;)

No Correspond Unigene

1.3.1.66(cis-dihydroethylcatechol dehydrogenase)

No Correspond Unigene

2.3.1.-(Transferases;)

ABCDEF_520	UniRef50_Q9FF86	0.0	Normal: 1672	85	2273	5056	3383	1211	BlastAnno: 1811	UniRef50_Q9FF86	484	64.06	448	149	6	1664	354	3	449	0.0	573
ABCDEF_68664	UniRef50_Q9ZVN2	0.0	Normal: 79	35	85	107	193	63	BlastAnno: 1488	UniRef50_Q9ZVN2	704	63.99	411	142	3	46	1275	298	703	0.0	540
ABCDEF_52245	UniRef50_Q9FG87	0.0	Normal: 1878	635	1833	3814	1432	1376	BlastAnno: 1758	UniRef50_Q9FG87	529	55.35	486	204	4	4	1428	34	517	0.0	530
ABCDEF_16730	UniRef50_Q3EC11	0.0	Normal: 281	204	237	365	377	458	BlastAnno: 2092	UniRef50_Q3EC11	536	59.33	536	204	8	196	1785	3	530	0.0	661
ABCDEF_4655	UniRef50_A5BMG1	0.0	Normal: 2426	1856	3606	6958	4608	2755	BlastAnno: 2261	UniRef50_A5BMG1	481	68.37	528	112	10	263	1843	3	476	0.0	667
ABCDEF_13860	UniRef50_Q9C533	0.0	Normal: 588	220	570	1451	1338	310	BlastAnno: 2648	UniRef50_Q9C533	596	63.37	606	177	12	2226	511	1	595	0.0	558
ABCDEF_28294	UniRef50_Q9SYZ0	0.0	Normal: 1038	4070	1427	6745	877	2797	BlastAnno: 1785	UniRef50_Q9SYZ0	493	52.37	485	223	3	1598	162	5	487	0.0	533
ABCDEF_47718	UniRef50_Q9C533	0.0	Normal: 1599	236	1657	2093	1231	588	BlastAnno: 2680	UniRef50_Q9C533	596	65.02	626	165	14	2280	472	1	595	0.0	634
ABCDEF_45080	UniRef50_Q9SYZ0	0.0	Normal: 1005	592	1079	2360	3762	2217	BlastAnno: 2130	UniRef50_Q9SYZ0	493	57.85	484	197	3	302	1735	5	487	0.0	533
ABCDEF_25377	UniRef50_Q9C6L5	0.0	Normal: 37	2090	138	2573	1026	1298	BlastAnno: 1334	UniRef50_Q9C6L5	492	79.74	385	76	1	1155	1	6	388	0.0	654
ABCDEF_33470	UniRef50_Q9LW26	0.0	Normal: 172	98	156	260	391	149	BlastAnno: 2993	UniRef50_Q9LW26	701	48.96	627	301	5	655	2508	85	701	0.0	581
ABCDEF_40654	UniRef50_Q9HOA0	0.0	Normal: 638	213	530	318	318	286	BlastAnno: 2216	UniRef50_Q9HOA0	1025	56.87	721	279	6	2127	13	91	795	0.0	756
ABCDEF_33460	UniRef50_Q9ZVN2	0.0	Normal: 144	71	119	216	306	97	BlastAnno: 2691	UniRef50_Q9ZVN2	704	61.50	587	191	4	379	2133	83	636	0.0	715
ABCDEF_14442	UniRef50_Q9SYZ0	0.0	Normal: 341	684	439	1040	1601	552	BlastAnno: 1779	UniRef50_Q9SYZ0	493	63.12	480	171	2	1570	149	5	484	0.0	628
ABCDEF_52626	UniRef50_B6SYP9	0.0	Normal: 815	346	1131	3015	1537	108	BlastAnno: 1861	UniRef50_B6SYP9	494	55.02	438	189	3	1538	249	51	488	0.0	536
ABCDEF_644	UniRef50_Q9SB58	0.0	Normal: 704	444	913	969	1431	848	BlastAnno: 1777	UniRef50_Q9SB58	407	64.15	424	129	8	1572	316	1	406	0.0	554
ABCDEF_27087	UniRef50_Q52T38	0.0	Normal: 324	221	233	419	343	412	BlastAnno: 1631	UniRef50_Q52T38	620	75.95	420	91	1	1409	150	1	410	0.0	681
ABCDEF_34187	UniRef50_Q8L5Y5	0.0	Normal: 497	415	588	523	291	433	BlastAnno: 2677	UniRef50_Q8L5Y5	718	57.30	733	251	15	202	2274	1	713	0.0	683
ABCDEF_47966	UniRef50_Q9SYZ0	0.0	Normal: 577	934	326	580	682	3440	BlastAnno: 2177	UniRef50_Q9SYZ0	493	56.40	484	204	3	1780	347	5	487	0.0	535
ABCDEF_51189	UniRef50_Q52T38	0.0	Normal: 135	152	128	181	219	327	BlastAnno: 1766	UniRef50_Q52T38	620	68.09	470	137	3	80	1483	130	588	0.0	641
ABCDEF_41288	UniRef50_Q9SB58	0.0	Normal: 307	183	332	637	649	322	BlastAnno: 1403	UniRef50_Q9SB58	407	67.56	410	110	6	1401	184	15	405	0.0	523
ABCDEF_33461	UniRef50_Q9ZVN2	0.0	Normal: 142	84	140	206	283	119	BlastAnno: 2904	UniRef50_Q9ZVN2	704	60.98	574	201	6	2271	559	83	636	0.0	686
ABCDEF_14334	UniRef50_Q9SYZ0	0.0	Normal: 2263	129	1724	3469	866	283	BlastAnno: 2039	UniRef50_Q9SYZ0	493	63.22	484	171	2	369	1802	5	487	0.0	627
ABCDEF_24844	UniRef50_Q9FF86	0.0	Normal: 1857	3686	2657	21940	11467	4090	BlastAnno: 1640	UniRef50_Q9FF86	484	66.89	447	135	6	1492	185	2	446	0.0	575
ABCDEF_51190	UniRef50_Q52T38	0.0	Normal: 145	169	142	210	232	335	BlastAnno: 1891	UniRef50_Q52T38	620	70.04	514	141	3	73	1608	86	588	0.0	726
ABCDEF_14085	UniRef50_Q8L5Y5	0.0	Normal: 353	41	384	527	331	280	BlastAnno: 2362	UniRef50_Q8L5Y5	718	62.07	704	220	12	2360	348	24	713	0.0	676
ABCDEF_33458	UniRef50_Q9LW26	0.0	Normal: 158	78	130	240	342	106	BlastAnno: 2822	UniRef50_Q9LW26	701	46.72	655	302	5	400	2337	85	701	0.0	568
ABCDEF_25193	UniRef50_Q9C6L5	0.0	Normal: 3256	368	3548	6230	5109	1288	BlastAnno: 1786	UniRef50_Q9C6L5	492	65.83	480	156	4	1668	241	17	492	0.0	668
ABCDEF_33457	UniRef50_Q9ZVN2	0.0	Normal: 168	83	137	250	375	112	BlastAnno: 2823	UniRef50_Q9ZVN2	704	65.12	496	168	3	379	1860	83	575	0.0	648
ABCDEF_7634	UniRef50_Q9C6L5	0.0	Normal: 2543	9303	2711	11578	5533	10032	BlastAnno: 2064	UniRef50_Q9C6L5	492	52.76	489	225	3	1734	268	1	483	0.0	535
ABCDEF_299	UniRef50_Q71N54	0.0	Normal: 569	1147	511	900	649	1879	BlastAnno: 2017	UniRef50_Q71N54	535	61.73	533	198	5	338	1924	4	534	0.0	677
ABCDEF_27086	UniRef50_Q52T38	0.0	Normal: 784	470	617	843	893	976	BlastAnno: 2398	UniRef50_Q52T38	620	70.80	637	165	4	225	2129	1	618	0.0	887
ABCDEF_51188	UniRef50_Q52T38	0.0	Normal: 273	327	255	415	349	441	BlastAnno: 2324	UniRef50_Q52T38	620	71.30	568	150	3	2041	344	32	588	0.0	815
ABCDEF_44812	UniRef50_Q6DR03	0.0	Normal: 982	902	1005	1071	959	2999	BlastAnno: 2450	UniRef50_Q6DR03	565	54.97	644	198	17	2142	220	1	555	0.0	540

2.3.1.16(acetyl-CoA C-acyltransferase;)

ABCDEF_47228	UniRef50_P09110	1e-153	Normal: 321	497	306	408	1144	369	BlastAnno: 1559	UniRef50_P09110	424	59.95	387	152	3	1397	237	37	420	1e-153	454

3.7.1.-(Hydrolases;)

No Correspond Unigene

4.1.3.39(4-hydroxy-2-oxovalerate aldolase;)

No Correspond Unigene

4.2.1.80(2-oxopent-4-enoate hydratase;)

No Correspond Unigene

6.2.1.-(Ligases;)

ABCDEF_7378	UniRef50_Q9FFE6	0.0	Normal: 1022	1422	746	576	680	1408	BlastAnno: 2117	UniRef50_Q9FFE6	552	67.57	552	167	5	144	1778	1	547	0.0	768
ABCDEF_47513	UniRef50_Q8RU95	0.0	Normal: 411	609	570	233	749	887	BlastAnno: 2280	UniRef50_Q8RU95	598	53.15	572	246	6	119	1777	28	596	0.0	543
ABCDEF_78742	UniRef50_Q84P25	0.0	Normal: 236	207	373	576	450	306	BlastAnno: 1534	UniRef50_Q84P25	565	60.39	462	168	5	1413	49	19	472	0.0	546
ABCDEF_10753	UniRef50_Q9FFE6	0.0	Normal: 394	401	336	253	292	348	BlastAnno: 1841	UniRef50_Q9FFE6	552	51.00	551	259	5	117	1751	1	546	0.0	553
ABCDEF_4431	UniRef50_Q9FFE6	0.0	Normal: 27	358	28	98	112	914	BlastAnno: 2075	UniRef50_Q9FFE6	552	50.71	560	264	7	1774	107	1	552	0.0	551
ABCDEF_38213	UniRef50_Q84P25	0.0	Normal: 342	337	597	798	722	505	BlastAnno: 1939	UniRef50_Q84P25	565	62.34	555	194	5	1818	175	19	565	0.0	690
ABCDEF_25221	UniRef50_Q0DV32	0.0	Normal: 971	826	1003	1543	1442	1545	BlastAnno: 2009	UniRef50_Q0DV32	552	57.20	542	226	4	183	1793	9	549	0.0	613
ABCDEF_23413	UniRef50_Q84P23	0.0	Normal: 123	237	158	149	213	658	BlastAnno: 3175	UniRef50_Q84P23	562	51.74	547	243	8	3030	1435	15	555	0.0	555
ABCDEF_20486	UniRef50_Q9FFE6	0.0	Normal: 580	912	312	324	864	1565	BlastAnno: 3530	UniRef50_Q9FFE6	552	66.61	557	171	5	352	1995	1	551	0.0	781

6.4.1.-(Ligases;)

No Correspond Unigene

>00643 Styrene degradation

1.13.11.2(catechol 2,3-dioxygenase;)

No Correspond Unigene

1.13.11.5(homogentisate 1,2-dioxygenase;)

ABCDEF_7368	UniRef50_Q93099	6e-173	Normal: 260	416	205	300	283	1362	BlastAnno: 1849	UniRef50_Q93099	445	57.85	446	173	8	239	1552	2	440	6e-173	508

1.14.12.-(Oxidoreductases;)

No Correspond Unigene

1.14.13.-(Oxidoreductases;)

ABCDEF_45538	UniRef50_O23024	3e-156	Normal: 110	6	165	774	133	36	BlastAnno: 1258	UniRef50_O23024	437	58.18	385	151	3	3	1157	37	411	3e-156	457

1.14.13.63(3-hydroxyphenylacetate 6-hydroxylase;)																					
No Correspond Unigene																					
1.2.1.39(phenylacetaldehyde dehydrogenase)																					
No Correspond Unigene																					
1.3.1.19(cis-1,2-dihydrobenzene-1,2-diol dehydrogenase;)																					
No Correspond Unigene																					
2.8.3.1(propionate CoA-transferase;)																					
No Correspond Unigene																					
2.8.3.12(glutaconate CoA-transferase)																					
No Correspond Unigene																					
3.5.1.4(amidase;)																					
ABCDEF_3151	UniRef50_B9RF45	0.0	Normal: 113	275	72	56	28	1014	BlastAnno: 1850	UniRef50_B9RF45	500	68.47	425	133	1	131	1402	68	492	0.0	547
ABCDEF_81937	UniRef50_B9RF45	0.0	Normal: 101	261	68	53	28	927	BlastAnno: 1739	UniRef50_B9RF45	500	68.47	425	133	1	131	1402	68	492	0.0	546
3.5.5.1(nitrilase;)																					
ABCDEF_3989	UniRef50_P46011	0.0	Normal: 806	816	966	1114	1416	1512	BlastAnno: 1581	UniRef50_P46011	355	79.10	354	71	3	255	1310	3	355	0.0	579
3.5.5.7(aliphatic nitrilase)																					
No Correspond Unigene																					
3.7.1.2(fumarylacetoacetase;)																					
ABCDEF_25631	UniRef50_P16930	2e-172	Normal: 2583	1744	2457	3093	7463	3464	BlastAnno: 1676	UniRef50_P16930	419	58.89	416	167	3	1435	194	2	415	2e-172	504
3.7.1.9(2-hydroxymuconate-semialdehyde hydrolase;)																					
No Correspond Unigene																					
4.2.1.54(lactoyl-CoA dehydratase;)																					
No Correspond Unigene																					
4.2.1.84(nitrile hydratase;)																					
No Correspond Unigene																					
4.99.1.7(phenylacetaldoxime dehydratase;)																					
No Correspond Unigene																					
5.2.1.2(maleylacetoacetate isomerase;)																					
ABCDEF_24780	UniRef50_O43708	2e-58	Normal: 315	636	278	477	702	802	BlastAnno: 1018	UniRef50_O43708	216	50.00	214	100	3	822	187	8	216	2e-58	196
ABCDEF_73734	UniRef50_O43708	2e-58	Normal: 201	411	188	302	537	754	BlastAnno: 949	UniRef50_O43708	216	50.00	214	100	3	822	187	8	216	2e-58	195
5.3.99.7(styrene-oxide isomerase;)																					
No Correspond Unigene																					
>00650 Butanoate metabolism																					
1.1.1.-(Oxidoreductases;)																					
ABCDEF_13453	UniRef50_Q9SYM5	0.0	Normal: 225	226	398	1950	2026	421	BlastAnno: 2578	UniRef50_Q9SYM5	669	86.61	672	87	2	298	2313	1	669	0.0	1226
ABCDEF_17342	UniRef50_Q84M96	0.0	Normal: 274	406	272	327	439	625	BlastAnno: 1277	UniRef50_Q84M96	346	73.33	345	91	1	1187	156	2	346	0.0	546
ABCDEF_31147	UniRef50_Q9SYM5	0.0	Normal: 4571	1591	1491	1401	1939	4327	BlastAnno: 2465	UniRef50_Q9SYM5	669	82.76	673	107	4	2397	394	1	669	0.0	1154
ABCDEF_17344	UniRef50_Q84M96	0.0	Normal: 252	373	257	297	380	540	BlastAnno: 1350	UniRef50_Q84M96	346	68.19	371	91	2	1263	154	2	346	0.0	530
ABCDEF_44674	UniRef50_Q9SYM5	0.0	Normal: 3581	2620	4329	9505	11447	4930	BlastAnno: 2634	UniRef50_Q9SYM5	669	87.80	672	78	3	253	2265	1	669	0.0	1241
1.1.1.157(3-hydroxybutyryl-CoA dehydrogenase;)																					
ABCDEF_7588	UniRef50_Q45223	6e-112	Normal: 227	754	413	644	1257	2201	BlastAnno: 1204	UniRef50_Q45223	293	56.14	285	125	0	101	955	3	287	6e-112	338
1.1.1.30(3-hydroxybutyrate dehydrogenase;)																					
No Correspond Unigene																					
1.1.1.303(diacetyl reductase [(R)-acetoin forming];)																					
No Correspond Unigene																					
1.1.1.304(diacetyl reductase [(S)-acetoin forming];)																					
No Correspond Unigene																					
1.1.1.35(3-hydroxyacyl-CoA dehydrogenase;)																					
ABCDEF_80649	UniRef50_Q1LD07	5e-120	Normal: 527	841	443	1308	1321	1188	BlastAnno: 2586	UniRef50_Q1LD07	715	37.34	699	420	9	2358	277	22	707	5e-120	389
1.1.1.36(acetoacetyl-CoA reductase;)																					
ABCDEF_34806	UniRef50_B7QGZ9	3e-25	Normal: 139	128	127	302	264	325	BlastAnno: 873	UniRef50_B7QGZ9	273	39.19	148	78	1	177	584	105	252	3e-25	108
1.1.1.4(No Name)																					
No Correspond Unigene																					
1.1.1.61(4-hydroxybutyrate dehydrogenase;)																					
ABCDEF_25589	UniRef50_Q94B07	9e-166	Normal: 1440	1755	1664	2327	3041	3552	BlastAnno: 1236	UniRef50_Q94B07	289	83.28	287	48	0	92	952	2	288	9e-166	476
1.1.1.76(No Name)																					
No Correspond Unigene																					
1.1.1.83(D-malate dehydrogenase (decarboxylating);)																					
No Correspond Unigene																					
1.1.99.2(2-hydroxyglutarate dehydrogenase;)																					
ABCDEF_603	UniRef50_B1KDC8	4e-51	Normal: 60	64	53	60	64	50	BlastAnno: 871	UniRef50_B1KDC8	378	36.21	290	158	5	870	7	75	339	4e-51	180
1.2.1.10(acetaldehyde dehydrogenase (acetylating);)																					
No Correspond Unigene																					
1.2.1.16(succinate-semialdehyde dehydrogenase [NAD(P)+];)																					
ABCDEF_35184	UniRef50_Q39Q67	0.0	Normal: 907	754	991	1776	1616	1278	BlastAnno: 2107	UniRef50_Q39Q67	509	57.38	481	205	0	1853	411	29	509	0.0	578
1.2.1.24(succinate-semialdehyde dehydrogenase (NAD+);)																					
ABCDEF_35184	UniRef50_Q9SAK4	0.0	Normal: 907	754	991	1776	1616	1278	BlastAnno: 2107	UniRef50_Q9SAK4	528	75.00	528	129	2	1991	408	2	526	0.0	778
1.2.1.57(butanal dehydrogenase)																					
No Correspond Unigene																					
1.2.4.1(pyruvate dehydrogenase (acetyl-transferring);)																					
ABCDEF_28990	UniRef50_Q8H1Y0	0.0	Normal: 569	351	802	1550	2572	992	BlastAnno: 1596	UniRef50_Q8H1Y0	393	80.62	356	69	0	1369	302	38	393	0.0	628
ABCDEF_11397	UniRef50_O24458	0.0	Normal: 1628	1528	1619	3502	3214	2274	BlastAnno: 1766	UniRef50_O24458	406	82.74	394	58	4	1349	180	19	406	0.0	610

ABCDEF_35811	UniRef50_Q8H1Y0	0.0	Normal: 1865	1480	2772	5588	6415	2489	BlastAnno: 1647	UniRef50_Q8H1Y0	393	77.66	394	83	3	138	1307	1	393	0.0	621
ABCDEF_15406	UniRef50_O24458	0.0	Normal: 297	358	305	389	271	1259	BlastAnno: 1486	UniRef50_O24458	406	80.73	410	72	4	19	1239	1	406	0.0	647
ABCDEF_49154	UniRef50_O24458	0.0	Normal: 304	369	317	391	293	1358	BlastAnno: 1412	UniRef50_O24458	406	80.73	410	72	4	1394	174	1	406	0.0	647
ABCDEF_63617	UniRef50_O24458	0.0	Normal: 518	490	543	916	834	1436	BlastAnno: 1398	UniRef50_O24458	406	80.68	409	72	4	1397	180	2	406	0.0	618

1.2.7.1(pyruvate synthase;)

No Correspond Unigene

1.3.1.44(trans-2-enoyl-CoA reductase (NAD+);)

No Correspond Unigene

1.3.99.1(succinate dehydrogenase;)

ABCDEF_47748	UniRef50_Q6F8K9	0.0	Normal: 903	791	1034	2782	3604	958	BlastAnno: 2360	UniRef50_Q6F8K9	632	51.24	603	252	8	1990	290	36	632	0.0	584
ABCDEF_47751	UniRef50_Q6F8K9	0.0	Normal: 776	655	916	2143	2907	810	BlastAnno: 2266	UniRef50_Q6F8K9	632	51.24	603	252	8	303	2003	36	632	0.0	585
ABCDEF_47750	UniRef50_Q6F8K9	0.0	Normal: 1478	889	1772	2151	3099	1241	BlastAnno: 2236	UniRef50_Q6F8K9	632	51.00	602	255	7	1898	198	36	632	0.0	571

1.3.99.2(butyryl-CoA dehydrogenase;)

ABCDEF_90276	UniRef50_Q0AYU5	1e-61	Normal: 0	0	0	0	0	80	BlastAnno: 757	UniRef50_Q0AYU5	380	45.63	252	126	5	3	755	44	285	1e-61	206

2.2.1.6(acetolactate synthase;)

ABCDEF_41851	UniRef50_B2UM25	0.0	Normal: 2693	2810	2333	1636	1298	4686	BlastAnno: 2340	UniRef50_B2UM25	617	52.19	594	263	7	364	2088	10	601	0.0	597
ABCDEF_6813	UniRef50_B2UM25	0.0	Normal: 935	649	1027	751	1655	773	BlastAnno: 2146	UniRef50_B2UM25	617	51.17	598	270	8	1943	204	10	603	0.0	584

2.3.1.-(Transferases;)

ABCDEF_520	UniRef50_Q9FF86	0.0	Normal: 1672	85	2273	5056	3383	1211	BlastAnno: 1811	UniRef50_Q9FF86	484	64.06	448	149	6	1664	354	3	449	0.0	573
ABCDEF_68664	UniRef50_Q9ZVN2	0.0	Normal: 79	35	85	107	193	63	BlastAnno: 1488	UniRef50_Q9ZVN2	704	63.99	411	142	3	46	1275	298	703	0.0	540
ABCDEF_52245	UniRef50_Q9FG87	0.0	Normal: 1878	635	1833	3814	1432	1376	BlastAnno: 1758	UniRef50_Q9FG87	529	55.35	486	204	4	4	1428	34	517	0.0	530
ABCDEF_16730	UniRef50_Q3EC11	0.0	Normal: 281	204	237	365	377	458	BlastAnno: 2092	UniRef50_Q3EC11	536	59.33	536	204	8	196	1785	3	530	0.0	661
ABCDEF_4655	UniRef50_A5BMG1	0.0	Normal: 2426	1856	3606	6958	4608	2755	BlastAnno: 2261	UniRef50_A5BMG1	481	68.37	528	112	10	263	1843	3	476	0.0	667
ABCDEF_13860	UniRef50_Q9C533	0.0	Normal: 588	220	570	1451	1338	310	BlastAnno: 2648	UniRef50_Q9C533	596	63.37	606	177	12	2226	511	1	595	0.0	558
ABCDEF_28294	UniRef50_Q9SYZ0	0.0	Normal: 1038	4070	1427	6745	877	2797	BlastAnno: 1785	UniRef50_Q9SYZ0	493	52.37	485	223	3	1598	162	5	487	0.0	533
ABCDEF_47718	UniRef50_Q9C533	0.0	Normal: 1599	236	1657	2093	1231	588	BlastAnno: 2680	UniRef50_Q9C533	596	65.02	626	165	14	2280	472	1	595	0.0	634
ABCDEF_45080	UniRef50_Q9SYZ0	0.0	Normal: 1005	592	1079	2360	3762	2217	BlastAnno: 2130	UniRef50_Q9SYZ0	493	57.85	484	197	3	302	1735	5	487	0.0	533
ABCDEF_25377	UniRef50_Q9C6L5	0.0	Normal: 37	2090	138	2573	1026	1298	BlastAnno: 1334	UniRef50_Q9C6L5	492	79.74	385	76	1	1155	1	6	388	0.0	654
ABCDEF_33470	UniRef50_Q9LW26	0.0	Normal: 172	98	156	260	391	149	BlastAnno: 2993	UniRef50_Q9LW26	701	48.96	627	301	5	655	2508	85	701	0.0	581
ABCDEF_40654	UniRef50_Q9H0A0	0.0	Normal: 638	213	530	318	318	286	BlastAnno: 2216	UniRef50_Q9H0A0	1025	56.87	721	279	6	2127	13	91	795	0.0	756
ABCDEF_33460	UniRef50_Q9ZVN2	0.0	Normal: 144	71	119	216	306	97	BlastAnno: 2691	UniRef50_Q9ZVN2	704	61.50	587	191	4	379	2133	83	636	0.0	715
ABCDEF_14442	UniRef50_Q9SYZ0	0.0	Normal: 341	684	439	1040	1601	552	BlastAnno: 1779	UniRef50_Q9SYZ0	493	63.12	480	171	2	1570	149	5	484	0.0	628
ABCDEF_52626	UniRef50_B6SYP9	0.0	Normal: 815	346	1131	3015	1537	108	BlastAnno: 1861	UniRef50_B6SYP9	494	55.02	438	189	3	1538	249	51	488	0.0	536
ABCDEF_644	UniRef50_Q9SB58	0.0	Normal: 704	444	913	969	1431	848	BlastAnno: 1777	UniRef50_Q9SB58	407	64.15	424	129	8	1572	316	1	406	0.0	554
ABCDEF_27087	UniRef50_Q52T38	0.0	Normal: 324	221	233	419	343	412	BlastAnno: 1631	UniRef50_Q52T38	620	75.95	420	91	1	1409	150	1	410	0.0	681
ABCDEF_34187	UniRef50_Q8L5Y5	0.0	Normal: 497	415	588	523	291	433	BlastAnno: 2677	UniRef50_Q8L5Y5	718	57.30	733	251	15	202	2274	1	713	0.0	683
ABCDEF_47966	UniRef50_Q9SYZ0	0.0	Normal: 577	934	326	580	682	3440	BlastAnno: 2177	UniRef50_Q9SYZ0	493	56.40	484	204	3	1780	347	5	487	0.0	535
ABCDEF_51189	UniRef50_Q52T38	0.0	Normal: 135	152	128	181	219	327	BlastAnno: 1766	UniRef50_Q52T38	620	68.09	470	137	3	80	1483	130	588	0.0	641
ABCDEF_41288	UniRef50_Q9SB58	0.0	Normal: 307	183	332	637	649	322	BlastAnno: 1403	UniRef50_Q9SB58	407	67.56	410	110	6	1401	184	15	405	0.0	523
ABCDEF_33461	UniRef50_Q9ZVN2	0.0	Normal: 142	84	140	206	283	119	BlastAnno: 2904	UniRef50_Q9ZVN2	704	60.98	574	201	6	2271	559	83	636	0.0	686
ABCDEF_14334	UniRef50_Q9SYZ0	0.0	Normal: 2263	129	1724	3469	866	283	BlastAnno: 2039	UniRef50_Q9SYZ0	493	63.22	484	171	2	369	1802	5	487	0.0	627
ABCDEF_24844	UniRef50_Q9FF86	0.0	Normal: 1857	3686	2657	21940	11467	4090	BlastAnno: 1640	UniRef50_Q9FF86	484	66.89	447	135	6	1492	185	2	446	0.0	575
ABCDEF_51190	UniRef50_Q52T38	0.0	Normal: 145	169	142	210	232	335	BlastAnno: 1891	UniRef50_Q52T38	620	70.04	514	141	3	73	1608	86	588	0.0	726
ABCDEF_14085	UniRef50_Q8L5Y5	0.0	Normal: 353	41	384	527	331	280	BlastAnno: 2362	UniRef50_Q8L5Y5	718	62.07	704	220	12	2360	348	24	713	0.0	676
ABCDEF_33458	UniRef50_Q9LW26	0.0	Normal: 158	78	130	240	342	106	BlastAnno: 2822	UniRef50_Q9LW26	701	46.72	655	302	5	400	2337	85	701	0.0	568
ABCDEF_25193	UniRef50_Q9C6L5	0.0	Normal: 3256	368	3548	6230	5109	1288	BlastAnno: 1786	UniRef50_Q9C6L5	492	65.83	480	156	4	1668	241	17	492	0.0	668
ABCDEF_33457	UniRef50_Q9ZVN2	0.0	Normal: 168	83	137	250	375	112	BlastAnno: 2823	UniRef50_Q9ZVN2	704	65.12	496	168	3	379	1860	83	575	0.0	648
ABCDEF_7634	UniRef50_Q9C6L5	0.0	Normal: 2543	9303	2711	11578	5533	10032	BlastAnno: 2064	UniRef50_Q9C6L5	492	52.76	489	225	3	1734	268	1	483	0.0	535
ABCDEF_299	UniRef50_Q71N54	0.0	Normal: 569	1147	511	900	649	1879	BlastAnno: 2017	UniRef50_Q71N54	535	61.73	533	198	5	338	1924	4	534	0.0	677
ABCDEF_27086	UniRef50_Q52T38	0.0	Normal: 784	470	617	843	893	976	BlastAnno: 2398	UniRef50_Q52T38	620	70.80	637	165	4	225	2129	1	618	0.0	887
ABCDEF_51188	UniRef50_Q52T38	0.0	Normal: 273	327	255	415	349	441	BlastAnno: 2324	UniRef50_Q52T38	620	71.30	568	150	3	2041	344	32	588	0.0	815
ABCDEF_44812	UniRef50_Q6DR03	0.0	Normal: 982	902	1005	1071	959	2999	BlastAnno: 2450	UniRef50_Q6DR03	565	54.97	644	198	17	2142	220	1	555	0.0	540

2.3.1.19(phosphate butyryltransferase;)

No Correspond Unigene

2.3.1.54(formate C-acetyltransferase;)

No Correspond Unigene

2.3.1.9(acetyl-CoA C-acetyltransferase;)

ABCDEF_34825	UniRef50_Q8S4Y1	0.0	Normal: 640	698	620	807	839	1164	BlastAnno: 1773	UniRef50_Q8S4Y1	403	78.11	402	87	1	1597	395	2	403	0.0	598
ABCDEF_14629	UniRef50_Q8S4Y1	0.0	Normal: 634	316	650	1021	1278	691	BlastAnno: 1514	UniRef50_Q8S4Y1	403	85.11	403	60	0	1367	159	1	403	0.0	620
ABCDEF_107	UniRef50_Q8S4Y1	0.0	Normal: 332	460	460	842	893	424	BlastAnno: 1214	UniRef50_Q8S4Y1	403	84.07	339	54	0	1212	196	42	380	0.0	596
ABCDEF_14630	UniRef50_Q8S4Y1	0.0	Normal: 562	282	568	959	1173	627	BlastAnno: 1796	UniRef50_Q8S4Y1	403	85.50	400	58	0	436	1635	4	403	0.0	642

2.3.3.10(hydroxymethylglutaryl-CoA synthase;)

ABCDEF_52282	UniRef50_A4GKI1	0.0	Normal: 1662	333	1405	2243	3244	1685	BlastAnno: 1805	UniRef50_A4GKI1	463	57.49	454	189	2	1606	251	2	453	0.0	536
ABCDEF_4202	UniRef50_A4GKI1	0.0	Normal: 1002	1241	821	1323	1992	1786	BlastAnno: 1998	UniRef50_A4GKI1	463	57.74	452	185	3	1636	290	2	450	0.0	531

2.6.1.19(4-aminobutyrate transaminase;)

ABCDEF_3775	UniRef50_B8CY58	4e-110	Normal: 473	159	414	817	611	782	BlastAnno: 1801	UniRef50_B8CY58	437	45.37	432	223	7	1570	293	6	430	4e-110	346

2.7.2.7(butyrate kinase)

No Correspond Unigene

2.8.3.12(glutaconate CoA-transferase)

No Correspond Unigene

2.8.3.5(3-oxoacid CoA-transferase;)

No Correspond Unigene

	2.8.3.8(acetate CoA-transferase;)																					
	No Correspond Unigene																					
	3.1.1.22(hydroxybutyrate-dimer hydrolase;)																					
	No Correspond Unigene																					
	3.1.1.75(poly(3-hydroxybutyrate) depolymerase;)																					
	No Correspond Unigene																					
	3.1.2.11(acetoacetyl-CoA hydrolase;)																					
	No Correspond Unigene																					
	4.1.1.15(glutamate decarboxylase;)																					
	ABCDEF_11564	UniRef50_Q42472	0.0	Normal: 1009	2635	783	1023	2784	3375	BlastAnno: 2048	UniRef50_Q42472	494	80.20	500	93	3	224	1723	1	494	0.0	846
	ABCDEF_38368	UniRef50_Q42472	0.0	Normal: 893	2322	667	877	2443	2996	BlastAnno: 2255	UniRef50_Q42472	494	86.09	338	46	1	2032	1019	1	337	0.0	624
	ABCDEF_38369	UniRef50_Q42472	0.0	Normal: 802	2006	619	863	2096	2472	BlastAnno: 2237	UniRef50_Q42472	494	89.35	310	33	0	1833	904	28	337	0.0	593
	ABCDEF_38367	UniRef50_Q42472	0.0	Normal: 952	2438	773	996	2638	3216	BlastAnno: 2145	UniRef50_Q42472	494	81.99	472	80	2	1741	326	28	494	0.0	815
	4.1.1.5(acetolactate decarboxylase;)																					
	No Correspond Unigene																					
	4.1.1.70(glutaconyl-CoA decarboxylase;)																					
	ABCDEF_8712	UniRef50_B6KHR8	3e-13	Normal: 16	0	5	0	35	34	BlastAnno: 338	UniRef50_B6KHR8	600	63.27	49	18	0	116	262	250	298	3e-13	71.6
	4.1.3.4(hydroxymethylglutaryl-CoA lyase;)																					
	ABCDEF_25184	UniRef50_P35915	3e-119	Normal: 682	2198	692	1164	701	2789	BlastAnno: 1741	UniRef50_P35915	298	63.51	296	108	0	1298	411	3	298	3e-119	363
	4.2.1.-(Lyases;)																					
	ABCDEF_48679	UniRef50_Q0RP29	5e-115	Normal: 568	325	563	941	1285	676	BlastAnno: 1424	UniRef50_Q0RP29	493	56.39	321	129	4	119	1075	39	350	5e-115	356
	4.2.1.17(enoyl-CoA hydratase;)																					
	ABCDEF_21259	UniRef50_Q8W1L6	0.0	Normal: 272	276	284	384	326	305	BlastAnno: 2641	UniRef50_Q8W1L6	726	47.57	721	375	3	114	2267	6	726	0.0	633
	ABCDEF_80649	UniRef50_Q8W1L6	0.0	Normal: 527	841	443	1308	1321	1188	BlastAnno: 2586	UniRef50_Q8W1L6	726	60.39	712	281	1	2403	268	1	711	0.0	818
	ABCDEF_466	UniRef50_Q8W1L6	0.0	Normal: 1014	1690	1094	1677	2438	2080	BlastAnno: 2453	UniRef50_Q8W1L6	726	71.24	699	201	0	2368	272	7	705	0.0	954
	ABCDEF_54834	UniRef50_Q8W1L6	0.0	Normal: 229	220	237	298	270	261	BlastAnno: 2405	UniRef50_Q8W1L6	726	47.52	606	315	3	274	2082	121	726	0.0	580
	ABCDEF_34756	UniRef50_Q8W1L6	0.0	Normal: 776	726	768	2922	4039	1079	BlastAnno: 2469	UniRef50_Q8W1L6	726	68.93	708	217	2	2396	273	7	711	0.0	957
	ABCDEF_4198	UniRef50_Q8W1L6	0.0	Normal: 609	929	532	1401	1568	1297	BlastAnno: 2667	UniRef50_Q8W1L6	726	60.45	713	281	1	2463	325	1	712	0.0	820
	4.2.1.27(acetylenecarboxylate hydratase;)																					
	No Correspond Unigene																					
	4.2.1.31(maleate hydratase;)																					
	No Correspond Unigene																					
	4.2.1.55(3-hydroxybutyryl-CoA dehydratase;)																					
	ABCDEF_31573	UniRef50_C0QBE8	2e-44	Normal: 159	151	158	345	356	262	BlastAnno: 1094	UniRef50_C0QBE8	240	40.00	220	124	3	228	875	11	226	2e-44	160
	5.1.2.3(3-hydroxybutyryl-CoA epimerase;)																					
	No Correspond Unigene																					
	5.1.2.4(acetoin racemase;)																					
	No Correspond Unigene																					
	5.2.1.1(maleate isomerase)																					
	No Correspond Unigene																					
	5.3.3.3(vinylacetyl-CoA Delta-isomerase;)																					
	No Correspond Unigene																					
	6.2.1.16(acetoacetate---CoA ligase;)																					
	ABCDEF_46067	UniRef50_Q0SJB1	9e-08	Normal: 10	11	0	0	48	18	BlastAnno: 390	UniRef50_Q0SJB1	666	30.17	116	74	3	41	388	38	146	9e-08	55.8
	6.2.1.2(butyrate---CoA ligase;)																					
	No Correspond Unigene																					
>00660	C5-Branched dibasic acid metabolism																					
	2.2.1.6(acetolactate synthase;)																					
	ABCDEF_41851	UniRef50_B2UM25	0.0	Normal: 2693	2810	2333	1636	1298	4686	BlastAnno: 2340	UniRef50_B2UM25	617	52.19	594	263	7	364	2088	10	601	0.0	597
	ABCDEF_6813	UniRef50_B2UM25	0.0	Normal: 935	649	1027	751	1655	773	BlastAnno: 2146	UniRef50_B2UM25	617	51.17	598	270	8	1943	204	10	603	0.0	584
	2.3.3.11(2-hydroxyglutarate synthase;)																					
	No Correspond Unigene																					
	2.8.3.11(citramalate CoA-transferase)																					
	No Correspond Unigene																					
	2.8.3.7(succinate---citramalate CoA-transferase;)																					
	No Correspond Unigene																					
	3.5.1.67(4-methyleneglutaminase;)																					
	No Correspond Unigene																					
	4.1.1.5(acetolactate decarboxylase;)																					
	No Correspond Unigene																					
	4.1.1.6(aconitate decarboxylase;)																					
	No Correspond Unigene																					
	4.1.3.17(4-hydroxy-4-methyl-2-oxoglutarate aldolase;)																					
	No Correspond Unigene																					
	4.1.3.22(citramalate lyase;)																					
	No Correspond Unigene																					
	4.1.3.24(malyl-CoA lyase;)																					
	No Correspond Unigene																					
	4.1.3.25(citramalyl-CoA lyase;)																					
	No Correspond Unigene																					
	4.2.1.34(No Name)																					
	No Correspond Unigene																					

4.2.1.35(No Name)

No Correspond Unigene

4.2.1.56(itaconyl-CoA hydratase;)

No Correspond Unigene

4.3.1.2(methylaspartate ammonia-lyase;)

No Correspond Unigene

5.4.99.1(methylaspartate mutase;)

No Correspond Unigene

6.2.1.5(succinate---CoA ligase (ADP-forming);)

ABCDEF_17836	UniRef50_O82662	0.0	Normal: 367	77	365	530	1198	144	BlastAnno: 1491	UniRef50_O82662	421	85.48	420	61	0	131	1390	1	420	0.0	741
ABCDEF_45671	UniRef50_O82662	0.0	Normal: 1899	1293	2339	3905	6798	2137	BlastAnno: 1668	UniRef50_O82662	421	87.38	420	53	0	164	1423	1	420	0.0	753

6.3.1.7(4-methyleneglutamate---ammonia ligase;)

No Correspond Unigene

>00670 One carbon pool by folate

1.5.1.15(methylenetetrahydrofolate dehydrogenase (NAD+);)

No Correspond Unigene

1.5.1.20(methylenetetrahydrofolate reductase [NAD(P)H];)

ABCDEF_18406	UniRef50_B9HG95	0.0	Normal: 5047	6500	5572	11857	13941	8245	BlastAnno: 2369	UniRef50_B9HG95	608	82.24	608	97	1	269	2059	1	608	0.0	1024

1.5.1.3(dihydrofolate reductase;)

ABCDEF_13008	UniRef50_Q27828	1e-156	Normal: 295	218	353	308	311	485	BlastAnno: 2378	UniRef50_Q27828	462	47.04	506	220	6	406	1923	5	462	1e-156	473

1.5.1.5(methylenetetrahydrofolate dehydrogenase (NADP+);)

ABCDEF_28394	UniRef50_P11586	0.0	Normal: 1686	2953	1497	2421	3359	6073	BlastAnno: 2493	UniRef50_P11586	935	68.58	627	194	3	383	2260	310	934	0.0	818

1.5.1.6(formyltetrahydrofolate dehydrogenase;)

No Correspond Unigene

1.5.7.1(methylenetetrahydrofolate reductase (ferredoxin);)

No Correspond Unigene

2.1.1.13(methionine synthase;)

No Correspond Unigene

2.1.1.148(thymidylate synthase (FAD);)

No Correspond Unigene

2.1.1.19(trimethylsulfonium---tetrahydrofolate N-methyltransferase;)

No Correspond Unigene

2.1.1.45(thymidylate synthase;)

ABCDEF_13009	UniRef50_P04818	2e-137	Normal: 222	170	310	238	266	400	BlastAnno: 1734	UniRef50_P04818	313	65.03	286	100	0	1313	456	28	313	2e-137	410

2.1.2.1(glycine hydroxymethyltransferase;)

ABCDEF_41184	UniRef50_C5KPS8	0.0	Normal: 509	437	722	1807	2474	561	BlastAnno: 2201	UniRef50_C5KPS8	607	62.56	446	164	2	1762	425	164	606	0.0	590
ABCDEF_11573	UniRef50_P34897	0.0	Normal: 1903	1387	2092	2286	3254	2865	BlastAnno: 2080	UniRef50_P34897	504	60.18	457	170	4	502	1845	48	501	0.0	573
ABCDEF_37629	UniRef50_Q9LM59	0.0	Normal: 412	460	499	689	962	979	BlastAnno: 2512	UniRef50_Q9LM59	599	66.00	597	187	5	369	2126	8	599	0.0	813
ABCDEF_7224	UniRef50_C5KPS8	0.0	Normal: 360	5392	231	401	266	13616	BlastAnno: 2246	UniRef50_C5KPS8	607	61.88	446	167	2	1828	491	164	606	0.0	577
ABCDEF_51490	UniRef50_Q9LM59	0.0	Normal: 497	502	504	755	1027	1332	BlastAnno: 2919	UniRef50_Q9LM59	599	64.38	584	197	2	578	2299	17	599	0.0	754
ABCDEF_1167	UniRef50_Q9LM59	0.0	Normal: 5116	9848	6479	16268	26840	14685	BlastAnno: 1891	UniRef50_Q9LM59	599	62.07	464	171	1	1747	371	134	597	0.0	611
ABCDEF_7223	UniRef50_C5KPS8	0.0	Normal: 395	5706	275	418	261	13912	BlastAnno: 2154	UniRef50_C5KPS8	607	61.88	446	167	2	1736	399	164	606	0.0	577
ABCDEF_28355	UniRef50_P34897	0.0	Normal: 255	239	370	837	1312	358	BlastAnno: 1812	UniRef50_P34897	504	57.73	466	153	3	1810	416	81	503	0.0	553

2.1.2.10(aminomethyltransferase;)

ABCDEF_22217	UniRef50_O65396	0.0	Normal: 1753	5094	1871	4253	3113	17559	BlastAnno: 1818	UniRef50_O65396	408	87.47	407	51	0	363	1583	1	407	0.0	752

2.1.2.2(phosphoribosylglycinamide formyltransferase;)

ABCDEF_21522	UniRef50_P52422	3e-110	Normal: 231	105	226	159	220	202	BlastAnno: 1269	UniRef50_P52422	292	72.69	216	58	1	904	260	76	291	3e-110	335

2.1.2.3(phosphoribosylaminoimidazolecarboxamide formyltransferase;)

ABCDEF_31348	UniRef50_A3DEU9	5e-160	Normal: 1610	679	1784	1352	1383	1416	BlastAnno: 2145	UniRef50_A3DEU9	514	46.50	529	261	7	1878	307	3	514	5e-160	481

2.1.2.4(glycine formimidoyltransferase;)

No Correspond Unigene

2.1.2.5(glutamate formimidoyltransferase;)

ABCDEF_24160	UniRef50_Q3AE85	1e-09	Normal: 112	179	129	133	48	463	BlastAnno: 872	UniRef50_Q3AE85	305	30.00	130	74	4	712	341	10	128	1e-09	63.9

2.1.2.7(D-alanine 2-hydroxymethyltransferase;)

No Correspond Unigene

2.1.2.8(deoxycytidylate 5-hydroxymethyltransferase;)

No Correspond Unigene

2.1.2.9(methionyl-tRNA formyltransferase;)

ABCDEF_3144	UniRef50_B8C483	1e-62	Normal: 46	41	49	56	55	232	BlastAnno: 1466	UniRef50_B8C483	337	38.39	336	195	8	1339	347	1	329	1e-62	215
ABCDEF_3142	UniRef50_B8C483	1e-62	Normal: 47	35	64	56	53	215	BlastAnno: 1477	UniRef50_B8C483	337	38.39	336	195	8	130	1122	1	329	1e-62	215

3.5.1.10(formyltetrahydrofolate deformylase)

ABCDEF_11642	UniRef50_B9SXD7	5e-153	Normal: 361	592	520	793	672	2287	BlastAnno: 1364	UniRef50_B9SXD7	341	77.42	279	60	2	113	949	4	279	5e-153	447

3.5.4.9(methenyltetrahydrofolate cyclohydrolase;)

No Correspond Unigene

4.3.1.4(formimidoyltetrahydrofolate cyclodeaminase;)

No Correspond Unigene

6.3.3.2(5-formyltetrahydrofolate cyclo-ligase;)

ABCDEF_48448	UniRef50_B9R910	9e-93	Normal: 90	54	103	190	333	218	BlastAnno: 1122	UniRef50_B9R910	292	62.55	251	82	3	139	870	27	272	9e-93	288

6.3.4.3(formate---tetrahydrofolate ligase;)

ABCDEF_28394	UniRef50_Q6UB35	0.0	Normal: 1686	2953	1497	2421	3359	6073	BlastAnno: 2493	UniRef50_Q6UB35	978	66.88	628	205	3	380	2260	352	977	0.0	792

>00680 Methane metabolism

1.1.1.244(methanol dehydrogenase)

No Correspond Unigene																					
1.1.1.272(No Name)																					
No Correspond Unigene																					
1.1.1.284(S-(hydroxymethyl)glutathione dehydrogenase;)																					
ABCDEF_21050	UniRef50_B9RYV6	0.0	Normal: 5	0	8	669	176	0	BlastAnno: 1535	UniRef50_B9RYV6	425	70.56	377	111	0	364	1494	49	425	0.0	528
1.1.1.29(glycerate dehydrogenase;)																					
ABCDEF_38443	UniRef50_P13443	0.0	Normal: 996	6551	645	477	900	18941	BlastAnno: 1745	UniRef50_P13443	382	89.21	380	41	0	326	1465	1	380	0.0	679
1.1.1.37(malate dehydrogenase;)																					
ABCDEF_25169	UniRef50_Q9SN86	0.0	Normal: 938	614	1116	1317	4446	973	BlastAnno: 1856	UniRef50_Q9SN86	403	75.50	400	88	2	1616	417	2	391	0.0	585
ABCDEF_31746	UniRef50_Q9ZP05	0.0	Normal: 611	6985	354	98	71	19818	BlastAnno: 1631	UniRef50_Q9ZP05	354	83.38	349	54	2	274	1317	9	354	0.0	578
ABCDEF_32230	UniRef50_P57106	0.0	Normal: 8408	4627	7877	10286	18779	11078	BlastAnno: 1523	UniRef50_P57106	332	93.07	332	23	0	262	1257	1	332	0.0	617
ABCDEF_57415	UniRef50_Q9ZP05	0.0	Normal: 403	336	343	761	845	611	BlastAnno: 1190	UniRef50_Q9ZP05	354	83.29	353	54	2	1058	3	1	349	0.0	579
ABCDEF_17941	UniRef50_Q9ZP05	0.0	Normal: 616	554	586	1025	1231	910	BlastAnno: 1567	UniRef50_Q9ZP05	354	83.63	342	51	2	1336	314	17	354	0.0	563
1.1.1.95(phosphoglycerate dehydrogenase;)																					
ABCDEF_13990	UniRef50_O04130	0.0	Normal: 487	278	487	447	1745	311	BlastAnno: 1815	UniRef50_O04130	624	80.46	481	93	1	1815	373	145	624	0.0	754
ABCDEF_35317	UniRef50_O04130	0.0	Normal: 3413	458	3803	2964	1953	680	BlastAnno: 1723	UniRef50_O04130	624	78.60	500	107	0	1	1500	125	624	0.0	736
ABCDEF_45312	UniRef50_O04130	0.0	Normal: 879	2767	1113	1869	8492	758	BlastAnno: 1813	UniRef50_O04130	624	83.82	513	83	0	1812	274	112	624	0.0	853
1.1.2.7(methanol dehydrogenase (cytochrome c);)																					
No Correspond Unigene																					
1.1.3.13(alcohol oxidase;)																					
No Correspond Unigene																					
1.11.1.6(catalase;)																					
ABCDEF_32232	UniRef50_Q96528	0.0	Normal: 5097	16083	8563	16179	24962	32510	BlastAnno: 1961	UniRef50_Q96528	492	75.41	492	121	0	97	1572	1	492	0.0	816
ABCDEF_45231	UniRef50_Q96528	0.0	Normal: 3120	1495	2311	3940	2336	3972	BlastAnno: 1786	UniRef50_Q96528	492	86.99	492	64	0	74	1549	1	492	0.0	921
ABCDEF_12422	UniRef50_Q96528	0.0	Normal: 3094	9467	5243	8875	15494	19272	BlastAnno: 1462	UniRef50_Q96528	492	72.90	369	99	1	64	1167	122	490	0.0	597
ABCDEF_1075	UniRef50_Q96528	0.0	Normal: 205	3077	104	25	17	5494	BlastAnno: 1543	UniRef50_Q96528	492	83.74	406	66	0	1543	326	87	492	0.0	720
ABCDEF_727	UniRef50_Q96528	0.0	Normal: 671	268	532	1260	1197	440	BlastAnno: 1428	UniRef50_Q96528	492	87.81	443	54	0	98	1426	1	443	0.0	837
1.11.1.7(peroxidase;)																					
ABCDEF_25672	UniRef50_Q9SB81	0.0	Normal: 7636	15838	4777	1134	3090	9628	BlastAnno: 1715	UniRef50_Q9SB81	330	85.71	301	43	0	342	1244	30	330	0.0	552
1.12.1.2(hydrogen dehydrogenase;)																					
No Correspond Unigene																					
1.12.2.-(Oxidoreductases;)																					
No Correspond Unigene																					
1.12.7.2(ferredoxin hydrogenase;)																					
ABCDEF_52859	UniRef50_Q22NP0	5e-12	Normal: 43	31	23	68	115	76	BlastAnno: 458	UniRef50_Q22NP0	488	37.08	89	45	3	218	457	2	88	5e-12	68.9
1.12.98.1(coenzyme F420 hydrogenase;)																					
No Correspond Unigene																					
1.12.98.2(5,10-methenyltetrahydromethanopterin hydrogenase;)																					
No Correspond Unigene																					
1.12.99.-(Oxidoreductases;)																					
No Correspond Unigene																					
1.14.13.25(methane monooxygenase;)																					
No Correspond Unigene																					
1.14.13.8(flavin-containing monooxygenase;)																					
No Correspond Unigene																					
1.2.1.2(formate dehydrogenase;)																					
ABCDEF_60816	UniRef50_P33160	7e-111	Normal: 1383	1133	1488	2129	3432	1561	BlastAnno: 1303	UniRef50_P33160	401	51.38	327	159	0	116	1096	49	375	7e-111	341
1.2.1.43(formate dehydrogenase (NADP+);)																					
No Correspond Unigene																					
1.2.1.46(formaldehyde dehydrogenase;)																					
No Correspond Unigene																					
1.2.7.1(pyruvate synthase;)																					
No Correspond Unigene																					
1.2.7.4(carbon-monoxide dehydrogenase (ferredoxin))																					
No Correspond Unigene																					
1.2.99.2(carbon-monoxide dehydrogenase (acceptor);)																					
No Correspond Unigene																					
1.2.99.5(formylmethanofuran dehydrogenase;)																					
No Correspond Unigene																					
1.4.99.3(amine dehydrogenase;)																					
No Correspond Unigene																					
1.5.1.-(Oxidoreductases;)																					
No Correspond Unigene																					
1.5.1.20(methylenetetrahydrofolate reductase [NAD(P)H];)																					
ABCDEF_18406	UniRef50_B9HG95	0.0	Normal: 5047	6500	5572	11857	13941	8245	BlastAnno: 2369	UniRef50_B9HG95	608	82.24	608	97	1	269	2059	1	608	0.0	1024
1.5.8.1(dimethylamine dehydrogenase)																					
No Correspond Unigene																					
1.5.8.2(trimethylamine dehydrogenase)																					
No Correspond Unigene																					
1.5.99.11(5,10-methylenetetrahydromethanopterin reductase;)																					
No Correspond Unigene																					
1.5.99.5(methylglutamate dehydrogenase;)																					

No Correspond Unigene

1.5.99.9(methylenetetrahydromethanopterin dehydrogenase;)

No Correspond Unigene

1.6.6.9(trimethylamine-N-oxide reductase;)

No Correspond Unigene

1.8.98.1(CoB---CoM heterodisulfide reductase;)

No Correspond Unigene

2.1.1.-(Transferases;)

ABCDEF_20806	UniRef50_Q94II3	0.0	Normal: 700	332	876	1458	1997	929	BlastAnno: 2275	UniRef50_Q94II3	600	73.57	594	153	4	2119	341	1	591	0.0	907
ABCDEF_45802	UniRef50_Q9ZPH9	0.0	Normal: 3543	3348	4296	9797	10830	5017	BlastAnno: 2642	UniRef50_Q9ZPH9	633	53.90	603	261	7	2216	456	23	624	0.0	676
ABCDEF_20070	UniRef50_Q944R7	0.0	Normal: 301	141	265	207	185	187	BlastAnno: 2675	UniRef50_Q944R7	724	58.28	719	275	9	127	2265	13	712	0.0	840
ABCDEF_34956	UniRef50_Q94II3	0.0	Normal: 1120	7269	2475	16033	22219	21461	BlastAnno: 2676	UniRef50_Q94II3	600	73.77	610	146	4	404	2230	1	597	0.0	957
ABCDEF_17121	UniRef50_Q9ZPH9	0.0	Normal: 124	77	109	284	199	132	BlastAnno: 2066	UniRef50_Q9ZPH9	633	53.24	601	259	8	161	1903	25	623	0.0	644
ABCDEF_6524	UniRef50_Q9ZPH9	0.0	Normal: 471	296	490	753	870	730	BlastAnno: 2602	UniRef50_Q9ZPH9	633	50.89	619	290	5	2221	404	6	623	0.0	659
ABCDEF_56017	UniRef50_Q9ZPH9	0.0	Normal: 302	167	286	596	748	529	BlastAnno: 2384	UniRef50_Q9ZPH9	633	51.37	619	287	5	213	2030	6	623	0.0	663
ABCDEF_44169	UniRef50_Q9SIZ3	0.0	Normal: 179	158	273	705	685	229	BlastAnno: 2300	UniRef50_Q9SIZ3	589	62.01	308	108	4	1219	296	285	583	0.0	394
ABCDEF_6521	UniRef50_Q9ZPH9	0.0	Normal: 339	198	314	641	767	582	BlastAnno: 2511	UniRef50_Q9ZPH9	633	51.37	619	287	5	2172	355	6	623	0.0	663
ABCDEF_44172	UniRef50_Q9SIZ3	0.0	Normal: 204	184	287	767	741	242	BlastAnno: 2317	UniRef50_Q9SIZ3	589	68.15	449	134	4	1637	291	144	583	0.0	641
ABCDEF_199	UniRef50_Q9SU94	0.0	Normal: 1076	275	1009	558	1247	306	BlastAnno: 1769	UniRef50_Q9SU94	390	78.45	348	74	1	284	1324	43	390	0.0	584
ABCDEF_52252	UniRef50_Q84W92	0.0	Normal: 1658	720	1653	1980	2289	1601	BlastAnno: 2170	UniRef50_Q84W92	535	72.49	469	120	3	409	1791	34	501	0.0	703
ABCDEF_51404	UniRef50_A3BMN9	0.0	Normal: 864	188	716	337	631	326	BlastAnno: 2192	UniRef50_A3BMN9	620	50.50	604	243	9	2008	251	55	620	0.0	555
ABCDEF_21386	UniRef50_Q94AI4	0.0	Normal: 2326	1250	2220	4294	2920	1983	BlastAnno: 3701	UniRef50_Q94AI4	775	47.27	842	332	16	567	2978	8	775	0.0	577
ABCDEF_35101	UniRef50_Q9MAT5	0.0	Normal: 1440	420	1268	576	1136	850	BlastAnno: 1634	UniRef50_Q9MAT5	383	69.03	352	109	0	1449	394	32	383	0.0	550
ABCDEF_960	UniRef50_Q9LN50	0.0	Normal: 3504	13198	3609	11156	19651	12155	BlastAnno: 2626	UniRef50_Q9LN50	724	49.14	525	246	5	883	2451	219	724	0.0	570
ABCDEF_48082	UniRef50_Q9LN50	0.0	Normal: 410	445	467	870	993	454	BlastAnno: 2340	UniRef50_Q9LN50	724	70.25	521	155	0	710	2272	204	724	0.0	821
ABCDEF_10473	UniRef50_Q08A71	0.0	Normal: 374	187	332	425	456	314	BlastAnno: 1764	UniRef50_Q08A71	435	79.23	366	75	1	344	1438	70	435	0.0	575
ABCDEF_7295	UniRef50_Q9ZPH9	0.0	Normal: 25	54	30	81	26	329	BlastAnno: 1191	UniRef50_Q9ZPH9	633	69.23	403	110	4	1191	22	225	626	0.0	599
ABCDEF_4160	UniRef50_Q94II3	0.0	Normal: 189	0	210	71	17	72	BlastAnno: 1424	UniRef50_Q94II3	600	77.28	449	101	1	1424	81	143	591	0.0	758
ABCDEF_44857	UniRef50_Q9ZPH9	0.0	Normal: 101	224	103	299	588	350	BlastAnno: 1582	UniRef50_Q9ZPH9	633	72.06	519	143	1	1571	21	114	632	0.0	822
ABCDEF_71541	UniRef50_Q9SIZ3	0.0	Normal: 187	167	296	765	724	237	BlastAnno: 2268	UniRef50_Q9SIZ3	589	62.01	308	108	4	1210	287	285	583	0.0	394
ABCDEF_44171	UniRef50_Q9SIZ3	0.0	Normal: 214	196	312	827	802	251	BlastAnno: 2339	UniRef50_Q9SIZ3	589	64.78	548	182	5	1978	335	47	583	0.0	717
ABCDEF_20805	UniRef50_Q94II3	0.0	Normal: 674	344	885	1417	2194	963	BlastAnno: 2400	UniRef50_Q94II3	600	78.21	436	94	1	879	2183	156	591	0.0	734
ABCDEF_18210	UniRef50_Q9SIZ3	0.0	Normal: 5584	3714	7379	17006	21892	7546	BlastAnno: 3199	UniRef50_Q9SIZ3	589	50.00	576	269	7	2145	424	27	585	0.0	574
ABCDEF_6526	UniRef50_Q9ZPH9	0.0	Normal: 627	185	837	2717	2815	1530	BlastAnno: 2611	UniRef50_Q9ZPH9	633	54.76	599	259	5	462	2222	25	623	0.0	689
ABCDEF_6525	UniRef50_Q9ZPH9	0.0	Normal: 602	165	760	2369	2605	1400	BlastAnno: 2545	UniRef50_Q9ZPH9	633	54.92	599	258	5	431	2191	25	623	0.0	689
ABCDEF_6523	UniRef50_Q9ZPH9	0.0	Normal: 314	175	304	641	784	552	BlastAnno: 2312	UniRef50_Q9ZPH9	633	52.42	599	272	4	201	1958	25	623	0.0	663
ABCDEF_67393	UniRef50_Q9ZPH9	0.0	Normal: 434	245	444	608	768	655	BlastAnno: 2346	UniRef50_Q9ZPH9	633	51.92	599	275	4	232	1989	25	623	0.0	659
ABCDEF_3579	UniRef50_O14744	0.0	Normal: 1361	326	984	568	791	440	BlastAnno: 2369	UniRef50_O14744	637	47.00	634	294	10	267	2120	30	637	0.0	553

2.1.1.21(methylamine---glutamate N-methyltransferase;)

No Correspond Unigene

2.1.1.86(tetrahydromethanopterin S-methyltransferase;)

No Correspond Unigene

2.1.1.90(methanol---5-hydroxybenzimidazolylcobamide Co-methyltransferase;)

No Correspond Unigene

2.1.2.1(glycine hydroxymethyltransferase;)

ABCDEF_41184	UniRef50_C5KPS8	0.0	Normal: 509	437	722	1807	2474	561	BlastAnno: 2201	UniRef50_C5KPS8	607	62.56	446	164	2	1762	425	164	606	0.0	590
ABCDEF_11573	UniRef50_P34897	0.0	Normal: 1903	1387	2092	2286	3254	2865	BlastAnno: 2080	UniRef50_P34897	504	60.18	457	170	4	502	1845	48	501	0.0	573
ABCDEF_37629	UniRef50_Q9LM59	0.0	Normal: 412	460	499	689	962	979	BlastAnno: 2512	UniRef50_Q9LM59	599	66.00	597	187	5	369	2126	8	599	0.0	813
ABCDEF_7224	UniRef50_C5KPS8	0.0	Normal: 360	5392	231	401	266	13616	BlastAnno: 2246	UniRef50_C5KPS8	607	61.88	446	167	2	1828	491	164	606	0.0	577
ABCDEF_51490	UniRef50_Q9LM59	0.0	Normal: 497	502	504	755	1027	1332	BlastAnno: 2919	UniRef50_Q9LM59	599	64.38	584	197	2	578	2299	17	599	0.0	754
ABCDEF_1167	UniRef50_Q9LM59	0.0	Normal: 5116	9848	6479	16268	26840	14685	BlastAnno: 1891	UniRef50_Q9LM59	599	62.07	464	171	1	1747	371	134	597	0.0	611
ABCDEF_7223	UniRef50_C5KPS8	0.0	Normal: 395	5706	275	418	261	13912	BlastAnno: 2154	UniRef50_C5KPS8	607	61.88	446	167	2	1736	399	164	606	0.0	577
ABCDEF_28355	UniRef50_P34897	0.0	Normal: 255	239	370	837	1312	358	BlastAnno: 1812	UniRef50_P34897	504	57.73	466	153	3	1810	416	81	503	0.0	553

2.2.1.3(formaldehyde transketolase;)

ABCDEF_33843	UniRef50_B9T0B2	0.0	Normal: 1647	3396	1878	3084	2916	10247	BlastAnno: 2902	UniRef50_B9T0B2	789	56.01	616	264	6	2523	691	126	739	0.0	637
ABCDEF_33840	UniRef50_B9T0B2	0.0	Normal: 1558	3002	1682	2815	2569	8787	BlastAnno: 2829	UniRef50_B9T0B2	789	56.01	616	264	6	381	2213	126	739	0.0	638

2.3.1.-(Transferases;)

ABCDEF_520	UniRef50_Q9FF86	0.0	Normal: 1672	85	2273	5056	3383	1211	BlastAnno: 1811	UniRef50_Q9FF86	484	64.06	448	149	6	1664	354	3	449	0.0	573
ABCDEF_68664	UniRef50_Q9ZVN2	0.0	Normal: 79	35	85	107	193	63	BlastAnno: 1488	UniRef50_Q9ZVN2	704	63.99	411	142	3	46	1275	298	703	0.0	540
ABCDEF_52245	UniRef50_Q9FG87	0.0	Normal: 1878	635	1833	3814	1432	1376	BlastAnno: 1758	UniRef50_Q9FG87	529	55.35	486	204	4	4	1428	34	517	0.0	530
ABCDEF_16730	UniRef50_Q3EC11	0.0	Normal: 281	204	237	365	377	458	BlastAnno: 2092	UniRef50_Q3EC11	536	59.33	536	204	8	196	1785	3	530	0.0	661
ABCDEF_4655	UniRef50_A5BMG1	0.0	Normal: 2426	1856	3606	6958	4608	2755	BlastAnno: 2261	UniRef50_A5BMG1	481	68.37	528	112	10	263	1843	3	476	0.0	667
ABCDEF_13860	UniRef50_Q9C533	0.0	Normal: 588	220	570	1451	1338	310	BlastAnno: 2648	UniRef50_Q9C533	596	63.37	606	177	12	2226	511	1	595	0.0	558
ABCDEF_28294	UniRef50_Q9SYZ0	0.0	Normal: 1038	4070	1427	6745	877	2797	BlastAnno: 1785	UniRef50_Q9SYZ0	493	52.37	485	223	3	1598	162	5	487	0.0	533
ABCDEF_47718	UniRef50_Q9C533	0.0	Normal: 1599	236	1657	2093	1231	588	BlastAnno: 2680	UniRef50_Q9C533	596	65.02	626	165	14	2280	472	1	595	0.0	634
ABCDEF_45080	UniRef50_Q9SYZ0	0.0	Normal: 1005	592	1079	2360	3762	2217	BlastAnno: 2130	UniRef50_Q9SYZ0	493	57.85	484	197	3	302	1735	5	487	0.0	533
ABCDEF_25377	UniRef50_Q9C6L5	0.0	Normal: 37	2090	138	2573	1026	1298	BlastAnno: 1334	UniRef50_Q9C6L5	492	79.74	385	76	1	1155	1	6	388	0.0	654
ABCDEF_33470	UniRef50_Q9LW26	0.0	Normal: 172	98	156	260	391	149	BlastAnno: 2993	UniRef50_Q9LW26	701	48.96	627	301	5	655	2508	85	701	0.0	581
ABCDEF_40654	UniRef50_Q9H0A0	0.0	Normal: 638	213	530	318	318	286	BlastAnno: 2216	UniRef50_Q9H0A0	1025	56.87	721	279	6	2127	13	91	795	0.0	756
ABCDEF_33460	UniRef50_Q9ZVN2	0.0	Normal: 144	71	119	216	306	97	BlastAnno: 2691	UniRef50_Q9ZVN2	704	61.50	587	191	4	379	2133	83	636	0.0	715
ABCDEF_14442	UniRef50_Q9SYZ0	0.0	Normal: 341	684	439	1040	1601	552	BlastAnno: 1779	UniRef50_Q9SYZ0	493	63.12	480	171	2	1570	149	5	484	0.0	628
ABCDEF_52626	UniRef50_B6SYP9	0.0	Normal: 815	346	1131	3015	1537	108	BlastAnno: 1861	UniRef50_B6SYP9	494	55.02	438	189	3	1538	249	51	488	0.0	536

ABCDEF_644	UniRef50_Q9SB58	0.0	Normal: 704	444	913	969	1431	848	BlastAnno: 1777	UniRef50_Q9SB58	407	64.15	424	129	8	1572	316	1	406	0.0	554
ABCDEF_27087	UniRef50_Q52T38	0.0	Normal: 324	221	233	419	343	412	BlastAnno: 1631	UniRef50_Q52T38	620	75.95	420	91	1	1409	150	1	410	0.0	681
ABCDEF_34187	UniRef50_Q8L5Y5	0.0	Normal: 497	415	588	523	291	433	BlastAnno: 2677	UniRef50_Q8L5Y5	718	57.30	733	251	15	202	2274	1	713	0.0	683
ABCDEF_47966	UniRef50_Q9SYZ0	0.0	Normal: 577	934	326	580	682	3440	BlastAnno: 2177	UniRef50_Q9SYZ0	493	56.40	484	204	3	1780	347	5	487	0.0	535
ABCDEF_51189	UniRef50_Q52T38	0.0	Normal: 135	152	128	181	219	327	BlastAnno: 1766	UniRef50_Q52T38	620	68.09	470	137	3	80	1483	130	588	0.0	641
ABCDEF_41288	UniRef50_Q9SB58	0.0	Normal: 307	183	332	637	649	322	BlastAnno: 1403	UniRef50_Q9SB58	407	67.56	410	110	6	1401	184	15	405	0.0	523
ABCDEF_33461	UniRef50_Q9ZVN2	0.0	Normal: 142	84	140	206	283	119	BlastAnno: 2904	UniRef50_Q9ZVN2	704	60.98	574	201	6	2271	559	83	636	0.0	686
ABCDEF_14334	UniRef50_Q9SYZ0	0.0	Normal: 2263	129	1724	3469	866	283	BlastAnno: 2039	UniRef50_Q9SYZ0	493	63.22	484	171	2	369	1802	5	487	0.0	627
ABCDEF_24844	UniRef50_Q9FF86	0.0	Normal: 1857	3686	2657	21940	11467	4090	BlastAnno: 1640	UniRef50_Q9FF86	484	66.89	447	135	6	1492	185	2	446	0.0	575
ABCDEF_51190	UniRef50_Q52T38	0.0	Normal: 145	169	142	210	232	335	BlastAnno: 1891	UniRef50_Q52T38	620	70.04	514	141	3	73	1608	86	588	0.0	726
ABCDEF_14085	UniRef50_Q8L5Y5	0.0	Normal: 353	41	384	527	331	280	BlastAnno: 2362	UniRef50_Q8L5Y5	718	62.07	704	220	12	2360	348	24	713	0.0	676
ABCDEF_33458	UniRef50_Q9LW26	0.0	Normal: 158	78	130	240	342	106	BlastAnno: 2822	UniRef50_Q9LW26	701	46.72	655	302	5	400	2337	85	701	0.0	568
ABCDEF_25193	UniRef50_Q9C6L5	0.0	Normal: 3256	368	3548	6230	5109	1288	BlastAnno: 1786	UniRef50_Q9C6L5	492	65.83	480	156	4	1668	241	17	492	0.0	668
ABCDEF_33457	UniRef50_Q9ZVN2	0.0	Normal: 168	83	137	250	375	112	BlastAnno: 2823	UniRef50_Q9ZVN2	704	65.12	496	168	3	379	1860	83	575	0.0	648
ABCDEF_7634	UniRef50_Q9C6L5	0.0	Normal: 2543	9303	2711	11578	5533	10032	BlastAnno: 2064	UniRef50_Q9C6L5	492	52.76	489	225	3	1734	268	1	483	0.0	535
ABCDEF_299	UniRef50_Q71N54	0.0	Normal: 569	1147	511	900	649	1879	BlastAnno: 2017	UniRef50_Q71N54	535	61.73	533	198	5	338	1924	4	534	0.0	677
ABCDEF_27086	UniRef50_Q52T38	0.0	Normal: 784	470	617	843	893	976	BlastAnno: 2398	UniRef50_Q52T38	620	70.80	637	165	4	225	2129	1	618	0.0	887
ABCDEF_51188	UniRef50_Q52T38	0.0	Normal: 273	327	255	415	349	441	BlastAnno: 2324	UniRef50_Q52T38	620	71.30	568	150	3	2041	344	32	588	0.0	815
ABCDEF_44812	UniRef50_Q6DR03	0.0	Normal: 982	902	1005	1071	959	2999	BlastAnno: 2450	UniRef50_Q6DR03	565	54.97	644	198	17	2142	220	1	555	0.0	540
2.3.1.101(formylmethanofuran---tetrahydromethanopterin N-formyltransferase;)																					
No Correspond Unigene																					
2.3.1.169(CO-methylating acetyl-CoA synthase)																					
No Correspond Unigene																					
2.3.1.8(phosphate acetyltransferase;)																					
ABCDEF_10009	UniRef50_B6KAY1	6e-23	Normal: 14	16	19	16	34	38	BlastAnno: 693	UniRef50_B6KAY1	1296	48.51	101	51	1	154	453	1128	1228	6e-23	104
2.6.1.45(serine---glyoxylate transaminase)																					
ABCDEF_9789	UniRef50_Q56YA5	0.0	Normal: 130	3261	66	26	15	8034	BlastAnno: 1692	UniRef50_Q56YA5	401	85.29	333	49	0	352	1350	69	401	0.0	583
ABCDEF_9785	UniRef50_Q56YA5	0.0	Normal: 191	4834	126	51	21	10532	BlastAnno: 1764	UniRef50_Q56YA5	401	85.79	401	57	0	1614	412	1	401	0.0	712
2.6.1.52(phosphoserine transaminase;)																					
ABCDEF_41302	UniRef50_Q96255	0.0	Normal: 1671	665	2009	2718	2176	624	BlastAnno: 1822	UniRef50_Q96255	430	80.05	391	73	3	1572	406	43	430	0.0	653
ABCDEF_28393	UniRef50_Q96255	0.0	Normal: 390	227	489	822	2225	158	BlastAnno: 1446	UniRef50_Q96255	430	78.83	392	79	2	5	1180	42	429	0.0	627
2.7.1.11(6-phosphofructokinase;)																					
ABCDEF_48463	UniRef50_Q94AA4	0.0	Normal: 273	212	275	545	518	593	BlastAnno: 1885	UniRef50_Q94AA4	489	71.85	437	122	1	403	1713	17	452	0.0	681
ABCDEF_48462	UniRef50_Q9FKG3	0.0	Normal: 197	165	201	395	395	441	BlastAnno: 1773	UniRef50_Q9FKG3	530	76.95	295	61	2	316	1197	44	332	0.0	469
ABCDEF_29376	UniRef50_Q94AA4	0.0	Normal: 0	44	0	0	20	222	BlastAnno: 1555	UniRef50_Q94AA4	489	81.00	458	82	3	99	1472	8	460	0.0	756
ABCDEF_49371	UniRef50_Q94AA4	0.0	Normal: 16	353	32	5	82	64	BlastAnno: 1513	UniRef50_Q94AA4	489	77.23	470	98	3	30	1421	10	476	0.0	752
ABCDEF_48301	UniRef50_Q8VYN6	0.0	Normal: 298	135	430	474	1016	200	BlastAnno: 2035	UniRef50_Q8VYN6	537	78.25	538	112	3	128	1729	1	537	0.0	844
ABCDEF_41341	UniRef50_Q94AA4	0.0	Normal: 889	655	932	1360	2142	1363	BlastAnno: 2130	UniRef50_Q94AA4	489	80.89	450	85	1	1832	483	6	454	0.0	755
2.7.1.29(glycerone kinase;)																					
ABCDEF_19659	UniRef50_Q3LXA3	6e-108	Normal: 200	133	145	233	228	183	BlastAnno: 2278	UniRef50_Q3LXA3	575	39.45	578	322	11	2026	317	1	558	6e-108	349
2.7.1.31(glycerate kinase;)																					
ABCDEF_10301	UniRef50_Q944I4	0.0	Normal: 259	552	306	527	545	1533	BlastAnno: 1595	UniRef50_Q944I4	456	72.85	372	99	1	272	1387	86	455	0.0	580
ABCDEF_10303	UniRef50_Q944I4	0.0	Normal: 266	510	294	565	522	1374	BlastAnno: 1563	UniRef50_Q944I4	456	68.43	396	99	2	1292	105	86	455	0.0	565
2.7.2.1(acetate kinase;)																					
No Correspond Unigene																					
2.7.9.2(pyruvate, water dikinase;)																					
No Correspond Unigene																					
2.8.4.1(coenzyme-B sulfoethylthiotransferase;)																					
No Correspond Unigene																					
3.1.2.12(S-formylglutathione hydrolase)																					
ABCDEF_38717	UniRef50_Q8LAS8	4e-166	Normal: 1716	1661	1554	1829	2661	3085	BlastAnno: 1286	UniRef50_Q8LAS8	284	80.71	280	54	0	1174	335	1	280	4e-166	477
3.1.3.11(fructose-bisphosphatase;)																					
ABCDEF_28302	UniRef50_P25851	0.0	Normal: 1013	1937	653	1129	1293	5773	BlastAnno: 1619	UniRef50_P25851	417	66.49	382	125	2	1417	281	36	417	0.0	544
ABCDEF_35263	UniRef50_Q9XGG5	0.0	Normal: 72	1508	41	126	415	7941	BlastAnno: 1577	UniRef50_Q9XGG5	329	92.62	325	24	0	373	1347	1	325	0.0	627
ABCDEF_7505	UniRef50_P25851	0.0	Normal: 1194	5939	568	263	269	21425	BlastAnno: 1662	UniRef50_P25851	417	81.75	389	66	3	1369	218	29	417	0.0	613
3.1.3.3(phosphoserine phosphatase)																					
ABCDEF_73777	UniRef50_O82796	8e-138	Normal: 234	113	241	451	692	447	BlastAnno: 1098	UniRef50_O82796	295	67.71	288	93	0	95	958	8	295	8e-138	403
ABCDEF_15135	UniRef50_O82796	8e-138	Normal: 265	126	260	482	787	488	BlastAnno: 1173	UniRef50_O82796	295	66.44	295	99	0	136	1020	1	295	8e-138	404
3.1.3.71(2-phosphosulfolactate phosphatase;)																					
No Correspond Unigene																					
3.5.4.27(methenyltetrahydromethanopterin cyclohydrolase;)																					
No Correspond Unigene																					
3.6.3.14(H+-transporting two-sector ATPase;)																					
ABCDEF_38143	UniRef50_C8W8D3	0.0	Normal: 188	240	462	512	364	584	BlastAnno: 1786	UniRef50_C8W8D3	557	53.40	530	217	5	1650	121	7	526	0.0	544
ABCDEF_750	UniRef50_Q3A946	0.0	Normal: 8200	4736	9322	16172	23565	9462	BlastAnno: 2234	UniRef50_Q3A946	473	71.62	451	111	3	441	1766	18	460	0.0	657
ABCDEF_31686	UniRef50_Q3A946	0.0	Normal: 4796	2678	5669	8971	9245	4913	BlastAnno: 2182	UniRef50_Q3A946	473	71.43	448	111	3	448	1764	18	457	0.0	650
ABCDEF_81842	UniRef50_P06576	0.0	Normal: 0	6	0	0	0	192	BlastAnno: 868	UniRef50_P06576	529	88.93	289	32	0	1	867	239	527	0.0	525
ABCDEF_6666	UniRef50_B8ZR40	0.0	Normal: 131	564	209	94	213	1741	BlastAnno: 2588	UniRef50_B8ZR40	558	57.06	503	206	2	772	2253	4	505	0.0	553
ABCDEF_44101	UniRef50_A2BKX6	0.0	Normal: 1138	1254	1508	2332	4942	1782	BlastAnno: 2483	UniRef50_A2BKX6	601	49.75	599	285	5	2117	327	5	589	0.0	576
ABCDEF_6665	UniRef50_C3W009	0.0	Normal: 363	1495	714	290	657	5105	BlastAnno: 3324	UniRef50_C3W009	507	71.65	462	131	0	1386	1	1	462	0.0	597
ABCDEF_44104	UniRef50_A2BKX6	0.0	Normal: 1709	1358	1590	2988	6608	3458	BlastAnno: 2470	UniRef50_A2BKX6	601	49.92	599	284	5	252	2042	5	589	0.0	562
ABCDEF_38059	UniRef50_Q3A946	0.0	Normal: 7449	20106	6978	6819	10389	50205	BlastAnno: 3486	UniRef50_Q3A946	473	70.43	460	124	2	3220	1856	18	470	0.0	659

	ABCDEF_64176	UniRef50_P38606	0.0	Normal: 625	683	721	1302	2664	866	BlastAnno: 1815	UniRef50_P38606	617	75.20	496	121	2	330	1814	11	505	0.0	749
4.1.1.31(phosphoenolpyruvate carboxylase;)																						
	ABCDEF_64086	UniRef50_Q6R2V6	0.0	Normal: 363	569	289	463	784	348	BlastAnno: 2123	UniRef50_Q6R2V6	1221	61.79	560	205	4	163	1821	664	1221	0.0	650
	ABCDEF_41385	UniRef50_B5FPX1	0.0	Normal: 4314	701	3865	3687	4278	3624	BlastAnno: 3374	UniRef50_B5FPX1	883	40.60	941	490	14	3058	260	4	883	0.0	634
	ABCDEF_62655	UniRef50_Q6R2V6	0.0	Normal: 335	542	261	432	710	315	BlastAnno: 2054	UniRef50_Q6R2V6	1221	61.79	560	205	4	163	1821	664	1221	0.0	650
	ABCDEF_5143	UniRef50_Q6R2V6	0.0	Normal: 847	1445	623	1238	1720	785	BlastAnno: 3592	UniRef50_Q6R2V6	1221	57.46	637	243	6	1451	3292	590	1221	0.0	661
	ABCDEF_34325	UniRef50_B5FPX1	0.0	Normal: 1019	396	1067	2133	3237	977	BlastAnno: 3428	UniRef50_B5FPX1	883	41.15	943	483	13	3011	210	4	883	0.0	656
	ABCDEF_49210	UniRef50_Q6R2V6	0.0	Normal: 884	1485	676	1279	1833	822	BlastAnno: 3747	UniRef50_Q6R2V6	1221	57.46	637	243	6	2261	420	590	1221	0.0	661
	ABCDEF_34323	UniRef50_B5FPX1	0.0	Normal: 1026	368	1024	2181	3307	986	BlastAnno: 3450	UniRef50_B5FPX1	883	41.15	943	483	13	440	3241	4	883	0.0	656
	ABCDEF_13966	UniRef50_P15804	0.0	Normal: 86	248	71	71	214	123	BlastAnno: 2130	UniRef50_P15804	960	74.85	672	161	4	2130	133	291	960	0.0	1010
	ABCDEF_13967	UniRef50_P15804	0.0	Normal: 75	236	61	55	191	97	BlastAnno: 2000	UniRef50_P15804	960	74.96	619	149	3	28	1872	344	960	0.0	925
4.1.1.79(sulfopyruvate decarboxylase;)																						
	No Correspond Unigene																					
4.1.2.13(fructose-bisphosphate aldolase;)																						
	ABCDEF_812	UniRef50_Q40677	0.0	Normal: 2391	1243	3255	6494	7322	1031	BlastAnno: 1720	UniRef50_Q40677	388	81.39	360	67	0	362	1441	29	388	0.0	612
	ABCDEF_4038	UniRef50_Q40677	0.0	Normal: 2914	23962	1997	525	623	51538	BlastAnno: 1570	UniRef50_Q40677	388	82.87	397	53	3	123	1301	3	388	0.0	641
	ABCDEF_35511	UniRef50_B9FU33	0.0	Normal: 18	1991	3	12	56	2290	BlastAnno: 1432	UniRef50_B9FU33	559	71.11	398	101	3	1423	245	171	559	0.0	536
	ABCDEF_14742	UniRef50_Q40677	0.0	Normal: 4220	14155	2670	281	395	26738	BlastAnno: 1587	UniRef50_Q40677	388	89.94	348	35	0	1291	248	41	388	0.0	657
4.1.2.32(trimethylamine-oxide aldolase;)																						
	No Correspond Unigene																					
4.1.2.43(3-hexulose-6-phosphate synthase;)																						
	No Correspond Unigene																					
4.1.2.9(phosphoketolase;)																						
	No Correspond Unigene																					
4.1.3.24(malyl-CoA lyase;)																						
	No Correspond Unigene																					
4.2.1.11(phosphopyruvate hydratase;)																						
	ABCDEF_204	UniRef50_A5B6U7	0.0	Normal: 577	318	663	998	1166	662	BlastAnno: 1992	UniRef50_A5B6U7	527	68.14	452	89	3	196	1551	1	397	0.0	613
	ABCDEF_51801	UniRef50_Q6BI20	0.0	Normal: 883	270	1626	3195	3612	300	BlastAnno: 1822	UniRef50_Q6BI20	439	64.27	431	143	6	1472	201	9	435	0.0	537
	ABCDEF_4784	UniRef50_P06733	0.0	Normal: 8739	4441	8587	11774	22445	7368	BlastAnno: 1781	UniRef50_P06733	434	70.81	442	115	8	1588	272	2	432	0.0	618
	ABCDEF_48464	UniRef50_P06733	0.0	Normal: 3032	1633	3013	4940	8155	2527	BlastAnno: 1829	UniRef50_P06733	434	71.27	442	113	8	242	1558	2	432	0.0	621
4.3.-.-(Lyases;)																						
	No Correspond Unigene																					
4.4.1.19(phosphosulfolactate synthase;)																						
	ABCDEF_42685	UniRef50_B8D0I9	3e-06	Normal: 45	36	16	12	82	62	BlastAnno: 559	UniRef50_B8D0I9	280	31.58	133	83	4	433	38	18	143	3e-06	52.0
4.4.1.22(S-(hydroxymethyl)glutathione synthase;)																						
	No Correspond Unigene																					
5.3.1.27(6-phospho-3-hexuloisomerase;)																						
	No Correspond Unigene																					
5.4.2.1(phosphoglycerate mutase;)																						
	ABCDEF_45789	UniRef50_O04499	0.0	Normal: 6087	3713	6051	11349	14624	5679	BlastAnno: 2153	UniRef50_O04499	557	83.81	556	90	0	1962	295	2	557	0.0	942
6.2.1.1(acetate---CoA ligase;)																						
	ABCDEF_65173	UniRef50_Q9VP61	0.0	Normal: 208	202	219	336	383	409	BlastAnno: 2249	UniRef50_Q9VP61	670	54.63	540	236	3	539	2131	4	543	0.0	629
	ABCDEF_51889	UniRef50_Q9VP61	0.0	Normal: 268	268	315	436	574	651	BlastAnno: 2148	UniRef50_Q9VP61	670	55.64	638	273	4	2060	174	26	662	0.0	723
	ABCDEF_51888	UniRef50_A8JFR9	0.0	Normal: 348	343	408	583	709	775	BlastAnno: 2704	UniRef50_A8JFR9	667	56.57	647	273	5	2090	168	20	664	0.0	712
	ABCDEF_59631	UniRef50_Q9VP61	0.0	Normal: 132	127	125	187	249	287	BlastAnno: 1733	UniRef50_Q9VP61	670	55.41	518	222	3	89	1615	26	543	0.0	610
6.2.1.9(malate---CoA ligase;)																						
	ABCDEF_10877	UniRef50_P53595	5e-94	Normal: 1436	691	1429	2257	2843	867	BlastAnno: 1600	UniRef50_P53595	296	52.04	294	137	2	1323	451	2	294	5e-94	297
>00710 Carbon fixation in photosynthetic organisms																						
1.1.1.37(malate dehydrogenase;)																						
	ABCDEF_25169	UniRef50_Q9SN86	0.0	Normal: 938	614	1116	1317	4446	973	BlastAnno: 1856	UniRef50_Q9SN86	403	75.50	400	88	2	1616	417	2	391	0.0	585
	ABCDEF_31746	UniRef50_Q9ZP05	0.0	Normal: 611	6985	354	98	71	19818	BlastAnno: 1631	UniRef50_Q9ZP05	354	83.38	349	54	2	274	1317	9	354	0.0	578
	ABCDEF_32230	UniRef50_P57106	0.0	Normal: 8408	4627	7877	10286	18779	11078	BlastAnno: 1523	UniRef50_P57106	332	93.07	332	23	0	262	1257	1	332	0.0	617
	ABCDEF_57415	UniRef50_Q9ZP05	0.0	Normal: 403	336	343	761	845	611	BlastAnno: 1190	UniRef50_Q9ZP05	354	83.29	353	54	2	1058	3	1	349	0.0	579
	ABCDEF_17941	UniRef50_Q9ZP05	0.0	Normal: 616	554	586	1025	1231	910	BlastAnno: 1567	UniRef50_Q9ZP05	354	83.63	342	51	2	1336	314	17	354	0.0	563
1.1.1.39(malate dehydrogenase (decarboxylating);)																						
	ABCDEF_76313	UniRef50_P37221	0.0	Normal: 942	772	1313	2259	2342	1405	BlastAnno: 2331	UniRef50_P37221	626	65.87	583	193	1	2153	423	42	624	0.0	661
	ABCDEF_11069	UniRef50_P37221	0.0	Normal: 913	1050	959	1783	2411	1423	BlastAnno: 2200	UniRef50_P37221	626	87.90	628	73	2	2190	310	1	626	0.0	1046
	ABCDEF_11068	UniRef50_P37221	0.0	Normal: 894	1051	947	1766	2300	1417	BlastAnno: 2244	UniRef50_P37221	626	87.90	628	73	2	2158	278	1	626	0.0	1045
	ABCDEF_35547	UniRef50_P37221	0.0	Normal: 1071	798	1206	2226	2090	1293	BlastAnno: 2532	UniRef50_P37221	626	66.04	583	192	1	399	2129	42	624	0.0	661
1.1.1.40(malate dehydrogenase (oxaloacetate-decarboxylating) (NADP+);)																						
	ABCDEF_7664	UniRef50_P43279	0.0	Normal: 1619	1000	1705	548	2995	3374	BlastAnno: 2126	UniRef50_P43279	639	78.41	565	117	1	131	1825	74	633	0.0	879
	ABCDEF_24790	UniRef50_P43279	0.0	Normal: 452	150	359	466	361	366	BlastAnno: 1480	UniRef50_P43279	639	78.02	373	75	2	1121	3	58	423	0.0	567
	ABCDEF_4172	UniRef50_Q16798	0.0	Normal: 3896	8020	3800	3763	4801	9522	BlastAnno: 2536	UniRef50_Q16798	604	50.56	540	264	3	1988	372	50	587	0.0	556
	ABCDEF_4544	UniRef50_P43279	0.0	Normal: 12	3	39	281	184	0	BlastAnno: 1263	UniRef50_P43279	639	78.18	417	91	0	2	1252	223	639	0.0	660
1.1.1.82(malate dehydrogenase (NADP+);)																						
	ABCDEF_11352	UniRef50_P37229	0.0	Normal: 857	3023	663	550	982	9189	BlastAnno: 1914	UniRef50_P37229	432	83.25	400	64	2	371	1561	32	431	0.0	661
1.2.1.13(glyceraldehyde-3-phosphate dehydrogenase (NADP+) (phosphorylating);)																						
	ABCDEF_28550	UniRef50_P25857	0.0	Normal: 5628	12889	3893	2309	3913	34587	BlastAnno: 1889	UniRef50_P25857	447	86.43	442	52	3	1545	232	10	447	0.0	705
1.2.1.59(glyceraldehyde-3-phosphate dehydrogenase (NAD(P)+) (phosphorylating);)																						
	No Correspond Unigene																					
2.2.1.1(transketolase;)																						

ABCDEF_33843	UniRef50_P45694	0.0	Normal: 1647	3396	1878	3084	2916	10247	BlastAnno: 2902	UniRef50_P45694	667	52.71	664	298	8	2559	583	1	653	0.0	620
ABCDEF_33839	UniRef50_Q43848	0.0	Normal: 564	1182	798	1094	1060	3501	BlastAnno: 1311	UniRef50_Q43848	741	92.11	304	23	1	1	909	426	729	0.0	537
ABCDEF_33840	UniRef50_P45694	0.0	Normal: 1558	3002	1682	2815	2569	8787	BlastAnno: 2829	UniRef50_P45694	667	52.71	664	298	8	345	2321	1	653	0.0	619
2.6.1.1(aspartate transaminase;)																					
ABCDEF_11690	UniRef50_P46248	0.0	Normal: 1745	1815	2022	4078	5299	6095	BlastAnno: 1778	UniRef50_P46248	453	86.09	417	58	0	297	1547	37	453	0.0	733
ABCDEF_1333	UniRef50_C0PSV4	0.0	Normal: 356	544	636	1119	2495	631	BlastAnno: 1594	UniRef50_C0PSV4	462	76.43	403	95	0	1401	193	60	462	0.0	662
ABCDEF_21786	UniRef50_C0PSV4	0.0	Normal: 268	668	330	546	1267	646	BlastAnno: 1736	UniRef50_C0PSV4	462	74.14	464	113	3	50	1441	4	460	0.0	711
ABCDEF_42262	UniRef50_P46643	0.0	Normal: 916	606	1656	2699	2553	856	BlastAnno: 1722	UniRef50_P46643	430	79.49	429	85	2	1635	355	1	428	0.0	731
ABCDEF_56929	UniRef50_P37833	0.0	Normal: 173	278	312	654	1243	297	BlastAnno: 1151	UniRef50_P37833	407	81.82	308	56	0	978	55	3	310	0.0	536
2.6.1.2(alanine transaminase;)																					
ABCDEF_32120	UniRef50_P34106	0.0	Normal: 1312	2052	1485	2115	4153	2982	BlastAnno: 2449	UniRef50_P34106	482	78.87	478	101	0	652	2085	5	482	0.0	763
2.7.1.14(sedoheptulokinase;)																					
No Correspond Unigene																					
2.7.1.19(phosphoribulokinase;)																					
ABCDEF_14565	UniRef50_P25697	0.0	Normal: 1506	10830	1092	553	1011	33693	BlastAnno: 2550	UniRef50_P25697	395	82.88	403	57	4	236	1432	1	395	0.0	689
2.7.1.40(pyruvate kinase;)																					
ABCDEF_27872	UniRef50_Q40545	0.0	Normal: 1220	1217	1378	1640	1974	4469	BlastAnno: 2233	UniRef50_Q40545	593	81.65	594	96	4	1951	206	1	593	0.0	979
ABCDEF_34901	UniRef50_B9EYF5	0.0	Normal: 3919	1884	3179	3852	4333	5138	BlastAnno: 2384	UniRef50_B9EYF5	606	80.36	555	84	2	2021	432	52	606	0.0	922
ABCDEF_10813	UniRef50_Q2QXR7	0.0	Normal: 2566	2379	2864	4497	6222	4202	BlastAnno: 2019	UniRef50_Q2QXR7	408	85.57	402	58	0	174	1379	1	402	0.0	727
ABCDEF_35590	UniRef50_Q9M057	0.0	Normal: 2212	2122	2749	5601	7830	2637	BlastAnno: 1980	UniRef50_Q9M057	510	69.58	503	148	4	1830	334	1	502	0.0	687
ABCDEF_27885	UniRef50_Q2QXR7	0.0	Normal: 924	752	1120	2229	3166	928	BlastAnno: 2102	UniRef50_Q2QXR7	408	87.31	402	51	0	182	1387	1	402	0.0	729
ABCDEF_21818	UniRef50_Q40545	0.0	Normal: 1982	1309	1439	2275	1999	2303	BlastAnno: 2062	UniRef50_Q40545	593	69.25	491	147	2	1594	134	103	593	0.0	716
ABCDEF_31300	UniRef50_Q40545	0.0	Normal: 1326	1019	1016	1433	1396	1865	BlastAnno: 1805	UniRef50_Q40545	593	91.70	494	41	0	1802	321	100	593	0.0	920
ABCDEF_45529	UniRef50_B9EYF5	0.0	Normal: 1474	625	1452	3814	2571	3121	BlastAnno: 2114	UniRef50_B9EYF5	606	78.56	569	89	3	1810	179	46	606	0.0	912
ABCDEF_45536	UniRef50_Q2QXR7	0.0	Normal: 1644	1099	1596	1290	5936	335	BlastAnno: 1959	UniRef50_Q2QXR7	408	81.59	402	74	0	1862	657	1	402	0.0	687
ABCDEF_27886	UniRef50_Q2QXR7	0.0	Normal: 771	640	954	1807	2731	814	BlastAnno: 1787	UniRef50_Q2QXR7	408	86.82	349	46	0	54	1100	54	402	0.0	632
ABCDEF_18187	UniRef50_Q9M057	0.0	Normal: 3137	1498	3397	5489	6703	1765	BlastAnno: 1912	UniRef50_Q9M057	510	67.21	494	158	3	216	1685	17	510	0.0	681
ABCDEF_45028	UniRef50_B9EYF5	0.0	Normal: 273	415	192	360	372	144	BlastAnno: 1785	UniRef50_B9EYF5	606	73.94	495	105	2	372	1784	111	605	0.0	729
2.7.2.3(phosphoglycerate kinase;)																					
ABCDEF_38934	UniRef50_B8LKQ1	0.0	Normal: 7822	4290	7908	11136	16389	8199	BlastAnno: 1790	UniRef50_B8LKQ1	472	76.90	407	58	2	261	1481	97	467	0.0	580
ABCDEF_28557	UniRef50_B8LKQ1	0.0	Normal: 2514	10526	2985	3890	5050	32250	BlastAnno: 2074	UniRef50_B8LKQ1	472	77.70	408	56	1	1523	300	98	470	0.0	630
2.7.9.1(pyruvate, phosphate dikinase;)																					
ABCDEF_21316	UniRef50_B9RSV8	0.0	Normal: 3	411	12	10	27	459	BlastAnno: 1538	UniRef50_B9RSV8	872	87.65	421	49	1	284	1537	68	488	0.0	774
ABCDEF_20732	UniRef50_A9F4I0	0.0	Normal: 9	376	9	13	38	528	BlastAnno: 1760	UniRef50_A9F4I0	920	59.76	497	187	5	1760	294	407	898	0.0	588
ABCDEF_20731	UniRef50_O23404	0.0	Normal: 14	241	6	10	27	401	BlastAnno: 1677	UniRef50_O23404	963	84.32	338	52	1	401	1414	626	962	0.0	604
3.1.3.11(fructose-bisphosphatase;)																					
ABCDEF_28302	UniRef50_P25851	0.0	Normal: 1013	1937	653	1129	1293	5773	BlastAnno: 1619	UniRef50_P25851	417	66.49	382	125	2	1417	281	36	417	0.0	544
ABCDEF_35263	UniRef50_Q9XGG5	0.0	Normal: 72	1508	41	126	415	7941	BlastAnno: 1577	UniRef50_Q9XGG5	329	92.62	325	24	0	373	1347	1	325	0.0	627
ABCDEF_7505	UniRef50_P25851	0.0	Normal: 1194	5939	568	263	269	21425	BlastAnno: 1662	UniRef50_P25851	417	81.75	389	66	3	1369	218	29	417	0.0	613
3.1.3.37(sedoheptulose-bisphosphatase;)																					
ABCDEF_7816	UniRef50_P46283	0.0	Normal: 1776	6982	1128	540	649	22164	BlastAnno: 1692	UniRef50_P46283	393	79.60	397	77	3	276	1466	1	393	0.0	632
4.1.1.31(phosphoenolpyruvate carboxylase;)																					
ABCDEF_64086	UniRef50_Q6R2V6	0.0	Normal: 363	569	289	463	784	348	BlastAnno: 2123	UniRef50_Q6R2V6	1221	61.79	560	205	4	163	1821	664	1221	0.0	650
ABCDEF_41385	UniRef50_B5FPX1	0.0	Normal: 4314	701	3865	3687	4278	3624	BlastAnno: 3374	UniRef50_B5FPX1	883	40.60	941	490	14	3058	260	4	883	0.0	634
ABCDEF_62655	UniRef50_Q6R2V6	0.0	Normal: 335	542	261	432	710	315	BlastAnno: 2054	UniRef50_Q6R2V6	1221	61.79	560	205	4	163	1821	664	1221	0.0	650
ABCDEF_5143	UniRef50_Q6R2V6	0.0	Normal: 847	1445	623	1238	1720	785	BlastAnno: 3592	UniRef50_Q6R2V6	1221	57.46	637	243	6	1451	3292	590	1221	0.0	661
ABCDEF_34325	UniRef50_B5FPX1	0.0	Normal: 1019	396	1067	2133	3237	977	BlastAnno: 3428	UniRef50_B5FPX1	883	41.15	943	483	13	3011	210	4	883	0.0	656
ABCDEF_49210	UniRef50_Q6R2V6	0.0	Normal: 884	1485	676	1279	1833	822	BlastAnno: 3747	UniRef50_Q6R2V6	1221	57.46	637	243	6	2261	420	590	1221	0.0	661
ABCDEF_34323	UniRef50_B5FPX1	0.0	Normal: 1026	368	1024	2181	3307	986	BlastAnno: 3450	UniRef50_B5FPX1	883	41.15	943	483	13	440	3241	4	883	0.0	656
ABCDEF_13966	UniRef50_P15804	0.0	Normal: 86	248	71	71	214	123	BlastAnno: 2130	UniRef50_P15804	960	74.85	672	161	4	2130	133	291	960	0.0	1010
ABCDEF_13967	UniRef50_P15804	0.0	Normal: 75	236	61	55	191	97	BlastAnno: 2000	UniRef50_P15804	960	74.96	619	149	3	28	1872	344	960	0.0	925
4.1.1.39(ribulose-bisphosphate carboxylase;)																					
ABCDEF_38101	UniRef50_Q8WKC9	0.0	Normal: 193	868	327	479	300	4925	BlastAnno: 1900	UniRef50_Q8WKC9	418	95.22	418	20	0	334	1587	1	418	0.0	798
4.1.1.49(phosphoenolpyruvate carboxykinase (ATP);)																					
ABCDEF_2793	UniRef50_P10963	0.0	Normal: 99	228	79	1160	39	318	BlastAnno: 2459	UniRef50_P10963	549	52.91	567	219	11	402	2084	24	548	0.0	564
ABCDEF_81273	UniRef50_Q9T074	0.0	Normal: 86	204	69	1021	35	282	BlastAnno: 2611	UniRef50_Q9T074	671	76.69	369	78	5	2606	1512	8	372	0.0	565
ABCDEF_81242	UniRef50_Q9T074	0.0	Normal: 95	225	76	1100	40	293	BlastAnno: 2538	UniRef50_Q9T074	671	75.83	480	102	7	2533	1124	8	483	0.0	693
ABCDEF_2794	UniRef50_Q9T074	0.0	Normal: 114	289	99	1477	44	337	BlastAnno: 2733	UniRef50_Q9T074	671	76.69	369	78	5	2614	1520	8	372	0.0	565
ABCDEF_2796	UniRef50_Q9T074	0.0	Normal: 127	322	105	1687	47	387	BlastAnno: 2614	UniRef50_Q9T074	671	90.23	399	39	0	1572	376	273	671	0.0	738
ABCDEF_85588	UniRef50_Q9T074	0.0	Normal: 41	99	29	443	15	186	BlastAnno: 2024	UniRef50_Q9T074	671	80.55	329	35	1	1362	376	372	671	0.0	550
ABCDEF_79512	UniRef50_Q9T074	0.0	Normal: 104	244	82	1189	43	318	BlastAnno: 2451	UniRef50_Q9T074	671	75.83	480	102	7	2446	1037	8	483	0.0	693
ABCDEF_2797	UniRef50_P10963	0.0	Normal: 136	339	120	1738	54	398	BlastAnno: 2486	UniRef50_P10963	549	55.58	538	220	10	1971	376	24	548	0.0	576
ABCDEF_89373	UniRef50_Q9T074	0.0	Normal: 55	130	40	611	21	222	BlastAnno: 1864	UniRef50_Q9T074	671	84.11	428	39	1	1659	376	273	671	0.0	721
ABCDEF_2795	UniRef50_Q9T074	0.0	Normal: 49	120	40	536	20	212	BlastAnno: 1946	UniRef50_Q9T074	671	88.33	300	35	0	1275	376	372	671	0.0	565
ABCDEF_2792	UniRef50_Q9T074	0.0	Normal: 88	218	76	1085	36	308	BlastAnno: 2532	UniRef50_Q9T074	671	88.33	300	35	0	1258	2157	372	671	0.0	565
4.1.2.13(fructose-bisphosphate aldolase;)																					
ABCDEF_812	UniRef50_Q40677	0.0	Normal: 2391	1243	3255	6494	7322	1031	BlastAnno: 1720	UniRef50_Q40677	388	81.39	360	67	0	362	1441	29	388	0.0	612
ABCDEF_4038	UniRef50_Q40677	0.0	Normal: 2914	23962	1997	525	623	51538	BlastAnno: 1570	UniRef50_Q40677	388	82.87	397	53	3	123	1301	3	388	0.0	641
ABCDEF_35511	UniRef50_B9FU33	0.0	Normal: 18	1991	3	12	56	2290	BlastAnno: 1432	UniRef50_B9FU33	559	71.11	398	101	3	1423	245	171	559	0.0	536
ABCDEF_14742	UniRef50_Q40677	0.0	Normal: 4220	14155	2670	281	395	26738	BlastAnno: 1587	UniRef50_Q40677	388	89.94	348	35	0	1291	248	41	388	0.0	657
4.1.2.22(fructose-6-phosphate phosphoketolase;)																					
No Correspond Unigene																					

4.1.2.9(phosphoketolase;)

No Correspond Unigene

5.1.3.1(ribulose-phosphate 3-epimerase;)

ABCDEF_45834	UniRef50_Q9ZTP5	4e-147	Normal: 2109	7922	1619	1068	1971	21649	BlastAnno: 1506	UniRef50_Q9ZTP5	274	91.77	243	18	1	440	1168	32	272	4e-147	431

5.3.1.1(triose-phosphate isomerase;)

ABCDEF_25548	UniRef50_Q9SKP6	8e-167	Normal: 879	1226	1007	1772	2081	5167	BlastAnno: 1419	UniRef50_Q9SKP6	315	75.95	316	70	3	1221	289	1	315	8e-167	482

5.3.1.6(ribose-5-phosphate isomerase;)

ABCDEF_35205	UniRef50_B8FL28	3e-60	Normal: 177	481	176	230	243	502	BlastAnno: 1012	UniRef50_B8FL28	230	51.07	233	103	4	905	207	3	224	3e-60	201

>00720 Carbon fixation pathways in prokaryotes

1.1.1.-(Oxidoreductases;)

ABCDEF_13453	UniRef50_Q9SYM5	0.0	Normal: 225	226	398	1950	2026	421	BlastAnno: 2578	UniRef50_Q9SYM5	669	86.61	672	87	2	298	2313	1	669	0.0	1226
ABCDEF_17342	UniRef50_Q84M96	0.0	Normal: 274	406	272	327	439	625	BlastAnno: 1277	UniRef50_Q84M96	346	73.33	345	91	1	1187	156	2	346	0.0	546
ABCDEF_31147	UniRef50_Q9SYM5	0.0	Normal: 4571	1591	1491	1401	1939	4327	BlastAnno: 2465	UniRef50_Q9SYM5	669	82.76	673	107	4	2397	394	1	669	0.0	1154
ABCDEF_17344	UniRef50_Q84M96	0.0	Normal: 252	373	257	297	380	540	BlastAnno: 1350	UniRef50_Q84M96	346	68.19	371	91	2	1263	154	2	346	0.0	530
ABCDEF_44674	UniRef50_Q9SYM5	0.0	Normal: 3581	2620	4329	9505	11447	4930	BlastAnno: 2634	UniRef50_Q9SYM5	669	87.80	672	78	3	253	2265	1	669	0.0	1241

1.1.1.298(3-hydroxypropionate dehydrogenase (NADP+))

No Correspond Unigene

1.1.1.35(3-hydroxyacyl-CoA dehydrogenase;)

ABCDEF_80649	UniRef50_Q1LD07	5e-120	Normal: 527	841	443	1308	1321	1188	BlastAnno: 2586	UniRef50_Q1LD07	715	37.34	699	420	9	2358	277	22	707	5e-120	389

1.1.1.37(malate dehydrogenase;)

ABCDEF_25169	UniRef50_Q9SN86	0.0	Normal: 938	614	1116	1317	4446	973	BlastAnno: 1856	UniRef50_Q9SN86	403	75.50	400	88	2	1616	417	2	391	0.0	585
ABCDEF_31746	UniRef50_Q9ZP05	0.0	Normal: 611	6985	354	98	71	19818	BlastAnno: 1631	UniRef50_Q9ZP05	354	83.38	349	54	2	274	1317	9	354	0.0	578
ABCDEF_32230	UniRef50_P57106	0.0	Normal: 8408	4627	7877	10286	18779	11078	BlastAnno: 1523	UniRef50_P57106	332	93.07	332	23	0	262	1257	1	332	0.0	617
ABCDEF_57415	UniRef50_Q9ZP05	0.0	Normal: 403	336	343	761	845	611	BlastAnno: 1190	UniRef50_Q9ZP05	354	83.29	353	54	2	1058	3	1	349	0.0	579
ABCDEF_17941	UniRef50_Q9ZP05	0.0	Normal: 616	554	586	1025	1231	910	BlastAnno: 1567	UniRef50_Q9ZP05	354	83.63	342	51	2	1336	314	17	354	0.0	563

1.1.1.42(isocitrate dehydrogenase (NADP+);)

ABCDEF_49419	UniRef50_O75874	0.0	Normal: 5183	5221	5740	12435	20083	7734	BlastAnno: 1704	UniRef50_O75874	414	68.12	389	123	1	1491	328	10	398	0.0	566
ABCDEF_51976	UniRef50_O75874	0.0	Normal: 551	408	828	1291	1909	1088	BlastAnno: 1799	UniRef50_O75874	414	66.17	405	135	2	321	1529	10	414	0.0	568

1.2.1.43(formate dehydrogenase (NADP+);)

No Correspond Unigene

1.2.1.75(malonyl CoA reductase (malonate semialdehyde-forming);)

No Correspond Unigene

1.2.1.76(succinate-semialdehyde dehydrogenase (acylating);)

No Correspond Unigene

1.2.7.1(pyruvate synthase;)

No Correspond Unigene

1.2.7.3(2-oxoglutarate synthase;)

No Correspond Unigene

1.2.7.4(carbon-monoxide dehydrogenase (ferredoxin))

No Correspond Unigene

1.2.99.2(carbon-monoxide dehydrogenase (acceptor);)

No Correspond Unigene

1.3.1.84(acrylyl-CoA reductase (NADPH))

No Correspond Unigene

1.3.99.1(succinate dehydrogenase;)

ABCDEF_47748	UniRef50_Q6F8K9	0.0	Normal: 903	791	1034	2782	3604	958	BlastAnno: 2360	UniRef50_Q6F8K9	632	51.24	603	252	8	1990	290	36	632	0.0	584
ABCDEF_47751	UniRef50_Q6F8K9	0.0	Normal: 776	655	916	2143	2907	810	BlastAnno: 2266	UniRef50_Q6F8K9	632	51.24	603	252	8	303	2003	36	632	0.0	585
ABCDEF_47750	UniRef50_Q6F8K9	0.0	Normal: 1478	889	1772	2151	3099	1241	BlastAnno: 2236	UniRef50_Q6F8K9	632	51.00	602	255	7	1898	198	36	632	0.0	571

1.5.1.20(methylenetetrahydrofolate reductase [NAD(P)H];)

ABCDEF_18406	UniRef50_B9HG95	0.0	Normal: 5047	6500	5572	11857	13941	8245	BlastAnno: 2369	UniRef50_B9HG95	608	82.24	608	97	1	269	2059	1	608	0.0	1024

1.5.1.5(methylenetetrahydrofolate dehydrogenase (NADP+);)

ABCDEF_28394	UniRef50_P11586	0.0	Normal: 1686	2953	1497	2421	3359	6073	BlastAnno: 2493	UniRef50_P11586	935	68.58	627	194	3	383	2260	310	934	0.0	818

2.3.1.169(CO-methylating acetyl-CoA synthase)

No Correspond Unigene

2.3.1.8(phosphate acetyltransferase;)

ABCDEF_10009	UniRef50_B6KAY1	6e-23	Normal: 14	16	19	16	34	38	BlastAnno: 693	UniRef50_B6KAY1	1296	48.51	101	51	1	154	453	1128	1228	6e-23	104

2.3.1.9(acetyl-CoA C-acetyltransferase;)

ABCDEF_34825	UniRef50_Q8S4Y1	0.0	Normal: 640	698	620	807	839	1164	BlastAnno: 1773	UniRef50_Q8S4Y1	403	78.11	402	87	1	1597	395	2	403	0.0	598
ABCDEF_14629	UniRef50_Q8S4Y1	0.0	Normal: 634	316	650	1021	1278	691	BlastAnno: 1514	UniRef50_Q8S4Y1	403	85.11	403	60	0	1367	159	1	403	0.0	620
ABCDEF_107	UniRef50_Q8S4Y1	0.0	Normal: 332	460	460	842	893	424	BlastAnno: 1214	UniRef50_Q8S4Y1	403	84.07	339	54	0	1212	196	42	380	0.0	596
ABCDEF_14630	UniRef50_Q8S4Y1	0.0	Normal: 562	282	568	959	1173	627	BlastAnno: 1796	UniRef50_Q8S4Y1	403	85.50	400	58	0	436	1635	4	403	0.0	642

2.3.3.8(ATP citrate synthase;)

ABCDEF_877	UniRef50_Q2QNG7	0.0	Normal: 2303	1262	2637	3893	6172	3374	BlastAnno: 1806	UniRef50_Q2QNG7	423	84.73	419	64	0	1525	269	1	419	0.0	725
ABCDEF_38288	UniRef50_Q2QNG7	0.0	Normal: 923	1139	998	1780	3136	1375	BlastAnno: 1899	UniRef50_Q2QNG7	423	90.02	421	42	0	1697	435	1	421	0.0	793
ABCDEF_38287	UniRef50_Q2QNG7	0.0	Normal: 1012	1211	1083	1973	3362	1439	BlastAnno: 1842	UniRef50_Q2QNG7	423	90.02	421	42	0	146	1408	1	421	0.0	793
ABCDEF_42417	UniRef50_A8I8X8	0.0	Normal: 4805	3069	5468	10656	12827	8213	BlastAnno: 2396	UniRef50_A8I8X8	534	64.13	605	142	3	192	2003	3	533	0.0	771

2.7.2.1(acetate kinase;)

No Correspond Unigene

2.7.9.2(pyruvate, water dikinase;)

No Correspond Unigene

2.8.3.-(Transferases;)

No Correspond Unigene

3.5.4.9(methenyltetrahydrofolate cyclohydrolase;)																					
No Correspond Unigene																					
4.1.1.31(phosphoenolpyruvate carboxylase;)																					
ABCDEF_64086	UniRef50_Q6R2V6	0.0	Normal: 363	569	289	463	784	348	BlastAnno: 2123	UniRef50_Q6R2V6	1221	61.79	560	205	4	163	1821	664	1221	0.0	650
ABCDEF_41385	UniRef50_B5FPX1	0.0	Normal: 4314	701	3865	3687	4278	3624	BlastAnno: 3374	UniRef50_B5FPX1	883	40.60	941	490	14	3058	260	4	883	0.0	634
ABCDEF_62655	UniRef50_Q6R2V6	0.0	Normal: 335	542	261	432	710	315	BlastAnno: 2054	UniRef50_Q6R2V6	1221	61.79	560	205	4	163	1821	664	1221	0.0	650
ABCDEF_5143	UniRef50_Q6R2V6	0.0	Normal: 847	1445	623	1238	1720	785	BlastAnno: 3592	UniRef50_Q6R2V6	1221	57.46	637	243	6	1451	3292	590	1221	0.0	661
ABCDEF_34325	UniRef50_B5FPX1	0.0	Normal: 1019	396	1067	2133	3237	977	BlastAnno: 3428	UniRef50_B5FPX1	883	41.15	943	483	13	3011	210	4	883	0.0	656
ABCDEF_49210	UniRef50_Q6R2V6	0.0	Normal: 884	1485	676	1279	1833	822	BlastAnno: 3747	UniRef50_Q6R2V6	1221	57.46	637	243	6	2261	420	590	1221	0.0	661
ABCDEF_34323	UniRef50_B5FPX1	0.0	Normal: 1026	368	1024	2181	3307	986	BlastAnno: 3450	UniRef50_B5FPX1	883	41.15	943	483	13	440	3241	4	883	0.0	656
ABCDEF_13966	UniRef50_P15804	0.0	Normal: 86	248	71	71	214	123	BlastAnno: 2130	UniRef50_P15804	960	74.85	672	161	4	2130	133	291	960	0.0	1010
ABCDEF_13967	UniRef50_P15804	0.0	Normal: 75	236	61	55	191	97	BlastAnno: 2000	UniRef50_P15804	960	74.96	619	149	3	28	1872	344	960	0.0	925
4.1.3.24(malyl-CoA lyase;)																					
No Correspond Unigene																					
4.2.1.116(3-hydroxypropionyl-CoA dehydratase)																					
No Correspond Unigene																					
4.2.1.120(4-hydroxybutanoyl-CoA dehydratase)																					
No Correspond Unigene																					
4.2.1.2(fumarate hydratase;)																					
ABCDEF_34405	UniRef50_Q92PB6	0.0	Normal: 238	139	294	638	963	297	BlastAnno: 1885	UniRef50_Q92PB6	463	65.23	463	157	3	1703	321	2	462	0.0	605
ABCDEF_34407	UniRef50_Q92PB6	0.0	Normal: 230	132	282	615	1071	309	BlastAnno: 2193	UniRef50_Q92PB6	463	65.01	463	158	3	1860	478	2	462	0.0	603
ABCDEF_34406	UniRef50_Q92PB6	0.0	Normal: 208	123	332	593	935	301	BlastAnno: 2063	UniRef50_Q92PB6	463	65.01	463	158	3	204	1586	2	462	0.0	605
ABCDEF_34404	UniRef50_Q92PB6	0.0	Normal: 305	139	336	555	956	377	BlastAnno: 1811	UniRef50_Q92PB6	463	65.01	463	158	3	226	1608	2	462	0.0	603
4.2.1.3(aconitate hydratase;)																					
ABCDEF_73976	UniRef50_Q90875	0.0	Normal: 1872	1426	2211	5893	8147	1851	BlastAnno: 3209	UniRef50_Q90875	889	61.77	892	335	3	3045	370	3	888	0.0	1114
ABCDEF_35907	UniRef50_Q90875	0.0	Normal: 1591	876	1677	4081	5832	1425	BlastAnno: 3358	UniRef50_Q90875	889	61.84	891	334	3	3181	509	3	887	0.0	1109
ABCDEF_17819	UniRef50_Q90875	0.0	Normal: 1842	942	1937	4916	6347	1527	BlastAnno: 3866	UniRef50_Q90875	889	61.84	891	334	3	3181	509	3	887	0.0	1110
ABCDEF_52401	UniRef50_Q90875	0.0	Normal: 2227	1825	2713	7829	9659	2079	BlastAnno: 3665	UniRef50_Q90875	889	61.77	892	335	3	3045	370	3	888	0.0	1114
4.2.1.55(3-hydroxybutyryl-CoA dehydratase;)																					
ABCDEF_31573	UniRef50_C0QBE8	2e-44	Normal: 159	151	158	345	356	262	BlastAnno: 1094	UniRef50_C0QBE8	240	40.00	220	124	3	228	875	11	226	2e-44	160
5.1.99.1(methylmalonyl-CoA epimerase;)																					
No Correspond Unigene																					
5.3.3.3(vinylacetyl-CoA Delta-isomerase;)																					
No Correspond Unigene																					
5.4.99.2(methylmalonyl-CoA mutase;)																					
No Correspond Unigene																					
6.2.1.-(Ligases;)																					
ABCDEF_7378	UniRef50_Q9FFE6	0.0	Normal: 1022	1422	746	576	680	1408	BlastAnno: 2117	UniRef50_Q9FFE6	552	67.57	552	167	5	144	1778	1	547	0.0	768
ABCDEF_47513	UniRef50_Q8RU95	0.0	Normal: 411	609	570	233	749	887	BlastAnno: 2280	UniRef50_Q8RU95	598	53.15	572	246	6	119	1777	28	596	0.0	543
ABCDEF_78742	UniRef50_Q84P25	0.0	Normal: 236	207	373	576	450	306	BlastAnno: 1534	UniRef50_Q84P25	565	60.39	462	168	5	1413	49	19	472	0.0	546
ABCDEF_10753	UniRef50_Q9FFE6	0.0	Normal: 394	401	336	253	292	348	BlastAnno: 1841	UniRef50_Q9FFE6	552	51.00	551	259	5	117	1751	1	546	0.0	553
ABCDEF_4431	UniRef50_Q9FFE6	0.0	Normal: 27	358	28	98	112	914	BlastAnno: 2075	UniRef50_Q9FFE6	552	50.71	560	264	7	1774	107	1	552	0.0	551
ABCDEF_38213	UniRef50_Q84P25	0.0	Normal: 342	337	597	798	722	505	BlastAnno: 1939	UniRef50_Q84P25	565	62.34	555	194	5	1818	175	19	565	0.0	690
ABCDEF_25221	UniRef50_Q0DV32	0.0	Normal: 971	826	1003	1543	1442	1545	BlastAnno: 2009	UniRef50_Q0DV32	552	57.20	542	226	4	183	1793	9	549	0.0	613
ABCDEF_23413	UniRef50_Q84P23	0.0	Normal: 123	237	158	149	213	658	BlastAnno: 3175	UniRef50_Q84P23	562	51.74	547	243	8	3030	1435	15	555	0.0	555
ABCDEF_20486	UniRef50_Q9FFE6	0.0	Normal: 580	912	312	324	864	1565	BlastAnno: 3530	UniRef50_Q9FFE6	552	66.61	557	171	5	352	1995	1	551	0.0	781
6.2.1.1(acetate---CoA ligase;)																					
ABCDEF_65173	UniRef50_Q9VP61	0.0	Normal: 208	202	219	336	383	409	BlastAnno: 2249	UniRef50_Q9VP61	670	54.63	540	236	3	539	2131	4	543	0.0	629
ABCDEF_51889	UniRef50_Q9VP61	0.0	Normal: 268	268	315	436	574	651	BlastAnno: 2148	UniRef50_Q9VP61	670	55.64	638	273	4	2060	174	26	662	0.0	723
ABCDEF_51888	UniRef50_A8JFR9	0.0	Normal: 348	343	408	583	709	775	BlastAnno: 2704	UniRef50_A8JFR9	667	56.57	647	273	5	2090	168	20	664	0.0	712
ABCDEF_59631	UniRef50_Q9VP61	0.0	Normal: 132	127	125	187	249	287	BlastAnno: 1733	UniRef50_Q9VP61	670	55.41	518	222	3	89	1615	26	543	0.0	610
6.2.1.36(3-hydroxypropionyl-CoA synthase;)																					
No Correspond Unigene																					
6.2.1.5(succinate---CoA ligase (ADP-forming);)																					
ABCDEF_17836	UniRef50_O82662	0.0	Normal: 367	77	365	530	1198	144	BlastAnno: 1491	UniRef50_O82662	421	85.48	420	61	0	131	1390	1	420	0.0	741
ABCDEF_45671	UniRef50_O82662	0.0	Normal: 1899	1293	2339	3905	6798	2137	BlastAnno: 1668	UniRef50_O82662	421	87.38	420	53	0	164	1423	1	420	0.0	753
6.3.4.3(formate---tetrahydrofolate ligase;)																					
ABCDEF_28394	UniRef50_Q6UB35	0.0	Normal: 1686	2953	1497	2421	3359	6073	BlastAnno: 2493	UniRef50_Q6UB35	978	66.88	628	205	3	380	2260	352	977	0.0	792
6.4.1.2(acetyl-CoA carboxylase;)																					
ABCDEF_27465	UniRef50_P78820	0.0	Normal: 4589	3602	6382	22310	7761	4482	BlastAnno: 7477	UniRef50_P78820	2280	40.33	2145	1127	43	7128	919	55	2121	0.0	1462
6.4.1.3(propionyl-CoA carboxylase;)																					
ABCDEF_21606	UniRef50_Q2S388	2e-150	Normal: 386	375	365	562	565	924	BlastAnno: 2365	UniRef50_Q2S388	698	45.92	527	261	3	251	1768	152	675	2e-150	465
>00730 Thiamine metabolism																					
1.1.3.23(thiamine oxidase;)																					
No Correspond Unigene																					
1.4.3.19(glycine oxidase)																					
ABCDEF_64504	UniRef50_B1HSZ2	2e-10	Normal: 63	83	32	42	36	87	BlastAnno: 677	UniRef50_B1HSZ2	401	31.29	147	83	4	545	105	207	335	2e-10	65.9
2.5.1.2(thiamine pyridinylase;)																					
No Correspond Unigene																					
2.5.1.3(thiamine-phosphate diphosphorylase;)																					
ABCDEF_51560	UniRef50_P61422	6e-59	Normal: 274	206	237	192	274	574	BlastAnno: 1842	UniRef50_P61422	490	50.38	260	124	4	1647	868	228	482	6e-59	213
2.7.1.49(hydroxymethylpyrimidine kinase;)																					

No Correspond Unigene																					
2.7.1.50(hydroxyethylthiazole kinase;)																					
ABCDEF_25469	UniRef50_Q0BVF4	2e-38	Normal: 63	23	96	33	50	62	BlastAnno: 683	UniRef50_Q0BVF4	274	50.00	166	80	2	2	499	99	261	2e-38	142
2.7.1.89(thiamine kinase;)																					
No Correspond Unigene																					
2.7.4.15(thiamine-diphosphate kinase;)																					
No Correspond Unigene																					
2.7.4.16(thiamine-phosphate kinase;)																					
No Correspond Unigene																					
2.7.4.7(phosphomethylpyrimidine kinase;)																					
ABCDEF_51560	UniRef50_Q5LCW2	6e-61	Normal: 274	206	237	192	274	574	BlastAnno: 1842	UniRef50_Q5LCW2	277	44.44	261	142	3	1653	871	5	262	6e-61	212
2.7.6.2(thiamine diphosphokinase;)																					
ABCDEF_51594	UniRef50_Q9H3S4	3e-50	Normal: 83	23	113	172	254	105	BlastAnno: 1072	UniRef50_Q9H3S4	243	40.08	247	132	6	857	132	7	242	3e-50	176
2.8.1.7(cysteine desulfurase;)																					
ABCDEF_307	UniRef50_Q93WX6	0.0	Normal: 565	651	475	601	604	2143	BlastAnno: 1771	UniRef50_Q93WX6	463	80.58	412	80	0	271	1506	52	463	0.0	681
ABCDEF_18191	UniRef50_Q9Y697	0.0	Normal: 1423	1090	1398	1576	1783	1230	BlastAnno: 1995	UniRef50_Q9Y697	457	70.32	401	119	0	319	1521	57	457	0.0	609
3.1.3.-(Hydrolases;)																					
ABCDEF_3128	UniRef50_Q9ZU49	5e-117	Normal: 118	143	108	91	149	56	BlastAnno: 1434	UniRef50_Q9ZU49	327	57.43	303	121	2	1184	294	20	320	5e-117	355
3.5.99.2(thiaminase;)																					
No Correspond Unigene																					
3.6.1.15(nucleoside-triphosphatase;)																					
ABCDEF_40935	UniRef50_B7PNN0	0.0	Normal: 1058	787	1081	1355	1280	1214	BlastAnno: 3814	UniRef50_B7PNN0	1042	57.14	812	311	14	2815	413	249	1034	0.0	867
3.6.1.28(thiamine-triphosphatase)																					
No Correspond Unigene																					
>00740 Riboflavin metabolism																					
1.1.1.193(5-amino-6-(5-phosphoribosylamino)uracil reductase;)																					
ABCDEF_3649	UniRef50_B1QUE3	3e-58	Normal: 395	282	487	567	624	1315	BlastAnno: 1676	UniRef50_B1QUE3	360	36.41	368	203	7	1254	217	2	360	3e-58	206
1.1.1.302(2,5-diamino-6-(ribosylamino)-4(3H)-pyrimidinone 5'-phosphate reductase;)																					
No Correspond Unigene																					
1.14.18.1(monophenol monooxygenase;)																					
No Correspond Unigene																					
1.14.99.40(5,6-dimethylbenzimidazole synthase;)																					
No Correspond Unigene																					
1.5.1.29(FMN reductase;)																					
ABCDEF_45774	UniRef50_B0EI57	0.0	Normal: 24227	11521	19203	23125	37117	17927	BlastAnno: 3112	UniRef50_B0EI57	970	54.60	837	315	5	2894	390	197	970	0.0	919
1.5.1.30(flavin reductase;)																					
No Correspond Unigene																					
2.5.1.78(6,7-dimethyl-8-ribityllumazine synthase;)																					
No Correspond Unigene																					
2.5.1.9(riboflavin synthase;)																					
ABCDEF_22166	UniRef50_O80575	1e-93	Normal: 1014	696	1117	1299	2207	2695	BlastAnno: 1039	UniRef50_O80575	227	62.90	221	63	1	127	789	26	227	1e-93	287
2.7.1.161(CTP-dependent riboflavin kinase;)																					
No Correspond Unigene																					
2.7.1.26(riboflavin kinase;)																					
ABCDEF_30077	UniRef50_Q969G6	4e-41	Normal: 202	124	222	211	316	188	BlastAnno: 1349	UniRef50_Q969G6	155	51.47	136	66	0	487	80	10	145	4e-41	151
ABCDEF_30075	UniRef50_Q969G6	4e-41	Normal: 196	122	217	207	316	184	BlastAnno: 1331	UniRef50_Q969G6	155	51.47	136	66	0	487	80	10	145	4e-41	151
2.7.7.2(FAD synthetase;)																					
ABCDEF_37997	UniRef50_Q9FMW8	9e-110	Normal: 309	250	296	276	418	594	BlastAnno: 1566	UniRef50_Q9FMW8	354	60.49	286	106	4	1114	275	69	353	9e-110	339
3.1.3.-(Hydrolases;)																					
ABCDEF_3128	UniRef50_Q9ZU49	5e-117	Normal: 118	143	108	91	149	56	BlastAnno: 1434	UniRef50_Q9ZU49	327	57.43	303	121	2	1184	294	20	320	5e-117	355
3.1.3.2(acid phosphatase;)																					
ABCDEF_48781	UniRef50_Q9LXI4	0.0	Normal: 3	205	0	171	142	375	BlastAnno: 1533	UniRef50_Q9LXI4	437	61.07	429	162	2	79	1365	5	428	0.0	540
ABCDEF_31834	UniRef50_Q9LJU7	0.0	Normal: 669	384	448	538	533	426	BlastAnno: 1818	UniRef50_Q9LJU7	437	64.83	435	148	3	344	1642	6	437	0.0	602
ABCDEF_41630	UniRef50_Q949Y3	0.0	Normal: 325	632	262	126	318	1578	BlastAnno: 1864	UniRef50_Q949Y3	475	61.81	432	162	2	1475	183	22	451	0.0	563
ABCDEF_28194	UniRef50_Q949Y3	0.0	Normal: 933	1694	869	1449	1788	3440	BlastAnno: 1940	UniRef50_Q949Y3	475	80.67	450	87	0	1654	305	20	469	0.0	759
ABCDEF_41635	UniRef50_Q949Y3	0.0	Normal: 1046	543	888	1413	1679	1200	BlastAnno: 1824	UniRef50_Q949Y3	475	59.45	439	169	4	1513	200	21	451	0.0	540
ABCDEF_45098	UniRef50_Q949Y3	0.0	Normal: 349	5170	534	294	420	27111	BlastAnno: 1905	UniRef50_Q949Y3	475	56.86	459	184	6	156	1520	4	452	0.0	537
ABCDEF_27775	UniRef50_Q949Y3	0.0	Normal: 108	180	98	136	195	803	BlastAnno: 1429	UniRef50_Q949Y3	475	60.51	428	166	2	1427	147	26	451	0.0	540
3.5.1.102(2-amino-5-formylamino-6-ribosylaminopyrimidin-4(3H)-one 5'-monophosphate deformylase;)																					
No Correspond Unigene																					
3.5.4.25(GTP cyclohydrolase II;)																					
ABCDEF_3107	UniRef50_Q6MIV8	3e-72	Normal: 34	44	42	55	29	396	BlastAnno: 1153	UniRef50_Q6MIV8	376	55.29	208	93	0	630	7	3	210	3e-72	238
3.5.4.26(diaminohydroxyphosphoribosylaminopyrimidine deaminase)																					
ABCDEF_3649	UniRef50_Q55158	1e-65	Normal: 395	282	487	567	624	1315	BlastAnno: 1676	UniRef50_Q55158	368	44.33	291	153	5	1254	403	4	292	1e-65	226
3.5.4.29(GTP cyclohydrolase IIa)																					
No Correspond Unigene																					
3.5.99.1(riboflavinase)																					
No Correspond Unigene																					
3.6.1.18(FAD diphosphatase;)																					
No Correspond Unigene																					
3.6.1.9(nucleotide diphosphatase;)																					
ABCDEF_27673	UniRef50_Q9SU83	0.0	Normal: 373	418	422	639	977	783	BlastAnno: 2094	UniRef50_Q9SU83	496	70.27	481	123	2	1775	393	15	495	0.0	679

ABCDEF_83044	UniRef50_Q9SU83	0.0	Normal: 191	238	231	367	469	401	BlastAnno: 1570	UniRef50_Q9SU83	496	70.42	480	122	2	162	1541	15	494	0.0	678
4.1.99.12(3,4-dihydroxy-2-butanone-4-phosphate synthase;)																					
ABCDEF_17700	UniRef50_P47924	0.0	Normal: 1107	1082	1309	1136	1062	2745	BlastAnno: 2195	UniRef50_P47924	543	77.56	499	102	4	1800	331	46	543	0.0	797
ABCDEF_3108	UniRef50_P47924	0.0	Normal: 99	110	89	105	103	990	BlastAnno: 1376	UniRef50_P47924	543	84.30	293	46	0	117	995	224	516	0.0	526
ABCDEF_10294	UniRef50_P47924	0.0	Normal: 70	151	80	164	225	786	BlastAnno: 1859	UniRef50_P47924	543	65.27	429	146	3	261	1541	99	526	0.0	563
ABCDEF_25781	UniRef50_P47924	0.0	Normal: 103	8	52	48	387	92	BlastAnno: 1122	UniRef50_P47924	543	88.22	297	35	0	1	891	233	529	0.0	558
>00750 Vitamin B6 metabolism																					
1.1.1.107(pyridoxal 4-dehydrogenase;)																					
No Correspond Unigene																					
1.1.1.262(4-hydroxythreonine-4-phosphate dehydrogenase;)																					
No Correspond Unigene																					
1.1.1.290(4-phosphoerythronate dehydrogenase;)																					
No Correspond Unigene																					
1.1.1.65(pyridoxine 4-dehydrogenase;)																					
ABCDEF_30846	UniRef50_B9REP9	3e-120	Normal: 27	87	17	14	25	545	BlastAnno: 1362	UniRef50_B9REP9	280	62.50	288	88	4	1282	425	9	278	3e-120	361
1.1.3.12(pyridoxine 4-oxidase;)																					
No Correspond Unigene																					
1.1.99.9(pyridoxine 5-dehydrogenase;)																					
No Correspond Unigene																					
1.14.12.4(3-hydroxy-2-methylpyridinecarboxylate dioxygenase;)																					
No Correspond Unigene																					
1.14.12.5(5-pyridoxate dioxygenase;)																					
No Correspond Unigene																					
1.2.1.72(erythrose-4-phosphate dehydrogenase;)																					
No Correspond Unigene																					
1.2.3.1(aldehyde oxidase;)																					
ABCDEF_37559	UniRef50_Q7G193	0.0	Normal: 208	649	188	236	120	436	BlastAnno: 2825	UniRef50_Q7G193	1368	58.36	927	352	11	2741	3	3	909	0.0	1063
ABCDEF_51652	UniRef50_Q7G193	0.0	Normal: 97	323	130	90	67	347	BlastAnno: 1575	UniRef50_Q7G193	1368	64.15	491	172	4	5	1468	871	1360	0.0	632
1.2.3.8(pyridoxal oxidase)																					
No Correspond Unigene																					
1.4.3.5(pyridoxal 5'-phosphate synthase;)																					
ABCDEF_48388	UniRef50_B0C079	1e-68	Normal: 580	386	581	621	1473	1292	BlastAnno: 1905	UniRef50_B0C079	219	52.94	221	95	3	925	269	6	219	1e-68	231
2.6.-.-(Transferases;)																					
ABCDEF_3637	UniRef50_Q8LAD0	1e-121	Normal: 102	196	78	84	162	412	BlastAnno: 922	UniRef50_Q8LAD0	255	78.44	218	47	0	870	217	1	218	1e-121	358
2.6.1.30(pyridoxamine---pyruvate transaminase;)																					
No Correspond Unigene																					
2.6.1.31(pyridoxamine---oxaloacetate transaminase)																					
No Correspond Unigene																					
2.6.1.52(phosphoserine transaminase;)																					
ABCDEF_41302	UniRef50_Q96255	0.0	Normal: 1671	665	2009	2718	2176	624	BlastAnno: 1822	UniRef50_Q96255	430	80.05	391	73	3	1572	406	43	430	0.0	653
ABCDEF_28393	UniRef50_Q96255	0.0	Normal: 390	227	489	822	2225	158	BlastAnno: 1446	UniRef50_Q96255	430	78.83	392	79	2	5	1180	42	429	0.0	627
2.6.1.54(pyridoxamine-phosphate transaminase;)																					
No Correspond Unigene																					
2.6.99.2(pyridoxine 5'-phosphate synthase;)																					
No Correspond Unigene																					
2.7.1.35(pyridoxal kinase;)																					
ABCDEF_7535	UniRef50_Q8W1X2	0.0	Normal: 242	241	289	545	940	613	BlastAnno: 1440	UniRef50_Q8W1X2	309	82.14	308	55	0	1079	156	1	308	0.0	523
3.1.1.27(4-pyridoxolactonase)																					
No Correspond Unigene																					
3.1.3.74(pyridoxal phosphatase;)																					
No Correspond Unigene																					
3.5.1.29(2-(acetamidomethylene)succinate hydrolase;)																					
No Correspond Unigene																					
3.5.1.66(2-(hydroxymethyl)-3-(acetamidomethylene)succinate hydrolase;)																					
No Correspond Unigene																					
4.-.-.-(Lyases)																					
ABCDEF_41738	UniRef50_A1UF85	2e-114	Normal: 1096	1526	732	626	2607	1174	BlastAnno: 1351	UniRef50_A1UF85	322	67.22	299	93	2	1186	293	26	320	2e-114	347
4.1.1.51(3-hydroxy-2-methylpyridine-4,5-dicarboxylate 4-decarboxylase;)																					
No Correspond Unigene																					
4.2.3.1(threonine synthase;)																					
ABCDEF_45313	UniRef50_Q9S7B5	0.0	Normal: 1544	576	1733	1123	1215	608	BlastAnno: 1882	UniRef50_Q9S7B5	526	86.14	469	61	2	1744	344	46	512	0.0	841
>00760 Nicotinate and nicotinamide metabolism																					
1.1.1.291(2-hydroxymethylglutarate dehydrogenase;)																					
No Correspond Unigene																					
1.13.11.9(2,5-dihydroxypyridine 5,6-dioxygenase;)																					
No Correspond Unigene																					
1.14.13.10(2,6-dihydroxypyridine 3-monooxygenase;)																					
No Correspond Unigene																					
1.14.18.-(Oxidoreductases;)																					
No Correspond Unigene																					
1.14.99.-(Oxidoreductases;)																					
ABCDEF_66639	UniRef50_Q0DUI8	0.0	Normal: 646	675	723	1956	1163	1303	BlastAnno: 1867	UniRef50_Q0DUI8	578	85.02	414	61	1	167	1408	158	570	0.0	734

ABCDEF_52814	UniRef50_Q0DUI8	0.0	Normal: 1118	1292	1150	3508	1987	2103	BlastAnno: 2562	UniRef50_Q0DUI8	578	77.22	575	124	4	370	2094	3	570	0.0	909
1.17.1.5(nicotinate dehydrogenase;)																					
No Correspond Unigene																					
1.17.3.3(6-hydroxynicotinate dehydrogenase;)																					
No Correspond Unigene																					
1.2.3.1(aldehyde oxidase;)																					
ABCDEF_37559	UniRef50_Q7G193	0.0	Normal: 208	649	188	236	120	436	BlastAnno: 2825	UniRef50_Q7G193	1368	58.36	927	352	11	2741	3	3	909	0.0	1063
ABCDEF_51652	UniRef50_Q7G193	0.0	Normal: 97	323	130	90	67	347	BlastAnno: 1575	UniRef50_Q7G193	1368	64.15	491	172	4	5	1468	871	1360	0.0	632
1.3.7.1(6-hydroxynicotinate reductase;)																					
No Correspond Unigene																					
1.4.1.21(aspartate dehydrogenase;)																					
No Correspond Unigene																					
1.4.3.16(L-aspartate oxidase;)																					
ABCDEF_10434	UniRef50_Q3B5D3	4e-157	Normal: 139	306	137	263	196	849	BlastAnno: 2600	UniRef50_Q3B5D3	537	46.32	544	264	7	1890	274	14	534	4e-157	479
1.5.3.5(No Name)																					
No Correspond Unigene																					
1.5.3.6(No Name)																					
No Correspond Unigene																					
1.5.99.4(nicotine dehydrogenase;)																					
No Correspond Unigene																					
1.6.1.1(NAD(P)+ transhydrogenase (B-specific);)																					
No Correspond Unigene																					
1.6.1.2(NAD(P)+ transhydrogenase (AB-specific);)																					
No Correspond Unigene																					
2.1.1.1(nicotinamide N-methyltransferase;)																					
No Correspond Unigene																					
2.1.1.7(nicotinate N-methyltransferase;)																					
No Correspond Unigene																					
2.4.2.1(purine-nucleoside phosphorylase;)																					
No Correspond Unigene																					
2.4.2.11(nicotinate phosphoribosyltransferase;)																					
ABCDEF_24586	UniRef50_Q9VQX4	0.0	Normal: 261	545	278	379	508	1153	BlastAnno: 2301	UniRef50_Q9VQX4	555	58.07	539	219	4	1910	297	22	554	0.0	640
2.4.2.12(nicotinamide phosphoribosyltransferase;)																					
No Correspond Unigene																					
2.4.2.19(nicotinate-nucleotide diphosphorylase (carboxylating);)																					
ABCDEF_38831	UniRef50_Q11NQ1	7e-77	Normal: 303	363	282	308	531	807	BlastAnno: 1456	UniRef50_Q11NQ1	301	47.81	297	134	5	1153	281	18	299	7e-77	251
2.7.1.-(Transferases;)																					
ABCDEF_19669	UniRef50_O04059	0.0	Normal: 81	66	74	92	96	76	BlastAnno: 1254	UniRef50_O04059	594	81.65	376	40	1	3	1130	84	430	0.0	571
ABCDEF_19662	UniRef50_O04059	0.0	Normal: 103	74	68	108	100	97	BlastAnno: 1553	UniRef50_O04059	594	89.77	430	44	0	1419	130	1	430	0.0	752
ABCDEF_47654	UniRef50_Q86C65	0.0	Normal: 2310	2222	1853	2946	2297	3238	BlastAnno: 6212	UniRef50_Q86C65	2380	48.37	2030	881	32	6204	241	476	2380	0.0	1769
ABCDEF_19665	UniRef50_O04059	0.0	Normal: 100	80	57	109	104	104	BlastAnno: 1759	UniRef50_O04059	594	89.21	380	41	0	1390	251	60	439	0.0	655
ABCDEF_19655	UniRef50_O04059	0.0	Normal: 197	122	157	224	212	154	BlastAnno: 2166	UniRef50_O04059	594	86.71	572	76	0	2032	317	1	572	0.0	914
ABCDEF_19659	UniRef50_O04059	0.0	Normal: 200	133	145	233	228	183	BlastAnno: 2278	UniRef50_O04059	594	86.89	572	75	0	2032	317	1	572	0.0	916
ABCDEF_19671	UniRef50_O04059	0.0	Normal: 130	125	102	159	150	145	BlastAnno: 1767	UniRef50_O04059	594	89.77	430	44	0	202	1491	1	430	0.0	751
2.7.1.22(ribosylnicotinamide kinase;)																					
No Correspond Unigene																					
2.7.1.23(NAD+ kinase;)																					
ABCDEF_47607	UniRef50_Q9C5W3	0.0	Normal: 274	705	213	302	264	899	BlastAnno: 3271	UniRef50_Q9C5W3	985	63.53	987	296	14	198	3107	46	985	0.0	1181
ABCDEF_47609	UniRef50_Q9C5W3	0.0	Normal: 270	689	208	299	247	845	BlastAnno: 3293	UniRef50_Q9C5W3	985	54.20	738	274	14	3096	934	46	736	0.0	719
ABCDEF_27303	UniRef50_Q56YN3	0.0	Normal: 524	660	622	1018	947	1004	BlastAnno: 2249	UniRef50_Q56YN3	524	71.18	517	136	4	1959	418	18	524	0.0	700
2.7.7.1(nicotinamide-nucleotide adenylyltransferase;)																					
ABCDEF_47812	UniRef50_Q9HAN9	2e-52	Normal: 181	136	273	150	124	429	BlastAnno: 1383	UniRef50_Q9HAN9	279	40.32	248	115	2	820	176	7	254	2e-52	185
2.7.7.18(nicotinate-nucleotide adenylyltransferase;)																					
ABCDEF_47812	UniRef50_Q96T66	1e-48	Normal: 181	136	273	150	124	429	BlastAnno: 1383	UniRef50_Q96T66	252	40.97	227	120	2	814	176	7	233	1e-48	174
3.1.3.-(Hydrolases;)																					
ABCDEF_3128	UniRef50_Q9ZU49	5e-117	Normal: 118	143	108	91	149	56	BlastAnno: 1434	UniRef50_Q9ZU49	327	57.43	303	121	2	1184	294	20	320	5e-117	355
3.1.3.5(5'-nucleotidase;)																					
ABCDEF_10187	UniRef50_A9F2C9	3e-85	Normal: 140	337	178	525	497	1064	BlastAnno: 2494	UniRef50_A9F2C9	618	34.02	535	278	12	792	2276	119	618	3e-85	292
3.2.2.1(purine nucleosidase;)																					
ABCDEF_52566	UniRef50_B9SSZ5	2e-160	Normal: 417	265	311	827	1190	504	BlastAnno: 1375	UniRef50_B9SSZ5	286	72.01	318	54	1	191	1144	1	283	2e-160	464
3.2.2.14(NMN nucleosidase;)																					
No Correspond Unigene																					
3.2.2.5(NAD+ nucleosidase;)																					
No Correspond Unigene																					
3.2.2.6(NAD(P)+ nucleosidase;)																					
No Correspond Unigene																					
3.5.1.107(maleamate amidohydrolase;)																					
No Correspond Unigene																					
3.5.1.19(nicotinamidase;)																					
No Correspond Unigene																					
3.5.1.42(nicotinamide-nucleotide amidase;)																					
No Correspond Unigene																					

3.5.2.18(enamidase)																					
No Correspond Unigene																					
3.6.1.22(NAD+ diphosphatase;)																					
ABCDEF_38040	UniRef50_Q94A82	0.0	Normal: 508	612	543	670	703	978	BlastAnno: 1812	UniRef50_Q94A82	438	71.13	388	106	3	229	1383	36	420	0.0	566
3.6.1.9(nucleotide diphosphatase;)																					
ABCDEF_27673	UniRef50_Q9SU83	0.0	Normal: 373	418	422	639	977	783	BlastAnno: 2094	UniRef50_Q9SU83	496	70.27	481	123	2	1775	393	15	495	0.0	679
ABCDEF_83044	UniRef50_Q9SU83	0.0	Normal: 191	238	231	367	469	401	BlastAnno: 1570	UniRef50_Q9SU83	496	70.42	480	122	2	162	1541	15	494	0.0	678
3.7.1.-(Hydrolases;)																					
No Correspond Unigene																					
4.1.3.32(2,3-dimethylmalate lyase;)																					
No Correspond Unigene																					
4.2.1.85(dimethylmaleate hydratase;)																					
No Correspond Unigene																					
5.2.1.1(maleate isomerase)																					
No Correspond Unigene																					
5.3.3.6(methylitaconate Delta-isomerase;)																					
No Correspond Unigene																					
5.4.99.4(2-methyleneglutarate mutase;)																					
No Correspond Unigene																					
6.3.1.5(NAD+ synthase;)																					
ABCDEF_47914	UniRef50_B6KCT7	0.0	Normal: 564	429	500	832	1072	838	BlastAnno: 2524	UniRef50_B6KCT7	862	47.30	778	317	13	2371	311	33	808	0.0	679
6.3.5.1(NAD+ synthase (glutamine-hydrolysing);)																					
ABCDEF_47914	UniRef50_B9RYQ0	0.0	Normal: 564	429	500	832	1072	838	BlastAnno: 2524	UniRef50_B9RYQ0	716	71.19	708	201	3	2377	260	1	707	0.0	1043
>00770 Pantothenate and CoA biosynthesis																					
1.1.1.106(pantoate 4-dehydrogenase;)																					
No Correspond Unigene																					
1.1.1.169(2-dehydropantoate 2-reductase;)																					
No Correspond Unigene																					
1.1.1.84(dimethylmalate dehydrogenase;)																					
No Correspond Unigene																					
1.1.1.86(ketol-acid reductoisomerase;)																					
ABCDEF_45798	UniRef50_Q05758	0.0	Normal: 8016	5924	6126	3207	6323	14773	BlastAnno: 2251	UniRef50_Q05758	591	90.13	537	49	2	1881	271	59	591	0.0	980
1.2.1.33(No Name)																					
No Correspond Unigene																					
1.3.1.1(dihydrouracil dehydrogenase (NAD+);)																					
ABCDEF_27630	UniRef50_Q01P61	9e-82	Normal: 448	273	523	806	1011	577	BlastAnno: 1916	UniRef50_Q01P61	446	41.85	368	198	4	1435	338	2	355	9e-82	273
1.3.1.2(dihydropyrimidine dehydrogenase (NADP+);)																					
ABCDEF_27630	UniRef50_A9GKC4	2e-84	Normal: 448	273	523	806	1011	577	BlastAnno: 1916	UniRef50_A9GKC4	462	42.13	356	190	5	1435	374	2	343	2e-84	280
2.1.2.11(3-methyl-2-oxobutanoate hydroxymethyltransferase;)																					
ABCDEF_4166	UniRef50_Q9Y7B6	2e-78	Normal: 443	443	422	461	505	284	BlastAnno: 1420	UniRef50_Q9Y7B6	349	47.77	291	147	3	969	97	60	345	2e-78	256
2.2.1.6(acetolactate synthase;)																					
ABCDEF_41851	UniRef50_B2UM25	0.0	Normal: 2693	2810	2333	1636	1298	4686	BlastAnno: 2340	UniRef50_B2UM25	617	52.19	594	263	7	364	2088	10	601	0.0	597
ABCDEF_6813	UniRef50_B2UM25	0.0	Normal: 935	649	1027	751	1655	773	BlastAnno: 2146	UniRef50_B2UM25	617	51.17	598	270	8	1943	204	10	603	0.0	584
2.6.1.42(branched-chain-amino-acid transaminase;)																					
ABCDEF_45709	UniRef50_Q9FYA6	1e-179	Normal: 1705	822	1358	1129	1148	2398	BlastAnno: 1596	UniRef50_Q9FYA6	415	74.08	355	91	1	1261	197	61	414	1e-179	521
2.6.1.6(leucine transaminase;)																					
No Correspond Unigene																					
2.7.1.24(dephospho-CoA kinase;)																					
ABCDEF_7740	UniRef50_Q71ZA4	4e-54	Normal: 312	229	320	408	390	498	BlastAnno: 803	UniRef50_Q71ZA4	200	46.77	186	99	0	677	120	3	188	4e-54	182
2.7.1.33(pantothenate kinase;)																					
ABCDEF_44176	UniRef50_Q8L5Y9	0.0	Normal: 703	558	674	1029	743	917	BlastAnno: 3140	UniRef50_Q8L5Y9	870	85.78	865	119	3	353	2941	8	870	0.0	1414
ABCDEF_44178	UniRef50_Q8L5Y9	0.0	Normal: 805	597	731	986	875	1073	BlastAnno: 3436	UniRef50_Q8L5Y9	870	84.50	471	69	3	333	1739	8	476	0.0	765
ABCDEF_44177	UniRef50_Q8L5Y9	0.0	Normal: 749	593	773	1080	777	927	BlastAnno: 3060	UniRef50_Q8L5Y9	870	85.78	865	119	3	273	2861	8	870	0.0	1414
2.7.1.34(pantetheine kinase;)																					
No Correspond Unigene																					
2.7.7.3(pantetheine-phosphate adenylyltransferase;)																					
ABCDEF_4183	UniRef50_Q9ZPV8	3e-81	Normal: 70	107	77	215	215	228	BlastAnno: 708	UniRef50_Q9ZPV8	176	77.02	161	37	0	99	581	3	163	3e-81	249
2.7.8.7(holo-[acyl-carrier-protein] synthase;)																					
No Correspond Unigene																					
3.1.4.14(No Name)																					
No Correspond Unigene																					
3.5.1.22(pantothenase;)																					
No Correspond Unigene																					
3.5.1.6(beta-ureidopropionase)																					
ABCDEF_17031	UniRef50_Q9UBR1	1e-142	Normal: 273	642	328	641	1094	595	BlastAnno: 2413	UniRef50_Q9UBR1	384	58.49	383	153	3	2133	991	6	384	1e-142	434
3.5.1.92(pantetheine hydrolase;)																					
No Correspond Unigene																					
3.5.2.2(dihydropyrimidinase;)																					
ABCDEF_44368	UniRef50_Q9FMP3	0.0	Normal: 229	259	223	421	456	369	BlastAnno: 1773	UniRef50_Q9FMP3	531	84.83	501	76	0	1554	52	27	527	0.0	864
ABCDEF_44370	UniRef50_Q9FMP3	0.0	Normal: 159	173	177	315	341	286	BlastAnno: 1537	UniRef50_Q9FMP3	531	85.98	435	61	0	1363	59	93	527	0.0	754
3.6.1.9(nucleotide diphosphatase;)																					
ABCDEF_27673	UniRef50_Q9SU83	0.0	Normal: 373	418	422	639	977	783	BlastAnno: 2094	UniRef50_Q9SU83	496	70.27	481	123	2	1775	393	15	495	0.0	679

ABCDEF_83044	UniRef50_Q9SU83	0.0	Normal: 191	238	231	367	469	401	BlastAnno: 1570	UniRef50_Q9SU83	496	70.42	480	122	2	162	1541	15	494	0.0	678
4.1.1.11(aspartate 1-decarboxylase;)																					
ABCDEF_80024	UniRef50_Q24062	1e-13	Normal: 0	0	0	0	0	10	BlastAnno: 237	UniRef50_Q24062	575	39.73	73	44	0	18	236	482	554	1e-13	71.2
4.1.1.30(pantothenoylcysteine decarboxylase;)																					
No Correspond Unigene																					
4.1.1.36(phosphopantothenoylcysteine decarboxylase;)																					
ABCDEF_38331	UniRef50_Q9SWE5	2e-114	Normal: 1127	483	996	1144	1398	1346	BlastAnno: 1173	UniRef50_Q9SWE5	209	80.42	189	37	0	253	819	14	202	2e-114	341
4.2.1.9(dihydroxy-acid dehydratase;)																					
ABCDEF_34628	UniRef50_P39522	0.0	Normal: 1310	368	1226	708	881	847	BlastAnno: 2244	UniRef50_P39522	585	58.77	570	226	3	291	1976	16	584	0.0	642
6.3.2.1(pantoate---beta-alanine ligase;)																					
ABCDEF_17131	UniRef50_B9S544	3e-153	Normal: 493	361	542	624	685	667	BlastAnno: 2119	UniRef50_B9S544	325	67.08	322	95	2	1855	920	5	325	3e-153	456
6.3.2.5(phosphopantothenate---cysteine ligase;)																					
ABCDEF_313	UniRef50_Q7KN99	2e-66	Normal: 227	143	291	285	664	387	BlastAnno: 1463	UniRef50_Q7KN99	313	39.54	306	175	3	1140	238	7	307	2e-66	224
>00780 Biotin metabolism																					
2.3.1.47(8-amino-7-oxononanoate synthase;)																					
ABCDEF_45523	UniRef50_B9RB57	2e-93	Normal: 211	37	165	124	76	158	BlastAnno: 881	UniRef50_B9RB57	484	78.53	191	41	0	879	307	206	396	2e-93	293
2.6.1.62(adenosylmethionine---8-amino-7-oxononanoate transaminase;)																					
ABCDEF_28231	UniRef50_A2R4Y0	3e-84	Normal: 177	89	131	88	115	206	BlastAnno: 1439	UniRef50_A2R4Y0	804	38.24	455	215	11	1438	209	363	796	3e-84	284
2.8.1.6(biotin synthase)																					
ABCDEF_8062	UniRef50_P54967	0.0	Normal: 1470	760	2013	2614	2386	1907	BlastAnno: 1476	UniRef50_P54967	378	87.10	341	43	1	1175	156	37	377	0.0	607
3.4.-.-(Hydrolases;)																					
ABCDEF_21645	UniRef50_Q1PF50	0.0	Normal: 654	512	669	909	994	888	BlastAnno: 1898	UniRef50_Q1PF50	417	66.35	416	138	1	1558	317	1	416	0.0	592
3.5.1.12(biotinidase;)																					
No Correspond Unigene																					
6.2.1.11(biotin---CoA ligase;)																					
No Correspond Unigene																					
6.2.1.14(6-carboxyhexanoate---CoA ligase;)																					
ABCDEF_28096	UniRef50_C4LK80	5e-31	Normal: 175	47	154	126	94	174	BlastAnno: 957	UniRef50_C4LK80	720	42.07	164	93	2	499	8	335	496	5e-31	129
6.3.3.3(dethiobiotin synthase;)																					
No Correspond Unigene																					
6.3.4.10(biotin---[propionyl-CoA-carboxylase (ATP-hydrolysing)] ligase;)																					
ABCDEF_6377	UniRef50_Q9SL92	4e-150	Normal: 145	148	166	204	216	227	BlastAnno: 1876	UniRef50_Q9SL92	367	60.11	356	131	4	661	1722	21	367	4e-150	447
6.3.4.11(biotin---[methylcrotonoyl-CoA-carboxylase] ligase;)																					
No Correspond Unigene																					
6.3.4.15(biotin---[acetyl-CoA-carboxylase] ligase;)																					
No Correspond Unigene																					
6.3.4.9(biotin---[methylmalonyl-CoA-carboxytransferase] ligase;)																					
No Correspond Unigene																					
>00785 Lipoic acid metabolism																					
2.3.1.181(lipoyl(octanoyl) transferase;)																					
ABCDEF_52279	UniRef50_Q9SXP7	8e-122	Normal: 97	67	106	172	197	352	BlastAnno: 1065	UniRef50_Q9SXP7	235	75.77	227	55	0	902	222	1	227	8e-122	359
2.7.7.63(lipoate---protein ligase;)																					
No Correspond Unigene																					
2.8.1.8(lipoyl synthase;)																					
ABCDEF_27381	UniRef50_B9RW49	0.0	Normal: 293	155	210	210	255	722	BlastAnno: 1380	UniRef50_B9RW49	348	87.54	297	37	0	1319	429	36	332	0.0	518
ABCDEF_24256	UniRef50_B9RW49	0.0	Normal: 306	388	455	735	569	1080	BlastAnno: 1864	UniRef50_B9RW49	348	90.40	302	29	0	1599	694	31	332	0.0	548
6.-.-.-(No Name)																					
ABCDEF_30078	UniRef50_Q6L4L4	0.0	Normal: 873	690	641	788	1014	1012	BlastAnno: 3221	UniRef50_Q6L4L4	875	50.18	815	380	18	3086	669	78	875	0.0	721
ABCDEF_30143	UniRef50_Q6L4L4	0.0	Normal: 95	103	72	139	90	112	BlastAnno: 1586	UniRef50_Q6L4L4	875	61.24	485	163	9	1467	28	3	467	0.0	618
ABCDEF_30141	UniRef50_Q6L4L4	0.0	Normal: 1434	827	1232	1415	1424	1692	BlastAnno: 5949	UniRef50_Q6L4L4	875	50.00	876	376	23	5791	3221	3	835	0.0	802
>00790 Folate biosynthesis																					
1.1.1.153(sepiapterin reductase;)																					
No Correspond Unigene																					
1.1.1.220(6-pyruvoyltetrahydropterin 2'-reductase;)																					
No Correspond Unigene																					
1.5.1.3(dihydrofolate reductase;)																					
ABCDEF_13008	UniRef50_Q27828	1e-156	Normal: 295	218	353	308	311	485	BlastAnno: 2378	UniRef50_Q27828	462	47.04	506	220	6	406	1923	5	462	1e-156	473
1.5.1.34(6,7-dihydropteridine reductase;)																					
No Correspond Unigene																					
2.5.1.15(dihydropteroate synthase;)																					
ABCDEF_24831	UniRef50_B5YCT5	8e-48	Normal: 110	57	106	73	105	168	BlastAnno: 904	UniRef50_B5YCT5	400	40.95	232	122	5	3	695	175	392	8e-48	172
2.6.1.85(aminodeoxychorismate synthase;)																					
ABCDEF_30231	UniRef50_B2J340	1e-167	Normal: 271	744	402	648	716	864	BlastAnno: 3039	UniRef50_B2J340	741	38.96	847	345	20	2671	230	1	708	1e-167	519
2.7.6.3(2-amino-4-hydroxy-6-hydroxymethyldihydropteridine diphosphokinase;)																					
No Correspond Unigene																					
3.1.3.1(alkaline phosphatase;)																					
ABCDEF_87884	UniRef50_P05186	1e-14	Normal: 0	0	0	0	0	16	BlastAnno: 228	UniRef50_P05186	524	46.15	78	40	1	1	228	411	488	1e-14	73.9
3.4.19.9(gamma-glutamyl hydrolase;)																					
ABCDEF_52466	UniRef50_O65355	9e-145	Normal: 1043	1339	1007	1474	1878	2047	BlastAnno: 1344	UniRef50_O65355	347	63.27	343	120	2	1213	200	5	346	9e-145	426
3.5.4.16(GTP cyclohydrolase I;)																					
ABCDEF_31349	UniRef50_C7QLK1	3e-32	Normal: 176	166	158	172	311	320	BlastAnno: 1949	UniRef50_C7QLK1	239	41.12	197	95	7	262	849	57	233	3e-32	131
ABCDEF_37862	UniRef50_C7QLK1	3e-32	Normal: 247	258	399	373	318	520	BlastAnno: 1789	UniRef50_C7QLK1	239	40.28	211	115	5	1003	1620	34	238	3e-32	130

3.6.1.-(Hydrolases;)																					
ABCDEF_43677	UniRef50_B6ADS3	0.0	Normal: 265	202	294	363	341	233	BlastAnno: 2843	UniRef50_B6ADS3	1078	62.19	685	246	6	2017	2	246	930	0.0	842
ABCDEF_40935	UniRef50_A7ASE9	0.0	Normal: 1058	787	1081	1355	1280	1214	BlastAnno: 3814	UniRef50_A7ASE9	931	63.21	647	232	3	2359	428	285	928	0.0	811
ABCDEF_37918	UniRef50_Q8L831	0.0	Normal: 1344	592	1122	994	975	978	BlastAnno: 2888	UniRef50_Q8L831	772	71.74	768	214	1	209	2503	4	771	0.0	1152
ABCDEF_43683	UniRef50_B6ADS3	0.0	Normal: 186	143	232	259	238	201	BlastAnno: 1783	UniRef50_B6ADS3	1078	66.53	478	159	1	1	1431	600	1077	0.0	698
ABCDEF_34064	UniRef50_A7AVM7	0.0	Normal: 2140	1397	1695	2129	1483	1897	BlastAnno: 4387	UniRef50_A7AVM7	1016	49.83	891	398	12	2923	368	80	960	0.0	799
ABCDEF_43671	UniRef50_B6ADS3	0.0	Normal: 152	130	166	197	197	139	BlastAnno: 1982	UniRef50_B6ADS3	1078	65.79	643	211	5	80	1981	298	940	0.0	856
ABCDEF_43679	UniRef50_B6ADS3	0.0	Normal: 245	175	259	298	300	234	BlastAnno: 2502	UniRef50_B6ADS3	1078	65.13	780	263	5	2423	111	298	1077	0.0	1053
ABCDEF_43672	UniRef50_B6ADS3	0.0	Normal: 334	268	365	470	391	268	BlastAnno: 3191	UniRef50_B6ADS3	1078	62.19	685	246	6	1175	3190	246	930	0.0	842
4.1.2.25(dihydroneopterin aldolase;)																					
ABCDEF_41656	UniRef50_Q9SF23	5e-49	Normal: 95	68	90	107	143	194	BlastAnno: 517	UniRef50_Q9SF23	146	61.16	121	46	1	381	22	21	141	5e-49	163
4.1.3.38(aminodeoxychorismate lyase;)																					
No Correspond Unigene																					
4.2.3.12(6-pyruvoyltetrahydropterin synthase;)																					
No Correspond Unigene																					
6.3.2.12(dihydrofolate synthase;)																					
ABCDEF_1498	UniRef50_B0VF02	1e-17	Normal: 0	0	4	0	12	6	BlastAnno: 319	UniRef50_B0VF02	426	51.25	80	36	1	265	26	112	188	1e-17	83.2
6.3.2.17(tetrahydrofolate synthase;)																					
ABCDEF_37925	UniRef50_B9HT54	0.0	Normal: 597	769	434	611	646	3201	BlastAnno: 2219	UniRef50_B9HT54	587	67.31	569	142	3	2095	509	6	570	0.0	746
>00791 Atrazine degradation																					
1.14.15.-(Oxidoreductases;)																					
No Correspond Unigene																					
3.5.1.5(urease)																					
ABCDEF_31241	UniRef50_B2W555	0.0	Normal: 284	167	262	380	250	289	BlastAnno: 1705	UniRef50_B2W555	793	59.82	555	181	5	1703	45	279	793	0.0	647
3.5.1.54(allophanate hydrolase;)																					
No Correspond Unigene																					
3.5.1.84(biuret amidohydrolase)																					
No Correspond Unigene																					
3.5.2.15(cyanuric acid amidohydrolase;)																					
No Correspond Unigene																					
3.5.4.-(Hydrolases;)																					
ABCDEF_6867	UniRef50_Q9S7I0	1e-80	Normal: 1248	676	1204	1899	1979	2300	BlastAnno: 4061	UniRef50_Q9S7I0	1307	69.49	236	69	3	915	211	1074	1307	1e-80	296
3.5.99.-(Hydrolases;)																					
No Correspond Unigene																					
3.5.99.3(hydroxydechloroatrazine ethylaminohydrolase;)																					
No Correspond Unigene																					
3.5.99.4(N-isopropylammelide isopropylaminohydrolase;)																					
No Correspond Unigene																					
3.8.1.-(Hydrolases;)																					
No Correspond Unigene																					
3.8.1.8(atrazine chlorohydrolase;)																					
No Correspond Unigene																					
4.2.1.69(cyanamide hydratase;)																					
No Correspond Unigene																					
>00830 Retinol metabolism																					
1.1.-.-(Oxidoreductases;)																					
ABCDEF_17646	UniRef50_O34948	5e-83	Normal: 372	339	320	294	487	1376	BlastAnno: 1685	UniRef50_O34948	288	47.31	279	145	2	1523	690	5	282	5e-83	269
1.1.1.-(Oxidoreductases;)																					
ABCDEF_13453	UniRef50_Q9SYM5	0.0	Normal: 225	226	398	1950	2026	421	BlastAnno: 2578	UniRef50_Q9SYM5	669	86.61	672	87	2	298	2313	1	669	0.0	1226
ABCDEF_17342	UniRef50_Q84M96	0.0	Normal: 274	406	272	327	439	625	BlastAnno: 1277	UniRef50_Q84M96	346	73.33	345	91	1	1187	156	2	346	0.0	546
ABCDEF_31147	UniRef50_Q9SYM5	0.0	Normal: 4571	1591	1491	1401	1939	4327	BlastAnno: 2465	UniRef50_Q9SYM5	669	82.76	673	107	4	2397	394	1	669	0.0	1154
ABCDEF_17344	UniRef50_Q84M96	0.0	Normal: 252	373	257	297	380	540	BlastAnno: 1350	UniRef50_Q84M96	346	68.19	371	91	2	1263	154	2	346	0.0	530
ABCDEF_44674	UniRef50_Q9SYM5	0.0	Normal: 3581	2620	4329	9505	11447	4930	BlastAnno: 2634	UniRef50_Q9SYM5	669	87.80	672	78	3	253	2265	1	669	0.0	1241
1.1.1.1(alcohol dehydrogenase;)																					
ABCDEF_31928	UniRef50_B9RHN6	0.0	Normal: 721	714	784	1329	1570	1819	BlastAnno: 1711	UniRef50_B9RHN6	437	77.55	392	87	1	360	1535	46	436	0.0	610
ABCDEF_10335	UniRef50_Q8LEB2	0.0	Normal: 285	158	514	810	352	183	BlastAnno: 1547	UniRef50_Q8LEB2	381	74.93	379	92	1	1317	181	5	380	0.0	598
1.14.14.1(unspecific monooxygenase;)																					
ABCDEF_41955	UniRef50_B9S0F8	2e-174	Normal: 70	140	28	0	0	150	BlastAnno: 1118	UniRef50_B9S0F8	506	66.67	366	115	5	2	1099	138	496	2e-174	504
1.14.15.3(alkane 1-monooxygenase;)																					
No Correspond Unigene																					
1.14.99.36(beta-carotene 15,15'-monooxygenase;)																					
ABCDEF_14018	UniRef50_Q3MBG7	3e-105	Normal: 277	1535	152	65	458	955	BlastAnno: 2090	UniRef50_Q3MBG7	765	38.36	511	252	14	370	1839	292	760	3e-105	346
1.2.1.36(retinal dehydrogenase;)																					
No Correspond Unigene																					
1.2.3.11(retinal oxidase;)																					
No Correspond Unigene																					
1.3.99.23(all-trans-retinol 13,14-reductase;)																					
No Correspond Unigene																					
2.3.1.135(phosphatidylcholine---retinol O-acyltransferase;)																					
No Correspond Unigene																					
2.3.1.76(retinol O-fatty-acyltransferase;)																					
No Correspond Unigene																					

2.4.1.17(glucuronosyltransferase;)

No Correspond Unigene

3.1.1.-(Hydrolases;)

ABCDEF_25737	UniRef50_Q9CA68	0.0	Normal: 104	11	8	393	426	18	BlastAnno: 1232	UniRef50_Q9CA68	366	69.89	352	103	2	1182	130	17	366	0.0	522
ABCDEF_525	UniRef50_Q9LIN2	0.0	Normal: 213	16	167	378	456	158	BlastAnno: 1254	UniRef50_Q9LIN2	380	69.27	358	110	0	1097	24	23	380	0.0	535
ABCDEF_28091	UniRef50_O23522	0.0	Normal: 1192	678	424	81	156	3170	BlastAnno: 1887	UniRef50_O23522	601	57.90	525	191	12	168	1721	3	504	0.0	568
ABCDEF_13432	UniRef50_Q3ECP6	0.0	Normal: 712	1725	466	588	811	6988	BlastAnno: 2479	UniRef50_Q3ECP6	408	66.83	401	104	2	2397	1282	8	408	0.0	567
ABCDEF_72624	UniRef50_Q3ECP6	0.0	Normal: 22	38	25	267	382	149	BlastAnno: 1342	UniRef50_Q3ECP6	408	66.83	401	104	2	53	1168	8	408	0.0	567
ABCDEF_4432	UniRef50_B9IQA0	0.0	Normal: 680	298	798	834	1447	644	BlastAnno: 1533	UniRef50_B9IQA0	354	78.82	321	68	0	214	1176	33	353	0.0	556

3.1.1.21(retinyl-palmitate esterase;)

No Correspond Unigene

3.1.1.63(11-cis-retinyl-palmitate hydrolase;)

No Correspond Unigene

3.1.1.64(all-trans-retinyl-palmitate hydrolase)

No Correspond Unigene

5.2.1.3(retinal isomerase;)

No Correspond Unigene

5.2.1.7(retinol isomerase;)

No Correspond Unigene

>00860 Porphyrin and chlorophyll metabolism

1.-.-.-(Oxidoreductases)

ABCDEF_345	UniRef50_Q6Z690	0.0	Normal: 442	146	449	340	272	314	BlastAnno: 1658	UniRef50_Q6Z690	849	63.42	462	150	5	1657	314	387	843	0.0	589
ABCDEF_24068	UniRef50_Q9FI41	0.0	Normal: 214	316	207	270	263	849	BlastAnno: 1742	UniRef50_Q9FI41	387	73.28	363	96	1	1440	355	4	366	0.0	554
ABCDEF_24070	UniRef50_Q9FI41	0.0	Normal: 189	290	182	231	232	751	BlastAnno: 1479	UniRef50_Q9FI41	387	73.28	363	96	1	1363	278	4	366	0.0	551
ABCDEF_13957	UniRef50_Q9CAE3	0.0	Normal: 399	318	392	489	456	586	BlastAnno: 2488	UniRef50_Q9CAE3	789	80.85	496	93	2	3	1487	285	779	0.0	768
ABCDEF_28410	UniRef50_Q9LIG0	0.0	Normal: 211	366	234	286	433	1504	BlastAnno: 1324	UniRef50_Q9LIG0	330	74.92	331	76	2	1147	173	1	330	0.0	521
ABCDEF_17581	UniRef50_Q8LGI2	0.0	Normal: 758	336	735	426	374	852	BlastAnno: 1543	UniRef50_Q8LGI2	454	68.78	442	136	2	71	1390	8	449	0.0	597
ABCDEF_23753	UniRef50_Q94A68	0.0	Normal: 284	559	215	224	164	519	BlastAnno: 1580	UniRef50_Q94A68	377	81.90	348	63	0	1349	306	30	377	0.0	576
ABCDEF_24071	UniRef50_Q9FI41	0.0	Normal: 235	377	230	278	289	948	BlastAnno: 2090	UniRef50_Q9FI41	387	73.28	363	96	1	1726	641	4	366	0.0	553

1.1.1.294(chlorophyll(ide) b reductase;)

ABCDEF_31907	UniRef50_Q93ZA0	0.0	Normal: 1371	1567	1225	2573	2435	4258	BlastAnno: 2090	UniRef50_Q93ZA0	496	75.00	420	105	0	1578	319	66	485	0.0	663

1.13.12.14(chlorophyllide-a oxygenase;)

No Correspond Unigene

1.14.12.20(pheophorbide a oxygenase;)

ABCDEF_44846	UniRef50_Q9FYC2	0.0	Normal: 358	707	410	718	705	1086	BlastAnno: 2219	UniRef50_Q9FYC2	537	73.69	498	123	4	1832	345	46	537	0.0	793

1.14.13.81(magnesium-protoporphyrin IX monomethyl ester (oxidative) cyclase;)

ABCDEF_16959	UniRef50_Q5EFU4	0.0	Normal: 852	2498	1175	652	479	15583	BlastAnno: 2685	UniRef50_Q5EFU4	417	78.74	414	77	4	82	1290	4	417	0.0	669
ABCDEF_16965	UniRef50_Q5EFU4	0.0	Normal: 537	1389	854	530	480	5092	BlastAnno: 2057	UniRef50_Q5EFU4	417	89.55	287	30	0	2056	1196	131	417	0.0	554
ABCDEF_16963	UniRef50_Q5EFU4	0.0	Normal: 516	1388	824	496	397	5093	BlastAnno: 2133	UniRef50_Q5EFU4	417	89.55	287	30	0	2132	1272	131	417	0.0	554
ABCDEF_16960	UniRef50_Q5EFU4	0.0	Normal: 857	2500	1023	539	497	15581	BlastAnno: 2607	UniRef50_Q5EFU4	417	78.74	414	77	4	2519	1311	4	417	0.0	669
ABCDEF_65358	UniRef50_Q5EFU4	0.0	Normal: 807	2380	870	474	431	9163	BlastAnno: 2567	UniRef50_Q5EFU4	417	82.26	389	67	2	5	1165	29	417	0.0	666

1.14.13.83(precorrin-3B synthase;)

No Correspond Unigene

1.14.99.3(heme oxygenase;)

ABCDEF_8106	UniRef50_O48782	5e-126	Normal: 853	1156	815	873	895	2801	BlastAnno: 1383	UniRef50_O48782	282	69.57	276	72	3	242	1033	7	282	5e-126	376

1.16.1.2(diferric-transferrin reductase;)

No Correspond Unigene

1.16.1.3(aquacobalamin reductase;)

No Correspond Unigene

1.16.1.4(cob(II)alamin reductase;)

No Correspond Unigene

1.16.1.5(aquacobalamin reductase (NADPH);)

No Correspond Unigene

1.16.1.6(cyanocobalamin reductase (cyanide-eliminating);)

No Correspond Unigene

1.16.3.1(ferroxidase;)

ABCDEF_49166	UniRef50_Q9S756	9e-110	Normal: 1136	782	532	446	716	3470	BlastAnno: 1115	UniRef50_Q9S756	259	72.54	244	63	3	1021	299	17	259	9e-110	330

1.16.8.1(cob(II)yrinic acid a,c-diamide reductase;)

No Correspond Unigene

1.18.-.-(Oxidoreductases;)

ABCDEF_14948	UniRef50_Q0I794	3e-51	Normal: 365	971	336	208	276	1833	BlastAnno: 848	UniRef50_Q0I794	140	68.47	111	35	0	544	212	28	138	3e-51	172

1.2.1.70(glutamyl-tRNA reductase)

ABCDEF_21092	UniRef50_P42804	0.0	Normal: 1124	2741	469	406	544	11829	BlastAnno: 2202	UniRef50_P42804	543	86.27	466	62	1	503	1900	80	543	0.0	767
ABCDEF_24562	UniRef50_P42804	0.0	Normal: 298	373	402	519	707	1960	BlastAnno: 2320	UniRef50_P42804	543	85.41	466	66	1	497	1894	80	543	0.0	749

1.3.1.24(biliverdin reductase)

No Correspond Unigene

1.3.1.33(protochlorophyllide reductase;)

ABCDEF_38417	UniRef50_Q42536	0.0	Normal: 1497	8066	316	27	66	45852	BlastAnno: 1568	UniRef50_Q42536	405	78.57	392	77	3	1456	302	14	405	0.0	607
ABCDEF_18586	UniRef50_Q42536	0.0	Normal: 1049	1993	667	418	850	11039	BlastAnno: 1642	UniRef50_Q42536	405	76.05	405	85	4	134	1312	1	405	0.0	581
ABCDEF_15994	UniRef50_Q42536	0.0	Normal: 835	1571	568	345	737	8292	BlastAnno: 1537	UniRef50_Q42536	405	78.76	372	74	3	108	1208	34	405	0.0	566

1.3.1.54(precorrin-6A reductase;)

No Correspond Unigene

1.3.1.75(divinyl chlorophyllide a 8-vinyl-reductase;)																				
No Correspond Unigene																				
1.3.1.76(precorrin-2 dehydrogenase;)																				
No Correspond Unigene																				
1.3.1.80(red chlorophyll catabolite reductase;)																				
ABCDEF_31142	UniRef50_Q8LDU4	2e-100	Normal: 806	840	1049	2270	1864	1473	BlastAnno: 2024	UniRef50_Q8LDU4	319	56.25	288	121	2	1860	1006	31	316	2e-100	319
1.3.3.3(coproporphyrinogen oxidase;)																				
ABCDEF_2436	UniRef50_Q9LR75	0.0	Normal: 110	241	67	49	84	1194	BlastAnno: 2924	UniRef50_Q9LR75	386	78.63	365	67	3	355	1422	24	386	0.0	593
ABCDEF_2440	UniRef50_Q9LR75	0.0	Normal: 135	253	92	65	116	1198	BlastAnno: 2951	UniRef50_Q9LR75	386	78.63	365	67	3	355	1422	24	386	0.0	593
ABCDEF_2442	UniRef50_Q9LR75	0.0	Normal: 141	216	68	61	120	958	BlastAnno: 2570	UniRef50_Q9LR75	386	78.63	365	67	3	2216	1149	24	386	0.0	593
ABCDEF_2435	UniRef50_Q9LR75	0.0	Normal: 118	235	70	52	92	1112	BlastAnno: 2805	UniRef50_Q9LR75	386	78.63	365	67	3	355	1422	24	386	0.0	593
ABCDEF_2444	UniRef50_Q9LR75	0.0	Normal: 82	199	44	45	61	874	BlastAnno: 2193	UniRef50_Q9LR75	386	78.63	365	67	3	1839	772	24	386	0.0	593
ABCDEF_2437	UniRef50_Q9LR75	0.0	Normal: 66	207	40	41	47	964	BlastAnno: 2312	UniRef50_Q9LR75	386	78.63	365	67	3	1958	891	24	386	0.0	593
ABCDEF_2441	UniRef50_Q9LR75	0.0	Normal: 195	261	111	79	167	1216	BlastAnno: 3328	UniRef50_Q9LR75	386	78.63	365	67	3	355	1422	24	386	0.0	593
ABCDEF_2434	UniRef50_Q9LR75	0.0	Normal: 144	240	85	76	106	1105	BlastAnno: 2601	UniRef50_Q9LR75	386	78.63	365	67	3	2247	1180	24	386	0.0	593
ABCDEF_2430	UniRef50_Q9LR75	0.0	Normal: 374	458	431	533	633	1879	BlastAnno: 1365	UniRef50_Q9LR75	386	83.97	343	47	3	1031	3	51	385	0.0	591
ABCDEF_2439	UniRef50_Q9LR75	0.0	Normal: 323	298	151	114	281	1442	BlastAnno: 4122	UniRef50_Q9LR75	386	78.63	365	67	3	355	1422	24	386	0.0	593
ABCDEF_2425	UniRef50_Q9LR75	0.0	Normal: 134	211	71	74	106	880	BlastAnno: 2246	UniRef50_Q9LR75	386	78.63	365	67	3	1941	874	24	386	0.0	593
ABCDEF_2426	UniRef50_Q9LR75	0.0	Normal: 84	206	52	60	51	884	BlastAnno: 2077	UniRef50_Q9LR75	386	78.63	365	67	3	1772	705	24	386	0.0	593
ABCDEF_2432	UniRef50_Q9LR75	0.0	Normal: 135	190	194	205	266	853	BlastAnno: 841	UniRef50_Q9LR75	386	88.89	279	26	2	839	3	112	385	0.0	520
ABCDEF_2438	UniRef50_Q9LR75	0.0	Normal: 328	298	134	127	296	1442	BlastAnno: 4003	UniRef50_Q9LR75	386	78.63	365	67	3	3649	2582	24	386	0.0	593
ABCDEF_2433	UniRef50_Q9LR75	0.0	Normal: 372	313	157	142	353	1294	BlastAnno: 3826	UniRef50_Q9LR75	386	78.63	365	67	3	3472	2405	24	386	0.0	593
ABCDEF_2443	UniRef50_Q9LR75	0.0	Normal: 237	271	126	98	222	1270	BlastAnno: 3485	UniRef50_Q9LR75	386	78.63	365	67	3	355	1422	24	386	0.0	593
1.3.3.4(protoporphyrinogen oxidase;)																				
ABCDEF_47868	UniRef50_O24164	0.0	Normal: 575	529	561	431	518	1829	BlastAnno: 2043	UniRef50_O24164	504	76.26	476	108	4	1932	514	30	503	0.0	753
ABCDEF_14353	UniRef50_P55826	0.0	Normal: 597	834	445	292	295	3392	BlastAnno: 2100	UniRef50_P55826	537	73.75	518	128	3	1824	292	21	537	0.0	755
1.3.3.5(bilirubin oxidase;)																				
No Correspond Unigene																				
1.3.7.2(15,16-dihydrobiliverdin:ferredoxin oxidoreductase;)																				
No Correspond Unigene																				
1.3.7.3(phycoerythrobilin:ferredoxin oxidoreductase;)																				
ABCDEF_34562	UniRef50_Q10UW6	6e-38	Normal: 213	201	246	167	154	530	BlastAnno: 1625	UniRef50_Q10UW6	256	33.19	238	151	3	1008	313	11	246	6e-38	146
1.3.7.4(phytochromobilin:ferredoxin oxidoreductase;)																				
ABCDEF_34562	UniRef50_Q9SR43	8e-114	Normal: 213	201	246	167	154	530	BlastAnno: 1625	UniRef50_Q9SR43	329	56.84	285	122	1	1023	169	46	329	8e-114	349
1.3.7.5(phycocyanobilin:ferredoxin oxidoreductase)																				
No Correspond Unigene																				
1.3.99.22(coproporphyrinogen dehydrogenase;)																				
No Correspond Unigene																				
2.1.1.-(Transferases;)																				
ABCDEF_20806	UniRef50_Q94II3	0.0	Normal: 700	332	876	1458	1997	929	BlastAnno: 2275	UniRef50_Q94II3	600	73.57	594	153	4	2119	341	1	591	0.0	907
ABCDEF_45802	UniRef50_Q9ZPH9	0.0	Normal: 3543	3348	4296	9797	10830	5017	BlastAnno: 2642	UniRef50_Q9ZPH9	633	53.90	603	261	7	2216	456	23	624	0.0	676
ABCDEF_20070	UniRef50_Q944R7	0.0	Normal: 301	141	265	207	185	187	BlastAnno: 2675	UniRef50_Q944R7	724	58.28	719	275	9	127	2265	13	712	0.0	840
ABCDEF_34956	UniRef50_Q94II3	0.0	Normal: 1120	7269	2475	16033	22219	21461	BlastAnno: 2676	UniRef50_Q94II3	600	73.77	610	146	4	404	2230	1	597	0.0	957
ABCDEF_17121	UniRef50_Q9ZPH9	0.0	Normal: 124	77	109	284	199	132	BlastAnno: 2066	UniRef50_Q9ZPH9	633	53.24	601	259	8	161	1903	25	623	0.0	644
ABCDEF_6524	UniRef50_Q9ZPH9	0.0	Normal: 471	296	490	753	870	730	BlastAnno: 2602	UniRef50_Q9ZPH9	633	50.89	619	290	5	2221	404	6	623	0.0	659
ABCDEF_56017	UniRef50_Q9ZPH9	0.0	Normal: 302	167	286	596	748	529	BlastAnno: 2384	UniRef50_Q9ZPH9	633	51.37	619	287	5	213	2030	6	623	0.0	663
ABCDEF_44169	UniRef50_Q9SIZ3	0.0	Normal: 179	158	273	705	685	229	BlastAnno: 2300	UniRef50_Q9SIZ3	589	62.01	308	108	4	1219	296	285	583	0.0	394
ABCDEF_6521	UniRef50_Q9ZPH9	0.0	Normal: 339	198	314	641	767	582	BlastAnno: 2511	UniRef50_Q9ZPH9	633	51.37	619	287	5	2172	355	6	623	0.0	663
ABCDEF_44172	UniRef50_Q9SIZ3	0.0	Normal: 204	184	287	767	741	242	BlastAnno: 2317	UniRef50_Q9SIZ3	589	68.15	449	134	4	1637	291	144	583	0.0	641
ABCDEF_199	UniRef50_Q9SU94	0.0	Normal: 1076	275	1009	558	1247	306	BlastAnno: 1769	UniRef50_Q9SU94	390	78.45	348	74	1	284	1324	43	390	0.0	584
ABCDEF_52252	UniRef50_Q84W92	0.0	Normal: 1658	720	1653	1980	2289	1601	BlastAnno: 2170	UniRef50_Q84W92	535	72.49	469	120	3	409	1791	34	501	0.0	703
ABCDEF_51404	UniRef50_A3BMN9	0.0	Normal: 864	188	716	337	631	326	BlastAnno: 2192	UniRef50_A3BMN9	620	50.50	604	243	9	2008	251	55	620	0.0	555
ABCDEF_21386	UniRef50_Q94AI4	0.0	Normal: 2326	1250	2220	4294	2920	1983	BlastAnno: 3701	UniRef50_Q94AI4	775	47.27	842	332	16	567	2978	8	775	0.0	577
ABCDEF_35101	UniRef50_Q9MAT5	0.0	Normal: 1440	420	1268	576	1136	850	BlastAnno: 1634	UniRef50_Q9MAT5	383	69.03	352	109	0	1449	394	32	383	0.0	550
ABCDEF_960	UniRef50_Q9LN50	0.0	Normal: 3504	13198	3609	11156	19651	12155	BlastAnno: 2626	UniRef50_Q9LN50	724	49.14	525	246	5	883	2451	219	724	0.0	570
ABCDEF_48082	UniRef50_Q9LN50	0.0	Normal: 410	445	467	870	993	454	BlastAnno: 2340	UniRef50_Q9LN50	724	70.25	521	155	0	710	2272	204	724	0.0	821
ABCDEF_10473	UniRef50_Q08A71	0.0	Normal: 374	187	332	425	456	314	BlastAnno: 1764	UniRef50_Q08A71	435	79.23	366	75	1	344	1438	70	435	0.0	575
ABCDEF_7295	UniRef50_Q9ZPH9	0.0	Normal: 25	54	30	81	26	329	BlastAnno: 1191	UniRef50_Q9ZPH9	633	69.23	403	110	4	1191	22	225	626	0.0	599
ABCDEF_4160	UniRef50_Q94II3	0.0	Normal: 189	0	210	71	17	72	BlastAnno: 1424	UniRef50_Q94II3	600	77.28	449	101	1	1424	81	143	591	0.0	758
ABCDEF_44857	UniRef50_Q9ZPH9	0.0	Normal: 101	224	103	299	588	350	BlastAnno: 1582	UniRef50_Q9ZPH9	633	72.06	519	143	1	1571	21	114	632	0.0	822
ABCDEF_71541	UniRef50_Q9SIZ3	0.0	Normal: 187	167	296	765	724	237	BlastAnno: 2268	UniRef50_Q9SIZ3	589	62.01	308	108	4	1210	287	285	583	0.0	394
ABCDEF_44171	UniRef50_Q9SIZ3	0.0	Normal: 214	196	312	827	802	251	BlastAnno: 2339	UniRef50_Q9SIZ3	589	64.78	548	182	5	1978	335	47	583	0.0	717
ABCDEF_20805	UniRef50_Q94II3	0.0	Normal: 674	344	885	1417	2194	963	BlastAnno: 2400	UniRef50_Q94II3	600	78.21	436	94	1	879	2183	156	591	0.0	734
ABCDEF_18210	UniRef50_Q9SIZ3	0.0	Normal: 5584	3714	7379	17006	21892	7546	BlastAnno: 3199	UniRef50_Q9SIZ3	589	50.00	576	269	7	2145	424	27	585	0.0	574
ABCDEF_6526	UniRef50_Q9ZPH9	0.0	Normal: 627	185	837	2717	2815	1530	BlastAnno: 2611	UniRef50_Q9ZPH9	633	54.76	599	259	5	462	2222	25	623	0.0	689
ABCDEF_6525	UniRef50_Q9ZPH9	0.0	Normal: 602	165	760	2369	2605	1400	BlastAnno: 2545	UniRef50_Q9ZPH9	633	54.92	599	258	5	431	2191	25	623	0.0	689
ABCDEF_6523	UniRef50_Q9ZPH9	0.0	Normal: 314	175	304	641	784	552	BlastAnno: 2312	UniRef50_Q9ZPH9	633	52.42	599	272	4	201	1958	25	623	0.0	663
ABCDEF_67393	UniRef50_Q9ZPH9	0.0	Normal: 434	245	444	608	768	655	BlastAnno: 2346	UniRef50_Q9ZPH9	633	51.92	599	275	4	232	1989	25	623	0.0	659
ABCDEF_3579	UniRef50_O14744	0.0	Normal: 1361	326	984	568	791	440	BlastAnno: 2369	UniRef50_O14744	637	47.00	634	294	10	267	2120	30	637	0.0	553
2.1.1.107(uroporphyrinogen-III C-methyltransferase;)																				
ABCDEF_37650	UniRef50_Q42606	1e-171	Normal: 267	206	381	472	608	438	BlastAnno: 1770	UniRef50_Q42606	369	73.32	371	94	4	260	1363	1	369	1e-171	501
2.1.1.11(magnesium protoporphyrin IX methyltransferase)																				

ABCDEF_18348	UniRef50_Q55467	7e-82	Normal: 1040	2323	635	598	785	10576	BlastAnno: 1119	UniRef50_Q55467	230	58.26	230	87	2	1084	395	8	228	7e-82	258
2.1.1.130(precorrin-2 C20-methyltransferase)																					
No Correspond Unigene																					
2.1.1.131(precorrin-3B C17-methyltransferase;)																					
No Correspond Unigene																					
2.1.1.132(precorrin-6Y C5,15-methyltransferase (decarboxylating);)																					
No Correspond Unigene																					
2.1.1.133(precorrin-4 C11-methyltransferase;)																					
No Correspond Unigene																					
2.1.1.151(cobalt-factor II C20-methyltransferase;)																					
No Correspond Unigene																					
2.1.1.152(precorrin-6A synthase (deacetylating);)																					
No Correspond Unigene																					
2.3.1.37(5-aminolevulinate synthase;)																					
No Correspond Unigene																					
2.4.1.17(glucuronosyltransferase;)																					
No Correspond Unigene																					
2.4.2.21(nicotinate-nucleotide---dimethylbenzimidazole phosphoribosyltransferase;)																					
No Correspond Unigene																					
2.5.1.-(Transferases;)																					
ABCDEF_48175	UniRef50_P72580	2e-123	Normal: 366	927	282	531	590	1811	BlastAnno: 1917	UniRef50_P72580	323	58.44	320	130	2	610	1569	6	322	2e-123	377
2.5.1.17(cob(I)yrinic acid a,c-diamide adenosyltransferase;)																					
No Correspond Unigene																					
2.5.1.61(hydroxymethylbilane synthase;)																					
ABCDEF_32160	UniRef50_Q43316	5e-171	Normal: 2183	2972	2484	2796	2784	19430	BlastAnno: 1515	UniRef50_Q43316	382	79.88	333	64	1	1298	300	53	382	5e-171	496
2.5.1.62(chlorophyll synthase)																					
ABCDEF_14926	UniRef50_Q38833	0.0	Normal: 962	1056	881	944	767	4240	BlastAnno: 1776	UniRef50_Q38833	387	78.34	374	64	4	160	1233	1	373	0.0	566
2.6.1.43(aminolevulinate transaminase;)																					
No Correspond Unigene																					
2.7.1.156(adenosylcobinamide kinase;)																					
No Correspond Unigene																					
2.7.7.62(adenosylcobinamide-phosphate guanylyltransferase;)																					
No Correspond Unigene																					
2.7.8.26(adenosylcobinamide-GDP ribazoletransferase;)																					
No Correspond Unigene																					
3.1.1.14(chlorophyllase;)																					
ABCDEF_41882	UniRef50_Q9M7I7	4e-123	Normal: 51	208	25	38	35	607	BlastAnno: 1306	UniRef50_Q9M7I7	318	59.67	305	111	5	129	1007	8	312	4e-123	369
3.1.1.82(pheophorbidase;)																					
ABCDEF_4509	UniRef50_O23512	3e-66	Normal: 405	579	502	1444	2358	952	BlastAnno: 1191	UniRef50_O23512	262	44.09	254	130	5	1011	262	12	257	3e-66	219
3.1.3.73(alpha-ribazole phosphatase;)																					
No Correspond Unigene																					
3.2.1.31(beta-glucuronidase;)																					
No Correspond Unigene																					
3.5.1.90(adenosylcobinamide hydrolase;)																					
No Correspond Unigene																					
4.-.-.-(Lyases)																					
ABCDEF_41738	UniRef50_A1UF85	2e-114	Normal: 1096	1526	732	626	2607	1174	BlastAnno: 1351	UniRef50_A1UF85	322	67.22	299	93	2	1186	293	26	320	2e-114	347
4.1.1.37(uroporphyrinogen decarboxylase;)																					
ABCDEF_1046	UniRef50_Q93ZB6	0.0	Normal: 1116	1189	971	1621	1773	7702	BlastAnno: 1453	UniRef50_Q93ZB6	418	75.63	398	87	3	160	1332	17	411	0.0	615
ABCDEF_4010	UniRef50_O22886	0.0	Normal: 1163	2808	930	804	934	16260	BlastAnno: 1610	UniRef50_O22886	394	83.10	361	60	1	1363	284	34	394	0.0	627
4.1.1.81(threonine-phosphate decarboxylase;)																					
No Correspond Unigene																					
4.2.1.-(Lyases;)																					
ABCDEF_48679	UniRef50_Q0RP29	5e-115	Normal: 568	325	563	941	1285	676	BlastAnno: 1424	UniRef50_Q0RP29	493	56.39	321	129	4	119	1075	39	350	5e-115	356
4.2.1.24(porphobilinogen synthase;)																					
ABCDEF_32088	UniRef50_Q5Z8V9	0.0	Normal: 1946	3328	1545	1614	1632	12405	BlastAnno: 1596	UniRef50_Q5Z8V9	426	88.33	317	37	0	1121	171	108	424	0.0	590
4.2.1.75(uroporphyrinogen-III synthase;)																					
ABCDEF_24084	UniRef50_O48721	5e-104	Normal: 298	161	195	96	165	619	BlastAnno: 1298	UniRef50_O48721	321	67.35	245	80	0	276	1010	65	309	5e-104	320
4.4.1.17(holocytochrome-c synthase;)																					
No Correspond Unigene																					
4.99.1.1(ferrochelatase;)																					
ABCDEF_731	UniRef50_P42043	0.0	Normal: 844	1184	761	992	1367	2187	BlastAnno: 2286	UniRef50_P42043	466	72.65	340	93	0	1800	781	84	423	0.0	537
ABCDEF_37993	UniRef50_P42043	0.0	Normal: 151	370	172	527	711	828	BlastAnno: 1708	UniRef50_P42043	466	73.98	392	99	2	384	1550	68	459	0.0	578
ABCDEF_37992	UniRef50_P42043	0.0	Normal: 210	517	257	733	1014	1070	BlastAnno: 1901	UniRef50_P42043	466	75.45	391	93	2	1340	177	69	459	0.0	595
4.99.1.3(sirohydrochlorin cobaltochelatase;)																					
ABCDEF_36964	UniRef50_B5YL78	2e-18	Normal: 36	30	26	20	27	108	BlastAnno: 850	UniRef50_B5YL78	125	36.80	125	74	2	683	318	2	124	2e-18	85.5
ABCDEF_36969	UniRef50_B5YL78	2e-18	Normal: 33	28	32	26	41	80	BlastAnno: 847	UniRef50_B5YL78	125	36.80	125	74	2	296	661	2	124	2e-18	85.5
ABCDEF_57531	UniRef50_B5YL78	2e-18	Normal: 35	27	23	18	16	84	BlastAnno: 835	UniRef50_B5YL78	125	36.80	125	74	2	641	276	2	124	2e-18	85.5
4.99.1.4(sirohydrochlorin ferrochelatase;)																					
ABCDEF_36970	UniRef50_P61817	2e-11	Normal: 12	10	8	4	8	24	BlastAnno: 526	UniRef50_P61817	266	31.25	96	64	1	95	382	53	146	2e-11	65.9
5.4.1.2(precorrin-8X methylmutase;)																					
No Correspond Unigene																					

5.4.3.8(glutamate-1-semialdehyde 2,1-aminomutase;)																					
ABCDEF_4514	UniRef50_P42799	0.0	Normal: 1760	1689	1708	2005	1922	11781	BlastAnno: 1778	UniRef50_P42799	474	84.79	434	66	0	1489	188	40	473	0.0	744
6.1.1.17(glutamate---tRNA ligase;)																					
ABCDEF_42065	UniRef50_Q9FEA2	0.0	Normal: 817	816	851	1388	1686	4658	BlastAnno: 2125	UniRef50_Q9FEA2	570	74.53	589	128	6	2121	358	2	569	0.0	915
ABCDEF_34003	UniRef50_O13775	0.0	Normal: 994	462	1011	964	1034	1372	BlastAnno: 2650	UniRef50_O13775	716	47.03	640	320	7	330	2240	84	707	0.0	595
ABCDEF_66061	UniRef50_P28668	0.0	Normal: 539	247	497	528	630	425	BlastAnno: 2402	UniRef50_P28668	1714	43.56	691	353	10	331	2340	56	730	0.0	584
ABCDEF_34004	UniRef50_P28668	0.0	Normal: 871	382	854	826	1146	475	BlastAnno: 2652	UniRef50_P28668	1714	43.04	704	364	10	341	2389	56	743	0.0	583
6.3.1.-(Ligases;)																					
No Correspond Unigene																					
6.3.1.10(adenosylcobinamide-phosphate synthase;)																					
No Correspond Unigene																					
6.3.5.10(adenosylcobyric acid synthase (glutamine-hydrolysing);)																					
No Correspond Unigene																					
6.3.5.9(hydrogenobyrinic acid a,c-diamide synthase (glutamine-hydrolysing);)																					
No Correspond Unigene																					
6.6.1.1(magnesium chelatase;)																					
ABCDEF_24630	UniRef50_O50314	0.0	Normal: 6603	29214	2306	489	438	67412	BlastAnno: 4506	UniRef50_O50314	1272	32.07	1347	784	38	313	4212	6	1268	0.0	705
ABCDEF_18527	UniRef50_P16127	0.0	Normal: 1530	4061	1133	1456	1994	19958	BlastAnno: 1614	UniRef50_P16127	424	77.05	427	94	2	1456	179	1	424	0.0	586
ABCDEF_35124	UniRef50_Q9SJE1	0.0	Normal: 1142	2028	954	1415	1208	8536	BlastAnno: 2676	UniRef50_Q9SJE1	760	78.79	363	69	1	2435	1371	36	398	0.0	583
6.6.1.2(cobaltochelatase;)																					
ABCDEF_24630	UniRef50_B8GCY7	0.0	Normal: 6603	29214	2306	489	438	67412	BlastAnno: 4506	UniRef50_B8GCY7	1269	34.96	1270	734	33	490	4212	62	1268	0.0	717
>00900 Terpenoid backbone biosynthesis																					
1.1.1.267(1-deoxy-D-xylulose-5-phosphate reductoisomerase;)																					
ABCDEF_52377	UniRef50_Q9XFS9	0.0	Normal: 926	1803	991	2235	2299	5311	BlastAnno: 1995	UniRef50_Q9XFS9	477	82.28	474	77	3	1737	334	2	474	0.0	781
1.1.1.34(hydroxymethylglutaryl-CoA reductase (NADPH);)																					
ABCDEF_25368	UniRef50_P14891	0.0	Normal: 1644	938	1408	4004	4555	1661	BlastAnno: 1833	UniRef50_P14891	592	69.42	605	139	5	65	1807	1	583	0.0	796
ABCDEF_10954	UniRef50_P14891	0.0	Normal: 13477	608	12495	15402	12563	1354	BlastAnno: 2430	UniRef50_P14891	592	70.42	612	145	7	2273	456	1	582	0.0	818
ABCDEF_35656	UniRef50_P14891	0.0	Normal: 36	683	10	25	43	206	BlastAnno: 1425	UniRef50_P14891	592	72.15	474	104	3	1424	3	104	549	0.0	669
ABCDEF_22574	UniRef50_P14891	0.0	Normal: 81	4	25	0	0	36	BlastAnno: 1075	UniRef50_P14891	592	83.04	342	58	0	48	1073	185	526	0.0	588
ABCDEF_14443	UniRef50_P14891	0.0	Normal: 81	115	41	20	256	356	BlastAnno: 1784	UniRef50_P14891	592	71.40	500	114	4	1782	286	111	582	0.0	693
1.1.1.88(hydroxymethylglutaryl-CoA reductase;)																					
No Correspond Unigene																					
1.17.1.2(4-hydroxy-3-methylbut-2-enyl diphosphate reductase;)																					
ABCDEF_6599	UniRef50_B0C4N8	0.0	Normal: 892	928	1006	2423	2035	1227	BlastAnno: 1821	UniRef50_B0C4N8	415	60.39	409	159	2	1492	275	1	409	0.0	530
ABCDEF_6600	UniRef50_B0C4N8	0.0	Normal: 583	951	722	1646	1274	2408	BlastAnno: 1768	UniRef50_B0C4N8	415	60.99	405	155	3	1546	341	1	405	0.0	527
ABCDEF_6597	UniRef50_B0C4N8	0.0	Normal: 717	776	783	1715	1640	1185	BlastAnno: 1824	UniRef50_B0C4N8	415	60.64	409	158	2	1566	349	1	409	0.0	531
ABCDEF_6601	UniRef50_B0C4N8	0.0	Normal: 984	1089	1157	2805	2238	1259	BlastAnno: 1799	UniRef50_B0C4N8	415	60.39	409	159	3	1556	339	1	409	0.0	531
1.17.7.1(No Name)																					
ABCDEF_7067	UniRef50_Q6MD85	0.0	Normal: 642	879	760	1226	1229	1636	BlastAnno: 2683	UniRef50_Q6MD85	654	52.08	674	291	10	2178	178	3	651	0.0	665
ABCDEF_7066	UniRef50_Q6MD85	0.0	Normal: 621	896	678	1287	1340	1637	BlastAnno: 2813	UniRef50_Q6MD85	654	52.08	674	291	10	636	2636	3	651	0.0	664
ABCDEF_7065	UniRef50_Q6MD85	0.0	Normal: 579	822	642	1191	1184	1523	BlastAnno: 2636	UniRef50_Q6MD85	654	52.08	674	291	10	459	2459	3	651	0.0	665
1.3.1.83(geranylgeranyl diphosphate reductase;)																					
ABCDEF_24705	UniRef50_Q9CA67	0.0	Normal: 3500	4587	3746	8400	3382	16286	BlastAnno: 1891	UniRef50_Q9CA67	467	80.69	466	86	3	184	1575	4	467	0.0	746
2.2.1.7(1-deoxy-D-xylulose-5-phosphate synthase;)																					
ABCDEF_55526	UniRef50_Q0C154	0.0	Normal: 1288	2189	708	1624	1104	6968	BlastAnno: 2055	UniRef50_Q0C154	640	54.36	631	241	7	2032	140	45	628	0.0	640
ABCDEF_20989	UniRef50_Q39RT4	0.0	Normal: 1897	3673	1203	2622	1772	11108	BlastAnno: 3024	UniRef50_Q39RT4	635	49.61	649	304	10	768	2699	4	634	0.0	572
ABCDEF_62323	UniRef50_C9KK37	0.0	Normal: 46	911	173	439	384	522	BlastAnno: 2400	UniRef50_C9KK37	629	49.77	639	290	11	2113	221	3	618	0.0	588
ABCDEF_17105	UniRef50_C9KK37	0.0	Normal: 30	730	143	403	319	394	BlastAnno: 2176	UniRef50_C9KK37	629	51.09	595	269	9	288	2057	3	580	0.0	568
2.3.1.9(acetyl-CoA C-acetyltransferase;)																					
ABCDEF_34825	UniRef50_Q8S4Y1	0.0	Normal: 640	698	620	807	839	1164	BlastAnno: 1773	UniRef50_Q8S4Y1	403	78.11	402	87	1	1597	395	2	403	0.0	598
ABCDEF_14629	UniRef50_Q8S4Y1	0.0	Normal: 634	316	650	1021	1278	691	BlastAnno: 1514	UniRef50_Q8S4Y1	403	85.11	403	60	0	1367	159	1	403	0.0	620
ABCDEF_107	UniRef50_Q8S4Y1	0.0	Normal: 332	460	460	842	893	424	BlastAnno: 1214	UniRef50_Q8S4Y1	403	84.07	339	54	0	1212	196	42	380	0.0	596
ABCDEF_14630	UniRef50_Q8S4Y1	0.0	Normal: 562	282	568	959	1173	627	BlastAnno: 1796	UniRef50_Q8S4Y1	403	85.50	400	58	0	436	1635	4	403	0.0	642
2.3.3.10(hydroxymethylglutaryl-CoA synthase;)																					
ABCDEF_52282	UniRef50_A4GKI1	0.0	Normal: 1662	333	1405	2243	3244	1685	BlastAnno: 1805	UniRef50_A4GKI1	463	57.49	454	189	2	1606	251	2	453	0.0	536
ABCDEF_4202	UniRef50_A4GKI1	0.0	Normal: 1002	1241	821	1323	1992	1786	BlastAnno: 1998	UniRef50_A4GKI1	463	57.74	452	185	3	1636	290	2	450	0.0	531
2.5.1.-(Transferases;)																					
ABCDEF_48175	UniRef50_P72580	2e-123	Normal: 366	927	282	531	590	1811	BlastAnno: 1917	UniRef50_P72580	323	58.44	320	130	2	610	1569	6	322	2e-123	377
2.5.1.1(dimethylallyltranstransferase;)																					
ABCDEF_75976	UniRef50_O04046	5e-136	Normal: 59	59	70	98	780	133	BlastAnno: 1334	UniRef50_O04046	376	66.33	294	95	2	345	1220	83	374	5e-136	404
2.5.1.10(No Name)																					
ABCDEF_7885	UniRef50_Q09152	0.0	Normal: 492	301	542	1067	2969	981	BlastAnno: 1316	UniRef50_Q09152	384	76.44	348	82	0	62	1105	37	384	0.0	566
ABCDEF_56213	UniRef50_Q09152	0.0	Normal: 484	278	496	987	2687	896	BlastAnno: 1308	UniRef50_Q09152	384	76.44	348	82	0	56	1099	37	384	0.0	566
ABCDEF_63994	UniRef50_Q09152	0.0	Normal: 642	280	422	841	2240	714	BlastAnno: 1298	UniRef50_Q09152	384	76.44	348	82	0	62	1105	37	384	0.0	566
ABCDEF_83164	UniRef50_Q09152	0.0	Normal: 404	252	478	936	2606	841	BlastAnno: 1301	UniRef50_Q09152	384	77.71	341	76	0	69	1091	44	384	0.0	564
2.5.1.20(rubber cis-polyprenylcistransferase;)																					
No Correspond Unigene																					
2.5.1.29(farnesyltranstransferase;)																					
ABCDEF_35530	UniRef50_B9T2V1	4e-45	Normal: 81	39	93	279	195	246	BlastAnno: 801	UniRef50_B9T2V1	308	45.52	268	113	8	776	45	47	305	4e-45	161
2.5.1.30(heptaprenyl diphosphate synthase;)																					
No Correspond Unigene																					
2.5.1.31(ditrans,polycis-undecaprenyl-diphosphate synthase [(2E,6E)-farnesyl-diphosphate specific];)																					

ABCDEF_34051 UniRef50_B9S6U3 9e-71 Normal: 26 12 19 21 17 22 BlastAnno: 644 UniRef50_B9S6U3 381 63.27 196 72 0 47 634 1 196 9e-71 228

2.5.1.68(No Name)

No Correspond Unigene

2.5.1.82(hexaprenyl diphosphate synthase [geranylgeranyl-diphosphate specific];)

No Correspond Unigene

2.5.1.83(hexaprenyl-diphosphate synthase [(2E,6E)-farnesyl-diphosphate specific];)

No Correspond Unigene

2.5.1.84(all-trans-nonaprenyl-diphosphate synthase [geranyl-diphosphate specific];)

No Correspond Unigene

2.5.1.85(all-trans-nonaprenyl diphosphate synthase [geranylgeranyl-diphosphate specific];)

ABCDEF_24254 UniRef50_Q76FS5 0.0 Normal: 392 1036 292 487 410 1538 BlastAnno: 1568 UniRef50_Q76FS5 417 72.19 392 102 3 382 1539 26 416 0.0 548

ABCDEF_48175 UniRef50_Q76FS5 0.0 Normal: 366 927 282 531 590 1811 BlastAnno: 1917 UniRef50_Q76FS5 417 72.31 372 99 2 460 1572 49 417 0.0 547

2.5.1.86(trans,polycis-decaprenyl diphosphate synthase;)

No Correspond Unigene

2.7.1.148(4-(cytidine 5'-diphospho)-2-C-methyl-D-erythritol kinase;)

ABCDEF_35406 UniRef50_O81014 1e-151 Normal: 515 516 559 741 798 1585 BlastAnno: 1443 UniRef50_O81014 383 74.48 337 86 0 336 1346 41 377 1e-151 446

2.7.1.36(mevalonate kinase;)

ABCDEF_27375 UniRef50_P46086 5e-162 Normal: 251 265 272 438 716 369 BlastAnno: 1711 UniRef50_P46086 378 66.49 385 120 5 1418 270 1 378 5e-162 476

2.7.4.2(phosphomevalonate kinase;)

No Correspond Unigene

2.7.7.60(2-C-methyl-D-erythritol 4-phosphate cytidylyltransferase;)

ABCDEF_41638 UniRef50_P69834 1e-117 Normal: 404 632 388 398 555 1016 BlastAnno: 1614 UniRef50_P69834 302 62.01 308 105 3 423 1340 2 299 1e-117 358

4.1.1.33(diphosphomevalonate decarboxylase;)

ABCDEF_37042 UniRef50_P53602 6e-112 Normal: 171 147 260 473 765 345 BlastAnno: 1637 UniRef50_P53602 400 49.29 420 189 8 130 1386 1 397 6e-112 347

4.2.3.27(isoprene synthase;)

No Correspond Unigene

4.6.1.12(2-C-methyl-D-erythritol 2,4-cyclodiphosphate synthase;)

ABCDEF_3957 UniRef50_Q9CAK8 1e-104 Normal: 318 477 346 473 482 1726 BlastAnno: 972 UniRef50_Q9CAK8 231 87.50 168 21 0 648 145 64 231 1e-104 314

5.3.3.2(isopentenyl-diphosphate Delta-isomerase;)

ABCDEF_42087 UniRef50_Q42553 8e-145 Normal: 680 587 690 903 1450 1773 BlastAnno: 1264 UniRef50_Q42553 284 83.19 238 39 1 257 970 48 284 8e-145 422

>00901 Indole alkaloid biosynthesis

1.1.1.273(vellosimine dehydrogenase)

No Correspond Unigene

1.14.11.20(deacetoxyvindoline 4-hydroxylase;)

No Correspond Unigene

1.14.13.73(tabersonine 16-hydroxylase;)

ABCDEF_7521 UniRef50_P98183 6e-163 Normal: 511 689 239 291 500 1769 BlastAnno: 1700 UniRef50_P98183 495 56.45 473 196 4 244 1650 26 492 6e-163 483

1.14.13.75(vinorine hydroxylase)

No Correspond Unigene

1.3.1.36(geissoschizine dehydrogenase)

No Correspond Unigene

1.3.1.73(1,2-dihydrovomilenine reductase)

No Correspond Unigene

1.5.1.32(vomilenine reductase)

No Correspond Unigene

2.1.1.94(tabersonine 16-O-methyltransferase;)

No Correspond Unigene

2.1.1.99(3-hydroxy-16-methoxy-2,3-dihydrotabersonine N-methyltransferase;)

No Correspond Unigene

2.3.1.107(deacetylvindoline O-acetyltransferase;)

No Correspond Unigene

2.3.1.160(vinorine synthase)

ABCDEF_8475 UniRef50_Q70PR7 3e-71 Normal: 112 14 35 0 0 653 BlastAnno: 1543 UniRef50_Q70PR7 421 39.86 429 216 12 1417 179 1 403 3e-71 241

2.4.1.219(vomilenine glucosyltransferase;)

No Correspond Unigene

3.1.1.78(polyneuridine-aldehyde esterase;)

ABCDEF_32045 UniRef50_Q9SE93 7e-111 Normal: 68 189 17 5 0 164 BlastAnno: 853 UniRef50_Q9SE93 264 58.33 252 105 0 88 843 10 261 7e-111 330

3.2.1.105(3alpha(S)-strictosidine beta-glucosidase)

No Correspond Unigene

3.2.1.125(raucaffricine beta-glucosidase;)

ABCDEF_41889 UniRef50_Q9SPP9 0.0 Normal: 0 68 1 11 33 921 BlastAnno: 1933 UniRef50_Q9SPP9 540 61.06 511 183 5 1812 289 16 513 0.0 612

4.1.1.28(aromatic-L-amino-acid decarboxylase;)

ABCDEF_28052 UniRef50_P54770 3e-145 Normal: 150 74 133 88 177 332 BlastAnno: 1237 UniRef50_P54770 533 55.61 374 141 2 1237 191 144 517 3e-145 433

4.3.3.2(strictosidine synthase;)

ABCDEF_17966 UniRef50_P94111 9e-50 Normal: 21 112 174 190 61 4216 BlastAnno: 1196 UniRef50_P94111 335 38.01 292 174 5 147 1016 38 324 9e-50 178

>00902 Monoterpenoid biosynthesis

1.1.1.207(No Name)

No Correspond Unigene

1.1.1.208(No Name)

ABCDEF_41901 UniRef50_B2X050 5e-122 Normal: 13 138 8 11 325 355 BlastAnno: 1159 UniRef50_B2X050 314 56.33 316 133 3 194 1135 2 314 5e-122 364

1.1.1.223(isopiperitenol dehydrogenase)

No Correspond Unigene

1.1.1.241(6-endo-hydroxycineole dehydrogenase)																					
No Correspond Unigene																					
1.1.1.243(carveol dehydrogenase;)																					
No Correspond Unigene																					
1.1.1.275(No Name)																					
No Correspond Unigene																					
1.14.13.47(No Name)																					
No Correspond Unigene																					
1.14.13.48(No Name)																					
ABCDEF_22561	UniRef50_Q6WKZ1	9e-128	Normal: 0	528	0	0	0	67	BlastAnno: 1856	UniRef50_Q6WKZ1	496	47.22	449	224	6	318	1664	56	491	9e-128	394
1.14.13.49(No Name)																					
No Correspond Unigene																					
1.14.13.51(6-oxocineole dehydrogenase;)																					
No Correspond Unigene																					
1.14.13.74(7-deoxyloganin 7-hydroxylase)																					
No Correspond Unigene																					
1.14.13.80(No Name)																					
No Correspond Unigene																					
1.3.1.82(No Name)																					
No Correspond Unigene																					
1.3.3.9(secologanin synthase)																					
ABCDEF_24989	UniRef50_Q05047	0.0	Normal: 632	1234	956	2042	2096	0	BlastAnno: 1929	UniRef50_Q05047	524	48.85	524	263	3	1921	359	1	522	0.0	548
ABCDEF_33620	UniRef50_Q05047	0.0	Normal: 191	477	188	337	398	331	BlastAnno: 1829	UniRef50_Q05047	524	52.48	524	241	5	93	1655	4	522	0.0	569
ABCDEF_45573	UniRef50_Q05047	0.0	Normal: 19	539	12	27	279	185	BlastAnno: 1850	UniRef50_Q05047	524	52.31	520	243	3	1797	244	6	522	0.0	561
ABCDEF_77074	UniRef50_Q05047	0.0	Normal: 149	338	139	264	325	248	BlastAnno: 1821	UniRef50_Q05047	524	52.48	524	241	5	39	1601	4	522	0.0	574
ABCDEF_17490	UniRef50_Q05047	0.0	Normal: 219	297	257	159	646	116	BlastAnno: 1937	UniRef50_Q05047	524	51.64	517	242	5	140	1678	12	524	0.0	562
2.1.1.50(loganate O-methyltransferase;)																					
No Correspond Unigene																					
3.1.7.3(monoterpenyl-diphosphatase;)																					
No Correspond Unigene																					
4.2.3.-(Lyases;)																					
ABCDEF_30616	UniRef50_Q0JA83	0.0	Normal: 514	201	395	484	549	320	BlastAnno: 3023	UniRef50_Q0JA83	739	47.55	736	339	12	660	2852	33	726	0.0	609
4.2.3.10(No Name)																					
No Correspond Unigene																					
4.2.3.11(sabinene-hydrate synthase;)																					
No Correspond Unigene																					
4.2.3.14(pinene synthase;)																					
No Correspond Unigene																					
4.2.3.15(myrcene synthase)																					
ABCDEF_64133	UniRef50_Q93X23	0.0	Normal: 13	674	12	9	22	9	BlastAnno: 1958	UniRef50_Q93X23	597	52.87	541	246	4	111	1715	57	594	0.0	563
ABCDEF_20253	UniRef50_Q93X23	0.0	Normal: 27	666	27	87	40	47	BlastAnno: 1962	UniRef50_Q93X23	597	51.64	550	250	9	1746	97	59	592	0.0	540
4.2.3.16(No Name)																					
ABCDEF_64133	UniRef50_A7IZZ1	4e-164	Normal: 13	674	12	9	22	9	BlastAnno: 1958	UniRef50_A7IZZ1	622	46.99	549	280	5	102	1715	74	622	4e-164	493
4.2.3.20(No Name)																					
ABCDEF_64133	UniRef50_Q8L5K3	5e-169	Normal: 13	674	12	9	22	9	BlastAnno: 1958	UniRef50_Q8L5K3	606	48.99	543	267	4	108	1715	52	591	5e-169	505
4.2.3.26(R-linalool synthase;)																					
ABCDEF_62661	UniRef50_Q9SPN1	6e-99	Normal: 9	379	6	5	13	0	BlastAnno: 1229	UniRef50_Q9SPN1	583	49.02	357	178	2	111	1169	45	401	6e-99	315
5.5.1.8(bornyl diphosphate synthase;)																					
ABCDEF_20253	UniRef50_O81192	8e-156	Normal: 27	666	27	87	40	47	BlastAnno: 1962	UniRef50_O81192	598	45.81	561	295	5	1782	100	45	596	8e-156	471
>00903 Limonene and pinene degradation																					
1.1.-.-(Oxidoreductases;)																					
ABCDEF_17646	UniRef50_O34948	5e-83	Normal: 372	339	320	294	487	1376	BlastAnno: 1685	UniRef50_O34948	288	47.31	279	145	2	1523	690	5	282	5e-83	269
1.1.1.144(perillyl-alcohol dehydrogenase;)																					
No Correspond Unigene																					
1.1.1.243(carveol dehydrogenase;)																					
No Correspond Unigene																					
1.1.1.275(No Name)																					
No Correspond Unigene																					
1.1.99.-(Oxidoreductases;)																					
ABCDEF_20521	UniRef50_Q8N465	0.0	Normal: 406	283	302	461	568	486	BlastAnno: 2144	UniRef50_Q8N465	521	58.16	478	194	5	354	1784	47	519	0.0	564
1.14.-.-(Oxidoreductases;)																					
ABCDEF_44739	UniRef50_O64989	0.0	Normal: 157	87	159	718	673	189	BlastAnno: 2185	UniRef50_O64989	513	70.75	482	112	3	1867	509	30	511	0.0	706
ABCDEF_31491	UniRef50_Q93VK5	0.0	Normal: 647	1826	671	1517	1770	5953	BlastAnno: 2278	UniRef50_Q93VK5	595	79.14	556	113	2	198	1862	35	588	0.0	902
ABCDEF_40554	UniRef50_P37122	0.0	Normal: 1574	1962	1829	1150	2882	2721	BlastAnno: 1828	UniRef50_P37122	505	60.52	504	198	1	1752	244	1	504	0.0	638
ABCDEF_34049	UniRef50_O23365	0.0	Normal: 363	384	349	442	549	1416	BlastAnno: 2101	UniRef50_O23365	580	76.17	579	116	5	230	1957	19	578	0.0	904
ABCDEF_21579	UniRef50_O48928	0.0	Normal: 1005	643	1606	4372	3226	1621	BlastAnno: 1895	UniRef50_O48928	513	69.72	502	151	1	185	1690	7	507	0.0	731
ABCDEF_37741	UniRef50_P37122	0.0	Normal: 12	760	9	24	34	576	BlastAnno: 1197	UniRef50_P37122	505	65.70	379	129	1	1195	59	127	504	0.0	526
ABCDEF_35618	UniRef50_O48928	0.0	Normal: 28	2648	17	142	1234	902	BlastAnno: 1746	UniRef50_O48928	513	66.40	506	168	2	1649	132	4	507	0.0	701
ABCDEF_24522	UniRef50_P37122	0.0	Normal: 334	2190	561	268	1391	2459	BlastAnno: 2075	UniRef50_P37122	505	61.52	460	176	1	1544	165	47	505	0.0	612
ABCDEF_14592	UniRef50_O23066	0.0	Normal: 1514	402	2245	7837	4742	1248	BlastAnno: 2378	UniRef50_O23066	553	72.71	546	140	4	2111	489	1	542	0.0	821
ABCDEF_14512	UniRef50_Q9FG65	0.0	Normal: 261	872	212	291	175	789	BlastAnno: 1878	UniRef50_Q9FG65	502	56.89	501	197	9	1738	266	11	502	0.0	532
ABCDEF_44487	UniRef50_Q940V4	0.0	Normal: 3	41	53	501	262	542	BlastAnno: 1712	UniRef50_Q940V4	465	69.37	382	116	1	38	1180	14	395	0.0	572

ABCDEF_17723	UniRef50_P37122	0.0	Normal: 240	1698	219	768	592	794	BlastAnno: 1757	UniRef50_P37122	505	63.40	459	166	2	228	1601	47	504	0.0	617
ABCDEF_37901	UniRef50_Q42569	0.0	Normal: 30	149	86	255	195	255	BlastAnno: 1246	UniRef50_Q42569	472	78.27	359	73	2	64	1140	43	396	0.0	567
ABCDEF_7260	UniRef50_O81117	0.0	Normal: 314	1085	466	3248	2575	1360	BlastAnno: 1768	UniRef50_O81117	514	59.92	474	188	1	145	1566	43	514	0.0	615
ABCDEF_40548	UniRef50_P37122	0.0	Normal: 2756	2467	2752	1072	3120	1735	BlastAnno: 1947	UniRef50_P37122	505	59.72	504	202	1	241	1749	1	504	0.0	636
ABCDEF_44485	UniRef50_Q940V4	0.0	Normal: 3	55	63	574	338	660	BlastAnno: 1607	UniRef50_Q940V4	465	68.58	452	141	1	42	1394	14	465	0.0	673
ABCDEF_40552	UniRef50_P37122	0.0	Normal: 2397	1870	2304	1249	10428	2291	BlastAnno: 1812	UniRef50_P37122	505	60.12	504	200	1	108	1616	1	504	0.0	640
ABCDEF_37902	UniRef50_Q42569	0.0	Normal: 84	524	254	725	670	1049	BlastAnno: 1812	UniRef50_Q42569	472	77.47	435	92	3	1675	371	43	471	0.0	681
ABCDEF_40556	UniRef50_P37122	0.0	Normal: 1907	2365	2163	1319	3678	3308	BlastAnno: 1944	UniRef50_P37122	505	61.04	498	193	1	264	1754	1	498	0.0	635
ABCDEF_40549	UniRef50_P37122	0.0	Normal: 1574	1962	1826	1150	2882	2723	BlastAnno: 1845	UniRef50_P37122	505	60.52	504	198	1	1754	246	1	504	0.0	638
ABCDEF_20301	UniRef50_O81117	0.0	Normal: 89	308	123	176	389	249	BlastAnno: 1637	UniRef50_O81117	514	64.04	470	164	3	1498	98	47	514	0.0	586
ABCDEF_3882	UniRef50_Q42569	0.0	Normal: 84	243	257	786	1561	182	BlastAnno: 1854	UniRef50_Q42569	472	72.51	422	107	1	1852	587	59	471	0.0	615
ABCDEF_20703	UniRef50_O49858	0.0	Normal: 115	636	20	0	0	597	BlastAnno: 1815	UniRef50_O49858	527	50.10	523	248	6	48	1607	12	524	0.0	533
ABCDEF_18295	UniRef50_O22203	0.0	Normal: 3667	1934	4977	10491	4588	1917	BlastAnno: 2006	UniRef50_O22203	508	74.85	493	123	1	214	1692	17	508	0.0	803
ABCDEF_48724	UniRef50_O23066	0.0	Normal: 104	11518	163	2083	3464	6356	BlastAnno: 2162	UniRef50_O23066	553	72.96	540	143	3	227	1840	1	539	0.0	807
ABCDEF_37738	UniRef50_P37122	0.0	Normal: 26	2447	19	30	33	993	BlastAnno: 1837	UniRef50_P37122	505	64.30	479	169	2	1685	249	28	504	0.0	639

1.14.13.-(Oxidoreductases;)

ABCDEF_45538	UniRef50_O23024	3e-156	Normal: 110	6	165	774	133	36	BlastAnno: 1258	UniRef50_O23024	437	58.18	385	151	3	3	1157	37	411	3e-156	457

1.14.13.48(No Name)

ABCDEF_22561	UniRef50_Q6WKZ1	9e-128	Normal: 0	528	0	0	0	67	BlastAnno: 1856	UniRef50_Q6WKZ1	496	47.22	449	224	6	318	1664	56	491	9e-128	394

1.14.13.49(No Name)

No Correspond Unigene

1.14.13.80(No Name)

No Correspond Unigene

1.2.-.-(Oxidoreductases;)

No Correspond Unigene

1.2.1.-(Oxidoreductases;)

ABCDEF_14573	UniRef50_B5ZBH4	2e-108	Normal: 2547	9518	1266	309	441	22896	BlastAnno: 1872	UniRef50_B5ZBH4	475	42.52	461	259	4	228	1598	16	474	2e-108	343

1.2.1.3(aldehyde dehydrogenase (NAD+);)

ABCDEF_52483	UniRef50_Q70E96	0.0	Normal: 28	851	41	0	3965	420	BlastAnno: 1750	UniRef50_Q70E96	484	54.56	482	219	0	107	1552	3	484	0.0	548
ABCDEF_17638	UniRef50_Q70E96	0.0	Normal: 580	718	749	2424	1317	775	BlastAnno: 1970	UniRef50_Q70E96	484	62.21	471	177	1	283	1692	2	472	0.0	625
ABCDEF_403	UniRef50_Q8W033	0.0	Normal: 69	355	98	39	223	1111	BlastAnno: 1749	UniRef50_Q8W033	550	65.96	473	161	0	1523	105	74	546	0.0	625
ABCDEF_45143	UniRef50_Q0WSF1	0.0	Normal: 1445	874	1200	1789	1940	1504	BlastAnno: 2450	UniRef50_Q0WSF1	596	79.97	594	115	2	2220	451	1	594	0.0	1015

1.3.-.-(Oxidoreductases;)

ABCDEF_37215	UniRef50_Q9VSM5	6e-76	Normal: 470	387	380	308	475	745	BlastAnno: 2204	UniRef50_Q9VSM5	316	44.33	300	155	4	228	1091	4	303	6e-76	255

2.3.1.-(Transferases;)

ABCDEF_520	UniRef50_Q9FF86	0.0	Normal: 1672	85	2273	5056	3383	1211	BlastAnno: 1811	UniRef50_Q9FF86	484	64.06	448	149	6	1664	354	3	449	0.0	573
ABCDEF_68664	UniRef50_Q9ZVN2	0.0	Normal: 79	35	85	107	193	63	BlastAnno: 1488	UniRef50_Q9ZVN2	704	63.99	411	142	3	46	1275	298	703	0.0	540
ABCDEF_52245	UniRef50_Q9FG87	0.0	Normal: 1878	635	1833	3814	1432	1376	BlastAnno: 1758	UniRef50_Q9FG87	529	55.35	486	204	4	4	1428	34	517	0.0	530
ABCDEF_16730	UniRef50_Q3EC11	0.0	Normal: 281	204	237	365	377	458	BlastAnno: 2092	UniRef50_Q3EC11	536	59.33	536	204	8	196	1785	3	530	0.0	661
ABCDEF_4655	UniRef50_A5BMG1	0.0	Normal: 2426	1856	3606	6958	4608	2755	BlastAnno: 2261	UniRef50_A5BMG1	481	68.37	528	112	10	263	1843	3	476	0.0	667
ABCDEF_13860	UniRef50_Q9C533	0.0	Normal: 588	220	570	1451	1338	310	BlastAnno: 2648	UniRef50_Q9C533	596	63.37	606	177	12	2226	511	1	595	0.0	558
ABCDEF_28294	UniRef50_Q9SYZ0	0.0	Normal: 1038	4070	1427	6745	877	2797	BlastAnno: 1785	UniRef50_Q9SYZ0	493	52.37	485	223	3	1598	162	5	487	0.0	533
ABCDEF_47718	UniRef50_Q9C533	0.0	Normal: 1599	236	1657	2093	1231	588	BlastAnno: 2680	UniRef50_Q9C533	596	65.02	626	165	14	2280	472	1	595	0.0	634
ABCDEF_45080	UniRef50_Q9SYZ0	0.0	Normal: 1005	592	1079	2360	3762	2217	BlastAnno: 2130	UniRef50_Q9SYZ0	493	57.85	484	197	3	302	1735	5	487	0.0	533
ABCDEF_25377	UniRef50_Q9C6L5	0.0	Normal: 37	2090	138	2573	1026	1298	BlastAnno: 1334	UniRef50_Q9C6L5	492	79.74	385	76	1	1155	1	6	388	0.0	654
ABCDEF_33470	UniRef50_Q9LW26	0.0	Normal: 172	98	156	260	391	149	BlastAnno: 2993	UniRef50_Q9LW26	701	48.96	627	301	5	655	2508	85	701	0.0	581
ABCDEF_40654	UniRef50_Q9H0A0	0.0	Normal: 638	213	530	318	318	286	BlastAnno: 2216	UniRef50_Q9H0A0	1025	56.87	721	279	6	2127	13	91	795	0.0	756
ABCDEF_33460	UniRef50_Q9ZVN2	0.0	Normal: 144	71	119	216	306	97	BlastAnno: 2691	UniRef50_Q9ZVN2	704	61.50	587	191	4	379	2133	83	636	0.0	715
ABCDEF_14442	UniRef50_Q9SYZ0	0.0	Normal: 341	684	439	1040	1601	552	BlastAnno: 1779	UniRef50_Q9SYZ0	493	63.12	480	171	2	1570	149	5	484	0.0	628
ABCDEF_52626	UniRef50_B6SYP9	0.0	Normal: 815	346	1131	3015	1537	108	BlastAnno: 1861	UniRef50_B6SYP9	494	55.02	438	189	3	1538	249	51	488	0.0	536
ABCDEF_644	UniRef50_Q9SB58	0.0	Normal: 704	444	913	969	1431	848	BlastAnno: 1777	UniRef50_Q9SB58	407	64.15	424	129	8	1572	316	1	406	0.0	554
ABCDEF_27087	UniRef50_Q52T38	0.0	Normal: 324	221	233	419	343	412	BlastAnno: 1631	UniRef50_Q52T38	620	75.95	420	91	1	1409	150	1	410	0.0	681
ABCDEF_34187	UniRef50_Q8L5Y5	0.0	Normal: 497	415	588	523	291	433	BlastAnno: 2677	UniRef50_Q8L5Y5	718	57.30	733	251	15	202	2274	1	713	0.0	683
ABCDEF_47966	UniRef50_Q9SYZ0	0.0	Normal: 577	934	326	580	682	3440	BlastAnno: 2177	UniRef50_Q9SYZ0	493	56.40	484	204	3	1780	347	5	487	0.0	535
ABCDEF_51189	UniRef50_Q52T38	0.0	Normal: 135	152	128	181	219	327	BlastAnno: 1766	UniRef50_Q52T38	620	68.09	470	137	3	80	1483	130	588	0.0	641
ABCDEF_41288	UniRef50_Q9SB58	0.0	Normal: 307	183	332	637	649	322	BlastAnno: 1403	UniRef50_Q9SB58	407	67.56	410	110	6	1401	184	15	405	0.0	523
ABCDEF_33461	UniRef50_Q9ZVN2	0.0	Normal: 142	84	140	206	283	119	BlastAnno: 2904	UniRef50_Q9ZVN2	704	60.98	574	201	6	2271	559	83	636	0.0	686
ABCDEF_14334	UniRef50_Q9SYZ0	0.0	Normal: 2263	129	1724	3469	866	283	BlastAnno: 2039	UniRef50_Q9SYZ0	493	63.22	484	171	2	369	1802	5	487	0.0	627
ABCDEF_24844	UniRef50_Q9FF86	0.0	Normal: 1857	3686	2657	21940	11467	4090	BlastAnno: 1640	UniRef50_Q9FF86	484	66.89	447	135	6	1492	185	2	446	0.0	575
ABCDEF_51190	UniRef50_Q52T38	0.0	Normal: 145	169	142	210	232	335	BlastAnno: 1891	UniRef50_Q52T38	620	70.04	514	141	3	73	1608	86	588	0.0	726
ABCDEF_14085	UniRef50_Q8L5Y5	0.0	Normal: 353	41	384	527	331	280	BlastAnno: 2362	UniRef50_Q8L5Y5	718	62.07	704	220	12	2360	348	24	713	0.0	676
ABCDEF_33458	UniRef50_Q9LW26	0.0	Normal: 158	78	130	240	342	106	BlastAnno: 2822	UniRef50_Q9LW26	701	46.72	655	302	5	400	2337	85	701	0.0	568
ABCDEF_25193	UniRef50_Q9C6L5	0.0	Normal: 3256	368	3548	6230	5109	1288	BlastAnno: 1786	UniRef50_Q9C6L5	492	65.83	480	156	4	1668	241	17	492	0.0	668
ABCDEF_33457	UniRef50_Q9ZVN2	0.0	Normal: 168	83	137	250	375	112	BlastAnno: 2823	UniRef50_Q9ZVN2	704	65.12	496	168	3	379	1860	83	575	0.0	648
ABCDEF_7634	UniRef50_Q9C6L5	0.0	Normal: 2543	9303	2711	11578	5533	10032	BlastAnno: 2064	UniRef50_Q9C6L5	492	52.76	489	225	3	1734	268	1	483	0.0	535
ABCDEF_299	UniRef50_Q71N54	0.0	Normal: 569	1147	511	900	649	1879	BlastAnno: 2017	UniRef50_Q71N54	535	61.73	533	198	5	338	1924	4	534	0.0	677
ABCDEF_27086	UniRef50_Q52T38	0.0	Normal: 784	470	617	843	893	976	BlastAnno: 2398	UniRef50_Q52T38	620	70.80	637	165	4	225	2129	1	618	0.0	887
ABCDEF_51188	UniRef50_Q52T38	0.0	Normal: 273	327	255	415	349	441	BlastAnno: 2324	UniRef50_Q52T38	620	71.30	568	150	3	2041	344	32	588	0.0	815
ABCDEF_44812	UniRef50_Q6DR03	0.0	Normal: 982	902	1005	1071	959	2999	BlastAnno: 2450	UniRef50_Q6DR03	565	54.97	644	198	17	2142	220	1	555	0.0	540

3.1.2.-(Hydrolases;)

ABCDEF_2988	UniRef50_Q39513	0.0	Normal: 684	868	676	1224	1298	1392	BlastAnno: 1728	UniRef50_Q39513	415	69.13	392	111	5	408	1574	12	396	0.0	554
ABCDEF_42638	UniRef50_Q9SHJ8	0.0	Normal: 183	172	331	565	390	214	BlastAnno: 1547	UniRef50_Q9SHJ8	395	68.62	376	94	2	196	1320	42	394	0.0	533

ABCDEF_56804	UniRef50_Q9SHJ8	0.0	Normal: 160	146	249	472	328	155	BlastAnno: 1439	UniRef50_Q9SHJ8	395	68.62	376	94	2	275	1399	42	394	0.0	533
ABCDEF_2987	UniRef50_Q39513	0.0	Normal: 608	561	472	1162	1435	954	BlastAnno: 1938	UniRef50_Q39513	415	67.26	394	119	5	408	1580	12	398	0.0	544
ABCDEF_20456	UniRef50_Q9LK08	0.0	Normal: 375	366	460	796	1014	454	BlastAnno: 1539	UniRef50_Q9LK08	418	64.52	420	140	3	1434	187	4	418	0.0	559
ABCDEF_49725	UniRef50_Q9SHJ8	0.0	Normal: 176	174	327	582	397	195	BlastAnno: 1556	UniRef50_Q9SHJ8	395	68.62	376	94	2	234	1358	42	394	0.0	533
ABCDEF_20453	UniRef50_Q9LK08	0.0	Normal: 396	358	461	824	1019	461	BlastAnno: 1565	UniRef50_Q9LK08	418	66.90	420	134	2	1459	200	4	418	0.0	586
3.3.2.8(limonene-1,2-epoxide hydrolase;)																					
No Correspond Unigene																					
3.7.1.-(Hydrolases;)																					
No Correspond Unigene																					
4.2.1.-(Lyases;)																					
ABCDEF_48679	UniRef50_Q0RP29	5e-115	Normal: 568	325	563	941	1285	676	BlastAnno: 1424	UniRef50_Q0RP29	493	56.39	321	129	4	119	1075	39	350	5e-115	356
4.2.1.17(enoyl-CoA hydratase;)																					
ABCDEF_21259	UniRef50_Q8W1L6	0.0	Normal: 272	276	284	384	326	305	BlastAnno: 2641	UniRef50_Q8W1L6	726	47.57	721	375	3	114	2267	6	726	0.0	633
ABCDEF_80649	UniRef50_Q8W1L6	0.0	Normal: 527	841	443	1308	1321	1188	BlastAnno: 2586	UniRef50_Q8W1L6	726	60.39	712	281	1	2403	268	1	711	0.0	818
ABCDEF_466	UniRef50_Q8W1L6	0.0	Normal: 1014	1690	1094	1677	2438	2080	BlastAnno: 2453	UniRef50_Q8W1L6	726	71.24	699	201	0	2368	272	7	705	0.0	954
ABCDEF_54834	UniRef50_Q8W1L6	0.0	Normal: 229	220	237	298	270	261	BlastAnno: 2405	UniRef50_Q8W1L6	726	47.52	606	315	3	274	2082	121	726	0.0	580
ABCDEF_34756	UniRef50_Q8W1L6	0.0	Normal: 776	726	768	2922	4039	1079	BlastAnno: 2469	UniRef50_Q8W1L6	726	68.93	708	217	2	2396	273	7	711	0.0	957
ABCDEF_4198	UniRef50_Q8W1L6	0.0	Normal: 609	929	532	1401	1568	1297	BlastAnno: 2667	UniRef50_Q8W1L6	726	60.45	713	281	1	2463	325	1	712	0.0	820
5.5.1.10(alpha-pinene-oxide decyclase;)																					
No Correspond Unigene																					
6.2.1.-(Ligases;)																					
ABCDEF_7378	UniRef50_Q9FFE6	0.0	Normal: 1022	1422	746	576	680	1408	BlastAnno: 2117	UniRef50_Q9FFE6	552	67.57	552	167	5	144	1778	1	547	0.0	768
ABCDEF_47513	UniRef50_Q8RU95	0.0	Normal: 411	609	570	233	749	887	BlastAnno: 2280	UniRef50_Q8RU95	598	53.15	572	246	6	119	1777	28	596	0.0	543
ABCDEF_78742	UniRef50_Q84P25	0.0	Normal: 236	207	373	576	450	306	BlastAnno: 1534	UniRef50_Q84P25	565	60.39	462	168	5	1413	49	19	472	0.0	546
ABCDEF_10753	UniRef50_Q9FFE6	0.0	Normal: 394	401	336	253	292	348	BlastAnno: 1841	UniRef50_Q9FFE6	552	51.00	551	259	5	117	1751	1	546	0.0	553
ABCDEF_4431	UniRef50_Q9FFE6	0.0	Normal: 27	358	28	98	112	914	BlastAnno: 2075	UniRef50_Q9FFE6	552	50.71	560	264	7	1774	107	1	552	0.0	551
ABCDEF_38213	UniRef50_Q84P25	0.0	Normal: 342	337	597	798	722	505	BlastAnno: 1939	UniRef50_Q84P25	565	62.34	555	194	5	1818	175	19	565	0.0	690
ABCDEF_25221	UniRef50_Q0DV32	0.0	Normal: 971	826	1003	1543	1442	1545	BlastAnno: 2009	UniRef50_Q0DV32	552	57.20	542	226	4	183	1793	9	549	0.0	613
ABCDEF_23413	UniRef50_Q84P23	0.0	Normal: 123	237	158	149	213	658	BlastAnno: 3175	UniRef50_Q84P23	562	51.74	547	243	8	3030	1435	15	555	0.0	555
ABCDEF_20486	UniRef50_Q9FFE6	0.0	Normal: 580	912	312	324	864	1565	BlastAnno: 3530	UniRef50_Q9FFE6	552	66.61	557	171	5	352	1995	1	551	0.0	781
>00904 Diterpenoid biosynthesis																					
1.1.1.295(momilactone-A synthase;)																					
No Correspond Unigene																					
1.14.-.-(Oxidoreductases;)																					
ABCDEF_44739	UniRef50_O64989	0.0	Normal: 157	87	159	718	673	189	BlastAnno: 2185	UniRef50_O64989	513	70.75	482	112	3	1867	509	30	511	0.0	706
ABCDEF_31491	UniRef50_Q93VK5	0.0	Normal: 647	1826	671	1517	1770	5953	BlastAnno: 2278	UniRef50_Q93VK5	595	79.14	556	113	2	198	1862	35	588	0.0	902
ABCDEF_40554	UniRef50_P37122	0.0	Normal: 1574	1962	1829	1150	2882	2721	BlastAnno: 1828	UniRef50_P37122	505	60.52	504	198	1	1752	244	1	504	0.0	638
ABCDEF_34049	UniRef50_O23365	0.0	Normal: 363	384	349	442	549	1416	BlastAnno: 2101	UniRef50_O23365	580	76.17	579	116	5	230	1957	19	578	0.0	904
ABCDEF_21579	UniRef50_O48928	0.0	Normal: 1005	643	1606	4372	3226	1621	BlastAnno: 1895	UniRef50_O48928	513	69.72	502	151	1	185	1690	7	507	0.0	731
ABCDEF_37741	UniRef50_P37122	0.0	Normal: 12	760	9	24	34	576	BlastAnno: 1197	UniRef50_P37122	505	65.70	379	129	1	1195	59	127	504	0.0	526
ABCDEF_35618	UniRef50_O48928	0.0	Normal: 28	2648	17	142	1234	902	BlastAnno: 1746	UniRef50_O48928	513	66.40	506	168	2	1649	132	4	507	0.0	701
ABCDEF_24522	UniRef50_P37122	0.0	Normal: 334	2190	561	268	1391	2459	BlastAnno: 2075	UniRef50_P37122	505	61.52	460	176	1	1544	165	47	505	0.0	612
ABCDEF_14592	UniRef50_O23066	0.0	Normal: 1514	402	2245	7837	4742	1248	BlastAnno: 2378	UniRef50_O23066	553	72.71	546	140	4	2111	489	1	542	0.0	821
ABCDEF_14512	UniRef50_Q9FG65	0.0	Normal: 261	872	212	291	175	789	BlastAnno: 1878	UniRef50_Q9FG65	502	56.89	501	197	9	1738	266	11	502	0.0	532
ABCDEF_44487	UniRef50_Q940V4	0.0	Normal: 3	41	53	501	262	542	BlastAnno: 1712	UniRef50_Q940V4	465	69.37	382	116	1	38	1180	14	395	0.0	572
ABCDEF_17723	UniRef50_P37122	0.0	Normal: 240	1698	219	768	592	794	BlastAnno: 1757	UniRef50_P37122	505	63.40	459	166	2	228	1601	47	504	0.0	617
ABCDEF_37901	UniRef50_Q42569	0.0	Normal: 30	149	86	255	195	255	BlastAnno: 1246	UniRef50_Q42569	472	78.27	359	73	2	64	1140	43	396	0.0	567
ABCDEF_7260	UniRef50_O81117	0.0	Normal: 314	1085	466	3248	2575	1360	BlastAnno: 1768	UniRef50_O81117	514	59.92	474	188	1	145	1566	43	514	0.0	615
ABCDEF_40548	UniRef50_P37122	0.0	Normal: 2756	2467	2752	1072	3120	1735	BlastAnno: 1947	UniRef50_P37122	505	59.72	504	202	1	241	1749	1	504	0.0	636
ABCDEF_44485	UniRef50_Q940V4	0.0	Normal: 3	55	63	574	338	660	BlastAnno: 1607	UniRef50_Q940V4	465	68.58	452	141	1	42	1394	14	465	0.0	673
ABCDEF_40552	UniRef50_P37122	0.0	Normal: 2397	1870	2304	1249	10428	2291	BlastAnno: 1812	UniRef50_P37122	505	60.12	504	200	1	108	1616	1	504	0.0	640
ABCDEF_37902	UniRef50_Q42569	0.0	Normal: 84	524	254	725	670	1049	BlastAnno: 1812	UniRef50_Q42569	472	77.47	435	92	3	1675	371	43	471	0.0	681
ABCDEF_40556	UniRef50_P37122	0.0	Normal: 1907	2365	2163	1319	3678	3308	BlastAnno: 1944	UniRef50_P37122	505	61.04	498	193	1	264	1754	1	498	0.0	635
ABCDEF_40549	UniRef50_P37122	0.0	Normal: 1574	1962	1826	1150	2882	2723	BlastAnno: 1845	UniRef50_P37122	505	60.52	504	198	1	1754	246	1	504	0.0	638
ABCDEF_20301	UniRef50_O81117	0.0	Normal: 89	308	123	176	389	249	BlastAnno: 1637	UniRef50_O81117	514	64.04	470	164	3	1498	98	47	514	0.0	586
ABCDEF_3882	UniRef50_Q42569	0.0	Normal: 84	243	257	786	1561	182	BlastAnno: 1854	UniRef50_Q42569	472	72.51	422	107	1	1852	587	59	471	0.0	615
ABCDEF_20703	UniRef50_O49858	0.0	Normal: 115	636	20	0	0	597	BlastAnno: 1815	UniRef50_O49858	527	50.10	523	248	6	48	1607	12	524	0.0	533
ABCDEF_18295	UniRef50_O22203	0.0	Normal: 3667	1934	4977	10491	4588	1917	BlastAnno: 2006	UniRef50_O22203	508	74.85	493	123	1	214	1692	17	508	0.0	803
ABCDEF_48724	UniRef50_O23066	0.0	Normal: 104	11518	163	2083	3464	6356	BlastAnno: 2162	UniRef50_O23066	553	72.96	540	143	3	227	1840	1	539	0.0	807
ABCDEF_37738	UniRef50_P37122	0.0	Normal: 26	2447	19	30	33	993	BlastAnno: 1837	UniRef50_P37122	505	64.30	479	169	2	1685	249	28	504	0.0	639
1.14.11.-(Oxidoreductases;)																					
ABCDEF_37086	UniRef50_Q8GUI6	0.0	Normal: 592	429	529	801	742	784	BlastAnno: 3943	UniRef50_Q8GUI6	954	56.60	553	227	4	613	2244	38	586	0.0	643
ABCDEF_51324	UniRef50_Q8GUI6	0.0	Normal: 461	353	534	1098	841	490	BlastAnno: 3289	UniRef50_Q8GUI6	954	60.79	584	214	5	2989	1262	12	588	0.0	698
1.14.11.12(gibberellin-44 dioxygenase;)																					
No Correspond Unigene																					
1.14.11.13(gibberellin 2beta-dioxygenase;)																					
ABCDEF_1155	UniRef50_Q9XG83	3e-153	Normal: 177	227	10	0	71	3312	BlastAnno: 1221	UniRef50_Q9XG83	332	64.56	333	116	2	1108	110	1	331	3e-153	445
1.14.11.15(gibberellin 3beta-dioxygenase;)																					
ABCDEF_75035	UniRef50_B9SME4	3e-132	Normal: 5	0	5	0	1039	10	BlastAnno: 1253	UniRef50_B9SME4	387	58.39	322	133	1	1252	287	65	385	3e-132	394
1.14.13.-(Oxidoreductases;)																					
ABCDEF_45538	UniRef50_O23024	3e-156	Normal: 110	6	165	774	133	36	BlastAnno: 1258	UniRef50_O23024	437	58.18	385	151	3	3	1157	37	411	3e-156	457
1.14.13.108(abietadiene hydroxylase)																					

No Correspond Unigene																					
1.14.13.109(abietadienol hydroxylase;)																					
No Correspond Unigene																					
1.14.13.110(geranylgeraniol 18-hydroxylase;)																					
No Correspond Unigene																					
1.14.13.76(taxane 10beta-hydroxylase)																					
ABCDEF_46241	UniRef50_B9SBP0	4e-156	Normal: 9	41	19	170	795	48	BlastAnno: 1696	UniRef50_B9SBP0	455	46.67	480	227	2	1565	129	1	452	4e-156	463
1.14.13.77(taxane 13alpha-hydroxylase)																					
ABCDEF_46241	UniRef50_B9SPR3	4e-171	Normal: 9	41	19	170	795	48	BlastAnno: 1696	UniRef50_B9SPR3	480	49.59	484	231	5	1565	129	1	476	4e-171	503
1.14.13.78(ent-kaurene oxidase)																					
ABCDEF_64567	UniRef50_Q93ZB2	0.0	Normal: 677	400	355	577	1342	57	BlastAnno: 1658	UniRef50_Q93ZB2	509	60.52	461	181	1	64	1443	47	507	0.0	600
ABCDEF_38222	UniRef50_Q93ZB2	0.0	Normal: 794	485	439	766	1572	55	BlastAnno: 2033	UniRef50_Q93ZB2	509	60.39	467	184	1	1658	261	41	507	0.0	607
1.14.13.79(ent-kaurenoic acid oxidase)																					
ABCDEF_28264	UniRef50_O23051	0.0	Normal: 161	219	120	35	1901	42	BlastAnno: 1737	UniRef50_O23051	490	70.09	468	135	4	1550	150	24	487	0.0	699
1.14.99.37(taxadiene 5alpha-hydroxylase)																					
ABCDEF_34158	UniRef50_Q6WG30	3e-60	Normal: 61	77	37	48	85	16	BlastAnno: 1639	UniRef50_Q6WG30	499	38.57	223	128	3	876	1520	245	466	3e-60	161
1.2.1.74(abietadienal dehydrogenase)																					
No Correspond Unigene																					
2.3.1.-(Transferases;)																					
ABCDEF_520	UniRef50_Q9FF86	0.0	Normal: 1672	85	2273	5056	3383	1211	BlastAnno: 1811	UniRef50_Q9FF86	484	64.06	448	149	6	1664	354	3	449	0.0	573
ABCDEF_68664	UniRef50_Q9ZVN2	0.0	Normal: 79	35	85	107	193	63	BlastAnno: 1488	UniRef50_Q9ZVN2	704	63.99	411	142	3	46	1275	298	703	0.0	540
ABCDEF_52245	UniRef50_Q9FG87	0.0	Normal: 1878	635	1833	3814	1432	1376	BlastAnno: 1758	UniRef50_Q9FG87	529	55.35	486	204	4	4	1428	34	517	0.0	530
ABCDEF_16730	UniRef50_Q3EC11	0.0	Normal: 281	204	237	365	377	458	BlastAnno: 2092	UniRef50_Q3EC11	536	59.33	536	204	8	196	1785	3	530	0.0	661
ABCDEF_4655	UniRef50_A5BMG1	0.0	Normal: 2426	1856	3606	6958	4608	2755	BlastAnno: 2261	UniRef50_A5BMG1	481	68.37	528	112	10	263	1843	3	476	0.0	667
ABCDEF_13860	UniRef50_Q9C533	0.0	Normal: 588	220	570	1451	1338	310	BlastAnno: 2648	UniRef50_Q9C533	596	63.37	606	177	12	2226	511	1	595	0.0	558
ABCDEF_28294	UniRef50_Q9SYZ0	0.0	Normal: 1038	4070	1427	6745	877	2797	BlastAnno: 1785	UniRef50_Q9SYZ0	493	52.37	485	223	3	1598	162	5	487	0.0	533
ABCDEF_47718	UniRef50_Q9C533	0.0	Normal: 1599	236	1657	2093	1231	588	BlastAnno: 2680	UniRef50_Q9C533	596	65.02	626	165	14	2280	472	1	595	0.0	634
ABCDEF_45080	UniRef50_Q9SYZ0	0.0	Normal: 1005	592	1079	2360	3762	2217	BlastAnno: 2130	UniRef50_Q9SYZ0	493	57.85	484	197	3	302	1735	5	487	0.0	533
ABCDEF_25377	UniRef50_Q9C6L5	0.0	Normal: 37	2090	138	2573	1026	1298	BlastAnno: 1334	UniRef50_Q9C6L5	492	79.74	385	76	1	1155	1	6	388	0.0	654
ABCDEF_33470	UniRef50_Q9LW26	0.0	Normal: 172	98	156	260	391	149	BlastAnno: 2993	UniRef50_Q9LW26	701	48.96	627	301	5	655	2508	85	701	0.0	581
ABCDEF_40654	UniRef50_Q9H0A0	0.0	Normal: 638	213	530	318	318	286	BlastAnno: 2216	UniRef50_Q9H0A0	1025	56.87	721	279	6	2127	13	91	795	0.0	756
ABCDEF_33460	UniRef50_Q9ZVN2	0.0	Normal: 144	71	119	216	306	97	BlastAnno: 2691	UniRef50_Q9ZVN2	704	61.50	587	191	4	379	2133	83	636	0.0	715
ABCDEF_14442	UniRef50_Q9SYZ0	0.0	Normal: 341	684	439	1040	1601	552	BlastAnno: 1779	UniRef50_Q9SYZ0	493	63.12	480	171	2	1570	149	5	484	0.0	628
ABCDEF_52626	UniRef50_B6SYP9	0.0	Normal: 815	346	1131	3015	1537	108	BlastAnno: 1861	UniRef50_B6SYP9	494	55.02	438	189	3	1538	249	51	488	0.0	536
ABCDEF_644	UniRef50_Q9SB58	0.0	Normal: 704	444	913	969	1431	848	BlastAnno: 1777	UniRef50_Q9SB58	407	64.15	424	129	8	1572	316	1	406	0.0	554
ABCDEF_27087	UniRef50_Q52T38	0.0	Normal: 324	221	233	419	343	412	BlastAnno: 1631	UniRef50_Q52T38	620	75.95	420	91	1	1409	150	1	410	0.0	681
ABCDEF_34187	UniRef50_Q8L5Y5	0.0	Normal: 497	415	588	523	291	433	BlastAnno: 2677	UniRef50_Q8L5Y5	718	57.30	733	251	15	202	2274	1	713	0.0	683
ABCDEF_47966	UniRef50_Q9SYZ0	0.0	Normal: 577	934	326	580	682	3440	BlastAnno: 2177	UniRef50_Q9SYZ0	493	56.40	484	204	3	1780	347	5	487	0.0	535
ABCDEF_51189	UniRef50_Q52T38	0.0	Normal: 135	152	128	181	219	327	BlastAnno: 1766	UniRef50_Q52T38	620	68.09	470	137	3	80	1483	130	588	0.0	641
ABCDEF_41288	UniRef50_Q9SB58	0.0	Normal: 307	183	332	637	649	322	BlastAnno: 1403	UniRef50_Q9SB58	407	67.56	410	110	6	1401	184	15	405	0.0	523
ABCDEF_33461	UniRef50_Q9ZVN2	0.0	Normal: 142	84	140	206	283	119	BlastAnno: 2904	UniRef50_Q9ZVN2	704	60.98	574	201	6	2271	559	83	636	0.0	686
ABCDEF_14334	UniRef50_Q9SYZ0	0.0	Normal: 2263	129	1724	3469	866	283	BlastAnno: 2039	UniRef50_Q9SYZ0	493	63.22	484	171	2	369	1802	5	487	0.0	627
ABCDEF_24844	UniRef50_Q9FF86	0.0	Normal: 1857	3686	2657	21940	11467	4090	BlastAnno: 1640	UniRef50_Q9FF86	484	66.89	447	135	6	1492	185	2	446	0.0	575
ABCDEF_51190	UniRef50_Q52T38	0.0	Normal: 145	169	142	210	232	335	BlastAnno: 1891	UniRef50_Q52T38	620	70.04	514	141	3	73	1608	86	588	0.0	726
ABCDEF_14085	UniRef50_Q8L5Y5	0.0	Normal: 353	41	384	527	331	280	BlastAnno: 2362	UniRef50_Q8L5Y5	718	62.07	704	220	12	2360	348	24	713	0.0	676
ABCDEF_33458	UniRef50_Q9LW26	0.0	Normal: 158	78	130	240	342	106	BlastAnno: 2822	UniRef50_Q9LW26	701	46.72	655	302	5	400	2337	85	701	0.0	568
ABCDEF_25193	UniRef50_Q9C6L5	0.0	Normal: 3256	368	3548	6230	5109	1288	BlastAnno: 1786	UniRef50_Q9C6L5	492	65.83	480	156	4	1668	241	17	492	0.0	668
ABCDEF_33457	UniRef50_Q9ZVN2	0.0	Normal: 168	83	137	250	375	112	BlastAnno: 2823	UniRef50_Q9ZVN2	704	65.12	496	168	3	379	1860	83	575	0.0	648
ABCDEF_7634	UniRef50_Q9C6L5	0.0	Normal: 2543	9303	2711	11578	5533	10032	BlastAnno: 2064	UniRef50_Q9C6L5	492	52.76	489	225	3	1734	268	1	483	0.0	535
ABCDEF_299	UniRef50_Q71N54	0.0	Normal: 569	1147	511	900	649	1879	BlastAnno: 2017	UniRef50_Q71N54	535	61.73	533	198	5	338	1924	4	534	0.0	677
ABCDEF_27086	UniRef50_Q52T38	0.0	Normal: 784	470	617	843	893	976	BlastAnno: 2398	UniRef50_Q52T38	620	70.80	637	165	4	225	2129	1	618	0.0	887
ABCDEF_51188	UniRef50_Q52T38	0.0	Normal: 273	327	255	415	349	441	BlastAnno: 2324	UniRef50_Q52T38	620	71.30	568	150	3	2041	344	32	588	0.0	815
ABCDEF_44812	UniRef50_Q6DR03	0.0	Normal: 982	902	1005	1071	959	2999	BlastAnno: 2450	UniRef50_Q6DR03	565	54.97	644	198	17	2142	220	1	555	0.0	540
2.3.1.162(taxadien-5alpha-ol O-acetyltransferase;)																					
ABCDEF_38214	UniRef50_B9SDF5	1e-166	Normal: 1318	2729	962	748	2864	1118	BlastAnno: 1685	UniRef50_B9SDF5	434	55.23	449	166	6	1616	288	1	420	1e-166	489
2.3.1.166(2alpha-hydroxytaxane 2-O-benzoyltransferase;)																					
No Correspond Unigene																					
2.3.1.167(10-deacetylbaccatin III 10-O-acetyltransferase)																					
No Correspond Unigene																					
2.3.2.-(Transferases;)																					
ABCDEF_41698	UniRef50_Q9M8T3	7e-62	Normal: 110	102	151	190	213	210	BlastAnno: 870	UniRef50_Q9M8T3	182	58.29	187	63	4	740	210	1	182	7e-62	202
3.1.7.4(sclareol cyclase;)																					
No Correspond Unigene																					
3.1.7.5(geranylgeranyl diphosphate diphosphatase;)																					
No Correspond Unigene																					
4.2.3.17(taxadiene synthase;)																					
No Correspond Unigene																					
4.2.3.18(abietadiene synthase;)																					
No Correspond Unigene																					
4.2.3.19(ent-kaurene synthase;)																					
ABCDEF_30616	UniRef50_B9SIL7	0.0	Normal: 514	201	395	484	549	320	BlastAnno: 3023	UniRef50_B9SIL7	816	46.13	737	381	8	636	2837	88	811	0.0	645
4.2.3.28(ent-cassa-12,15-diene synthase;)																					

No Correspond Unigene																					
4.2.3.29(ent-sandaracopimaradiene synthase;)																					
No Correspond Unigene																					
4.2.3.30(ent-pimara-8(14),15-diene synthase;)																					
No Correspond Unigene																					
4.2.3.32(levopimaradiene synthase;)																					
ABCDEF_30194	UniRef50_Q947C4	3e-128	Normal: 4	218	3	4	184	0	BlastAnno: 2093	UniRef50_Q947C4	873	41.75	515	287	8	4	1536	211	716	3e-128	410
4.2.3.33(stemar-13-ene synthase;)																					
No Correspond Unigene																					
4.2.3.34(stemod-13(17)-ene synthase;)																					
No Correspond Unigene																					
4.2.3.35(syn-pimara-7,15-diene synthase;)																					
ABCDEF_30616	UniRef50_Q0JEZ8	0.0	Normal: 514	201	395	484	549	320	BlastAnno: 3023	UniRef50_Q0JEZ8	842	44.43	745	399	10	627	2837	100	837	0.0	600
4.2.3.42(aphidicolan-16beta-ol synthase;)																					
No Correspond Unigene																					
4.2.3.43(fusicocca-2,10(14)-diene synthase;)																					
No Correspond Unigene																					
4.2.3.44(isopimara-7,15-diene synthase;)																					
No Correspond Unigene																					
4.2.3.8(casbene synthase;)																					
ABCDEF_12021	UniRef50_P59287	9e-140	Normal: 2436	29	818	0	600	0	BlastAnno: 1866	UniRef50_P59287	601	39.27	550	323	10	1817	186	57	601	9e-140	429
5.5.1.12(copalyl diphosphate synthase)																					
No Correspond Unigene																					
5.5.1.13(ent-copalyl diphosphate synthase;)																					
ABCDEF_30194	UniRef50_Q38802	0.0	Normal: 4	218	3	4	184	0	BlastAnno: 2093	UniRef50_Q38802	802	54.03	633	268	7	1	1872	184	802	0.0	708
5.5.1.14(syn-copalyl-diphosphate synthase;)																					
ABCDEF_30194	UniRef50_Q0JF02	9e-177	Normal: 4	218	3	4	184	0	BlastAnno: 2093	UniRef50_Q0JF02	767	44.09	635	304	11	1	1872	172	766	9e-177	532
>00905 Brassinosteroid biosynthesis																					
1.14.-.-(Oxidoreductases;)																					
ABCDEF_44739	UniRef50_O64989	0.0	Normal: 157	87	159	718	673	189	BlastAnno: 2185	UniRef50_O64989	513	70.75	482	112	3	1867	509	30	511	0.0	706
ABCDEF_31491	UniRef50_Q93VK5	0.0	Normal: 647	1826	671	1517	1770	5953	BlastAnno: 2278	UniRef50_Q93VK5	595	79.14	556	113	2	198	1862	35	588	0.0	902
ABCDEF_40554	UniRef50_P37122	0.0	Normal: 1574	1962	1829	1150	2882	2721	BlastAnno: 1828	UniRef50_P37122	505	60.52	504	198	1	1752	244	1	504	0.0	638
ABCDEF_34049	UniRef50_O23365	0.0	Normal: 363	384	349	442	549	1416	BlastAnno: 2101	UniRef50_O23365	580	76.17	579	116	5	230	1957	19	578	0.0	904
ABCDEF_21579	UniRef50_O48928	0.0	Normal: 1005	643	1606	4372	3226	1621	BlastAnno: 1895	UniRef50_O48928	513	69.72	502	151	1	185	1690	7	507	0.0	731
ABCDEF_37741	UniRef50_P37122	0.0	Normal: 12	760	9	24	34	576	BlastAnno: 1197	UniRef50_P37122	505	65.70	379	129	1	1195	59	127	504	0.0	526
ABCDEF_35618	UniRef50_O48928	0.0	Normal: 28	2648	17	142	1234	902	BlastAnno: 1746	UniRef50_O48928	513	66.40	506	168	2	1649	132	4	507	0.0	701
ABCDEF_24522	UniRef50_P37122	0.0	Normal: 334	2190	561	268	1391	2459	BlastAnno: 2075	UniRef50_P37122	505	61.52	460	176	1	1544	165	47	505	0.0	612
ABCDEF_14592	UniRef50_O23066	0.0	Normal: 1514	402	2245	7837	4742	1248	BlastAnno: 2378	UniRef50_O23066	553	72.71	546	140	4	2111	489	1	542	0.0	821
ABCDEF_14512	UniRef50_Q9FG65	0.0	Normal: 261	872	212	291	175	789	BlastAnno: 1878	UniRef50_Q9FG65	502	56.89	501	197	9	1738	266	11	502	0.0	532
ABCDEF_44487	UniRef50_Q940V4	0.0	Normal: 3	41	53	501	262	542	BlastAnno: 1712	UniRef50_Q940V4	465	69.37	382	116	1	38	1180	14	395	0.0	572
ABCDEF_17723	UniRef50_P37122	0.0	Normal: 240	1698	219	768	592	794	BlastAnno: 1757	UniRef50_P37122	505	63.40	459	166	2	228	1601	47	504	0.0	617
ABCDEF_37901	UniRef50_Q42569	0.0	Normal: 30	149	86	255	195	255	BlastAnno: 1246	UniRef50_Q42569	472	78.27	359	73	2	64	1140	43	396	0.0	567
ABCDEF_7260	UniRef50_O81117	0.0	Normal: 314	1085	466	3248	2575	1360	BlastAnno: 1768	UniRef50_O81117	514	59.92	474	188	1	145	1566	43	514	0.0	615
ABCDEF_40548	UniRef50_P37122	0.0	Normal: 2756	2467	2752	1072	3120	1735	BlastAnno: 1947	UniRef50_P37122	505	59.72	504	202	1	241	1749	1	504	0.0	636
ABCDEF_44485	UniRef50_Q940V4	0.0	Normal: 3	55	63	574	338	660	BlastAnno: 1607	UniRef50_Q940V4	465	68.58	452	141	1	42	1394	14	465	0.0	673
ABCDEF_40552	UniRef50_P37122	0.0	Normal: 2397	1870	2304	1249	10428	2291	BlastAnno: 1812	UniRef50_P37122	505	60.12	504	200	1	108	1616	1	504	0.0	640
ABCDEF_37902	UniRef50_Q42569	0.0	Normal: 84	524	254	725	670	1049	BlastAnno: 1812	UniRef50_Q42569	472	77.47	435	92	3	1675	371	43	471	0.0	681
ABCDEF_40556	UniRef50_P37122	0.0	Normal: 1907	2365	2163	1319	3678	3308	BlastAnno: 1944	UniRef50_P37122	505	61.04	498	193	1	264	1754	1	498	0.0	635
ABCDEF_40549	UniRef50_P37122	0.0	Normal: 1574	1962	1826	1150	2882	2723	BlastAnno: 1845	UniRef50_P37122	505	60.52	504	198	1	1754	246	1	504	0.0	638
ABCDEF_20301	UniRef50_O81117	0.0	Normal: 89	308	123	176	389	249	BlastAnno: 1637	UniRef50_O81117	514	64.04	470	164	3	1498	98	47	514	0.0	586
ABCDEF_3882	UniRef50_Q42569	0.0	Normal: 84	243	257	786	1561	182	BlastAnno: 1854	UniRef50_Q42569	472	72.51	422	107	1	1852	587	59	471	0.0	615
ABCDEF_20703	UniRef50_O49858	0.0	Normal: 115	636	20	0	0	597	BlastAnno: 1815	UniRef50_O49858	527	50.10	523	248	6	48	1607	12	524	0.0	533
ABCDEF_18295	UniRef50_O22203	0.0	Normal: 3667	1934	4977	10491	4588	1917	BlastAnno: 2006	UniRef50_O22203	508	74.85	493	123	1	214	1692	17	508	0.0	803
ABCDEF_48724	UniRef50_O23066	0.0	Normal: 104	11518	163	2083	3464	6356	BlastAnno: 2162	UniRef50_O23066	553	72.96	540	143	3	227	1840	1	539	0.0	807
ABCDEF_37738	UniRef50_P37122	0.0	Normal: 26	2447	19	30	33	993	BlastAnno: 1837	UniRef50_P37122	505	64.30	479	169	2	1685	249	28	504	0.0	639
1.14.13.-(Oxidoreductases;)																					
ABCDEF_45538	UniRef50_O23024	3e-156	Normal: 110	6	165	774	133	36	BlastAnno: 1258	UniRef50_O23024	437	58.18	385	151	3	3	1157	37	411	3e-156	457
1.14.13.112(3-epi-6-deoxocathasterone 23-monooxygenase;)																					
ABCDEF_354	UniRef50_Q9M066	0.0	Normal: 334	343	609	1743	1285	642	BlastAnno: 2004	UniRef50_Q9M066	524	65.92	446	146	3	1734	400	69	509	0.0	607
1.3.99.-(Oxidoreductases;)																					
ABCDEF_4448	UniRef50_Q6JQN1	0.0	Normal: 1778	1683	1752	2720	2409	2803	BlastAnno: 2780	UniRef50_Q6JQN1	1059	48.13	802	354	14	231	2564	293	1056	0.0	718
>00906 Carotenoid biosynthesis																					
1.-.-.-(Oxidoreductases)																					
ABCDEF_345	UniRef50_Q6Z690	0.0	Normal: 442	146	449	340	272	314	BlastAnno: 1658	UniRef50_Q6Z690	849	63.42	462	150	5	1657	314	387	843	0.0	589
ABCDEF_24068	UniRef50_Q9FI41	0.0	Normal: 214	316	207	270	263	849	BlastAnno: 1742	UniRef50_Q9FI41	387	73.28	363	96	1	1440	355	4	366	0.0	554
ABCDEF_24070	UniRef50_Q9FI41	0.0	Normal: 189	290	182	231	232	751	BlastAnno: 1479	UniRef50_Q9FI41	387	73.28	363	96	1	1363	278	4	366	0.0	551
ABCDEF_13957	UniRef50_Q9CAE3	0.0	Normal: 399	318	392	489	456	586	BlastAnno: 2488	UniRef50_Q9CAE3	789	80.85	496	93	2	3	1487	285	779	0.0	768
ABCDEF_28410	UniRef50_Q9LIG0	0.0	Normal: 211	366	234	286	433	1504	BlastAnno: 1324	UniRef50_Q9LIG0	330	74.92	331	76	2	1147	173	1	330	0.0	521
ABCDEF_17581	UniRef50_Q8LGI2	0.0	Normal: 758	336	735	426	374	852	BlastAnno: 1543	UniRef50_Q8LGI2	454	68.78	442	136	2	71	1390	8	449	0.0	597
ABCDEF_23753	UniRef50_Q94A68	0.0	Normal: 284	559	215	224	164	519	BlastAnno: 1580	UniRef50_Q94A68	377	81.90	348	63	0	1349	306	30	377	0.0	576
ABCDEF_24071	UniRef50_Q9FI41	0.0	Normal: 235	377	230	278	289	948	BlastAnno: 2090	UniRef50_Q9FI41	387	73.28	363	96	1	1726	641	4	366	0.0	553
1.1.1.288(xanthoxin dehydrogenase;)																					

ABCDEF_25531	UniRef50_Q9C826	2e-127	Normal: 682	590	352	248	546	2051	BlastAnno: 1184	UniRef50_Q9C826	285	72.22	270	72	2	1042	242	16	285	2e-127	377
1.10.99.3(violaxanthin de-epoxidase;)																					
ABCDEF_41221	UniRef50_Q39249	5e-179	Normal: 302	746	255	461	486	2211	BlastAnno: 1888	UniRef50_Q39249	462	76.20	353	79	2	1259	216	100	452	5e-179	525
1.13.11.51(9-cis-epoxycarotenoid dioxygenase;)																					
ABCDEF_38468	UniRef50_Q9M9F5	0.0	Normal: 536	357	44	15	25	1417	BlastAnno: 2143	UniRef50_Q9M9F5	657	74.32	514	127	3	1785	247	146	655	0.0	813
1.14.-.-(Oxidoreductases;)																					
ABCDEF_44739	UniRef50_O64989	0.0	Normal: 157	87	159	718	673	189	BlastAnno: 2185	UniRef50_O64989	513	70.75	482	112	3	1867	509	30	511	0.0	706
ABCDEF_31491	UniRef50_Q93VK5	0.0	Normal: 647	1826	671	1517	1770	5953	BlastAnno: 2278	UniRef50_Q93VK5	595	79.14	556	113	2	198	1862	35	588	0.0	902
ABCDEF_40554	UniRef50_P37122	0.0	Normal: 1574	1962	1829	1150	2882	2721	BlastAnno: 1828	UniRef50_P37122	505	60.52	504	198	1	1752	244	1	504	0.0	638
ABCDEF_34049	UniRef50_O23365	0.0	Normal: 363	384	349	442	549	1416	BlastAnno: 2101	UniRef50_O23365	580	76.17	579	116	5	230	1957	19	578	0.0	904
ABCDEF_21579	UniRef50_O48928	0.0	Normal: 1005	643	1606	4372	3226	1621	BlastAnno: 1895	UniRef50_O48928	513	69.72	502	151	1	185	1690	7	507	0.0	731
ABCDEF_37741	UniRef50_P37122	0.0	Normal: 12	760	9	24	34	576	BlastAnno: 1197	UniRef50_P37122	505	65.70	379	129	1	1195	59	127	504	0.0	526
ABCDEF_35618	UniRef50_O48928	0.0	Normal: 28	2648	17	142	1234	902	BlastAnno: 1746	UniRef50_O48928	513	66.40	506	168	2	1649	132	4	507	0.0	701
ABCDEF_24522	UniRef50_P37122	0.0	Normal: 334	2190	561	268	1391	2459	BlastAnno: 2075	UniRef50_P37122	505	61.52	460	176	1	1544	165	47	505	0.0	612
ABCDEF_14592	UniRef50_O23066	0.0	Normal: 1514	402	2245	7837	4742	1248	BlastAnno: 2378	UniRef50_O23066	553	72.71	546	140	4	2111	489	1	542	0.0	821
ABCDEF_14512	UniRef50_Q9FG65	0.0	Normal: 261	872	212	291	175	789	BlastAnno: 1878	UniRef50_Q9FG65	502	56.89	501	197	9	1738	266	11	502	0.0	532
ABCDEF_44487	UniRef50_Q940V4	0.0	Normal: 3	41	53	501	262	542	BlastAnno: 1712	UniRef50_Q940V4	465	69.37	382	116	1	38	1180	14	395	0.0	572
ABCDEF_17723	UniRef50_P37122	0.0	Normal: 240	1698	219	768	592	794	BlastAnno: 1757	UniRef50_P37122	505	63.40	459	166	2	228	1601	47	504	0.0	617
ABCDEF_37901	UniRef50_Q42569	0.0	Normal: 30	149	86	255	195	255	BlastAnno: 1246	UniRef50_Q42569	472	78.27	359	73	2	64	1140	43	396	0.0	567
ABCDEF_7260	UniRef50_O81117	0.0	Normal: 314	1085	466	3248	2575	1360	BlastAnno: 1768	UniRef50_O81117	514	59.92	474	188	1	145	1566	43	514	0.0	615
ABCDEF_40548	UniRef50_P37122	0.0	Normal: 2756	2467	2752	1072	3120	1735	BlastAnno: 1947	UniRef50_P37122	505	59.72	504	202	1	241	1749	1	504	0.0	636
ABCDEF_44485	UniRef50_Q940V4	0.0	Normal: 3	55	63	574	338	660	BlastAnno: 1607	UniRef50_Q940V4	465	68.58	452	141	1	42	1394	14	465	0.0	673
ABCDEF_40552	UniRef50_P37122	0.0	Normal: 2397	1870	2304	1249	10428	2291	BlastAnno: 1812	UniRef50_P37122	505	60.12	504	200	1	108	1616	1	504	0.0	640
ABCDEF_37902	UniRef50_Q42569	0.0	Normal: 84	524	254	725	670	1049	BlastAnno: 1812	UniRef50_Q42569	472	77.47	435	92	3	1675	371	43	471	0.0	681
ABCDEF_40556	UniRef50_P37122	0.0	Normal: 1907	2365	2163	1319	3678	3308	BlastAnno: 1944	UniRef50_P37122	505	61.04	498	193	1	264	1754	1	498	0.0	635
ABCDEF_40549	UniRef50_P37122	0.0	Normal: 1574	1962	1826	1150	2882	2723	BlastAnno: 1845	UniRef50_P37122	505	60.52	504	198	1	1754	246	1	504	0.0	638
ABCDEF_20301	UniRef50_O81117	0.0	Normal: 89	308	123	176	389	249	BlastAnno: 1637	UniRef50_O81117	514	64.04	470	164	3	1498	98	47	514	0.0	586
ABCDEF_3882	UniRef50_Q42569	0.0	Normal: 84	243	257	786	1561	182	BlastAnno: 1854	UniRef50_Q42569	472	72.51	422	107	1	1852	587	59	471	0.0	615
ABCDEF_20703	UniRef50_O49858	0.0	Normal: 115	636	20	0	0	597	BlastAnno: 1815	UniRef50_O49858	527	50.10	523	248	6	48	1607	12	524	0.0	533
ABCDEF_18295	UniRef50_O22203	0.0	Normal: 3667	1934	4977	10491	4588	1917	BlastAnno: 2006	UniRef50_O22203	508	74.85	493	123	1	214	1692	17	508	0.0	803
ABCDEF_48724	UniRef50_O23066	0.0	Normal: 104	11518	163	2083	3464	6356	BlastAnno: 2162	UniRef50_O23066	553	72.96	540	143	3	227	1840	1	539	0.0	807
ABCDEF_37738	UniRef50_P37122	0.0	Normal: 26	2447	19	30	33	993	BlastAnno: 1837	UniRef50_P37122	505	64.30	479	169	2	1685	249	28	504	0.0	639
1.14.13.-(Oxidoreductases;)																					
ABCDEF_45538	UniRef50_O23024	3e-156	Normal: 110	6	165	774	133	36	BlastAnno: 1258	UniRef50_O23024	437	58.18	385	151	3	3	1157	37	411	3e-156	457
1.14.13.90(zeaxanthin epoxidase;)																					
ABCDEF_14394	UniRef50_B9H6G3	0.0	Normal: 1058	4977	757	657	1067	8368	BlastAnno: 2442	UniRef50_B9H6G3	692	69.86	700	173	6	2206	194	1	691	0.0	929
1.14.13.93(No Name)																					
ABCDEF_76458	UniRef50_Q6ZDE3	0.0	Normal: 59	88	36	31	78	17	BlastAnno: 1371	UniRef50_Q6ZDE3	510	60.29	413	155	3	1290	79	80	492	0.0	539
ABCDEF_17772	UniRef50_O81077	0.0	Normal: 1	53	62	273	520	1966	BlastAnno: 1935	UniRef50_O81077	482	61.42	438	161	4	153	1448	46	481	0.0	556
ABCDEF_56894	UniRef50_Q6ZDE3	0.0	Normal: 54	88	26	29	58	15	BlastAnno: 1539	UniRef50_Q6ZDE3	510	62.44	426	151	3	191	1441	44	469	0.0	576
ABCDEF_34157	UniRef50_O81077	0.0	Normal: 77	121	49	52	104	23	BlastAnno: 1763	UniRef50_O81077	482	59.17	436	177	1	206	1510	43	478	0.0	541
1.14.99.-(Oxidoreductases;)																					
ABCDEF_66639	UniRef50_Q0DUI8	0.0	Normal: 646	675	723	1956	1163	1303	BlastAnno: 1867	UniRef50_Q0DUI8	578	85.02	414	61	1	167	1408	158	570	0.0	734
ABCDEF_52814	UniRef50_Q0DUI8	0.0	Normal: 1118	1292	1150	3508	1987	2103	BlastAnno: 2562	UniRef50_Q0DUI8	578	77.22	575	124	4	370	2094	3	570	0.0	909
1.14.99.30(carotene 7,8-desaturase;)																					
No Correspond Unigene																					
1.2.3.14(abscisic-aldehyde oxidase;)																					
No Correspond Unigene																					
2.1.1.-(Transferases;)																					
ABCDEF_20806	UniRef50_Q94II3	0.0	Normal: 700	332	876	1458	1997	929	BlastAnno: 2275	UniRef50_Q94II3	600	73.57	594	153	4	2119	341	1	591	0.0	907
ABCDEF_45802	UniRef50_Q9ZPH9	0.0	Normal: 3543	3348	4296	9797	10830	5017	BlastAnno: 2642	UniRef50_Q9ZPH9	633	53.90	603	261	7	2216	456	23	624	0.0	676
ABCDEF_20070	UniRef50_Q944R7	0.0	Normal: 301	141	265	207	185	187	BlastAnno: 2675	UniRef50_Q944R7	724	58.28	719	275	9	127	2265	13	712	0.0	840
ABCDEF_34956	UniRef50_Q94II3	0.0	Normal: 1120	7269	2475	16033	22219	21461	BlastAnno: 2676	UniRef50_Q94II3	600	73.77	610	146	4	404	2230	1	597	0.0	957
ABCDEF_17121	UniRef50_Q9ZPH9	0.0	Normal: 124	77	109	284	199	132	BlastAnno: 2066	UniRef50_Q9ZPH9	633	53.24	601	259	8	161	1903	25	623	0.0	644
ABCDEF_6524	UniRef50_Q9ZPH9	0.0	Normal: 471	296	490	753	870	730	BlastAnno: 2602	UniRef50_Q9ZPH9	633	50.89	619	290	5	2221	404	6	623	0.0	659
ABCDEF_56017	UniRef50_Q9ZPH9	0.0	Normal: 302	167	286	596	748	529	BlastAnno: 2384	UniRef50_Q9ZPH9	633	51.37	619	287	5	213	2030	6	623	0.0	663
ABCDEF_44169	UniRef50_Q9SIZ3	0.0	Normal: 179	158	273	705	685	229	BlastAnno: 2300	UniRef50_Q9SIZ3	589	62.01	308	108	4	1219	296	285	583	0.0	394
ABCDEF_6521	UniRef50_Q9ZPH9	0.0	Normal: 339	198	314	641	767	582	BlastAnno: 2511	UniRef50_Q9ZPH9	633	51.37	619	287	5	2172	355	6	623	0.0	663
ABCDEF_44172	UniRef50_Q9SIZ3	0.0	Normal: 204	184	287	767	741	242	BlastAnno: 2317	UniRef50_Q9SIZ3	589	68.15	449	134	4	1637	291	144	583	0.0	641
ABCDEF_199	UniRef50_Q9SU94	0.0	Normal: 1076	275	1009	558	1247	306	BlastAnno: 1769	UniRef50_Q9SU94	390	78.45	348	74	1	284	1324	43	390	0.0	584
ABCDEF_52252	UniRef50_Q84W92	0.0	Normal: 1658	720	1653	1980	2289	1601	BlastAnno: 2170	UniRef50_Q84W92	535	72.49	469	120	3	409	1791	34	501	0.0	703
ABCDEF_51404	UniRef50_A3BMN9	0.0	Normal: 864	188	716	337	631	326	BlastAnno: 2192	UniRef50_A3BMN9	620	50.50	620	243	9	2008	251	55	620	0.0	555
ABCDEF_21386	UniRef50_Q94AI4	0.0	Normal: 2326	1250	2220	4294	2920	1983	BlastAnno: 3701	UniRef50_Q94AI4	775	47.27	842	332	16	567	2978	8	775	0.0	577
ABCDEF_35101	UniRef50_Q9MAT5	0.0	Normal: 1440	420	1268	576	1136	850	BlastAnno: 1634	UniRef50_Q9MAT5	383	69.03	352	109	0	1449	394	32	383	0.0	550
ABCDEF_960	UniRef50_Q9LN50	0.0	Normal: 3504	13198	3609	11156	19651	12155	BlastAnno: 2626	UniRef50_Q9LN50	724	49.14	525	246	5	883	2451	219	724	0.0	570
ABCDEF_48082	UniRef50_Q9LN50	0.0	Normal: 410	445	467	870	993	454	BlastAnno: 2340	UniRef50_Q9LN50	724	70.25	521	155	0	710	2272	204	724	0.0	821
ABCDEF_10473	UniRef50_Q08A71	0.0	Normal: 374	187	332	425	456	314	BlastAnno: 1764	UniRef50_Q08A71	435	79.23	366	75	1	344	1438	70	435	0.0	575
ABCDEF_7295	UniRef50_Q9ZPH9	0.0	Normal: 25	54	30	81	26	329	BlastAnno: 1191	UniRef50_Q9ZPH9	633	69.23	403	110	4	1191	22	225	626	0.0	599
ABCDEF_4160	UniRef50_Q94II3	0.0	Normal: 189	0	210	71	17	72	BlastAnno: 1424	UniRef50_Q94II3	600	77.28	449	101	1	1424	81	143	591	0.0	758
ABCDEF_44857	UniRef50_Q9ZPH9	0.0	Normal: 101	224	103	299	588	350	BlastAnno: 1582	UniRef50_Q9ZPH9	633	72.06	519	143	1	1571	21	114	632	0.0	822
ABCDEF_71541	UniRef50_Q9SIZ3	0.0	Normal: 187	167	296	765	724	237	BlastAnno: 2268	UniRef50_Q9SIZ3	589	62.01	308	108	4	1210	287	285	583	0.0	394
ABCDEF_44171	UniRef50_Q9SIZ3	0.0	Normal: 214	196	312	827	802	251	BlastAnno: 2339	UniRef50_Q9SIZ3	589	64.78	548	182	5	1978	335	47	583	0.0	717

ABCDEF_20805	UniRef50_Q94II3	0.0	Normal: 674	344	885	1417	2194	963	BlastAnno: 2400	UniRef50_Q94II3	600	78.21	436	94	1	879	2183	156	591	0.0	734
ABCDEF_18210	UniRef50_Q9SIZ3	0.0	Normal: 5584	3714	7379	17006	21892	7546	BlastAnno: 3199	UniRef50_Q9SIZ3	589	50.00	576	269	7	2145	424	27	585	0.0	574
ABCDEF_6526	UniRef50_Q9ZPH9	0.0	Normal: 627	185	837	2717	2815	1530	BlastAnno: 2611	UniRef50_Q9ZPH9	633	54.76	599	259	5	462	2222	25	623	0.0	689
ABCDEF_6525	UniRef50_Q9ZPH9	0.0	Normal: 602	165	760	2369	2605	1400	BlastAnno: 2545	UniRef50_Q9ZPH9	633	54.92	599	258	5	431	2191	25	623	0.0	689
ABCDEF_6523	UniRef50_Q9ZPH9	0.0	Normal: 314	175	304	641	784	552	BlastAnno: 2312	UniRef50_Q9ZPH9	633	52.42	599	272	4	201	1958	25	623	0.0	663
ABCDEF_67393	UniRef50_Q9ZPH9	0.0	Normal: 434	245	444	608	768	655	BlastAnno: 2346	UniRef50_Q9ZPH9	633	51.92	599	275	4	232	1989	25	623	0.0	659
ABCDEF_3579	UniRef50_O14744	0.0	Normal: 1361	326	984	568	791	440	BlastAnno: 2369	UniRef50_O14744	637	47.00	634	294	10	267	2120	30	637	0.0	553
2.3.1.-(Transferases;)																					
ABCDEF_520	UniRef50_Q9FF86	0.0	Normal: 1672	85	2273	5056	3383	1211	BlastAnno: 1811	UniRef50_Q9FF86	484	64.06	448	149	6	1664	354	3	449	0.0	573
ABCDEF_68664	UniRef50_Q9ZVN2	0.0	Normal: 79	35	85	107	193	63	BlastAnno: 1488	UniRef50_Q9ZVN2	704	63.99	411	142	3	46	1275	298	703	0.0	540
ABCDEF_52245	UniRef50_Q9FG87	0.0	Normal: 1878	635	1833	3814	1432	1376	BlastAnno: 1758	UniRef50_Q9FG87	529	55.35	486	204	4	4	1428	34	517	0.0	530
ABCDEF_16730	UniRef50_Q3EC11	0.0	Normal: 281	204	237	365	377	458	BlastAnno: 2092	UniRef50_Q3EC11	536	59.33	536	204	8	196	1785	3	530	0.0	661
ABCDEF_4655	UniRef50_A5BMG1	0.0	Normal: 2426	1856	3606	6958	4608	2755	BlastAnno: 2261	UniRef50_A5BMG1	481	68.37	528	112	10	263	1843	3	476	0.0	667
ABCDEF_13860	UniRef50_Q9C533	0.0	Normal: 588	220	570	1451	1338	310	BlastAnno: 2648	UniRef50_Q9C533	596	63.37	606	177	12	2226	511	1	595	0.0	558
ABCDEF_28294	UniRef50_Q9SYZ0	0.0	Normal: 1038	4070	1427	6745	877	2797	BlastAnno: 1785	UniRef50_Q9SYZ0	493	52.37	485	223	3	1598	162	5	487	0.0	533
ABCDEF_47718	UniRef50_Q9C533	0.0	Normal: 1599	236	1657	2093	1231	588	BlastAnno: 2680	UniRef50_Q9C533	596	65.02	626	165	14	2280	472	1	595	0.0	634
ABCDEF_45080	UniRef50_Q9SYZ0	0.0	Normal: 1005	592	1079	2360	3762	2217	BlastAnno: 2130	UniRef50_Q9SYZ0	493	57.85	484	197	3	302	1735	5	487	0.0	533
ABCDEF_25377	UniRef50_Q9C6L5	0.0	Normal: 37	2090	138	2573	1026	1298	BlastAnno: 1334	UniRef50_Q9C6L5	492	79.74	385	76	1	1155	1	6	388	0.0	654
ABCDEF_33470	UniRef50_Q9LW26	0.0	Normal: 172	98	156	260	391	149	BlastAnno: 2993	UniRef50_Q9LW26	701	48.96	627	301	5	655	2508	85	701	0.0	581
ABCDEF_40654	UniRef50_Q9H0A0	0.0	Normal: 638	213	530	318	318	286	BlastAnno: 2216	UniRef50_Q9H0A0	1025	56.87	721	279	6	2127	13	91	795	0.0	756
ABCDEF_33460	UniRef50_Q9ZVN2	0.0	Normal: 144	71	119	216	306	97	BlastAnno: 2691	UniRef50_Q9ZVN2	704	61.50	587	191	4	379	2133	83	636	0.0	715
ABCDEF_14442	UniRef50_Q9SYZ0	0.0	Normal: 341	684	439	1040	1601	552	BlastAnno: 1779	UniRef50_Q9SYZ0	493	63.12	480	171	2	1570	149	5	484	0.0	628
ABCDEF_52626	UniRef50_B6SYP9	0.0	Normal: 815	346	1131	3015	1537	108	BlastAnno: 1861	UniRef50_B6SYP9	494	55.02	438	189	3	1538	249	51	488	0.0	536
ABCDEF_644	UniRef50_Q9SB58	0.0	Normal: 704	444	913	969	1431	848	BlastAnno: 1777	UniRef50_Q9SB58	407	64.15	424	129	8	1572	316	1	406	0.0	554
ABCDEF_27087	UniRef50_Q52T38	0.0	Normal: 324	221	233	419	343	412	BlastAnno: 1631	UniRef50_Q52T38	620	75.95	420	91	1	1409	150	1	410	0.0	681
ABCDEF_34187	UniRef50_Q8L5Y5	0.0	Normal: 497	415	588	523	291	433	BlastAnno: 2677	UniRef50_Q8L5Y5	718	57.30	733	251	15	202	2274	1	713	0.0	683
ABCDEF_47966	UniRef50_Q9SYZ0	0.0	Normal: 577	934	326	580	682	3440	BlastAnno: 2177	UniRef50_Q9SYZ0	493	56.40	484	204	3	1780	347	5	487	0.0	535
ABCDEF_51189	UniRef50_Q52T38	0.0	Normal: 135	152	128	181	219	327	BlastAnno: 1766	UniRef50_Q52T38	620	68.09	470	137	3	80	1483	130	588	0.0	641
ABCDEF_41288	UniRef50_Q9SB58	0.0	Normal: 307	183	332	637	649	322	BlastAnno: 1403	UniRef50_Q9SB58	407	67.56	410	110	6	1401	184	15	405	0.0	523
ABCDEF_33461	UniRef50_Q9ZVN2	0.0	Normal: 142	84	140	206	283	119	BlastAnno: 2904	UniRef50_Q9ZVN2	704	60.98	574	201	6	2271	559	83	636	0.0	686
ABCDEF_14334	UniRef50_Q9SYZ0	0.0	Normal: 2263	129	1724	3469	866	283	BlastAnno: 2039	UniRef50_Q9SYZ0	493	63.22	484	171	2	369	1802	5	487	0.0	627
ABCDEF_24844	UniRef50_Q9FF86	0.0	Normal: 1857	3686	2657	21940	11467	4090	BlastAnno: 1640	UniRef50_Q9FF86	484	66.89	447	135	6	1492	185	2	446	0.0	575
ABCDEF_51190	UniRef50_Q52T38	0.0	Normal: 145	169	142	210	232	335	BlastAnno: 1891	UniRef50_Q52T38	620	70.04	514	141	3	73	1608	86	588	0.0	726
ABCDEF_14085	UniRef50_Q8L5Y5	0.0	Normal: 353	41	384	527	331	280	BlastAnno: 2362	UniRef50_Q8L5Y5	718	62.07	704	220	12	2360	348	24	713	0.0	676
ABCDEF_33458	UniRef50_Q9LW26	0.0	Normal: 158	78	130	240	342	106	BlastAnno: 2822	UniRef50_Q9LW26	701	46.72	655	302	5	400	2337	85	701	0.0	568
ABCDEF_25193	UniRef50_Q9C6L5	0.0	Normal: 3256	368	3548	6230	5109	1288	BlastAnno: 1786	UniRef50_Q9C6L5	492	65.83	480	156	4	1668	241	17	492	0.0	668
ABCDEF_33457	UniRef50_Q9ZVN2	0.0	Normal: 168	83	137	250	375	112	BlastAnno: 2823	UniRef50_Q9ZVN2	704	65.12	496	168	3	379	1860	83	575	0.0	648
ABCDEF_7634	UniRef50_Q9C6L5	0.0	Normal: 2543	9303	2711	11578	5533	10032	BlastAnno: 2064	UniRef50_Q9C6L5	492	52.76	489	225	3	1734	268	1	483	0.0	535
ABCDEF_299	UniRef50_Q71N54	0.0	Normal: 569	1147	511	900	649	1879	BlastAnno: 2017	UniRef50_Q71N54	535	61.73	533	198	5	338	1924	4	534	0.0	677
ABCDEF_27086	UniRef50_Q52T38	0.0	Normal: 784	470	617	843	893	976	BlastAnno: 2398	UniRef50_Q52T38	620	70.80	637	165	4	225	2129	1	618	0.0	887
ABCDEF_51188	UniRef50_Q52T38	0.0	Normal: 273	327	255	415	349	441	BlastAnno: 2324	UniRef50_Q52T38	620	71.30	568	150	3	2041	344	32	588	0.0	815
ABCDEF_44812	UniRef50_Q6DR03	0.0	Normal: 982	902	1005	1071	959	2999	BlastAnno: 2450	UniRef50_Q6DR03	565	54.97	644	198	17	2142	220	1	555	0.0	540
2.4.1.-(Transferases;)																					
ABCDEF_18004	UniRef50_Q6YWK8	0.0	Normal: 1562	1736	2009	3452	4438	2400	BlastAnno: 2298	UniRef50_Q6YWK8	570	65.27	429	136	1	1941	694	57	485	0.0	608
ABCDEF_52379	UniRef50_Q9LHZ7	0.0	Normal: 4015	4921	4546	10520	13430	4731	BlastAnno: 3479	UniRef50_Q9LHZ7	1170	48.04	893	342	16	825	3242	299	1156	0.0	787
ABCDEF_48593	UniRef50_Q9SK82	0.0	Normal: 12	420	19	343	763	253	BlastAnno: 1834	UniRef50_Q9SK82	489	60.08	481	186	4	1714	278	10	486	0.0	633
ABCDEF_13375	UniRef50_Q8VZK9	0.0	Normal: 108	123	208	526	34	294	BlastAnno: 1738	UniRef50_Q8VZK9	729	53.62	552	234	8	94	1737	18	551	0.0	582
ABCDEF_40982	UniRef50_Q96301	0.0	Normal: 1037	706	819	1249	1084	920	BlastAnno: 3418	UniRef50_Q96301	914	79.60	902	173	3	243	2948	1	891	0.0	1506
ABCDEF_47674	UniRef50_Q9LHZ7	0.0	Normal: 274	92	202	599	505	280	BlastAnno: 2526	UniRef50_Q9LHZ7	1170	75.93	777	151	8	2260	2	15	779	0.0	1177
ABCDEF_34097	UniRef50_Q9LE59	0.0	Normal: 626	613	923	1797	1829	692	BlastAnno: 2521	UniRef50_Q9LE59	673	60.92	458	178	1	1687	317	215	672	0.0	630
ABCDEF_34098	UniRef50_Q9LE59	0.0	Normal: 457	464	697	1283	1312	539	BlastAnno: 2199	UniRef50_Q9LE59	673	60.92	458	178	1	1687	317	215	672	0.0	628
ABCDEF_10848	UniRef50_Q9M156	0.0	Normal: 157	2988	163	408	772	1566	BlastAnno: 1810	UniRef50_Q9M156	480	60.09	456	176	4	1700	336	4	454	0.0	553
ABCDEF_41182	UniRef50_Q9LHZ7	0.0	Normal: 150	2169	221	814	1407	495	BlastAnno: 3604	UniRef50_Q9LHZ7	1170	51.03	870	333	15	2822	456	299	1156	0.0	826
ABCDEF_40981	UniRef50_Q96301	0.0	Normal: 156	97	110	160	179	173	BlastAnno: 1225	UniRef50_Q96301	914	86.92	344	45	0	1112	81	394	737	0.0	652
ABCDEF_9506	UniRef50_Q9LHZ7	0.0	Normal: 1302	2128	1494	3356	4644	2206	BlastAnno: 3751	UniRef50_Q9LHZ7	1170	49.38	891	343	17	927	3359	299	1161	0.0	812
ABCDEF_9500	UniRef50_Q9LHZ7	0.0	Normal: 183	188	186	576	498	260	BlastAnno: 1717	UniRef50_Q9LHZ7	1170	47.98	644	234	14	3	1715	418	1033	0.0	571
ABCDEF_100	UniRef50_Q9M8Y0	0.0	Normal: 228	309	224	416	461	141	BlastAnno: 2109	UniRef50_Q9M8Y0	977	77.49	533	120	0	1610	12	50	582	0.0	884
ABCDEF_33659	UniRef50_Q9LHZ7	0.0	Normal: 1282	1948	2248	5826	10642	2083	BlastAnno: 4289	UniRef50_Q9LHZ7	1170	48.70	887	345	15	2955	538	299	1156	0.0	788
ABCDEF_37667	UniRef50_Q9LHZ7	0.0	Normal: 248	3617	251	1237	2078	970	BlastAnno: 3161	UniRef50_Q9LHZ7	1170	49.72	885	335	14	2671	260	301	1156	0.0	810
ABCDEF_47673	UniRef50_Q9LHZ7	0.0	Normal: 876	296	828	1628	1721	1172	BlastAnno: 3878	UniRef50_Q9LHZ7	1170	80.88	1161	186	8	3642	232	15	1163	0.0	1865
ABCDEF_99	UniRef50_Q9M8Y0	0.0	Normal: 272	330	222	398	423	145	BlastAnno: 2094	UniRef50_Q9M8Y0	977	77.36	561	127	0	1683	1	50	610	0.0	929
ABCDEF_20658	UniRef50_Q8VZK9	0.0	Normal: 373	33	193	64	503	141	BlastAnno: 2316	UniRef50_Q8VZK9	729	53.35	508	221	7	47	1564	11	504	0.0	559
ABCDEF_10183	UniRef50_A7XDQ9	0.0	Normal: 422	455	346	577	378	610	BlastAnno: 2362	UniRef50_A7XDQ9	684	50.94	693	307	12	281	2287	1	684	0.0	670
ABCDEF_30745	UniRef50_A7XDQ9	0.0	Normal: 1209	351	1031	1645	1226	635	BlastAnno: 2646	UniRef50_A7XDQ9	684	57.58	554	227	6	546	2189	133	684	0.0	664
ABCDEF_13336	UniRef50_Q7PC69	0.0	Normal: 148	563	194	957	1575	816	BlastAnno: 2584	UniRef50_Q7PC69	745	71.56	559	136	6	744	2351	187	745	0.0	730
ABCDEF_21426	UniRef50_Q8RU27	0.0	Normal: 2658	1979	3417	6157	9464	2599	BlastAnno: 1530	UniRef50_Q8RU27	366	89.44	360	34	2	1358	282	1	357	0.0	667
ABCDEF_71242	UniRef50_Q9ZV71	0.0	Normal: 134	122	179	480	754	119	BlastAnno: 1835	UniRef50_Q9ZV71	409	73.35	409	103	4	231	1439	1	409	0.0	587
ABCDEF_52020	UniRef50_Q6YWK8	0.0	Normal: 1635	1534	1975	4284	10353	2289	BlastAnno: 2394	UniRef50_Q6YWK8	570	58.37	514	185	2	403	1857	57	570	0.0	607
ABCDEF_51269	UniRef50_Q6YWK8	0.0	Normal: 147	85	103	21	33	438	BlastAnno: 1864	UniRef50_Q6YWK8	570	54.28	514	204	2	1646	198	57	570	0.0	586
ABCDEF_30753	UniRef50_Q9ZV71	0.0	Normal: 290	497	417	629	1013	634	BlastAnno: 1502	UniRef50_Q9ZV71	409	72.32	401	107	2	311	1501	1	401	0.0	590
ABCDEF_4233	UniRef50_Q9ZV71	0.0	Normal: 369	412	550	936	860	750	BlastAnno: 2004	UniRef50_Q9ZV71	409	72.13	409	110	2	370	1584	1	409	0.0	613
ABCDEF_17525	UniRef50_Q8RU27	0.0	Normal: 1500	1289	2149	3711	4446	1069	BlastAnno: 1507	UniRef50_Q8RU27	366	91.35	347	30	0	1371	331	3	349	0.0	671

ABCDEF_18005	UniRef50_Q6YWK8	0.0	Normal: 1842	2042	2515	4214	5208	2842	BlastAnno: 2305	UniRef50_Q6YWK8	570	59.14	514	180	2	1948	497	57	570	0.0	642
ABCDEF_41140	UniRef50_Q6YWK8	0.0	Normal: 86	1314	93	753	1787	2076	BlastAnno: 2346	UniRef50_Q6YWK8	570	60.00	510	174	2	431	1870	57	566	0.0	669
ABCDEF_18002	UniRef50_Q9LHZ7	0.0	Normal: 376	127	367	601	1190	520	BlastAnno: 1605	UniRef50_Q9LHZ7	1170	89.75	400	41	0	1603	404	771	1170	0.0	732
ABCDEF_13339	UniRef50_Q7PC69	0.0	Normal: 127	814	183	541	1135	1144	BlastAnno: 2610	UniRef50_Q7PC69	745	70.48	559	141	6	725	2329	187	745	0.0	729
ABCDEF_41141	UniRef50_Q6YWK8	0.0	Normal: 67	1107	84	708	1690	1854	BlastAnno: 2461	UniRef50_Q6YWK8	570	60.00	510	174	2	1916	477	57	566	0.0	668
ABCDEF_44583	UniRef50_Q9LE59	0.0	Normal: 681	561	559	947	1745	789	BlastAnno: 2470	UniRef50_Q9LE59	673	59.21	456	185	1	1749	385	214	669	0.0	621
ABCDEF_3869	UniRef50_Q9LE59	0.0	Normal: 333	393	415	809	907	754	BlastAnno: 2136	UniRef50_Q9LE59	673	59.05	613	227	4	1827	1	81	673	0.0	699
ABCDEF_28947	UniRef50_Q9SK82	0.0	Normal: 0	851	3	0	0	116	BlastAnno: 1567	UniRef50_Q9SK82	489	62.40	484	176	4	1524	85	1	482	0.0	652
ABCDEF_31119	UniRef50_Q9M8Y0	0.0	Normal: 2212	1279	1917	2933	3341	1803	BlastAnno: 3695	UniRef50_Q9M8Y0	977	81.08	925	174	1	392	3163	50	974	0.0	1583
ABCDEF_37311	UniRef50_Q9LE59	0.0	Normal: 175	133	191	549	421	366	BlastAnno: 1612	UniRef50_Q9LE59	673	62.96	486	153	4	1406	3	4	480	0.0	542
ABCDEF_24190	UniRef50_Q8L7F9	0.0	Normal: 397	265	492	583	553	371	BlastAnno: 2564	UniRef50_Q8L7F9	643	63.91	640	218	4	2353	473	1	640	0.0	850
ABCDEF_30548	UniRef50_Q9FH36	0.0	Normal: 17	916	27	151	420	126	BlastAnno: 1963	UniRef50_Q9FH36	535	72.47	534	138	3	102	1676	1	534	0.0	767
ABCDEF_27416	UniRef50_Q7PC69	0.0	Normal: 575	837	875	1344	2015	936	BlastAnno: 2454	UniRef50_Q7PC69	745	66.11	416	115	4	2337	1168	126	541	0.0	518
ABCDEF_48373	UniRef50_A7XDQ9	0.0	Normal: 1364	773	1336	1451	1791	1202	BlastAnno: 3042	UniRef50_A7XDQ9	684	63.68	691	234	6	2405	360	1	683	0.0	915
ABCDEF_3868	UniRef50_Q9LE59	0.0	Normal: 224	274	301	555	686	524	BlastAnno: 1670	UniRef50_Q9LE59	673	65.31	516	176	1	1556	9	161	673	0.0	696
ABCDEF_31288	UniRef50_Q940V3	0.0	Normal: 247	309	183	101	124	655	BlastAnno: 1658	UniRef50_Q940V3	470	56.01	466	197	7	1614	232	8	470	0.0	537
ABCDEF_52574	UniRef50_Q09332	0.0	Normal: 885	474	1068	1835	1853	816	BlastAnno: 3720	UniRef50_Q09332	1548	47.89	687	310	14	2551	533	859	1511	0.0	598
ABCDEF_79752	UniRef50_Q9ZV71	0.0	Normal: 158	183	245	400	379	352	BlastAnno: 1266	UniRef50_Q9ZV71	409	71.39	360	99	2	31	1098	1	360	0.0	526
ABCDEF_9509	UniRef50_Q9LHZ7	0.0	Normal: 1296	2171	1498	3410	4683	2232	BlastAnno: 3897	UniRef50_Q9LHZ7	1170	49.38	891	343	17	1073	3505	299	1161	0.0	812
ABCDEF_48200	UniRef50_Q6YWK8	0.0	Normal: 1768	576	3156	4431	6634	491	BlastAnno: 2274	UniRef50_Q6YWK8	570	57.98	514	185	4	291	1739	57	570	0.0	642
ABCDEF_24155	UniRef50_Q9ZVI7	0.0	Normal: 503	247	607	1676	1833	578	BlastAnno: 2708	UniRef50_Q9ZVI7	619	52.33	621	264	11	306	2123	15	618	0.0	601
ABCDEF_21246	UniRef50_Q9SKT6	0.0	Normal: 92	107	188	380	834	84	BlastAnno: 1791	UniRef50_Q9SKT6	536	56.61	484	204	5	123	1562	52	533	0.0	568
ABCDEF_32317	UniRef50_Q8RU27	0.0	Normal: 12	9	3	118	800	62	BlastAnno: 1076	UniRef50_Q8RU27	366	87.02	285	37	0	1075	221	64	348	0.0	535
ABCDEF_9502	UniRef50_Q9LHZ7	0.0	Normal: 610	486	568	1331	1403	591	BlastAnno: 1961	UniRef50_Q9LHZ7	1170	48.64	625	249	13	3	1742	564	1161	0.0	570
ABCDEF_23298	UniRef50_Q9FH36	0.0	Normal: 128	527	130	223	314	864	BlastAnno: 2832	UniRef50_Q9FH36	535	83.16	196	33	0	696	1283	340	535	0.0	357
ABCDEF_40595	UniRef50_O80898	0.0	Normal: 229	135	103	25	307	325	BlastAnno: 2413	UniRef50_O80898	757	44.68	761	378	13	95	2299	10	753	0.0	587
ABCDEF_63947	UniRef50_Q9SK82	0.0	Normal: 0	267	0	0	71	0	BlastAnno: 1446	UniRef50_Q9SK82	489	58.33	468	189	4	60	1445	1	468	0.0	592
ABCDEF_67847	UniRef50_Q9M8Y0	0.0	Normal: 794	476	871	1081	1401	868	BlastAnno: 1591	UniRef50_Q9M8Y0	977	84.86	370	56	0	1589	480	605	974	0.0	685
ABCDEF_65666	UniRef50_Q09332	0.0	Normal: 800	441	946	1606	1637	735	BlastAnno: 3518	UniRef50_Q09332	1548	47.54	671	305	13	1162	3087	859	1511	0.0	586
ABCDEF_38641	UniRef50_Q9FWA4	0.0	Normal: 674	967	1162	2603	4404	2475	BlastAnno: 1599	UniRef50_Q9FWA4	561	81.35	386	72	0	1	1158	176	561	0.0	641
ABCDEF_17914	UniRef50_Q9SKT6	0.0	Normal: 784	567	886	1447	1439	1424	BlastAnno: 2049	UniRef50_Q9SKT6	536	72.95	536	140	2	204	1796	1	536	0.0	785
ABCDEF_18286	UniRef50_Q6NQB7	0.0	Normal: 1131	1108	1225	1872	1722	3329	BlastAnno: 1752	UniRef50_Q6NQB7	393	73.80	397	81	6	1527	367	10	393	0.0	607
ABCDEF_119	UniRef50_Q8RU27	0.0	Normal: 1613	1155	2249	3558	7663	1775	BlastAnno: 1532	UniRef50_Q8RU27	366	88.33	360	38	2	1306	230	1	357	0.0	663
ABCDEF_47603	UniRef50_Q9M9Y5	0.0	Normal: 2609	8779	1664	1103	2085	22991	BlastAnno: 2830	UniRef50_Q9M9Y5	589	54.04	594	267	3	2475	694	1	588	0.0	654
ABCDEF_24192	UniRef50_Q8L7F9	0.0	Normal: 391	244	465	553	497	345	BlastAnno: 2477	UniRef50_Q8L7F9	643	58.99	434	165	4	212	1474	1	434	0.0	513
ABCDEF_17482	UniRef50_Q9SKT6	0.0	Normal: 615	379	816	1068	1470	852	BlastAnno: 2083	UniRef50_Q9SKT6	536	71.11	540	147	4	1915	311	1	536	0.0	786
ABCDEF_85909	UniRef50_Q8VZK9	0.0	Normal: 28	45	9	10	44	127	BlastAnno: 1473	UniRef50_Q8VZK9	729	55.37	475	198	6	1419	1	11	473	0.0	536
ABCDEF_9503	UniRef50_Q9LHZ7	0.0	Normal: 487	544	524	1242	1320	833	BlastAnno: 2087	UniRef50_Q9LHZ7	1170	50.35	574	236	8	57	1712	615	1161	0.0	567
ABCDEF_40594	UniRef50_O80898	0.0	Normal: 256	127	102	41	331	326	BlastAnno: 2417	UniRef50_O80898	757	45.76	719	348	12	213	2291	51	753	0.0	585
ABCDEF_17141	UniRef50_Q9LHZ7	0.0	Normal: 74	1734	69	380	701	333	BlastAnno: 3337	UniRef50_Q9LHZ7	1170	50.92	868	335	14	2539	179	299	1156	0.0	816
ABCDEF_33658	UniRef50_Q9LHZ7	0.0	Normal: 384	308	395	805	1003	602	BlastAnno: 2961	UniRef50_Q9LHZ7	1170	48.76	888	343	14	204	2621	299	1156	0.0	792
ABCDEF_20579	UniRef50_Q7PC69	0.0	Normal: 1007	795	1238	2311	2309	1345	BlastAnno: 3113	UniRef50_Q7PC69	745	59.97	767	229	14	2667	535	1	745	0.0	768
ABCDEF_31118	UniRef50_Q9M8Y0	0.0	Normal: 2135	1267	1900	3004	3329	1757	BlastAnno: 3787	UniRef50_Q9M8Y0	977	81.10	709	133	1	1132	3255	266	974	0.0	1217
ABCDEF_9499	UniRef50_Q9LHZ7	0.0	Normal: 205	207	215	632	538	275	BlastAnno: 1794	UniRef50_Q9LHZ7	1170	48.88	667	240	14	11	1792	395	1033	0.0	605
ABCDEF_48199	UniRef50_Q6YWK8	0.0	Normal: 1506	467	2429	3094	4233	439	BlastAnno: 1997	UniRef50_Q6YWK8	570	63.10	439	146	2	291	1559	57	495	0.0	610
ABCDEF_40983	UniRef50_Q96301	0.0	Normal: 406	261	343	392	415	433	BlastAnno: 2265	UniRef50_Q96301	914	79.17	504	99	1	114	1625	394	891	0.0	854
ABCDEF_17140	UniRef50_Q9LHZ7	0.0	Normal: 45	1049	49	216	386	208	BlastAnno: 2293	UniRef50_Q9LHZ7	1170	52.34	770	281	13	2138	57	397	1156	0.0	739
ABCDEF_18352	UniRef50_Q9FWA4	0.0	Normal: 2427	2688	2969	5519	6547	4855	BlastAnno: 2224	UniRef50_Q9FWA4	561	69.31	505	148	3	1887	382	61	561	0.0	683
ABCDEF_27767	UniRef50_Q9LHZ7	0.0	Normal: 2576	5141	2841	10249	14583	5031	BlastAnno: 3981	UniRef50_Q9LHZ7	1170	49.66	888	337	16	2927	504	299	1156	0.0	818
ABCDEF_33661	UniRef50_Q9LHZ7	0.0	Normal: 414	340	507	879	790	783	BlastAnno: 2185	UniRef50_Q9LHZ7	1170	48.66	748	277	14	2184	169	440	1156	0.0	662
ABCDEF_14685	UniRef50_A7XDQ9	0.0	Normal: 233	308	134	573	292	334	BlastAnno: 1947	UniRef50_A7XDQ9	684	56.84	468	186	6	569	1945	1	461	0.0	545
ABCDEF_20660	UniRef50_Q8VZK9	0.0	Normal: 22	48	13	15	52	144	BlastAnno: 1473	UniRef50_Q8VZK9	729	55.16	475	199	6	55	1473	11	473	0.0	537
ABCDEF_21315	UniRef50_Q9LHZ7	0.0	Normal: 3412	252	1450	0	79	1228	BlastAnno: 3765	UniRef50_Q9LHZ7	1170	63.78	1096	340	15	308	3508	70	1137	0.0	1333
ABCDEF_533	UniRef50_Q9C8W3	0.0	Normal: 230	204	269	505	411	301	BlastAnno: 1688	UniRef50_Q9C8W3	401	70.33	364	94	2	393	1475	47	399	0.0	555
ABCDEF_34099	UniRef50_Q9LE59	0.0	Normal: 478	491	735	1356	1350	548	BlastAnno: 2158	UniRef50_Q9LE59	673	60.92	458	178	1	1610	240	215	672	0.0	630
ABCDEF_47984	UniRef50_Q7PC69	0.0	Normal: 663	457	734	1362	2147	696	BlastAnno: 2838	UniRef50_Q7PC69	745	59.27	712	226	9	373	2316	34	745	0.0	702
ABCDEF_51451	UniRef50_Q9LE59	0.0	Normal: 1883	1782	2202	4337	4350	2667	BlastAnno: 2874	UniRef50_Q9LE59	673	74.16	685	165	6	2559	505	1	673	0.0	982
ABCDEF_13370	UniRef50_Q8VZK9	0.0	Normal: 290	87	317	377	105	258	BlastAnno: 2033	UniRef50_Q8VZK9	729	51.65	635	278	11	2031	154	112	726	0.0	612
ABCDEF_48239	UniRef50_Q9LHZ7	0.0	Normal: 511	163	422	1154	1316	510	BlastAnno: 2714	UniRef50_Q9LHZ7	1170	76.20	769	156	9	478	2712	41	806	0.0	1201
ABCDEF_13338	UniRef50_Q7PC69	0.0	Normal: 335	468	417	2639	4443	616	BlastAnno: 2525	UniRef50_Q7PC69	745	62.45	727	209	12	298	2292	21	745	0.0	781
ABCDEF_24193	UniRef50_Q8L7F9	0.0	Normal: 113	72	132	203	180	119	BlastAnno: 1679	UniRef50_Q8L7F9	643	60.54	479	176	4	212	1609	1	479	0.0	592
ABCDEF_48514	UniRef50_Q9ZV71	0.0	Normal: 540	580	601	771	1033	926	BlastAnno: 1920	UniRef50_Q9ZV71	409	78.14	398	85	2	1618	431	12	409	0.0	637

2.5.1.-(Transferases;)

ABCDEF_48175	UniRef50_P72580	2e-123	Normal: 366	927	282	531	590	1811	BlastAnno: 1917	UniRef50_P72580	323	58.44	320	130	2	610	1569	6	322	2e-123	377

2.5.1.32(phytoene synthase;)

ABCDEF_11297	UniRef50_P08196	0.0	Normal: 1327	3381	743	814	2119	12185	BlastAnno: 2134	UniRef50_P08196	412	76.21	433	79	3	1561	263	1	409	0.0	650

5.-.-.-(Isomerases)

ABCDEF_37668	UniRef50_A8DJY4	1e-109	Normal: 240	328	262	459	844	1032	BlastAnno: 2373	UniRef50_A8DJY4	512	37.90	504	291	6	1825	359	12	508	1e-109	353

5.3.99.8(capsanthin/capsorubin synthase;)

No Correspond Unigene

5.3.99.9(neoxanthin synthase;)

No Correspond Unigene

>00908 Zeatin biosynthesis

1.3.1.69(zeatin reductase)

No Correspond Unigene

1.5.99.12(cytokinin dehydrogenase;)

ABCDEF_21247	UniRef50_Q9FUJ1	0.0	Normal: 130	23	127	167	83	68	BlastAnno: 1487	UniRef50_Q9FUJ1	524	66.27	421	135	4	3	1247	104	523	0.0	569
ABCDEF_25439	UniRef50_O22213	0.0	Normal: 0	67	0	0	0	680	BlastAnno: 1827	UniRef50_O22213	575	64.43	537	177	5	1745	168	10	543	0.0	735
ABCDEF_18740	UniRef50_O22213	0.0	Normal: 88	1155	17	0	15	61	BlastAnno: 2112	UniRef50_O22213	575	63.87	537	175	3	1837	281	11	546	0.0	698
ABCDEF_18739	UniRef50_O22213	0.0	Normal: 37	596	7	0	4	33	BlastAnno: 1577	UniRef50_O22213	575	64.72	428	132	3	1302	73	11	437	0.0	551
ABCDEF_5276	UniRef50_Q67YU0	0.0	Normal: 0	16	1	0	76	602	BlastAnno: 1434	UniRef50_Q67YU0	540	76.37	402	92	1	2	1207	122	520	0.0	655

2.4.1.-(Transferases;)

ABCDEF_18004	UniRef50_Q6YWK8	0.0	Normal: 1562	1736	2009	3452	4438	2400	BlastAnno: 2298	UniRef50_Q6YWK8	570	65.27	429	136	1	1941	694	57	485	0.0	608
ABCDEF_52379	UniRef50_Q9LHZ7	0.0	Normal: 4015	4921	4546	10520	13430	4731	BlastAnno: 3479	UniRef50_Q9LHZ7	1170	48.04	893	342	16	825	3242	299	1156	0.0	787
ABCDEF_48593	UniRef50_Q9SK82	0.0	Normal: 12	420	19	343	763	253	BlastAnno: 1834	UniRef50_Q9SK82	489	60.08	481	186	4	1714	278	10	486	0.0	633
ABCDEF_13375	UniRef50_Q8VZK9	0.0	Normal: 108	123	208	526	34	294	BlastAnno: 1738	UniRef50_Q8VZK9	729	53.62	552	234	8	94	1737	18	551	0.0	582
ABCDEF_40982	UniRef50_Q96301	0.0	Normal: 1037	706	819	1249	1084	920	BlastAnno: 3418	UniRef50_Q96301	914	79.60	902	173	3	243	2948	1	891	0.0	1506
ABCDEF_47674	UniRef50_Q9LHZ7	0.0	Normal: 274	92	202	599	505	280	BlastAnno: 2526	UniRef50_Q9LHZ7	1170	75.93	777	151	8	2260	2	15	779	0.0	1177
ABCDEF_34097	UniRef50_Q9LE59	0.0	Normal: 626	613	923	1797	1829	692	BlastAnno: 2521	UniRef50_Q9LE59	673	60.92	458	178	1	1687	317	215	672	0.0	630
ABCDEF_34098	UniRef50_Q9LE59	0.0	Normal: 457	464	697	1283	1312	539	BlastAnno: 2199	UniRef50_Q9LE59	673	60.92	458	178	1	1687	317	215	672	0.0	628
ABCDEF_10848	UniRef50_Q9M156	0.0	Normal: 157	2988	163	408	772	1566	BlastAnno: 1810	UniRef50_Q9M156	480	60.09	456	176	4	1700	336	4	454	0.0	553
ABCDEF_41182	UniRef50_Q9LHZ7	0.0	Normal: 150	2169	221	814	1407	495	BlastAnno: 3604	UniRef50_Q9LHZ7	1170	51.03	870	333	15	2822	456	299	1156	0.0	826
ABCDEF_40981	UniRef50_Q96301	0.0	Normal: 156	97	110	160	179	173	BlastAnno: 1225	UniRef50_Q96301	914	86.92	344	45	0	1112	81	394	737	0.0	652
ABCDEF_9506	UniRef50_Q9LHZ7	0.0	Normal: 1302	2128	1494	3356	4644	2206	BlastAnno: 3751	UniRef50_Q9LHZ7	1170	49.38	891	343	17	927	3359	299	1161	0.0	812
ABCDEF_9500	UniRef50_Q9LHZ7	0.0	Normal: 183	188	186	576	498	260	BlastAnno: 1717	UniRef50_Q9LHZ7	1170	47.98	644	234	14	3	1715	418	1033	0.0	571
ABCDEF_100	UniRef50_Q9M8Y0	0.0	Normal: 228	309	224	416	461	141	BlastAnno: 2109	UniRef50_Q9M8Y0	977	77.49	533	120	0	1610	12	50	582	0.0	884
ABCDEF_33659	UniRef50_Q9LHZ7	0.0	Normal: 1282	1948	2248	5826	10642	2083	BlastAnno: 4289	UniRef50_Q9LHZ7	1170	48.70	887	345	15	2955	538	299	1156	0.0	788
ABCDEF_37667	UniRef50_Q9LHZ7	0.0	Normal: 248	3617	251	1237	2078	970	BlastAnno: 3161	UniRef50_Q9LHZ7	1170	49.72	885	335	14	2671	260	301	1156	0.0	810
ABCDEF_47673	UniRef50_Q9LHZ7	0.0	Normal: 876	296	828	1628	1721	1172	BlastAnno: 3878	UniRef50_Q9LHZ7	1170	80.88	1161	186	8	3642	232	15	1163	0.0	1865
ABCDEF_99	UniRef50_Q9M8Y0	0.0	Normal: 272	330	222	398	423	145	BlastAnno: 2094	UniRef50_Q9M8Y0	977	77.36	561	127	0	1683	1	50	610	0.0	929
ABCDEF_20658	UniRef50_Q8VZK9	0.0	Normal: 373	33	193	64	503	141	BlastAnno: 2316	UniRef50_Q8VZK9	729	53.35	508	221	7	47	1564	11	504	0.0	559
ABCDEF_10183	UniRef50_A7XDQ9	0.0	Normal: 422	455	346	577	378	610	BlastAnno: 2362	UniRef50_A7XDQ9	684	50.94	693	307	12	281	2287	1	684	0.0	670
ABCDEF_30745	UniRef50_A7XDQ9	0.0	Normal: 1209	351	1031	1645	1226	635	BlastAnno: 2646	UniRef50_A7XDQ9	684	57.58	554	227	6	546	2189	133	684	0.0	664
ABCDEF_13336	UniRef50_Q7PC69	0.0	Normal: 148	563	194	957	1575	816	BlastAnno: 2584	UniRef50_Q7PC69	745	71.56	559	136	6	744	2351	187	745	0.0	730
ABCDEF_21426	UniRef50_Q8RU27	0.0	Normal: 2658	1979	3417	6157	9464	2599	BlastAnno: 1530	UniRef50_Q8RU27	366	89.44	360	34	2	1358	282	1	357	0.0	667
ABCDEF_71242	UniRef50_Q9ZV71	0.0	Normal: 134	122	179	480	754	119	BlastAnno: 1835	UniRef50_Q9ZV71	409	73.35	409	103	4	231	1439	1	409	0.0	587
ABCDEF_52020	UniRef50_Q6YWK8	0.0	Normal: 1635	1534	1975	4284	10353	2289	BlastAnno: 2394	UniRef50_Q6YWK8	570	58.37	514	185	2	403	1857	57	570	0.0	607
ABCDEF_51269	UniRef50_Q6YWK8	0.0	Normal: 147	85	103	21	33	438	BlastAnno: 1864	UniRef50_Q6YWK8	570	54.28	514	204	2	1646	198	57	570	0.0	586
ABCDEF_30753	UniRef50_Q9ZV71	0.0	Normal: 290	497	417	629	1013	634	BlastAnno: 1502	UniRef50_Q9ZV71	409	72.32	401	107	2	311	1501	1	401	0.0	590
ABCDEF_4233	UniRef50_Q9ZV71	0.0	Normal: 369	412	550	936	860	750	BlastAnno: 2004	UniRef50_Q9ZV71	409	72.13	409	110	2	370	1584	1	409	0.0	613
ABCDEF_17525	UniRef50_Q8RU27	0.0	Normal: 1500	1289	2149	3711	4446	1069	BlastAnno: 1507	UniRef50_Q8RU27	366	91.35	347	30	0	1371	331	3	349	0.0	671
ABCDEF_18005	UniRef50_Q6YWK8	0.0	Normal: 1842	2042	2515	4214	5208	2842	BlastAnno: 2305	UniRef50_Q6YWK8	570	59.14	514	180	2	1948	497	57	570	0.0	642
ABCDEF_41140	UniRef50_Q6YWK8	0.0	Normal: 86	1314	93	753	1787	2076	BlastAnno: 2346	UniRef50_Q6YWK8	570	60.00	510	174	2	431	1870	57	566	0.0	669
ABCDEF_18002	UniRef50_Q9LHZ7	0.0	Normal: 376	127	367	601	1190	520	BlastAnno: 1605	UniRef50_Q9LHZ7	1170	89.75	400	41	0	1603	404	771	1170	0.0	732
ABCDEF_13339	UniRef50_Q7PC69	0.0	Normal: 127	814	183	541	1135	1144	BlastAnno: 2610	UniRef50_Q7PC69	745	70.48	559	141	6	725	2329	187	745	0.0	729
ABCDEF_41141	UniRef50_Q6YWK8	0.0	Normal: 67	1107	84	708	1690	1854	BlastAnno: 2461	UniRef50_Q6YWK8	570	60.00	510	174	2	1916	477	57	566	0.0	668
ABCDEF_44583	UniRef50_Q9LE59	0.0	Normal: 681	561	559	947	1745	789	BlastAnno: 2470	UniRef50_Q9LE59	673	59.21	456	185	1	1749	385	214	669	0.0	621
ABCDEF_3869	UniRef50_Q9LE59	0.0	Normal: 333	393	415	809	907	754	BlastAnno: 2136	UniRef50_Q9LE59	673	59.05	613	227	4	1827	1	81	673	0.0	699
ABCDEF_28947	UniRef50_Q9SK82	0.0	Normal: 0	851	3	0	0	116	BlastAnno: 1567	UniRef50_Q9SK82	489	62.40	484	176	4	1524	85	1	482	0.0	652
ABCDEF_31119	UniRef50_Q9M8Y0	0.0	Normal: 2212	1279	1917	2933	3341	1803	BlastAnno: 3695	UniRef50_Q9M8Y0	977	81.08	925	174	1	392	3163	50	974	0.0	1583
ABCDEF_37311	UniRef50_Q9LE59	0.0	Normal: 175	133	191	549	421	366	BlastAnno: 1612	UniRef50_Q9LE59	673	62.96	486	153	4	1406	3	4	480	0.0	542
ABCDEF_24190	UniRef50_Q8L7F9	0.0	Normal: 397	265	492	583	553	371	BlastAnno: 2564	UniRef50_Q8L7F9	643	63.91	640	218	4	2353	473	1	640	0.0	850
ABCDEF_30548	UniRef50_Q9FH36	0.0	Normal: 17	916	27	151	420	126	BlastAnno: 1963	UniRef50_Q9FH36	535	72.47	534	138	3	102	1676	1	534	0.0	767
ABCDEF_27416	UniRef50_Q7PC69	0.0	Normal: 575	837	875	1344	2015	936	BlastAnno: 2454	UniRef50_Q7PC69	745	66.11	416	115	4	2337	1168	126	541	0.0	518
ABCDEF_48373	UniRef50_A7XDQ9	0.0	Normal: 1364	773	1336	1451	1791	1202	BlastAnno: 3042	UniRef50_A7XDQ9	684	63.68	691	234	6	2405	360	1	683	0.0	915
ABCDEF_3868	UniRef50_Q9LE59	0.0	Normal: 224	274	301	555	686	524	BlastAnno: 1670	UniRef50_Q9LE59	673	65.31	516	176	1	1556	9	161	673	0.0	696
ABCDEF_31288	UniRef50_Q940V3	0.0	Normal: 247	309	183	101	124	655	BlastAnno: 1658	UniRef50_Q940V3	470	56.01	466	197	7	1614	232	8	470	0.0	537
ABCDEF_52574	UniRef50_Q09332	0.0	Normal: 885	474	1068	1835	1853	816	BlastAnno: 3720	UniRef50_Q09332	1548	47.89	687	310	14	2551	533	859	1511	0.0	598
ABCDEF_79752	UniRef50_Q9ZV71	0.0	Normal: 158	183	245	400	379	352	BlastAnno: 1266	UniRef50_Q9ZV71	409	71.39	360	99	2	31	1098	1	360	0.0	526
ABCDEF_9509	UniRef50_Q9LHZ7	0.0	Normal: 1296	2171	1498	3410	4683	2232	BlastAnno: 3897	UniRef50_Q9LHZ7	1170	49.38	891	343	17	1073	3505	299	1161	0.0	812
ABCDEF_48200	UniRef50_Q6YWK8	0.0	Normal: 1768	576	3156	4431	6634	491	BlastAnno: 2274	UniRef50_Q6YWK8	570	57.98	514	185	4	291	1739	57	570	0.0	642
ABCDEF_24155	UniRef50_Q9ZVI7	0.0	Normal: 503	247	607	1676	1833	578	BlastAnno: 2708	UniRef50_Q9ZVI7	619	52.33	621	264	11	306	2123	15	618	0.0	601
ABCDEF_21246	UniRef50_Q9SKT6	0.0	Normal: 92	107	188	380	834	84	BlastAnno: 1791	UniRef50_Q9SKT6	536	56.61	484	204	5	123	1562	52	533	0.0	568
ABCDEF_32317	UniRef50_Q8RU27	0.0	Normal: 12	9	3	118	800	62	BlastAnno: 1076	UniRef50_Q8RU27	366	87.02	285	37	0	1075	221	64	348	0.0	535
ABCDEF_9502	UniRef50_Q9LHZ7	0.0	Normal: 610	486	568	1331	1403	591	BlastAnno: 1961	UniRef50_Q9LHZ7	1170	48.64	625	249	13	3	1742	564	1161	0.0	570
ABCDEF_23298	UniRef50_Q9FH36	0.0	Normal: 128	527	130	223	314	864	BlastAnno: 2832	UniRef50_Q9FH36	535	83.16	196	33	0	696	1283	340	535	0.0	357
ABCDEF_40595	UniRef50_O80898	0.0	Normal: 229	135	103	25	307	325	BlastAnno: 2413	UniRef50_O80898	757	44.68	761	378	13	95	2299	10	753	0.0	587
ABCDEF_63947	UniRef50_Q9SK82	0.0	Normal: 0	267	0	0	71	0	BlastAnno: 1446	UniRef50_Q9SK82	489	58.33	468	189	4	60	1445	1	468	0.0	592
ABCDEF_67847	UniRef50_Q9M8Y0	0.0	Normal: 794	476	871	1081	1401	868	BlastAnno: 1591	UniRef50_Q9M8Y0	977	84.86	370	56	0	1589	480	605	974	0.0	685
ABCDEF_65666	UniRef50_Q09332	0.0	Normal: 800	441	946	1606	1637	735	BlastAnno: 3518	UniRef50_Q09332	1548	47.54	671	305	13	1162	3087	859	1511	0.0	586
ABCDEF_38641	UniRef50_Q9FWA4	0.0	Normal: 674	967	1162	2603	4404	2475	BlastAnno: 1599	UniRef50_Q9FWA4	561	81.35	386	72	0	1	1158	176	561	0.0	641
ABCDEF_17914	UniRef50_Q9SKT6	0.0	Normal: 784	567	886	1447	1439	1424	BlastAnno: 2049	UniRef50_Q9SKT6	536	72.95	536	140	2	204	1796	1	536	0.0	785
ABCDEF_18286	UniRef50_Q6NQB7	0.0	Normal: 1131	1108	1225	1872	1722	3329	BlastAnno: 1752	UniRef50_Q6NQB7	393	73.80	397	81	6	1527	367	10	393	0.0	607
ABCDEF_119	UniRef50_Q8RU27	0.0	Normal: 1613	1155	2249	3558	7663	1775	BlastAnno: 1532	UniRef50_Q8RU27	366	88.33	360	38	2	1306	230	1	357	0.0	663

ABCDEF_47603	UniRef50_Q9M9Y5	0.0	Normal: 2609	8779	1664	1103	2085	22991	BlastAnno: 2830	UniRef50_Q9M9Y5	589	54.04	594	267	3	2475	694	1	588	0.0	654
ABCDEF_24192	UniRef50_Q8L7F9	0.0	Normal: 391	244	465	553	497	345	BlastAnno: 2477	UniRef50_Q8L7F9	643	58.99	434	165	4	212	1474	1	434	0.0	513
ABCDEF_17482	UniRef50_Q9SKT6	0.0	Normal: 615	379	816	1068	1470	852	BlastAnno: 2083	UniRef50_Q9SKT6	536	71.11	540	147	4	1915	311	1	536	0.0	786
ABCDEF_85909	UniRef50_Q8VZK9	0.0	Normal: 28	45	9	10	44	127	BlastAnno: 1473	UniRef50_Q8VZK9	729	55.37	475	198	6	1419	1	11	473	0.0	536
ABCDEF_9503	UniRef50_Q9LHZ7	0.0	Normal: 487	544	524	1242	1320	833	BlastAnno: 2087	UniRef50_Q9LHZ7	1170	50.35	574	236	8	57	1712	615	1161	0.0	567
ABCDEF_40594	UniRef50_O80898	0.0	Normal: 256	127	102	41	331	326	BlastAnno: 2417	UniRef50_O80898	757	45.76	719	348	12	213	2291	51	753	0.0	585
ABCDEF_17141	UniRef50_Q9LHZ7	0.0	Normal: 74	1734	69	380	701	333	BlastAnno: 3337	UniRef50_Q9LHZ7	1170	50.92	868	335	14	2539	179	299	1156	0.0	816
ABCDEF_33658	UniRef50_Q9LHZ7	0.0	Normal: 384	308	395	805	1003	602	BlastAnno: 2961	UniRef50_Q9LHZ7	1170	48.76	888	343	14	204	2621	299	1156	0.0	792
ABCDEF_20579	UniRef50_Q7PC69	0.0	Normal: 1007	795	1238	2311	2309	1345	BlastAnno: 3113	UniRef50_Q7PC69	745	59.97	767	229	14	2667	535	1	745	0.0	768
ABCDEF_31118	UniRef50_Q9M8Y0	0.0	Normal: 2135	1267	1900	3004	3329	1757	BlastAnno: 3787	UniRef50_Q9M8Y0	977	81.10	709	133	1	1132	3255	266	974	0.0	1217
ABCDEF_9499	UniRef50_Q9LHZ7	0.0	Normal: 205	207	215	632	538	275	BlastAnno: 1794	UniRef50_Q9LHZ7	1170	48.88	667	240	14	11	1792	395	1033	0.0	605
ABCDEF_48199	UniRef50_Q6YWK8	0.0	Normal: 1506	467	2429	3094	4233	439	BlastAnno: 1997	UniRef50_Q6YWK8	570	63.10	439	146	2	291	1559	57	495	0.0	610
ABCDEF_40983	UniRef50_Q96301	0.0	Normal: 406	261	343	392	415	433	BlastAnno: 2265	UniRef50_Q96301	914	79.17	504	99	1	114	1625	394	891	0.0	854
ABCDEF_17140	UniRef50_Q9LHZ7	0.0	Normal: 45	1049	49	216	386	208	BlastAnno: 2293	UniRef50_Q9LHZ7	1170	52.34	770	281	13	2138	57	397	1156	0.0	739
ABCDEF_18352	UniRef50_Q9FWA4	0.0	Normal: 2427	2688	2969	5519	6547	4855	BlastAnno: 2224	UniRef50_Q9FWA4	561	69.31	505	148	3	1887	382	61	561	0.0	683
ABCDEF_27767	UniRef50_Q9LHZ7	0.0	Normal: 2576	5141	2841	10249	14583	5031	BlastAnno: 3981	UniRef50_Q9LHZ7	1170	49.66	888	337	16	2927	504	299	1156	0.0	818
ABCDEF_33661	UniRef50_Q9LHZ7	0.0	Normal: 414	340	507	879	790	783	BlastAnno: 2185	UniRef50_Q9LHZ7	1170	48.66	748	277	14	2184	169	440	1156	0.0	662
ABCDEF_14685	UniRef50_A7XDQ9	0.0	Normal: 233	308	134	573	292	334	BlastAnno: 1947	UniRef50_A7XDQ9	684	56.84	468	186	6	569	1945	1	461	0.0	545
ABCDEF_20660	UniRef50_Q8VZK9	0.0	Normal: 22	48	13	15	52	144	BlastAnno: 1473	UniRef50_Q8VZK9	729	55.16	475	199	6	55	1473	11	473	0.0	537
ABCDEF_21315	UniRef50_Q9LHZ7	0.0	Normal: 3412	252	1450	0	79	1228	BlastAnno: 3765	UniRef50_Q9LHZ7	1170	63.78	1096	340	15	308	3508	70	1137	0.0	1333
ABCDEF_533	UniRef50_Q9C8W3	0.0	Normal: 230	204	269	505	411	301	BlastAnno: 1688	UniRef50_Q9C8W3	401	70.33	364	94	2	393	1475	47	399	0.0	555
ABCDEF_34099	UniRef50_Q9LE59	0.0	Normal: 478	491	735	1356	1350	548	BlastAnno: 2158	UniRef50_Q9LE59	673	60.92	458	178	1	1610	240	215	672	0.0	630
ABCDEF_47984	UniRef50_Q7PC69	0.0	Normal: 663	457	734	1362	2147	696	BlastAnno: 2838	UniRef50_Q7PC69	745	59.27	712	226	9	373	2316	34	745	0.0	702
ABCDEF_51451	UniRef50_Q9LE59	0.0	Normal: 1883	1782	2202	4337	4350	2667	BlastAnno: 2874	UniRef50_Q9LE59	673	74.16	685	165	6	2559	505	1	673	0.0	982
ABCDEF_13370	UniRef50_Q8VZK9	0.0	Normal: 290	87	317	377	105	258	BlastAnno: 2033	UniRef50_Q8VZK9	729	51.65	635	278	11	2031	154	112	726	0.0	612
ABCDEF_48239	UniRef50_Q9LHZ7	0.0	Normal: 511	163	422	1154	1316	510	BlastAnno: 2714	UniRef50_Q9LHZ7	1170	76.20	769	156	9	478	2712	41	806	0.0	1201
ABCDEF_13338	UniRef50_Q7PC69	0.0	Normal: 335	468	417	2639	4443	616	BlastAnno: 2525	UniRef50_Q7PC69	745	62.45	727	209	12	298	2292	21	745	0.0	781
ABCDEF_24193	UniRef50_Q8L7F9	0.0	Normal: 113	72	132	203	180	119	BlastAnno: 1679	UniRef50_Q8L7F9	643	60.54	479	176	4	212	1609	1	479	0.0	592
ABCDEF_48514	UniRef50_Q9ZV71	0.0	Normal: 540	580	601	771	1033	926	BlastAnno: 1920	UniRef50_Q9ZV71	409	78.14	398	85	2	1618	431	12	409	0.0	637
2.4.1.203(trans-zeatin O-beta-D-glucosyltransferase;)																					
ABCDEF_28462	UniRef50_B9T1L8	1e-163	Normal: 90	42	48	20	51	386	BlastAnno: 1317	UniRef50_B9T1L8	456	56.77	384	157	3	3	1139	76	455	1e-163	478
2.4.1.215(cis-zeatin O-beta-D-glucosyltransferase)																					
ABCDEF_3470	UniRef50_Q93XP7	9e-97	Normal: 70	326	56	88	170	310	BlastAnno: 1469	UniRef50_Q93XP7	467	37.76	474	262	12	58	1413	5	467	9e-97	308
2.4.2.40(zeatin O-beta-D-xylosyltransferase;)																					
No Correspond Unigene																					
2.5.1.24(discadenine synthase;)																					
No Correspond Unigene																					
2.5.1.27(adenylate dimethylallyltransferase;)																					
ABCDEF_25246	UniRef50_Q94ID3	4e-34	Normal: 11	0	5	40	19	0	BlastAnno: 468	UniRef50_Q94ID3	357	57.52	113	47	1	133	468	51	163	4e-34	129
2.5.1.50(zeatin 9-aminocarboxyethyltransferase;)																					
No Correspond Unigene																					
2.5.1.75(tRNA dimethylallyltransferase;)																					
ABCDEF_20862	UniRef50_Q6MBT2	1e-60	Normal: 195	161	161	118	169	1579	BlastAnno: 1699	UniRef50_Q6MBT2	344	41.09	348	162	9	1520	483	35	341	1e-60	212
>00909 Sesquiterpenoid biosynthesis																					
1.1.1.216(farnesol dehydrogenase;)																					
No Correspond Unigene																					
1.14.13.106(epi-isozizaene 5-monooxygenase;)																					
No Correspond Unigene																					
3.1.7.-(No Name)																					
No Correspond Unigene																					
4.2.3.-(Lyases;)																					
ABCDEF_30616	UniRef50_Q0JA83	0.0	Normal: 514	201	395	484	549	320	BlastAnno: 3023	UniRef50_Q0JA83	739	47.55	736	339	12	660	2852	33	726	0.0	609
4.2.3.13(No Name)																					
ABCDEF_54972	UniRef50_Q43714	0.0	Normal: 439	0	37	0	0	0	BlastAnno: 1731	UniRef50_Q43714	555	48.19	552	280	3	73	1728	9	554	0.0	533
ABCDEF_792	UniRef50_Q43714	0.0	Normal: 0	123	0	0	7	3329	BlastAnno: 1888	UniRef50_Q43714	555	50.63	557	269	4	1820	150	3	553	0.0	566
ABCDEF_793	UniRef50_Q43714	0.0	Normal: 3	244	8	0	64	2842	BlastAnno: 1936	UniRef50_Q43714	555	50.63	557	269	4	69	1739	3	553	0.0	565
4.2.3.21(vetispiradiene synthase;)																					
ABCDEF_19346	UniRef50_Q39979	8e-75	Normal: 0	132	0	0	10	34	BlastAnno: 548	UniRef50_Q39979	300	70.78	154	43	1	48	509	1	152	8e-75	235
4.2.3.22(germacradienol synthase;)																					
No Correspond Unigene																					
4.2.3.23(germacrene-A synthase;)																					
No Correspond Unigene																					
4.2.3.24(amorpha-4,11-diene synthase;)																					
No Correspond Unigene																					
4.2.3.37(epi-isozizaene synthase;)																					
No Correspond Unigene																					
4.2.3.38(alpha-bisabolene synthase;)																					
No Correspond Unigene																					
4.2.3.39(epi-cedrol synthase;)																					
ABCDEF_82642	UniRef50_Q9LLR9	8e-13	Normal: 0	0	0	0	0	22	BlastAnno: 259	UniRef50_Q9LLR9	547	56.82	44	19	0	255	124	234	277	8e-13	58.9
4.2.3.46(alpha-farnesene synthase;)																					
No Correspond Unigene																					

4.2.3.47(beta-farnesene synthase;)

ABCDEF_54972	UniRef50_Q94JS8	1e-161	Normal: 439	0	37	0	0	0	BlastAnno: 1731	UniRef50_Q94JS8	560	43.37	558	301	5	73	1725	9	558	1e-161	482

4.2.3.6(trichodiene synthase;)
No Correspond Unigene
4.2.3.7(pentalenene synthase;)
No Correspond Unigene
4.2.3.9(aristolochene synthase;)
No Correspond Unigene
5.2.1.9(farnesol 2-isomerase;)
No Correspond Unigene

>00910 Nitrogen metabolism

1.13.12.-(Oxidoreductases;)
No Correspond Unigene
1.13.12.16(nitronate monooxygenase;)
No Correspond Unigene
1.14.12.1(anthranilate 1,2-dioxygenase (deaminating, decarboxylating);)
No Correspond Unigene
1.14.13.35(anthranilate 3-monooxygenase (deaminating);)
No Correspond Unigene
1.18.6.1(nitrogenase)
No Correspond Unigene
1.19.6.1(nitrogenase (flavodoxin))
No Correspond Unigene
1.4.1.13(glutamate synthase (NADPH);)

ABCDEF_9685	UniRef50_C5P2L2	0.0	Normal: 1255	805	1346	3025	1481	566	BlastAnno: 6164	UniRef50_C5P2L2	2137	57.29	1934	742	18	6164	375	250	2103	0.0	2102
ABCDEF_66114	UniRef50_C5P2L2	0.0	Normal: 1307	821	1417	3088	1567	591	BlastAnno: 6039	UniRef50_C5P2L2	2137	58.59	1862	724	16	59	5632	285	2103	0.0	2092
ABCDEF_9687	UniRef50_C5P2L2	0.0	Normal: 1563	1073	1590	4123	1731	640	BlastAnno: 7405	UniRef50_C5P2L2	2137	56.45	2117	832	25	6616	377	40	2103	0.0	2256
ABCDEF_9683	UniRef50_C5P2L2	0.0	Normal: 1525	975	1564	3728	1698	627	BlastAnno: 7188	UniRef50_C5P2L2	2137	56.45	2117	832	25	540	6779	40	2103	0.0	2258
ABCDEF_76911	UniRef50_C5P2L2	0.0	Normal: 1308	820	1416	3108	1574	593	BlastAnno: 6032	UniRef50_C5P2L2	2137	58.29	1870	733	16	6007	410	277	2103	0.0	2092
ABCDEF_9684	UniRef50_C5P2L2	0.0	Normal: 1255	813	1342	2996	1487	561	BlastAnno: 6155	UniRef50_C5P2L2	2137	57.90	1893	744	17	131	5779	254	2103	0.0	2097
ABCDEF_23790	UniRef50_A5UXJ6	0.0	Normal: 1509	3497	1157	1553	1291	9842	BlastAnno: 5662	UniRef50_A5UXJ6	1554	47.52	1553	718	23	5114	627	18	1530	0.0	1355

1.4.1.14(glutamate synthase (NADH);)

ABCDEF_9685	UniRef50_Q03460	0.0	Normal: 1255	805	1346	3025	1481	566	BlastAnno: 6164	UniRef50_Q03460	2194	82.36	1962	296	5	6164	306	274	2194	0.0	3175
ABCDEF_66114	UniRef50_Q03460	0.0	Normal: 1307	821	1417	3088	1567	591	BlastAnno: 6039	UniRef50_Q03460	2194	83.91	1896	291	4	41	5701	304	2194	0.0	3140
ABCDEF_9687	UniRef50_Q03460	0.0	Normal: 1563	1073	1590	4123	1731	640	BlastAnno: 7405	UniRef50_Q03460	2194	82.91	2159	351	6	6754	308	44	2194	0.0	3558
ABCDEF_9683	UniRef50_Q03460	0.0	Normal: 1525	975	1564	3728	1698	627	BlastAnno: 7188	UniRef50_Q03460	2194	82.91	2159	351	6	402	6848	44	2194	0.0	3555
ABCDEF_76911	UniRef50_Q03460	0.0	Normal: 1308	820	1416	3108	1574	593	BlastAnno: 6032	UniRef50_Q03460	2194	83.86	1896	292	4	6001	341	304	2194	0.0	3145
ABCDEF_9684	UniRef50_Q03460	0.0	Normal: 1255	813	1342	2996	1487	561	BlastAnno: 6155	UniRef50_Q03460	2194	82.84	1929	309	7	92	5848	278	2194	0.0	3147

1.4.1.2(glutamate dehydrogenase;)

ABCDEF_52465	UniRef50_Q5HHC7	4e-126	Normal: 404	402	745	2021	3684	1711	BlastAnno: 1651	UniRef50_Q5HHC7	414	44.01	409	227	2	213	1436	6	413	4e-126	384

1.4.1.3(glutamate dehydrogenase [NAD(P)+];)

ABCDEF_25196	UniRef50_Q43314	0.0	Normal: 271	16	291	548	795	8	BlastAnno: 1412	UniRef50_Q43314	411	78.84	378	80	0	1410	277	34	411	0.0	642
ABCDEF_52465	UniRef50_Q43314	0.0	Normal: 404	402	745	2021	3684	1711	BlastAnno: 1651	UniRef50_Q43314	411	79.81	411	83	0	210	1442	1	411	0.0	699
ABCDEF_24937	UniRef50_Q43314	0.0	Normal: 94	39	122	768	1065	114	BlastAnno: 1144	UniRef50_Q43314	411	87.97	349	42	0	1047	1	1	349	0.0	649
ABCDEF_32993	UniRef50_Q43314	0.0	Normal: 127	299	296	1133	320	898	BlastAnno: 1450	UniRef50_Q43314	411	89.78	411	42	0	1382	150	1	411	0.0	775
ABCDEF_42257	UniRef50_Q43314	0.0	Normal: 140	355	359	1267	415	1032	BlastAnno: 1644	UniRef50_Q43314	411	89.78	411	42	0	1454	222	1	411	0.0	775

1.4.1.4(glutamate dehydrogenase (NADP+);)

ABCDEF_41247	UniRef50_P43793	1e-117	Normal: 929	608	918	1231	1278	890	BlastAnno: 2684	UniRef50_P43793	449	42.26	452	250	6	950	2284	1	448	1e-117	374

1.4.2.-(Oxidoreductases;)
No Correspond Unigene
1.4.7.1(glutamate synthase (ferredoxin);)

ABCDEF_9685	UniRef50_P55037	0.0	Normal: 1255	805	1346	3025	1481	566	BlastAnno: 6164	UniRef50_P55037	1550	54.51	1387	549	18	6164	2013	215	1522	0.0	1392
ABCDEF_66114	UniRef50_P55037	0.0	Normal: 1307	821	1417	3088	1567	591	BlastAnno: 6039	UniRef50_P55037	1550	56.27	1315	530	16	59	3994	250	1522	0.0	1385
ABCDEF_76911	UniRef50_P55037	0.0	Normal: 1308	820	1416	3108	1574	593	BlastAnno: 6032	UniRef50_P55037	1550	56.19	1317	532	16	5989	2048	248	1522	0.0	1385
ABCDEF_9684	UniRef50_P55037	0.0	Normal: 1255	813	1342	2996	1487	561	BlastAnno: 6155	UniRef50_P55037	1550	56.19	1317	532	16	200	4141	248	1522	0.0	1384
ABCDEF_9687	UniRef50_P55037	0.0	Normal: 1563	1073	1590	4123	1731	640	BlastAnno: 7405	UniRef50_P55037	1550	54.06	1539	654	19	6616	2015	32	1522	0.0	1573
ABCDEF_9683	UniRef50_P55037	0.0	Normal: 1525	975	1564	3728	1698	627	BlastAnno: 7188	UniRef50_P55037	1550	54.06	1539	654	19	540	5141	32	1522	0.0	1573
ABCDEF_23790	UniRef50_A9AZQ4	0.0	Normal: 1509	3497	1157	1553	1291	9842	BlastAnno: 5662	UniRef50_A9AZQ4	1485	45.86	1511	745	20	5114	630	11	1464	0.0	1277

1.4.99.1(D-amino-acid dehydrogenase;)
No Correspond Unigene
1.7.1.1(nitrate reductase (NADH);)

ABCDEF_38826	UniRef50_P11035	0.0	Normal: 70	224	0	17	71	28	BlastAnno: 1108	UniRef50_P11035	917	86.60	373	46	2	1	1107	234	606	0.0	686

1.7.1.10(hydroxylamine reductase (NADH);)
No Correspond Unigene
1.7.1.2(nitrate reductase [NAD(P)H];)
No Correspond Unigene
1.7.1.3(nitrate reductase (NADPH);)

ABCDEF_38826	UniRef50_P39864	4e-110	Normal: 70	224	0	17	71	28	BlastAnno: 1108	UniRef50_P39864	902	47.86	374	171	6	31	1101	233	599	4e-110	351

1.7.1.4(nitrite reductase [NAD(P)H];)
No Correspond Unigene
1.7.2.1(nitrite reductase (NO-forming);)
No Correspond Unigene

1.7.2.2(nitrite reductase (cytochrome; ammonia-forming);)
No Correspond Unigene
1.7.3.1(nitroalkane oxidase;)
No Correspond Unigene
1.7.3.4(hydroxylamine oxidase;)
No Correspond Unigene
1.7.7.1(ferredoxin---nitrite reductase)

ABCDEF_44808	UniRef50_Q39161	0.0	Normal: 334	170	264	942	2919	206	BlastAnno: 2107	UniRef50_Q39161	586	76.61	590	133	2	1961	195	1	586	0.0	956
ABCDEF_41624	UniRef50_Q39161	0.0	Normal: 132	856	57	799	605	1885	BlastAnno: 2069	UniRef50_Q39161	586	76.53	588	135	2	135	1895	1	586	0.0	943

1.7.7.2(ferredoxin---nitrate reductase;)
No Correspond Unigene
1.7.99.1(hydroxylamine reductase;)
No Correspond Unigene
1.7.99.4(nitrate reductase;)
No Correspond Unigene
1.7.99.6(nitrous-oxide reductase;)
No Correspond Unigene
1.7.99.7(nitric-oxide reductase;)
No Correspond Unigene
1.9.6.1(nitrate reductase (cytochrome);)
No Correspond Unigene
2.1.2.10(aminomethyltransferase;)

ABCDEF_22217	UniRef50_O65396	0.0	Normal: 1753	5094	1871	4253	3113	17559	BlastAnno: 1818	UniRef50_O65396	408	87.47	407	51	0	363	1583	1	407	0.0	752

2.7.2.2(carbamate kinase;)
No Correspond Unigene
3.5.1.1(asparaginase;)

ABCDEF_13981	UniRef50_Q0E667	3e-157	Normal: 943	1644	1236	3106	2580	499	BlastAnno: 1218	UniRef50_Q0E667	375	61.50	374	93	2	1108	140	2	375	3e-157	457

3.5.1.2(glutaminase;)

ABCDEF_56484	UniRef50_B0TVW4	4e-16	Normal: 9	0	0	0	0	0	BlastAnno: 289	UniRef50_B0TVW4	514	43.37	83	47	0	252	4	428	510	4e-16	79.0

3.5.1.38(glutamin-(asparagin-)ase)
No Correspond Unigene
3.5.1.49(formamidase)

ABCDEF_60429	UniRef50_Q9SZE9	0.0	Normal: 13	40	13	16	7	67	BlastAnno: 1646	UniRef50_Q9SZE9	432	70.39	456	93	4	108	1421	1	432	0.0	619
ABCDEF_23263	UniRef50_Q9SZE9	0.0	Normal: 18	37	10	15	9	48	BlastAnno: 1541	UniRef50_Q9SZE9	432	77.32	366	63	1	1405	308	1	346	0.0	570
ABCDEF_23268	UniRef50_Q9SZE9	0.0	Normal: 19	60	15	18	9	96	BlastAnno: 1677	UniRef50_Q9SZE9	432	68.42	456	102	4	139	1452	1	432	0.0	592
ABCDEF_63714	UniRef50_Q9SZE9	0.0	Normal: 24	55	17	24	11	83	BlastAnno: 1689	UniRef50_Q9SZE9	432	80.24	420	63	1	108	1367	1	400	0.0	689
ABCDEF_23269	UniRef50_Q9SZE9	0.0	Normal: 59	149	58	65	24	188	BlastAnno: 1719	UniRef50_Q9SZE9	432	78.54	452	77	1	139	1494	1	432	0.0	719

3.5.5.1(nitrilase;)

ABCDEF_3989	UniRef50_P46011	0.0	Normal: 806	816	966	1114	1416	1512	BlastAnno: 1581	UniRef50_P46011	355	79.10	354	71	3	255	1310	3	355	0.0	579

3.5.5.2(ricinine nitrilase)
No Correspond Unigene
4.1.99.1(tryptophanase;)
No Correspond Unigene
4.1.99.2(tyrosine phenol-lyase;)
No Correspond Unigene
4.2.1.1(carbonate dehydratase;)

ABCDEF_32852	UniRef50_P27140	2e-150	Normal: 0	4879	0	0	0	57554	BlastAnno: 1566	UniRef50_P27140	347	78.10	274	60	0	431	1252	56	329	2e-150	443

4.2.1.104(cyanase;)

ABCDEF_8227	UniRef50_O22683	9e-71	Normal: 593	442	800	1545	2271	1929	BlastAnno: 926	UniRef50_O22683	168	73.01	163	40	1	820	332	6	164	9e-71	225

4.3.1.1(aspartate ammonia-lyase;)
No Correspond Unigene
4.3.1.2(methylaspartate ammonia-lyase;)
No Correspond Unigene
4.3.1.23(tyrosine ammonia-lyase;)
No Correspond Unigene
4.3.1.24(phenylalanine ammonia-lyase;)

ABCDEF_34414	UniRef50_Q6GZ04	0.0	Normal: 1997	3260	3067	14209	6957	2185	BlastAnno: 2627	UniRef50_Q6GZ04	698	47.67	644	318	7	2160	253	48	680	0.0	591
ABCDEF_35119	UniRef50_Q6GZ04	0.0	Normal: 2644	1363	3193	6969	3673	1650	BlastAnno: 2515	UniRef50_Q6GZ04	698	46.71	683	344	7	264	2285	9	680	0.0	597
ABCDEF_21563	UniRef50_C5YCD7	0.0	Normal: 707	225	993	972	1864	182	BlastAnno: 1483	UniRef50_C5YCD7	688	74.23	489	100	1	1482	16	225	687	0.0	758
ABCDEF_59755	UniRef50_Q6GZ04	0.0	Normal: 1915	2750	3275	9429	2466	3052	BlastAnno: 2458	UniRef50_Q6GZ04	698	46.86	685	338	10	2178	163	9	680	0.0	595
ABCDEF_7556	UniRef50_Q6GZ04	0.0	Normal: 2012	2842	3468	9812	2712	3290	BlastAnno: 2512	UniRef50_Q6GZ04	698	46.43	700	349	10	285	2345	9	695	0.0	595

4.3.1.25(phenylalanine/tyrosine ammonia-lyase;)
No Correspond Unigene
4.3.1.3(histidine ammonia-lyase;)

ABCDEF_77305	UniRef50_B5XIY2	3e-13	Normal: 218	337	343	1269	246	230	BlastAnno: 612	UniRef50_B5XIY2	513	36.80	125	70	3	241	609	5	122	3e-13	73.9

4.4.1.1(cystathionine gamma-lyase;)

ABCDEF_72071	UniRef50_Q55DV9	1e-80	Normal: 173	154	160	241	209	225	BlastAnno: 1568	UniRef50_Q55DV9	387	47.49	259	135	1	662	1435	126	384	1e-80	260
ABCDEF_72428	UniRef50_Q55DV9	1e-80	Normal: 135	117	119	172	170	180	BlastAnno: 1527	UniRef50_Q55DV9	387	47.49	259	135	1	621	1394	126	384	1e-80	260

4.4.1.2(homocysteine desulfhydrase;)
No Correspond Unigene
4.4.1.8(cystathionine beta-lyase;)

ABCDEF_71418	UniRef50_P53780	0.0	Normal: 160	139	136	188	195	199	BlastAnno: 1556	UniRef50_P53780	464	72.22	450	117	2	86	1435	22	463	0.0	637

ABCDEF_40739	UniRef50_P53780	0.0	Normal: 202	178	179	256	234	247	BlastAnno: 1653	UniRef50_P53780	464	71.24	459	124	2	130	1506	13	463	0.0	644
6.3.1.1(aspartate---ammonia ligase;)																					
No Correspond Unigene																					
6.3.1.2(glutamate---ammonia ligase;)																					
ABCDEF_27409	UniRef50_Q56WN1	0.0	Normal: 792	857	694	764	746	4126	BlastAnno: 1892	UniRef50_Q56WN1	356	80.76	343	66	0	460	1488	1	343	0.0	547
ABCDEF_60010	UniRef50_Q56WN1	0.0	Normal: 722	827	669	717	712	3883	BlastAnno: 1644	UniRef50_Q56WN1	356	80.76	343	66	0	290	1318	1	343	0.0	547
ABCDEF_27406	UniRef50_Q56WN1	0.0	Normal: 1322	8376	1449	1550	2297	27785	BlastAnno: 1946	UniRef50_Q56WN1	356	79.49	356	73	0	537	1604	1	356	0.0	546
ABCDEF_38019	UniRef50_Q56WN1	0.0	Normal: 65	126	230	858	520	162	BlastAnno: 1336	UniRef50_Q56WN1	356	85.11	356	53	0	77	1144	1	356	0.0	654
ABCDEF_28058	UniRef50_Q56WN1	0.0	Normal: 999	55	1008	535	2834	260	BlastAnno: 1368	UniRef50_Q56WN1	356	86.30	343	47	0	93	1121	14	356	0.0	587
ABCDEF_28620	UniRef50_Q56WN1	0.0	Normal: 2577	3235	3447	8812	12665	2225	BlastAnno: 1455	UniRef50_Q56WN1	356	91.12	338	30	0	169	1182	16	353	0.0	595
6.3.1.4(aspartate---ammonia ligase (ADP-forming);)																					
No Correspond Unigene																					
6.3.1.5(NAD+ synthase;)																					
ABCDEF_47914	UniRef50_B6KCT7	0.0	Normal: 564	429	500	832	1072	838	BlastAnno: 2524	UniRef50_B6KCT7	862	47.30	778	317	13	2371	311	33	808	0.0	679
6.3.4.16(carbamoyl-phosphate synthase (ammonia);)																					
No Correspond Unigene																					
6.3.5.4(asparagine synthase (glutamine-hydrolysing);)																					
ABCDEF_9351	UniRef50_Q83S76	0.0	Normal: 45	29	8	0	0	5615	BlastAnno: 2191	UniRef50_Q83S76	515	53.32	557	216	7	131	1795	1	515	0.0	556
ABCDEF_82324	UniRef50_Q83S76	0.0	Normal: 7	19	0	0	0	2399	BlastAnno: 2218	UniRef50_Q83S76	515	52.96	557	218	7	186	1850	1	515	0.0	550
ABCDEF_11540	UniRef50_P49078	0.0	Normal: 2080	820	1576	1695	1260	2315	BlastAnno: 2183	UniRef50_P49078	584	78.38	555	120	0	1989	325	1	555	0.0	896
>00920 Sulfur metabolism																					
1.13.11.18(sulfur dioxygenase;)																					
No Correspond Unigene																					
1.8.1.2(sulfite reductase (NADPH);)																					
ABCDEF_14065	UniRef50_A2C1J2	2e-179	Normal: 1146	570	1258	1446	3426	377	BlastAnno: 2475	UniRef50_A2C1J2	714	47.89	593	295	9	2190	439	121	708	2e-179	542
1.8.2.1(sulfite dehydrogenase;)																					
No Correspond Unigene																					
1.8.3.1(sulfite oxidase)																					
ABCDEF_32244	UniRef50_Q9S850	0.0	Normal: 1118	1361	1137	1433	1965	3518	BlastAnno: 1610	UniRef50_Q9S850	393	78.68	394	83	1	163	1344	1	393	0.0	667
1.8.4.8(phosphoadenylyl-sulfate reductase (thioredoxin);)																					
ABCDEF_50910	UniRef50_A5W608	2e-92	Normal: 118	167	261	297	375	650	BlastAnno: 1572	UniRef50_A5W608	244	55.41	231	103	0	1296	604	10	240	2e-92	291
1.8.7.1(sulfite reductase (ferredoxin);)																					
ABCDEF_14065	UniRef50_P72854	0.0	Normal: 1146	570	1258	1446	3426	377	BlastAnno: 2475	UniRef50_P72854	635	53.60	584	256	7	2163	442	2	580	0.0	630
ABCDEF_30811	UniRef50_P72854	0.0	Normal: 441	890	361	322	307	5643	BlastAnno: 2549	UniRef50_P72854	635	51.39	574	267	7	2187	496	6	577	0.0	592
1.8.99.1(sulfite reductase;)																					
No Correspond Unigene																					
1.8.99.2(adenylyl-sulfate reductase;)																					
No Correspond Unigene																					
2.3.1.30(serine O-acetyltransferase;)																					
ABCDEF_31726	UniRef50_B9S9Q4	3e-161	Normal: 94	985	55	43	120	1929	BlastAnno: 1549	UniRef50_B9S9Q4	396	63.93	377	104	6	1287	250	19	394	3e-161	473
2.3.1.31(homoserine O-acetyltransferase;)																					
No Correspond Unigene																					
2.3.1.46(homoserine O-succinyltransferase;)																					
No Correspond Unigene																					
2.5.1.47(cysteine synthase;)																					
ABCDEF_4276	UniRef50_P47999	0.0	Normal: 991	1556	999	1021	1482	4746	BlastAnno: 1414	UniRef50_P47999	392	77.08	349	76	1	146	1192	47	391	0.0	533
2.5.1.48(cystathionine gamma-synthase;)																					
ABCDEF_31946	UniRef50_P55217	0.0	Normal: 3795	4731	4027	7276	7940	8571	BlastAnno: 1625	UniRef50_P55217	563	84.28	407	60	1	1399	179	161	563	0.0	719
2.5.1.65(O-phosphoserine sulfhydrylase;)																					
No Correspond Unigene																					
2.7.1.25(adenylyl-sulfate kinase;)																					
ABCDEF_31787	UniRef50_B8CBW8	2e-117	Normal: 1065	1161	1322	2258	1986	3606	BlastAnno: 2042	UniRef50_B8CBW8	968	46.74	430	200	5	1602	397	310	738	2e-117	383
2.7.7.4(sulfate adenylyltransferase;)																					
ABCDEF_10695	UniRef50_B6KN22	0.0	Normal: 1568	429	1310	1338	1351	785	BlastAnno: 3382	UniRef50_B6KN22	1008	53.54	1003	431	10	3195	274	5	1001	0.0	1061
ABCDEF_48012	UniRef50_B6KBK6	0.0	Normal: 909	375	986	1182	1437	436	BlastAnno: 2606	UniRef50_B6KBK6	877	54.57	722	296	6	2316	247	148	869	0.0	791
ABCDEF_38032	UniRef50_Q43870	0.0	Normal: 611	1036	642	1131	1509	1791	BlastAnno: 1947	UniRef50_Q43870	476	73.00	426	115	0	1636	359	42	467	0.0	661
ABCDEF_31663	UniRef50_Q43870	0.0	Normal: 1022	1780	1065	1614	1663	3264	BlastAnno: 1893	UniRef50_Q43870	476	74.21	411	106	0	1723	491	57	467	0.0	663
ABCDEF_31787	UniRef50_Q43870	0.0	Normal: 1065	1161	1322	2258	1986	3606	BlastAnno: 2042	UniRef50_Q43870	476	76.60	483	106	3	1809	361	1	476	0.0	771
ABCDEF_10694	UniRef50_B6KN22	0.0	Normal: 1188	323	1053	981	1089	607	BlastAnno: 2848	UniRef50_B6KN22	1008	55.54	884	365	7	21	2588	118	1001	0.0	1015
2.7.7.5(sulfate adenylyltransferase (ADP);)																					
No Correspond Unigene																					
2.8.1.5(thiosulfate---dithiol sulfurtransferase;)																					
No Correspond Unigene																					
2.8.2.1(aryl sulfotransferase;)																					
No Correspond Unigene																					
2.8.2.2(alcohol sulfotransferase;)																					
ABCDEF_50672	UniRef50_B9SUK2	6e-80	Normal: 233	351	355	724	181	516	BlastAnno: 1433	UniRef50_B9SUK2	317	45.26	285	138	6	1194	343	39	306	6e-80	259
2.8.2.3(amine sulfotransferase;)																					
No Correspond Unigene																					
2.8.2.4(estrone sulfotransferase;)																					
ABCDEF_60620	UniRef50_B9RE91	1e-103	Normal: 240	393	445	400	902	151	BlastAnno: 1191	UniRef50_B9RE91	354	50.59	340	156	5	31	1050	27	354	1e-103	319
2.8.2.5(chondroitin 4-sulfotransferase;)																					

No Correspond Unigene																					
2.8.2.6(choline sulfotransferase;)																					
No Correspond Unigene																					
3.1.3.7(3'(2'),5'-bisphosphate nucleotidase;)																					
ABCDEF_51283	UniRef50_Q42546	5e-172	Normal: 359	210	299	280	398	472	BlastAnno: 1368	UniRef50_Q42546	353	78.67	347	69	2	175	1200	1	347	5e-172	496
3.12.1.1(trithionate hydrolase)																					
No Correspond Unigene																					
3.6.2.1(adenylylsulfatase;)																					
No Correspond Unigene																					
3.6.2.2(phosphoadenylylsulfatase;)																					
No Correspond Unigene																					
4.4.1.2(homocysteine desulfhydrase;)																					
No Correspond Unigene																					
4.4.1.8(cystathionine beta-lyase;)																					
ABCDEF_71418	UniRef50_P53780	0.0	Normal: 160	139	136	188	195	199	BlastAnno: 1556	UniRef50_P53780	464	72.22	450	117	2	86	1435	22	463	0.0	637
ABCDEF_40739	UniRef50_P53780	0.0	Normal: 202	178	179	256	234	247	BlastAnno: 1653	UniRef50_P53780	464	71.24	459	124	2	130	1506	13	463	0.0	644
>00930 Caprolactam degradation																					
1.1.1.174(cyclohexane-1,2-diol dehydrogenase)																					
No Correspond Unigene																					
1.1.1.2(alcohol dehydrogenase (NADP+);)																					
ABCDEF_27285	UniRef50_O57380	5e-94	Normal: 119	81	124	95	252	18	BlastAnno: 1489	UniRef50_O57380	373	46.11	373	197	4	1279	161	3	371	5e-94	298
1.1.1.245(cyclohexanol dehydrogenase)																					
No Correspond Unigene																					
1.1.1.258(6-hydroxyhexanoate dehydrogenase)																					
No Correspond Unigene																					
1.1.1.35(3-hydroxyacyl-CoA dehydrogenase;)																					
ABCDEF_80649	UniRef50_Q1LD07	5e-120	Normal: 527	841	443	1308	1321	1188	BlastAnno: 2586	UniRef50_Q1LD07	715	37.34	699	420	9	2358	277	22	707	5e-120	389
1.1.1.90(aryl-alcohol dehydrogenase;)																					
No Correspond Unigene																					
1.14.13.22(cyclohexanone monooxygenase;)																					
No Correspond Unigene																					
1.14.15.-(Oxidoreductases;)																					
No Correspond Unigene																					
1.2.1.4(aldehyde dehydrogenase (NADP+);)																					
No Correspond Unigene																					
1.2.1.63(6-oxohexanoate dehydrogenase)																					
No Correspond Unigene																					
1.3.99.-(Oxidoreductases;)																					
ABCDEF_4448	UniRef50_Q6JQN1	0.0	Normal: 1778	1683	1752	2720	2409	2803	BlastAnno: 2780	UniRef50_Q6JQN1	1059	48.13	802	354	14	231	2564	293	1056	0.0	718
1.4.3.12(cyclohexylamine oxidase)																					
No Correspond Unigene																					
2.6.1.-(Transferases;)																					
ABCDEF_3716	UniRef50_Q9LQ10	0.0	Normal: 224	353	140	47	76	606	BlastAnno: 2230	UniRef50_Q9LQ10	557	61.35	533	189	8	250	1812	30	557	0.0	609
ABCDEF_37756	UniRef50_Q9LQ10	0.0	Normal: 197	168	149	152	186	490	BlastAnno: 1840	UniRef50_Q9LQ10	557	57.49	534	196	7	1638	109	28	554	0.0	619
3.1.1.17(gluconolactonase;)																					
ABCDEF_17917	UniRef50_A1TXZ0	5e-64	Normal: 303	575	152	163	236	2055	BlastAnno: 1384	UniRef50_A1TXZ0	363	36.89	347	200	6	1216	200	23	358	5e-64	219
3.10.1.2(cyclamate sulfohydrolase;)																					
No Correspond Unigene																					
3.5.1.-(Hydrolases;)																					
ABCDEF_41579	UniRef50_O81641	0.0	Normal: 384	122	265	320	313	150	BlastAnno: 1456	UniRef50_O81641	428	65.16	399	138	1	1313	120	23	421	0.0	543
ABCDEF_16924	UniRef50_Q8VYX0	0.0	Normal: 135	349	460	1325	685	527	BlastAnno: 2002	UniRef50_Q8VYX0	464	71.72	396	109	2	1612	428	68	461	0.0	549
ABCDEF_24290	UniRef50_O04373	0.0	Normal: 296	238	403	498	698	330	BlastAnno: 1710	UniRef50_O04373	440	65.24	443	151	3	67	1395	1	440	0.0	595
3.5.2.-(Hydrolases;)																					
No Correspond Unigene																					
3.7.1.11(cyclohexane-1,2-dione hydrolase)																					
No Correspond Unigene																					
4.2.1.103(cyclohexyl-isocyanide hydratase;)																					
No Correspond Unigene																					
4.2.1.17(enoyl-CoA hydratase;)																					
ABCDEF_21259	UniRef50_Q8W1L6	0.0	Normal: 272	276	284	384	326	305	BlastAnno: 2641	UniRef50_Q8W1L6	726	47.57	721	375	3	114	2267	6	726	0.0	633
ABCDEF_80649	UniRef50_Q8W1L6	0.0	Normal: 527	841	443	1308	1321	1188	BlastAnno: 2586	UniRef50_Q8W1L6	726	60.39	712	281	1	2403	268	1	711	0.0	818
ABCDEF_466	UniRef50_Q8W1L6	0.0	Normal: 1014	1690	1094	1677	2438	2080	BlastAnno: 2453	UniRef50_Q8W1L6	726	71.24	699	201	0	2368	272	7	705	0.0	954
ABCDEF_54834	UniRef50_Q8W1L6	0.0	Normal: 229	220	237	298	270	261	BlastAnno: 2405	UniRef50_Q8W1L6	726	47.52	606	315	3	274	2082	121	726	0.0	580
ABCDEF_34756	UniRef50_Q8W1L6	0.0	Normal: 776	726	768	2922	4039	1079	BlastAnno: 2469	UniRef50_Q8W1L6	726	68.93	708	217	2	2396	273	7	711	0.0	957
ABCDEF_4198	UniRef50_Q8W1L6	0.0	Normal: 609	929	532	1401	1568	1297	BlastAnno: 2667	UniRef50_Q8W1L6	726	60.45	713	281	1	2463	325	1	712	0.0	820
6.2.1.-(Ligases;)																					
ABCDEF_7378	UniRef50_Q9FFE6	0.0	Normal: 1022	1422	746	576	680	1408	BlastAnno: 2117	UniRef50_Q9FFE6	552	67.57	552	167	5	144	1778	1	547	0.0	768
ABCDEF_47513	UniRef50_Q8RU95	0.0	Normal: 411	609	570	233	749	887	BlastAnno: 2280	UniRef50_Q8RU95	598	53.15	572	246	6	119	1777	28	596	0.0	543
ABCDEF_78742	UniRef50_Q84P25	0.0	Normal: 236	207	373	576	450	306	BlastAnno: 1534	UniRef50_Q84P25	565	60.39	462	168	5	1413	49	19	472	0.0	546
ABCDEF_10753	UniRef50_Q9FFE6	0.0	Normal: 394	401	336	253	292	348	BlastAnno: 1841	UniRef50_Q9FFE6	552	51.00	551	259	5	117	1751	1	546	0.0	553
ABCDEF_4431	UniRef50_Q9FFE6	0.0	Normal: 27	358	28	98	112	914	BlastAnno: 2075	UniRef50_Q9FFE6	552	50.71	560	264	7	1774	107	1	552	0.0	551
ABCDEF_38213	UniRef50_Q84P25	0.0	Normal: 342	337	597	798	722	505	BlastAnno: 1939	UniRef50_Q84P25	565	62.34	555	194	5	1818	175	19	565	0.0	690

ABCDEF_25221	UniRef50_Q0DV32	0.0	Normal: 971	826	1003	1543	1442	1545	BlastAnno: 2009	UniRef50_Q0DV32	552	57.20	542	226	4	183	1793	9	549	0.0	613
ABCDEF_23413	UniRef50_Q84P23	0.0	Normal: 123	237	158	149	213	658	BlastAnno: 3175	UniRef50_Q84P23	562	51.74	547	243	8	3030	1435	15	555	0.0	555
ABCDEF_20486	UniRef50_Q9FFE6	0.0	Normal: 580	912	312	324	864	1565	BlastAnno: 3530	UniRef50_Q9FFE6	552	66.61	557	171	5	352	1995	1	551	0.0	781
>00940 Phenylpropanoid biosynthesis																					
1.1.1.195(cinnamyl-alcohol dehydrogenase;)																					
ABCDEF_36163	UniRef50_Q9CAI3	0.0	Normal: 146	182	143	193	285	358	BlastAnno: 1440	UniRef50_Q9CAI3	355	76.69	356	81	2	132	1196	1	355	0.0	541
ABCDEF_843	UniRef50_Q9CAI3	0.0	Normal: 165	196	152	228	294	375	BlastAnno: 1489	UniRef50_Q9CAI3	355	76.69	356	81	2	1310	246	1	355	0.0	540
ABCDEF_53286	UniRef50_Q9CAI3	0.0	Normal: 148	176	144	209	272	360	BlastAnno: 1486	UniRef50_Q9CAI3	355	76.69	356	81	2	1310	246	1	355	0.0	540
1.11.1.7(peroxidase;)																					
ABCDEF_25672	UniRef50_Q9SB81	0.0	Normal: 7636	15838	4777	1134	3090	9628	BlastAnno: 1715	UniRef50_Q9SB81	330	85.71	301	43	0	342	1244	30	330	0.0	552
1.13.11.22(caffeate 3,4-dioxygenase)																					
No Correspond Unigene																					
1.14.-.-(Oxidoreductases;)																					
ABCDEF_44739	UniRef50_O64989	0.0	Normal: 157	87	159	718	673	189	BlastAnno: 2185	UniRef50_O64989	513	70.75	482	112	3	1867	509	30	511	0.0	706
ABCDEF_31491	UniRef50_Q93VK5	0.0	Normal: 647	1826	671	1517	1770	5953	BlastAnno: 2278	UniRef50_Q93VK5	595	79.14	556	113	2	198	1862	35	588	0.0	902
ABCDEF_40554	UniRef50_P37122	0.0	Normal: 1574	1962	1829	1150	2882	2721	BlastAnno: 1828	UniRef50_P37122	505	60.52	504	198	1	1752	244	1	504	0.0	638
ABCDEF_34049	UniRef50_O23365	0.0	Normal: 363	384	349	442	549	1416	BlastAnno: 2101	UniRef50_O23365	580	76.17	579	116	5	230	1957	19	578	0.0	904
ABCDEF_21579	UniRef50_O48928	0.0	Normal: 1005	643	1606	4372	3226	1621	BlastAnno: 1895	UniRef50_O48928	513	69.72	502	151	1	185	1690	7	507	0.0	731
ABCDEF_37741	UniRef50_P37122	0.0	Normal: 12	760	9	24	34	576	BlastAnno: 1197	UniRef50_P37122	505	65.70	379	129	1	1195	59	127	504	0.0	526
ABCDEF_35618	UniRef50_O48928	0.0	Normal: 28	2648	17	142	1234	902	BlastAnno: 1746	UniRef50_O48928	513	66.40	506	168	2	1649	132	4	507	0.0	701
ABCDEF_24522	UniRef50_P37122	0.0	Normal: 334	2190	561	268	1391	2459	BlastAnno: 2075	UniRef50_P37122	505	61.52	460	176	1	1544	165	47	505	0.0	612
ABCDEF_14592	UniRef50_O23066	0.0	Normal: 1514	402	2245	7837	4742	1248	BlastAnno: 2378	UniRef50_O23066	553	72.71	546	140	4	2111	489	1	542	0.0	821
ABCDEF_14512	UniRef50_Q9FG65	0.0	Normal: 261	872	212	291	175	789	BlastAnno: 1878	UniRef50_Q9FG65	502	56.89	501	197	9	1738	266	11	502	0.0	532
ABCDEF_44487	UniRef50_Q940V4	0.0	Normal: 3	41	53	501	262	542	BlastAnno: 1712	UniRef50_Q940V4	465	69.37	382	116	1	38	1180	14	395	0.0	572
ABCDEF_17723	UniRef50_P37122	0.0	Normal: 240	1698	219	768	592	794	BlastAnno: 1757	UniRef50_P37122	505	63.40	459	166	2	228	1601	47	504	0.0	617
ABCDEF_37901	UniRef50_Q42569	0.0	Normal: 30	149	86	255	195	255	BlastAnno: 1246	UniRef50_Q42569	472	78.27	359	73	2	64	1140	43	396	0.0	567
ABCDEF_7260	UniRef50_O81117	0.0	Normal: 314	1085	466	3248	2575	1360	BlastAnno: 1768	UniRef50_O81117	514	59.92	474	188	1	145	1566	43	514	0.0	615
ABCDEF_40548	UniRef50_P37122	0.0	Normal: 2756	2467	2752	1072	3120	1735	BlastAnno: 1947	UniRef50_P37122	505	59.72	504	202	1	241	1749	1	504	0.0	636
ABCDEF_44485	UniRef50_Q940V4	0.0	Normal: 3	55	63	574	338	660	BlastAnno: 1607	UniRef50_Q940V4	465	68.58	452	141	1	42	1394	14	465	0.0	673
ABCDEF_40552	UniRef50_P37122	0.0	Normal: 2397	1870	2304	1249	10428	2291	BlastAnno: 1812	UniRef50_P37122	505	60.12	504	200	1	108	1616	1	504	0.0	640
ABCDEF_37902	UniRef50_Q42569	0.0	Normal: 84	524	254	725	670	1049	BlastAnno: 1812	UniRef50_Q42569	472	77.47	435	92	3	1675	371	43	471	0.0	681
ABCDEF_40556	UniRef50_P37122	0.0	Normal: 1907	2365	2163	1319	3678	3308	BlastAnno: 1944	UniRef50_P37122	505	61.04	498	193	1	264	1754	1	498	0.0	635
ABCDEF_40549	UniRef50_P37122	0.0	Normal: 1574	1962	1826	1150	2882	2723	BlastAnno: 1845	UniRef50_P37122	505	60.52	504	198	1	1754	246	1	504	0.0	638
ABCDEF_20301	UniRef50_O81117	0.0	Normal: 89	308	123	176	389	249	BlastAnno: 1637	UniRef50_O81117	514	64.04	470	164	3	1498	98	47	514	0.0	586
ABCDEF_3882	UniRef50_Q42569	0.0	Normal: 84	243	257	786	1561	182	BlastAnno: 1854	UniRef50_Q42569	472	72.51	422	107	1	1852	587	59	471	0.0	615
ABCDEF_20703	UniRef50_O49858	0.0	Normal: 115	636	20	0	0	597	BlastAnno: 1815	UniRef50_O49858	527	50.10	523	248	6	48	1607	12	524	0.0	533
ABCDEF_18295	UniRef50_O22203	0.0	Normal: 3667	1934	4977	10491	4588	1917	BlastAnno: 2006	UniRef50_O22203	508	74.85	493	123	1	214	1692	17	508	0.0	803
ABCDEF_48724	UniRef50_O23066	0.0	Normal: 104	11518	163	2083	3464	6356	BlastAnno: 2162	UniRef50_O23066	553	72.96	540	143	3	227	1840	1	539	0.0	807
ABCDEF_37738	UniRef50_P37122	0.0	Normal: 26	2447	19	30	33	993	BlastAnno: 1837	UniRef50_P37122	505	64.30	479	169	2	1685	249	28	504	0.0	639
1.14.13.-(Oxidoreductases;)																					
ABCDEF_45538	UniRef50_O23024	3e-156	Normal: 110	6	165	774	133	36	BlastAnno: 1258	UniRef50_O23024	437	58.18	385	151	3	3	1157	37	411	3e-156	457
1.14.13.11(trans-cinnamate 4-monooxygenase;)																					
ABCDEF_71482	UniRef50_P92994	0.0	Normal: 2048	1264	3828	9981	3885	695	BlastAnno: 1893	UniRef50_P92994	505	84.49	490	76	0	107	1576	14	503	0.0	797
ABCDEF_41092	UniRef50_P92994	0.0	Normal: 2785	1577	4558	11362	4868	724	BlastAnno: 2059	UniRef50_P92994	505	83.70	503	82	0	1826	318	1	503	0.0	788
ABCDEF_41091	UniRef50_P92994	0.0	Normal: 946	733	2221	5924	1901	1102	BlastAnno: 1557	UniRef50_P92994	505	87.56	442	55	0	1556	231	62	503	0.0	754
ABCDEF_41093	UniRef50_P92994	0.0	Normal: 1164	920	2732	7588	2245	1278	BlastAnno: 1828	UniRef50_P92994	505	86.56	491	66	0	1722	250	14	504	0.0	814
1.14.13.14(trans-cinnamate 2-monooxygenase;)																					
No Correspond Unigene																					
1.14.13.36(5-O-(4-coumaroyl)-D-quinate 3'-monooxygenase;)																					
No Correspond Unigene																					
1.2.1.44(cinnamoyl-CoA reductase;)																					
ABCDEF_38603	UniRef50_Q8H1H5	3e-106	Normal: 391	237	554	703	145	1672	BlastAnno: 1349	UniRef50_Q8H1H5	221	74.09	220	56	1	106	765	1	219	3e-106	323
1.2.1.68(coniferyl-aldehyde dehydrogenase)																					
No Correspond Unigene																					
2.1.1.-(Transferases;)																					
ABCDEF_20806	UniRef50_Q94II3	0.0	Normal: 700	332	876	1458	1997	929	BlastAnno: 2275	UniRef50_Q94II3	600	73.57	594	153	4	2119	341	1	591	0.0	907
ABCDEF_45802	UniRef50_Q9ZPH9	0.0	Normal: 3543	3348	4296	9797	10830	5017	BlastAnno: 2642	UniRef50_Q9ZPH9	633	53.90	603	261	7	2216	456	23	624	0.0	676
ABCDEF_20070	UniRef50_Q944R7	0.0	Normal: 301	141	265	207	185	187	BlastAnno: 2675	UniRef50_Q944R7	724	58.28	719	275	9	127	2265	13	712	0.0	840
ABCDEF_34956	UniRef50_Q94II3	0.0	Normal: 1120	7269	2475	16033	22219	21461	BlastAnno: 2676	UniRef50_Q94II3	600	73.77	610	146	4	404	2230	1	597	0.0	957
ABCDEF_17121	UniRef50_Q9ZPH9	0.0	Normal: 124	77	109	284	199	132	BlastAnno: 2066	UniRef50_Q9ZPH9	633	53.24	601	259	8	161	1903	25	623	0.0	644
ABCDEF_6524	UniRef50_Q9ZPH9	0.0	Normal: 471	296	490	753	870	730	BlastAnno: 2602	UniRef50_Q9ZPH9	633	50.89	619	290	5	2221	404	6	623	0.0	659
ABCDEF_56017	UniRef50_Q9ZPH9	0.0	Normal: 302	167	286	596	748	529	BlastAnno: 2384	UniRef50_Q9ZPH9	633	51.37	619	287	5	213	2030	6	623	0.0	663
ABCDEF_44169	UniRef50_Q9SIZ3	0.0	Normal: 179	158	273	705	685	229	BlastAnno: 2300	UniRef50_Q9SIZ3	589	62.01	308	108	4	1219	296	285	583	0.0	394
ABCDEF_6521	UniRef50_Q9ZPH9	0.0	Normal: 339	198	314	641	767	582	BlastAnno: 2511	UniRef50_Q9ZPH9	633	51.37	619	287	5	2172	355	6	623	0.0	663
ABCDEF_44172	UniRef50_Q9SIZ3	0.0	Normal: 204	184	287	767	741	242	BlastAnno: 2317	UniRef50_Q9SIZ3	589	68.15	449	134	4	1637	291	144	583	0.0	641
ABCDEF_199	UniRef50_Q9SU94	0.0	Normal: 1076	275	1009	558	1247	306	BlastAnno: 1769	UniRef50_Q9SU94	390	78.45	348	74	1	284	1324	43	390	0.0	584
ABCDEF_52252	UniRef50_Q84W92	0.0	Normal: 1658	720	1653	1980	2289	1601	BlastAnno: 2170	UniRef50_Q84W92	535	72.49	469	120	3	409	1791	34	501	0.0	703
ABCDEF_51404	UniRef50_A3BMN9	0.0	Normal: 864	188	716	337	631	326	BlastAnno: 2192	UniRef50_A3BMN9	620	50.50	604	243	9	2008	251	55	620	0.0	555
ABCDEF_21386	UniRef50_Q94AI4	0.0	Normal: 2326	1250	2220	4294	2920	1983	BlastAnno: 3701	UniRef50_Q94AI4	775	47.27	842	332	16	567	2978	8	775	0.0	577
ABCDEF_35101	UniRef50_Q9MAT5	0.0	Normal: 1440	420	1268	576	1136	850	BlastAnno: 1634	UniRef50_Q9MAT5	383	69.03	352	109	0	1449	394	32	383	0.0	550
ABCDEF_960	UniRef50_Q9LN50	0.0	Normal: 3504	13198	3609	11156	19651	12155	BlastAnno: 2626	UniRef50_Q9LN50	724	49.14	525	246	5	883	2451	219	724	0.0	570
ABCDEF_48082	UniRef50_Q9LN50	0.0	Normal: 410	445	467	870	993	454	BlastAnno: 2340	UniRef50_Q9LN50	724	70.25	521	155	0	710	2272	204	724	0.0	821

ABCDEF_10473	UniRef50_Q08A71	0.0	Normal: 374	187	332	425	456	314	BlastAnno: 1764	UniRef50_Q08A71	435	79.23	366	75	1	344	1438	70	435	0.0	575	
ABCDEF_7295	UniRef50_Q9ZPH9	0.0	Normal: 25	54	30	81	26	329	BlastAnno: 1191	UniRef50_Q9ZPH9	633	69.23	403	110	4	1191	22	225	626	0.0	599	
ABCDEF_4160	UniRef50_Q94II3	0.0	Normal: 189	0	210	71	17	72	BlastAnno: 1424	UniRef50_Q94II3	600	77.28	449	101	1	1424	81	143	591	0.0	758	
ABCDEF_44857	UniRef50_Q9ZPH9	0.0	Normal: 101	224	103	299	588	350	BlastAnno: 1582	UniRef50_Q9ZPH9	633	72.06	519	143	1	1571	21	114	632	0.0	822	
ABCDEF_71541	UniRef50_Q9SIZ3	0.0	Normal: 187	167	296	765	724	237	BlastAnno: 2268	UniRef50_Q9SIZ3	589	62.01	308	108	4	1210	287	285	583	0.0	394	
ABCDEF_44171	UniRef50_Q9SIZ3	0.0	Normal: 214	196	312	827	802	251	BlastAnno: 2339	UniRef50_Q9SIZ3	589	64.78	548	182	5	1978	335	47	583	0.0	717	
ABCDEF_20805	UniRef50_Q94II3	0.0	Normal: 674	344	885	1417	2194	963	BlastAnno: 2400	UniRef50_Q94II3	600	78.21	436	94	1	879	2183	156	591	0.0	734	
ABCDEF_18210	UniRef50_Q9SIZ3	0.0	Normal: 5584	3714	7379	17006	21892	7546	BlastAnno: 3199	UniRef50_Q9SIZ3	589	50.00	576	269	7	2145	424	27	585	0.0	574	
ABCDEF_6526	UniRef50_Q9ZPH9	0.0	Normal: 627	185	837	2717	2815	1530	BlastAnno: 2611	UniRef50_Q9ZPH9	633	54.76	599	259	5	462	2222	25	623	0.0	689	
ABCDEF_6525	UniRef50_Q9ZPH9	0.0	Normal: 602	165	760	2369	2605	1400	BlastAnno: 2545	UniRef50_Q9ZPH9	633	54.92	599	258	5	431	2191	25	623	0.0	689	
ABCDEF_6523	UniRef50_Q9ZPH9	0.0	Normal: 314	175	304	641	784	552	BlastAnno: 2312	UniRef50_Q9ZPH9	633	52.42	599	272	4	201	1958	25	623	0.0	663	
ABCDEF_67393	UniRef50_Q9ZPH9	0.0	Normal: 434	245	444	608	768	655	BlastAnno: 2346	UniRef50_Q9ZPH9	633	51.92	599	275	4	232	1989	25	623	0.0	659	
ABCDEF_3579	UniRef50_O14744	0.0	Normal: 1361	326	984	568	791	440	BlastAnno: 2369	UniRef50_O14744	637	47.00	634	294	10	267	2120	30	637	0.0	553	
2.1.1.104(caffeoyl-CoA O-methyltransferase;)																						
ABCDEF_62277	UniRef50_O65862	2e-162	Normal: 19	362	17	166	258	203	BlastAnno: 1005	UniRef50_O65862	247	89.84	246	20	1	103	825	1	246	2e-162	462	
ABCDEF_34441	UniRef50_O65862	2e-162	Normal: 22	354	16	164	260	194	BlastAnno: 992	UniRef50_O65862	247	89.84	246	20	1	103	825	1	246	2e-162	462	
2.1.1.68(caffeate O-methyltransferase;)																						
ABCDEF_3867	UniRef50_Q00763	0.0	Normal: 43	1755	68	456	3254	474	BlastAnno: 1421	UniRef50_Q00763	365	81.04	364	68	1	1351	260	1	363	0.0	619	
2.3.1.133(shikimate O-hydroxycinnamoyltransferase;)																						
ABCDEF_31225	UniRef50_B9GVM3	0.0	Normal: 101	1473	304	2685	639	932	BlastAnno: 1783	UniRef50_B9GVM3	457	80.32	432	85	0	1664	369	24	455	0.0	741	
ABCDEF_17902	UniRef50_B9GVM3	0.0	Normal: 586	820	1166	3765	692	1830	BlastAnno: 1735	UniRef50_B9GVM3	457	59.28	442	174	2	172	1482	17	457	0.0	534	
ABCDEF_34985	UniRef50_B9GVM3	0.0	Normal: 2887	3227	1901	629	1634	3256	BlastAnno: 1801	UniRef50_B9GVM3	457	61.47	436	163	1	184	1476	20	455	0.0	577	
2.3.1.91(sinapoylglucose---choline O-sinapoyltransferase;)																						
No Correspond Unigene																						
2.3.1.92(sinapoylglucose---malate O-sinapoyltransferase;)																						
No Correspond Unigene																						
2.3.1.99(quinate O-hydroxycinnamoyltransferase;)																						
No Correspond Unigene																						
2.4.1.111(coniferyl-alcohol glucosyltransferase;)																						
No Correspond Unigene																						
2.4.1.114(2-coumarate O-beta-glucosyltransferase;)																						
No Correspond Unigene																						
2.4.1.120(sinapate 1-glucosyltransferase;)																						
No Correspond Unigene																						
2.4.1.128(scopoletin glucosyltransferase;)																						
No Correspond Unigene																						
3.1.1.49(sinapine esterase;)																						
No Correspond Unigene																						
3.2.1.126(coniferin beta-glucosidase;)																						
No Correspond Unigene																						
3.2.1.21(beta-glucosidase;)																						
ABCDEF_24901	UniRef50_Q9LV34	0.0	Normal: 696	459	604	917	1518	3129	BlastAnno: 1698	UniRef50_Q9LV34	495	69.82	487	133	4	1576	131	17	494	0.0	696	
ABCDEF_3707	UniRef50_Q8L7J2	0.0	Normal: 55	614	80	478	1267	616	BlastAnno: 1950	UniRef50_Q8L7J2	521	68.39	484	143	2	365	1786	36	519	0.0	738	
ABCDEF_50691	UniRef50_O80690	0.0	Normal: 29	230	24	31	176	222	BlastAnno: 1737	UniRef50_O80690	527	56.05	512	197	4	1723	194	39	524	0.0	593	
ABCDEF_6978	UniRef50_Q9LV34	0.0	Normal: 132	59	114	74	74	76	BlastAnno: 1368	UniRef50_Q9LV34	495	65.54	444	136	5	1311	1	27	460	0.0	616	
ABCDEF_7820	UniRef50_B3H5Q1	0.0	Normal: 1199	176	254	446	677	1138	BlastAnno: 1699	UniRef50_B3H5Q1	521	55.14	486	190	4	97	1470	25	510	0.0	549	
ABCDEF_52515	UniRef50_O64882	0.0	Normal: 14	184	426	593	18106	970	BlastAnno: 1946	UniRef50_O64882	517	55.39	482	214	1	128	1570	32	513	0.0	574	
ABCDEF_50689	UniRef50_O80690	0.0	Normal: 42	287	26	32	235	244	BlastAnno: 1949	UniRef50_O80690	527	57.58	521	205	4	1711	194	5	524	0.0	608	
ABCDEF_24900	UniRef50_Q9LV34	0.0	Normal: 661	448	581	908	1414	2839	BlastAnno: 1678	UniRef50_Q9LV34	495	69.82	487	133	4	1557	112	17	494	0.0	696	
ABCDEF_9495	UniRef50_O80690	0.0	Normal: 261	104	1144	3869	396	26	BlastAnno: 1691	UniRef50_O80690	527	52.12	495	196	7	220	1686	59	518	0.0	533	
ABCDEF_9464	UniRef50_O80690	0.0	Normal: 733	82	1949	4567	726	6	BlastAnno: 2104	UniRef50_O80690	527	53.91	473	206	6	269	1669	59	525	0.0	542	
4.1.1.-(Lyases;)																						
ABCDEF_57496	UniRef50_O96569	9e-20	Normal: 4	0	0	0	0	9	BlastAnno: 223	UniRef50_O96569	439	66.67	72	22	1	210	1	19	90	9e-20	87.4	
4.3.1.23(tyrosine ammonia-lyase;)																						
No Correspond Unigene																						
4.3.1.24(phenylalanine ammonia-lyase;)																						
ABCDEF_34414	UniRef50_Q6GZ04	0.0	Normal: 1997	3260	3067	14209	6957	2185	BlastAnno: 2627	UniRef50_Q6GZ04	698	47.67	644	318	7	2160	253	48	680	0.0	591	
ABCDEF_35119	UniRef50_Q6GZ04	0.0	Normal: 2644	1363	3193	6969	3673	1650	BlastAnno: 2515	UniRef50_Q6GZ04	698	46.71	683	344	7	264	2285	9	680	0.0	597	
ABCDEF_21563	UniRef50_C5YCD7	0.0	Normal: 707	225	993	972	1864	182	BlastAnno: 1483	UniRef50_C5YCD7	688	74.23	489	100	1	1482	16	225	687	0.0	758	
ABCDEF_59755	UniRef50_Q6GZ04	0.0	Normal: 1915	2750	3275	9429	2466	3052	BlastAnno: 2458	UniRef50_Q6GZ04	698	46.86	685	338	10	2178	163	9	680	0.0	595	
ABCDEF_7556	UniRef50_Q6GZ04	0.0	Normal: 2012	2842	3468	9812	2712	3290	BlastAnno: 2512	UniRef50_Q6GZ04	698	46.43	700	349	10	285	2345	9	695	0.0	595	
4.3.1.25(phenylalanine/tyrosine ammonia-lyase;)																						
No Correspond Unigene																						
5.2.1.-(Isomerases;)																						
No Correspond Unigene																						
6.2.1.12(4-coumarate---CoA ligase;)																						
ABCDEF_52459	UniRef50_Q9LU36	0.0	Normal: 157	704	147	540	677	726	BlastAnno: 1521	UniRef50_Q9LU36	570	65.20	431	139	3	9	1274	136	564	0.0	554	
ABCDEF_41628	UniRef50_Q9LU36	0.0	Normal: 140	1561	151	856	1760	1204	BlastAnno: 2088	UniRef50_Q9LU36	570	60.04	548	203	5	152	1753	21	566	0.0	660	
>00941 Flavonoid biosynthesis																						
1.1.1.219(dihydrokaempferol 4-reductase;)																						
ABCDEF_31933	UniRef50_B9S058	2e-167	Normal: 265	160	160	96	243	496	BlastAnno: 1034	UniRef50_B9S058	334	78.38	296	60	2	4	885	34	327	2e-167	479	
1.1.1.234(flavanone 4-reductase)																						

No Correspond Unigene																					
1.14.11.19(leucocyanidin oxygenase;)																					
ABCDEF_4484	UniRef50_Q96323	0.0	Normal: 472	0	4079	17694	899	0	BlastAnno: 1445	UniRef50_Q96323	356	77.91	344	76	0	124	1155	6	349	0.0	525
1.14.11.22(flavone synthase)																					
No Correspond Unigene																					
1.14.11.23(flavonol synthase)																					
ABCDEF_3215	UniRef50_B9RKI5	1e-168	Normal: 124	110	105	152	146	193	BlastAnno: 1426	UniRef50_B9RKI5	340	74.92	319	80	0	186	1142	17	335	1e-168	488
1.14.11.9(flavanone 3-dioxygenase;)																					
ABCDEF_27750	UniRef50_Q9S818	0.0	Normal: 1057	31	3292	11145	6466	73	BlastAnno: 1409	UniRef50_Q9S818	358	80.56	360	61	2	1354	275	5	355	0.0	607
ABCDEF_74599	UniRef50_Q9S818	0.0	Normal: 837	26	2851	8754	4660	95	BlastAnno: 1257	UniRef50_Q9S818	358	83.58	341	55	1	1202	180	5	344	0.0	601
ABCDEF_27751	UniRef50_Q9S818	0.0	Normal: 478	392	918	1437	3714	607	BlastAnno: 1227	UniRef50_Q9S818	358	76.25	341	80	1	1180	158	5	344	0.0	553
ABCDEF_41152	UniRef50_Q9S818	0.0	Normal: 164	0	575	2160	56	3	BlastAnno: 1228	UniRef50_Q9S818	358	78.39	361	73	3	1163	84	1	357	0.0	578
ABCDEF_27748	UniRef50_Q9S818	0.0	Normal: 1510	32	4126	14241	8425	56	BlastAnno: 1434	UniRef50_Q9S818	358	81.06	359	60	1	90	1166	5	355	0.0	612
1.14.13.-(Oxidoreductases;)																					
ABCDEF_45538	UniRef50_O23024	3e-156	Normal: 110	6	165	774	133	36	BlastAnno: 1258	UniRef50_O23024	437	58.18	385	151	3	3	1157	37	411	3e-156	457
1.14.13.11(trans-cinnamate 4-monooxygenase;)																					
ABCDEF_71482	UniRef50_P92994	0.0	Normal: 2048	1264	3828	9981	3885	695	BlastAnno: 1893	UniRef50_P92994	505	84.49	490	76	0	107	1576	14	503	0.0	797
ABCDEF_41092	UniRef50_P92994	0.0	Normal: 2785	1577	4558	11362	4868	724	BlastAnno: 2059	UniRef50_P92994	505	83.70	503	82	0	1826	318	1	503	0.0	788
ABCDEF_41091	UniRef50_P92994	0.0	Normal: 946	733	2221	5924	1901	1102	BlastAnno: 1557	UniRef50_P92994	505	87.56	442	55	0	1556	231	62	503	0.0	754
ABCDEF_41093	UniRef50_P92994	0.0	Normal: 1164	920	2732	7588	2245	1278	BlastAnno: 1828	UniRef50_P92994	505	86.56	491	66	0	1722	250	14	504	0.0	814
1.14.13.21(flavonoid 3'-monooxygenase;)																					
ABCDEF_35618	UniRef50_B9SY12	0.0	Normal: 28	2648	17	142	1234	902	BlastAnno: 1746	UniRef50_B9SY12	482	63.04	506	155	3	1649	132	9	482	0.0	637
ABCDEF_21579	UniRef50_B9SY12	0.0	Normal: 1005	643	1606	4372	3226	1621	BlastAnno: 1895	UniRef50_B9SY12	482	65.94	502	140	2	185	1690	12	482	0.0	655
ABCDEF_18295	UniRef50_B9R991	0.0	Normal: 3667	1934	4977	10491	4588	1917	BlastAnno: 2006	UniRef50_B9R991	511	55.28	492	213	4	217	1686	22	508	0.0	564
ABCDEF_45242	UniRef50_Q9SD85	0.0	Normal: 2190	674	4992	10924	6248	146	BlastAnno: 1929	UniRef50_Q9SD85	513	65.01	523	165	7	73	1626	1	510	0.0	661
1.14.13.36(5-O-(4-coumaroyl)-D-quinate 3'-monooxygenase;)																					
No Correspond Unigene																					
1.14.13.88(flavonoid 3',5'-hydroxylase)																					
ABCDEF_37738	UniRef50_B9SRN0	3e-174	Normal: 26	2447	19	30	33	993	BlastAnno: 1837	UniRef50_B9SRN0	524	50.64	466	227	1	1634	246	56	521	3e-174	514
1.17.1.3(leucoanthocyanidin reductase;)																					
ABCDEF_18485	UniRef50_Q84V83	9e-80	Normal: 390	3288	770	723	18366	1745	BlastAnno: 1311	UniRef50_Q84V83	382	43.32	307	171	2	1160	246	12	317	9e-80	260
1.21.3.6(aureusidin synthase)																					
No Correspond Unigene																					
1.3.1.77(anthocyanidin reductase;)																					
ABCDEF_58207	UniRef50_Q9SEV0	1e-56	Normal: 141	0	1397	4774	261	0	BlastAnno: 1159	UniRef50_Q9SEV0	340	40.15	274	152	4	1157	366	66	337	1e-56	196
2.1.1.-(Transferases;)																					
ABCDEF_20806	UniRef50_Q94II3	0.0	Normal: 700	332	876	1458	1997	929	BlastAnno: 2275	UniRef50_Q94II3	600	73.57	594	153	4	2119	341	1	591	0.0	907
ABCDEF_45802	UniRef50_Q9ZPH9	0.0	Normal: 3543	3348	4296	9797	10830	5017	BlastAnno: 2642	UniRef50_Q9ZPH9	633	53.90	603	261	7	2216	456	23	624	0.0	676
ABCDEF_20070	UniRef50_Q944R7	0.0	Normal: 301	141	265	207	185	187	BlastAnno: 2675	UniRef50_Q944R7	724	58.28	719	275	9	127	2265	13	712	0.0	840
ABCDEF_34956	UniRef50_Q94II3	0.0	Normal: 1120	7269	2475	16033	22219	21461	BlastAnno: 2676	UniRef50_Q94II3	600	73.77	610	146	4	404	2230	1	597	0.0	957
ABCDEF_17121	UniRef50_Q9ZPH9	0.0	Normal: 124	77	109	284	199	132	BlastAnno: 2066	UniRef50_Q9ZPH9	633	53.24	601	259	8	161	1903	25	623	0.0	644
ABCDEF_6524	UniRef50_Q9ZPH9	0.0	Normal: 471	296	490	753	870	730	BlastAnno: 2602	UniRef50_Q9ZPH9	633	50.89	619	290	5	2221	404	6	623	0.0	659
ABCDEF_56017	UniRef50_Q9ZPH9	0.0	Normal: 302	167	286	596	748	529	BlastAnno: 2384	UniRef50_Q9ZPH9	633	51.37	619	287	5	213	2030	6	623	0.0	663
ABCDEF_44169	UniRef50_Q9SIZ3	0.0	Normal: 179	158	273	705	685	229	BlastAnno: 2300	UniRef50_Q9SIZ3	589	62.01	308	108	4	1219	296	285	583	0.0	394
ABCDEF_6521	UniRef50_Q9ZPH9	0.0	Normal: 339	198	314	641	767	582	BlastAnno: 2511	UniRef50_Q9ZPH9	633	51.37	619	287	5	2172	355	6	623	0.0	663
ABCDEF_44172	UniRef50_Q9SIZ3	0.0	Normal: 204	184	287	767	741	242	BlastAnno: 2317	UniRef50_Q9SIZ3	589	68.15	449	134	4	1637	291	144	583	0.0	641
ABCDEF_199	UniRef50_Q9SU94	0.0	Normal: 1076	275	1009	558	1247	306	BlastAnno: 1769	UniRef50_Q9SU94	390	78.45	348	74	1	284	1324	43	390	0.0	584
ABCDEF_52252	UniRef50_Q84W92	0.0	Normal: 1658	720	1653	1980	2289	1601	BlastAnno: 2170	UniRef50_Q84W92	535	72.49	469	120	3	409	1791	34	501	0.0	703
ABCDEF_51404	UniRef50_A3BMN9	0.0	Normal: 864	188	716	337	631	326	BlastAnno: 2192	UniRef50_A3BMN9	620	50.50	604	243	9	2008	251	55	620	0.0	555
ABCDEF_21386	UniRef50_Q94AI4	0.0	Normal: 2326	1250	2220	4294	2920	1983	BlastAnno: 3701	UniRef50_Q94AI4	775	47.27	842	332	16	567	2978	8	775	0.0	577
ABCDEF_35101	UniRef50_Q9MAT5	0.0	Normal: 1440	420	1268	576	1136	850	BlastAnno: 1634	UniRef50_Q9MAT5	383	69.03	352	109	0	1449	394	32	383	0.0	550
ABCDEF_960	UniRef50_Q9LN50	0.0	Normal: 3504	13198	3609	11156	19651	12155	BlastAnno: 2626	UniRef50_Q9LN50	724	49.14	525	246	5	883	2451	219	724	0.0	570
ABCDEF_48082	UniRef50_Q9LN50	0.0	Normal: 410	445	467	870	993	454	BlastAnno: 2340	UniRef50_Q9LN50	724	70.25	521	155	0	710	2272	204	724	0.0	821
ABCDEF_10473	UniRef50_Q08A71	0.0	Normal: 374	187	332	425	456	314	BlastAnno: 1764	UniRef50_Q08A71	435	79.23	366	75	1	344	1438	70	435	0.0	575
ABCDEF_7295	UniRef50_Q9ZPH9	0.0	Normal: 25	54	30	81	26	329	BlastAnno: 1191	UniRef50_Q9ZPH9	633	69.23	403	110	4	1191	22	225	626	0.0	599
ABCDEF_4160	UniRef50_Q94II3	0.0	Normal: 189	0	210	71	17	72	BlastAnno: 1424	UniRef50_Q94II3	600	77.28	449	101	1	1424	81	143	591	0.0	758
ABCDEF_44857	UniRef50_Q9ZPH9	0.0	Normal: 101	224	103	299	588	350	BlastAnno: 1582	UniRef50_Q9ZPH9	633	72.06	519	143	1	1571	21	114	632	0.0	822
ABCDEF_71541	UniRef50_Q9SIZ3	0.0	Normal: 187	167	296	765	724	237	BlastAnno: 2268	UniRef50_Q9SIZ3	589	62.01	308	108	4	1210	287	285	583	0.0	394
ABCDEF_44171	UniRef50_Q9SIZ3	0.0	Normal: 214	196	312	827	802	251	BlastAnno: 2339	UniRef50_Q9SIZ3	589	64.78	548	182	5	1978	335	47	583	0.0	717
ABCDEF_20805	UniRef50_Q94II3	0.0	Normal: 674	344	885	1417	2194	963	BlastAnno: 2400	UniRef50_Q94II3	600	78.21	436	94	1	879	2183	156	591	0.0	734
ABCDEF_18210	UniRef50_Q9SIZ3	0.0	Normal: 5584	3714	7379	17006	21892	7546	BlastAnno: 3199	UniRef50_Q9SIZ3	589	50.00	576	269	7	2145	424	27	585	0.0	574
ABCDEF_6526	UniRef50_Q9ZPH9	0.0	Normal: 627	185	837	2717	2815	1530	BlastAnno: 2611	UniRef50_Q9ZPH9	633	54.76	599	259	5	462	2222	25	623	0.0	689
ABCDEF_6525	UniRef50_Q9ZPH9	0.0	Normal: 602	165	760	2369	2605	1400	BlastAnno: 2545	UniRef50_Q9ZPH9	633	54.92	599	258	5	431	2191	25	623	0.0	689
ABCDEF_6523	UniRef50_Q9ZPH9	0.0	Normal: 314	175	304	641	784	552	BlastAnno: 2312	UniRef50_Q9ZPH9	633	52.42	599	272	4	201	1958	25	623	0.0	663
ABCDEF_67393	UniRef50_Q9ZPH9	0.0	Normal: 434	245	444	608	768	655	BlastAnno: 2346	UniRef50_Q9ZPH9	633	51.92	599	275	4	232	1989	25	623	0.0	659
ABCDEF_3579	UniRef50_O14744	0.0	Normal: 1361	326	984	568	791	440	BlastAnno: 2369	UniRef50_O14744	637	47.00	634	294	10	267	2120	30	637	0.0	553
2.1.1.104(caffeoyl-CoA O-methyltransferase;)																					
ABCDEF_62277	UniRef50_O65862	2e-162	Normal: 19	362	17	166	258	203	BlastAnno: 1005	UniRef50_O65862	247	89.84	246	20	1	103	825	1	246	2e-162	462
ABCDEF_34441	UniRef50_O65862	2e-162	Normal: 22	354	16	164	260	194	BlastAnno: 992	UniRef50_O65862	247	89.84	246	20	1	103	825	1	246	2e-162	462
2.3.1.133(shikimate O-hydroxycinnamoyltransferase;)																					
ABCDEF_31225	UniRef50_B9GVM3	0.0	Normal: 101	1473	304	2685	639	932	BlastAnno: 1783	UniRef50_B9GVM3	457	80.32	432	85	0	1664	369	24	455	0.0	741
ABCDEF_17902	UniRef50_B9GVM3	0.0	Normal: 586	820	1166	3765	692	1830	BlastAnno: 1735	UniRef50_B9GVM3	457	59.28	442	174	2	172	1482	17	457	0.0	534

ABCDEF_34985	UniRef50_B9GVM3	0.0	Normal: 2887	3227	1901	629	1634	3256	BlastAnno: 1801	UniRef50_B9GVM3	457	61.47	436	163	1	184	1476	20	455	0.0	577
2.3.1.170(6'-deoxychalcone synthase)																					
ABCDEF_42198	UniRef50_P26690	7e-113	Normal: 311	470	413	606	1080	440	BlastAnno: 1263	UniRef50_P26690	315	57.05	298	117	4	1153	269	8	297	7e-113	342
2.3.1.74(naringenin-chalcone synthase;)																					
ABCDEF_38379	UniRef50_P13114	0.0	Normal: 123	1	1680	7195	1325	6	BlastAnno: 1460	UniRef50_P13114	395	85.03	334	48	2	1234	239	61	394	0.0	568
ABCDEF_22514	UniRef50_P13114	0.0	Normal: 783	0	236	0	421	0	BlastAnno: 1516	UniRef50_P13114	395	67.28	382	124	1	1350	208	14	395	0.0	542
ABCDEF_52368	UniRef50_P13114	0.0	Normal: 5935	229	6666	2345	25408	350	BlastAnno: 1734	UniRef50_P13114	395	83.94	386	61	1	1514	360	8	393	0.0	669
ABCDEF_38380	UniRef50_P13114	0.0	Normal: 214	6	3245	19196	2381	10	BlastAnno: 1526	UniRef50_P13114	395	83.72	387	61	2	134	1288	8	394	0.0	660
2.4.1.-(Transferases;)																					
ABCDEF_18004	UniRef50_Q6YWK8	0.0	Normal: 1562	1736	2009	3452	4438	2400	BlastAnno: 2298	UniRef50_Q6YWK8	570	65.27	429	136	1	1941	694	57	485	0.0	608
ABCDEF_52379	UniRef50_Q9LHZ7	0.0	Normal: 4015	4921	4546	10520	13430	4731	BlastAnno: 3479	UniRef50_Q9LHZ7	1170	48.04	893	342	16	825	3242	299	1156	0.0	787
ABCDEF_48593	UniRef50_Q9SK82	0.0	Normal: 12	420	19	343	763	253	BlastAnno: 1834	UniRef50_Q9SK82	489	60.08	481	186	4	1714	278	10	486	0.0	633
ABCDEF_13375	UniRef50_Q8VZK9	0.0	Normal: 108	123	208	526	34	294	BlastAnno: 1738	UniRef50_Q8VZK9	729	53.62	552	234	8	94	1737	18	551	0.0	582
ABCDEF_40982	UniRef50_Q96301	0.0	Normal: 1037	706	819	1249	1084	920	BlastAnno: 3418	UniRef50_Q96301	914	79.60	902	173	3	243	2948	1	891	0.0	1506
ABCDEF_47674	UniRef50_Q9LHZ7	0.0	Normal: 274	92	202	599	505	280	BlastAnno: 2526	UniRef50_Q9LHZ7	1170	75.93	777	151	8	2260	2	15	779	0.0	1177
ABCDEF_34097	UniRef50_Q9LE59	0.0	Normal: 626	613	923	1797	1829	692	BlastAnno: 2521	UniRef50_Q9LE59	673	60.92	458	178	1	1687	317	215	672	0.0	630
ABCDEF_34098	UniRef50_Q9LE59	0.0	Normal: 457	464	697	1283	1312	539	BlastAnno: 2199	UniRef50_Q9LE59	673	60.92	458	178	1	1687	317	215	672	0.0	628
ABCDEF_10848	UniRef50_Q9M156	0.0	Normal: 157	2988	163	408	772	1566	BlastAnno: 1810	UniRef50_Q9M156	480	60.09	456	176	4	1700	336	4	454	0.0	553
ABCDEF_41182	UniRef50_Q9LHZ7	0.0	Normal: 150	2169	221	814	1407	495	BlastAnno: 3604	UniRef50_Q9LHZ7	1170	51.03	870	333	15	2822	456	299	1156	0.0	826
ABCDEF_40981	UniRef50_Q96301	0.0	Normal: 156	97	110	160	179	173	BlastAnno: 1225	UniRef50_Q96301	914	86.92	344	45	0	1112	81	394	737	0.0	652
ABCDEF_9506	UniRef50_Q9LHZ7	0.0	Normal: 1302	2128	1494	3356	4644	2206	BlastAnno: 3751	UniRef50_Q9LHZ7	1170	49.38	891	343	17	927	3359	299	1161	0.0	812
ABCDEF_9500	UniRef50_Q9LHZ7	0.0	Normal: 183	188	186	576	498	260	BlastAnno: 1717	UniRef50_Q9LHZ7	1170	47.98	644	234	14	3	1715	418	1033	0.0	571
ABCDEF_100	UniRef50_Q9M8Y0	0.0	Normal: 228	309	224	416	461	141	BlastAnno: 2109	UniRef50_Q9M8Y0	977	77.49	533	120	0	1610	12	50	582	0.0	884
ABCDEF_33659	UniRef50_Q9LHZ7	0.0	Normal: 1282	1948	2248	5826	10642	2083	BlastAnno: 4289	UniRef50_Q9LHZ7	1170	48.70	887	345	15	2955	538	299	1156	0.0	788
ABCDEF_37667	UniRef50_Q9LHZ7	0.0	Normal: 248	3617	251	1237	2078	970	BlastAnno: 3161	UniRef50_Q9LHZ7	1170	49.72	885	335	14	2671	260	301	1156	0.0	810
ABCDEF_47673	UniRef50_Q9LHZ7	0.0	Normal: 876	296	828	1628	1721	1172	BlastAnno: 3878	UniRef50_Q9LHZ7	1170	80.88	1161	186	8	3642	232	15	1163	0.0	1865
ABCDEF_99	UniRef50_Q9M8Y0	0.0	Normal: 272	330	222	398	423	145	BlastAnno: 2094	UniRef50_Q9M8Y0	977	77.36	561	127	0	1683	1	50	610	0.0	929
ABCDEF_20658	UniRef50_Q8VZK9	0.0	Normal: 373	33	193	64	503	141	BlastAnno: 2316	UniRef50_Q8VZK9	729	53.35	508	221	7	47	1564	11	504	0.0	559
ABCDEF_10183	UniRef50_A7XDQ9	0.0	Normal: 422	455	346	577	378	610	BlastAnno: 2362	UniRef50_A7XDQ9	684	50.94	693	307	12	281	2287	1	684	0.0	670
ABCDEF_30745	UniRef50_A7XDQ9	0.0	Normal: 1209	351	1031	1645	1226	635	BlastAnno: 2646	UniRef50_A7XDQ9	684	57.58	554	227	6	546	2189	133	684	0.0	664
ABCDEF_13336	UniRef50_Q7PC69	0.0	Normal: 148	563	194	957	1575	816	BlastAnno: 2584	UniRef50_Q7PC69	745	71.56	559	136	6	744	2351	187	745	0.0	730
ABCDEF_21426	UniRef50_Q8RU27	0.0	Normal: 2658	1979	3417	6157	9464	2599	BlastAnno: 1530	UniRef50_Q8RU27	366	89.44	360	34	2	1358	282	1	357	0.0	667
ABCDEF_71242	UniRef50_Q9ZV71	0.0	Normal: 134	122	179	480	754	119	BlastAnno: 1835	UniRef50_Q9ZV71	409	73.35	409	103	4	231	1439	1	409	0.0	587
ABCDEF_52020	UniRef50_Q6YWK8	0.0	Normal: 1635	1534	1975	4284	10353	2289	BlastAnno: 2394	UniRef50_Q6YWK8	570	58.37	514	185	2	403	1857	57	570	0.0	607
ABCDEF_51269	UniRef50_Q6YWK8	0.0	Normal: 147	85	103	21	33	438	BlastAnno: 1864	UniRef50_Q6YWK8	570	54.28	514	204	2	1646	198	57	570	0.0	586
ABCDEF_30753	UniRef50_Q9ZV71	0.0	Normal: 290	497	417	629	1013	634	BlastAnno: 1502	UniRef50_Q9ZV71	409	72.32	401	107	2	311	1501	1	401	0.0	590
ABCDEF_4233	UniRef50_Q9ZV71	0.0	Normal: 369	412	550	936	860	750	BlastAnno: 2004	UniRef50_Q9ZV71	409	72.13	409	110	2	370	1584	1	409	0.0	613
ABCDEF_17525	UniRef50_Q8RU27	0.0	Normal: 1500	1289	2149	3711	4446	1069	BlastAnno: 1507	UniRef50_Q8RU27	366	91.35	347	30	0	1371	331	3	349	0.0	671
ABCDEF_18005	UniRef50_Q6YWK8	0.0	Normal: 1842	2042	2515	4214	5208	2842	BlastAnno: 2305	UniRef50_Q6YWK8	570	59.14	514	180	2	1948	497	57	570	0.0	642
ABCDEF_41140	UniRef50_Q6YWK8	0.0	Normal: 86	1314	93	753	1787	2076	BlastAnno: 2346	UniRef50_Q6YWK8	570	60.00	510	174	2	431	1870	57	566	0.0	669
ABCDEF_18002	UniRef50_Q9LHZ7	0.0	Normal: 376	127	367	601	1190	520	BlastAnno: 1605	UniRef50_Q9LHZ7	1170	89.75	400	41	0	1603	404	771	1170	0.0	732
ABCDEF_13339	UniRef50_Q7PC69	0.0	Normal: 127	814	183	541	1135	1144	BlastAnno: 2610	UniRef50_Q7PC69	745	70.48	559	141	6	725	2329	187	745	0.0	729
ABCDEF_41141	UniRef50_Q6YWK8	0.0	Normal: 67	1107	84	708	1690	1854	BlastAnno: 2461	UniRef50_Q6YWK8	570	60.00	510	174	2	1916	477	57	566	0.0	668
ABCDEF_44583	UniRef50_Q9LE59	0.0	Normal: 681	561	559	947	1745	789	BlastAnno: 2470	UniRef50_Q9LE59	673	59.21	456	185	1	1749	385	214	669	0.0	621
ABCDEF_3869	UniRef50_Q9LE59	0.0	Normal: 333	393	415	809	907	754	BlastAnno: 2136	UniRef50_Q9LE59	673	59.05	613	227	4	1827	1	81	673	0.0	699
ABCDEF_28947	UniRef50_Q9SK82	0.0	Normal: 0	851	3	0	0	116	BlastAnno: 1567	UniRef50_Q9SK82	489	62.40	484	176	4	1524	85	1	482	0.0	652
ABCDEF_31119	UniRef50_Q9M8Y0	0.0	Normal: 2212	1279	1917	2933	3341	1803	BlastAnno: 3695	UniRef50_Q9M8Y0	977	81.08	925	174	1	392	3163	50	974	0.0	1583
ABCDEF_37311	UniRef50_Q9LE59	0.0	Normal: 175	133	191	549	421	366	BlastAnno: 1612	UniRef50_Q9LE59	673	62.96	486	153	4	1406	3	4	480	0.0	542
ABCDEF_24190	UniRef50_Q8L7F9	0.0	Normal: 397	265	492	583	553	371	BlastAnno: 2564	UniRef50_Q8L7F9	643	63.91	640	218	4	2353	473	1	640	0.0	850
ABCDEF_30548	UniRef50_Q9FH36	0.0	Normal: 17	916	27	151	420	126	BlastAnno: 1963	UniRef50_Q9FH36	535	72.47	534	138	3	102	1676	1	534	0.0	767
ABCDEF_27416	UniRef50_Q7PC69	0.0	Normal: 575	837	875	1344	2015	936	BlastAnno: 2454	UniRef50_Q7PC69	745	66.11	416	115	4	2337	1168	126	541	0.0	518
ABCDEF_48373	UniRef50_A7XDQ9	0.0	Normal: 1364	773	1336	1451	1791	1202	BlastAnno: 3042	UniRef50_A7XDQ9	684	63.68	691	234	6	2405	360	1	683	0.0	915
ABCDEF_3868	UniRef50_Q9LE59	0.0	Normal: 224	274	301	555	686	524	BlastAnno: 1670	UniRef50_Q9LE59	673	65.31	516	176	1	1556	9	161	673	0.0	696
ABCDEF_31288	UniRef50_Q940V3	0.0	Normal: 247	309	183	101	124	655	BlastAnno: 1658	UniRef50_Q940V3	470	56.01	466	197	7	1614	232	8	470	0.0	537
ABCDEF_52574	UniRef50_Q09332	0.0	Normal: 885	474	1068	1835	1853	816	BlastAnno: 3720	UniRef50_Q09332	1548	47.89	687	310	14	2551	533	859	1511	0.0	598
ABCDEF_79752	UniRef50_Q9ZV71	0.0	Normal: 158	183	245	400	379	352	BlastAnno: 1266	UniRef50_Q9ZV71	409	71.39	360	99	2	31	1098	1	360	0.0	526
ABCDEF_9509	UniRef50_Q9LHZ7	0.0	Normal: 1296	2171	1498	3410	4683	2232	BlastAnno: 3897	UniRef50_Q9LHZ7	1170	49.38	891	343	17	1073	3505	299	1161	0.0	812
ABCDEF_48200	UniRef50_Q6YWK8	0.0	Normal: 1768	576	3156	4431	6634	491	BlastAnno: 2274	UniRef50_Q6YWK8	570	57.98	514	185	4	291	1739	57	570	0.0	642
ABCDEF_24155	UniRef50_Q9ZVI7	0.0	Normal: 503	247	607	1676	1833	578	BlastAnno: 2708	UniRef50_Q9ZVI7	619	52.33	621	264	11	306	2123	15	618	0.0	601
ABCDEF_21246	UniRef50_Q9SKT6	0.0	Normal: 92	107	188	380	834	84	BlastAnno: 1791	UniRef50_Q9SKT6	536	56.61	484	204	5	123	1562	52	533	0.0	568
ABCDEF_32317	UniRef50_Q8RU27	0.0	Normal: 12	9	3	118	800	62	BlastAnno: 1076	UniRef50_Q8RU27	366	87.02	285	37	0	1075	221	64	348	0.0	535
ABCDEF_9502	UniRef50_Q9LHZ7	0.0	Normal: 610	486	568	1331	1403	591	BlastAnno: 1961	UniRef50_Q9LHZ7	1170	48.64	625	249	13	3	1742	564	1161	0.0	570
ABCDEF_23298	UniRef50_Q9FH36	0.0	Normal: 128	527	130	223	314	864	BlastAnno: 2832	UniRef50_Q9FH36	535	83.16	196	33	0	696	1283	340	535	0.0	357
ABCDEF_40595	UniRef50_O80898	0.0	Normal: 229	135	103	25	307	325	BlastAnno: 2413	UniRef50_O80898	757	44.68	761	378	13	95	2299	10	753	0.0	587
ABCDEF_63947	UniRef50_Q9SK82	0.0	Normal: 0	267	0	0	71	0	BlastAnno: 1446	UniRef50_Q9SK82	489	58.33	468	189	4	60	1445	1	468	0.0	592
ABCDEF_67847	UniRef50_Q9M8Y0	0.0	Normal: 794	476	871	1081	1401	868	BlastAnno: 1591	UniRef50_Q9M8Y0	977	84.86	370	56	0	1589	480	605	974	0.0	685
ABCDEF_65666	UniRef50_Q09332	0.0	Normal: 800	441	946	1606	1637	735	BlastAnno: 3518	UniRef50_Q09332	1548	47.54	671	305	13	1162	3087	859	1511	0.0	586
ABCDEF_38641	UniRef50_Q9FWA4	0.0	Normal: 674	967	1162	2603	4404	2475	BlastAnno: 1599	UniRef50_Q9FWA4	561	81.35	386	72	0	1	1158	176	561	0.0	641
ABCDEF_17914	UniRef50_Q9SKT6	0.0	Normal: 784	567	886	1447	1439	1424	BlastAnno: 2049	UniRef50_Q9SKT6	536	72.95	536	140	2	204	1796	1	536	0.0	785
ABCDEF_18286	UniRef50_Q6NQB7	0.0	Normal: 1131	1108	1225	1872	1722	3329	BlastAnno: 1752	UniRef50_Q6NQB7	393	73.80	397	81	6	1527	367	10	393	0.0	607
ABCDEF_119	UniRef50_Q8RU27	0.0	Normal: 1613	1155	2249	3558	7663	1775	BlastAnno: 1532	UniRef50_Q8RU27	366	88.33	360	38	2	1306	230	1	357	0.0	663
ABCDEF_47603	UniRef50_Q9M9Y5	0.0	Normal: 2609	8779	1664	1103	2085	22991	BlastAnno: 2830	UniRef50_Q9M9Y5	589	54.04	594	267	3	2475	694	1	588	0.0	654

ABCDEF_24192	UniRef50_Q8L7F9	0.0	Normal: 391	244	465	553	497	345	BlastAnno: 2477	UniRef50_Q8L7F9	643	58.99	434	165	4	212	1474	1	434	0.0	513
ABCDEF_17482	UniRef50_Q9SKT6	0.0	Normal: 615	379	816	1068	1470	852	BlastAnno: 2083	UniRef50_Q9SKT6	536	71.11	540	147	4	1915	311	1	536	0.0	786
ABCDEF_85909	UniRef50_Q8VZK9	0.0	Normal: 28	45	9	10	44	127	BlastAnno: 1473	UniRef50_Q8VZK9	729	55.37	475	198	6	1419	1	11	473	0.0	536
ABCDEF_9503	UniRef50_Q9LHZ7	0.0	Normal: 487	544	524	1242	1320	833	BlastAnno: 2087	UniRef50_Q9LHZ7	1170	50.35	574	236	8	57	1712	615	1161	0.0	567
ABCDEF_40594	UniRef50_O80898	0.0	Normal: 256	127	102	41	331	326	BlastAnno: 2417	UniRef50_O80898	757	45.76	719	348	12	213	2291	51	753	0.0	585
ABCDEF_17141	UniRef50_Q9LHZ7	0.0	Normal: 74	1734	69	380	701	333	BlastAnno: 3337	UniRef50_Q9LHZ7	1170	50.92	868	335	14	2539	179	299	1156	0.0	816
ABCDEF_33658	UniRef50_Q9LHZ7	0.0	Normal: 384	308	395	805	1003	602	BlastAnno: 2961	UniRef50_Q9LHZ7	1170	48.76	888	343	14	204	2621	299	1156	0.0	792
ABCDEF_20579	UniRef50_Q7PC69	0.0	Normal: 1007	795	1238	2311	2309	1345	BlastAnno: 3113	UniRef50_Q7PC69	745	59.97	767	229	14	2667	535	1	745	0.0	768
ABCDEF_31118	UniRef50_Q9M8Y0	0.0	Normal: 2135	1267	1900	3004	3329	1757	BlastAnno: 3787	UniRef50_Q9M8Y0	977	81.10	709	133	1	1132	3255	266	974	0.0	1217
ABCDEF_9499	UniRef50_Q9LHZ7	0.0	Normal: 205	207	215	632	538	275	BlastAnno: 1794	UniRef50_Q9LHZ7	1170	48.88	667	240	14	11	1792	395	1033	0.0	605
ABCDEF_48199	UniRef50_Q6YWK8	0.0	Normal: 1506	467	2429	3094	4233	439	BlastAnno: 1997	UniRef50_Q6YWK8	570	63.10	439	146	2	291	1559	57	495	0.0	610
ABCDEF_40983	UniRef50_Q96301	0.0	Normal: 406	261	343	392	415	433	BlastAnno: 2265	UniRef50_Q96301	914	79.17	504	99	1	114	1625	394	891	0.0	854
ABCDEF_17140	UniRef50_Q9LHZ7	0.0	Normal: 45	1049	49	216	386	208	BlastAnno: 2293	UniRef50_Q9LHZ7	1170	52.34	770	281	13	2138	57	397	1156	0.0	739
ABCDEF_18352	UniRef50_Q9FWA4	0.0	Normal: 2427	2688	2969	5519	6547	4855	BlastAnno: 2224	UniRef50_Q9FWA4	561	69.31	505	148	3	1887	382	61	561	0.0	683
ABCDEF_27767	UniRef50_Q9LHZ7	0.0	Normal: 2576	5141	2841	10249	14583	5031	BlastAnno: 3981	UniRef50_Q9LHZ7	1170	49.66	888	337	16	2927	504	299	1156	0.0	818
ABCDEF_33661	UniRef50_Q9LHZ7	0.0	Normal: 414	340	507	879	790	783	BlastAnno: 2185	UniRef50_Q9LHZ7	1170	48.66	748	277	14	2184	169	440	1156	0.0	662
ABCDEF_14685	UniRef50_A7XDQ9	0.0	Normal: 233	308	134	573	292	334	BlastAnno: 1947	UniRef50_A7XDQ9	684	56.84	468	186	6	569	1945	1	461	0.0	545
ABCDEF_20660	UniRef50_Q8VZK9	0.0	Normal: 22	48	13	15	52	144	BlastAnno: 1473	UniRef50_Q8VZK9	729	55.16	475	199	6	55	1473	11	473	0.0	537
ABCDEF_21315	UniRef50_Q9LHZ7	0.0	Normal: 3412	252	1450	0	79	1228	BlastAnno: 3765	UniRef50_Q9LHZ7	1170	63.78	1096	340	15	308	3508	70	1137	0.0	1333
ABCDEF_533	UniRef50_Q9C8W3	0.0	Normal: 230	204	269	505	411	301	BlastAnno: 1688	UniRef50_Q9C8W3	401	70.33	364	94	2	393	1475	47	399	0.0	555
ABCDEF_34099	UniRef50_Q9LE59	0.0	Normal: 478	491	735	1356	1350	548	BlastAnno: 2158	UniRef50_Q9LE59	673	60.92	458	178	1	1610	240	215	672	0.0	630
ABCDEF_47984	UniRef50_Q7PC69	0.0	Normal: 663	457	734	1362	2147	696	BlastAnno: 2838	UniRef50_Q7PC69	745	59.27	712	226	9	373	2316	34	745	0.0	702
ABCDEF_51451	UniRef50_Q9LE59	0.0	Normal: 1883	1782	2202	4337	4350	2667	BlastAnno: 2874	UniRef50_Q9LE59	673	74.16	685	165	6	2559	505	1	673	0.0	982
ABCDEF_13370	UniRef50_Q8VZK9	0.0	Normal: 290	87	317	377	105	258	BlastAnno: 2033	UniRef50_Q8VZK9	729	51.65	635	278	11	2031	154	112	726	0.0	612
ABCDEF_48239	UniRef50_Q9LHZ7	0.0	Normal: 511	163	422	1154	1316	510	BlastAnno: 2714	UniRef50_Q9LHZ7	1170	76.20	769	156	9	478	2712	41	806	0.0	1201
ABCDEF_13338	UniRef50_Q7PC69	0.0	Normal: 335	468	417	2639	4443	616	BlastAnno: 2525	UniRef50_Q7PC69	745	62.45	727	209	12	298	2292	21	745	0.0	781
ABCDEF_24193	UniRef50_Q8L7F9	0.0	Normal: 113	72	132	203	180	119	BlastAnno: 1679	UniRef50_Q8L7F9	643	60.54	479	176	4	212	1609	1	479	0.0	592
ABCDEF_48514	UniRef50_Q9ZV71	0.0	Normal: 540	580	601	771	1033	926	BlastAnno: 1920	UniRef50_Q9ZV71	409	78.14	398	85	2	1618	431	12	409	0.0	637
2.4.1.185(flavanone 7-O-beta-glucosyltransferase;)																					
No Correspond Unigene																					
2.4.1.236(flavanone 7-O-glucoside 2''-O-beta-L-rhamnosyltransferase;)																					
ABCDEF_24628	UniRef50_Q8GVE3	5e-98	Normal: 362	43	68	125	130	118	BlastAnno: 1488	UniRef50_Q8GVE3	452	41.06	436	235	10	1377	97	11	433	5e-98	311
5.5.1.6(chalcone isomerase;)																					
ABCDEF_34946	UniRef50_P41088	1e-86	Normal: 1328	102	3395	10046	8742	153	BlastAnno: 1187	UniRef50_P41088	246	60.65	216	85	0	925	278	14	229	1e-86	271
>00942 Anthocyanin biosynthesis																					
2.1.1.-(Transferases;)																					
ABCDEF_20806	UniRef50_Q94II3	0.0	Normal: 700	332	876	1458	1997	929	BlastAnno: 2275	UniRef50_Q94II3	600	73.57	594	153	4	2119	341	1	591	0.0	907
ABCDEF_45802	UniRef50_Q9ZPH9	0.0	Normal: 3543	3348	4296	9797	10830	5017	BlastAnno: 2642	UniRef50_Q9ZPH9	633	53.90	603	261	7	2216	456	23	624	0.0	676
ABCDEF_20070	UniRef50_Q944R7	0.0	Normal: 301	141	265	207	185	187	BlastAnno: 2675	UniRef50_Q944R7	724	58.28	719	275	9	127	2265	13	712	0.0	840
ABCDEF_34956	UniRef50_Q94II3	0.0	Normal: 1120	7269	2475	16033	22219	21461	BlastAnno: 2676	UniRef50_Q94II3	600	73.77	610	146	4	404	2230	1	597	0.0	957
ABCDEF_17121	UniRef50_Q9ZPH9	0.0	Normal: 124	77	109	284	199	132	BlastAnno: 2066	UniRef50_Q9ZPH9	633	53.24	601	259	8	161	1903	25	623	0.0	644
ABCDEF_6524	UniRef50_Q9ZPH9	0.0	Normal: 471	296	490	753	870	730	BlastAnno: 2602	UniRef50_Q9ZPH9	633	50.89	619	290	5	2221	404	6	623	0.0	659
ABCDEF_56017	UniRef50_Q9ZPH9	0.0	Normal: 302	167	286	596	748	529	BlastAnno: 2384	UniRef50_Q9ZPH9	633	51.37	619	287	5	213	2030	6	623	0.0	663
ABCDEF_44169	UniRef50_Q9SIZ3	0.0	Normal: 179	158	273	705	685	229	BlastAnno: 2300	UniRef50_Q9SIZ3	589	62.01	308	108	4	1219	296	285	583	0.0	394
ABCDEF_6521	UniRef50_Q9ZPH9	0.0	Normal: 339	198	314	641	767	582	BlastAnno: 2511	UniRef50_Q9ZPH9	633	51.37	619	287	5	2172	355	6	623	0.0	663
ABCDEF_44172	UniRef50_Q9SIZ3	0.0	Normal: 204	184	287	767	741	242	BlastAnno: 2317	UniRef50_Q9SIZ3	589	68.15	449	134	4	1637	291	144	583	0.0	641
ABCDEF_199	UniRef50_Q9SU94	0.0	Normal: 1076	275	1009	558	1247	306	BlastAnno: 1769	UniRef50_Q9SU94	390	78.45	348	74	1	284	1324	43	390	0.0	584
ABCDEF_52252	UniRef50_Q84W92	0.0	Normal: 1658	720	1653	1980	2289	1601	BlastAnno: 2170	UniRef50_Q84W92	535	72.49	469	120	3	409	1791	34	501	0.0	703
ABCDEF_51404	UniRef50_A3BMN9	0.0	Normal: 864	188	716	337	631	326	BlastAnno: 2192	UniRef50_A3BMN9	620	50.50	604	243	9	2008	251	55	620	0.0	555
ABCDEF_21386	UniRef50_Q94AI4	0.0	Normal: 2326	1250	2220	4294	2920	1983	BlastAnno: 3701	UniRef50_Q94AI4	775	47.27	842	332	16	567	2978	8	775	0.0	577
ABCDEF_35101	UniRef50_Q9MAT5	0.0	Normal: 1440	420	1268	576	1136	850	BlastAnno: 1634	UniRef50_Q9MAT5	383	69.03	352	109	0	1449	394	32	383	0.0	550
ABCDEF_960	UniRef50_Q9LN50	0.0	Normal: 3504	13198	3609	11156	19651	12155	BlastAnno: 2626	UniRef50_Q9LN50	724	49.14	525	246	5	883	2451	219	724	0.0	570
ABCDEF_48082	UniRef50_Q9LN50	0.0	Normal: 410	445	467	870	993	454	BlastAnno: 2340	UniRef50_Q9LN50	724	70.25	521	155	0	710	2272	204	724	0.0	821
ABCDEF_10473	UniRef50_Q08A71	0.0	Normal: 374	187	332	425	456	314	BlastAnno: 1764	UniRef50_Q08A71	435	79.23	366	75	1	344	1438	70	435	0.0	575
ABCDEF_7295	UniRef50_Q9ZPH9	0.0	Normal: 25	54	30	81	26	329	BlastAnno: 1191	UniRef50_Q9ZPH9	633	69.23	403	110	4	1191	22	225	626	0.0	599
ABCDEF_4160	UniRef50_Q94II3	0.0	Normal: 189	0	210	71	17	72	BlastAnno: 1424	UniRef50_Q94II3	600	77.28	449	101	1	1424	81	143	591	0.0	758
ABCDEF_44857	UniRef50_Q9ZPH9	0.0	Normal: 101	224	103	299	588	350	BlastAnno: 1582	UniRef50_Q9ZPH9	633	72.06	519	143	1	1571	21	114	632	0.0	822
ABCDEF_71541	UniRef50_Q9SIZ3	0.0	Normal: 187	167	296	765	724	237	BlastAnno: 2268	UniRef50_Q9SIZ3	589	62.01	308	108	4	1210	287	285	583	0.0	394
ABCDEF_44171	UniRef50_Q9SIZ3	0.0	Normal: 214	196	312	827	802	251	BlastAnno: 2339	UniRef50_Q9SIZ3	589	64.78	548	182	5	1978	335	47	583	0.0	717
ABCDEF_20805	UniRef50_Q94II3	0.0	Normal: 674	344	885	1417	2194	963	BlastAnno: 2400	UniRef50_Q94II3	600	78.21	436	94	1	879	2183	156	591	0.0	734
ABCDEF_18210	UniRef50_Q9SIZ3	0.0	Normal: 5584	3714	7379	17006	21892	7546	BlastAnno: 3199	UniRef50_Q9SIZ3	589	50.00	576	269	7	2145	424	27	585	0.0	574
ABCDEF_6526	UniRef50_Q9ZPH9	0.0	Normal: 627	185	837	2717	2815	1530	BlastAnno: 2611	UniRef50_Q9ZPH9	633	54.76	599	259	5	462	2222	25	623	0.0	689
ABCDEF_6525	UniRef50_Q9ZPH9	0.0	Normal: 602	165	760	2369	2605	1400	BlastAnno: 2545	UniRef50_Q9ZPH9	633	54.92	599	258	5	431	2191	25	623	0.0	689
ABCDEF_6523	UniRef50_Q9ZPH9	0.0	Normal: 314	175	304	641	784	552	BlastAnno: 2312	UniRef50_Q9ZPH9	633	52.42	599	272	4	201	1958	25	623	0.0	663
ABCDEF_67393	UniRef50_Q9ZPH9	0.0	Normal: 434	245	444	608	768	655	BlastAnno: 2346	UniRef50_Q9ZPH9	633	51.92	599	275	4	232	1989	25	623	0.0	659
ABCDEF_3579	UniRef50_O14744	0.0	Normal: 1361	326	984	568	791	440	BlastAnno: 2369	UniRef50_O14744	637	47.00	634	294	10	267	2120	30	637	0.0	553
2.3.1.-(Transferases;)																					
ABCDEF_520	UniRef50_Q9FF86	0.0	Normal: 1672	85	2273	5056	3383	1211	BlastAnno: 1811	UniRef50_Q9FF86	484	64.06	448	149	6	1664	354	3	449	0.0	573
ABCDEF_68664	UniRef50_Q9ZVN2	0.0	Normal: 79	35	85	107	193	63	BlastAnno: 1488	UniRef50_Q9ZVN2	704	63.99	411	142	3	46	1275	298	703	0.0	540
ABCDEF_52245	UniRef50_Q9FG87	0.0	Normal: 1878	635	1833	3814	1432	1376	BlastAnno: 1758	UniRef50_Q9FG87	529	55.35	486	204	4	4	1428	34	517	0.0	530
ABCDEF_16730	UniRef50_Q3EC11	0.0	Normal: 281	204	237	365	377	458	BlastAnno: 2092	UniRef50_Q3EC11	536	59.33	536	204	8	196	1785	3	530	0.0	661
ABCDEF_4655	UniRef50_A5BMG1	0.0	Normal: 2426	1856	3606	6958	4608	2755	BlastAnno: 2261	UniRef50_A5BMG1	481	68.37	528	112	10	263	1843	3	476	0.0	667

ABCDEF_13860	UniRef50_Q9C533	0.0	Normal: 588	220	570	1451	1338	310	BlastAnno: 2648	UniRef50_Q9C533	596	63.37	606	177	12	2226	511	1	595	0.0	558
ABCDEF_28294	UniRef50_Q9SYZ0	0.0	Normal: 1038	4070	1427	6745	877	2797	BlastAnno: 1785	UniRef50_Q9SYZ0	493	52.37	485	223	3	1598	162	5	487	0.0	533
ABCDEF_47718	UniRef50_Q9C533	0.0	Normal: 1599	236	1657	2093	1231	588	BlastAnno: 2680	UniRef50_Q9C533	596	65.02	626	165	14	2280	472	1	595	0.0	634
ABCDEF_45080	UniRef50_Q9SYZ0	0.0	Normal: 1005	592	1079	2360	3762	2217	BlastAnno: 2130	UniRef50_Q9SYZ0	493	57.85	484	197	3	302	1735	5	487	0.0	533
ABCDEF_25377	UniRef50_Q9C6L5	0.0	Normal: 37	2090	138	2573	1026	1298	BlastAnno: 1334	UniRef50_Q9C6L5	492	79.74	385	76	1	1155	1	6	388	0.0	654
ABCDEF_33470	UniRef50_Q9LW26	0.0	Normal: 172	98	156	260	391	149	BlastAnno: 2993	UniRef50_Q9LW26	701	48.96	627	301	5	655	2508	85	701	0.0	581
ABCDEF_40654	UniRef50_Q9H0A0	0.0	Normal: 638	213	530	318	318	286	BlastAnno: 2216	UniRef50_Q9H0A0	1025	56.87	721	279	6	2127	13	91	795	0.0	756
ABCDEF_33460	UniRef50_Q9ZVN2	0.0	Normal: 144	71	119	216	306	97	BlastAnno: 2691	UniRef50_Q9ZVN2	704	61.50	587	191	4	379	2133	83	636	0.0	715
ABCDEF_14442	UniRef50_Q9SYZ0	0.0	Normal: 341	684	439	1040	1601	552	BlastAnno: 1779	UniRef50_Q9SYZ0	493	63.12	480	171	2	1570	149	5	484	0.0	628
ABCDEF_52626	UniRef50_B6SYP9	0.0	Normal: 815	346	1131	3015	1537	108	BlastAnno: 1861	UniRef50_B6SYP9	494	55.02	438	189	3	1538	249	51	488	0.0	536
ABCDEF_644	UniRef50_Q9SB58	0.0	Normal: 704	444	913	969	1431	848	BlastAnno: 1777	UniRef50_Q9SB58	407	64.15	424	129	8	1572	316	1	406	0.0	554
ABCDEF_27087	UniRef50_Q52T38	0.0	Normal: 324	221	233	419	343	412	BlastAnno: 1631	UniRef50_Q52T38	620	75.95	420	91	1	1409	150	1	410	0.0	681
ABCDEF_34187	UniRef50_Q8L5Y5	0.0	Normal: 497	415	588	523	291	433	BlastAnno: 2677	UniRef50_Q8L5Y5	718	57.30	733	251	15	202	2274	1	713	0.0	683
ABCDEF_47966	UniRef50_Q9SYZ0	0.0	Normal: 577	934	326	580	682	3440	BlastAnno: 2177	UniRef50_Q9SYZ0	493	56.40	484	204	3	1780	347	5	487	0.0	535
ABCDEF_51189	UniRef50_Q52T38	0.0	Normal: 135	152	128	181	219	327	BlastAnno: 1766	UniRef50_Q52T38	620	68.09	470	137	3	80	1483	130	588	0.0	641
ABCDEF_41288	UniRef50_Q9SB58	0.0	Normal: 307	183	332	637	649	322	BlastAnno: 1403	UniRef50_Q9SB58	407	67.56	410	110	6	1401	184	15	405	0.0	523
ABCDEF_33461	UniRef50_Q9ZVN2	0.0	Normal: 142	84	140	206	283	119	BlastAnno: 2904	UniRef50_Q9ZVN2	704	60.98	574	201	6	2271	559	83	636	0.0	686
ABCDEF_14334	UniRef50_Q9SYZ0	0.0	Normal: 2263	129	1724	3469	866	283	BlastAnno: 2039	UniRef50_Q9SYZ0	493	63.22	484	171	2	369	1802	5	487	0.0	627
ABCDEF_24844	UniRef50_Q9FF86	0.0	Normal: 1857	3686	2657	21940	11467	4090	BlastAnno: 1640	UniRef50_Q9FF86	484	66.89	447	135	6	1492	185	2	446	0.0	575
ABCDEF_51190	UniRef50_Q52T38	0.0	Normal: 145	169	142	210	232	335	BlastAnno: 1891	UniRef50_Q52T38	620	70.04	514	141	3	73	1608	86	588	0.0	726
ABCDEF_14085	UniRef50_Q8L5Y5	0.0	Normal: 353	41	384	527	331	280	BlastAnno: 2362	UniRef50_Q8L5Y5	718	62.07	704	220	12	2360	348	24	713	0.0	676
ABCDEF_33458	UniRef50_Q9LW26	0.0	Normal: 158	78	130	240	342	106	BlastAnno: 2822	UniRef50_Q9LW26	701	46.72	655	302	5	400	2337	85	701	0.0	568
ABCDEF_25193	UniRef50_Q9C6L5	0.0	Normal: 3256	368	3548	6230	5109	1288	BlastAnno: 1786	UniRef50_Q9C6L5	492	65.83	480	156	4	1668	241	17	492	0.0	668
ABCDEF_33457	UniRef50_Q9ZVN2	0.0	Normal: 168	83	137	250	375	112	BlastAnno: 2823	UniRef50_Q9ZVN2	704	65.12	496	168	3	379	1860	83	575	0.0	648
ABCDEF_7634	UniRef50_Q9C6L5	0.0	Normal: 2543	9303	2711	11578	5533	10032	BlastAnno: 2064	UniRef50_Q9C6L5	492	52.76	489	225	3	1734	268	1	483	0.0	535
ABCDEF_299	UniRef50_Q71N54	0.0	Normal: 569	1147	511	900	649	1879	BlastAnno: 2017	UniRef50_Q71N54	535	61.73	533	198	5	338	1924	4	534	0.0	677
ABCDEF_27086	UniRef50_Q52T38	0.0	Normal: 784	470	617	843	893	976	BlastAnno: 2398	UniRef50_Q52T38	620	70.80	637	165	4	225	2129	1	618	0.0	887
ABCDEF_51188	UniRef50_Q52T38	0.0	Normal: 273	327	255	415	349	441	BlastAnno: 2324	UniRef50_Q52T38	620	71.30	568	150	3	2041	344	32	588	0.0	815
ABCDEF_44812	UniRef50_Q6DR03	0.0	Normal: 982	902	1005	1071	959	2999	BlastAnno: 2450	UniRef50_Q6DR03	565	54.97	644	198	17	2142	220	1	555	0.0	540

2.3.1.153(anthocyanin 5-aromatic acyltransferase)

ABCDEF_7539	UniRef50_Q9ZWR8	2e-117	Normal: 150	339	165	1357	751	80	BlastAnno: 1725	UniRef50_Q9ZWR8	469	40.38	473	265	7	1649	255	5	468	2e-117	365

2.3.1.171(anthocyanin 6''-O-malonyltransferase)

No Correspond Unigene

2.3.1.172(anthocyanin 5-O-glucoside 6'''-O-malonyltransferase)

ABCDEF_7539	UniRef50_Q8W1W9	9e-105	Normal: 150	339	165	1357	751	80	BlastAnno: 1725	UniRef50_Q8W1W9	462	39.07	453	256	8	1613	258	14	447	9e-105	332

2.4.1.-(Transferases;)

ABCDEF_18004	UniRef50_Q6YWK8	0.0	Normal: 1562	1736	2009	3452	4438	2400	BlastAnno: 2298	UniRef50_Q6YWK8	570	65.27	429	136	1	1941	694	57	485	0.0	608
ABCDEF_52379	UniRef50_Q9LHZ7	0.0	Normal: 4015	4921	4546	10520	13430	4731	BlastAnno: 3479	UniRef50_Q9LHZ7	1170	48.04	893	342	16	825	3242	299	1156	0.0	787
ABCDEF_48593	UniRef50_Q9SK82	0.0	Normal: 12	420	19	343	763	253	BlastAnno: 1834	UniRef50_Q9SK82	489	60.08	481	186	4	1714	278	10	486	0.0	633
ABCDEF_13375	UniRef50_Q8VZK9	0.0	Normal: 108	123	208	526	34	294	BlastAnno: 1738	UniRef50_Q8VZK9	729	53.62	552	234	8	94	1737	18	551	0.0	582
ABCDEF_40982	UniRef50_Q96301	0.0	Normal: 1037	706	819	1249	1084	920	BlastAnno: 3418	UniRef50_Q96301	914	79.60	902	173	3	243	2948	1	891	0.0	1506
ABCDEF_47674	UniRef50_Q9LHZ7	0.0	Normal: 274	92	202	599	505	280	BlastAnno: 2526	UniRef50_Q9LHZ7	1170	75.93	777	151	8	2260	2	15	779	0.0	1177
ABCDEF_34097	UniRef50_Q9LE59	0.0	Normal: 626	613	923	1797	1829	692	BlastAnno: 2521	UniRef50_Q9LE59	673	60.92	458	178	1	1687	317	215	672	0.0	630
ABCDEF_34098	UniRef50_Q9LE59	0.0	Normal: 457	464	697	1283	1312	539	BlastAnno: 2199	UniRef50_Q9LE59	673	60.92	458	178	1	1687	317	215	672	0.0	628
ABCDEF_10848	UniRef50_Q9M156	0.0	Normal: 157	2988	163	408	772	1566	BlastAnno: 1810	UniRef50_Q9M156	480	60.09	456	176	4	1700	336	4	454	0.0	553
ABCDEF_41182	UniRef50_Q9LHZ7	0.0	Normal: 150	2169	221	814	1407	495	BlastAnno: 3604	UniRef50_Q9LHZ7	1170	51.03	870	333	15	2822	456	299	1156	0.0	826
ABCDEF_40981	UniRef50_Q96301	0.0	Normal: 156	97	110	160	179	173	BlastAnno: 1225	UniRef50_Q96301	914	86.92	344	45	0	1112	81	394	737	0.0	652
ABCDEF_9506	UniRef50_Q9LHZ7	0.0	Normal: 1302	2128	1494	3356	4644	2206	BlastAnno: 3751	UniRef50_Q9LHZ7	1170	49.38	891	343	17	927	3359	299	1161	0.0	812
ABCDEF_9500	UniRef50_Q9LHZ7	0.0	Normal: 183	188	186	576	498	260	BlastAnno: 1717	UniRef50_Q9LHZ7	1170	47.98	644	234	14	3	1715	418	1033	0.0	571
ABCDEF_100	UniRef50_Q9M8Y0	0.0	Normal: 228	309	224	416	461	141	BlastAnno: 2109	UniRef50_Q9M8Y0	977	77.49	533	120	0	1610	12	50	582	0.0	884
ABCDEF_33659	UniRef50_Q9LHZ7	0.0	Normal: 1282	1948	2248	5826	10642	2083	BlastAnno: 4289	UniRef50_Q9LHZ7	1170	48.70	887	345	15	2955	538	299	1156	0.0	788
ABCDEF_37667	UniRef50_Q9LHZ7	0.0	Normal: 248	3617	251	1237	2078	970	BlastAnno: 3161	UniRef50_Q9LHZ7	1170	49.72	885	335	14	2671	260	301	1156	0.0	810
ABCDEF_47673	UniRef50_Q9LHZ7	0.0	Normal: 876	296	828	1628	1721	1172	BlastAnno: 3878	UniRef50_Q9LHZ7	1170	80.88	1161	186	8	3642	232	15	1163	0.0	1865
ABCDEF_99	UniRef50_Q9M8Y0	0.0	Normal: 272	330	222	398	423	145	BlastAnno: 2094	UniRef50_Q9M8Y0	977	77.36	561	127	0	1683	1	50	610	0.0	929
ABCDEF_20658	UniRef50_Q8VZK9	0.0	Normal: 373	33	193	64	503	141	BlastAnno: 2316	UniRef50_Q8VZK9	729	53.35	508	221	7	47	1564	11	504	0.0	559
ABCDEF_10183	UniRef50_A7XDQ9	0.0	Normal: 422	455	346	577	378	610	BlastAnno: 2362	UniRef50_A7XDQ9	684	50.94	693	307	12	281	2287	1	684	0.0	670
ABCDEF_30745	UniRef50_A7XDQ9	0.0	Normal: 1209	351	1031	1645	1226	635	BlastAnno: 2646	UniRef50_A7XDQ9	684	57.58	554	227	6	546	2189	133	684	0.0	664
ABCDEF_13336	UniRef50_Q7PC69	0.0	Normal: 148	563	194	957	1575	816	BlastAnno: 2584	UniRef50_Q7PC69	745	71.56	559	136	6	744	2351	187	745	0.0	730
ABCDEF_21426	UniRef50_Q8RU27	0.0	Normal: 2658	1979	3417	6157	9464	2599	BlastAnno: 1530	UniRef50_Q8RU27	366	89.44	360	34	2	1358	282	1	357	0.0	667
ABCDEF_71242	UniRef50_Q9ZV71	0.0	Normal: 134	122	179	480	754	119	BlastAnno: 1835	UniRef50_Q9ZV71	409	73.35	409	103	4	231	1439	1	409	0.0	587
ABCDEF_52020	UniRef50_Q6YWK8	0.0	Normal: 1635	1534	1975	4284	10353	2289	BlastAnno: 2394	UniRef50_Q6YWK8	570	58.37	514	185	2	403	1857	57	570	0.0	607
ABCDEF_51269	UniRef50_Q6YWK8	0.0	Normal: 147	85	103	21	33	438	BlastAnno: 1864	UniRef50_Q6YWK8	570	54.28	514	204	2	1646	198	57	570	0.0	586
ABCDEF_30753	UniRef50_Q9ZV71	0.0	Normal: 290	497	417	629	1013	634	BlastAnno: 1502	UniRef50_Q9ZV71	409	72.32	401	107	2	311	1501	1	401	0.0	590
ABCDEF_4233	UniRef50_Q9ZV71	0.0	Normal: 369	412	550	936	860	750	BlastAnno: 2004	UniRef50_Q9ZV71	409	72.13	409	110	2	370	1584	1	409	0.0	613
ABCDEF_17525	UniRef50_Q8RU27	0.0	Normal: 1500	1289	2149	3711	4446	1069	BlastAnno: 1507	UniRef50_Q8RU27	366	91.35	347	30	0	1371	331	3	349	0.0	671
ABCDEF_18005	UniRef50_Q6YWK8	0.0	Normal: 1842	2042	2515	4214	5208	2842	BlastAnno: 2305	UniRef50_Q6YWK8	570	59.14	514	180	2	1948	497	57	570	0.0	642
ABCDEF_41140	UniRef50_Q6YWK8	0.0	Normal: 86	1314	93	753	1787	2076	BlastAnno: 2346	UniRef50_Q6YWK8	570	60.00	510	174	2	431	1870	57	566	0.0	669
ABCDEF_18002	UniRef50_Q9LHZ7	0.0	Normal: 376	127	367	601	1190	520	BlastAnno: 1605	UniRef50_Q9LHZ7	1170	89.75	400	41	0	1603	404	771	1170	0.0	732
ABCDEF_13339	UniRef50_Q7PC69	0.0	Normal: 127	814	183	541	1135	1144	BlastAnno: 2610	UniRef50_Q7PC69	745	70.48	559	141	6	725	2329	187	745	0.0	729
ABCDEF_41141	UniRef50_Q6YWK8	0.0	Normal: 67	1107	84	708	1690	1854	BlastAnno: 2461	UniRef50_Q6YWK8	570	60.00	510	174	2	1916	477	57	566	0.0	668
ABCDEF_44583	UniRef50_Q9LE59	0.0	Normal: 681	561	559	947	1745	789	BlastAnno: 2470	UniRef50_Q9LE59	673	59.21	456	185	1	1749	385	214	669	0.0	621
ABCDEF_3869	UniRef50_Q9LE59	0.0	Normal: 333	393	415	809	907	754	BlastAnno: 2136	UniRef50_Q9LE59	673	59.05	613	227	4	1827	1	81	673	0.0	699

ABCDEF_28947	UniRef50_Q9SK82	0.0	Normal: 0	851	3	0	0	116	BlastAnno: 1567	UniRef50_Q9SK82	489	62.40	484	176	4	1524	85	1	482	0.0	652
ABCDEF_31119	UniRef50_Q9M8Y0	0.0	Normal: 2212	1279	1917	2933	3341	1803	BlastAnno: 3695	UniRef50_Q9M8Y0	977	81.08	925	174	1	392	3163	50	974	0.0	1583
ABCDEF_37311	UniRef50_Q9LE59	0.0	Normal: 175	133	191	549	421	366	BlastAnno: 1612	UniRef50_Q9LE59	673	62.96	486	153	4	1406	3	4	480	0.0	542
ABCDEF_24190	UniRef50_Q8L7F9	0.0	Normal: 397	265	492	583	553	371	BlastAnno: 2564	UniRef50_Q8L7F9	643	63.91	640	218	4	2353	473	1	640	0.0	850
ABCDEF_30548	UniRef50_Q9FH36	0.0	Normal: 17	916	27	151	420	126	BlastAnno: 1963	UniRef50_Q9FH36	535	72.47	534	138	3	102	1676	1	534	0.0	767
ABCDEF_27416	UniRef50_Q7PC69	0.0	Normal: 575	837	875	1344	2015	936	BlastAnno: 2454	UniRef50_Q7PC69	745	66.11	416	115	4	2337	1168	126	541	0.0	518
ABCDEF_48373	UniRef50_A7XDQ9	0.0	Normal: 1364	773	1336	1451	1791	1202	BlastAnno: 3042	UniRef50_A7XDQ9	684	63.68	691	234	6	2405	360	1	683	0.0	915
ABCDEF_3868	UniRef50_Q9LE59	0.0	Normal: 224	274	301	555	686	524	BlastAnno: 1670	UniRef50_Q9LE59	673	65.31	516	176	1	1556	9	161	673	0.0	696
ABCDEF_31288	UniRef50_Q940V3	0.0	Normal: 247	309	183	101	124	655	BlastAnno: 1658	UniRef50_Q940V3	470	56.01	466	197	7	1614	232	8	470	0.0	537
ABCDEF_52574	UniRef50_Q09332	0.0	Normal: 885	474	1068	1835	1853	816	BlastAnno: 3720	UniRef50_Q09332	1548	47.89	687	310	14	2551	533	859	1511	0.0	598
ABCDEF_79752	UniRef50_Q9ZV71	0.0	Normal: 158	183	245	400	379	352	BlastAnno: 1266	UniRef50_Q9ZV71	409	71.39	360	99	2	31	1098	1	360	0.0	526
ABCDEF_9509	UniRef50_Q9LHZ7	0.0	Normal: 1296	2171	1498	3410	4683	2232	BlastAnno: 3897	UniRef50_Q9LHZ7	1170	49.38	891	343	17	1073	3505	299	1161	0.0	812
ABCDEF_48200	UniRef50_Q6YWK8	0.0	Normal: 1768	576	3156	4431	6634	491	BlastAnno: 2274	UniRef50_Q6YWK8	570	57.98	514	185	4	291	1739	57	570	0.0	642
ABCDEF_24155	UniRef50_Q9ZVI7	0.0	Normal: 503	247	607	1676	1833	578	BlastAnno: 2708	UniRef50_Q9ZVI7	619	52.33	621	264	11	306	2123	15	618	0.0	601
ABCDEF_21246	UniRef50_Q9SKT6	0.0	Normal: 92	107	188	380	834	84	BlastAnno: 1791	UniRef50_Q9SKT6	536	56.61	484	204	5	123	1562	52	533	0.0	568
ABCDEF_32317	UniRef50_Q8RU27	0.0	Normal: 12	9	3	118	800	62	BlastAnno: 1076	UniRef50_Q8RU27	366	87.02	285	37	0	1075	221	64	348	0.0	535
ABCDEF_9502	UniRef50_Q9LHZ7	0.0	Normal: 610	486	568	1331	1403	591	BlastAnno: 1961	UniRef50_Q9LHZ7	1170	48.64	625	249	13	3	1742	564	1161	0.0	570
ABCDEF_23298	UniRef50_Q9FH36	0.0	Normal: 128	527	130	223	314	864	BlastAnno: 2832	UniRef50_Q9FH36	535	83.16	196	33	0	696	1283	340	535	0.0	357
ABCDEF_40595	UniRef50_O80898	0.0	Normal: 229	135	103	25	307	325	BlastAnno: 2413	UniRef50_O80898	757	44.68	761	378	13	95	2299	10	753	0.0	587
ABCDEF_63947	UniRef50_Q9SK82	0.0	Normal: 0	267	0	0	71	0	BlastAnno: 1446	UniRef50_Q9SK82	489	58.33	468	189	4	60	1445	1	468	0.0	592
ABCDEF_67847	UniRef50_Q9M8Y0	0.0	Normal: 794	476	871	1081	1401	868	BlastAnno: 1591	UniRef50_Q9M8Y0	977	84.86	370	56	0	1589	480	605	974	0.0	685
ABCDEF_65666	UniRef50_Q09332	0.0	Normal: 800	441	946	1606	1637	735	BlastAnno: 3518	UniRef50_Q09332	1548	47.54	671	305	13	1162	3087	859	1511	0.0	586
ABCDEF_38641	UniRef50_Q9FWA4	0.0	Normal: 674	967	1162	2603	4404	2475	BlastAnno: 1599	UniRef50_Q9FWA4	561	81.35	386	72	0	1	1158	176	561	0.0	641
ABCDEF_17914	UniRef50_Q9SKT6	0.0	Normal: 784	567	886	1447	1439	1424	BlastAnno: 2049	UniRef50_Q9SKT6	536	72.95	536	140	2	204	1796	1	536	0.0	785
ABCDEF_18286	UniRef50_Q6NQB7	0.0	Normal: 1131	1108	1225	1872	1722	3329	BlastAnno: 1752	UniRef50_Q6NQB7	393	73.80	397	81	6	1527	367	10	393	0.0	607
ABCDEF_119	UniRef50_Q8RU27	0.0	Normal: 1613	1155	2249	3558	7663	1775	BlastAnno: 1532	UniRef50_Q8RU27	366	88.33	360	38	2	1306	230	1	357	0.0	663
ABCDEF_47603	UniRef50_Q9M9Y5	0.0	Normal: 2609	8779	1664	1103	2085	22991	BlastAnno: 2830	UniRef50_Q9M9Y5	589	54.04	594	267	3	2475	694	1	588	0.0	654
ABCDEF_24192	UniRef50_Q8L7F9	0.0	Normal: 391	244	465	553	497	345	BlastAnno: 2477	UniRef50_Q8L7F9	643	58.99	434	165	4	212	1474	1	434	0.0	513
ABCDEF_17482	UniRef50_Q9SKT6	0.0	Normal: 615	379	816	1068	1470	852	BlastAnno: 2083	UniRef50_Q9SKT6	536	71.11	540	147	4	1915	311	1	536	0.0	786
ABCDEF_85909	UniRef50_Q8VZK9	0.0	Normal: 28	45	9	10	44	127	BlastAnno: 1473	UniRef50_Q8VZK9	729	55.37	475	198	6	1419	1	11	473	0.0	536
ABCDEF_9503	UniRef50_Q9LHZ7	0.0	Normal: 487	544	524	1242	1320	833	BlastAnno: 2087	UniRef50_Q9LHZ7	1170	50.35	574	236	8	57	1712	615	1161	0.0	567
ABCDEF_40594	UniRef50_O80898	0.0	Normal: 256	127	102	41	331	326	BlastAnno: 2417	UniRef50_O80898	757	45.76	719	348	12	213	2291	51	753	0.0	585
ABCDEF_17141	UniRef50_Q9LHZ7	0.0	Normal: 74	1734	69	380	701	333	BlastAnno: 3337	UniRef50_Q9LHZ7	1170	50.92	868	335	14	2539	179	299	1156	0.0	816
ABCDEF_33658	UniRef50_Q9LHZ7	0.0	Normal: 384	308	395	805	1003	602	BlastAnno: 2961	UniRef50_Q9LHZ7	1170	48.76	888	343	14	204	2621	299	1156	0.0	792
ABCDEF_20579	UniRef50_Q7PC69	0.0	Normal: 1007	795	1238	2311	2309	1345	BlastAnno: 3113	UniRef50_Q7PC69	745	59.97	767	229	14	2667	535	1	745	0.0	768
ABCDEF_31118	UniRef50_Q9M8Y0	0.0	Normal: 2135	1267	1900	3004	3329	1757	BlastAnno: 3787	UniRef50_Q9M8Y0	977	81.10	709	133	1	1132	3255	266	974	0.0	1217
ABCDEF_9499	UniRef50_Q9LHZ7	0.0	Normal: 205	207	215	632	538	275	BlastAnno: 1794	UniRef50_Q9LHZ7	1170	48.88	667	240	14	11	1792	395	1033	0.0	605
ABCDEF_48199	UniRef50_Q6YWK8	0.0	Normal: 1506	467	2429	3094	4233	439	BlastAnno: 1997	UniRef50_Q6YWK8	570	63.10	439	146	2	291	1559	57	495	0.0	610
ABCDEF_40983	UniRef50_Q96301	0.0	Normal: 406	261	343	392	415	433	BlastAnno: 2265	UniRef50_Q96301	914	79.17	504	99	1	114	1625	394	891	0.0	854
ABCDEF_17140	UniRef50_Q9LHZ7	0.0	Normal: 45	1049	49	216	386	208	BlastAnno: 2293	UniRef50_Q9LHZ7	1170	52.34	770	281	13	2138	57	397	1156	0.0	739
ABCDEF_18352	UniRef50_Q9FWA4	0.0	Normal: 2427	2688	2969	5519	6547	4855	BlastAnno: 2224	UniRef50_Q9FWA4	561	69.31	505	148	3	1887	382	61	561	0.0	683
ABCDEF_27767	UniRef50_Q9LHZ7	0.0	Normal: 2576	5141	2841	10249	14583	5031	BlastAnno: 3981	UniRef50_Q9LHZ7	1170	49.66	888	337	16	2927	504	299	1156	0.0	818
ABCDEF_33661	UniRef50_Q9LHZ7	0.0	Normal: 414	340	507	879	790	783	BlastAnno: 2185	UniRef50_Q9LHZ7	1170	48.66	748	277	14	2184	169	440	1156	0.0	662
ABCDEF_14685	UniRef50_A7XDQ9	0.0	Normal: 233	308	134	573	292	334	BlastAnno: 1947	UniRef50_A7XDQ9	684	56.84	468	186	6	569	1945	1	461	0.0	545
ABCDEF_20660	UniRef50_Q8VZK9	0.0	Normal: 22	48	13	15	52	144	BlastAnno: 1473	UniRef50_Q8VZK9	729	55.16	475	199	6	55	1473	11	473	0.0	537
ABCDEF_21315	UniRef50_Q9LHZ7	0.0	Normal: 3412	252	1450	0	79	1228	BlastAnno: 3765	UniRef50_Q9LHZ7	1170	63.78	1096	340	15	308	3508	70	1137	0.0	1333
ABCDEF_533	UniRef50_Q9C8W3	0.0	Normal: 230	204	269	505	411	301	BlastAnno: 1688	UniRef50_Q9C8W3	401	70.33	364	94	2	393	1475	47	399	0.0	555
ABCDEF_34099	UniRef50_Q9LE59	0.0	Normal: 478	491	735	1356	1350	548	BlastAnno: 2158	UniRef50_Q9LE59	673	60.92	458	178	1	1610	240	215	672	0.0	630
ABCDEF_47984	UniRef50_Q7PC69	0.0	Normal: 663	457	734	1362	2147	696	BlastAnno: 2838	UniRef50_Q7PC69	745	59.27	712	226	9	373	2316	34	745	0.0	702
ABCDEF_51451	UniRef50_Q9LE59	0.0	Normal: 1883	1782	2202	4337	4350	2667	BlastAnno: 2874	UniRef50_Q9LE59	673	74.16	685	165	6	2559	505	1	673	0.0	982
ABCDEF_13370	UniRef50_Q8VZK9	0.0	Normal: 290	87	317	377	105	258	BlastAnno: 2033	UniRef50_Q8VZK9	729	51.65	635	278	11	2031	154	112	726	0.0	612
ABCDEF_48239	UniRef50_Q9LHZ7	0.0	Normal: 511	163	422	1154	1316	510	BlastAnno: 2714	UniRef50_Q9LHZ7	1170	76.20	769	156	9	478	2712	41	806	0.0	1201
ABCDEF_13338	UniRef50_Q7PC69	0.0	Normal: 335	468	417	2639	4443	616	BlastAnno: 2525	UniRef50_Q7PC69	745	62.45	727	209	12	298	2292	21	745	0.0	781
ABCDEF_24193	UniRef50_Q8L7F9	0.0	Normal: 113	72	132	203	180	119	BlastAnno: 1679	UniRef50_Q8L7F9	643	60.54	479	176	4	212	1609	1	479	0.0	592
ABCDEF_48514	UniRef50_Q9ZV71	0.0	Normal: 540	580	601	771	1033	926	BlastAnno: 1920	UniRef50_Q9ZV71	409	78.14	398	85	2	1618	431	12	409	0.0	637

2.4.1.115(anthocyanidin 3-O-glucosyltransferase;)

ABCDEF_34091	UniRef50_B9SUM4	0.0	Normal: 919	313	1164	1012	309	546	BlastAnno: 1654	UniRef50_B9SUM4	505	55.49	501	207	6	1565	102	3	500	0.0	575
ABCDEF_78405	UniRef50_B9SUM4	0.0	Normal: 532	232	589	760	225	610	BlastAnno: 1338	UniRef50_B9SUM4	505	58.53	434	168	4	1289	24	5	438	0.0	533
ABCDEF_34092	UniRef50_B9SUM4	0.0	Normal: 676	279	746	821	254	524	BlastAnno: 1738	UniRef50_B9SUM4	505	56.22	498	205	5	1653	199	3	500	0.0	576
ABCDEF_25617	UniRef50_Q43716	0.0	Normal: 141	815	39	25	25	1156	BlastAnno: 1635	UniRef50_Q43716	473	71.28	470	129	3	32	1423	2	471	0.0	674

2.4.1.116(cyanidin 3-O-rutinoside 5-O-glucosyltransferase;)

No Correspond Unigene

2.4.1.238(anthocyanin 3'-O-beta-glucosyltransferase;)

ABCDEF_72483	UniRef50_Q8H0F2	0.0	Normal: 44	299	68	802	339	250	BlastAnno: 1753	UniRef50_Q8H0F2	482	58.93	448	178	4	189	1517	1	447	0.0	531

>00943 Isoflavonoid biosynthesis

1.1.1.246(pterocarpin synthase;)

No Correspond Unigene

1.14.11.22(flavone synthase)

No Correspond Unigene

1.14.13.-(Oxidoreductases;)

ABCDEF_45538	UniRef50_O23024	3e-156	Normal: 110	6	165	774	133	36	BlastAnno: 1258	UniRef50_O23024	437	58.18	385	151	3	3	1157	37	411	3e-156	457

1.14.13.28(3,9-dihydroxypterocarpan 6a-monooxygenase;)

No Correspond Unigene																					
1.14.13.52(isoflavone 3'-hydroxylase;)																					
No Correspond Unigene																					
1.14.13.85(glyceollin synthase)																					
No Correspond Unigene																					
1.14.13.86(2-hydroxyisoflavanone synthase)																					
No Correspond Unigene																					
1.14.13.89(isoflavone 2'-hydroxylase;)																					
ABCDEF_14512	UniRef50_B9SG23	1e-172	Normal: 261	872	212	291	175	789	BlastAnno: 1878	UniRef50_B9SG23	452	56.29	485	157	6	1696	257	18	452	1e-172	508
1.3.1.-(Oxidoreductases;)																					
ABCDEF_45552	UniRef50_P52581	0.0	Normal: 213	2112	262	1058	1208	1322	BlastAnno: 1284	UniRef50_P52581	312	80.45	312	61	0	185	1120	1	312	0.0	530
ABCDEF_47970	UniRef50_Q9T0J6	0.0	Normal: 1040	443	1171	941	1234	650	BlastAnno: 2557	UniRef50_Q9T0J6	700	60.52	699	232	9	2337	271	36	700	0.0	860
1.3.1.45(2'-hydroxyisoflavone reductase;)																					
No Correspond Unigene																					
1.3.1.46(biochanin-A reductase)																					
No Correspond Unigene																					
1.3.1.51(2'-hydroxydaidzein reductase;)																					
No Correspond Unigene																					
2.1.1.150(isoflavone 7-O-methyltransferase)																					
ABCDEF_38684	UniRef50_O22308	1e-100	Normal: 0	0	177	2779	45	0	BlastAnno: 1362	UniRef50_O22308	352	46.48	355	176	5	1255	191	12	352	1e-100	313
2.1.1.46(isoflavone 4'-O-methyltransferase;)																					
No Correspond Unigene																					
2.3.1.115(isoflavone-7-O-beta-glucoside 6''-O-malonyltransferase;)																					
No Correspond Unigene																					
2.4.1.170(isoflavone 7-O-glucosyltransferase;)																					
No Correspond Unigene																					
2.5.1.36(trihydroxypterocarpan dimethylallyltransferase;)																					
No Correspond Unigene																					
4.2.1.105(2-hydroxyisoflavanone dehydratase;)																					
No Correspond Unigene																					
>00944 Flavone and flavonol biosynthesis																					
1.14.13.21(flavonoid 3'-monooxygenase;)																					
ABCDEF_35618	UniRef50_B9SY12	0.0	Normal: 28	2648	17	142	1234	902	BlastAnno: 1746	UniRef50_B9SY12	482	63.04	506	155	3	1649	132	9	482	0.0	637
ABCDEF_21579	UniRef50_B9SY12	0.0	Normal: 1005	643	1606	4372	3226	1621	BlastAnno: 1895	UniRef50_B9SY12	482	65.94	502	140	2	185	1690	12	482	0.0	655
ABCDEF_18295	UniRef50_B9R991	0.0	Normal: 3667	1934	4977	10491	4588	1917	BlastAnno: 2006	UniRef50_B9R991	511	55.28	492	213	4	217	1686	22	508	0.0	564
ABCDEF_45242	UniRef50_Q9SD85	0.0	Normal: 2190	674	4992	10924	6248	146	BlastAnno: 1929	UniRef50_Q9SD85	513	65.01	523	165	7	73	1626	1	510	0.0	661
1.14.13.88(flavonoid 3',5'-hydroxylase)																					
ABCDEF_37738	UniRef50_B9SRN0	3e-174	Normal: 26	2447	19	30	33	993	BlastAnno: 1837	UniRef50_B9SRN0	524	50.64	466	227	1	1634	246	56	521	3e-174	514
2.1.1.149(myricetin O-methyltransferase)																					
No Correspond Unigene																					
2.1.1.75(apigenin 4'-O-methyltransferase;)																					
No Correspond Unigene																					
2.1.1.76(quercetin 3-O-methyltransferase;)																					
ABCDEF_3867	UniRef50_Q9FK25	0.0	Normal: 43	1755	68	456	3254	474	BlastAnno: 1421	UniRef50_Q9FK25	363	75.00	364	88	2	1351	260	1	361	0.0	565
2.1.1.82(3-methylquercetin 7-O-methyltransferase;)																					
No Correspond Unigene																					
2.1.1.83(3,7-dimethylquercetin 4'-O-methyltransferase;)																					
No Correspond Unigene																					
2.3.1.115(isoflavone-7-O-beta-glucoside 6''-O-malonyltransferase;)																					
No Correspond Unigene																					
2.3.1.116(flavonol-3-O-beta-glucoside O-malonyltransferase;)																					
No Correspond Unigene																					
2.4.1.-(Transferases;)																					
ABCDEF_18004	UniRef50_Q6YWK8	0.0	Normal: 1562	1736	2009	3452	4438	2400	BlastAnno: 2298	UniRef50_Q6YWK8	570	65.27	429	136	1	1941	694	57	485	0.0	608
ABCDEF_52379	UniRef50_Q9LHZ7	0.0	Normal: 4015	4921	4546	10520	13430	4731	BlastAnno: 3479	UniRef50_Q9LHZ7	1170	48.04	893	342	16	825	3242	299	1156	0.0	787
ABCDEF_48593	UniRef50_Q9SK82	0.0	Normal: 12	420	19	343	763	253	BlastAnno: 1834	UniRef50_Q9SK82	489	60.08	481	186	4	1714	278	10	486	0.0	633
ABCDEF_13375	UniRef50_Q8VZK9	0.0	Normal: 108	123	208	526	34	294	BlastAnno: 1738	UniRef50_Q8VZK9	729	53.62	552	234	8	94	1737	18	551	0.0	582
ABCDEF_40982	UniRef50_Q96301	0.0	Normal: 1037	706	819	1249	1084	920	BlastAnno: 3418	UniRef50_Q96301	914	79.60	902	173	3	243	2948	1	891	0.0	1506
ABCDEF_47674	UniRef50_Q9LHZ7	0.0	Normal: 274	92	202	599	505	280	BlastAnno: 2526	UniRef50_Q9LHZ7	1170	75.93	777	151	8	2260	2	15	779	0.0	1177
ABCDEF_34097	UniRef50_Q9LE59	0.0	Normal: 626	613	923	1797	1829	692	BlastAnno: 2521	UniRef50_Q9LE59	673	60.92	458	178	1	1687	317	215	672	0.0	630
ABCDEF_34098	UniRef50_Q9LE59	0.0	Normal: 457	464	697	1283	1312	539	BlastAnno: 2199	UniRef50_Q9LE59	673	60.92	458	178	1	1687	317	215	672	0.0	628
ABCDEF_10848	UniRef50_Q9M156	0.0	Normal: 157	2988	163	408	772	1566	BlastAnno: 1810	UniRef50_Q9M156	480	60.09	456	176	4	1700	336	4	454	0.0	553
ABCDEF_41182	UniRef50_Q9LHZ7	0.0	Normal: 150	2169	221	814	1407	495	BlastAnno: 3604	UniRef50_Q9LHZ7	1170	51.03	870	333	15	2822	456	299	1156	0.0	826
ABCDEF_40981	UniRef50_Q96301	0.0	Normal: 156	97	110	160	179	173	BlastAnno: 1225	UniRef50_Q96301	914	86.92	344	45	0	1112	81	394	737	0.0	652
ABCDEF_9506	UniRef50_Q9LHZ7	0.0	Normal: 1302	2128	1494	3356	4644	2206	BlastAnno: 3751	UniRef50_Q9LHZ7	1170	49.38	891	343	17	927	3359	299	1161	0.0	812
ABCDEF_9500	UniRef50_Q9LHZ7	0.0	Normal: 183	188	186	576	498	260	BlastAnno: 1717	UniRef50_Q9LHZ7	1170	47.98	644	234	14	3	1715	418	1033	0.0	571
ABCDEF_100	UniRef50_Q9M8Y0	0.0	Normal: 228	309	224	416	461	141	BlastAnno: 2109	UniRef50_Q9M8Y0	977	77.49	533	120	0	1610	12	50	582	0.0	884
ABCDEF_33659	UniRef50_Q9LHZ7	0.0	Normal: 1282	1948	2248	5826	10642	2083	BlastAnno: 4289	UniRef50_Q9LHZ7	1170	48.70	887	345	15	2955	538	299	1156	0.0	788
ABCDEF_37667	UniRef50_Q9LHZ7	0.0	Normal: 248	3617	251	1237	2078	970	BlastAnno: 3161	UniRef50_Q9LHZ7	1170	49.72	885	335	14	2671	260	301	1156	0.0	810
ABCDEF_47673	UniRef50_Q9LHZ7	0.0	Normal: 876	296	828	1628	1721	1172	BlastAnno: 3878	UniRef50_Q9LHZ7	1170	80.88	1161	186	8	3642	232	15	1163	0.0	1865
ABCDEF_99	UniRef50_Q9M8Y0	0.0	Normal: 272	330	222	398	423	145	BlastAnno: 2094	UniRef50_Q9M8Y0	977	77.36	561	127	0	1683	1	50	610	0.0	929
ABCDEF_20658	UniRef50_Q8VZK9	0.0	Normal: 373	33	193	64	503	141	BlastAnno: 2316	UniRef50_Q8VZK9	729	53.35	508	221	7	47	1564	11	504	0.0	559

ABCDEF_10183	UniRef50_A7XDQ9	0.0	Normal: 422	455	346	577	378	610	BlastAnno: 2362	UniRef50_A7XDQ9	684	50.94	693	307	12	281	2287	1	684	0.0	670
ABCDEF_30745	UniRef50_A7XDQ9	0.0	Normal: 1209	351	1031	1645	1226	635	BlastAnno: 2646	UniRef50_A7XDQ9	684	57.58	554	227	6	546	2189	133	684	0.0	664
ABCDEF_13336	UniRef50_Q7PC69	0.0	Normal: 148	563	194	957	1575	816	BlastAnno: 2584	UniRef50_Q7PC69	745	71.56	559	136	6	744	2351	187	745	0.0	730
ABCDEF_21426	UniRef50_Q8RU27	0.0	Normal: 2658	1979	3417	6157	9464	2599	BlastAnno: 1530	UniRef50_Q8RU27	366	89.44	360	34	2	1358	282	1	357	0.0	667
ABCDEF_71242	UniRef50_Q9ZV71	0.0	Normal: 134	122	179	480	754	119	BlastAnno: 1835	UniRef50_Q9ZV71	409	73.35	409	103	4	231	1439	1	409	0.0	587
ABCDEF_52020	UniRef50_Q6YWK8	0.0	Normal: 1635	1534	1975	4284	10353	2289	BlastAnno: 2394	UniRef50_Q6YWK8	570	58.37	514	185	2	403	1857	57	570	0.0	607
ABCDEF_51269	UniRef50_Q6YWK8	0.0	Normal: 147	85	103	21	33	438	BlastAnno: 1864	UniRef50_Q6YWK8	570	54.28	514	204	2	1646	198	57	570	0.0	586
ABCDEF_30753	UniRef50_Q9ZV71	0.0	Normal: 290	497	417	629	1013	634	BlastAnno: 1502	UniRef50_Q9ZV71	409	72.32	401	107	2	311	1501	1	401	0.0	590
ABCDEF_4233	UniRef50_Q9ZV71	0.0	Normal: 369	412	550	936	860	750	BlastAnno: 2004	UniRef50_Q9ZV71	409	72.13	409	110	2	370	1584	1	409	0.0	613
ABCDEF_17525	UniRef50_Q8RU27	0.0	Normal: 1500	1289	2149	3711	4446	1069	BlastAnno: 1507	UniRef50_Q8RU27	366	91.35	347	30	0	1371	331	3	349	0.0	671
ABCDEF_18005	UniRef50_Q6YWK8	0.0	Normal: 1842	2042	2515	4214	5208	2842	BlastAnno: 2305	UniRef50_Q6YWK8	570	59.14	514	180	2	1948	497	57	570	0.0	642
ABCDEF_41140	UniRef50_Q6YWK8	0.0	Normal: 86	1314	93	753	1787	2076	BlastAnno: 2346	UniRef50_Q6YWK8	570	60.00	510	174	2	431	1870	57	566	0.0	669
ABCDEF_18002	UniRef50_Q9LHZ7	0.0	Normal: 376	127	367	601	1190	520	BlastAnno: 1605	UniRef50_Q9LHZ7	1170	89.75	400	41	0	1603	404	771	1170	0.0	732
ABCDEF_13339	UniRef50_Q7PC69	0.0	Normal: 127	814	183	541	1135	1144	BlastAnno: 2610	UniRef50_Q7PC69	745	70.48	559	141	6	725	2329	187	745	0.0	729
ABCDEF_41141	UniRef50_Q6YWK8	0.0	Normal: 67	1107	84	708	1690	1854	BlastAnno: 2461	UniRef50_Q6YWK8	570	60.00	510	174	2	1916	477	57	566	0.0	668
ABCDEF_44583	UniRef50_Q9LE59	0.0	Normal: 681	561	559	947	1745	789	BlastAnno: 2470	UniRef50_Q9LE59	673	59.21	456	185	1	1749	385	214	669	0.0	621
ABCDEF_3869	UniRef50_Q9LE59	0.0	Normal: 333	393	415	809	907	754	BlastAnno: 2136	UniRef50_Q9LE59	673	59.05	613	227	4	1827	1	81	673	0.0	699
ABCDEF_28947	UniRef50_Q9SK82	0.0	Normal: 0	851	3	0	0	116	BlastAnno: 1567	UniRef50_Q9SK82	489	62.40	484	176	4	1524	85	1	482	0.0	652
ABCDEF_31119	UniRef50_Q9M8Y0	0.0	Normal: 2212	1279	1917	2933	3341	1803	BlastAnno: 3695	UniRef50_Q9M8Y0	977	81.08	925	174	1	392	3163	50	974	0.0	1583
ABCDEF_37311	UniRef50_Q9LE59	0.0	Normal: 175	133	191	549	421	366	BlastAnno: 1612	UniRef50_Q9LE59	673	62.96	486	153	4	1406	3	4	480	0.0	542
ABCDEF_24190	UniRef50_Q8L7F9	0.0	Normal: 397	265	492	583	553	371	BlastAnno: 2564	UniRef50_Q8L7F9	643	63.91	640	218	4	2353	473	1	640	0.0	850
ABCDEF_30548	UniRef50_Q9FH36	0.0	Normal: 17	916	27	151	420	126	BlastAnno: 1963	UniRef50_Q9FH36	535	72.47	534	138	3	102	1676	1	534	0.0	767
ABCDEF_27416	UniRef50_Q7PC69	0.0	Normal: 575	837	875	1344	2015	936	BlastAnno: 2454	UniRef50_Q7PC69	745	66.11	416	115	4	2337	1168	126	541	0.0	518
ABCDEF_48373	UniRef50_A7XDQ9	0.0	Normal: 1364	773	1336	1451	1791	1202	BlastAnno: 3042	UniRef50_A7XDQ9	684	63.68	691	234	6	2405	360	1	683	0.0	915
ABCDEF_3868	UniRef50_Q9LE59	0.0	Normal: 224	274	301	555	686	524	BlastAnno: 1670	UniRef50_Q9LE59	673	65.31	516	176	1	1556	9	161	673	0.0	696
ABCDEF_31288	UniRef50_Q940V3	0.0	Normal: 247	309	183	101	124	655	BlastAnno: 1658	UniRef50_Q940V3	470	56.01	466	197	7	1614	232	8	470	0.0	537
ABCDEF_52574	UniRef50_Q09332	0.0	Normal: 885	474	1068	1835	1853	816	BlastAnno: 3720	UniRef50_Q09332	1548	47.89	687	310	14	2551	533	859	1511	0.0	598
ABCDEF_79752	UniRef50_Q9ZV71	0.0	Normal: 158	183	245	400	379	352	BlastAnno: 1266	UniRef50_Q9ZV71	409	71.39	360	99	2	31	1098	1	360	0.0	526
ABCDEF_9509	UniRef50_Q9LHZ7	0.0	Normal: 1296	2171	1498	3410	4683	2232	BlastAnno: 3897	UniRef50_Q9LHZ7	1170	49.38	891	343	17	1073	3505	299	1161	0.0	812
ABCDEF_48200	UniRef50_Q6YWK8	0.0	Normal: 1768	576	3156	4431	6634	491	BlastAnno: 2274	UniRef50_Q6YWK8	570	57.98	514	185	4	291	1739	57	570	0.0	642
ABCDEF_24155	UniRef50_Q9ZVI7	0.0	Normal: 503	247	607	1676	1833	578	BlastAnno: 2708	UniRef50_Q9ZVI7	619	52.33	621	264	11	306	2123	15	618	0.0	601
ABCDEF_21246	UniRef50_Q9SKT6	0.0	Normal: 92	107	188	380	834	84	BlastAnno: 1791	UniRef50_Q9SKT6	536	56.61	484	204	5	123	1562	52	533	0.0	568
ABCDEF_32317	UniRef50_Q8RU27	0.0	Normal: 12	9	3	118	800	62	BlastAnno: 1076	UniRef50_Q8RU27	366	87.02	285	37	0	1075	221	64	348	0.0	535
ABCDEF_9502	UniRef50_Q9LHZ7	0.0	Normal: 610	486	568	1331	1403	591	BlastAnno: 1961	UniRef50_Q9LHZ7	1170	48.64	625	249	13	3	1742	564	1161	0.0	570
ABCDEF_23298	UniRef50_Q9FH36	0.0	Normal: 128	527	130	223	314	864	BlastAnno: 2832	UniRef50_Q9FH36	535	83.16	196	33	0	696	1283	340	535	0.0	357
ABCDEF_40595	UniRef50_O80898	0.0	Normal: 229	135	103	25	307	325	BlastAnno: 2413	UniRef50_O80898	757	44.68	761	378	13	95	2299	10	753	0.0	587
ABCDEF_63947	UniRef50_Q9SK82	0.0	Normal: 0	267	0	0	71	0	BlastAnno: 1446	UniRef50_Q9SK82	489	58.33	468	189	4	60	1445	1	468	0.0	592
ABCDEF_67847	UniRef50_Q9M8Y0	0.0	Normal: 794	476	871	1081	1401	868	BlastAnno: 1591	UniRef50_Q9M8Y0	977	84.86	370	56	0	1589	480	605	974	0.0	685
ABCDEF_65666	UniRef50_Q09332	0.0	Normal: 800	441	946	1606	1637	735	BlastAnno: 3518	UniRef50_Q09332	1548	47.54	671	305	13	1162	3087	859	1511	0.0	586
ABCDEF_38641	UniRef50_Q9FWA4	0.0	Normal: 674	967	1162	2603	4404	2475	BlastAnno: 1599	UniRef50_Q9FWA4	561	81.35	386	72	0	1	1158	176	561	0.0	641
ABCDEF_17914	UniRef50_Q9SKT6	0.0	Normal: 784	567	886	1447	1439	1424	BlastAnno: 2049	UniRef50_Q9SKT6	536	72.95	536	140	2	204	1796	1	536	0.0	785
ABCDEF_18286	UniRef50_Q6NQB7	0.0	Normal: 1131	1108	1225	1872	1722	3329	BlastAnno: 1752	UniRef50_Q6NQB7	393	73.80	397	81	6	1527	367	10	393	0.0	607
ABCDEF_119	UniRef50_Q8RU27	0.0	Normal: 1613	1155	2249	3558	7663	1775	BlastAnno: 1532	UniRef50_Q8RU27	366	88.33	360	38	2	1306	230	1	357	0.0	663
ABCDEF_47603	UniRef50_Q9M9Y5	0.0	Normal: 2609	8779	1664	1103	2085	22991	BlastAnno: 2830	UniRef50_Q9M9Y5	589	54.04	594	267	3	2475	694	1	588	0.0	654
ABCDEF_24192	UniRef50_Q8L7F9	0.0	Normal: 391	244	465	553	497	345	BlastAnno: 2477	UniRef50_Q8L7F9	643	58.99	434	165	4	212	1474	1	434	0.0	513
ABCDEF_17482	UniRef50_Q9SKT6	0.0	Normal: 615	379	816	1068	1470	852	BlastAnno: 2083	UniRef50_Q9SKT6	536	71.11	540	147	4	1915	311	1	536	0.0	786
ABCDEF_85909	UniRef50_Q8VZK9	0.0	Normal: 28	45	9	10	44	127	BlastAnno: 1473	UniRef50_Q8VZK9	729	55.37	475	198	6	1419	1	11	473	0.0	536
ABCDEF_9503	UniRef50_Q9LHZ7	0.0	Normal: 487	544	524	1242	1320	833	BlastAnno: 2087	UniRef50_Q9LHZ7	1170	50.35	574	236	8	57	1712	615	1161	0.0	567
ABCDEF_40594	UniRef50_O80898	0.0	Normal: 256	127	102	41	331	326	BlastAnno: 2417	UniRef50_O80898	757	45.76	719	348	12	213	2291	51	753	0.0	585
ABCDEF_17141	UniRef50_Q9LHZ7	0.0	Normal: 74	1734	69	380	701	333	BlastAnno: 3337	UniRef50_Q9LHZ7	1170	50.92	868	335	14	2539	179	299	1156	0.0	816
ABCDEF_33658	UniRef50_Q9LHZ7	0.0	Normal: 384	308	395	805	1003	602	BlastAnno: 2961	UniRef50_Q9LHZ7	1170	48.76	888	343	14	204	2621	299	1156	0.0	792
ABCDEF_20579	UniRef50_Q7PC69	0.0	Normal: 1007	795	1238	2311	2309	1345	BlastAnno: 3113	UniRef50_Q7PC69	745	59.97	767	229	14	2667	535	1	745	0.0	768
ABCDEF_31118	UniRef50_Q9M8Y0	0.0	Normal: 2135	1267	1900	3004	3329	1757	BlastAnno: 3787	UniRef50_Q9M8Y0	977	81.10	709	133	1	1132	3255	266	974	0.0	1217
ABCDEF_9499	UniRef50_Q9LHZ7	0.0	Normal: 205	207	215	632	538	275	BlastAnno: 1794	UniRef50_Q9LHZ7	1170	48.88	667	240	14	11	1792	395	1033	0.0	605
ABCDEF_48199	UniRef50_Q6YWK8	0.0	Normal: 1506	467	2429	3094	4233	439	BlastAnno: 1997	UniRef50_Q6YWK8	570	63.10	439	146	2	291	1559	57	495	0.0	610
ABCDEF_40983	UniRef50_Q96301	0.0	Normal: 406	261	343	392	415	433	BlastAnno: 2265	UniRef50_Q96301	914	79.17	504	99	1	114	1625	394	891	0.0	854
ABCDEF_17140	UniRef50_Q9LHZ7	0.0	Normal: 45	1049	49	216	386	208	BlastAnno: 2293	UniRef50_Q9LHZ7	1170	52.34	770	281	13	2138	57	397	1156	0.0	739
ABCDEF_18352	UniRef50_Q9FWA4	0.0	Normal: 2427	2688	2969	5519	6547	4855	BlastAnno: 2224	UniRef50_Q9FWA4	561	69.31	505	148	3	1887	382	61	561	0.0	683
ABCDEF_27767	UniRef50_Q9LHZ7	0.0	Normal: 2576	5141	2841	10249	14583	5031	BlastAnno: 3981	UniRef50_Q9LHZ7	1170	49.66	888	337	16	2927	504	299	1156	0.0	818
ABCDEF_33661	UniRef50_Q9LHZ7	0.0	Normal: 414	340	507	879	790	783	BlastAnno: 2185	UniRef50_Q9LHZ7	1170	48.66	748	277	14	2184	169	440	1156	0.0	662
ABCDEF_14685	UniRef50_A7XDQ9	0.0	Normal: 233	308	134	573	292	334	BlastAnno: 1947	UniRef50_A7XDQ9	684	56.84	468	186	6	569	1945	1	461	0.0	545
ABCDEF_20660	UniRef50_Q8VZK9	0.0	Normal: 22	48	13	15	52	144	BlastAnno: 1473	UniRef50_Q8VZK9	729	55.16	475	199	6	55	1473	11	473	0.0	537
ABCDEF_21315	UniRef50_Q9LHZ7	0.0	Normal: 3412	252	1450	0	79	1228	BlastAnno: 3765	UniRef50_Q9LHZ7	1170	63.78	1096	340	15	308	3508	70	1137	0.0	1333
ABCDEF_533	UniRef50_Q9C8W3	0.0	Normal: 230	204	269	505	411	301	BlastAnno: 1688	UniRef50_Q9C8W3	401	70.33	364	94	2	393	1475	47	399	0.0	555
ABCDEF_34099	UniRef50_Q9LE59	0.0	Normal: 478	491	735	1356	1350	548	BlastAnno: 2158	UniRef50_Q9LE59	673	60.92	458	178	1	1610	240	215	672	0.0	630
ABCDEF_47984	UniRef50_Q7PC69	0.0	Normal: 663	457	734	1362	2147	696	BlastAnno: 2838	UniRef50_Q7PC69	745	59.27	712	226	9	373	2316	34	745	0.0	702
ABCDEF_51451	UniRef50_Q9LE59	0.0	Normal: 1883	1782	2202	4337	4350	2667	BlastAnno: 2874	UniRef50_Q9LE59	673	74.16	685	165	6	2559	505	1	673	0.0	982
ABCDEF_13370	UniRef50_Q8VZK9	0.0	Normal: 290	87	317	377	105	258	BlastAnno: 2033	UniRef50_Q8VZK9	729	51.65	635	278	11	2031	154	112	726	0.0	612
ABCDEF_48239	UniRef50_Q9LHZ7	0.0	Normal: 511	163	422	1154	1316	510	BlastAnno: 2714	UniRef50_Q9LHZ7	1170	76.20	769	156	9	478	2712	41	806	0.0	1201
ABCDEF_13338	UniRef50_Q7PC69	0.0	Normal: 335	468	417	2639	4443	616	BlastAnno: 2525	UniRef50_Q7PC69	745	62.45	727	209	12	298	2292	21	745	0.0	781
ABCDEF_24193	UniRef50_Q8L7F9	0.0	Normal: 113	72	132	203	180	119	BlastAnno: 1679	UniRef50_Q8L7F9	643	60.54	479	176	4	212	1609	1	479	0.0	592
ABCDEF_48514	UniRef50_Q9ZV71	0.0	Normal: 540	580	601	771	1033	926	BlastAnno: 1920	UniRef50_Q9ZV71	409	78.14	398	85	2	1618	431	12	409	0.0	637

2.4.1.159(flavonol-3-O-glucoside L-rhamnosyltransferase;)
No Correspond Unigene
2.4.1.189(luteolin 7-O-glucuronosyltransferase;)
No Correspond Unigene
2.4.1.190(luteolin-7-O-glucuronide 2’’-O-glucuronosyltransferase;)
No Correspond Unigene
2.4.1.191(luteolin-7-O-diglucuronide 4’-O-glucuronosyltransferase;)
No Correspond Unigene
2.4.1.234(kaempferol 3-O-galactosyltransferase;)
No Correspond Unigene
2.4.1.239(flavonol-3-O-glucoside glucosyltransferase;)
No Correspond Unigene
2.4.1.240(flavonol-3-O-glycoside glucosyltransferase)
No Correspond Unigene
2.4.1.81(flavone 7-O-beta-glucosyltransferase;)
No Correspond Unigene
2.4.1.91(flavonol 3-O-glucosyltransferase;)

ABCDEF_34091	UniRef50_Q6WFW1	2e-105	Normal: 919	313	1164	1012	309	546	BlastAnno: 1654	UniRef50_Q6WFW1	475	42.17	460	232	13	1553	222	2	443	2e-105	333

2.4.2.25(flavone apiosyltransferase;)
No Correspond Unigene
2.8.2.25(flavonol 3-sulfotransferase)
No Correspond Unigene
2.8.2.26(quercetin-3-sulfate 3’-sulfotransferase;)
No Correspond Unigene
2.8.2.27(quercetin-3-sulfate 4’-sulfotransferase;)

ABCDEF_18473	UniRef50_P52837	2e-84	Normal: 326	485	563	486	1413	239	BlastAnno: 1205	UniRef50_P52837	320	47.32	298	144	5	1040	156	32	319	2e-84	269

2.8.2.28(quercetin-3,3’-bissulfate 7-sulfotransferase;)
No Correspond Unigene
3.2.1.31(beta-glucuronidase;)
No Correspond Unigene

>00945 Stilbenoid, diarylheptanoid and gingerol biosynthesis

1.14.-.-(Oxidoreductases;)

ABCDEF_44739	UniRef50_O64989	0.0	Normal: 157	87	159	718	673	189	BlastAnno: 2185	UniRef50_O64989	513	70.75	482	112	3	1867	509	30	511	0.0	706
ABCDEF_31491	UniRef50_Q93VK5	0.0	Normal: 647	1826	671	1517	1770	5953	BlastAnno: 2278	UniRef50_Q93VK5	595	79.14	556	113	2	198	1862	35	588	0.0	902
ABCDEF_40554	UniRef50_P37122	0.0	Normal: 1574	1962	1829	1150	2882	2721	BlastAnno: 1828	UniRef50_P37122	505	60.52	504	198	1	1752	244	1	504	0.0	638
ABCDEF_34049	UniRef50_O23365	0.0	Normal: 363	384	349	442	549	1416	BlastAnno: 2101	UniRef50_O23365	580	76.17	579	116	5	230	1957	19	578	0.0	904
ABCDEF_21579	UniRef50_O48928	0.0	Normal: 1005	643	1606	4372	3226	1621	BlastAnno: 1895	UniRef50_O48928	513	69.72	502	151	1	185	1690	7	507	0.0	731
ABCDEF_37741	UniRef50_P37122	0.0	Normal: 12	760	9	24	34	576	BlastAnno: 1197	UniRef50_P37122	505	65.70	379	129	1	1195	59	127	504	0.0	526
ABCDEF_35618	UniRef50_O48928	0.0	Normal: 28	2648	17	142	1234	902	BlastAnno: 1746	UniRef50_O48928	513	66.40	506	168	2	1649	132	4	507	0.0	701
ABCDEF_24522	UniRef50_P37122	0.0	Normal: 334	2190	561	268	1391	2459	BlastAnno: 2075	UniRef50_P37122	505	61.52	460	176	1	1544	165	47	505	0.0	612
ABCDEF_14592	UniRef50_O23066	0.0	Normal: 1514	402	2245	7837	4742	1248	BlastAnno: 2378	UniRef50_O23066	553	72.71	546	140	4	2111	489	1	542	0.0	821
ABCDEF_14512	UniRef50_Q9FG65	0.0	Normal: 261	872	212	291	175	789	BlastAnno: 1878	UniRef50_Q9FG65	502	56.89	501	197	9	1738	266	11	502	0.0	532
ABCDEF_44487	UniRef50_Q940V4	0.0	Normal: 3	41	53	501	262	542	BlastAnno: 1712	UniRef50_Q940V4	465	69.37	382	116	1	38	1180	14	395	0.0	572
ABCDEF_17723	UniRef50_P37122	0.0	Normal: 240	1698	219	768	592	794	BlastAnno: 1757	UniRef50_P37122	505	63.40	459	166	2	228	1601	47	504	0.0	617
ABCDEF_37901	UniRef50_Q42569	0.0	Normal: 30	149	86	255	195	255	BlastAnno: 1246	UniRef50_Q42569	472	78.27	359	73	2	64	1140	43	396	0.0	567
ABCDEF_7260	UniRef50_O81117	0.0	Normal: 314	1085	466	3248	2575	1360	BlastAnno: 1768	UniRef50_O81117	514	59.92	474	188	1	145	1566	43	514	0.0	615
ABCDEF_40548	UniRef50_P37122	0.0	Normal: 2756	2467	2752	1072	3120	1735	BlastAnno: 1947	UniRef50_P37122	505	59.72	504	202	1	241	1749	1	504	0.0	636
ABCDEF_44485	UniRef50_Q940V4	0.0	Normal: 3	55	63	574	338	660	BlastAnno: 1607	UniRef50_Q940V4	465	68.58	452	141	1	42	1394	14	465	0.0	673
ABCDEF_40552	UniRef50_P37122	0.0	Normal: 2397	1870	2304	1249	10428	2291	BlastAnno: 1812	UniRef50_P37122	505	60.12	504	200	1	108	1616	1	504	0.0	640
ABCDEF_37902	UniRef50_Q42569	0.0	Normal: 84	524	254	725	670	1049	BlastAnno: 1812	UniRef50_Q42569	472	77.47	435	92	3	1675	371	43	471	0.0	681
ABCDEF_40556	UniRef50_P37122	0.0	Normal: 1907	2365	2163	1319	3678	3308	BlastAnno: 1944	UniRef50_P37122	505	61.04	498	193	1	264	1754	1	498	0.0	635
ABCDEF_40549	UniRef50_P37122	0.0	Normal: 1574	1962	1826	1150	2882	2723	BlastAnno: 1845	UniRef50_P37122	505	60.52	504	198	1	1754	246	1	504	0.0	638
ABCDEF_20301	UniRef50_O81117	0.0	Normal: 89	308	123	176	389	249	BlastAnno: 1637	UniRef50_O81117	514	64.04	470	164	3	1498	98	47	514	0.0	586
ABCDEF_3882	UniRef50_Q42569	0.0	Normal: 84	243	257	786	1561	182	BlastAnno: 1854	UniRef50_Q42569	472	72.51	422	107	1	1852	587	59	471	0.0	615
ABCDEF_20703	UniRef50_O49858	0.0	Normal: 115	636	20	0	0	597	BlastAnno: 1815	UniRef50_O49858	527	50.10	523	248	6	48	1607	12	524	0.0	533
ABCDEF_18295	UniRef50_O22203	0.0	Normal: 3667	1934	4977	10491	4588	1917	BlastAnno: 2006	UniRef50_O22203	508	74.85	493	123	1	214	1692	17	508	0.0	803
ABCDEF_48724	UniRef50_O23066	0.0	Normal: 104	11518	163	2083	3464	6356	BlastAnno: 2162	UniRef50_O23066	553	72.96	540	143	3	227	1840	1	539	0.0	807
ABCDEF_37738	UniRef50_P37122	0.0	Normal: 26	2447	19	30	33	993	BlastAnno: 1837	UniRef50_P37122	505	64.30	479	169	2	1685	249	28	504	0.0	639

1.14.13.-(Oxidoreductases;)

ABCDEF_45538	UniRef50_O23024	3e-156	Normal: 110	6	165	774	133	36	BlastAnno: 1258	UniRef50_O23024	437	58.18	385	151	3	3	1157	37	411	3e-156	457

1.14.13.11(trans-cinnamate 4-monooxygenase;)

ABCDEF_71482	UniRef50_P92994	0.0	Normal: 2048	1264	3828	9981	3885	695	BlastAnno: 1893	UniRef50_P92994	505	84.49	490	76	0	107	1576	14	503	0.0	797
ABCDEF_41092	UniRef50_P92994	0.0	Normal: 2785	1577	4558	11362	4868	724	BlastAnno: 2059	UniRef50_P92994	505	83.70	503	82	0	1826	318	1	503	0.0	788
ABCDEF_41091	UniRef50_P92994	0.0	Normal: 946	733	2221	5924	1901	1102	BlastAnno: 1557	UniRef50_P92994	505	87.56	442	55	0	1556	231	62	503	0.0	754
ABCDEF_41093	UniRef50_P92994	0.0	Normal: 1164	920	2732	7588	2245	1278	BlastAnno: 1828	UniRef50_P92994	505	86.56	491	66	0	1722	250	14	504	0.0	814

1.14.13.36(5-O-(4-coumaroyl)-D-quinate 3’-monooxygenase;)
No Correspond Unigene
2.1.1.-(Transferases;)

ABCDEF_20806	UniRef50_Q94II3	0.0	Normal: 700	332	876	1458	1997	929	BlastAnno: 2275	UniRef50_Q94II3	600	73.57	594	153	4	2119	341	1	591	0.0	907
ABCDEF_45802	UniRef50_Q9ZPH9	0.0	Normal: 3543	3348	4296	9797	10830	5017	BlastAnno: 2642	UniRef50_Q9ZPH9	633	53.90	603	261	7	2216	456	23	624	0.0	676
ABCDEF_20070	UniRef50_Q944R7	0.0	Normal: 301	141	265	207	185	187	BlastAnno: 2675	UniRef50_Q944R7	724	58.28	719	275	9	127	2265	13	712	0.0	840
ABCDEF_34956	UniRef50_Q94II3	0.0	Normal: 1120	7269	2475	16033	22219	21461	BlastAnno: 2676	UniRef50_Q94II3	600	73.77	610	146	4	404	2230	1	597	0.0	957

ABCDEF_17121	UniRef50_Q9ZPH9	0.0	Normal: 124	77	109	284	199	132	BlastAnno: 2066	UniRef50_Q9ZPH9	633	53.24	601	259	8	161	1903	25	623	0.0	644
ABCDEF_6524	UniRef50_Q9ZPH9	0.0	Normal: 471	296	490	753	870	730	BlastAnno: 2602	UniRef50_Q9ZPH9	633	50.89	619	290	5	2221	404	6	623	0.0	659
ABCDEF_56017	UniRef50_Q9ZPH9	0.0	Normal: 302	167	286	596	748	529	BlastAnno: 2384	UniRef50_Q9ZPH9	633	51.37	619	287	5	213	2030	6	623	0.0	663
ABCDEF_44169	UniRef50_Q9SIZ3	0.0	Normal: 179	158	273	705	685	229	BlastAnno: 2300	UniRef50_Q9SIZ3	589	62.01	308	108	4	1219	296	285	583	0.0	394
ABCDEF_6521	UniRef50_Q9ZPH9	0.0	Normal: 339	198	314	641	767	582	BlastAnno: 2511	UniRef50_Q9ZPH9	633	51.37	619	287	5	2172	355	6	623	0.0	663
ABCDEF_44172	UniRef50_Q9SIZ3	0.0	Normal: 204	184	287	767	741	242	BlastAnno: 2317	UniRef50_Q9SIZ3	589	68.15	449	134	4	1637	291	144	583	0.0	641
ABCDEF_199	UniRef50_Q9SU94	0.0	Normal: 1076	275	1009	558	1247	306	BlastAnno: 1769	UniRef50_Q9SU94	390	78.45	348	74	1	284	1324	43	390	0.0	584
ABCDEF_52252	UniRef50_Q84W92	0.0	Normal: 1658	720	1653	1980	2289	1601	BlastAnno: 2170	UniRef50_Q84W92	535	72.49	469	120	3	409	1791	34	501	0.0	703
ABCDEF_51404	UniRef50_A3BMN9	0.0	Normal: 864	188	716	337	631	326	BlastAnno: 2192	UniRef50_A3BMN9	620	50.50	604	243	9	2008	251	55	620	0.0	555
ABCDEF_21386	UniRef50_Q94AI4	0.0	Normal: 2326	1250	2220	4294	2920	1983	BlastAnno: 3701	UniRef50_Q94AI4	775	47.27	842	332	16	567	2978	8	775	0.0	577
ABCDEF_35101	UniRef50_Q9MAT5	0.0	Normal: 1440	420	1268	576	1136	850	BlastAnno: 1634	UniRef50_Q9MAT5	383	69.03	352	109	0	1449	394	32	383	0.0	550
ABCDEF_960	UniRef50_Q9LN50	0.0	Normal: 3504	13198	3609	11156	19651	12155	BlastAnno: 2626	UniRef50_Q9LN50	724	49.14	525	246	5	883	2451	219	724	0.0	570
ABCDEF_48082	UniRef50_Q9LN50	0.0	Normal: 410	445	467	870	993	454	BlastAnno: 2340	UniRef50_Q9LN50	724	70.25	521	155	0	710	2272	204	724	0.0	821
ABCDEF_10473	UniRef50_Q08A71	0.0	Normal: 374	187	332	425	456	314	BlastAnno: 1764	UniRef50_Q08A71	435	79.23	366	75	1	344	1438	70	435	0.0	575
ABCDEF_7295	UniRef50_Q9ZPH9	0.0	Normal: 25	54	30	81	26	329	BlastAnno: 1191	UniRef50_Q9ZPH9	633	69.23	403	110	4	1191	22	225	626	0.0	599
ABCDEF_4160	UniRef50_Q94II3	0.0	Normal: 189	0	210	71	17	72	BlastAnno: 1424	UniRef50_Q94II3	600	77.28	449	101	1	1424	81	143	591	0.0	758
ABCDEF_44857	UniRef50_Q9ZPH9	0.0	Normal: 101	224	103	299	588	350	BlastAnno: 1582	UniRef50_Q9ZPH9	633	72.06	519	143	1	1571	21	114	632	0.0	822
ABCDEF_71541	UniRef50_Q9SIZ3	0.0	Normal: 187	167	296	765	724	237	BlastAnno: 2268	UniRef50_Q9SIZ3	589	62.01	308	108	4	1210	287	285	583	0.0	394
ABCDEF_44171	UniRef50_Q9SIZ3	0.0	Normal: 214	196	312	827	802	251	BlastAnno: 2339	UniRef50_Q9SIZ3	589	64.78	548	182	5	1978	335	47	583	0.0	717
ABCDEF_20805	UniRef50_Q94II3	0.0	Normal: 674	344	885	1417	2194	963	BlastAnno: 2400	UniRef50_Q94II3	600	78.21	436	94	1	879	2183	156	591	0.0	734
ABCDEF_18210	UniRef50_Q9SIZ3	0.0	Normal: 5584	3714	7379	17006	21892	7546	BlastAnno: 3199	UniRef50_Q9SIZ3	589	50.00	576	269	7	2145	424	27	585	0.0	574
ABCDEF_6526	UniRef50_Q9ZPH9	0.0	Normal: 627	185	837	2717	2815	1530	BlastAnno: 2611	UniRef50_Q9ZPH9	633	54.76	599	259	5	462	2222	25	623	0.0	689
ABCDEF_6525	UniRef50_Q9ZPH9	0.0	Normal: 602	165	760	2369	2605	1400	BlastAnno: 2545	UniRef50_Q9ZPH9	633	54.92	599	258	5	431	2191	25	623	0.0	689
ABCDEF_6523	UniRef50_Q9ZPH9	0.0	Normal: 314	175	304	641	784	552	BlastAnno: 2312	UniRef50_Q9ZPH9	633	52.42	599	272	4	201	1958	25	623	0.0	663
ABCDEF_67393	UniRef50_Q9ZPH9	0.0	Normal: 434	245	444	608	768	655	BlastAnno: 2346	UniRef50_Q9ZPH9	633	51.92	599	275	4	232	1989	25	623	0.0	659
ABCDEF_3579	UniRef50_O14744	0.0	Normal: 1361	326	984	568	791	440	BlastAnno: 2369	UniRef50_O14744	637	47.00	634	294	10	267	2120	30	637	0.0	553
2.1.1.104(caffeoyl-CoA O-methyltransferase;)																					
ABCDEF_62277	UniRef50_O65862	2e-162	Normal: 19	362	17	166	258	203	BlastAnno: 1005	UniRef50_O65862	247	89.84	246	20	1	103	825	1	246	2e-162	462
ABCDEF_34441	UniRef50_O65862	2e-162	Normal: 22	354	16	164	260	194	BlastAnno: 992	UniRef50_O65862	247	89.84	246	20	1	103	825	1	246	2e-162	462
2.3.1.-(Transferases;)																					
ABCDEF_520	UniRef50_Q9FF86	0.0	Normal: 1672	85	2273	5056	3383	1211	BlastAnno: 1811	UniRef50_Q9FF86	484	64.06	448	149	6	1664	354	3	449	0.0	573
ABCDEF_68664	UniRef50_Q9ZVN2	0.0	Normal: 79	35	85	107	193	63	BlastAnno: 1488	UniRef50_Q9ZVN2	704	63.99	411	142	3	46	1275	298	703	0.0	540
ABCDEF_52245	UniRef50_Q9FG87	0.0	Normal: 1878	635	1833	3814	1432	1376	BlastAnno: 1758	UniRef50_Q9FG87	529	55.35	486	204	4	4	1428	34	517	0.0	530
ABCDEF_16730	UniRef50_Q3EC11	0.0	Normal: 281	204	237	365	377	458	BlastAnno: 2092	UniRef50_Q3EC11	536	59.33	536	204	8	196	1785	3	530	0.0	661
ABCDEF_4655	UniRef50_A5BMG1	0.0	Normal: 2426	1856	3606	6958	4608	2755	BlastAnno: 2261	UniRef50_A5BMG1	481	68.37	528	112	10	263	1843	3	476	0.0	667
ABCDEF_13860	UniRef50_Q9C533	0.0	Normal: 588	220	570	1451	1338	310	BlastAnno: 2648	UniRef50_Q9C533	596	63.37	606	177	12	2226	511	1	595	0.0	558
ABCDEF_28294	UniRef50_Q9SYZ0	0.0	Normal: 1038	4070	1427	6745	877	2797	BlastAnno: 1785	UniRef50_Q9SYZ0	493	52.37	485	223	3	1598	162	5	487	0.0	533
ABCDEF_47718	UniRef50_Q9C533	0.0	Normal: 1599	236	1657	2093	1231	588	BlastAnno: 2680	UniRef50_Q9C533	596	65.02	626	165	14	2280	472	1	595	0.0	634
ABCDEF_45080	UniRef50_Q9SYZ0	0.0	Normal: 1005	592	1079	2360	3762	2217	BlastAnno: 2130	UniRef50_Q9SYZ0	493	57.85	484	197	3	302	1735	5	487	0.0	533
ABCDEF_25377	UniRef50_Q9C6L5	0.0	Normal: 37	2090	138	2573	1026	1298	BlastAnno: 1334	UniRef50_Q9C6L5	492	79.74	385	76	1	1155	1	6	388	0.0	654
ABCDEF_33470	UniRef50_Q9LW26	0.0	Normal: 172	98	156	260	391	149	BlastAnno: 2993	UniRef50_Q9LW26	701	48.96	627	301	5	655	2508	85	701	0.0	581
ABCDEF_40654	UniRef50_Q9H0A0	0.0	Normal: 638	213	530	318	318	286	BlastAnno: 2216	UniRef50_Q9H0A0	1025	56.87	721	279	6	2127	13	91	795	0.0	756
ABCDEF_33460	UniRef50_Q9ZVN2	0.0	Normal: 144	71	119	216	306	97	BlastAnno: 2691	UniRef50_Q9ZVN2	704	61.50	587	191	4	379	2133	83	636	0.0	715
ABCDEF_14442	UniRef50_Q9SYZ0	0.0	Normal: 341	684	439	1040	1601	552	BlastAnno: 1779	UniRef50_Q9SYZ0	493	63.12	480	171	2	1570	149	5	484	0.0	628
ABCDEF_52626	UniRef50_B6SYP9	0.0	Normal: 815	346	1131	3015	1537	108	BlastAnno: 1861	UniRef50_B6SYP9	494	55.02	438	189	3	1538	249	51	488	0.0	536
ABCDEF_644	UniRef50_Q9SB58	0.0	Normal: 704	444	913	969	1431	848	BlastAnno: 1777	UniRef50_Q9SB58	407	64.15	424	129	8	1572	316	1	406	0.0	554
ABCDEF_27087	UniRef50_Q52T38	0.0	Normal: 324	221	233	419	343	412	BlastAnno: 1631	UniRef50_Q52T38	620	75.95	420	91	1	1409	150	1	410	0.0	681
ABCDEF_34187	UniRef50_Q8L5Y5	0.0	Normal: 497	415	588	523	291	433	BlastAnno: 2677	UniRef50_Q8L5Y5	718	57.30	733	251	15	202	2274	1	713	0.0	683
ABCDEF_47966	UniRef50_Q9SYZ0	0.0	Normal: 577	934	326	580	682	3440	BlastAnno: 2177	UniRef50_Q9SYZ0	493	56.40	484	204	3	1780	347	5	487	0.0	535
ABCDEF_51189	UniRef50_Q52T38	0.0	Normal: 135	152	128	181	219	327	BlastAnno: 1766	UniRef50_Q52T38	620	68.09	470	137	3	80	1483	130	588	0.0	641
ABCDEF_41288	UniRef50_Q9SB58	0.0	Normal: 307	183	332	637	649	322	BlastAnno: 1403	UniRef50_Q9SB58	407	67.56	410	110	6	1401	184	15	405	0.0	523
ABCDEF_33461	UniRef50_Q9ZVN2	0.0	Normal: 142	84	140	206	283	119	BlastAnno: 2904	UniRef50_Q9ZVN2	704	60.98	574	201	6	2271	559	83	636	0.0	686
ABCDEF_14334	UniRef50_Q9SYZ0	0.0	Normal: 2263	129	1724	3469	866	283	BlastAnno: 2039	UniRef50_Q9SYZ0	493	63.22	484	171	2	369	1802	5	487	0.0	627
ABCDEF_24844	UniRef50_Q9FF86	0.0	Normal: 1857	3686	2657	21940	11467	4090	BlastAnno: 1640	UniRef50_Q9FF86	484	66.89	447	135	6	1492	185	2	446	0.0	575
ABCDEF_51190	UniRef50_Q52T38	0.0	Normal: 145	169	142	210	232	335	BlastAnno: 1891	UniRef50_Q52T38	620	70.04	514	141	3	73	1608	86	588	0.0	726
ABCDEF_14085	UniRef50_Q8L5Y5	0.0	Normal: 353	41	384	527	331	280	BlastAnno: 2362	UniRef50_Q8L5Y5	718	62.07	704	220	12	2360	348	24	713	0.0	676
ABCDEF_33458	UniRef50_Q9LW26	0.0	Normal: 158	78	130	240	342	106	BlastAnno: 2822	UniRef50_Q9LW26	701	46.72	655	302	5	400	2337	85	701	0.0	568
ABCDEF_25193	UniRef50_Q9C6L5	0.0	Normal: 3256	368	3548	6230	5109	1288	BlastAnno: 1786	UniRef50_Q9C6L5	492	65.83	480	156	4	1668	241	17	492	0.0	668
ABCDEF_33457	UniRef50_Q9ZVN2	0.0	Normal: 168	83	137	250	375	112	BlastAnno: 2823	UniRef50_Q9ZVN2	704	65.12	496	168	3	379	1860	83	575	0.0	648
ABCDEF_7634	UniRef50_Q9C6L5	0.0	Normal: 2543	9303	2711	11578	5533	10032	BlastAnno: 2064	UniRef50_Q9C6L5	492	52.76	489	225	3	1734	268	1	483	0.0	535
ABCDEF_299	UniRef50_Q71N54	0.0	Normal: 569	1147	511	900	649	1879	BlastAnno: 2017	UniRef50_Q71N54	535	61.73	533	198	5	338	1924	4	534	0.0	677
ABCDEF_27086	UniRef50_Q52T38	0.0	Normal: 784	470	617	843	893	976	BlastAnno: 2398	UniRef50_Q52T38	620	70.80	452	165	4	225	2129	1	618	0.0	887
ABCDEF_51188	UniRef50_Q52T38	0.0	Normal: 273	327	255	415	349	441	BlastAnno: 2324	UniRef50_Q52T38	620	71.30	568	150	3	2041	344	32	588	0.0	815
ABCDEF_44812	UniRef50_Q6DR03	0.0	Normal: 982	902	1005	1071	959	2999	BlastAnno: 2450	UniRef50_Q6DR03	565	54.97	644	198	17	2142	220	1	555	0.0	540
2.3.1.133(shikimate O-hydroxycinnamoyltransferase;)																					
ABCDEF_31225	UniRef50_B9GVM3	0.0	Normal: 101	1473	304	2685	639	932	BlastAnno: 1783	UniRef50_B9GVM3	457	80.32	432	85	0	1664	369	24	455	0.0	741
ABCDEF_17902	UniRef50_B9GVM3	0.0	Normal: 586	820	1166	3765	692	1830	BlastAnno: 1735	UniRef50_B9GVM3	457	59.28	442	174	2	172	1482	17	457	0.0	534
ABCDEF_34985	UniRef50_B9GVM3	0.0	Normal: 2887	3227	1901	629	1634	3256	BlastAnno: 1801	UniRef50_B9GVM3	457	61.47	436	163	1	184	1476	20	455	0.0	577
2.3.1.146(pinosylvin synthase;)																					
No Correspond Unigene																					
2.3.1.95(trihydroxystilbene synthase;)																					
No Correspond Unigene																					

2.3.1.99(quinate O-hydroxycinnamoyltransferase;)

No Correspond Unigene

2.4.1.-(Transferases;)

ABCDEF_18004	UniRef50_Q6YWK8	0.0	Normal: 1562	1736	2009	3452	4438	2400	BlastAnno: 2298	UniRef50_Q6YWK8	570	65.27	429	136	1	1941	694	57	485	0.0	608
ABCDEF_52379	UniRef50_Q9LHZ7	0.0	Normal: 4015	4921	4546	10520	13430	4731	BlastAnno: 3479	UniRef50_Q9LHZ7	1170	48.04	893	342	16	825	3242	299	1156	0.0	787
ABCDEF_48593	UniRef50_Q9SK82	0.0	Normal: 12	420	19	343	763	253	BlastAnno: 1834	UniRef50_Q9SK82	489	60.08	481	186	4	1714	278	10	486	0.0	633
ABCDEF_13375	UniRef50_Q8VZK9	0.0	Normal: 108	123	208	526	34	294	BlastAnno: 1738	UniRef50_Q8VZK9	729	53.62	552	234	8	94	1737	18	551	0.0	582
ABCDEF_40982	UniRef50_Q96301	0.0	Normal: 1037	706	819	1249	1084	920	BlastAnno: 3418	UniRef50_Q96301	914	79.60	902	173	3	243	2948	1	891	0.0	1506
ABCDEF_47674	UniRef50_Q9LHZ7	0.0	Normal: 274	92	202	599	505	280	BlastAnno: 2526	UniRef50_Q9LHZ7	1170	75.93	777	151	8	2260	2	15	779	0.0	1177
ABCDEF_34097	UniRef50_Q9LE59	0.0	Normal: 626	613	923	1797	1829	692	BlastAnno: 2521	UniRef50_Q9LE59	673	60.92	458	178	1	1687	317	215	672	0.0	630
ABCDEF_34098	UniRef50_Q9LE59	0.0	Normal: 457	464	697	1283	1312	539	BlastAnno: 2199	UniRef50_Q9LE59	673	60.92	458	178	1	1687	317	215	672	0.0	628
ABCDEF_10848	UniRef50_Q9M156	0.0	Normal: 157	2988	163	408	772	1566	BlastAnno: 1810	UniRef50_Q9M156	480	60.09	456	176	4	1700	336	4	454	0.0	553
ABCDEF_41182	UniRef50_Q9LHZ7	0.0	Normal: 150	2169	221	814	1407	495	BlastAnno: 3604	UniRef50_Q9LHZ7	1170	51.03	870	333	15	2822	456	299	1156	0.0	826
ABCDEF_40981	UniRef50_Q96301	0.0	Normal: 156	97	110	160	179	173	BlastAnno: 1225	UniRef50_Q96301	914	86.92	344	45	0	1112	81	394	737	0.0	652
ABCDEF_9506	UniRef50_Q9LHZ7	0.0	Normal: 1302	2128	1494	3356	4644	2206	BlastAnno: 3751	UniRef50_Q9LHZ7	1170	49.38	891	343	17	927	3359	299	1161	0.0	812
ABCDEF_9500	UniRef50_Q9LHZ7	0.0	Normal: 183	188	186	576	498	260	BlastAnno: 1717	UniRef50_Q9LHZ7	1170	47.98	644	234	14	3	1715	418	1033	0.0	571
ABCDEF_100	UniRef50_Q9M8Y0	0.0	Normal: 228	309	224	416	461	141	BlastAnno: 2109	UniRef50_Q9M8Y0	977	77.49	533	120	0	1610	12	50	582	0.0	884
ABCDEF_33659	UniRef50_Q9LHZ7	0.0	Normal: 1282	1948	2248	5826	10642	2083	BlastAnno: 4289	UniRef50_Q9LHZ7	1170	48.70	887	345	15	2955	538	299	1156	0.0	788
ABCDEF_37667	UniRef50_Q9LHZ7	0.0	Normal: 248	3617	251	1237	2078	970	BlastAnno: 3161	UniRef50_Q9LHZ7	1170	49.72	885	335	14	2671	260	301	1156	0.0	810
ABCDEF_47673	UniRef50_Q9LHZ7	0.0	Normal: 876	296	828	1628	1721	1172	BlastAnno: 3878	UniRef50_Q9LHZ7	1170	80.88	1161	186	8	3642	232	15	1163	0.0	1865
ABCDEF_99	UniRef50_Q9M8Y0	0.0	Normal: 272	330	222	398	423	145	BlastAnno: 2094	UniRef50_Q9M8Y0	977	77.36	561	127	0	1683	1	50	610	0.0	929
ABCDEF_20658	UniRef50_Q8VZK9	0.0	Normal: 373	33	193	64	503	141	BlastAnno: 2316	UniRef50_Q8VZK9	729	53.35	508	221	7	47	1564	11	504	0.0	559
ABCDEF_10183	UniRef50_A7XDQ9	0.0	Normal: 422	455	346	577	378	610	BlastAnno: 2362	UniRef50_A7XDQ9	684	50.94	693	307	12	281	2287	1	684	0.0	670
ABCDEF_30745	UniRef50_A7XDQ9	0.0	Normal: 1209	351	1031	1645	1226	635	BlastAnno: 2646	UniRef50_A7XDQ9	684	57.58	554	227	6	546	2189	133	684	0.0	664
ABCDEF_13336	UniRef50_Q7PC69	0.0	Normal: 148	563	194	957	1575	816	BlastAnno: 2584	UniRef50_Q7PC69	745	71.56	559	136	6	744	2351	187	745	0.0	730
ABCDEF_21426	UniRef50_Q8RU27	0.0	Normal: 2658	1979	3417	6157	9464	2599	BlastAnno: 1530	UniRef50_Q8RU27	366	89.44	360	34	2	1358	282	1	357	0.0	667
ABCDEF_71242	UniRef50_Q9ZV71	0.0	Normal: 134	122	179	480	754	119	BlastAnno: 1835	UniRef50_Q9ZV71	409	73.35	409	103	4	231	1439	1	409	0.0	587
ABCDEF_52020	UniRef50_Q6YWK8	0.0	Normal: 1635	1534	1975	4284	10353	2289	BlastAnno: 2394	UniRef50_Q6YWK8	570	58.37	514	185	2	403	1857	57	570	0.0	607
ABCDEF_51269	UniRef50_Q6YWK8	0.0	Normal: 147	85	103	21	33	438	BlastAnno: 1864	UniRef50_Q6YWK8	570	54.28	514	204	2	1646	198	57	570	0.0	586
ABCDEF_30753	UniRef50_Q9ZV71	0.0	Normal: 290	497	417	629	1013	634	BlastAnno: 1502	UniRef50_Q9ZV71	409	72.32	401	107	2	311	1501	1	401	0.0	590
ABCDEF_4233	UniRef50_Q9ZV71	0.0	Normal: 369	412	550	936	860	750	BlastAnno: 2004	UniRef50_Q9ZV71	409	72.13	409	110	2	370	1584	1	409	0.0	613
ABCDEF_17525	UniRef50_Q8RU27	0.0	Normal: 1500	1289	2149	3711	4446	1069	BlastAnno: 1507	UniRef50_Q8RU27	366	91.35	347	30	0	1371	331	3	349	0.0	671
ABCDEF_18005	UniRef50_Q6YWK8	0.0	Normal: 1842	2042	2515	4214	5208	2842	BlastAnno: 2305	UniRef50_Q6YWK8	570	59.14	514	180	2	1948	497	57	570	0.0	642
ABCDEF_41140	UniRef50_Q6YWK8	0.0	Normal: 86	1314	93	753	1787	2076	BlastAnno: 2346	UniRef50_Q6YWK8	570	60.00	510	174	2	431	1870	57	566	0.0	669
ABCDEF_18002	UniRef50_Q9LHZ7	0.0	Normal: 376	127	367	601	1190	520	BlastAnno: 1605	UniRef50_Q9LHZ7	1170	89.75	400	41	0	1603	404	771	1170	0.0	732
ABCDEF_13339	UniRef50_Q7PC69	0.0	Normal: 127	814	183	541	1135	1144	BlastAnno: 2610	UniRef50_Q7PC69	745	70.48	559	141	6	725	2329	187	745	0.0	729
ABCDEF_41141	UniRef50_Q6YWK8	0.0	Normal: 67	1107	84	708	1690	1854	BlastAnno: 2461	UniRef50_Q6YWK8	570	60.00	510	174	2	1916	477	57	566	0.0	668
ABCDEF_44583	UniRef50_Q9LE59	0.0	Normal: 681	561	559	947	1745	789	BlastAnno: 2470	UniRef50_Q9LE59	673	59.21	456	185	1	1749	385	214	669	0.0	621
ABCDEF_3869	UniRef50_Q9LE59	0.0	Normal: 333	393	415	809	907	754	BlastAnno: 2136	UniRef50_Q9LE59	673	59.05	613	227	4	1827	1	81	673	0.0	699
ABCDEF_28947	UniRef50_Q9SK82	0.0	Normal: 0	851	3	0	0	116	BlastAnno: 1567	UniRef50_Q9SK82	489	62.40	484	176	4	1524	85	1	482	0.0	652
ABCDEF_31119	UniRef50_Q9M8Y0	0.0	Normal: 2212	1279	1917	2933	3341	1803	BlastAnno: 3695	UniRef50_Q9M8Y0	977	81.08	925	174	1	392	3163	50	974	0.0	1583
ABCDEF_37311	UniRef50_Q9LE59	0.0	Normal: 175	133	191	549	421	366	BlastAnno: 1612	UniRef50_Q9LE59	673	62.96	486	153	4	1406	3	4	480	0.0	542
ABCDEF_24190	UniRef50_Q8L7F9	0.0	Normal: 397	265	492	583	553	371	BlastAnno: 2564	UniRef50_Q8L7F9	643	63.91	640	218	4	2353	473	1	640	0.0	850
ABCDEF_30548	UniRef50_Q9FH36	0.0	Normal: 17	916	27	151	420	126	BlastAnno: 1963	UniRef50_Q9FH36	535	72.47	534	138	3	102	1676	1	534	0.0	767
ABCDEF_27416	UniRef50_Q7PC69	0.0	Normal: 575	837	875	1344	2015	936	BlastAnno: 2454	UniRef50_Q7PC69	745	66.11	416	115	4	2337	1168	126	541	0.0	518
ABCDEF_48373	UniRef50_A7XDQ9	0.0	Normal: 1364	773	1336	1451	1791	1202	BlastAnno: 3042	UniRef50_A7XDQ9	684	63.68	691	234	6	2405	360	1	683	0.0	915
ABCDEF_3868	UniRef50_Q9LE59	0.0	Normal: 224	274	301	555	686	524	BlastAnno: 1670	UniRef50_Q9LE59	673	65.31	516	176	1	1556	9	161	673	0.0	696
ABCDEF_31288	UniRef50_Q940V3	0.0	Normal: 247	309	183	101	124	655	BlastAnno: 1658	UniRef50_Q940V3	470	56.01	466	197	7	1614	232	8	470	0.0	537
ABCDEF_52574	UniRef50_Q09332	0.0	Normal: 885	474	1068	1835	1853	816	BlastAnno: 3720	UniRef50_Q09332	1548	47.89	687	310	14	2551	533	859	1511	0.0	598
ABCDEF_79752	UniRef50_Q9ZV71	0.0	Normal: 158	183	245	400	379	352	BlastAnno: 1266	UniRef50_Q9ZV71	409	71.39	360	99	2	31	1098	1	360	0.0	526
ABCDEF_9509	UniRef50_Q9LHZ7	0.0	Normal: 1296	2171	1498	3410	4683	2232	BlastAnno: 3897	UniRef50_Q9LHZ7	1170	49.38	891	343	17	1073	3505	299	1161	0.0	812
ABCDEF_48200	UniRef50_Q6YWK8	0.0	Normal: 1768	576	3156	4431	6634	491	BlastAnno: 2274	UniRef50_Q6YWK8	570	57.98	514	185	4	291	1739	57	570	0.0	642
ABCDEF_24155	UniRef50_Q9ZVI7	0.0	Normal: 503	247	607	1676	1833	578	BlastAnno: 2708	UniRef50_Q9ZVI7	619	52.33	621	264	11	306	2123	15	618	0.0	601
ABCDEF_21246	UniRef50_Q9SKT6	0.0	Normal: 92	107	188	380	834	84	BlastAnno: 1791	UniRef50_Q9SKT6	536	56.61	484	204	5	123	1562	52	533	0.0	568
ABCDEF_32317	UniRef50_Q8RU27	0.0	Normal: 12	9	3	118	800	62	BlastAnno: 1076	UniRef50_Q8RU27	366	87.02	285	37	0	1075	221	64	348	0.0	535
ABCDEF_9502	UniRef50_Q9LHZ7	0.0	Normal: 610	486	568	1331	1403	591	BlastAnno: 1961	UniRef50_Q9LHZ7	1170	48.64	625	249	13	3	1742	564	1161	0.0	570
ABCDEF_23298	UniRef50_Q9FH36	0.0	Normal: 128	527	130	223	314	864	BlastAnno: 2832	UniRef50_Q9FH36	535	83.16	196	33	0	696	1283	340	535	0.0	357
ABCDEF_40595	UniRef50_O80898	0.0	Normal: 229	135	103	25	307	325	BlastAnno: 2413	UniRef50_O80898	757	44.68	761	378	13	95	2299	10	753	0.0	587
ABCDEF_63947	UniRef50_Q9SK82	0.0	Normal: 0	267	0	0	71	0	BlastAnno: 1446	UniRef50_Q9SK82	489	58.33	468	189	4	60	1445	1	468	0.0	592
ABCDEF_67847	UniRef50_Q9M8Y0	0.0	Normal: 794	476	871	1081	1401	868	BlastAnno: 1591	UniRef50_Q9M8Y0	977	84.86	370	56	0	1589	480	605	974	0.0	685
ABCDEF_65666	UniRef50_Q09332	0.0	Normal: 800	441	946	1606	1637	735	BlastAnno: 3518	UniRef50_Q09332	1548	47.54	671	305	13	1162	3087	859	1511	0.0	586
ABCDEF_38641	UniRef50_Q9FWA4	0.0	Normal: 674	967	1162	2603	4404	2475	BlastAnno: 1599	UniRef50_Q9FWA4	561	81.35	386	72	0	1	1158	176	561	0.0	641
ABCDEF_17914	UniRef50_Q9SKT6	0.0	Normal: 784	567	886	1447	1439	1424	BlastAnno: 2049	UniRef50_Q9SKT6	536	72.95	536	140	2	204	1796	1	536	0.0	785
ABCDEF_18286	UniRef50_Q6NQB7	0.0	Normal: 1131	1108	1225	1872	1722	3329	BlastAnno: 1752	UniRef50_Q6NQB7	393	73.80	397	81	6	1527	367	10	393	0.0	607
ABCDEF_119	UniRef50_Q8RU27	0.0	Normal: 1613	1155	2249	3558	7663	1775	BlastAnno: 1532	UniRef50_Q8RU27	366	88.33	360	38	2	1306	230	1	357	0.0	663
ABCDEF_47603	UniRef50_Q9M9Y5	0.0	Normal: 2609	8779	1664	1103	2085	22991	BlastAnno: 2830	UniRef50_Q9M9Y5	589	54.04	594	267	3	2475	694	1	588	0.0	654
ABCDEF_24192	UniRef50_Q8L7F9	0.0	Normal: 391	244	465	553	497	345	BlastAnno: 2477	UniRef50_Q8L7F9	643	58.99	434	165	4	212	1474	1	434	0.0	513
ABCDEF_17482	UniRef50_Q9SKT6	0.0	Normal: 615	379	816	1068	1470	852	BlastAnno: 2083	UniRef50_Q9SKT6	536	71.11	540	147	4	1915	311	1	536	0.0	786
ABCDEF_85909	UniRef50_Q8VZK9	0.0	Normal: 28	45	9	10	44	127	BlastAnno: 1473	UniRef50_Q8VZK9	729	55.37	475	198	6	1419	1	11	473	0.0	536
ABCDEF_9503	UniRef50_Q9LHZ7	0.0	Normal: 487	544	524	1242	1320	833	BlastAnno: 2087	UniRef50_Q9LHZ7	1170	50.35	574	236	8	57	1712	615	1161	0.0	567
ABCDEF_40594	UniRef50_O80898	0.0	Normal: 256	127	102	41	331	326	BlastAnno: 2417	UniRef50_O80898	757	45.76	719	348	12	213	2291	51	753	0.0	585
ABCDEF_17141	UniRef50_Q9LHZ7	0.0	Normal: 74	1734	69	380	701	333	BlastAnno: 3337	UniRef50_Q9LHZ7	1170	50.92	868	335	14	2539	179	299	1156	0.0	816

ABCDEF_33658	UniRef50_Q9LHZ7	0.0	Normal: 384	308	395	805	1003	602	BlastAnno: 2961	UniRef50_Q9LHZ7	1170	48.76	888	343	14	204	2621	299	1156	0.0	792
ABCDEF_20579	UniRef50_Q7PC69	0.0	Normal: 1007	795	1238	2311	2309	1345	BlastAnno: 3113	UniRef50_Q7PC69	745	59.97	767	229	14	2667	535	1	745	0.0	768
ABCDEF_31118	UniRef50_Q9M8Y0	0.0	Normal: 2135	1267	1900	3004	3329	1757	BlastAnno: 3787	UniRef50_Q9M8Y0	977	81.10	709	133	1	1132	3255	266	974	0.0	1217
ABCDEF_9499	UniRef50_Q9LHZ7	0.0	Normal: 205	207	215	632	538	275	BlastAnno: 1794	UniRef50_Q9LHZ7	1170	48.88	667	240	14	11	1792	395	1033	0.0	605
ABCDEF_48199	UniRef50_Q6YWK8	0.0	Normal: 1506	467	2429	3094	4233	439	BlastAnno: 1997	UniRef50_Q6YWK8	570	63.10	439	146	2	291	1559	57	495	0.0	610
ABCDEF_40983	UniRef50_Q96301	0.0	Normal: 406	261	343	392	415	433	BlastAnno: 2265	UniRef50_Q96301	914	79.17	504	99	1	114	1625	394	891	0.0	854
ABCDEF_17140	UniRef50_Q9LHZ7	0.0	Normal: 45	1049	49	216	386	208	BlastAnno: 2293	UniRef50_Q9LHZ7	1170	52.34	770	281	13	2138	57	397	1156	0.0	739
ABCDEF_18352	UniRef50_Q9FWA4	0.0	Normal: 2427	2688	2969	5519	6547	4855	BlastAnno: 2224	UniRef50_Q9FWA4	561	69.31	505	148	3	1887	382	61	561	0.0	683
ABCDEF_27767	UniRef50_Q9LHZ7	0.0	Normal: 2576	5141	2841	10249	14583	5031	BlastAnno: 3981	UniRef50_Q9LHZ7	1170	49.66	888	337	16	2927	504	299	1156	0.0	818
ABCDEF_33661	UniRef50_Q9LHZ7	0.0	Normal: 414	340	507	879	790	783	BlastAnno: 2185	UniRef50_Q9LHZ7	1170	48.66	748	277	14	2184	169	440	1156	0.0	662
ABCDEF_14685	UniRef50_A7XDQ9	0.0	Normal: 233	308	134	573	292	334	BlastAnno: 1947	UniRef50_A7XDQ9	684	56.84	468	186	6	569	1945	1	461	0.0	545
ABCDEF_20660	UniRef50_Q8VZK9	0.0	Normal: 22	48	13	15	52	144	BlastAnno: 1473	UniRef50_Q8VZK9	729	55.16	475	199	6	55	1473	11	473	0.0	537
ABCDEF_21315	UniRef50_Q9LHZ7	0.0	Normal: 3412	252	1450	0	79	1228	BlastAnno: 3765	UniRef50_Q9LHZ7	1170	63.78	1096	340	15	308	3508	70	1137	0.0	1333
ABCDEF_533	UniRef50_Q9C8W3	0.0	Normal: 230	204	269	505	411	301	BlastAnno: 1688	UniRef50_Q9C8W3	401	70.33	364	94	2	393	1475	47	399	0.0	555
ABCDEF_34099	UniRef50_Q9LE59	0.0	Normal: 478	491	735	1356	1350	548	BlastAnno: 2158	UniRef50_Q9LE59	673	60.92	458	178	1	1610	240	215	672	0.0	630
ABCDEF_47984	UniRef50_Q7PC69	0.0	Normal: 663	457	734	1362	2147	696	BlastAnno: 2838	UniRef50_Q7PC69	745	59.27	712	226	9	373	2316	34	745	0.0	702
ABCDEF_51451	UniRef50_Q9LE59	0.0	Normal: 1883	1782	2202	4337	4350	2667	BlastAnno: 2874	UniRef50_Q9LE59	673	74.16	685	165	6	2559	505	1	673	0.0	982
ABCDEF_13370	UniRef50_Q8VZK9	0.0	Normal: 290	87	317	377	105	258	BlastAnno: 2033	UniRef50_Q8VZK9	729	51.65	635	278	11	2031	154	112	726	0.0	612
ABCDEF_48239	UniRef50_Q9LHZ7	0.0	Normal: 511	163	422	1154	1316	510	BlastAnno: 2714	UniRef50_Q9LHZ7	1170	76.20	769	156	9	478	2712	41	806	0.0	1201
ABCDEF_13338	UniRef50_Q7PC69	0.0	Normal: 335	468	417	2639	4443	616	BlastAnno: 2525	UniRef50_Q7PC69	745	62.45	727	209	12	298	2292	21	745	0.0	781
ABCDEF_24193	UniRef50_Q8L7F9	0.0	Normal: 113	72	132	203	180	119	BlastAnno: 1679	UniRef50_Q8L7F9	643	60.54	479	176	4	212	1609	1	479	0.0	592
ABCDEF_48514	UniRef50_Q9ZV71	0.0	Normal: 540	580	601	771	1033	926	BlastAnno: 1920	UniRef50_Q9ZV71	409	78.14	398	85	2	1618	431	12	409	0.0	637
>00950 Isoquinoline alkaloid biosynthesis																					
1.-.-.-(Oxidoreductases)																					
ABCDEF_345	UniRef50_Q6Z690	0.0	Normal: 442	146	449	340	272	314	BlastAnno: 1658	UniRef50_Q6Z690	849	63.42	462	150	5	1657	314	387	843	0.0	589
ABCDEF_24068	UniRef50_Q9FI41	0.0	Normal: 214	316	207	270	263	849	BlastAnno: 1742	UniRef50_Q9FI41	387	73.28	363	96	1	1440	355	4	366	0.0	554
ABCDEF_24070	UniRef50_Q9FI41	0.0	Normal: 189	290	182	231	232	751	BlastAnno: 1479	UniRef50_Q9FI41	387	73.28	363	96	1	1363	278	4	366	0.0	551
ABCDEF_13957	UniRef50_Q9CAE3	0.0	Normal: 399	318	392	489	456	586	BlastAnno: 2488	UniRef50_Q9CAE3	789	80.85	496	93	2	3	1487	285	779	0.0	768
ABCDEF_28410	UniRef50_Q9LIG0	0.0	Normal: 211	366	234	286	433	1504	BlastAnno: 1324	UniRef50_Q9LIG0	330	74.92	331	76	2	1147	173	1	330	0.0	521
ABCDEF_17581	UniRef50_Q8LGI2	0.0	Normal: 758	336	735	426	374	852	BlastAnno: 1543	UniRef50_Q8LGI2	454	68.78	442	136	2	71	1390	8	449	0.0	597
ABCDEF_23753	UniRef50_Q94A68	0.0	Normal: 284	559	215	224	164	519	BlastAnno: 1580	UniRef50_Q94A68	377	81.90	348	63	0	1349	306	30	377	0.0	576
ABCDEF_24071	UniRef50_Q9FI41	0.0	Normal: 235	377	230	278	289	948	BlastAnno: 2090	UniRef50_Q9FI41	387	73.28	363	96	1	1726	641	4	366	0.0	553
1.1.1.-(Oxidoreductases;)																					
ABCDEF_13453	UniRef50_Q9SYM5	0.0	Normal: 225	226	398	1950	2026	421	BlastAnno: 2578	UniRef50_Q9SYM5	669	86.61	672	87	2	298	2313	1	669	0.0	1226
ABCDEF_17342	UniRef50_Q84M96	0.0	Normal: 274	406	272	327	439	625	BlastAnno: 1277	UniRef50_Q84M96	346	73.33	345	91	1	1187	156	2	346	0.0	546
ABCDEF_31147	UniRef50_Q9SYM5	0.0	Normal: 4571	1591	1491	1401	1939	4327	BlastAnno: 2465	UniRef50_Q9SYM5	669	82.76	673	107	4	2397	394	1	669	0.0	1154
ABCDEF_17344	UniRef50_Q84M96	0.0	Normal: 252	373	257	297	380	540	BlastAnno: 1350	UniRef50_Q84M96	346	68.19	371	91	2	1263	154	2	346	0.0	530
ABCDEF_44674	UniRef50_Q9SYM5	0.0	Normal: 3581	2620	4329	9505	11447	4930	BlastAnno: 2634	UniRef50_Q9SYM5	669	87.80	672	78	3	253	2265	1	669	0.0	1241
1.1.1.218(morphine 6-dehydrogenase;)																					
No Correspond Unigene																					
1.1.1.247(codeinone reductase (NADPH))																					
No Correspond Unigene																					
1.1.1.248(salutaridine reductase (NADPH))																					
No Correspond Unigene																					
1.10.3.1(catechol oxidase;)																					
ABCDEF_45625	UniRef50_P43311	9e-157	Normal: 20202	1798	18188	15443	32697	6972	BlastAnno: 2080	UniRef50_P43311	607	49.32	584	252	14	1895	243	34	606	9e-157	475
1.14.13.-(Oxidoreductases;)																					
ABCDEF_45538	UniRef50_O23024	3e-156	Normal: 110	6	165	774	133	36	BlastAnno: 1258	UniRef50_O23024	437	58.18	385	151	3	3	1157	37	411	3e-156	457
1.14.13.37(methyltetrahydroprotoberberine 14-monooxygenase;)																					
No Correspond Unigene																					
1.14.13.55(protopine 6-monooxygenase;)																					
No Correspond Unigene																					
1.14.13.56(dihydrosanguinarine 10-monooxygenase;)																					
No Correspond Unigene																					
1.14.13.57(dihydrochelirubine 12-monooxygenase;)																					
No Correspond Unigene																					
1.14.13.71(N-methylcoclaurine 3'-monooxygenase;)																					
ABCDEF_49517	UniRef50_O64900	4e-71	Normal: 19	273	41	86	43	14	BlastAnno: 1286	UniRef50_O64900	488	43.06	346	183	5	259	1284	41	376	4e-71	240
1.14.16.2(tyrosine 3-monooxygenase;)																					
No Correspond Unigene																					
1.14.18.1(monophenol monooxygenase;)																					
No Correspond Unigene																					
1.14.21.-(Oxidoreductases;)																					
No Correspond Unigene																					
1.14.21.1(No Name)																					
No Correspond Unigene																					
1.14.21.2(No Name)																					
No Correspond Unigene																					
1.14.21.3(berbamunine synthase;)																					
No Correspond Unigene																					
1.14.21.4(salutaridine synthase;)																					
No Correspond Unigene																					

1.14.21.5(No Name)																					
No Correspond Unigene																					
1.21.3.2(columbamine oxidase;)																					
No Correspond Unigene																					
1.21.3.3(reticuline oxidase;)																					
ABCDEF_595	UniRef50_B9SAZ3	5e-148	Normal: 164	2820	133	756	469	762	BlastAnno: 1895	UniRef50_B9SAZ3	639	48.59	496	242	4	108	1595	4	486	5e-148	452
1.3.3.8(tetrahydroberberine oxidase;)																					
No Correspond Unigene																					
1.4.3.2(L-amino-acid oxidase;)																					
ABCDEF_34035	UniRef50_B9R844	0.0	Normal: 973	600	948	1073	764	972	BlastAnno: 3436	UniRef50_B9R844	1947	69.56	611	156	9	1050	2867	1361	1946	0.0	707
1.4.3.21(primary-amine oxidase;)																					
ABCDEF_3522	UniRef50_Q8H1H9	0.0	Normal: 405	723	295	151	922	46	BlastAnno: 2086	UniRef50_Q8H1H9	712	64.54	595	204	6	2	1774	116	707	0.0	811
ABCDEF_3521	UniRef50_Q8H1H9	0.0	Normal: 322	637	263	137	709	53	BlastAnno: 1768	UniRef50_Q8H1H9	712	68.24	510	156	5	1630	113	200	707	0.0	744
1.4.3.4(monoamine oxidase;)																					
ABCDEF_11940	UniRef50_Q8LL67	3e-122	Normal: 41	0	82	2	541	4	BlastAnno: 1557	UniRef50_Q8LL67	496	45.61	410	204	3	2	1183	74	480	3e-122	376
1.4.99.4(aralkylamine dehydrogenase;)																					
No Correspond Unigene																					
1.5.-.-(Oxidoreductases;)																					
No Correspond Unigene																					
1.5.1.27(1,2-dehydroreticulinium reductase (NADPH);)																					
No Correspond Unigene																					
1.5.1.31(berberine reductase;)																					
No Correspond Unigene																					
1.5.3.12(dihydrobenzophenanthridine oxidase)																					
No Correspond Unigene																					
2.1.1.-(Transferases;)																					
ABCDEF_20806	UniRef50_Q94II3	0.0	Normal: 700	332	876	1458	1997	929	BlastAnno: 2275	UniRef50_Q94II3	600	73.57	594	153	4	2119	341	1	591	0.0	907
ABCDEF_45802	UniRef50_Q9ZPH9	0.0	Normal: 3543	3348	4296	9797	10830	5017	BlastAnno: 2642	UniRef50_Q9ZPH9	633	53.90	603	261	7	2216	456	23	624	0.0	676
ABCDEF_20070	UniRef50_Q944R7	0.0	Normal: 301	141	265	207	185	187	BlastAnno: 2675	UniRef50_Q944R7	724	58.28	719	275	9	127	2265	13	712	0.0	840
ABCDEF_34956	UniRef50_Q94II3	0.0	Normal: 1120	7269	2475	16033	22219	21461	BlastAnno: 2676	UniRef50_Q94II3	600	73.77	610	146	4	404	2230	1	597	0.0	957
ABCDEF_17121	UniRef50_Q9ZPH9	0.0	Normal: 124	77	109	284	199	132	BlastAnno: 2066	UniRef50_Q9ZPH9	633	53.24	601	259	8	161	1903	25	623	0.0	644
ABCDEF_6524	UniRef50_Q9ZPH9	0.0	Normal: 471	296	490	753	870	730	BlastAnno: 2602	UniRef50_Q9ZPH9	633	50.89	619	290	5	2221	404	6	623	0.0	659
ABCDEF_56017	UniRef50_Q9ZPH9	0.0	Normal: 302	167	286	596	748	529	BlastAnno: 2384	UniRef50_Q9ZPH9	633	51.37	619	287	5	213	2030	6	623	0.0	663
ABCDEF_44169	UniRef50_Q9SIZ3	0.0	Normal: 179	158	273	705	685	229	BlastAnno: 2300	UniRef50_Q9SIZ3	589	62.01	308	108	4	1219	296	285	583	0.0	394
ABCDEF_6521	UniRef50_Q9ZPH9	0.0	Normal: 339	198	314	641	767	582	BlastAnno: 2511	UniRef50_Q9ZPH9	633	51.37	619	287	5	2172	355	6	623	0.0	663
ABCDEF_44172	UniRef50_Q9SIZ3	0.0	Normal: 204	184	287	767	741	242	BlastAnno: 2317	UniRef50_Q9SIZ3	589	68.15	449	134	4	1637	291	144	583	0.0	641
ABCDEF_199	UniRef50_Q9SU94	0.0	Normal: 1076	275	1009	558	1247	306	BlastAnno: 1769	UniRef50_Q9SU94	390	78.45	348	74	1	284	1324	43	390	0.0	584
ABCDEF_52252	UniRef50_Q84W92	0.0	Normal: 1658	720	1653	1980	2289	1601	BlastAnno: 2170	UniRef50_Q84W92	535	72.49	469	120	3	409	1791	34	501	0.0	703
ABCDEF_51404	UniRef50_A3BMN9	0.0	Normal: 864	188	716	337	631	326	BlastAnno: 2192	UniRef50_A3BMN9	620	50.50	604	243	9	2008	251	55	620	0.0	555
ABCDEF_21386	UniRef50_Q94AI4	0.0	Normal: 2326	1250	2220	4294	2920	1983	BlastAnno: 3701	UniRef50_Q94AI4	775	47.27	842	332	16	567	2978	8	775	0.0	577
ABCDEF_35101	UniRef50_Q9MAT5	0.0	Normal: 1440	420	1268	576	1136	850	BlastAnno: 1634	UniRef50_Q9MAT5	383	69.03	352	109	0	1449	394	32	383	0.0	550
ABCDEF_960	UniRef50_Q9LN50	0.0	Normal: 3504	13198	3609	11156	19651	12155	BlastAnno: 2626	UniRef50_Q9LN50	724	49.14	525	246	5	883	2451	219	724	0.0	570
ABCDEF_48082	UniRef50_Q9LN50	0.0	Normal: 410	445	467	870	993	454	BlastAnno: 2340	UniRef50_Q9LN50	724	70.25	521	155	0	710	2272	204	724	0.0	821
ABCDEF_10473	UniRef50_Q08A71	0.0	Normal: 374	187	332	425	456	314	BlastAnno: 1764	UniRef50_Q08A71	435	79.23	366	75	1	344	1438	70	435	0.0	575
ABCDEF_7295	UniRef50_Q9ZPH9	0.0	Normal: 25	54	30	81	26	329	BlastAnno: 1191	UniRef50_Q9ZPH9	633	69.23	403	110	4	1191	22	225	626	0.0	599
ABCDEF_4160	UniRef50_Q94II3	0.0	Normal: 189	0	210	71	17	72	BlastAnno: 1424	UniRef50_Q94II3	600	77.28	449	101	1	1424	81	143	591	0.0	758
ABCDEF_44857	UniRef50_Q9ZPH9	0.0	Normal: 101	224	103	299	588	350	BlastAnno: 1582	UniRef50_Q9ZPH9	633	72.06	519	143	1	1571	21	114	632	0.0	822
ABCDEF_71541	UniRef50_Q9SIZ3	0.0	Normal: 187	167	296	765	724	237	BlastAnno: 2268	UniRef50_Q9SIZ3	589	62.01	308	108	4	1210	287	285	583	0.0	394
ABCDEF_44171	UniRef50_Q9SIZ3	0.0	Normal: 214	196	312	827	802	251	BlastAnno: 2339	UniRef50_Q9SIZ3	589	64.78	548	182	5	1978	335	47	583	0.0	717
ABCDEF_20805	UniRef50_Q94II3	0.0	Normal: 674	344	885	1417	2194	963	BlastAnno: 2400	UniRef50_Q94II3	600	78.21	436	94	1	879	2183	156	591	0.0	734
ABCDEF_18210	UniRef50_Q9SIZ3	0.0	Normal: 5584	3714	7379	17006	21892	7546	BlastAnno: 3199	UniRef50_Q9SIZ3	589	50.00	576	269	7	2145	424	27	585	0.0	574
ABCDEF_6526	UniRef50_Q9ZPH9	0.0	Normal: 627	185	837	2717	2815	1530	BlastAnno: 2611	UniRef50_Q9ZPH9	633	54.76	599	259	5	462	2222	25	623	0.0	689
ABCDEF_6525	UniRef50_Q9ZPH9	0.0	Normal: 602	165	760	2369	2605	1400	BlastAnno: 2545	UniRef50_Q9ZPH9	633	54.92	599	258	5	431	2191	25	623	0.0	689
ABCDEF_6523	UniRef50_Q9ZPH9	0.0	Normal: 314	175	304	641	784	552	BlastAnno: 2312	UniRef50_Q9ZPH9	633	52.42	599	272	4	201	1958	25	623	0.0	663
ABCDEF_67393	UniRef50_Q9ZPH9	0.0	Normal: 434	245	444	608	768	655	BlastAnno: 2346	UniRef50_Q9ZPH9	633	51.92	599	275	4	232	1989	25	623	0.0	659
ABCDEF_3579	UniRef50_O14744	0.0	Normal: 1361	326	984	568	791	440	BlastAnno: 2369	UniRef50_O14744	637	47.00	634	294	10	267	2120	30	637	0.0	553
2.1.1.115(No Name)																					
No Correspond Unigene																					
2.1.1.116(3'-hydroxy-N-methyl-(S)-coclaurine 4'-O-methyltransferase)																					
ABCDEF_7591	UniRef50_Q9LEL5	7e-72	Normal: 450	1688	372	261	811	936	BlastAnno: 1380	UniRef50_Q9LEL5	350	41.21	347	193	7	1291	257	12	349	7e-72	239
2.1.1.117(No Name)																					
ABCDEF_77382	UniRef50_Q39522	4e-114	Normal: 43	125	33	89	146	144	BlastAnno: 1430	UniRef50_Q39522	381	47.81	366	189	2	259	1353	14	378	4e-114	350
2.1.1.118(columbamine O-methyltransferase)																					
No Correspond Unigene																					
2.1.1.119(10-hydroxydihydrosanguinarine 10-O-methyltransferase)																					
No Correspond Unigene																					
2.1.1.120(12-hydroxydihydrochelirubine 12-O-methyltransferase)																					
No Correspond Unigene																					
2.1.1.121(6-O-methylnorlaudanosoline 5'-O-methyltransferase)																					
No Correspond Unigene																					
2.1.1.122(No Name)																					
No Correspond Unigene																					

2.1.1.128(No Name)																						
	ABCDEF_7591	UniRef50_Q9LEL6	1e-73	Normal: 450	1688	372	261	811	936	BlastAnno: 1380	UniRef50_Q9LEL6	347	43.43	350	189	7	1303	257	4	345	1e-73	243
2.1.1.140(No Name)																						
	No Correspond Unigene																					
2.1.1.147(corydaline synthase)																						
	No Correspond Unigene																					
2.1.1.89(tetrahydrocolumbamine 2-O-methyltransferase;)																						
	No Correspond Unigene																					
2.3.1.-(Transferases;)																						
	ABCDEF_520	UniRef50_Q9FF86	0.0	Normal: 1672	85	2273	5056	3383	1211	BlastAnno: 1811	UniRef50_Q9FF86	484	64.06	448	149	6	1664	354	3	449	0.0	573
	ABCDEF_68664	UniRef50_Q9ZVN2	0.0	Normal: 79	35	85	107	193	63	BlastAnno: 1488	UniRef50_Q9ZVN2	704	63.99	411	142	3	46	1275	298	703	0.0	540
	ABCDEF_52245	UniRef50_Q9FG87	0.0	Normal: 1878	635	1833	3814	1432	1376	BlastAnno: 1758	UniRef50_Q9FG87	529	55.35	486	204	4	4	1428	34	517	0.0	530
	ABCDEF_16730	UniRef50_Q3EC11	0.0	Normal: 281	204	237	365	377	458	BlastAnno: 2092	UniRef50_Q3EC11	536	59.33	536	204	8	196	1785	3	530	0.0	661
	ABCDEF_4655	UniRef50_A5BMG1	0.0	Normal: 2426	1856	3606	6958	4608	2755	BlastAnno: 2261	UniRef50_A5BMG1	481	68.37	528	112	10	263	1843	3	476	0.0	667
	ABCDEF_13860	UniRef50_Q9C533	0.0	Normal: 588	220	570	1451	1338	310	BlastAnno: 2648	UniRef50_Q9C533	596	63.37	606	177	12	2226	511	1	595	0.0	558
	ABCDEF_28294	UniRef50_Q9SYZ0	0.0	Normal: 1038	4070	1427	6745	877	2797	BlastAnno: 1785	UniRef50_Q9SYZ0	493	52.37	485	223	3	1598	162	5	487	0.0	533
	ABCDEF_47718	UniRef50_Q9C533	0.0	Normal: 1599	236	1657	2093	1231	588	BlastAnno: 2680	UniRef50_Q9C533	596	65.02	626	165	14	2280	472	1	595	0.0	634
	ABCDEF_45080	UniRef50_Q9SYZ0	0.0	Normal: 1005	592	1079	2360	3762	2217	BlastAnno: 2130	UniRef50_Q9SYZ0	493	57.85	484	197	3	302	1735	5	487	0.0	533
	ABCDEF_25377	UniRef50_Q9C6L5	0.0	Normal: 37	2090	138	2573	1026	1298	BlastAnno: 1334	UniRef50_Q9C6L5	492	79.74	385	76	1	1155	1	6	388	0.0	654
	ABCDEF_33470	UniRef50_Q9LW26	0.0	Normal: 172	98	156	260	391	149	BlastAnno: 2993	UniRef50_Q9LW26	701	48.96	627	301	5	655	2508	85	701	0.0	581
	ABCDEF_40654	UniRef50_Q9H0A0	0.0	Normal: 638	213	530	318	318	286	BlastAnno: 2216	UniRef50_Q9H0A0	1025	56.87	721	279	6	2127	13	91	795	0.0	756
	ABCDEF_33460	UniRef50_Q9ZVN2	0.0	Normal: 144	71	119	216	306	97	BlastAnno: 2691	UniRef50_Q9ZVN2	704	61.50	587	191	4	379	2133	83	636	0.0	715
	ABCDEF_14442	UniRef50_Q9SYZ0	0.0	Normal: 341	684	439	1040	1601	552	BlastAnno: 1779	UniRef50_Q9SYZ0	493	63.12	480	171	2	1570	149	5	484	0.0	628
	ABCDEF_52626	UniRef50_B6SYP9	0.0	Normal: 815	346	1131	3015	1537	108	BlastAnno: 1861	UniRef50_B6SYP9	494	55.02	438	189	3	1538	249	51	488	0.0	536
	ABCDEF_644	UniRef50_Q9SB58	0.0	Normal: 704	444	913	969	1431	848	BlastAnno: 1777	UniRef50_Q9SB58	407	64.15	424	129	8	1572	316	1	406	0.0	554
	ABCDEF_27087	UniRef50_Q52T38	0.0	Normal: 324	221	233	419	343	412	BlastAnno: 1631	UniRef50_Q52T38	620	75.95	420	91	1	1409	150	1	410	0.0	681
	ABCDEF_34187	UniRef50_Q8L5Y5	0.0	Normal: 497	415	588	523	291	433	BlastAnno: 2677	UniRef50_Q8L5Y5	718	57.30	733	251	15	202	2274	1	713	0.0	683
	ABCDEF_47966	UniRef50_Q9SYZ0	0.0	Normal: 577	934	326	580	682	3440	BlastAnno: 2177	UniRef50_Q9SYZ0	493	56.40	484	204	3	1780	347	5	487	0.0	535
	ABCDEF_51189	UniRef50_Q52T38	0.0	Normal: 135	152	128	181	219	327	BlastAnno: 1766	UniRef50_Q52T38	620	68.09	470	137	3	80	1483	130	588	0.0	641
	ABCDEF_41288	UniRef50_Q9SB58	0.0	Normal: 307	183	332	637	649	322	BlastAnno: 1403	UniRef50_Q9SB58	407	67.56	410	110	6	1401	184	15	405	0.0	523
	ABCDEF_33461	UniRef50_Q9ZVN2	0.0	Normal: 142	84	140	206	283	119	BlastAnno: 2904	UniRef50_Q9ZVN2	704	60.98	574	201	6	2271	559	83	636	0.0	686
	ABCDEF_14334	UniRef50_Q9SYZ0	0.0	Normal: 2263	129	1724	3469	866	283	BlastAnno: 2039	UniRef50_Q9SYZ0	493	63.22	484	171	2	369	1802	5	487	0.0	627
	ABCDEF_24844	UniRef50_Q9FF86	0.0	Normal: 1857	3686	2657	21940	11467	4090	BlastAnno: 1640	UniRef50_Q9FF86	484	66.89	447	135	6	1492	185	2	446	0.0	575
	ABCDEF_51190	UniRef50_Q52T38	0.0	Normal: 145	169	142	210	232	335	BlastAnno: 1891	UniRef50_Q52T38	620	70.04	514	141	3	73	1608	86	588	0.0	726
	ABCDEF_14085	UniRef50_Q8L5Y5	0.0	Normal: 353	41	384	527	331	280	BlastAnno: 2362	UniRef50_Q8L5Y5	718	62.07	704	220	12	2360	348	24	713	0.0	676
	ABCDEF_33458	UniRef50_Q9LW26	0.0	Normal: 158	78	130	240	342	106	BlastAnno: 2822	UniRef50_Q9LW26	701	46.72	655	302	5	400	2337	85	701	0.0	568
	ABCDEF_25193	UniRef50_Q9C6L5	0.0	Normal: 3256	368	3548	6230	5109	1288	BlastAnno: 1786	UniRef50_Q9C6L5	492	65.83	480	156	4	1668	241	17	492	0.0	668
	ABCDEF_33457	UniRef50_Q9ZVN2	0.0	Normal: 168	83	137	250	375	112	BlastAnno: 2823	UniRef50_Q9ZVN2	704	65.12	496	168	3	379	1860	83	575	0.0	648
	ABCDEF_7634	UniRef50_Q9C6L5	0.0	Normal: 2543	9303	2711	11578	5533	10032	BlastAnno: 2064	UniRef50_Q9C6L5	492	52.76	489	225	3	1734	268	1	483	0.0	535
	ABCDEF_299	UniRef50_Q71N54	0.0	Normal: 569	1147	511	900	649	1879	BlastAnno: 2017	UniRef50_Q71N54	535	61.73	533	198	5	338	1924	4	534	0.0	677
	ABCDEF_27086	UniRef50_Q52T38	0.0	Normal: 784	470	617	843	893	976	BlastAnno: 2398	UniRef50_Q52T38	620	70.80	637	165	4	225	2129	1	618	0.0	887
	ABCDEF_51188	UniRef50_Q52T38	0.0	Normal: 273	327	255	415	349	441	BlastAnno: 2324	UniRef50_Q52T38	620	71.30	568	150	3	2041	344	32	588	0.0	815
	ABCDEF_44812	UniRef50_Q6DR03	0.0	Normal: 982	902	1005	1071	959	2999	BlastAnno: 2450	UniRef50_Q6DR03	565	54.97	644	198	17	2142	220	1	555	0.0	540
2.3.1.150(salutaridinol 7-O-acetyltransferase)																						
	ABCDEF_8475	UniRef50_B9RMK7	4e-80	Normal: 112	14	35	0	0	653	BlastAnno: 1543	UniRef50_B9RMK7	435	38.57	420	238	9	1417	179	1	407	4e-80	265
2.6.1.1(aspartate transaminase;)																						
	ABCDEF_11690	UniRef50_P46248	0.0	Normal: 1745	1815	2022	4078	5299	6095	BlastAnno: 1778	UniRef50_P46248	453	86.09	417	58	0	297	1547	37	453	0.0	733
	ABCDEF_1333	UniRef50_C0PSV4	0.0	Normal: 356	544	636	1119	2495	631	BlastAnno: 1594	UniRef50_C0PSV4	462	76.43	403	95	0	1401	193	60	462	0.0	662
	ABCDEF_21786	UniRef50_C0PSV4	0.0	Normal: 268	668	330	546	1267	646	BlastAnno: 1736	UniRef50_C0PSV4	462	74.14	464	113	3	50	1441	4	460	0.0	711
	ABCDEF_42262	UniRef50_P46643	0.0	Normal: 916	606	1656	2699	2553	856	BlastAnno: 1722	UniRef50_P46643	430	79.49	429	85	2	1635	355	1	428	0.0	731
	ABCDEF_56929	UniRef50_P37833	0.0	Normal: 173	278	312	654	1243	297	BlastAnno: 1151	UniRef50_P37833	407	81.82	308	56	0	978	55	3	310	0.0	536
2.6.1.5(tyrosine transaminase;)																						
	ABCDEF_52650	UniRef50_Q9LVY1	0.0	Normal: 493	358	455	302	300	892	BlastAnno: 1442	UniRef50_Q9LVY1	420	61.26	413	160	0	65	1303	5	417	0.0	539
2.6.1.57(aromatic-amino-acid transaminase;)																						
	No Correspond Unigene																					
4.1.1.25(tyrosine decarboxylase;)																						
	ABCDEF_28052	UniRef50_Q8RY79	0.0	Normal: 150	74	133	88	177	332	BlastAnno: 1237	UniRef50_Q8RY79	490	74.93	355	89	0	1237	173	134	488	0.0	551
4.1.1.28(aromatic-L-amino-acid decarboxylase;)																						
	ABCDEF_28052	UniRef50_P54770	3e-145	Normal: 150	74	133	88	177	332	BlastAnno: 1237	UniRef50_P54770	533	55.61	374	141	2	1237	191	144	517	3e-145	433
4.1.1.80(4-hydroxyphenylpyruvate decarboxylase;)																						
	No Correspond Unigene																					
4.2.1.78(No Name)																						
	ABCDEF_34762	UniRef50_A2A1A0	1e-95	Normal: 35	173	14	34	214	628	BlastAnno: 1587	UniRef50_A2A1A0	352	45.01	351	177	6	261	1283	8	352	1e-95	303
4.3.3.3(deacetylisoipecoside synthase;)																						
	No Correspond Unigene																					
4.3.3.4(deacetylipecoside synthase;)																						
	No Correspond Unigene																					
>00960 Tropane, piperidine and pyridine alkaloid biosynthesis																						
1.1.1.206(tropinone reductase I;)																						
	ABCDEF_60933	UniRef50_B2J733	9e-58	Normal: 7	69	3	7	4	132	BlastAnno: 674	UniRef50_B2J733	262	55.15	165	74	0	27	521	98	262	9e-58	191
1.1.1.222(No Name)																						
	No Correspond Unigene																					

1.1.1.236(tropinone reductase II;)																					
ABCDEF_37248	UniRef50_P50163	6e-103	Normal: 278	67	153	130	0	6	BlastAnno: 890	UniRef50_P50163	260	56.47	255	110	1	795	34	3	257	6e-103	310
1.1.1.237(hydroxyphenylpyruvate reductase;)																					
No Correspond Unigene																					
1.14.11.11(hyoscyamine (6S)-dioxygenase;)																					
No Correspond Unigene																					
1.14.11.14(6beta-hydroxyhyoscyamine epoxidase;)																					
No Correspond Unigene																					
1.14.13.101(senecionine N-oxygenase;)																					
No Correspond Unigene																					
1.4.1.18(lysine 6-dehydrogenase;)																					
No Correspond Unigene																					
1.4.3.14(L-lysine oxidase;)																					
No Correspond Unigene																					
1.4.3.21(primary-amine oxidase;)																					
ABCDEF_3522	UniRef50_Q8H1H9	0.0	Normal: 405	723	295	151	922	46	BlastAnno: 2086	UniRef50_Q8H1H9	712	64.54	595	204	6	2	1774	116	707	0.0	811
ABCDEF_3521	UniRef50_Q8H1H9	0.0	Normal: 322	637	263	137	709	53	BlastAnno: 1768	UniRef50_Q8H1H9	712	68.24	510	156	5	1630	113	200	707	0.0	744
1.5.1.1(pyrroline-2-carboxylate reductase;)																					
No Correspond Unigene																					
1.5.1.21(Delta1-piperideine-2-carboxylate reductase;)																					
No Correspond Unigene																					
2.1.1.53(putrescine N-methyltransferase;)																					
ABCDEF_48978	UniRef50_Q9SEH5	0.0	Normal: 144	209	131	0	84	2384	BlastAnno: 1287	UniRef50_Q9SEH5	381	78.32	346	53	4	79	1095	50	380	0.0	522
2.3.1.-(Transferases;)																					
ABCDEF_520	UniRef50_Q9FF86	0.0	Normal: 1672	85	2273	5056	3383	1211	BlastAnno: 1811	UniRef50_Q9FF86	484	64.06	448	149	6	1664	354	3	449	0.0	573
ABCDEF_68664	UniRef50_Q9ZVN2	0.0	Normal: 79	35	85	107	193	63	BlastAnno: 1488	UniRef50_Q9ZVN2	704	63.99	411	142	3	46	1275	298	703	0.0	540
ABCDEF_52245	UniRef50_Q9FG87	0.0	Normal: 1878	635	1833	3814	1432	1376	BlastAnno: 1758	UniRef50_Q9FG87	529	55.35	486	204	4	4	1428	34	517	0.0	530
ABCDEF_16730	UniRef50_Q3EC11	0.0	Normal: 281	204	237	365	377	458	BlastAnno: 2092	UniRef50_Q3EC11	536	59.33	536	204	8	196	1785	3	530	0.0	661
ABCDEF_4655	UniRef50_A5BMG1	0.0	Normal: 2426	1856	3606	6958	4608	2755	BlastAnno: 2261	UniRef50_A5BMG1	481	68.37	528	112	10	263	1843	3	476	0.0	667
ABCDEF_13860	UniRef50_Q9C533	0.0	Normal: 588	220	570	1451	1338	310	BlastAnno: 2648	UniRef50_Q9C533	596	63.37	606	177	12	2226	511	1	595	0.0	558
ABCDEF_28294	UniRef50_Q9SYZ0	0.0	Normal: 1038	4070	1427	6745	877	2797	BlastAnno: 1785	UniRef50_Q9SYZ0	493	52.37	485	223	3	1598	162	5	487	0.0	533
ABCDEF_47718	UniRef50_Q9C533	0.0	Normal: 1599	236	1657	2093	1231	588	BlastAnno: 2680	UniRef50_Q9C533	596	65.02	626	165	14	2280	472	1	595	0.0	634
ABCDEF_45080	UniRef50_Q9SYZ0	0.0	Normal: 1005	592	1079	2360	3762	2217	BlastAnno: 2130	UniRef50_Q9SYZ0	493	57.85	484	197	3	302	1735	5	487	0.0	533
ABCDEF_25377	UniRef50_Q9C6L5	0.0	Normal: 37	2090	138	2573	1026	1298	BlastAnno: 1334	UniRef50_Q9C6L5	492	79.74	385	76	1	1155	1	6	388	0.0	654
ABCDEF_33470	UniRef50_Q9LW26	0.0	Normal: 172	98	156	260	391	149	BlastAnno: 2993	UniRef50_Q9LW26	701	48.96	627	301	5	655	2508	85	701	0.0	581
ABCDEF_40654	UniRef50_Q9H0A0	0.0	Normal: 638	213	530	318	318	286	BlastAnno: 2216	UniRef50_Q9H0A0	1025	56.87	721	279	6	2127	13	91	795	0.0	756
ABCDEF_33460	UniRef50_Q9ZVN2	0.0	Normal: 144	71	119	216	306	97	BlastAnno: 2691	UniRef50_Q9ZVN2	704	61.50	587	191	4	379	2133	83	636	0.0	715
ABCDEF_14442	UniRef50_Q9SYZ0	0.0	Normal: 341	684	439	1040	1601	552	BlastAnno: 1779	UniRef50_Q9SYZ0	493	63.12	480	171	2	1570	149	5	484	0.0	628
ABCDEF_52626	UniRef50_B6SYP9	0.0	Normal: 815	346	1131	3015	1537	108	BlastAnno: 1861	UniRef50_B6SYP9	494	55.02	438	189	3	1538	249	51	488	0.0	536
ABCDEF_644	UniRef50_Q9SB58	0.0	Normal: 704	444	913	969	1431	848	BlastAnno: 1777	UniRef50_Q9SB58	407	64.15	424	129	8	1572	316	1	406	0.0	554
ABCDEF_27087	UniRef50_Q52T38	0.0	Normal: 324	221	233	419	343	412	BlastAnno: 1631	UniRef50_Q52T38	620	75.95	420	91	1	1409	150	1	410	0.0	681
ABCDEF_34187	UniRef50_Q8L5Y5	0.0	Normal: 497	415	588	523	291	433	BlastAnno: 2677	UniRef50_Q8L5Y5	718	57.30	733	251	15	202	2274	1	713	0.0	683
ABCDEF_47966	UniRef50_Q9SYZ0	0.0	Normal: 577	934	326	580	682	3440	BlastAnno: 2177	UniRef50_Q9SYZ0	493	56.40	484	204	3	1780	347	5	487	0.0	535
ABCDEF_51189	UniRef50_Q52T38	0.0	Normal: 135	152	128	181	219	327	BlastAnno: 1766	UniRef50_Q52T38	620	68.09	470	137	3	80	1483	130	588	0.0	641
ABCDEF_41288	UniRef50_Q9SB58	0.0	Normal: 307	183	332	637	649	322	BlastAnno: 1403	UniRef50_Q9SB58	407	67.56	410	110	6	1401	184	15	405	0.0	523
ABCDEF_33461	UniRef50_Q9ZVN2	0.0	Normal: 142	84	140	206	283	119	BlastAnno: 2904	UniRef50_Q9ZVN2	704	60.98	574	201	6	2271	559	83	636	0.0	686
ABCDEF_14334	UniRef50_Q9SYZ0	0.0	Normal: 2263	129	1724	3469	866	283	BlastAnno: 2039	UniRef50_Q9SYZ0	493	63.22	484	171	2	369	1802	5	487	0.0	627
ABCDEF_24844	UniRef50_Q9FF86	0.0	Normal: 1857	3686	2657	21940	11467	4090	BlastAnno: 1640	UniRef50_Q9FF86	484	66.89	447	135	6	1492	185	2	446	0.0	575
ABCDEF_51190	UniRef50_Q52T38	0.0	Normal: 145	169	142	210	232	335	BlastAnno: 1891	UniRef50_Q52T38	620	70.04	514	141	3	73	1608	86	588	0.0	726
ABCDEF_14085	UniRef50_Q8L5Y5	0.0	Normal: 353	41	384	527	331	280	BlastAnno: 2362	UniRef50_Q8L5Y5	718	62.07	704	220	12	2360	348	24	713	0.0	676
ABCDEF_33458	UniRef50_Q9LW26	0.0	Normal: 158	78	130	240	342	106	BlastAnno: 2822	UniRef50_Q9LW26	701	46.72	655	302	5	400	2337	85	701	0.0	568
ABCDEF_25193	UniRef50_Q9C6L5	0.0	Normal: 3256	368	3548	6230	5109	1288	BlastAnno: 1786	UniRef50_Q9C6L5	492	65.83	480	156	4	1668	241	17	492	0.0	668
ABCDEF_33457	UniRef50_Q9ZVN2	0.0	Normal: 168	83	137	250	375	112	BlastAnno: 2823	UniRef50_Q9ZVN2	704	65.12	496	168	3	379	1860	83	575	0.0	648
ABCDEF_7634	UniRef50_Q9C6L5	0.0	Normal: 2543	9303	2711	11578	5533	10032	BlastAnno: 2064	UniRef50_Q9C6L5	492	52.76	489	225	3	1734	268	1	483	0.0	535
ABCDEF_299	UniRef50_Q71N54	0.0	Normal: 569	1147	511	900	649	1879	BlastAnno: 2017	UniRef50_Q71N54	535	61.73	533	198	5	338	1924	4	534	0.0	677
ABCDEF_27086	UniRef50_Q52T38	0.0	Normal: 784	470	617	843	893	976	BlastAnno: 2398	UniRef50_Q52T38	620	70.80	637	165	4	225	2129	1	618	0.0	887
ABCDEF_51188	UniRef50_Q52T38	0.0	Normal: 273	327	255	415	349	441	BlastAnno: 2324	UniRef50_Q52T38	620	71.30	568	150	3	2041	344	32	588	0.0	815
ABCDEF_44812	UniRef50_Q6DR03	0.0	Normal: 982	902	1005	1071	959	2999	BlastAnno: 2450	UniRef50_Q6DR03	565	54.97	644	198	17	2142	220	1	555	0.0	540
2.3.1.145(piperidine N-piperoyltransferase;)																					
No Correspond Unigene																					
2.3.1.185(tropine acyltransferase;)																					
No Correspond Unigene																					
2.3.1.186(pseudotropine acyltransferase;)																					
No Correspond Unigene																					
2.3.1.93(13-hydroxylupinine O-tigloyltransferase;)																					
No Correspond Unigene																					
2.5.1.44(homospermidine synthase)																					
No Correspond Unigene																					
2.6.1.1(aspartate transaminase;)																					
ABCDEF_11690	UniRef50_P46248	0.0	Normal: 1745	1815	2022	4078	5299	6095	BlastAnno: 1778	UniRef50_P46248	453	86.09	417	58	0	297	1547	37	453	0.0	733
ABCDEF_1333	UniRef50_C0PSV4	0.0	Normal: 356	544	636	1119	2495	631	BlastAnno: 1594	UniRef50_C0PSV4	462	76.43	403	95	0	1401	193	60	462	0.0	662
ABCDEF_21786	UniRef50_C0PSV4	0.0	Normal: 268	668	330	546	1267	646	BlastAnno: 1736	UniRef50_C0PSV4	462	74.14	464	113	3	50	1441	4	460	0.0	711

ABCDEF_42262	UniRef50_P46643	0.0	Normal: 916	606	1656	2699	2553	856	BlastAnno: 1722	UniRef50_P46643	430	79.49	429	85	2	1635	355	1	428	0.0	731
ABCDEF_56929	UniRef50_P37833	0.0	Normal: 173	278	312	654	1243	297	BlastAnno: 1151	UniRef50_P37833	407	81.82	308	56	0	978	55	3	310	0.0	536
2.6.1.5(tyrosine transaminase;)																					
ABCDEF_52650	UniRef50_Q9LVY1	0.0	Normal: 493	358	455	302	300	892	BlastAnno: 1442	UniRef50_Q9LVY1	420	61.26	413	160	0	65	1303	5	417	0.0	539
2.6.1.57(aromatic-amino-acid transaminase;)																					
No Correspond Unigene																					
2.6.1.58(phenylalanine(histidine) transaminase;)																					
No Correspond Unigene																					
2.6.1.9(histidinol-phosphate transaminase;)																					
ABCDEF_45962	UniRef50_A7NFV2	2e-99	Normal: 595	558	686	955	1251	1312	BlastAnno: 1635	UniRef50_A7NFV2	371	48.76	363	178	4	336	1403	7	368	2e-99	313
2.8.3.17(cinnamoyl-CoA:phenyllactate CoA-transferase;)																					
No Correspond Unigene																					
3.1.1.-(Hydrolases;)																					
ABCDEF_25737	UniRef50_Q9CA68	0.0	Normal: 104	11	8	393	426	18	BlastAnno: 1232	UniRef50_Q9CA68	366	69.89	352	103	2	1182	130	17	366	0.0	522
ABCDEF_525	UniRef50_Q9LIN2	0.0	Normal: 213	16	167	378	456	158	BlastAnno: 1254	UniRef50_Q9LIN2	380	69.27	358	110	0	1097	24	23	380	0.0	535
ABCDEF_28091	UniRef50_O23522	0.0	Normal: 1192	678	424	81	156	3170	BlastAnno: 1887	UniRef50_O23522	601	57.90	525	191	12	168	1721	3	504	0.0	568
ABCDEF_13432	UniRef50_Q3ECP6	0.0	Normal: 712	1725	466	588	811	6988	BlastAnno: 2479	UniRef50_Q3ECP6	408	66.83	401	104	2	2397	1282	8	408	0.0	567
ABCDEF_72624	UniRef50_Q3ECP6	0.0	Normal: 22	38	25	267	382	149	BlastAnno: 1342	UniRef50_Q3ECP6	408	66.83	401	104	2	53	1168	8	408	0.0	567
ABCDEF_4432	UniRef50_B9IQA0	0.0	Normal: 680	298	798	834	1447	644	BlastAnno: 1533	UniRef50_B9IQA0	354	78.82	321	68	0	214	1176	33	353	0.0	556
3.1.1.1(carboxylesterase;)																					
ABCDEF_10516	UniRef50_Q9LK21	0.0	Normal: 352	181	343	573	669	294	BlastAnno: 1627	UniRef50_Q9LK21	460	68.21	475	108	10	3	1373	11	460	0.0	617
ABCDEF_51842	UniRef50_Q9LK21	0.0	Normal: 401	363	273	253	190	769	BlastAnno: 1697	UniRef50_Q9LK21	460	62.77	462	128	7	109	1368	1	460	0.0	563
3.1.1.10(tropinesterase;)																					
No Correspond Unigene																					
4.1.1.18(lysine decarboxylase;)																					
No Correspond Unigene																					
5.1.-.-(Isomerases;)																					
ABCDEF_43198	UniRef50_Q9DCG6	9e-15	Normal: 9	1	0	2	59	304	BlastAnno: 591	UniRef50_Q9DCG6	288	40.00	140	70	3	49	468	6	131	9e-15	76.3
6.2.1.-(Ligases;)																					
ABCDEF_7378	UniRef50_Q9FFE6	0.0	Normal: 1022	1422	746	576	680	1408	BlastAnno: 2117	UniRef50_Q9FFE6	552	67.57	552	167	5	144	1778	1	547	0.0	768
ABCDEF_47513	UniRef50_Q8RU95	0.0	Normal: 411	609	570	233	749	887	BlastAnno: 2280	UniRef50_Q8RU95	598	53.15	572	246	6	119	1777	28	596	0.0	543
ABCDEF_78742	UniRef50_Q84P25	0.0	Normal: 236	207	373	576	450	306	BlastAnno: 1534	UniRef50_Q84P25	565	60.39	462	168	5	1413	49	19	472	0.0	546
ABCDEF_10753	UniRef50_Q9FFE6	0.0	Normal: 394	401	336	253	292	348	BlastAnno: 1841	UniRef50_Q9FFE6	552	51.00	551	259	5	117	1751	1	546	0.0	553
ABCDEF_4431	UniRef50_Q9FFE6	0.0	Normal: 27	358	28	98	112	914	BlastAnno: 2075	UniRef50_Q9FFE6	552	50.71	560	264	7	1774	107	1	552	0.0	551
ABCDEF_38213	UniRef50_Q84P25	0.0	Normal: 342	337	597	798	722	505	BlastAnno: 1939	UniRef50_Q84P25	565	62.34	555	194	5	1818	175	19	565	0.0	690
ABCDEF_25221	UniRef50_Q0DV32	0.0	Normal: 971	826	1003	1543	1442	1545	BlastAnno: 2009	UniRef50_Q0DV32	552	57.20	542	226	4	183	1793	9	549	0.0	613
ABCDEF_23413	UniRef50_Q84P23	0.0	Normal: 123	237	158	149	213	658	BlastAnno: 3175	UniRef50_Q84P23	562	51.74	547	243	8	3030	1435	15	555	0.0	555
ABCDEF_20486	UniRef50_Q9FFE6	0.0	Normal: 580	912	312	324	864	1565	BlastAnno: 3530	UniRef50_Q9FFE6	552	66.61	557	171	5	352	1995	1	551	0.0	781
>00965 Betalain biosynthesis																					
1.10.3.-(Oxidoreductases;)																					
ABCDEF_45687	UniRef50_Q00624	0.0	Normal: 2597	1174	3902	13161	17999	2817	BlastAnno: 2156	UniRef50_Q00624	555	55.41	518	221	4	1840	314	24	540	0.0	576
ABCDEF_85228	UniRef50_Q00624	0.0	Normal: 47	55	24	16	59	389	BlastAnno: 1993	UniRef50_Q00624	555	54.87	503	213	6	245	1720	41	540	0.0	538
ABCDEF_37702	UniRef50_Q00624	0.0	Normal: 303	163	411	498	1042	1519	BlastAnno: 2056	UniRef50_Q00624	555	55.66	521	213	5	1775	255	24	540	0.0	576
ABCDEF_7339	UniRef50_Q00624	0.0	Normal: 1973	108	2117	11689	7999	246	BlastAnno: 2208	UniRef50_Q00624	555	54.32	521	229	4	1899	361	24	543	0.0	567
ABCDEF_41293	UniRef50_Q00624	0.0	Normal: 114	527	425	2664	2274	2864	BlastAnno: 2131	UniRef50_Q00624	555	54.63	518	225	4	316	1842	24	540	0.0	567
ABCDEF_41446	UniRef50_Q00624	0.0	Normal: 1149	179	484	181	771	508	BlastAnno: 1931	UniRef50_Q00624	555	54.44	518	226	4	105	1631	24	540	0.0	553
ABCDEF_48453	UniRef50_Q00624	0.0	Normal: 1968	8	2283	8826	11798	216	BlastAnno: 2052	UniRef50_Q00624	555	54.04	520	229	4	1778	246	25	543	0.0	558
ABCDEF_18058	UniRef50_Q00624	0.0	Normal: 1235	4137	1524	639	10823	1526	BlastAnno: 2082	UniRef50_Q00624	555	54.05	518	228	4	280	1806	24	540	0.0	563
1.13.11.-(Oxidoreductases;)																					
No Correspond Unigene																					
1.14.18.-(Oxidoreductases;)																					
No Correspond Unigene																					
1.14.18.1(monophenol monooxygenase;)																					
No Correspond Unigene																					
2.1.1.6(catechol O-methyltransferase;)																					
ABCDEF_3867	UniRef50_Q9ZTU1	4e-129	Normal: 43	1755	68	456	3254	474	BlastAnno: 1421	UniRef50_Q9ZTU1	351	51.57	351	167	2	1306	260	2	351	4e-129	387
2.3.1.-(Transferases;)																					
ABCDEF_520	UniRef50_Q9FF86	0.0	Normal: 1672	85	2273	5056	3383	1211	BlastAnno: 1811	UniRef50_Q9FF86	484	64.06	448	149	6	1664	354	3	449	0.0	573
ABCDEF_68664	UniRef50_Q9ZVN2	0.0	Normal: 79	35	85	107	193	63	BlastAnno: 1488	UniRef50_Q9ZVN2	704	63.99	411	142	3	46	1275	298	703	0.0	540
ABCDEF_52245	UniRef50_Q9FG87	0.0	Normal: 1878	635	1833	3814	1432	1376	BlastAnno: 1758	UniRef50_Q9FG87	529	55.35	486	204	4	4	1428	34	517	0.0	530
ABCDEF_16730	UniRef50_Q3EC11	0.0	Normal: 281	204	237	365	377	458	BlastAnno: 2092	UniRef50_Q3EC11	536	59.33	536	204	8	196	1785	3	530	0.0	661
ABCDEF_4655	UniRef50_A5BMG1	0.0	Normal: 2426	1856	3606	6958	4608	2755	BlastAnno: 2261	UniRef50_A5BMG1	481	68.37	528	112	10	263	1843	3	476	0.0	667
ABCDEF_13860	UniRef50_Q9C533	0.0	Normal: 588	220	570	1451	1338	310	BlastAnno: 2648	UniRef50_Q9C533	596	63.37	606	177	12	2226	511	1	595	0.0	558
ABCDEF_28294	UniRef50_Q9SYZ0	0.0	Normal: 1038	4070	1427	6745	877	2797	BlastAnno: 1785	UniRef50_Q9SYZ0	493	52.37	485	223	3	1598	162	5	487	0.0	533
ABCDEF_47718	UniRef50_Q9C533	0.0	Normal: 1599	236	1657	2093	1231	588	BlastAnno: 2680	UniRef50_Q9C533	596	65.02	626	165	14	2280	472	1	595	0.0	634
ABCDEF_45080	UniRef50_Q9SYZ0	0.0	Normal: 1005	592	1079	2360	3762	2217	BlastAnno: 2130	UniRef50_Q9SYZ0	493	57.85	484	197	3	302	1735	5	487	0.0	533
ABCDEF_25377	UniRef50_Q9C6L5	0.0	Normal: 37	2090	138	2573	1026	1298	BlastAnno: 1334	UniRef50_Q9C6L5	492	79.74	385	76	1	1155	1	6	388	0.0	654
ABCDEF_33470	UniRef50_Q9LW26	0.0	Normal: 172	98	156	260	391	149	BlastAnno: 2993	UniRef50_Q9LW26	701	48.96	627	301	5	655	2508	85	701	0.0	581
ABCDEF_40654	UniRef50_Q9H0A0	0.0	Normal: 638	213	530	318	318	286	BlastAnno: 2216	UniRef50_Q9H0A0	1025	56.87	721	279	6	2127	13	91	795	0.0	756
ABCDEF_33460	UniRef50_Q9ZVN2	0.0	Normal: 144	71	119	216	306	97	BlastAnno: 2691	UniRef50_Q9ZVN2	704	61.50	587	191	4	379	2133	83	636	0.0	715
ABCDEF_14442	UniRef50_Q9SYZ0	0.0	Normal: 341	684	439	1040	1601	552	BlastAnno: 1779	UniRef50_Q9SYZ0	493	63.12	480	171	2	1570	149	5	484	0.0	628
ABCDEF_52626	UniRef50_B6SYP9	0.0	Normal: 815	346	1131	3015	1537	108	BlastAnno: 1861	UniRef50_B6SYP9	494	55.02	438	189	3	1538	249	51	488	0.0	536

ABCDEF_644	UniRef50_Q9SB58	0.0	Normal: 704	444	913	969	1431	848	BlastAnno: 1777	UniRef50_Q9SB58	407	64.15	424	129	8	1572	316	1	406	0.0	554
ABCDEF_27087	UniRef50_Q52T38	0.0	Normal: 324	221	233	419	343	412	BlastAnno: 1631	UniRef50_Q52T38	620	75.95	420	91	1	1409	150	1	410	0.0	681
ABCDEF_34187	UniRef50_Q8L5Y5	0.0	Normal: 497	415	588	523	291	433	BlastAnno: 2677	UniRef50_Q8L5Y5	718	57.30	733	251	15	202	2274	1	713	0.0	683
ABCDEF_47966	UniRef50_Q9SYZ0	0.0	Normal: 577	934	326	580	682	3440	BlastAnno: 2177	UniRef50_Q9SYZ0	493	56.40	484	204	3	1780	347	5	487	0.0	535
ABCDEF_51189	UniRef50_Q52T38	0.0	Normal: 135	152	128	181	219	327	BlastAnno: 1766	UniRef50_Q52T38	620	68.09	470	137	3	80	1483	130	588	0.0	641
ABCDEF_41288	UniRef50_Q9SB58	0.0	Normal: 307	183	332	637	649	322	BlastAnno: 1403	UniRef50_Q9SB58	407	67.56	410	110	6	1401	184	15	405	0.0	523
ABCDEF_33461	UniRef50_Q9ZVN2	0.0	Normal: 142	84	140	206	283	119	BlastAnno: 2904	UniRef50_Q9ZVN2	704	60.98	574	201	6	2271	559	83	636	0.0	686
ABCDEF_14334	UniRef50_Q9SYZ0	0.0	Normal: 2263	129	1724	3469	866	283	BlastAnno: 2039	UniRef50_Q9SYZ0	493	63.22	484	171	2	369	1802	5	487	0.0	627
ABCDEF_24844	UniRef50_Q9FF86	0.0	Normal: 1857	3686	2657	21940	11467	4090	BlastAnno: 1640	UniRef50_Q9FF86	484	66.89	447	135	6	1492	185	2	446	0.0	575
ABCDEF_51190	UniRef50_Q52T38	0.0	Normal: 145	169	142	210	232	335	BlastAnno: 1891	UniRef50_Q52T38	620	70.04	514	141	3	73	1608	86	588	0.0	726
ABCDEF_14085	UniRef50_Q8L5Y5	0.0	Normal: 353	41	384	527	331	280	BlastAnno: 2362	UniRef50_Q8L5Y5	718	62.07	704	220	12	2360	348	24	713	0.0	676
ABCDEF_33458	UniRef50_Q9LW26	0.0	Normal: 158	78	130	240	342	106	BlastAnno: 2822	UniRef50_Q9LW26	701	46.72	655	302	5	400	2337	85	701	0.0	568
ABCDEF_25193	UniRef50_Q9C6L5	0.0	Normal: 3256	368	3548	6230	5109	1288	BlastAnno: 1786	UniRef50_Q9C6L5	492	65.83	480	156	4	1668	241	17	492	0.0	668
ABCDEF_33457	UniRef50_Q9ZVN2	0.0	Normal: 168	83	137	250	375	112	BlastAnno: 2823	UniRef50_Q9ZVN2	704	65.12	496	168	3	379	1860	83	575	0.0	648
ABCDEF_7634	UniRef50_Q9C6L5	0.0	Normal: 2543	9303	2711	11578	5533	10032	BlastAnno: 2064	UniRef50_Q9C6L5	492	52.76	489	225	3	1734	268	1	483	0.0	535
ABCDEF_299	UniRef50_Q71N54	0.0	Normal: 569	1147	511	900	649	1879	BlastAnno: 2017	UniRef50_Q71N54	535	61.73	533	198	5	338	1924	4	534	0.0	677
[truncated: 1,441,674 more chars]
